# Metabolomics Analysis of Metabolic Effects of Nicotinamide Phosphoribosyltransferase (NAMPT) Inhibition on Human Cancer Cells

**DOI:** 10.1371/journal.pone.0114019

**Published:** 2014-12-08

**Authors:** Vladimir Tolstikov, Alexander Nikolayev, Sucai Dong, Genshi Zhao, Ming-Shang Kuo

**Affiliations:** 1 Discovery Chemistry and Technologies, Lilly Research Laboratories, Eli Lilly and Company, 893 S Delaware St, Indianapolis, IN 46285, United States of America; 2 Cancer Signaling & Metabolism, Lilly Research Laboratories, Eli Lilly and Company, 893 S Delaware St, Indianapolis, IN 46285, United States of America; Spanish National Cancer Center, Spain

## Abstract

Nicotinamide phosphoribosyltransferase (NAMPT) plays an important role in cellular bioenergetics. It is responsible for converting nicotinamide to nicotinamide adenine dinucleotide, an essential molecule in cellular metabolism. NAMPT has been extensively studied over the past decade due to its role as a key regulator of nicotinamide adenine dinucleotide–consuming enzymes. NAMPT is also known as a potential target for therapeutic intervention due to its involvement in disease. In the current study, we used a global mass spectrometry–based metabolomic approach to investigate the effects of FK866, a small molecule inhibitor of NAMPT currently in clinical trials, on metabolic perturbations in human cancer cells. We treated A2780 (ovarian cancer) and HCT-116 (colorectal cancer) cell lines with FK866 in the presence and absence of nicotinic acid. Significant changes were observed in the amino acids metabolism and the purine and pyrimidine metabolism. We also observed metabolic alterations in glycolysis, the citric acid cycle (TCA), and the pentose phosphate pathway. To expand the range of the detected polar metabolites and improve data confidence, we applied a global metabolomics profiling platform by using both non-targeted and targeted hydrophilic (HILIC)-LC-MS and GC-MS analysis. We used Ingenuity Knowledge Base to facilitate the projection of metabolomics data onto metabolic pathways. Several metabolic pathways showed differential responses to FK866 based on several matches to the list of annotated metabolites. This study suggests that global metabolomics can be a useful tool in pharmacological studies of the mechanism of action of drugs at a cellular level.

## Introduction

As a continuation to a previous study on the pharmacological inhibition of nicotinamide phosphoribosyltransferase (NAMPT) describing the metabolic basis of NAMPT inhibition [1], we report here the results of a global metabolomics analysis that revealed the metabolic alterations of NAMPT inhibition in human cancer cells. The nicotinamide adenine dinucleotide (NAD) cofactor is essential for a variety of cellular processes. In mammals, NAD can be synthesized from nicotinamide, nicotinic acid, or tryptophan [2]–[5]. The in vivo concentration of nicotinic acid is low due to its rapid excretion and metabolism, suggesting that the utilization of nicotinic acid for NAD biosynthesis as compared to nicotinamide is limited in mammals [3]. The de novo biosynthesis of NAD from tryptophan mainly occurs in the liver [4]. Therefore, the two-step salvage pathway that converts nicotinamide to NAD represents the major route to NAD biosynthesis in mammals [6]–[8]. NAMPT, originally identified as a pre-B-cell colony-enhancing factor [9], is the rate-limiting enzyme that catalyzes the first step in the biosynthesis of NAD from nicotinamide [10], [11]. Recent studies have demonstrated that NAMPT-mediated NAD biosynthesis in cancer cells plays a crucial role in several physiological processes, including metabolism, energy generation, survival, apoptosis, DNA repair, and inflammation [2], [12]–[14]. It was demonstrated that NAMPT is overexpressed in several types of tumors, including breast, colorectal, gastric, lung, prostate, and other carcinomas [15]–[18], and its expression appears to be associated with tumor progression [19]. In the cell, NAMPT is abundant in the cytosol and present in the nucleus. It has been adequately reported that NAD turnover in cancer or proliferating cells is significantly increased over healthy or non-proliferating cells [1], [7]. These observations on the possible involvement of NAMPT in disease have now been supported by various approaches in cancer cells studies [10]–[14]. The down-regulation of NAMPT suppresses tumor cell growth in vitro and in vivo and sensitizes cells to oxidative stress and DNA-damaging agents [8], [15], [18], [20]–[22]. The inhibition of NAMPT also leads to the attenuation of tumor growth and induction of apoptosis due to NAD depletion [8], [21]–[24]. Taken together, NAMPT exemplifies a promising therapeutic target for the development of potential novel cancer drugs.

NAD is a substrate for dehydrogenases, poly(ADP-ribose) polymerases, sirtuins, mono(ADP-ribosyl) transferases, and ADP-ribosyl cyclases [2], [4], [12]. In most cancer cells, poly(ADP-ribose) polymerase, a key protein required for DNA repair that is also involved in apoptosis, is activated due to DNA damage and genome instability [2], [25]–[27]. The activation of poly(ADP-ribose) polymerase leads to NAD depletion in cancer cells [2], [8], [25]–[27]. As a result, the down-regulation of NAMPT sensitizes cancer cells to DNA-damaging agents and apoptosis [10], [21]. Similarly, Sir2 protein also serves as a key downstream effector of NAMPT that regulates a variety of cellular functions, including survival and inflammation [28]–[30]. Recent studies have demonstrated that Sir2 proteins regulate cytokine production [30], which in turn reduces NAD levels through the inhibition of NAMPT. Furthermore, a NAMPT inhibitor has shown anti-inflammatory effects in animal models of inflammation [20], [30]. Finally, the key mechanism of action of NAMPT inhibition is the blockade of glycolysis at the glyceraldehydes-3-phosphate dehydrogenase step responsible for adenosine triphosphate (ATP) depletion, metabolic perturbation, and subsequent tumor growth inhibition [1]. However, how modulating the NAMPT activity in cancer cells affects cellular metabolism, outside of energy metabolism as previously reported [1], remains unknown. FK866, a small molecule inhibitor of NAMPT, has been the subject of extensive studies [2], [21], [31]. The molecule has been co-crystallized with and found to be bound to the nicotinamide binding pocket of NAMPT, thereby demonstrating its mechanism as a competitive inhibitor of NAMPT with respect to nicotinamide [32]. Several studies also suggest that FK866 specifically inhibits NAMPT in the cell and exhibits anti-tumor activity in preclinical tumor models [11], [14], [20], [30], [32]–[35]. Thus, FK866 appears to be an ideal tool molecule for assessing the physiological function of NAMPT in the cell. In the current study, we wanted to further assess the global effects of NAMPT inhibition by FK866 on cancer metabolism by using global mass spectrometry–based metabolic profiling. We have chosen two human cancer cell lines that differ in the way they use nicotinic acid and nicotinamide for NAD formation, assuming that the addition of nicotinic acid to the growth medium can abolish NAMPT inhibition in one cell line but not in the other. We have found that the inhibition of NAMPT by FK866 results in the alteration of numerous metabolic pathways far beyond energy metabolism. Therefore, the global mass spectrometry–based metabolic profiling approach is a useful tool for mechanistic studies of drug actions and for identifying potential pharmacodynamics markers.

## Materials and Methods

### Study Design

Each of two cell lines were treated with 5 and 50 nM of FK866 (in 0.1% DMSO) and 0.1% DMSO with and without nicotinic acid (10 µM) for 24 hours. Each group was powered with 6 biological replicates. Cell lines that were not treated with drug and nicotinic acid served as controls.

### Cancer Cells

A2780 (NCI DCTD), an ovarian cancer cell line, and HCT-116 (ATCC), a colorectal cancer cell line, were cultured in RPMI 1640 (Invitrogen 30–2001) and McCoys 5a (Hyclone SH30200), respectively, in the presence of 10% FBS. Cells were seeded into a 6-well culture plate at a density of 1.0×10^6^ cells per well and incubated at 37°C in 5% CO_2_ for 24 hours and then treated with FK866 in DMSO at various concentrations for 24 hours. FK866 was synthesized as described previously [36], [37]. As a part of the study design, cells were also grown and treated with nicotinic acid (10 µM) and FK866 for 24 hours before harvest. Cell viability was assessed by measuring total protein content using a CytoScan SRB Cytotoxicity Assay Kit (Cat. No. 786-213; G-Biosciences, St. Louis, MO). After 24 hours of treatment, the medium was removed and pre-chilled at −20°C; next, 500 µL of 80% methanol was added to each well. The cells were scraped and collected at −20°C into 1.5 mL eppendorf tubes. After 60 minutes, the eppendorf tubes were centrifuged for 5 minutes at 14,000 rotations per minute and placed supernatant into new tubes for further analysis.

### Metabolomics Analysis

Analyses were performed using non-targeted and targeted protocols using GC-TOF-HRMS, hydrophilic (HILIC)-LC-HRMS and HILIC-LC-MS/MS instrumentation implementing previously reported methodology [38]–[40]. A standard quality control (QC) sample containing a mixture of amino acids and organic acids was injected daily to monitor mass spectrometer response. The pooled QC sample was obtained by taking an aliquot of the same volume of all samples from the study. The pooled QC sample was injected daily with a batch of analyzed samples and was used to determine the optimal dilution of the batch samples and to validate metabolite identification and peak integration (S1 File). Supernatants of cell extracts were divided in to three parts: 75 µL for GC-TOF-MS analysis, 175 µL for HILIC-LC/MS analysis, and 175 µL for HILIC-LC/MS/MS analysis.

### Sample Derivatization and GC-TOF-HRMS Analysis

Extracts were dried using SpeedVac Concentrator Savant DNA120 (ThermoScientific, San Jose, CA) with the bath temperature set below 30°C. Pre-chilled samples were further dried over 1 hour using a Free-Zone lyophilizer (Labconco, Kansas City, MO). Dried sample derivatization with methoxylamine hydrochloride in pyridine and N-methyl-N-trimethylsilyltrifluoroacetamide was performed as described by Tong et al. [37]. Gas chromatography was performed using an Agilent 7890A gas chromatograph (Agilent, Palo Alto, CA) interfaced to a high-resolution time-of-flight Pegasus GC-HRT mass spectrometer (Leco, St. Joseph, MI). Automated injections were performed using an MPS2 programmable robotic multipurpose sampler (Gerstel, Muhlheim an der Ruhr, Germany). The GC system was fitted with a Gerstel temperature-programmed injector, cooled injection system (model CIS 4). An automated liner exchange (ALEX) (Gerstel) was used to eliminate cross-contamination from the sample matrix that was occurring between sample runs. Multiple deactivated baffled liners for the GC inlet were used. The Gerstel injector was programmed for the following sequence: initial temperature 50°C, hold for 0.1 minute, increase temperature at a rate of 10°Cs-1 to a final temperature of 330°C, and hold time 15 minutes). Injections of 1 µL were made in the splitless mode. Chromatography was performed on an Rtx-5Sil MS column (length: 30 m; ID: 0.25 mm; df: 0.25 µm) with an Integra-Guard column (Restek, Bellefonte, PA). Helium carrier gas was used at a constant flow of 1 mL min-1. The GC oven temperature was programed for the following sequence: 50°C initial temperature with a 1-minute hold time and then ramping at 10°C per minute to a temperature of 140°C, then ramping at 4°C per minute to a temperature of 240°C, and then ramping at 10°C per minute to a temperature of 300°C with an 8-minute hold time. Both the transfer line and the source temperatures were 250°C. Ion source operated at −70 kV filament voltage. After a solvent delay of 500 seconds, mass spectra were acquired at 2.4 spectra per second with an extraction frequency of 2 kHz and a mass range of 60 to 520 m/z. Resolution was set to 25 K. Mass accuracy was controlled with PFTBA tuning at a sub-ppm level during the whole run time. The standard QCs and pooled sample QCs were used to monitor GC-TOF-HRMS data acquisition (S1 File) [41]–[43]. Mass spectrometer calibration was performed on daily basis using vendor protocol. Mass accuracy was routinely maintained in a sub-ppm level at a resolution of 30 to 40 K. Data analysis was performed with vendor software ChromaTof (LECO, St. Joseph, MI) and AnalyzerPro (SpectralWorks Ltd, Runcorn, UK) using the latest NIST-MS database (http://chemdata.nist.gov/).

### HILIC-LC-MS/MS Analysis

Extracts were used without any further derivatization. HILIC-LC-MS/MS data acquisition and processing were monitored using standard QCs [42], [43] and pooled sample QCs (S1 File). Liquid chromatography was performed using the NEXERA UPLC system (Shimadzu, Columbia, MD) coupled with the Triple Quad 5500 System (AB Sciex, Framingham, MA). HILIC separations were achieved using a polyamine-bonded polymeric gel column (apHera NH2 Polymer) with a 150×2 mm, 5-µm particle size, equipped with a guard column (apHera NH2 Polymer) with a 10×2 mm, 5-µm particle size (SUPELCO, Bellefonte, PA). The mobile phases were acetonitrile (A) and 50 mM ammonium bicarbonate (pH 9.4, adjusted with ammonium hydroxide) (B) at the flow rates of 0.25 mL/minutes at 30°C. After 3-minute isocratic run at 15% B, a gradient to 30% B was concluded at 12 minutes and a gradient to 60% B was concluded at 15 minutes. After that, a gradient to 75% B was concluded at 21 minutes and a gradient to 98% B was completed at 22 minutes. Following column wash, the run was concluded with 98% B at 29 minutes. Column equilibration with a starting buffer took 6 minutes before the next injection. Data acquisition was performed using scheduled MRMs. Total list contained more than 200 MRM transitions generated with authentic standards in positive and in negative modes [43] (S1 File).

### HILIC-LC-HRMS Analysis

Extracts were used without any further derivatization. The standards QCs and pooled sample QCs were used to monitor HILIC-LC-HRMS data acquisition as previously described (S1 File) [42]–[44]. Liquid chromatography was performed using the WATERS UPLC system (WATERS, Milford, MA) coupled with the Triple TOF 5600 System (AB Sciex). HILIC separations were achieved using the above described protocols. The Information Dependent Acquisition experiment was used for data acquisition within a 70 to 800 m/z mass range for positive and negative ions separately. Data were processed using MarkerView version 1.2.1 software (AB Sciex). Spiking pooled samples with commercially available authentic standards allowed for the identification of sample components. Data were collected for positive and negative ions separately within a 70 to 800 m/z mass range, implementing experiment intended to fragment all the ions exceeding selected threshold while applying rolling fragmentation energy. Mass spectrometer calibration was performed on daily basis using the vendor protocol for positive and negative ions. Mass accuracy was routinely maintained at 5 ppm level at a resolution of 20 to 30K. Component identification was accomplished with spiking authentic standards when available. Unknown components were identified using recalibrated spectral data with the assistance of PeakView version 1.2.0.3 software (AB Sciex). Batch recalibration was performed using previously identified components having different masses and retention times as internal standards. This protocol allowed for the routine achievement of a 2 to 5 ppm mass accuracy deviation for parent ions and their fragments (S1 File). METLIN (http://metlin.scripps.edu/index.php), HMDB (http://www.hmdb.ca/), MASSBANK (http://www.massbank.jp/), NIST-MS (http://chemdata.nist.gov/), IDEOME (http://mzmatch.sourceforge.net/ideom.php), mzCloud (https://mzcloud.org/), and in-house generated HRMS and HRMS/MS databases were used for the elemental composition assignment, spectral data comparisons, and detailed manual interpretation. This discovery protocol allowed for the annotation of metabolites, which were not commercially available (S1 File). Annotated data were further processed with MarkerView version 1.2.1 software (AB Sciex) to calculate the metabolite peak area. Unidentified features were routinely excluded from the peak list. HILIC-LC-HRMS data were used to clarify the cross-talk observed during HILIC-LC-MS/MS analysis, and it was used for the measurement of metabolites that had concentrations above linear range during the HILIC-LC-MS/MS analysis. It was also used for assignment retention times for isomers (S1 File).

### Data Processing and Statistical Analysis

**Figure 1:**
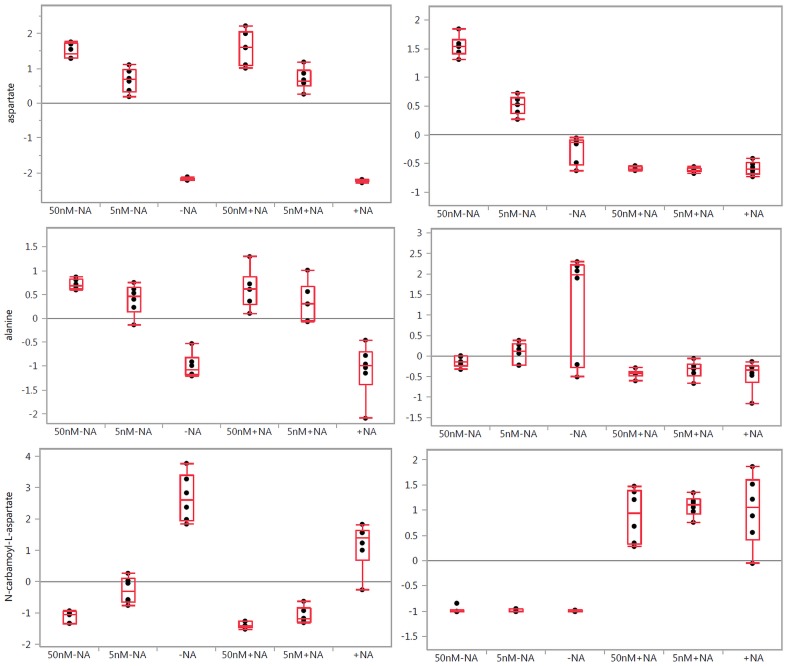
One-way analysis of variance box plots illustrating the observed changes in the levels of aspartate, alanine, and N-carbamoyl-L-aspartate when treated with FK866 at 50 and 5 nM concentrations in A2780 (left panels) and HCT-116 (right panels) cells, respectively, with (+NA) and without (−NA) nicotinic acid addition. Grand mean is shown as a horizontal line located within panel. The y-axis illustrates normalized, log transformed, and scaled peak area. Dots represent samples. Group mean shown as a horizontal line within the box.

GC-MS data analysis using ChromaTof allowed for peak lists generation, which were further extracted across datasheets and combined into a master list. In addition, the data processing was performed with AnalyzerPro and a matrix analyzer add-on was used to generate a matrix target library. The NIST-MS database was used to generate this library. This protocol resulted in the generation of several thousand features. Data were further filtered as described in Chan et al. [41] and Dunn et al. [42] and merged with the data generated with ChromaTof assistance. Manual inspection and duplicates removal was performed to form a final metabolites peak table identified and measured with the GC-MS method. Median data normalization (row scaling), logarithmic transformation, and data scaling (Pareto) for GC-MS and LC-MS data were performed using MetaboAnalyst version 2.0 software [45] in order to compensate for inter- and intra-instrumental variation. A final metabolite peak table was constructed by merging data acquired with the all methods. Peak list, generated with LC-MS/MS method, served as a basis for data merging and integration (S1 File). Duplicates introduced with the complementary methods (GC-HRMS and HILIC-LC-HRMS) were removed after manual inspection. Metabolite intensities from individual samples were normalized to corresponding protein concentration prior to further statistical analysis. Statistical analyses were performed using univariate and multivariate approaches. MetaboAnalyst version 2.0 [45] and JMP version 11 packages were used for statistical analyses. Analysis of variance (ANOVA) followed by Dunnett's Multiple Comparison Test was performed for data comparisons between groups, defined with the study design as follows: control (designated as −NA) and treated cells (designated as +NA, 50 nM-NA, 5 nM-NA, 50 nM+NA, 5 nM+NA). Figures containing illustrations of the ANOVA results have graphic information explained as follows: differences between groups were considered statistically significant when p <0.05. Grand mean is shown as a horizontal line located within the panel (Fig. 1). The y-axis illustrates normalized, log transformed, and scaled peak area. Experimental groups at the different treatments were further classified using unsupervised hierarchical clustering, principal component analysis, and supervised multivariate analysis partial least squares-discriminant analysis (S3 File).

### Pathway and Network Analysis

Identified metabolites were subjected to IPA analysis (Ingenuity System, Redwood City, CA). Accession numbers of detected metabolites (HMDB, PubChem, and KEGG Identifiers) were listed in MS Excel and imported into IPA to map the canonical pathways and generate networks of interacting biological entities. Data were submitted as fold change values (ratios) calculated against the control group (designated as −NA). Comprehensive pathway and network analyses were performed. Downstream biological processes were scored in accordance to the ontology support using Ingenuity Knowledge Base (http://www.ingenuity.com/science/knowledge-base). IPA analysis settings were as follows:

- Reference set: Ingenuity Knowledge Base (endogenous chemicals).
- Relationship to include: direct and indirect.
- Includes endogenous chemicals.
- Filter summary: Consider only relationships where confidence is experimentally observed or high (predicted).
- Analysis setting included data collected from human cells.

P-value explanation is as follows: a measure of the likelihood that the association between a set of metabolites in the dataset and a related function is due to random association. The smaller the p-value, the less likely it is that the association is random and the more significant the association. In general, p-values <0.05 indicate a statistically significant, non-random association. IPA uses the activation z-score algorithm to make predictions. The z-score algorithm is designed to produce either a prediction of activation or inhibition (or no prediction), as well as to reduce the chance that random data will generate significant predictions.

## Results

To assess the effects of NAMPT inhibition on cancer metabolism, we used two different cell lines: A2780, a NARPT-negative cell line that can only utilize the NAMPT-mediated nicotinamide pathway for NAD biosynthesis, and HCT-116, a NAPRT-positive cell line that can use both NAMPT-mediated nicotinamide and NAPRT-mediated nicotinic acid pathways for NAD biosynthesis. Because HCT-116 can use nicotinamide and nicotinic acid for NAD formation, the addition of nicotinic acid to the growth medium can abolish NAMPT inhibition in HCT-116 but not in A2780. Therefore, nicotinic acid was used in the current study to assess the specificity of NAMPT inhibitors.

### Amino Acids Metabolism

Using global metabolomics protocols, we observed significant changes in amino acid metabolism on NAMPT inhibition with FK866 in both cell lines, with the exception of glycine and serine metabolism, arginine metabolism, and histidine metabolism (S2–S5 Files). As shown in Fig. 1, the alanine and aspartate metabolism were significantly impacted. Drug dose dependent induced changes in aspartate, alanine, and N-carbamoyl-aspartate levels were observed. The addition of nicotinic acid completely abolished these effects observed in HCT-116 but not in A2780 (Fig. 1, S4 File and S5 Files), indicating that these effects were due to NAMPT inhibition. It is interesting that asparagine and glutamate levels were not significantly altered. A marginal decrease in glutamate and glutamine levels in HCT-116 cells was also observed. Elevations in threonine levels were observed (S2 File) and reversed with nicotinic acid treatment in HCT-116 cells, supporting the connection to aspartate, which is a source for threonine biosynthesis.

Interestingly, the levels of N-acetylglutamine were elevated in both cell lines when treated with FK866 and only the effects in HCT-116 were rescued by addition of nicotinic acid. Similar results were obtained for lysine, N-acetyl-lysine, phenylalanine, tryptophan, tyrosine, leucine, isoleucine, methionine, SAM, and proline (S3 File, S4 File and S5 Files). SAM is known to participate in cysteine and methionine metabolism, arginine and proline metabolism, the biosynthesis of amino acids, and in transmethylation, transsulfuration, and aminopropylation. It was found that alterations of SAM levels are strikingly similar to changes in aspartate levels (S3 File, S4 File and S5 Files). Finally, NAMPT inhibition also leads to altered polyamine metabolism because spermine and spermidine levels were elevated in a dose-dependent manner on treatment with FK866. The addition of nicotinic acid completely abolished the effects observed in HCT-116 cells but not in A2780 cells (S3 File, S4 File and S5 Files). Thus, NAMPT inhibition appears to perturb several amino acid pathways and some of the effects appear to be cell line specific.

### Purine and Pyrimidine Metabolism

**Figure 2:**
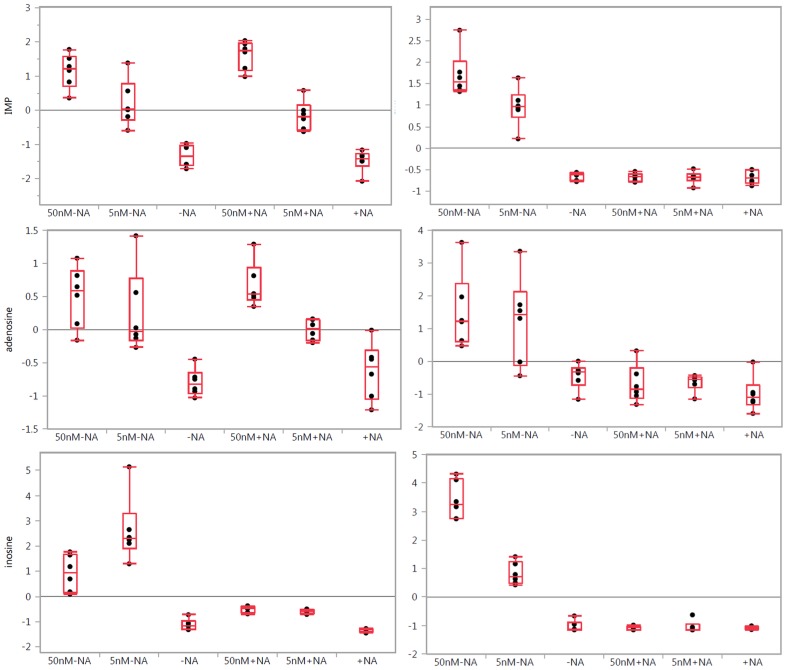
One-way analysis of variance box plots illustrating the observed level changes in the selected purines: inosine monophosphate (IMP), adenosine, and inosine after FK866 treatment at 50 and 5 nM concentrations in A2780 (left panels) and HCT-116 (right panels) cells, respectively, with (+NA) and without (−NA) nicotinic acid addition. Grand mean is shown as a horizontal line located within panel. The y-axis illustrates normalized, log transformed, and scaled peak area. Dots represent samples. Group mean shown as a horizontal line within the box.

Because the several steps of purine and pyrimidine biosynthesis require NAD and intermediates that derive from carbohydrate metabolism, we assessed the effects of NAMPT inhibition on nucleotide-related metabolism.As shown in Fig. 2 and in S3 File, S4 File and S5 Files, we observed changes in levels of purines in response to FK866 treatment because there was a dose-dependent increase in levels of adenine, cytidine, cytosine, adenosine, 1-methyladenosine, inosine, adenosine monophosphate (AMP), adenosine diphosphate (ADP), inosine monophosphate (IMP), inosine diphosphate (IDP), cytidine diphosphate (CDP), deoxyguanosine diphosphate (dGDP), 5-Aminoimidazole-4-carboxamide ribonucleotide (AICAR), AICAR 3′,5′-cyclic phosphate, N-formylglycinamide ribonucleotide, orotidine, purine, thymidine, and uridine in both cell lines, similar to the increase in amino acids levels. Interestingly, the addition of nicotinic acid significantly diminished the elevated levels of inosine in both cell lines (Fig. 2, S2 File, S3 File, S4 File and S5 Files). Levels of xanthine were not changed in A2780 cells but increased in a dose-dependent manner in HCT-116 cells, suggesting a cell line specific difference in xanthine-related metabolism. There was a dose dependent decrease in 7-methylguanosine, deoxyadenosine, deoxyuridine, guanosine monophosphate (GMP), cytidine monophosphate (CMP), deoxycytidine monophosphate (dCMP), deoxyadenosine monophosphate (dAMP), and uridine triphosphate (UTP) levels. These effects were due to NAMPT inhibition because the addition of nicotinic acid abolished these effects in HCT-116 cells but not in A2780 cells. Interestingly, treatment with 5 nM of FK866 increased dCMP levels comparable to those of 50 nM of FK866 in A2780 cells. Again, there was a cell line specific effect observed because NAMPT inhibition caused a decrease in levels cyclic AMP and AICAR ribotide in A2780 cells but not in HCT-116 cells (S2 File, S3 File, S4 File and S5 Files).

### Creatine Metabolism

NAMPT inhibition led to a dose-dependent increase in creatine and creatinine levels in both cell lines similar to amino acids levels. Consistent with this, the addition of nicotinic acid abolished these effects in HCT-116 cells but not in A2780 cells (S2 File, S3 File, S4 File and S5 Files).

### Lipid Metabolism

Fatty acid biosynthesis requires a large amount of NADP/NADPH derived from NAD and citrate (a TCA intermediate); we observed significant changes in lipid metabolism in response to FK866 treatment. Palmitic and stearic acid levels were elevated in a dose dependent manner in both cell lines. Interestingly, the addition of nicotinic acid abolished these effects in both cell lines. The treatment with FK866 led to increased levels of choline, phosphorylcholine, and phoshatydylglycerol in HCT-116 cells but not in A2780 cells. These effects observed in HCT-116 cells were a result of NAMPT inhibition because the effects were abolished with the addition of nicotinic acid to the growth medium. Interestingly, we observed a strong dose-dependent increase in glycerophosphocholine and glycerol-3-phosphate levels in A2780 cells but not in HCT-116 cells. The FK866 treatment also resulted in a significant dose-dependent decrease in the lyso-PC and PC levels detected in A2780 cells but marginal changes in HCT-116 cells (S2 File, S3 File, S4 File and S5 Files).

**Figure 3:**
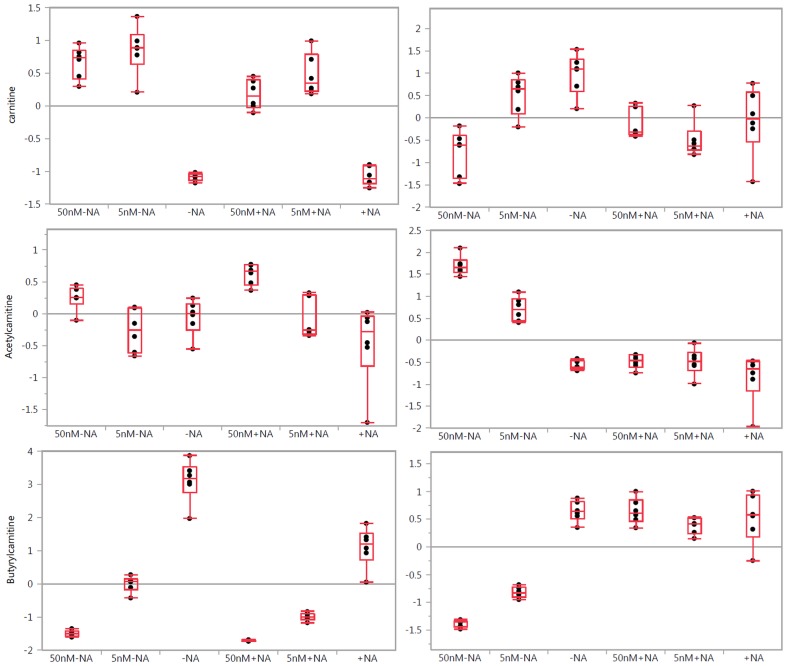
One-way analysis of variance box plots illustrating the observed level changes in the detected carnitines after FK866 treatment at 50 and 5 nM concentrations in A2780 (left panels) and HCT-116 (right panels) cells, respectively, with (+NA) and without (−NA) nicotinic acid addition. Grand mean is shown as a horizontal line located within panel. The y-axis illustrates normalized, log transformed, and scaled peak area. Dots represent samples. Group mean shown as a horizontal line within the box.

These observations might have reflected a cell line–specific difference in these particular metabolic pathways. Finally, NAMPT inhibition also significantly impacted carnitine metabolism in a dose-dependent manner (Fig. 3).

Carnitine is an important molecule for the regulation of the cellular energy metabolism of fatty acids and glucose. Carnitine is involved in long-chain fatty acid transport across the inner membrane of mitochondria, and it facilitates the transport of chain-shortened acyl groups produced in peroxisomes to the mitochondria for further energy production. Cell-specific alterations in carnitine metabolism were observed, indicating a different impact of FK866 administration on carnitine biosynthesis and degradation. Acetylcarnitine derives from acetyl-CoA, the universal degradation product of all metabolic substrates. Acetylcarnitine changes were found to be similar to acetyl-CoA changes (S2 File, S3 File, S4 File and S5 Files). Butyrylcarnitine can be derived from both fatty acids and amino acids. Its alterations were found to be dose dependent, which may reflect observed alterations in amino acids and fatty acids metabolism.

### Glycolysis, the Pentose Phosphate Pathway, and the TCA Cycle

It was recently demonstrated that NAMPT inhibition attenuates glyceraldehydes-3-phosphate dehydrogenase activity, which in turn affects glycolysis, the pentose phosphate pathway, and the TCA cycle and their downstream pathways in cancer cells [1]. The results of this study are in close agreement with those of the previous study [1] (S2–S5 Filess).

### Clustering Analysis

**Figure 4:**
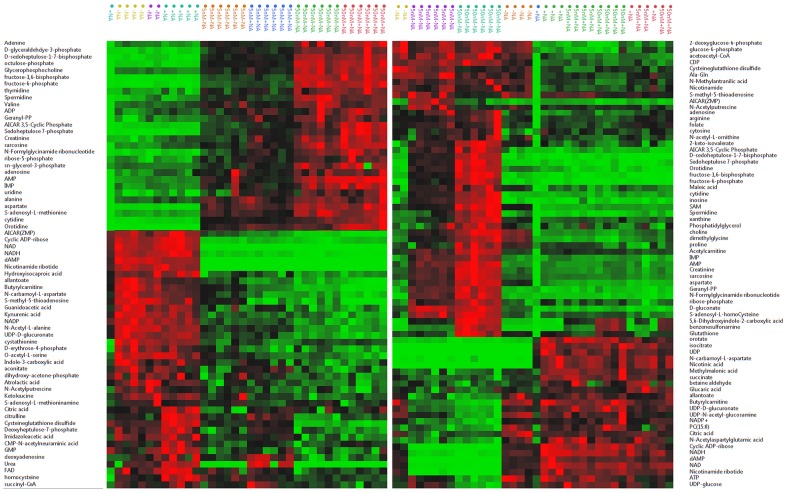
Unsupervised hierarchical clustering analysis of the metabolite levels in A2780 (left panels) and HCT-116 (right panels) cells after FK866 treatment at 50 and 5 nM concentrations, respectively, with (+NA) and without (−NA) nicotinic acid addition. Elevated metabolite levels are red and depressed ones are green. Each square represents the Pearson correlation coefficient between the metabolite in the row with the group in the column.

To further understand the metabolic connectivity and to capture the global effects, we performed an unsupervised hierarchical cluster analysis. Fig. 4 illustrates the selected metabolites heat maps. The color intensities of the maps show that the NAMPT inhibition with FK866 is cell specific and Fig. 4 shows nicotinic acid treatment. Heat maps illustrate the effects of NAMPT inhibition on different metabolic pathways in both cell lines, as reflected by the altered metabolite levels. Clustering patterns and the color intensities illustrate the impact of NAMPT inhibition after FK866 treatment on NAD biosynthesis, amino acids and purine metabolism, glycolysis, the TCA cycle, the pentose phosphate pathway, and transmethylation (Fig. 4). Full-size generated heat maps can be found in S3 File. It is also interesting to note that nicotinic acid alone differently impacted several metabolic pathways in both cell lines. In general, the HCT-116 cell line seems more impacted than A2780 with the action of nicotinic acid. This is probably because HCT-116, a NAPRT-positive cell line, can metabolize nicotinic acid for NAD biosynthesis and possibly for the other metabolites. Consistent with this assumption, we found that nicotinic acid levels in A2780 cells were more than 100-fold higher than those in HCT-116 cells.

### Pathway and Network Analysis

**Figure 5:**
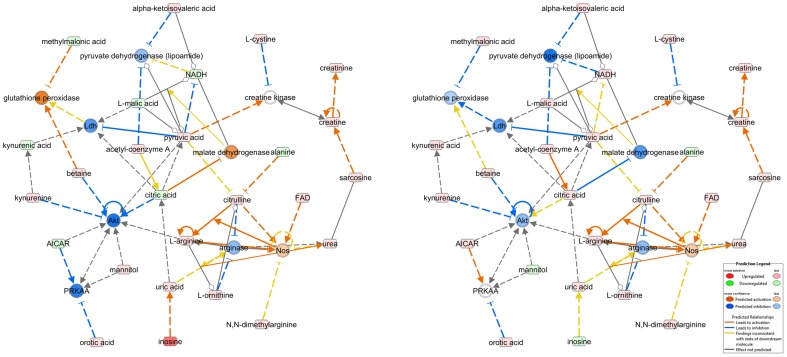
Network Analysis. Molecule activity prediction for amino acid metabolism, lipid metabolism, and molecular transport networks illustrating the treatment of HCT-116 cells with 50 nM of FK866 without nicotinic acid (left panel) and with addition of nicotinic acid (right panel). Blue nodes color reflects inhibition. Orange nodes explain activation. Color intensity depicts the degree of relative concentration for metabolites or activation for the proteins/complexes. Red metabolite nodes illustrate the elevated levels measured. Green metabolite nodes illustrate the decreased levels measured. Solid lines represent the direct relationships between nodes. Dotted lines represent indirect relationships between nodes. Red colored lines point to activation and blue to inhibition. Yellow lines indicate an inconsistent state of a downstream molecule. Ldh – lactate dehydrogenase, Akt - protein kinase B, FAD - flavin adenine dinucleotide, Nos - nitric oxide synthase, PRKAA - AMP-activated protein kinase, AICAR - 5-Aminoimidazole-4-carboxamide ribonucleotide.

To document the most significant biological functions revealed by enrichment analysis and to identify novel relationships between metabolites detected and biological entities related to observed metabolic alterations, we performed an IPA analysis of all of the metabolite alterations detected. Pathway analysis revealed the signaling and metabolic pathways and biological processes that are most significantly perturbed at treatment, such as glycolysis, gluconeogenesis, transfer ribonucleic acid charging, purine metabolism, super pathway of methionine degradation, the TCA cycle, and others. In addition, we found that FK866 treatment impacted some metabolic pathways differently in both cell lines (S3 File, S4 File and S5 Files). Network analysis identified 25 well ranked networks derived for each cell line. The highly ranked ones are those related to amino acids metabolism, lipid metabolism, molecular transport, carbohydrate metabolism, small molecule biochemistry, cell signaling, and others (S3 File). One of these networks is shown in Fig. 5. It depicts the predicted changes in enzyme activities based on the observed metabolic alterations. It is predicted that malate dehydrogenase activity should be inhibited in HCT-116 cells when treated with nicotinic acid after NAMPT inhibition with FK866, thus proposing the rescue process illustration in this particular cell line. Finally, the network analysis with IPA suggested potential links between nicotinic acid rescue action and activities of NADPH oxidase, 5' adenosine monophosphate-activated protein kinase, and other biological entities in both cell lines when NAMPT inhibition was performed with FK866 administration (S3 File).

## Discussion

In this study, we have shown that NAMPT inhibition leads to numerous metabolic perturbations in cancer cells and demonstrated resourcefulness of global metabolomics as a useful data extraction protocol for studying cellular metabolism. The profound effects of NAMPT inhibition on cancer cell metabolism could serve as a basis for further understanding of the physiological role of NAMPT in cancer cell metabolism. NAMPT seems to play a key role in maintaining cellular reduced form of nicotinamide adenine dinucleotide (NADH) levels. The important role of NAMPT in the regulation of cellular NADH levels explains its potential as a target for developing novel drugs [8], [21], [35], [36]. The metabolic basis of NAMPT inhibition was recently investigated [1]. It was reported that some cancer cells, such as A2780, only use nicotinamide for NAD biosynthesis but others, like HCT-116, can also utilize nicotinic acid via the alternative nicotinic acid phosphoribosyltransferase-mediated pathway to produce NAD [1], [8]. FK866 specificity as a NAMPT inhibitor was confirmed with addition of nicotinic acid to the growth medium that, in turn, abolished FK866 inhibitory activity against HCT-116 cells but not A2780 cells [1]. It was further demonstrated that NAMPT inhibition primarily results in the attenuation of glycolysis. This, in turn, leads to an accumulation in glycolytic intermediates before the glyceraldehyde-3-phosphate dehydrogenase step, with a corresponding decrease in intermediates after the glyceraldehyde-3-phosphate dehydrogenase step [1]. As a result, a major portion of glycolysis is significantly inhibited. Our current data are in agreement with these findings. It the current study, we demonstrated that glycolysis and pentose phosphate pathways are affected in a similar fashion, as described earlier [1] (S2 File). Similarly, we have found that NAD biosynthesis; glycolysis, pentose phosphate pathway alterations, and other metabolic perturbations are dependent on the dose of NAMPT inhibitor and can be reversed in HCT-116 cells with nicotinic acid treatment (S2 File). Therefore, the current study has confirmed the findings from our previous study [1].

We report for the first time that NAMPT inhibition leads to significant changes in nucleotide and amino acid metabolism, specifically aspartate and alanine metabolism (Figs. 1 and 4) and purine and pyrimidine metabolism (Figs. 2 and 4). Consistent with our findings, a recent study suggests that NAD depletion with the NAMPT inhibitor GNE-618, developed by Genentech, led to decreased nucleotide, lipid, and amino acid synthesis, which may have contributed to the cell cycle effects arising from NAD depletion in non-small-cell lung carcinoma cell lines [46]. It was also recently reported that phosphodiesterase 5 inhibitor Zaprinast, developed by May Baker Ltd, caused massive accumulation of aspartate at the expense of glutamate in the retina [47] when there was no aspartate in the media. On the basis of this reported event, it was proposed that Zaprinast inhibits the mitochondrial pyruvate carrier activity. As a result, pyruvate entry into the TCA cycle is attenuated. This led to increased oxaloacetate levels in the mitochondria, which in turn increased aspartate transaminase activity to generate more aspartate at the expense of glutamate [47]. In our study, we found that NAMPT inhibition attenuates glycolysis, thereby limiting pyruvate entry into the TCA cycle. This event may result in increased aspartate levels. Because aspartate is not an essential amino acid, we hypothesize that aspartate was synthesized in the cells and the attenuation of glycolysis by FK866 may have impacted the synthesis of aspartate. Consistent with that, the effects on aspartate and alanine metabolism were a result of NAMPT inhibition; these effects were abolished by nicotinic acid in HCT-116 cells but not in A2780 cells. We have found that the impact on the alanine, aspartate, and glutamate metabolism is dose dependent (Fig. 1, S3 File, S4 File and S5 Files) and cell line dependent. Interestingly, glutamine levels were not significantly affected with these treatments (S4 File and S5 Files), suggesting that it may not be the particular case described for the impact of Zaprinast on the amino acids metabolism. Network analysis, performed with IPA, strongly suggests that nicotinic acid treatment can also alter amino acid metabolism. For example, malate dehydrogenase activity is predicted to be elevated in HCT-116 cells treated with FK866 but suppressed when HCT-116 cells are treated with nicotinic acid (Fig. 5). Network analysis connected malate dehydrogenase activity with changes in the levels of malate, citrate, and NADH. This offers a correlation with the observed aspartate level changes in our study. The impact of FK866 on alanine, aspartate, and glutamate metabolism on A2780 cells is found to be different from HCT-116 cells. Observed changes in alanine and N-carbamoyl-L-aspartate levels suggest different activities of aspartate 4-decarboxylase and aspartate carbamoyl transferase in the investigated cell lines (Fig. 5). However, the levels of glutamine, asparagine, gamma-aminobutyric acid (GABA), and glutamate were not significantly altered (S4 File and S5 Files), which suggests corresponding enzymes activity tolerance to the applied treatments. Impact on methionine metabolism was found to be similar to aspartate and alanine metabolism, showing dose-dependent metabolic alterations in methionine SAM, SAH, and S-methyl-5′-thioadenosine levels that were abolished with nicotinic acid treatment in HCT-116 cells but not in A2780 cells (Fig. 1, S2 File, S3 File, S4 File and S5 Files). We hypothesize that those amino acids alterations, which can be abolished with nicotinic acid, are NAMPT dependent directly or indirectly through the intercellular metabolic networks.

**Figure 6:**
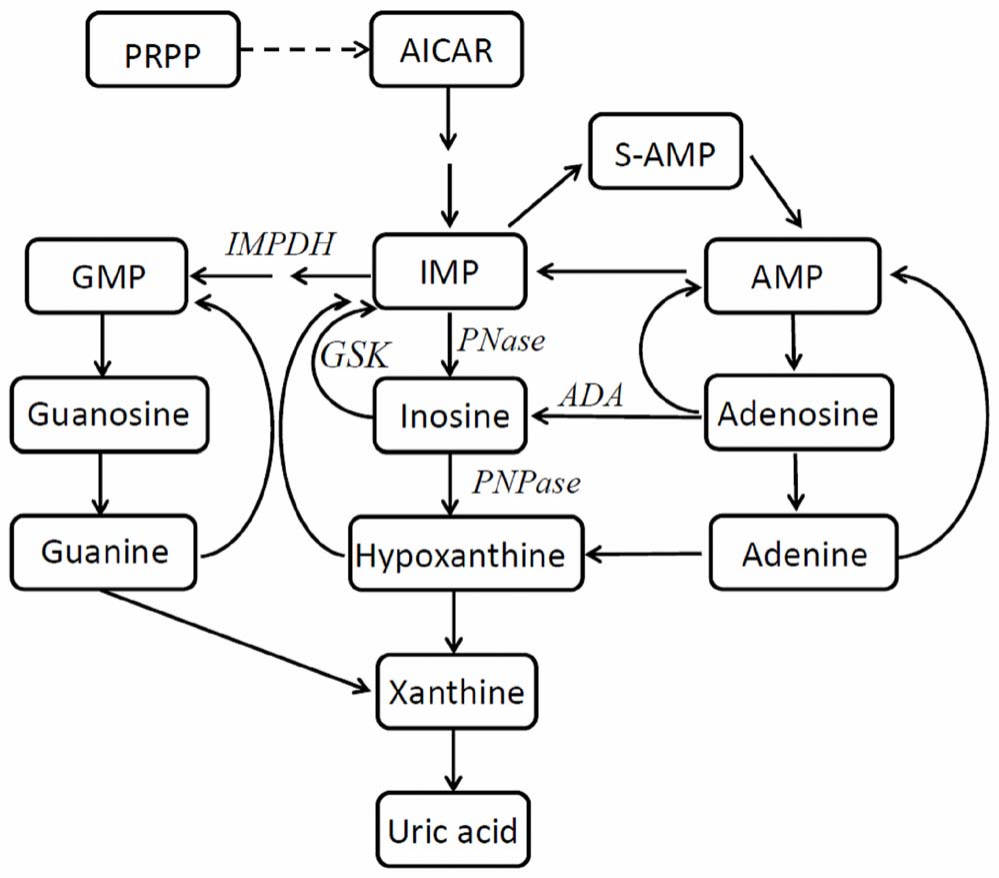
Purine metabolism scheme. ADA, adenosine deaminase; AICAR, 5-aminoimidazole-4-carboxamide ribonucleotide; AMP, adenosine monophosphate; GMP, guanosine monophosphate; GSK, inosine kinase; IMP, inosine monophosphate; IMPDH, inosine 5′-monophosphate dehydrogenase; PNase, purine 5′-nucleotidase; PNPase, purine-nucleoside phosphorylase; PRPP, 5-phospho-α-ribosyl-1-pyrophosphate; S-AMP, succinyl adenosine monophosphate.

We found that NAMPT inhibition significantly alters purine biosynthesis, exemplified by the increased IMP levels. This particular increase was probably due to the inhibition of IMP dehydrogenase (IMPDH) resulting from NAD depletion as this enzyme requires NAD for activity. We observed similar patterns of accumulation for adenosine, adenine, AMP, and ADP. These accumulations can be explained with the suppression of NAD biosynthesis, which can be reversed in HCT-116 cells with the addition of nicotinic acid (Fig. 2, S2 File, S3 File, S4 File and S5 Files). A similar pattern of change in other purine and pyrimidine levels indicates purine/pyrimidine biosynthesis connections with NAMPT activity. In support of this hypothesis, we offer observed a dose-dependent decrease in GMP levels that was restored with nicotinic acid treatment in HCT166 cells but not in A2780 cells. Interestingly, the strong inosine accumulation in both cells is abolished with nicotinic acid as well. Synthesis of the purine nucleotides begins with 5-phospho-α-ribosyl-1-pyrophosphate (PRPP) and leads to the first fully formed nucleotide: IMP (Fig. 2 and 6). Through a series of reactions using ATP, tetrahydrofolate derivatives, glutamine, glycine, and aspartate, this pathway yields IMP, which represents a branch point for purine biosynthesis because it can be converted into either AMP or GMP through two distinct reaction pathways. One of the branches was controlled by IMPDH with the cofactor of NAD. We observed that the alterations in the levels of IMP are due to the depletion of NAD followed by the blockage of IMPDH. Catabolism of purine nucleotides ultimately leads to the production of uric acid, but those levels were unchanged. Its precursor, xanthine, has an elevated level in response to FK866 treatment only in HCT-116 cells. This was abolished with nicotinic acid treatment (S4 File and S5 Files). This observation is in alignment with the cell-specific response to drug treatments. In this study, the abnormal inosine accumulation observed upstream to hypoxanthine could be explained with upregulated activity of cytosolic purine 5′-nucleotidase, adenosine deaminase, or both, or, more logically, with the selective inhibition of inosine kinase or purine-nucleoside phosphorylase with FK866 (Fig. 6). However, the nicotinic acid rescue effect suggests the involvement of NAMPT in the regulation of this chain of events. However, it is unclear how inosine levels could be regulated with nicotinic acid action in A2780 cells. The impact of nicotinic acid supplementation was further investigated by comparison of metabolic changes in both cell lines without NAMPT inhibition, illustrating the absence of the impact of nicotinic acid administration on inosine levels (Fig. 2, (S2 File, S3 File, S4 File and S5 Files). This makes the alternative hypothesis of NAMPT activity involvement through metabolic networks relevant to the observed phenomena.

We found that fatty acids metabolism and lipid metabolism were significantly affected as well (Fig. 3, S3 File, S4 File and S5 Files). We observed changes in fatty acids transport that utilizes carnitine. Significantly impacted carnitine metabolism illustrates a cell-specific response to the treatments (Fig. 3). The dose-dependent response of the changes in carnitine levels to NAMPT inhibition is different in two cell lines, as is the response to nicotinic acid. An increase in carnitine levels in A2780 cells due to NAMPT inhibition may be explained by the observed upregulated levels of lysine and methionine, which are precursors in carnitine biosynthesis. However, a dose-dependent decrease in carnitine levels in HCT-116 cells in response to NAMPT inhibition may require an alternative hypothesis based on the cell-specific alteration of metabolic pathways. Acylcarnitines were found to respond to FK866 treatment in a similar fashion for both cell lines. Significantly decreased levels of butyrylcarnitine in a dose-dependent fashion (Fig. 3) suggest a NAMPT inhibition effect on *de novo* fatty acid synthesis, which was earlier reported to be reduced in different cancer cells when treated with FK866 [48]. In our study, the observed effects were abolished with the addition of nicotinic acid to the growth medium in HCT-116 cells but not in A2780 cells (Fig. 3). Beta-oxidation requires NAD and flavin adenine dinucleotide (FAD) participation after activation and transport are provided. Therefore, fatty acids oxidation is strongly influenced by NAMPT inhibition. Interestingly, FAD levels were decreased in a dose-response fashion at NAMPT inhibition and did not respond to nicotinic acid treatment in A2780 cells. FAD levels were elevated in a dose-response manner at NAMPT inhibition and responded to nicotinic acid treatment in HCT-116 cells (S3 File, S4 File and S5 Files). FAD participates in the formation of succinate from fumarate as well. We observed similar trends in succinate level alterations (S2 File, S3 File, S4 File and S5 Files), which again suggested cell-specific responses to applied treatments. Fatty acid biosynthesis is NADPH dependent. We observed elevated levels of palmitic and stearic acids in a dose-dependent manner in both cell lines. Interestingly, the addition of nicotinic acid abolished these effects in both cell lines (S3 File, S4 File and S5 Files). This non-specific response to nicotinic acid treatment requires an alternative hypothesis. Increased levels of choline, phosphorylcholine, and phoshatydylglycerol in HCT-116 cells are associated with NAMT inhibition. We observed a strong dose-dependent increase in glycerophosphocholine and glycerol-3-phosphate levels and a dose-dependent decrease in the levels of detected lyso-PC and PC only in A2780 cells, suggesting a cell-specific response of lipid metabolism to NAMPT inhibition with FK866 (S2 File, S3 File, S4 File and S5 Files). The impact of the NAMPT inhibition on lipogenesis in cancer cell lines was previously reported [48]. In our study, we observed a similar impact on PC metabolism neutralized with the nicotinic acid treatment.

In general, the results of the pathway analysis of the metabolic alterations due to FK866 action with and without nicotinic acid treatment suggested that HCT-116 cells respond to nicotinic acid treatment with an upregulation of the various metabolic pathways showing how compensation of FK866 action impacted cellular metabolism. That is not the case for a majority of metabolic pathways of A2780 cells (S3 File, S4 File and S5 Files). It was found that nicotinic acid treatment did not reverse NAMPT inhibition induced with FK866 action in A2780 cells. Glycolysis, gluconeogenesis, the TCA cycle, urea cycle, NAD salvation pathway, adenine and adenosine salvage pathway, and the others were upregulated at nicotinic acid action, therefore compensating the NAMPT inhibition in HCT-116 cells treated with FK866 in comparison to A2780 cells treated with FK866 (S3 File). Network analysis allowed for suggesting a hypothesis that connects the metabolites and enzymes/complexes involved in metabolism. Fig. 5 illustrates predicted changes in enzyme activities when HCT-116 cells were treated with FK866 (left panel) and with FK866 at the presence of nicotinic acid (right panel). Measured levels of metabolites were the basis for the computer-assisted network analysis and predictions implying transformations and enzymes participation/activity described in the reviewed literature (http://www.ingenuity.com/science/knowledge-base). Projected reduced activity of malate dehydrogenase (blue node on the right panel; Fig. 5), activated beforehand as an outcome of NAMPT inhibition with FK866, is found to be directly associated with the elevated levels of NADH, citrate, and malate (red nodes on the right panel in comparison to green nodes on the left panel; Fig. 5), which in turn are the results of nicotinic acid administration. A nicotinic acid induced change in levels of methylmalonic acid predicts the inhibition of glutathione peroxidase activated as a result of NAMPT inhibition with FK866 (Fig. 5). Network analysis also proposed a scenario of the rescue with nicotinic acid administration for the HCT-116 cell line, which is a NAPRT-positive cell line that can use both NAMPT-mediated nicotinamide and NAPRT-mediated nicotinic acid pathways for NAD biosynthesis. It was proposed that by using MAP protocol, the nicotinic acid treatment should induce the activation of NADPH oxidase and the deactivation of AMPK activity. In contrast to these projections for HCT-116 cells, nicotinic acid action on A2780 cells, which lack NAPRT, proposed no changes in NADPH oxidase and AMPK activity. Activities of NADPH oxidase are known to be dependent on NADH and NADPH, for which the levels were shown to be depleted in both cell lines when treated with FK866 and rescued to the levels of control cells upon addition of nicotinic acid only in the HCT-116 cell line (S2 File, S3 File, S4 File and S5 Files). (Therefore, the network analysis results supported this observation. It is known that the activation of AMPK, a master regulator of energy homeostasis, is initiated through increased AMP/ATP and ADP/ATP ratios, which we found were affected with NAMPT inhibition in both cell lines. We have found that the level of ATP was decreased in a dose-dependent manner in the HCT-116 cell line when treated with FK866. However, the levels of AMP and ADP were increased in a dose-dependent manner, increasing the AMP/ATP and ADP/ATP ratios (S3 File and S4 File). Nicotinic acid action abolished this alteration, suggesting NAMPT involvement in AMPK regulation through metabolic networking. We found that the level of ATP was moderately increased in the A2780 cell line in response to FK866 treatment. However, the levels of AMP and ADP were increased as well in a dose-dependent manner, thus keeping the AMP/ATP and ADP/ATP ratios at almost similar levels. Nicotinic acid action did not abolish this alteration, except for ATP levels (S3 File, S4 File and S5 Files). Therefore, when activated with NAMPT inhibition, AMPK was not proposed inhibited when treated with nicotinic acid. This explains the IPA MAP prediction for both cell lines. IPA-assisted generated hypotheses would require experimental confirmation utilizing appropriate assays, which we plan to perform in the future.

The results of this study support knowledge about an important role of NAMPT in cancer cells' proliferation and its attractiveness as anticancer target for new, more specific, and less toxic drug molecules. The high dependence of cancer cells' viability on NAD levels makes tumor cells more susceptible to NAMPT inhibition than normal cells. It is reported that nicotinic acid coadministration with FK866 and GMX1777, both nicotinamide phosphoribosyltransferase inhibitors, rescues toxicity associated with NAMPT inhibition and enhances the therapeutic index [6], [8]. For the possibility of using nicotinic acid as a chemoprotectant in high-dose treatment with NAMPT inhibitors, it is important to discriminate positive and negative reactivity for NAPRT and cell-specific responses to treatments. NAPRT expression was absent in cell lines, which were not protected by nicotinic acid from toxicity induced with NAMPT inhibitors administration. In this study, we demonstrated cell-specific metabolic responses to FK866 treatment at 50 and 5 nM concentrations for 24 hours and ability to recuperate with nicotinic acid treatment for human cancer cells having NAPRT pathway available. To the authors' knowledge, this is the first report of significant alterations in amino acids metabolism and purine and pyrimidine metabolism induced with FK866 treatment in tested cell lines. We have also demonstrated NAMPT involvement in the regulation of the majority of the reported impacted metabolic pathways. We showed that nicotinic acid's impact on cells' metabolism and the metabolic alterations observed within this study is more complicated than only feeding NAPRT for upregulation of NADH biosynthesis. It is illustrated with the metabolic alterations of purine and pyrimidine metabolism, as well as and the fatty acids metabolism and transport, detected in this study. Taking into account the great complexity of drugs' impact on metabolism, even in the controlled environment of cell-based studies, we suggest that global metabolomics can be a useful tool in a pharmacological drug action study at the cellular level. The described metabolic alterations resulting from FK866 action could be used as potential pharmacodynamic markers for the evaluation of NAMPT inhibitors in the clinic. This could assist in pursuing a tailored approach in anticancer drug development and patient's segregation.

## Supporting Information

S1 File **Analytical performance of standards, quality control calibration curves.**
(XLSX)

S2 File **Results on nicotinamide adenine dinucleotidebiosynthesis, adenosine triphosphate depletion, glycolysis, pentose phosphate pathway, TCA cycle, and serine biosynthesis.**
(DOCX)

S3 File **Analytical performance of detected metabolites.** Multivariate analysis results. Pathway analysis results. Network analysis results.
(XLSX)

S4 File **A2780 one-way analysis of variance results.**
(PDF)

S5 File **HCT-116 one-way analysis of variance results.**
(PDF)
