# Supplementary material for: Metabolomics Analysis of Metabolic Effects of Nicotinamide Phosphoribosyltransferase (NAMPT) Inhibition on Human Cancer Cells
Source: PLoS One. 2014 Dec 8;9(12):e114019. doi: 10.1371/journal.pone.0114019 (PMC4259317; doi:10.1371/journal.pone.0114019)
Supplement: S2 File — Results on nicotinamide adenine dinucleotidebiosynthesis, adenosine triphosphate depletion, glycolysis, pentose phosphate pathway, TCA cycle, and serine biosynthesis. (DOCX) [file pone.0114019.s002.docx]

*Nicotinamide adenine dinucleotide (NAD) Biosynthesis*

It was previously shown that FK866 inhibited NAD biosynthesis in both cell lines with the IC50 values of 0.5 and 1.4 nM for A2780 and 0.5 and 3.0 nM for HCT116, respectively [1]. It was also shown that the addition of nicotinic acid to the growth medium can reverse FK866 inhibitory activity against HCT116 cells but not A2780 cells [2]. Our metabolomics study confirmed this observation. Changes in the levels of NAD and the related detected metabolites are illustrated in Supplementary Figure 1. Depletion of NAD, reduced nicotinamide adenine dinucleotide (NADH), and nicotinamide adenine dinucleotide phosphate (NADP) at nicotinamide phosphoribosyltransferase (NAMPT) inhibition with FK866 can be reversed with the addition of nicotinic acid for HCT116 cells but for A2780 cells. The same changes in levels of nicotinamide mononucleotide (NMN), cyclic adenosine aiphosphate ribose, uridine diphosphoglucuronic acid, and uridine diphospho n-acetyl glucosamine were observed (Supplemental Material S3-S5).


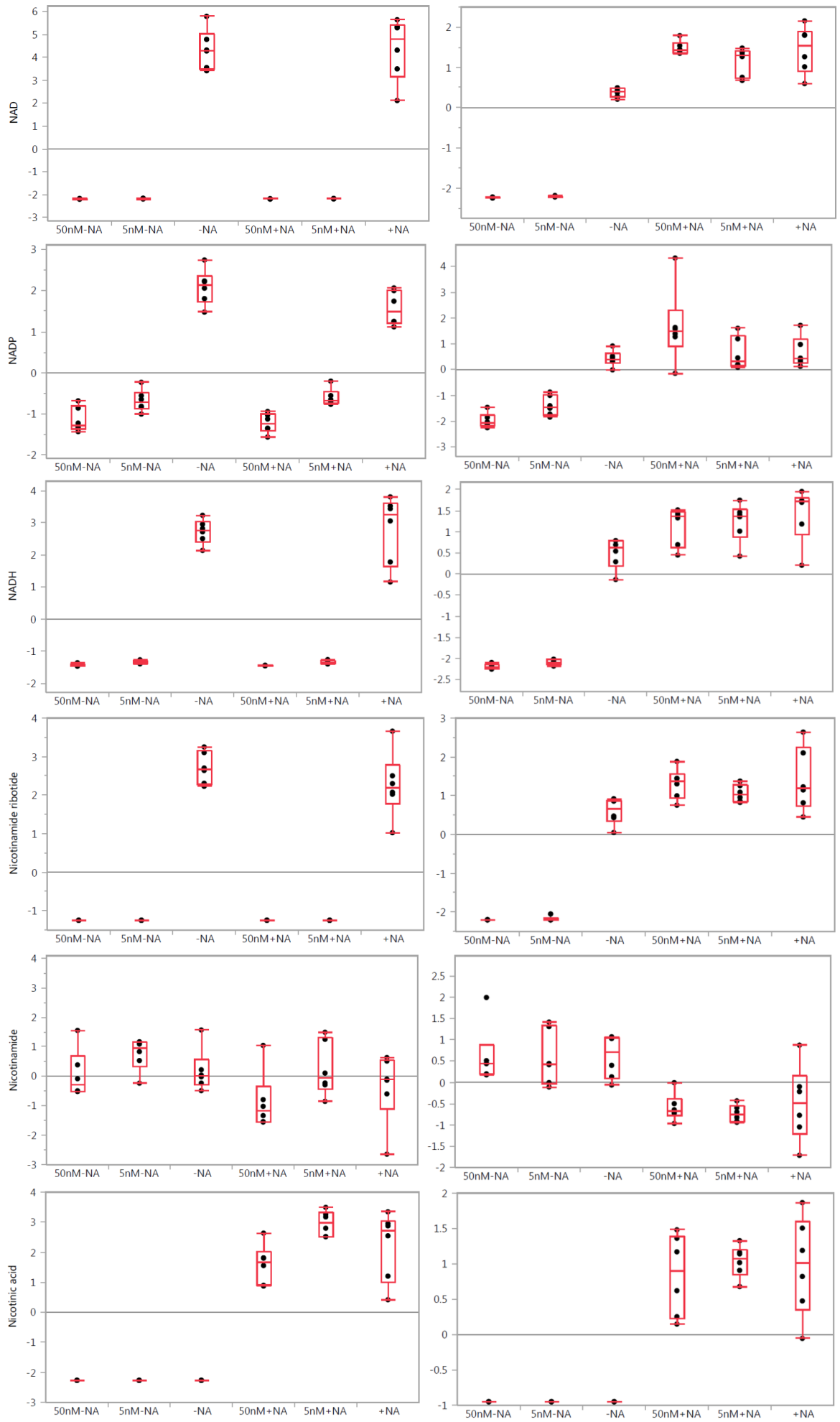


Supplementary Figure 1. One-way analysis of variance box plots illustrating the observed level changes of NAD, NADP, NADH, NMN, nicotinamide, and nicotinic acid levels after FK866 treatment at 50 and 5 nM concentrations in A2780 (left panels) and HCT116 (right panels) cells, respectively, with (+NA) and without (–NA) nicotinic acid addition. Grand mean is shown as a horizontal line located within panel. The y-axis illustrates the normalized, log transformed, and scaled peak area. Dots represent samples. Group mean is shown as a horizontal line within the box.

*Adenosine Triphosphate (ATP) Alterations*

It was reported that FK866 can induce a delayed cell death of A2780 human cancer cells at 100 nM concentration after 60 hours of exposure [2,3]. It was thought to be associated with a delayed ATP level decrease in cancer cells. ATP and NAD levels were reported to decrease 40 hours after treatment [2]. Our study showed that in A2780 cells, the ATP levels were not depleted within 24 hours. However, in HCT116 cells, the ATP levels were reduced after treatment with FK866 and observed changes in the ATP levels were alleviated with the addition of nicotinic acid (Supplementary Figure 2), indicating that the HCT116 cells are more sensitive than the A2780 cells. Interestingly, a slight increase of ATP levels was observed in A2780 cells in response to treatment with FK866. Nicotinic acid treatment abolished this effect. This observation confirms a cell-specific response to the applied treatments.


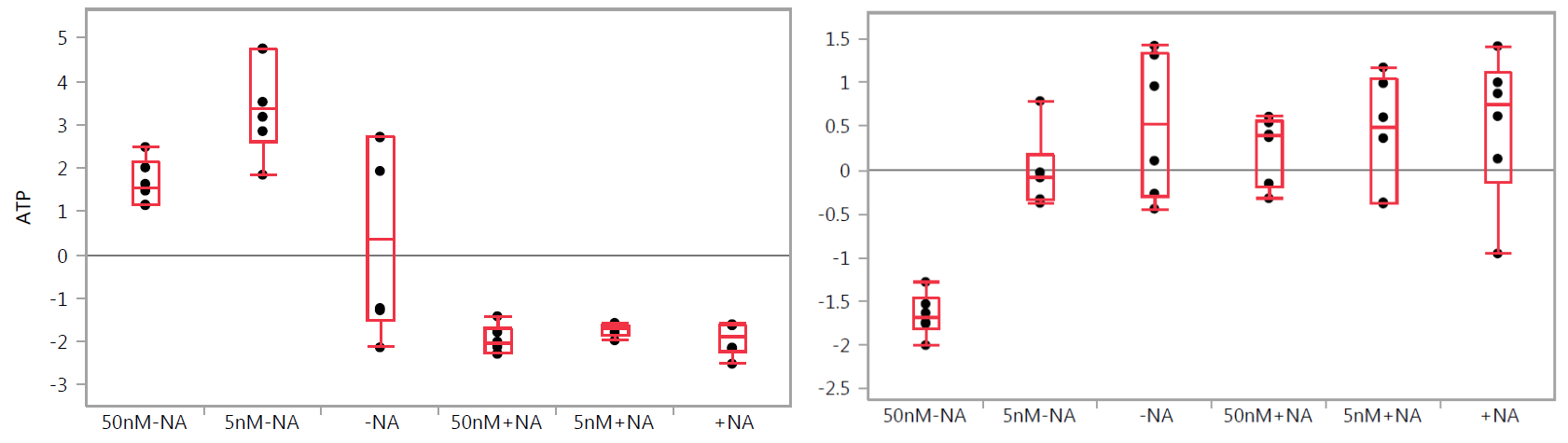


Supplementary Figure 2. One-way analysis of variance box plots illustrating observed changes of ATP levels after FK866 treatment at 50 and 5 nM concentrations in A2780 (left panel) and HCT116 (right panel) cells, respectively, with (+NA) and without (–NA) nicotinic acid addition. Grand mean is shown as a horizontal line located within panel. The y-axis illustrates the normalized, log transformed, and scaled peak area. Dots represent samples. Group mean is shown as a horizontal line within the box.

*Glycolysis*

It was recently demonstrated that NAMPT inhibition attenuates glyceraldehydes-3-phosphate dehydrogenase activity, which in turn affects glycolysis and its downstream pathways in cancer cells [1]. We observed a significant increase in fructose monophosphate, fructose 1,6-bisphosphate, and glyceraldehydes-3-phosphate levels and a decrease in 1,3-bisphosphoglycerate. The latter was dose dependent in A2780 cells and showed no significant changes in HCT116 cells. The levels of glucose-6-phosphate and 3-phosphoglycerate were not significantly changed, and no marked changes were observed in phosphoenolpyruvate levels (Supplemental Material S4 and S5). The addition of nicotinic acid completely abolished the observed effects in HCT116 cells but not in A2780 cells (Supplementary Figure 3).


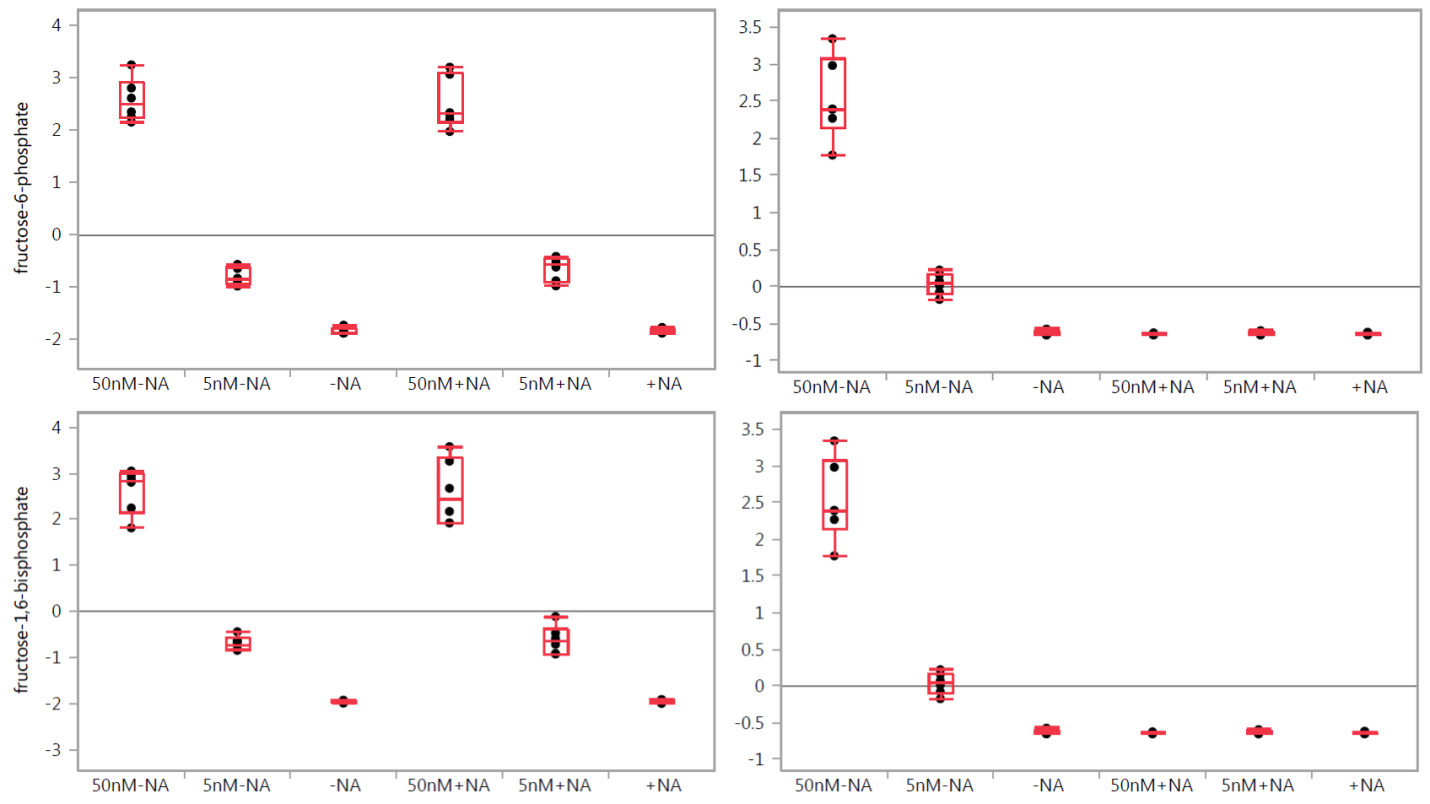


Supplementary Figure 3. One-way analysis of variance box plots illustrating the observed level changes of fructose monophosphate and fructose 1,6-bisphosphate levels after FK866 treatment at 50 and 5 nM concentrations in A2780 (left panel) and HCT116 (right panel) cells, respectively, with (+NA) and without (–NA) nicotinic acid addition. Grand mean is shown as a horizontal line located within panel. The y-axis illustrates the normalized, log transformed, and scaled peak area. Dots represent samples. Group mean is shown as a horizontal line within the box.

*Pentose Phosphate Pathway*

It is known that many cancer cells rely on glycolysis for ATP production to fuel their rapid growth and proliferation [4,5]. We observed an alteration in the levels of metabolites involved in the pentose phosphate pathway (PPP) with treatment cells with FK866. Levels of deoxysedoheptulose 7-phosphate were decreased in a dose-dependent manner in both cell lines. The levels of deoxyribose 5-phosphate were not significantly changed in either cell line. PPP upregulation due to NAMPT inhibition is consistent with the build-up of glycolytic metabolites, which serve as substrates for the PPP shunt. Levels of ribose-5-phosphate, sedoheptulose monophosphate, sedoheptulose 1, 7-bisphosphate, and octulose monophosphate were significantly increased in a dose-dependent manner in both cell lines. The addition of nicotinic acid abolished these effects in HCT116 cells but not in A2780 cells (Supplementary Figure 4).


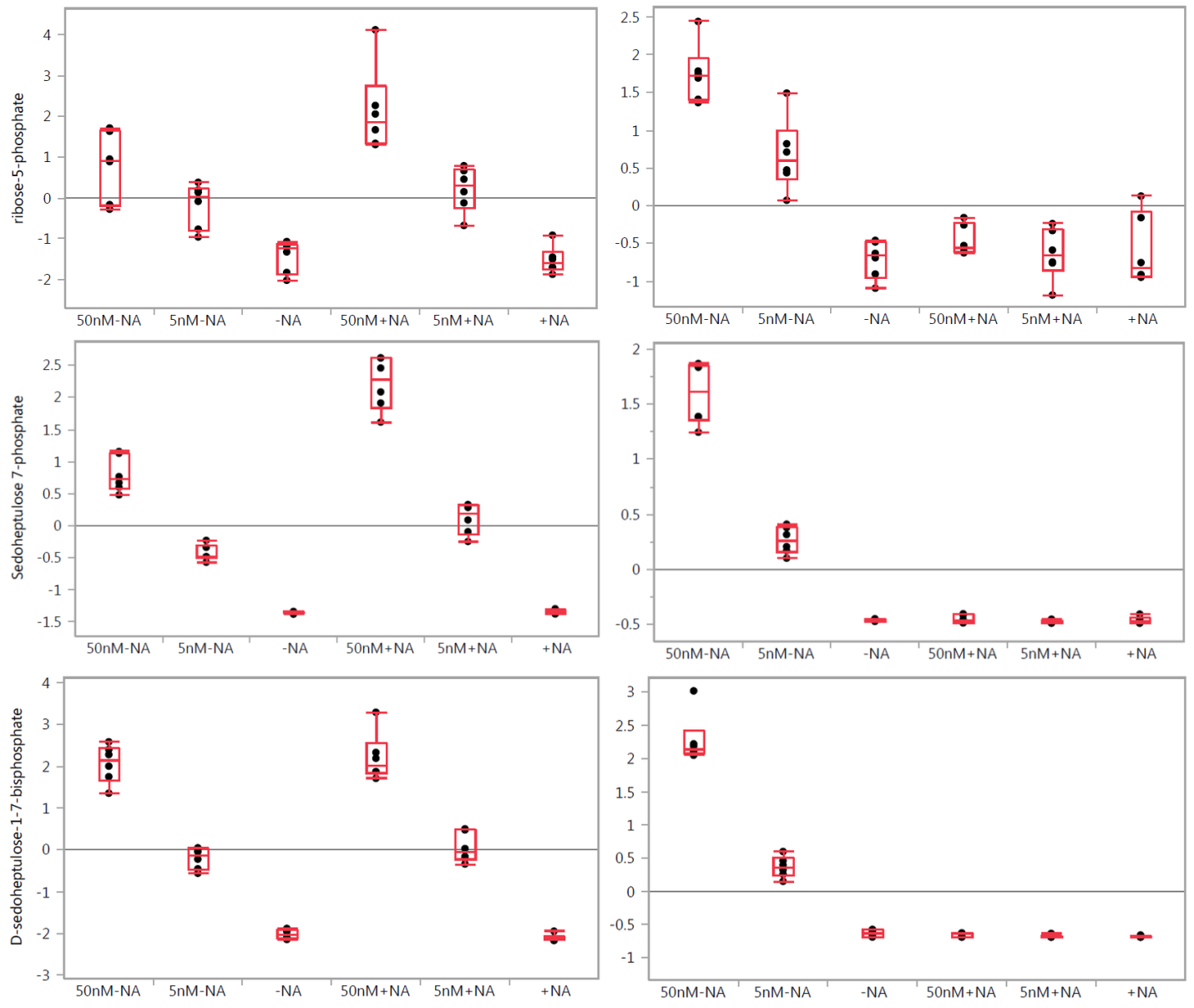


Supplementary Figure 4. One-way analysis of variance box plots illustrating the observed level changes of ribose-5-phosphate, sedoheptulose monophosphate, and sedoheptulose 1,7-bisphosphate after FK866 treatment at 50 and 5 nM concentrations in A2780 (left panel) and HCT116 (right panel) cells, respectively, with (+NA) and without (–NA) nicotinic acid addition. Grand mean is shown as a horizontal line located within panel. The y-axis illustrates the normalized, log transformed, and scaled peak area. Dots represent samples. Group mean is shown as a horizontal line within the box.

*Citric Acid Cycle(TCA)*

NAMPT inhibition affects glycolysis, which in turn could reduce pyruvate supply to the TCA cycle. The conversion of pyruvate to acetyl-CoA represents the first step in the entry of pyruvate into the TCA cycle, in which multiple steps require NAD participation. We observed a decrease in citrate levels more pronounced in HCT116 cells when treated with FK866. A dose-dependent decrease in aconitate, fumarate, and malate levels was observed in A2780 cells (Supplemental material S4, S5). A decrease in citrate, isocitrate, and succinate levels was observed in HCT116 cells, whereas this effect was abolished at the presence of nicotinic acid (Supplementary Figure 5). Acetyl-CoA levels were decreased in A2780 cells and increased in a dose-dependent manner in HCT116 cells. This increase was abolished by the addition of nicotinic acid. Pyruvate levels were not changed in HCT116 cells but were decreased in a dose-dependent manner in A2780 cells (Supplemental Figure 5). These observations illustrate a cell-specific response to the applied treatments.


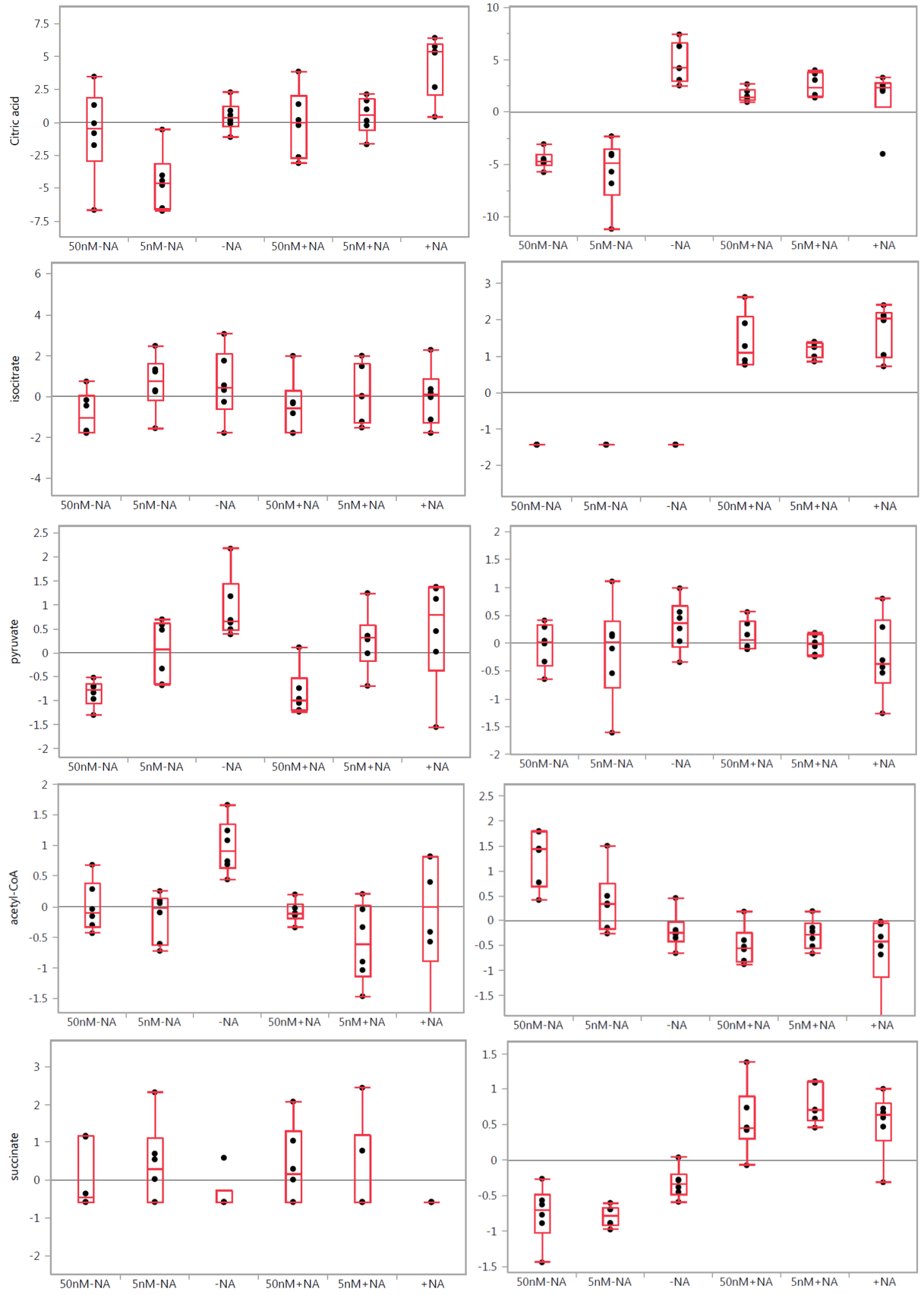


Supplementary Figure 5. One-way analysis of variance plots illustrating the observed metabolic alterations in TCA cycle after FK866 treatment at 50 and 5 nM concentrations in A2780 (left panels) and HCT116 (right panels) cells, respectively, with (+NA) and without (–NA) nicotinic acid addition. Grand mean is shown as a horizontal line located within panel. The y-axis illustrates the normalized, log transformed, and scaled peak area. Dots represent samples. Group mean is shown as a horizontal line within the box.

*Serine Biosynthesis*

Levels of ketoglutarate were elevated in A2780 cells but not in HCT116 cells (Supplementary Figure 5). Levels of glycine and serine were not significantly changed in either cell line. Phosphoserine levels were marginally elevated in both cell lines. The addition of nicotinic acid abolished these effects in HCT116 cells but not in A2780 cells (Supplemental material S3-S5.). Interestingly, threonine levels were elevated in response to FK866 treatment in both cell lines (Supplementary Figure 6). This effect was neutralized with nicotinic acid treatment, supporting NAMPT dependence of threonine biosynthesis starting from aspartate.


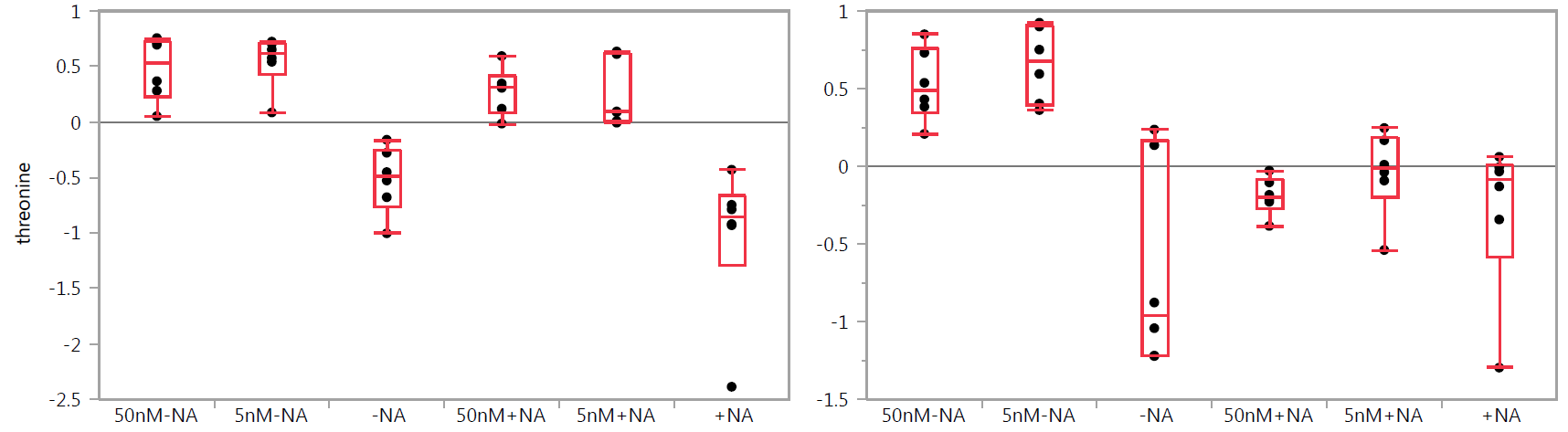


Supplementary Figure 6. One-way analysis of variance plots illustrating the observed changes in threonine levels after FK866 treatment at 50 and 5 nM concentrations in A2780 (left panel) and HCT116 (right panel) cells, respectively, with (+NA) and without (–NA) nicotinic acid addition. Grand mean is shown as a horizontal line located within panel. The y-axis illustrates the normalized, log transformed, and scaled peak area. Dots represent samples. Group mean is shown as a horizontal line within the box.

**References**

1. Watson M, Roulston A, Bélec L, Billot X, et al. (2009) The small molecule GMX1778 is a potent inhibitor of NAD+ biosynthesis: strategy for enhanced therapy in nicotinic acid phosphoribosyltransferase 1-deficient tumors. Mol Cell Biol 29: 5872-5888.
2. Tan B, Young DA, Lu ZH, Wang T, Meier TI, et al. (2013) Pharmacological inhibition of nicotinamide phosphoribosyltransferase (NAMPT), an enzyme essential for NAD biosynthesis, in human cancer cells: metabolic basis and potential clinical implications. J Biol Chem 288: 3500-3511.
3. Hasmann M, Schemainda I (2003) FK866, a highly specific noncompetitive inhibitor of nicotinamide phosphoribosyltransferase, represents a novel mechanism for induction of tumor cell apoptosis. Cancer Res 63: 7436-7442.
4. Olesen UH, Thougaard AV, Jensen PB, Sehested M (2010) A preclinical study on the rescue of normal tissue by nicotinic acid in high dose treatment with APO866, a specific nicotinamide phosphoribosyltransferase inhibitor. Mol Cancer Ther 9: 1609-1617.
5. Busso N, Karababa M, Nobile M, Rolaz A, Van Gool F, et al. (2008) Pharmacological inhibition of nicotinamide phosphoribosyltransferase/visfatin enzymatic activity identifies a new inflammatory pathway linked to NAD. PLoS One 3: e2267.
