# Supplementary material for: Metabolomics Analysis of Metabolic Effects of Nicotinamide Phosphoribosyltransferase (NAMPT) Inhibition on Human Cancer Cells
Source: PLoS One. 2014 Dec 8;9(12):e114019. doi: 10.1371/journal.pone.0114019 (PMC4259317; doi:10.1371/journal.pone.0114019)
Supplement: S4 File — A2780 one-way analysis of variance results. (PDF) [file pone.0114019.s004.pdf]

# Oneway Analysis of 1-(5Z,8Z,11Z,14Z-eicosatetraenoyl)-sn-glycero-3-phosphocholine By Labe

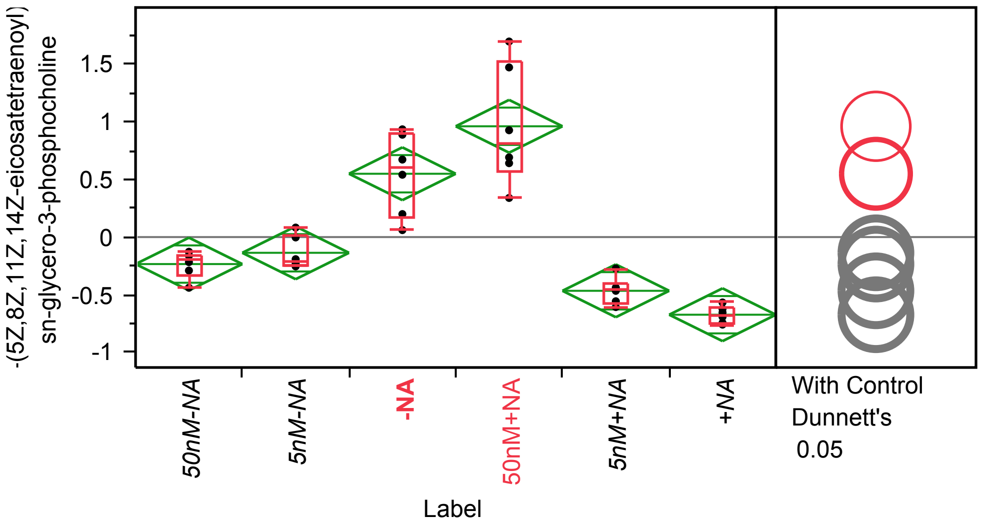


## Quantiles

| Level | Minimum | 10% | 25% | Median | 75% | 90% | Maximum |
|---|---|---|---|---|---|---|---|
| 50nM-NA | -0.44019 | -0.44019 | -0.32821 | -0.19323 | -0.15345 | -0.12762 | -0.12762 |
| 5nM-NA | -0.25461 | -0.25461 | -0.23942 | -0.20512 | 0.018235 | 0.082412 | 0.082412 |
| -NA | 0.060494 | 0.060494 | 0.164884 | 0.607386 | 0.900878 | 0.93608 | 0.93608 |
| 50nM+NA | 0.339986 | 0.339986 | 0.566507 | 0.810113 | 1.530201 | 1.698774 | 1.698774 |
| 5nM+NA | -0.60816 | -0.60816 | -0.57031 | -0.46121 | -0.39766 | -0.27322 | -0.27322 |
| +NA | -0.76048 | -0.76048 | -0.74889 | -0.67463 | -0.61032 | -0.56831 | -0.56831 |

## Oneway Anova

### Summary of Fit

| | |
|---|---|
| Rsquare | 0.839325 |
| Adj Rsquare | 0.812546 |
| Root Mean Square Error | 0.275021 |
| Mean of Response | 2.47e-17 |
| Observations (or Sum Wgts) | 36 |

### Analysis of Variance

| Source | DF | Sum of Squares | Mean Square | F Ratio | Prob > F |
|---|---|---|---|---|---|
| Label | 5 | 11.853238 | 2.37065 | 31.3425 | <.0001 * |
| Error | 30 | 2.269103 | 0.07564 | | |
| C. Total | 35 | 14.122341 | | | |

### Means for Oneway Anova

| Level | Number | Mean | Std Error | Lower 95% | Upper 95% |
|---|---|---|---|---|---|
| 50nM-NA | 6 | -0.23453 | 0.11228 | -0.4638 | -0.0052 |
| 5nM-NA | 6 | -0.13666 | 0.11228 | -0.3660 | 0.093 |
| -NA | 6 | 0.55003 | 0.11228 | 0.3207 | 0.779 |
| 50nM+NA | 6 | 0.96250 | 0.11228 | 0.7332 | 1.192 |
| 5nM+NA | 6 | -0.46677 | 0.11228 | -0.6961 | -0.237 |
| +NA | 6 | -0.67457 | 0.11228 | -0.9039 | -0.445 |

Std Error uses a pooled estimate of error variance

## Means Comparisons

### Comparisons with a control using Dunnett's Method

## Oneway Analysis of 1,3-diphopshateglycerate By Label

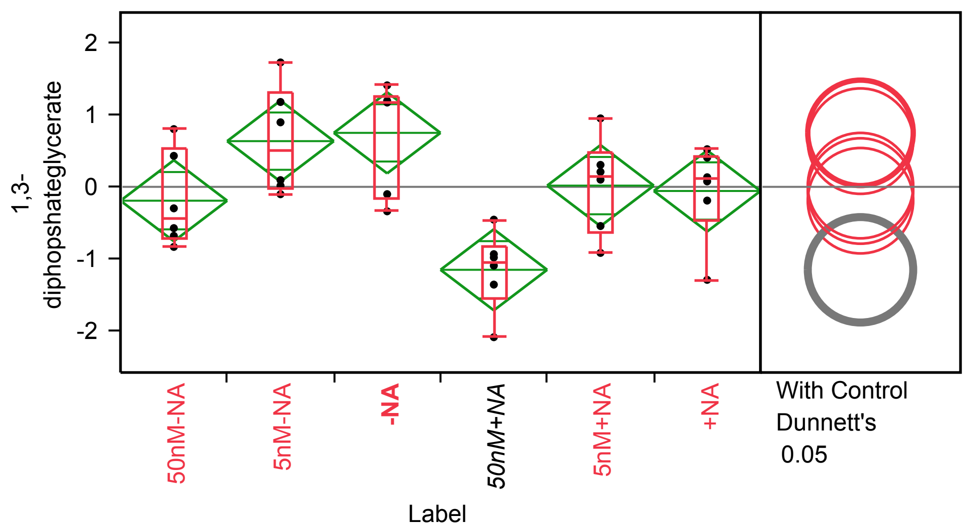


### Quantiles

| Level | Minimum | 10% | 25% | Median | 75% | 90% | Maximum |
|---|---|---|---|---|---|---|---|
| 50nM-NA | -0.83522 | -0.83522 | -0.7202 | -0.43644 | 0.523987 | 0.803017 | 0.803017 |
| 5nM-NA | -0.10242 | -0.10242 | -0.01339 | 0.49556 | 1.317685 | 1.729966 | 1.729966 |
| -NA | -0.34009 | -0.34009 | -0.16123 | 1.169604 | 1.250151 | 1.411665 | 1.411665 |
| 50nM+NA | -2.09097 | -2.09097 | -1.54247 | -1.03944 | -0.81622 | -0.45671 | -0.45671 |
| 5nM+NA | -0.91848 | -0.91848 | -0.6383 | 0.154178 | 0.467099 | 0.952202 | 0.952202 |
| +NA | -1.29328 | -1.29328 | -0.46655 | 0.104647 | 0.435666 | 0.523541 | 0.523541 |

### Oneway Anova

#### Summary of Fit

| | |
|---|---|
| Rsquare | 0.50598 |
| Adj Rsquare | 0.423643 |
| Root Mean Square Error | 0.675966 |
| Mean of Response | 2.78e-10 |
| Observations (or Sum Wgts) | 36 |

#### Analysis of Variance

| Source | DF | Sum of Squares | Mean Square | F Ratio | Prob > F |
|---|---|---|---|---|---|
| Label | 5 | 14.039775 | 2.80796 | 6.1453 | 0.0005 * |
| Error | 30 | 13.707920 | 0.45693 | | |
| C. Total | 35 | 27.747695 | | | |

#### Means for Oneway Anova

| Level | Number | Mean | Std Error | Lower 95% | Upper 95% |
|---|---|---|---|---|---|
| 50nM-NA | 6 | -0.1927 | 0.27596 | -0.756 | 0.371 |
| 5nM-NA | 6 | 0.6359 | 0.27596 | 0.072 | 1.199 |
| -NA | 6 | 0.7509 | 0.27596 | 0.187 | 1.315 |
| 50nM+NA | 6 | -1.1537 | 0.27596 | -1.717 | -0.590 |
| 5nM+NA | 6 | 0.0171 | 0.27596 | -0.546 | 0.581 |
| +NA | 6 | -0.0575 | 0.27596 | -0.621 | 0.506 |

Std Error uses a pooled estimate of error variance

### Means Comparisons

#### Comparisons with a control using Dunnett's Method

## Oneway Analysis of 1-Aminocyclopropanecarboxylic acid By Label

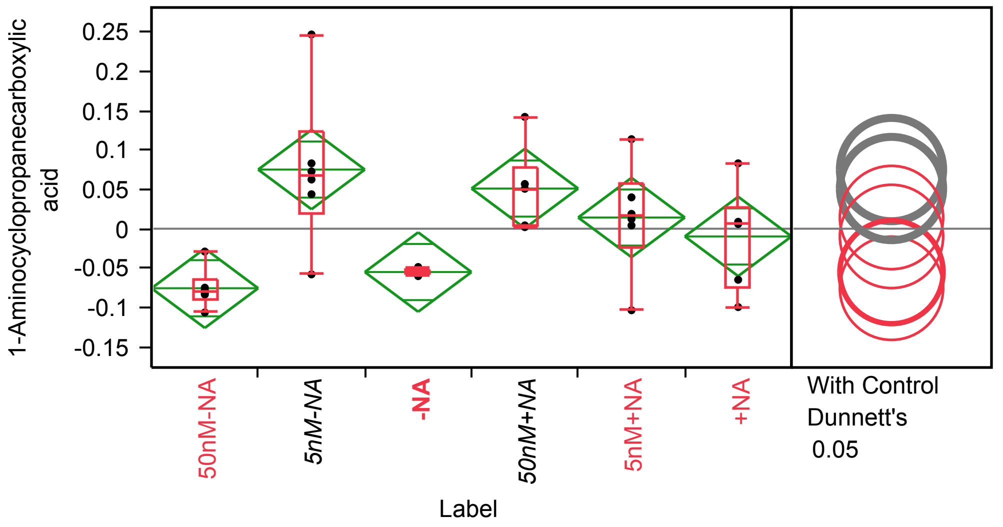


### Quantiles

| Level | Minimum | 10% | 25% | Median | 75% | 90% | Maximum |
|---|---|---|---|---|---|---|---|
| 50nM-NA | -0.10638 | -0.10638 | -0.08932 | -0.07992 | -0.06347 | -0.02907 | -0.02907 |
| 5nM-NA | -0.058 | -0.058 | 0.018364 | 0.06767 | 0.124014 | 0.246799 | 0.246799 |
| -NA | -0.06024 | -0.06024 | -0.058 | -0.05514 | -0.05262 | -0.04865 | -0.04865 |
| 50nM+NA | 0.002025 | 0.002025 | 0.003683 | 0.050625 | 0.078143 | 0.14209 | 0.14209 |
| 5nM+NA | -0.10359 | -0.10359 | -0.02276 | 0.015626 | 0.058336 | 0.113769 | 0.113769 |
| +NA | -0.0996 | -0.0996 | -0.07355 | 0.006943 | 0.02735 | 0.083087 | 0.083087 |

### Oneway Anova

#### Summary of Fit

| | |
|---|---|
| Rsquare | 0.486075 |
| Adj Rsquare | 0.40042 |
| Root Mean Square Error | 0.060504 |
| Mean of Response | -2.8e-11 |
| Observations (or Sum Wgts) | 36 |

#### Analysis of Variance

| Source | DF | Sum of Squares | Mean Square | F Ratio | Prob > F |
|---|---|---|---|---|---|
| Label | 5 | 0.10386893 | 0.020774 | 5.6748 | 0.0008 * |
| Error | 30 | 0.10982031 | 0.003661 | | |
| C. Total | 35 | 0.21368924 | | | |

#### Means for Oneway Anova

| Level | Number | Mean | Std Error | Lower 95% | Upper 95% |
|---|---|---|---|---|---|
| 50nM-NA | 6 | -0.07564 | 0.02470 | -0.1261 | -0.0252 |
| 5nM-NA | 6 | 0.07517 | 0.02470 | 0.0247 | 0.1256 |
| -NA | 6 | -0.05506 | 0.02470 | -0.1055 | -0.0046 |
| 50nM+NA | 6 | 0.05107 | 0.02470 | 0.00063 | 0.1015 |
| 5nM+NA | 6 | 0.01424 | 0.02470 | -0.0362 | 0.0647 |
| +NA | 6 | -0.00979 | 0.02470 | -0.0602 | 0.0407 |

Std Error uses a pooled estimate of error variance

### Means Comparisons

#### Comparisons with a control using Dunnett's Method

## Oneway Analysis of 1-Hexadecyl-2-arachidonoyl-glycerol By Label

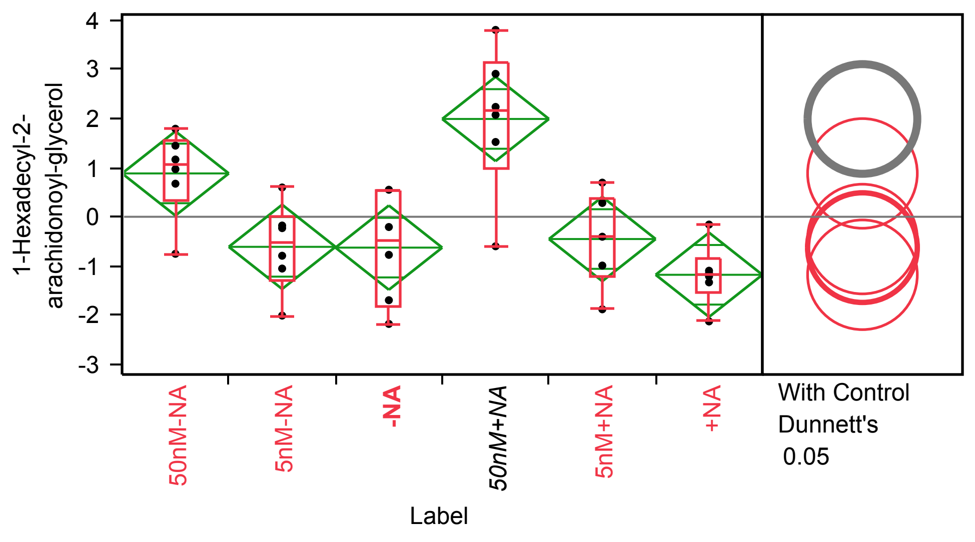


## Quantiles

| Level | Minimum | 10% | 25% | Median | 75% | 90% | Maximum |
|---|---|---|---|---|---|---|---|
| 50nM-NA | -0.75173 | -0.75173 | 0.31588 | 1.065503 | 1.53032 | 1.787532 | 1.787532 |
| 5nM-NA | -2.00659 | -2.00659 | -1.29038 | -0.51095 | 0.01535 | 0.594904 | 0.594904 |
| -NA | -2.17586 | -2.17586 | -1.81476 | -0.49051 | 0.545714 | 0.54885 | 0.54885 |
| 50nM+NA | -0.5986 | -0.5986 | 0.989672 | 2.150331 | 3.125968 | 3.794784 | 3.794784 |
| 5nM+NA | -1.87873 | -1.87873 | -1.21245 | -0.40833 | 0.383646 | 0.692954 | 0.692954 |
| +NA | -2.1245 | -2.1245 | -1.52987 | -1.17947 | -0.86134 | -0.15824 | -0.15824 |

## Oneway Anova

### Summary of Fit

| | |
|---|---|
| Rsquare | 0.573316 |
| Adj Rsquare | 0.502202 |
| Root Mean Square Error | 1.026488 |
| Mean of Response | 8.333e-9 |
| Observations (or Sum Wgts) | 36 |

### Analysis of Variance

| Source | DF | Sum of Squares | Mean Square | F Ratio | Prob > F |
|---|---|---|---|---|---|
| Label | 5 | 42.473310 | 8.49466 | 8.0619 | <.0001 * |
| Error | 30 | 31.610313 | 1.05368 | | |
| C. Total | 35 | 74.083623 | | | |

### Means for Oneway Anova

| Level | Number | Mean | Std Error | Lower 95% | Upper 95% |
|---|---|---|---|---|---|
| 50nM-NA | 6 | 0.8805 | 0.41906 | 0.025 | 1.736 |
| 5nM-NA | 6 | -0.6105 | 0.41906 | -1.466 | 0.245 |
| -NA | 6 | -0.6263 | 0.41906 | -1.482 | 0.230 |
| 50nM+NA | 6 | 1.9865 | 0.41906 | 1.131 | 2.842 |
| 5nM+NA | 6 | -0.4520 | 0.41906 | -1.308 | 0.404 |
| +NA | 6 | -1.1782 | 0.41906 | -2.034 | -0.322 |

Std Error uses a pooled estimate of error variance

## Means Comparisons

### Comparisons with a control using Dunnett's Method

## Oneway Analysis of 1-Methyladenosine By Label

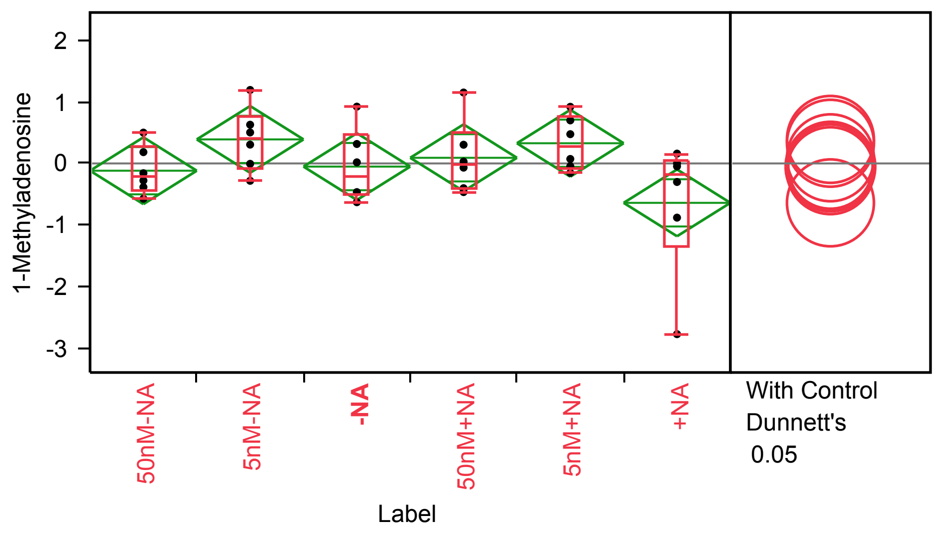


### Quantiles

| Level | Minimum | 10% | 25% | Median | 75% | 90% | Maximum |
|---|---|---|---|---|---|---|---|
| 50nM-NA | -0.57262 | -0.57262 | -0.43101 | -0.21782 | 0.262424 | 0.500071 | 0.500071 |
| 5nM-NA | -0.28172 | -0.28172 | -0.07517 | 0.404088 | 0.769274 | 1.192352 | 1.192352 |
| -NA | -0.62935 | -0.62935 | -0.5088 | -0.22373 | 0.467101 | 0.921483 | 0.921483 |
| 50nM+NA | -0.46508 | -0.46508 | -0.41781 | -0.01926 | 0.515246 | 1.150932 | 1.150932 |
| 5nM+NA | -0.15328 | -0.15328 | -0.0724 | 0.273701 | 0.753904 | 0.919208 | 0.919208 |
| +NA | -2.77138 | -2.77138 | -1.35284 | -0.17401 | 0.040715 | 0.159188 | 0.159188 |

### Oneway Anova

#### Summary of Fit

| | |
|---|---|
| Rsquare | 0.247159 |
| Adj Rsquare | 0.121685 |
| Root Mean Square Error | 0.650204 |
| Mean of Response | 2.78e-11 |
| Observations (or Sum Wgts) | 36 |

#### Analysis of Variance

| Source | DF | Sum of Squares | Mean Square | F Ratio | Prob > F |
|---|---|---|---|---|---|
| Label | 5 | 4.163836 | 0.832767 | 1.9698 | 0.1121 |
| Error | 30 | 12.682969 | 0.422766 | | |
| C. Total | 35 | 16.846805 | | | |

#### Means for Oneway Anova

| Level | Number | Mean | Std Error | Lower 95% | Upper 95% |
|---|---|---|---|---|---|
| 50nM-NA | 6 | -0.11813 | 0.26544 | -0.660 | 0.4240 |
| 5nM-NA | 6 | 0.39012 | 0.26544 | -0.152 | 0.9322 |
| -NA | 6 | -0.05138 | 0.26544 | -0.593 | 0.4907 |
| 50nM+NA | 6 | 0.09144 | 0.26544 | -0.451 | 0.6335 |
| 5nM+NA | 6 | 0.32778 | 0.26544 | -0.214 | 0.8699 |
| +NA | 6 | -0.63983 | 0.26544 | -1.182 | -0.0977 |

Std Error uses a pooled estimate of error variance

### Means Comparisons

#### Comparisons with a control using Dunnett's Method

## Oneway Analysis of 1-Methyl-Histidine By Label

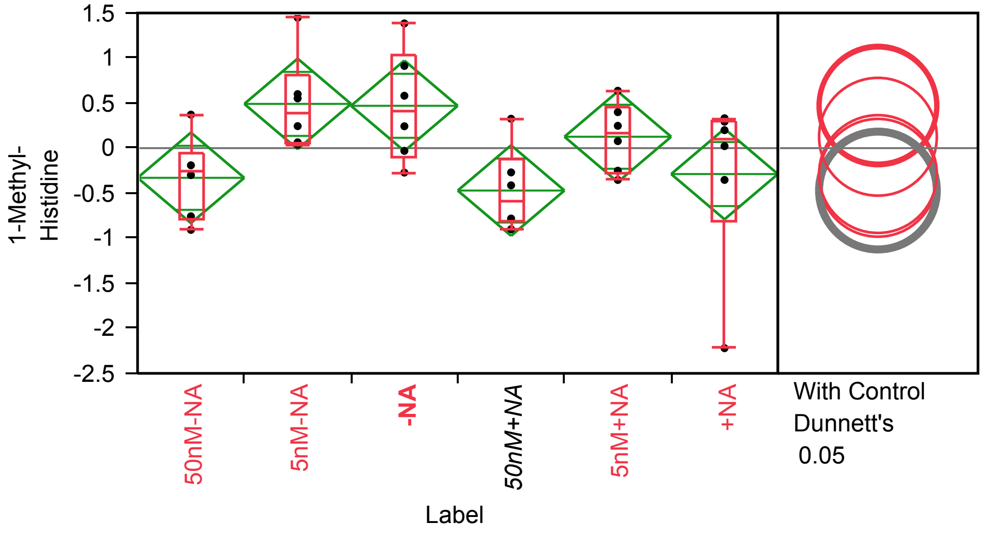


### Quantiles

| Level | Minimum | 10% | 25% | Median | 75% | 90% | Maximum |
|---|---|---|---|---|---|---|---|
| 50nM-NA | -0.90729 | -0.90729 | -0.79465 | -0.24659 | -0.04987 | 0.371832 | 0.371832 |
| 5nM-NA | 0.03814 | 0.03814 | 0.058013 | 0.398363 | 0.811807 | 1.44776 | 1.44776 |
| -NA | -0.27105 | -0.27105 | -0.09035 | 0.412185 | 1.030425 | 1.384041 | 1.384041 |
| 50nM+NA | -0.90041 | -0.90041 | -0.81424 | -0.59734 | -0.12049 | 0.32622 | 0.32622 |
| 5nM+NA | -0.35303 | -0.35303 | -0.27623 | 0.162939 | 0.459017 | 0.636851 | 0.636851 |
| +NA | -2.22052 | -2.22052 | -0.81953 | 0.110959 | 0.303224 | 0.331671 | 0.331671 |

### Oneway Anova

#### Summary of Fit

| | |
|---|---|
| Rsquare | 0.329034 |
| Adj Rsquare | 0.217206 |
| Root Mean Square Error | 0.602753 |
| Mean of Response | 5.56e-10 |
| Observations (or Sum Wgts) | 36 |

#### Analysis of Variance

| Source | DF | Sum of Squares | Mean Square | F Ratio | Prob > F |
|---|---|---|---|---|---|
| Label | 5 | 5.344907 | 1.06898 | 2.9423 | 0.0281 * |
| Error | 30 | 10.899353 | 0.36331 | | |
| C. Total | 35 | 16.244260 | | | |

#### Means for Oneway Anova

| Level | Number | Mean | Std Error | Lower 95% | Upper 95% |
|---|---|---|---|---|---|
| 50nM-NA | 6 | -0.32936 | 0.24607 | -0.8319 | 0.17319 |
| 5nM-NA | 6 | 0.49118 | 0.24607 | -0.0114 | 0.99373 |
| -NA | 6 | 0.46996 | 0.24607 | -0.0326 | 0.97251 |
| 50nM+NA | 6 | -0.47063 | 0.24607 | -0.9732 | 0.03192 |
| 5nM+NA | 6 | 0.12647 | 0.24607 | -0.3761 | 0.62902 |
| +NA | 6 | -0.28762 | 0.24607 | -0.7902 | 0.21493 |

Std Error uses a pooled estimate of error variance

### Means Comparisons

#### Comparisons with a control using Dunnett's Method

## Oneway Analysis of 1-Octadecanoyl-sn-glycero-3-phosphocholine By Label

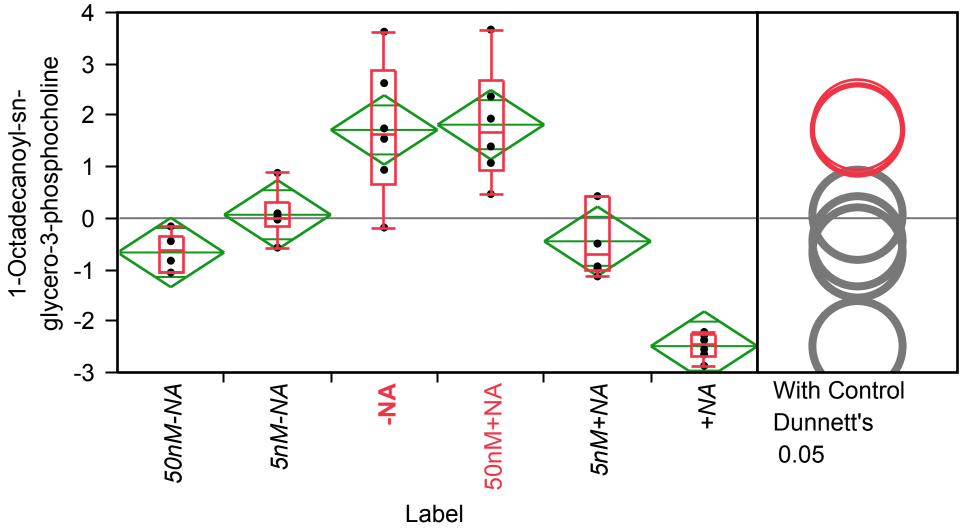


### Quantiles

| Level | Minimum | 10% | 25% | Median | 75% | 90% | Maximum |
|---|---|---|---|---|---|---|---|
| 50nM-NA | -1.06315 | -1.06315 | -1.05826 | -0.63592 | -0.37387 | -0.15971 | -0.15971 |
| 5nM-NA | -0.57199 | -0.57199 | -0.16313 | 0.012927 | 0.291024 | 0.881329 | 0.881329 |
| -NA | -0.18168 | -0.18168 | 0.660042 | 1.644728 | 2.876532 | 3.626184 | 3.626184 |
| 50nM+NA | 0.469931 | 0.469931 | 0.923482 | 1.665239 | 2.692588 | 3.667638 | 3.667638 |
| 5nM+NA | -1.13118 | -1.13118 | -1.02127 | -0.71502 | 0.424549 | 0.429226 | 0.429226 |
| +NA | -2.87058 | -2.87058 | -2.70384 | -2.45888 | -2.2593 | -2.21232 | -2.21232 |

### Oneway Anova

#### Summary of Fit

| | |
|---|---|
| Rsquare | 0.800224 |
| Adj Rsquare | 0.766928 |
| Root Mean Square Error | 0.808464 |
| Mean of Response | 2.78e-11 |
| Observations (or Sum Wgts) | 36 |

#### Analysis of Variance

| Source | DF | Sum of Squares | Mean Square | F Ratio | Prob > F |
|---|---|---|---|---|---|
| Label | 5 | 78.543698 | 15.7087 | 24.0337 | <.0001 * |
| Error | 30 | 19.608424 | 0.6536 | | |
| C. Total | 35 | 98.152123 | | | |

#### Means for Oneway Anova

| Level | Number | Mean | Std Error | Lower 95% | Upper 95% |
|---|---|---|---|---|---|
| 50nM-NA | 6 | -0.6661 | 0.33005 | -1.340 | 0.00796 |
| 5nM-NA | 6 | 0.0671 | 0.33005 | -0.607 | 0.741 |
| -NA | 6 | 1.7169 | 0.33005 | 1.043 | 2.391 |
| 50nM+NA | 6 | 1.8184 | 0.33005 | 1.144 | 2.492 |
| 5nM+NA | 6 | -0.4489 | 0.33005 | -1.123 | 0.225 |
| +NA | 6 | -2.4873 | 0.33005 | -3.161 | -1.813 |

Std Error uses a pooled estimate of error variance

### Means Comparisons

#### Comparisons with a control using Dunnett's Method

## Oneway Analysis of 1-oleoyl-2-hydroxy-sn-glycero-3-phosphocholine By Label

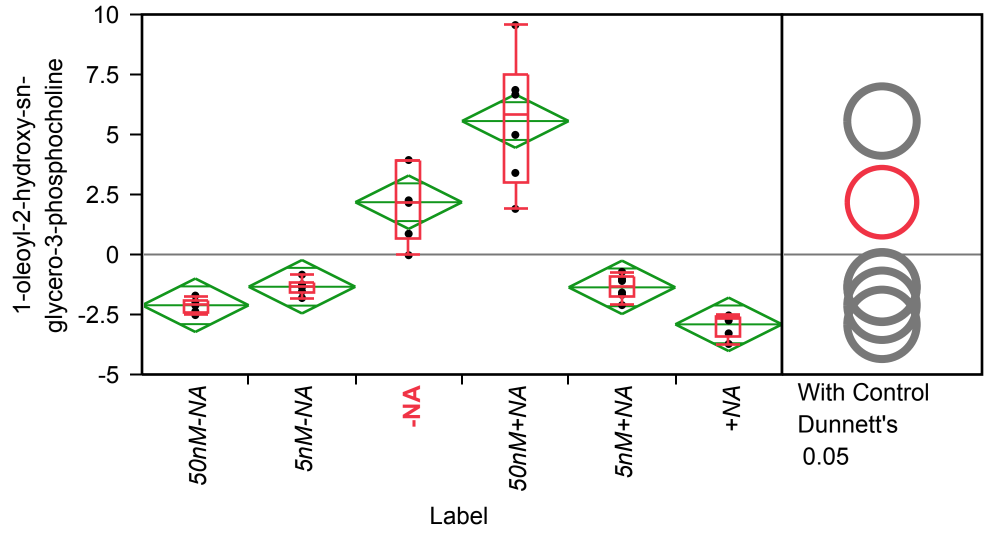


## Quantiles

| Level | Minimum | 10% | 25% | Median | 75% | 90% | Maximum |
|---|---|---|---|---|---|---|---|
| 50nM-NA | -2.52389 | -2.52389 | -2.41175 | -2.05805 | -1.89999 | -1.71279 | -1.71279 |
| 5nM-NA | -1.81414 | -1.81414 | -1.606 | -1.31592 | -1.13889 | -0.84171 | -0.84171 |
| -NA | -0.03 | -0.03 | 0.640034 | 2.2034 | 3.905622 | 3.935722 | 3.935722 |
| 50nM+NA | 1.901309 | 1.901309 | 3.024569 | 5.821837 | 7.531543 | 9.558603 | 9.558603 |
| 5nM+NA | -2.10916 | -2.10916 | -1.78924 | -1.34247 | -0.94148 | -0.72344 | -0.72344 |
| +NA | -3.72739 | -3.72739 | -3.39634 | -2.67736 | -2.55269 | -2.5382 | -2.5382 |

## Oneway Anova

### Summary of Fit

| | |
|---|---|
| Rsquare | 0.854594 |
| Adj Rsquare | 0.830359 |
| Root Mean Square Error | 1.33383 |
| Mean of Response | -2.8e-10 |
| Observations (or Sum Wgts) | 36 |

### Analysis of Variance

| Source | DF | Sum of Squares | Mean Square | F Ratio | Prob > F |
|---|---|---|---|---|---|
| Label | 5 | 313.68889 | 62.7378 | 35.2637 | <.0001 * |
| Error | 30 | 53.37309 | 1.7791 | | |
| C. Total | 35 | 367.06198 | | | |

### Means for Oneway Anova

| Level | Number | Mean | Std Error | Lower 95% | Upper 95% |
|---|---|---|---|---|---|
| 50nM-NA | 6 | -2.1149 | 0.54453 | -3.227 | -1.003 |
| 5nM-NA | 6 | -1.3437 | 0.54453 | -2.456 | -0.232 |
| -NA | 6 | 2.1786 | 0.54453 | 1.066 | 3.291 |
| 50nM+NA | 6 | 5.5597 | 0.54453 | 4.448 | 6.672 |
| 5nM+NA | 6 | -1.3691 | 0.54453 | -2.481 | -0.257 |
| +NA | 6 | -2.9106 | 0.54453 | -4.023 | -1.799 |

Std Error uses a pooled estimate of error variance

## Means Comparisons

### Comparisons with a control using Dunnett's Method

## Oneway Analysis of 2-Aminooctanoic acid By Label

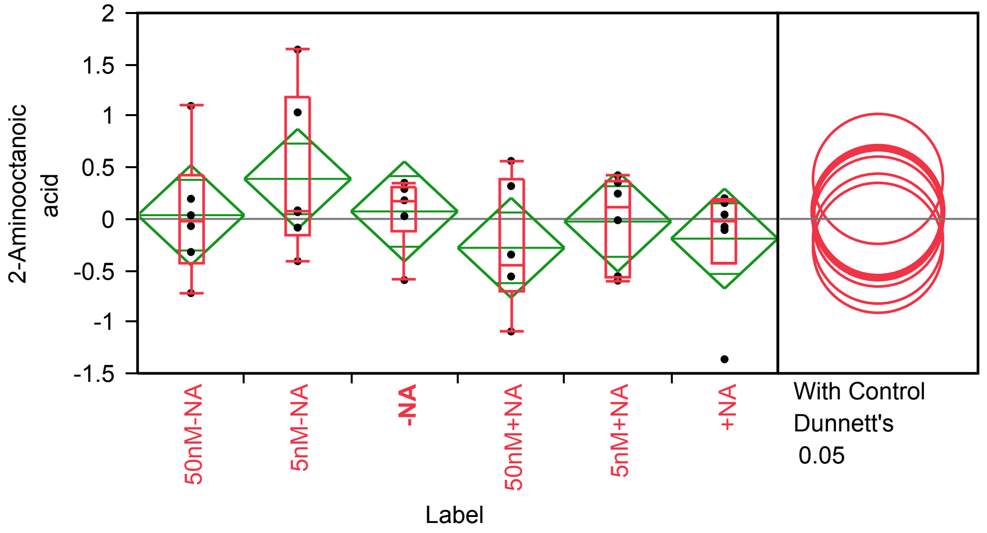


### Quantiles

| Level | Minimum | 10% | 25% | Median | 75% | 90% | Maximum |
|---|---|---|---|---|---|---|---|
| 50nM-NA | -0.72035 | -0.72035 | -0.42104 | -0.01676 | 0.42079 | 1.097218 | 1.097218 |
| 5nM-NA | -0.41027 | -0.41027 | -0.16585 | 0.074338 | 1.186919 | 1.644153 | 1.644153 |
| -NA | -0.59373 | -0.59373 | -0.12804 | 0.180462 | 0.305854 | 0.351042 | 0.351042 |
| 50nM+NA | -1.09324 | -1.09324 | -0.69947 | -0.45267 | 0.380068 | 0.563062 | 0.563062 |
| 5nM+NA | -0.59948 | -0.59948 | -0.56775 | 0.116462 | 0.369275 | 0.417888 | 0.417888 |
| +NA | -1.36315 | -1.36315 | -0.42143 | -0.01674 | 0.166099 | 0.200741 | 0.200741 |

### Oneway Anova

#### Summary of Fit

| | |
|---|---|
| Rsquare | 0.139307 |
| Adj Rsquare | -0.00414 |
| Root Mean Square Error | 0.581765 |
| Mean of Response | -2.8e-10 |
| Observations (or Sum Wgts) | 36 |

#### Analysis of Variance

| Source | DF | Sum of Squares | Mean Square | F Ratio | Prob > F |
|---|---|---|---|---|---|
| Label | 5 | 1.643398 | 0.328680 | 0.9711 | 0.4512 |
| Error | 30 | 10.153532 | 0.338451 | | |
| C. Total | 35 | 11.796930 | | | |

#### Means for Oneway Anova

| Level | Number | Mean | Std Error | Lower 95% | Upper 95% |
|---|---|---|---|---|---|
| 50nM-NA | 6 | 0.03623 | 0.23750 | -0.4488 | 0.52128 |
| 5nM-NA | 6 | 0.38878 | 0.23750 | -0.0963 | 0.87383 |
| -NA | 6 | 0.07270 | 0.23750 | -0.4123 | 0.55775 |
| 50nM+NA | 6 | -0.28078 | 0.23750 | -0.7658 | 0.20427 |
| 5nM+NA | 6 | -0.02546 | 0.23750 | -0.5105 | 0.45959 |
| +NA | 6 | -0.19148 | 0.23750 | -0.6765 | 0.29357 |

Std Error uses a pooled estimate of error variance

### Means Comparisons

#### Comparisons with a control using Dunnett's Method

## Oneway Analysis of 2-dehydro-D-gluconate By Label

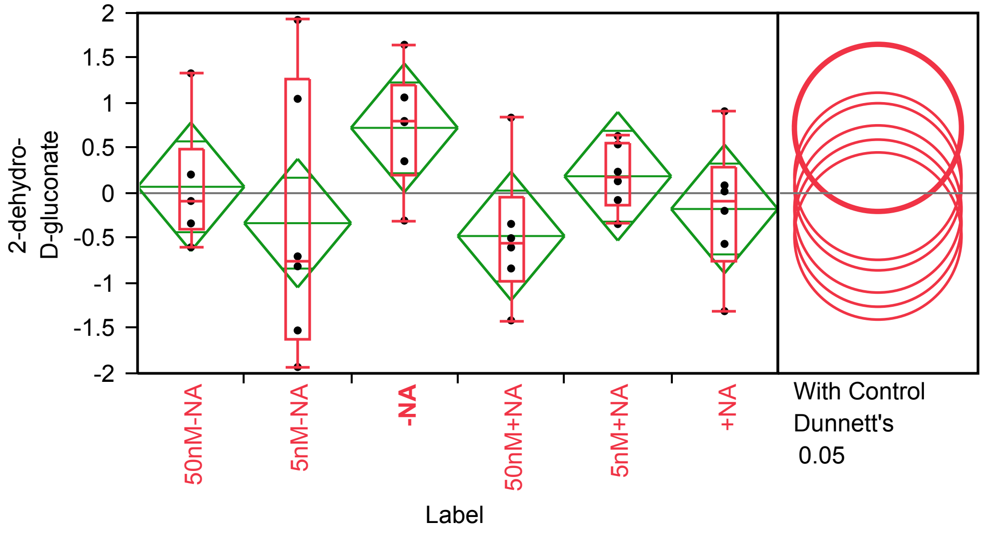


## Quantiles

| Level | Minimum | 10% | 25% | Median | 75% | 90% | Maximum |
|---|---|---|---|---|---|---|---|
| 50nM-NA | -0.60077 | -0.60077 | -0.4017 | -0.08783 | 0.487689 | 1.330371 | 1.330371 |
| 5nM-NA | -1.93006 | -1.93006 | -1.62533 | -0.75673 | 1.266272 | 1.923214 | 1.923214 |
| -NA | -0.30407 | -0.30407 | 0.190013 | 0.7949 | 1.208738 | 1.647399 | 1.647399 |
| 50nM+NA | -1.41223 | -1.41223 | -0.97975 | -0.55108 | -0.04651 | 0.838023 | 0.838023 |
| 5nM+NA | -0.34271 | -0.34271 | -0.14296 | 0.185077 | 0.564855 | 0.634486 | 0.634486 |
| +NA | -1.30966 | -1.30966 | -0.74966 | -0.08793 | 0.2922 | 0.909109 | 0.909109 |

## Oneway Anova

### Summary of Fit

| | |
|---|---|
| Rsquare | 0.203406 |
| Adj Rsquare | 0.07064 |
| Root Mean Square Error | 0.855186 |
| Mean of Response | -2.5e-17 |
| Observations (or Sum Wgts) | 36 |

### Analysis of Variance

| Source | DF | Sum of Squares | Mean Square | F Ratio | Prob > F |
|---|---|---|---|---|---|
| Label | 5 | 5.602339 | 1.12047 | 1.5321 | 0.2097 |
| Error | 30 | 21.940303 | 0.73134 | | |
| C. Total | 35 | 27.542642 | | | |

### Means for Oneway Anova

| Level | Number | Mean | Std Error | Lower 95% | Upper 95% |
|---|---|---|---|---|---|
| 50nM-NA | 6 | 0.07090 | 0.34913 | -0.642 | 0.7839 |
| 5nM-NA | 6 | -0.33280 | 0.34913 | -1.046 | 0.3802 |
| -NA | 6 | 0.72506 | 0.34913 | 0.012 | 1.4381 |
| 50nM+NA | 6 | -0.47555 | 0.34913 | -1.189 | 0.2375 |
| 5nM+NA | 6 | 0.18787 | 0.34913 | -0.525 | 0.9009 |
| +NA | 6 | -0.17548 | 0.34913 | -0.888 | 0.5375 |

Std Error uses a pooled estimate of error variance

## Means Comparisons

### Comparisons with a control using Dunnett's Method

## Oneway Analysis of 2-deoxyglucose-6-phosphate By Label

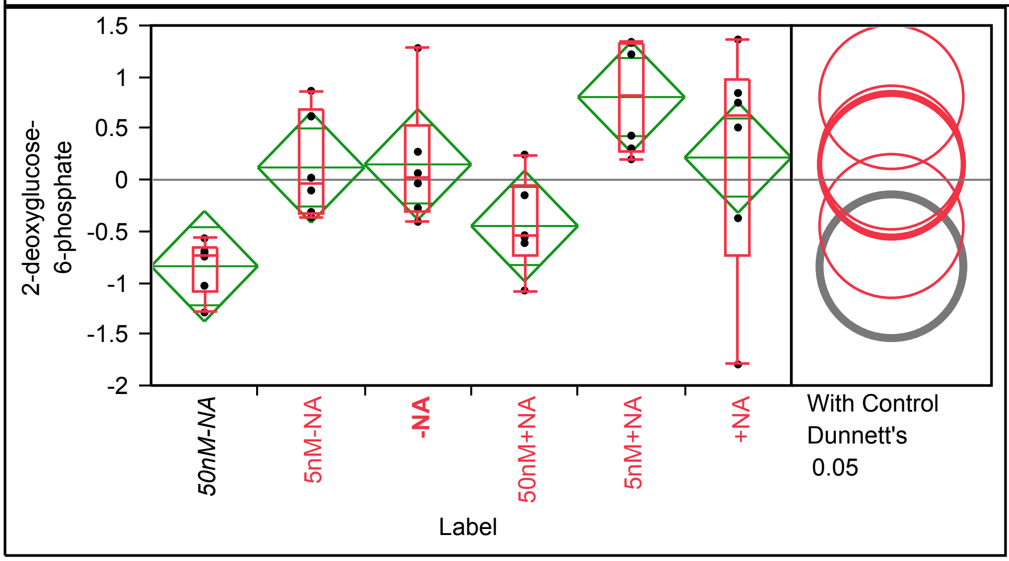


### Quantiles

| Level | Minimum | 10% | 25% | Median | 75% | 90% | Maximum |
|---|---|---|---|---|---|---|---|
| 50nM-NA | -1.28966 | -1.28966 | -1.09506 | -0.72976 | -0.66296 | -0.56902 | -0.56902 |
| 5nM-NA | -0.3649 | -0.3649 | -0.32577 | -0.04189 | 0.677897 | 0.864631 | 0.864631 |
| -NA | -0.40841 | -0.40841 | -0.30822 | 0.01459 | 0.524095 | 1.280223 | 1.280223 |
| 50nM+NA | -1.07641 | -1.07641 | -0.73058 | -0.54921 | -0.05153 | 0.244543 | 0.244543 |
| 5nM+NA | 0.199542 | 0.199542 | 0.278759 | 0.824308 | 1.333065 | 1.340506 | 1.340506 |
| +NA | -1.79493 | -1.79493 | -0.72863 | 0.628978 | 0.973617 | 1.363469 | 1.363469 |

### Oneway Anova

#### Summary of Fit

| | |
|---|---|
| Rsquare | 0.441323 |
| Adj Rsquare | 0.34821 |
| Root Mean Square Error | 0.644029 |
| Mean of Response | 2.78e-10 |
| Observations (or Sum Wgts) | 36 |

#### Analysis of Variance

| Source | DF | Sum of Squares | Mean Square | F Ratio | Prob > F |
|---|---|---|---|---|---|
| Label | 5 | 9.829424 | 1.96588 | 4.7397 | 0.0026 * |
| Error | 30 | 12.443209 | 0.41477 | | |
| C. Total | 35 | 22.272633 | | | |

#### Means for Oneway Anova

| Level | Number | Mean | Std Error | Lower 95% | Upper 95% |
|---|---|---|---|---|---|
| 50nM-NA | 6 | -0.84044 | 0.26292 | -1.377 | -0.303 |
| 5nM-NA | 6 | 0.11981 | 0.26292 | -0.417 | 0.657 |
| -NA | 6 | 0.14970 | 0.26292 | -0.387 | 0.687 |
| 50nM+NA | 6 | -0.44930 | 0.26292 | -0.986 | 0.088 |
| 5nM+NA | 6 | 0.80407 | 0.26292 | 0.267 | 1.341 |
| +NA | 6 | 0.21616 | 0.26292 | -0.321 | 0.753 |

Std Error uses a pooled estimate of error variance

### Means Comparisons

#### Comparisons with a control using Dunnett's Method

## Oneway Analysis of 2-Hydroxy-2-methylbutanedioic acid By Label

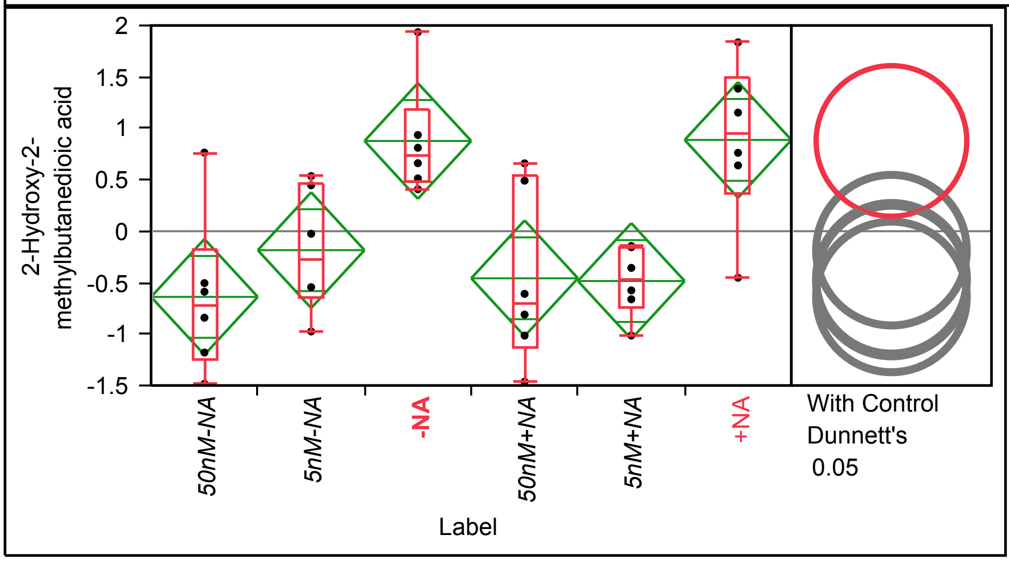


### Quantiles

| Level | Minimum | 10% | 25% | Median | 75% | 90% | Maximum |
|---|---|---|---|---|---|---|---|
| 50nM-NA | -1.48285 | -1.48285 | -1.25534 | -0.71477 | -0.18727 | 0.764637 | 0.764637 |
| 5nM-NA | -0.97433 | -0.97433 | -0.65242 | -0.28465 | 0.469082 | 0.536051 | 0.536051 |
| -NA | 0.408084 | 0.408084 | 0.487222 | 0.736287 | 1.185382 | 1.935237 | 1.935237 |
| 50nM+NA | -1.46276 | -1.46276 | -1.12674 | -0.70973 | 0.533529 | 0.659715 | 0.659715 |
| 5nM+NA | -1.01364 | -1.01364 | -0.74963 | -0.46587 | -0.15264 | -0.14367 | -0.14367 |
| +NA | -0.45251 | -0.45251 | 0.366659 | 0.957737 | 1.498541 | 1.836291 | 1.836291 |

### Oneway Anova

#### Summary of Fit

| | |
|---|---|
| Rsquare | 0.517708 |
| Adj Rsquare | 0.437326 |
| Root Mean Square Error | 0.674737 |
| Mean of Response | -1.11e-9 |
| Observations (or Sum Wgts) | 36 |

#### Analysis of Variance

| Source | DF | Sum of Squares | Mean Square | F Ratio | Prob > F |
|---|---|---|---|---|---|
| Label | 5 | 14.661036 | 2.93221 | 6.4406 | 0.0004 * |
| Error | 30 | 13.658088 | 0.45527 | | |
| C. Total | 35 | 28.319124 | | | |

#### Means for Oneway Anova

| Level | Number | Mean | Std Error | Lower 95% | Upper 95% |
|---|---|---|---|---|---|
| 50nM-NA | 6 | -0.63864 | 0.27546 | -1.201 | -0.076 |
| 5nM-NA | 6 | -0.18432 | 0.27546 | -0.747 | 0.378 |
| -NA | 6 | 0.87749 | 0.27546 | 0.315 | 1.440 |
| 50nM+NA | 6 | -0.45763 | 0.27546 | -1.020 | 0.105 |
| 5nM+NA | 6 | -0.48439 | 0.27546 | -1.047 | 0.078 |
| +NA | 6 | 0.88749 | 0.27546 | 0.325 | 1.450 |

Std Error uses a pooled estimate of error variance

### Means Comparisons

#### Comparisons with a control using Dunnett's Method

## Oneway Analysis of 2-hydroxyglutarate By Label

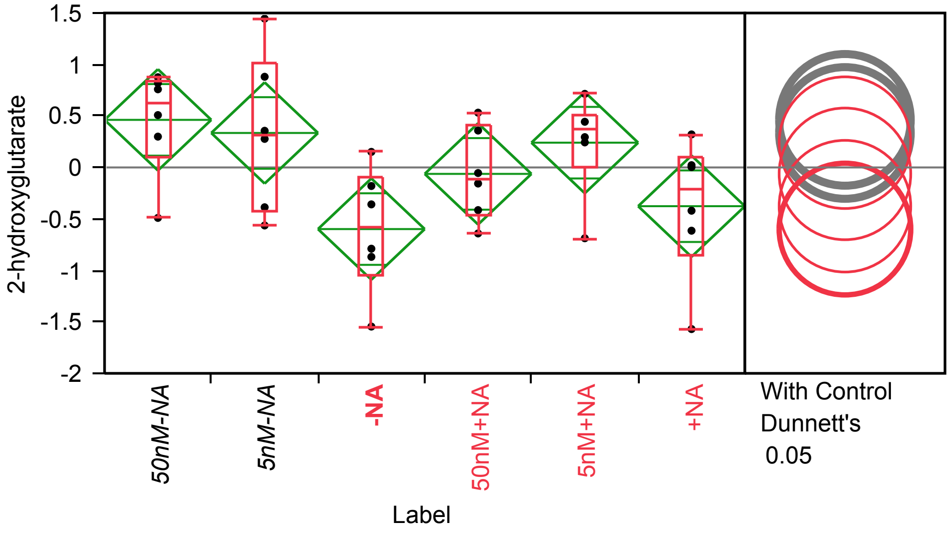


## Quantiles

| Level | Minimum | 10% | 25% | Median | 75% | 90% | Maximum |
|---|---|---|---|---|---|---|---|
| 50nM-NA | -0.49002 | -0.49002 | 0.101943 | 0.633754 | 0.834007 | 0.876246 | 0.876246 |
| 5nM-NA | -0.5636 | -0.5636 | -0.43103 | 0.316745 | 1.021839 | 1.445551 | 1.445551 |
| -NA | -1.54564 | -1.54564 | -1.03794 | -0.57408 | -0.09736 | 0.150921 | 0.150921 |
| 50nM+NA | -0.64098 | -0.64098 | -0.47111 | -0.10527 | 0.401857 | 0.531968 | 0.531968 |
| 5nM+NA | -0.68772 | -0.68772 | 0.01054 | 0.364878 | 0.511249 | 0.7145 | 0.7145 |
| +NA | -1.5698 | -1.5698 | -0.85327 | -0.20837 | 0.096677 | 0.320781 | 0.320781 |

## Oneway Anova

### Summary of Fit

| | |
|---|---|
| Rsquare | 0.338092 |
| Adj Rsquare | 0.227774 |
| Root Mean Square Error | 0.589496 |
| Mean of Response | 0 |
| Observations (or Sum Wgts) | 36 |

### Analysis of Variance

| Source | DF | Sum of Squares | Mean Square | F Ratio | Prob > F |
|---|---|---|---|---|---|
| Label | 5 | 5.325017 | 1.06500 | 3.0647 | 0.0237 * |
| Error | 30 | 10.425175 | 0.34751 | | |
| C. Total | 35 | 15.750193 | | | |

### Means for Oneway Anova

| Level | Number | Mean | Std Error | Lower 95% | Upper 95% |
|---|---|---|---|---|---|
| 50nM-NA | 6 | 0.46215 | 0.24066 | -0.029 | 0.9536 |
| 5nM-NA | 6 | 0.33487 | 0.24066 | -0.157 | 0.8264 |
| -NA | 6 | -0.59862 | 0.24066 | -1.090 | -0.1071 |
| 50nM+NA | 6 | -0.06259 | 0.24066 | -0.554 | 0.4289 |
| 5nM+NA | 6 | 0.24055 | 0.24066 | -0.251 | 0.7320 |
| +NA | 6 | -0.37637 | 0.24066 | -0.868 | 0.1151 |

Std Error uses a pooled estimate of error variance

## Means Comparisons

### Comparisons with a control using Dunnett's Method

## Oneway Analysis of 2-Isopropylmalic acid By Label

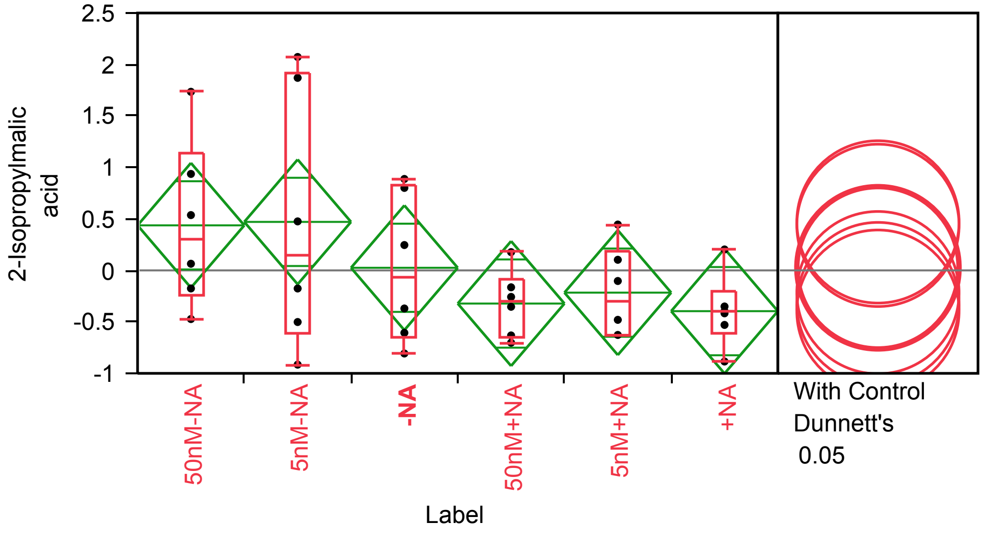


## Quantiles

| Level | Minimum | 10% | 25% | Median | 75% | 90% | Maximum |
|---|---|---|---|---|---|---|---|
| 50nM-NA | -0.47261 | -0.47261 | -0.2498 | 0.300859 | 1.136352 | 1.734583 | 1.734583 |
| 5nM-NA | -0.91544 | -0.91544 | -0.60498 | 0.150109 | 1.920846 | 2.072873 | 2.072873 |
| -NA | -0.80713 | -0.80713 | -0.65666 | -0.06184 | 0.822969 | 0.888124 | 0.888124 |
| 50nM+NA | -0.70253 | -0.70253 | -0.64955 | -0.30547 | -0.07886 | 0.177279 | 0.177279 |
| 5nM+NA | -0.6338 | -0.6338 | -0.62873 | -0.29164 | 0.188647 | 0.445353 | 0.445353 |
| +NA | -0.88503 | -0.88503 | -0.61844 | -0.40681 | -0.21206 | 0.203249 | 0.203249 |

## Oneway Anova

### Summary of Fit

| | |
|---|---|
| Rsquare | 0.214504 |
| Adj Rsquare | 0.083588 |
| Root Mean Square Error | 0.726699 |
| Mean of Response | -2.8e-10 |
| Observations (or Sum Wgts) | 36 |

### Analysis of Variance

| Source | DF | Sum of Squares | Mean Square | F Ratio | Prob > F |
|---|---|---|---|---|---|
| Label | 5 | 4.326360 | 0.865272 | 1.6385 | 0.1802 |
| Error | 30 | 15.842754 | 0.528092 | | |
| C. Total | 35 | 20.169114 | | | |

### Means for Oneway Anova

| Level | Number | Mean | Std Error | Lower 95% | Upper 95% |
|---|---|---|---|---|---|
| 50nM-NA | 6 | 0.43752 | 0.29667 | -0.168 | 1.0434 |
| 5nM-NA | 6 | 0.47106 | 0.29667 | -0.135 | 1.0769 |
| -NA | 6 | 0.02534 | 0.29667 | -0.581 | 0.6312 |
| 50nM+NA | 6 | -0.32206 | 0.29667 | -0.928 | 0.2838 |
| 5nM+NA | 6 | -0.21595 | 0.29667 | -0.822 | 0.3899 |
| +NA | 6 | -0.39591 | 0.29667 | -1.002 | 0.2100 |

Std Error uses a pooled estimate of error variance

## Means Comparisons

### Comparisons with a control using Dunnett's Method

## Oneway Analysis of 2-ketoglutarate By Label

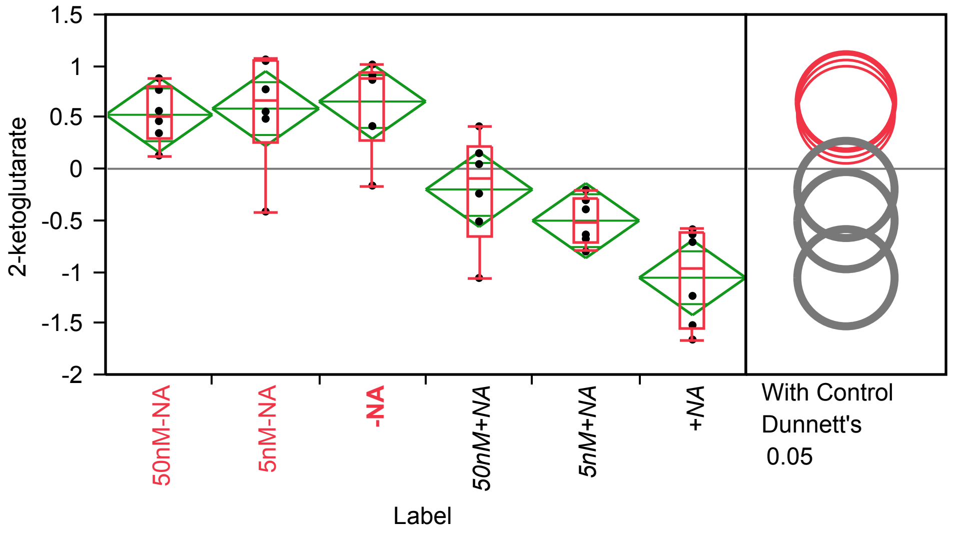


### Quantiles

| Level | Minimum | 10% | 25% | Median | 75% | 90% | Maximum |
|---|---|---|---|---|---|---|---|
| 50nM-NA | 0.128982 | 0.128982 | 0.292164 | 0.513335 | 0.795199 | 0.879979 | 0.879979 |
| 5nM-NA | -0.41587 | -0.41587 | 0.260855 | 0.664277 | 1.051997 | 1.062897 | 1.062897 |
| -NA | -0.16343 | -0.16343 | 0.270493 | 0.873766 | 0.937905 | 1.013418 | 1.013418 |
| 50nM+NA | -1.06157 | -1.06157 | -0.64938 | -0.09769 | 0.21705 | 0.412934 | 0.412934 |
| 5nM+NA | -0.80339 | -0.80339 | -0.71149 | -0.51648 | -0.27969 | -0.20427 | -0.20427 |
| +NA | -1.66045 | -1.66045 | -1.55535 | -0.97373 | -0.6256 | -0.58801 | -0.58801 |

### Oneway Anova

#### Summary of Fit

| | |
|---|---|
| Rsquare | 0.72177 |
| Adj Rsquare | 0.675399 |
| Root Mean Square Error | 0.435681 |
| Mean of Response | 5.56e-10 |
| Observations (or Sum Wgts) | 36 |

#### Analysis of Variance

| Source | DF | Sum of Squares | Mean Square | F Ratio | Prob > F |
|---|---|---|---|---|---|
| Label | 5 | 14.772491 | 2.95450 | 15.5649 | <.0001 * |
| Error | 30 | 5.694533 | 0.18982 | | |
| C. Total | 35 | 20.467024 | | | |

#### Means for Oneway Anova

| Level | Number | Mean | Std Error | Lower 95% | Upper 95% |
|---|---|---|---|---|---|
| 50nM-NA | 6 | 0.5249 | 0.17787 | 0.162 | 0.888 |
| 5nM-NA | 6 | 0.5851 | 0.17787 | 0.222 | 0.948 |
| -NA | 6 | 0.6542 | 0.17787 | 0.291 | 1.017 |
| 50nM+NA | 6 | -0.2007 | 0.17787 | -0.564 | 0.163 |
| 5nM+NA | 6 | -0.5044 | 0.17787 | -0.868 | -0.141 |
| +NA | 6 | -1.0591 | 0.17787 | -1.422 | -0.696 |

Std Error uses a pooled estimate of error variance

### Means Comparisons

#### Comparisons with a control using Dunnett's Method

## Oneway Analysis of 2-Oxo-4-hydroxy-5-aminovalerate By Label

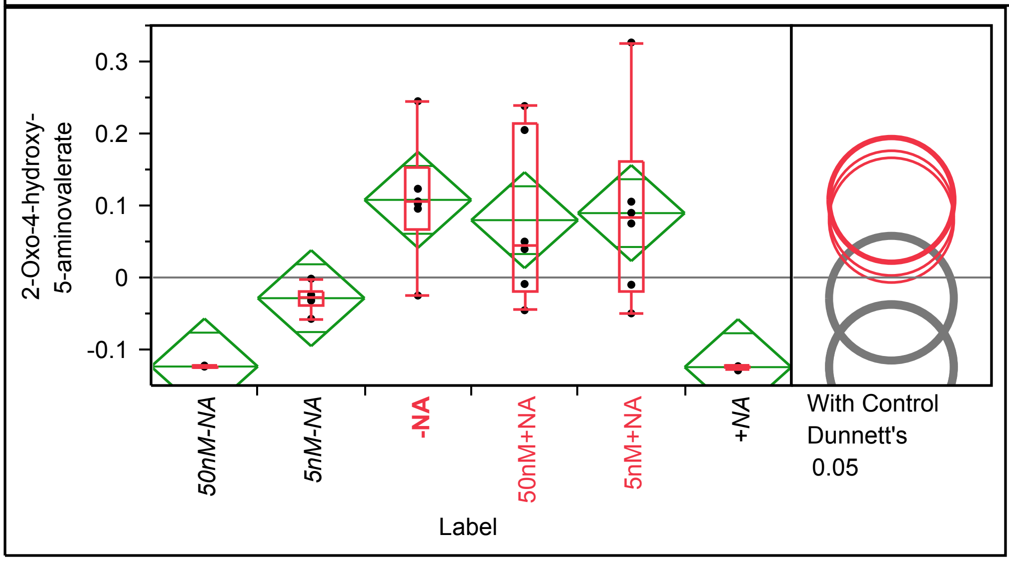


## Quantiles

| Level | Minimum | 10% | 25% | Median | 75% | 90% | Maximum |
|---|---|---|---|---|---|---|---|
| 50nM-NA | -0.12441 | -0.12441 | -0.12416 | -0.12388 | -0.12307 | -0.12235 | -0.12235 |
| 5nM-NA | -0.05741 | -0.05741 | -0.03838 | -0.02859 | -0.01842 | -0.00168 | -0.00168 |
| -NA | -0.02507 | -0.02507 | 0.065361 | 0.104178 | 0.153622 | 0.244839 | 0.244839 |
| 50nM+NA | -0.04546 | -0.04546 | -0.01812 | 0.044654 | 0.213157 | 0.238159 | 0.238159 |
| 5nM+NA | -0.04981 | -0.04981 | -0.02001 | 0.082395 | 0.16057 | 0.326361 | 0.326361 |
| +NA | -0.129 | -0.129 | -0.12585 | -0.12337 | -0.12324 | -0.12319 | -0.12319 |

## Oneway Anova

### Summary of Fit

| | |
|---|---|
| Rsquare | 0.643757 |
| Adj Rsquare | 0.584383 |
| Root Mean Square Error | 0.079827 |
| Mean of Response | 3.08e-18 |
| Observations (or Sum Wgts) | 36 |

### Analysis of Variance

| Source | DF | Sum of Squares | Mean Square | F Ratio | Prob > F |
|---|---|---|---|---|---|
| Label | 5 | 0.34545608 | 0.069091 | 10.8424 | <.0001 * |
| Error | 30 | 0.19116869 | 0.006372 | | |
| C. Total | 35 | 0.53662477 | | | |

### Means for Oneway Anova

| Level | Number | Mean | Std Error | Lower 95% | Upper 95% |
|---|---|---|---|---|---|
| 50nM-NA | 6 | -0.12365 | 0.03259 | -0.1902 | -0.0571 |
| 5nM-NA | 6 | -0.02872 | 0.03259 | -0.0953 | 0.0378 |
| -NA | 6 | 0.10781 | 0.03259 | 0.0413 | 0.1744 |
| 50nM+NA | 6 | 0.07964 | 0.03259 | 0.0131 | 0.1462 |
| 5nM+NA | 6 | 0.08943 | 0.03259 | 0.0229 | 0.1560 |
| +NA | 6 | -0.12450 | 0.03259 | -0.1911 | -0.0579 |

Std Error uses a pooled estimate of error variance

## Means Comparisons

### Comparisons with a control using Dunnett's Method

## Oneway Analysis of 2-oxobutanoate By Label

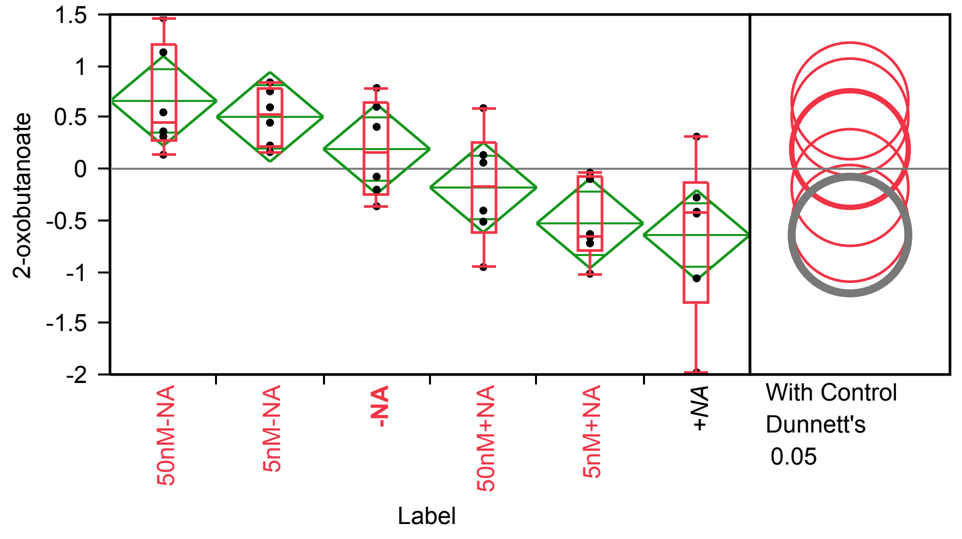


## Quantiles

| Level | Minimum | 10% | 25% | Median | 75% | 90% | Maximum |
|---|---|---|---|---|---|---|---|
| 50nM-NA | 0.136511 | 0.136511 | 0.269092 | 0.456175 | 1.21537 | 1.464117 | 1.464117 |
| 5nM-NA | 0.162949 | 0.162949 | 0.210347 | 0.522992 | 0.774793 | 0.839897 | 0.839897 |
| -NA | -0.36441 | -0.36441 | -0.24358 | 0.165318 | 0.648469 | 0.785597 | 0.785597 |
| 50nM+NA | -0.95511 | -0.95511 | -0.62434 | -0.17318 | 0.247768 | 0.589833 | 0.589833 |
| 5nM+NA | -1.02042 | -1.02042 | -0.7986 | -0.6494 | -0.0842 | -0.03843 | -0.03843 |
| +NA | -1.9795 | -1.9795 | -1.29347 | -0.42646 | -0.1316 | 0.312532 | 0.312532 |

## Oneway Anova

### Summary of Fit

| | |
|---|---|
| Rsquare | 0.515823 |
| Adj Rsquare | 0.435126 |
| Root Mean Square Error | 0.52281 |
| Mean of Response | -2.8e-10 |
| Observations (or Sum Wgts) | 36 |

### Analysis of Variance

| Source | DF | Sum of Squares | Mean Square | F Ratio | Prob > F |
|---|---|---|---|---|---|
| Label | 5 | 8.735857 | 1.74717 | 6.3922 | 0.0004 * |
| Error | 30 | 8.199919 | 0.27333 | | |
| C. Total | 35 | 16.935776 | | | |

### Means for Oneway Anova

| Level | Number | Mean | Std Error | Lower 95% | Upper 95% |
|---|---|---|---|---|---|
| 50nM-NA | 6 | 0.65979 | 0.21344 | 0.224 | 1.096 |
| 5nM-NA | 6 | 0.50468 | 0.21344 | 0.069 | 0.941 |
| -NA | 6 | 0.19188 | 0.21344 | -0.244 | 0.628 |
| 50nM+NA | 6 | -0.18199 | 0.21344 | -0.618 | 0.254 |
| 5nM+NA | 6 | -0.53029 | 0.21344 | -0.966 | -0.094 |
| +NA | 6 | -0.64406 | 0.21344 | -1.080 | -0.208 |

Std Error uses a pooled estimate of error variance

## Means Comparisons

### Comparisons with a control using Dunnett's Method

## Oneway Analysis of 3-phosphoglycerate By Label

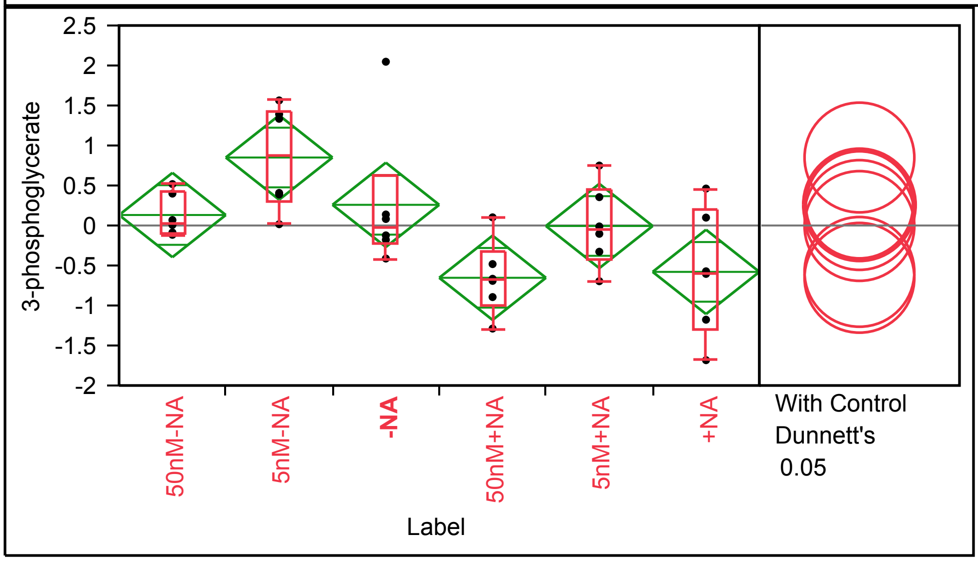


### Quantiles

| Level | Minimum | 10% | 25% | Median | 75% | 90% | Maximum |
|---|---|---|---|---|---|---|---|
| 50nM-NA | -0.11627 | -0.11627 | -0.09452 | 0.037201 | 0.428484 | 0.517465 | 0.517465 |
| 5nM-NA | 0.016316 | 0.016316 | 0.294981 | 0.870428 | 1.433625 | 1.563337 | 1.563337 |
| -NA | -0.413 | -0.413 | -0.23703 | -0.02107 | 0.614816 | 2.047001 | 2.047001 |
| 50nM+NA | -1.28828 | -1.28828 | -0.99321 | -0.67909 | -0.33635 | 0.101817 | 0.101817 |
| 5nM+NA | -0.6961 | -0.6961 | -0.42013 | -0.05787 | 0.453087 | 0.748826 | 0.748826 |
| +NA | -1.68329 | -1.68329 | -1.30401 | -0.58875 | 0.189314 | 0.461515 | 0.461515 |

### Oneway Anova

#### Summary of Fit

| | |
|---|---|
| Rsquare | 0.439828 |
| Adj Rsquare | 0.346466 |
| Root Mean Square Error | 0.632296 |
| Mean of Response | 1.11e-10 |
| Observations (or Sum Wgts) | 36 |

#### Analysis of Variance

| Source | DF | Sum of Squares | Mean Square | F Ratio | Prob > F |
|---|---|---|---|---|---|
| Label | 5 | 9.417239 | 1.88345 | 4.7110 | 0.0027 * |
| Error | 30 | 11.993952 | 0.39980 | | |
| C. Total | 35 | 21.411191 | | | |

#### Means for Oneway Anova

| Level | Number | Mean | Std Error | Lower 95% | Upper 95% |
|---|---|---|---|---|---|
| 50nM-NA | 6 | 0.13119 | 0.25813 | -0.396 | 0.658 |
| 5nM-NA | 6 | 0.84979 | 0.25813 | 0.323 | 1.377 |
| -NA | 6 | 0.25849 | 0.25813 | -0.269 | 0.786 |
| 50nM+NA | 6 | -0.65365 | 0.25813 | -1.181 | -0.126 |
| 5nM+NA | 6 | -0.00611 | 0.25813 | -0.533 | 0.521 |
| +NA | 6 | -0.57971 | 0.25813 | -1.107 | -0.053 |

Std Error uses a pooled estimate of error variance

### Means Comparisons

#### Comparisons with a control using Dunnett's Method

## Oneway Analysis of 3-phospho-serine By Label

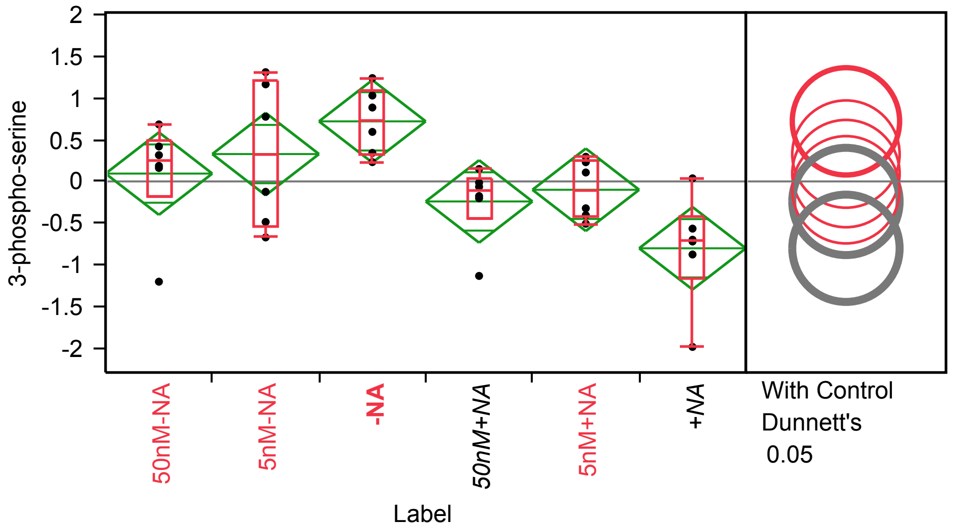


### Quantiles

| Level | Minimum | 10% | 25% | Median | 75% | 90% | Maximum |
|---|---|---|---|---|---|---|---|
| 50nM-NA | -1.20211 | -1.20211 | -0.1796 | 0.250815 | 0.485202 | 0.683513 | 0.683513 |
| 5nM-NA | -0.67114 | -0.67114 | -0.53239 | 0.325432 | 1.200464 | 1.309678 | 1.309678 |
| -NA | 0.231335 | 0.231335 | 0.314278 | 0.739725 | 1.08177 | 1.238625 | 1.238625 |
| 50nM+NA | -1.1329 | -1.1329 | -0.43459 | -0.12013 | 0.026349 | 0.148617 | 0.148617 |
| 5nM+NA | -0.50715 | -0.50715 | -0.43019 | -0.10761 | 0.246504 | 0.294967 | 0.294967 |
| +NA | -1.98417 | -1.98417 | -1.15304 | -0.70979 | -0.41538 | 0.036666 | 0.036666 |

### Oneway Anova

#### Summary of Fit

| | |
|---|---|
| Rsquare | 0.433551 |
| Adj Rsquare | 0.339143 |
| Root Mean Square Error | 0.592856 |
| Mean of Response | -1.39e-9 |
| Observations (or Sum Wgts) | 36 |

#### Analysis of Variance

| Source | DF | Sum of Squares | Mean Square | F Ratio | Prob > F |
|---|---|---|---|---|---|
| Label | 5 | 8.070477 | 1.61410 | 4.5923 | 0.0031 * |
| Error | 30 | 10.544344 | 0.35148 | | |
| C. Total | 35 | 18.614822 | | | |

#### Means for Oneway Anova

| Level | Number | Mean | Std Error | Lower 95% | Upper 95% |
|---|---|---|---|---|---|
| 50nM-NA | 6 | 0.09390 | 0.24203 | -0.400 | 0.588 |
| 5nM-NA | 6 | 0.32789 | 0.24203 | -0.166 | 0.822 |
| -NA | 6 | 0.72014 | 0.24203 | 0.226 | 1.214 |
| 50nM+NA | 6 | -0.24013 | 0.24203 | -0.734 | 0.254 |
| 5nM+NA | 6 | -0.10027 | 0.24203 | -0.595 | 0.394 |
| +NA | 6 | -0.80152 | 0.24203 | -1.296 | -0.307 |

Std Error uses a pooled estimate of error variance

### Means Comparisons

#### Comparisons with a control using Dunnett's Method

## Oneway Analysis of 4-aminobutyrate By Label

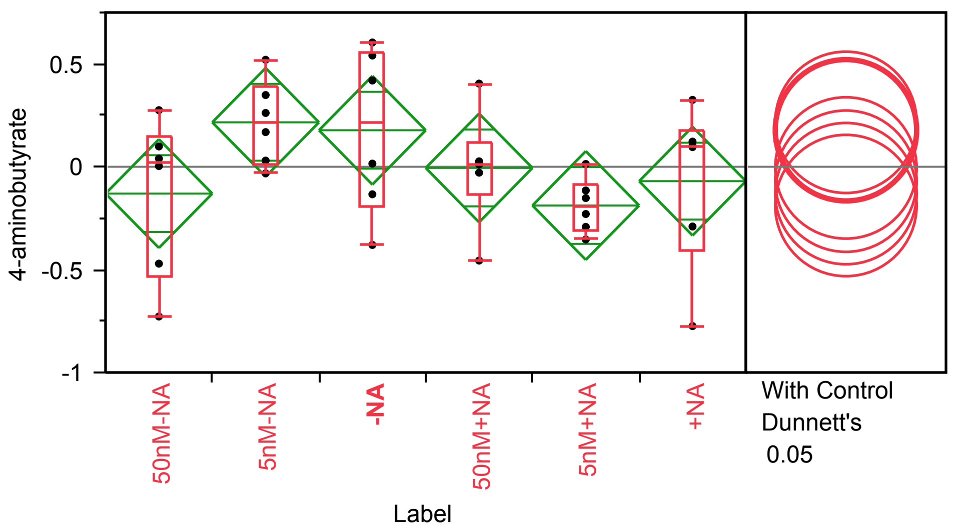


## Quantiles

| Level | Minimum | 10% | 25% | Median | 75% | 90% | Maximum |
|---|---|---|---|---|---|---|---|
| 50nM-NA | -0.72781 | -0.72781 | -0.53511 | 0.021487 | 0.142881 | 0.275201 | 0.275201 |
| 5nM-NA | -0.03173 | -0.03173 | 0.014503 | 0.215073 | 0.391579 | 0.520172 | 0.520172 |
| -NA | -0.38006 | -0.38006 | -0.19531 | 0.217374 | 0.556699 | 0.604051 | 0.604051 |
| 50nM+NA | -0.45593 | -0.45593 | -0.13537 | 0.010233 | 0.120878 | 0.404299 | 0.404299 |
| 5nM+NA | -0.35344 | -0.35344 | -0.30746 | -0.19085 | -0.08297 | 0.01349 | 0.01349 |
| +NA | -0.77441 | -0.77441 | -0.4114 | 0.099078 | 0.172963 | 0.325228 | 0.325228 |

## Oneway Anova

### Summary of Fit

| | |
|---|---|
| Rsquare | 0.212844 |
| Adj Rsquare | 0.081651 |
| Root Mean Square Error | 0.316691 |
| Mean of Response | -5.6e-11 |
| Observations (or Sum Wgts) | 36 |

### Analysis of Variance

| Source | DF | Sum of Squares | Mean Square | F Ratio | Prob > F |
|---|---|---|---|---|---|
| Label | 5 | 0.8135673 | 0.162713 | 1.6224 | 0.1844 |
| Error | 30 | 3.0088008 | 0.100293 | | |
| C. Total | 35 | 3.8223681 | | | |

### Means for Oneway Anova

| Level | Number | Mean | Std Error | Lower 95% | Upper 95% |
|---|---|---|---|---|---|
| 50nM-NA | 6 | -0.13029 | 0.12929 | -0.3943 | 0.13375 |
| 5nM-NA | 6 | 0.21620 | 0.12929 | -0.0478 | 0.48025 |
| -NA | 6 | 0.17766 | 0.12929 | -0.0864 | 0.44170 |
| 50nM+NA | 6 | -0.00555 | 0.12929 | -0.2696 | 0.25850 |
| 5nM+NA | 6 | -0.18815 | 0.12929 | -0.4522 | 0.07589 |
| +NA | 6 | -0.06987 | 0.12929 | -0.3339 | 0.19417 |

Std Error uses a pooled estimate of error variance

## Means Comparisons

### Comparisons with a control using Dunnett's Method

## Oneway Analysis of 4-Pyridoxic acid By Label

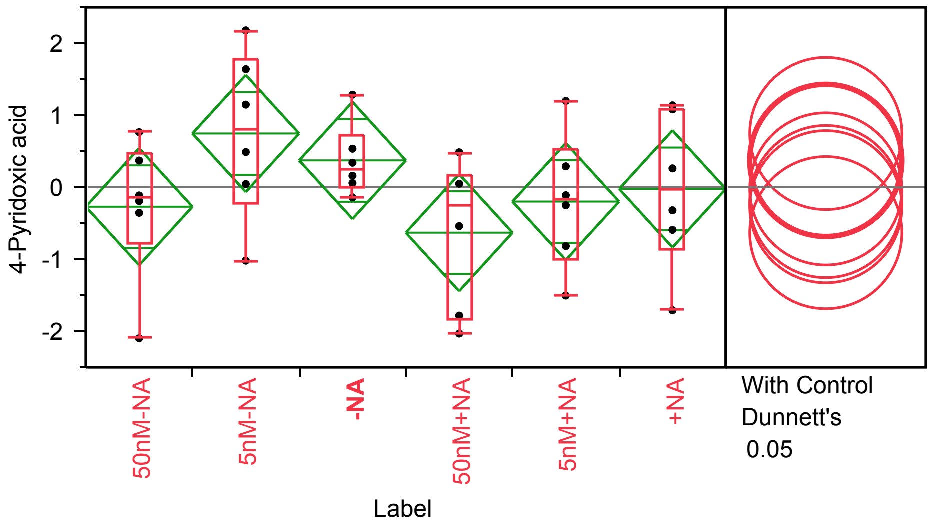


## Quantiles

| Level | Minimum | 10% | 25% | Median | 75% | 90% | Maximum |
|---|---|---|---|---|---|---|---|
| 50nM-NA | -2.09668 | -2.09668 | -0.78998 | -0.15234 | 0.470133 | 0.765591 | 0.765591 |
| 5nM-NA | -1.0189 | -1.0189 | -0.22111 | 0.818525 | 1.775289 | 2.179573 | 2.179573 |
| -NA | -0.13966 | -0.13966 | 0.009428 | 0.250177 | 0.722824 | 1.285486 | 1.285486 |
| 50nM+NA | -2.03149 | -2.03149 | -1.8446 | -0.25094 | 0.158566 | 0.485773 | 0.485773 |
| 5nM+NA | -1.50358 | -1.50358 | -0.98838 | -0.18016 | 0.518195 | 1.199578 | 1.199578 |
| +NA | -1.70761 | -1.70761 | -0.87079 | -0.02825 | 1.09678 | 1.138162 | 1.138162 |

## Oneway Anova

### Summary of Fit

| | |
|---|---|
| Rsquare | 0.202891 |
| Adj Rsquare | 0.07004 |
| Root Mean Square Error | 0.973983 |
| Mean of Response | -1.9e-17 |
| Observations (or Sum Wgts) | 36 |

### Analysis of Variance

| Source | DF | Sum of Squares | Mean Square | F Ratio | Prob > F |
|---|---|---|---|---|---|
| Label | 5 | 7.243848 | 1.44877 | 1.5272 | 0.2111 |
| Error | 30 | 28.459264 | 0.94864 | | |
| C. Total | 35 | 35.703113 | | | |

### Means for Oneway Anova

| Level | Number | Mean | Std Error | Lower 95% | Upper 95% |
|---|---|---|---|---|---|
| 50nM-NA | 6 | -0.26976 | 0.39763 | -1.082 | 0.5423 |
| 5nM-NA | 6 | 0.74718 | 0.39763 | -0.065 | 1.5592 |
| -NA | 6 | 0.37343 | 0.39763 | -0.439 | 1.1855 |
| 50nM+NA | 6 | -0.63006 | 0.39763 | -1.442 | 0.1820 |
| 5nM+NA | 6 | -0.19832 | 0.39763 | -1.010 | 0.6137 |
| +NA | 6 | -0.02247 | 0.39763 | -0.835 | 0.7896 |

Std Error uses a pooled estimate of error variance

## Means Comparisons

### Comparisons with a control using Dunnett's Method

## Oneway Analysis of 6-phospho-D-gluconate By Label

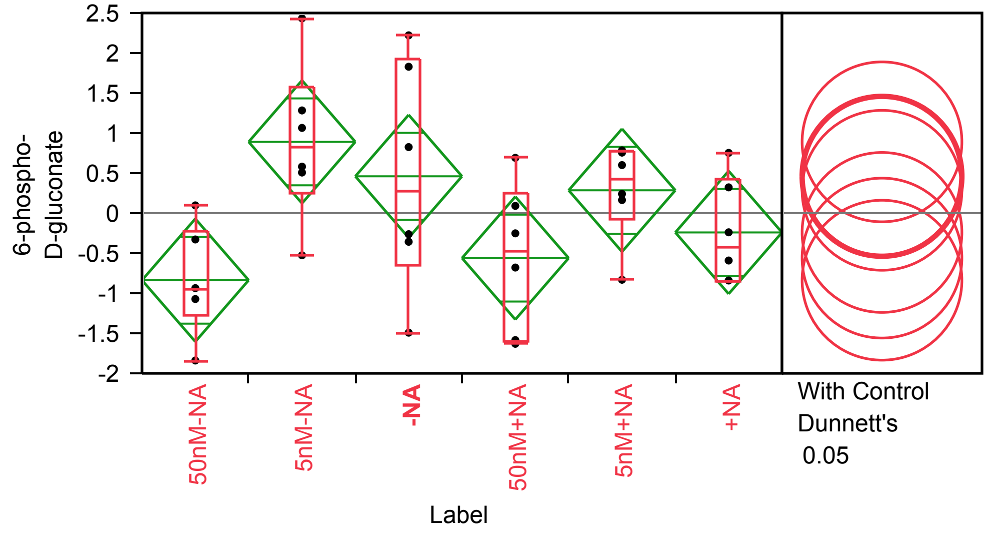


### Quantiles

| Level | Minimum | 10% | 25% | Median | 75% | 90% | Maximum |
|---|---|---|---|---|---|---|---|
| 50nM-NA | -1.83954 | -1.83954 | -1.26393 | -0.93907 | -0.22174 | 0.097472 | 0.097472 |
| 5nM-NA | -0.5255 | -0.5255 | 0.249468 | 0.8234 | 1.570698 | 2.433183 | 2.433183 |
| -NA | -1.49187 | -1.49187 | -0.64058 | 0.281691 | 1.926964 | 2.221025 | 2.221025 |
| 50nM+NA | -1.63105 | -1.63105 | -1.59637 | -0.46518 | 0.241328 | 0.691366 | 0.691366 |
| 5nM+NA | -0.83109 | -0.83109 | -0.08451 | 0.420821 | 0.763483 | 0.784206 | 0.784206 |
| +NA | -0.84769 | -0.84769 | -0.84221 | -0.41484 | 0.430294 | 0.753209 | 0.753209 |

### Oneway Anova

#### Summary of Fit

| | |
|---|---|
| Rsquare | 0.33768 |
| Adj Rsquare | 0.227293 |
| Root Mean Square Error | 0.920488 |
| Mean of Response | 2.78e-10 |
| Observations (or Sum Wgts) | 36 |

#### Analysis of Variance

| Source | DF | Sum of Squares | Mean Square | F Ratio | Prob > F |
|---|---|---|---|---|---|
| Label | 5 | 12.959677 | 2.59194 | 3.0591 | 0.0239 * |
| Error | 30 | 25.418932 | 0.84730 | | |
| C. Total | 35 | 38.378608 | | | |

#### Means for Oneway Anova

| Level | Number | Mean | Std Error | Lower 95% | Upper 95% |
|---|---|---|---|---|---|
| 50nM-NA | 6 | -0.83673 | 0.37579 | -1.604 | -0.069 |
| 5nM-NA | 6 | 0.89091 | 0.37579 | 0.123 | 1.658 |
| -NA | 6 | 0.46078 | 0.37579 | -0.307 | 1.228 |
| 50nM+NA | 6 | -0.56059 | 0.37579 | -1.328 | 0.207 |
| 5nM+NA | 6 | 0.28595 | 0.37579 | -0.482 | 1.053 |
| +NA | 6 | -0.24031 | 0.37579 | -1.008 | 0.527 |

Std Error uses a pooled estimate of error variance

### Means Comparisons

#### Comparisons with a control using Dunnett's Method

## Oneway Analysis of 6-phospho-D-glucono-1,5-lactone By Label

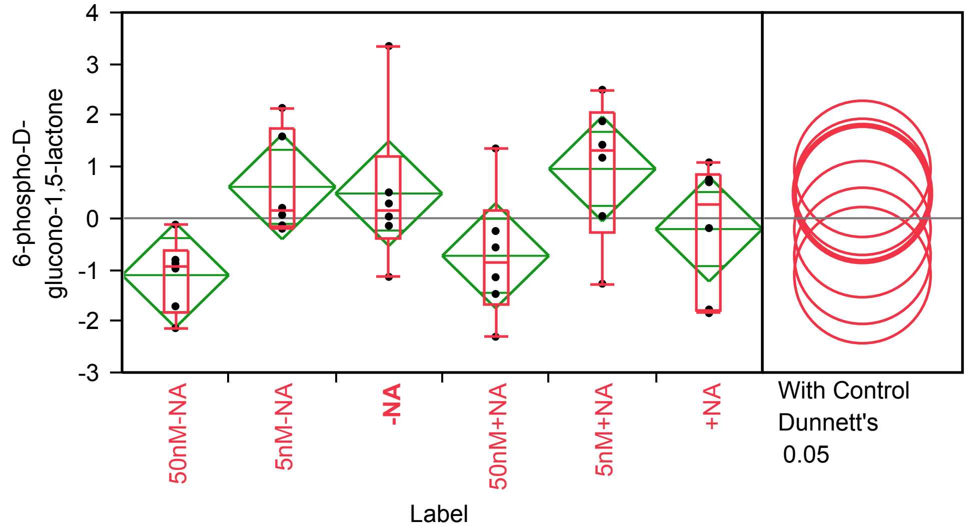


### Quantiles

| Level | Minimum | 10% | 25% | Median | 75% | 90% | Maximum |
|---|---|---|---|---|---|---|---|
| 50nM-NA | -2.13711 | -2.13711 | -1.81884 | -0.92865 | -0.63815 | -0.12513 | -0.12513 |
| 5nM-NA | -0.20387 | -0.20387 | -0.15473 | 0.133062 | 1.72876 | 2.144123 | 2.144123 |
| -NA | -1.14043 | -1.14043 | -0.3954 | 0.159516 | 1.21373 | 3.347747 | 3.347747 |
| 50nM+NA | -2.30485 | -2.30485 | -1.68085 | -0.85736 | 0.152372 | 1.356705 | 1.356705 |
| 5nM+NA | -1.27281 | -1.27281 | -0.28658 | 1.301995 | 2.03819 | 2.496518 | 2.496518 |
| +NA | -1.84528 | -1.84528 | -1.79275 | 0.253843 | 0.840071 | 1.085525 | 1.085525 |

### Oneway Anova

#### Summary of Fit

| | |
|---|---|
| Rsquare | 0.308547 |
| Adj Rsquare | 0.193305 |
| Root Mean Square Error | 1.221051 |
| Mean of Response | -3.1e-17 |
| Observations (or Sum Wgts) | 36 |

#### Analysis of Variance

| Source | DF | Sum of Squares | Mean Square | F Ratio | Prob > F |
|---|---|---|---|---|---|
| Label | 5 | 19.959405 | 3.99188 | 2.6774 | 0.0408 * |
| Error | 30 | 44.728993 | 1.49097 | | |
| C. Total | 35 | 64.688398 | | | |

#### Means for Oneway Anova

| Level | Number | Mean | Std Error | Lower 95% | Upper 95% |
|---|---|---|---|---|---|
| 50nM-NA | 6 | -1.1069 | 0.49849 | -2.125 | -0.089 |
| 5nM-NA | 6 | 0.6097 | 0.49849 | -0.408 | 1.628 |
| -NA | 6 | 0.4803 | 0.49849 | -0.538 | 1.498 |
| 50nM+NA | 6 | -0.7308 | 0.49849 | -1.749 | 0.287 |
| 5nM+NA | 6 | 0.9592 | 0.49849 | -0.059 | 1.977 |
| +NA | 6 | -0.2115 | 0.49849 | -1.230 | 0.807 |

Std Error uses a pooled estimate of error variance

### Means Comparisons

#### Comparisons with a control using Dunnett's Method

## Oneway Analysis of 7-methylguanosine By Label

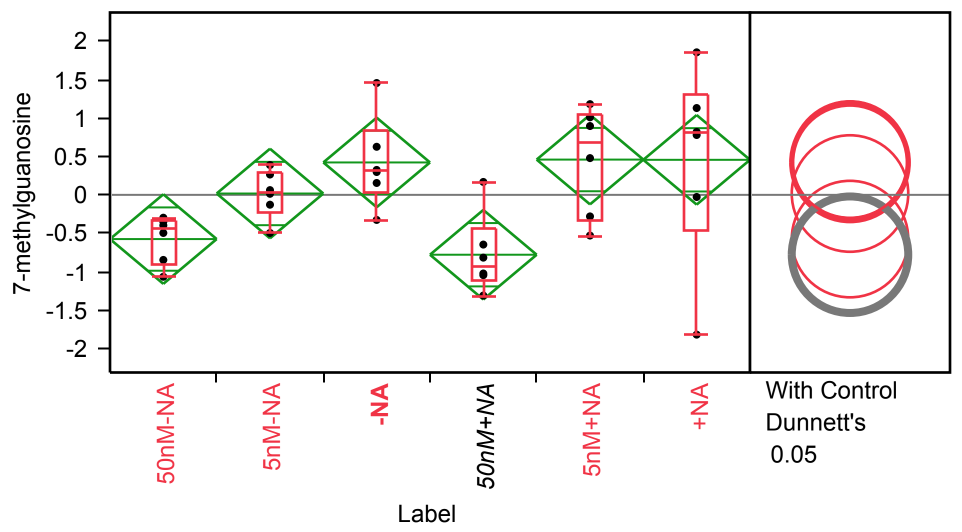


## Quantiles

| Level | Minimum | 10% | 25% | Median | 75% | 90% | Maximum |
|---|---|---|---|---|---|---|---|
| 50nM-NA | -1.06775 | -1.06775 | -0.90331 | -0.44752 | -0.33656 | -0.29791 | -0.29791 |
| 5nM-NA | -0.4967 | -0.4967 | -0.22045 | 0.0379 | 0.296151 | 0.389088 | 0.389088 |
| -NA | -0.32675 | -0.32675 | 0.034621 | 0.311469 | 0.834644 | 1.45811 | 1.45811 |
| 50nM+NA | -1.31248 | -1.31248 | -1.1134 | -0.92198 | -0.44405 | 0.168313 | 0.168313 |
| 5nM+NA | -0.53041 | -0.53041 | -0.3433 | 0.688308 | 1.054113 | 1.182878 | 1.182878 |
| +NA | -1.82244 | -1.82244 | -0.47633 | 0.800548 | 1.313654 | 1.858333 | 1.858333 |

## Oneway Anova

### Summary of Fit

| | |
|---|---|
| Rsquare | 0.385926 |
| Adj Rsquare | 0.283581 |
| Root Mean Square Error | 0.700239 |
| Mean of Response | 5.56e-10 |
| Observations (or Sum Wgts) | 36 |

### Analysis of Variance

| Source | DF | Sum of Squares | Mean Square | F Ratio | Prob > F |
|---|---|---|---|---|---|
| Label | 5 | 9.244811 | 1.84896 | 3.7708 | 0.0091 * |
| Error | 30 | 14.710041 | 0.49033 | | |
| C. Total | 35 | 23.954852 | | | |

### Means for Oneway Anova

| Level | Number | Mean | Std Error | Lower 95% | Upper 95% |
|---|---|---|---|---|---|
| 50nM-NA | 6 | -0.57644 | 0.28587 | -1.160 | 0.00739 |
| 5nM-NA | 6 | 0.01750 | 0.28587 | -0.566 | 0.601 |
| -NA | 6 | 0.42270 | 0.28587 | -0.161 | 1.007 |
| 50nM+NA | 6 | -0.78056 | 0.28587 | -1.364 | -0.197 |
| 5nM+NA | 6 | 0.45989 | 0.28587 | -0.124 | 1.044 |
| +NA | 6 | 0.45691 | 0.28587 | -0.127 | 1.041 |

Std Error uses a pooled estimate of error variance

## Means Comparisons

### Comparisons with a control using Dunnett's Method

## Oneway Analysis of acetoacetate By Label

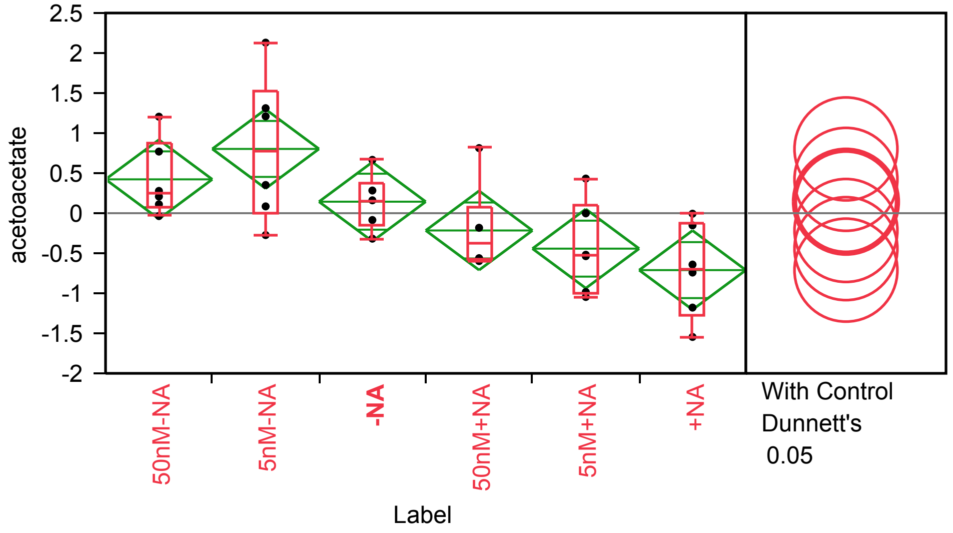


### Quantiles

| Level | Minimum | 10% | 25% | Median | 75% | 90% | Maximum |
|---|---|---|---|---|---|---|---|
| 50nM-NA | -0.03569 | -0.03569 | 0.074264 | 0.243195 | 0.878597 | 1.205337 | 1.205337 |
| 5nM-NA | -0.27308 | -0.27308 | -0.00449 | 0.78114 | 1.516133 | 2.129953 | 2.129953 |
| -NA | -0.31787 | -0.31787 | -0.14342 | 0.157949 | 0.379028 | 0.666075 | 0.666075 |
| 50nM+NA | -0.59755 | -0.59755 | -0.57936 | -0.37716 | 0.066705 | 0.812989 | 0.812989 |
| 5nM+NA | -1.04604 | -1.04604 | -0.99947 | -0.5279 | 0.106561 | 0.4325 | 0.4325 |
| +NA | -1.54537 | -1.54537 | -1.2705 | -0.69131 | -0.11534 | -0.00637 | -0.00637 |

### Oneway Anova

#### Summary of Fit

| | |
|---|---|
| Rsquare | 0.475246 |
| Adj Rsquare | 0.387787 |
| Root Mean Square Error | 0.592791 |
| Mean of Response | 5.56e-11 |
| Observations (or Sum Wgts) | 36 |

#### Analysis of Variance

| Source | DF | Sum of Squares | Mean Square | F Ratio | Prob > F |
|---|---|---|---|---|---|
| Label | 5 | 9.547441 | 1.90949 | 5.4339 | 0.0011 * |
| Error | 30 | 10.542044 | 0.35140 | | |
| C. Total | 35 | 20.089485 | | | |

#### Means for Oneway Anova

| Level | Number | Mean | Std Error | Lower 95% | Upper 95% |
|---|---|---|---|---|---|
| 50nM-NA | 6 | 0.42277 | 0.24201 | -0.071 | 0.917 |
| 5nM-NA | 6 | 0.80262 | 0.24201 | 0.308 | 1.297 |
| -NA | 6 | 0.14370 | 0.24201 | -0.351 | 0.638 |
| 50nM+NA | 6 | -0.21571 | 0.24201 | -0.710 | 0.279 |
| 5nM+NA | 6 | -0.44256 | 0.24201 | -0.937 | 0.052 |
| +NA | 6 | -0.71082 | 0.24201 | -1.205 | -0.217 |

Std Error uses a pooled estimate of error variance

### Means Comparisons

#### Comparisons with a control using Dunnett's Method

## Oneway Analysis of Acetylcarnitine By Label

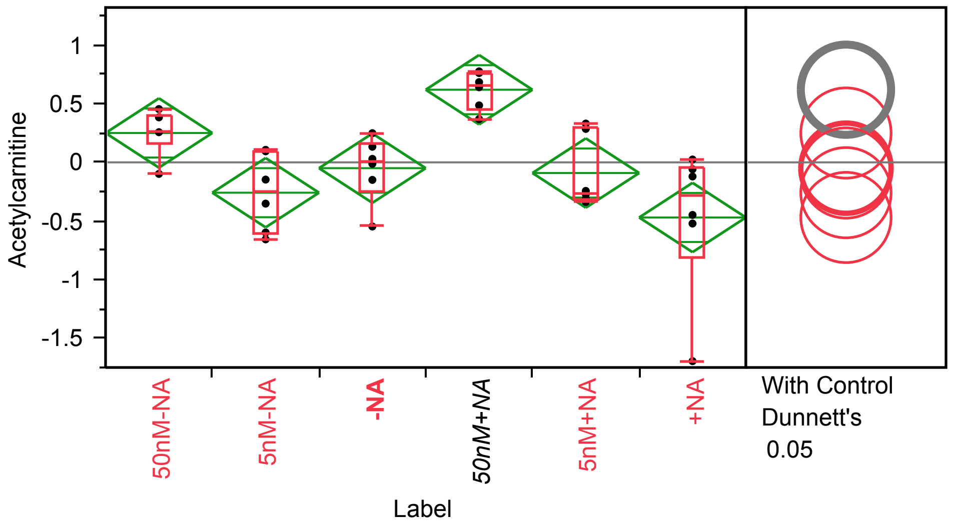


## Quantiles

| Level | Minimum | 10% | 25% | Median | 75% | 90% | Maximum |
|---|---|---|---|---|---|---|---|
| 50nM-NA | -0.09849 | -0.09849 | 0.162544 | 0.256481 | 0.401422 | 0.454408 | 0.454408 |
| 5nM-NA | -0.65681 | -0.65681 | -0.61309 | -0.2496 | 0.09747 | 0.106517 | 0.106517 |
| -NA | -0.54722 | -0.54722 | -0.24907 | 0.008503 | 0.162 | 0.248556 | 0.248556 |
| 50nM+NA | 0.368979 | 0.368979 | 0.45669 | 0.662367 | 0.765262 | 0.775754 | 0.775754 |
| 5nM+NA | -0.33761 | -0.33761 | -0.31625 | -0.25767 | 0.296632 | 0.33058 | 0.33058 |
| +NA | -1.69575 | -1.69575 | -0.81534 | -0.28451 | -0.03812 | 0.024084 | 0.024084 |

## Oneway Anova

### Summary of Fit

| | |
|---|---|
| Rsquare | 0.542182 |
| Adj Rsquare | 0.465879 |
| Root Mean Square Error | 0.354804 |
| Mean of Response | -2.8e-10 |
| Observations (or Sum Wgts) | 36 |

### Analysis of Variance

| Source | DF | Sum of Squares | Mean Square | F Ratio | Prob > F |
|---|---|---|---|---|---|
| Label | 5 | 4.4724954 | 0.894499 | 7.1057 | 0.0002 * |
| Error | 30 | 3.7765682 | 0.125886 | | |
| C. Total | 35 | 8.2490637 | | | |

### Means for Oneway Anova

| Level | Number | Mean | Std Error | Lower 95% | Upper 95% |
|---|---|---|---|---|---|
| 50nM-NA | 6 | 0.25037 | 0.14485 | -0.0455 | 0.5462 |
| 5nM-NA | 6 | -0.25893 | 0.14485 | -0.5547 | 0.0369 |
| -NA | 6 | -0.04970 | 0.14485 | -0.3455 | 0.2461 |
| 50nM+NA | 6 | 0.61953 | 0.14485 | 0.3237 | 0.9153 |
| 5nM+NA | 6 | -0.09103 | 0.14485 | -0.3868 | 0.2048 |
| +NA | 6 | -0.47023 | 0.14485 | -0.7661 | -0.1744 |

Std Error uses a pooled estimate of error variance

## Means Comparisons

### Comparisons with a control using Dunnett's Method

## Oneway Analysis of acetyl-CoA By Label

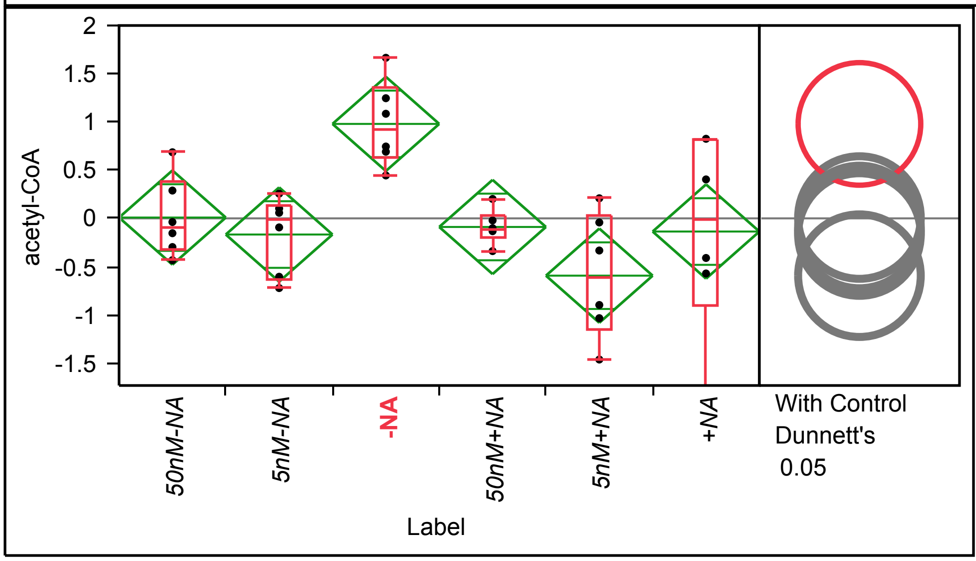


### Quantiles

| Level | Minimum | 10% | 25% | Median | 75% | 90% | Maximum |
|---|---|---|---|---|---|---|---|
| 50nM-NA | -0.43338 | -0.43338 | -0.33154 | -0.09612 | 0.387605 | 0.685471 | 0.685471 |
| 5nM-NA | -0.72285 | -0.72285 | -0.63503 | -0.01813 | 0.140952 | 0.256186 | 0.256186 |
| -NA | 0.445089 | 0.445089 | 0.629514 | 0.914786 | 1.350688 | 1.665058 | 1.665058 |
| 50nM+NA | -0.3395 | -0.3395 | -0.1905 | -0.11759 | 0.034405 | 0.201147 | 0.201147 |
| 5nM+NA | -1.46387 | -1.46387 | -1.14238 | -0.61604 | 0.020031 | 0.208098 | 0.208098 |
| +NA | -1.88359 | -1.88359 | -0.89974 | -0.00355 | 0.819459 | 0.825145 | 0.825145 |

### Oneway Anova

#### Summary of Fit

| | |
|---|---|
| Rsquare | 0.443389 |
| Adj Rsquare | 0.35062 |
| Root Mean Square Error | 0.585908 |
| Mean of Response | 2.78e-10 |
| Observations (or Sum Wgts) | 36 |

#### Analysis of Variance

| Source | DF | Sum of Squares | Mean Square | F Ratio | Prob > F |
|---|---|---|---|---|---|
| Label | 5 | 8.203755 | 1.64075 | 4.7795 | 0.0025 * |
| Error | 30 | 10.298640 | 0.34329 | | |
| C. Total | 35 | 18.502395 | | | |

#### Means for Oneway Anova

| Level | Number | Mean | Std Error | Lower 95% | Upper 95% |
|---|---|---|---|---|---|
| 50nM-NA | 6 | 0.00843 | 0.23920 | -0.480 | 0.497 |
| 5nM-NA | 6 | -0.16769 | 0.23920 | -0.656 | 0.321 |
| -NA | 6 | 0.97943 | 0.23920 | 0.491 | 1.468 |
| 50nM+NA | 6 | -0.08926 | 0.23920 | -0.578 | 0.399 |
| 5nM+NA | 6 | -0.59429 | 0.23920 | -1.083 | -0.106 |
| +NA | 6 | -0.13663 | 0.23920 | -0.625 | 0.352 |

Std Error uses a pooled estimate of error variance

### Means Comparisons

#### Comparisons with a control using Dunnett's Method

## Oneway Analysis of Acetyllysine By Label

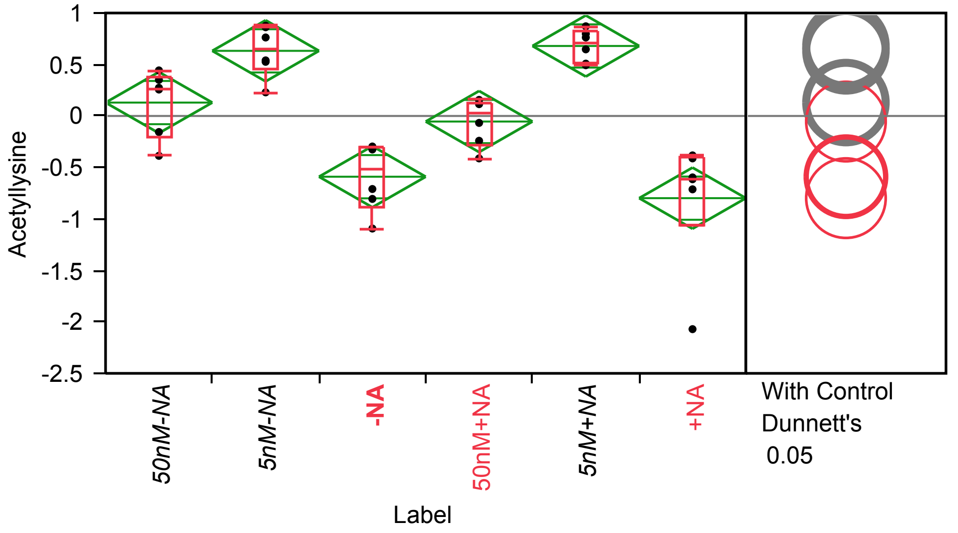


## Quantiles

| Level | Minimum | 10% | 25% | Median | 75% | 90% | Maximum |
|---|---|---|---|---|---|---|---|
| 50nM-NA | -0.38694 | -0.38694 | -0.21368 | 0.265195 | 0.373401 | 0.442474 | 0.442474 |
| 5nM-NA | 0.229527 | 0.229527 | 0.451838 | 0.65107 | 0.86542 | 0.876003 | 0.876003 |
| -NA | -1.09332 | -1.09332 | -0.87756 | -0.51737 | -0.30672 | -0.29681 | -0.29681 |
| 50nM+NA | -0.41205 | -0.41205 | -0.28384 | 0.023745 | 0.13298 | 0.155985 | 0.155985 |
| 5nM+NA | 0.492905 | 0.492905 | 0.504225 | 0.704768 | 0.815861 | 0.870952 | 0.870952 |
| +NA | -2.07068 | -2.07068 | -1.0521 | -0.60576 | -0.40476 | -0.38314 | -0.38314 |

## Oneway Anova

### Summary of Fit

| | |
|---|---|
| Rsquare | 0.746537 |
| Adj Rsquare | 0.704294 |
| Root Mean Square Error | 0.356118 |
| Mean of Response | 8.33e-10 |
| Observations (or Sum Wgts) | 36 |

### Analysis of Variance

| Source | DF | Sum of Squares | Mean Square | F Ratio | Prob > F |
|---|---|---|---|---|---|
| Label | 5 | 11.205929 | 2.24119 | 17.6721 | <.0001 * |
| Error | 30 | 3.804610 | 0.12682 | | |
| C. Total | 35 | 15.010539 | | | |

### Means for Oneway Anova

| Level | Number | Mean | Std Error | Lower 95% | Upper 95% |
|---|---|---|---|---|---|
| 50nM-NA | 6 | 0.13006 | 0.14538 | -0.167 | 0.4270 |
| 5nM-NA | 6 | 0.63258 | 0.14538 | 0.336 | 0.9295 |
| -NA | 6 | -0.59009 | 0.14538 | -0.887 | -0.2932 |
| 50nM+NA | 6 | -0.05406 | 0.14538 | -0.351 | 0.2429 |
| 5nM+NA | 6 | 0.67981 | 0.14538 | 0.383 | 0.9767 |
| +NA | 6 | -0.79831 | 0.14538 | -1.095 | -0.5014 |

Std Error uses a pooled estimate of error variance

## Means Comparisons

### Comparisons with a control using Dunnett's Method

## Oneway Analysis of aconitate By Label

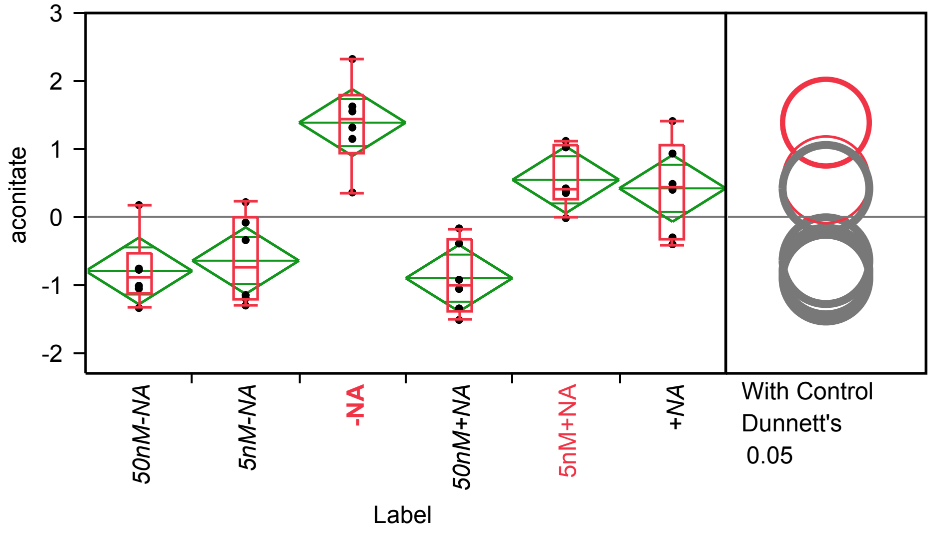


## Quantiles

| Level | Minimum | 10% | 25% | Median | 75% | 90% | Maximum |
|---|---|---|---|---|---|---|---|
| 50nM-NA | -1.34334 | -1.34334 | -1.12878 | -0.89842 | -0.52961 | 0.171812 | 0.171812 |
| 5nM-NA | -1.30649 | -1.30649 | -1.22845 | -0.74918 | -0.01076 | 0.215249 | 0.215249 |
| -NA | 0.358004 | 0.358004 | 0.94953 | 1.432818 | 1.798408 | 2.322271 | 2.322271 |
| 50nM+NA | -1.51932 | -1.51932 | -1.3919 | -0.99527 | -0.33742 | -0.17085 | -0.17085 |
| 5nM+NA | -0.01955 | -0.01955 | 0.257829 | 0.396963 | 1.045656 | 1.115989 | 1.115989 |
| +NA | -0.40465 | -0.40465 | -0.32951 | 0.440378 | 1.050314 | 1.408354 | 1.408354 |

## Oneway Anova

### Summary of Fit

| | |
|---|---|
| Rsquare | 0.71119 |
| Adj Rsquare | 0.663055 |
| Root Mean Square Error | 0.588476 |
| Mean of Response | 2.47e-17 |
| Observations (or Sum Wgts) | 36 |

### Analysis of Variance

| Source | DF | Sum of Squares | Mean Square | F Ratio | Prob > F |
|---|---|---|---|---|---|
| Label | 5 | 25.583035 | 5.11661 | 14.7749 | <.0001 * |
| Error | 30 | 10.389120 | 0.34630 | | |
| C. Total | 35 | 35.972156 | | | |

### Means for Oneway Anova

| Level | Number | Mean | Std Error | Lower 95% | Upper 95% |
|---|---|---|---|---|---|
| 50nM-NA | 6 | -0.7982 | 0.24024 | -1.289 | -0.308 |
| 5nM-NA | 6 | -0.6464 | 0.24024 | -1.137 | -0.156 |
| -NA | 6 | 1.3861 | 0.24024 | 0.895 | 1.877 |
| 50nM+NA | 6 | -0.9038 | 0.24024 | -1.394 | -0.413 |
| 5nM+NA | 6 | 0.5438 | 0.24024 | 0.053 | 1.034 |
| +NA | 6 | 0.4185 | 0.24024 | -0.072 | 0.909 |

Std Error uses a pooled estimate of error variance

## Means Comparisons

### Comparisons with a control using Dunnett's Method

## Oneway Analysis of Adenine By Label

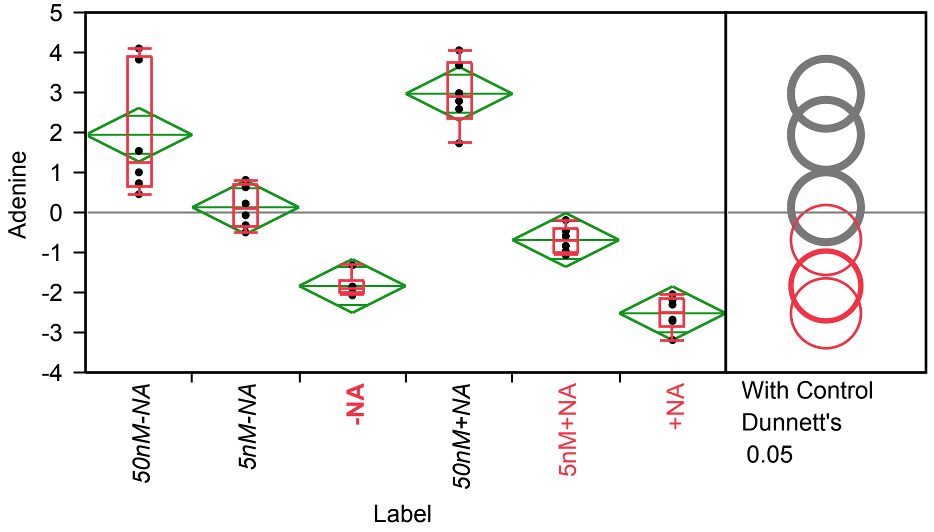


### Quantiles

| Level | Minimum | 10% | 25% | Median | 75% | 90% | Maximum |
|---|---|---|---|---|---|---|---|
| 50nM-NA | 0.456816 | 0.456816 | 0.663089 | 1.271783 | 3.892978 | 4.100561 | 4.100561 |
| 5nM-NA | -0.49656 | -0.49656 | -0.36343 | 0.077191 | 0.677009 | 0.814795 | 0.814795 |
| -NA | -2.07141 | -2.07141 | -2.02251 | -1.88688 | -1.71827 | -1.3169 | -1.3169 |
| 50nM+NA | 1.730777 | 1.730777 | 2.372974 | 2.884047 | 3.771701 | 4.054288 | 4.054288 |
| 5nM+NA | -1.06602 | -1.06602 | -0.99464 | -0.71406 | -0.39686 | -0.18963 | -0.18963 |
| +NA | -3.18796 | -3.18796 | -2.83123 | -2.49152 | -2.15351 | -2.04506 | -2.04506 |

### Oneway Anova

#### Summary of Fit

| | |
|---|---|
| Rsquare | 0.875433 |
| Adj Rsquare | 0.854672 |
| Root Mean Square Error | 0.805582 |
| Mean of Response | -8.33e-9 |
| Observations (or Sum Wgts) | 36 |

#### Analysis of Variance

| Source | DF | Sum of Squares | Mean Square | F Ratio | Prob > F |
|---|---|---|---|---|---|
| Label | 5 | 136.82390 | 27.3648 | 42.1670 | <.0001 * |
| Error | 30 | 19.46886 | 0.6490 | | |
| C. Total | 35 | 156.29276 | | | |

#### Means for Oneway Anova

| Level | Number | Mean | Std Error | Lower 95% | Upper 95% |
|---|---|---|---|---|---|
| 50nM-NA | 6 | 1.9428 | 0.32888 | 1.271 | 2.614 |
| 5nM-NA | 6 | 0.1308 | 0.32888 | -0.541 | 0.802 |
| -NA | 6 | -1.8367 | 0.32888 | -2.508 | -1.165 |
| 50nM+NA | 6 | 2.9696 | 0.32888 | 2.298 | 3.641 |
| 5nM+NA | 6 | -0.6868 | 0.32888 | -1.358 | -0.015 |
| +NA | 6 | -2.5197 | 0.32888 | -3.191 | -1.848 |

Std Error uses a pooled estimate of error variance

### Means Comparisons

#### Comparisons with a control using Dunnett's Method

## Oneway Analysis of adenosine By Label

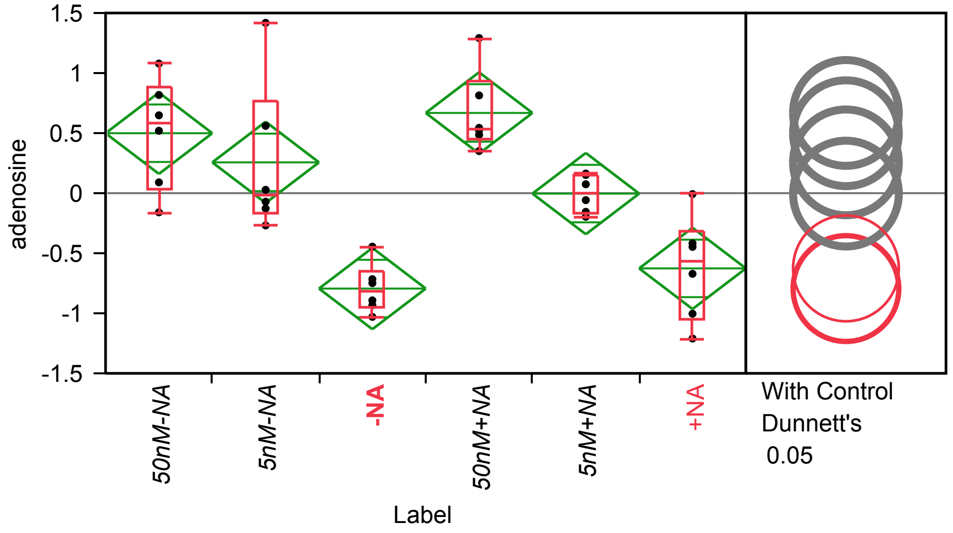


## Quantiles

| Level | Minimum | 10% | 25% | Median | 75% | 90% | Maximum |
|---|---|---|---|---|---|---|---|
| 50nM-NA | -0.15926 | -0.15926 | 0.027575 | 0.583929 | 0.883014 | 1.07912 | 1.07912 |
| 5nM-NA | -0.26829 | -0.26829 | -0.16287 | -0.02154 | 0.774911 | 1.417908 | 1.417908 |
| -NA | -1.0297 | -1.0297 | -0.95696 | -0.82047 | -0.64835 | -0.44538 | -0.44538 |
| 50nM+NA | 0.350952 | 0.350952 | 0.451802 | 0.533269 | 0.933369 | 1.291286 | 1.291286 |
| 5nM+NA | -0.19646 | -0.19646 | -0.16586 | 0.008241 | 0.154536 | 0.163279 | 0.163279 |
| +NA | -1.20978 | -1.20978 | -1.05524 | -0.55919 | -0.31527 | -0.0077 | -0.0077 |

## Oneway Anova

### Summary of Fit

| | |
|---|---|
| Rsquare | 0.684054 |
| Adj Rsquare | 0.631397 |
| Root Mean Square Error | 0.405964 |
| Mean of Response | -1.2e-17 |
| Observations (or Sum Wgts) | 36 |

### Analysis of Variance

| Source | DF | Sum of Squares | Mean Square | F Ratio | Prob > F |
|---|---|---|---|---|---|
| Label | 5 | 10.704728 | 2.14095 | 12.9906 | <.0001 * |
| Error | 30 | 4.944215 | 0.16481 | | |
| C. Total | 35 | 15.648943 | | | |

### Means for Oneway Anova

| Level | Number | Mean | Std Error | Lower 95% | Upper 95% |
|---|---|---|---|---|---|
| 50nM-NA | 6 | 0.49920 | 0.16573 | 0.161 | 0.838 |
| 5nM-NA | 6 | 0.25656 | 0.16573 | -0.082 | 0.595 |
| -NA | 6 | -0.79413 | 0.16573 | -1.133 | -0.456 |
| 50nM+NA | 6 | 0.66804 | 0.16573 | 0.330 | 1.007 |
| 5nM+NA | 6 | -0.00346 | 0.16573 | -0.342 | 0.335 |
| +NA | 6 | -0.62623 | 0.16573 | -0.965 | -0.288 |

Std Error uses a pooled estimate of error variance

## Means Comparisons

### Comparisons with a control using Dunnett's Method

## Oneway Analysis of Adipic acid By Label

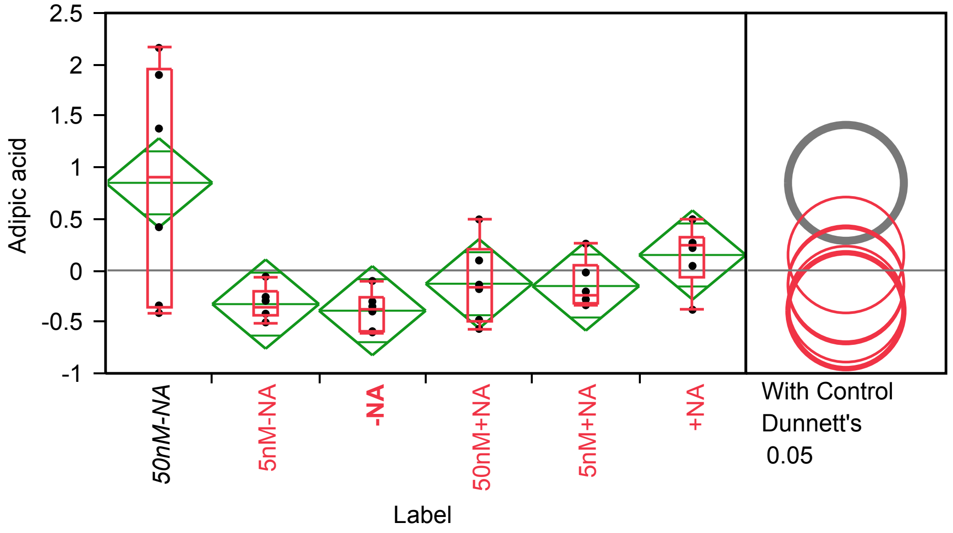


## Quantiles

| Level | Minimum | 10% | 25% | Median | 75% | 90% | Maximum |
|---|---|---|---|---|---|---|---|
| 50nM-NA | -0.41373 | -0.41373 | -0.35906 | 0.898748 | 1.96434 | 2.161242 | 2.161242 |
| 5nM-NA | -0.50494 | -0.50494 | -0.44572 | -0.35806 | -0.20813 | -0.06097 | -0.06097 |
| -NA | -0.6022 | -0.6022 | -0.59415 | -0.37466 | -0.25253 | -0.10269 | -0.10269 |
| 50nM+NA | -0.56807 | -0.56807 | -0.50244 | -0.15998 | 0.196263 | 0.495227 | 0.495227 |
| 5nM+NA | -0.33826 | -0.33826 | -0.32869 | -0.24299 | 0.049614 | 0.259356 | 0.259356 |
| +NA | -0.3822 | -0.3822 | -0.06291 | 0.235881 | 0.325292 | 0.496698 | 0.496698 |

## Oneway Anova

### Summary of Fit

| | |
|---|---|
| Rsquare | 0.437256 |
| Adj Rsquare | 0.343466 |
| Root Mean Square Error | 0.518869 |
| Mean of Response | -2.8e-10 |
| Observations (or Sum Wgts) | 36 |

### Analysis of Variance

| Source | DF | Sum of Squares | Mean Square | F Ratio | Prob > F |
|---|---|---|---|---|---|
| Label | 5 | 6.275702 | 1.25514 | 4.6620 | 0.0029 * |
| Error | 30 | 8.076754 | 0.26923 | | |
| C. Total | 35 | 14.352456 | | | |

### Means for Oneway Anova

| Level | Number | Mean | Std Error | Lower 95% | Upper 95% |
|---|---|---|---|---|---|
| 50nM-NA | 6 | 0.85048 | 0.21183 | 0.4179 | 1.2831 |
| 5nM-NA | 6 | -0.32753 | 0.21183 | -0.7601 | 0.1051 |
| -NA | 6 | -0.39136 | 0.21183 | -0.8240 | 0.0412 |
| 50nM+NA | 6 | -0.12946 | 0.21183 | -0.5621 | 0.3031 |
| 5nM+NA | 6 | -0.15178 | 0.21183 | -0.5844 | 0.2808 |
| +NA | 6 | 0.14966 | 0.21183 | -0.2830 | 0.5823 |

Std Error uses a pooled estimate of error variance

## Means Comparisons

### Comparisons with a control using Dunnett's Method

## Oneway Analysis of ADP By Label

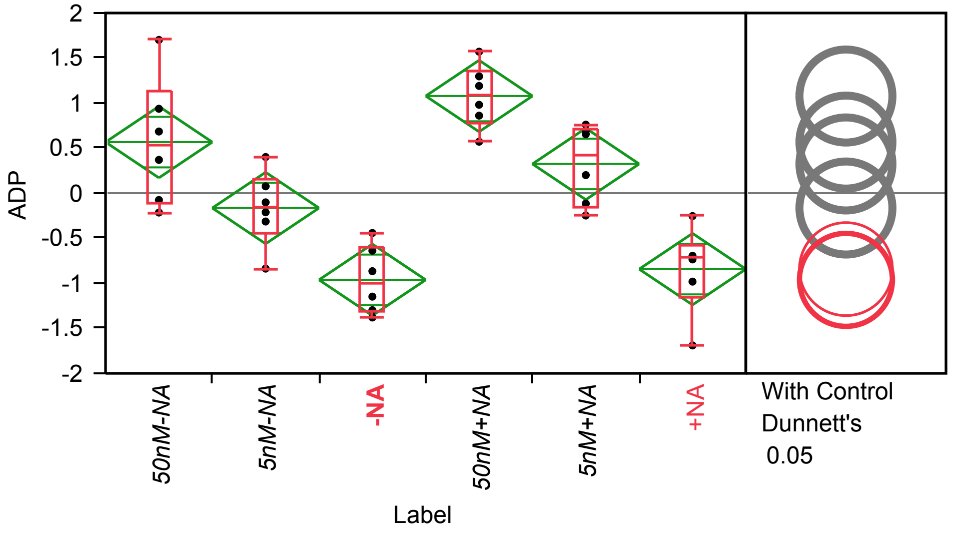


## Quantiles

| Level | Minimum | 10% | 25% | Median | 75% | 90% | Maximum |
|---|---|---|---|---|---|---|---|
| 50nM-NA | -0.21488 | -0.21488 | -0.11034 | 0.527025 | 1.128126 | 1.701416 | 1.701416 |
| 5nM-NA | -0.83739 | -0.83739 | -0.44526 | -0.15701 | 0.157366 | 0.401352 | 0.401352 |
| -NA | -1.37986 | -1.37986 | -1.31902 | -1.00715 | -0.58914 | -0.4418 | -0.4418 |
| 50nM+NA | 0.571568 | 0.571568 | 0.788331 | 1.085865 | 1.365217 | 1.570461 | 1.570461 |
| 5nM+NA | -0.248 | -0.248 | -0.14881 | 0.427756 | 0.710731 | 0.76027 | 0.76027 |
| +NA | -1.68996 | -1.68996 | -1.15896 | -0.71827 | -0.58503 | -0.25426 | -0.25426 |

## Oneway Anova

### Summary of Fit

| | |
|---|---|
| Rsquare | 0.741841 |
| Adj Rsquare | 0.698814 |
| Root Mean Square Error | 0.475859 |
| Mean of Response | -2.8e-10 |
| Observations (or Sum Wgts) | 36 |

### Analysis of Variance

| Source | DF | Sum of Squares | Mean Square | F Ratio | Prob > F |
|---|---|---|---|---|---|
| Label | 5 | 19.520957 | 3.90419 | 17.2415 | <.0001 * |
| Error | 30 | 6.793253 | 0.22644 | | |
| C. Total | 35 | 26.314211 | | | |

### Means for Oneway Anova

| Level | Number | Mean | Std Error | Lower 95% | Upper 95% |
|---|---|---|---|---|---|
| 50nM-NA | 6 | 0.5670 | 0.19427 | 0.170 | 0.964 |
| 5nM-NA | 6 | -0.1648 | 0.19427 | -0.562 | 0.232 |
| -NA | 6 | -0.9622 | 0.19427 | -1.359 | -0.565 |
| 50nM+NA | 6 | 1.0785 | 0.19427 | 0.682 | 1.475 |
| 5nM+NA | 6 | 0.3244 | 0.19427 | -0.072 | 0.721 |
| +NA | 6 | -0.8430 | 0.19427 | -1.240 | -0.446 |

Std Error uses a pooled estimate of error variance

## Means Comparisons

### Comparisons with a control using Dunnett's Method

## Oneway Analysis of AICAR 3,5-Cyclic Phosphate By Label

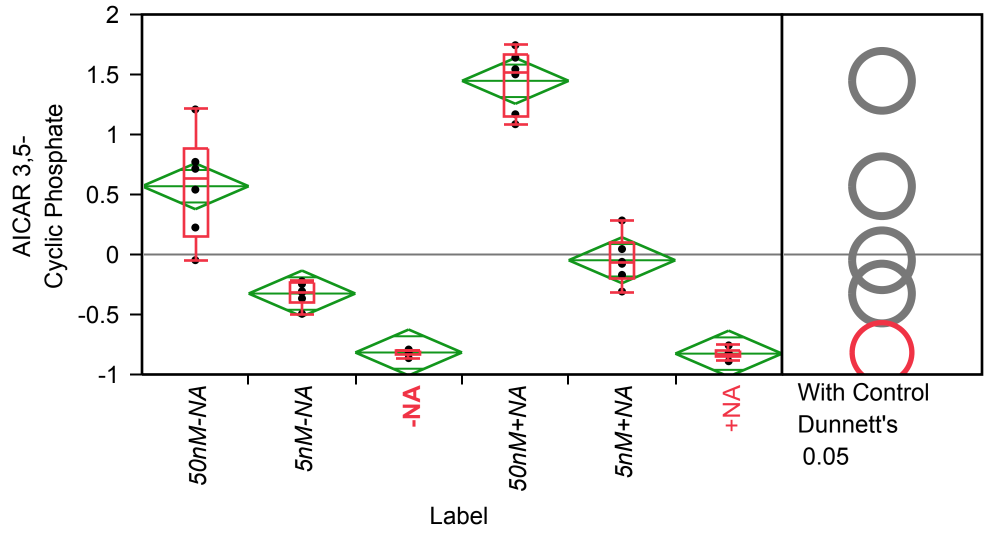


### Quantiles

| Level | Minimum | 10% | 25% | Median | 75% | 90% | Maximum |
|---|---|---|---|---|---|---|---|
| 50nM-NA | -0.04722 | -0.04722 | 0.156797 | 0.627453 | 0.88099 | 1.208936 | 1.208936 |
| 5nM-NA | -0.49429 | -0.49429 | -0.39834 | -0.31096 | -0.24046 | -0.22362 | -0.22362 |
| -NA | -0.86358 | -0.86358 | -0.83586 | -0.80684 | -0.80092 | -0.79207 | -0.79207 |
| 50nM+NA | 1.085752 | 1.085752 | 1.147775 | 1.522403 | 1.666335 | 1.743915 | 1.743915 |
| 5nM+NA | -0.30837 | -0.30837 | -0.20452 | -0.06924 | 0.104874 | 0.283546 | 0.283546 |
| +NA | -0.88942 | -0.88942 | -0.84976 | -0.82572 | -0.80558 | -0.75758 | -0.75758 |

### Oneway Anova

#### Summary of Fit

| | |
|---|---|
| Rsquare | 0.936436 |
| Adj Rsquare | 0.925841 |
| Root Mean Square Error | 0.229377 |
| Mean of Response | -5.6e-10 |
| Observations (or Sum Wgts) | 36 |

#### Analysis of Variance

| Source | DF | Sum of Squares | Mean Square | F Ratio | Prob > F |
|---|---|---|---|---|---|
| Label | 5 | 23.253395 | 4.65068 | 88.3924 | <.0001 * |
| Error | 30 | 1.578421 | 0.05261 | | |
| C. Total | 35 | 24.831816 | | | |

#### Means for Oneway Anova

| Level | Number | Mean | Std Error | Lower 95% | Upper 95% |
|---|---|---|---|---|---|
| 50nM-NA | 6 | 0.5689 | 0.09364 | 0.378 | 0.760 |
| 5nM-NA | 6 | -0.3254 | 0.09364 | -0.517 | -0.134 |
| -NA | 6 | -0.8166 | 0.09364 | -1.008 | -0.625 |
| 50nM+NA | 6 | 1.4472 | 0.09364 | 1.256 | 1.638 |
| 5nM+NA | 6 | -0.0480 | 0.09364 | -0.239 | 0.143 |
| +NA | 6 | -0.8261 | 0.09364 | -1.017 | -0.635 |

Std Error uses a pooled estimate of error variance

### Means Comparisons

#### Comparisons with a control using Dunnett's Method

## Oneway Analysis of Ala-Ala By Label

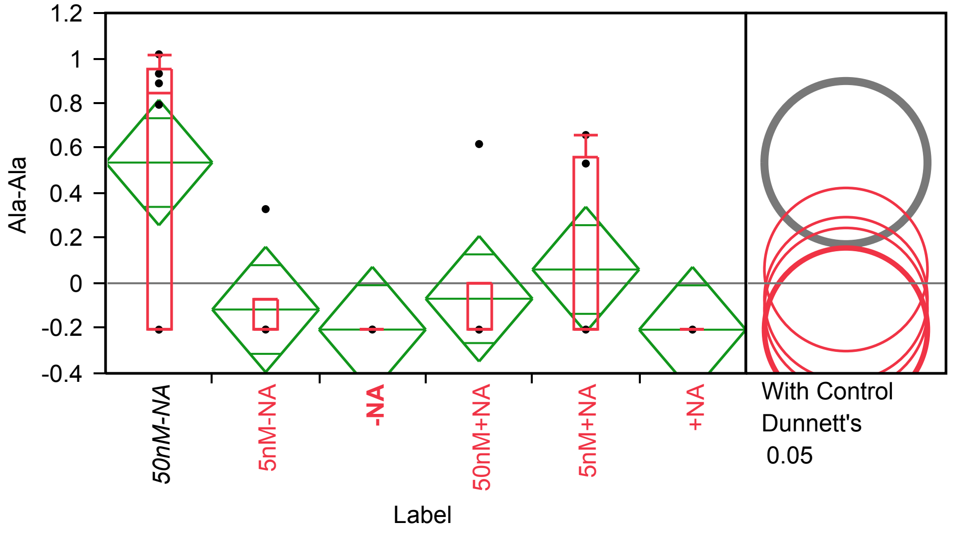


### Quantiles

| Level | Minimum | 10% | 25% | Median | 75% | 90% | Maximum |
|---|---|---|---|---|---|---|---|
| 50nM-NA | -0.20614 | -0.20614 | -0.20606 | 0.840199 | 0.952331 | 1.016762 | 1.016762 |
| 5nM-NA | -0.20611 | -0.20611 | -0.20586 | -0.20565 | -0.07179 | 0.329272 | 0.329272 |
| -NA | -0.20587 | -0.20587 | -0.20578 | -0.20567 | -0.20556 | -0.2054 | -0.2054 |
| 50nM+NA | -0.20611 | -0.20611 | -0.20604 | -0.20598 | 0.000126 | 0.61812 | 0.61812 |
| 5nM+NA | -0.20614 | -0.20614 | -0.20596 | -0.20571 | 0.563285 | 0.658197 | 0.658197 |
| +NA | -0.20755 | -0.20755 | -0.20658 | -0.20582 | -0.20578 | -0.20576 | -0.20576 |

### Oneway Anova

#### Summary of Fit

| | |
|---|---|
| Rsquare | 0.414101 |
| Adj Rsquare | 0.316451 |
| Root Mean Square Error | 0.333945 |
| Mean of Response | 2.778e-9 |
| Observations (or Sum Wgts) | 36 |

#### Analysis of Variance

| Source | DF | Sum of Squares | Mean Square | F Ratio | Prob > F |
|---|---|---|---|---|---|
| Label | 5 | 2.3645791 | 0.472916 | 4.2407 | 0.0049 * |
| Error | 30 | 3.3455721 | 0.111519 | | |
| C. Total | 35 | 5.7101512 | | | |

#### Means for Oneway Anova

| Level | Number | Mean | Std Error | Lower 95% | Upper 95% |
|---|---|---|---|---|---|
| 50nM-NA | 6 | 0.53597 | 0.13633 | 0.2575 | 0.81440 |
| 5nM-NA | 6 | -0.11657 | 0.13633 | -0.3950 | 0.16186 |
| -NA | 6 | -0.20566 | 0.13633 | -0.4841 | 0.07276 |
| 50nM+NA | 6 | -0.06864 | 0.13633 | -0.3471 | 0.20979 |
| 5nM+NA | 6 | 0.06106 | 0.13633 | -0.2174 | 0.33949 |
| +NA | 6 | -0.20616 | 0.13633 | -0.4846 | 0.07226 |

Std Error uses a pooled estimate of error variance

### Means Comparisons

#### Comparisons with a control using Dunnett's Method

## Oneway Analysis of Ala-Gly By Label

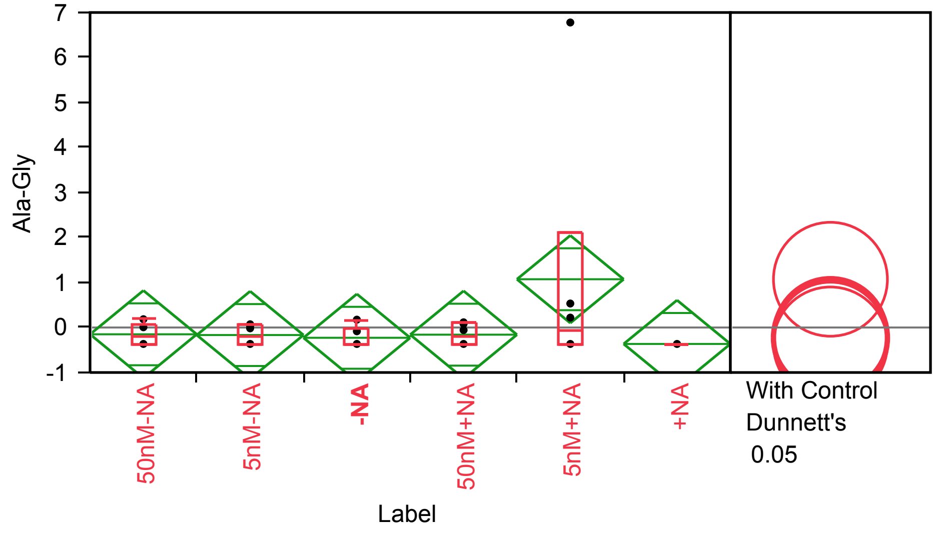


## Quantiles

| Level | Minimum | 10% | 25% | Median | 75% | 90% | Maximum |
|---|---|---|---|---|---|---|---|
| 50nM-NA | -0.36544 | -0.36544 | -0.36539 | -0.18271 | 0.050437 | 0.183814 | 0.183814 |
| 5nM-NA | -0.36543 | -0.36543 | -0.36535 | -0.19181 | 0.045148 | 0.071653 | 0.071653 |
| -NA | -0.36535 | -0.36535 | -0.36531 | -0.36523 | -0.01916 | 0.173762 | 0.173762 |
| 50nM+NA | -0.36543 | -0.36543 | -0.3654 | -0.21144 | 0.089646 | 0.114983 | 0.114983 |
| 5nM+NA | -0.36547 | -0.36547 | -0.36545 | -0.07303 | 2.0941 | 6.77809 | 6.77809 |
| +NA | -0.36592 | -0.36592 | -0.36559 | -0.36533 | -0.36532 | -0.36531 | -0.36531 |

## Oneway Anova

### Summary of Fit

| | |
|---|---|
| Rsquare | 0.171807 |
| Adj Rsquare | 0.033775 |
| Root Mean Square Error | 1.166765 |
| Mean of Response | -8.3e-11 |
| Observations (or Sum Wgts) | 36 |

### Analysis of Variance

| Source | DF | Sum of Squares | Mean Square | F Ratio | Prob > F |
|---|---|---|---|---|---|
| Label | 5 | 8.472228 | 1.69445 | 1.2447 | 0.3133 |
| Error | 30 | 40.840220 | 1.36134 | | |
| C. Total | 35 | 49.312448 | | | |

### Means for Oneway Anova

| Level | Number | Mean | Std Error | Lower 95% | Upper 95% |
|---|---|---|---|---|---|
| 50nM-NA | 6 | -0.1511 | 0.47633 | -1.124 | 0.8217 |
| 5nM-NA | 6 | -0.1677 | 0.47633 | -1.141 | 0.8051 |
| -NA | 6 | -0.2285 | 0.47633 | -1.201 | 0.7443 |
| 50nM+NA | 6 | -0.1596 | 0.47633 | -1.132 | 0.8132 |
| 5nM+NA | 6 | 1.0723 | 0.47633 | 0.100 | 2.0451 |
| +NA | 6 | -0.3655 | 0.47633 | -1.338 | 0.6073 |

Std Error uses a pooled estimate of error variance

## Means Comparisons

### Comparisons with a control using Dunnett's Method

## Oneway Analysis of alanine By Label

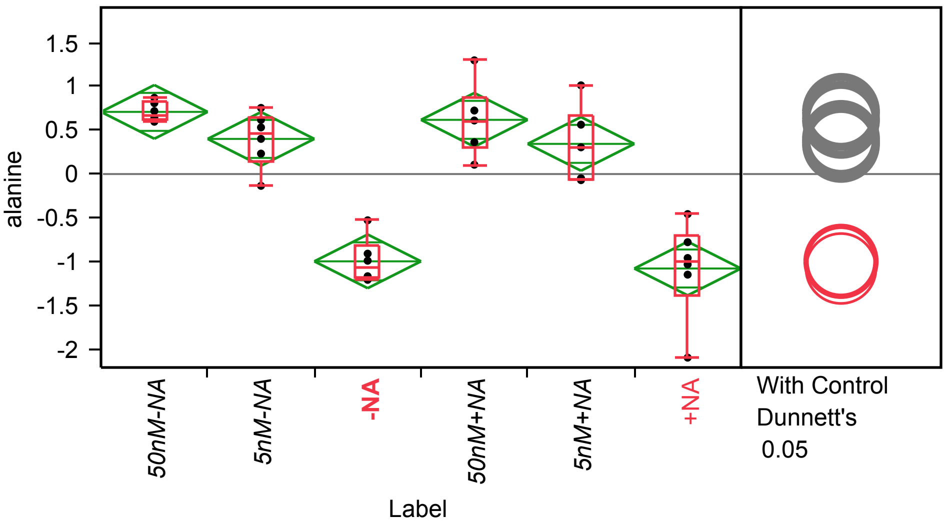


## Quantiles

| Level | Minimum | 10% | 25% | Median | 75% | 90% | Maximum |
|---|---|---|---|---|---|---|---|
| 50nM-NA | 0.596735 | 0.596735 | 0.615932 | 0.678944 | 0.824098 | 0.870025 | 0.870025 |
| 5nM-NA | -0.13413 | -0.13413 | 0.141323 | 0.466771 | 0.652219 | 0.754712 | 0.754712 |
| -NA | -1.20637 | -1.20637 | -1.18461 | -1.07714 | -0.81308 | -0.52748 | -0.52748 |
| 50nM+NA | 0.106742 | 0.106742 | 0.29918 | 0.609463 | 0.867733 | 1.297418 | 1.297418 |
| 5nM+NA | -0.06937 | -0.06937 | -0.05339 | 0.303989 | 0.675544 | 1.012091 | 1.012091 |
| +NA | -2.09418 | -2.09418 | -1.38411 | -0.99446 | -0.69703 | -0.45648 | -0.45648 |

## Oneway Anova

### Summary of Fit

| | |
|---|---|
| Rsquare | 0.830888 |
| Adj Rsquare | 0.802703 |
| Root Mean Square Error | 0.367449 |
| Mean of Response | -5.6e-10 |
| Observations (or Sum Wgts) | 36 |

### Analysis of Variance

| Source | DF | Sum of Squares | Mean Square | F Ratio | Prob > F |
|---|---|---|---|---|---|
| Label | 5 | 19.901406 | 3.98028 | 29.4795 | <.0001 * |
| Error | 30 | 4.050555 | 0.13502 | | |
| C. Total | 35 | 23.951961 | | | |

### Means for Oneway Anova

| Level | Number | Mean | Std Error | Lower 95% | Upper 95% |
|---|---|---|---|---|---|
| 50nM-NA | 6 | 0.7093 | 0.15001 | 0.403 | 1.016 |
| 5nM-NA | 6 | 0.4009 | 0.15001 | 0.095 | 0.707 |
| -NA | 6 | -0.9956 | 0.15001 | -1.302 | -0.689 |
| 50nM+NA | 6 | 0.6185 | 0.15001 | 0.312 | 0.925 |
| 5nM+NA | 6 | 0.3443 | 0.15001 | 0.038 | 0.651 |
| +NA | 6 | -1.0774 | 0.15001 | -1.384 | -0.771 |

Std Error uses a pooled estimate of error variance

## Means Comparisons

### Comparisons with a control using Dunnett's Method

## Oneway Analysis of allantoate By Label

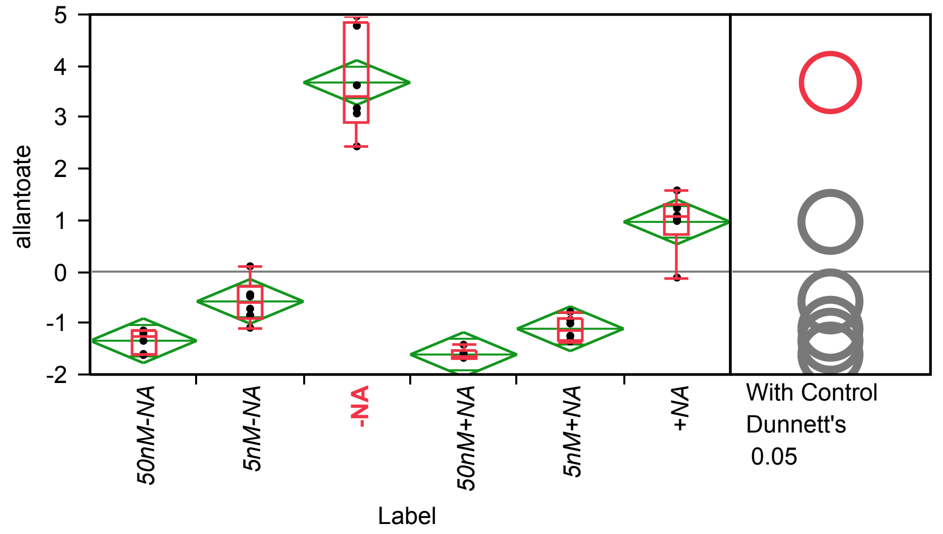


## Quantiles

| Level | Minimum | 10% | 25% | Median | 75% | 90% | Maximum |
|---|---|---|---|---|---|---|---|
| 50nM-NA | -1.62105 | -1.62105 | -1.6063 | -1.27481 | -1.14415 | -1.14384 | -1.14384 |
| 5nM-NA | -1.09071 | -1.09071 | -0.90703 | -0.60074 | -0.30473 | 0.104393 | 0.104393 |
| -NA | 2.435976 | 2.435976 | 2.916249 | 3.401909 | 4.827772 | 4.966276 | 4.966276 |
| 50nM+NA | -1.68277 | -1.68277 | -1.67968 | -1.65289 | -1.54181 | -1.42074 | -1.42074 |
| 5nM+NA | -1.35893 | -1.35893 | -1.34158 | -1.12735 | -0.89532 | -0.78078 | -0.78078 |
| +NA | -0.11506 | -0.11506 | 0.710763 | 1.058848 | 1.322054 | 1.579852 | 1.579852 |

## Oneway Anova

### Summary of Fit

| | |
|---|---|
| Rsquare | 0.938038 |
| Adj Rsquare | 0.927712 |
| Root Mean Square Error | 0.519514 |
| Mean of Response | 2.778e-9 |
| Observations (or Sum Wgts) | 36 |

### Analysis of Variance

| Source | DF | Sum of Squares | Mean Square | F Ratio | Prob > F |
|---|---|---|---|---|---|
| Label | 5 | 122.57833 | 24.5157 | 90.8342 | <.0001 * |
| Error | 30 | 8.09684 | 0.2699 | | |
| C. Total | 35 | 130.67517 | | | |

### Means for Oneway Anova

| Level | Number | Mean | Std Error | Lower 95% | Upper 95% |
|---|---|---|---|---|---|
| 50nM-NA | 6 | -1.3434 | 0.21209 | -1.777 | -0.910 |
| 5nM-NA | 6 | -0.5791 | 0.21209 | -1.012 | -0.146 |
| -NA | 6 | 3.6773 | 0.21209 | 3.244 | 4.110 |
| 50nM+NA | 6 | -1.6117 | 0.21209 | -2.045 | -1.179 |
| 5nM+NA | 6 | -1.1106 | 0.21209 | -1.544 | -0.677 |
| +NA | 6 | 0.9674 | 0.21209 | 0.534 | 1.401 |

Std Error uses a pooled estimate of error variance

## Means Comparisons

### Comparisons with a control using Dunnett's Method

## Oneway Analysis of Aminoadipic acid By Label

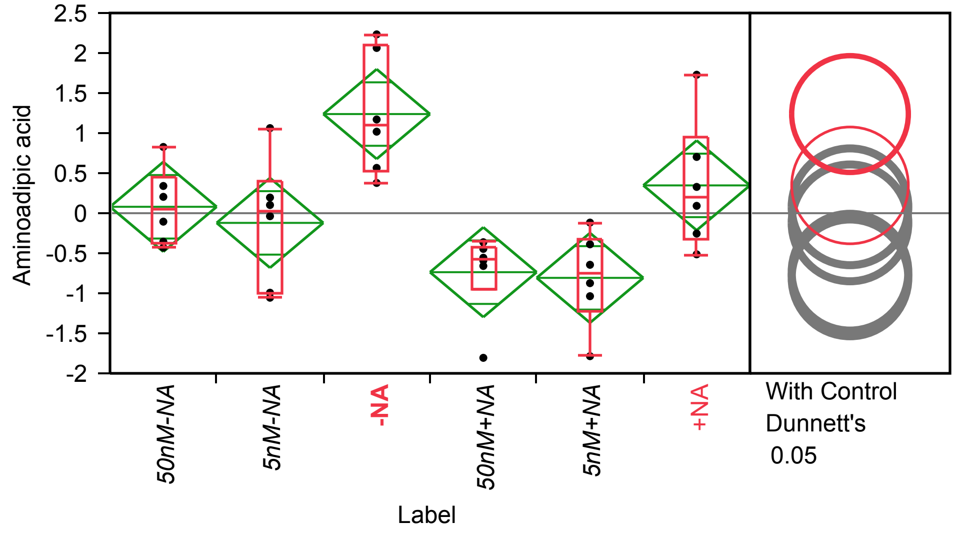


## Quantiles

| Level | Minimum | 10% | 25% | Median | 75% | 90% | Maximum |
|---|---|---|---|---|---|---|---|
| 50nM-NA | -0.42977 | -0.42977 | -0.37434 | 0.049233 | 0.461708 | 0.827022 | 0.827022 |
| 5nM-NA | -1.05532 | -1.05532 | -1.00865 | 0.032324 | 0.412188 | 1.062405 | 1.062405 |
| -NA | 0.378035 | 0.378035 | 0.518728 | 1.094183 | 2.106095 | 2.233133 | 2.233133 |
| 50nM+NA | -1.80618 | -1.80618 | -0.94577 | -0.57335 | -0.42474 | -0.36248 | -0.36248 |
| 5nM+NA | -1.78542 | -1.78542 | -1.22372 | -0.75889 | -0.32101 | -0.11737 | -0.11737 |
| +NA | -0.51359 | -0.51359 | -0.31947 | 0.209926 | 0.95923 | 1.728751 | 1.728751 |

## Oneway Anova

### Summary of Fit

| | |
|---|---|
| Rsquare | 0.559469 |
| Adj Rsquare | 0.486047 |
| Root Mean Square Error | 0.672233 |
| Mean of Response | -1.2e-17 |
| Observations (or Sum Wgts) | 36 |

### Analysis of Variance

| Source | DF | Sum of Squares | Mean Square | F Ratio | Prob > F |
|---|---|---|---|---|---|
| Label | 5 | 17.217106 | 3.44342 | 7.6199 | 0.0001 * |
| Error | 30 | 13.556906 | 0.45190 | | |
| C. Total | 35 | 30.774012 | | | |

### Means for Oneway Anova

| Level | Number | Mean | Std Error | Lower 95% | Upper 95% |
|---|---|---|---|---|---|
| 50nM-NA | 6 | 0.0800 | 0.27444 | -0.481 | 0.640 |
| 5nM-NA | 6 | -0.1210 | 0.27444 | -0.681 | 0.439 |
| -NA | 6 | 1.2382 | 0.27444 | 0.678 | 1.799 |
| 50nM+NA | 6 | -0.7366 | 0.27444 | -1.297 | -0.176 |
| 5nM+NA | 6 | -0.8077 | 0.27444 | -1.368 | -0.247 |
| +NA | 6 | 0.3472 | 0.27444 | -0.213 | 0.908 |

Std Error uses a pooled estimate of error variance

## Means Comparisons

### Comparisons with a control using Dunnett's Method

## Oneway Analysis of AICAR(ZMP) By Label

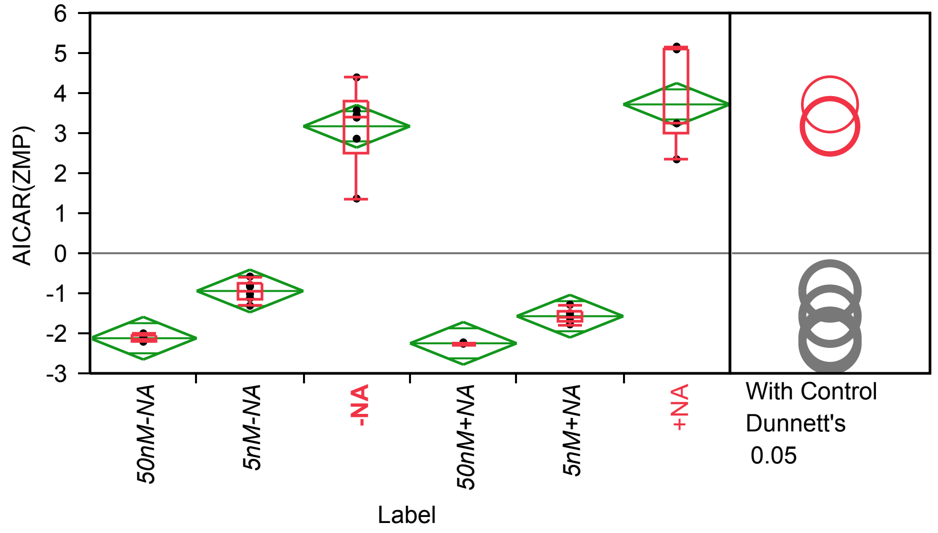


### Quantiles

| Level | Minimum | 10% | 25% | Median | 75% | 90% | Maximum |
|---|---|---|---|---|---|---|---|
| 50nM-NA | -2.21009 | -2.21009 | -2.17723 | -2.13695 | -2.0644 | -2.00136 | -2.00136 |
| 5nM-NA | -1.31157 | -1.31157 | -1.16844 | -0.92642 | -0.7383 | -0.5817 | -0.5817 |
| -NA | 1.365982 | 1.365982 | 2.486574 | 3.418112 | 3.776089 | 4.391648 | 4.391648 |
| 50nM+NA | -2.28559 | -2.28559 | -2.26686 | -2.24432 | -2.23536 | -2.22713 | -2.22713 |
| 5nM+NA | -1.77715 | -1.77715 | -1.71496 | -1.59015 | -1.4452 | -1.28532 | -1.28532 |
| +NA | 2.345733 | 2.345733 | 3.005039 | 3.240868 | 5.109774 | 5.159685 | 5.159685 |

### Oneway Anova

#### Summary of Fit

| | |
|---|---|
| Rsquare | 0.947673 |
| Adj Rsquare | 0.938952 |
| Root Mean Square Error | 0.637532 |
| Mean of Response | 0 |
| Observations (or Sum Wgts) | 36 |

#### Analysis of Variance

| Source | DF | Sum of Squares | Mean Square | F Ratio | Prob > F |
|---|---|---|---|---|---|
| Label | 5 | 220.83024 | 44.1660 | 108.6638 | <.0001 * |
| Error | 30 | 12.19341 | 0.4064 | | |
| C. Total | 35 | 233.02365 | | | |

#### Means for Oneway Anova

| Level | Number | Mean | Std Error | Lower 95% | Upper 95% |
|---|---|---|---|---|---|
| 50nM-NA | 6 | -2.1228 | 0.26027 | -2.654 | -1.591 |
| 5nM-NA | 6 | -0.9429 | 0.26027 | -1.474 | -0.411 |
| -NA | 6 | 3.1708 | 0.26027 | 2.639 | 3.702 |
| 50nM+NA | 6 | -2.2500 | 0.26027 | -2.782 | -1.718 |
| 5nM+NA | 6 | -1.5726 | 0.26027 | -2.104 | -1.041 |
| +NA | 6 | 3.7175 | 0.26027 | 3.186 | 4.249 |

Std Error uses a pooled estimate of error variance

### Means Comparisons

#### Comparisons with a control using Dunnett's Method

## Oneway Analysis of Aminomalonic acid By Label

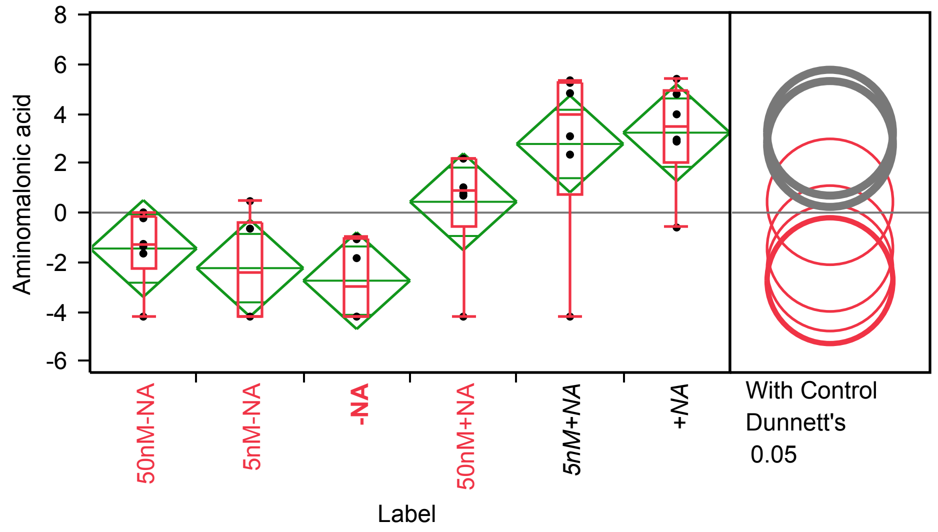


### Quantiles

| Level | Minimum | 10% | 25% | Median | 75% | 90% | Maximum |
|---|---|---|---|---|---|---|---|
| 50nM-NA | -4.19776 | -4.19776 | -2.28802 | -1.31305 | -0.16776 | 0.009186 | 0.009186 |
| 5nM-NA | -4.19774 | -4.19774 | -4.19772 | -2.43214 | -0.36874 | 0.463641 | 0.463641 |
| -NA | -4.19776 | -4.19776 | -4.19775 | -3.01733 | -1.04862 | -0.97768 | -0.97768 |
| 50nM+NA | -4.19778 | -4.19778 | -0.53658 | 0.897304 | 2.166758 | 2.17914 | 2.17914 |
| 5nM+NA | -4.19773 | -4.19773 | 0.706493 | 3.951793 | 5.264413 | 5.339218 | 5.339218 |
| +NA | -0.59964 | -0.59964 | 2.000886 | 3.458402 | 4.936844 | 5.399555 | 5.399555 |

### Oneway Anova

#### Summary of Fit

| | |
|---|---|
| Rsquare | 0.545919 |
| Adj Rsquare | 0.470239 |
| Root Mean Square Error | 2.341266 |
| Mean of Response | -9.9e-17 |
| Observations (or Sum Wgts) | 36 |

#### Analysis of Variance

| Source | DF | Sum of Squares | Mean Square | F Ratio | Prob > F |
|---|---|---|---|---|---|
| Label | 5 | 197.70527 | 39.5411 | 7.2135 | 0.0002 * |
| Error | 30 | 164.44580 | 5.4815 | | |
| C. Total | 35 | 362.15108 | | | |

#### Means for Oneway Anova

| Level | Number | Mean | Std Error | Lower 95% | Upper 95% |
|---|---|---|---|---|---|
| 50nM-NA | 6 | -1.4488 | 0.95582 | -3.401 | 0.503 |
| 5nM-NA | 6 | -2.2404 | 0.95582 | -4.192 | -0.288 |
| -NA | 6 | -2.7467 | 0.95582 | -4.699 | -0.795 |
| 50nM+NA | 6 | 0.4371 | 0.95582 | -1.515 | 2.389 |
| 5nM+NA | 6 | 2.7710 | 0.95582 | 0.819 | 4.723 |
| +NA | 6 | 3.2278 | 0.95582 | 1.276 | 5.180 |

Std Error uses a pooled estimate of error variance

### Means Comparisons

#### Comparisons with a control using Dunnett's Method

## Oneway Analysis of AMP By Label

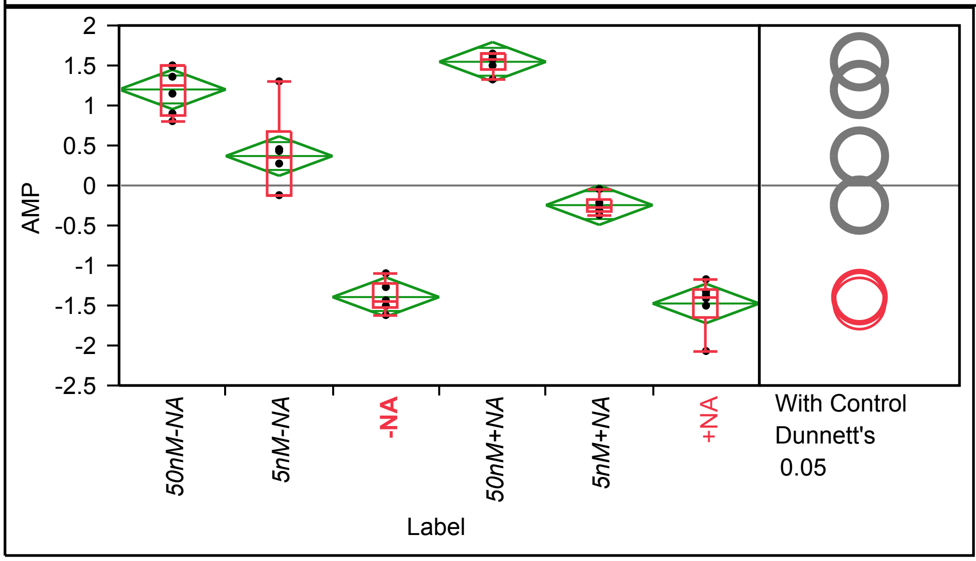


## Quantiles

| Level | Minimum | 10% | 25% | Median | 75% | 90% | Maximum |
|---|---|---|---|---|---|---|---|
| 50nM-NA | 0.804621 | 0.804621 | 0.876673 | 1.254101 | 1.491079 | 1.501597 | 1.501597 |
| 5nM-NA | -0.1326 | -0.1326 | -0.12391 | 0.352823 | 0.666539 | 1.302244 | 1.302244 |
| -NA | -1.61949 | -1.61949 | -1.53277 | -1.43933 | -1.22608 | -1.09688 | -1.09688 |
| 50nM+NA | 1.326028 | 1.326028 | 1.456487 | 1.583835 | 1.638922 | 1.64954 | 1.64954 |
| 5nM+NA | -0.36915 | -0.36915 | -0.32466 | -0.26682 | -0.17193 | -0.04523 | -0.04523 |
| +NA | -2.06874 | -2.06874 | -1.64315 | -1.39006 | -1.28919 | -1.17163 | -1.17163 |

## Oneway Anova

### Summary of Fit

| | |
|---|---|
| Rsquare | 0.949196 |
| Adj Rsquare | 0.940728 |
| Root Mean Square Error | 0.295353 |
| Mean of Response | 2.78e-10 |
| Observations (or Sum Wgts) | 36 |

### Analysis of Variance

| Source | DF | Sum of Squares | Mean Square | F Ratio | Prob > F |
|---|---|---|---|---|---|
| Label | 5 | 48.894370 | 9.77887 | 112.1002 | <.0001 * |
| Error | 30 | 2.617000 | 0.08723 | | |
| C. Total | 35 | 51.511370 | | | |

### Means for Oneway Anova

| Level | Number | Mean | Std Error | Lower 95% | Upper 95% |
|---|---|---|---|---|---|
| 50nM-NA | 6 | 1.2004 | 0.12058 | 0.954 | 1.447 |
| 5nM-NA | 6 | 0.3682 | 0.12058 | 0.122 | 0.614 |
| -NA | 6 | -1.3947 | 0.12058 | -1.641 | -1.148 |
| 50nM+NA | 6 | 1.5464 | 0.12058 | 1.300 | 1.793 |
| 5nM+NA | 6 | -0.2453 | 0.12058 | -0.492 | 0.00092 |
| +NA | 6 | -1.4750 | 0.12058 | -1.721 | -1.229 |

Std Error uses a pooled estimate of error variance

## Means Comparisons

### Comparisons with a control using Dunnett's Method

## Oneway Analysis of Anhydrosorbitol By Label

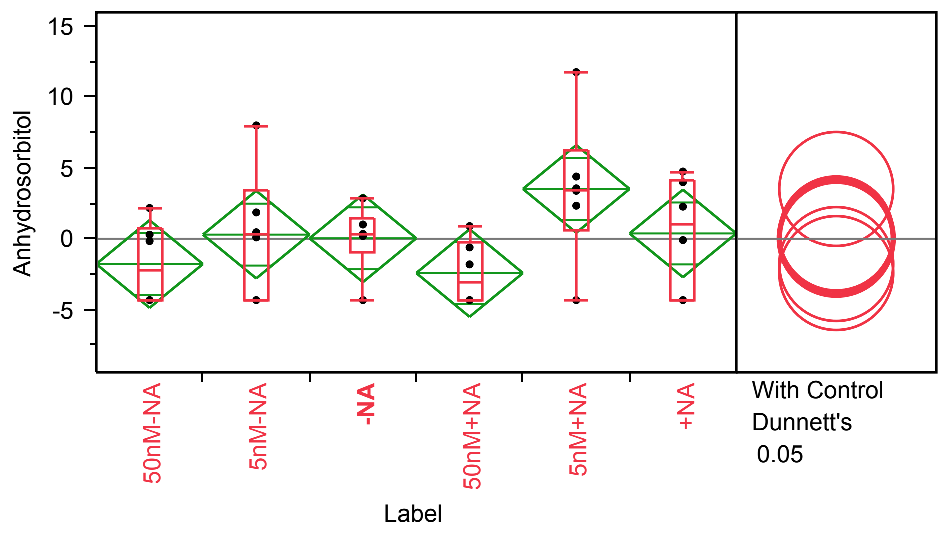


### Quantiles

| Level | Minimum | 10% | 25% | Median | 75% | 90% | Maximum |
|---|---|---|---|---|---|---|---|
| 50nM-NA | -4.33587 | -4.33587 | -4.33586 | -2.25187 | 0.755378 | 2.169058 | 2.169058 |
| 5nM-NA | -4.33585 | -4.33585 | -4.33582 | 0.275899 | 3.395682 | 8.005105 | 8.005105 |
| -NA | -4.33581 | -4.33581 | -0.99241 | 0.265467 | 1.467042 | 2.852432 | 2.852432 |
| 50nM+NA | -4.33588 | -4.33588 | -4.33587 | -3.07429 | -0.23685 | 0.890167 | 0.890167 |
| 5nM+NA | -4.33584 | -4.33584 | 0.665653 | 3.473047 | 6.237559 | 11.76941 | 11.76941 |
| +NA | -4.33586 | -4.33586 | -4.33585 | 1.085018 | 4.182573 | 4.746672 | 4.746672 |

### Oneway Anova

#### Summary of Fit

| | |
|---|---|
| Rsquare | 0.239429 |
| Adj Rsquare | 0.112667 |
| Root Mean Square Error | 3.710194 |
| Mean of Response | 2.78e-10 |
| Observations (or Sum Wgts) | 36 |

#### Analysis of Variance

| Source | DF | Sum of Squares | Mean Square | F Ratio | Prob > F |
|---|---|---|---|---|---|
| Label | 5 | 130.00210 | 26.0004 | 1.8888 | 0.1259 |
| Error | 30 | 412.96617 | 13.7655 | | |
| C. Total | 35 | 542.96827 | | | |

#### Means for Oneway Anova

| Level | Number | Mean | Std Error | Lower 95% | Upper 95% |
|---|---|---|---|---|---|
| 50nM-NA | 6 | -1.7870 | 1.5147 | -4.880 | 1.3063 |
| 5nM-NA | 6 | 0.2907 | 1.5147 | -2.803 | 3.3841 |
| -NA | 6 | 0.0291 | 1.5147 | -3.064 | 3.1225 |
| 50nM+NA | 6 | -2.4238 | 1.5147 | -5.517 | 0.6696 |
| 5nM+NA | 6 | 3.5177 | 1.5147 | 0.424 | 6.6111 |
| +NA | 6 | 0.3733 | 1.5147 | -2.720 | 3.4666 |

Std Error uses a pooled estimate of error variance

### Means Comparisons

#### Comparisons with a control using Dunnett's Method

## Oneway Analysis of Arabitol By Label

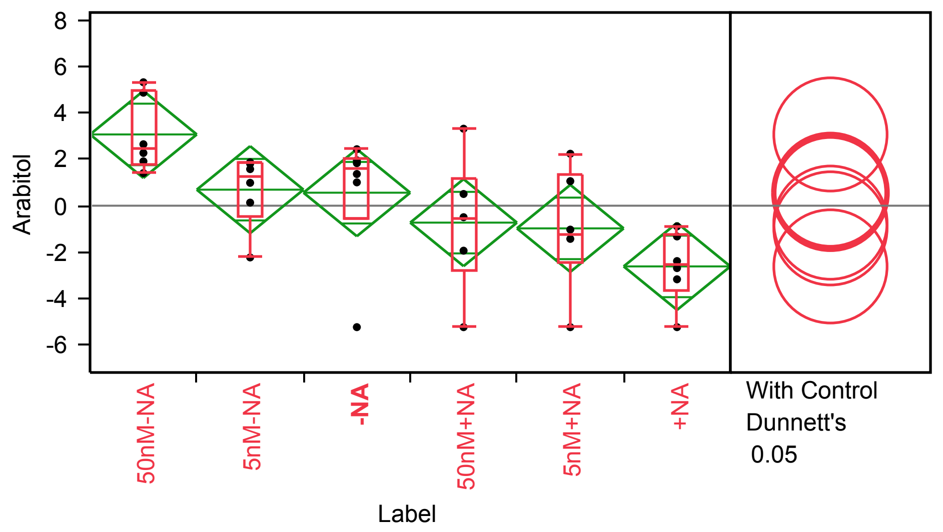


## Quantiles

| Level | Minimum | 10% | 25% | Median | 75% | 90% | Maximum |
|---|---|---|---|---|---|---|---|
| 50nM-NA | 1.407161 | 1.407161 | 1.787215 | 2.462121 | 4.986505 | 5.323787 | 5.323787 |
| 5nM-NA | -2.23137 | -2.23137 | -0.45838 | 1.282188 | 1.825411 | 1.873102 | 1.873102 |
| -NA | -5.23865 | -5.23865 | -0.55434 | 1.600656 | 2.078054 | 2.436184 | 2.436184 |
| 50nM+NA | -5.23871 | -5.23871 | -2.765 | -0.51236 | 1.208945 | 3.320171 | 3.320171 |
| 5nM+NA | -5.23871 | -5.23871 | -2.40867 | -1.23112 | 1.354142 | 2.240349 | 2.240349 |
| +NA | -5.23868 | -5.23868 | -3.69081 | -2.53975 | -1.21744 | -0.89108 | -0.89108 |

## Oneway Anova

### Summary of Fit

| | |
|---|---|
| Rsquare | 0.423172 |
| Adj Rsquare | 0.327034 |
| Root Mean Square Error | 2.250972 |
| Mean of Response | 1.48e-16 |
| Observations (or Sum Wgts) | 36 |

### Analysis of Variance

| Source | DF | Sum of Squares | Mean Square | F Ratio | Prob > F |
|---|---|---|---|---|---|
| Label | 5 | 111.51479 | 22.3030 | 4.4017 | 0.0040 * |
| Error | 30 | 152.00619 | 5.0669 | | |
| C. Total | 35 | 263.52099 | | | |

### Means for Oneway Anova

| Level | Number | Mean | Std Error | Lower 95% | Upper 95% |
|---|---|---|---|---|---|
| 50nM-NA | 6 | 3.0739 | 0.91896 | 1.197 | 4.951 |
| 5nM-NA | 6 | 0.6914 | 0.91896 | -1.185 | 2.568 |
| -NA | 6 | 0.5608 | 0.91896 | -1.316 | 2.438 |
| 50nM+NA | 6 | -0.7297 | 0.91896 | -2.607 | 1.147 |
| 5nM+NA | 6 | -0.9779 | 0.91896 | -2.855 | 0.899 |
| +NA | 6 | -2.6184 | 0.91896 | -4.495 | -0.742 |

Std Error uses a pooled estimate of error variance

## Means Comparisons

### Comparisons with a control using Dunnett's Method

## Oneway Analysis of Arginine By Label

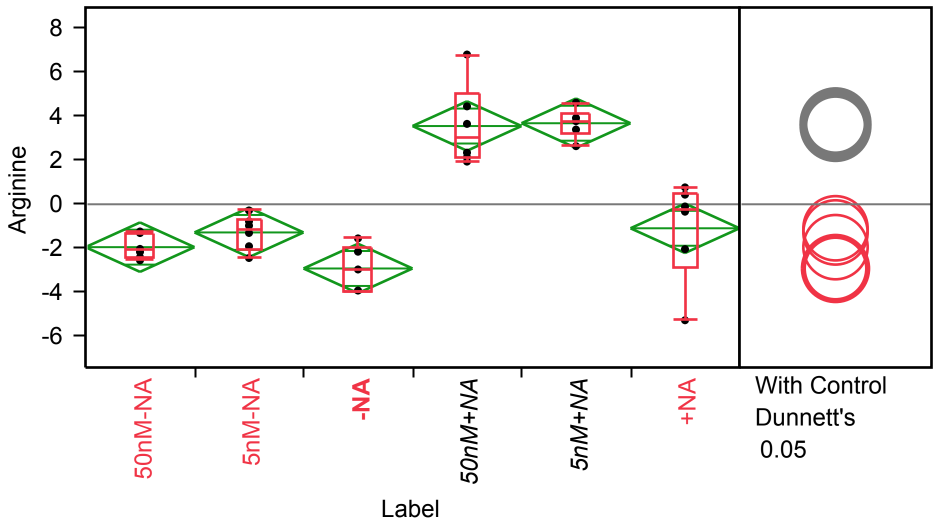


## Quantiles

| Level | Minimum | 10% | 25% | Median | 75% | 90% | Maximum |
|---|---|---|---|---|---|---|---|
| 50nM-NA | -2.54322 | -2.54322 | -2.44105 | -2.09381 | -1.28902 | -1.23618 | -1.23618 |
| 5nM-NA | -2.43894 | -2.43894 | -2.03919 | -1.13636 | -0.64932 | -0.28214 | -0.28214 |
| -NA | -3.95092 | -3.95092 | -3.92502 | -2.96027 | -2.0024 | -1.55037 | -1.55037 |
| 50nM+NA | 1.937607 | 1.937607 | 2.08705 | 2.996594 | 5.032613 | 6.797178 | 6.797178 |
| 5nM+NA | 2.638329 | 2.638329 | 3.203396 | 3.756947 | 4.092146 | 4.610685 | 4.610685 |
| +NA | -5.26524 | -5.26524 | -2.84421 | -0.22081 | 0.519817 | 0.766728 | 0.766728 |

## Oneway Anova

### Summary of Fit

| | |
|---|---|
| Rsquare | 0.820106 |
| Adj Rsquare | 0.790124 |
| Root Mean Square Error | 1.345398 |
| Mean of Response | -5.56e-9 |
| Observations (or Sum Wgts) | 36 |

### Analysis of Variance

| Source | DF | Sum of Squares | Mean Square | F Ratio | Prob > F |
|---|---|---|---|---|---|
| Label | 5 | 247.55795 | 49.5116 | 27.3530 | <.0001 * |
| Error | 30 | 54.30288 | 1.8101 | | |
| C. Total | 35 | 301.86082 | | | |

### Means for Oneway Anova

| Level | Number | Mean | Std Error | Lower 95% | Upper 95% |
|---|---|---|---|---|---|
| 50nM-NA | 6 | -1.9468 | 0.54926 | -3.069 | -0.825 |
| 5nM-NA | 6 | -1.2786 | 0.54926 | -2.400 | -0.157 |
| -NA | 6 | -2.9152 | 0.54926 | -4.037 | -1.793 |
| 50nM+NA | 6 | 3.5515 | 0.54926 | 2.430 | 4.673 |
| 5nM+NA | 6 | 3.6790 | 0.54926 | 2.557 | 4.801 |
| +NA | 6 | -1.0900 | 0.54926 | -2.212 | 0.032 |

Std Error uses a pooled estimate of error variance

## Means Comparisons

### Comparisons with a control using Dunnett's Method

## Oneway Analysis of Asparagine By Label

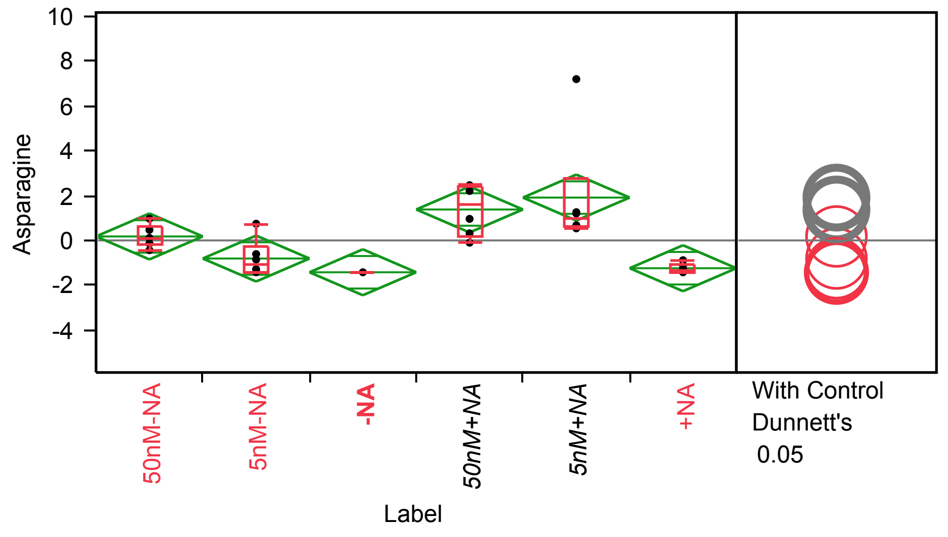


## Quantiles

| Level | Minimum | 10% | 25% | Median | 75% | 90% | Maximum |
|---|---|---|---|---|---|---|---|
| 50nM-NA | -0.43287 | -0.43287 | -0.20856 | 0.095661 | 0.595477 | 0.970184 | 0.970184 |
| 5nM-NA | -1.42114 | -1.42114 | -1.42112 | -1.06922 | -0.2715 | 0.743378 | 0.743378 |
| -NA | -1.42123 | -1.42123 | -1.42121 | -1.42118 | -1.42116 | -1.42112 | -1.42112 |
| 50nM+NA | -0.1003 | -0.1003 | 0.210756 | 1.58487 | 2.440553 | 2.46399 | 2.46399 |
| 5nM+NA | 0.539904 | 0.539904 | 0.578426 | 0.940581 | 2.744446 | 7.197166 | 7.197166 |
| +NA | -1.42121 | -1.42121 | -1.42121 | -1.30263 | -1.05334 | -0.88415 | -0.88415 |

## Oneway Anova

### Summary of Fit

| | |
|---|---|
| Rsquare | 0.564729 |
| Adj Rsquare | 0.492184 |
| Root Mean Square Error | 1.229172 |
| Mean of Response | 2.78e-10 |
| Observations (or Sum Wgts) | 36 |

### Analysis of Variance

| Source | DF | Sum of Squares | Mean Square | F Ratio | Prob > F |
|---|---|---|---|---|---|
| Label | 5 | 58.80667 | 11.7613 | 7.7845 | <.0001 * |
| Error | 30 | 45.32593 | 1.5109 | | |
| C. Total | 35 | 104.13260 | | | |

### Means for Oneway Anova

| Level | Number | Mean | Std Error | Lower 95% | Upper 95% |
|---|---|---|---|---|---|
| 50nM-NA | 6 | 0.1776 | 0.50181 | -0.847 | 1.202 |
| 5nM-NA | 6 | -0.8079 | 0.50181 | -1.833 | 0.217 |
| -NA | 6 | -1.4212 | 0.50181 | -2.446 | -0.396 |
| 50nM+NA | 6 | 1.3801 | 0.50181 | 0.355 | 2.405 |
| 5nM+NA | 6 | 1.9116 | 0.50181 | 0.887 | 2.936 |
| +NA | 6 | -1.2403 | 0.50181 | -2.265 | -0.215 |

Std Error uses a pooled estimate of error variance

## Means Comparisons

### Comparisons with a control using Dunnett's Method

## Oneway Analysis of aspartate By Label

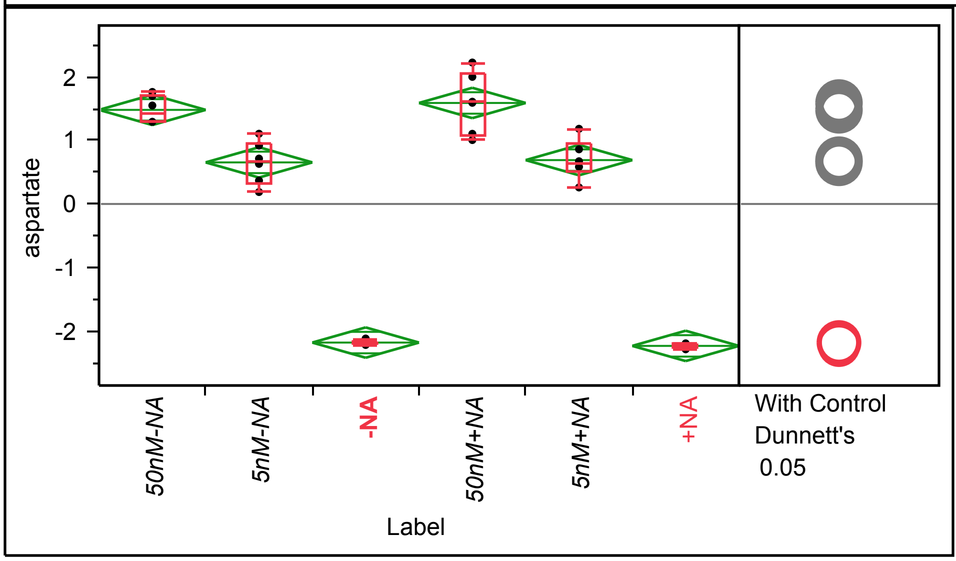


### Quantiles

| Level | Minimum | 10% | 25% | Median | 75% | 90% | Maximum |
|---|---|---|---|---|---|---|---|
| 50nM-NA | 1.285323 | 1.285323 | 1.290129 | 1.419915 | 1.713264 | 1.76084 | 1.76084 |
| 5nM-NA | 0.187828 | 0.187828 | 0.322628 | 0.671903 | 0.964545 | 1.103681 | 1.103681 |
| -NA | -2.2161 | -2.2161 | -2.20729 | -2.187 | -2.15139 | -2.11562 | -2.11562 |
| 50nM+NA | 1.005036 | 1.005036 | 1.078297 | 1.59886 | 2.054409 | 2.223257 | 2.223257 |
| 5nM+NA | 0.259766 | 0.259766 | 0.500775 | 0.627468 | 0.940011 | 1.181576 | 1.181576 |
| +NA | -2.28442 | -2.28442 | -2.25035 | -2.23668 | -2.19651 | -2.19161 | -2.19161 |

### Oneway Anova

#### Summary of Fit

| | |
|---|---|
| Rsquare | 0.974029 |
| Adj Rsquare | 0.969701 |
| Root Mean Square Error | 0.285984 |
| Mean of Response | -1.11e-8 |
| Observations (or Sum Wgts) | 36 |

#### Analysis of Variance

| Source | DF | Sum of Squares | Mean Square | F Ratio | Prob > F |
|---|---|---|---|---|---|
| Label | 5 | 92.021599 | 18.4043 | 225.0277 | <.0001 * |
| Error | 30 | 2.453607 | 0.0818 | | |
| C. Total | 35 | 94.475207 | | | |

#### Means for Oneway Anova

| Level | Number | Mean | Std Error | Lower 95% | Upper 95% |
|---|---|---|---|---|---|
| 50nM-NA | 6 | 1.4792 | 0.11675 | 1.241 | 1.718 |
| 5nM-NA | 6 | 0.6535 | 0.11675 | 0.415 | 0.892 |
| -NA | 6 | -2.1789 | 0.11675 | -2.417 | -1.940 |
| 50nM+NA | 6 | 1.5878 | 0.11675 | 1.349 | 1.826 |
| 5nM+NA | 6 | 0.6895 | 0.11675 | 0.451 | 0.928 |
| +NA | 6 | -2.2311 | 0.11675 | -2.470 | -1.993 |

Std Error uses a pooled estimate of error variance

### Means Comparisons

#### Comparisons with a control using Dunnett's Method

## Oneway Analysis of ATP By Label

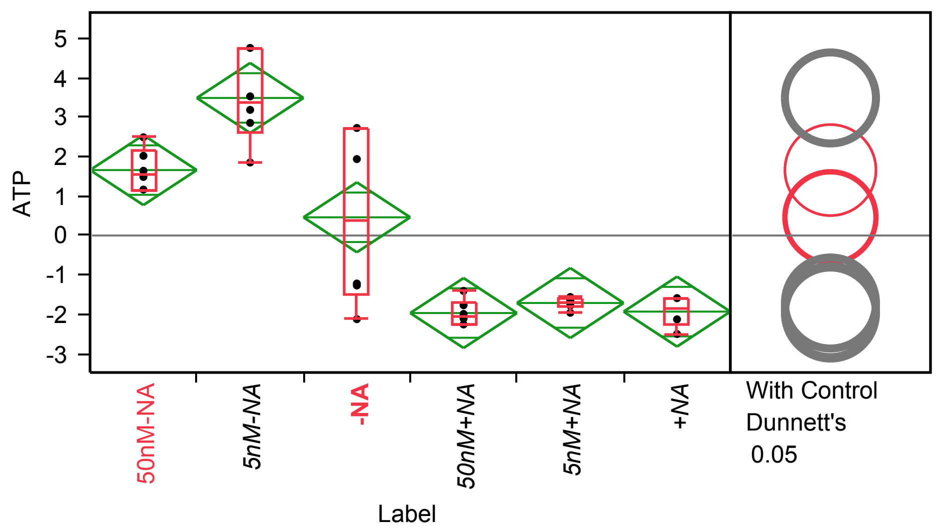


### Quantiles

| Level | Minimum | 10% | 25% | Median | 75% | 90% | Maximum |
|---|---|---|---|---|---|---|---|
| 50nM-NA | 1.154482 | 1.154482 | 1.163262 | 1.5663 | 2.141964 | 2.495491 | 2.495491 |
| 5nM-NA | 1.855749 | 1.855749 | 2.609769 | 3.363714 | 4.762448 | 4.766207 | 4.766207 |
| -NA | -2.12583 | -2.12583 | -1.48382 | 0.360479 | 2.720274 | 2.732486 | 2.732486 |
| 50nM+NA | -2.27653 | -2.27653 | -2.26473 | -2.05142 | -1.68195 | -1.40857 | -1.40857 |
| 5nM+NA | -1.96269 | -1.96269 | -1.8245 | -1.67954 | -1.61253 | -1.56314 | -1.56314 |
| +NA | -2.50367 | -2.50367 | -2.23709 | -1.87298 | -1.60912 | -1.59158 | -1.59158 |

### Oneway Anova

#### Summary of Fit

| | |
|---|---|
| Rsquare | 0.819263 |
| Adj Rsquare | 0.78914 |
| Root Mean Square Error | 1.066078 |
| Mean of Response | 0 |
| Observations (or Sum Wgts) | 36 |

#### Analysis of Variance

| Source | DF | Sum of Squares | Mean Square | F Ratio | Prob > F |
|---|---|---|---|---|---|
| Label | 5 | 154.55253 | 30.9105 | 27.1974 | <.0001 * |
| Error | 30 | 34.09568 | 1.1365 | | |
| C. Total | 35 | 188.64822 | | | |

#### Means for Oneway Anova

| Level | Number | Mean | Std Error | Lower 95% | Upper 95% |
|---|---|---|---|---|---|
| 50nM-NA | 6 | 1.6621 | 0.43522 | 0.773 | 2.551 |
| 5nM-NA | 6 | 3.4953 | 0.43522 | 2.606 | 4.384 |
| -NA | 6 | 0.4623 | 0.43522 | -0.427 | 1.351 |
| 50nM+NA | 6 | -1.9703 | 0.43522 | -2.859 | -1.081 |
| 5nM+NA | 6 | -1.7154 | 0.43522 | -2.604 | -0.827 |
| +NA | 6 | -1.9341 | 0.43522 | -2.823 | -1.045 |

Std Error uses a pooled estimate of error variance

### Means Comparisons

#### Comparisons with a control using Dunnett's Method

## Oneway Analysis of Atrolactic acid By Label

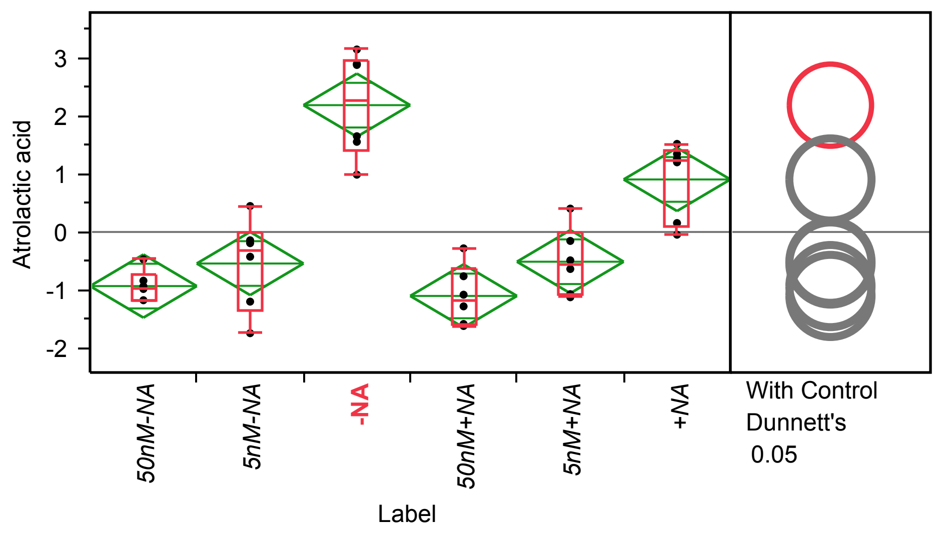


## Quantiles

| Level | Minimum | 10% | 25% | Median | 75% | 90% | Maximum |
|---|---|---|---|---|---|---|---|
| 50nM-NA | -1.18652 | -1.18652 | -1.17713 | -0.95729 | -0.74618 | -0.47038 | -0.47038 |
| 5nM-NA | -1.74081 | -1.74081 | -1.33396 | -0.30921 | 0.004143 | 0.446285 | 0.446285 |
| -NA | 0.985637 | 0.985637 | 1.40941 | 2.25961 | 2.954186 | 3.138685 | 3.138685 |
| 50nM+NA | -1.61707 | -1.61707 | -1.59127 | -1.17923 | -0.64125 | -0.28161 | -0.28161 |
| 5nM+NA | -1.12259 | -1.12259 | -1.08278 | -0.56219 | -0.01518 | 0.401655 | 0.401655 |
| +NA | -0.04666 | -0.04666 | 0.103367 | 1.228888 | 1.384978 | 1.514965 | 1.514965 |

## Oneway Anova

### Summary of Fit

| | |
|---|---|
| Rsquare | 0.794624 |
| Adj Rsquare | 0.760395 |
| Root Mean Square Error | 0.651289 |
| Mean of Response | -4.9e-17 |
| Observations (or Sum Wgts) | 36 |

### Analysis of Variance

| Source | DF | Sum of Squares | Mean Square | F Ratio | Prob > F |
|---|---|---|---|---|---|
| Label | 5 | 49.235756 | 9.84715 | 23.2147 | <.0001 * |
| Error | 30 | 12.725312 | 0.42418 | | |
| C. Total | 35 | 61.961068 | | | |

### Means for Oneway Anova

| Level | Number | Mean | Std Error | Lower 95% | Upper 95% |
|---|---|---|---|---|---|
| 50nM-NA | 6 | -0.9306 | 0.26589 | -1.474 | -0.388 |
| 5nM-NA | 6 | -0.5424 | 0.26589 | -1.085 | 0.00059 |
| -NA | 6 | 2.1811 | 0.26589 | 1.638 | 2.724 |
| 50nM+NA | 6 | -1.1002 | 0.26589 | -1.643 | -0.557 |
| 5nM+NA | 6 | -0.5115 | 0.26589 | -1.055 | 0.032 |
| +NA | 6 | 0.9035 | 0.26589 | 0.361 | 1.447 |

Std Error uses a pooled estimate of error variance

## Means Comparisons

### Comparisons with a control using Dunnett's Method

## Oneway Analysis of Benzoic acid By Label

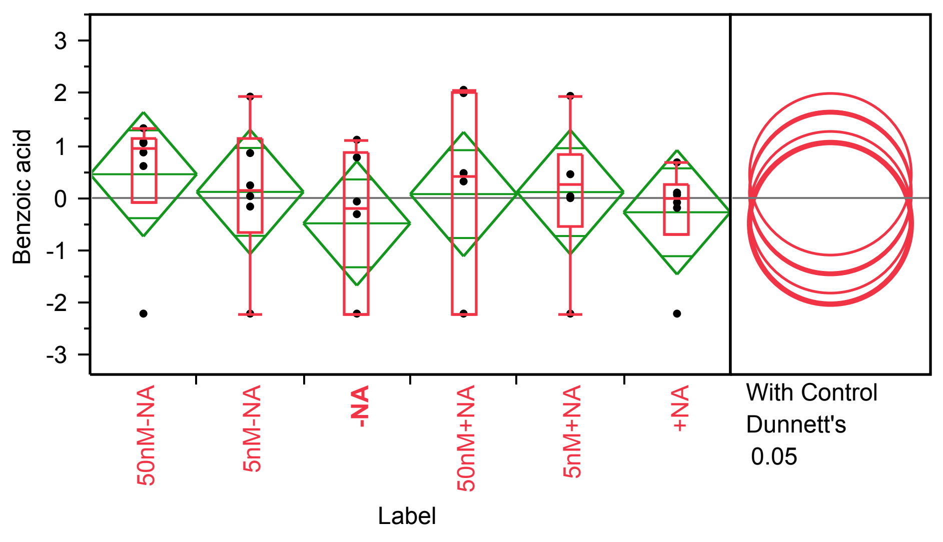


## Quantiles

| Level | Minimum | 10% | 25% | Median | 75% | 90% | Maximum |
|---|---|---|---|---|---|---|---|
| 50nM-NA | -2.20544 | -2.20544 | -0.0943 | 0.957931 | 1.133159 | 1.330905 | 1.330905 |
| 5nM-NA | -2.20539 | -2.20539 | -0.67405 | 0.138344 | 1.126117 | 1.933419 | 1.933419 |
| -NA | -2.20539 | -2.20539 | -2.20537 | -0.18736 | 0.85827 | 1.110674 | 1.110674 |
| 50nM+NA | -2.20549 | -2.20549 | -2.20547 | 0.395846 | 2.016248 | 2.062659 | 2.062659 |
| 5nM+NA | -2.20543 | -2.20543 | -0.55311 | 0.240249 | 0.826788 | 1.94723 | 1.94723 |
| +NA | -2.20592 | -2.20592 | -0.69252 | -0.01132 | 0.247583 | 0.680437 | 0.680437 |

## Oneway Anova

### Summary of Fit

| | |
|---|---|
| Rsquare | 0.051184 |
| Adj Rsquare | -0.10695 |
| Root Mean Square Error | 1.420943 |
| Mean of Response | -5.6e-10 |
| Observations (or Sum Wgts) | 36 |

### Analysis of Variance

| Source | DF | Sum of Squares | Mean Square | F Ratio | Prob > F |
|---|---|---|---|---|---|
| Label | 5 | 3.267581 | 0.65352 | 0.3237 | 0.8947 |
| Error | 30 | 60.572400 | 2.01908 | | |
| C. Total | 35 | 63.839981 | | | |

### Means for Oneway Anova

| Level | Number | Mean | Std Error | Lower 95% | Upper 95% |
|---|---|---|---|---|---|
| 50nM-NA | 6 | 0.45300 | 0.58010 | -0.732 | 1.6377 |
| 5nM-NA | 6 | 0.11635 | 0.58010 | -1.068 | 1.3011 |
| -NA | 6 | -0.48344 | 0.58010 | -1.668 | 0.7013 |
| 50nM+NA | 6 | 0.07403 | 0.58010 | -1.111 | 1.2587 |
| 5nM+NA | 6 | 0.11221 | 0.58010 | -1.073 | 1.2969 |
| +NA | 6 | -0.27215 | 0.58010 | -1.457 | 0.9126 |

Std Error uses a pooled estimate of error variance

## Means Comparisons

### Comparisons with a control using Dunnett's Method

## Oneway Analysis of beta-Alanine By Label

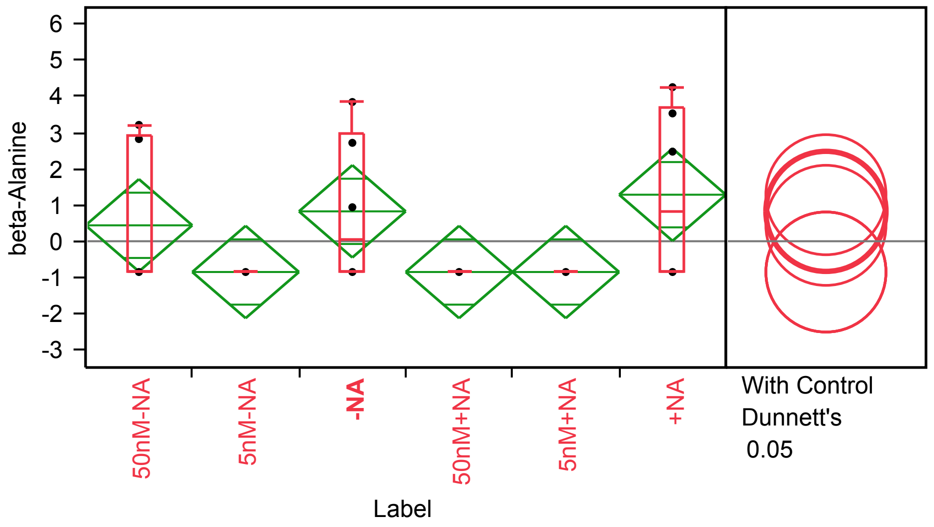


## Quantiles

| Level | Minimum | 10% | 25% | Median | 75% | 90% | Maximum |
|---|---|---|---|---|---|---|---|
| 50nM-NA | -0.84986 | -0.84986 | -0.84984 | -0.8498 | 2.919985 | 3.212418 | 3.212418 |
| 5nM-NA | -0.84986 | -0.84986 | -0.8498 | -0.84974 | -0.8497 | -0.84968 | -0.84968 |
| -NA | -0.84976 | -0.84976 | -0.84974 | 0.044461 | 2.999245 | 3.844765 | 3.844765 |
| 50nM+NA | -0.84986 | -0.84986 | -0.84984 | -0.84982 | -0.8498 | -0.8498 | -0.8498 |
| 5nM+NA | -0.84988 | -0.84988 | -0.84987 | -0.84982 | -0.84977 | -0.84974 | -0.84974 |
| +NA | -0.84978 | -0.84978 | -0.84978 | 0.811989 | 3.711258 | 4.254528 | 4.254528 |

## Oneway Anova

### Summary of Fit

| | |
|---|---|
| Rsquare | 0.286534 |
| Adj Rsquare | 0.167623 |
| Root Mean Square Error | 1.528488 |
| Mean of Response | -5.56e-9 |
| Observations (or Sum Wgts) | 36 |

### Analysis of Variance

| Source | DF | Sum of Squares | Mean Square | F Ratio | Prob > F |
|---|---|---|---|---|---|
| Label | 5 | 28.148008 | 5.62960 | 2.4096 | 0.0597 |
| Error | 30 | 70.088245 | 2.33627 | | |
| C. Total | 35 | 98.236253 | | | |

### Means for Oneway Anova

| Level | Number | Mean | Std Error | Lower 95% | Upper 95% |
|---|---|---|---|---|---|
| 50nM-NA | 6 | 0.4393 | 0.62400 | -0.835 | 1.7137 |
| 5nM-NA | 6 | -0.8498 | 0.62400 | -2.124 | 0.4246 |
| -NA | 6 | 0.8253 | 0.62400 | -0.449 | 2.0996 |
| 50nM+NA | 6 | -0.8498 | 0.62400 | -2.124 | 0.4246 |
| 5nM+NA | 6 | -0.8498 | 0.62400 | -2.124 | 0.4246 |
| +NA | 6 | 1.2849 | 0.62400 | 0.010 | 2.5592 |

Std Error uses a pooled estimate of error variance

## Means Comparisons

### Comparisons with a control using Dunnett's Method

## Oneway Analysis of beta-D-Glucopyranose By Label

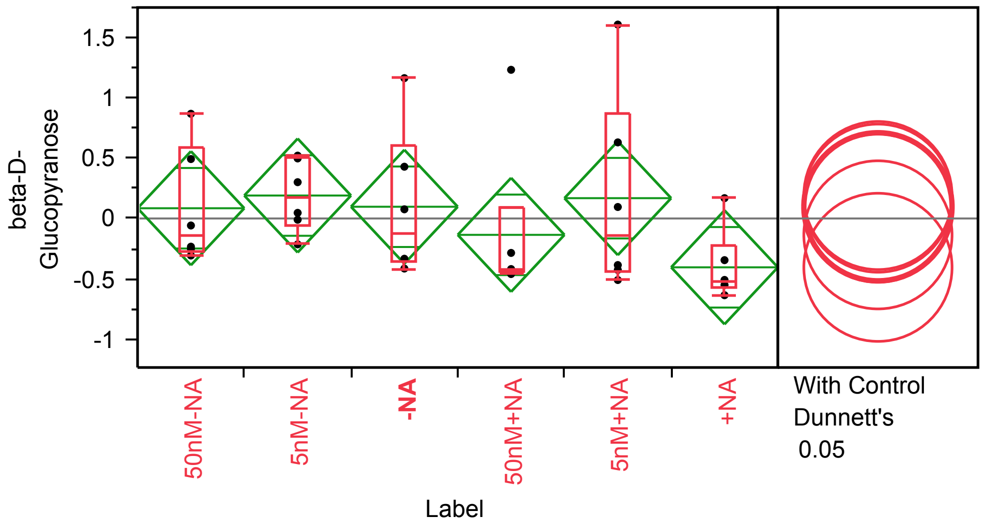


### Quantiles

| Level | Minimum | 10% | 25% | Median | 75% | 90% | Maximum |
|---|---|---|---|---|---|---|---|
| 50nM-NA | -0.30717 | -0.30717 | -0.26618 | -0.14616 | 0.586781 | 0.868374 | 0.868374 |
| 5nM-NA | -0.21459 | -0.21459 | -0.06142 | 0.173874 | 0.503795 | 0.521092 | 0.521092 |
| -NA | -0.41528 | -0.41528 | -0.35617 | -0.12896 | 0.612972 | 1.164071 | 1.164071 |
| 50nM+NA | -0.46091 | -0.46091 | -0.44624 | -0.43009 | 0.094005 | 1.233101 | 1.233101 |
| 5nM+NA | -0.51042 | -0.51042 | -0.44773 | -0.1456 | 0.875364 | 1.608279 | 1.608279 |
| +NA | -0.6365 | -0.6365 | -0.57586 | -0.53075 | -0.2171 | 0.168599 | 0.168599 |

### Oneway Anova

#### Summary of Fit

| | |
|---|---|
| Rsquare | 0.141275 |
| Adj Rsquare | -0.00185 |
| Root Mean Square Error | 0.566351 |
| Mean of Response | 0 |
| Observations (or Sum Wgts) | 36 |

#### Analysis of Variance

| Source | DF | Sum of Squares | Mean Square | F Ratio | Prob > F |
|---|---|---|---|---|---|
| Label | 5 | 1.583087 | 0.316617 | 0.9871 | 0.4420 |
| Error | 30 | 9.622618 | 0.320754 | | |
| C. Total | 35 | 11.205705 | | | |

#### Means for Oneway Anova

| Level | Number | Mean | Std Error | Lower 95% | Upper 95% |
|---|---|---|---|---|---|
| 50nM-NA | 6 | 0.08488 | 0.23121 | -0.3873 | 0.55708 |
| 5nM-NA | 6 | 0.19032 | 0.23121 | -0.2819 | 0.66252 |
| -NA | 6 | 0.09728 | 0.23121 | -0.3749 | 0.56948 |
| 50nM+NA | 6 | -0.13584 | 0.23121 | -0.6080 | 0.33636 |
| 5nM+NA | 6 | 0.16848 | 0.23121 | -0.3037 | 0.64068 |
| +NA | 6 | -0.40512 | 0.23121 | -0.8773 | 0.06708 |

Std Error uses a pooled estimate of error variance

### Means Comparisons

#### Comparisons with a control using Dunnett's Method

## Oneway Analysis of betaine By Label

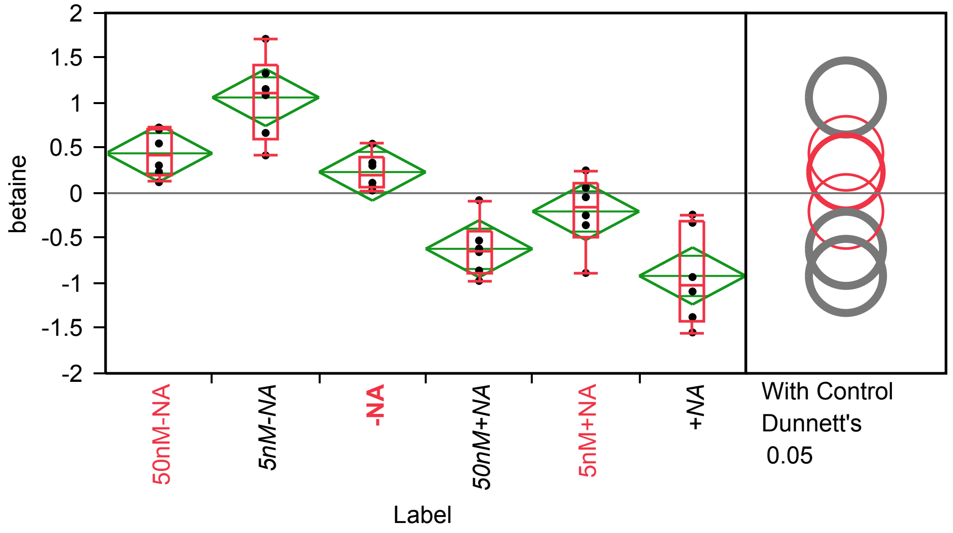


### Quantiles

| Level | Minimum | 10% | 25% | Median | 75% | 90% | Maximum |
|---|---|---|---|---|---|---|---|
| 50nM-NA | 0.122285 | 0.122285 | 0.208063 | 0.429937 | 0.712065 | 0.728499 | 0.728499 |
| 5nM-NA | 0.419249 | 0.419249 | 0.606293 | 1.121939 | 1.425465 | 1.712836 | 1.712836 |
| -NA | 0.030016 | 0.030016 | 0.060448 | 0.209543 | 0.390912 | 0.548527 | 0.548527 |
| 50nM+NA | -0.97364 | -0.97364 | -0.88736 | -0.63458 | -0.41413 | -0.07872 | -0.07872 |
| 5nM+NA | -0.88552 | -0.88552 | -0.48763 | -0.14516 | 0.108727 | 0.252686 | 0.252686 |
| +NA | -1.54572 | -1.54572 | -1.41836 | -1.01128 | -0.30608 | -0.2385 | -0.2385 |

### Oneway Anova

#### Summary of Fit

| | |
|---|---|
| Rsquare | 0.786169 |
| Adj Rsquare | 0.750531 |
| Root Mean Square Error | 0.379368 |
| Mean of Response | 5.56e-10 |
| Observations (or Sum Wgts) | 36 |

#### Analysis of Variance

| Source | DF | Sum of Squares | Mean Square | F Ratio | Prob > F |
|---|---|---|---|---|---|
| Label | 5 | 15.874087 | 3.17482 | 22.0596 | <.0001 * |
| Error | 30 | 4.317605 | 0.14392 | | |
| C. Total | 35 | 20.191691 | | | |

#### Means for Oneway Anova

| Level | Number | Mean | Std Error | Lower 95% | Upper 95% |
|---|---|---|---|---|---|
| 50nM-NA | 6 | 0.4423 | 0.15488 | 0.126 | 0.759 |
| 5nM-NA | 6 | 1.0624 | 0.15488 | 0.746 | 1.379 |
| -NA | 6 | 0.2344 | 0.15488 | -0.082 | 0.551 |
| 50nM+NA | 6 | -0.6177 | 0.15488 | -0.934 | -0.301 |
| 5nM+NA | 6 | -0.2029 | 0.15488 | -0.519 | 0.113 |
| +NA | 6 | -0.9186 | 0.15488 | -1.235 | -0.602 |

Std Error uses a pooled estimate of error variance

### Means Comparisons

#### Comparisons with a control using Dunnett's Method

## Oneway Analysis of betaine aldehyde By Label

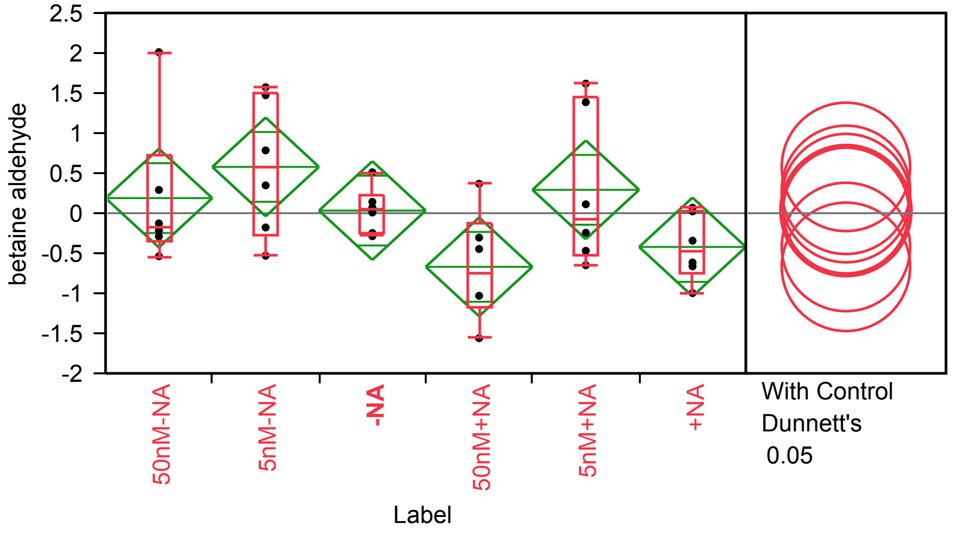


## Quantiles

| Level | Minimum | 10% | 25% | Median | 75% | 90% | Maximum |
|---|---|---|---|---|---|---|---|
| 50nM-NA | -0.53801 | -0.53801 | -0.35174 | -0.17195 | 0.720723 | 2.011233 | 2.011233 |
| 5nM-NA | -0.53087 | -0.53087 | -0.26632 | 0.566034 | 1.498464 | 1.57253 | 1.57253 |
| -NA | -0.28744 | -0.28744 | -0.25734 | 0.03969 | 0.233767 | 0.510583 | 0.510583 |
| 50nM+NA | -1.56096 | -1.56096 | -1.17148 | -0.73924 | -0.13703 | 0.367492 | 0.367492 |
| 5nM+NA | -0.64936 | -0.64936 | -0.51477 | -0.06647 | 1.444096 | 1.617597 | 1.617597 |
| +NA | -0.99717 | -0.99717 | -0.74672 | -0.47959 | 0.033967 | 0.067726 | 0.067726 |

## Oneway Anova

### Summary of Fit

| | |
|---|---|
| Rsquare | 0.283705 |
| Adj Rsquare | 0.164323 |
| Root Mean Square Error | 0.739318 |
| Mean of Response | 2.78e-11 |
| Observations (or Sum Wgts) | 36 |

### Analysis of Variance

| Source | DF | Sum of Squares | Mean Square | F Ratio | Prob > F |
|---|---|---|---|---|---|
| Label | 5 | 6.494701 | 1.29894 | 2.3764 | 0.0625 |
| Error | 30 | 16.397737 | 0.54659 | | |
| C. Total | 35 | 22.892439 | | | |

### Means for Oneway Anova

| Level | Number | Mean | Std Error | Lower 95% | Upper 95% |
|---|---|---|---|---|---|
| 50nM-NA | 6 | 0.18837 | 0.30183 | -0.428 | 0.805 |
| 5nM-NA | 6 | 0.57823 | 0.30183 | -0.038 | 1.195 |
| -NA | 6 | 0.03278 | 0.30183 | -0.584 | 0.649 |
| 50nM+NA | 6 | -0.66980 | 0.30183 | -1.286 | -0.053 |
| 5nM+NA | 6 | 0.29194 | 0.30183 | -0.324 | 0.908 |
| +NA | 6 | -0.42152 | 0.30183 | -1.038 | 0.195 |

Std Error uses a pooled estimate of error variance

## Means Comparisons

### Comparisons with a control using Dunnett's Method

## Oneway Analysis of Butyrylcarnitine By Label

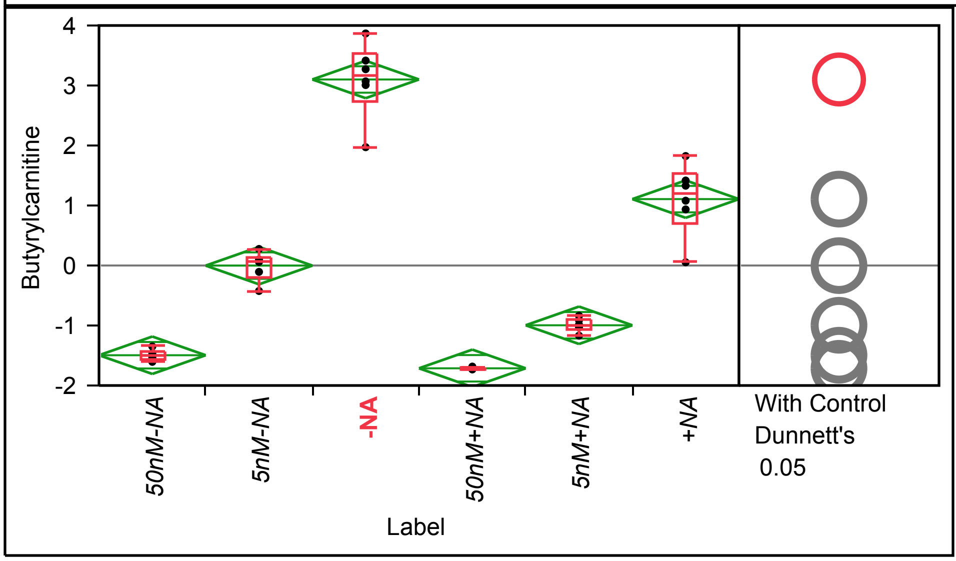


## Quantiles

| Level | Minimum | 10% | 25% | Median | 75% | 90% | Maximum |
|---|---|---|---|---|---|---|---|
| 50nM-NA | -1.60572 | -1.60572 | -1.567 | -1.50035 | -1.43485 | -1.34971 | -1.34971 |
| 5nM-NA | -0.42467 | -0.42467 | -0.18574 | 0.072688 | 0.143446 | 0.275603 | 0.275603 |
| -NA | 1.971726 | 1.971726 | 2.748522 | 3.170196 | 3.528478 | 3.867563 | 3.867563 |
| 50nM+NA | -1.73207 | -1.73207 | -1.72397 | -1.71572 | -1.70432 | -1.68498 | -1.68498 |
| 5nM+NA | -1.16932 | -1.16932 | -1.07267 | -1.00928 | -0.89289 | -0.83727 | -0.83727 |
| +NA | 0.053647 | 0.053647 | 0.714519 | 1.204953 | 1.518238 | 1.824189 | 1.824189 |

## Oneway Anova

### Summary of Fit

| | |
|---|---|
| Rsquare | 0.960505 |
| Adj Rsquare | 0.953922 |
| Root Mean Square Error | 0.373927 |
| Mean of Response | 5.56e-10 |
| Observations (or Sum Wgts) | 36 |

### Analysis of Variance

| Source | DF | Sum of Squares | Mean Square | F Ratio | Prob > F |
|---|---|---|---|---|---|
| Label | 5 | 102.01113 | 20.4022 | 145.9165 | <.0001 * |
| Error | 30 | 4.19464 | 0.1398 | | |
| C. Total | 35 | 106.20577 | | | |

### Means for Oneway Anova

| Level | Number | Mean | Std Error | Lower 95% | Upper 95% |
|---|---|---|---|---|---|
| 50nM-NA | 6 | -1.4956 | 0.15265 | -1.807 | -1.184 |
| 5nM-NA | 6 | -0.0017 | 0.15265 | -0.313 | 0.310 |
| -NA | 6 | 3.1004 | 0.15265 | 2.789 | 3.412 |
| 50nM+NA | 6 | -1.7134 | 0.15265 | -2.025 | -1.402 |
| 5nM+NA | 6 | -0.9962 | 0.15265 | -1.308 | -0.684 |
| +NA | 6 | 1.1065 | 0.15265 | 0.795 | 1.418 |

Std Error uses a pooled estimate of error variance

## Means Comparisons

### Comparisons with a control using Dunnett's Method

## Oneway Analysis of Carbamoyl phosphate By Label

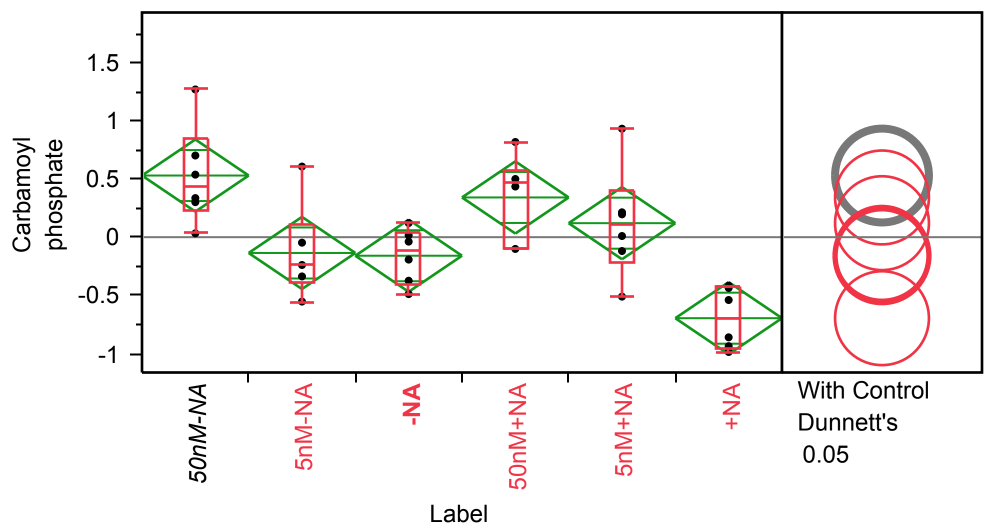


## Quantiles

| Level | Minimum | 10% | 25% | Median | 75% | 90% | Maximum |
|---|---|---|---|---|---|---|---|
| 50nM-NA | 0.033141 | 0.033141 | 0.234063 | 0.436542 | 0.843483 | 1.269066 | 1.269066 |
| 5nM-NA | -0.55337 | -0.55337 | -0.39141 | -0.24154 | 0.116193 | 0.606559 | 0.606559 |
| -NA | -0.48896 | -0.48896 | -0.40307 | -0.1154 | 0.042646 | 0.121189 | 0.121189 |
| 50nM+NA | -0.10122 | -0.10122 | -0.1012 | 0.466416 | 0.579295 | 0.817818 | 0.817818 |
| 5nM+NA | -0.5108 | -0.5108 | -0.21691 | 0.103047 | 0.393193 | 0.931902 | 0.931902 |
| +NA | -0.98641 | -0.98641 | -0.94892 | -0.70026 | -0.4315 | -0.41705 | -0.41705 |

## Oneway Anova

### Summary of Fit

| | |
|---|---|
| Rsquare | 0.576316 |
| Adj Rsquare | 0.505702 |
| Root Mean Square Error | 0.371757 |
| Mean of Response | -2.8e-11 |
| Observations (or Sum Wgts) | 36 |

### Analysis of Variance

| Source | DF | Sum of Squares | Mean Square | F Ratio | Prob > F |
|---|---|---|---|---|---|
| Label | 5 | 5.6397367 | 1.12795 | 8.1615 | <.0001 * |
| Error | 30 | 4.1461004 | 0.13820 | | |
| C. Total | 35 | 9.7858370 | | | |

### Means for Oneway Anova

| Level | Number | Mean | Std Error | Lower 95% | Upper 95% |
|---|---|---|---|---|---|
| 50nM-NA | 6 | 0.52966 | 0.15177 | 0.220 | 0.8396 |
| 5nM-NA | 6 | -0.13576 | 0.15177 | -0.446 | 0.1742 |
| -NA | 6 | -0.15942 | 0.15177 | -0.469 | 0.1505 |
| 50nM+NA | 6 | 0.34134 | 0.15177 | 0.031 | 0.6513 |
| 5nM+NA | 6 | 0.12031 | 0.15177 | -0.190 | 0.4303 |
| +NA | 6 | -0.69612 | 0.15177 | -1.006 | -0.3862 |

Std Error uses a pooled estimate of error variance

## Means Comparisons

### Comparisons with a control using Dunnett's Method

## Oneway Analysis of carnitine By Label

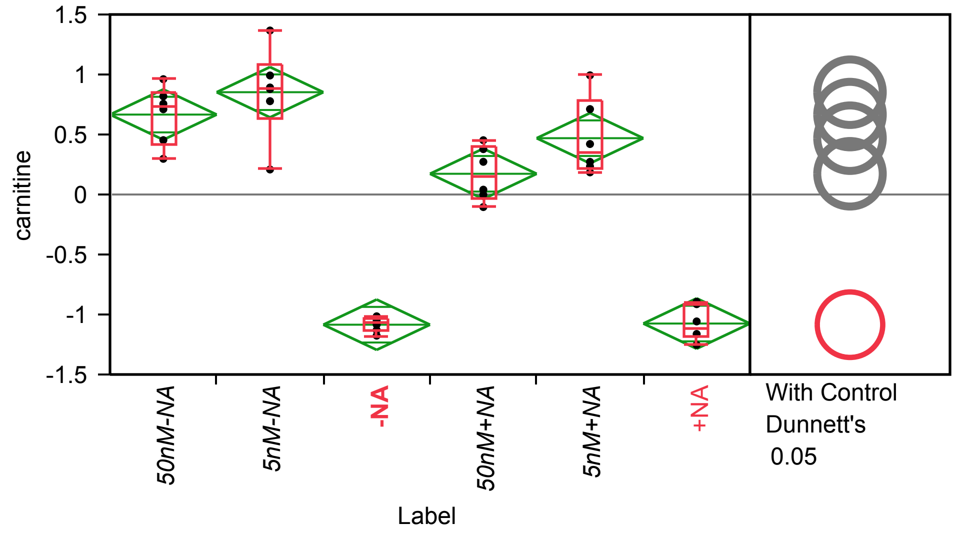


### Quantiles

| Level | Minimum | 10% | 25% | Median | 75% | 90% | Maximum |
|---|---|---|---|---|---|---|---|
| 50nM-NA | 0.297477 | 0.297477 | 0.413949 | 0.733194 | 0.853926 | 0.960944 | 0.960944 |
| 5nM-NA | 0.208587 | 0.208587 | 0.63546 | 0.88511 | 1.085496 | 1.366386 | 1.366386 |
| -NA | -1.17626 | -1.17626 | -1.14148 | -1.07321 | -1.03567 | -1.01511 | -1.01511 |
| 50nM+NA | -0.10464 | -0.10464 | -0.02778 | 0.156171 | 0.396651 | 0.452214 | 0.452214 |
| 5nM+NA | 0.184757 | 0.184757 | 0.2214 | 0.346315 | 0.78194 | 0.99303 | 0.99303 |
| +NA | -1.25298 | -1.25298 | -1.19069 | -1.11025 | -0.90906 | -0.89464 | -0.89464 |

### Oneway Anova

#### Summary of Fit

| | |
|---|---|
| Rsquare | 0.922266 |
| Adj Rsquare | 0.909311 |
| Root Mean Square Error | 0.251553 |
| Mean of Response | -2.8e-11 |
| Observations (or Sum Wgts) | 36 |

#### Analysis of Variance

| Source | DF | Sum of Squares | Mean Square | F Ratio | Prob > F |
|---|---|---|---|---|---|
| Label | 5 | 22.523141 | 4.50463 | 71.1867 | <.0001 * |
| Error | 30 | 1.898372 | 0.06328 | | |
| C. Total | 35 | 24.421513 | | | |

#### Means for Oneway Anova

| Level | Number | Mean | Std Error | Lower 95% | Upper 95% |
|---|---|---|---|---|---|
| 50nM-NA | 6 | 0.6660 | 0.10270 | 0.456 | 0.876 |
| 5nM-NA | 6 | 0.8525 | 0.10270 | 0.643 | 1.062 |
| -NA | 6 | -1.0850 | 0.10270 | -1.295 | -0.875 |
| 50nM+NA | 6 | 0.1726 | 0.10270 | -0.037 | 0.382 |
| 5nM+NA | 6 | 0.4693 | 0.10270 | 0.260 | 0.679 |
| +NA | 6 | -1.0753 | 0.10270 | -1.285 | -0.866 |

Std Error uses a pooled estimate of error variance

### Means Comparisons

#### Comparisons with a control using Dunnett's Method

## Oneway Analysis of CDP By Label

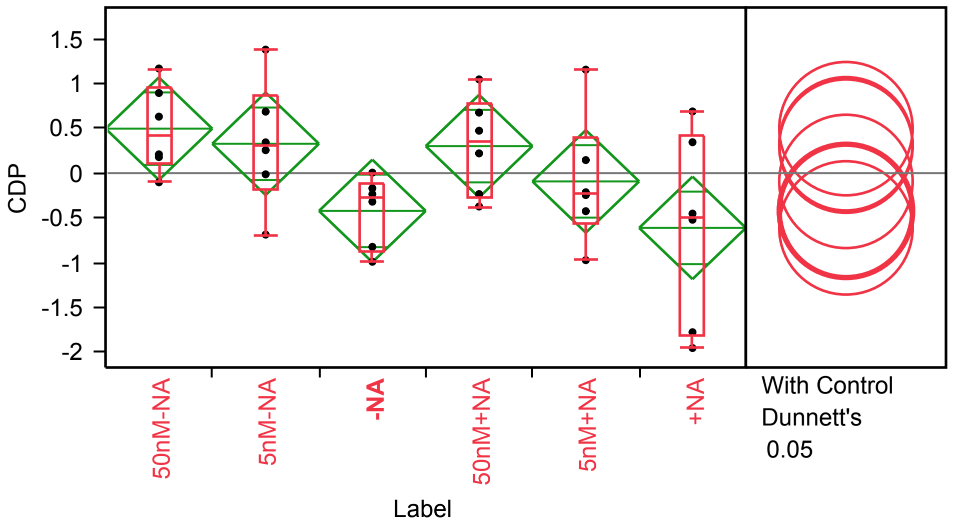


## Quantiles

| Level | Minimum | 10% | 25% | Median | 75% | 90% | Maximum |
|---|---|---|---|---|---|---|---|
| 50nM-NA | -0.10233 | -0.10233 | 0.108562 | 0.420131 | 0.963178 | 1.168386 | 1.168386 |
| 5nM-NA | -0.68668 | -0.68668 | -0.18172 | 0.300116 | 0.859747 | 1.37908 | 1.37908 |
| -NA | -0.9901 | -0.9901 | -0.86533 | -0.27698 | -0.12546 | 0.005174 | 0.005174 |
| 50nM+NA | -0.37285 | -0.37285 | -0.26851 | 0.347117 | 0.768736 | 1.046851 | 1.046851 |
| 5nM+NA | -0.97205 | -0.97205 | -0.562 | -0.22936 | 0.398156 | 1.155479 | 1.155479 |
| +NA | -1.95389 | -1.95389 | -1.82126 | -0.48644 | 0.431072 | 0.688817 | 0.688817 |

## Oneway Anova

### Summary of Fit

| | |
|---|---|
| Rsquare | 0.299106 |
| Adj Rsquare | 0.182291 |
| Root Mean Square Error | 0.686557 |
| Mean of Response | -1.1e-10 |
| Observations (or Sum Wgts) | 36 |

### Analysis of Variance

| Source | DF | Sum of Squares | Mean Square | F Ratio | Prob > F |
|---|---|---|---|---|---|
| Label | 5 | 6.034591 | 1.20692 | 2.5605 | 0.0481 * |
| Error | 30 | 14.140805 | 0.47136 | | |
| C. Total | 35 | 20.175396 | | | |

### Means for Oneway Anova

| Level | Number | Mean | Std Error | Lower 95% | Upper 95% |
|---|---|---|---|---|---|
| 50nM-NA | 6 | 0.49666 | 0.28029 | -0.076 | 1.069 |
| 5nM-NA | 6 | 0.32764 | 0.28029 | -0.245 | 0.900 |
| -NA | 6 | -0.42194 | 0.28029 | -0.994 | 0.150 |
| 50nM+NA | 6 | 0.30176 | 0.28029 | -0.271 | 0.874 |
| 5nM+NA | 6 | -0.09248 | 0.28029 | -0.665 | 0.480 |
| +NA | 6 | -0.61164 | 0.28029 | -1.184 | -0.039 |

Std Error uses a pooled estimate of error variance

## Means Comparisons

### Comparisons with a control using Dunnett's Method

## Oneway Analysis of Cholesterol By Label

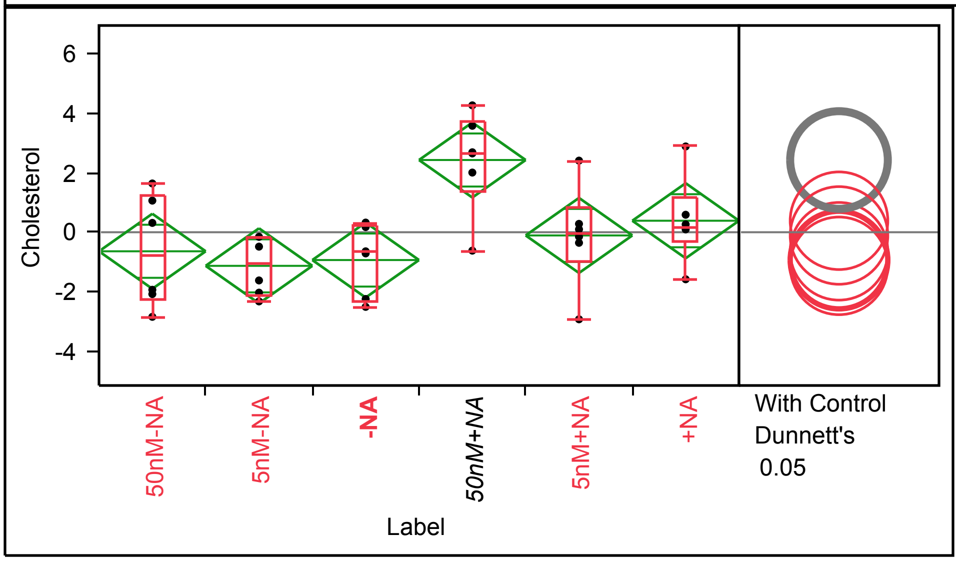


### Quantiles

| Level | Minimum | 10% | 25% | Median | 75% | 90% | Maximum |
|---|---|---|---|---|---|---|---|
| 50nM-NA | -2.84656 | -2.84656 | -2.27238 | -0.81164 | 1.207565 | 1.63996 | 1.63996 |
| 5nM-NA | -2.32711 | -2.32711 | -2.10679 | -1.05359 | -0.16226 | -0.15559 | -0.15559 |
| -NA | -2.51119 | -2.51119 | -2.31333 | -0.67774 | 0.21173 | 0.324181 | 0.324181 |
| 50nM+NA | -0.61897 | -0.61897 | 1.350805 | 2.670509 | 3.753075 | 4.268537 | 4.268537 |
| 5nM+NA | -2.92899 | -2.92899 | -0.99927 | -0.02673 | 0.813017 | 2.408627 | 2.408627 |
| +NA | -1.58309 | -1.58309 | -0.3423 | 0.179221 | 1.16235 | 2.885683 | 2.885683 |

### Oneway Anova

#### Summary of Fit

| | |
|---|---|
| Rsquare | 0.429515 |
| Adj Rsquare | 0.334434 |
| Root Mean Square Error | 1.514315 |
| Mean of Response | 5.56e-10 |
| Observations (or Sum Wgts) | 36 |

#### Analysis of Variance

| Source | DF | Sum of Squares | Mean Square | F Ratio | Prob > F |
|---|---|---|---|---|---|
| Label | 5 | 51.79491 | 10.3590 | 4.5174 | 0.0035 * |
| Error | 30 | 68.79446 | 2.2931 | | |
| C. Total | 35 | 120.58937 | | | |

#### Means for Oneway Anova

| Level | Number | Mean | Std Error | Lower 95% | Upper 95% |
|---|---|---|---|---|---|
| 50nM-NA | 6 | -0.6412 | 0.61822 | -1.904 | 0.6213 |
| 5nM-NA | 6 | -1.1313 | 0.61822 | -2.394 | 0.1313 |
| -NA | 6 | -0.9359 | 0.61822 | -2.199 | 0.3266 |
| 50nM+NA | 6 | 2.4299 | 0.61822 | 1.167 | 3.6924 |
| 5nM+NA | 6 | -0.1081 | 0.61822 | -1.371 | 1.1544 |
| +NA | 6 | 0.3867 | 0.61822 | -0.876 | 1.6493 |

Std Error uses a pooled estimate of error variance

### Means Comparisons

#### Comparisons with a control using Dunnett's Method

## Oneway Analysis of choline By Label

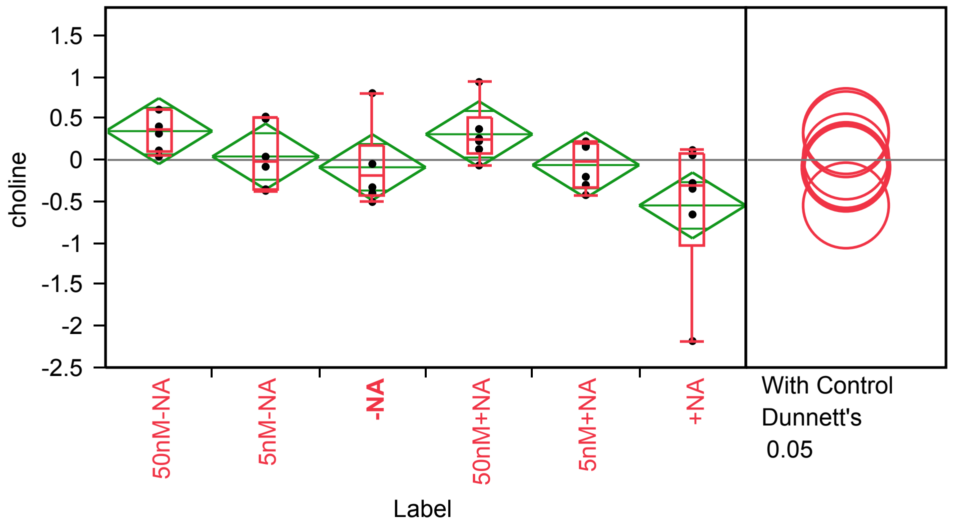


## Quantiles

| Level | Minimum | 10% | 25% | Median | 75% | 90% | Maximum |
|---|---|---|---|---|---|---|---|
| 50nM-NA | 0.043262 | 0.043262 | 0.09603 | 0.360174 | 0.600594 | 0.605887 | 0.605887 |
| 5nM-NA | -0.37177 | -0.37177 | -0.35829 | -0.0212 | 0.502189 | 0.521495 | 0.521495 |
| -NA | -0.50617 | -0.50617 | -0.42757 | -0.18973 | 0.164954 | 0.804578 | 0.804578 |
| 50nM+NA | -0.06664 | -0.06664 | 0.080181 | 0.242078 | 0.513551 | 0.938341 | 0.938341 |
| 5nM+NA | -0.42345 | -0.42345 | -0.33114 | -0.02266 | 0.187595 | 0.223817 | 0.223817 |
| +NA | -2.18158 | -2.18158 | -1.03824 | -0.31263 | 0.073002 | 0.118409 | 0.118409 |

## Oneway Anova

### Summary of Fit

| | |
|---|---|
| Rsquare | 0.31943 |
| Adj Rsquare | 0.206002 |
| Root Mean Square Error | 0.475129 |
| Mean of Response | -5.6e-10 |
| Observations (or Sum Wgts) | 36 |

### Analysis of Variance

| Source | DF | Sum of Squares | Mean Square | F Ratio | Prob > F |
|---|---|---|---|---|---|
| Label | 5 | 3.1786961 | 0.635739 | 2.8161 | 0.0335 * |
| Error | 30 | 6.7724408 | 0.225748 | | |
| C. Total | 35 | 9.9511369 | | | |

### Means for Oneway Anova

| Level | Number | Mean | Std Error | Lower 95% | Upper 95% |
|---|---|---|---|---|---|
| 50nM-NA | 6 | 0.34699 | 0.19397 | -0.0492 | 0.7431 |
| 5nM-NA | 6 | 0.04155 | 0.19397 | -0.3546 | 0.4377 |
| -NA | 6 | -0.08844 | 0.19397 | -0.4846 | 0.3077 |
| 50nM+NA | 6 | 0.30949 | 0.19397 | -0.0867 | 0.7056 |
| 5nM+NA | 6 | -0.06163 | 0.19397 | -0.4578 | 0.3345 |
| +NA | 6 | -0.54795 | 0.19397 | -0.9441 | -0.1518 |

Std Error uses a pooled estimate of error variance

## Means Comparisons

### Comparisons with a control using Dunnett's Method

## Oneway Analysis of Chondroitin By Label

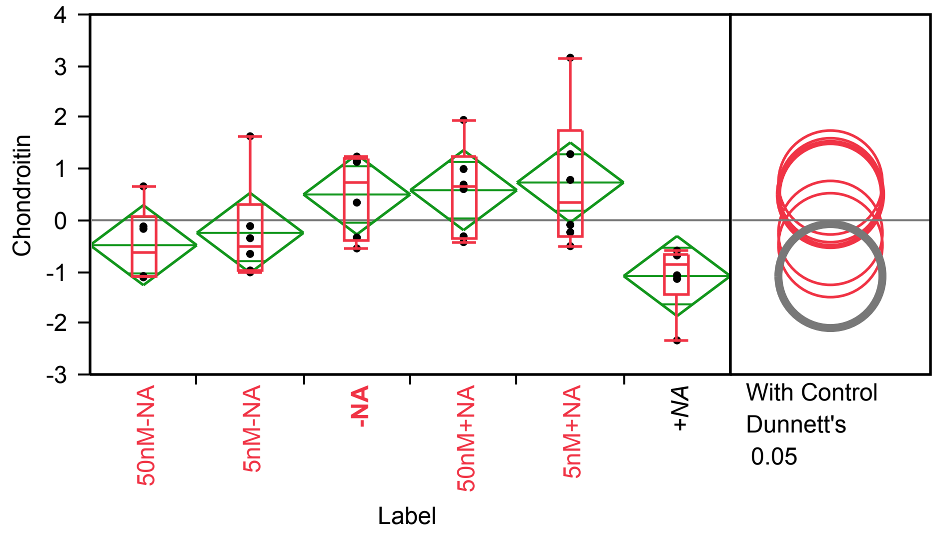


## Quantiles

| Level | Minimum | 10% | 25% | Median | 75% | 90% | Maximum |
|---|---|---|---|---|---|---|---|
| 50nM-NA | -1.10974 | -1.10974 | -1.09217 | -0.62632 | 0.073825 | 0.658421 | 0.658421 |
| 5nM-NA | -1.01308 | -1.01308 | -0.9864 | -0.50235 | 0.321889 | 1.632012 | 1.632012 |
| -NA | -0.54869 | -0.54869 | -0.38923 | 0.738717 | 1.188874 | 1.236908 | 1.236908 |
| 50nM+NA | -0.42544 | -0.42544 | -0.34159 | 0.648759 | 1.233935 | 1.949238 | 1.949238 |
| 5nM+NA | -0.50437 | -0.50437 | -0.30145 | 0.34644 | 1.753304 | 3.158821 | 3.158821 |
| +NA | -2.33675 | -2.33675 | -1.43992 | -0.88005 | -0.66182 | -0.59139 | -0.59139 |

## Oneway Anova

### Summary of Fit

| | |
|---|---|
| Rsquare | 0.374824 |
| Adj Rsquare | 0.270628 |
| Root Mean Square Error | 0.931954 |
| Mean of Response | 5.56e-10 |
| Observations (or Sum Wgts) | 36 |

### Analysis of Variance

| Source | DF | Sum of Squares | Mean Square | F Ratio | Prob > F |
|---|---|---|---|---|---|
| Label | 5 | 15.621952 | 3.12439 | 3.5973 | 0.0115 * |
| Error | 30 | 26.056154 | 0.86854 | | |
| C. Total | 35 | 41.678106 | | | |

### Means for Oneway Anova

| Level | Number | Mean | Std Error | Lower 95% | Upper 95% |
|---|---|---|---|---|---|
| 50nM-NA | 6 | -0.4852 | 0.38047 | -1.262 | 0.292 |
| 5nM-NA | 6 | -0.2464 | 0.38047 | -1.023 | 0.531 |
| -NA | 6 | 0.5004 | 0.38047 | -0.277 | 1.277 |
| 50nM+NA | 6 | 0.5839 | 0.38047 | -0.193 | 1.361 |
| 5nM+NA | 6 | 0.7331 | 0.38047 | -0.044 | 1.510 |
| +NA | 6 | -1.0858 | 0.38047 | -1.863 | -0.309 |

Std Error uses a pooled estimate of error variance

## Means Comparisons

### Comparisons with a control using Dunnett's Method

## Oneway Analysis of Citric acid By Label

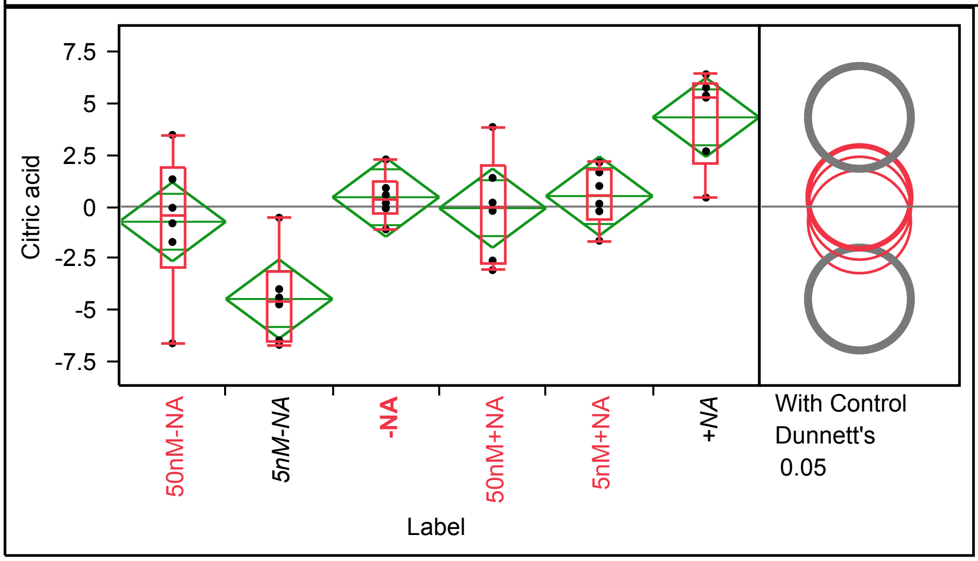


## Quantiles

| Level | Minimum | 10% | 25% | Median | 75% | 90% | Maximum |
|---|---|---|---|---|---|---|---|
| 50nM-NA | -6.62705 | -6.62705 | -2.94419 | -0.43342 | 1.862288 | 3.474572 | 3.474572 |
| 5nM-NA | -6.70506 | -6.70506 | -6.54058 | -4.57774 | -3.1368 | -0.53948 | -0.53948 |
| -NA | -1.10502 | -1.10502 | -0.34079 | 0.364703 | 1.24942 | 2.287295 | 2.287295 |
| 50nM+NA | -3.0793 | -3.0793 | -2.73258 | -0.0071 | 2.008354 | 3.859498 | 3.859498 |
| 5nM+NA | -1.65604 | -1.65604 | -0.58288 | 0.57058 | 1.781055 | 2.140037 | 2.140037 |
| +NA | 0.434736 | 0.434736 | 2.120807 | 5.335073 | 5.926992 | 6.417313 | 6.417313 |

## Oneway Anova

### Summary of Fit

| | |
|---|---|
| Rsquare | 0.601362 |
| Adj Rsquare | 0.534923 |
| Root Mean Square Error | 2.29792 |
| Mean of Response | 5.56e-10 |
| Observations (or Sum Wgts) | 36 |

### Analysis of Variance

| Source | DF | Sum of Squares | Mean Square | F Ratio | Prob > F |
|---|---|---|---|---|---|
| Label | 5 | 238.97313 | 47.7946 | 9.0513 | <.0001 * |
| Error | 30 | 158.41315 | 5.2804 | | |
| C. Total | 35 | 397.38628 | | | |

### Means for Oneway Anova

| Level | Number | Mean | Std Error | Lower 95% | Upper 95% |
|---|---|---|---|---|---|
| 50nM-NA | 6 | -0.7352 | 0.93812 | -2.651 | 1.181 |
| 5nM-NA | 6 | -4.4814 | 0.93812 | -6.397 | -2.565 |
| -NA | 6 | 0.4548 | 0.93812 | -1.461 | 2.371 |
| 50nM+NA | 6 | -0.0766 | 0.93812 | -1.993 | 1.839 |
| 5nM+NA | 6 | 0.5102 | 0.93812 | -1.406 | 2.426 |
| +NA | 6 | 4.3281 | 0.93812 | 2.412 | 6.244 |

Std Error uses a pooled estimate of error variance

## Means Comparisons

### Comparisons with a control using Dunnett's Method

## Oneway Analysis of citrulline By Label

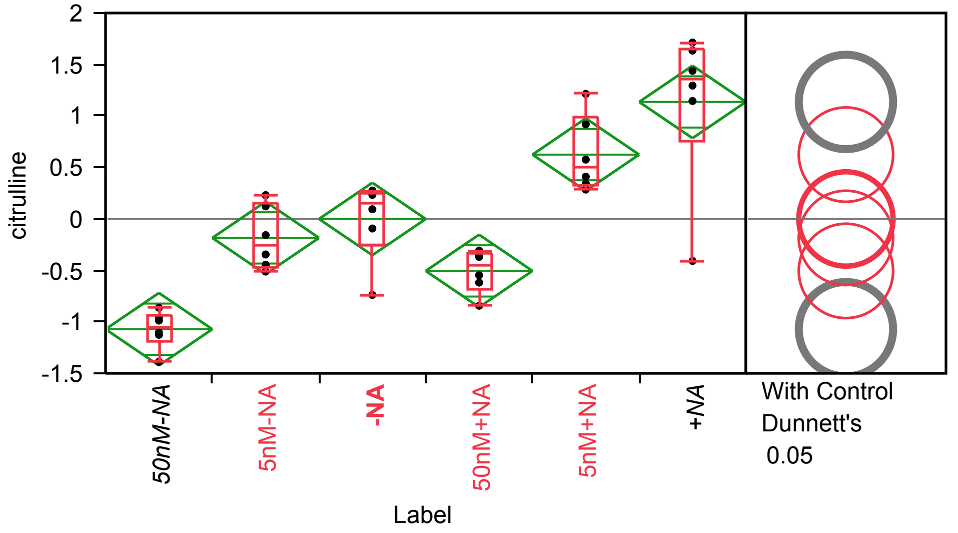


## Quantiles

| Level | Minimum | 10% | 25% | Median | 75% | 90% | Maximum |
|---|---|---|---|---|---|---|---|
| 50nM-NA | -1.38779 | -1.38779 | -1.19132 | -1.04526 | -0.93604 | -0.8626 | -0.8626 |
| 5nM-NA | -0.51127 | -0.51127 | -0.46178 | -0.25138 | 0.149323 | 0.2312 | 0.2312 |
| -NA | -0.73838 | -0.73838 | -0.25443 | 0.160125 | 0.24393 | 0.275551 | 0.275551 |
| 50nM+NA | -0.84316 | -0.84316 | -0.67467 | -0.45839 | -0.335 | -0.30783 | -0.30783 |
| 5nM+NA | 0.285188 | 0.285188 | 0.32869 | 0.493201 | 0.992546 | 1.214149 | 1.214149 |
| +NA | -0.40826 | -0.40826 | 0.757019 | 1.366643 | 1.654383 | 1.713091 | 1.713091 |

## Oneway Anova

### Summary of Fit

| | |
|---|---|
| Rsquare | 0.777572 |
| Adj Rsquare | 0.740501 |
| Root Mean Square Error | 0.42236 |
| Mean of Response | 2.78e-10 |
| Observations (or Sum Wgts) | 36 |

### Analysis of Variance

| Source | DF | Sum of Squares | Mean Square | F Ratio | Prob > F |
|---|---|---|---|---|---|
| Label | 5 | 18.708482 | 3.74170 | 20.9750 | <.0001 * |
| Error | 30 | 5.351647 | 0.17839 | | |
| C. Total | 35 | 24.060129 | | | |

### Means for Oneway Anova

| Level | Number | Mean | Std Error | Lower 95% | Upper 95% |
|---|---|---|---|---|---|
| 50nM-NA | 6 | -1.0712 | 0.17243 | -1.423 | -0.719 |
| 5nM-NA | 6 | -0.1843 | 0.17243 | -0.536 | 0.168 |
| -NA | 6 | -0.00038 | 0.17243 | -0.353 | 0.352 |
| 50nM+NA | 6 | -0.5051 | 0.17243 | -0.857 | -0.153 |
| 5nM+NA | 6 | 0.6246 | 0.17243 | 0.272 | 0.977 |
| +NA | 6 | 1.1364 | 0.17243 | 0.784 | 1.489 |

Std Error uses a pooled estimate of error variance

## Means Comparisons

### Comparisons with a control using Dunnett's Method

## Oneway Analysis of CMP By Label

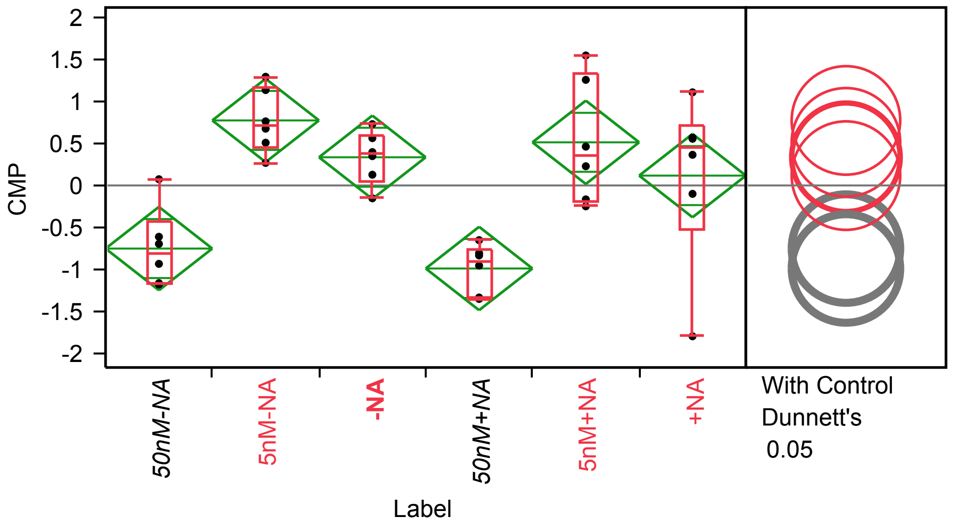


## Quantiles

| Level | Minimum | 10% | 25% | Median | 75% | 90% | Maximum |
|---|---|---|---|---|---|---|---|
| 50nM-NA | -1.17491 | -1.17491 | -1.1632 | -0.81191 | -0.43835 | 0.073696 | 0.073696 |
| 5nM-NA | 0.267453 | 0.267453 | 0.446982 | 0.716868 | 1.170945 | 1.288873 | 1.288873 |
| -NA | -0.15263 | -0.15263 | 0.057233 | 0.371648 | 0.602737 | 0.727137 | 0.727137 |
| 50nM+NA | -1.3465 | -1.3465 | -1.33385 | -0.89078 | -0.76552 | -0.64941 | -0.64941 |
| 5nM+NA | -0.24748 | -0.24748 | -0.18608 | 0.344264 | 1.325127 | 1.541926 | 1.541926 |
| +NA | -1.78938 | -1.78938 | -0.52227 | 0.458579 | 0.701649 | 1.104427 | 1.104427 |

## Oneway Anova

### Summary of Fit

| | |
|---|---|
| Rsquare | 0.588134 |
| Adj Rsquare | 0.51949 |
| Root Mean Square Error | 0.593367 |
| Mean of Response | 5.56e-10 |
| Observations (or Sum Wgts) | 36 |

### Analysis of Variance

| Source | DF | Sum of Squares | Mean Square | F Ratio | Prob > F |
|---|---|---|---|---|---|
| Label | 5 | 15.083044 | 3.01661 | 8.5679 | <.0001 * |
| Error | 30 | 10.562532 | 0.35208 | | |
| C. Total | 35 | 25.645576 | | | |

### Means for Oneway Anova

| Level | Number | Mean | Std Error | Lower 95% | Upper 95% |
|---|---|---|---|---|---|
| 50nM-NA | 6 | -0.74889 | 0.24224 | -1.244 | -0.254 |
| 5nM-NA | 6 | 0.77142 | 0.24224 | 0.277 | 1.266 |
| -NA | 6 | 0.33438 | 0.24224 | -0.160 | 0.829 |
| 50nM+NA | 6 | -0.98522 | 0.24224 | -1.480 | -0.490 |
| 5nM+NA | 6 | 0.51170 | 0.24224 | 0.017 | 1.006 |
| +NA | 6 | 0.11662 | 0.24224 | -0.378 | 0.611 |

Std Error uses a pooled estimate of error variance

## Means Comparisons

### Comparisons with a control using Dunnett's Method

## Oneway Analysis of CMP-N-acetylneuraminic acid By Label

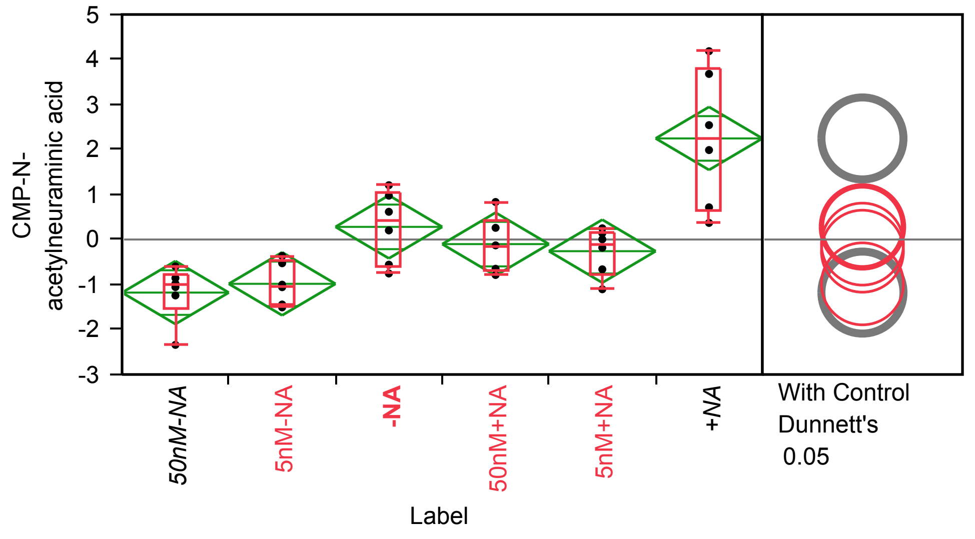


## Quantiles

| Level | Minimum | 10% | 25% | Median | 75% | 90% | Maximum |
|---|---|---|---|---|---|---|---|
| 50nM-NA | -2.34093 | -2.34093 | -1.51728 | -1.01894 | -0.79247 | -0.59843 | -0.59843 |
| 5nM-NA | -1.50791 | -1.50791 | -1.46066 | -1.03219 | -0.48708 | -0.36561 | -0.36561 |
| -NA | -0.75488 | -0.75488 | -0.60851 | 0.408864 | 1.031955 | 1.206422 | 1.206422 |
| 50nM+NA | -0.7881 | -0.7881 | -0.68523 | -0.1342 | 0.402918 | 0.833351 | 0.833351 |
| 5nM+NA | -1.10564 | -1.10564 | -0.77339 | -0.08897 | 0.166474 | 0.248714 | 0.248714 |
| +NA | 0.369068 | 0.369068 | 0.628897 | 2.264318 | 3.805741 | 4.184549 | 4.184549 |

## Oneway Anova

### Summary of Fit

| | |
|---|---|
| Rsquare | 0.682107 |
| Adj Rsquare | 0.629125 |
| Root Mean Square Error | 0.839648 |
| Mean of Response | 4.93e-17 |
| Observations (or Sum Wgts) | 36 |

### Analysis of Variance

| Source | DF | Sum of Squares | Mean Square | F Ratio | Prob > F |
|---|---|---|---|---|---|
| Label | 5 | 45.382383 | 9.07648 | 12.8743 | <.0001 * |
| Error | 30 | 21.150263 | 0.70501 | | |
| C. Total | 35 | 66.532646 | | | |

### Means for Oneway Anova

| Level | Number | Mean | Std Error | Lower 95% | Upper 95% |
|---|---|---|---|---|---|
| 50nM-NA | 6 | -1.1795 | 0.34278 | -1.880 | -0.479 |
| 5nM-NA | 6 | -0.9851 | 0.34278 | -1.685 | -0.285 |
| -NA | 6 | 0.2806 | 0.34278 | -0.420 | 0.981 |
| 50nM+NA | 6 | -0.1024 | 0.34278 | -0.802 | 0.598 |
| 5nM+NA | 6 | -0.2597 | 0.34278 | -0.960 | 0.440 |
| +NA | 6 | 2.2462 | 0.34278 | 1.546 | 2.946 |

Std Error uses a pooled estimate of error variance

## Means Comparisons

### Comparisons with a control using Dunnett's Method

## Oneway Analysis of creatine By Label

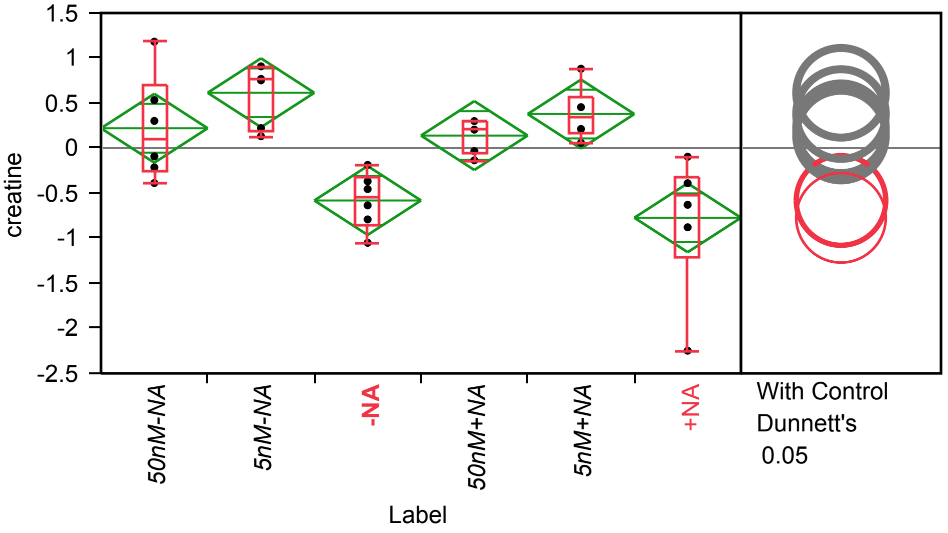


### Quantiles

| Level | Minimum | 10% | 25% | Median | 75% | 90% | Maximum |
|---|---|---|---|---|---|---|---|
| 50nM-NA | -0.38706 | -0.38706 | -0.25685 | 0.105417 | 0.695104 | 1.181597 | 1.181597 |
| 5nM-NA | 0.132105 | 0.132105 | 0.199799 | 0.760616 | 0.90465 | 0.908161 | 0.908161 |
| -NA | -1.05054 | -1.05054 | -0.85623 | -0.54481 | -0.32263 | -0.18635 | -0.18635 |
| 50nM+NA | -0.135 | -0.135 | -0.05867 | 0.204615 | 0.297704 | 0.30013 | 0.30013 |
| 5nM+NA | 0.058881 | 0.058881 | 0.171289 | 0.33375 | 0.561454 | 0.881403 | 0.881403 |
| +NA | -2.24841 | -2.24841 | -1.22106 | -0.51173 | -0.31579 | -0.09602 | -0.09602 |

### Oneway Anova

#### Summary of Fit

| | |
|---|---|
| Rsquare | 0.591588 |
| Adj Rsquare | 0.523519 |
| Root Mean Square Error | 0.458677 |
| Mean of Response | -2.8e-10 |
| Observations (or Sum Wgts) | 36 |

#### Analysis of Variance

| Source | DF | Sum of Squares | Mean Square | F Ratio | Prob > F |
|---|---|---|---|---|---|
| Label | 5 | 9.142294 | 1.82846 | 8.6910 | <.0001 * |
| Error | 30 | 6.311532 | 0.21038 | | |
| C. Total | 35 | 15.453826 | | | |

#### Means for Oneway Anova

| Level | Number | Mean | Std Error | Lower 95% | Upper 95% |
|---|---|---|---|---|---|
| 50nM-NA | 6 | 0.22081 | 0.18725 | -0.162 | 0.6032 |
| 5nM-NA | 6 | 0.61456 | 0.18725 | 0.232 | 0.9970 |
| -NA | 6 | -0.58100 | 0.18725 | -0.963 | -0.1986 |
| 50nM+NA | 6 | 0.13967 | 0.18725 | -0.243 | 0.5221 |
| 5nM+NA | 6 | 0.37856 | 0.18725 | -0.0039 | 0.7610 |
| +NA | 6 | -0.77259 | 0.18725 | -1.155 | -0.3902 |

Std Error uses a pooled estimate of error variance

### Means Comparisons

#### Comparisons with a control using Dunnett's Method

## Oneway Analysis of Creatinine By Label

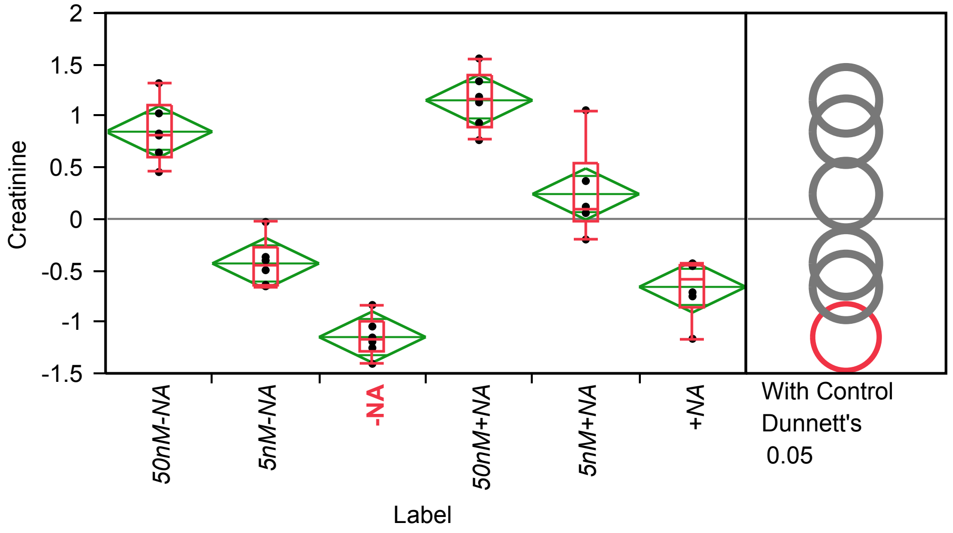


### Quantiles

| Level | Minimum | 10% | 25% | Median | 75% | 90% | Maximum |
|---|---|---|---|---|---|---|---|
| 50nM-NA | 0.455337 | 0.455337 | 0.597391 | 0.819742 | 1.098537 | 1.317918 | 1.317918 |
| 5nM-NA | -0.65442 | -0.65442 | -0.64397 | -0.45072 | -0.28449 | -0.02979 | -0.02979 |
| -NA | -1.40703 | -1.40703 | -1.29172 | -1.17235 | -0.99274 | -0.83623 | -0.83623 |
| 50nM+NA | 0.765479 | 0.765479 | 0.890815 | 1.158928 | 1.392774 | 1.558608 | 1.558608 |
| 5nM+NA | -0.19971 | -0.19971 | -0.01268 | 0.087853 | 0.5399 | 1.055384 | 1.055384 |
| +NA | -1.16426 | -1.16426 | -0.85514 | -0.58522 | -0.44091 | -0.43148 | -0.43148 |

### Oneway Anova

#### Summary of Fit

| | |
|---|---|
| Rsquare | 0.901042 |
| Adj Rsquare | 0.884548 |
| Root Mean Square Error | 0.298024 |
| Mean of Response | -5.6e-10 |
| Observations (or Sum Wgts) | 36 |

#### Analysis of Variance

| Source | DF | Sum of Squares | Mean Square | F Ratio | Prob > F |
|---|---|---|---|---|---|
| Label | 5 | 24.261330 | 4.85227 | 54.6315 | <.0001 * |
| Error | 30 | 2.664542 | 0.08882 | | |
| C. Total | 35 | 26.925872 | | | |

#### Means for Oneway Anova

| Level | Number | Mean | Std Error | Lower 95% | Upper 95% |
|---|---|---|---|---|---|
| 50nM-NA | 6 | 0.8471 | 0.12167 | 0.599 | 1.096 |
| 5nM-NA | 6 | -0.4326 | 0.12167 | -0.681 | -0.184 |
| -NA | 6 | -1.1477 | 0.12167 | -1.396 | -0.899 |
| 50nM+NA | 6 | 1.1520 | 0.12167 | 0.904 | 1.400 |
| 5nM+NA | 6 | 0.2415 | 0.12167 | -0.007 | 0.490 |
| +NA | 6 | -0.6604 | 0.12167 | -0.909 | -0.412 |

Std Error uses a pooled estimate of error variance

### Means Comparisons

#### Comparisons with a control using Dunnett's Method

## Oneway Analysis of Cyclic ADP-ribose By Label

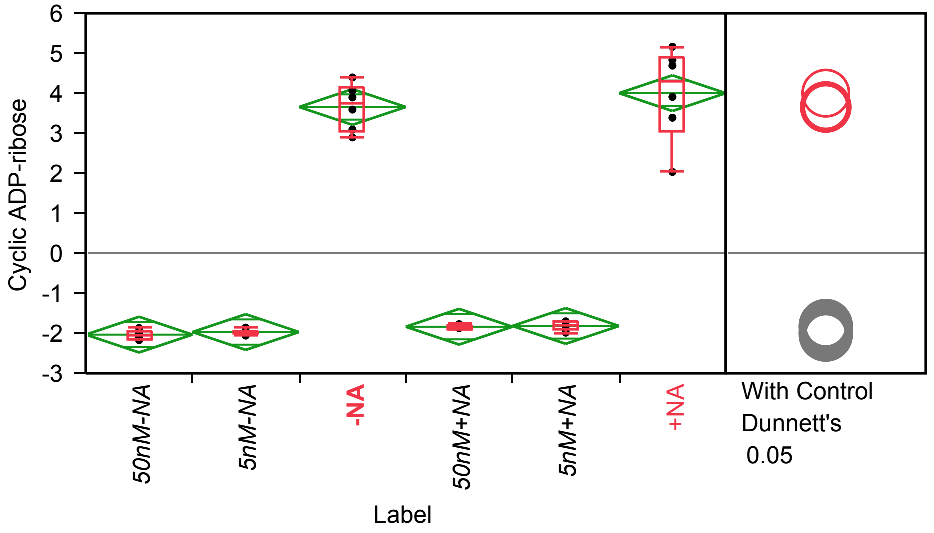


### Quantiles

| Level | Minimum | 10% | 25% | Median | 75% | 90% | Maximum |
|---|---|---|---|---|---|---|---|
| 50nM-NA | -2.172 | -2.172 | -2.12586 | -2.03842 | -1.95071 | -1.8682 | -1.8682 |
| 5nM-NA | -2.0585 | -2.0585 | -2.01869 | -1.97391 | -1.92517 | -1.85947 | -1.85947 |
| -NA | 2.896764 | 2.896764 | 3.043147 | 3.743374 | 4.149887 | 4.394103 | 4.394103 |
| 50nM+NA | -1.88855 | -1.88855 | -1.87866 | -1.84855 | -1.78292 | -1.7729 | -1.7729 |
| 5nM+NA | -1.98573 | -1.98573 | -1.91724 | -1.80541 | -1.7113 | -1.7022 | -1.7022 |
| +NA | 2.032615 | 2.032615 | 3.048999 | 4.299434 | 4.915025 | 5.161477 | 5.161477 |

### Oneway Anova

#### Summary of Fit

| | |
|---|---|
| Rsquare | 0.968536 |
| Adj Rsquare | 0.963292 |
| Root Mean Square Error | 0.535179 |
| Mean of Response | -9.9e-17 |
| Observations (or Sum Wgts) | 36 |

#### Analysis of Variance

| Source | DF | Sum of Squares | Mean Square | F Ratio | Prob > F |
|---|---|---|---|---|---|
| Label | 5 | 264.50011 | 52.9000 | 184.6963 | <.0001 * |
| Error | 30 | 8.59249 | 0.2864 | | |
| C. Total | 35 | 273.09260 | | | |

#### Means for Oneway Anova

| Level | Number | Mean | Std Error | Lower 95% | Upper 95% |
|---|---|---|---|---|---|
| 50nM-NA | 6 | -2.0343 | 0.21849 | -2.480 | -1.588 |
| 5nM-NA | 6 | -1.9697 | 0.21849 | -2.416 | -1.524 |
| -NA | 6 | 3.6563 | 0.21849 | 3.210 | 4.103 |
| 50nM+NA | 6 | -1.8367 | 0.21849 | -2.283 | -1.390 |
| 5nM+NA | 6 | -1.8179 | 0.21849 | -2.264 | -1.372 |
| +NA | 6 | 4.0023 | 0.21849 | 3.556 | 4.448 |

Std Error uses a pooled estimate of error variance

### Means Comparisons

#### Comparisons with a control using Dunnett's Method

## Oneway Analysis of cyclic-AMP By Label

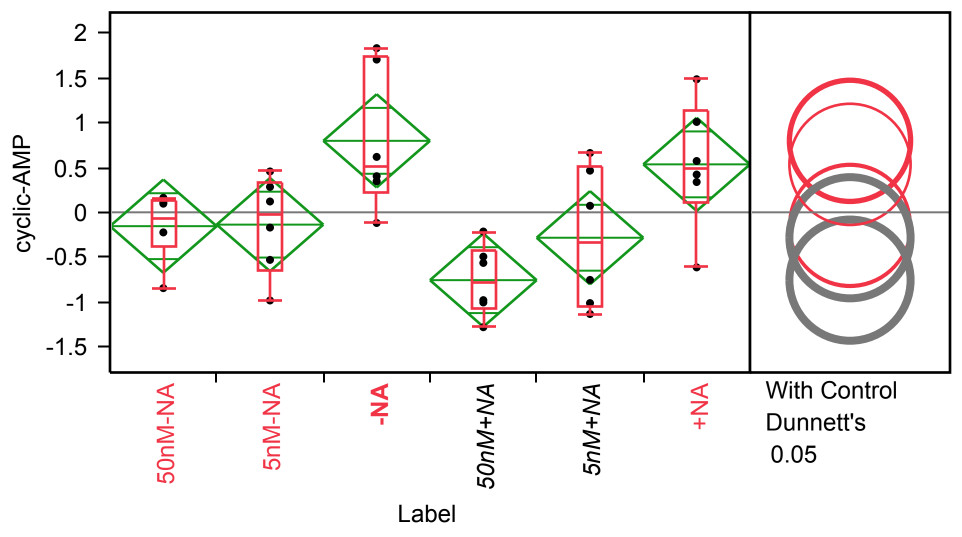


## Quantiles

| Level | Minimum | 10% | 25% | Median | 75% | 90% | Maximum |
|---|---|---|---|---|---|---|---|
| 50nM-NA | -0.8474 | -0.8474 | -0.38266 | -0.06399 | 0.124588 | 0.161753 | 0.161753 |
| 5nM-NA | -0.98525 | -0.98525 | -0.64497 | -0.02474 | 0.327129 | 0.456646 | 0.456646 |
| -NA | -0.11915 | -0.11915 | 0.230988 | 0.511526 | 1.739032 | 1.834879 | 1.834879 |
| 50nM+NA | -1.28413 | -1.28413 | -1.07674 | -0.77366 | -0.4253 | -0.21476 | -0.21476 |
| 5nM+NA | -1.13627 | -1.13627 | -1.04508 | -0.34127 | 0.51489 | 0.662758 | 0.662758 |
| +NA | -0.61543 | -0.61543 | 0.100053 | 0.497681 | 1.129356 | 1.487129 | 1.487129 |

## Oneway Anova

### Summary of Fit

| | |
|---|---|
| Rsquare | 0.454475 |
| Adj Rsquare | 0.363555 |
| Root Mean Square Error | 0.624416 |
| Mean of Response | -2.5e-17 |
| Observations (or Sum Wgts) | 36 |

### Analysis of Variance

| Source | DF | Sum of Squares | Mean Square | F Ratio | Prob > F |
|---|---|---|---|---|---|
| Label | 5 | 9.744623 | 1.94892 | 4.9986 | 0.0019 * |
| Error | 30 | 11.696853 | 0.38990 | | |
| C. Total | 35 | 21.441476 | | | |

### Means for Oneway Anova

| Level | Number | Mean | Std Error | Lower 95% | Upper 95% |
|---|---|---|---|---|---|
| 50nM-NA | 6 | -0.15486 | 0.25492 | -0.675 | 0.366 |
| 5nM-NA | 6 | -0.13761 | 0.25492 | -0.658 | 0.383 |
| -NA | 6 | 0.79893 | 0.25492 | 0.278 | 1.320 |
| 50nM+NA | 6 | -0.75822 | 0.25492 | -1.279 | -0.238 |
| 5nM+NA | 6 | -0.28419 | 0.25492 | -0.805 | 0.236 |
| +NA | 6 | 0.53595 | 0.25492 | 0.015 | 1.057 |

Std Error uses a pooled estimate of error variance

## Means Comparisons

### Comparisons with a control using Dunnett's Method

## Oneway Analysis of cystathionine By Label

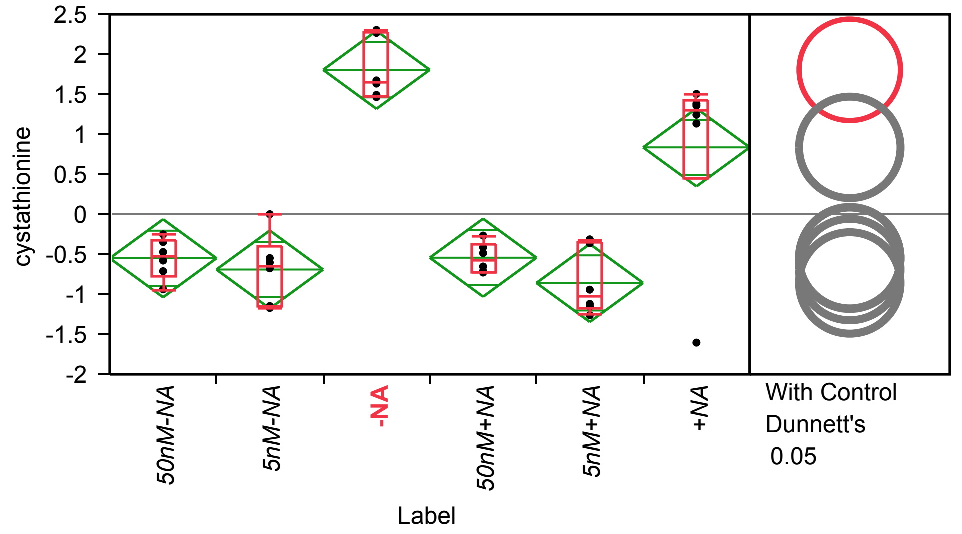


### Quantiles

| Level | Minimum | 10% | 25% | Median | 75% | 90% | Maximum |
|---|---|---|---|---|---|---|---|
| 50nM-NA | -0.93875 | -0.93875 | -0.76835 | -0.52511 | -0.32306 | -0.25125 | -0.25125 |
| 5nM-NA | -1.17119 | -1.17119 | -1.15347 | -0.63947 | -0.41047 | 0.000707 | 0.000707 |
| -NA | 1.46475 | 1.46475 | 1.484678 | 1.65323 | 2.276402 | 2.303491 | 2.303491 |
| 50nM+NA | -0.72832 | -0.72832 | -0.7154 | -0.56901 | -0.37641 | -0.26632 | -0.26632 |
| 5nM+NA | -1.26106 | -1.26106 | -1.17795 | -1.03138 | -0.34998 | -0.31357 | -0.31357 |
| +NA | -1.60417 | -1.60417 | 0.449182 | 1.30051 | 1.412565 | 1.503918 | 1.503918 |

### Oneway Anova

#### Summary of Fit

| | |
|---|---|
| Rsquare | 0.771611 |
| Adj Rsquare | 0.733546 |
| Root Mean Square Error | 0.584434 |
| Mean of Response | 0 |
| Observations (or Sum Wgts) | 36 |

#### Analysis of Variance

| Source | DF | Sum of Squares | Mean Square | F Ratio | Prob > F |
|---|---|---|---|---|---|
| Label | 5 | 34.618988 | 6.92380 | 20.2709 | <.0001 * |
| Error | 30 | 10.246891 | 0.34156 | | |
| C. Total | 35 | 44.865879 | | | |

#### Means for Oneway Anova

| Level | Number | Mean | Std Error | Lower 95% | Upper 95% |
|---|---|---|---|---|---|
| 50nM-NA | 6 | -0.5498 | 0.23859 | -1.037 | -0.063 |
| 5nM-NA | 6 | -0.6908 | 0.23859 | -1.178 | -0.203 |
| -NA | 6 | 1.8056 | 0.23859 | 1.318 | 2.293 |
| 50nM+NA | 6 | -0.5428 | 0.23859 | -1.030 | -0.056 |
| 5nM+NA | 6 | -0.8583 | 0.23859 | -1.346 | -0.371 |
| +NA | 6 | 0.8361 | 0.23859 | 0.349 | 1.323 |

Std Error uses a pooled estimate of error variance

### Means Comparisons

#### Comparisons with a control using Dunnett's Method

# Oneway Analysis of Cysteineglutathione disulfide By Label

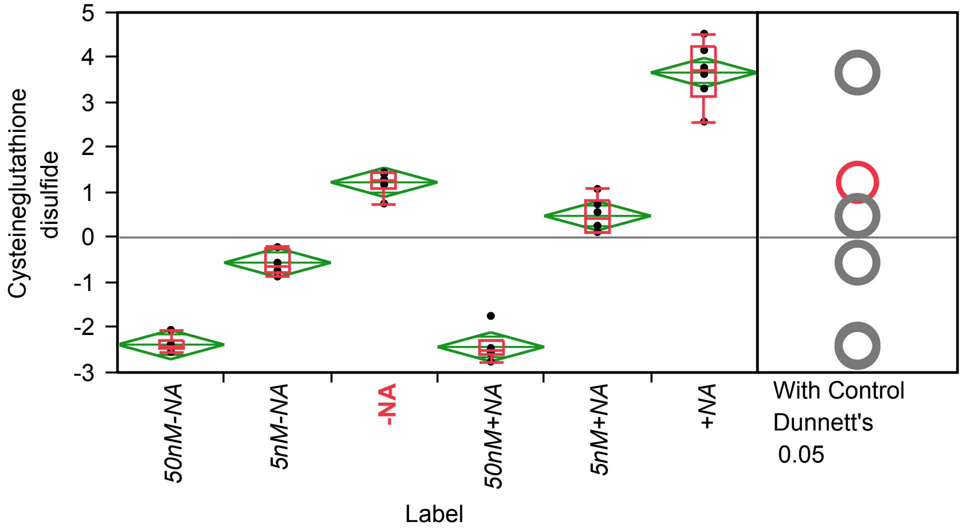


## Quantiles

| Level | Minimum | 10% | 25% | Median | 75% | 90% | Maximum |
|---|---|---|---|---|---|---|---|
| 50nM-NA | -2.54351 | -2.54351 | -2.4694 | -2.43557 | -2.2913 | -2.05319 | -2.05319 |
| 5nM-NA | -0.86662 | -0.86662 | -0.77467 | -0.64582 | -0.23064 | -0.2141 | -0.2141 |
| -NA | 0.754695 | 0.754695 | 1.072733 | 1.278969 | 1.423362 | 1.451949 | 1.451949 |
| 50nM+NA | -2.75962 | -2.75962 | -2.62139 | -2.52932 | -2.27773 | -1.74254 | -1.74254 |
| 5nM+NA | 0.119035 | 0.119035 | 0.12056 | 0.412715 | 0.82395 | 1.077268 | 1.077268 |
| +NA | 2.575387 | 2.575387 | 3.128589 | 3.70415 | 4.254489 | 4.528792 | 4.528792 |

## Oneway Anova

### Summary of Fit

| | |
|---|---|
| Rsquare | 0.9729 |
| Adj Rsquare | 0.968384 |
| Root Mean Square Error | 0.388256 |
| Mean of Response | 2.778e-9 |
| Observations (or Sum Wgts) | 36 |

### Analysis of Variance

| Source | DF | Sum of Squares | Mean Square | F Ratio | Prob > F |
|---|---|---|---|---|---|
| Label | 5 | 162.35235 | 32.4705 | 215.4036 | <.0001 * |
| Error | 30 | 4.52227 | 0.1507 | | |
| C. Total | 35 | 166.87462 | | | |

### Means for Oneway Anova

| Level | Number | Mean | Std Error | Lower 95% | Upper 95% |
|---|---|---|---|---|---|
| 50nM-NA | 6 | -2.3805 | 0.15850 | -2.704 | -2.057 |
| 5nM-NA | 6 | -0.5588 | 0.15850 | -0.882 | -0.235 |
| -NA | 6 | 1.2262 | 0.15850 | 0.902 | 1.550 |
| 50nM+NA | 6 | -2.4320 | 0.15850 | -2.756 | -2.108 |
| 5nM+NA | 6 | 0.4804 | 0.15850 | 0.157 | 0.804 |
| +NA | 6 | 3.6648 | 0.15850 | 3.341 | 3.988 |

Std Error uses a pooled estimate of error variance

## Means Comparisons

### Comparisons with a control using Dunnett's Method

## Oneway Analysis of Cystine By Label

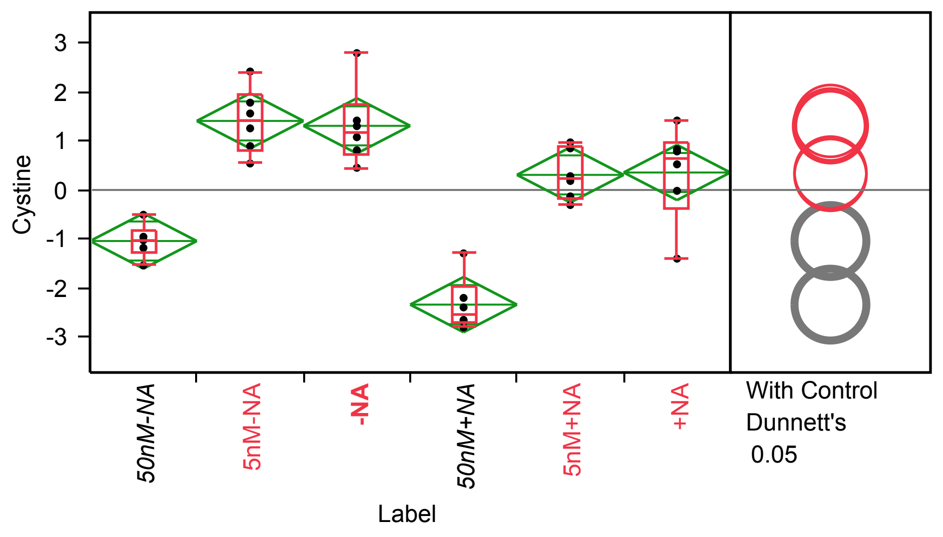


## Quantiles

| Level | Minimum | 10% | 25% | Median | 75% | 90% | Maximum |
|---|---|---|---|---|---|---|---|
| 50nM-NA | -1.54447 | -1.54447 | -1.27552 | -1.03707 | -0.84593 | -0.50813 | -0.50813 |
| 5nM-NA | 0.542279 | 0.542279 | 0.809405 | 1.413857 | 1.946733 | 2.425012 | 2.425012 |
| -NA | 0.453678 | 0.453678 | 0.723268 | 1.19366 | 1.766129 | 2.80153 | 2.80153 |
| 50nM+NA | -2.81499 | -2.81499 | -2.72606 | -2.53218 | -1.98218 | -1.29841 | -1.29841 |
| 5nM+NA | -0.30813 | -0.30813 | -0.17587 | 0.233285 | 0.883031 | 0.974067 | 0.974067 |
| +NA | -1.40628 | -1.40628 | -0.36496 | 0.657084 | 0.980455 | 1.420906 | 1.420906 |

## Oneway Anova

### Summary of Fit

| | |
|---|---|
| Rsquare | 0.821157 |
| Adj Rsquare | 0.79135 |
| Root Mean Square Error | 0.677801 |
| Mean of Response | -2.8e-10 |
| Observations (or Sum Wgts) | 36 |

### Analysis of Variance

| Source | DF | Sum of Squares | Mean Square | F Ratio | Prob > F |
|---|---|---|---|---|---|
| Label | 5 | 63.282213 | 12.6564 | 27.5491 | <.0001 * |
| Error | 30 | 13.782443 | 0.4594 | | |
| C. Total | 35 | 77.064656 | | | |

### Means for Oneway Anova

| Level | Number | Mean | Std Error | Lower 95% | Upper 95% |
|---|---|---|---|---|---|
| 50nM-NA | 6 | -1.0452 | 0.27671 | -1.610 | -0.480 |
| 5nM-NA | 6 | 1.4135 | 0.27671 | 0.848 | 1.979 |
| -NA | 6 | 1.3128 | 0.27671 | 0.748 | 1.878 |
| 50nM+NA | 6 | -2.3474 | 0.27671 | -2.912 | -1.782 |
| 5nM+NA | 6 | 0.3089 | 0.27671 | -0.256 | 0.874 |
| +NA | 6 | 0.3574 | 0.27671 | -0.208 | 0.923 |

Std Error uses a pooled estimate of error variance

## Means Comparisons

### Comparisons with a control using Dunnett's Method

## Oneway Analysis of cytidine By Label

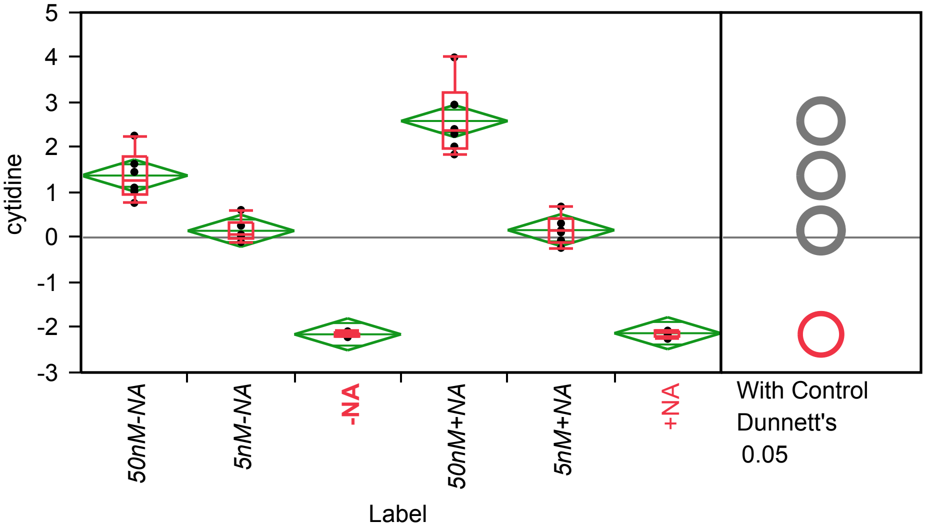


## Quantiles

| Level | Minimum | 10% | 25% | Median | 75% | 90% | Maximum |
|---|---|---|---|---|---|---|---|
| 50nM-NA | 0.768863 | 0.768863 | 0.961987 | 1.282933 | 1.793094 | 2.263768 | 2.263768 |
| 5nM-NA | -0.10217 | -0.10217 | -0.01055 | 0.050832 | 0.349909 | 0.611528 | 0.611528 |
| -NA | -2.21469 | -2.21469 | -2.19591 | -2.16379 | -2.09116 | -2.08499 | -2.08499 |
| 50nM+NA | 1.846733 | 1.846733 | 1.977855 | 2.355754 | 3.214898 | 4.002038 | 4.002038 |
| 5nM+NA | -0.23075 | -0.23075 | -0.10386 | 0.137178 | 0.407649 | 0.686947 | 0.686947 |
| +NA | -2.25181 | -2.25181 | -2.18849 | -2.09795 | -2.07577 | -2.06963 | -2.06963 |

## Oneway Anova

### Summary of Fit

| | |
|---|---|
| Rsquare | 0.951787 |
| Adj Rsquare | 0.943751 |
| Root Mean Square Error | 0.424684 |
| Mean of Response | 2.78e-10 |
| Observations (or Sum Wgts) | 36 |

### Analysis of Variance

| Source | DF | Sum of Squares | Mean Square | F Ratio | Prob > F |
|---|---|---|---|---|---|
| Label | 5 | 106.81361 | 21.3627 | 118.4469 | <.0001 * |
| Error | 30 | 5.41071 | 0.1804 | | |
| C. Total | 35 | 112.22431 | | | |

### Means for Oneway Anova

| Level | Number | Mean | Std Error | Lower 95% | Upper 95% |
|---|---|---|---|---|---|
| 50nM-NA | 6 | 1.3768 | 0.17338 | 1.023 | 1.731 |
| 5nM-NA | 6 | 0.1490 | 0.17338 | -0.205 | 0.503 |
| -NA | 6 | -2.1517 | 0.17338 | -2.506 | -1.798 |
| 50nM+NA | 6 | 2.5891 | 0.17338 | 2.235 | 2.943 |
| 5nM+NA | 6 | 0.1639 | 0.17338 | -0.190 | 0.518 |
| +NA | 6 | -2.1271 | 0.17338 | -2.481 | -1.773 |

Std Error uses a pooled estimate of error variance

## Means Comparisons

### Comparisons with a control using Dunnett's Method

## Oneway Analysis of cytosine By Label

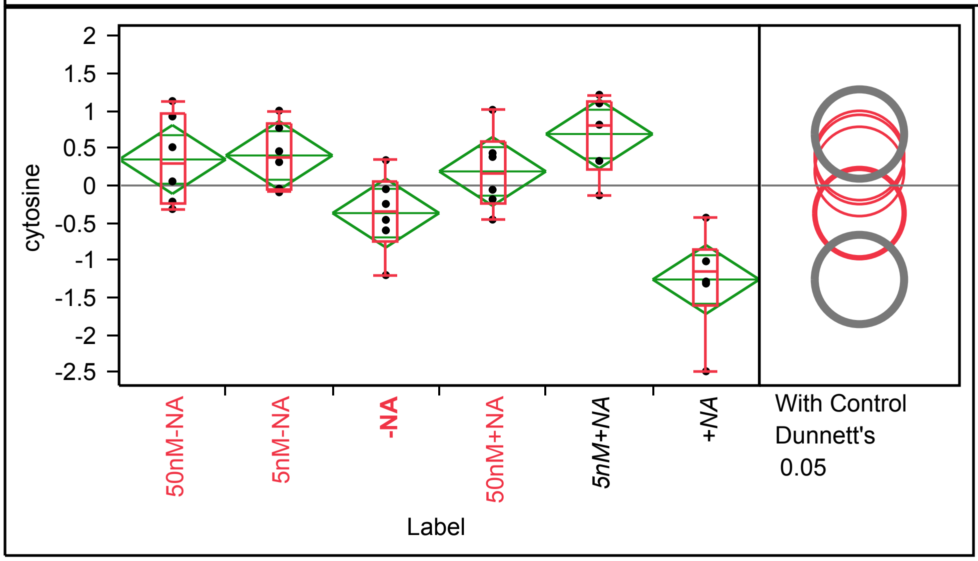


### Quantiles

| Level | Minimum | 10% | 25% | Median | 75% | 90% | Maximum |
|---|---|---|---|---|---|---|---|
| 50nM-NA | -0.31561 | -0.31561 | -0.24107 | 0.284265 | 0.976821 | 1.131485 | 1.131485 |
| 5nM-NA | -0.09087 | -0.09087 | -0.05487 | 0.386488 | 0.83011 | 1.002679 | 1.002679 |
| -NA | -1.20129 | -1.20129 | -0.75113 | -0.35399 | 0.047937 | 0.339415 | 0.339415 |
| 50nM+NA | -0.45695 | -0.45695 | -0.25296 | 0.162965 | 0.579006 | 1.014027 | 1.014027 |
| 5nM+NA | -0.13165 | -0.13165 | 0.214249 | 0.812554 | 1.13009 | 1.215709 | 1.215709 |
| +NA | -2.4901 | -2.4901 | -1.60797 | -1.15417 | -0.86986 | -0.43189 | -0.43189 |

### Oneway Anova

#### Summary of Fit

| | |
|---|---|
| Rsquare | 0.62328 |
| Adj Rsquare | 0.560494 |
| Root Mean Square Error | 0.551928 |
| Mean of Response | -5.6e-10 |
| Observations (or Sum Wgts) | 36 |

#### Analysis of Variance

| Source | DF | Sum of Squares | Mean Square | F Ratio | Prob > F |
|---|---|---|---|---|---|
| Label | 5 | 15.120001 | 3.02400 | 9.9270 | <.0001 * |
| Error | 30 | 9.138751 | 0.30463 | | |
| C. Total | 35 | 24.258752 | | | |

#### Means for Oneway Anova

| Level | Number | Mean | Std Error | Lower 95% | Upper 95% |
|---|---|---|---|---|---|
| 50nM-NA | 6 | 0.3489 | 0.22532 | -0.111 | 0.809 |
| 5nM-NA | 6 | 0.4024 | 0.22532 | -0.058 | 0.863 |
| -NA | 6 | -0.3700 | 0.22532 | -0.830 | 0.090 |
| 50nM+NA | 6 | 0.1887 | 0.22532 | -0.271 | 0.649 |
| 5nM+NA | 6 | 0.6900 | 0.22532 | 0.230 | 1.150 |
| +NA | 6 | -1.2600 | 0.22532 | -1.720 | -0.800 |

Std Error uses a pooled estimate of error variance

### Means Comparisons

#### Comparisons with a control using Dunnett's Method

## Oneway Analysis of dAMP By Label

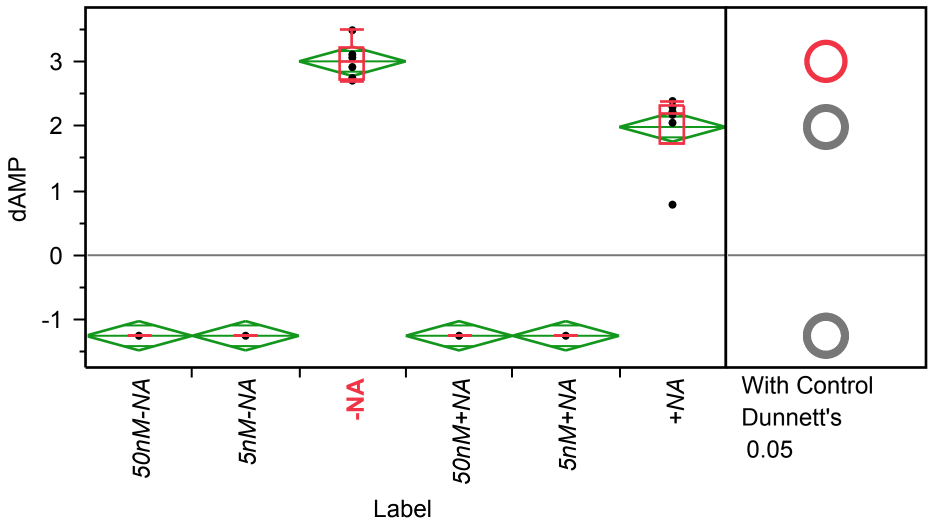


### Quantiles

| Level | Minimum | 10% | 25% | Median | 75% | 90% | Maximum |
|---|---|---|---|---|---|---|---|
| 50nM-NA | -1.24608 | -1.24608 | -1.24607 | -1.24605 | -1.24599 | -1.24594 | -1.24594 |
| 5nM-NA | -1.24608 | -1.24608 | -1.24602 | -1.24597 | -1.24593 | -1.24592 | -1.24592 |
| -NA | 2.701832 | 2.701832 | 2.732119 | 2.984835 | 3.199126 | 3.481271 | 3.481271 |
| 50nM+NA | -1.24608 | -1.24608 | -1.24606 | -1.24605 | -1.24602 | -1.24602 | -1.24602 |
| 5nM+NA | -1.2461 | -1.2461 | -1.24609 | -1.24605 | -1.246 | -1.24597 | -1.24597 |
| +NA | 0.782468 | 0.782468 | 1.732472 | 2.19562 | 2.318335 | 2.385869 | 2.385869 |

### Oneway Anova

#### Summary of Fit

| | |
|---|---|
| Rsquare | 0.981155 |
| Adj Rsquare | 0.978014 |
| Root Mean Square Error | 0.271202 |
| Mean of Response | 5.556e-9 |
| Observations (or Sum Wgts) | 36 |

#### Analysis of Variance

| Source | DF | Sum of Squares | Mean Square | F Ratio | Prob > F |
|---|---|---|---|---|---|
| Label | 5 | 114.88196 | 22.9764 | 312.3881 | <.0001 * |
| Error | 30 | 2.20652 | 0.0736 | | |
| C. Total | 35 | 117.08849 | | | |

#### Means for Oneway Anova

| Level | Number | Mean | Std Error | Lower 95% | Upper 95% |
|---|---|---|---|---|---|
| 50nM-NA | 6 | -1.2460 | 0.11072 | -1.472 | -1.020 |
| 5nM-NA | 6 | -1.2460 | 0.11072 | -1.472 | -1.020 |
| -NA | 6 | 3.0000 | 0.11072 | 2.774 | 3.226 |
| 50nM+NA | 6 | -1.2460 | 0.11072 | -1.472 | -1.020 |
| 5nM+NA | 6 | -1.2460 | 0.11072 | -1.472 | -1.020 |
| +NA | 6 | 1.9841 | 0.11072 | 1.758 | 2.210 |

Std Error uses a pooled estimate of error variance

### Means Comparisons

#### Comparisons with a control using Dunnett's Method

## Oneway Analysis of dCMP By Label

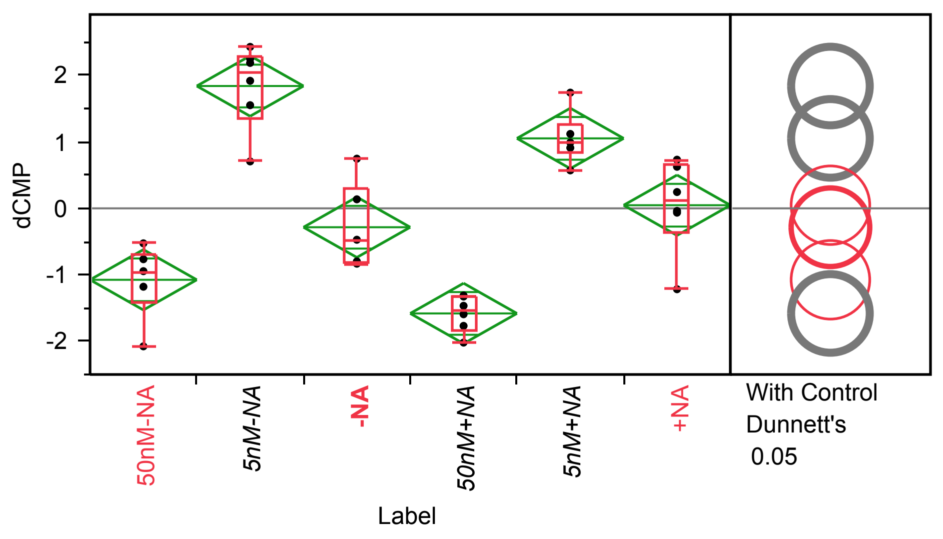


### Quantiles

| Level | Minimum | 10% | 25% | Median | 75% | 90% | Maximum |
|---|---|---|---|---|---|---|---|
| 50nM-NA | -2.07627 | -2.07627 | -1.40357 | -0.95173 | -0.70564 | -0.52662 | -0.52662 |
| 5nM-NA | 0.706389 | 0.706389 | 1.340632 | 2.053578 | 2.294051 | 2.429346 | 2.429346 |
| -NA | -0.83241 | -0.83241 | -0.80683 | -0.47854 | 0.288659 | 0.747092 | 0.747092 |
| 50nM+NA | -2.01978 | -2.01978 | -1.83133 | -1.53 | -1.32301 | -1.31155 | -1.31155 |
| 5nM+NA | 0.571618 | 0.571618 | 0.825493 | 0.987304 | 1.274819 | 1.740636 | 1.740636 |
| +NA | -1.21685 | -1.21685 | -0.35306 | 0.103348 | 0.652207 | 0.731706 | 0.731706 |

### Oneway Anova

#### Summary of Fit

| | |
|---|---|
| Rsquare | 0.847259 |
| Adj Rsquare | 0.821802 |
| Root Mean Square Error | 0.544904 |
| Mean of Response | 2.47e-17 |
| Observations (or Sum Wgts) | 36 |

#### Analysis of Variance

| Source | DF | Sum of Squares | Mean Square | F Ratio | Prob > F |
|---|---|---|---|---|---|
| Label | 5 | 49.410784 | 9.88216 | 33.2822 | <.0001 * |
| Error | 30 | 8.907618 | 0.29692 | | |
| C. Total | 35 | 58.318402 | | | |

#### Means for Oneway Anova

| Level | Number | Mean | Std Error | Lower 95% | Upper 95% |
|---|---|---|---|---|---|
| 50nM-NA | 6 | -1.0752 | 0.22246 | -1.529 | -0.621 |
| 5nM-NA | 6 | 1.8406 | 0.22246 | 1.386 | 2.295 |
| -NA | 6 | -0.2841 | 0.22246 | -0.738 | 0.170 |
| 50nM+NA | 6 | -1.5811 | 0.22246 | -2.035 | -1.127 |
| 5nM+NA | 6 | 1.0528 | 0.22246 | 0.598 | 1.507 |
| +NA | 6 | 0.0470 | 0.22246 | -0.407 | 0.501 |

Std Error uses a pooled estimate of error variance

### Means Comparisons

#### Comparisons with a control using Dunnett's Method

## Oneway Analysis of deoxyadenosine By Label

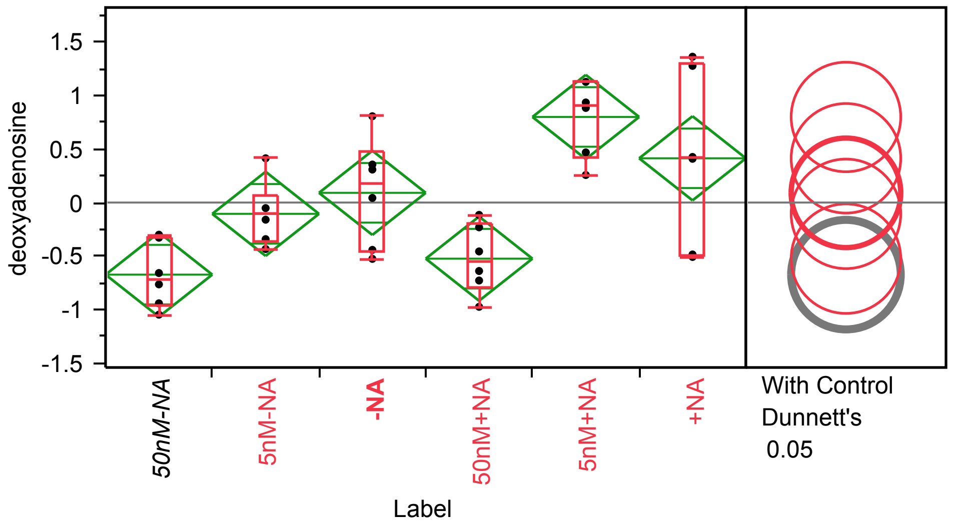


### Quantiles

| Level | Minimum | 10% | 25% | Median | 75% | 90% | Maximum |
|---|---|---|---|---|---|---|---|
| 50nM-NA | -1.04657 | -1.04657 | -0.96732 | -0.71103 | -0.32137 | -0.30058 | -0.30058 |
| 5nM-NA | -0.43376 | -0.43376 | -0.36469 | -0.10942 | 0.064264 | 0.413081 | 0.413081 |
| -NA | -0.52563 | -0.52563 | -0.46293 | 0.175573 | 0.467296 | 0.805535 | 0.805535 |
| 50nM+NA | -0.97343 | -0.97343 | -0.7894 | -0.54784 | -0.20232 | -0.11531 | -0.11531 |
| 5nM+NA | 0.257093 | 0.257093 | 0.415242 | 0.908729 | 1.125219 | 1.126446 | 1.126446 |
| +NA | -0.50791 | -0.50791 | -0.49066 | 0.416959 | 1.297201 | 1.361041 | 1.361041 |

### Oneway Anova

#### Summary of Fit

| | |
|---|---|
| Rsquare | 0.583412 |
| Adj Rsquare | 0.513981 |
| Root Mean Square Error | 0.471368 |
| Mean of Response | 5.56e-10 |
| Observations (or Sum Wgts) | 36 |

#### Analysis of Variance

| Source | DF | Sum of Squares | Mean Square | F Ratio | Prob > F |
|---|---|---|---|---|---|
| Label | 5 | 9.334925 | 1.86699 | 8.4027 | <.0001 * |
| Error | 30 | 6.665641 | 0.22219 | | |
| C. Total | 35 | 16.000566 | | | |

#### Means for Oneway Anova

| Level | Number | Mean | Std Error | Lower 95% | Upper 95% |
|---|---|---|---|---|---|
| 50nM-NA | 6 | -0.67307 | 0.19244 | -1.066 | -0.280 |
| 5nM-NA | 6 | -0.10553 | 0.19244 | -0.499 | 0.287 |
| -NA | 6 | 0.09059 | 0.19244 | -0.302 | 0.484 |
| 50nM+NA | 6 | -0.52397 | 0.19244 | -0.917 | -0.131 |
| 5nM+NA | 6 | 0.79896 | 0.19244 | 0.406 | 1.192 |
| +NA | 6 | 0.41301 | 0.19244 | 0.020 | 0.806 |

Std Error uses a pooled estimate of error variance

### Means Comparisons

#### Comparisons with a control using Dunnett's Method

## Oneway Analysis of Deoxyheptulose-7-phosphate By Label

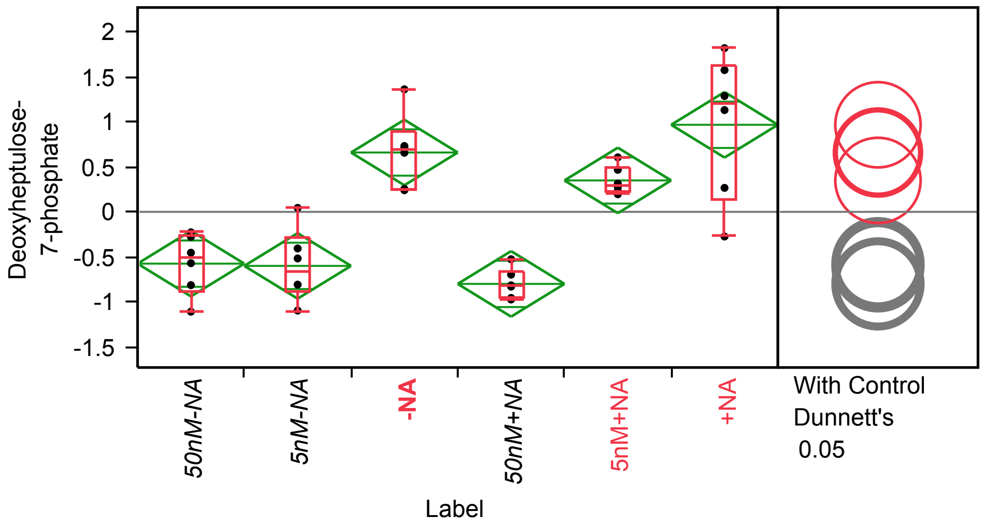


### Quantiles

| Level | Minimum | 10% | 25% | Median | 75% | 90% | Maximum |
|---|---|---|---|---|---|---|---|
| 50nM-NA | -1.10995 | -1.10995 | -0.88868 | -0.51251 | -0.26521 | -0.22767 | -0.22767 |
| 5nM-NA | -1.0974 | -1.0974 | -0.88693 | -0.66352 | -0.29429 | 0.043753 | 0.043753 |
| -NA | 0.240826 | 0.240826 | 0.25146 | 0.68456 | 0.889557 | 1.362124 | 1.362124 |
| 50nM+NA | -0.97426 | -0.97426 | -0.96151 | -0.82431 | -0.65534 | -0.52717 | -0.52717 |
| 5nM+NA | 0.195796 | 0.195796 | 0.225555 | 0.296387 | 0.50199 | 0.605859 | 0.605859 |
| +NA | -0.27377 | -0.27377 | 0.131542 | 1.20966 | 1.637324 | 1.820114 | 1.820114 |

### Oneway Anova

#### Summary of Fit

| | |
|---|---|
| Rsquare | 0.747697 |
| Adj Rsquare | 0.705646 |
| Root Mean Square Error | 0.436932 |
| Mean of Response | -5.6e-10 |
| Observations (or Sum Wgts) | 36 |

#### Analysis of Variance

| Source | DF | Sum of Squares | Mean Square | F Ratio | Prob > F |
|---|---|---|---|---|---|
| Label | 5 | 16.972772 | 3.39455 | 17.7809 | <.0001 * |
| Error | 30 | 5.727297 | 0.19091 | | |
| C. Total | 35 | 22.700069 | | | |

#### Means for Oneway Anova

| Level | Number | Mean | Std Error | Lower 95% | Upper 95% |
|---|---|---|---|---|---|
| 50nM-NA | 6 | -0.57588 | 0.17838 | -0.940 | -0.212 |
| 5nM-NA | 6 | -0.60074 | 0.17838 | -0.965 | -0.236 |
| -NA | 6 | 0.65985 | 0.17838 | 0.296 | 1.024 |
| 50nM+NA | 6 | -0.80090 | 0.17838 | -1.165 | -0.437 |
| 5nM+NA | 6 | 0.34954 | 0.17838 | -0.015 | 0.714 |
| +NA | 6 | 0.96812 | 0.17838 | 0.604 | 1.332 |

Std Error uses a pooled estimate of error variance

### Means Comparisons

#### Comparisons with a control using Dunnett's Method

## Oneway Analysis of deoxyinosine By Label

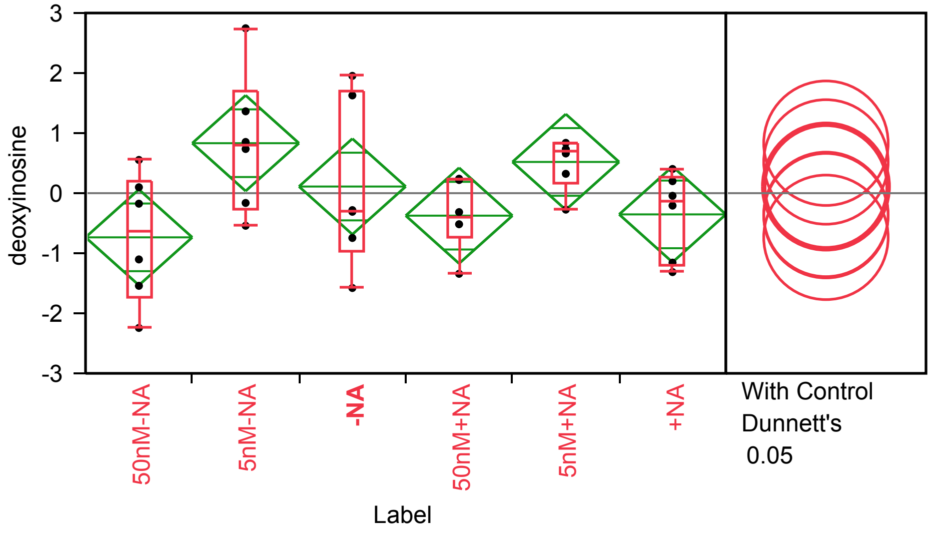


## Quantiles

| Level | Minimum | 10% | 25% | Median | 75% | 90% | Maximum |
|---|---|---|---|---|---|---|---|
| 50nM-NA | -2.24372 | -2.24372 | -1.71772 | -0.63899 | 0.212386 | 0.55319 | 0.55319 |
| 5nM-NA | -0.54212 | -0.54212 | -0.25767 | 0.794415 | 1.70885 | 2.746039 | 2.746039 |
| -NA | -1.5787 | -1.5787 | -0.95402 | -0.29608 | 1.709311 | 1.952691 | 1.952691 |
| 50nM+NA | -1.34218 | -1.34218 | -0.73388 | -0.41636 | 0.224969 | 0.240293 | 0.240293 |
| 5nM+NA | -0.27349 | -0.27349 | 0.173412 | 0.697726 | 0.837747 | 0.837755 | 0.837755 |
| +NA | -1.31382 | -1.31382 | -1.19609 | -0.12551 | 0.250591 | 0.402694 | 0.402694 |

## Oneway Anova

### Summary of Fit

| | |
|---|---|
| Rsquare | 0.280579 |
| Adj Rsquare | 0.160676 |
| Root Mean Square Error | 0.955602 |
| Mean of Response | -8.3e-10 |
| Observations (or Sum Wgts) | 36 |

### Analysis of Variance

| Source | DF | Sum of Squares | Mean Square | F Ratio | Prob > F |
|---|---|---|---|---|---|
| Label | 5 | 10.684345 | 2.13687 | 2.3400 | 0.0659 |
| Error | 30 | 27.395239 | 0.91317 | | |
| C. Total | 35 | 38.079584 | | | |

### Means for Oneway Anova

| Level | Number | Mean | Std Error | Lower 95% | Upper 95% |
|---|---|---|---|---|---|
| 50nM-NA | 6 | -0.73535 | 0.39012 | -1.532 | 0.0614 |
| 5nM-NA | 6 | 0.83217 | 0.39012 | 0.035 | 1.6289 |
| -NA | 6 | 0.11070 | 0.39012 | -0.686 | 0.9074 |
| 50nM+NA | 6 | -0.37431 | 0.39012 | -1.171 | 0.4224 |
| 5nM+NA | 6 | 0.51997 | 0.39012 | -0.277 | 1.3167 |
| +NA | 6 | -0.35319 | 0.39012 | -1.150 | 0.4436 |

Std Error uses a pooled estimate of error variance

## Means Comparisons

### Comparisons with a control using Dunnett's Method

## Oneway Analysis of deoxyribose-phosphate By Label

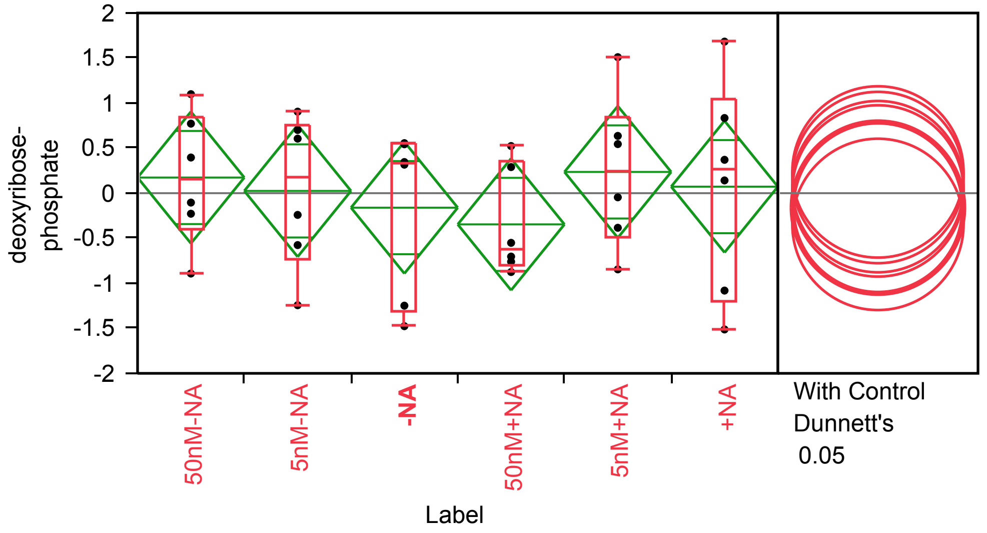


### Quantiles

| Level | Minimum | 10% | 25% | Median | 75% | 90% | Maximum |
|---|---|---|---|---|---|---|---|
| 50nM-NA | -0.89205 | -0.89205 | -0.39408 | 0.145815 | 0.853665 | 1.098976 | 1.098976 |
| 5nM-NA | -1.24351 | -1.24351 | -0.74304 | 0.182169 | 0.752217 | 0.904672 | 0.904672 |
| -NA | -1.47648 | -1.47648 | -1.30566 | 0.331155 | 0.546203 | 0.555417 | 0.555417 |
| 50nM+NA | -0.87525 | -0.87525 | -0.78939 | -0.62765 | 0.348161 | 0.523297 | 0.523297 |
| 5nM+NA | -0.8456 | -0.8456 | -0.49957 | 0.249627 | 0.854214 | 1.509638 | 1.509638 |
| +NA | -1.5127 | -1.5127 | -1.18904 | 0.255952 | 1.046393 | 1.68222 | 1.68222 |

### Oneway Anova

#### Summary of Fit

| | |
|---|---|
| Rsquare | 0.058197 |
| Adj Rsquare | -0.09877 |
| Root Mean Square Error | 0.876697 |
| Mean of Response | 2.78e-10 |
| Observations (or Sum Wgts) | 36 |

#### Analysis of Variance

| Source | DF | Sum of Squares | Mean Square | F Ratio | Prob > F |
|---|---|---|---|---|---|
| Label | 5 | 1.424815 | 0.284963 | 0.3708 | 0.8646 |
| Error | 30 | 23.057928 | 0.768598 | | |
| C. Total | 35 | 24.482743 | | | |

#### Means for Oneway Anova

| Level | Number | Mean | Std Error | Lower 95% | Upper 95% |
|---|---|---|---|---|---|
| 50nM-NA | 6 | 0.17373 | 0.35791 | -0.557 | 0.90468 |
| 5nM-NA | 6 | 0.02511 | 0.35791 | -0.706 | 0.75606 |
| -NA | 6 | -0.16072 | 0.35791 | -0.892 | 0.57023 |
| 50nM+NA | 6 | -0.34637 | 0.35791 | -1.077 | 0.38458 |
| 5nM+NA | 6 | 0.23580 | 0.35791 | -0.495 | 0.96675 |
| +NA | 6 | 0.07245 | 0.35791 | -0.658 | 0.80340 |

Std Error uses a pooled estimate of error variance

### Means Comparisons

#### Comparisons with a control using Dunnett's Method

## Oneway Analysis of D-erythrose-4-phosphate By Label

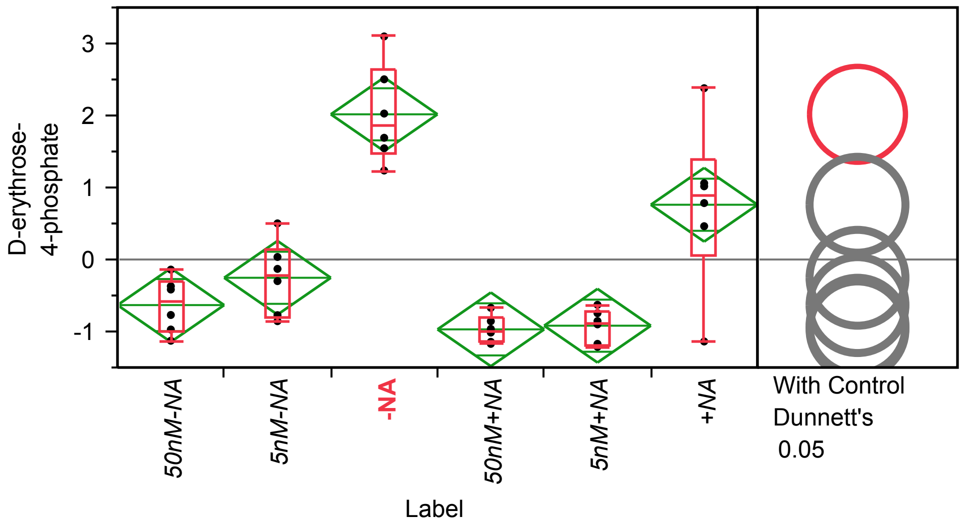


## Quantiles

| Level | Minimum | 10% | 25% | Median | 75% | 90% | Maximum |
|---|---|---|---|---|---|---|---|
| 50nM-NA | -1.1282 | -1.1282 | -1.01166 | -0.59371 | -0.31306 | -0.14052 | -0.14052 |
| 5nM-NA | -0.85632 | -0.85632 | -0.79407 | -0.21515 | 0.151681 | 0.501459 | 0.501459 |
| -NA | 1.235995 | 1.235995 | 1.467615 | 1.858588 | 2.65158 | 3.098131 | 3.098131 |
| 50nM+NA | -1.17135 | -1.17135 | -1.15065 | -0.98949 | -0.8103 | -0.67137 | -0.67137 |
| 5nM+NA | -1.2138 | -1.2138 | -1.18226 | -0.87802 | -0.71673 | -0.62931 | -0.62931 |
| +NA | -1.13857 | -1.13857 | 0.062101 | 0.900166 | 1.389521 | 2.379116 | 2.379116 |

## Oneway Anova

### Summary of Fit

| | |
|---|---|
| Rsquare | 0.785483 |
| Adj Rsquare | 0.74973 |
| Root Mean Square Error | 0.61377 |
| Mean of Response | 5.56e-10 |
| Observations (or Sum Wgts) | 36 |

### Analysis of Variance

| Source | DF | Sum of Squares | Mean Square | F Ratio | Prob > F |
|---|---|---|---|---|---|
| Label | 5 | 41.381630 | 8.27633 | 21.9698 | <.0001 * |
| Error | 30 | 11.301420 | 0.37671 | | |
| C. Total | 35 | 52.683050 | | | |

### Means for Oneway Anova

| Level | Number | Mean | Std Error | Lower 95% | Upper 95% |
|---|---|---|---|---|---|
| 50nM-NA | 6 | -0.6333 | 0.25057 | -1.145 | -0.122 |
| 5nM-NA | 6 | -0.2539 | 0.25057 | -0.766 | 0.258 |
| -NA | 6 | 2.0165 | 0.25057 | 1.505 | 2.528 |
| 50nM+NA | 6 | -0.9703 | 0.25057 | -1.482 | -0.459 |
| 5nM+NA | 6 | -0.9195 | 0.25057 | -1.431 | -0.408 |
| +NA | 6 | 0.7605 | 0.25057 | 0.249 | 1.272 |

Std Error uses a pooled estimate of error variance

## Means Comparisons

### Comparisons with a control using Dunnett's Method

## Oneway Analysis of dGDP By Label

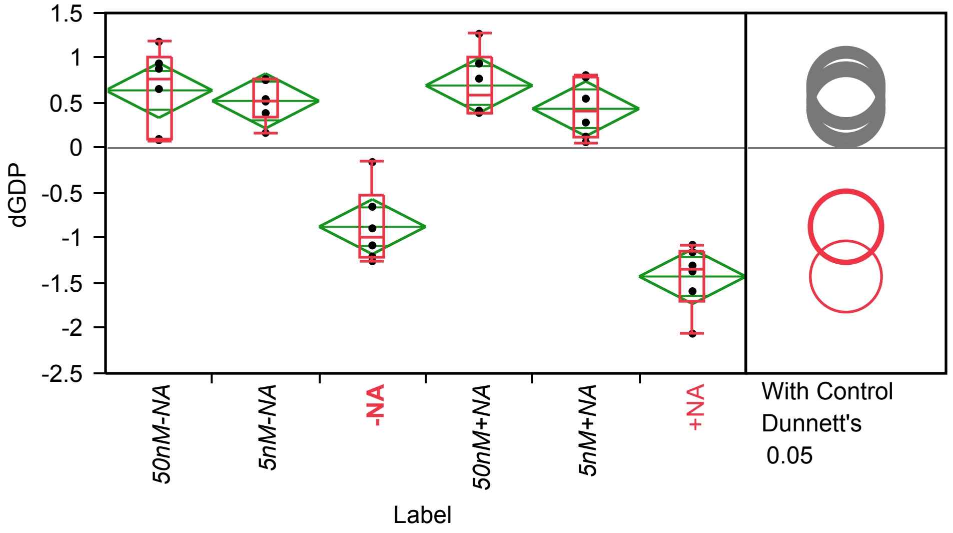


### Quantiles

| Level | Minimum | 10% | 25% | Median | 75% | 90% | Maximum |
|---|---|---|---|---|---|---|---|
| 50nM-NA | 0.088755 | 0.088755 | 0.097955 | 0.768651 | 1.000195 | 1.180446 | 1.180446 |
| 5nM-NA | 0.169532 | 0.169532 | 0.333779 | 0.530678 | 0.758143 | 0.767669 | 0.767669 |
| -NA | -1.25486 | -1.25486 | -1.2173 | -0.98511 | -0.52657 | -0.15514 | -0.15514 |
| 50nM+NA | 0.386117 | 0.386117 | 0.390083 | 0.593485 | 1.021414 | 1.268711 | 1.268711 |
| 5nM+NA | 0.066291 | 0.066291 | 0.112002 | 0.416843 | 0.792208 | 0.809808 | 0.809808 |
| +NA | -2.05872 | -2.05872 | -1.70621 | -1.33645 | -1.13521 | -1.07385 | -1.07385 |

### Oneway Anova

#### Summary of Fit

| | |
|---|---|
| Rsquare | 0.861995 |
| Adj Rsquare | 0.838994 |
| Root Mean Square Error | 0.364623 |
| Mean of Response | -5.6e-10 |
| Observations (or Sum Wgts) | 36 |

#### Analysis of Variance

| Source | DF | Sum of Squares | Mean Square | F Ratio | Prob > F |
|---|---|---|---|---|---|
| Label | 5 | 24.912530 | 4.98251 | 37.4765 | <.0001 * |
| Error | 30 | 3.988501 | 0.13295 | | |
| C. Total | 35 | 28.901031 | | | |

#### Means for Oneway Anova

| Level | Number | Mean | Std Error | Lower 95% | Upper 95% |
|---|---|---|---|---|---|
| 50nM-NA | 6 | 0.6413 | 0.14886 | 0.337 | 0.945 |
| 5nM-NA | 6 | 0.5237 | 0.14886 | 0.220 | 0.828 |
| -NA | 6 | -0.8726 | 0.14886 | -1.177 | -0.569 |
| 50nM+NA | 6 | 0.6954 | 0.14886 | 0.391 | 0.999 |
| 5nM+NA | 6 | 0.4372 | 0.14886 | 0.133 | 0.741 |
| +NA | 6 | -1.4250 | 0.14886 | -1.729 | -1.121 |

Std Error uses a pooled estimate of error variance

### Means Comparisons

#### Comparisons with a control using Dunnett's Method

## Oneway Analysis of D-gluconate By Label

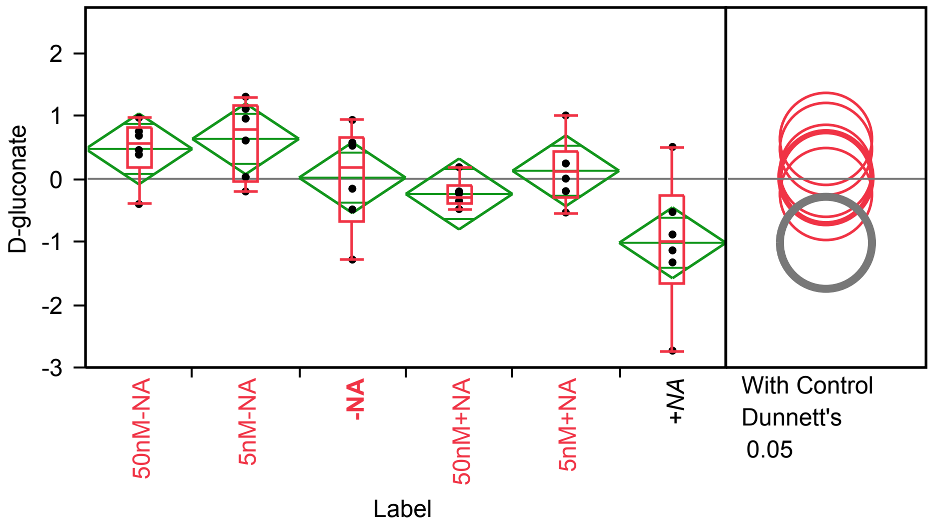


### Quantiles

| Level | Minimum | 10% | 25% | Median | 75% | 90% | Maximum |
|---|---|---|---|---|---|---|---|
| 50nM-NA | -0.4012 | -0.4012 | 0.188891 | 0.572243 | 0.809922 | 0.971483 | 0.971483 |
| 5nM-NA | -0.19993 | -0.19993 | -0.02824 | 0.783601 | 1.160399 | 1.305663 | 1.305663 |
| -NA | -1.28392 | -1.28392 | -0.6863 | 0.184807 | 0.666171 | 0.936187 | 0.936187 |
| 50nM+NA | -0.48089 | -0.48089 | -0.39413 | -0.29315 | -0.1033 | 0.185715 | 0.185715 |
| 5nM+NA | -0.53527 | -0.53527 | -0.28076 | 0.123632 | 0.435295 | 1.00767 | 1.00767 |
| +NA | -2.73212 | -2.73212 | -1.67931 | -1.0112 | -0.26901 | 0.507537 | 0.507537 |

### Oneway Anova

#### Summary of Fit

| | |
|---|---|
| Rsquare | 0.433398 |
| Adj Rsquare | 0.338964 |
| Root Mean Square Error | 0.674551 |
| Mean of Response | 0 |
| Observations (or Sum Wgts) | 36 |

#### Analysis of Variance

| Source | DF | Sum of Squares | Mean Square | F Ratio | Prob > F |
|---|---|---|---|---|---|
| Label | 5 | 10.441417 | 2.08828 | 4.5894 | 0.0032 * |
| Error | 30 | 13.650578 | 0.45502 | | |
| C. Total | 35 | 24.091995 | | | |

#### Means for Oneway Anova

| Level | Number | Mean | Std Error | Lower 95% | Upper 95% |
|---|---|---|---|---|---|
| 50nM-NA | 6 | 0.4761 | 0.27538 | -0.086 | 1.038 |
| 5nM-NA | 6 | 0.6356 | 0.27538 | 0.073 | 1.198 |
| -NA | 6 | 0.0185 | 0.27538 | -0.544 | 0.581 |
| 50nM+NA | 6 | -0.2411 | 0.27538 | -0.803 | 0.321 |
| 5nM+NA | 6 | 0.1280 | 0.27538 | -0.434 | 0.690 |
| +NA | 6 | -1.0172 | 0.27538 | -1.580 | -0.455 |

Std Error uses a pooled estimate of error variance

### Means Comparisons

#### Comparisons with a control using Dunnett's Method

## Oneway Analysis of D-glyceraldehdye-3-phosphate By Label

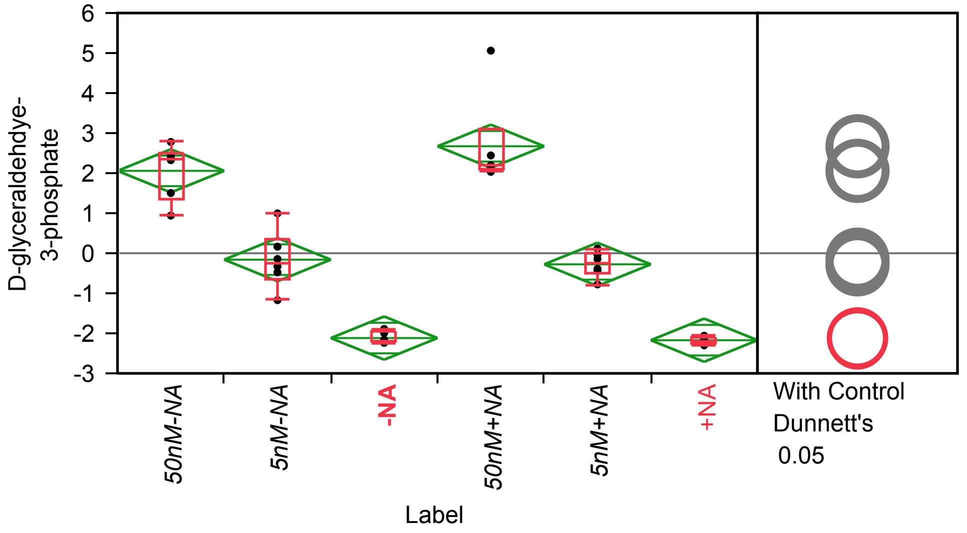


## Quantiles

| Level | Minimum | 10% | 25% | Median | 75% | 90% | Maximum |
|---|---|---|---|---|---|---|---|
| 50nM-NA | 0.940811 | 0.940811 | 1.362641 | 2.348663 | 2.51284 | 2.779431 | 2.779431 |
| 5nM-NA | -1.17182 | -1.17182 | -0.65098 | -0.23659 | 0.371801 | 0.994755 | 0.994755 |
| -NA | -2.23808 | -2.23808 | -2.225 | -2.18478 | -1.96814 | -1.89042 | -1.89042 |
| 50nM+NA | 2.033092 | 2.033092 | 2.096857 | 2.187855 | 3.096649 | 5.060323 | 5.060323 |
| 5nM+NA | -0.77896 | -0.77896 | -0.51949 | -0.26674 | 0.001863 | 0.116704 | 0.116704 |
| +NA | -2.29982 | -2.29982 | -2.23524 | -2.17963 | -2.08959 | -2.06079 | -2.06079 |

## Oneway Anova

### Summary of Fit

| | |
|---|---|
| Rsquare | 0.908004 |
| Adj Rsquare | 0.892672 |
| Root Mean Square Error | 0.647346 |
| Mean of Response | 2.78e-10 |
| Observations (or Sum Wgts) | 36 |

### Analysis of Variance

| Source | DF | Sum of Squares | Mean Square | F Ratio | Prob > F |
|---|---|---|---|---|---|
| Label | 5 | 124.08356 | 24.8167 | 59.2203 | <.0001 * |
| Error | 30 | 12.57172 | 0.4191 | | |
| C. Total | 35 | 136.65528 | | | |

### Means for Oneway Anova

| Level | Number | Mean | Std Error | Lower 95% | Upper 95% |
|---|---|---|---|---|---|
| 50nM-NA | 6 | 2.0575 | 0.26428 | 1.518 | 2.597 |
| 5nM-NA | 6 | -0.1606 | 0.26428 | -0.700 | 0.379 |
| -NA | 6 | -2.1188 | 0.26428 | -2.659 | -1.579 |
| 50nM+NA | 6 | 2.6716 | 0.26428 | 2.132 | 3.211 |
| 5nM+NA | 6 | -0.2775 | 0.26428 | -0.817 | 0.262 |
| +NA | 6 | -2.1721 | 0.26428 | -2.712 | -1.632 |

Std Error uses a pooled estimate of error variance

## Means Comparisons

### Comparisons with a control using Dunnett's Method

## Oneway Analysis of dGTP By Label

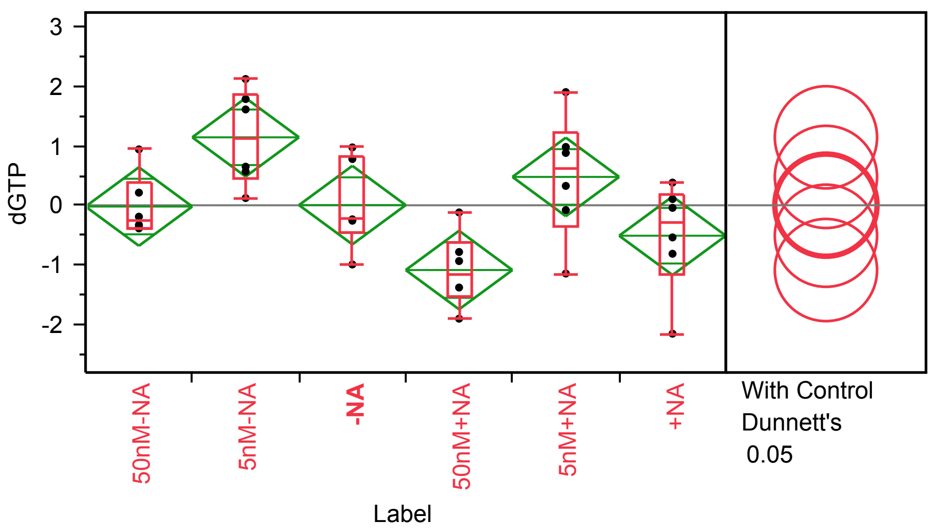


## Quantiles

| Level | Minimum | 10% | 25% | Median | 75% | 90% | Maximum |
|---|---|---|---|---|---|---|---|
| 50nM-NA | -0.38935 | -0.38935 | -0.37483 | -0.26165 | 0.395085 | 0.942199 | 0.942199 |
| 5nM-NA | 0.121861 | 0.121861 | 0.456449 | 1.132992 | 1.875055 | 2.128342 | 2.128342 |
| -NA | -0.99813 | -0.99813 | -0.44489 | -0.23941 | 0.828566 | 0.975298 | 0.975298 |
| 50nM+NA | -1.91208 | -1.91208 | -1.53055 | -1.16371 | -0.621 | -0.12105 | -0.12105 |
| 5nM+NA | -1.15057 | -1.15057 | -0.34863 | 0.604312 | 1.214293 | 1.907911 | 1.907911 |
| +NA | -2.16481 | -2.16481 | -1.15439 | -0.29264 | 0.174208 | 0.381906 | 0.381906 |

## Oneway Anova

### Summary of Fit

| | |
|---|---|
| Rsquare | 0.487299 |
| Adj Rsquare | 0.401848 |
| Root Mean Square Error | 0.794226 |
| Mean of Response | -2.8e-10 |
| Observations (or Sum Wgts) | 36 |

### Analysis of Variance

| Source | DF | Sum of Squares | Mean Square | F Ratio | Prob > F |
|---|---|---|---|---|---|
| Label | 5 | 17.986250 | 3.59725 | 5.7027 | 0.0008 * |
| Error | 30 | 18.923870 | 0.63080 | | |
| C. Total | 35 | 36.910120 | | | |

### Means for Oneway Anova

| Level | Number | Mean | Std Error | Lower 95% | Upper 95% |
|---|---|---|---|---|---|
| 50nM-NA | 6 | -0.0213 | 0.32424 | -0.683 | 0.641 |
| 5nM-NA | 6 | 1.1458 | 0.32424 | 0.484 | 1.808 |
| -NA | 6 | 0.0029 | 0.32424 | -0.659 | 0.665 |
| 50nM+NA | 6 | -1.0919 | 0.32424 | -1.754 | -0.430 |
| 5nM+NA | 6 | 0.4780 | 0.32424 | -0.184 | 1.140 |
| +NA | 6 | -0.5135 | 0.32424 | -1.176 | 0.149 |

Std Error uses a pooled estimate of error variance

## Means Comparisons

### Comparisons with a control using Dunnett's Method

## Oneway Analysis of dihydroxy-acetone-phosphate By Label

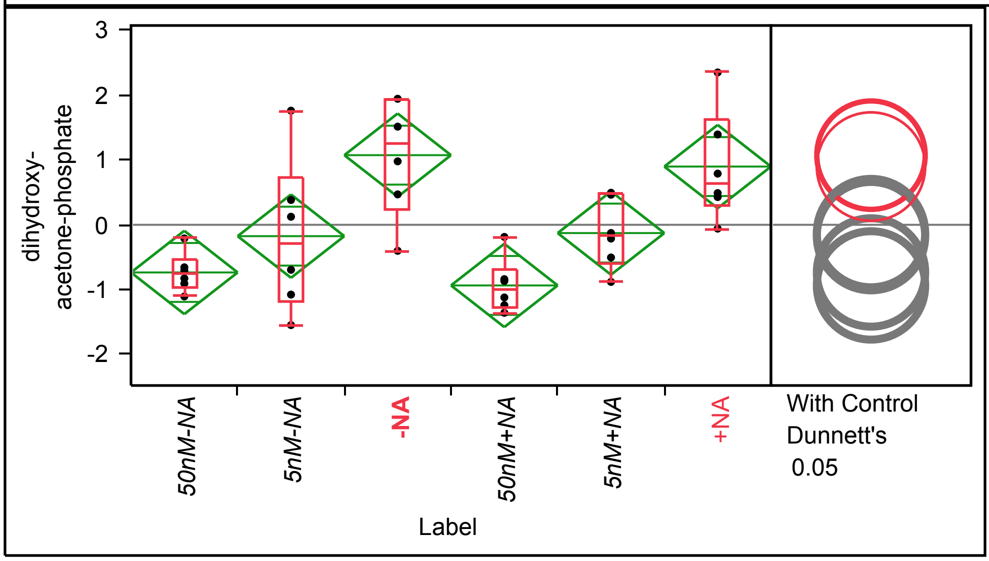


## Quantiles

| Level | Minimum | 10% | 25% | Median | 75% | 90% | Maximum |
|---|---|---|---|---|---|---|---|
| 50nM-NA | -1.10598 | -1.10598 | -0.95604 | -0.76625 | -0.54455 | -0.21006 | -0.21006 |
| 5nM-NA | -1.54722 | -1.54722 | -1.19439 | -0.28511 | 0.727289 | 1.76064 | 1.76064 |
| -NA | -0.40119 | -0.40119 | 0.251516 | 1.246775 | 1.941798 | 1.944512 | 1.944512 |
| 50nM+NA | -1.35776 | -1.35776 | -1.27009 | -0.9941 | -0.67565 | -0.18686 | -0.18686 |
| 5nM+NA | -0.88076 | -0.88076 | -0.59842 | -0.16952 | 0.471642 | 0.494408 | 0.494408 |
| +NA | -0.05883 | -0.05883 | 0.306372 | 0.639444 | 1.633046 | 2.350306 | 2.350306 |

## Oneway Anova

### Summary of Fit

| | |
|---|---|
| Rsquare | 0.535405 |
| Adj Rsquare | 0.457972 |
| Root Mean Square Error | 0.77092 |
| Mean of Response | -2.8e-10 |
| Observations (or Sum Wgts) | 36 |

### Analysis of Variance

| Source | DF | Sum of Squares | Mean Square | F Ratio | Prob > F |
|---|---|---|---|---|---|
| Label | 5 | 20.546938 | 4.10939 | 6.9145 | 0.0002 * |
| Error | 30 | 17.829507 | 0.59432 | | |
| C. Total | 35 | 38.376445 | | | |

### Means for Oneway Anova

| Level | Number | Mean | Std Error | Lower 95% | Upper 95% |
|---|---|---|---|---|---|
| 50nM-NA | 6 | -0.7351 | 0.31473 | -1.378 | -0.092 |
| 5nM-NA | 6 | -0.1751 | 0.31473 | -0.818 | 0.468 |
| -NA | 6 | 1.0745 | 0.31473 | 0.432 | 1.717 |
| 50nM+NA | 6 | -0.9354 | 0.31473 | -1.578 | -0.293 |
| 5nM+NA | 6 | -0.1276 | 0.31473 | -0.770 | 0.515 |
| +NA | 6 | 0.8987 | 0.31473 | 0.256 | 1.541 |

Std Error uses a pooled estimate of error variance

## Means Comparisons

### Comparisons with a control using Dunnett's Method

## Oneway Analysis of dimethylglycine By Label

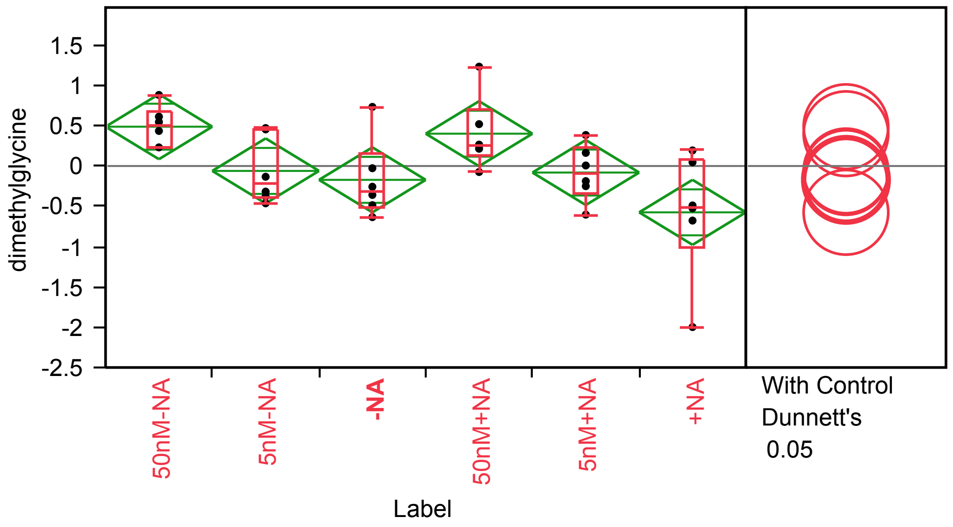


### Quantiles

| Level | Minimum | 10% | 25% | Median | 75% | 90% | Maximum |
|---|---|---|---|---|---|---|---|
| 50nM-NA | 0.21976 | 0.21976 | 0.228211 | 0.490754 | 0.679666 | 0.881557 | 0.881557 |
| 5nM-NA | -0.46277 | -0.46277 | -0.39808 | -0.2256 | 0.458566 | 0.47065 | 0.47065 |
| -NA | -0.63447 | -0.63447 | -0.52319 | -0.30808 | 0.162693 | 0.733288 | 0.733288 |
| 50nM+NA | -0.07434 | -0.07434 | 0.14188 | 0.256931 | 0.697742 | 1.232406 | 1.232406 |
| 5nM+NA | -0.60319 | -0.60319 | -0.33833 | -0.09049 | 0.21831 | 0.384377 | 0.384377 |
| +NA | -1.99817 | -1.99817 | -1.00735 | -0.50655 | 0.083327 | 0.193121 | 0.193121 |

### Oneway Anova

#### Summary of Fit

| | |
|---|---|
| Rsquare | 0.396623 |
| Adj Rsquare | 0.29606 |
| Root Mean Square Error | 0.483623 |
| Mean of Response | 8.33e-11 |
| Observations (or Sum Wgts) | 36 |

#### Analysis of Variance

| Source | DF | Sum of Squares | Mean Square | F Ratio | Prob > F |
|---|---|---|---|---|---|
| Label | 5 | 4.612367 | 0.922473 | 3.9440 | 0.0072 * |
| Error | 30 | 7.016729 | 0.233891 | | |
| C. Total | 35 | 11.629096 | | | |

#### Means for Oneway Anova

| Level | Number | Mean | Std Error | Lower 95% | Upper 95% |
|---|---|---|---|---|---|
| 50nM-NA | 6 | 0.48770 | 0.19744 | 0.0845 | 0.8909 |
| 5nM-NA | 6 | -0.06088 | 0.19744 | -0.4641 | 0.3423 |
| -NA | 6 | -0.17182 | 0.19744 | -0.5750 | 0.2314 |
| 50nM+NA | 6 | 0.40090 | 0.19744 | -0.0023 | 0.8041 |
| 5nM+NA | 6 | -0.08115 | 0.19744 | -0.4844 | 0.3221 |
| +NA | 6 | -0.57475 | 0.19744 | -0.9780 | -0.1715 |

Std Error uses a pooled estimate of error variance

### Means Comparisons

#### Comparisons with a control using Dunnett's Method

## Oneway Analysis of DL-Pipecolic acid By Label

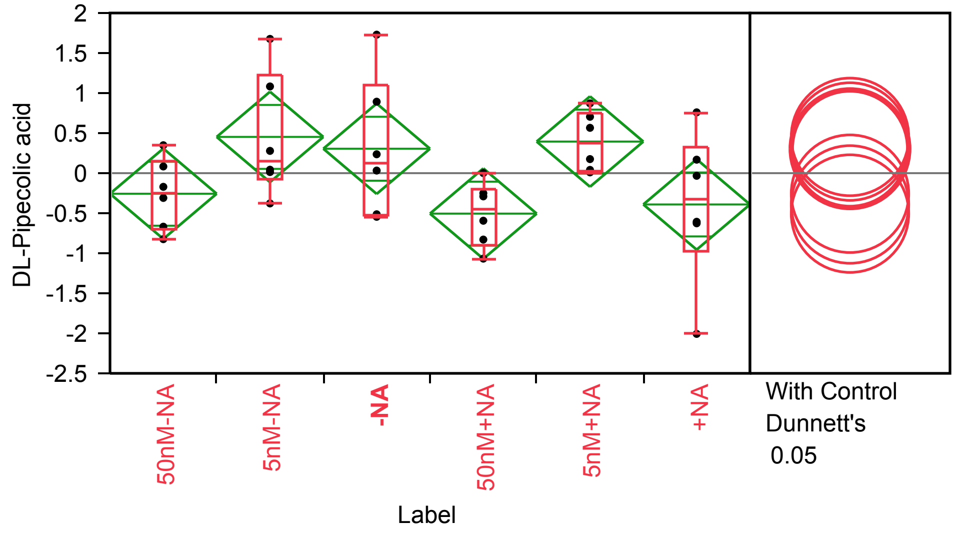


## Quantiles

| Level | Minimum | 10% | 25% | Median | 75% | 90% | Maximum |
|---|---|---|---|---|---|---|---|
| 50nM-NA | -0.824 | -0.824 | -0.70746 | -0.24008 | 0.149892 | 0.34863 | 0.34863 |
| 5nM-NA | -0.37774 | -0.37774 | -0.08397 | 0.160451 | 1.231251 | 1.676498 | 1.676498 |
| -NA | -0.54485 | -0.54485 | -0.52146 | 0.134136 | 1.101557 | 1.729192 | 1.729192 |
| 50nM+NA | -1.06713 | -1.06713 | -0.8889 | -0.44201 | -0.18773 | 0.000941 | 0.000941 |
| 5nM+NA | 0.010117 | 0.010117 | 0.032988 | 0.37091 | 0.74603 | 0.871402 | 0.871402 |
| +NA | -2.00694 | -2.00694 | -0.97139 | -0.31997 | 0.316237 | 0.759495 | 0.759495 |

## Oneway Anova

### Summary of Fit

| | |
|---|---|
| Rsquare | 0.288124 |
| Adj Rsquare | 0.169477 |
| Root Mean Square Error | 0.67706 |
| Mean of Response | -2.8e-11 |
| Observations (or Sum Wgts) | 36 |

### Analysis of Variance

| Source | DF | Sum of Squares | Mean Square | F Ratio | Prob > F |
|---|---|---|---|---|---|
| Label | 5 | 5.566091 | 1.11322 | 2.4284 | 0.0581 |
| Error | 30 | 13.752325 | 0.45841 | | |
| C. Total | 35 | 19.318416 | | | |

### Means for Oneway Anova

| Level | Number | Mean | Std Error | Lower 95% | Upper 95% |
|---|---|---|---|---|---|
| 50nM-NA | 6 | -0.25675 | 0.27641 | -0.821 | 0.3078 |
| 5nM-NA | 6 | 0.45274 | 0.27641 | -0.112 | 1.0172 |
| -NA | 6 | 0.30521 | 0.27641 | -0.259 | 0.8697 |
| 50nM+NA | 6 | -0.50506 | 0.27641 | -1.070 | 0.0594 |
| 5nM+NA | 6 | 0.39470 | 0.27641 | -0.170 | 0.9592 |
| +NA | 6 | -0.39085 | 0.27641 | -0.955 | 0.1737 |

Std Error uses a pooled estimate of error variance

## Means Comparisons

### Comparisons with a control using Dunnett's Method

## Oneway Analysis of D-Mannitol By Label

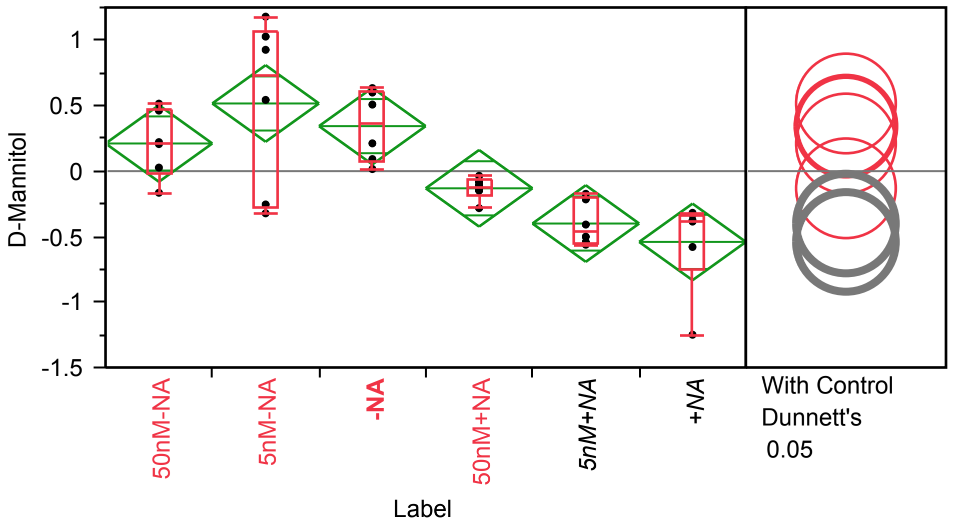


## Quantiles

| Level | Minimum | 10% | 25% | Median | 75% | 90% | Maximum |
|---|---|---|---|---|---|---|---|
| 50nM-NA | -0.16513 | -0.16513 | -0.02111 | 0.214091 | 0.475676 | 0.515389 | 0.515389 |
| 5nM-NA | -0.32218 | -0.32218 | -0.27161 | 0.735415 | 1.065787 | 1.179949 | 1.179949 |
| -NA | 0.015846 | 0.015846 | 0.073012 | 0.360265 | 0.608914 | 0.63426 | 0.63426 |
| 50nM+NA | -0.28265 | -0.28265 | -0.18256 | -0.1188 | -0.0702 | -0.03574 | -0.03574 |
| 5nM+NA | -0.56205 | -0.56205 | -0.54663 | -0.45448 | -0.20507 | -0.17333 | -0.17333 |
| +NA | -1.24965 | -1.24965 | -0.74689 | -0.38053 | -0.33133 | -0.3186 | -0.3186 |

## Oneway Anova

### Summary of Fit

| | |
|---|---|
| Rsquare | 0.594023 |
| Adj Rsquare | 0.52636 |
| Root Mean Square Error | 0.350711 |
| Mean of Response | 8.33e-10 |
| Observations (or Sum Wgts) | 36 |

### Analysis of Variance

| Source | DF | Sum of Squares | Mean Square | F Ratio | Prob > F |
|---|---|---|---|---|---|
| Label | 5 | 5.3991069 | 1.07982 | 8.7792 | <.0001 * |
| Error | 30 | 3.6899429 | 0.12300 | | |
| C. Total | 35 | 9.0890498 | | | |

### Means for Oneway Anova

| Level | Number | Mean | Std Error | Lower 95% | Upper 95% |
|---|---|---|---|---|---|
| 50nM-NA | 6 | 0.21130 | 0.14318 | -0.0811 | 0.5037 |
| 5nM-NA | 6 | 0.51693 | 0.14318 | 0.2245 | 0.8093 |
| -NA | 6 | 0.34386 | 0.14318 | 0.0515 | 0.6363 |
| 50nM+NA | 6 | -0.13114 | 0.14318 | -0.4235 | 0.1613 |
| 5nM+NA | 6 | -0.40025 | 0.14318 | -0.6927 | -0.1078 |
| +NA | 6 | -0.54070 | 0.14318 | -0.8331 | -0.2483 |

Std Error uses a pooled estimate of error variance

## Means Comparisons

### Comparisons with a control using Dunnett's Method

## Oneway Analysis of Sedoheptulose-1-7-bisphosphate By Label

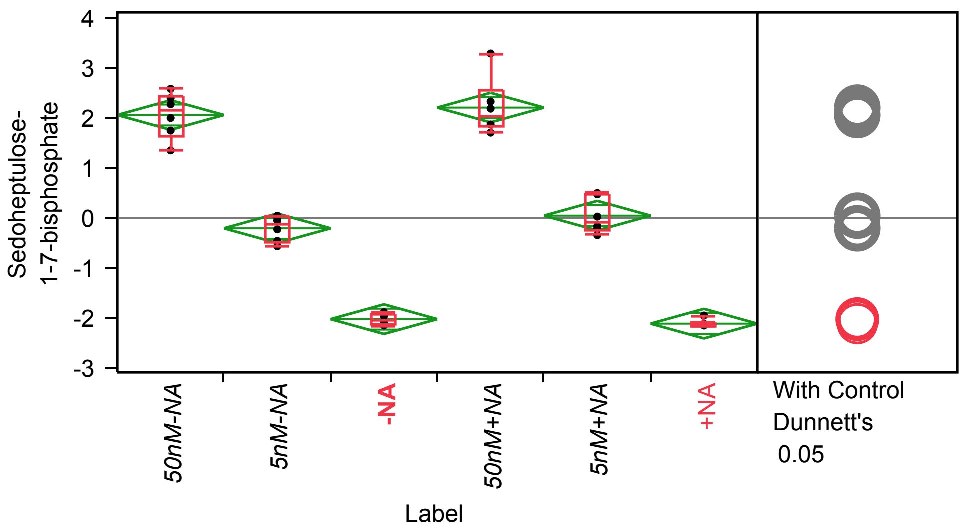


### Quantiles

| Level | Minimum | 10% | 25% | Median | 75% | 90% | Maximum |
|---|---|---|---|---|---|---|---|
| 50nM-NA | 1.35624 | 1.35624 | 1.652999 | 2.141451 | 2.448232 | 2.585122 | 2.585122 |
| 5nM-NA | -0.55963 | -0.55963 | -0.47954 | -0.13338 | 0.027501 | 0.049673 | 0.049673 |
| -NA | -2.14222 | -2.14222 | -2.11876 | -2.02626 | -1.90811 | -1.87901 | -1.87901 |
| 50nM+NA | 1.711324 | 1.711324 | 1.82937 | 2.032054 | 2.571414 | 3.292073 | 3.292073 |
| 5nM+NA | -0.33809 | -0.33809 | -0.2405 | -0.06383 | 0.48433 | 0.503066 | 0.503066 |
| +NA | -2.16324 | -2.16324 | -2.16323 | -2.13043 | -2.06838 | -1.94476 | -1.94476 |

### Oneway Anova

#### Summary of Fit

| | |
|---|---|
| Rsquare | 0.965665 |
| Adj Rsquare | 0.959943 |
| Root Mean Square Error | 0.354747 |
| Mean of Response | -2.8e-10 |
| Observations (or Sum Wgts) | 36 |

#### Analysis of Variance

| Source | DF | Sum of Squares | Mean Square | F Ratio | Prob > F |
|---|---|---|---|---|---|
| Label | 5 | 106.18309 | 21.2366 | 168.7512 | <.0001 * |
| Error | 30 | 3.77537 | 0.1258 | | |
| C. Total | 35 | 109.95846 | | | |

#### Means for Oneway Anova

| Level | Number | Mean | Std Error | Lower 95% | Upper 95% |
|---|---|---|---|---|---|
| 50nM-NA | 6 | 2.0631 | 0.14483 | 1.767 | 2.359 |
| 5nM-NA | 6 | -0.2016 | 0.14483 | -0.497 | 0.094 |
| -NA | 6 | -2.0171 | 0.14483 | -2.313 | -1.721 |
| 50nM+NA | 6 | 2.2112 | 0.14483 | 1.915 | 2.507 |
| 5nM+NA | 6 | 0.0512 | 0.14483 | -0.245 | 0.347 |
| +NA | 6 | -2.1069 | 0.14483 | -2.403 | -1.811 |

Std Error uses a pooled estimate of error variance

### Means Comparisons

#### Comparisons with a control using Dunnett's Method

## Oneway Analysis of FAD By Label

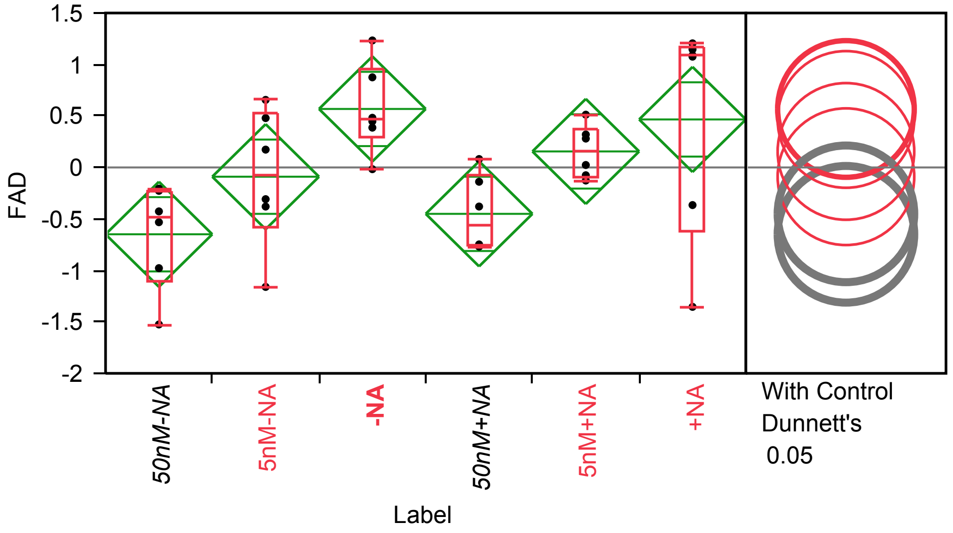


### Quantiles

| Level | Minimum | 10% | 25% | Median | 75% | 90% | Maximum |
|---|---|---|---|---|---|---|---|
| 50nM-NA | -1.52517 | -1.52517 | -1.11496 | -0.47941 | -0.22173 | -0.20625 | -0.20625 |
| 5nM-NA | -1.16044 | -1.16044 | -0.57498 | -0.06752 | 0.523634 | 0.656017 | 0.656017 |
| -NA | -0.01682 | -0.01682 | 0.285811 | 0.464987 | 0.964827 | 1.233442 | 1.233442 |
| 50nM+NA | -0.76902 | -0.76902 | -0.75531 | -0.56368 | -0.08382 | 0.081409 | 0.081409 |
| 5nM+NA | -0.12692 | -0.12692 | -0.08866 | 0.152078 | 0.367934 | 0.512256 | 0.512256 |
| +NA | -1.35419 | -1.35419 | -0.61264 | 1.082648 | 1.161463 | 1.205267 | 1.205267 |

### Oneway Anova

#### Summary of Fit

| | |
|---|---|
| Rsquare | 0.389986 |
| Adj Rsquare | 0.288317 |
| Root Mean Square Error | 0.611975 |
| Mean of Response | 2.47e-17 |
| Observations (or Sum Wgts) | 36 |

#### Analysis of Variance

| Source | DF | Sum of Squares | Mean Square | F Ratio | Prob > F |
|---|---|---|---|---|---|
| Label | 5 | 7.182881 | 1.43658 | 3.8358 | 0.0083 * |
| Error | 30 | 11.235411 | 0.37451 | | |
| C. Total | 35 | 18.418293 | | | |

#### Means for Oneway Anova

| Level | Number | Mean | Std Error | Lower 95% | Upper 95% |
|---|---|---|---|---|---|
| 50nM-NA | 6 | -0.64922 | 0.24984 | -1.159 | -0.139 |
| 5nM-NA | 6 | -0.08997 | 0.24984 | -0.600 | 0.420 |
| -NA | 6 | 0.56810 | 0.24984 | 0.058 | 1.078 |
| 50nM+NA | 6 | -0.45077 | 0.24984 | -0.961 | 0.059 |
| 5nM+NA | 6 | 0.15557 | 0.24984 | -0.355 | 0.666 |
| +NA | 6 | 0.46630 | 0.24984 | -0.044 | 0.977 |

Std Error uses a pooled estimate of error variance

### Means Comparisons

#### Comparisons with a control using Dunnett's Method

## Oneway Analysis of folate By Label

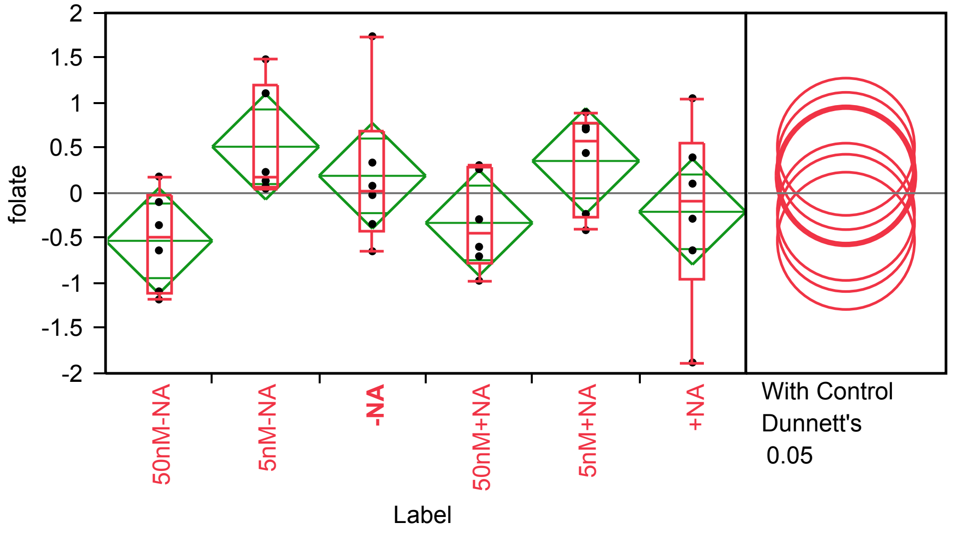


### Quantiles

| Level | Minimum | 10% | 25% | Median | 75% | 90% | Maximum |
|---|---|---|---|---|---|---|---|
| 50nM-NA | -1.17971 | -1.17971 | -1.11262 | -0.49488 | -0.02765 | 0.184424 | 0.184424 |
| 5nM-NA | 0.049748 | 0.049748 | 0.068396 | 0.186619 | 1.203456 | 1.485941 | 1.485941 |
| -NA | -0.64481 | -0.64481 | -0.41791 | 0.030622 | 0.69023 | 1.74183 | 1.74183 |
| 50nM+NA | -0.97168 | -0.97168 | -0.76849 | -0.44247 | 0.278064 | 0.310688 | 0.310688 |
| 5nM+NA | -0.40994 | -0.40994 | -0.27575 | 0.576967 | 0.774992 | 0.898184 | 0.898184 |
| +NA | -1.87953 | -1.87953 | -0.94584 | -0.08873 | 0.56142 | 1.053888 | 1.053888 |

### Oneway Anova

#### Summary of Fit

| | |
|---|---|
| Rsquare | 0.259022 |
| Adj Rsquare | 0.135525 |
| Root Mean Square Error | 0.702267 |
| Mean of Response | -1.2e-17 |
| Observations (or Sum Wgts) | 36 |

#### Analysis of Variance

| Source | DF | Sum of Squares | Mean Square | F Ratio | Prob > F |
|---|---|---|---|---|---|
| Label | 5 | 5.171975 | 1.03439 | 2.0974 | 0.0933 |
| Error | 30 | 14.795361 | 0.49318 | | |
| C. Total | 35 | 19.967336 | | | |

#### Means for Oneway Anova

| Level | Number | Mean | Std Error | Lower 95% | Upper 95% |
|---|---|---|---|---|---|
| 50nM-NA | 6 | -0.52894 | 0.28670 | -1.114 | 0.0566 |
| 5nM-NA | 6 | 0.51547 | 0.28670 | -0.070 | 1.1010 |
| -NA | 6 | 0.19261 | 0.28670 | -0.393 | 0.7781 |
| 50nM+NA | 6 | -0.32992 | 0.28670 | -0.915 | 0.2556 |
| 5nM+NA | 6 | 0.35751 | 0.28670 | -0.228 | 0.9430 |
| +NA | 6 | -0.20674 | 0.28670 | -0.792 | 0.3788 |

Std Error uses a pooled estimate of error variance

### Means Comparisons

#### Comparisons with a control using Dunnett's Method

## Oneway Analysis of fructose-1,6-bisphosphate By Label

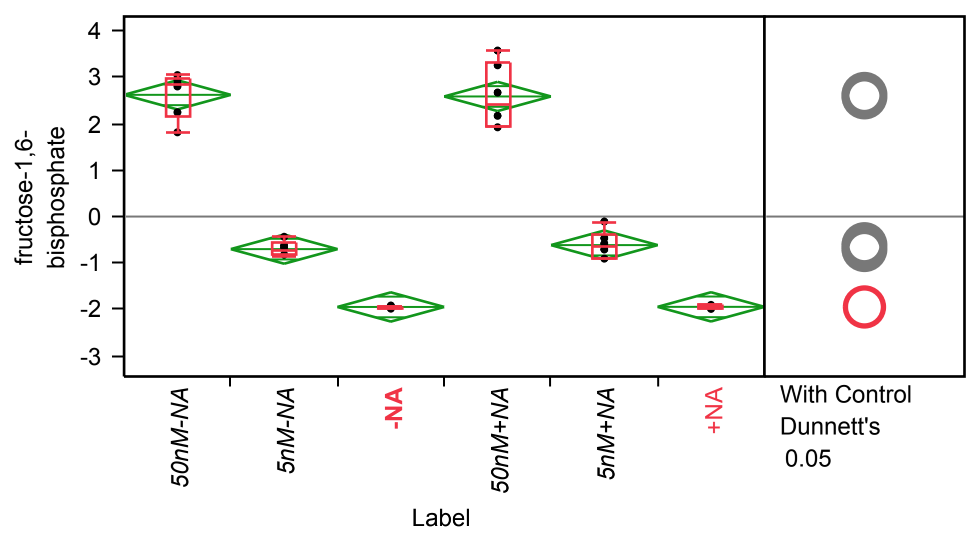


### Quantiles

| Level | Minimum | 10% | 25% | Median | 75% | 90% | Maximum |
|---|---|---|---|---|---|---|---|
| 50nM-NA | 1.815708 | 1.815708 | 2.138996 | 2.838381 | 2.97503 | 3.04779 | 3.04779 |
| 5nM-NA | -0.84018 | -0.84018 | -0.82789 | -0.74361 | -0.57611 | -0.43621 | -0.43621 |
| -NA | -1.98441 | -1.98441 | -1.96101 | -1.94617 | -1.93544 | -1.91772 | -1.91772 |
| 50nM+NA | 1.922608 | 1.922608 | 1.923234 | 2.422012 | 3.339519 | 3.576942 | 3.576942 |
| 5nM+NA | -0.91928 | -0.91928 | -0.90987 | -0.64571 | -0.37616 | -0.10794 | -0.10794 |
| +NA | -1.98973 | -1.98973 | -1.9781 | -1.94882 | -1.90814 | -1.90398 | -1.90398 |

### Oneway Anova

#### Summary of Fit

| | |
|---|---|
| Rsquare | 0.968682 |
| Adj Rsquare | 0.963462 |
| Root Mean Square Error | 0.37742 |
| Mean of Response | 5.556e-9 |
| Observations (or Sum Wgts) | 36 |

#### Analysis of Variance

| Source | DF | Sum of Squares | Mean Square | F Ratio | Prob > F |
|---|---|---|---|---|---|
| Label | 5 | 132.17793 | 26.4356 | 185.5838 | <.0001 * |
| Error | 30 | 4.27337 | 0.1424 | | |
| C. Total | 35 | 136.45130 | | | |

#### Means for Oneway Anova

| Level | Number | Mean | Std Error | Lower 95% | Upper 95% |
|---|---|---|---|---|---|
| 50nM-NA | 6 | 2.6230 | 0.15408 | 2.308 | 2.938 |
| 5nM-NA | 6 | -0.7017 | 0.15408 | -1.016 | -0.387 |
| -NA | 6 | -1.9482 | 0.15408 | -2.263 | -1.633 |
| 50nM+NA | 6 | 2.5879 | 0.15408 | 2.273 | 2.903 |
| 5nM+NA | 6 | -0.6152 | 0.15408 | -0.930 | -0.300 |
| +NA | 6 | -1.9459 | 0.15408 | -2.261 | -1.631 |

Std Error uses a pooled estimate of error variance

### Means Comparisons

#### Comparisons with a control using Dunnett's Method

## Oneway Analysis of fructose-6-phosphate By Label

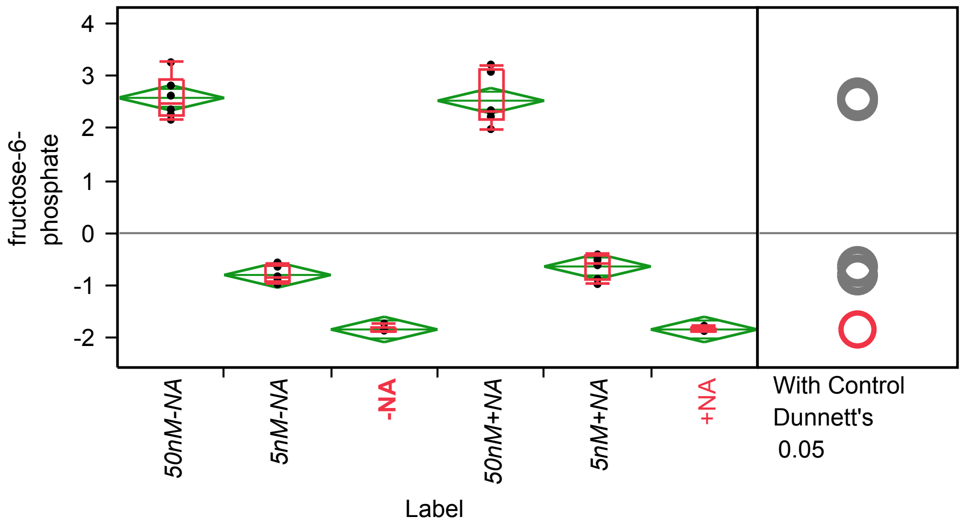


### Quantiles

| Level | Minimum | 10% | 25% | Median | 75% | 90% | Maximum |
|---|---|---|---|---|---|---|---|
| 50nM-NA | 2.161456 | 2.161456 | 2.234418 | 2.48937 | 2.923656 | 3.256588 | 3.256588 |
| 5nM-NA | -0.97397 | -0.97397 | -0.93481 | -0.84297 | -0.61878 | -0.5594 | -0.5594 |
| -NA | -1.87167 | -1.87167 | -1.87163 | -1.86426 | -1.79384 | -1.72155 | -1.72155 |
| 50nM+NA | 1.982973 | 1.982973 | 2.162828 | 2.326146 | 3.109268 | 3.212324 | 3.212324 |
| 5nM+NA | -0.96968 | -0.96968 | -0.89642 | -0.55999 | -0.43378 | -0.405 | -0.405 |
| +NA | -1.87155 | -1.87155 | -1.87101 | -1.84975 | -1.7961 | -1.76374 | -1.76374 |

### Oneway Anova

#### Summary of Fit

| | |
|---|---|
| Rsquare | 0.980236 |
| Adj Rsquare | 0.976942 |
| Root Mean Square Error | 0.289562 |
| Mean of Response | 9.87e-17 |
| Observations (or Sum Wgts) | 36 |

#### Analysis of Variance

| Source | DF | Sum of Squares | Mean Square | F Ratio | Prob > F |
|---|---|---|---|---|---|
| Label | 5 | 124.75501 | 24.9510 | 297.5802 | <.0001 * |
| Error | 30 | 2.51539 | 0.0838 | | |
| C. Total | 35 | 127.27040 | | | |

#### Means for Oneway Anova

| Level | Number | Mean | Std Error | Lower 95% | Upper 95% |
|---|---|---|---|---|---|
| 50nM-NA | 6 | 2.5780 | 0.11821 | 2.337 | 2.819 |
| 5nM-NA | 6 | -0.7966 | 0.11821 | -1.038 | -0.555 |
| -NA | 6 | -1.8352 | 0.11821 | -2.077 | -1.594 |
| 50nM+NA | 6 | 2.5242 | 0.11821 | 2.283 | 2.766 |
| 5nM+NA | 6 | -0.6350 | 0.11821 | -0.876 | -0.394 |
| +NA | 6 | -1.8354 | 0.11821 | -2.077 | -1.594 |

Std Error uses a pooled estimate of error variance

### Means Comparisons

#### Comparisons with a control using Dunnett's Method

## Oneway Analysis of fumarate By Label

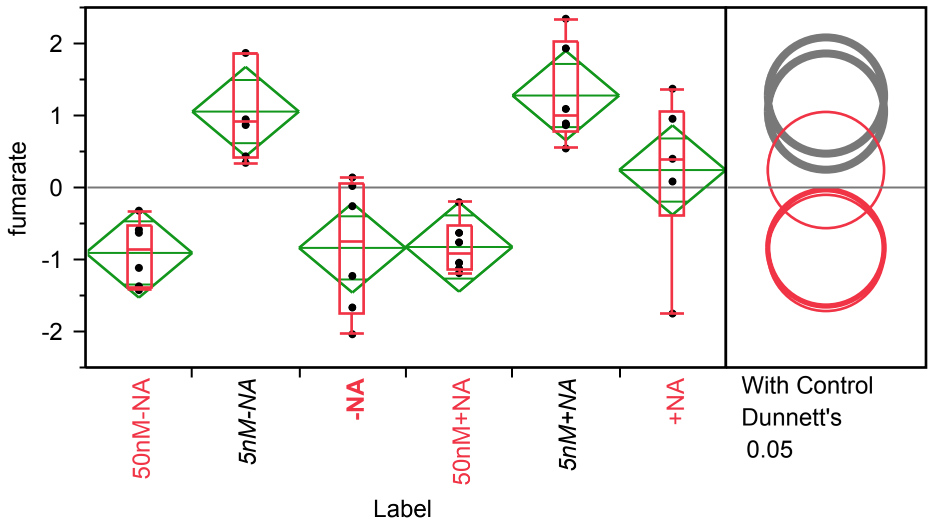


### Quantiles

| Level | Minimum | 10% | 25% | Median | 75% | 90% | Maximum |
|---|---|---|---|---|---|---|---|
| 50nM-NA | -1.42502 | -1.42502 | -1.3857 | -0.87287 | -0.52057 | -0.32228 | -0.32228 |
| 5nM-NA | 0.342189 | 0.342189 | 0.409723 | 0.907196 | 1.868651 | 1.868702 | 1.868702 |
| -NA | -2.03832 | -2.03832 | -1.75967 | -0.74407 | 0.050336 | 0.139156 | 0.139156 |
| 50nM+NA | -1.18908 | -1.18908 | -1.14217 | -0.9038 | -0.52398 | -0.20416 | -0.20416 |
| 5nM+NA | 0.542694 | 0.542694 | 0.785049 | 0.989884 | 2.034817 | 2.343568 | 2.343568 |
| +NA | -1.75068 | -1.75068 | -0.37518 | 0.397556 | 1.058552 | 1.371861 | 1.371861 |

### Oneway Anova

#### Summary of Fit

| | |
|---|---|
| Rsquare | 0.644253 |
| Adj Rsquare | 0.584962 |
| Root Mean Square Error | 0.744144 |
| Mean of Response | 1.23e-17 |
| Observations (or Sum Wgts) | 36 |

#### Analysis of Variance

| Source | DF | Sum of Squares | Mean Square | F Ratio | Prob > F |
|---|---|---|---|---|---|
| Label | 5 | 30.085104 | 6.01702 | 10.8659 | <.0001 * |
| Error | 30 | 16.612525 | 0.55375 | | |
| C. Total | 35 | 46.697629 | | | |

#### Means for Oneway Anova

| Level | Number | Mean | Std Error | Lower 95% | Upper 95% |
|---|---|---|---|---|---|
| 50nM-NA | 6 | -0.9087 | 0.30380 | -1.529 | -0.288 |
| 5nM-NA | 6 | 1.0544 | 0.30380 | 0.434 | 1.675 |
| -NA | 6 | -0.8389 | 0.30380 | -1.459 | -0.218 |
| 50nM+NA | 6 | -0.8263 | 0.30380 | -1.447 | -0.206 |
| 5nM+NA | 6 | 1.2773 | 0.30380 | 0.657 | 1.898 |
| +NA | 6 | 0.2423 | 0.30380 | -0.378 | 0.863 |

Std Error uses a pooled estimate of error variance

### Means Comparisons

#### Comparisons with a control using Dunnett's Method

## Oneway Analysis of Geranyl-PP By Label

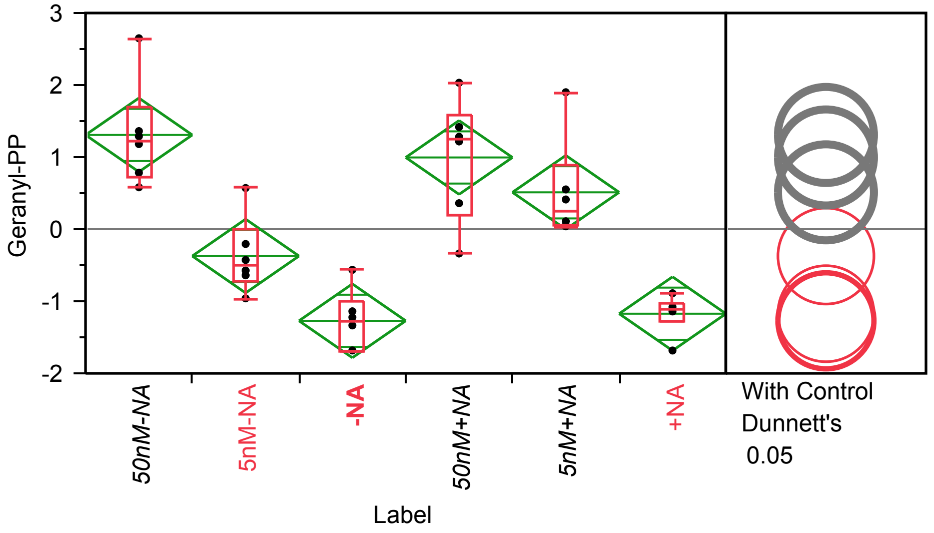


### Quantiles

| Level | Minimum | 10% | 25% | Median | 75% | 90% | Maximum |
|---|---|---|---|---|---|---|---|
| 50nM-NA | 0.581891 | 0.581891 | 0.733458 | 1.235544 | 1.683385 | 2.649513 | 2.649513 |
| 5nM-NA | -0.95924 | -0.95924 | -0.71982 | -0.50014 | -0.01088 | 0.571684 | 0.571684 |
| -NA | -1.68251 | -1.68251 | -1.68243 | -1.27877 | -0.99527 | -0.56391 | -0.56391 |
| 50nM+NA | -0.33705 | -0.33705 | 0.186203 | 1.25028 | 1.571487 | 2.032188 | 2.032188 |
| 5nM+NA | 0.040395 | 0.040395 | 0.05188 | 0.261174 | 0.889233 | 1.900668 | 1.900668 |
| +NA | -1.68314 | -1.68314 | -1.28644 | -1.11575 | -1.03026 | -0.88857 | -0.88857 |

### Oneway Anova

#### Summary of Fit

| | |
|---|---|
| Rsquare | 0.763616 |
| Adj Rsquare | 0.724219 |
| Root Mean Square Error | 0.614171 |
| Mean of Response | 5.56e-10 |
| Observations (or Sum Wgts) | 36 |

#### Analysis of Variance

| Source | DF | Sum of Squares | Mean Square | F Ratio | Prob > F |
|---|---|---|---|---|---|
| Label | 5 | 36.555843 | 7.31117 | 19.3824 | <.0001 * |
| Error | 30 | 11.316168 | 0.37721 | | |
| C. Total | 35 | 47.872012 | | | |

#### Means for Oneway Anova

| Level | Number | Mean | Std Error | Lower 95% | Upper 95% |
|---|---|---|---|---|---|
| 50nM-NA | 6 | 1.3080 | 0.25073 | 0.796 | 1.820 |
| 5nM-NA | 6 | -0.3722 | 0.25073 | -0.884 | 0.140 |
| -NA | 6 | -1.2709 | 0.25073 | -1.783 | -0.759 |
| 50nM+NA | 6 | 0.9957 | 0.25073 | 0.484 | 1.508 |
| 5nM+NA | 6 | 0.5119 | 0.25073 | -0.0002 | 1.024 |
| +NA | 6 | -1.1725 | 0.25073 | -1.685 | -0.660 |

Std Error uses a pooled estimate of error variance

### Means Comparisons

#### Comparisons with a control using Dunnett's Method

## Oneway Analysis of Glucaric acid By Label

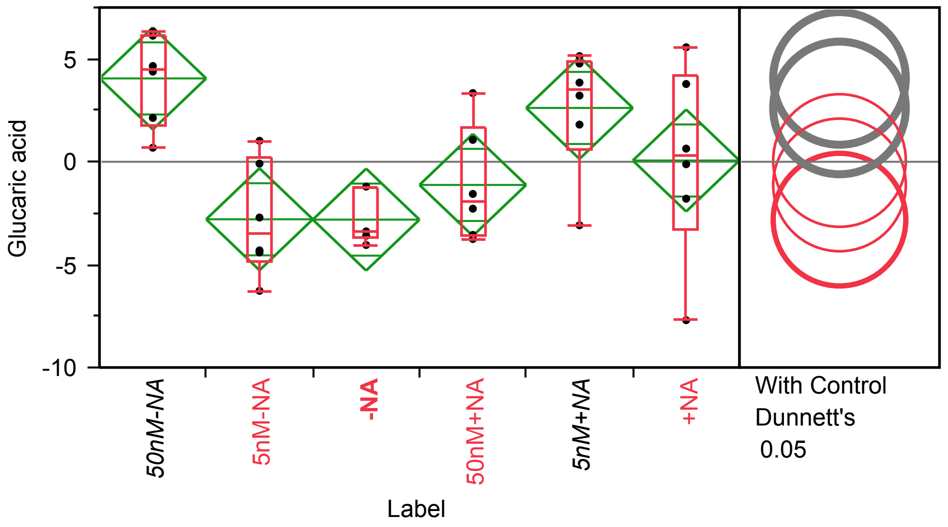


## Quantiles

| Level | Minimum | 10% | 25% | Median | 75% | 90% | Maximum |
|---|---|---|---|---|---|---|---|
| 50nM-NA | 0.684608 | 0.684608 | 1.772114 | 4.52028 | 6.177898 | 6.322774 | 6.322774 |
| 5nM-NA | -6.27366 | -6.27366 | -4.87422 | -3.50935 | 0.188373 | 1.019477 | 1.019477 |
| -NA | -4.0433 | -4.0433 | -3.70644 | -3.39257 | -1.23062 | -1.20731 | -1.20731 |
| 50nM+NA | -3.75367 | -3.75367 | -3.60546 | -1.92176 | 1.633933 | 3.338858 | 3.338858 |
| 5nM+NA | -3.08468 | -3.08468 | 0.584452 | 3.533454 | 4.866845 | 5.13664 | 5.13664 |
| +NA | -7.6995 | -7.6995 | -3.27201 | 0.259938 | 4.226762 | 5.566565 | 5.566565 |

## Oneway Anova

### Summary of Fit

| | |
|---|---|
| Rsquare | 0.476817 |
| Adj Rsquare | 0.389619 |
| Root Mean Square Error | 2.972112 |
| Mean of Response | 2.78e-10 |
| Observations (or Sum Wgts) | 36 |

### Analysis of Variance

| Source | DF | Sum of Squares | Mean Square | F Ratio | Prob > F |
|---|---|---|---|---|---|
| Label | 5 | 241.51767 | 48.3035 | 5.4683 | 0.0011 * |
| Error | 30 | 265.00343 | 8.8334 | | |
| C. Total | 35 | 506.52110 | | | |

### Means for Oneway Anova

| Level | Number | Mean | Std Error | Lower 95% | Upper 95% |
|---|---|---|---|---|---|
| 50nM-NA | 6 | 4.0520 | 1.2134 | 1.574 | 6.530 |
| 5nM-NA | 6 | -2.7949 | 1.2134 | -5.273 | -0.317 |
| -NA | 6 | -2.8114 | 1.2134 | -5.289 | -0.333 |
| 50nM+NA | 6 | -1.1248 | 1.2134 | -3.603 | 1.353 |
| 5nM+NA | 6 | 2.6172 | 1.2134 | 0.139 | 5.095 |
| +NA | 6 | 0.0618 | 1.2134 | -2.416 | 2.540 |

Std Error uses a pooled estimate of error variance

## Means Comparisons

### Comparisons with a control using Dunnett's Method

## Oneway Analysis of glucono-D-lactone By Label

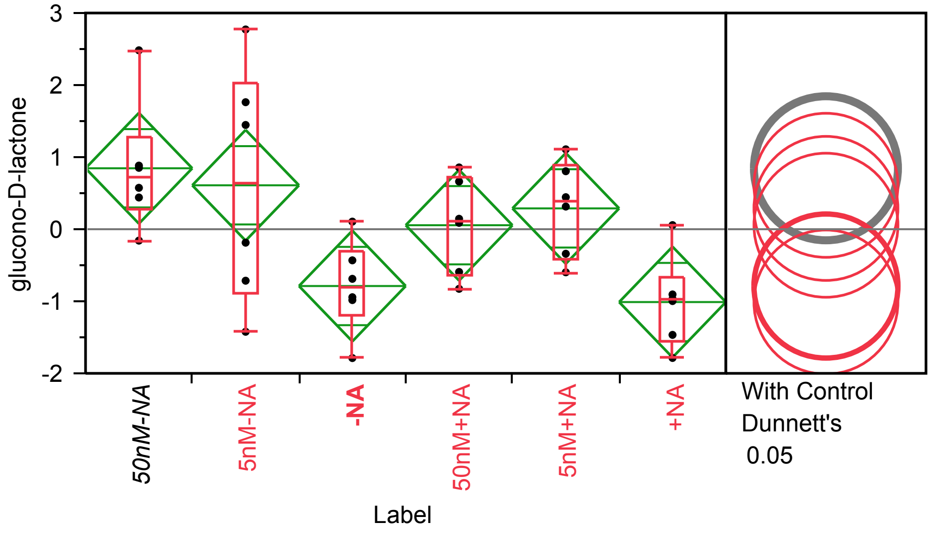


### Quantiles

| Level | Minimum | 10% | 25% | Median | 75% | 90% | Maximum |
|---|---|---|---|---|---|---|---|
| 50nM-NA | -0.15804 | -0.15804 | 0.290652 | 0.713142 | 1.282506 | 2.482587 | 2.482587 |
| 5nM-NA | -1.42288 | -1.42288 | -0.89161 | 0.629809 | 2.014644 | 2.771418 | 2.771418 |
| -NA | -1.78786 | -1.78786 | -1.18435 | -0.81613 | -0.29755 | 0.105095 | 0.105095 |
| 50nM+NA | -0.82679 | -0.82679 | -0.65154 | 0.116524 | 0.710656 | 0.85696 | 0.85696 |
| 5nM+NA | -0.59764 | -0.59764 | -0.40488 | 0.379353 | 0.88059 | 1.108281 | 1.108281 |
| +NA | -1.78639 | -1.78639 | -1.54628 | -0.98185 | -0.66481 | 0.056678 | 0.056678 |

### Oneway Anova

#### Summary of Fit

| | |
|---|---|
| Rsquare | 0.398831 |
| Adj Rsquare | 0.298636 |
| Root Mean Square Error | 0.921383 |
| Mean of Response | -5.6e-10 |
| Observations (or Sum Wgts) | 36 |

#### Analysis of Variance

| Source | DF | Sum of Squares | Mean Square | F Ratio | Prob > F |
|---|---|---|---|---|---|
| Label | 5 | 16.896426 | 3.37929 | 3.9806 | 0.0069 * |
| Error | 30 | 25.468424 | 0.84895 | | |
| C. Total | 35 | 42.364850 | | | |

#### Means for Oneway Anova

| Level | Number | Mean | Std Error | Lower 95% | Upper 95% |
|---|---|---|---|---|---|
| 50nM-NA | 6 | 0.8456 | 0.37615 | 0.077 | 1.614 |
| 5nM-NA | 6 | 0.6093 | 0.37615 | -0.159 | 1.378 |
| -NA | 6 | -0.7883 | 0.37615 | -1.557 | -0.020 |
| 50nM+NA | 6 | 0.0553 | 0.37615 | -0.713 | 0.824 |
| 5nM+NA | 6 | 0.2889 | 0.37615 | -0.479 | 1.057 |
| +NA | 6 | -1.0108 | 0.37615 | -1.779 | -0.243 |

Std Error uses a pooled estimate of error variance

### Means Comparisons

#### Comparisons with a control using Dunnett's Method

## Oneway Analysis of Glucose By Label

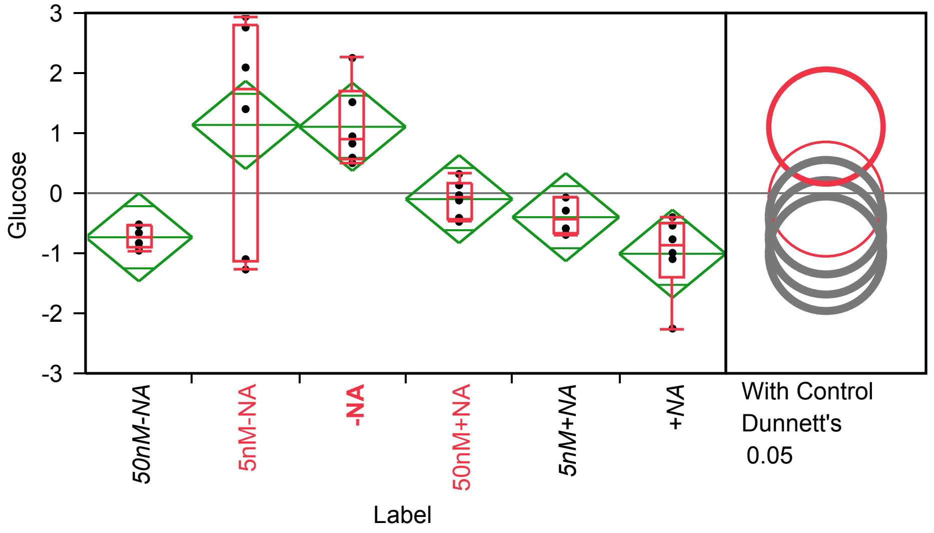


## Quantiles

| Level | Minimum | 10% | 25% | Median | 75% | 90% | Maximum |
|---|---|---|---|---|---|---|---|
| 50nM-NA | -0.95643 | -0.95643 | -0.91506 | -0.74549 | -0.53338 | -0.51937 | -0.51937 |
| 5nM-NA | -1.27143 | -1.27143 | -1.14215 | 1.746402 | 2.803557 | 2.936364 | 2.936364 |
| -NA | 0.500336 | 0.500336 | 0.568221 | 0.88643 | 1.69954 | 2.251277 | 2.251277 |
| 50nM+NA | -0.47607 | -0.47607 | -0.42808 | -0.07782 | 0.178886 | 0.318574 | 0.318574 |
| 5nM+NA | -0.69512 | -0.69512 | -0.68137 | -0.43873 | -0.07379 | -0.07324 | -0.07324 |
| +NA | -2.2544 | -2.2544 | -1.3861 | -0.88135 | -0.50469 | -0.39998 | -0.39998 |

## Oneway Anova

### Summary of Fit

| | |
|---|---|
| Rsquare | 0.523529 |
| Adj Rsquare | 0.444117 |
| Root Mean Square Error | 0.878411 |
| Mean of Response | 4.93e-17 |
| Observations (or Sum Wgts) | 36 |

### Analysis of Variance

| Source | DF | Sum of Squares | Mean Square | F Ratio | Prob > F |
|---|---|---|---|---|---|
| Label | 5 | 25.434322 | 5.08686 | 6.5926 | 0.0003 * |
| Error | 30 | 23.148175 | 0.77161 | | |
| C. Total | 35 | 48.582497 | | | |

### Means for Oneway Anova

| Level | Number | Mean | Std Error | Lower 95% | Upper 95% |
|---|---|---|---|---|---|
| 50nM-NA | 6 | -0.7343 | 0.35861 | -1.467 | -0.002 |
| 5nM-NA | 6 | 1.1363 | 0.35861 | 0.404 | 1.869 |
| -NA | 6 | 1.1052 | 0.35861 | 0.373 | 1.838 |
| 50nM+NA | 6 | -0.0988 | 0.35861 | -0.831 | 0.634 |
| 5nM+NA | 6 | -0.3994 | 0.35861 | -1.132 | 0.333 |
| +NA | 6 | -1.0089 | 0.35861 | -1.741 | -0.277 |

Std Error uses a pooled estimate of error variance

## Means Comparisons

### Comparisons with a control using Dunnett's Method

## Oneway Analysis of glucose-6-phosphate By Label

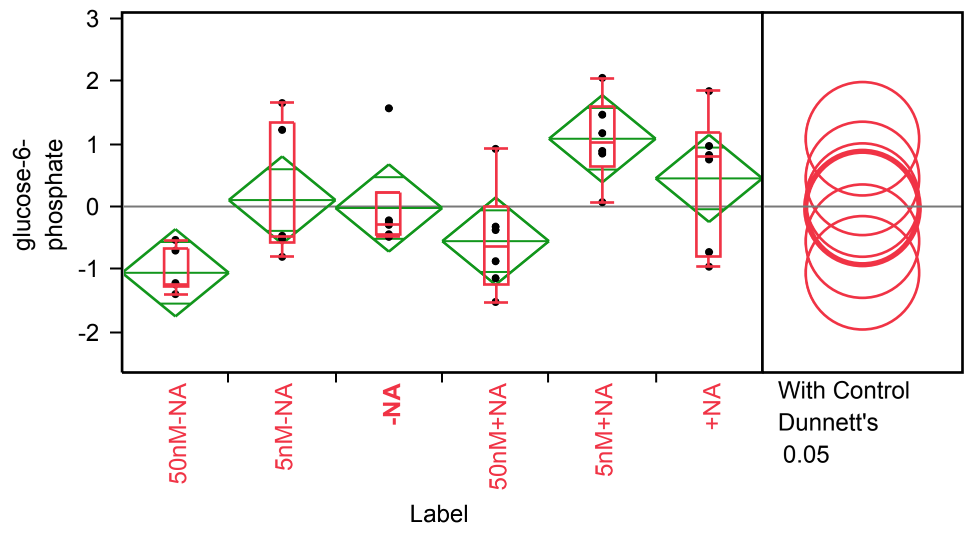


## Quantiles

| Level | Minimum | 10% | 25% | Median | 75% | 90% | Maximum |
|---|---|---|---|---|---|---|---|
| 50nM-NA | -1.39951 | -1.39951 | -1.2847 | -1.23451 | -0.66118 | -0.53382 | -0.53382 |
| 5nM-NA | -0.80385 | -0.80385 | -0.58618 | -0.47012 | 1.331548 | 1.653114 | 1.653114 |
| -NA | -0.48472 | -0.48472 | -0.45388 | -0.28061 | 0.226439 | 1.56948 | 1.56948 |
| 50nM+NA | -1.52494 | -1.52494 | -1.2398 | -0.62466 | -0.01101 | 0.920878 | 0.920878 |
| 5nM+NA | 0.069722 | 0.069722 | 0.652097 | 1.029735 | 1.613948 | 2.054974 | 2.054974 |
| +NA | -0.96128 | -0.96128 | -0.78585 | 0.787382 | 1.186046 | 1.843356 | 1.843356 |

## Oneway Anova

### Summary of Fit

| | |
|---|---|
| Rsquare | 0.446446 |
| Adj Rsquare | 0.354187 |
| Root Mean Square Error | 0.835194 |
| Mean of Response | 2.78e-10 |
| Observations (or Sum Wgts) | 36 |

### Analysis of Variance

| Source | DF | Sum of Squares | Mean Square | F Ratio | Prob > F |
|---|---|---|---|---|---|
| Label | 5 | 16.877344 | 3.37547 | 4.8390 | 0.0023 * |
| Error | 30 | 20.926455 | 0.69755 | | |
| C. Total | 35 | 37.803799 | | | |

### Means for Oneway Anova

| Level | Number | Mean | Std Error | Lower 95% | Upper 95% |
|---|---|---|---|---|---|
| 50nM-NA | 6 | -1.0587 | 0.34097 | -1.755 | -0.362 |
| 5nM-NA | 6 | 0.1033 | 0.34097 | -0.593 | 0.800 |
| -NA | 6 | -0.0235 | 0.34097 | -0.720 | 0.673 |
| 50nM+NA | 6 | -0.5533 | 0.34097 | -1.250 | 0.143 |
| 5nM+NA | 6 | 1.0829 | 0.34097 | 0.387 | 1.779 |
| +NA | 6 | 0.4494 | 0.34097 | -0.247 | 1.146 |

Std Error uses a pooled estimate of error variance

## Means Comparisons

### Comparisons with a control using Dunnett's Method

## Oneway Analysis of glutamate By Label

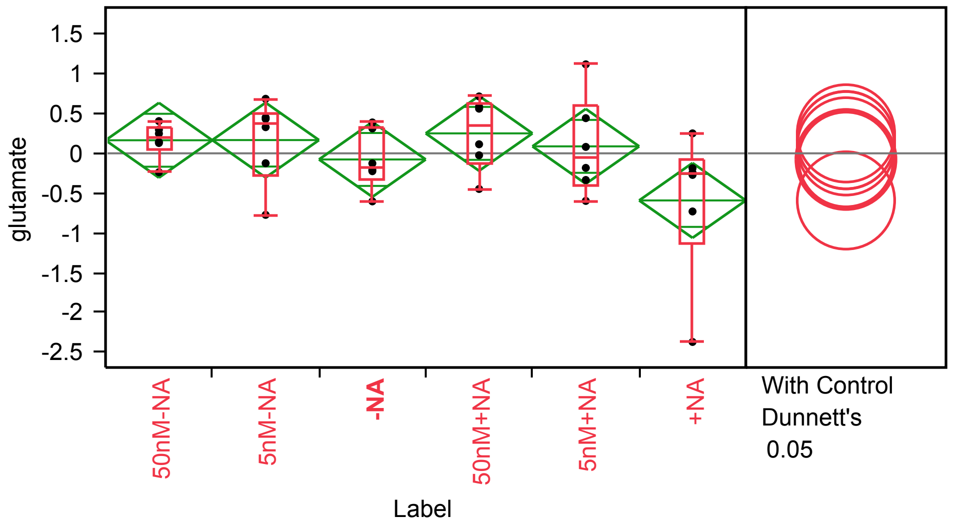


## Quantiles

| Level | Minimum | 10% | 25% | Median | 75% | 90% | Maximum |
|---|---|---|---|---|---|---|---|
| 50nM-NA | -0.23742 | -0.23742 | 0.037912 | 0.195374 | 0.324834 | 0.404835 | 0.404835 |
| 5nM-NA | -0.77353 | -0.77353 | -0.28672 | 0.380603 | 0.509447 | 0.685491 | 0.685491 |
| -NA | -0.60399 | -0.60399 | -0.31945 | -0.16767 | 0.331996 | 0.388237 | 0.388237 |
| 50nM+NA | -0.44786 | -0.44786 | -0.13044 | 0.337629 | 0.619058 | 0.715437 | 0.715437 |
| 5nM+NA | -0.59814 | -0.59814 | -0.4009 | -0.05124 | 0.611371 | 1.118727 | 1.118727 |
| +NA | -2.37134 | -2.37134 | -1.14097 | -0.25724 | -0.07793 | 0.250758 | 0.250758 |

## Oneway Anova

### Summary of Fit

| | |
|---|---|
| Rsquare | 0.232402 |
| Adj Rsquare | 0.104469 |
| Root Mean Square Error | 0.564286 |
| Mean of Response | 0 |
| Observations (or Sum Wgts) | 36 |

### Analysis of Variance

| Source | DF | Sum of Squares | Mean Square | F Ratio | Prob > F |
|---|---|---|---|---|---|
| Label | 5 | 2.892174 | 0.578435 | 1.8166 | 0.1397 |
| Error | 30 | 9.552547 | 0.318418 | | |
| C. Total | 35 | 12.444721 | | | |

### Means for Oneway Anova

| Level | Number | Mean | Std Error | Lower 95% | Upper 95% |
|---|---|---|---|---|---|
| 50nM-NA | 6 | 0.16434 | 0.23037 | -0.306 | 0.6348 |
| 5nM-NA | 6 | 0.16658 | 0.23037 | -0.304 | 0.6371 |
| -NA | 6 | -0.07707 | 0.23037 | -0.548 | 0.3934 |
| 50nM+NA | 6 | 0.25086 | 0.23037 | -0.220 | 0.7213 |
| 5nM+NA | 6 | 0.08753 | 0.23037 | -0.383 | 0.5580 |
| +NA | 6 | -0.59223 | 0.23037 | -1.063 | -0.1218 |

Std Error uses a pooled estimate of error variance

## Means Comparisons

### Comparisons with a control using Dunnett's Method

## Oneway Analysis of glutamine By Label

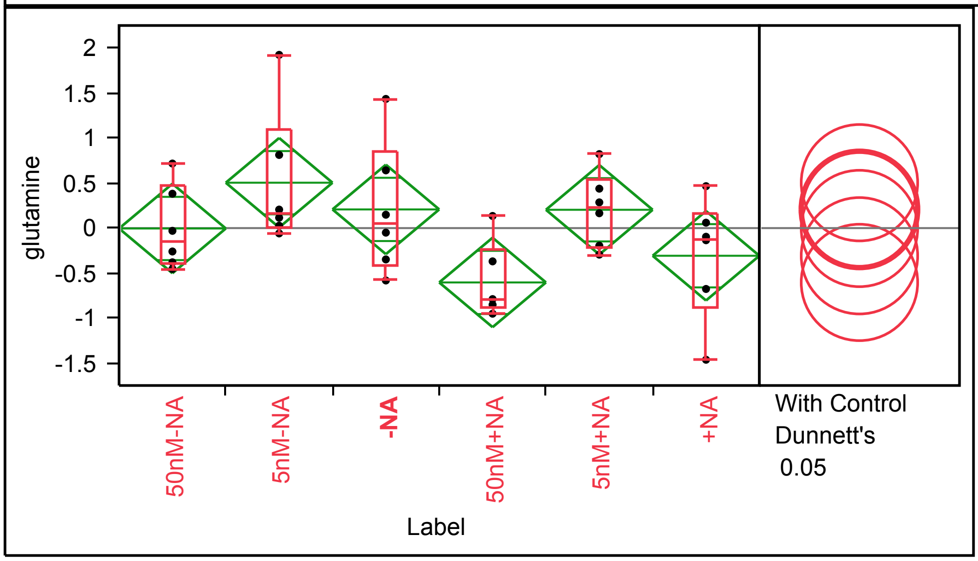


## Quantiles

| Level | Minimum | 10% | 25% | Median | 75% | 90% | Maximum |
|---|---|---|---|---|---|---|---|
| 50nM-NA | -0.45404 | -0.45404 | -0.39874 | -0.14527 | 0.465999 | 0.717626 | 0.717626 |
| 5nM-NA | -0.0589 | -0.0589 | 0.00513 | 0.160437 | 1.091673 | 1.926207 | 1.926207 |
| -NA | -0.58232 | -0.58232 | -0.40673 | 0.04912 | 0.840682 | 1.436242 | 1.436242 |
| 50nM+NA | -0.95174 | -0.95174 | -0.87687 | -0.79327 | -0.2437 | 0.134729 | 0.134729 |
| 5nM+NA | -0.29481 | -0.29481 | -0.2202 | 0.225612 | 0.53444 | 0.822534 | 0.822534 |
| +NA | -1.46374 | -1.46374 | -0.87356 | -0.11565 | 0.161488 | 0.466089 | 0.466089 |

## Oneway Anova

### Summary of Fit

| | |
|---|---|
| Rsquare | 0.30993 |
| Adj Rsquare | 0.194918 |
| Root Mean Square Error | 0.596516 |
| Mean of Response | 1.111e-9 |
| Observations (or Sum Wgts) | 36 |

### Analysis of Variance

| Source | DF | Sum of Squares | Mean Square | F Ratio | Prob > F |
|---|---|---|---|---|---|
| Label | 5 | 4.794407 | 0.958881 | 2.6948 | 0.0398 * |
| Error | 30 | 10.674923 | 0.355831 | | |
| C. Total | 35 | 15.469330 | | | |

### Means for Oneway Anova

| Level | Number | Mean | Std Error | Lower 95% | Upper 95% |
|---|---|---|---|---|---|
| 50nM-NA | 6 | -0.00419 | 0.24353 | -0.502 | 0.493 |
| 5nM-NA | 6 | 0.50469 | 0.24353 | 0.00734 | 1.002 |
| -NA | 6 | 0.20769 | 0.24353 | -0.290 | 0.705 |
| 50nM+NA | 6 | -0.60422 | 0.24353 | -1.102 | -0.107 |
| 5nM+NA | 6 | 0.20367 | 0.24353 | -0.294 | 0.701 |
| +NA | 6 | -0.30764 | 0.24353 | -0.805 | 0.190 |

Std Error uses a pooled estimate of error variance

## Means Comparisons

### Comparisons with a control using Dunnett's Method

## Oneway Analysis of glutathione By Label

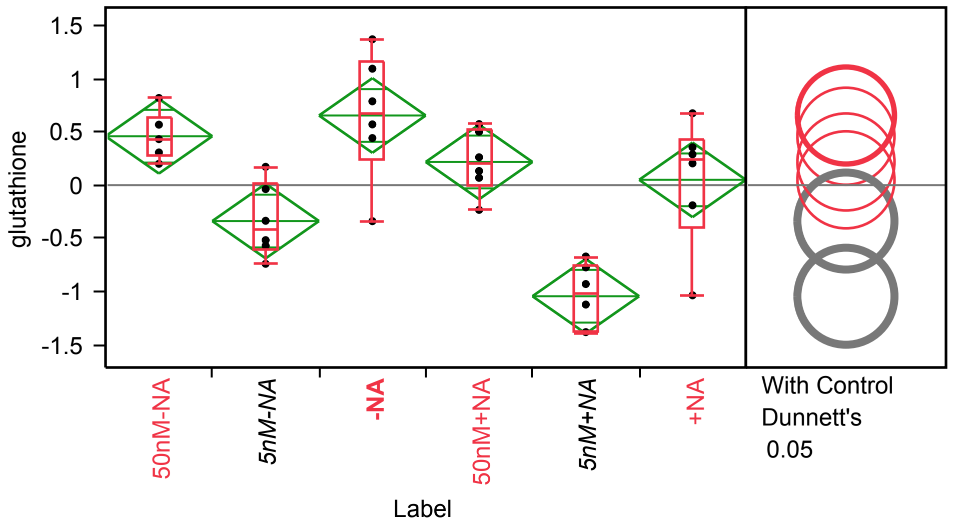


## Quantiles

| Level | Minimum | 10% | 25% | Median | 75% | 90% | Maximum |
|---|---|---|---|---|---|---|---|
| 50nM-NA | 0.200151 | 0.200151 | 0.279911 | 0.430789 | 0.629773 | 0.81765 | 0.81765 |
| 5nM-NA | -0.73801 | -0.73801 | -0.61104 | -0.42536 | 0.015183 | 0.171698 | 0.171698 |
| -NA | -0.33818 | -0.33818 | 0.246631 | 0.678218 | 1.161399 | 1.3685 | 1.3685 |
| 50nM+NA | -0.23224 | -0.23224 | -0.0067 | 0.197041 | 0.519192 | 0.572976 | 0.572976 |
| 5nM+NA | -1.38542 | -1.38542 | -1.38074 | -1.02433 | -0.74608 | -0.66986 | -0.66986 |
| +NA | -1.0338 | -1.0338 | -0.4004 | 0.24653 | 0.434652 | 0.674314 | 0.674314 |

## Oneway Anova

### Summary of Fit

| | |
|---|---|
| Rsquare | 0.681924 |
| Adj Rsquare | 0.628911 |
| Root Mean Square Error | 0.419632 |
| Mean of Response | -2.8e-10 |
| Observations (or Sum Wgts) | 36 |

### Analysis of Variance

| Source | DF | Sum of Squares | Mean Square | F Ratio | Prob > F |
|---|---|---|---|---|---|
| Label | 5 | 11.325625 | 2.26513 | 12.8634 | <.0001 * |
| Error | 30 | 5.282722 | 0.17609 | | |
| C. Total | 35 | 16.608347 | | | |

### Means for Oneway Anova

| Level | Number | Mean | Std Error | Lower 95% | Upper 95% |
|---|---|---|---|---|---|
| 50nM-NA | 6 | 0.4588 | 0.17131 | 0.109 | 0.809 |
| 5nM-NA | 6 | -0.3371 | 0.17131 | -0.687 | 0.013 |
| -NA | 6 | 0.6534 | 0.17131 | 0.304 | 1.003 |
| 50nM+NA | 6 | 0.2174 | 0.17131 | -0.132 | 0.567 |
| 5nM+NA | 6 | -1.0424 | 0.17131 | -1.392 | -0.693 |
| +NA | 6 | 0.0498 | 0.17131 | -0.300 | 0.400 |

Std Error uses a pooled estimate of error variance

## Means Comparisons

### Comparisons with a control using Dunnett's Method

## Oneway Analysis of glutathione disulfide By Label

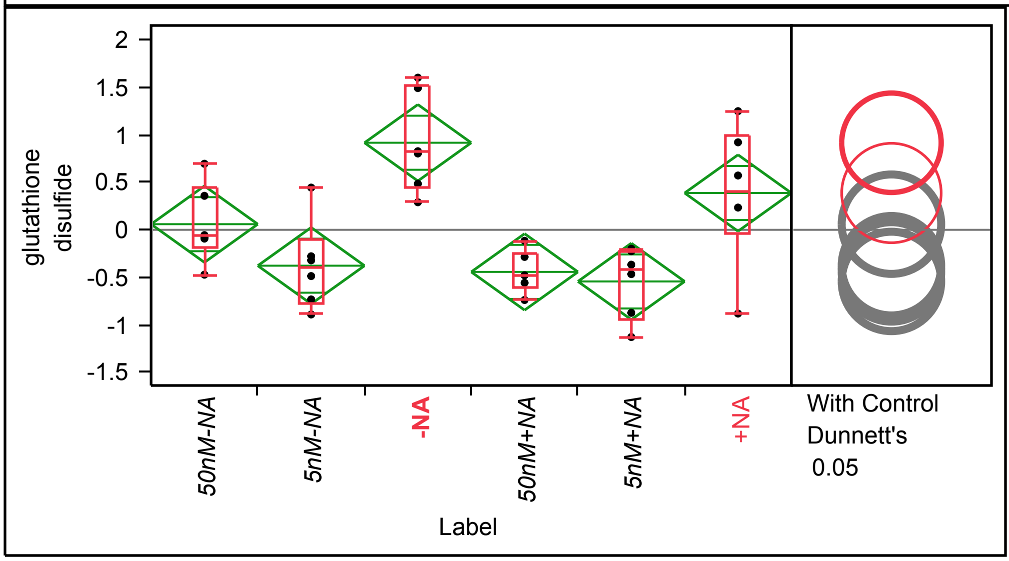


## Quantiles

| Level | Minimum | 10% | 25% | Median | 75% | 90% | Maximum |
|---|---|---|---|---|---|---|---|
| 50nM-NA | -0.47295 | -0.47295 | -0.18766 | -0.06601 | 0.443323 | 0.696976 | 0.696976 |
| 5nM-NA | -0.89281 | -0.89281 | -0.77386 | -0.4048 | -0.09803 | 0.445432 | 0.445432 |
| -NA | 0.29114 | 0.29114 | 0.437112 | 0.817306 | 1.522529 | 1.605017 | 1.605017 |
| 50nM+NA | -0.74014 | -0.74014 | -0.60432 | -0.48153 | -0.24325 | -0.1159 | -0.1159 |
| 5nM+NA | -1.13089 | -1.13089 | -0.93943 | -0.41744 | -0.21869 | -0.19988 | -0.19988 |
| +NA | -0.88312 | -0.88312 | -0.04786 | 0.404003 | 1.005462 | 1.25187 | 1.25187 |

## Oneway Anova

### Summary of Fit

| | |
|---|---|
| Rsquare | 0.583091 |
| Adj Rsquare | 0.513606 |
| Root Mean Square Error | 0.483519 |
| Mean of Response | 8.33e-10 |
| Observations (or Sum Wgts) | 36 |

### Analysis of Variance

| Source | DF | Sum of Squares | Mean Square | F Ratio | Prob > F |
|---|---|---|---|---|---|
| Label | 5 | 9.809395 | 1.96188 | 8.3916 | <.0001 * |
| Error | 30 | 7.013711 | 0.23379 | | |
| C. Total | 35 | 16.823106 | | | |

### Means for Oneway Anova

| Level | Number | Mean | Std Error | Lower 95% | Upper 95% |
|---|---|---|---|---|---|
| 50nM-NA | 6 | 0.05970 | 0.19740 | -0.3434 | 0.463 |
| 5nM-NA | 6 | -0.37840 | 0.19740 | -0.7815 | 0.025 |
| -NA | 6 | 0.91860 | 0.19740 | 0.5155 | 1.322 |
| 50nM+NA | 6 | -0.44397 | 0.19740 | -0.8471 | -0.041 |
| 5nM+NA | 6 | -0.54437 | 0.19740 | -0.9475 | -0.141 |
| +NA | 6 | 0.38844 | 0.19740 | -0.0147 | 0.792 |

Std Error uses a pooled estimate of error variance

## Means Comparisons

### Comparisons with a control using Dunnett's Method

## Oneway Analysis of Glycerophosphocholine By Label

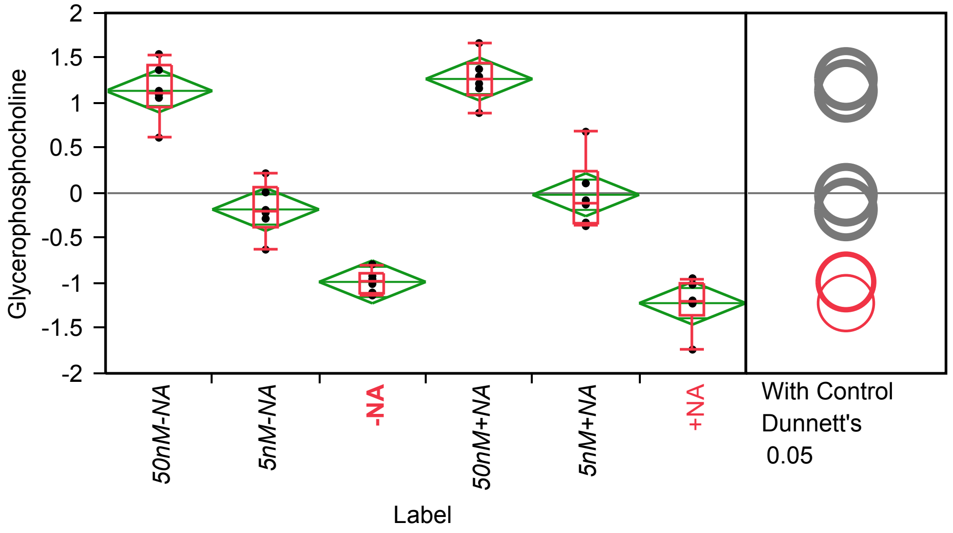


### Quantiles

| Level | Minimum | 10% | 25% | Median | 75% | 90% | Maximum |
|---|---|---|---|---|---|---|---|
| 50nM-NA | 0.616597 | 0.616597 | 0.947771 | 1.115027 | 1.411294 | 1.543703 | 1.543703 |
| 5nM-NA | -0.62624 | -0.62624 | -0.36866 | -0.20186 | 0.061982 | 0.22056 | 0.22056 |
| -NA | -1.1353 | -1.1353 | -1.11126 | -0.98115 | -0.88721 | -0.78986 | -0.78986 |
| 50nM+NA | 0.890782 | 0.890782 | 1.095057 | 1.255634 | 1.449334 | 1.665503 | 1.665503 |
| 5nM+NA | -0.3652 | -0.3652 | -0.33632 | -0.10276 | 0.252484 | 0.68192 | 0.68192 |
| +NA | -1.73617 | -1.73617 | -1.35286 | -1.2016 | -0.99723 | -0.94495 | -0.94495 |

### Oneway Anova

#### Summary of Fit

| | |
|---|---|
| Rsquare | 0.930019 |
| Adj Rsquare | 0.918356 |
| Root Mean Square Error | 0.284847 |
| Mean of Response | 2.78e-11 |
| Observations (or Sum Wgts) | 36 |

#### Analysis of Variance

| Source | DF | Sum of Squares | Mean Square | F Ratio | Prob > F |
|---|---|---|---|---|---|
| Label | 5 | 32.348912 | 6.46978 | 79.7379 | <.0001 * |
| Error | 30 | 2.434142 | 0.08114 | | |
| C. Total | 35 | 34.783054 | | | |

#### Means for Oneway Anova

| Level | Number | Mean | Std Error | Lower 95% | Upper 95% |
|---|---|---|---|---|---|
| 50nM-NA | 6 | 1.1359 | 0.11629 | 0.898 | 1.373 |
| 5nM-NA | 6 | -0.1805 | 0.11629 | -0.418 | 0.057 |
| -NA | 6 | -0.9851 | 0.11629 | -1.223 | -0.748 |
| 50nM+NA | 6 | 1.2680 | 0.11629 | 1.031 | 1.505 |
| 5nM+NA | 6 | -0.0177 | 0.11629 | -0.255 | 0.220 |
| +NA | 6 | -1.2207 | 0.11629 | -1.458 | -0.983 |

Std Error uses a pooled estimate of error variance

### Means Comparisons

#### Comparisons with a control using Dunnett's Method

## Oneway Analysis of glycine By Label

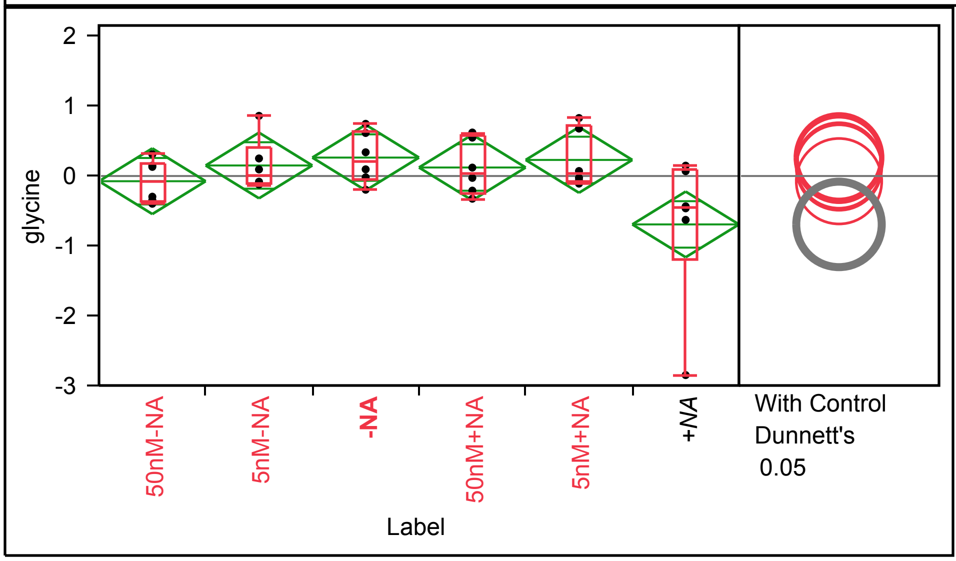


### Quantiles

| Level | Minimum | 10% | 25% | Median | 75% | 90% | Maximum |
|---|---|---|---|---|---|---|---|
| 50nM-NA | -0.39711 | -0.39711 | -0.35776 | -0.08279 | 0.189717 | 0.311414 | 0.311414 |
| 5nM-NA | -0.12222 | -0.12222 | -0.10343 | 0.00639 | 0.404021 | 0.861497 | 0.861497 |
| -NA | -0.19602 | -0.19602 | -0.063 | 0.21785 | 0.649028 | 0.747119 | 0.747119 |
| 50nM+NA | -0.32494 | -0.32494 | -0.23831 | 0.046884 | 0.568065 | 0.622719 | 0.622719 |
| 5nM+NA | -0.10874 | -0.10874 | -0.07562 | 0.022787 | 0.717243 | 0.830244 | 0.830244 |
| +NA | -2.85094 | -2.85094 | -1.18358 | -0.44844 | 0.089085 | 0.148 | 0.148 |

### Oneway Anova

#### Summary of Fit

| | |
|---|---|
| Rsquare | 0.288833 |
| Adj Rsquare | 0.170305 |
| Root Mean Square Error | 0.564164 |
| Mean of Response | 8.33e-10 |
| Observations (or Sum Wgts) | 36 |

#### Analysis of Variance

| Source | DF | Sum of Squares | Mean Square | F Ratio | Prob > F |
|---|---|---|---|---|---|
| Label | 5 | 3.877994 | 0.775599 | 2.4368 | 0.0574 |
| Error | 30 | 9.548432 | 0.318281 | | |
| C. Total | 35 | 13.426425 | | | |

#### Means for Oneway Anova

| Level | Number | Mean | Std Error | Lower 95% | Upper 95% |
|---|---|---|---|---|---|
| 50nM-NA | 6 | -0.07446 | 0.23032 | -0.545 | 0.3959 |
| 5nM-NA | 6 | 0.15107 | 0.23032 | -0.319 | 0.6214 |
| -NA | 6 | 0.26408 | 0.23032 | -0.206 | 0.7345 |
| 50nM+NA | 6 | 0.12199 | 0.23032 | -0.348 | 0.5924 |
| 5nM+NA | 6 | 0.23034 | 0.23032 | -0.240 | 0.7007 |
| +NA | 6 | -0.69303 | 0.23032 | -1.163 | -0.2227 |

Std Error uses a pooled estimate of error variance

### Means Comparisons

#### Comparisons with a control using Dunnett's Method

## Oneway Analysis of GMP By Label

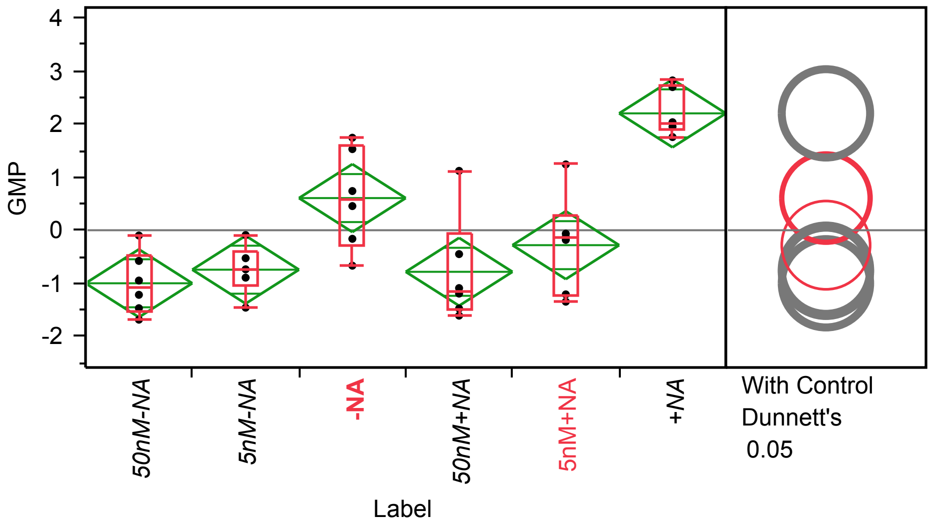


## Quantiles

| Level | Minimum | 10% | 25% | Median | 75% | 90% | Maximum |
|---|---|---|---|---|---|---|---|
| 50nM-NA | -1.68423 | -1.68423 | -1.52234 | -1.08207 | -0.45813 | -0.09972 | -0.09972 |
| 5nM-NA | -1.45784 | -1.45784 | -1.03421 | -0.74462 | -0.41786 | -0.09167 | -0.09167 |
| -NA | -0.6674 | -0.6674 | -0.28813 | 0.596286 | 1.58124 | 1.741673 | 1.741673 |
| 50nM+NA | -1.60801 | -1.60801 | -1.50864 | -1.14135 | -0.05772 | 1.117746 | 1.117746 |
| 5nM+NA | -1.34541 | -1.34541 | -1.24254 | -0.15404 | 0.260975 | 1.239301 | 1.239301 |
| +NA | 1.75467 | 1.75467 | 1.900623 | 1.99202 | 2.727128 | 2.82139 | 2.82139 |

## Oneway Anova

### Summary of Fit

| | |
|---|---|
| Rsquare | 0.717165 |
| Adj Rsquare | 0.670026 |
| Root Mean Square Error | 0.766706 |
| Mean of Response | 2.78e-10 |
| Observations (or Sum Wgts) | 36 |

### Analysis of Variance

| Source | DF | Sum of Squares | Mean Square | F Ratio | Prob > F |
|---|---|---|---|---|---|
| Label | 5 | 44.716131 | 8.94323 | 15.2138 | <.0001 * |
| Error | 30 | 17.635136 | 0.58784 | | |
| C. Total | 35 | 62.351267 | | | |

### Means for Oneway Anova

| Level | Number | Mean | Std Error | Lower 95% | Upper 95% |
|---|---|---|---|---|---|
| 50nM-NA | 6 | -0.9990 | 0.31301 | -1.638 | -0.360 |
| 5nM-NA | 6 | -0.7431 | 0.31301 | -1.382 | -0.104 |
| -NA | 6 | 0.6055 | 0.31301 | -0.034 | 1.245 |
| 50nM+NA | 6 | -0.7830 | 0.31301 | -1.422 | -0.144 |
| 5nM+NA | 6 | -0.2813 | 0.31301 | -0.921 | 0.358 |
| +NA | 6 | 2.2008 | 0.31301 | 1.562 | 2.840 |

Std Error uses a pooled estimate of error variance

## Means Comparisons

### Comparisons with a control using Dunnett's Method

## Oneway Analysis of Guanidoacetic acid By Label

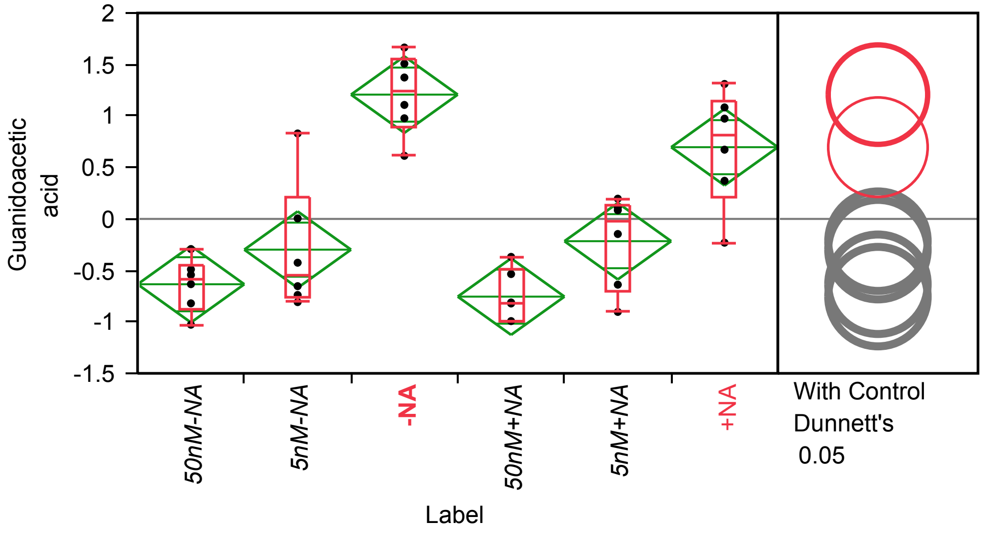


## Quantiles

| Level | Minimum | 10% | 25% | Median | 75% | 90% | Maximum |
|---|---|---|---|---|---|---|---|
| 50nM-NA | -1.02743 | -1.02743 | -0.87202 | -0.58845 | -0.44093 | -0.29312 | -0.29312 |
| 5nM-NA | -0.80808 | -0.80808 | -0.75625 | -0.53925 | 0.21124 | 0.829887 | 0.829887 |
| -NA | 0.61294 | 0.61294 | 0.886313 | 1.240441 | 1.547318 | 1.664759 | 1.664759 |
| 50nM+NA | -0.99903 | -0.99903 | -0.99411 | -0.8165 | -0.49376 | -0.36874 | -0.36874 |
| 5nM+NA | -0.90316 | -0.90316 | -0.70477 | -0.03136 | 0.132221 | 0.19648 | 0.19648 |
| +NA | -0.23066 | -0.23066 | 0.22008 | 0.823308 | 1.14009 | 1.310698 | 1.310698 |

## Oneway Anova

### Summary of Fit

| | |
|---|---|
| Rsquare | 0.754632 |
| Adj Rsquare | 0.713737 |
| Root Mean Square Error | 0.445457 |
| Mean of Response | 2.78e-11 |
| Observations (or Sum Wgts) | 36 |

### Analysis of Variance

| Source | DF | Sum of Squares | Mean Square | F Ratio | Prob > F |
|---|---|---|---|---|---|
| Label | 5 | 18.308360 | 3.66167 | 18.4530 | <.0001 * |
| Error | 30 | 5.952956 | 0.19843 | | |
| C. Total | 35 | 24.261315 | | | |

### Means for Oneway Anova

| Level | Number | Mean | Std Error | Lower 95% | Upper 95% |
|---|---|---|---|---|---|
| 50nM-NA | 6 | -0.6346 | 0.18186 | -1.006 | -0.263 |
| 5nM-NA | 6 | -0.2984 | 0.18186 | -0.670 | 0.073 |
| -NA | 6 | 1.2074 | 0.18186 | 0.836 | 1.579 |
| 50nM+NA | 6 | -0.7548 | 0.18186 | -1.126 | -0.383 |
| 5nM+NA | 6 | -0.2162 | 0.18186 | -0.588 | 0.155 |
| +NA | 6 | 0.6967 | 0.18186 | 0.325 | 1.068 |

Std Error uses a pooled estimate of error variance

## Means Comparisons

### Comparisons with a control using Dunnett's Method

## Oneway Analysis of histidine By Label

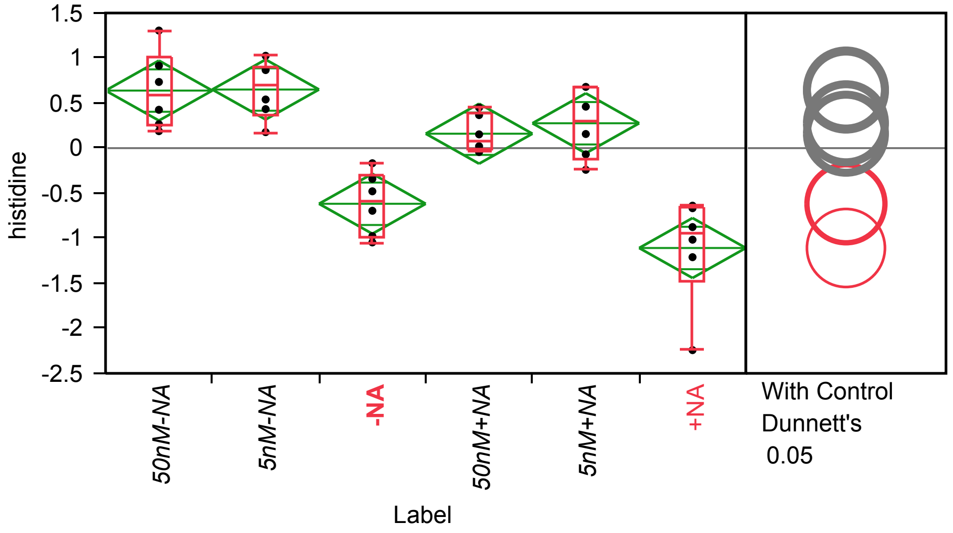


### Quantiles

| Level | Minimum | 10% | 25% | Median | 75% | 90% | Maximum |
|---|---|---|---|---|---|---|---|
| 50nM-NA | 0.188544 | 0.188544 | 0.246292 | 0.580182 | 1.012277 | 1.305121 | 1.305121 |
| 5nM-NA | 0.177592 | 0.177592 | 0.369319 | 0.70083 | 0.906263 | 1.024813 | 1.024813 |
| -NA | -1.04647 | -1.04647 | -0.9941 | -0.58727 | -0.29737 | -0.16766 | -0.16766 |
| 50nM+NA | -0.04307 | -0.04307 | -0.00061 | 0.0871 | 0.387698 | 0.449766 | 0.449766 |
| 5nM+NA | -0.2402 | -0.2402 | -0.11189 | 0.308772 | 0.671635 | 0.679872 | 0.679872 |
| +NA | -2.24214 | -2.24214 | -1.46836 | -0.94625 | -0.65978 | -0.639 | -0.639 |

### Oneway Anova

#### Summary of Fit

| | |
|---|---|
| Rsquare | 0.76143 |
| Adj Rsquare | 0.721669 |
| Root Mean Square Error | 0.39926 |
| Mean of Response | 2.78e-10 |
| Observations (or Sum Wgts) | 36 |

#### Analysis of Variance

| Source | DF | Sum of Squares | Mean Square | F Ratio | Prob > F |
|---|---|---|---|---|---|
| Label | 5 | 15.263273 | 3.05265 | 19.1499 | <.0001 * |
| Error | 30 | 4.782252 | 0.15941 | | |
| C. Total | 35 | 20.045525 | | | |

#### Means for Oneway Anova

| Level | Number | Mean | Std Error | Lower 95% | Upper 95% |
|---|---|---|---|---|---|
| 50nM-NA | 6 | 0.6390 | 0.16300 | 0.306 | 0.9719 |
| 5nM-NA | 6 | 0.6507 | 0.16300 | 0.318 | 0.9836 |
| -NA | 6 | -0.6177 | 0.16300 | -0.951 | -0.2848 |
| 50nM+NA | 6 | 0.1602 | 0.16300 | -0.173 | 0.4931 |
| 5nM+NA | 6 | 0.2762 | 0.16300 | -0.057 | 0.6090 |
| +NA | 6 | -1.1085 | 0.16300 | -1.441 | -0.7756 |

Std Error uses a pooled estimate of error variance

### Means Comparisons

#### Comparisons with a control using Dunnett's Method

## Oneway Analysis of homocysteine By Label

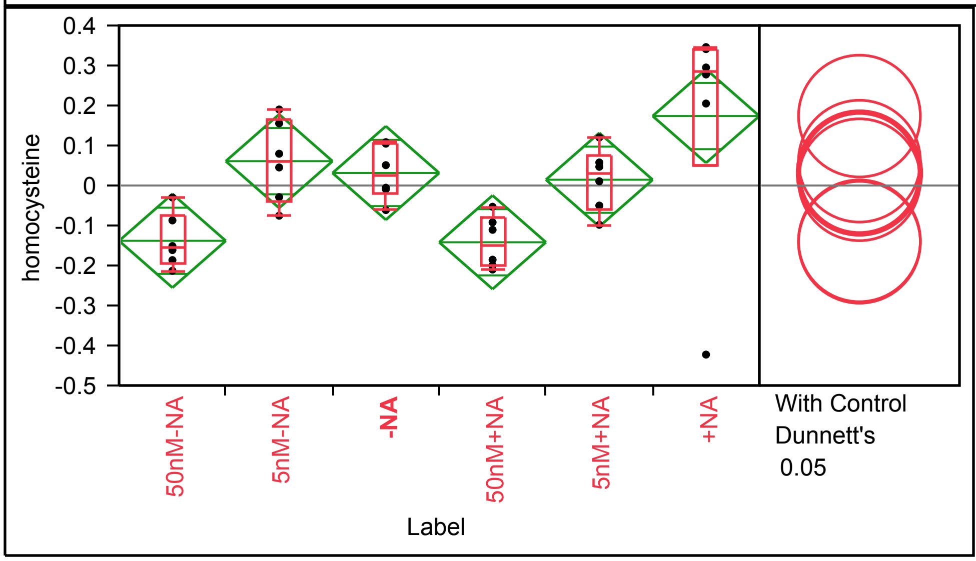


### Quantiles

| Level | Minimum | 10% | 25% | Median | 75% | 90% | Maximum |
|---|---|---|---|---|---|---|---|
| 50nM-NA | -0.21313 | -0.21313 | -0.19321 | -0.15641 | -0.07291 | -0.02998 | -0.02998 |
| 5nM-NA | -0.07551 | -0.07551 | -0.04067 | 0.062259 | 0.163968 | 0.189963 | 0.189963 |
| -NA | -0.06142 | -0.06142 | -0.02224 | 0.022615 | 0.105541 | 0.108318 | 0.108318 |
| 50nM+NA | -0.20998 | -0.20998 | -0.20222 | -0.14817 | -0.08244 | -0.05319 | -0.05319 |
| 5nM+NA | -0.09821 | -0.09821 | -0.06221 | 0.028716 | 0.073163 | 0.120032 | 0.120032 |
| +NA | -0.42277 | -0.42277 | 0.047982 | 0.286275 | 0.34226 | 0.345992 | 0.345992 |

### Oneway Anova

#### Summary of Fit

| | |
|---|---|
| Rsquare | 0.429871 |
| Adj Rsquare | 0.334849 |
| Root Mean Square Error | 0.140376 |
| Mean of Response | -5.6e-11 |
| Observations (or Sum Wgts) | 36 |

#### Analysis of Variance

| Source | DF | Sum of Squares | Mean Square | F Ratio | Prob > F |
|---|---|---|---|---|---|
| Label | 5 | 0.4457292 | 0.089146 | 4.5239 | 0.0034 * |
| Error | 30 | 0.5911622 | 0.019705 | | |
| C. Total | 35 | 1.0368914 | | | |

#### Means for Oneway Anova

| Level | Number | Mean | Std Error | Lower 95% | Upper 95% |
|---|---|---|---|---|---|
| 50nM-NA | 6 | -0.13829 | 0.05731 | -0.2553 | -0.0213 |
| 5nM-NA | 6 | 0.06087 | 0.05731 | -0.0562 | 0.1779 |
| -NA | 6 | 0.03126 | 0.05731 | -0.0858 | 0.1483 |
| 50nM+NA | 6 | -0.14189 | 0.05731 | -0.2589 | -0.0248 |
| 5nM+NA | 6 | 0.01443 | 0.05731 | -0.1026 | 0.1315 |
| +NA | 6 | 0.17361 | 0.05731 | 0.0566 | 0.2907 |

Std Error uses a pooled estimate of error variance

### Means Comparisons

#### Comparisons with a control using Dunnett's Method

## Oneway Analysis of homoserine By Label

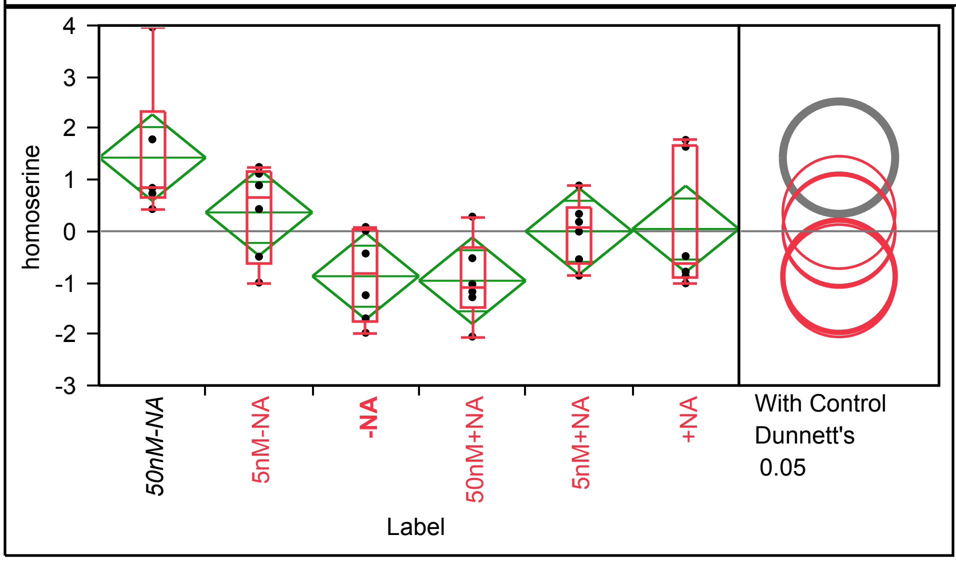


### Quantiles

| Level | Minimum | 10% | 25% | Median | 75% | 90% | Maximum |
|---|---|---|---|---|---|---|---|
| 50nM-NA | 0.431205 | 0.431205 | 0.658795 | 0.833375 | 2.330996 | 3.964943 | 3.964943 |
| 5nM-NA | -0.99817 | -0.99817 | -0.62099 | 0.661439 | 1.150897 | 1.247832 | 1.247832 |
| -NA | -1.97502 | -1.97502 | -1.76445 | -0.83852 | 0.029726 | 0.082439 | 0.082439 |
| 50nM+NA | -2.04913 | -2.04913 | -1.47506 | -1.10237 | -0.32277 | 0.282959 | 0.282959 |
| 5nM+NA | -0.86347 | -0.86347 | -0.62469 | 0.086919 | 0.474927 | 0.888309 | 0.888309 |
| +NA | -1.01295 | -1.01295 | -0.90814 | -0.63534 | 1.675417 | 1.775182 | 1.775182 |

### Oneway Anova

#### Summary of Fit

| | |
|---|---|
| Rsquare | 0.433275 |
| Adj Rsquare | 0.338821 |
| Root Mean Square Error | 1.007022 |
| Mean of Response | -2.8e-11 |
| Observations (or Sum Wgts) | 36 |

#### Analysis of Variance

| Source | DF | Sum of Squares | Mean Square | F Ratio | Prob > F |
|---|---|---|---|---|---|
| Label | 5 | 23.258975 | 4.65179 | 4.5872 | 0.0032 * |
| Error | 30 | 30.422778 | 1.01409 | | |
| C. Total | 35 | 53.681753 | | | |

#### Means for Oneway Anova

| Level | Number | Mean | Std Error | Lower 95% | Upper 95% |
|---|---|---|---|---|---|
| 50nM-NA | 6 | 1.4307 | 0.41111 | 0.591 | 2.270 |
| 5nM-NA | 6 | 0.3660 | 0.41111 | -0.474 | 1.206 |
| -NA | 6 | -0.8753 | 0.41111 | -1.715 | -0.036 |
| 50nM+NA | 6 | -0.9632 | 0.41111 | -1.803 | -0.124 |
| 5nM+NA | 6 | -0.0015 | 0.41111 | -0.841 | 0.838 |
| +NA | 6 | 0.0434 | 0.41111 | -0.796 | 0.883 |

Std Error uses a pooled estimate of error variance

### Means Comparisons

#### Comparisons with a control using Dunnett's Method

## Oneway Analysis of Hydroxyisocaproic acid By Label

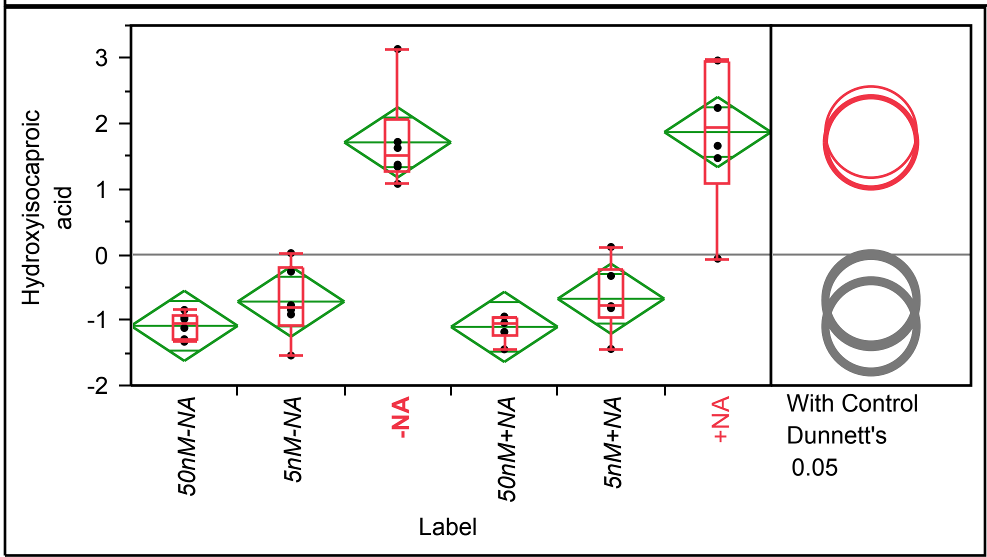


### Quantiles

| Level | Minimum | 10% | 25% | Median | 75% | 90% | Maximum |
|---|---|---|---|---|---|---|---|
| 50nM-NA | -1.33163 | -1.33163 | -1.30245 | -1.05243 | -0.92846 | -0.84554 | -0.84554 |
| 5nM-NA | -1.54157 | -1.54157 | -1.0711 | -0.80749 | -0.18914 | 0.022529 | 0.022529 |
| -NA | 1.080477 | 1.080477 | 1.276696 | 1.501376 | 2.07629 | 3.140562 | 3.140562 |
| 50nM+NA | -1.44743 | -1.44743 | -1.24713 | -1.05234 | -0.9473 | -0.94694 | -0.94694 |
| 5nM+NA | -1.44196 | -1.44196 | -0.97404 | -0.79092 | -0.21542 | 0.116368 | 0.116368 |
| +NA | -0.06276 | -0.06276 | 1.089942 | 1.951166 | 2.95438 | 2.965314 | 2.965314 |

### Oneway Anova

#### Summary of Fit

| | |
|---|---|
| Rsquare | 0.826311 |
| Adj Rsquare | 0.797363 |
| Root Mean Square Error | 0.642482 |
| Mean of Response | 5.56e-10 |
| Observations (or Sum Wgts) | 36 |

#### Analysis of Variance

| Source | DF | Sum of Squares | Mean Square | F Ratio | Prob > F |
|---|---|---|---|---|---|
| Label | 5 | 58.913387 | 11.7827 | 28.5445 | <.0001 * |
| Error | 30 | 12.383502 | 0.4128 | | |
| C. Total | 35 | 71.296889 | | | |

#### Means for Oneway Anova

| Level | Number | Mean | Std Error | Lower 95% | Upper 95% |
|---|---|---|---|---|---|
| 50nM-NA | 6 | -1.0885 | 0.26229 | -1.624 | -0.553 |
| 5nM-NA | 6 | -0.7180 | 0.26229 | -1.254 | -0.182 |
| -NA | 6 | 1.7146 | 0.26229 | 1.179 | 2.250 |
| 50nM+NA | 6 | -1.1045 | 0.26229 | -1.640 | -0.569 |
| 5nM+NA | 6 | -0.6753 | 0.26229 | -1.211 | -0.140 |
| +NA | 6 | 1.8716 | 0.26229 | 1.336 | 2.407 |

Std Error uses a pooled estimate of error variance

### Means Comparisons

#### Comparisons with a control using Dunnett's Method

## Oneway Analysis of Hydroxylamine By Label

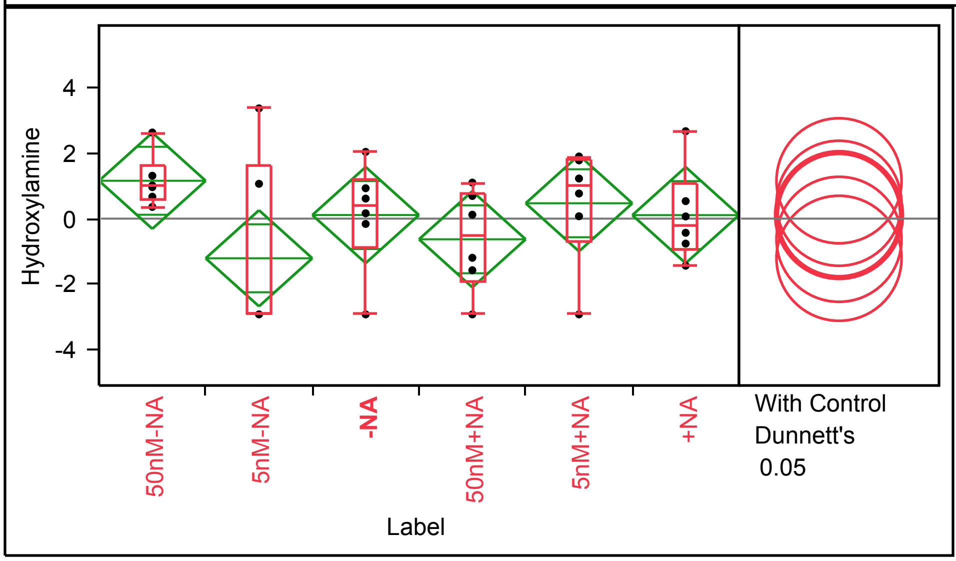


## Quantiles

| Level | Minimum | 10% | 25% | Median | 75% | 90% | Maximum |
|---|---|---|---|---|---|---|---|
| 50nM-NA | 0.356315 | 0.356315 | 0.588245 | 0.980738 | 1.630286 | 2.615176 | 2.615176 |
| 5nM-NA | -2.90823 | -2.90823 | -2.90823 | -2.90818 | 1.630733 | 3.349005 | 3.349005 |
| -NA | -2.90824 | -2.90824 | -0.84785 | 0.38551 | 1.199078 | 2.027783 | 2.027783 |
| 50nM+NA | -2.90831 | -2.90831 | -1.90259 | -0.53543 | 0.789628 | 1.093404 | 1.093404 |
| 5nM+NA | -2.90832 | -2.90832 | -0.673 | 0.990872 | 1.798883 | 1.887163 | 1.887163 |
| +NA | -1.42909 | -1.42909 | -0.92654 | -0.18145 | 1.062158 | 2.655594 | 2.655594 |

## Oneway Anova

### Summary of Fit

| | |
|---|---|
| Rsquare | 0.181888 |
| Adj Rsquare | 0.045536 |
| Root Mean Square Error | 1.750907 |
| Mean of Response | -4.6e-17 |
| Observations (or Sum Wgts) | 36 |

### Analysis of Variance

| Source | DF | Sum of Squares | Mean Square | F Ratio | Prob > F |
|---|---|---|---|---|---|
| Label | 5 | 20.44746 | 4.08949 | 1.3340 | 0.2769 |
| Error | 30 | 91.97026 | 3.06568 | | |
| C. Total | 35 | 112.41773 | | | |

### Means for Oneway Anova

| Level | Number | Mean | Std Error | Lower 95% | Upper 95% |
|---|---|---|---|---|---|
| 50nM-NA | 6 | 1.1501 | 0.71480 | -0.310 | 2.6099 |
| 5nM-NA | 6 | -1.2043 | 0.71480 | -2.664 | 0.2555 |
| -NA | 6 | 0.1087 | 0.71480 | -1.351 | 1.5686 |
| 50nM+NA | 6 | -0.6275 | 0.71480 | -2.087 | 0.8324 |
| 5nM+NA | 6 | 0.4670 | 0.71480 | -0.993 | 1.9268 |
| +NA | 6 | 0.1059 | 0.71480 | -1.354 | 1.5658 |

Std Error uses a pooled estimate of error variance

## Means Comparisons

### Comparisons with a control using Dunnett's Method

## Oneway Analysis of hydroxyphenylpyruvate By Label

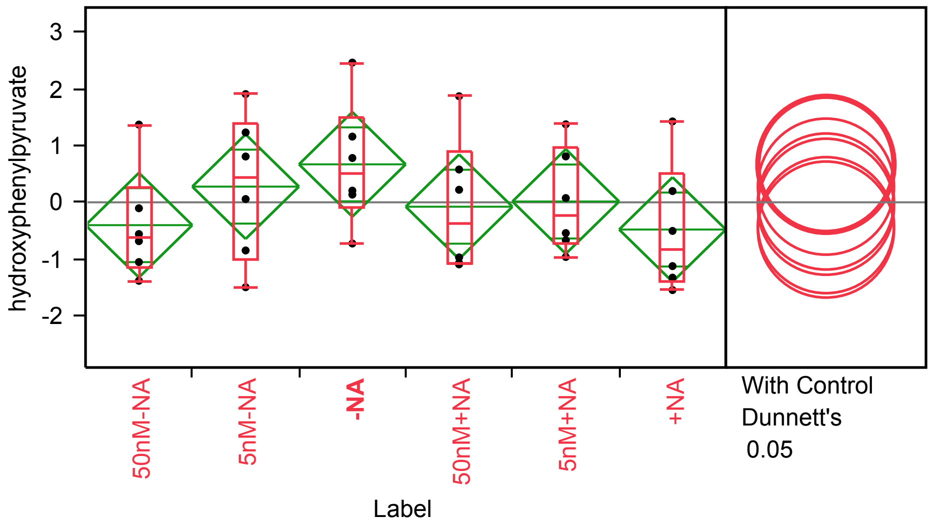


### Quantiles

| Level | Minimum | 10% | 25% | Median | 75% | 90% | Maximum |
|---|---|---|---|---|---|---|---|
| 50nM-NA | -1.37977 | -1.37977 | -1.13288 | -0.62036 | 0.263373 | 1.3661 | 1.3661 |
| 5nM-NA | -1.4927 | -1.4927 | -1.00815 | 0.431298 | 1.396975 | 1.90258 | 1.90258 |
| -NA | -0.72407 | -0.72407 | -0.0794 | 0.491917 | 1.480216 | 2.456141 | 2.456141 |
| 50nM+NA | -1.0906 | -1.0906 | -1.07258 | -0.37431 | 0.898592 | 1.86477 | 1.86477 |
| 5nM+NA | -0.96092 | -0.96092 | -0.73992 | -0.23312 | 0.94836 | 1.371255 | 1.371255 |
| +NA | -1.54279 | -1.54279 | -1.37922 | -0.8123 | 0.503996 | 1.421881 | 1.421881 |

### Oneway Anova

#### Summary of Fit

| | |
|---|---|
| Rsquare | 0.131286 |
| Adj Rsquare | -0.0135 |
| Root Mean Square Error | 1.102473 |
| Mean of Response | -1.2e-17 |
| Observations (or Sum Wgts) | 36 |

#### Analysis of Variance

| Source | DF | Sum of Squares | Mean Square | F Ratio | Prob > F |
|---|---|---|---|---|---|
| Label | 5 | 5.510603 | 1.10212 | 0.9068 | 0.4897 |
| Error | 30 | 36.463431 | 1.21545 | | |
| C. Total | 35 | 41.974034 | | | |

#### Means for Oneway Anova

| Level | Number | Mean | Std Error | Lower 95% | Upper 95% |
|---|---|---|---|---|---|
| 50nM-NA | 6 | -0.40153 | 0.45008 | -1.321 | 0.5177 |
| 5nM-NA | 6 | 0.27571 | 0.45008 | -0.643 | 1.1949 |
| -NA | 6 | 0.66772 | 0.45008 | -0.251 | 1.5869 |
| 50nM+NA | 6 | -0.07742 | 0.45008 | -0.997 | 0.8418 |
| 5nM+NA | 6 | 0.01421 | 0.45008 | -0.905 | 0.9334 |
| +NA | 6 | -0.47869 | 0.45008 | -1.398 | 0.4405 |

Std Error uses a pooled estimate of error variance

### Means Comparisons

#### Comparisons with a control using Dunnett's Method

## Oneway Analysis of hydroxyproline By Label

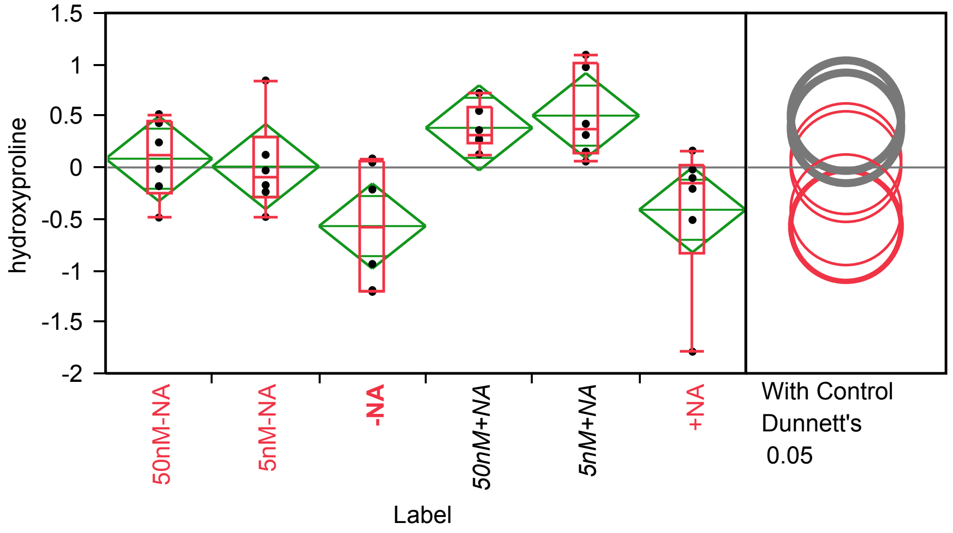


### Quantiles

| Level | Minimum | 10% | 25% | Median | 75% | 90% | Maximum |
|---|---|---|---|---|---|---|---|
| 50nM-NA | -0.48669 | -0.48669 | -0.25814 | 0.114666 | 0.45238 | 0.517859 | 0.517859 |
| 5nM-NA | -0.48039 | -0.48039 | -0.29806 | -0.10049 | 0.303252 | 0.844695 | 0.844695 |
| -NA | -1.208 | -1.208 | -1.19597 | -0.57618 | 0.057737 | 0.087113 | 0.087113 |
| 50nM+NA | 0.128504 | 0.128504 | 0.230966 | 0.320636 | 0.592397 | 0.723025 | 0.723025 |
| 5nM+NA | 0.057384 | 0.057384 | 0.129749 | 0.369825 | 1.004346 | 1.094039 | 1.094039 |
| +NA | -1.78979 | -1.78979 | -0.83009 | -0.15514 | 0.025868 | 0.163425 | 0.163425 |

### Oneway Anova

#### Summary of Fit

| | |
|---|---|
| Rsquare | 0.423957 |
| Adj Rsquare | 0.32795 |
| Root Mean Square Error | 0.495017 |
| Mean of Response | -8.3e-10 |
| Observations (or Sum Wgts) | 36 |

#### Analysis of Variance

| Source | DF | Sum of Squares | Mean Square | F Ratio | Prob > F |
|---|---|---|---|---|---|
| Label | 5 | 5.410399 | 1.08208 | 4.4159 | 0.0039 * |
| Error | 30 | 7.351266 | 0.24504 | | |
| C. Total | 35 | 12.761665 | | | |

#### Means for Oneway Anova

| Level | Number | Mean | Std Error | Lower 95% | Upper 95% |
|---|---|---|---|---|---|
| 50nM-NA | 6 | 0.08485 | 0.20209 | -0.3279 | 0.4976 |
| 5nM-NA | 6 | 0.00814 | 0.20209 | -0.4046 | 0.4209 |
| -NA | 6 | -0.56954 | 0.20209 | -0.9823 | -0.1568 |
| 50nM+NA | 6 | 0.38446 | 0.20209 | -0.0283 | 0.7972 |
| 5nM+NA | 6 | 0.50323 | 0.20209 | 0.0905 | 0.9160 |
| +NA | 6 | -0.41114 | 0.20209 | -0.8239 | 0.0016 |

Std Error uses a pooled estimate of error variance

### Means Comparisons

#### Comparisons with a control using Dunnett's Method

## Oneway Analysis of IDP By Label

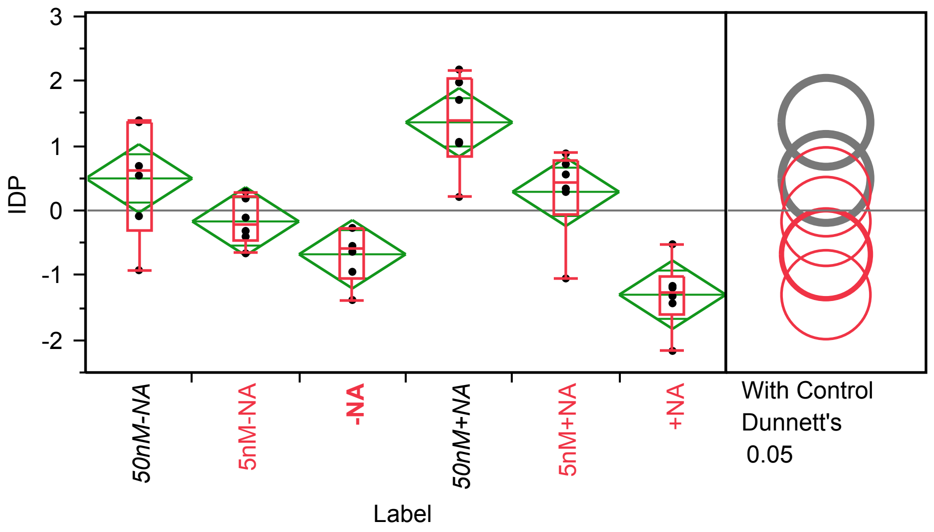


### Quantiles

| Level | Minimum | 10% | 25% | Median | 75% | 90% | Maximum |
|---|---|---|---|---|---|---|---|
| 50nM-NA | -0.92141 | -0.92141 | -0.29564 | 0.612922 | 1.369479 | 1.390958 | 1.390958 |
| 5nM-NA | -0.66069 | -0.66069 | -0.46703 | -0.2125 | 0.211969 | 0.289657 | 0.289657 |
| -NA | -1.38136 | -1.38136 | -1.055 | -0.59192 | -0.27226 | -0.26917 | -0.26917 |
| 50nM+NA | 0.209471 | 0.209471 | 0.830978 | 1.382597 | 2.026167 | 2.175191 | 2.175191 |
| 5nM+NA | -1.04806 | -1.04806 | -0.04719 | 0.447102 | 0.756666 | 0.88056 | 0.88056 |
| +NA | -2.16651 | -2.16651 | -1.61537 | -1.25689 | -1.00355 | -0.52098 | -0.52098 |

### Oneway Anova

#### Summary of Fit

| | |
|---|---|
| Rsquare | 0.685313 |
| Adj Rsquare | 0.632866 |
| Root Mean Square Error | 0.632314 |
| Mean of Response | 5.56e-10 |
| Observations (or Sum Wgts) | 36 |

#### Analysis of Variance

| Source | DF | Sum of Squares | Mean Square | F Ratio | Prob > F |
|---|---|---|---|---|---|
| Label | 5 | 26.121512 | 5.22430 | 13.0666 | <.0001 * |
| Error | 30 | 11.994645 | 0.39982 | | |
| C. Total | 35 | 38.116157 | | | |

#### Means for Oneway Anova

| Level | Number | Mean | Std Error | Lower 95% | Upper 95% |
|---|---|---|---|---|---|
| 50nM-NA | 6 | 0.4951 | 0.25814 | -0.032 | 1.022 |
| 5nM-NA | 6 | -0.1687 | 0.25814 | -0.696 | 0.358 |
| -NA | 6 | -0.6756 | 0.25814 | -1.203 | -0.148 |
| 50nM+NA | 6 | 1.3607 | 0.25814 | 0.834 | 1.888 |
| 5nM+NA | 6 | 0.2881 | 0.25814 | -0.239 | 0.815 |
| +NA | 6 | -1.2996 | 0.25814 | -1.827 | -0.772 |

Std Error uses a pooled estimate of error variance

### Means Comparisons

#### Comparisons with a control using Dunnett's Method

## Oneway Analysis of Imidazoleacetic acid By Label

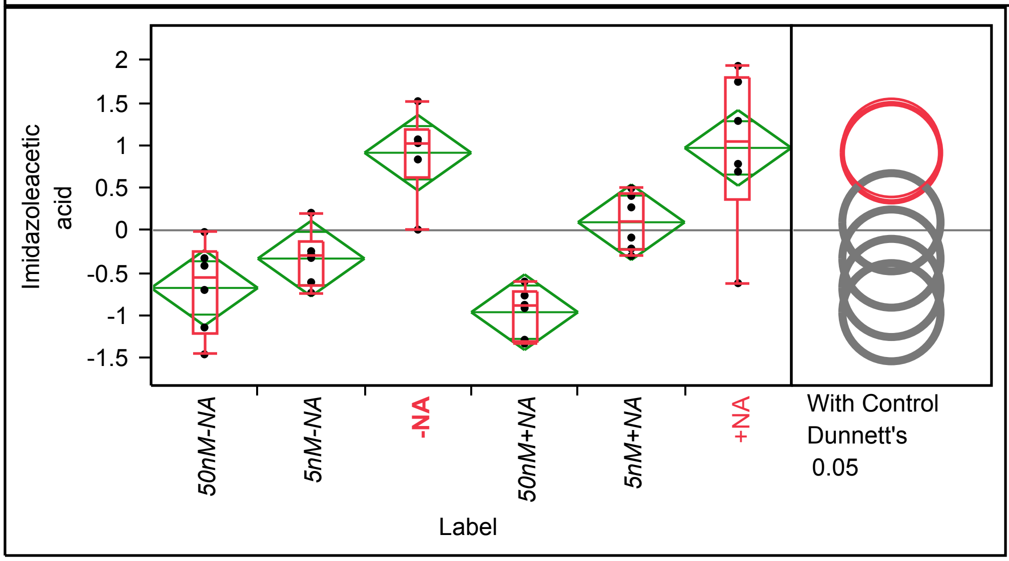


### Quantiles

| Level | Minimum | 10% | 25% | Median | 75% | 90% | Maximum |
|---|---|---|---|---|---|---|---|
| 50nM-NA | -1.45894 | -1.45894 | -1.22216 | -0.5593 | -0.25125 | -0.02059 | -0.02059 |
| 5nM-NA | -0.73543 | -0.73543 | -0.64176 | -0.30479 | -0.13315 | 0.205416 | 0.205416 |
| -NA | 0.007729 | 0.007729 | 0.62688 | 1.021149 | 1.180975 | 1.517922 | 1.517922 |
| 50nM+NA | -1.33171 | -1.33171 | -1.29904 | -0.89571 | -0.72635 | -0.6036 | -0.6036 |
| 5nM+NA | -0.30137 | -0.30137 | -0.23699 | 0.091949 | 0.426615 | 0.49406 | 0.49406 |
| +NA | -0.6221 | -0.6221 | 0.361357 | 1.033371 | 1.792756 | 1.932845 | 1.932845 |

### Oneway Anova

#### Summary of Fit

| | |
|---|---|
| Rsquare | 0.6983 |
| Adj Rsquare | 0.648017 |
| Root Mean Square Error | 0.532207 |
| Mean of Response | 2.47e-17 |
| Observations (or Sum Wgts) | 36 |

#### Analysis of Variance

| Source | DF | Sum of Squares | Mean Square | F Ratio | Prob > F |
|---|---|---|---|---|---|
| Label | 5 | 19.667547 | 3.93351 | 13.8873 | <.0001 * |
| Error | 30 | 8.497330 | 0.28324 | | |
| C. Total | 35 | 28.164878 | | | |

#### Means for Oneway Anova

| Level | Number | Mean | Std Error | Lower 95% | Upper 95% |
|---|---|---|---|---|---|
| 50nM-NA | 6 | -0.67825 | 0.21727 | -1.122 | -0.235 |
| 5nM-NA | 6 | -0.33269 | 0.21727 | -0.776 | 0.111 |
| -NA | 6 | 0.91165 | 0.21727 | 0.468 | 1.355 |
| 50nM+NA | 6 | -0.96369 | 0.21727 | -1.407 | -0.520 |
| 5nM+NA | 6 | 0.09420 | 0.21727 | -0.350 | 0.538 |
| +NA | 6 | 0.96879 | 0.21727 | 0.525 | 1.413 |

Std Error uses a pooled estimate of error variance

### Means Comparisons

#### Comparisons with a control using Dunnett's Method

## Oneway Analysis of IMP By Label

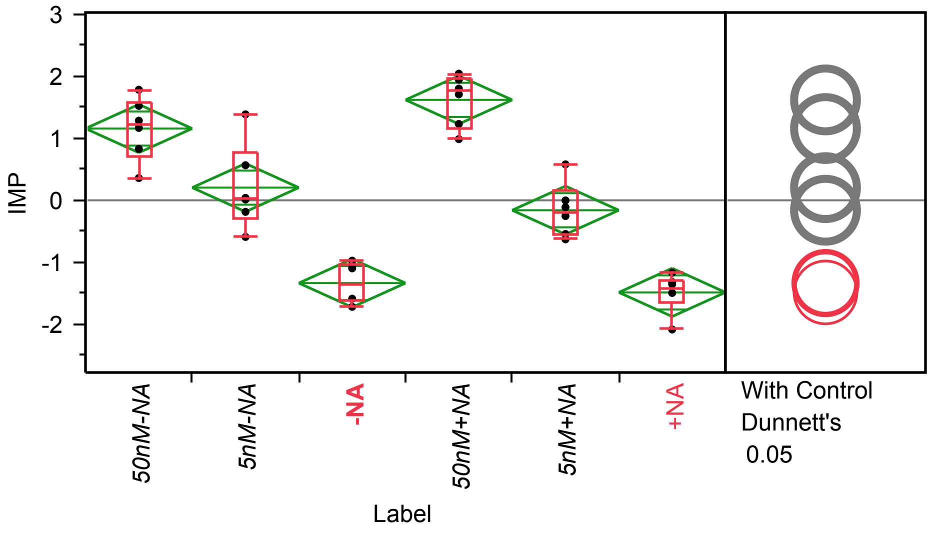


### Quantiles

| Level | Minimum | 10% | 25% | Median | 75% | 90% | Maximum |
|---|---|---|---|---|---|---|---|
| 50nM-NA | 0.358518 | 0.358518 | 0.710882 | 1.225074 | 1.587062 | 1.782003 | 1.782003 |
| 5nM-NA | -0.59035 | -0.59035 | -0.2877 | 0.026074 | 0.770412 | 1.385265 | 1.385265 |
| -NA | -1.71782 | -1.71782 | -1.62158 | -1.34181 | -1.02681 | -0.97379 | -0.97379 |
| 50nM+NA | 0.986201 | 0.986201 | 1.169468 | 1.754325 | 1.969526 | 2.041595 | 2.041595 |
| 5nM+NA | -0.62901 | -0.62901 | -0.56504 | -0.18277 | 0.143192 | 0.579272 | 0.579272 |
| +NA | -2.08297 | -2.08297 | -1.64121 | -1.42343 | -1.28024 | -1.16723 | -1.16723 |

### Oneway Anova

#### Summary of Fit

| | |
|---|---|
| Rsquare | 0.88031 |
| Adj Rsquare | 0.860361 |
| Root Mean Square Error | 0.466782 |
| Mean of Response | 4.93e-17 |
| Observations (or Sum Wgts) | 36 |

#### Analysis of Variance

| Source | DF | Sum of Squares | Mean Square | F Ratio | Prob > F |
|---|---|---|---|---|---|
| Label | 5 | 48.075701 | 9.61514 | 44.1294 | <.0001 * |
| Error | 30 | 6.536560 | 0.21789 | | |
| C. Total | 35 | 54.612261 | | | |

#### Means for Oneway Anova

| Level | Number | Mean | Std Error | Lower 95% | Upper 95% |
|---|---|---|---|---|---|
| 50nM-NA | 6 | 1.1568 | 0.19056 | 0.768 | 1.546 |
| 5nM-NA | 6 | 0.2043 | 0.19056 | -0.185 | 0.593 |
| -NA | 6 | -1.3349 | 0.19056 | -1.724 | -0.946 |
| 50nM+NA | 6 | 1.6188 | 0.19056 | 1.230 | 2.008 |
| 5nM+NA | 6 | -0.1602 | 0.19056 | -0.549 | 0.229 |
| +NA | 6 | -1.4848 | 0.19056 | -1.874 | -1.096 |

Std Error uses a pooled estimate of error variance

### Means Comparisons

#### Comparisons with a control using Dunnett's Method

## Oneway Analysis of indole By Label

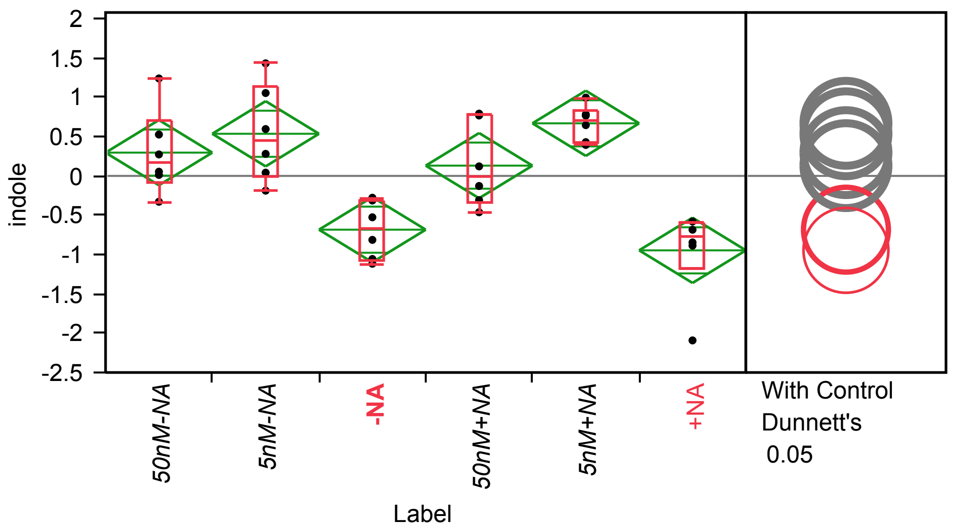


## Quantiles

| Level | Minimum | 10% | 25% | Median | 75% | 90% | Maximum |
|---|---|---|---|---|---|---|---|
| 50nM-NA | -0.32879 | -0.32879 | -0.07273 | 0.16321 | 0.704116 | 1.238955 | 1.238955 |
| 5nM-NA | -0.18749 | -0.18749 | -0.01613 | 0.437952 | 1.148042 | 1.430902 | 1.430902 |
| -NA | -1.11692 | -1.11692 | -1.07173 | -0.67006 | -0.30395 | -0.27771 | -0.27771 |
| 50nM+NA | -0.46206 | -0.46206 | -0.34666 | -0.00374 | 0.774324 | 0.79188 | 0.79188 |
| 5nM+NA | 0.393845 | 0.393845 | 0.420236 | 0.706154 | 0.83505 | 0.995396 | 0.995396 |
| +NA | -2.09193 | -2.09193 | -1.18785 | -0.7654 | -0.58796 | -0.57995 | -0.57995 |

## Oneway Anova

### Summary of Fit

| | |
|---|---|
| Rsquare | 0.640362 |
| Adj Rsquare | 0.580423 |
| Root Mean Square Error | 0.4974 |
| Mean of Response | -2.8e-10 |
| Observations (or Sum Wgts) | 36 |

### Analysis of Variance

| Source | DF | Sum of Squares | Mean Square | F Ratio | Prob > F |
|---|---|---|---|---|---|
| Label | 5 | 13.215795 | 2.64316 | 10.6835 | <.0001 * |
| Error | 30 | 7.422196 | 0.24741 | | |
| C. Total | 35 | 20.637992 | | | |

### Means for Oneway Anova

| Level | Number | Mean | Std Error | Lower 95% | Upper 95% |
|---|---|---|---|---|---|
| 50nM-NA | 6 | 0.29584 | 0.20306 | -0.119 | 0.711 |
| 5nM-NA | 6 | 0.53568 | 0.20306 | 0.121 | 0.950 |
| -NA | 6 | -0.68402 | 0.20306 | -1.099 | -0.269 |
| 50nM+NA | 6 | 0.13044 | 0.20306 | -0.284 | 0.545 |
| 5nM+NA | 6 | 0.66870 | 0.20306 | 0.254 | 1.083 |
| +NA | 6 | -0.94663 | 0.20306 | -1.361 | -0.532 |

Std Error uses a pooled estimate of error variance

## Means Comparisons

### Comparisons with a control using Dunnett's Method

## Oneway Analysis of Indole-3-carboxylic acid By Label

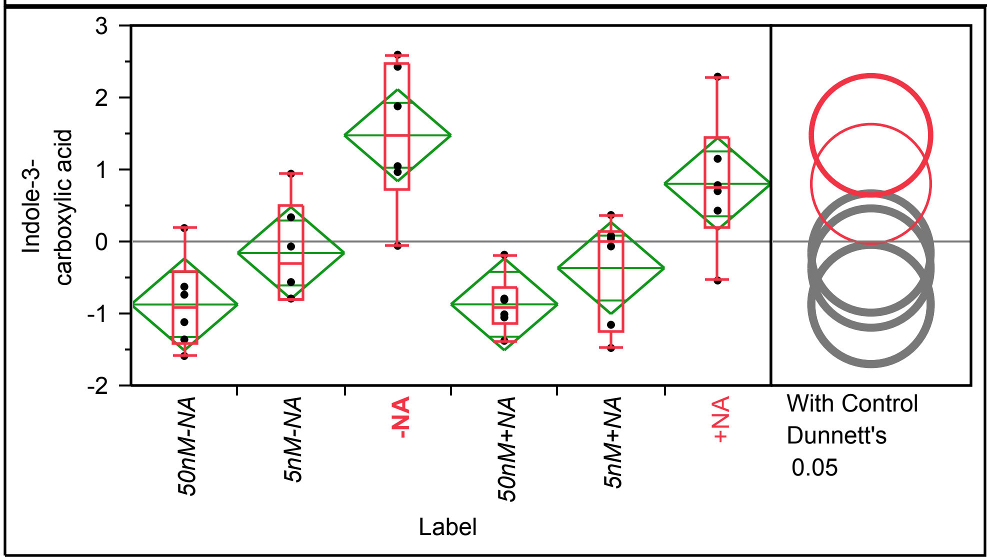


### Quantiles

| Level | Minimum | 10% | 25% | Median | 75% | 90% | Maximum |
|---|---|---|---|---|---|---|---|
| 50nM-NA | -1.59005 | -1.59005 | -1.41836 | -0.9294 | -0.42444 | 0.186262 | 0.186262 |
| 5nM-NA | -0.8063 | -0.8063 | -0.79612 | -0.31694 | 0.48718 | 0.940789 | 0.940789 |
| -NA | -0.06025 | -0.06025 | 0.708731 | 1.461897 | 2.46879 | 2.593926 | 2.593926 |
| 50nM+NA | -1.37947 | -1.37947 | -1.13604 | -0.91184 | -0.64088 | -0.18423 | -0.18423 |
| 5nM+NA | -1.47846 | -1.47846 | -1.23724 | -0.01243 | 0.152238 | 0.36722 | 0.36722 |
| +NA | -0.54149 | -0.54149 | 0.185936 | 0.740883 | 1.43369 | 2.288602 | 2.288602 |

### Oneway Anova

#### Summary of Fit

| | |
|---|---|
| Rsquare | 0.606813 |
| Adj Rsquare | 0.541282 |
| Root Mean Square Error | 0.764148 |
| Mean of Response | -7.4e-17 |
| Observations (or Sum Wgts) | 36 |

#### Analysis of Variance

| Source | DF | Sum of Squares | Mean Square | F Ratio | Prob > F |
|---|---|---|---|---|---|
| Label | 5 | 27.035334 | 5.40707 | 9.2599 | <.0001 * |
| Error | 30 | 17.517671 | 0.58392 | | |
| C. Total | 35 | 44.553004 | | | |

#### Means for Oneway Anova

| Level | Number | Mean | Std Error | Lower 95% | Upper 95% |
|---|---|---|---|---|---|
| 50nM-NA | 6 | -0.8753 | 0.31196 | -1.512 | -0.238 |
| 5nM-NA | 6 | -0.1594 | 0.31196 | -0.796 | 0.478 |
| -NA | 6 | 1.4749 | 0.31196 | 0.838 | 2.112 |
| 50nM+NA | 6 | -0.8726 | 0.31196 | -1.510 | -0.235 |
| 5nM+NA | 6 | -0.3687 | 0.31196 | -1.006 | 0.268 |
| +NA | 6 | 0.8010 | 0.31196 | 0.164 | 1.438 |

Std Error uses a pooled estimate of error variance

### Means Comparisons

#### Comparisons with a control using Dunnett's Method

## Oneway Analysis of Indoleacrylic acid By Label

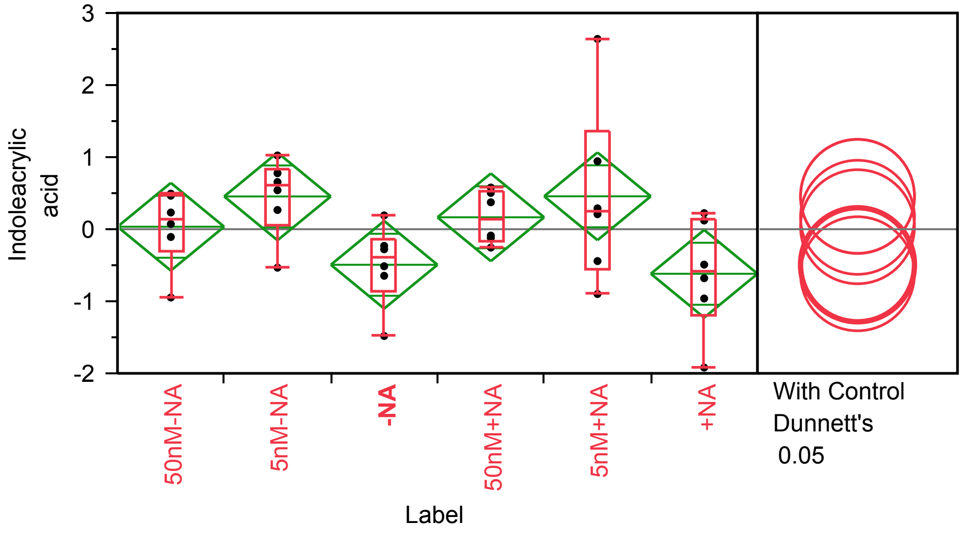


## Quantiles

| Level | Minimum | 10% | 25% | Median | 75% | 90% | Maximum |
|---|---|---|---|---|---|---|---|
| 50nM-NA | -0.94755 | -0.94755 | -0.31776 | 0.151871 | 0.471961 | 0.493348 | 0.493348 |
| 5nM-NA | -0.53357 | -0.53357 | 0.066643 | 0.597815 | 0.838781 | 1.02236 | 1.02236 |
| -NA | -1.48144 | -1.48144 | -0.85493 | -0.39639 | -0.12549 | 0.191641 | 0.191641 |
| 50nM+NA | -0.25472 | -0.25472 | -0.15338 | 0.142522 | 0.522269 | 0.578258 | 0.578258 |
| 5nM+NA | -0.89884 | -0.89884 | -0.55624 | 0.248948 | 1.367532 | 2.640111 | 2.640111 |
| +NA | -1.92004 | -1.92004 | -1.20119 | -0.58561 | 0.146679 | 0.223247 | 0.223247 |

## Oneway Anova

### Summary of Fit

| | |
|---|---|
| Rsquare | 0.286649 |
| Adj Rsquare | 0.167757 |
| Root Mean Square Error | 0.729551 |
| Mean of Response | 8.33e-10 |
| Observations (or Sum Wgts) | 36 |

### Analysis of Variance

| Source | DF | Sum of Squares | Mean Square | F Ratio | Prob > F |
|---|---|---|---|---|---|
| Label | 5 | 6.416220 | 1.28324 | 2.4110 | 0.0595 |
| Error | 30 | 15.967340 | 0.53224 | | |
| C. Total | 35 | 22.383560 | | | |

### Means for Oneway Anova

| Level | Number | Mean | Std Error | Lower 95% | Upper 95% |
|---|---|---|---|---|---|
| 50nM-NA | 6 | 0.03442 | 0.29784 | -0.574 | 0.643 |
| 5nM-NA | 6 | 0.45479 | 0.29784 | -0.153 | 1.063 |
| -NA | 6 | -0.49331 | 0.29784 | -1.102 | 0.115 |
| 50nM+NA | 6 | 0.16543 | 0.29784 | -0.443 | 0.774 |
| 5nM+NA | 6 | 0.45674 | 0.29784 | -0.152 | 1.065 |
| +NA | 6 | -0.61807 | 0.29784 | -1.226 | -0.0098 |

Std Error uses a pooled estimate of error variance

## Means Comparisons

### Comparisons with a control using Dunnett's Method

## Oneway Analysis of inosine By Label

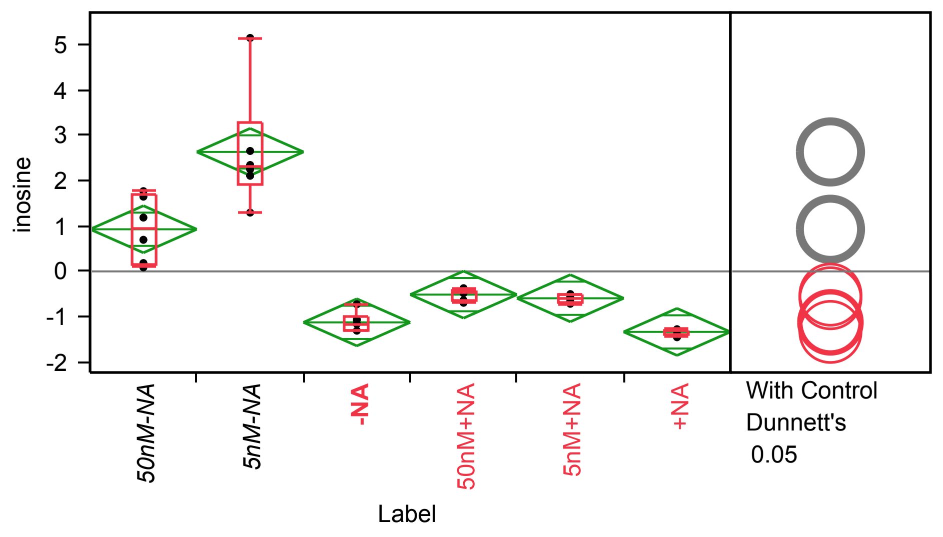


### Quantiles

| Level | Minimum | 10% | 25% | Median | 75% | 90% | Maximum |
|---|---|---|---|---|---|---|---|
| 50nM-NA | 0.089975 | 0.089975 | 0.159784 | 0.941052 | 1.677092 | 1.768554 | 1.768554 |
| 5nM-NA | 1.295879 | 1.295879 | 1.904558 | 2.297163 | 3.276728 | 5.144731 | 5.144731 |
| -NA | -1.31656 | -1.31656 | -1.30899 | -1.16456 | -0.97927 | -0.72385 | -0.72385 |
| 50nM+NA | -0.69116 | -0.69116 | -0.65075 | -0.48025 | -0.40168 | -0.3709 | -0.3709 |
| 5nM+NA | -0.71345 | -0.71345 | -0.67755 | -0.58643 | -0.50117 | -0.50005 | -0.50005 |
| +NA | -1.45108 | -1.45108 | -1.40732 | -1.29812 | -1.28352 | -1.27444 | -1.27444 |

### Oneway Anova

#### Summary of Fit

| | |
|---|---|
| Rsquare | 0.855672 |
| Adj Rsquare | 0.831617 |
| Root Mean Square Error | 0.6214 |
| Mean of Response | -5.6e-10 |
| Observations (or Sum Wgts) | 36 |

#### Analysis of Variance

| Source | DF | Sum of Squares | Mean Square | F Ratio | Prob > F |
|---|---|---|---|---|---|
| Label | 5 | 68.678164 | 13.7356 | 35.5719 | <.0001 * |
| Error | 30 | 11.584123 | 0.3861 | | |
| C. Total | 35 | 80.262287 | | | |

#### Means for Oneway Anova

| Level | Number | Mean | Std Error | Lower 95% | Upper 95% |
|---|---|---|---|---|---|
| 50nM-NA | 6 | 0.9284 | 0.25369 | 0.410 | 1.446 |
| 5nM-NA | 6 | 2.6327 | 0.25369 | 2.115 | 3.151 |
| -NA | 6 | -1.1234 | 0.25369 | -1.641 | -0.605 |
| 50nM+NA | 6 | -0.5120 | 0.25369 | -1.030 | 0.00613 |
| 5nM+NA | 6 | -0.5923 | 0.25369 | -1.110 | -0.074 |
| +NA | 6 | -1.3335 | 0.25369 | -1.852 | -0.815 |

Std Error uses a pooled estimate of error variance

### Means Comparisons

#### Comparisons with a control using Dunnett's Method

## Oneway Analysis of isocitrate By Label

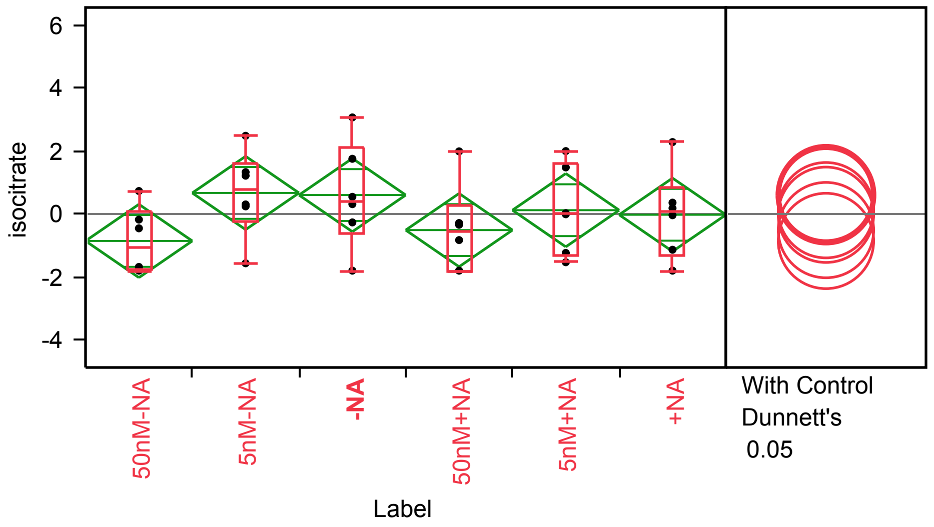


## Quantiles

| Level | Minimum | 10% | 25% | Median | 75% | 90% | Maximum |
|---|---|---|---|---|---|---|---|
| 50nM-NA | -1.79541 | -1.79541 | -1.76125 | -1.06529 | 0.04909 | 0.728292 | 0.728292 |
| 5nM-NA | -1.56152 | -1.56152 | -0.20895 | 0.758671 | 1.614567 | 2.477935 | 2.477935 |
| -NA | -1.79535 | -1.79535 | -0.64844 | 0.427554 | 2.077753 | 3.061227 | 3.061227 |
| 50nM+NA | -1.79546 | -1.79546 | -1.79543 | -0.58449 | 0.281865 | 1.984964 | 1.984964 |
| 5nM+NA | -1.52868 | -1.52868 | -1.30746 | 0.008809 | 1.605758 | 1.99351 | 1.99351 |
| +NA | -1.79537 | -1.79537 | -1.29893 | 0.071702 | 0.839196 | 2.27944 | 2.27944 |

## Oneway Anova

### Summary of Fit

| | |
|---|---|
| Rsquare | 0.157242 |
| Adj Rsquare | 0.016783 |
| Root Mean Square Error | 1.393264 |
| Mean of Response | 6.17e-18 |
| Observations (or Sum Wgts) | 36 |

### Analysis of Variance

| Source | DF | Sum of Squares | Mean Square | F Ratio | Prob > F |
|---|---|---|---|---|---|
| Label | 5 | 10.865631 | 2.17313 | 1.1195 | 0.3712 |
| Error | 30 | 58.235506 | 1.94118 | | |
| C. Total | 35 | 69.101138 | | | |

### Means for Oneway Anova

| Level | Number | Mean | Std Error | Lower 95% | Upper 95% |
|---|---|---|---|---|---|
| 50nM-NA | 6 | -0.85414 | 0.56880 | -2.016 | 0.3075 |
| 5nM-NA | 6 | 0.66707 | 0.56880 | -0.495 | 1.8287 |
| -NA | 6 | 0.60080 | 0.56880 | -0.561 | 1.7624 |
| 50nM+NA | 6 | -0.51012 | 0.56880 | -1.672 | 0.6515 |
| 5nM+NA | 6 | 0.12087 | 0.56880 | -1.041 | 1.2825 |
| +NA | 6 | -0.02448 | 0.56880 | -1.186 | 1.1372 |

Std Error uses a pooled estimate of error variance

## Means Comparisons

### Comparisons with a control using Dunnett's Method

## Oneway Analysis of Isoleucine By Label

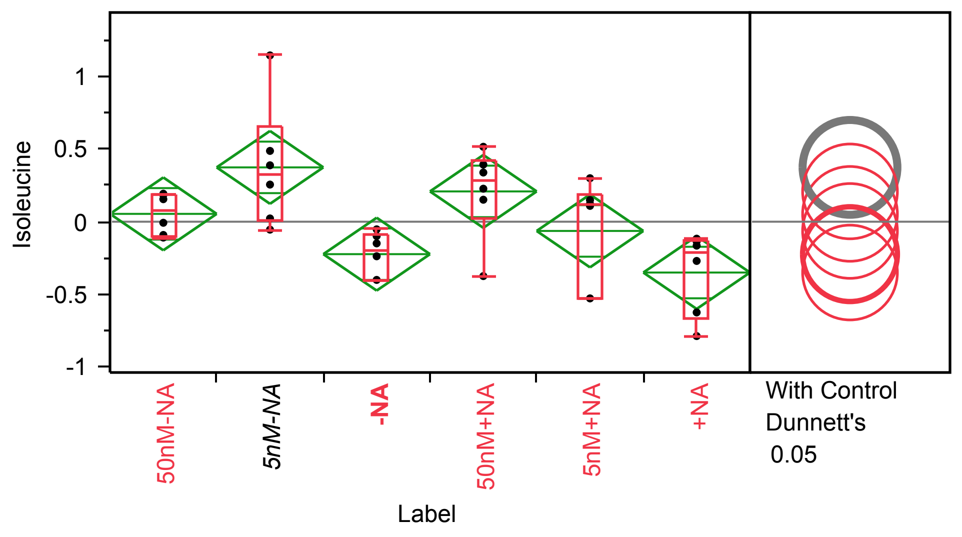


### Quantiles

| Level | Minimum | 10% | 25% | Median | 75% | 90% | Maximum |
|---|---|---|---|---|---|---|---|
| 50nM-NA | -0.10923 | -0.10923 | -0.09585 | 0.073898 | 0.18632 | 0.192968 | 0.192968 |
| 5nM-NA | -0.05452 | -0.05452 | 0.003261 | 0.322243 | 0.652205 | 1.145669 | 1.145669 |
| -NA | -0.40111 | -0.40111 | -0.40097 | -0.19363 | -0.0873 | -0.05259 | -0.05259 |
| 50nM+NA | -0.3746 | -0.3746 | 0.019938 | 0.283109 | 0.424121 | 0.516092 | 0.516092 |
| 5nM+NA | -0.53391 | -0.53391 | -0.52965 | 0.116114 | 0.186606 | 0.299607 | 0.299607 |
| +NA | -0.78774 | -0.78774 | -0.66558 | -0.21755 | -0.13116 | -0.11706 | -0.11706 |

### Oneway Anova

#### Summary of Fit

| | |
|---|---|
| Rsquare | 0.445011 |
| Adj Rsquare | 0.352513 |
| Root Mean Square Error | 0.300969 |
| Mean of Response | 5.56e-11 |
| Observations (or Sum Wgts) | 36 |

#### Analysis of Variance

| Source | DF | Sum of Squares | Mean Square | F Ratio | Prob > F |
|---|---|---|---|---|---|
| Label | 5 | 2.1789743 | 0.435795 | 4.8110 | 0.0024 * |
| Error | 30 | 2.7174760 | 0.090583 | | |
| C. Total | 35 | 4.8964503 | | | |

#### Means for Oneway Anova

| Level | Number | Mean | Std Error | Lower 95% | Upper 95% |
|---|---|---|---|---|---|
| 50nM-NA | 6 | 0.05404 | 0.12287 | -0.1969 | 0.3050 |
| 5nM-NA | 6 | 0.37431 | 0.12287 | 0.1234 | 0.6252 |
| -NA | 6 | -0.22346 | 0.12287 | -0.4744 | 0.0275 |
| 50nM+NA | 6 | 0.20877 | 0.12287 | -0.0422 | 0.4597 |
| 5nM+NA | 6 | -0.06356 | 0.12287 | -0.3145 | 0.1874 |
| +NA | 6 | -0.35010 | 0.12287 | -0.6010 | -0.0992 |

Std Error uses a pooled estimate of error variance

### Means Comparisons

#### Comparisons with a control using Dunnett's Method

## Oneway Analysis of Ketohexanoic acid By Label

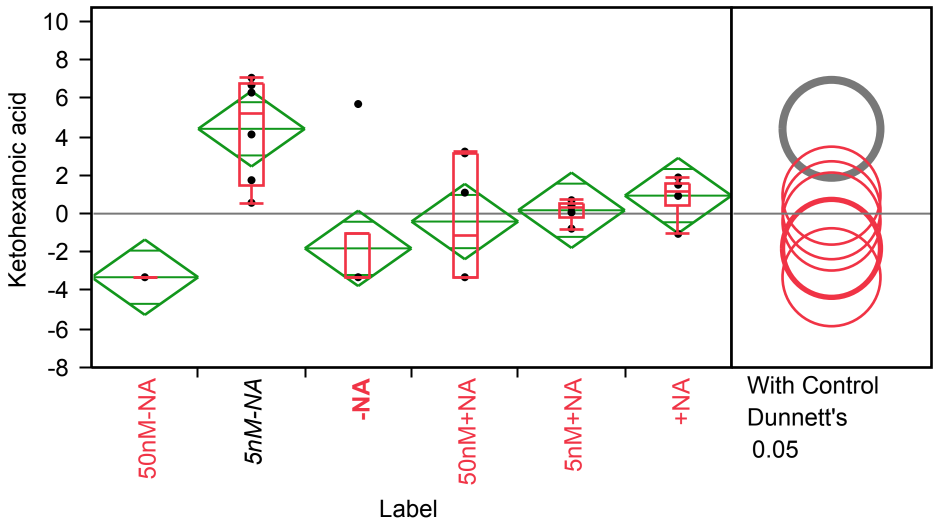


## Quantiles

| Level | Minimum | 10% | 25% | Median | 75% | 90% | Maximum |
|---|---|---|---|---|---|---|---|
| 50nM-NA | -3.31088 | -3.31088 | -3.31087 | -3.31086 | -3.31083 | -3.3108 | -3.3108 |
| 5nM-NA | 0.564491 | 0.564491 | 1.447323 | 5.212808 | 6.788121 | 7.074387 | 7.074387 |
| -NA | -3.31083 | -3.31083 | -3.31083 | -3.31081 | -1.05633 | 5.70705 | 5.70705 |
| 50nM+NA | -3.31087 | -3.31087 | -3.31086 | -1.10883 | 3.162229 | 3.230753 | 3.230753 |
| 5nM+NA | -0.78138 | -0.78138 | -0.14957 | 0.315604 | 0.509474 | 0.699221 | 0.699221 |
| +NA | -1.05285 | -1.05285 | 0.418991 | 1.189194 | 1.600948 | 1.884804 | 1.884804 |

## Oneway Anova

### Summary of Fit

| | |
|---|---|
| Rsquare | 0.557537 |
| Adj Rsquare | 0.483793 |
| Root Mean Square Error | 2.35071 |
| Mean of Response | -2.8e-10 |
| Observations (or Sum Wgts) | 36 |

### Analysis of Variance

| Source | DF | Sum of Squares | Mean Square | F Ratio | Prob > F |
|---|---|---|---|---|---|
| Label | 5 | 208.88898 | 41.7778 | 7.5604 | 0.0001 * |
| Error | 30 | 165.77509 | 5.5258 | | |
| C. Total | 35 | 374.66407 | | | |

### Means for Oneway Anova

| Level | Number | Mean | Std Error | Lower 95% | Upper 95% |
|---|---|---|---|---|---|
| 50nM-NA | 6 | -3.3108 | 0.95967 | -5.271 | -1.351 |
| 5nM-NA | 6 | 4.4165 | 0.95967 | 2.457 | 6.376 |
| -NA | 6 | -1.8078 | 0.95967 | -3.768 | 0.152 |
| 50nM+NA | 6 | -0.4115 | 0.95967 | -2.371 | 1.548 |
| 5nM+NA | 6 | 0.1760 | 0.95967 | -1.784 | 2.136 |
| +NA | 6 | 0.9377 | 0.95967 | -1.022 | 2.898 |

Std Error uses a pooled estimate of error variance

## Means Comparisons

### Comparisons with a control using Dunnett's Method

## Oneway Analysis of Ketoleucine By Label

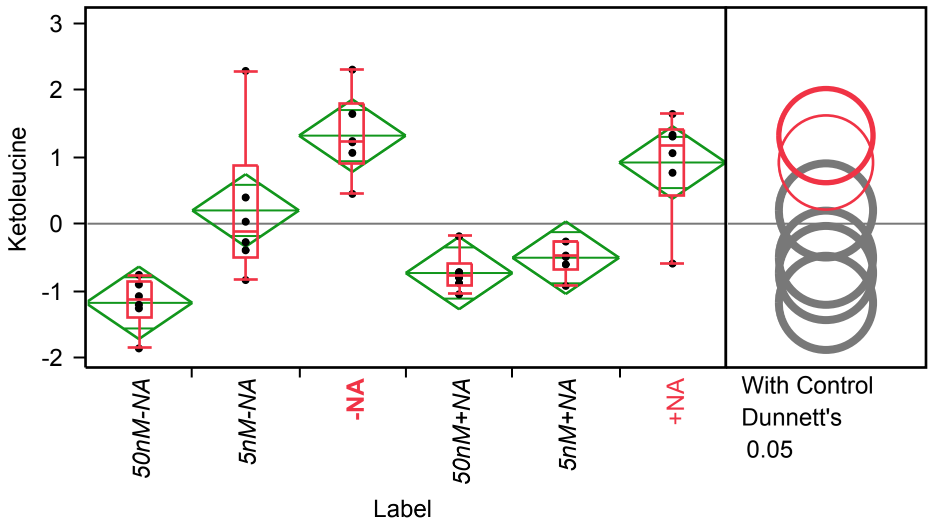


### Quantiles

| Level | Minimum | 10% | 25% | Median | 75% | 90% | Maximum |
|---|---|---|---|---|---|---|---|
| 50nM-NA | -1.86031 | -1.86031 | -1.41235 | -1.14529 | -0.87167 | -0.76368 | -0.76368 |
| 5nM-NA | -0.84057 | -0.84057 | -0.50767 | -0.1228 | 0.862674 | 2.278405 | 2.278405 |
| -NA | 0.444412 | 0.444412 | 0.903585 | 1.223028 | 1.802126 | 2.29753 | 2.29753 |
| 50nM+NA | -1.05376 | -1.05376 | -0.92963 | -0.78133 | -0.58831 | -0.18813 | -0.18813 |
| 5nM+NA | -0.9278 | -0.9278 | -0.68808 | -0.48552 | -0.27085 | -0.26656 | -0.26656 |
| +NA | -0.59487 | -0.59487 | 0.420958 | 1.176126 | 1.402737 | 1.63563 | 1.63563 |

### Oneway Anova

#### Summary of Fit

| | |
|---|---|
| Rsquare | 0.695655 |
| Adj Rsquare | 0.644931 |
| Root Mean Square Error | 0.647504 |
| Mean of Response | 5.56e-10 |
| Observations (or Sum Wgts) | 36 |

#### Analysis of Variance

| Source | DF | Sum of Squares | Mean Square | F Ratio | Prob > F |
|---|---|---|---|---|---|
| Label | 5 | 28.749723 | 5.74994 | 13.7145 | <.0001 * |
| Error | 30 | 12.577825 | 0.41926 | | |
| C. Total | 35 | 41.327548 | | | |

#### Means for Oneway Anova

| Level | Number | Mean | Std Error | Lower 95% | Upper 95% |
|---|---|---|---|---|---|
| 50nM-NA | 6 | -1.1809 | 0.26434 | -1.721 | -0.641 |
| 5nM-NA | 6 | 0.1977 | 0.26434 | -0.342 | 0.738 |
| -NA | 6 | 1.3136 | 0.26434 | 0.774 | 1.853 |
| 50nM+NA | 6 | -0.7358 | 0.26434 | -1.276 | -0.196 |
| 5nM+NA | 6 | -0.5076 | 0.26434 | -1.048 | 0.032 |
| +NA | 6 | 0.9129 | 0.26434 | 0.373 | 1.453 |

Std Error uses a pooled estimate of error variance

### Means Comparisons

#### Comparisons with a control using Dunnett's Method

## Oneway Analysis of Kynurenic acid By Label

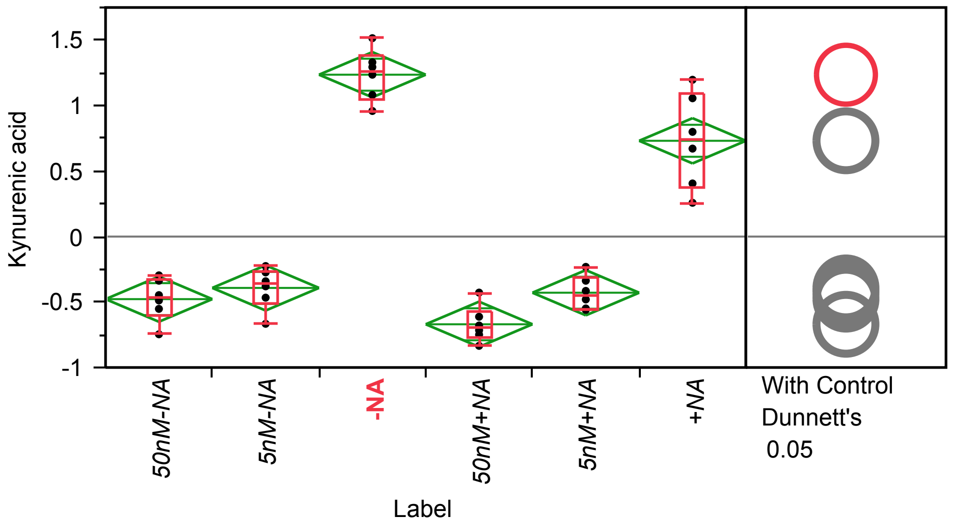


### Quantiles

| Level | Minimum | 10% | 25% | Median | 75% | 90% | Maximum |
|---|---|---|---|---|---|---|---|
| 50nM-NA | -0.74606 | -0.74606 | -0.60066 | -0.46759 | -0.32679 | -0.29614 | -0.29614 |
| 5nM-NA | -0.66612 | -0.66612 | -0.5164 | -0.36065 | -0.26146 | -0.22521 | -0.22521 |
| -NA | 0.959528 | 0.959528 | 1.051276 | 1.265735 | 1.377325 | 1.515564 | 1.515564 |
| 50nM+NA | -0.83444 | -0.83444 | -0.77063 | -0.69739 | -0.56618 | -0.42792 | -0.42792 |
| 5nM+NA | -0.55979 | -0.55979 | -0.55167 | -0.44753 | -0.31079 | -0.23138 | -0.23138 |
| +NA | 0.258324 | 0.258324 | 0.368951 | 0.736048 | 1.092051 | 1.19775 | 1.19775 |

### Oneway Anova

#### Summary of Fit

| | |
|---|---|
| Rsquare | 0.934822 |
| Adj Rsquare | 0.923959 |
| Root Mean Square Error | 0.207214 |
| Mean of Response | -5.56e-9 |
| Observations (or Sum Wgts) | 36 |

#### Analysis of Variance

| Source | DF | Sum of Squares | Mean Square | F Ratio | Prob > F |
|---|---|---|---|---|---|
| Label | 5 | 18.475181 | 3.69504 | 86.0555 | <.0001 * |
| Error | 30 | 1.288134 | 0.04294 | | |
| C. Total | 35 | 19.763315 | | | |

#### Means for Oneway Anova

| Level | Number | Mean | Std Error | Lower 95% | Upper 95% |
|---|---|---|---|---|---|
| 50nM-NA | 6 | -0.4778 | 0.08459 | -0.651 | -0.305 |
| 5nM-NA | 6 | -0.3921 | 0.08459 | -0.565 | -0.219 |
| -NA | 6 | 1.2366 | 0.08459 | 1.064 | 1.409 |
| 50nM+NA | 6 | -0.6698 | 0.08459 | -0.843 | -0.497 |
| 5nM+NA | 6 | -0.4287 | 0.08459 | -0.602 | -0.256 |
| +NA | 6 | 0.7318 | 0.08459 | 0.559 | 0.905 |

Std Error uses a pooled estimate of error variance

### Means Comparisons

#### Comparisons with a control using Dunnett's Method

## Oneway Analysis of Kynurenine By Label

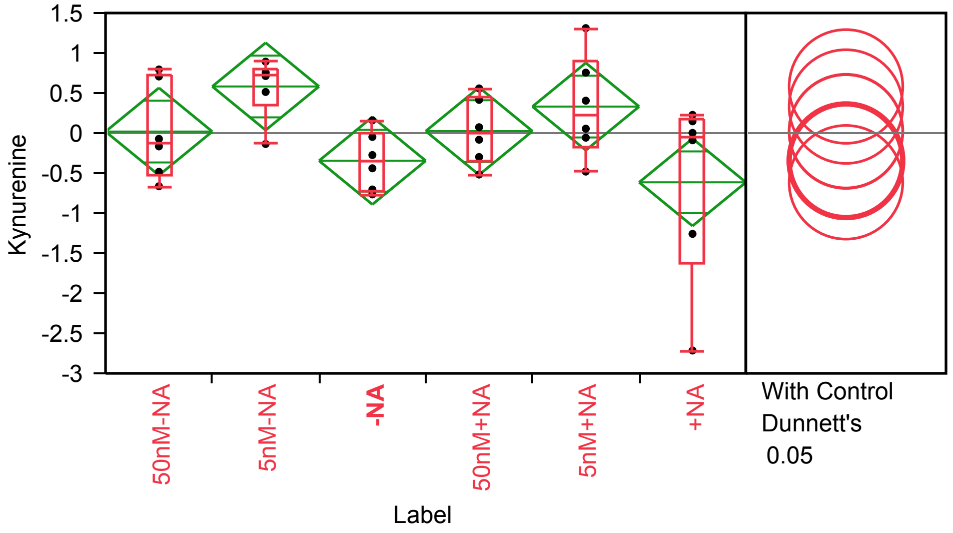


## Quantiles

| Level | Minimum | 10% | 25% | Median | 75% | 90% | Maximum |
|---|---|---|---|---|---|---|---|
| 50nM-NA | -0.66348 | -0.66348 | -0.52865 | -0.11869 | 0.729267 | 0.794855 | 0.794855 |
| 5nM-NA | -0.13547 | -0.13547 | 0.351598 | 0.733668 | 0.791098 | 0.892751 | 0.892751 |
| -NA | -0.76462 | -0.76462 | -0.71928 | -0.35639 | 0.004359 | 0.159724 | 0.159724 |
| 50nM+NA | -0.51677 | -0.51677 | -0.35272 | -0.00437 | 0.450968 | 0.558133 | 0.558133 |
| 5nM+NA | -0.48012 | -0.48012 | -0.1628 | 0.230267 | 0.894295 | 1.311209 | 1.311209 |
| +NA | -2.71471 | -2.71471 | -1.62237 | -0.04313 | 0.167627 | 0.227676 | 0.227676 |

## Oneway Anova

### Summary of Fit

| | |
|---|---|
| Rsquare | 0.306567 |
| Adj Rsquare | 0.190994 |
| Root Mean Square Error | 0.654319 |
| Mean of Response | -2.8e-11 |
| Observations (or Sum Wgts) | 36 |

### Analysis of Variance

| Source | DF | Sum of Squares | Mean Square | F Ratio | Prob > F |
|---|---|---|---|---|---|
| Label | 5 | 5.678322 | 1.13566 | 2.6526 | 0.0422 * |
| Error | 30 | 12.843994 | 0.42813 | | |
| C. Total | 35 | 18.522316 | | | |

### Means for Oneway Anova

| Level | Number | Mean | Std Error | Lower 95% | Upper 95% |
|---|---|---|---|---|---|
| 50nM-NA | 6 | 0.01961 | 0.26712 | -0.526 | 0.565 |
| 5nM-NA | 6 | 0.58263 | 0.26712 | 0.037 | 1.128 |
| -NA | 6 | -0.34488 | 0.26712 | -0.890 | 0.201 |
| 50nM+NA | 6 | 0.02497 | 0.26712 | -0.521 | 0.571 |
| 5nM+NA | 6 | 0.33165 | 0.26712 | -0.214 | 0.877 |
| +NA | 6 | -0.61399 | 0.26712 | -1.160 | -0.068 |

Std Error uses a pooled estimate of error variance

## Means Comparisons

### Comparisons with a control using Dunnett's Method

## Oneway Analysis of Lactic acid By Label

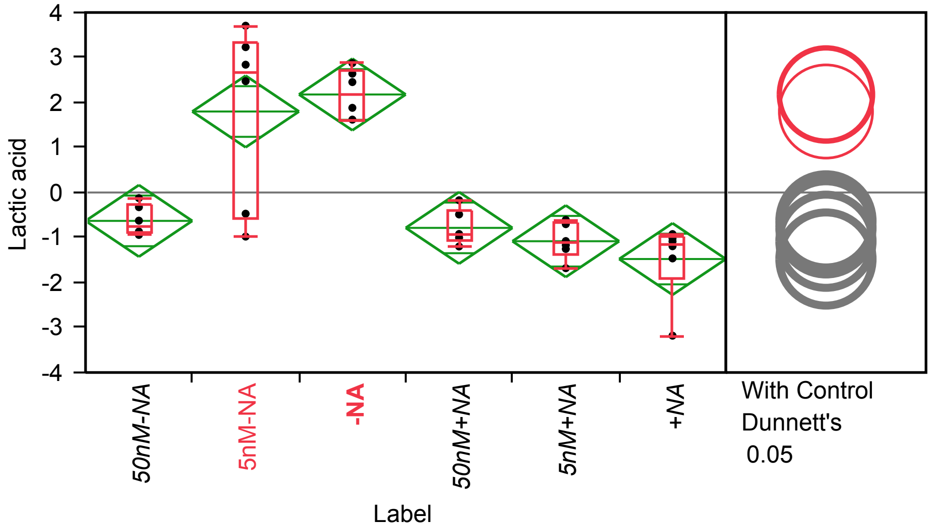


## Quantiles

| Level | Minimum | 10% | 25% | Median | 75% | 90% | Maximum |
|---|---|---|---|---|---|---|---|
| 50nM-NA | -0.94238 | -0.94238 | -0.90881 | -0.74627 | -0.27559 | -0.12474 | -0.12474 |
| 5nM-NA | -0.97952 | -0.97952 | -0.59848 | 2.65681 | 3.349276 | 3.70579 | 3.70579 |
| -NA | 1.597497 | 1.597497 | 1.613839 | 2.16759 | 2.699438 | 2.874866 | 2.874866 |
| 50nM+NA | -1.19934 | -1.19934 | -1.05313 | -0.92655 | -0.40875 | -0.1724 | -0.1724 |
| 5nM+NA | -1.68133 | -1.68133 | -1.36056 | -1.12475 | -0.67986 | -0.60879 | -0.60879 |
| +NA | -3.18171 | -3.18171 | -1.89387 | -1.14038 | -0.99483 | -0.92462 | -0.92462 |

## Oneway Anova

### Summary of Fit

| | |
|---|---|
| Rsquare | 0.730681 |
| Adj Rsquare | 0.685794 |
| Root Mean Square Error | 0.954397 |
| Mean of Response | 5.556e-9 |
| Observations (or Sum Wgts) | 36 |

### Analysis of Variance

| Source | DF | Sum of Squares | Mean Square | F Ratio | Prob > F |
|---|---|---|---|---|---|
| Label | 5 | 74.13783 | 14.8276 | 16.2784 | <.0001 * |
| Error | 30 | 27.32624 | 0.9109 | | |
| C. Total | 35 | 101.46407 | | | |

### Means for Oneway Anova

| Level | Number | Mean | Std Error | Lower 95% | Upper 95% |
|---|---|---|---|---|---|
| 50nM-NA | 6 | -0.6305 | 0.38963 | -1.426 | 0.165 |
| 5nM-NA | 6 | 1.7998 | 0.38963 | 1.004 | 2.596 |
| -NA | 6 | 2.1780 | 0.38963 | 1.382 | 2.974 |
| 50nM+NA | 6 | -0.7861 | 0.38963 | -1.582 | 0.0096 |
| 5nM+NA | 6 | -1.0828 | 0.38963 | -1.879 | -0.287 |
| +NA | 6 | -1.4783 | 0.38963 | -2.274 | -0.683 |

Std Error uses a pooled estimate of error variance

## Means Comparisons

### Comparisons with a control using Dunnett's Method

## Oneway Analysis of leucine By Label

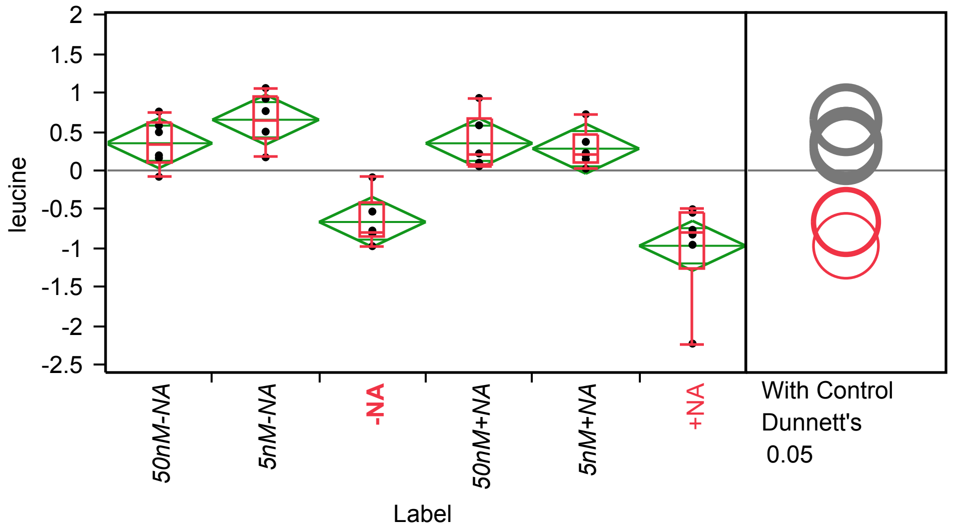


### Quantiles

| Level | Minimum | 10% | 25% | Median | 75% | 90% | Maximum |
|---|---|---|---|---|---|---|---|
| 50nM-NA | -0.08093 | -0.08093 | 0.099766 | 0.343746 | 0.622299 | 0.75803 | 0.75803 |
| 5nM-NA | 0.169542 | 0.169542 | 0.416817 | 0.631775 | 0.95772 | 1.060596 | 1.060596 |
| -NA | -0.97438 | -0.97438 | -0.8544 | -0.78701 | -0.41874 | -0.08815 | -0.08815 |
| 50nM+NA | 0.053712 | 0.053712 | 0.087902 | 0.215498 | 0.669281 | 0.932744 | 0.932744 |
| 5nM+NA | 0.0235 | 0.0235 | 0.115132 | 0.210656 | 0.457085 | 0.723368 | 0.723368 |
| +NA | -2.22873 | -2.22873 | -1.27482 | -0.79487 | -0.53282 | -0.49913 | -0.49913 |

### Oneway Anova

#### Summary of Fit

| | |
|---|---|
| Rsquare | 0.741919 |
| Adj Rsquare | 0.698905 |
| Root Mean Square Error | 0.384954 |
| Mean of Response | 8.33e-10 |
| Observations (or Sum Wgts) | 36 |

#### Analysis of Variance

| Source | DF | Sum of Squares | Mean Square | F Ratio | Prob > F |
|---|---|---|---|---|---|
| Label | 5 | 12.780239 | 2.55605 | 17.2485 | <.0001 * |
| Error | 30 | 4.445690 | 0.14819 | | |
| C. Total | 35 | 17.225929 | | | |

#### Means for Oneway Anova

| Level | Number | Mean | Std Error | Lower 95% | Upper 95% |
|---|---|---|---|---|---|
| 50nM-NA | 6 | 0.35027 | 0.15716 | 0.029 | 0.6712 |
| 5nM-NA | 6 | 0.65273 | 0.15716 | 0.332 | 0.9737 |
| -NA | 6 | -0.66332 | 0.15716 | -0.984 | -0.3424 |
| 50nM+NA | 6 | 0.34970 | 0.15716 | 0.029 | 0.6707 |
| 5nM+NA | 6 | 0.28036 | 0.15716 | -0.041 | 0.6013 |
| +NA | 6 | -0.96975 | 0.15716 | -1.291 | -0.6488 |

Std Error uses a pooled estimate of error variance

### Means Comparisons

#### Comparisons with a control using Dunnett's Method

## Oneway Analysis of lysine By Label

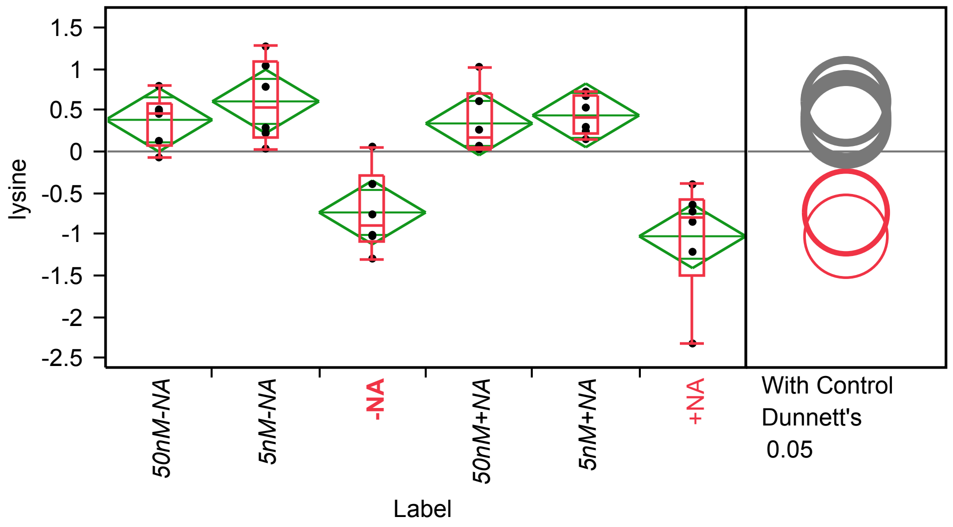


## Quantiles

| Level | Minimum | 10% | 25% | Median | 75% | 90% | Maximum |
|---|---|---|---|---|---|---|---|
| 50nM-NA | -0.07187 | -0.07187 | 0.078038 | 0.470336 | 0.579936 | 0.794278 | 0.794278 |
| 5nM-NA | 0.033944 | 0.033944 | 0.174815 | 0.536157 | 1.097618 | 1.272508 | 1.272508 |
| -NA | -1.29786 | -1.29786 | -1.09323 | -0.88679 | -0.28198 | 0.057806 | 0.057806 |
| 50nM+NA | 0.027511 | 0.027511 | 0.039236 | 0.166039 | 0.713873 | 1.024971 | 1.024971 |
| 5nM+NA | 0.149263 | 0.149263 | 0.220725 | 0.414745 | 0.685891 | 0.726219 | 0.726219 |
| +NA | -2.32634 | -2.32634 | -1.4937 | -0.78982 | -0.5834 | -0.39808 | -0.39808 |

## Oneway Anova

### Summary of Fit

| | |
|---|---|
| Rsquare | 0.693886 |
| Adj Rsquare | 0.642867 |
| Root Mean Square Error | 0.46239 |
| Mean of Response | 2.78e-10 |
| Observations (or Sum Wgts) | 36 |

### Analysis of Variance

| Source | DF | Sum of Squares | Mean Square | F Ratio | Prob > F |
|---|---|---|---|---|---|
| Label | 5 | 14.539286 | 2.90786 | 13.6005 | <.0001 * |
| Error | 30 | 6.414142 | 0.21380 | | |
| C. Total | 35 | 20.953428 | | | |

### Means for Oneway Anova

| Level | Number | Mean | Std Error | Lower 95% | Upper 95% |
|---|---|---|---|---|---|
| 50nM-NA | 6 | 0.3833 | 0.18877 | -0.0023 | 0.7688 |
| 5nM-NA | 6 | 0.6066 | 0.18877 | 0.221 | 0.9922 |
| -NA | 6 | -0.7390 | 0.18877 | -1.125 | -0.3535 |
| 50nM+NA | 6 | 0.3396 | 0.18877 | -0.046 | 0.7252 |
| 5nM+NA | 6 | 0.4370 | 0.18877 | 0.051 | 0.8225 |
| +NA | 6 | -1.0276 | 0.18877 | -1.413 | -0.6420 |

Std Error uses a pooled estimate of error variance

## Means Comparisons

### Comparisons with a control using Dunnett's Method

## Oneway Analysis of LysoPC(20:4) By Label

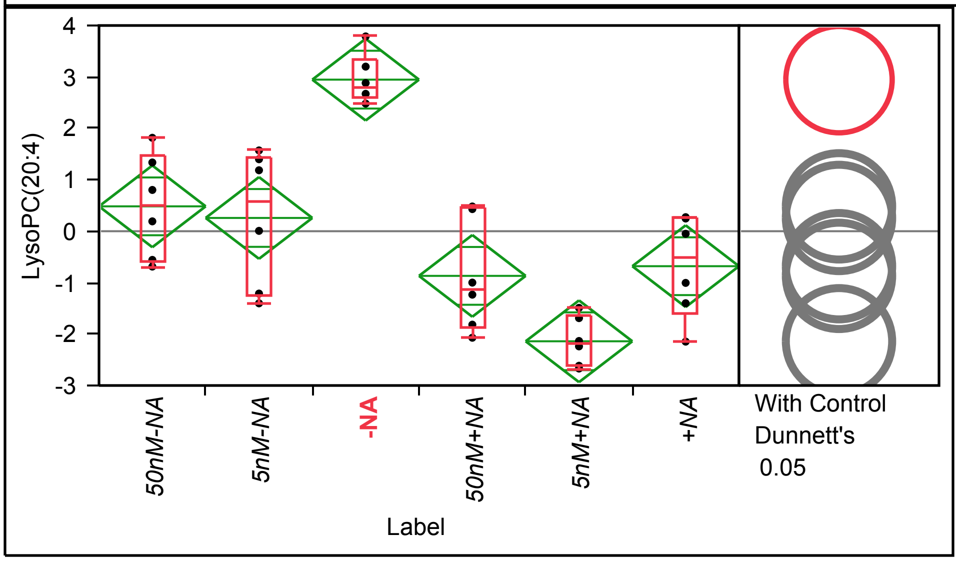


### Quantiles

| Level | Minimum | 10% | 25% | Median | 75% | 90% | Maximum |
|---|---|---|---|---|---|---|---|
| 50nM-NA | -0.68757 | -0.68757 | -0.59036 | 0.497069 | 1.456597 | 1.816381 | 1.816381 |
| 5nM-NA | -1.40219 | -1.40219 | -1.26004 | 0.596048 | 1.442515 | 1.570914 | 1.570914 |
| -NA | 2.481484 | 2.481484 | 2.615831 | 2.776543 | 3.347178 | 3.787462 | 3.787462 |
| 50nM+NA | -2.07107 | -2.07107 | -1.88071 | -1.1166 | 0.442889 | 0.480621 | 0.480621 |
| 5nM+NA | -2.67018 | -2.67018 | -2.62995 | -2.18703 | -1.63978 | -1.48993 | -1.48993 |
| +NA | -2.14682 | -2.14682 | -1.58668 | -0.52754 | 0.253664 | 0.273091 | 0.273091 |

### Oneway Anova

#### Summary of Fit

| | |
|---|---|
| Rsquare | 0.764959 |
| Adj Rsquare | 0.725785 |
| Root Mean Square Error | 0.953128 |
| Mean of Response | 5.56e-11 |
| Observations (or Sum Wgts) | 36 |

#### Analysis of Variance

| Source | DF | Sum of Squares | Mean Square | F Ratio | Prob > F |
|---|---|---|---|---|---|
| Label | 5 | 88.69876 | 17.7398 | 19.5274 | <.0001 * |
| Error | 30 | 27.25357 | 0.9085 | | |
| C. Total | 35 | 115.95233 | | | |

#### Means for Oneway Anova

| Level | Number | Mean | Std Error | Lower 95% | Upper 95% |
|---|---|---|---|---|---|
| 50nM-NA | 6 | 0.4836 | 0.38911 | -0.311 | 1.278 |
| 5nM-NA | 6 | 0.2580 | 0.38911 | -0.537 | 1.053 |
| -NA | 6 | 2.9472 | 0.38911 | 2.153 | 3.742 |
| 50nM+NA | 6 | -0.8684 | 0.38911 | -1.663 | -0.074 |
| 5nM+NA | 6 | -2.1401 | 0.38911 | -2.935 | -1.345 |
| +NA | 6 | -0.6803 | 0.38911 | -1.475 | 0.114 |

Std Error uses a pooled estimate of error variance

### Means Comparisons

#### Comparisons with a control using Dunnett's Method

## Oneway Analysis of Maleic acid By Label

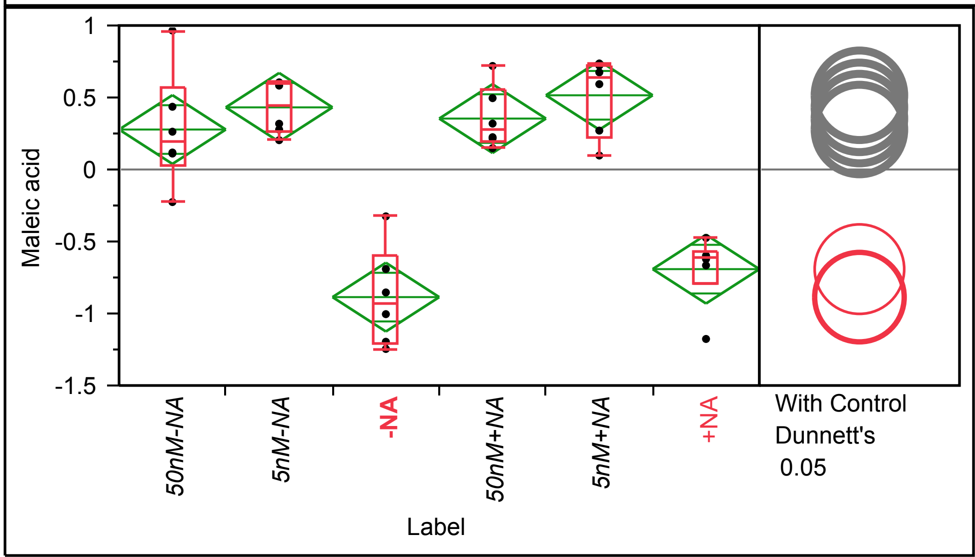


## Quantiles

| Level | Minimum | 10% | 25% | Median | 75% | 90% | Maximum |
|---|---|---|---|---|---|---|---|
| 50nM-NA | -0.22611 | -0.22611 | 0.026592 | 0.190076 | 0.567952 | 0.964944 | 0.964944 |
| 5nM-NA | 0.203693 | 0.203693 | 0.258875 | 0.450246 | 0.60323 | 0.605204 | 0.605204 |
| -NA | -1.2462 | -1.2462 | -1.20877 | -0.92917 | -0.59964 | -0.32553 | -0.32553 |
| 50nM+NA | 0.148406 | 0.148406 | 0.199026 | 0.271596 | 0.55138 | 0.718834 | 0.718834 |
| 5nM+NA | 0.0972 | 0.0972 | 0.226793 | 0.633571 | 0.72539 | 0.736205 | 0.736205 |
| +NA | -1.17682 | -1.17682 | -0.79355 | -0.61764 | -0.5676 | -0.47519 | -0.47519 |

## Oneway Anova

### Summary of Fit

| | |
|---|---|
| Rsquare | 0.823787 |
| Adj Rsquare | 0.794418 |
| Root Mean Square Error | 0.28646 |
| Mean of Response | -2.5e-17 |
| Observations (or Sum Wgts) | 36 |

### Analysis of Variance

| Source | DF | Sum of Squares | Mean Square | F Ratio | Prob > F |
|---|---|---|---|---|---|
| Label | 5 | 11.508686 | 2.30174 | 28.0497 | <.0001 * |
| Error | 30 | 2.461781 | 0.08206 | | |
| C. Total | 35 | 13.970467 | | | |

### Means for Oneway Anova

| Level | Number | Mean | Std Error | Lower 95% | Upper 95% |
|---|---|---|---|---|---|
| 50nM-NA | 6 | 0.27757 | 0.11695 | 0.039 | 0.5164 |
| 5nM-NA | 6 | 0.43154 | 0.11695 | 0.193 | 0.6704 |
| -NA | 6 | -0.88623 | 0.11695 | -1.125 | -0.6474 |
| 50nM+NA | 6 | 0.35365 | 0.11695 | 0.115 | 0.5925 |
| 5nM+NA | 6 | 0.51539 | 0.11695 | 0.277 | 0.7542 |
| +NA | 6 | -0.69192 | 0.11695 | -0.931 | -0.4531 |

Std Error uses a pooled estimate of error variance

## Means Comparisons

### Comparisons with a control using Dunnett's Method

## Oneway Analysis of Malic acid By Label

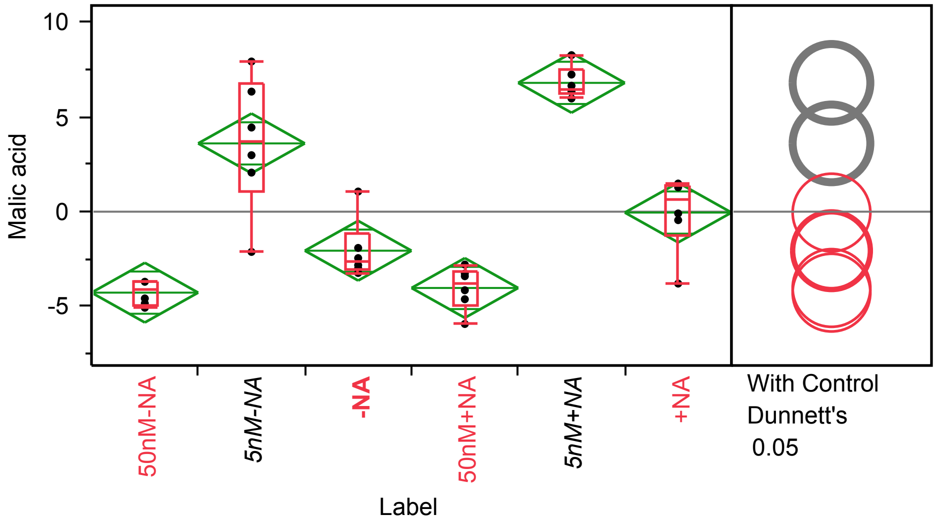


## Quantiles

| Level | Minimum | 10% | 25% | Median | 75% | 90% | Maximum |
|---|---|---|---|---|---|---|---|
| 50nM-NA | -5.06722 | -5.06722 | -4.89809 | -4.14658 | -3.71156 | -3.69727 | -3.69727 |
| 5nM-NA | -2.12111 | -2.12111 | 1.016244 | 3.709513 | 6.729322 | 7.919709 | 7.919709 |
| -NA | -3.23211 | -3.23211 | -3.05362 | -2.63486 | -1.16868 | 1.062083 | 1.062083 |
| 50nM+NA | -5.92247 | -5.92247 | -4.94361 | -3.77361 | -3.14453 | -2.78929 | -2.78929 |
| 5nM+NA | 5.971429 | 5.971429 | 6.236444 | 6.489706 | 7.489553 | 8.256018 | 8.256018 |
| +NA | -3.79074 | -3.79074 | -1.28163 | 0.595585 | 1.334503 | 1.483029 | 1.483029 |

## Oneway Anova

### Summary of Fit

| | |
|---|---|
| Rsquare | 0.845715 |
| Adj Rsquare | 0.820001 |
| Root Mean Square Error | 1.888874 |
| Mean of Response | 2.78e-10 |
| Observations (or Sum Wgts) | 36 |

### Analysis of Variance

| Source | DF | Sum of Squares | Mean Square | F Ratio | Prob > F |
|---|---|---|---|---|---|
| Label | 5 | 586.71671 | 117.343 | 32.8891 | <.0001 * |
| Error | 30 | 107.03537 | 3.568 | | |
| C. Total | 35 | 693.75208 | | | |

### Means for Oneway Anova

| Level | Number | Mean | Std Error | Lower 95% | Upper 95% |
|---|---|---|---|---|---|
| 50nM-NA | 6 | -4.2693 | 0.77113 | -5.844 | -2.694 |
| 5nM-NA | 6 | 3.6020 | 0.77113 | 2.027 | 5.177 |
| -NA | 6 | -2.0577 | 0.77113 | -3.633 | -0.483 |
| 50nM+NA | 6 | -4.0232 | 0.77113 | -5.598 | -2.448 |
| 5nM+NA | 6 | 6.7943 | 0.77113 | 5.219 | 8.369 |
| +NA | 6 | -0.0461 | 0.77113 | -1.621 | 1.529 |

Std Error uses a pooled estimate of error variance

## Means Comparisons

### Comparisons with a control using Dunnett's Method

## Oneway Analysis of Mannose By Label

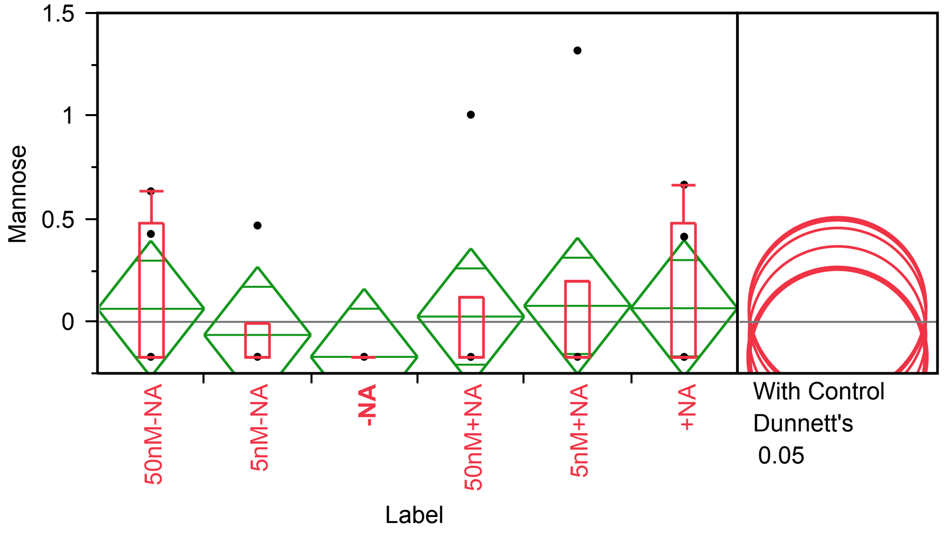


### Quantiles

| Level | Minimum | 10% | 25% | Median | 75% | 90% | Maximum |
|---|---|---|---|---|---|---|---|
| 50nM-NA | -0.17041 | -0.17041 | -0.17026 | -0.1699 | 0.478864 | 0.634123 | 0.634123 |
| 5nM-NA | -0.17003 | -0.17003 | -0.16993 | -0.16979 | -0.0102 | 0.468043 | 0.468043 |
| -NA | -0.17013 | -0.17013 | -0.17003 | -0.16991 | -0.1698 | -0.16963 | -0.16963 |
| 50nM+NA | -0.17039 | -0.17039 | -0.17031 | -0.17019 | 0.123957 | 1.006217 | 1.006217 |
| 5nM+NA | -0.17051 | -0.17051 | -0.17037 | -0.17011 | 0.202082 | 1.317954 | 1.317954 |
| +NA | -0.17192 | -0.17192 | -0.17054 | -0.17004 | 0.47675 | 0.666096 | 0.666096 |

### Oneway Anova

#### Summary of Fit

| | |
|---|---|
| Rsquare | 0.057579 |
| Adj Rsquare | -0.09949 |
| Root Mean Square Error | 0.3966 |
| Mean of Response | 2.778e-9 |
| Observations (or Sum Wgts) | 36 |

#### Analysis of Variance

| Source | DF | Sum of Squares | Mean Square | F Ratio | Prob > F |
|---|---|---|---|---|---|
| Label | 5 | 0.2882985 | 0.057660 | 0.3666 | 0.8674 |
| Error | 30 | 4.7187391 | 0.157291 | | |
| C. Total | 35 | 5.0070376 | | | |

#### Means for Oneway Anova

| Level | Number | Mean | Std Error | Lower 95% | Upper 95% |
|---|---|---|---|---|---|
| 50nM-NA | 6 | 0.06347 | 0.16191 | -0.2672 | 0.39414 |
| 5nM-NA | 6 | -0.06351 | 0.16191 | -0.3942 | 0.26715 |
| -NA | 6 | -0.16991 | 0.16191 | -0.5006 | 0.16076 |
| 50nM+NA | 6 | 0.02584 | 0.16191 | -0.3048 | 0.35650 |
| 5nM+NA | 6 | 0.07784 | 0.16191 | -0.2528 | 0.40851 |
| +NA | 6 | 0.06628 | 0.16191 | -0.2644 | 0.39694 |

Std Error uses a pooled estimate of error variance

### Means Comparisons

#### Comparisons with a control using Dunnett's Method

## Oneway Analysis of methionine By Label

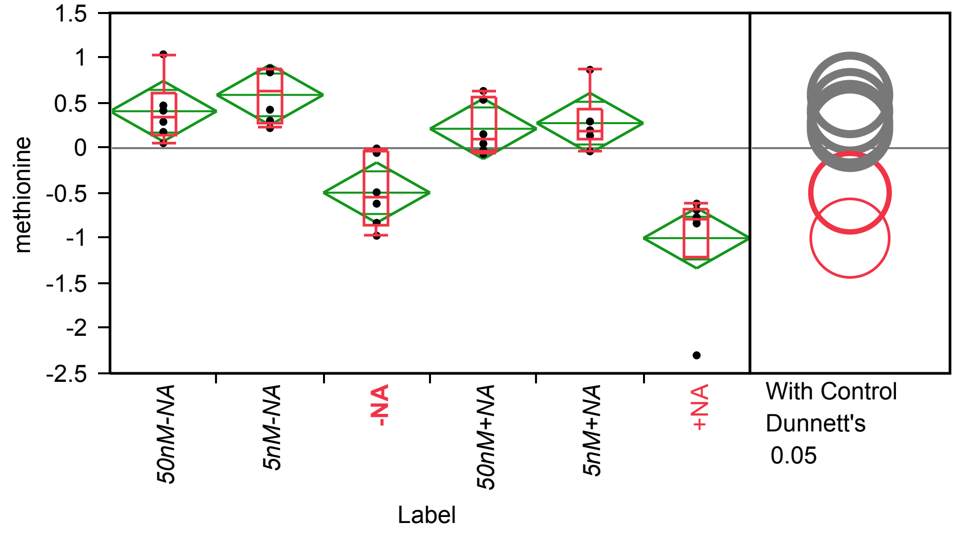


### Quantiles

| Level | Minimum | 10% | 25% | Median | 75% | 90% | Maximum |
|---|---|---|---|---|---|---|---|
| 50nM-NA | 0.055737 | 0.055737 | 0.14861 | 0.353738 | 0.615322 | 1.041237 | 1.041237 |
| 5nM-NA | 0.223117 | 0.223117 | 0.284309 | 0.633722 | 0.87051 | 0.885188 | 0.885188 |
| -NA | -0.97198 | -0.97198 | -0.86514 | -0.55337 | -0.04063 | -0.00402 | -0.00402 |
| 50nM+NA | -0.06443 | -0.06443 | -0.03228 | 0.102113 | 0.56145 | 0.634411 | 0.634411 |
| 5nM+NA | -0.03542 | -0.03542 | 0.095448 | 0.198481 | 0.439079 | 0.869414 | 0.869414 |
| +NA | -2.30008 | -2.30008 | -1.20174 | -0.77879 | -0.66739 | -0.61597 | -0.61597 |

### Oneway Anova

#### Summary of Fit

| | |
|---|---|
| Rsquare | 0.699934 |
| Adj Rsquare | 0.649923 |
| Root Mean Square Error | 0.401752 |
| Mean of Response | 5.56e-11 |
| Observations (or Sum Wgts) | 36 |

#### Analysis of Variance

| Source | DF | Sum of Squares | Mean Square | F Ratio | Prob > F |
|---|---|---|---|---|---|
| Label | 5 | 11.294746 | 2.25895 | 13.9956 | <.0001 * |
| Error | 30 | 4.842135 | 0.16140 | | |
| C. Total | 35 | 16.136881 | | | |

#### Means for Oneway Anova

| Level | Number | Mean | Std Error | Lower 95% | Upper 95% |
|---|---|---|---|---|---|
| 50nM-NA | 6 | 0.40956 | 0.16401 | 0.075 | 0.7445 |
| 5nM-NA | 6 | 0.59101 | 0.16401 | 0.256 | 0.9260 |
| -NA | 6 | -0.49418 | 0.16401 | -0.829 | -0.1592 |
| 50nM+NA | 6 | 0.21496 | 0.16401 | -0.120 | 0.5499 |
| 5nM+NA | 6 | 0.27761 | 0.16401 | -0.057 | 0.6126 |
| +NA | 6 | -0.99896 | 0.16401 | -1.334 | -0.6640 |

Std Error uses a pooled estimate of error variance

### Means Comparisons

#### Comparisons with a control using Dunnett's Method

## Oneway Analysis of Methionine sulfoxide By Label

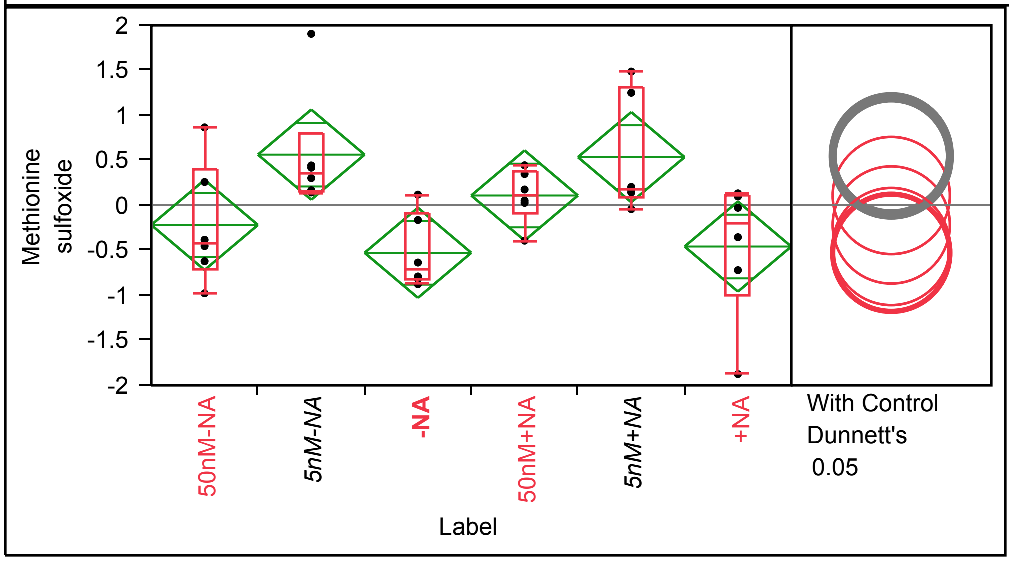


## Quantiles

| Level | Minimum | 10% | 25% | Median | 75% | 90% | Maximum |
|---|---|---|---|---|---|---|---|
| 50nM-NA | -0.97783 | -0.97783 | -0.71068 | -0.41877 | 0.410022 | 0.865178 | 0.865178 |
| 5nM-NA | 0.142539 | 0.142539 | 0.160503 | 0.359625 | 0.80791 | 1.904708 | 1.904708 |
| -NA | -0.87712 | -0.87712 | -0.83225 | -0.71403 | -0.09353 | 0.117917 | 0.117917 |
| 50nM+NA | -0.39329 | -0.39329 | -0.07846 | 0.113 | 0.371247 | 0.444102 | 0.444102 |
| 5nM+NA | -0.04287 | -0.04287 | 0.096199 | 0.186472 | 1.308613 | 1.485223 | 1.485223 |
| +NA | -1.87567 | -1.87567 | -1.01012 | -0.19157 | 0.105969 | 0.132121 | 0.132121 |

## Oneway Anova

### Summary of Fit

| | |
|---|---|
| Rsquare | 0.389818 |
| Adj Rsquare | 0.28812 |
| Root Mean Square Error | 0.600289 |
| Mean of Response | 8.33e-10 |
| Observations (or Sum Wgts) | 36 |

### Analysis of Variance

| Source | DF | Sum of Squares | Mean Square | F Ratio | Prob > F |
|---|---|---|---|---|---|
| Label | 5 | 6.906267 | 1.38125 | 3.8331 | 0.0084 * |
| Error | 30 | 10.810398 | 0.36035 | | |
| C. Total | 35 | 17.716665 | | | |

### Means for Oneway Anova

| Level | Number | Mean | Std Error | Lower 95% | Upper 95% |
|---|---|---|---|---|---|
| 50nM-NA | 6 | -0.21892 | 0.24507 | -0.719 | 0.282 |
| 5nM-NA | 6 | 0.56255 | 0.24507 | 0.062 | 1.063 |
| -NA | 6 | -0.52810 | 0.24507 | -1.029 | -0.028 |
| 50nM+NA | 6 | 0.10837 | 0.24507 | -0.392 | 0.609 |
| 5nM+NA | 6 | 0.53460 | 0.24507 | 0.034 | 1.035 |
| +NA | 6 | -0.45851 | 0.24507 | -0.959 | 0.042 |

Std Error uses a pooled estimate of error variance

## Means Comparisons

### Comparisons with a control using Dunnett's Method

## Oneway Analysis of Methylcysteine By Label

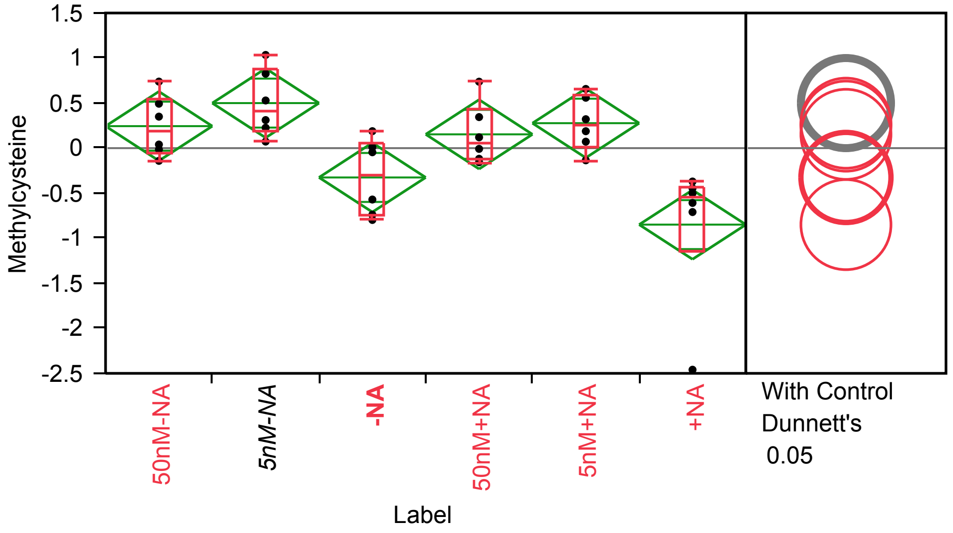


### Quantiles

| Level | Minimum | 10% | 25% | Median | 75% | 90% | Maximum |
|---|---|---|---|---|---|---|---|
| 50nM-NA | -0.14084 | -0.14084 | -0.04686 | 0.19482 | 0.554505 | 0.73839 | 0.73839 |
| 5nM-NA | 0.072946 | 0.072946 | 0.187991 | 0.420278 | 0.877954 | 1.034341 | 1.034341 |
| -NA | -0.79718 | -0.79718 | -0.75333 | -0.30801 | 0.05539 | 0.188193 | 0.188193 |
| 50nM+NA | -0.15676 | -0.15676 | -0.12791 | 0.055993 | 0.442851 | 0.73727 | 0.73727 |
| 5nM+NA | -0.13498 | -0.13498 | 0.020095 | 0.254518 | 0.583903 | 0.657002 | 0.657002 |
| +NA | -2.45986 | -2.45986 | -1.14701 | -0.55439 | -0.42633 | -0.37065 | -0.37065 |

### Oneway Anova

#### Summary of Fit

| | |
|---|---|
| Rsquare | 0.537806 |
| Adj Rsquare | 0.460773 |
| Root Mean Square Error | 0.460995 |
| Mean of Response | -8.3e-11 |
| Observations (or Sum Wgts) | 36 |

#### Analysis of Variance

| Source | DF | Sum of Squares | Mean Square | F Ratio | Prob > F |
|---|---|---|---|---|---|
| Label | 5 | 7.418486 | 1.48370 | 6.9816 | 0.0002 * |
| Error | 30 | 6.375503 | 0.21252 | | |
| C. Total | 35 | 13.793989 | | | |

#### Means for Oneway Anova

| Level | Number | Mean | Std Error | Lower 95% | Upper 95% |
|---|---|---|---|---|---|
| 50nM-NA | 6 | 0.24414 | 0.18820 | -0.140 | 0.6285 |
| 5nM-NA | 6 | 0.50000 | 0.18820 | 0.116 | 0.8844 |
| -NA | 6 | -0.32543 | 0.18820 | -0.710 | 0.0589 |
| 50nM+NA | 6 | 0.15315 | 0.18820 | -0.231 | 0.5375 |
| 5nM+NA | 6 | 0.27706 | 0.18820 | -0.107 | 0.6614 |
| +NA | 6 | -0.84893 | 0.18820 | -1.233 | -0.4646 |

Std Error uses a pooled estimate of error variance

### Means Comparisons

#### Comparisons with a control using Dunnett's Method

## Oneway Analysis of Methylmalonic acid By Label

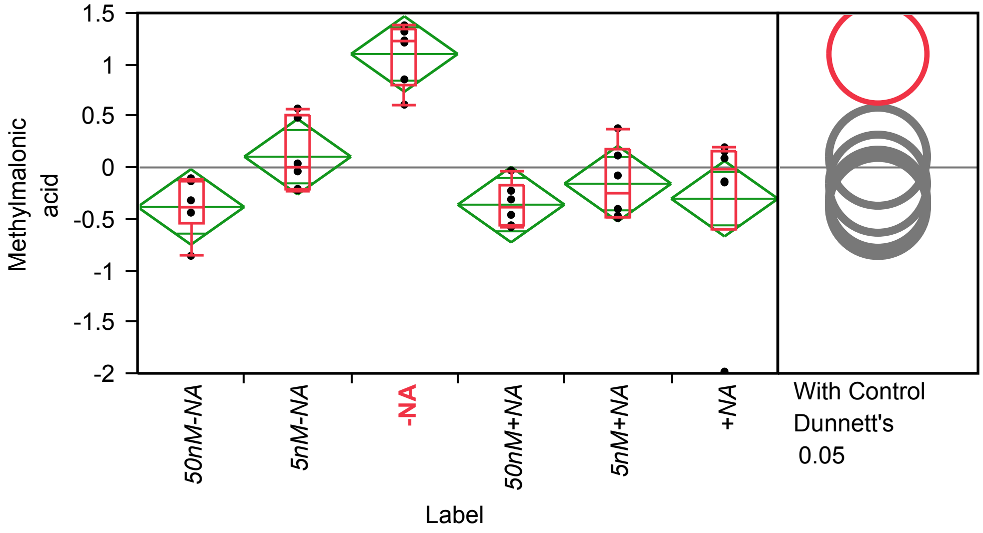


### Quantiles

| Level | Minimum | 10% | 25% | Median | 75% | 90% | Maximum |
|---|---|---|---|---|---|---|---|
| 50nM-NA | -0.85843 | -0.85843 | -0.55019 | -0.37903 | -0.12671 | -0.10529 | -0.10529 |
| 5nM-NA | -0.2219 | -0.2219 | -0.21164 | 0.000335 | 0.506098 | 0.572424 | 0.572424 |
| -NA | 0.609749 | 0.609749 | 0.793786 | 1.22333 | 1.334723 | 1.380203 | 1.380203 |
| 50nM+NA | -0.57936 | -0.57936 | -0.56634 | -0.38539 | -0.17706 | -0.02955 | -0.02955 |
| 5nM+NA | -0.49151 | -0.49151 | -0.47527 | -0.24062 | 0.182129 | 0.38041 | 0.38041 |
| +NA | -1.98319 | -1.98319 | -0.60477 | -0.02267 | 0.166626 | 0.195816 | 0.195816 |

### Oneway Anova

#### Summary of Fit

| | |
|---|---|
| Rsquare | 0.626877 |
| Adj Rsquare | 0.56469 |
| Root Mean Square Error | 0.439137 |
| Mean of Response | -8.3e-10 |
| Observations (or Sum Wgts) | 36 |

#### Analysis of Variance

| Source | DF | Sum of Squares | Mean Square | F Ratio | Prob > F |
|---|---|---|---|---|---|
| Label | 5 | 9.719703 | 1.94394 | 10.0805 | <.0001 * |
| Error | 30 | 5.785246 | 0.19284 | | |
| C. Total | 35 | 15.504948 | | | |

#### Means for Oneway Anova

| Level | Number | Mean | Std Error | Lower 95% | Upper 95% |
|---|---|---|---|---|---|
| 50nM-NA | 6 | -0.3838 | 0.17928 | -0.7500 | -0.018 |
| 5nM-NA | 6 | 0.1045 | 0.17928 | -0.2616 | 0.471 |
| -NA | 6 | 1.1019 | 0.17928 | 0.7358 | 1.468 |
| 50nM+NA | 6 | -0.3613 | 0.17928 | -0.7275 | 0.00481 |
| 5nM+NA | 6 | -0.1577 | 0.17928 | -0.5238 | 0.208 |
| +NA | 6 | -0.3035 | 0.17928 | -0.6697 | 0.063 |

Std Error uses a pooled estimate of error variance

### Means Comparisons

#### Comparisons with a control using Dunnett's Method

## Oneway Analysis of myo-inositol By Label

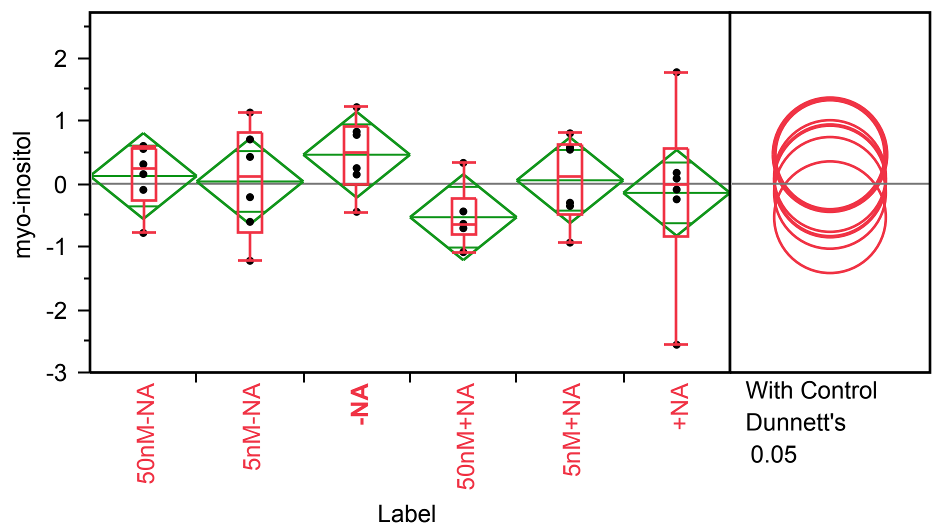


## Quantiles

| Level | Minimum | 10% | 25% | Median | 75% | 90% | Maximum |
|---|---|---|---|---|---|---|---|
| 50nM-NA | -0.7816 | -0.7816 | -0.26809 | 0.231785 | 0.56512 | 0.600507 | 0.600507 |
| 5nM-NA | -1.2244 | -1.2244 | -0.76022 | 0.10684 | 0.811245 | 1.131279 | 1.131279 |
| -NA | -0.44601 | -0.44601 | -0.00125 | 0.512243 | 0.924499 | 1.217106 | 1.217106 |
| 50nM+NA | -1.08706 | -1.08706 | -0.80202 | -0.64355 | -0.24748 | 0.331799 | 0.331799 |
| 5nM+NA | -0.93537 | -0.93537 | -0.49833 | 0.119787 | 0.633867 | 0.801669 | 0.801669 |
| +NA | -2.55885 | -2.55885 | -0.82512 | -0.00486 | 0.57355 | 1.772618 | 1.772618 |

## Oneway Anova

### Summary of Fit

| | |
|---|---|
| Rsquare | 0.13811 |
| Adj Rsquare | -0.00554 |
| Root Mean Square Error | 0.818123 |
| Mean of Response | -2.8e-10 |
| Observations (or Sum Wgts) | 36 |

### Analysis of Variance

| Source | DF | Sum of Squares | Mean Square | F Ratio | Prob > F |
|---|---|---|---|---|---|
| Label | 5 | 3.217596 | 0.643519 | 0.9614 | 0.4568 |
| Error | 30 | 20.079748 | 0.669325 | | |
| C. Total | 35 | 23.297345 | | | |

### Means for Oneway Anova

| Level | Number | Mean | Std Error | Lower 95% | Upper 95% |
|---|---|---|---|---|---|
| 50nM-NA | 6 | 0.12315 | 0.33400 | -0.559 | 0.8053 |
| 5nM-NA | 6 | 0.03661 | 0.33400 | -0.646 | 0.7187 |
| -NA | 6 | 0.46159 | 0.33400 | -0.221 | 1.1437 |
| 50nM+NA | 6 | -0.53166 | 0.33400 | -1.214 | 0.1505 |
| 5nM+NA | 6 | 0.05519 | 0.33400 | -0.627 | 0.7373 |
| +NA | 6 | -0.14488 | 0.33400 | -0.827 | 0.5372 |

Std Error uses a pooled estimate of error variance

## Means Comparisons

### Comparisons with a control using Dunnett's Method

## Oneway Analysis of N6-Acetyl-L-lysine By Label

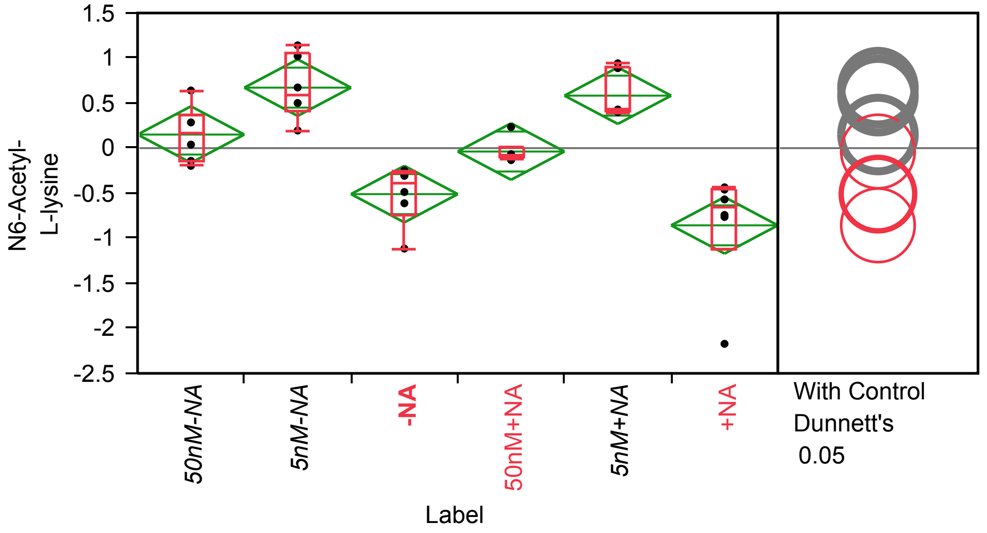


### Quantiles

| Level | Minimum | 10% | 25% | Median | 75% | 90% | Maximum |
|---|---|---|---|---|---|---|---|
| 50nM-NA | -0.1965 | -0.1965 | -0.15519 | 0.159107 | 0.374012 | 0.63945 | 0.63945 |
| 5nM-NA | 0.195988 | 0.195988 | 0.421793 | 0.587098 | 1.0507 | 1.140843 | 1.140843 |
| -NA | -1.11493 | -1.11493 | -0.7377 | -0.39721 | -0.28298 | -0.24675 | -0.24675 |
| 50nM+NA | -0.13275 | -0.13275 | -0.10762 | -0.08031 | 0.008725 | 0.234295 | 0.234295 |
| 5nM+NA | 0.399221 | 0.399221 | 0.410041 | 0.42688 | 0.901507 | 0.941211 | 0.941211 |
| +NA | -2.17219 | -2.17219 | -1.11587 | -0.65564 | -0.45364 | -0.43519 | -0.43519 |

### Oneway Anova

#### Summary of Fit

| | |
|---|---|
| Rsquare | 0.718822 |
| Adj Rsquare | 0.671959 |
| Root Mean Square Error | 0.376268 |
| Mean of Response | 2.47e-17 |
| Observations (or Sum Wgts) | 36 |

#### Analysis of Variance

| Source | DF | Sum of Squares | Mean Square | F Ratio | Prob > F |
|---|---|---|---|---|---|
| Label | 5 | 10.858128 | 2.17163 | 15.3388 | <.0001 * |
| Error | 30 | 4.247319 | 0.14158 | | |
| C. Total | 35 | 15.105447 | | | |

#### Means for Oneway Anova

| Level | Number | Mean | Std Error | Lower 95% | Upper 95% |
|---|---|---|---|---|---|
| 50nM-NA | 6 | 0.15088 | 0.15361 | -0.163 | 0.4646 |
| 5nM-NA | 6 | 0.67146 | 0.15361 | 0.358 | 0.9852 |
| -NA | 6 | -0.51052 | 0.15361 | -0.824 | -0.1968 |
| 50nM+NA | 6 | -0.03746 | 0.15361 | -0.351 | 0.2763 |
| 5nM+NA | 6 | 0.58269 | 0.15361 | 0.269 | 0.8964 |
| +NA | 6 | -0.85704 | 0.15361 | -1.171 | -0.5433 |

Std Error uses a pooled estimate of error variance

### Means Comparisons

#### Comparisons with a control using Dunnett's Method

## Oneway Analysis of N-Acetylaspartylglutamic acid By Label

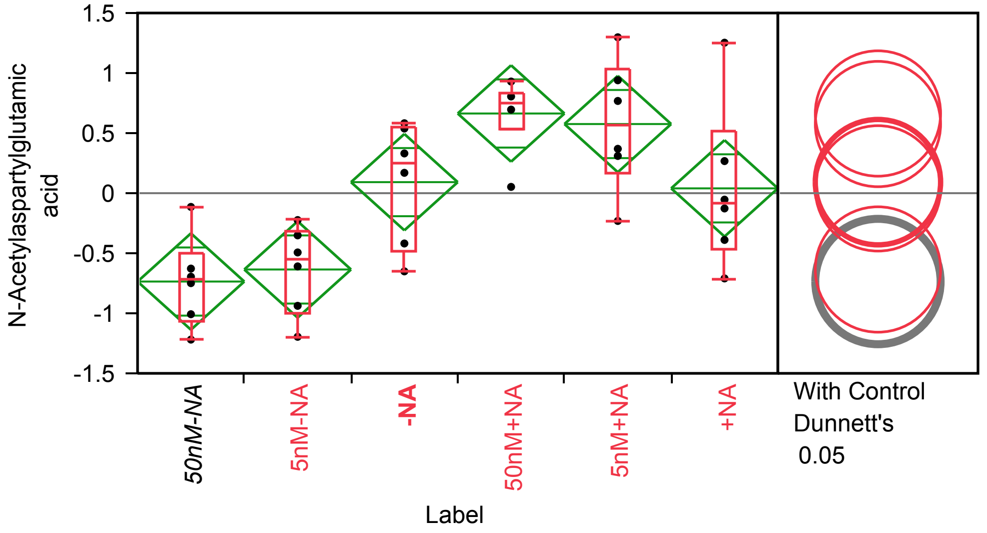


### Quantiles

| Level | Minimum | 10% | 25% | Median | 75% | 90% | Maximum |
|---|---|---|---|---|---|---|---|
| 50nM-NA | -1.219 | -1.219 | -1.06054 | -0.72239 | -0.49955 | -0.11446 | -0.11446 |
| 5nM-NA | -1.19578 | -1.19578 | -1.00238 | -0.55151 | -0.3194 | -0.22398 | -0.22398 |
| -NA | -0.65086 | -0.65086 | -0.47685 | 0.250233 | 0.54927 | 0.582236 | 0.582236 |
| 50nM+NA | 0.052286 | 0.052286 | 0.53484 | 0.748972 | 0.836183 | 0.929371 | 0.929371 |
| 5nM+NA | -0.23105 | -0.23105 | 0.174717 | 0.568565 | 1.029464 | 1.297836 | 1.297836 |
| +NA | -0.70965 | -0.70965 | -0.46933 | -0.09048 | 0.513631 | 1.25276 | 1.25276 |

### Oneway Anova

#### Summary of Fit

| | |
|---|---|
| Rsquare | 0.598629 |
| Adj Rsquare | 0.531734 |
| Root Mean Square Error | 0.481138 |
| Mean of Response | 2.78e-10 |
| Observations (or Sum Wgts) | 36 |

#### Analysis of Variance

| Source | DF | Sum of Squares | Mean Square | F Ratio | Prob > F |
|---|---|---|---|---|---|
| Label | 5 | 10.357924 | 2.07158 | 8.9488 | <.0001 * |
| Error | 30 | 6.944823 | 0.23149 | | |
| C. Total | 35 | 17.302748 | | | |

#### Means for Oneway Anova

| Level | Number | Mean | Std Error | Lower 95% | Upper 95% |
|---|---|---|---|---|---|
| 50nM-NA | 6 | -0.73564 | 0.19642 | -1.137 | -0.334 |
| 5nM-NA | 6 | -0.63532 | 0.19642 | -1.036 | -0.234 |
| -NA | 6 | 0.09188 | 0.19642 | -0.309 | 0.493 |
| 50nM+NA | 6 | 0.66340 | 0.19642 | 0.262 | 1.065 |
| 5nM+NA | 6 | 0.57565 | 0.19642 | 0.174 | 0.977 |
| +NA | 6 | 0.04003 | 0.19642 | -0.361 | 0.441 |

Std Error uses a pooled estimate of error variance

### Means Comparisons

#### Comparisons with a control using Dunnett's Method

## Oneway Analysis of N-acetyl-glucosamine-1-phosphate By Label

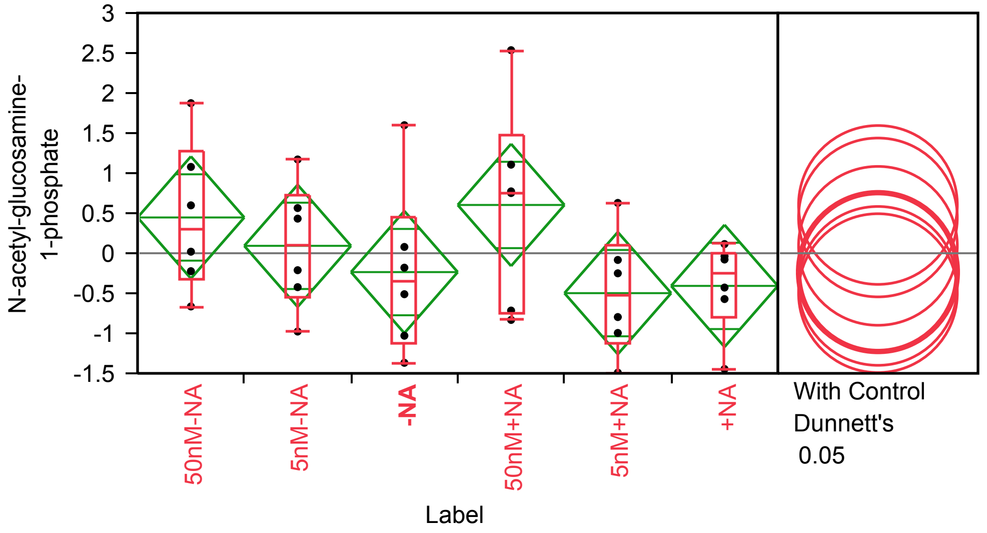


## Quantiles

| Level | Minimum | 10% | 25% | Median | 75% | 90% | Maximum |
|---|---|---|---|---|---|---|---|
| 50nM-NA | -0.66465 | -0.66465 | -0.33428 | 0.308593 | 1.276982 | 1.873894 | 1.873894 |
| 5nM-NA | -0.97775 | -0.97775 | -0.56208 | 0.110056 | 0.715893 | 1.170779 | 1.170779 |
| -NA | -1.36827 | -1.36827 | -1.1146 | -0.34609 | 0.458572 | 1.598815 | 1.598815 |
| 50nM+NA | -0.83275 | -0.83275 | -0.74528 | 0.762042 | 1.463448 | 2.535569 | 2.535569 |
| 5nM+NA | -1.49141 | -1.49141 | -1.11959 | -0.52383 | 0.094202 | 0.62829 | 0.62829 |
| +NA | -1.44612 | -1.44612 | -0.7906 | -0.25355 | 0.000943 | 0.114674 | 0.114674 |

## Oneway Anova

### Summary of Fit

| | |
|---|---|
| Rsquare | 0.199509 |
| Adj Rsquare | 0.066094 |
| Root Mean Square Error | 0.914298 |
| Mean of Response | 0 |
| Observations (or Sum Wgts) | 36 |

### Analysis of Variance

| Source | DF | Sum of Squares | Mean Square | F Ratio | Prob > F |
|---|---|---|---|---|---|
| Label | 5 | 6.250319 | 1.25006 | 1.4954 | 0.2208 |
| Error | 30 | 25.078215 | 0.83594 | | |
| C. Total | 35 | 31.328534 | | | |

### Means for Oneway Anova

| Level | Number | Mean | Std Error | Lower 95% | Upper 95% |
|---|---|---|---|---|---|
| 50nM-NA | 6 | 0.44671 | 0.37326 | -0.316 | 1.2090 |
| 5nM-NA | 6 | 0.09231 | 0.37326 | -0.670 | 0.8546 |
| -NA | 6 | -0.23553 | 0.37326 | -0.998 | 0.5268 |
| 50nM+NA | 6 | 0.60281 | 0.37326 | -0.159 | 1.3651 |
| 5nM+NA | 6 | -0.49838 | 0.37326 | -1.261 | 0.2639 |
| +NA | 6 | -0.40793 | 0.37326 | -1.170 | 0.3544 |

Std Error uses a pooled estimate of error variance

## Means Comparisons

### Comparisons with a control using Dunnett's Method

## Oneway Analysis of N-acetyl-glutamine By Label

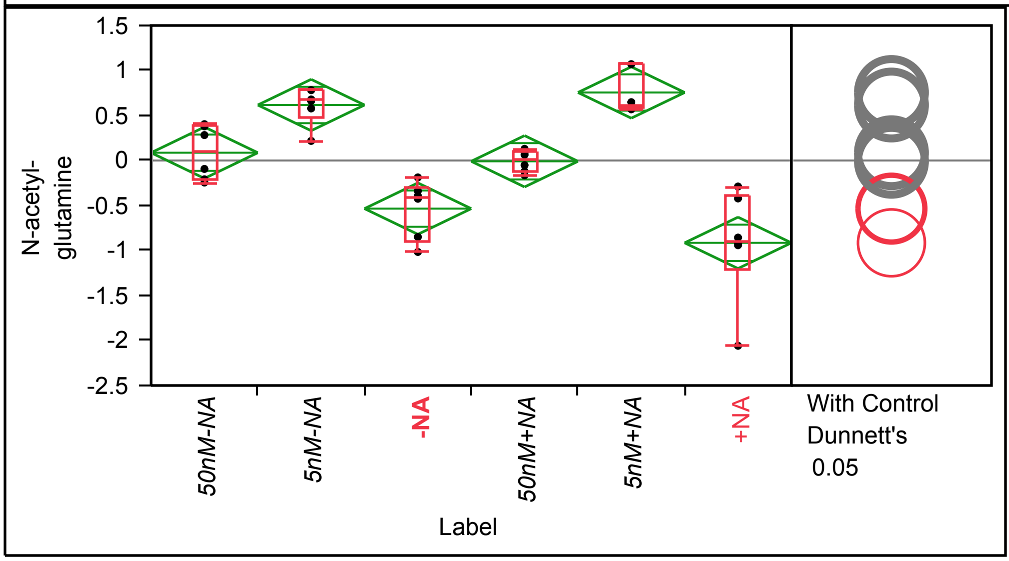


## Quantiles

| Level | Minimum | 10% | 25% | Median | 75% | 90% | Maximum |
|---|---|---|---|---|---|---|---|
| 50nM-NA | -0.24466 | -0.24466 | -0.21796 | 0.094377 | 0.385639 | 0.403666 | 0.403666 |
| 5nM-NA | 0.217653 | 0.217653 | 0.488325 | 0.670034 | 0.781307 | 0.781409 | 0.781409 |
| -NA | -1.01621 | -1.01621 | -0.89121 | -0.40882 | -0.30044 | -0.18789 | -0.18789 |
| 50nM+NA | -0.16298 | -0.16298 | -0.13086 | 0.005734 | 0.099365 | 0.129503 | 0.129503 |
| 5nM+NA | 0.568824 | 0.568824 | 0.580297 | 0.621266 | 1.068584 | 1.070638 | 1.070638 |
| +NA | -2.05865 | -2.05865 | -1.22058 | -0.88926 | -0.38859 | -0.28907 | -0.28907 |

## Oneway Anova

### Summary of Fit

| | |
|---|---|
| Rsquare | 0.780386 |
| Adj Rsquare | 0.743784 |
| Root Mean Square Error | 0.342295 |
| Mean of Response | 1.111e-9 |
| Observations (or Sum Wgts) | 36 |

### Analysis of Variance

| Source | DF | Sum of Squares | Mean Square | F Ratio | Prob > F |
|---|---|---|---|---|---|
| Label | 5 | 12.490286 | 2.49806 | 21.3207 | <.0001 * |
| Error | 30 | 3.514981 | 0.11717 | | |
| C. Total | 35 | 16.005268 | | | |

### Means for Oneway Anova

| Level | Number | Mean | Std Error | Lower 95% | Upper 95% |
|---|---|---|---|---|---|
| 50nM-NA | 6 | 0.08639 | 0.13974 | -0.199 | 0.372 |
| 5nM-NA | 6 | 0.61649 | 0.13974 | 0.331 | 0.902 |
| -NA | 6 | -0.53487 | 0.13974 | -0.820 | -0.249 |
| 50nM+NA | 6 | -0.00881 | 0.13974 | -0.294 | 0.277 |
| 5nM+NA | 6 | 0.75567 | 0.13974 | 0.470 | 1.041 |
| +NA | 6 | -0.91487 | 0.13974 | -1.200 | -0.629 |

Std Error uses a pooled estimate of error variance

## Means Comparisons

### Comparisons with a control using Dunnett's Method

## Oneway Analysis of N-Acetyl-L-alanine By Label

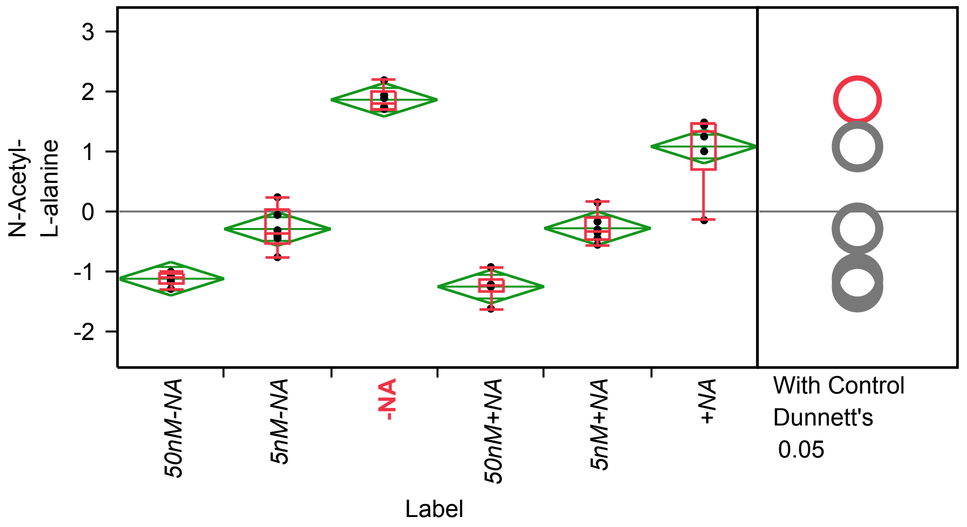


## Quantiles

| Level | Minimum | 10% | 25% | Median | 75% | 90% | Maximum |
|---|---|---|---|---|---|---|---|
| 50nM-NA | -1.29042 | -1.29042 | -1.20764 | -1.11172 | -1.02094 | -1.0039 | -1.0039 |
| 5nM-NA | -0.7615 | -0.7615 | -0.52435 | -0.36236 | 0.016974 | 0.237483 | 0.237483 |
| -NA | 1.705468 | 1.705468 | 1.707203 | 1.815349 | 2.002501 | 2.188442 | 2.188442 |
| 50nM+NA | -1.61963 | -1.61963 | -1.34809 | -1.24706 | -1.14438 | -0.92613 | -0.92613 |
| 5nM+NA | -0.55485 | -0.55485 | -0.47564 | -0.32805 | -0.0887 | 0.150169 | 0.150169 |
| +NA | -0.14537 | -0.14537 | 0.716445 | 1.341311 | 1.474145 | 1.482768 | 1.482768 |

## Oneway Anova

### Summary of Fit

| | |
|---|---|
| Rsquare | 0.931693 |
| Adj Rsquare | 0.920308 |
| Root Mean Square Error | 0.334426 |
| Mean of Response | 0 |
| Observations (or Sum Wgts) | 36 |

### Analysis of Variance

| Source | DF | Sum of Squares | Mean Square | F Ratio | Prob > F |
|---|---|---|---|---|---|
| Label | 5 | 45.764548 | 9.15291 | 81.8385 | <.0001 * |
| Error | 30 | 3.355232 | 0.11184 | | |
| C. Total | 35 | 49.119780 | | | |

### Means for Oneway Anova

| Level | Number | Mean | Std Error | Lower 95% | Upper 95% |
|---|---|---|---|---|---|
| 50nM-NA | 6 | -1.1207 | 0.13653 | -1.400 | -0.842 |
| 5nM-NA | 6 | -0.2918 | 0.13653 | -0.571 | -0.013 |
| -NA | 6 | 1.8622 | 0.13653 | 1.583 | 2.141 |
| 50nM+NA | 6 | -1.2524 | 0.13653 | -1.531 | -0.974 |
| 5nM+NA | 6 | -0.2797 | 0.13653 | -0.559 | -0.0009 |
| +NA | 6 | 1.0825 | 0.13653 | 0.804 | 1.361 |

Std Error uses a pooled estimate of error variance

## Means Comparisons

### Comparisons with a control using Dunnett's Method

## Oneway Analysis of N-acetyl-L-ornithine By Label

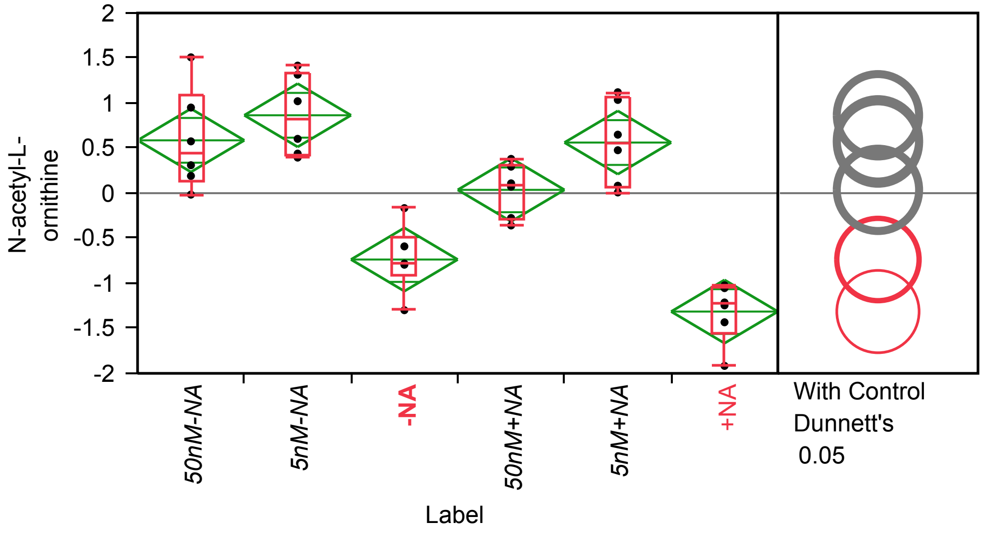


## Quantiles

| Level | Minimum | 10% | 25% | Median | 75% | 90% | Maximum |
|---|---|---|---|---|---|---|---|
| 50nM-NA | -0.01583 | -0.01583 | 0.139655 | 0.442901 | 1.089819 | 1.507972 | 1.507972 |
| 5nM-NA | 0.39513 | 0.39513 | 0.428143 | 0.811869 | 1.340642 | 1.415675 | 1.415675 |
| -NA | -1.29884 | -1.29884 | -0.9202 | -0.78448 | -0.48456 | -0.16601 | -0.16601 |
| 50nM+NA | -0.35832 | -0.35832 | -0.29619 | 0.090173 | 0.316032 | 0.380529 | 0.380529 |
| 5nM+NA | 0.008275 | 0.008275 | 0.06399 | 0.563154 | 1.056605 | 1.12049 | 1.12049 |
| +NA | -1.91711 | -1.91711 | -1.55409 | -1.22931 | -1.04597 | -1.02265 | -1.02265 |

## Oneway Anova

### Summary of Fit

| | |
|---|---|
| Rsquare | 0.805611 |
| Adj Rsquare | 0.773213 |
| Root Mean Square Error | 0.42136 |
| Mean of Response | 5.56e-11 |
| Observations (or Sum Wgts) | 36 |

### Analysis of Variance

| Source | DF | Sum of Squares | Mean Square | F Ratio | Prob > F |
|---|---|---|---|---|---|
| Label | 5 | 22.074050 | 4.41481 | 24.8660 | <.0001 * |
| Error | 30 | 5.326319 | 0.17754 | | |
| C. Total | 35 | 27.400368 | | | |

### Means for Oneway Anova

| Level | Number | Mean | Std Error | Lower 95% | Upper 95% |
|---|---|---|---|---|---|
| 50nM-NA | 6 | 0.5866 | 0.17202 | 0.235 | 0.938 |
| 5nM-NA | 6 | 0.8649 | 0.17202 | 0.514 | 1.216 |
| -NA | 6 | -0.7364 | 0.17202 | -1.088 | -0.385 |
| 50nM+NA | 6 | 0.0369 | 0.17202 | -0.314 | 0.388 |
| 5nM+NA | 6 | 0.5622 | 0.17202 | 0.211 | 0.913 |
| +NA | 6 | -1.3142 | 0.17202 | -1.666 | -0.963 |

Std Error uses a pooled estimate of error variance

## Means Comparisons

### Comparisons with a control using Dunnett's Method

## Oneway Analysis of N-Acetylputrescine By Label

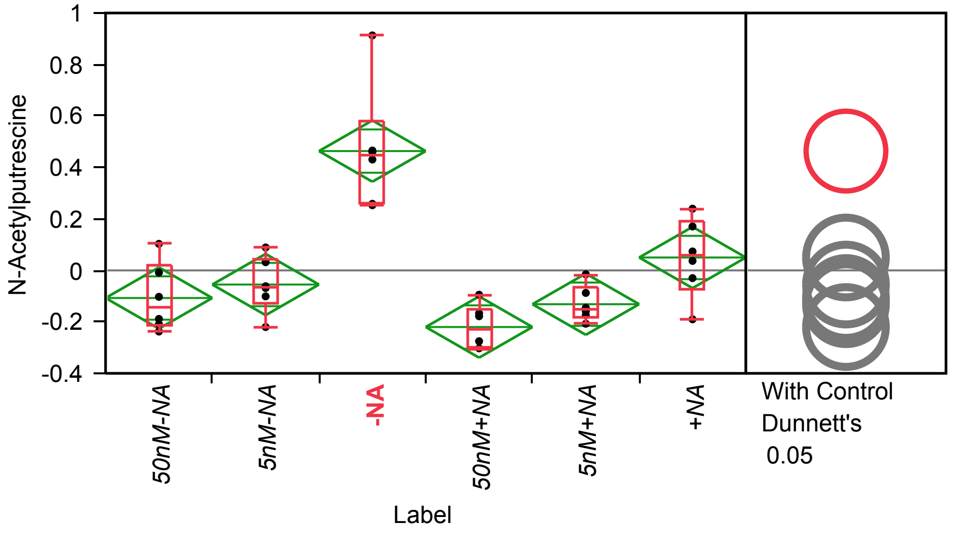


## Quantiles

| Level | Minimum | 10% | 25% | Median | 75% | 90% | Maximum |
|---|---|---|---|---|---|---|---|
| 50nM-NA | -0.23748 | -0.23748 | -0.21664 | -0.14566 | 0.019355 | 0.103052 | 0.103052 |
| 5nM-NA | -0.21999 | -0.21999 | -0.13068 | -0.06502 | 0.046194 | 0.089047 | 0.089047 |
| -NA | 0.253256 | 0.253256 | 0.257317 | 0.445633 | 0.577363 | 0.912818 | 0.912818 |
| 50nM+NA | -0.30278 | -0.30278 | -0.3027 | -0.22669 | -0.1488 | -0.09466 | -0.09466 |
| 5nM+NA | -0.20716 | -0.20716 | -0.18224 | -0.15215 | -0.06937 | -0.01524 | -0.01524 |
| +NA | -0.19003 | -0.19003 | -0.07003 | 0.055242 | 0.187966 | 0.239648 | 0.239648 |

## Oneway Anova

### Summary of Fit

| | |
|---|---|
| Rsquare | 0.744998 |
| Adj Rsquare | 0.702498 |
| Root Mean Square Error | 0.142746 |
| Mean of Response | -1.1e-10 |
| Observations (or Sum Wgts) | 36 |

### Analysis of Variance

| Source | DF | Sum of Squares | Mean Square | F Ratio | Prob > F |
|---|---|---|---|---|---|
| Label | 5 | 1.7859141 | 0.357183 | 17.5293 | <.0001 * |
| Error | 30 | 0.6112913 | 0.020376 | | |
| C. Total | 35 | 2.3972054 | | | |

### Means for Oneway Anova

| Level | Number | Mean | Std Error | Lower 95% | Upper 95% |
|---|---|---|---|---|---|
| 50nM-NA | 6 | -0.10733 | 0.05828 | -0.2263 | 0.0117 |
| 5nM-NA | 6 | -0.05500 | 0.05828 | -0.1740 | 0.0640 |
| -NA | 6 | 0.46359 | 0.05828 | 0.3446 | 0.5826 |
| 50nM+NA | 6 | -0.22006 | 0.05828 | -0.3391 | -0.1010 |
| 5nM+NA | 6 | -0.13134 | 0.05828 | -0.2504 | -0.0123 |
| +NA | 6 | 0.05014 | 0.05828 | -0.0689 | 0.1692 |

Std Error uses a pooled estimate of error variance

## Means Comparisons

### Comparisons with a control using Dunnett's Method

## Oneway Analysis of NAD By Label

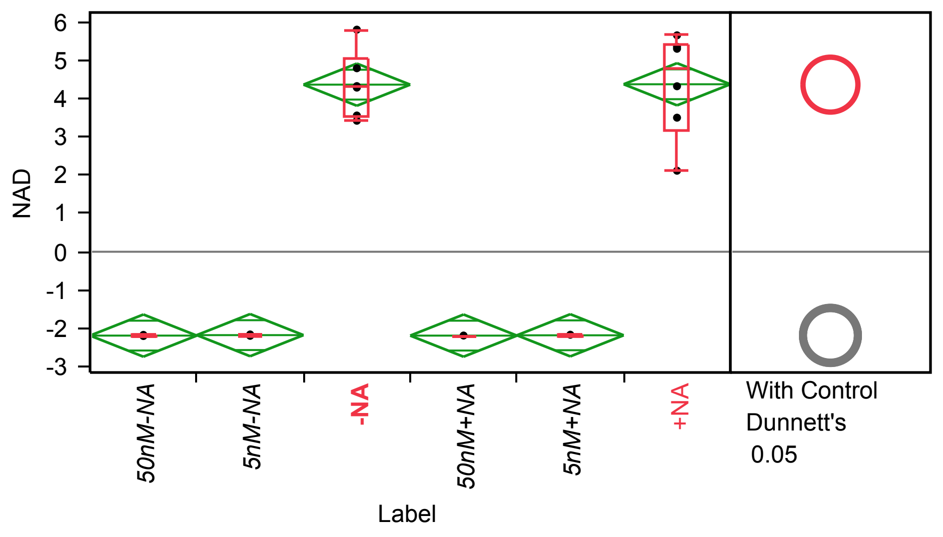


## Quantiles

| Level | Minimum | 10% | 25% | Median | 75% | 90% | Maximum |
|---|---|---|---|---|---|---|---|
| 50nM-NA | -2.20143 | -2.20143 | -2.19685 | -2.19217 | -2.18349 | -2.17179 | -2.17179 |
| 5nM-NA | -2.19576 | -2.19576 | -2.18665 | -2.17684 | -2.17058 | -2.16045 | -2.16045 |
| -NA | 3.422451 | 3.422451 | 3.524903 | 4.307548 | 5.045661 | 5.801577 | 5.801577 |
| 50nM+NA | -2.19131 | -2.19131 | -2.19124 | -2.19079 | -2.18988 | -2.18876 | -2.18876 |
| 5nM+NA | -2.19002 | -2.19002 | -2.18773 | -2.18421 | -2.1776 | -2.16768 | -2.16768 |
| +NA | 2.117008 | 2.117008 | 3.155877 | 4.813003 | 5.429035 | 5.653624 | 5.653624 |

## Oneway Anova

### Summary of Fit

| | |
|---|---|
| Rsquare | 0.963266 |
| Adj Rsquare | 0.957143 |
| Root Mean Square Error | 0.661089 |
| Mean of Response | -2.78e-8 |
| Observations (or Sum Wgts) | 36 |

### Analysis of Variance

| Source | DF | Sum of Squares | Mean Square | F Ratio | Prob > F |
|---|---|---|---|---|---|
| Label | 5 | 343.80869 | 68.7617 | 157.3356 | <.0001 * |
| Error | 30 | 13.11116 | 0.4370 | | |
| C. Total | 35 | 356.91985 | | | |

### Means for Oneway Anova

| Level | Number | Mean | Std Error | Lower 95% | Upper 95% |
|---|---|---|---|---|---|
| 50nM-NA | 6 | -2.1900 | 0.26989 | -2.741 | -1.639 |
| 5nM-NA | 6 | -2.1779 | 0.26989 | -2.729 | -1.627 |
| -NA | 6 | 4.3653 | 0.26989 | 3.814 | 4.916 |
| 50nM+NA | 6 | -2.1905 | 0.26989 | -2.742 | -1.639 |
| 5nM+NA | 6 | -2.1823 | 0.26989 | -2.734 | -1.631 |
| +NA | 6 | 4.3755 | 0.26989 | 3.824 | 4.927 |

Std Error uses a pooled estimate of error variance

## Means Comparisons

### Comparisons with a control using Dunnett's Method

## Oneway Analysis of NADH By Label

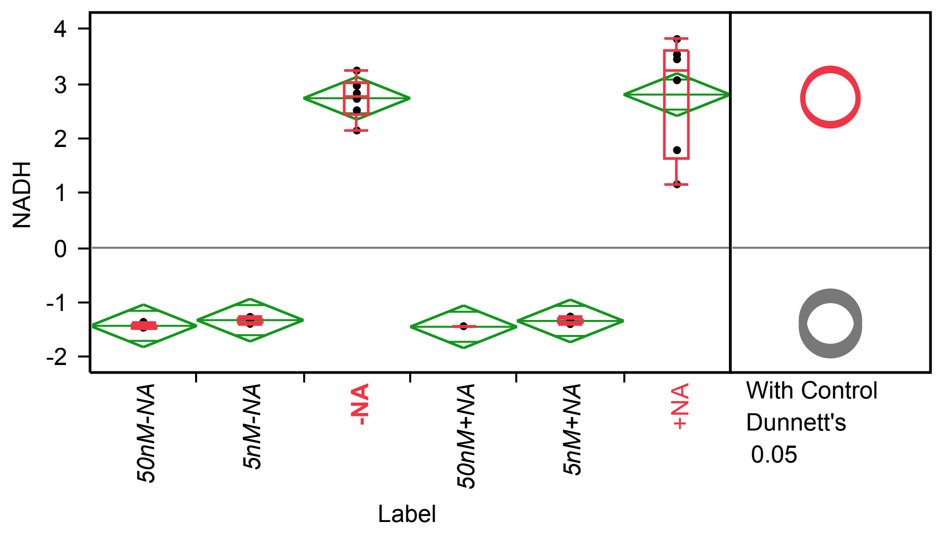


### Quantiles

| Level | Minimum | 10% | 25% | Median | 75% | 90% | Maximum |
|---|---|---|---|---|---|---|---|
| 50nM-NA | -1.46195 | -1.46195 | -1.46149 | -1.43946 | -1.3925 | -1.35619 | -1.35619 |
| 5nM-NA | -1.39043 | -1.39043 | -1.37456 | -1.30916 | -1.2882 | -1.26429 | -1.26429 |
| -NA | 2.14431 | 2.14431 | 2.420294 | 2.77214 | 3.02977 | 3.238229 | 3.238229 |
| 50nM+NA | -1.45087 | -1.45087 | -1.44879 | -1.4468 | -1.439 | -1.43396 | -1.43396 |
| 5nM+NA | -1.39448 | -1.39448 | -1.38389 | -1.34903 | -1.28623 | -1.25871 | -1.25871 |
| +NA | 1.160839 | 1.160839 | 1.628253 | 3.253598 | 3.601238 | 3.813845 | 3.813845 |

### Oneway Anova

#### Summary of Fit

| | |
|---|---|
| Rsquare | 0.9549 |
| Adj Rsquare | 0.947383 |
| Root Mean Square Error | 0.465818 |
| Mean of Response | 1.97e-16 |
| Observations (or Sum Wgts) | 36 |

#### Analysis of Variance

| Source | DF | Sum of Squares | Mean Square | F Ratio | Prob > F |
|---|---|---|---|---|---|
| Label | 5 | 137.82562 | 27.5651 | 127.0364 | <.0001 * |
| Error | 30 | 6.50958 | 0.2170 | | |
| C. Total | 35 | 144.33520 | | | |

#### Means for Oneway Anova

| Level | Number | Mean | Std Error | Lower 95% | Upper 95% |
|---|---|---|---|---|---|
| 50nM-NA | 6 | -1.4272 | 0.19017 | -1.816 | -1.039 |
| 5nM-NA | 6 | -1.3231 | 0.19017 | -1.711 | -0.935 |
| -NA | 6 | 2.7332 | 0.19017 | 2.345 | 3.122 |
| 50nM+NA | 6 | -1.4445 | 0.19017 | -1.833 | -1.056 |
| 5nM+NA | 6 | -1.3378 | 0.19017 | -1.726 | -0.949 |
| +NA | 6 | 2.7994 | 0.19017 | 2.411 | 3.188 |

Std Error uses a pooled estimate of error variance

### Means Comparisons

#### Comparisons with a control using Dunnett's Method

## Oneway Analysis of NADP By Label

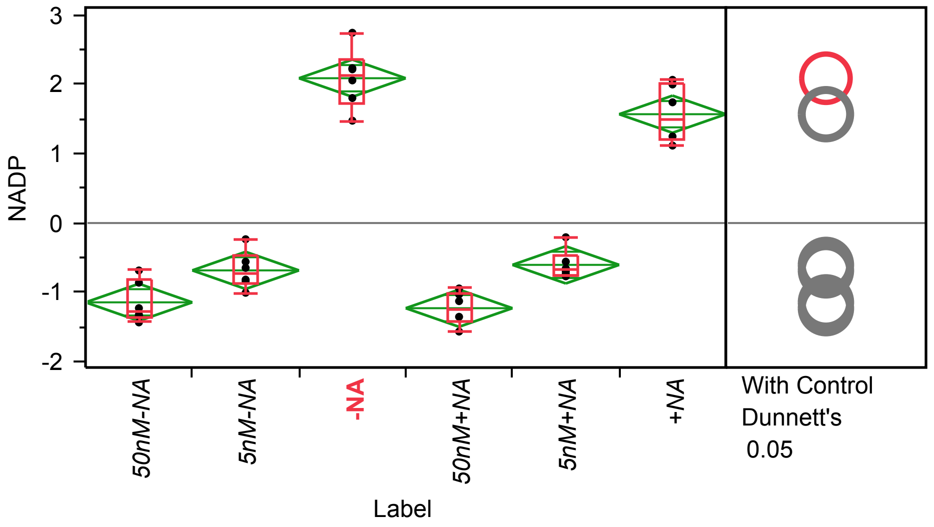


## Quantiles

| Level | Minimum | 10% | 25% | Median | 75% | 90% | Maximum |
|---|---|---|---|---|---|---|---|
| 50nM-NA | -1.4339 | -1.4339 | -1.362 | -1.27532 | -0.81682 | -0.68454 | -0.68454 |
| 5nM-NA | -1.0064 | -1.0064 | -0.87989 | -0.73197 | -0.47723 | -0.23394 | -0.23394 |
| -NA | 1.477346 | 1.477346 | 1.722864 | 2.139119 | 2.36285 | 2.746087 | 2.746087 |
| 50nM+NA | -1.56628 | -1.56628 | -1.40695 | -1.24027 | -1.00906 | -0.94418 | -0.94418 |
| 5nM+NA | -0.76877 | -0.76877 | -0.74466 | -0.67748 | -0.46944 | -0.20514 | -0.20514 |
| +NA | 1.118233 | 1.118233 | 1.215428 | 1.49641 | 2.016624 | 2.066547 | 2.066547 |

## Oneway Anova

### Summary of Fit

| | |
|---|---|
| Rsquare | 0.952949 |
| Adj Rsquare | 0.945108 |
| Root Mean Square Error | 0.321845 |
| Mean of Response | -8.33e-9 |
| Observations (or Sum Wgts) | 36 |

### Analysis of Variance

| Source | DF | Sum of Squares | Mean Square | F Ratio | Prob > F |
|---|---|---|---|---|---|
| Label | 5 | 62.939003 | 12.5878 | 121.5221 | <.0001 * |
| Error | 30 | 3.107534 | 0.1036 | | |
| C. Total | 35 | 66.046537 | | | |

### Means for Oneway Anova

| Level | Number | Mean | Std Error | Lower 95% | Upper 95% |
|---|---|---|---|---|---|
| 50nM-NA | 6 | -1.1447 | 0.13139 | -1.413 | -0.876 |
| 5nM-NA | 6 | -0.6834 | 0.13139 | -0.952 | -0.415 |
| -NA | 6 | 2.0902 | 0.13139 | 1.822 | 2.359 |
| 50nM+NA | 6 | -1.2293 | 0.13139 | -1.498 | -0.961 |
| 5nM+NA | 6 | -0.6038 | 0.13139 | -0.872 | -0.335 |
| +NA | 6 | 1.5709 | 0.13139 | 1.303 | 1.839 |

Std Error uses a pooled estimate of error variance

## Means Comparisons

### Comparisons with a control using Dunnett's Method

## Oneway Analysis of N-carbamoyl-L-aspartate By Label

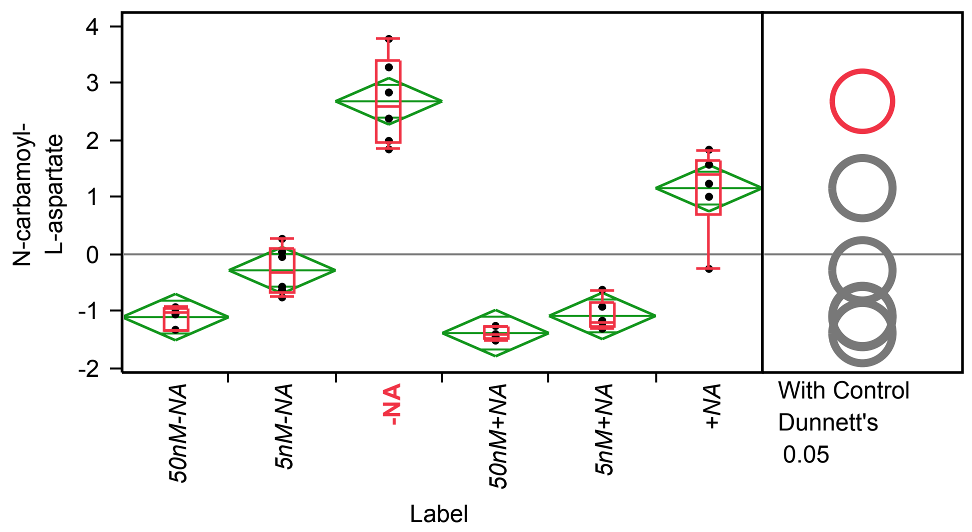


### Quantiles

| Level | Minimum | 10% | 25% | Median | 75% | 90% | Maximum |
|---|---|---|---|---|---|---|---|
| 50nM-NA | -1.32824 | -1.32824 | -1.32324 | -1.03294 | -0.94752 | -0.92624 | -0.92624 |
| 5nM-NA | -0.75415 | -0.75415 | -0.65472 | -0.30791 | 0.098687 | 0.270195 | 0.270195 |
| -NA | 1.837594 | 1.837594 | 1.94864 | 2.603852 | 3.397625 | 3.769718 | 3.769718 |
| 50nM+NA | -1.511 | -1.511 | -1.46231 | -1.40188 | -1.25984 | -1.25395 | -1.25395 |
| 5nM+NA | -1.30283 | -1.30283 | -1.27853 | -1.1786 | -0.84411 | -0.62212 | -0.62212 |
| +NA | -0.25515 | -0.25515 | 0.691355 | 1.399701 | 1.633619 | 1.830179 | 1.830179 |

### Oneway Anova

#### Summary of Fit

| | |
|---|---|
| Rsquare | 0.915808 |
| Adj Rsquare | 0.901776 |
| Root Mean Square Error | 0.486483 |
| Mean of Response | -7.4e-17 |
| Observations (or Sum Wgts) | 36 |

#### Analysis of Variance

| Source | DF | Sum of Squares | Mean Square | F Ratio | Prob > F |
|---|---|---|---|---|---|
| Label | 5 | 77.230605 | 15.4461 | 65.2657 | <.0001 * |
| Error | 30 | 7.099960 | 0.2367 | | |
| C. Total | 35 | 84.330566 | | | |

#### Means for Oneway Anova

| Level | Number | Mean | Std Error | Lower 95% | Upper 95% |
|---|---|---|---|---|---|
| 50nM-NA | 6 | -1.0994 | 0.19861 | -1.505 | -0.694 |
| 5nM-NA | 6 | -0.2800 | 0.19861 | -0.686 | 0.126 |
| -NA | 6 | 2.6790 | 0.19861 | 2.273 | 3.085 |
| 50nM+NA | 6 | -1.3794 | 0.19861 | -1.785 | -0.974 |
| 5nM+NA | 6 | -1.0784 | 0.19861 | -1.484 | -0.673 |
| +NA | 6 | 1.1582 | 0.19861 | 0.753 | 1.564 |

Std Error uses a pooled estimate of error variance

### Means Comparisons

#### Comparisons with a control using Dunnett's Method

## Oneway Analysis of N-Formylglycinamide ribonucleotide By Label

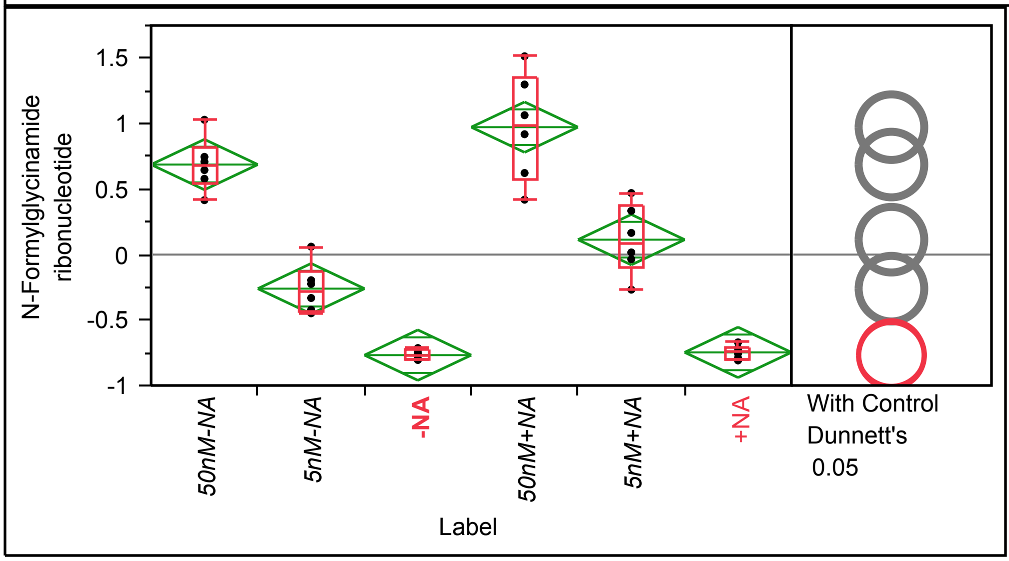


## Quantiles

| Level | Minimum | 10% | 25% | Median | 75% | 90% | Maximum |
|---|---|---|---|---|---|---|---|
| 50nM-NA | 0.41588 | 0.41588 | 0.53836 | 0.67752 | 0.817303 | 1.029653 | 1.029653 |
| 5nM-NA | -0.44818 | -0.44818 | -0.42834 | -0.27781 | -0.13183 | 0.060256 | 0.060256 |
| -NA | -0.80729 | -0.80729 | -0.80574 | -0.77581 | -0.72605 | -0.71407 | -0.71407 |
| 50nM+NA | 0.419433 | 0.419433 | 0.572236 | 0.992035 | 1.353033 | 1.514853 | 1.514853 |
| 5nM+NA | -0.26775 | -0.26775 | -0.09336 | 0.090977 | 0.368204 | 0.46999 | 0.46999 |
| +NA | -0.80846 | -0.80846 | -0.79479 | -0.74791 | -0.70452 | -0.6703 | -0.6703 |

## Oneway Anova

### Summary of Fit

| | |
|---|---|
| Rsquare | 0.908566 |
| Adj Rsquare | 0.893328 |
| Root Mean Square Error | 0.230894 |
| Mean of Response | 5.56e-10 |
| Observations (or Sum Wgts) | 36 |

### Analysis of Variance

| Source | DF | Sum of Squares | Mean Square | F Ratio | Prob > F |
|---|---|---|---|---|---|
| Label | 5 | 15.892695 | 3.17854 | 59.6214 | <.0001 * |
| Error | 30 | 1.599361 | 0.05331 | | |
| C. Total | 35 | 17.492055 | | | |

### Means for Oneway Anova

| Level | Number | Mean | Std Error | Lower 95% | Upper 95% |
|---|---|---|---|---|---|
| 50nM-NA | 6 | 0.68771 | 0.09426 | 0.4952 | 0.880 |
| 5nM-NA | 6 | -0.26019 | 0.09426 | -0.4527 | -0.068 |
| -NA | 6 | -0.76804 | 0.09426 | -0.9606 | -0.576 |
| 50nM+NA | 6 | 0.97344 | 0.09426 | 0.7809 | 1.166 |
| 5nM+NA | 6 | 0.11387 | 0.09426 | -0.0786 | 0.306 |
| +NA | 6 | -0.74679 | 0.09426 | -0.9393 | -0.554 |

Std Error uses a pooled estimate of error variance

## Means Comparisons

### Comparisons with a control using Dunnett's Method

## Oneway Analysis of Ng,NG-dimethyl-L-arginine By Label

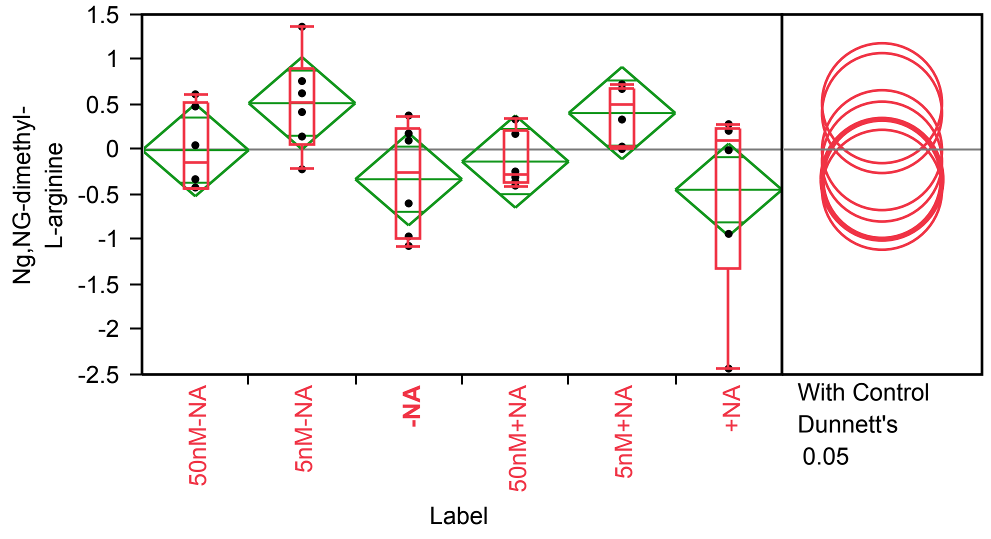


### Quantiles

| Level | Minimum | 10% | 25% | Median | 75% | 90% | Maximum |
|---|---|---|---|---|---|---|---|
| 50nM-NA | -0.43008 | -0.43008 | -0.42427 | -0.14138 | 0.512049 | 0.613396 | 0.613396 |
| 5nM-NA | -0.21992 | -0.21992 | 0.052959 | 0.52001 | 0.910874 | 1.363612 | 1.363612 |
| -NA | -1.07182 | -1.07182 | -0.99283 | -0.25005 | 0.228126 | 0.377531 | 0.377531 |
| 50nM+NA | -0.40148 | -0.40148 | -0.36896 | -0.27618 | 0.213151 | 0.333683 | 0.333683 |
| 5nM+NA | 0.005081 | 0.005081 | 0.025302 | 0.497443 | 0.685426 | 0.723487 | 0.723487 |
| +NA | -2.43449 | -2.43449 | -1.31168 | 0.099134 | 0.226165 | 0.28148 | 0.28148 |

### Oneway Anova

#### Summary of Fit

| | |
|---|---|
| Rsquare | 0.286088 |
| Adj Rsquare | 0.167102 |
| Root Mean Square Error | 0.614263 |
| Mean of Response | 2.78e-11 |
| Observations (or Sum Wgts) | 36 |

#### Analysis of Variance

| Source | DF | Sum of Squares | Mean Square | F Ratio | Prob > F |
|---|---|---|---|---|---|
| Label | 5 | 4.536113 | 0.907223 | 2.4044 | 0.0601 |
| Error | 30 | 11.319556 | 0.377319 | | |
| C. Total | 35 | 15.855669 | | | |

#### Means for Oneway Anova

| Level | Number | Mean | Std Error | Lower 95% | Upper 95% |
|---|---|---|---|---|---|
| 50nM-NA | 6 | -0.00725 | 0.25077 | -0.5194 | 0.5049 |
| 5nM-NA | 6 | 0.51460 | 0.25077 | 0.0025 | 1.0267 |
| -NA | 6 | -0.33043 | 0.25077 | -0.8426 | 0.1817 |
| 50nM+NA | 6 | -0.13422 | 0.25077 | -0.6464 | 0.3779 |
| 5nM+NA | 6 | 0.40471 | 0.25077 | -0.1074 | 0.9168 |
| +NA | 6 | -0.44740 | 0.25077 | -0.9595 | 0.0647 |

Std Error uses a pooled estimate of error variance

### Means Comparisons

#### Comparisons with a control using Dunnett's Method

## Oneway Analysis of Nicotinamide By Label

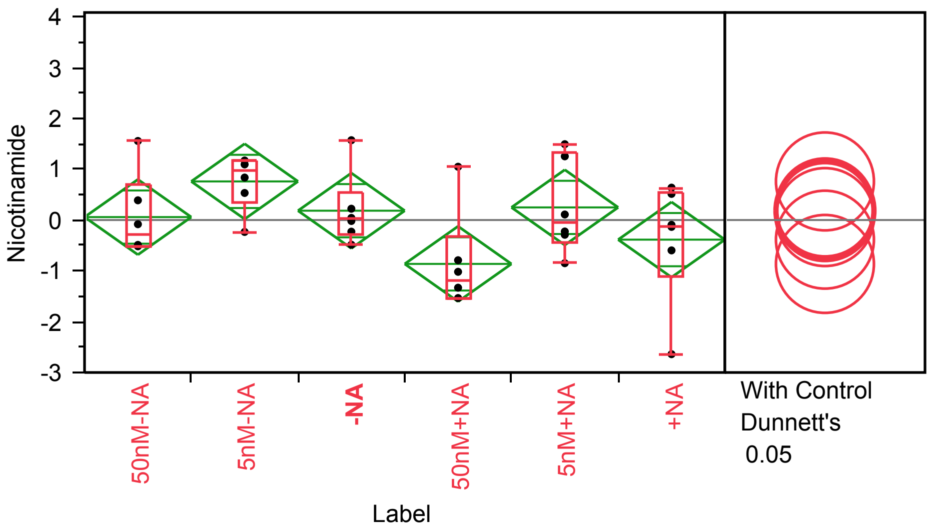


### Quantiles

| Level | Minimum | 10% | 25% | Median | 75% | 90% | Maximum |
|---|---|---|---|---|---|---|---|
| 50nM-NA | -0.51632 | -0.51632 | -0.50968 | -0.28593 | 0.679843 | 1.555235 | 1.555235 |
| 5nM-NA | -0.23445 | -0.23445 | 0.340704 | 0.964811 | 1.161922 | 1.171148 | 1.171148 |
| -NA | -0.48564 | -0.48564 | -0.29321 | 0.011468 | 0.559331 | 1.570586 | 1.570586 |
| 50nM+NA | -1.55211 | -1.55211 | -1.53951 | -1.17506 | -0.33143 | 1.047969 | 1.047969 |
| 5nM+NA | -0.84899 | -0.84899 | -0.42959 | -0.05838 | 1.31304 | 1.492062 | 1.492062 |
| +NA | -2.63956 | -2.63956 | -1.10783 | -0.11501 | 0.54515 | 0.636875 | 0.636875 |

### Oneway Anova

#### Summary of Fit

| | |
|---|---|
| Rsquare | 0.284782 |
| Adj Rsquare | 0.165579 |
| Root Mean Square Error | 0.888079 |
| Mean of Response | 1.23e-17 |
| Observations (or Sum Wgts) | 36 |

#### Analysis of Variance

| Source | DF | Sum of Squares | Mean Square | F Ratio | Prob > F |
|---|---|---|---|---|---|
| Label | 5 | 9.421021 | 1.88420 | 2.3890 | 0.0614 |
| Error | 30 | 23.660545 | 0.78868 | | |
| C. Total | 35 | 33.081566 | | | |

#### Means for Oneway Anova

| Level | Number | Mean | Std Error | Lower 95% | Upper 95% |
|---|---|---|---|---|---|
| 50nM-NA | 6 | 0.05794 | 0.36256 | -0.683 | 0.798 |
| 5nM-NA | 6 | 0.75960 | 0.36256 | 0.019 | 1.500 |
| -NA | 6 | 0.18351 | 0.36256 | -0.557 | 0.924 |
| 50nM+NA | 6 | -0.86346 | 0.36256 | -1.604 | -0.123 |
| 5nM+NA | 6 | 0.24831 | 0.36256 | -0.492 | 0.989 |
| +NA | 6 | -0.38590 | 0.36256 | -1.126 | 0.355 |

Std Error uses a pooled estimate of error variance

### Means Comparisons

#### Comparisons with a control using Dunnett's Method

## Oneway Analysis of Nicotinamide ribotide By Label

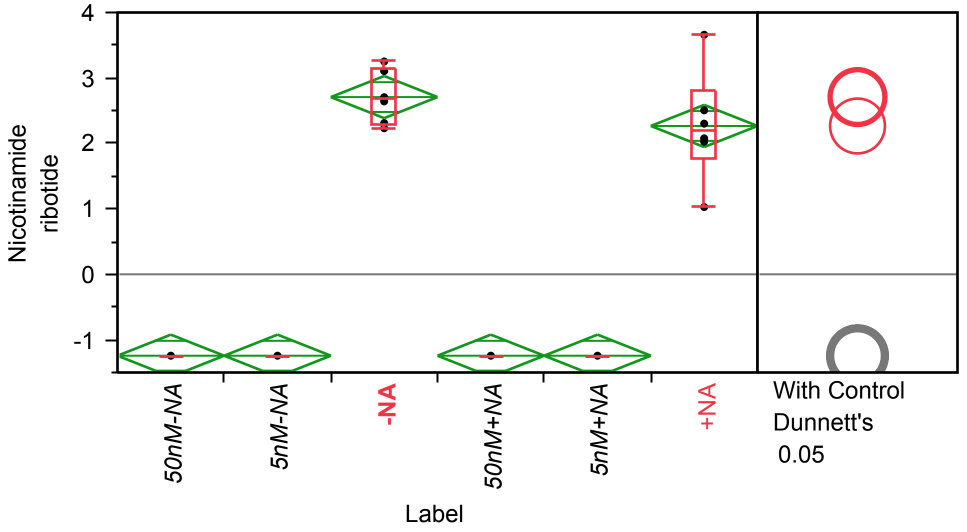


## Quantiles

| Level | Minimum | 10% | 25% | Median | 75% | 90% | Maximum |
|---|---|---|---|---|---|---|---|
| 50nM-NA | -1.24358 | -1.24358 | -1.24356 | -1.24354 | -1.24349 | -1.24344 | -1.24344 |
| 5nM-NA | -1.24357 | -1.24357 | -1.24352 | -1.24347 | -1.24343 | -1.24341 | -1.24341 |
| -NA | 2.231892 | 2.231892 | 2.290642 | 2.67388 | 3.141905 | 3.25152 | 3.25152 |
| 50nM+NA | -1.24357 | -1.24357 | -1.24356 | -1.24355 | -1.24352 | -1.24352 | -1.24352 |
| 5nM+NA | -1.2436 | -1.2436 | -1.24358 | -1.24354 | -1.24349 | -1.24347 | -1.24347 |
| +NA | 1.028179 | 1.028179 | 1.776601 | 2.18939 | 2.793607 | 3.659868 | 3.659868 |

## Oneway Anova

### Summary of Fit

| | |
|---|---|
| Rsquare | 0.961602 |
| Adj Rsquare | 0.955202 |
| Root Mean Square Error | 0.385971 |
| Mean of Response | -2.78e-8 |
| Observations (or Sum Wgts) | 36 |

### Analysis of Variance

| Source | DF | Sum of Squares | Mean Square | F Ratio | Prob > F |
|---|---|---|---|---|---|
| Label | 5 | 111.92166 | 22.3843 | 150.2574 | <.0001 * |
| Error | 30 | 4.46920 | 0.1490 | | |
| C. Total | 35 | 116.39086 | | | |

### Means for Oneway Anova

| Level | Number | Mean | Std Error | Lower 95% | Upper 95% |
|---|---|---|---|---|---|
| 50nM-NA | 6 | -1.2435 | 0.15757 | -1.565 | -0.922 |
| 5nM-NA | 6 | -1.2435 | 0.15757 | -1.565 | -0.922 |
| -NA | 6 | 2.7078 | 0.15757 | 2.386 | 3.030 |
| 50nM+NA | 6 | -1.2435 | 0.15757 | -1.565 | -0.922 |
| 5nM+NA | 6 | -1.2435 | 0.15757 | -1.565 | -0.922 |
| +NA | 6 | 2.2663 | 0.15757 | 1.944 | 2.588 |

Std Error uses a pooled estimate of error variance

## Means Comparisons

### Comparisons with a control using Dunnett's Method

## Oneway Analysis of Nicotinic acid By Label

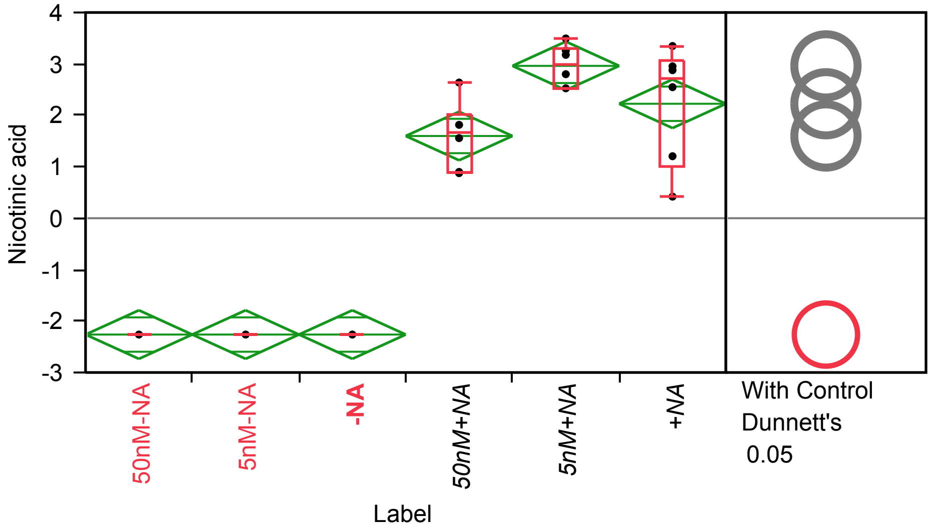


## Quantiles

| Level | Minimum | 10% | 25% | Median | 75% | 90% | Maximum |
|---|---|---|---|---|---|---|---|
| 50nM-NA | -2.26218 | -2.26218 | -2.26217 | -2.26215 | -2.26211 | -2.26207 | -2.26207 |
| 5nM-NA | -2.26218 | -2.26218 | -2.26213 | -2.2621 | -2.26206 | -2.26205 | -2.26205 |
| -NA | -2.26214 | -2.26214 | -2.26212 | -2.2621 | -2.26208 | -2.26206 | -2.26206 |
| 50nM+NA | 0.87653 | 0.87653 | 0.894293 | 1.68106 | 2.021086 | 2.637433 | 2.637433 |
| 5nM+NA | 2.515783 | 2.515783 | 2.523405 | 2.988662 | 3.318307 | 3.494872 | 3.494872 |
| +NA | 0.419808 | 0.419808 | 1.009014 | 2.713184 | 3.051871 | 3.347998 | 3.347998 |

## Oneway Anova

### Summary of Fit

| | |
|---|---|
| Rsquare | 0.951694 |
| Adj Rsquare | 0.943643 |
| Root Mean Square Error | 0.566692 |
| Mean of Response | -1.39e-8 |
| Observations (or Sum Wgts) | 36 |

### Analysis of Variance

| Source | DF | Sum of Squares | Mean Square | F Ratio | Prob > F |
|---|---|---|---|---|---|
| Label | 5 | 189.80795 | 37.9616 | 118.2088 | <.0001 * |
| Error | 30 | 9.63420 | 0.3211 | | |
| C. Total | 35 | 199.44216 | | | |

### Means for Oneway Anova

| Level | Number | Mean | Std Error | Lower 95% | Upper 95% |
|---|---|---|---|---|---|
| 50nM-NA | 6 | -2.2621 | 0.23135 | -2.735 | -1.790 |
| 5nM-NA | 6 | -2.2621 | 0.23135 | -2.735 | -1.790 |
| -NA | 6 | -2.2621 | 0.23135 | -2.735 | -1.790 |
| 50nM+NA | 6 | 1.5987 | 0.23135 | 1.126 | 2.071 |
| 5nM+NA | 6 | 2.9622 | 0.23135 | 2.490 | 3.435 |
| +NA | 6 | 2.2255 | 0.23135 | 1.753 | 2.698 |

Std Error uses a pooled estimate of error variance

## Means Comparisons

### Comparisons with a control using Dunnett's Method

## Oneway Analysis of Nicotyrine By Label

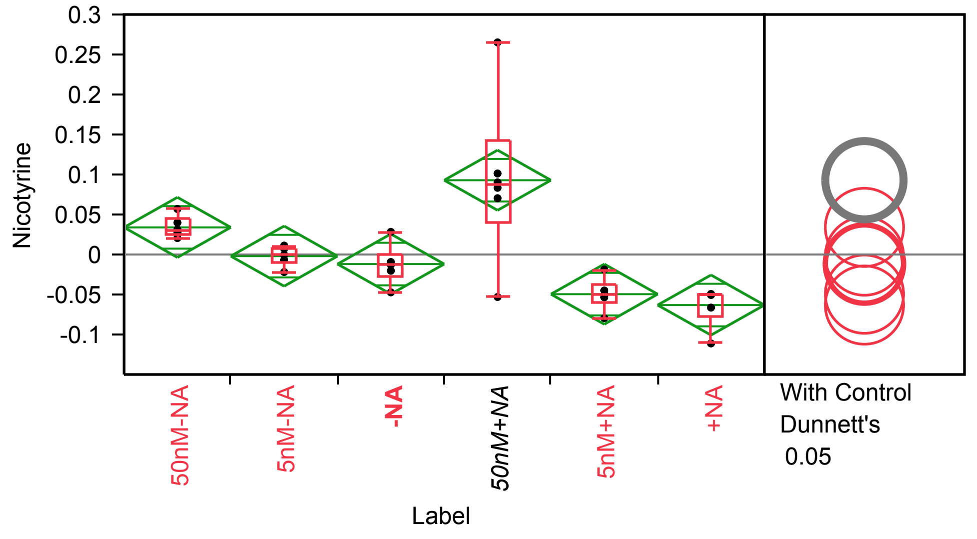


### Quantiles

| Level | Minimum | 10% | 25% | Median | 75% | 90% | Maximum |
|---|---|---|---|---|---|---|---|
| 50nM-NA | 0.020548 | 0.020548 | 0.024921 | 0.02988 | 0.044095 | 0.056877 | 0.056877 |
| 5nM-NA | -0.02192 | -0.02192 | -0.01062 | -0.00084 | 0.00816 | 0.01114 | 0.01114 |
| -NA | -0.0473 | -0.0473 | -0.02706 | -0.01146 | 2.938e-5 | 0.028264 | 0.028264 |
| 50nM+NA | -0.05314 | -0.05314 | 0.039472 | 0.086759 | 0.142246 | 0.265133 | 0.265133 |
| 5nM+NA | -0.07952 | -0.07952 | -0.06003 | -0.05033 | -0.03845 | -0.01878 | -0.01878 |
| +NA | -0.11118 | -0.11118 | -0.07758 | -0.05116 | -0.04986 | -0.04933 | -0.04933 |

### Oneway Anova

#### Summary of Fit

| | |
|---|---|
| Rsquare | 0.616981 |
| Adj Rsquare | 0.553145 |
| Root Mean Square Error | 0.045086 |
| Mean of Response | -2.8e-11 |
| Observations (or Sum Wgts) | 36 |

#### Analysis of Variance

| Source | DF | Sum of Squares | Mean Square | F Ratio | Prob > F |
|---|---|---|---|---|---|
| Label | 5 | 0.09823175 | 0.019646 | 9.6650 | <.0001 * |
| Error | 30 | 0.06098181 | 0.002033 | | |
| C. Total | 35 | 0.15921356 | | | |

#### Means for Oneway Anova

| Level | Number | Mean | Std Error | Lower 95% | Upper 95% |
|---|---|---|---|---|---|
| 50nM-NA | 6 | 0.03390 | 0.01841 | -0.0037 | 0.0715 |
| 5nM-NA | 6 | -0.00202 | 0.01841 | -0.0396 | 0.0356 |
| -NA | 6 | -0.01194 | 0.01841 | -0.0495 | 0.0257 |
| 50nM+NA | 6 | 0.09286 | 0.01841 | 0.0553 | 0.1304 |
| 5nM+NA | 6 | -0.04958 | 0.01841 | -0.0872 | -0.0120 |
| +NA | 6 | -0.06321 | 0.01841 | -0.1008 | -0.0256 |

Std Error uses a pooled estimate of error variance

### Means Comparisons

#### Comparisons with a control using Dunnett's Method

## Oneway Analysis of O-acetyl-L-serine By Label

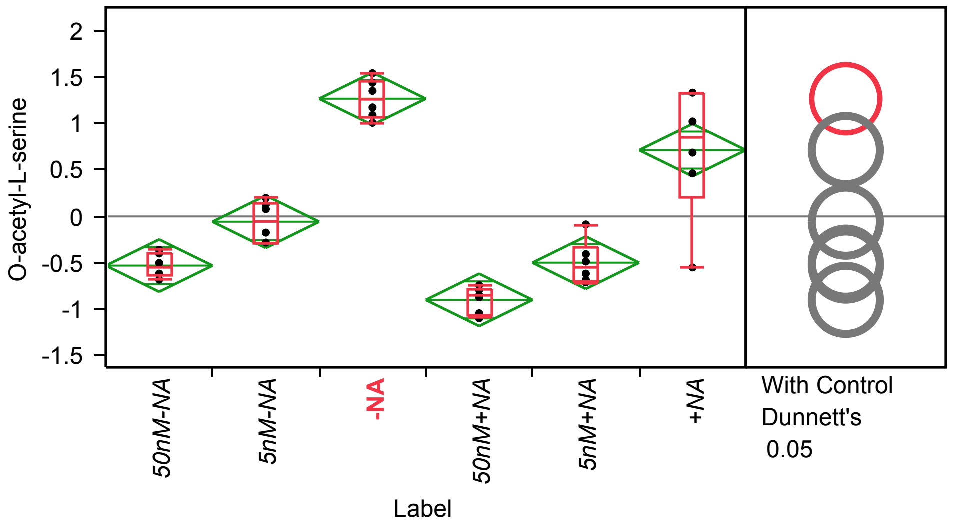


## Quantiles

| Level | Minimum | 10% | 25% | Median | 75% | 90% | Maximum |
|---|---|---|---|---|---|---|---|
| 50nM-NA | -0.68063 | -0.68063 | -0.63676 | -0.55844 | -0.38893 | -0.35904 | -0.35904 |
| 5nM-NA | -0.2897 | -0.2897 | -0.28344 | -0.04786 | 0.145303 | 0.198979 | 0.198979 |
| -NA | 1.008457 | 1.008457 | 1.070962 | 1.263285 | 1.465169 | 1.542661 | 1.542661 |
| 50nM+NA | -1.09704 | -1.09704 | -1.05649 | -0.85992 | -0.77944 | -0.74117 | -0.74117 |
| 5nM+NA | -0.71041 | -0.71041 | -0.69016 | -0.55103 | -0.326 | -0.08594 | -0.08594 |
| +NA | -0.54943 | -0.54943 | 0.210541 | 0.8553 | 1.332379 | 1.332959 | 1.332959 |

## Oneway Anova

### Summary of Fit

| | |
|---|---|
| Rsquare | 0.858081 |
| Adj Rsquare | 0.834428 |
| Root Mean Square Error | 0.33826 |
| Mean of Response | 2.78e-10 |
| Observations (or Sum Wgts) | 36 |

### Analysis of Variance

| Source | DF | Sum of Squares | Mean Square | F Ratio | Prob > F |
|---|---|---|---|---|---|
| Label | 5 | 20.754450 | 4.15089 | 36.2778 | <.0001 * |
| Error | 30 | 3.432591 | 0.11442 | | |
| C. Total | 35 | 24.187041 | | | |

### Means for Oneway Anova

| Level | Number | Mean | Std Error | Lower 95% | Upper 95% |
|---|---|---|---|---|---|
| 50nM-NA | 6 | -0.5296 | 0.13809 | -0.812 | -0.248 |
| 5nM-NA | 6 | -0.0567 | 0.13809 | -0.339 | 0.225 |
| -NA | 6 | 1.2681 | 0.13809 | 0.986 | 1.550 |
| 50nM+NA | 6 | -0.8989 | 0.13809 | -1.181 | -0.617 |
| 5nM+NA | 6 | -0.4980 | 0.13809 | -0.780 | -0.216 |
| +NA | 6 | 0.7150 | 0.13809 | 0.433 | 0.997 |

Std Error uses a pooled estimate of error variance

## Means Comparisons

### Comparisons with a control using Dunnett's Method

## Oneway Analysis of octulose-phosphate By Label

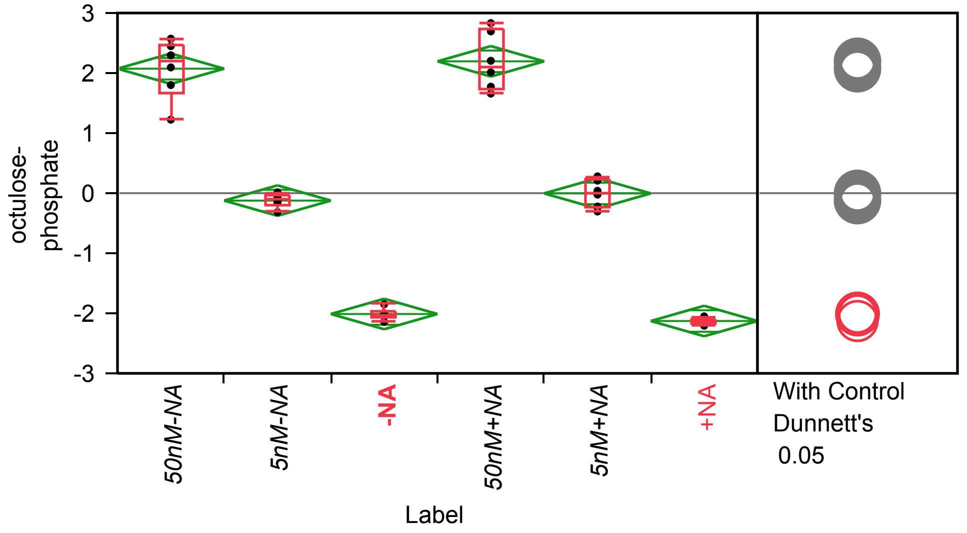


## Quantiles

| Level | Minimum | 10% | 25% | Median | 75% | 90% | Maximum |
|---|---|---|---|---|---|---|---|
| 50nM-NA | 1.224747 | 1.224747 | 1.656478 | 2.195423 | 2.479337 | 2.571939 | 2.571939 |
| 5nM-NA | -0.31623 | -0.31623 | -0.19785 | -0.1054 | -0.0486 | 0.013375 | 0.013375 |
| -NA | -2.14056 | -2.14056 | -2.07016 | -2.01719 | -1.96387 | -1.84932 | -1.84932 |
| 50nM+NA | 1.658176 | 1.658176 | 1.74415 | 2.108096 | 2.728821 | 2.831246 | 2.831246 |
| 5nM+NA | -0.30502 | -0.30502 | -0.24323 | 0.007717 | 0.227063 | 0.279156 | 0.279156 |
| +NA | -2.20626 | -2.20626 | -2.17492 | -2.12687 | -2.08469 | -2.05167 | -2.05167 |

## Oneway Anova

### Summary of Fit

| | |
|---|---|
| Rsquare | 0.974546 |
| Adj Rsquare | 0.970304 |
| Root Mean Square Error | 0.304174 |
| Mean of Response | -5.6e-10 |
| Observations (or Sum Wgts) | 36 |

### Analysis of Variance

| Source | DF | Sum of Squares | Mean Square | F Ratio | Prob > F |
|---|---|---|---|---|---|
| Label | 5 | 106.27025 | 21.2540 | 229.7195 | <.0001 * |
| Error | 30 | 2.77565 | 0.0925 | | |
| C. Total | 35 | 109.04590 | | | |

### Means for Oneway Anova

| Level | Number | Mean | Std Error | Lower 95% | Upper 95% |
|---|---|---|---|---|---|
| 50nM-NA | 6 | 2.0727 | 0.12418 | 1.819 | 2.326 |
| 5nM-NA | 6 | -0.1236 | 0.12418 | -0.377 | 0.130 |
| -NA | 6 | -2.0122 | 0.12418 | -2.266 | -1.759 |
| 50nM+NA | 6 | 2.1955 | 0.12418 | 1.942 | 2.449 |
| 5nM+NA | 6 | -0.0039 | 0.12418 | -0.257 | 0.250 |
| +NA | 6 | -2.1286 | 0.12418 | -2.382 | -1.875 |

Std Error uses a pooled estimate of error variance

## Means Comparisons

### Comparisons with a control using Dunnett's Method

## Oneway Analysis of ornithine By Label

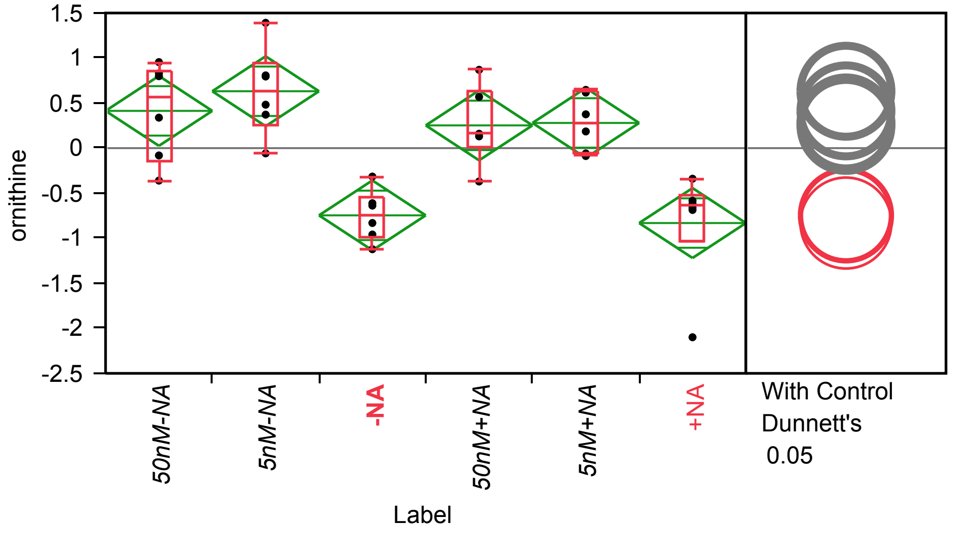


## Quantiles

| Level | Minimum | 10% | 25% | Median | 75% | 90% | Maximum |
|---|---|---|---|---|---|---|---|
| 50nM-NA | -0.36028 | -0.36028 | -0.15084 | 0.56858 | 0.86184 | 0.954999 | 0.954999 |
| 5nM-NA | -0.05774 | -0.05774 | 0.264364 | 0.638646 | 0.953364 | 1.390956 | 1.390956 |
| -NA | -1.12187 | -1.12187 | -0.99983 | -0.7344 | -0.53732 | -0.31567 | -0.31567 |
| 50nM+NA | -0.37021 | -0.37021 | 0.005487 | 0.158533 | 0.644164 | 0.869162 | 0.869162 |
| 5nM+NA | -0.08753 | -0.08753 | -0.06607 | 0.280442 | 0.624445 | 0.646662 | 0.646662 |
| +NA | -2.09943 | -2.09943 | -1.03962 | -0.63985 | -0.52254 | -0.3398 | -0.3398 |

## Oneway Anova

### Summary of Fit

| | |
|---|---|
| Rsquare | 0.644171 |
| Adj Rsquare | 0.584866 |
| Root Mean Square Error | 0.465342 |
| Mean of Response | -1.11e-9 |
| Observations (or Sum Wgts) | 36 |

### Analysis of Variance

| Source | DF | Sum of Squares | Mean Square | F Ratio | Prob > F |
|---|---|---|---|---|---|
| Label | 5 | 11.760472 | 2.35209 | 10.8620 | <.0001 * |
| Error | 30 | 6.496289 | 0.21654 | | |
| C. Total | 35 | 18.256761 | | | |

### Means for Oneway Anova

| Level | Number | Mean | Std Error | Lower 95% | Upper 95% |
|---|---|---|---|---|---|
| 50nM-NA | 6 | 0.41361 | 0.18997 | 0.026 | 0.802 |
| 5nM-NA | 6 | 0.63162 | 0.18997 | 0.244 | 1.020 |
| -NA | 6 | -0.74612 | 0.18997 | -1.134 | -0.358 |
| 50nM+NA | 6 | 0.25265 | 0.18997 | -0.135 | 0.641 |
| 5nM+NA | 6 | 0.27969 | 0.18997 | -0.108 | 0.668 |
| +NA | 6 | -0.83145 | 0.18997 | -1.219 | -0.443 |

Std Error uses a pooled estimate of error variance

## Means Comparisons

### Comparisons with a control using Dunnett's Method

## Oneway Analysis of orotate By Label

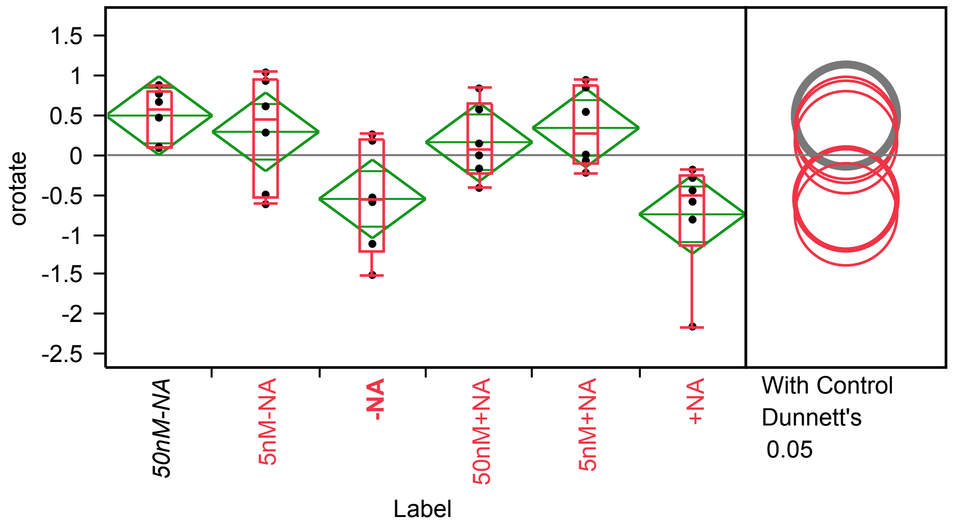


### Quantiles

| Level | Minimum | 10% | 25% | Median | 75% | 90% | Maximum |
|---|---|---|---|---|---|---|---|
| 50nM-NA | 0.090386 | 0.090386 | 0.10034 | 0.569662 | 0.797848 | 0.879819 | 0.879819 |
| 5nM-NA | -0.6171 | -0.6171 | -0.5263 | 0.448569 | 0.95975 | 1.039757 | 1.039757 |
| -NA | -1.50686 | -1.50686 | -1.21329 | -0.56146 | 0.200992 | 0.260315 | 0.260315 |
| 50nM+NA | -0.41159 | -0.41159 | -0.22978 | 0.071532 | 0.640088 | 0.838755 | 0.838755 |
| 5nM+NA | -0.22167 | -0.22167 | -0.10832 | 0.274714 | 0.874039 | 0.944524 | 0.944524 |
| +NA | -2.15493 | -2.15493 | -1.14631 | -0.51489 | -0.26033 | -0.18322 | -0.18322 |

### Oneway Anova

#### Summary of Fit

| | |
|---|---|
| Rsquare | 0.432125 |
| Adj Rsquare | 0.337479 |
| Root Mean Square Error | 0.59194 |
| Mean of Response | 0 |
| Observations (or Sum Wgts) | 36 |

#### Analysis of Variance

| Source | DF | Sum of Squares | Mean Square | F Ratio | Prob > F |
|---|---|---|---|---|---|
| Label | 5 | 7.998948 | 1.59979 | 4.5657 | 0.0033 * |
| Error | 30 | 10.511794 | 0.35039 | | |
| C. Total | 35 | 18.510741 | | | |

#### Means for Oneway Anova

| Level | Number | Mean | Std Error | Lower 95% | Upper 95% |
|---|---|---|---|---|---|
| 50nM-NA | 6 | 0.49729 | 0.24166 | 0.00375 | 0.9908 |
| 5nM-NA | 6 | 0.29281 | 0.24166 | -0.201 | 0.7863 |
| -NA | 6 | -0.55061 | 0.24166 | -1.044 | -0.0571 |
| 50nM+NA | 6 | 0.16249 | 0.24166 | -0.331 | 0.6560 |
| 5nM+NA | 6 | 0.34205 | 0.24166 | -0.151 | 0.8356 |
| +NA | 6 | -0.74401 | 0.24166 | -1.238 | -0.2505 |

Std Error uses a pooled estimate of error variance

### Means Comparisons

#### Comparisons with a control using Dunnett's Method

## Oneway Analysis of Orotidine By Label

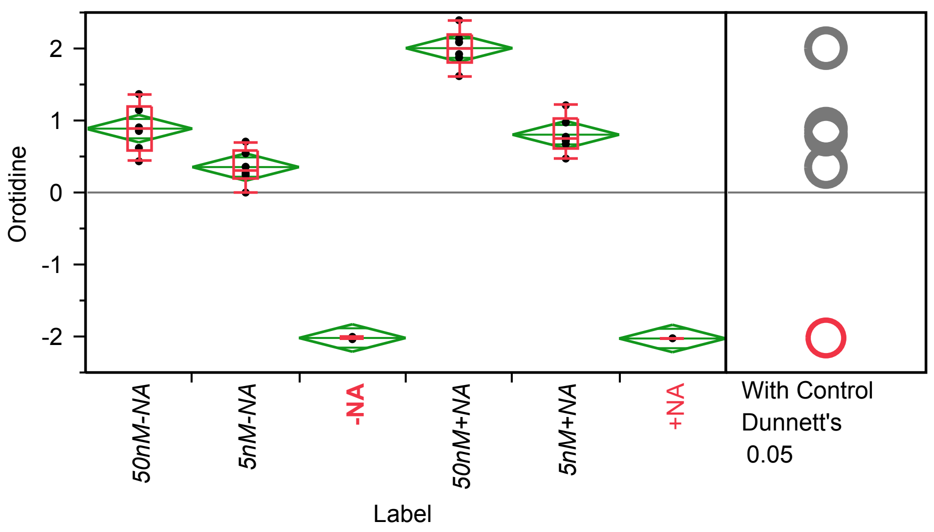


## Quantiles

| Level | Minimum | 10% | 25% | Median | 75% | 90% | Maximum |
|---|---|---|---|---|---|---|---|
| 50nM-NA | 0.433871 | 0.433871 | 0.572553 | 0.879427 | 1.199571 | 1.366525 | 1.366525 |
| 5nM-NA | -0.00023 | -0.00023 | 0.18203 | 0.312903 | 0.584632 | 0.706531 | 0.706531 |
| -NA | -2.03694 | -2.03694 | -2.02602 | -2.01964 | -2.01493 | -2.00528 | -2.00528 |
| 50nM+NA | 1.616391 | 1.616391 | 1.809963 | 2.00413 | 2.201423 | 2.392233 | 2.392233 |
| 5nM+NA | 0.471583 | 0.471583 | 0.621258 | 0.746078 | 1.032284 | 1.210951 | 1.210951 |
| +NA | -2.03404 | -2.03404 | -2.03048 | -2.02647 | -2.0253 | -2.02486 | -2.02486 |

## Oneway Anova

### Summary of Fit

| | |
|---|---|
| Rsquare | 0.981464 |
| Adj Rsquare | 0.978374 |
| Root Mean Square Error | 0.228061 |
| Mean of Response | 5.56e-11 |
| Observations (or Sum Wgts) | 36 |

### Analysis of Variance

| Source | DF | Sum of Squares | Mean Square | F Ratio | Prob > F |
|---|---|---|---|---|---|
| Label | 5 | 82.616967 | 16.5234 | 317.6865 | <.0001 * |
| Error | 30 | 1.560349 | 0.0520 | | |
| C. Total | 35 | 84.177316 | | | |

### Means for Oneway Anova

| Level | Number | Mean | Std Error | Lower 95% | Upper 95% |
|---|---|---|---|---|---|
| 50nM-NA | 6 | 0.8870 | 0.09311 | 0.697 | 1.077 |
| 5nM-NA | 6 | 0.3531 | 0.09311 | 0.163 | 0.543 |
| -NA | 6 | -2.0203 | 0.09311 | -2.210 | -1.830 |
| 50nM+NA | 6 | 2.0049 | 0.09311 | 1.815 | 2.195 |
| 5nM+NA | 6 | 0.8031 | 0.09311 | 0.613 | 0.993 |
| +NA | 6 | -2.0278 | 0.09311 | -2.218 | -1.838 |

Std Error uses a pooled estimate of error variance

## Means Comparisons

### Comparisons with a control using Dunnett's Method

## Oneway Analysis of Palmitic acid By Label

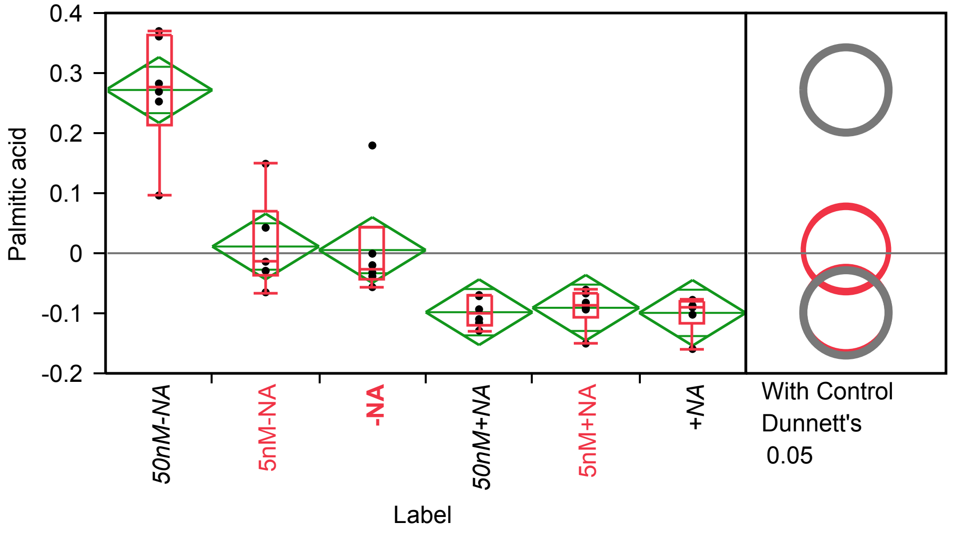


## Quantiles

| Level | Minimum | 10% | 25% | Median | 75% | 90% | Maximum |
|---|---|---|---|---|---|---|---|
| 50nM-NA | 0.09617 | 0.09617 | 0.213489 | 0.27577 | 0.363153 | 0.369836 | 0.369836 |
| 5nM-NA | -0.0651 | -0.0651 | -0.03826 | -0.01489 | 0.069125 | 0.148924 | 0.148924 |
| -NA | -0.05649 | -0.05649 | -0.04292 | -0.02606 | 0.044325 | 0.179439 | 0.179439 |
| 50nM+NA | -0.12895 | -0.12895 | -0.11916 | -0.10156 | -0.07103 | -0.06966 | -0.06966 |
| 5nM+NA | -0.15042 | -0.15042 | -0.10789 | -0.08649 | -0.06548 | -0.06086 | -0.06086 |
| +NA | -0.15943 | -0.15943 | -0.11656 | -0.08884 | -0.07854 | -0.07772 | -0.07772 |

## Oneway Anova

### Summary of Fit

| | |
|---|---|
| Rsquare | 0.82612 |
| Adj Rsquare | 0.79714 |
| Root Mean Square Error | 0.065465 |
| Mean of Response | -5.6e-11 |
| Observations (or Sum Wgts) | 36 |

### Analysis of Variance

| Source | DF | Sum of Squares | Mean Square | F Ratio | Prob > F |
|---|---|---|---|---|---|
| Label | 5 | 0.61085102 | 0.122170 | 28.5066 | <.0001 * |
| Error | 30 | 0.12857062 | 0.004286 | | |
| C. Total | 35 | 0.73942164 | | | |

### Means for Oneway Anova

| Level | Number | Mean | Std Error | Lower 95% | Upper 95% |
|---|---|---|---|---|---|
| 50nM-NA | 6 | 0.27184 | 0.02673 | 0.2173 | 0.3264 |
| 5nM-NA | 6 | 0.01121 | 0.02673 | -0.0434 | 0.0658 |
| -NA | 6 | 0.00529 | 0.02673 | -0.0493 | 0.0599 |
| 50nM+NA | 6 | -0.09819 | 0.02673 | -0.1528 | -0.0436 |
| 5nM+NA | 6 | -0.09083 | 0.02673 | -0.1454 | -0.0363 |
| +NA | 6 | -0.09932 | 0.02673 | -0.1539 | -0.0447 |

Std Error uses a pooled estimate of error variance

## Means Comparisons

### Comparisons with a control using Dunnett's Method

## Oneway Analysis of pantothenate By Label

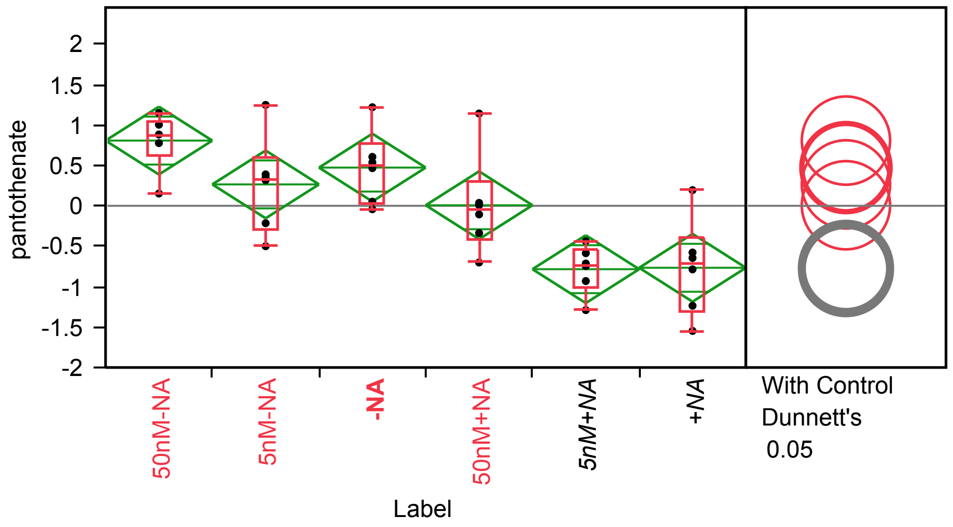


## Quantiles

| Level | Minimum | 10% | 25% | Median | 75% | 90% | Maximum |
|---|---|---|---|---|---|---|---|
| 50nM-NA | 0.152299 | 0.152299 | 0.619909 | 0.879822 | 1.039305 | 1.148697 | 1.148697 |
| 5nM-NA | -0.49981 | -0.49981 | -0.28642 | 0.33363 | 0.60352 | 1.247651 | 1.247651 |
| -NA | -0.0425 | -0.0425 | 0.02766 | 0.501327 | 0.758632 | 1.21896 | 1.21896 |
| 50nM+NA | -0.70109 | -0.70109 | -0.42834 | -0.04897 | 0.312197 | 1.141537 | 1.141537 |
| 5nM+NA | -1.28966 | -1.28966 | -1.02051 | -0.73016 | -0.54891 | -0.43762 | -0.43762 |
| +NA | -1.54738 | -1.54738 | -1.31402 | -0.7156 | -0.38524 | 0.193216 | 0.193216 |

## Oneway Anova

### Summary of Fit

| | |
|---|---|
| Rsquare | 0.629207 |
| Adj Rsquare | 0.567408 |
| Root Mean Square Error | 0.50297 |
| Mean of Response | -5.6e-11 |
| Observations (or Sum Wgts) | 36 |

### Analysis of Variance

| Source | DF | Sum of Squares | Mean Square | F Ratio | Prob > F |
|---|---|---|---|---|---|
| Label | 5 | 12.878557 | 2.57571 | 10.1815 | <.0001 * |
| Error | 30 | 7.589370 | 0.25298 | | |
| C. Total | 35 | 20.467927 | | | |

### Means for Oneway Anova

| Level | Number | Mean | Std Error | Lower 95% | Upper 95% |
|---|---|---|---|---|---|
| 50nM-NA | 6 | 0.80654 | 0.20534 | 0.387 | 1.226 |
| 5nM-NA | 6 | 0.26477 | 0.20534 | -0.155 | 0.684 |
| -NA | 6 | 0.47256 | 0.20534 | 0.053 | 0.892 |
| 50nM+NA | 6 | 0.00681 | 0.20534 | -0.413 | 0.426 |
| 5nM+NA | 6 | -0.78407 | 0.20534 | -1.203 | -0.365 |
| +NA | 6 | -0.76661 | 0.20534 | -1.186 | -0.347 |

Std Error uses a pooled estimate of error variance

## Means Comparisons

### Comparisons with a control using Dunnett's Method

## Oneway Analysis of PC (13:0) By Label

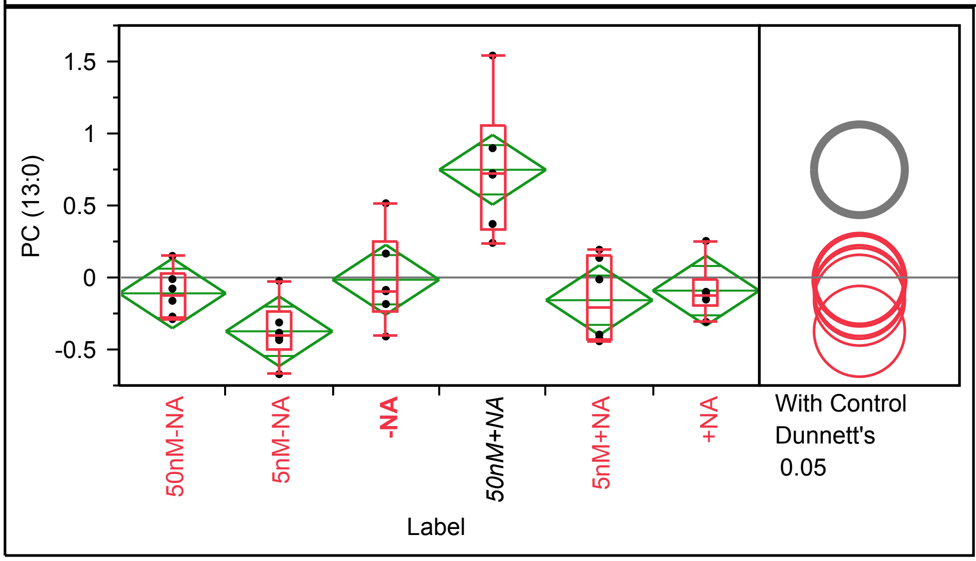


## Quantiles

| Level | Minimum | 10% | 25% | Median | 75% | 90% | Maximum |
|---|---|---|---|---|---|---|---|
| 50nM-NA | -0.28878 | -0.28878 | -0.27569 | -0.11952 | 0.029123 | 0.14999 | 0.14999 |
| 5nM-NA | -0.67121 | -0.67121 | -0.49355 | -0.40013 | -0.2402 | -0.02343 | -0.02343 |
| -NA | -0.40934 | -0.40934 | -0.24051 | -0.09074 | 0.253587 | 0.515477 | 0.515477 |
| 50nM+NA | 0.240384 | 0.240384 | 0.338829 | 0.718746 | 1.058802 | 1.541048 | 1.541048 |
| 5nM+NA | -0.44239 | -0.44239 | -0.42724 | -0.20497 | 0.150193 | 0.192814 | 0.192814 |
| +NA | -0.30814 | -0.30814 | -0.19196 | -0.12029 | -0.01121 | 0.253954 | 0.253954 |

## Oneway Anova

### Summary of Fit

| | |
|---|---|
| Rsquare | 0.638318 |
| Adj Rsquare | 0.578038 |
| Root Mean Square Error | 0.290517 |
| Mean of Response | 5.56e-10 |
| Observations (or Sum Wgts) | 36 |

### Analysis of Variance

| Source | DF | Sum of Squares | Mean Square | F Ratio | Prob > F |
|---|---|---|---|---|---|
| Label | 5 | 4.4686458 | 0.893729 | 10.5892 | <.0001 * |
| Error | 30 | 2.5320083 | 0.084400 | | |
| C. Total | 35 | 7.0006542 | | | |

### Means for Oneway Anova

| Level | Number | Mean | Std Error | Lower 95% | Upper 95% |
|---|---|---|---|---|---|
| 50nM-NA | 6 | -0.11005 | 0.11860 | -0.3523 | 0.1322 |
| 5nM-NA | 6 | -0.37362 | 0.11860 | -0.6158 | -0.1314 |
| -NA | 6 | -0.01555 | 0.11860 | -0.2578 | 0.2267 |
| 50nM+NA | 6 | 0.74810 | 0.11860 | 0.5059 | 0.9903 |
| 5nM+NA | 6 | -0.15762 | 0.11860 | -0.3998 | 0.0846 |
| +NA | 6 | -0.09127 | 0.11860 | -0.3335 | 0.1510 |

Std Error uses a pooled estimate of error variance

## Means Comparisons

### Comparisons with a control using Dunnett's Method

## Oneway Analysis of PC (14:0) By Label

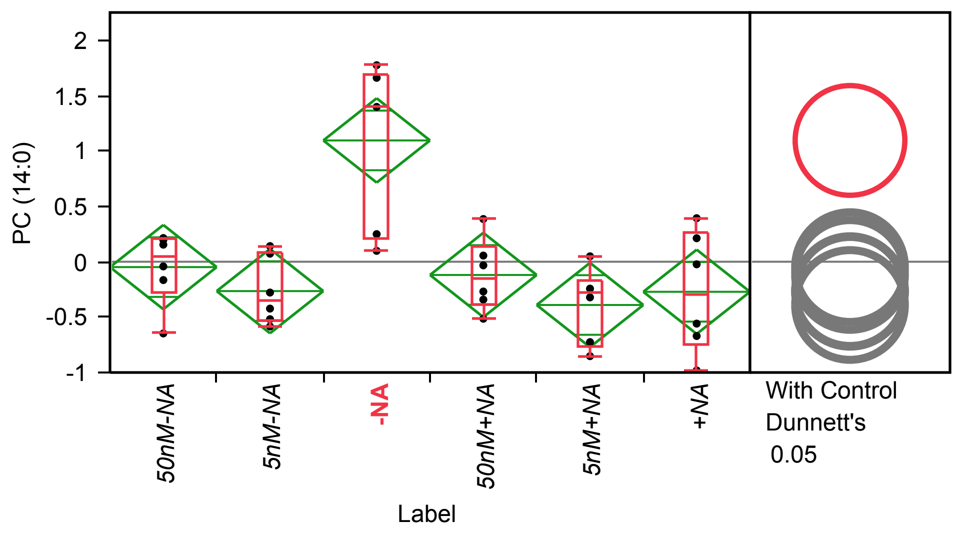


### Quantiles

| Level | Minimum | 10% | 25% | Median | 75% | 90% | Maximum |
|---|---|---|---|---|---|---|---|
| 50nM-NA | -0.6472 | -0.6472 | -0.28596 | 0.055845 | 0.200989 | 0.213768 | 0.213768 |
| 5nM-NA | -0.58425 | -0.58425 | -0.53517 | -0.35071 | 0.089793 | 0.141167 | 0.141167 |
| -NA | 0.098294 | 0.098294 | 0.211379 | 1.394311 | 1.688748 | 1.774035 | 1.774035 |
| 50nM+NA | -0.51453 | -0.51453 | -0.38509 | -0.15088 | 0.137973 | 0.385407 | 0.385407 |
| 5nM+NA | -0.85234 | -0.85234 | -0.75739 | -0.28537 | -0.16998 | 0.048913 | 0.048913 |
| +NA | -0.98147 | -0.98147 | -0.74826 | -0.29107 | 0.257371 | 0.393015 | 0.393015 |

### Oneway Anova

#### Summary of Fit

| | |
|---|---|
| Rsquare | 0.592104 |
| Adj Rsquare | 0.524122 |
| Root Mean Square Error | 0.45646 |
| Mean of Response | -2.8e-10 |
| Observations (or Sum Wgts) | 36 |

#### Analysis of Variance

| Source | DF | Sum of Squares | Mean Square | F Ratio | Prob > F |
|---|---|---|---|---|---|
| Label | 5 | 9.073509 | 1.81470 | 8.7096 | <.0001 * |
| Error | 30 | 6.250663 | 0.20836 | | |
| C. Total | 35 | 15.324171 | | | |

#### Means for Oneway Anova

| Level | Number | Mean | Std Error | Lower 95% | Upper 95% |
|---|---|---|---|---|---|
| 50nM-NA | 6 | -0.0484 | 0.18635 | -0.4290 | 0.332 |
| 5nM-NA | 6 | -0.2651 | 0.18635 | -0.6457 | 0.115 |
| -NA | 6 | 1.0951 | 0.18635 | 0.7145 | 1.476 |
| 50nM+NA | 6 | -0.1196 | 0.18635 | -0.5001 | 0.261 |
| 5nM+NA | 6 | -0.3905 | 0.18635 | -0.7711 | -0.0099 |
| +NA | 6 | -0.2715 | 0.18635 | -0.6521 | 0.109 |

Std Error uses a pooled estimate of error variance

### Means Comparisons

#### Comparisons with a control using Dunnett's Method

## Oneway Analysis of PC (15:1) By Label

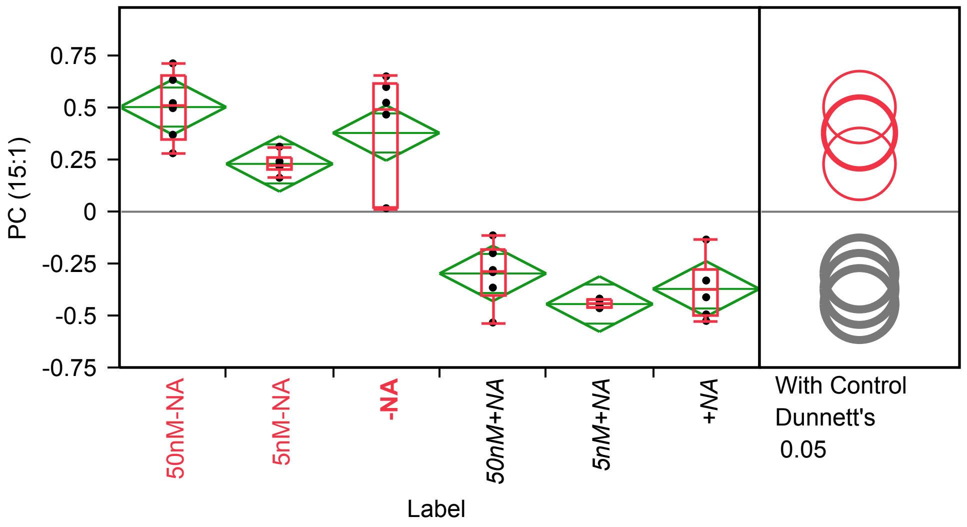


### Quantiles

| Level | Minimum | 10% | 25% | Median | 75% | 90% | Maximum |
|---|---|---|---|---|---|---|---|
| 50nM-NA | 0.281556 | 0.281556 | 0.348271 | 0.51097 | 0.654648 | 0.71442 | 0.71442 |
| 5nM-NA | 0.163423 | 0.163423 | 0.203834 | 0.223143 | 0.259941 | 0.313095 | 0.313095 |
| -NA | 0.013256 | 0.013256 | 0.015944 | 0.496435 | 0.613402 | 0.651854 | 0.651854 |
| 50nM+NA | -0.53335 | -0.53335 | -0.40737 | -0.28582 | -0.17807 | -0.11372 | -0.11372 |
| 5nM+NA | -0.46572 | -0.46572 | -0.46475 | -0.44663 | -0.42398 | -0.41842 | -0.41842 |
| +NA | -0.52553 | -0.52553 | -0.50209 | -0.37224 | -0.28195 | -0.13409 | -0.13409 |

### Oneway Anova

#### Summary of Fit

| | |
|---|---|
| Rsquare | 0.872917 |
| Adj Rsquare | 0.851736 |
| Root Mean Square Error | 0.159608 |
| Mean of Response | 0 |
| Observations (or Sum Wgts) | 36 |

#### Analysis of Variance

| Source | DF | Sum of Squares | Mean Square | F Ratio | Prob > F |
|---|---|---|---|---|---|
| Label | 5 | 5.2494317 | 1.04989 | 41.2131 | <.0001 * |
| Error | 30 | 0.7642382 | 0.02547 | | |
| C. Total | 35 | 6.0136699 | | | |

#### Means for Oneway Anova

| Level | Number | Mean | Std Error | Lower 95% | Upper 95% |
|---|---|---|---|---|---|
| 50nM-NA | 6 | 0.50386 | 0.06516 | 0.3708 | 0.6369 |
| 5nM-NA | 6 | 0.23039 | 0.06516 | 0.0973 | 0.3635 |
| -NA | 6 | 0.37923 | 0.06516 | 0.2462 | 0.5123 |
| 50nM+NA | 6 | -0.29727 | 0.06516 | -0.4303 | -0.1642 |
| 5nM+NA | 6 | -0.44461 | 0.06516 | -0.5777 | -0.3115 |
| +NA | 6 | -0.37160 | 0.06516 | -0.5047 | -0.2385 |

Std Error uses a pooled estimate of error variance

### Means Comparisons

#### Comparisons with a control using Dunnett's Method

## Oneway Analysis of PC(15:0) By Label

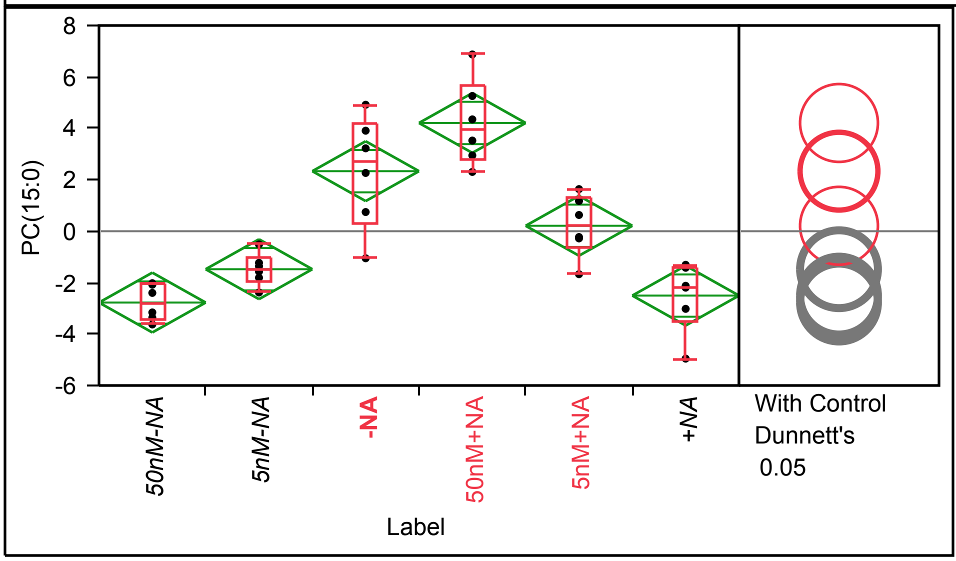


### Quantiles

| Level | Minimum | 10% | 25% | Median | 75% | 90% | Maximum |
|---|---|---|---|---|---|---|---|
| 50nM-NA | -3.62606 | -3.62606 | -3.42624 | -2.77871 | -2.06592 | -2.02123 | -2.02123 |
| 5nM-NA | -2.38262 | -2.38262 | -1.95178 | -1.46651 | -1.0499 | -0.51418 | -0.51418 |
| -NA | -1.05182 | -1.05182 | 0.296243 | 2.744888 | 4.160696 | 4.917106 | 4.917106 |
| 50nM+NA | 2.305569 | 2.305569 | 2.786148 | 3.937029 | 5.661365 | 6.873391 | 6.873391 |
| 5nM+NA | -1.67103 | -1.67103 | -0.62736 | 0.202492 | 1.290179 | 1.637127 | 1.637127 |
| +NA | -4.95535 | -4.95535 | -3.50298 | -2.15892 | -1.39274 | -1.3071 | -1.3071 |

### Oneway Anova

#### Summary of Fit

| | |
|---|---|
| Rsquare | 0.801707 |
| Adj Rsquare | 0.768659 |
| Root Mean Square Error | 1.395411 |
| Mean of Response | -5.56e-9 |
| Observations (or Sum Wgts) | 36 |

#### Analysis of Variance

| Source | DF | Sum of Squares | Mean Square | F Ratio | Prob > F |
|---|---|---|---|---|---|
| Label | 5 | 236.17538 | 47.2351 | 24.2583 | <.0001 * |
| Error | 30 | 58.41512 | 1.9472 | | |
| C. Total | 35 | 294.59051 | | | |

#### Means for Oneway Anova

| Level | Number | Mean | Std Error | Lower 95% | Upper 95% |
|---|---|---|---|---|---|
| 50nM-NA | 6 | -2.7742 | 0.56967 | -3.938 | -1.611 |
| 5nM-NA | 6 | -1.4777 | 0.56967 | -2.641 | -0.314 |
| -NA | 6 | 2.3349 | 0.56967 | 1.171 | 3.498 |
| 50nM+NA | 6 | 4.2095 | 0.56967 | 3.046 | 5.373 |
| 5nM+NA | 6 | 0.2110 | 0.56967 | -0.952 | 1.374 |
| +NA | 6 | -2.5034 | 0.56967 | -3.667 | -1.340 |

Std Error uses a pooled estimate of error variance

### Means Comparisons

#### Comparisons with a control using Dunnett's Method

## Oneway Analysis of PC(16:1) By Label

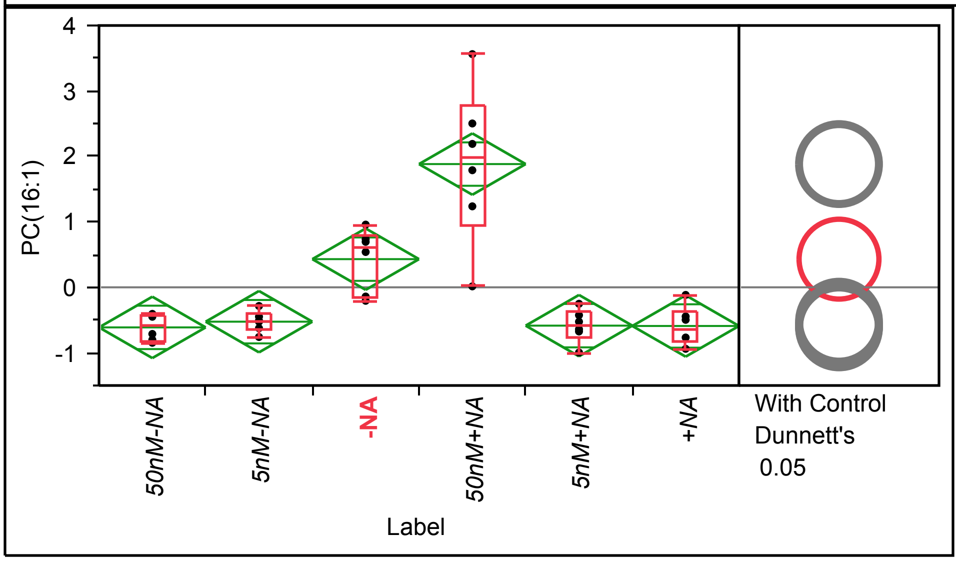


### Quantiles

| Level | Minimum | 10% | 25% | Median | 75% | 90% | Maximum |
|---|---|---|---|---|---|---|---|
| 50nM-NA | -0.84711 | -0.84711 | -0.81266 | -0.58453 | -0.43884 | -0.40586 | -0.40586 |
| 5nM-NA | -0.75901 | -0.75901 | -0.65807 | -0.51666 | -0.40738 | -0.28535 | -0.28535 |
| -NA | -0.20562 | -0.20562 | -0.15731 | 0.617295 | 0.792318 | 0.957695 | 0.957695 |
| 50nM+NA | 0.014647 | 0.014647 | 0.931618 | 1.989932 | 2.767945 | 3.562421 | 3.562421 |
| 5nM+NA | -0.99598 | -0.99598 | -0.75492 | -0.58248 | -0.38223 | -0.25172 | -0.25172 |
| +NA | -0.93923 | -0.93923 | -0.81968 | -0.63171 | -0.36286 | -0.11627 | -0.11627 |

### Oneway Anova

#### Summary of Fit

| | |
|---|---|
| Rsquare | 0.762354 |
| Adj Rsquare | 0.722746 |
| Root Mean Square Error | 0.562364 |
| Mean of Response | -5.6e-10 |
| Observations (or Sum Wgts) | 36 |

#### Analysis of Variance

| Source | DF | Sum of Squares | Mean Square | F Ratio | Prob > F |
|---|---|---|---|---|---|
| Label | 5 | 30.435656 | 6.08713 | 19.2477 | <.0001 * |
| Error | 30 | 9.487596 | 0.31625 | | |
| C. Total | 35 | 39.923253 | | | |

#### Means for Oneway Anova

| Level | Number | Mean | Std Error | Lower 95% | Upper 95% |
|---|---|---|---|---|---|
| 50nM-NA | 6 | -0.6122 | 0.22958 | -1.081 | -0.143 |
| 5nM-NA | 6 | -0.5250 | 0.22958 | -0.994 | -0.056 |
| -NA | 6 | 0.4304 | 0.22958 | -0.038 | 0.899 |
| 50nM+NA | 6 | 1.8829 | 0.22958 | 1.414 | 2.352 |
| 5nM+NA | 6 | -0.5855 | 0.22958 | -1.054 | -0.117 |
| +NA | 6 | -0.5906 | 0.22958 | -1.060 | -0.122 |

Std Error uses a pooled estimate of error variance

### Means Comparisons

#### Comparisons with a control using Dunnett's Method

## Oneway Analysis of PC(17:1) By Label

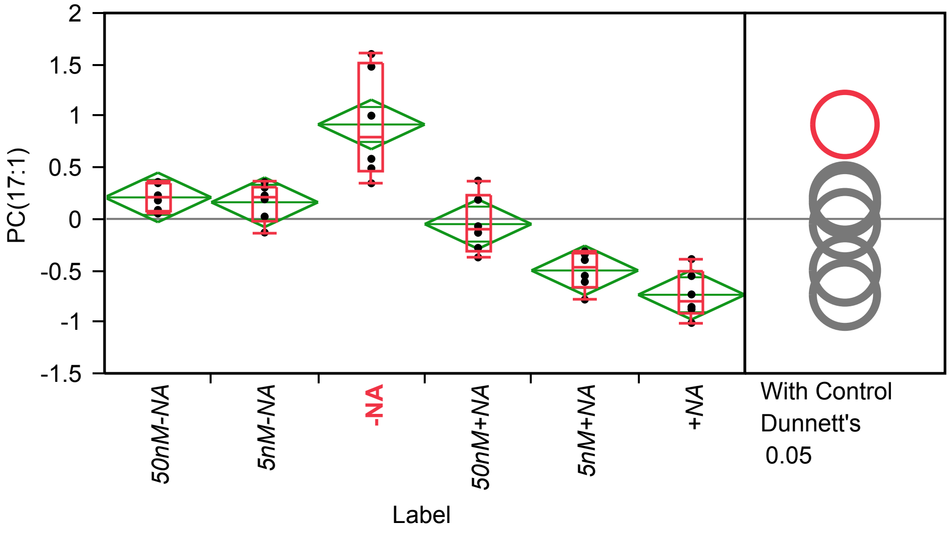


### Quantiles

| Level | Minimum | 10% | 25% | Median | 75% | 90% | Maximum |
|---|---|---|---|---|---|---|---|
| 50nM-NA | 0.052221 | 0.052221 | 0.078403 | 0.204219 | 0.350232 | 0.358904 | 0.358904 |
| 5nM-NA | -0.13216 | -0.13216 | -0.01588 | 0.208972 | 0.317732 | 0.357187 | 0.357187 |
| -NA | 0.346088 | 0.346088 | 0.454962 | 0.793062 | 1.509326 | 1.601554 | 1.601554 |
| 50nM+NA | -0.37383 | -0.37383 | -0.30457 | -0.10284 | 0.23266 | 0.371895 | 0.371895 |
| 5nM+NA | -0.78313 | -0.78313 | -0.65419 | -0.47552 | -0.33683 | -0.3152 | -0.3152 |
| +NA | -1.01028 | -1.01028 | -0.91036 | -0.79419 | -0.51354 | -0.39027 | -0.39027 |

### Oneway Anova

#### Summary of Fit

| | |
|---|---|
| Rsquare | 0.804653 |
| Adj Rsquare | 0.772095 |
| Root Mean Square Error | 0.287923 |
| Mean of Response | 0 |
| Observations (or Sum Wgts) | 36 |

#### Analysis of Variance

| Source | DF | Sum of Squares | Mean Square | F Ratio | Prob > F |
|---|---|---|---|---|---|
| Label | 5 | 10.244183 | 2.04884 | 24.7146 | <.0001 * |
| Error | 30 | 2.486996 | 0.08290 | | |
| C. Total | 35 | 12.731179 | | | |

#### Means for Oneway Anova

| Level | Number | Mean | Std Error | Lower 95% | Upper 95% |
|---|---|---|---|---|---|
| 50nM-NA | 6 | 0.20901 | 0.11754 | -0.0311 | 0.449 |
| 5nM-NA | 6 | 0.16174 | 0.11754 | -0.0783 | 0.402 |
| -NA | 6 | 0.91727 | 0.11754 | 0.6772 | 1.157 |
| 50nM+NA | 6 | -0.05047 | 0.11754 | -0.2905 | 0.190 |
| 5nM+NA | 6 | -0.50077 | 0.11754 | -0.7408 | -0.261 |
| +NA | 6 | -0.73677 | 0.11754 | -0.9768 | -0.497 |

Std Error uses a pooled estimate of error variance

### Means Comparisons

#### Comparisons with a control using Dunnett's Method

## Oneway Analysis of PC(17:2) By Label

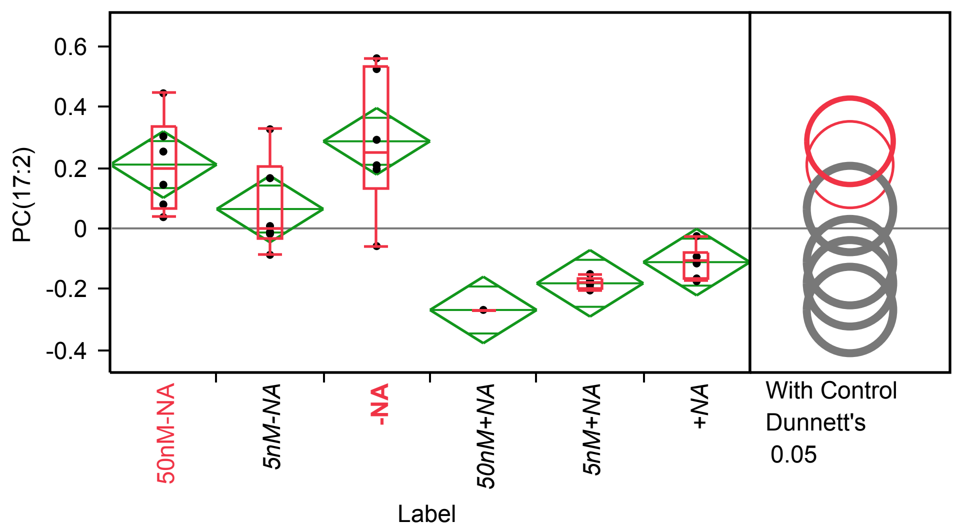


## Quantiles

| Level | Minimum | 10% | 25% | Median | 75% | 90% | Maximum |
|---|---|---|---|---|---|---|---|
| 50nM-NA | 0.037015 | 0.037015 | 0.068344 | 0.198104 | 0.337964 | 0.444397 | 0.444397 |
| 5nM-NA | -0.08702 | -0.08702 | -0.03523 | -0.00212 | 0.205457 | 0.326074 | 0.326074 |
| -NA | -0.05826 | -0.05826 | 0.131352 | 0.249463 | 0.532806 | 0.559442 | 0.559442 |
| 50nM+NA | -0.26856 | -0.26856 | -0.26845 | -0.26835 | -0.26816 | -0.26816 | -0.26816 |
| 5nM+NA | -0.20412 | -0.20412 | -0.19775 | -0.1807 | -0.1669 | -0.15003 | -0.15003 |
| +NA | -0.17369 | -0.17369 | -0.16686 | -0.1046 | -0.07611 | -0.02542 | -0.02542 |

## Oneway Anova

### Summary of Fit

| | |
|---|---|
| Rsquare | 0.742067 |
| Adj Rsquare | 0.699078 |
| Root Mean Square Error | 0.131054 |
| Mean of Response | -2.8e-11 |
| Observations (or Sum Wgts) | 36 |

### Analysis of Variance

| Source | DF | Sum of Squares | Mean Square | F Ratio | Prob > F |
|---|---|---|---|---|---|
| Label | 5 | 1.4823787 | 0.296476 | 17.2618 | <.0001 * |
| Error | 30 | 0.5152568 | 0.017175 | | |
| C. Total | 35 | 1.9976356 | | | |

### Means for Oneway Anova

| Level | Number | Mean | Std Error | Lower 95% | Upper 95% |
|---|---|---|---|---|---|
| 50nM-NA | 6 | 0.20982 | 0.05350 | 0.1005 | 0.3191 |
| 5nM-NA | 6 | 0.06368 | 0.05350 | -0.0456 | 0.1730 |
| -NA | 6 | 0.28643 | 0.05350 | 0.1772 | 0.3957 |
| 50nM+NA | 6 | -0.26833 | 0.05350 | -0.3776 | -0.1591 |
| 5nM+NA | 6 | -0.18062 | 0.05350 | -0.2899 | -0.0714 |
| +NA | 6 | -0.11098 | 0.05350 | -0.2203 | -0.0017 |

Std Error uses a pooled estimate of error variance

## Means Comparisons

### Comparisons with a control using Dunnett's Method

## Oneway Analysis of p-coumaric acid By Label

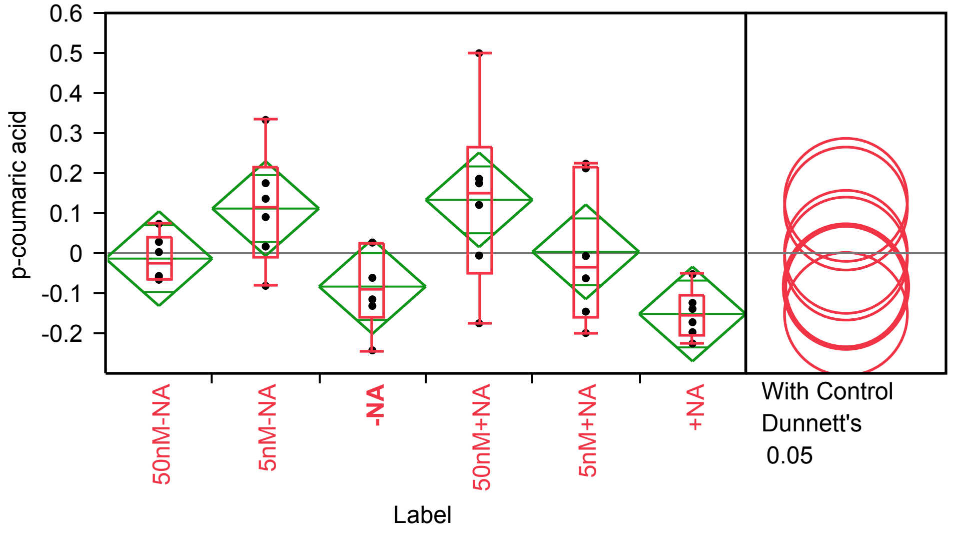


### Quantiles

| Level | Minimum | 10% | 25% | Median | 75% | 90% | Maximum |
|---|---|---|---|---|---|---|---|
| 50nM-NA | -0.06622 | -0.06622 | -0.06308 | -0.02701 | 0.039582 | 0.073696 | 0.073696 |
| 5nM-NA | -0.08132 | -0.08132 | -0.00777 | 0.113216 | 0.214434 | 0.332856 | 0.332856 |
| -NA | -0.24252 | -0.24252 | -0.15935 | -0.08831 | 0.02505 | 0.026568 | 0.026568 |
| 50nM+NA | -0.17491 | -0.17491 | -0.04811 | 0.14761 | 0.264325 | 0.499533 | 0.499533 |
| 5nM+NA | -0.19917 | -0.19917 | -0.15921 | -0.0348 | 0.214857 | 0.223523 | 0.223523 |
| +NA | -0.22545 | -0.22545 | -0.20426 | -0.15568 | -0.10612 | -0.05235 | -0.05235 |

### Oneway Anova

#### Summary of Fit

| | |
|---|---|
| Rsquare | 0.375969 |
| Adj Rsquare | 0.271963 |
| Root Mean Square Error | 0.141578 |
| Mean of Response | -2.8e-11 |
| Observations (or Sum Wgts) | 36 |

#### Analysis of Variance

| Source | DF | Sum of Squares | Mean Square | F Ratio | Prob > F |
|---|---|---|---|---|---|
| Label | 5 | 0.36229182 | 0.072458 | 3.6149 | 0.0112 * |
| Error | 30 | 0.60133086 | 0.020044 | | |
| C. Total | 35 | 0.96362267 | | | |

#### Means for Oneway Anova

| Level | Number | Mean | Std Error | Lower 95% | Upper 95% |
|---|---|---|---|---|---|
| 50nM-NA | 6 | -0.01339 | 0.05780 | -0.1314 | 0.1046 |
| 5nM-NA | 6 | 0.11161 | 0.05780 | -0.0064 | 0.2297 |
| -NA | 6 | -0.08328 | 0.05780 | -0.2013 | 0.0348 |
| 50nM+NA | 6 | 0.13332 | 0.05780 | 0.0153 | 0.2514 |
| 5nM+NA | 6 | 0.00347 | 0.05780 | -0.1146 | 0.1215 |
| +NA | 6 | -0.15173 | 0.05780 | -0.2698 | -0.0337 |

Std Error uses a pooled estimate of error variance

### Means Comparisons

#### Comparisons with a control using Dunnett's Method

## Oneway Analysis of Pentadecanal By Label

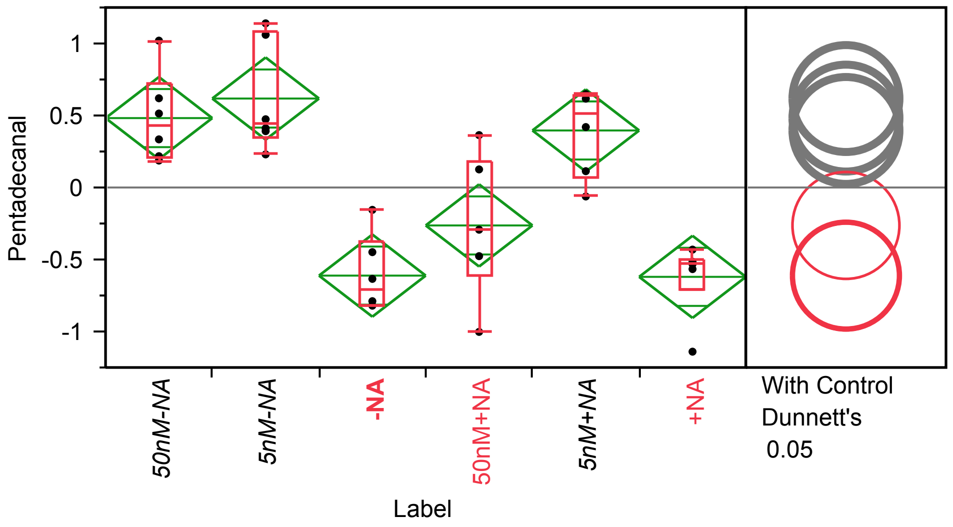


## Quantiles

| Level | Minimum | 10% | 25% | Median | 75% | 90% | Maximum |
|---|---|---|---|---|---|---|---|
| 50nM-NA | 0.187142 | 0.187142 | 0.209815 | 0.423916 | 0.719713 | 1.019773 | 1.019773 |
| 5nM-NA | 0.229972 | 0.229972 | 0.350967 | 0.442608 | 1.080268 | 1.139939 | 1.139939 |
| -NA | -0.82261 | -0.82261 | -0.82108 | -0.712 | -0.3755 | -0.15536 | -0.15536 |
| 50nM+NA | -1.00263 | -1.00263 | -0.60816 | -0.29545 | 0.185156 | 0.363859 | 0.363859 |
| 5nM+NA | -0.06227 | -0.06227 | 0.06943 | 0.518273 | 0.64246 | 0.648473 | 0.648473 |
| +NA | -1.14017 | -1.14017 | -0.71002 | -0.53457 | -0.49457 | -0.43143 | -0.43143 |

## Oneway Anova

### Summary of Fit

| | |
|---|---|
| Rsquare | 0.732089 |
| Adj Rsquare | 0.687437 |
| Root Mean Square Error | 0.342176 |
| Mean of Response | 5.56e-10 |
| Observations (or Sum Wgts) | 36 |

### Analysis of Variance

| Source | DF | Sum of Squares | Mean Square | F Ratio | Prob > F |
|---|---|---|---|---|---|
| Label | 5 | 9.598275 | 1.91965 | 16.3955 | <.0001 * |
| Error | 30 | 3.512529 | 0.11708 | | |
| C. Total | 35 | 13.110804 | | | |

### Means for Oneway Anova

| Level | Number | Mean | Std Error | Lower 95% | Upper 95% |
|---|---|---|---|---|---|
| 50nM-NA | 6 | 0.48197 | 0.13969 | 0.1967 | 0.7673 |
| 5nM-NA | 6 | 0.61780 | 0.13969 | 0.3325 | 0.9031 |
| -NA | 6 | -0.61190 | 0.13969 | -0.8972 | -0.3266 |
| 50nM+NA | 6 | -0.26346 | 0.13969 | -0.5487 | 0.0218 |
| 5nM+NA | 6 | 0.39609 | 0.13969 | 0.1108 | 0.6814 |
| +NA | 6 | -0.62050 | 0.13969 | -0.9058 | -0.3352 |

Std Error uses a pooled estimate of error variance

## Means Comparisons

### Comparisons with a control using Dunnett's Method

## Oneway Analysis of phenylalanine By Label

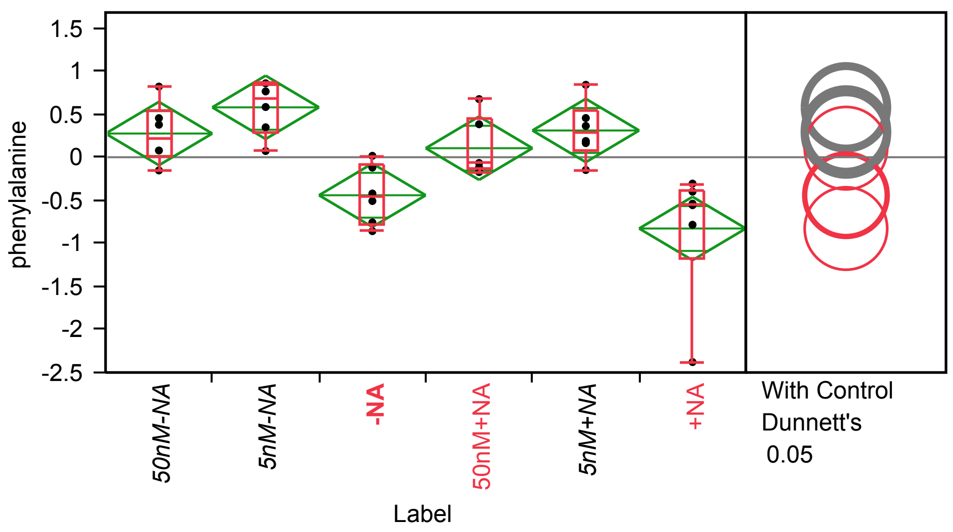


## Quantiles

| Level | Minimum | 10% | 25% | Median | 75% | 90% | Maximum |
|---|---|---|---|---|---|---|---|
| 50nM-NA | -0.15326 | -0.15326 | 0.017205 | 0.227536 | 0.5451 | 0.819849 | 0.819849 |
| 5nM-NA | 0.072142 | 0.072142 | 0.278425 | 0.67346 | 0.853108 | 0.85819 | 0.85819 |
| -NA | -0.85816 | -0.85816 | -0.7826 | -0.46515 | -0.08611 | 0.016003 | 0.016003 |
| 50nM+NA | -0.17207 | -0.17207 | -0.13245 | -0.06879 | 0.455893 | 0.674616 | 0.674616 |
| 5nM+NA | -0.14823 | -0.14823 | 0.085781 | 0.275682 | 0.551398 | 0.8406 | 0.8406 |
| +NA | -2.37867 | -2.37867 | -1.1831 | -0.54831 | -0.3765 | -0.30661 | -0.30661 |

## Oneway Anova

### Summary of Fit

| | |
|---|---|
| Rsquare | 0.589293 |
| Adj Rsquare | 0.520841 |
| Root Mean Square Error | 0.441543 |
| Mean of Response | -2.8e-10 |
| Observations (or Sum Wgts) | 36 |

### Analysis of Variance

| Source | DF | Sum of Squares | Mean Square | F Ratio | Prob > F |
|---|---|---|---|---|---|
| Label | 5 | 8.391999 | 1.67840 | 8.6089 | <.0001 * |
| Error | 30 | 5.848800 | 0.19496 | | |
| C. Total | 35 | 14.240799 | | | |

### Means for Oneway Anova

| Level | Number | Mean | Std Error | Lower 95% | Upper 95% |
|---|---|---|---|---|---|
| 50nM-NA | 6 | 0.27487 | 0.18026 | -0.093 | 0.6430 |
| 5nM-NA | 6 | 0.57931 | 0.18026 | 0.211 | 0.9474 |
| -NA | 6 | -0.44167 | 0.18026 | -0.810 | -0.0735 |
| 50nM+NA | 6 | 0.10478 | 0.18026 | -0.263 | 0.4729 |
| 5nM+NA | 6 | 0.31042 | 0.18026 | -0.058 | 0.6786 |
| +NA | 6 | -0.82771 | 0.18026 | -1.196 | -0.4596 |

Std Error uses a pooled estimate of error variance

## Means Comparisons

### Comparisons with a control using Dunnett's Method

## Oneway Analysis of Phenyllactic acid By Label

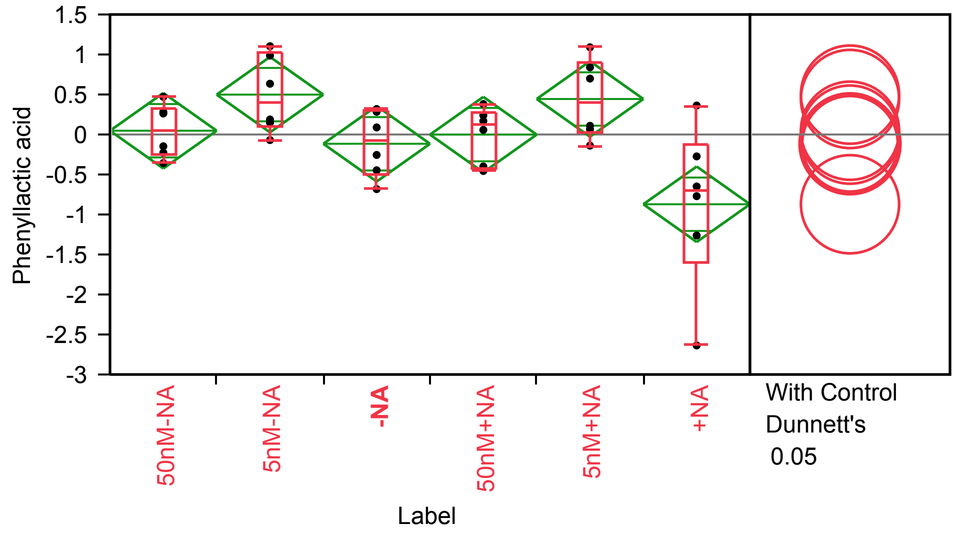


## Quantiles

| Level | Minimum | 10% | 25% | Median | 75% | 90% | Maximum |
|---|---|---|---|---|---|---|---|
| 50nM-NA | -0.35702 | -0.35702 | -0.25939 | 0.057623 | 0.328391 | 0.470482 | 0.470482 |
| 5nM-NA | -0.06938 | -0.06938 | 0.093769 | 0.410891 | 1.01563 | 1.103634 | 1.103634 |
| -NA | -0.68255 | -0.68255 | -0.50613 | -0.08357 | 0.292036 | 0.317403 | 0.317403 |
| 50nM+NA | -0.45563 | -0.45563 | -0.41374 | 0.113248 | 0.27458 | 0.380871 | 0.380871 |
| 5nM+NA | -0.13917 | -0.13917 | 0.014328 | 0.403603 | 0.902166 | 1.089904 | 1.089904 |
| +NA | -2.63667 | -2.63667 | -1.60616 | -0.71026 | -0.11475 | 0.362072 | 0.362072 |

## Oneway Anova

### Summary of Fit

| | |
|---|---|
| Rsquare | 0.432784 |
| Adj Rsquare | 0.338248 |
| Root Mean Square Error | 0.565807 |
| Mean of Response | 0 |
| Observations (or Sum Wgts) | 36 |

### Analysis of Variance

| Source | DF | Sum of Squares | Mean Square | F Ratio | Prob > F |
|---|---|---|---|---|---|
| Label | 5 | 7.327931 | 1.46559 | 4.5780 | 0.0032 * |
| Error | 30 | 9.604135 | 0.32014 | | |
| C. Total | 35 | 16.932066 | | | |

### Means for Oneway Anova

| Level | Number | Mean | Std Error | Lower 95% | Upper 95% |
|---|---|---|---|---|---|
| 50nM-NA | 6 | 0.04715 | 0.23099 | -0.425 | 0.5189 |
| 5nM-NA | 6 | 0.49841 | 0.23099 | 0.027 | 0.9702 |
| -NA | 6 | -0.11600 | 0.23099 | -0.588 | 0.3557 |
| 50nM+NA | 6 | -0.00148 | 0.23099 | -0.473 | 0.4703 |
| 5nM+NA | 6 | 0.44384 | 0.23099 | -0.028 | 0.9156 |
| +NA | 6 | -0.87191 | 0.23099 | -1.344 | -0.4002 |

Std Error uses a pooled estimate of error variance

## Means Comparisons

### Comparisons with a control using Dunnett's Method

## Oneway Analysis of Phosphate By Label

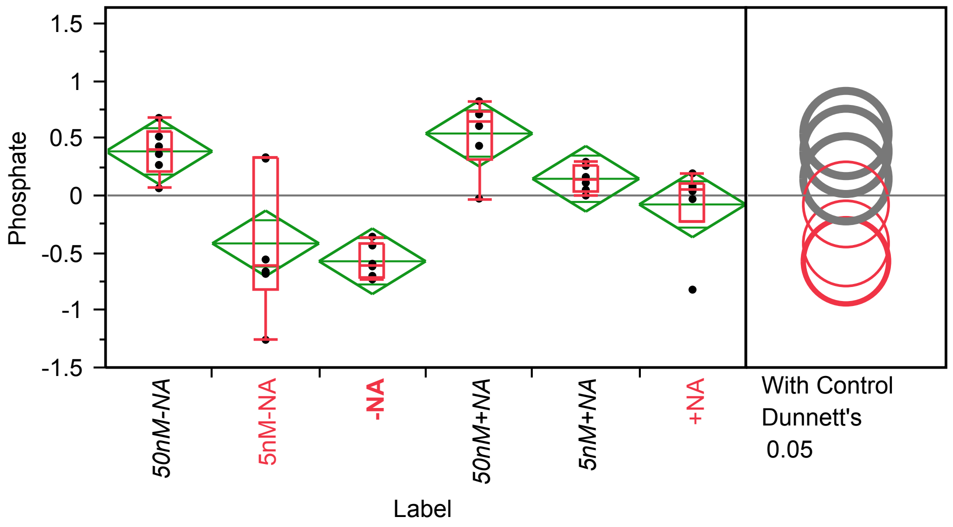


### Quantiles

| Level | Minimum | 10% | 25% | Median | 75% | 90% | Maximum |
|---|---|---|---|---|---|---|---|
| 50nM-NA | 0.063032 | 0.063032 | 0.215231 | 0.39455 | 0.553783 | 0.676341 | 0.676341 |
| 5nM-NA | -1.25844 | -1.25844 | -0.82716 | -0.61039 | 0.324118 | 0.332706 | 0.332706 |
| -NA | -0.73178 | -0.73178 | -0.70888 | -0.60707 | -0.41698 | -0.35981 | -0.35981 |
| 50nM+NA | -0.02733 | -0.02733 | 0.317877 | 0.653252 | 0.735967 | 0.822233 | 0.822233 |
| 5nM+NA | -0.0007 | -0.0007 | 0.036754 | 0.135788 | 0.267471 | 0.293476 | 0.293476 |
| +NA | -0.82129 | -0.82129 | -0.22897 | 0.052859 | 0.111608 | 0.192947 | 0.192947 |

### Oneway Anova

#### Summary of Fit

| | |
|---|---|
| Rsquare | 0.623185 |
| Adj Rsquare | 0.560382 |
| Root Mean Square Error | 0.342698 |
| Mean of Response | -2.8e-11 |
| Observations (or Sum Wgts) | 36 |

#### Analysis of Variance

| Source | DF | Sum of Squares | Mean Square | F Ratio | Prob > F |
|---|---|---|---|---|---|
| Label | 5 | 5.8268306 | 1.16537 | 9.9229 | <.0001 * |
| Error | 30 | 3.5232564 | 0.11744 | | |
| C. Total | 35 | 9.3500870 | | | |

#### Means for Oneway Anova

| Level | Number | Mean | Std Error | Lower 95% | Upper 95% |
|---|---|---|---|---|---|
| 50nM-NA | 6 | 0.38456 | 0.13991 | 0.0988 | 0.6703 |
| 5nM-NA | 6 | -0.41811 | 0.13991 | -0.7038 | -0.1324 |
| -NA | 6 | -0.57383 | 0.13991 | -0.8596 | -0.2881 |
| 50nM+NA | 6 | 0.54026 | 0.13991 | 0.2545 | 0.8260 |
| 5nM+NA | 6 | 0.14540 | 0.13991 | -0.1403 | 0.4311 |
| +NA | 6 | -0.07828 | 0.13991 | -0.3640 | 0.2074 |

Std Error uses a pooled estimate of error variance

### Means Comparisons

#### Comparisons with a control using Dunnett's Method

## Oneway Analysis of Phosphatidylglycerol By Label

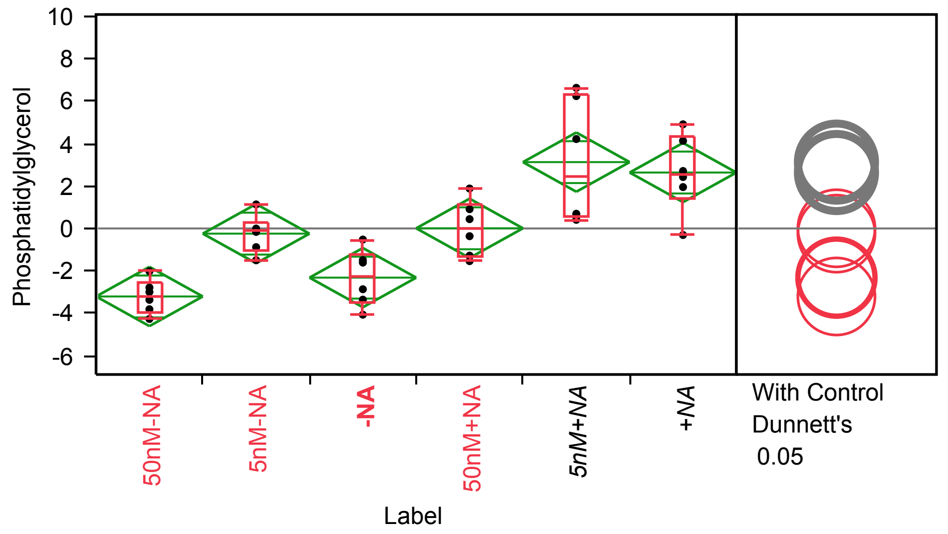


## Quantiles

| Level | Minimum | 10% | 25% | Median | 75% | 90% | Maximum |
|---|---|---|---|---|---|---|---|
| 50nM-NA | -4.27087 | -4.27087 | -3.92838 | -3.18478 | -2.59754 | -2.0217 | -2.0217 |
| 5nM-NA | -1.49746 | -1.49746 | -1.03631 | -0.10915 | 0.278613 | 1.133416 | 1.133416 |
| -NA | -4.07039 | -4.07039 | -3.53933 | -2.24071 | -1.25149 | -0.52408 | -0.52408 |
| 50nM+NA | -1.54221 | -1.54221 | -1.34882 | 0.037544 | 1.153647 | 1.883733 | 1.883733 |
| 5nM+NA | 0.408172 | 0.408172 | 0.537758 | 2.457097 | 6.345582 | 6.636919 | 6.636919 |
| +NA | -0.30793 | -0.30793 | 1.387477 | 2.569655 | 4.332758 | 4.915485 | 4.915485 |

## Oneway Anova

### Summary of Fit

| | |
|---|---|
| Rsquare | 0.698131 |
| Adj Rsquare | 0.64782 |
| Root Mean Square Error | 1.677407 |
| Mean of Response | 0 |
| Observations (or Sum Wgts) | 36 |

### Analysis of Variance

| Source | DF | Sum of Squares | Mean Square | F Ratio | Prob > F |
|---|---|---|---|---|---|
| Label | 5 | 195.21684 | 39.0434 | 13.8762 | <.0001 * |
| Error | 30 | 84.41081 | 2.8137 | | |
| C. Total | 35 | 279.62765 | | | |

### Means for Oneway Anova

| Level | Number | Mean | Std Error | Lower 95% | Upper 95% |
|---|---|---|---|---|---|
| 50nM-NA | 6 | -3.2110 | 0.68480 | -4.610 | -1.812 |
| 5nM-NA | 6 | -0.2452 | 0.68480 | -1.644 | 1.153 |
| -NA | 6 | -2.3220 | 0.68480 | -3.721 | -0.923 |
| 50nM+NA | 6 | 0.0071 | 0.68480 | -1.391 | 1.406 |
| 5nM+NA | 6 | 3.1315 | 0.68480 | 1.733 | 4.530 |
| +NA | 6 | 2.6397 | 0.68480 | 1.241 | 4.038 |

Std Error uses a pooled estimate of error variance

## Means Comparisons

### Comparisons with a control using Dunnett's Method

## Oneway Analysis of phosphoenolpyruvate By Label

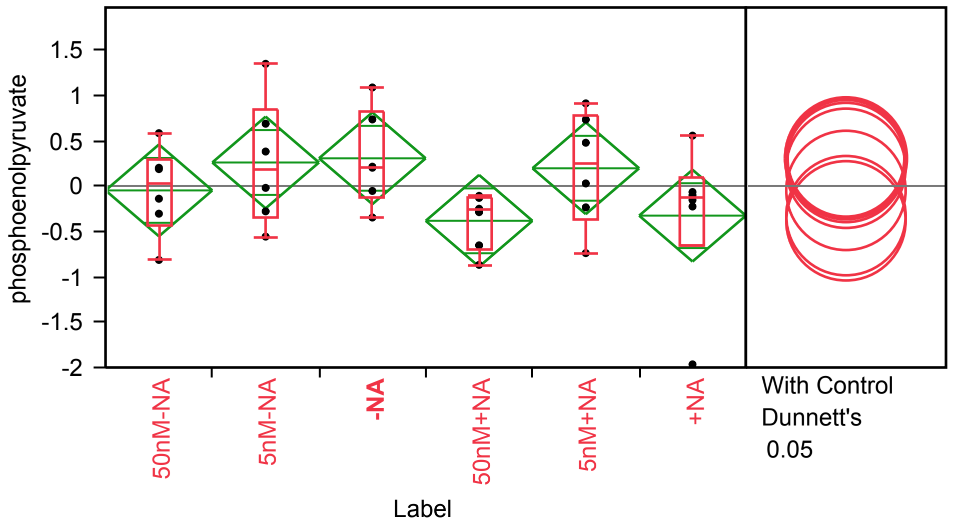


### Quantiles

| Level | Minimum | 10% | 25% | Median | 75% | 90% | Maximum |
|---|---|---|---|---|---|---|---|
| 50nM-NA | -0.81492 | -0.81492 | -0.43275 | 0.02288 | 0.295897 | 0.581953 | 0.581953 |
| 5nM-NA | -0.55785 | -0.55785 | -0.34906 | 0.179394 | 0.85093 | 1.346075 | 1.346075 |
| -NA | -0.34727 | -0.34727 | -0.12789 | 0.206828 | 0.821441 | 1.085366 | 1.085366 |
| 50nM+NA | -0.86885 | -0.86885 | -0.70778 | -0.2696 | -0.12566 | -0.10873 | -0.10873 |
| 5nM+NA | -0.73966 | -0.73966 | -0.3615 | 0.25224 | 0.775799 | 0.910218 | 0.910218 |
| +NA | -1.96556 | -1.96556 | -0.66093 | -0.12822 | 0.088565 | 0.554521 | 0.554521 |

### Oneway Anova

#### Summary of Fit

| | |
|---|---|
| Rsquare | 0.198416 |
| Adj Rsquare | 0.064818 |
| Root Mean Square Error | 0.605993 |
| Mean of Response | -5.6e-10 |
| Observations (or Sum Wgts) | 36 |

#### Analysis of Variance

| Source | DF | Sum of Squares | Mean Square | F Ratio | Prob > F |
|---|---|---|---|---|---|
| Label | 5 | 2.726983 | 0.545397 | 1.4852 | 0.2241 |
| Error | 30 | 11.016815 | 0.367227 | | |
| C. Total | 35 | 13.743798 | | | |

#### Means for Oneway Anova

| Level | Number | Mean | Std Error | Lower 95% | Upper 95% |
|---|---|---|---|---|---|
| 50nM-NA | 6 | -0.04867 | 0.24740 | -0.5539 | 0.45658 |
| 5nM-NA | 6 | 0.25890 | 0.24740 | -0.2463 | 0.76415 |
| -NA | 6 | 0.30508 | 0.24740 | -0.2002 | 0.81032 |
| 50nM+NA | 6 | -0.38369 | 0.24740 | -0.8889 | 0.12155 |
| 5nM+NA | 6 | 0.19510 | 0.24740 | -0.3102 | 0.70035 |
| +NA | 6 | -0.32671 | 0.24740 | -0.8320 | 0.17854 |

Std Error uses a pooled estimate of error variance

### Means Comparisons

#### Comparisons with a control using Dunnett's Method

## Oneway Analysis of Phosphorylcholine By Label

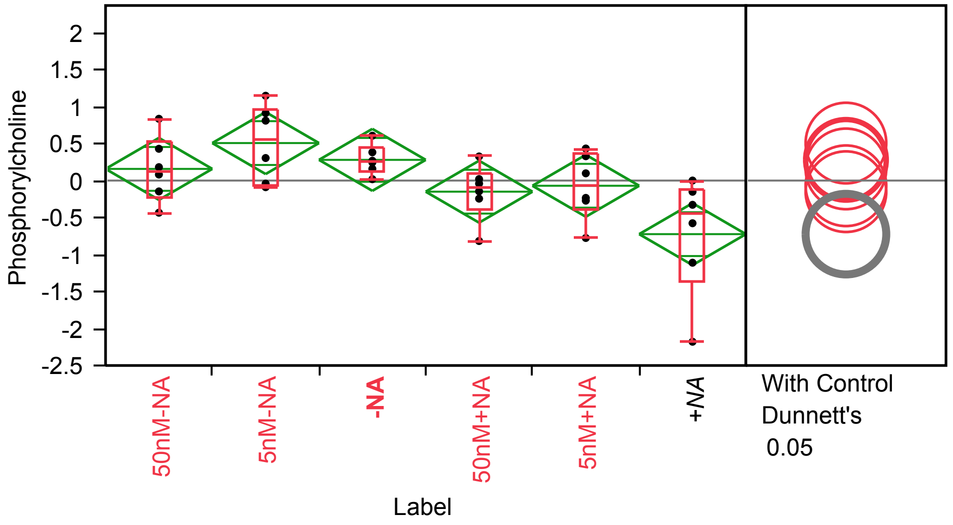


### Quantiles

| Level | Minimum | 10% | 25% | Median | 75% | 90% | Maximum |
|---|---|---|---|---|---|---|---|
| 50nM-NA | -0.43434 | -0.43434 | -0.21875 | 0.131437 | 0.530925 | 0.83511 | 0.83511 |
| 5nM-NA | -0.08938 | -0.08938 | -0.05341 | 0.557359 | 0.970436 | 1.146465 | 1.146465 |
| -NA | 0.018445 | 0.018445 | 0.123931 | 0.256701 | 0.438264 | 0.606373 | 0.606373 |
| 50nM+NA | -0.81712 | -0.81712 | -0.38757 | -0.0929 | 0.09643 | 0.326047 | 0.326047 |
| 5nM+NA | -0.77599 | -0.77599 | -0.39764 | -0.06816 | 0.355188 | 0.433249 | 0.433249 |
| +NA | -2.17879 | -2.17879 | -1.37763 | -0.45226 | -0.11358 | 0.000225 | 0.000225 |

### Oneway Anova

#### Summary of Fit

| | |
|---|---|
| Rsquare | 0.419613 |
| Adj Rsquare | 0.322882 |
| Root Mean Square Error | 0.502353 |
| Mean of Response | 1.11e-10 |
| Observations (or Sum Wgts) | 36 |

#### Analysis of Variance

| Source | DF | Sum of Squares | Mean Square | F Ratio | Prob > F |
|---|---|---|---|---|---|
| Label | 5 | 5.473583 | 1.09472 | 4.3379 | 0.0043 * |
| Error | 30 | 7.570765 | 0.25236 | | |
| C. Total | 35 | 13.044348 | | | |

#### Means for Oneway Anova

| Level | Number | Mean | Std Error | Lower 95% | Upper 95% |
|---|---|---|---|---|---|
| 50nM-NA | 6 | 0.15772 | 0.20508 | -0.261 | 0.5766 |
| 5nM-NA | 6 | 0.50702 | 0.20508 | 0.088 | 0.9259 |
| -NA | 6 | 0.27992 | 0.20508 | -0.139 | 0.6988 |
| 50nM+NA | 6 | -0.15023 | 0.20508 | -0.569 | 0.2686 |
| 5nM+NA | 6 | -0.07024 | 0.20508 | -0.489 | 0.3486 |
| +NA | 6 | -0.72420 | 0.20508 | -1.143 | -0.3054 |

Std Error uses a pooled estimate of error variance

### Means Comparisons

#### Comparisons with a control using Dunnett's Method

## Oneway Analysis of p-hydroxybenzoate By Label

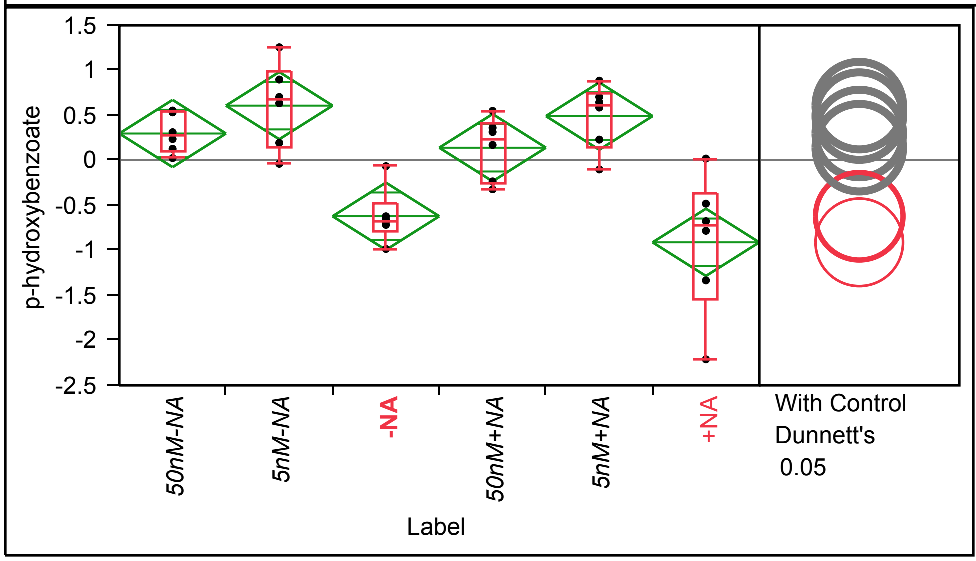


## Quantiles

| Level | Minimum | 10% | 25% | Median | 75% | 90% | Maximum |
|---|---|---|---|---|---|---|---|
| 50nM-NA | 0.024063 | 0.024063 | 0.101415 | 0.273584 | 0.537309 | 0.552034 | 0.552034 |
| 5nM-NA | -0.03914 | -0.03914 | 0.135293 | 0.667981 | 0.986375 | 1.255105 | 1.255105 |
| -NA | -0.98542 | -0.98542 | -0.78472 | -0.671 | -0.48369 | -0.0661 | -0.0661 |
| 50nM+NA | -0.32225 | -0.32225 | -0.2569 | 0.242042 | 0.40845 | 0.548618 | 0.548618 |
| 5nM+NA | -0.10197 | -0.10197 | 0.146536 | 0.6146 | 0.748329 | 0.884228 | 0.884228 |
| +NA | -2.21198 | -2.21198 | -1.55412 | -0.73053 | -0.35713 | 0.018491 | 0.018491 |

## Oneway Anova

### Summary of Fit

| | |
|---|---|
| Rsquare | 0.658289 |
| Adj Rsquare | 0.601337 |
| Root Mean Square Error | 0.448318 |
| Mean of Response | -5.6e-10 |
| Observations (or Sum Wgts) | 36 |

### Analysis of Variance

| Source | DF | Sum of Squares | Mean Square | F Ratio | Prob > F |
|---|---|---|---|---|---|
| Label | 5 | 11.615853 | 2.32317 | 11.5587 | <.0001 * |
| Error | 30 | 6.029665 | 0.20099 | | |
| C. Total | 35 | 17.645518 | | | |

### Means for Oneway Anova

| Level | Number | Mean | Std Error | Lower 95% | Upper 95% |
|---|---|---|---|---|---|
| 50nM-NA | 6 | 0.29714 | 0.18302 | -0.077 | 0.6709 |
| 5nM-NA | 6 | 0.60703 | 0.18302 | 0.233 | 0.9808 |
| -NA | 6 | -0.62237 | 0.18302 | -0.996 | -0.2486 |
| 50nM+NA | 6 | 0.13951 | 0.18302 | -0.234 | 0.5133 |
| 5nM+NA | 6 | 0.49064 | 0.18302 | 0.117 | 0.8644 |
| +NA | 6 | -0.91195 | 0.18302 | -1.286 | -0.5382 |

Std Error uses a pooled estimate of error variance

## Means Comparisons

### Comparisons with a control using Dunnett's Method

## Oneway Analysis of PI(16:0/20:2) By Label

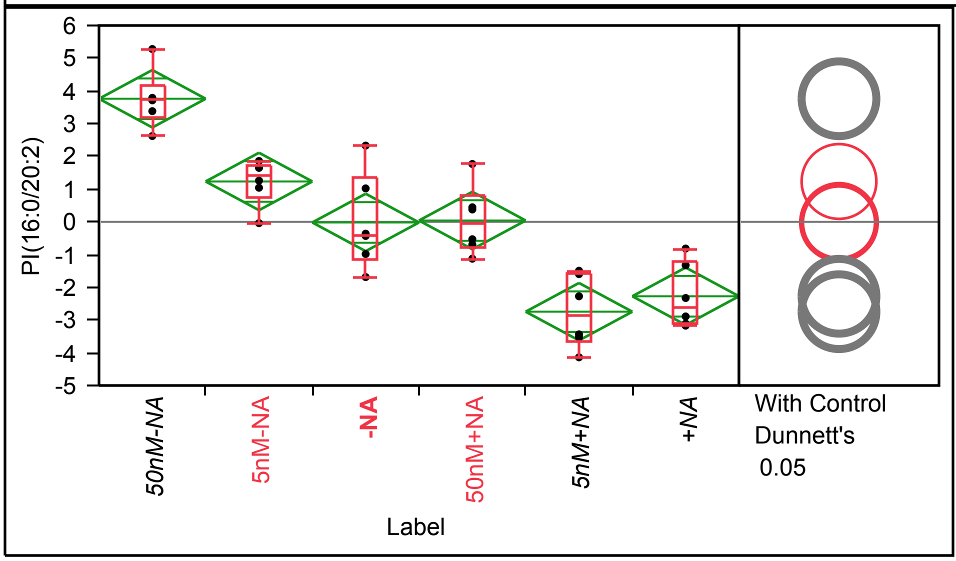


## Quantiles

| Level | Minimum | 10% | 25% | Median | 75% | 90% | Maximum |
|---|---|---|---|---|---|---|---|
| 50nM-NA | 2.622976 | 2.622976 | 3.192662 | 3.746502 | 4.162221 | 5.281024 | 5.281024 |
| 5nM-NA | -0.03878 | -0.03878 | 0.772407 | 1.445814 | 1.70191 | 1.860337 | 1.860337 |
| -NA | -1.68671 | -1.68671 | -1.15856 | -0.39627 | 1.349289 | 2.328989 | 2.328989 |
| 50nM+NA | -1.12559 | -1.12559 | -0.8001 | -0.07598 | 0.776144 | 1.767703 | 1.767703 |
| 5nM+NA | -4.13872 | -4.13872 | -3.67955 | -2.8559 | -1.57151 | -1.49447 | -1.49447 |
| +NA | -3.17158 | -3.17158 | -3.12473 | -2.60835 | -1.19849 | -0.816 | -0.816 |

## Oneway Anova

### Summary of Fit

| | |
|---|---|
| Rsquare | 0.836584 |
| Adj Rsquare | 0.809348 |
| Root Mean Square Error | 1.052876 |
| Mean of Response | 2.78e-10 |
| Observations (or Sum Wgts) | 36 |

### Analysis of Variance

| Source | DF | Sum of Squares | Mean Square | F Ratio | Prob > F |
|---|---|---|---|---|---|
| Label | 5 | 170.25160 | 34.0503 | 30.7161 | <.0001 * |
| Error | 30 | 33.25643 | 1.1085 | | |
| C. Total | 35 | 203.50803 | | | |

### Means for Oneway Anova

| Level | Number | Mean | Std Error | Lower 95% | Upper 95% |
|---|---|---|---|---|---|
| 50nM-NA | 6 | 3.7615 | 0.42983 | 2.884 | 4.639 |
| 5nM-NA | 6 | 1.2342 | 0.42983 | 0.356 | 2.112 |
| -NA | 6 | -0.0183 | 0.42983 | -0.896 | 0.859 |
| 50nM+NA | 6 | 0.0407 | 0.42983 | -0.837 | 0.919 |
| 5nM+NA | 6 | -2.7448 | 0.42983 | -3.623 | -1.867 |
| +NA | 6 | -2.2732 | 0.42983 | -3.151 | -1.395 |

Std Error uses a pooled estimate of error variance

## Means Comparisons

### Comparisons with a control using Dunnett's Method

## Oneway Analysis of PI(16:0/22:4) By Label

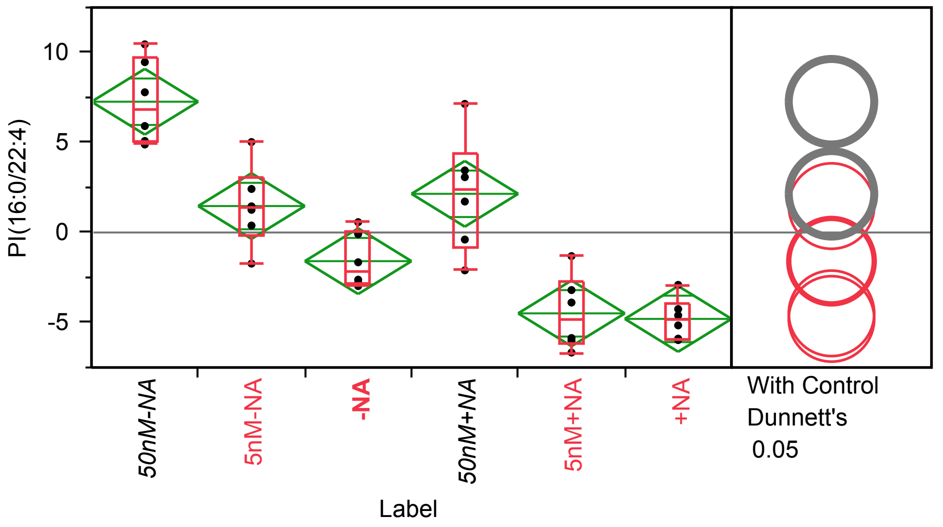


## Quantiles

| Level | Minimum | 10% | 25% | Median | 75% | 90% | Maximum |
|---|---|---|---|---|---|---|---|
| 50nM-NA | 4.889046 | 4.889046 | 5.036596 | 6.852175 | 9.710947 | 10.45267 | 10.45267 |
| 5nM-NA | -1.72795 | -1.72795 | -0.143 | 1.356983 | 3.070265 | 5.024486 | 5.024486 |
| -NA | -2.97463 | -2.97463 | -2.83312 | -2.13876 | 0.076361 | 0.573977 | 0.573977 |
| 50nM+NA | -2.1021 | -2.1021 | -0.81975 | 2.397221 | 4.372343 | 7.143888 | 7.143888 |
| 5nM+NA | -6.67021 | -6.67021 | -6.17586 | -4.8782 | -2.72752 | -1.31286 | -1.31286 |
| +NA | -5.97582 | -5.97582 | -5.91734 | -4.88064 | -3.91814 | -2.91044 | -2.91044 |

## Oneway Anova

### Summary of Fit

| | |
|---|---|
| Rsquare | 0.814697 |
| Adj Rsquare | 0.783813 |
| Root Mean Square Error | 2.18882 |
| Mean of Response | 2.778e-9 |
| Observations (or Sum Wgts) | 36 |

### Analysis of Variance

| Source | DF | Sum of Squares | Mean Square | F Ratio | Prob > F |
|---|---|---|---|---|---|
| Label | 5 | 631.90747 | 126.381 | 26.3793 | <.0001 * |
| Error | 30 | 143.72794 | 4.791 | | |
| C. Total | 35 | 775.63542 | | | |

### Means for Oneway Anova

| Level | Number | Mean | Std Error | Lower 95% | Upper 95% |
|---|---|---|---|---|---|
| 50nM-NA | 6 | 7.2659 | 0.89358 | 5.441 | 9.091 |
| 5nM-NA | 6 | 1.4691 | 0.89358 | -0.356 | 3.294 |
| -NA | 6 | -1.5923 | 0.89358 | -3.417 | 0.233 |
| 50nM+NA | 6 | 2.1487 | 0.89358 | 0.324 | 3.974 |
| 5nM+NA | 6 | -4.4916 | 0.89358 | -6.317 | -2.667 |
| +NA | 6 | -4.7999 | 0.89358 | -6.625 | -2.975 |

Std Error uses a pooled estimate of error variance

## Means Comparisons

### Comparisons with a control using Dunnett's Method

## Oneway Analysis of PI(16:1/22:4) By Label

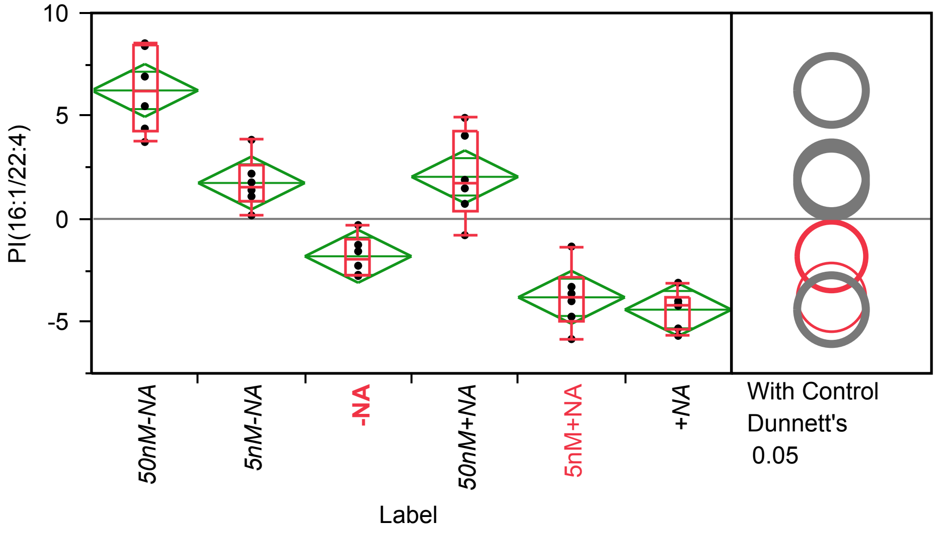


### Quantiles

| Level | Minimum | 10% | 25% | Median | 75% | 90% | Maximum |
|---|---|---|---|---|---|---|---|
| 50nM-NA | 3.735954 | 3.735954 | 4.224711 | 6.195558 | 8.430433 | 8.530166 | 8.530166 |
| 5nM-NA | 0.172032 | 0.172032 | 0.865309 | 1.585636 | 2.607733 | 3.839856 | 3.839856 |
| -NA | -2.77131 | -2.77131 | -2.73079 | -1.92346 | -1.01944 | -0.29857 | -0.29857 |
| 50nM+NA | -0.79038 | -0.79038 | 0.351371 | 1.688251 | 4.257241 | 4.906469 | 4.906469 |
| 5nM+NA | -5.83738 | -5.83738 | -5.01974 | -3.81062 | -2.8106 | -1.34221 | -1.34221 |
| +NA | -5.67752 | -5.67752 | -5.4091 | -4.17643 | -3.76872 | -3.10075 | -3.10075 |

### Oneway Anova

#### Summary of Fit

| | |
|---|---|
| Rsquare | 0.875779 |
| Adj Rsquare | 0.855076 |
| Root Mean Square Error | 1.538043 |
| Mean of Response | -3e-16 |
| Observations (or Sum Wgts) | 36 |

#### Analysis of Variance

| Source | DF | Sum of Squares | Mean Square | F Ratio | Prob > F |
|---|---|---|---|---|---|
| Label | 5 | 500.33369 | 100.067 | 42.3012 | <.0001 * |
| Error | 30 | 70.96731 | 2.366 | | |
| C. Total | 35 | 571.30100 | | | |

#### Means for Oneway Anova

| Level | Number | Mean | Std Error | Lower 95% | Upper 95% |
|---|---|---|---|---|---|
| 50nM-NA | 6 | 6.2403 | 0.62790 | 4.958 | 7.523 |
| 5nM-NA | 6 | 1.7461 | 0.62790 | 0.464 | 3.028 |
| -NA | 6 | -1.8156 | 0.62790 | -3.098 | -0.533 |
| 50nM+NA | 6 | 2.0442 | 0.62790 | 0.762 | 3.327 |
| 5nM+NA | 6 | -3.8080 | 0.62790 | -5.090 | -2.526 |
| +NA | 6 | -4.4070 | 0.62790 | -5.689 | -3.125 |

Std Error uses a pooled estimate of error variance

### Means Comparisons

#### Comparisons with a control using Dunnett's Method

## Oneway Analysis of Piperidine By Label

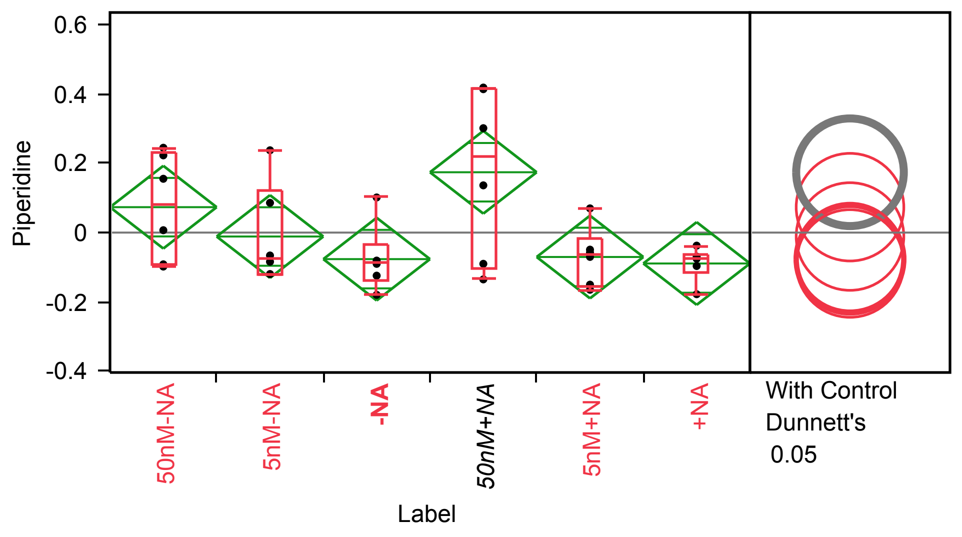


## Quantiles

| Level | Minimum | 10% | 25% | Median | 75% | 90% | Maximum |
|---|---|---|---|---|---|---|---|
| 50nM-NA | -0.0982 | -0.0982 | -0.09338 | 0.081181 | 0.22872 | 0.245163 | 0.245163 |
| 5nM-NA | -0.12162 | -0.12162 | -0.12026 | -0.07525 | 0.123956 | 0.237835 | 0.237835 |
| -NA | -0.17995 | -0.17995 | -0.13836 | -0.08814 | -0.03534 | 0.101488 | 0.101488 |
| 50nM+NA | -0.13523 | -0.13523 | -0.1015 | 0.219133 | 0.415654 | 0.419119 | 0.419119 |
| 5nM+NA | -0.1652 | -0.1652 | -0.15419 | -0.06402 | -0.01951 | 0.069946 | 0.069946 |
| +NA | -0.17804 | -0.17804 | -0.11694 | -0.077 | -0.06148 | -0.03778 | -0.03778 |

## Oneway Anova

### Summary of Fit

| | |
|---|---|
| Rsquare | 0.347614 |
| Adj Rsquare | 0.238883 |
| Root Mean Square Error | 0.143382 |
| Mean of Response | -2.8e-11 |
| Observations (or Sum Wgts) | 36 |

### Analysis of Variance

| Source | DF | Sum of Squares | Mean Square | F Ratio | Prob > F |
|---|---|---|---|---|---|
| Label | 5 | 0.32862516 | 0.065725 | 3.1970 | 0.0197 * |
| Error | 30 | 0.61674784 | 0.020558 | | |
| C. Total | 35 | 0.94537300 | | | |

### Means for Oneway Anova

| Level | Number | Mean | Std Error | Lower 95% | Upper 95% |
|---|---|---|---|---|---|
| 50nM-NA | 6 | 0.07347 | 0.05854 | -0.0461 | 0.19301 |
| 5nM-NA | 6 | -0.01135 | 0.05854 | -0.1309 | 0.10820 |
| -NA | 6 | -0.07670 | 0.05854 | -0.1962 | 0.04285 |
| 50nM+NA | 6 | 0.17440 | 0.05854 | 0.0549 | 0.29394 |
| 5nM+NA | 6 | -0.07053 | 0.05854 | -0.1901 | 0.04902 |
| +NA | 6 | -0.08930 | 0.05854 | -0.2088 | 0.03025 |

Std Error uses a pooled estimate of error variance

## Means Comparisons

### Comparisons with a control using Dunnett's Method

## Oneway Analysis of proline By Label

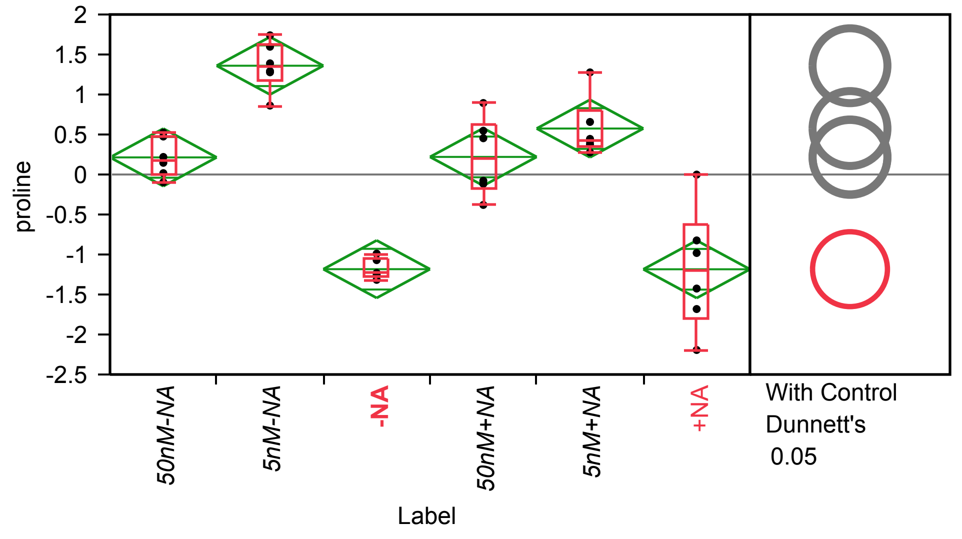


## Quantiles

| Level | Minimum | 10% | 25% | Median | 75% | 90% | Maximum |
|---|---|---|---|---|---|---|---|
| 50nM-NA | -0.09636 | -0.09636 | -0.01155 | 0.182685 | 0.486843 | 0.522844 | 0.522844 |
| 5nM-NA | 0.861628 | 0.861628 | 1.173658 | 1.340739 | 1.632742 | 1.73968 | 1.73968 |
| -NA | -1.31672 | -1.31672 | -1.27342 | -1.23246 | -1.04997 | -0.98916 | -0.98916 |
| 50nM+NA | -0.38013 | -0.38013 | -0.18254 | 0.187776 | 0.633393 | 0.893313 | 0.893313 |
| 5nM+NA | 0.27727 | 0.27727 | 0.352838 | 0.429861 | 0.811611 | 1.276194 | 1.276194 |
| +NA | -2.1934 | -2.1934 | -1.80965 | -1.20345 | -0.61844 | -0.00162 | -0.00162 |

## Oneway Anova

### Summary of Fit

| | |
|---|---|
| Rsquare | 0.845114 |
| Adj Rsquare | 0.8193 |
| Root Mean Square Error | 0.431361 |
| Mean of Response | -2.8e-10 |
| Observations (or Sum Wgts) | 36 |

### Analysis of Variance

| Source | DF | Sum of Squares | Mean Square | F Ratio | Prob > F |
|---|---|---|---|---|---|
| Label | 5 | 30.458380 | 6.09168 | 32.7383 | <.0001 * |
| Error | 30 | 5.582159 | 0.18607 | | |
| C. Total | 35 | 36.040540 | | | |

### Means for Oneway Anova

| Level | Number | Mean | Std Error | Lower 95% | Upper 95% |
|---|---|---|---|---|---|
| 50nM-NA | 6 | 0.2139 | 0.17610 | -0.146 | 0.574 |
| 5nM-NA | 6 | 1.3596 | 0.17610 | 1.000 | 1.719 |
| -NA | 6 | -1.1833 | 0.17610 | -1.543 | -0.824 |
| 50nM+NA | 6 | 0.2198 | 0.17610 | -0.140 | 0.579 |
| 5nM+NA | 6 | 0.5747 | 0.17610 | 0.215 | 0.934 |
| +NA | 6 | -1.1846 | 0.17610 | -1.544 | -0.825 |

Std Error uses a pooled estimate of error variance

## Means Comparisons

### Comparisons with a control using Dunnett's Method

## Oneway Analysis of purine By Label

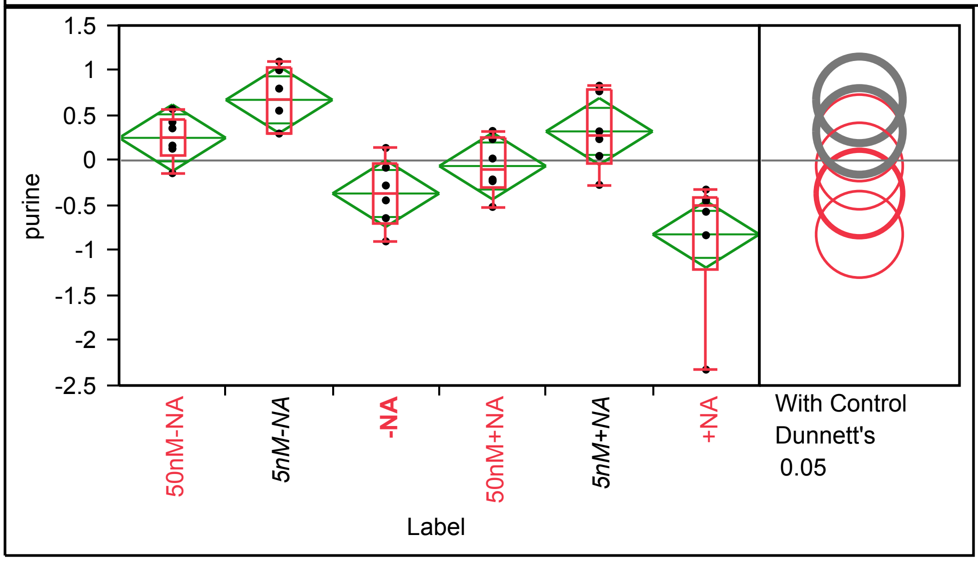


## Quantiles

| Level | Minimum | 10% | 25% | Median | 75% | 90% | Maximum |
|---|---|---|---|---|---|---|---|
| 50nM-NA | -0.14121 | -0.14121 | 0.062804 | 0.261477 | 0.462102 | 0.570996 | 0.570996 |
| 5nM-NA | 0.289841 | 0.289841 | 0.296705 | 0.676261 | 1.027298 | 1.09884 | 1.09884 |
| -NA | -0.89841 | -0.89841 | -0.70501 | -0.36025 | -0.02526 | 0.139435 | 0.139435 |
| 50nM+NA | -0.516 | -0.516 | -0.30126 | -0.09457 | 0.260297 | 0.328202 | 0.328202 |
| 5nM+NA | -0.27092 | -0.27092 | -0.02976 | 0.280697 | 0.784122 | 0.830723 | 0.830723 |
| +NA | -2.32355 | -2.32355 | -1.20366 | -0.50966 | -0.40152 | -0.32354 | -0.32354 |

## Oneway Anova

### Summary of Fit

| | |
|---|---|
| Rsquare | 0.593446 |
| Adj Rsquare | 0.525687 |
| Root Mean Square Error | 0.443242 |
| Mean of Response | -8.3e-10 |
| Observations (or Sum Wgts) | 36 |

### Analysis of Variance

| Source | DF | Sum of Squares | Mean Square | F Ratio | Prob > F |
|---|---|---|---|---|---|
| Label | 5 | 8.603320 | 1.72066 | 8.7582 | <.0001 * |
| Error | 30 | 5.893895 | 0.19646 | | |
| C. Total | 35 | 14.497215 | | | |

### Means for Oneway Anova

| Level | Number | Mean | Std Error | Lower 95% | Upper 95% |
|---|---|---|---|---|---|
| 50nM-NA | 6 | 0.25156 | 0.18095 | -0.118 | 0.621 |
| 5nM-NA | 6 | 0.67394 | 0.18095 | 0.304 | 1.043 |
| -NA | 6 | -0.36669 | 0.18095 | -0.736 | 0.00286 |
| 50nM+NA | 6 | -0.06149 | 0.18095 | -0.431 | 0.308 |
| 5nM+NA | 6 | 0.32340 | 0.18095 | -0.046 | 0.693 |
| +NA | 6 | -0.82071 | 0.18095 | -1.190 | -0.451 |

Std Error uses a pooled estimate of error variance

## Means Comparisons

### Comparisons with a control using Dunnett's Method

## Oneway Analysis of pyridoxine By Label

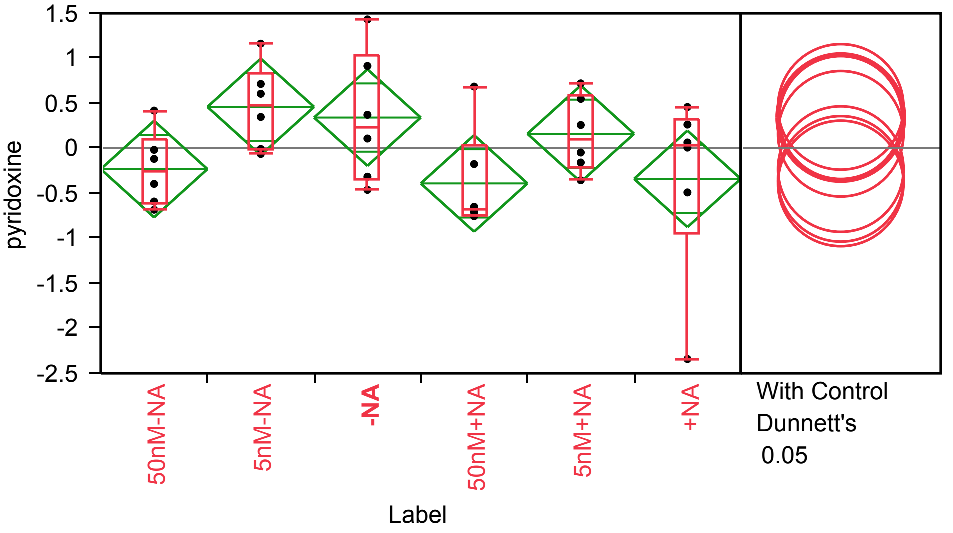


## Quantiles

| Level | Minimum | 10% | 25% | Median | 75% | 90% | Maximum |
|---|---|---|---|---|---|---|---|
| 50nM-NA | -0.68268 | -0.68268 | -0.61464 | -0.25708 | 0.091251 | 0.418396 | 0.418396 |
| 5nM-NA | -0.06332 | -0.06332 | -0.02197 | 0.476575 | 0.826284 | 1.161902 | 1.161902 |
| -NA | -0.46226 | -0.46226 | -0.3526 | 0.239913 | 1.043693 | 1.431506 | 1.431506 |
| 50nM+NA | -0.75473 | -0.75473 | -0.74563 | -0.678 | 0.039592 | 0.685129 | 0.685129 |
| 5nM+NA | -0.35551 | -0.35551 | -0.2078 | 0.105579 | 0.591727 | 0.720972 | 0.720972 |
| +NA | -2.34202 | -2.34202 | -0.95355 | 0.035745 | 0.311857 | 0.457978 | 0.457978 |

## Oneway Anova

### Summary of Fit

| | |
|---|---|
| Rsquare | 0.246268 |
| Adj Rsquare | 0.120646 |
| Root Mean Square Error | 0.642997 |
| Mean of Response | 5.56e-11 |
| Observations (or Sum Wgts) | 36 |

### Analysis of Variance

| Source | DF | Sum of Squares | Mean Square | F Ratio | Prob > F |
|---|---|---|---|---|---|
| Label | 5 | 4.052565 | 0.810513 | 1.9604 | 0.1136 |
| Error | 30 | 12.403367 | 0.413446 | | |
| C. Total | 35 | 16.455932 | | | |

### Means for Oneway Anova

| Level | Number | Mean | Std Error | Lower 95% | Upper 95% |
|---|---|---|---|---|---|
| 50nM-NA | 6 | -0.23137 | 0.26250 | -0.7675 | 0.30473 |
| 5nM-NA | 6 | 0.45966 | 0.26250 | -0.0764 | 0.99576 |
| -NA | 6 | 0.34124 | 0.26250 | -0.1949 | 0.87734 |
| 50nM+NA | 6 | -0.39063 | 0.26250 | -0.9267 | 0.14547 |
| 5nM+NA | 6 | 0.16112 | 0.26250 | -0.3750 | 0.69722 |
| +NA | 6 | -0.34002 | 0.26250 | -0.8761 | 0.19608 |

Std Error uses a pooled estimate of error variance

## Means Comparisons

### Comparisons with a control using Dunnett's Method

## Oneway Analysis of Pyroglutamate By Label

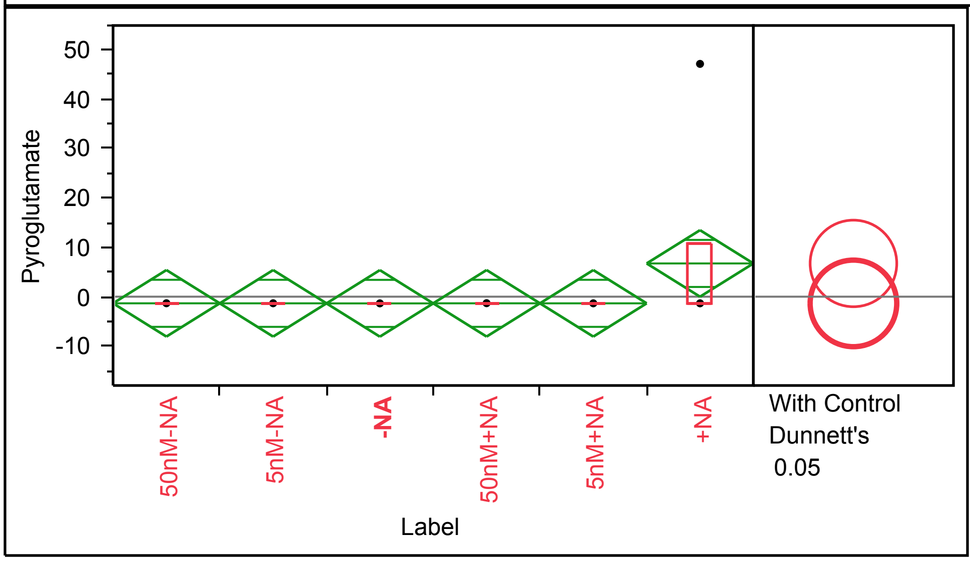


### Quantiles

| Level | Minimum | 10% | 25% | Median | 75% | 90% | Maximum |
|---|---|---|---|---|---|---|---|
| 50nM-NA | -1.35239 | -1.35239 | -1.35082 | -1.34217 | -1.33362 | -1.32511 | -1.32511 |
| 5nM-NA | -1.36094 | -1.36094 | -1.35335 | -1.34344 | -1.33717 | -1.32914 | -1.32914 |
| -NA | -1.36308 | -1.36308 | -1.36059 | -1.35914 | -1.35392 | -1.34949 | -1.34949 |
| 50nM+NA | -1.3608 | -1.3608 | -1.36079 | -1.34393 | -1.32067 | -1.31275 | -1.31275 |
| 5nM+NA | -1.34875 | -1.34875 | -1.34785 | -1.34671 | -1.34287 | -1.34006 | -1.34006 |
| +NA | -1.35867 | -1.35867 | -1.35412 | -1.34264 | 10.77417 | 47.11846 | 47.11846 |

### Oneway Anova

#### Summary of Fit

| | |
|---|---|
| Rsquare | 0.142815 |
| Adj Rsquare | -4.88e-5 |
| Root Mean Square Error | 8.077655 |
| Mean of Response | -2.78e-8 |
| Observations (or Sum Wgts) | 36 |

#### Analysis of Variance

| Source | DF | Sum of Squares | Mean Square | F Ratio | Prob > F |
|---|---|---|---|---|---|
| Label | 5 | 326.1312 | 65.2262 | 0.9997 | 0.4348 |
| Error | 30 | 1957.4553 | 65.2485 | | |
| C. Total | 35 | 2283.5865 | | | |

#### Means for Oneway Anova

| Level | Number | Mean | Std Error | Lower 95% | Upper 95% |
|---|---|---|---|---|---|
| 50nM-NA | 6 | -1.3414 | 3.2977 | -8.076 | 5.393 |
| 5nM-NA | 6 | -1.3446 | 3.2977 | -8.079 | 5.390 |
| -NA | 6 | -1.3577 | 3.2977 | -8.092 | 5.377 |
| 50nM+NA | 6 | -1.3409 | 3.2977 | -8.076 | 5.394 |
| 5nM+NA | 6 | -1.3456 | 3.2977 | -8.080 | 5.389 |
| +NA | 6 | 6.7302 | 3.2977 | -0.0046 | 13.465 |

Std Error uses a pooled estimate of error variance

### Means Comparisons

#### Comparisons with a control using Dunnett's Method

## Oneway Analysis of pyruvate By Label

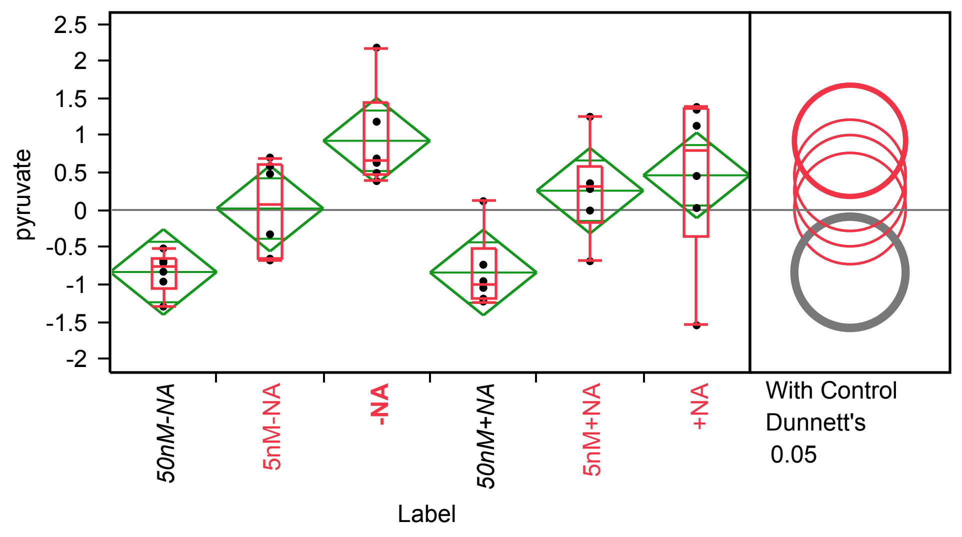


## Quantiles

| Level | Minimum | 10% | 25% | Median | 75% | 90% | Maximum |
|---|---|---|---|---|---|---|---|
| 50nM-NA | -1.29624 | -1.29624 | -1.0452 | -0.76858 | -0.64175 | -0.51627 | -0.51627 |
| 5nM-NA | -0.67587 | -0.67587 | -0.65976 | 0.078146 | 0.617025 | 0.702632 | 0.702632 |
| -NA | 0.387943 | 0.387943 | 0.469669 | 0.661469 | 1.432757 | 2.178318 | 2.178318 |
| 50nM+NA | -1.2284 | -1.2284 | -1.19992 | -1.00031 | -0.52178 | 0.117766 | 0.117766 |
| 5nM+NA | -0.68714 | -0.68714 | -0.17746 | 0.319419 | 0.581307 | 1.250071 | 1.250071 |
| +NA | -1.54742 | -1.54742 | -0.36693 | 0.792507 | 1.354652 | 1.381439 | 1.381439 |

## Oneway Anova

### Summary of Fit

| | |
|---|---|
| Rsquare | 0.517854 |
| Adj Rsquare | 0.437497 |
| Root Mean Square Error | 0.68818 |
| Mean of Response | -2.8e-11 |
| Observations (or Sum Wgts) | 36 |

### Analysis of Variance

| Source | DF | Sum of Squares | Mean Square | F Ratio | Prob > F |
|---|---|---|---|---|---|
| Label | 5 | 15.260007 | 3.05200 | 6.4444 | 0.0004 * |
| Error | 30 | 14.207745 | 0.47359 | | |
| C. Total | 35 | 29.467753 | | | |

### Means for Oneway Anova

| Level | Number | Mean | Std Error | Lower 95% | Upper 95% |
|---|---|---|---|---|---|
| 50nM-NA | 6 | -0.83246 | 0.28095 | -1.406 | -0.259 |
| 5nM-NA | 6 | 0.01952 | 0.28095 | -0.554 | 0.593 |
| -NA | 6 | 0.92839 | 0.28095 | 0.355 | 1.502 |
| 50nM+NA | 6 | -0.83944 | 0.28095 | -1.413 | -0.266 |
| 5nM+NA | 6 | 0.25876 | 0.28095 | -0.315 | 0.833 |
| +NA | 6 | 0.46522 | 0.28095 | -0.109 | 1.039 |

Std Error uses a pooled estimate of error variance

## Means Comparisons

### Comparisons with a control using Dunnett's Method

## Oneway Analysis of riboflavin By Label

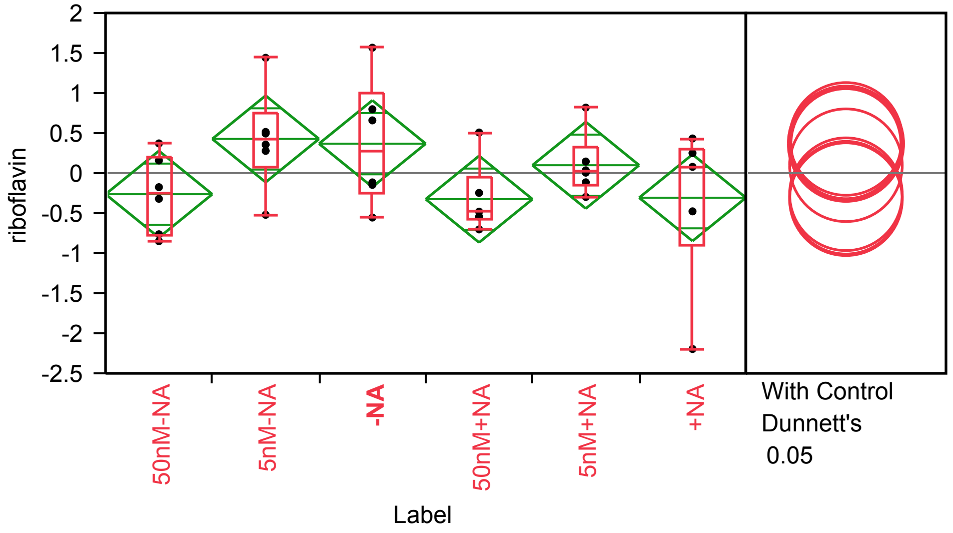


### Quantiles

| Level | Minimum | 10% | 25% | Median | 75% | 90% | Maximum |
|---|---|---|---|---|---|---|---|
| 50nM-NA | -0.84942 | -0.84942 | -0.78472 | -0.24765 | 0.211871 | 0.371273 | 0.371273 |
| 5nM-NA | -0.51995 | -0.51995 | 0.07864 | 0.425552 | 0.74631 | 1.440585 | 1.440585 |
| -NA | -0.55408 | -0.55408 | -0.24815 | 0.271815 | 0.990314 | 1.566474 | 1.566474 |
| 50nM+NA | -0.7022 | -0.7022 | -0.58343 | -0.48249 | -0.05799 | 0.507374 | 0.507374 |
| 5nM+NA | -0.2967 | -0.2967 | -0.15998 | 0.020783 | 0.313513 | 0.818381 | 0.818381 |
| +NA | -2.19603 | -2.19603 | -0.90674 | 0.075172 | 0.296213 | 0.433065 | 0.433065 |

### Oneway Anova

#### Summary of Fit

| | |
|---|---|
| Rsquare | 0.220932 |
| Adj Rsquare | 0.091087 |
| Root Mean Square Error | 0.648713 |
| Mean of Response | -5.6e-11 |
| Observations (or Sum Wgts) | 36 |

#### Analysis of Variance

| Source | DF | Sum of Squares | Mean Square | F Ratio | Prob > F |
|---|---|---|---|---|---|
| Label | 5 | 3.580207 | 0.716041 | 1.7015 | 0.1647 |
| Error | 30 | 12.624846 | 0.420828 | | |
| C. Total | 35 | 16.205053 | | | |

#### Means for Oneway Anova

| Level | Number | Mean | Std Error | Lower 95% | Upper 95% |
|---|---|---|---|---|---|
| 50nM-NA | 6 | -0.26298 | 0.26484 | -0.8038 | 0.27789 |
| 5nM-NA | 6 | 0.42747 | 0.26484 | -0.1134 | 0.96833 |
| -NA | 6 | 0.36802 | 0.26484 | -0.1728 | 0.90888 |
| 50nM+NA | 6 | -0.32502 | 0.26484 | -0.8659 | 0.21585 |
| 5nM+NA | 6 | 0.09901 | 0.26484 | -0.4419 | 0.63988 |
| +NA | 6 | -0.30650 | 0.26484 | -0.8474 | 0.23437 |

Std Error uses a pooled estimate of error variance

### Means Comparisons

#### Comparisons with a control using Dunnett's Method

## Oneway Analysis of ribose-5-phosphate By Label

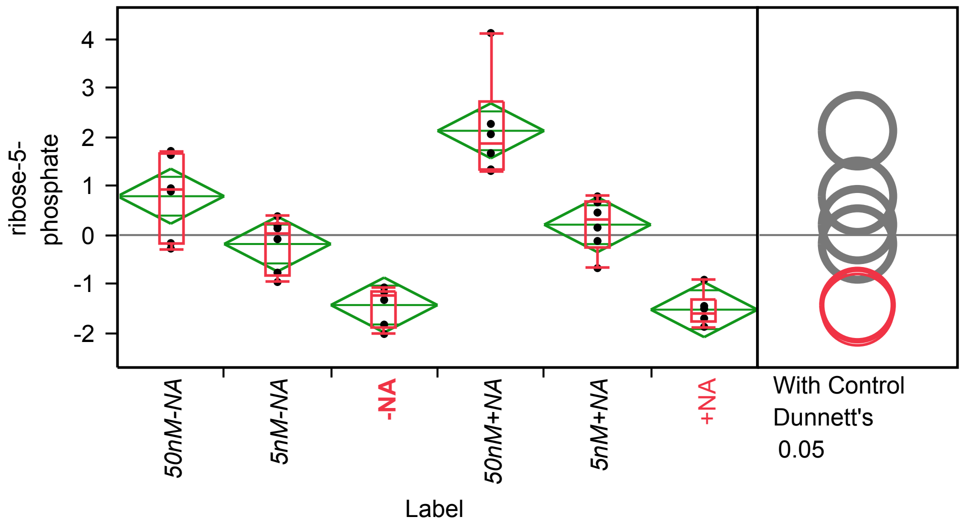


## Quantiles

| Level | Minimum | 10% | 25% | Median | 75% | 90% | Maximum |
|---|---|---|---|---|---|---|---|
| 50nM-NA | -0.27581 | -0.27581 | -0.19197 | 0.922658 | 1.655226 | 1.716226 | 1.716226 |
| 5nM-NA | -0.95844 | -0.95844 | -0.81508 | 0.026119 | 0.242212 | 0.383851 | 0.383851 |
| -NA | -2.01872 | -2.01872 | -1.87541 | -1.24699 | -1.13818 | -1.06908 | -1.06908 |
| 50nM+NA | 1.308132 | 1.308132 | 1.332259 | 1.864634 | 2.732473 | 4.12368 | 4.12368 |
| 5nM+NA | -0.67468 | -0.67468 | -0.25942 | 0.305554 | 0.698301 | 0.789853 | 0.789853 |
| +NA | -1.87289 | -1.87289 | -1.74361 | -1.60048 | -1.31385 | -0.9125 | -0.9125 |

## Oneway Anova

### Summary of Fit

| | |
|---|---|
| Rsquare | 0.809638 |
| Adj Rsquare | 0.777911 |
| Root Mean Square Error | 0.671705 |
| Mean of Response | 2.78e-10 |
| Observations (or Sum Wgts) | 36 |

### Analysis of Variance

| Source | DF | Sum of Squares | Mean Square | F Ratio | Prob > F |
|---|---|---|---|---|---|
| Label | 5 | 57.569161 | 11.5138 | 25.5189 | <.0001 * |
| Error | 30 | 13.535636 | 0.4512 | | |
| C. Total | 35 | 71.104797 | | | |

### Means for Oneway Anova

| Level | Number | Mean | Std Error | Lower 95% | Upper 95% |
|---|---|---|---|---|---|
| 50nM-NA | 6 | 0.7928 | 0.27422 | 0.233 | 1.353 |
| 5nM-NA | 6 | -0.1824 | 0.27422 | -0.742 | 0.378 |
| -NA | 6 | -1.4284 | 0.27422 | -1.988 | -0.868 |
| 50nM+NA | 6 | 2.1284 | 0.27422 | 1.568 | 2.688 |
| 5nM+NA | 6 | 0.2122 | 0.27422 | -0.348 | 0.772 |
| +NA | 6 | -1.5224 | 0.27422 | -2.082 | -0.962 |

Std Error uses a pooled estimate of error variance

## Means Comparisons

### Comparisons with a control using Dunnett's Method

## Oneway Analysis of S-adenosyl-L-homoCysteine By Label

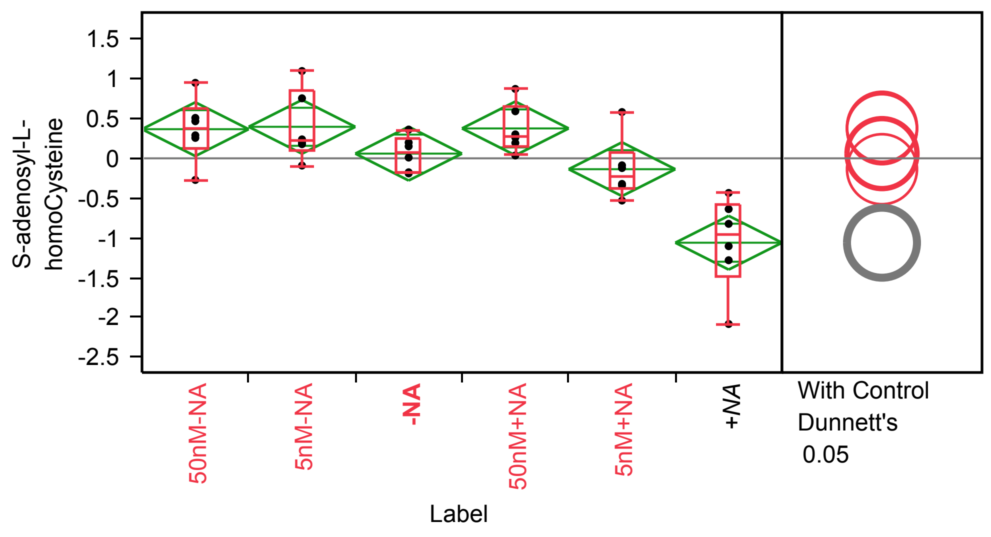


### Quantiles

| Level | Minimum | 10% | 25% | Median | 75% | 90% | Maximum |
|---|---|---|---|---|---|---|---|
| 50nM-NA | -0.27162 | -0.27162 | 0.124768 | 0.377743 | 0.618435 | 0.948932 | 0.948932 |
| 5nM-NA | -0.09083 | -0.09083 | 0.110726 | 0.219301 | 0.840427 | 1.097526 | 1.097526 |
| -NA | -0.18976 | -0.18976 | -0.17913 | 0.080312 | 0.239159 | 0.361099 | 0.361099 |
| 50nM+NA | 0.035687 | 0.035687 | 0.153718 | 0.281262 | 0.661905 | 0.872448 | 0.872448 |
| 5nM+NA | -0.53072 | -0.53072 | -0.38727 | -0.22202 | 0.079541 | 0.581545 | 0.581545 |
| +NA | -2.08286 | -2.08286 | -1.48175 | -0.96405 | -0.58747 | -0.43521 | -0.43521 |

### Oneway Anova

#### Summary of Fit

| | |
|---|---|
| Rsquare | 0.657798 |
| Adj Rsquare | 0.600764 |
| Root Mean Square Error | 0.405504 |
| Mean of Response | 0 |
| Observations (or Sum Wgts) | 36 |

#### Analysis of Variance

| Source | DF | Sum of Squares | Mean Square | F Ratio | Prob > F |
|---|---|---|---|---|---|
| Label | 5 | 9.482468 | 1.89649 | 11.5335 | <.0001 * |
| Error | 30 | 4.933004 | 0.16443 | | |
| C. Total | 35 | 14.415472 | | | |

#### Means for Oneway Anova

| Level | Number | Mean | Std Error | Lower 95% | Upper 95% |
|---|---|---|---|---|---|
| 50nM-NA | 6 | 0.3663 | 0.16555 | 0.028 | 0.7044 |
| 5nM-NA | 6 | 0.3963 | 0.16555 | 0.058 | 0.7344 |
| -NA | 6 | 0.0591 | 0.16555 | -0.279 | 0.3972 |
| 50nM+NA | 6 | 0.3759 | 0.16555 | 0.038 | 0.7140 |
| 5nM+NA | 6 | -0.1367 | 0.16555 | -0.475 | 0.2013 |
| +NA | 6 | -1.0610 | 0.16555 | -1.399 | -0.7229 |

Std Error uses a pooled estimate of error variance

### Means Comparisons

#### Comparisons with a control using Dunnett's Method

## Oneway Analysis of S-adenosyl-L-methioninamine By Label

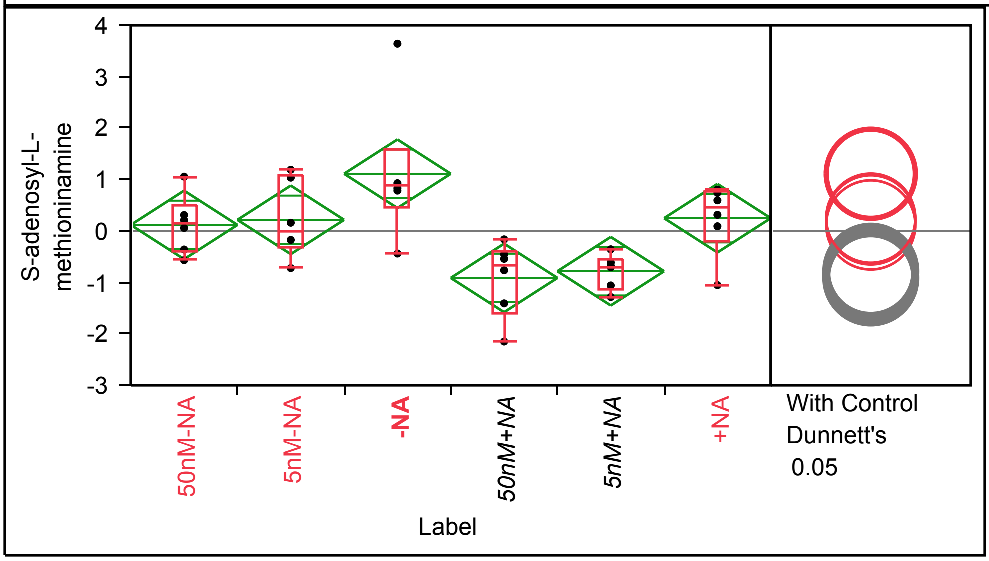


### Quantiles

| Level | Minimum | 10% | 25% | Median | 75% | 90% | Maximum |
|---|---|---|---|---|---|---|---|
| 50nM-NA | -0.56732 | -0.56732 | -0.41238 | 0.134853 | 0.496433 | 1.055808 | 1.055808 |
| 5nM-NA | -0.7212 | -0.7212 | -0.32523 | -0.00684 | 1.075006 | 1.19003 | 1.19003 |
| -NA | -0.43834 | -0.43834 | 0.47923 | 0.87931 | 1.607004 | 3.641227 | 3.641227 |
| 50nM+NA | -2.14965 | -2.14965 | -1.59315 | -0.65303 | -0.37998 | -0.16305 | -0.16305 |
| 5nM+NA | -1.28403 | -1.28403 | -1.11552 | -0.69062 | -0.54926 | -0.35649 | -0.35649 |
| +NA | -1.05293 | -1.05293 | -0.19462 | 0.454758 | 0.756285 | 0.803024 | 0.803024 |

### Oneway Anova

#### Summary of Fit

| | |
|---|---|
| Rsquare | 0.467728 |
| Adj Rsquare | 0.379016 |
| Root Mean Square Error | 0.799209 |
| Mean of Response | 0 |
| Observations (or Sum Wgts) | 36 |

#### Analysis of Variance

| Source | DF | Sum of Squares | Mean Square | F Ratio | Prob > F |
|---|---|---|---|---|---|
| Label | 5 | 16.838417 | 3.36768 | 5.2724 | 0.0014 * |
| Error | 30 | 19.162028 | 0.63873 | | |
| C. Total | 35 | 36.000445 | | | |

#### Means for Oneway Anova

| Level | Number | Mean | Std Error | Lower 95% | Upper 95% |
|---|---|---|---|---|---|
| 50nM-NA | 6 | 0.1179 | 0.32628 | -0.548 | 0.784 |
| 5nM-NA | 6 | 0.2164 | 0.32628 | -0.450 | 0.883 |
| -NA | 6 | 1.1126 | 0.32628 | 0.446 | 1.779 |
| 50nM+NA | 6 | -0.9131 | 0.32628 | -1.579 | -0.247 |
| 5nM+NA | 6 | -0.7824 | 0.32628 | -1.449 | -0.116 |
| +NA | 6 | 0.2486 | 0.32628 | -0.418 | 0.915 |

Std Error uses a pooled estimate of error variance

### Means Comparisons

#### Comparisons with a control using Dunnett's Method

## Oneway Analysis of S-adenosyl-L-methionine By Label

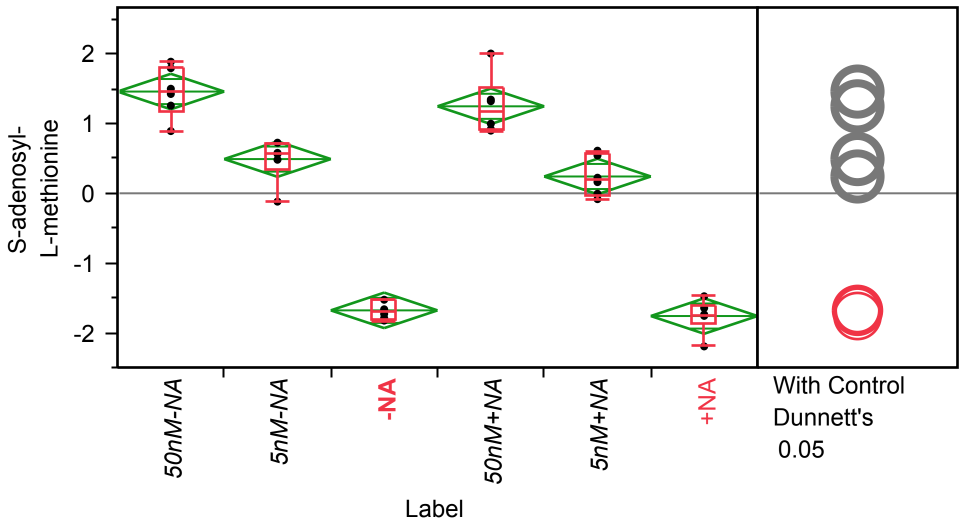


### Quantiles

| Level | Minimum | 10% | 25% | Median | 75% | 90% | Maximum |
|---|---|---|---|---|---|---|---|
| 50nM-NA | 0.892936 | 0.892936 | 1.167066 | 1.463023 | 1.818878 | 1.88829 | 1.88829 |
| 5nM-NA | -0.11938 | -0.11938 | 0.331596 | 0.571816 | 0.71792 | 0.726332 | 0.726332 |
| -NA | -1.82686 | -1.82686 | -1.79896 | -1.69843 | -1.53643 | -1.52748 | -1.52748 |
| 50nM+NA | 0.899371 | 0.899371 | 0.915422 | 1.160153 | 1.509943 | 2.005553 | 2.005553 |
| 5nM+NA | -0.0838 | -0.0838 | -0.02574 | 0.192143 | 0.560195 | 0.611283 | 0.611283 |
| +NA | -2.19729 | -2.19729 | -1.86862 | -1.74363 | -1.60438 | -1.47793 | -1.47793 |

### Oneway Anova

#### Summary of Fit

| | |
|---|---|
| Rsquare | 0.955085 |
| Adj Rsquare | 0.947599 |
| Root Mean Square Error | 0.305384 |
| Mean of Response | 2.78e-11 |
| Observations (or Sum Wgts) | 36 |

#### Analysis of Variance

| Source | DF | Sum of Squares | Mean Square | F Ratio | Prob > F |
|---|---|---|---|---|---|
| Label | 5 | 59.492950 | 11.8986 | 127.5858 | <.0001 * |
| Error | 30 | 2.797786 | 0.0933 | | |
| C. Total | 35 | 62.290736 | | | |

#### Means for Oneway Anova

| Level | Number | Mean | Std Error | Lower 95% | Upper 95% |
|---|---|---|---|---|---|
| 50nM-NA | 6 | 1.4602 | 0.12467 | 1.206 | 1.715 |
| 5nM-NA | 6 | 0.4913 | 0.12467 | 0.237 | 0.746 |
| -NA | 6 | -1.6800 | 0.12467 | -1.935 | -1.425 |
| 50nM+NA | 6 | 1.2485 | 0.12467 | 0.994 | 1.503 |
| 5nM+NA | 6 | 0.2414 | 0.12467 | -0.013 | 0.496 |
| +NA | 6 | -1.7613 | 0.12467 | -2.016 | -1.507 |

Std Error uses a pooled estimate of error variance

### Means Comparisons

#### Comparisons with a control using Dunnett's Method

## Oneway Analysis of sarcosine By Label

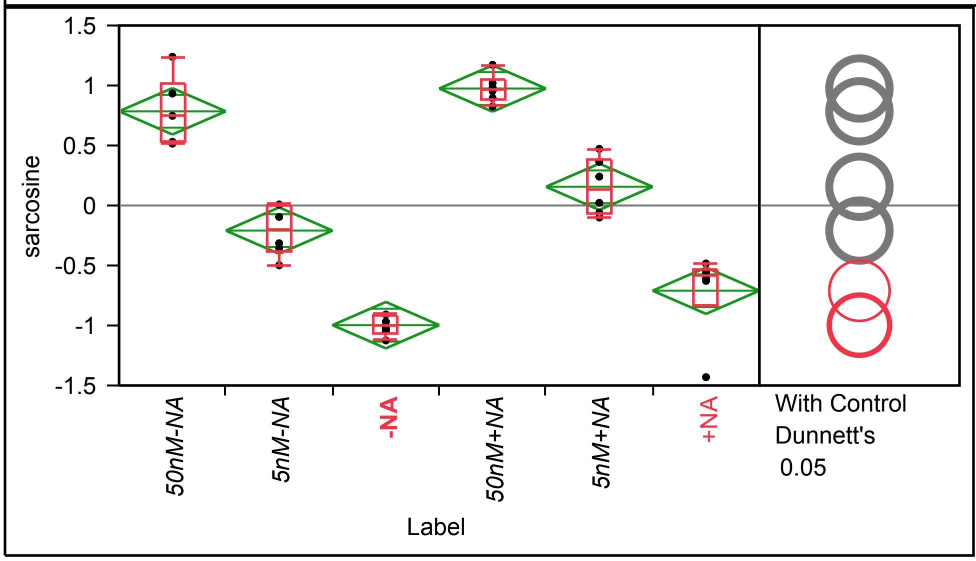


## Quantiles

| Level | Minimum | 10% | 25% | Median | 75% | 90% | Maximum |
|---|---|---|---|---|---|---|---|
| 50nM-NA | 0.515654 | 0.515654 | 0.526971 | 0.745606 | 1.009568 | 1.239164 | 1.239164 |
| 5nM-NA | -0.49816 | -0.49816 | -0.39093 | -0.20492 | 0.002713 | 0.008469 | 0.008469 |
| -NA | -1.12466 | -1.12466 | -1.0653 | -0.99635 | -0.91007 | -0.90832 | -0.90832 |
| 50nM+NA | 0.825332 | 0.825332 | 0.877911 | 0.969126 | 1.057009 | 1.171985 | 1.171985 |
| 5nM+NA | -0.10014 | -0.10014 | -0.07083 | 0.131454 | 0.390166 | 0.471782 | 0.471782 |
| +NA | -1.43059 | -1.43059 | -0.8286 | -0.58311 | -0.53488 | -0.48341 | -0.48341 |

## Oneway Anova

### Summary of Fit

| | |
|---|---|
| Rsquare | 0.921082 |
| Adj Rsquare | 0.907929 |
| Root Mean Square Error | 0.231698 |
| Mean of Response | -5.6e-11 |
| Observations (or Sum Wgts) | 36 |

### Analysis of Variance

| Source | DF | Sum of Squares | Mean Square | F Ratio | Prob > F |
|---|---|---|---|---|---|
| Label | 5 | 18.797010 | 3.75940 | 70.0282 | <.0001 * |
| Error | 30 | 1.610523 | 0.05368 | | |
| C. Total | 35 | 20.407533 | | | |

### Means for Oneway Anova

| Level | Number | Mean | Std Error | Lower 95% | Upper 95% |
|---|---|---|---|---|---|
| 50nM-NA | 6 | 0.78497 | 0.09459 | 0.592 | 0.978 |
| 5nM-NA | 6 | -0.20899 | 0.09459 | -0.402 | -0.016 |
| -NA | 6 | -0.99697 | 0.09459 | -1.190 | -0.804 |
| 50nM+NA | 6 | 0.97495 | 0.09459 | 0.782 | 1.168 |
| 5nM+NA | 6 | 0.15608 | 0.09459 | -0.037 | 0.349 |
| +NA | 6 | -0.71003 | 0.09459 | -0.903 | -0.517 |

Std Error uses a pooled estimate of error variance

## Means Comparisons

### Comparisons with a control using Dunnett's Method

## Oneway Analysis of Sedoheptulose 7-phosphate By Label

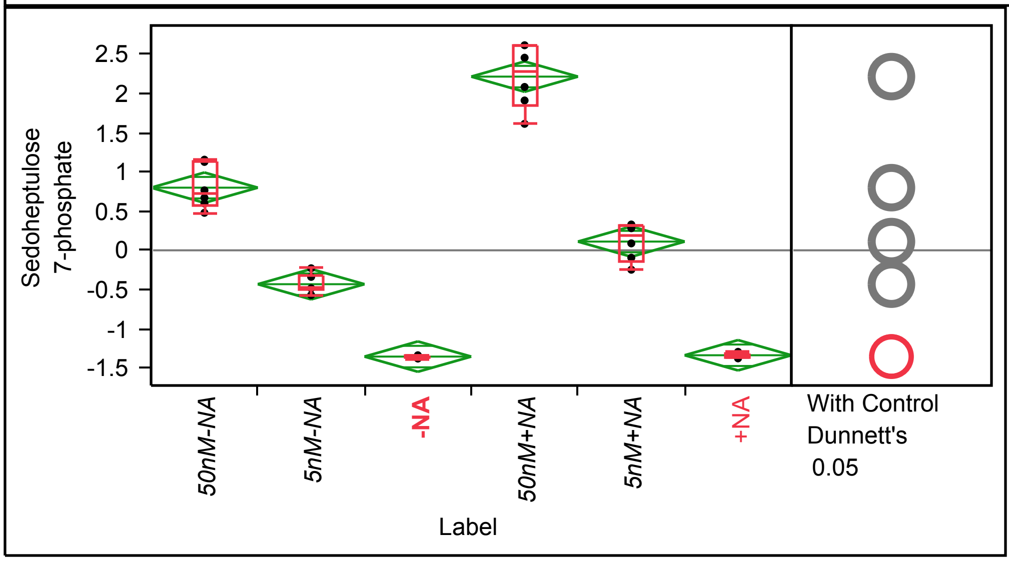


## Quantiles

| Level | Minimum | 10% | 25% | Median | 75% | 90% | Maximum |
|---|---|---|---|---|---|---|---|
| 50nM-NA | 0.478688 | 0.478688 | 0.568123 | 0.717802 | 1.135072 | 1.156223 | 1.156223 |
| 5nM-NA | -0.57117 | -0.57117 | -0.50702 | -0.48434 | -0.3117 | -0.22919 | -0.22919 |
| -NA | -1.38123 | -1.38123 | -1.3705 | -1.35381 | -1.34078 | -1.33931 | -1.33931 |
| 50nM+NA | 1.614224 | 1.614224 | 1.837646 | 2.271085 | 2.61454 | 2.616759 | 2.616759 |
| 5nM+NA | -0.24742 | -0.24742 | -0.13259 | 0.185391 | 0.315088 | 0.329218 | 0.329218 |
| +NA | -1.3785 | -1.3785 | -1.37362 | -1.33847 | -1.30696 | -1.29587 | -1.29587 |

## Oneway Anova

### Summary of Fit

| | |
|---|---|
| Rsquare | 0.972048 |
| Adj Rsquare | 0.96739 |
| Root Mean Square Error | 0.2323 |
| Mean of Response | -5.6e-10 |
| Observations (or Sum Wgts) | 36 |

### Analysis of Variance

| Source | DF | Sum of Squares | Mean Square | F Ratio | Prob > F |
|---|---|---|---|---|---|
| Label | 5 | 56.298573 | 11.2597 | 208.6557 | <.0001 * |
| Error | 30 | 1.618894 | 0.0540 | | |
| C. Total | 35 | 57.917466 | | | |

### Means for Oneway Anova

| Level | Number | Mean | Std Error | Lower 95% | Upper 95% |
|---|---|---|---|---|---|
| 50nM-NA | 6 | 0.7994 | 0.09484 | 0.606 | 0.993 |
| 5nM-NA | 6 | -0.4323 | 0.09484 | -0.626 | -0.239 |
| -NA | 6 | -1.3561 | 0.09484 | -1.550 | -1.162 |
| 50nM+NA | 6 | 2.2165 | 0.09484 | 2.023 | 2.410 |
| 5nM+NA | 6 | 0.1114 | 0.09484 | -0.082 | 0.305 |
| +NA | 6 | -1.3390 | 0.09484 | -1.533 | -1.145 |

Std Error uses a pooled estimate of error variance

## Means Comparisons

### Comparisons with a control using Dunnett's Method

## Oneway Analysis of serine By Label

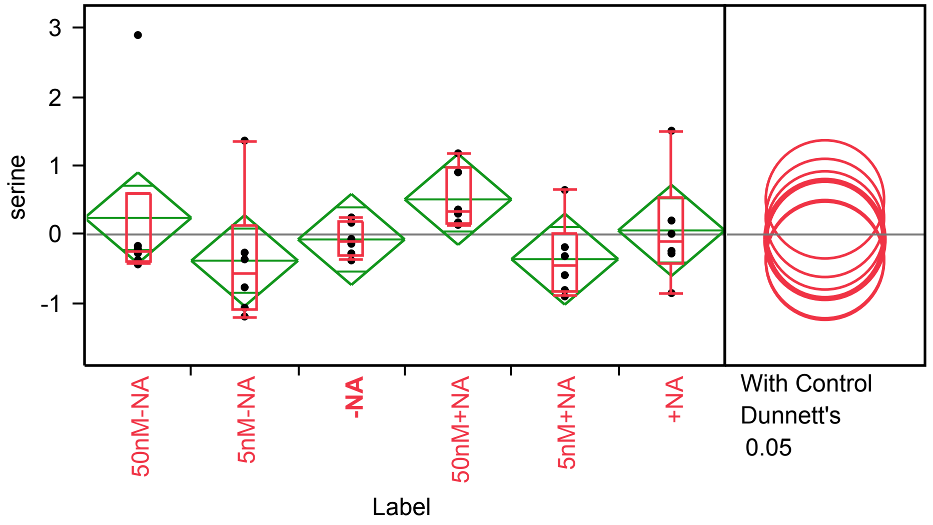


## Quantiles

| Level | Minimum | 10% | 25% | Median | 75% | 90% | Maximum |
|---|---|---|---|---|---|---|---|
| 50nM-NA | -0.4334 | -0.4334 | -0.38687 | -0.24036 | 0.599907 | 2.895512 | 2.895512 |
| 5nM-NA | -1.19091 | -1.19091 | -1.09558 | -0.56369 | 0.143071 | 1.362679 | 1.362679 |
| -NA | -0.37563 | -0.37563 | -0.29998 | -0.09951 | 0.189795 | 0.247586 | 0.247586 |
| 50nM+NA | 0.139118 | 0.139118 | 0.166225 | 0.332263 | 0.972137 | 1.178513 | 1.178513 |
| 5nM+NA | -0.89818 | -0.89818 | -0.82955 | -0.45184 | 0.023427 | 0.645867 | 0.645867 |
| +NA | -0.85081 | -0.85081 | -0.4191 | -0.11416 | 0.528842 | 1.506914 | 1.506914 |

## Oneway Anova

### Summary of Fit

| | |
|---|---|
| Rsquare | 0.160773 |
| Adj Rsquare | 0.020902 |
| Root Mean Square Error | 0.791226 |
| Mean of Response | 2.78e-10 |
| Observations (or Sum Wgts) | 36 |

### Analysis of Variance

| Source | DF | Sum of Squares | Mean Square | F Ratio | Prob > F |
|---|---|---|---|---|---|
| Label | 5 | 3.597966 | 0.719593 | 1.1494 | 0.3566 |
| Error | 30 | 18.781177 | 0.626039 | | |
| C. Total | 35 | 22.379143 | | | |

### Means for Oneway Anova

| Level | Number | Mean | Std Error | Lower 95% | Upper 95% |
|---|---|---|---|---|---|
| 50nM-NA | 6 | 0.24079 | 0.32302 | -0.419 | 0.9005 |
| 5nM-NA | 6 | -0.38048 | 0.32302 | -1.040 | 0.2792 |
| -NA | 6 | -0.07188 | 0.32302 | -0.732 | 0.5878 |
| 50nM+NA | 6 | 0.51013 | 0.32302 | -0.150 | 1.1698 |
| 5nM+NA | 6 | -0.35779 | 0.32302 | -1.017 | 0.3019 |
| +NA | 6 | 0.05923 | 0.32302 | -0.600 | 0.7189 |

Std Error uses a pooled estimate of error variance

## Means Comparisons

### Comparisons with a control using Dunnett's Method

## Oneway Analysis of S-methyl-5-thioadenosine By Label

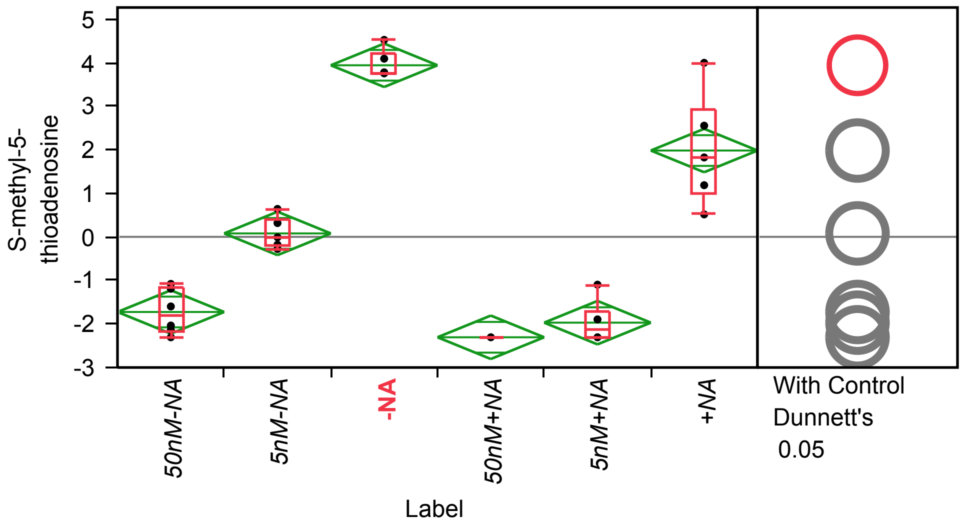


## Quantiles

| Level | Minimum | 10% | 25% | Median | 75% | 90% | Maximum |
|---|---|---|---|---|---|---|---|
| 50nM-NA | -2.30605 | -2.30605 | -2.18061 | -1.81741 | -1.16312 | -1.08015 | -1.08015 |
| 5nM-NA | -0.27768 | -0.27768 | -0.20465 | -0.01276 | 0.397849 | 0.64082 | 0.64082 |
| -NA | 3.731959 | 3.731959 | 3.748611 | 3.769813 | 4.19938 | 4.52303 | 4.52303 |
| 50nM+NA | -2.30606 | -2.30606 | -2.30605 | -2.30604 | -2.30602 | -2.30602 | -2.30602 |
| 5nM+NA | -2.30605 | -2.30605 | -2.30604 | -2.10787 | -1.69599 | -1.09813 | -1.09813 |
| +NA | 0.516094 | 0.516094 | 1.018509 | 1.821133 | 2.913863 | 3.99621 | 3.99621 |

## Oneway Anova

### Summary of Fit

| | |
|---|---|
| Rsquare | 0.946489 |
| Adj Rsquare | 0.93757 |
| Root Mean Square Error | 0.598087 |
| Mean of Response | -2.8e-10 |
| Observations (or Sum Wgts) | 36 |

### Analysis of Variance

| Source | DF | Sum of Squares | Mean Square | F Ratio | Prob > F |
|---|---|---|---|---|---|
| Label | 5 | 189.81061 | 37.9621 | 106.1262 | <.0001 * |
| Error | 30 | 10.73123 | 0.3577 | | |
| C. Total | 35 | 200.54183 | | | |

### Means for Oneway Anova

| Level | Number | Mean | Std Error | Lower 95% | Upper 95% |
|---|---|---|---|---|---|
| 50nM-NA | 6 | -1.7251 | 0.24417 | -2.224 | -1.226 |
| 5nM-NA | 6 | 0.0790 | 0.24417 | -0.420 | 0.578 |
| -NA | 6 | 3.9400 | 0.24417 | 3.441 | 4.439 |
| 50nM+NA | 6 | -2.3060 | 0.24417 | -2.805 | -1.807 |
| 5nM+NA | 6 | -1.9702 | 0.24417 | -2.469 | -1.472 |
| +NA | 6 | 1.9823 | 0.24417 | 1.484 | 2.481 |

Std Error uses a pooled estimate of error variance

## Means Comparisons

### Comparisons with a control using Dunnett's Method

## Oneway Analysis of sn-glycerol-3-phosphate By Label

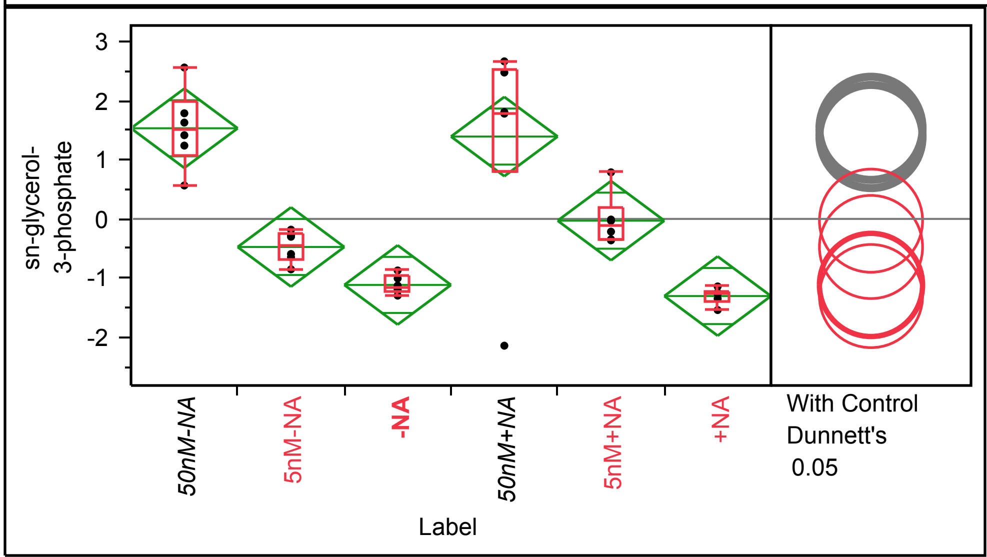


### Quantiles

| Level | Minimum | 10% | 25% | Median | 75% | 90% | Maximum |
|---|---|---|---|---|---|---|---|
| 50nM-NA | 0.565834 | 0.565834 | 1.072402 | 1.524288 | 1.982651 | 2.563416 | 2.563416 |
| 5nM-NA | -0.85367 | -0.85367 | -0.69989 | -0.44836 | -0.24865 | -0.18271 | -0.18271 |
| -NA | -1.29876 | -1.29876 | -1.23522 | -1.15687 | -0.97371 | -0.87299 | -0.87299 |
| 50nM+NA | -2.14679 | -2.14679 | 0.794685 | 1.796583 | 2.526011 | 2.667695 | 2.667695 |
| 5nM+NA | -0.36287 | -0.36287 | -0.34981 | -0.12408 | 0.188988 | 0.788942 | 0.788942 |
| +NA | -1.54324 | -1.54324 | -1.39936 | -1.27738 | -1.22013 | -1.1433 | -1.1433 |

### Oneway Anova

#### Summary of Fit

| | |
|---|---|
| Rsquare | 0.697787 |
| Adj Rsquare | 0.647418 |
| Root Mean Square Error | 0.805122 |
| Mean of Response | 2.78e-10 |
| Observations (or Sum Wgts) | 36 |

#### Analysis of Variance

| Source | DF | Sum of Squares | Mean Square | F Ratio | Prob > F |
|---|---|---|---|---|---|
| Label | 5 | 44.900765 | 8.98015 | 13.8535 | <.0001 * |
| Error | 30 | 19.446658 | 0.64822 | | |
| C. Total | 35 | 64.347423 | | | |

#### Means for Oneway Anova

| Level | Number | Mean | Std Error | Lower 95% | Upper 95% |
|---|---|---|---|---|---|
| 50nM-NA | 6 | 1.5347 | 0.32869 | 0.863 | 2.206 |
| 5nM-NA | 6 | -0.4754 | 0.32869 | -1.147 | 0.196 |
| -NA | 6 | -1.1178 | 0.32869 | -1.789 | -0.447 |
| 50nM+NA | 6 | 1.3947 | 0.32869 | 0.723 | 2.066 |
| 5nM+NA | 6 | -0.0298 | 0.32869 | -0.701 | 0.642 |
| +NA | 6 | -1.3064 | 0.32869 | -1.978 | -0.635 |

Std Error uses a pooled estimate of error variance

### Means Comparisons

#### Comparisons with a control using Dunnett's Method

## Oneway Analysis of Spermidine By Label

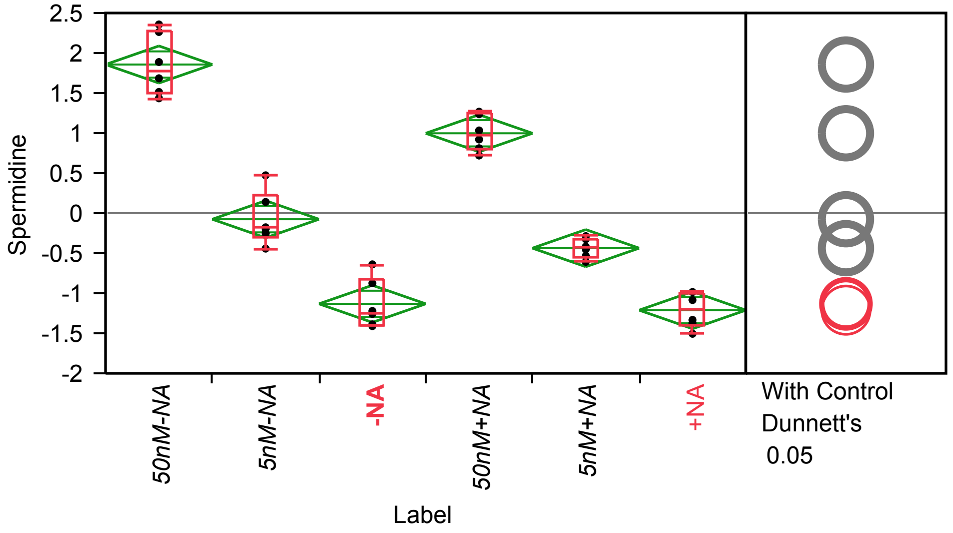


### Quantiles

| Level | Minimum | 10% | 25% | Median | 75% | 90% | Maximum |
|---|---|---|---|---|---|---|---|
| 50nM-NA | 1.434964 | 1.434964 | 1.494392 | 1.785927 | 2.286584 | 2.356894 | 2.356894 |
| 5nM-NA | -0.44217 | -0.44217 | -0.29921 | -0.18602 | 0.223392 | 0.472794 | 0.472794 |
| -NA | -1.40986 | -1.40986 | -1.3935 | -1.23949 | -0.81604 | -0.6392 | -0.6392 |
| 50nM+NA | 0.720422 | 0.720422 | 0.788359 | 0.976708 | 1.243664 | 1.267491 | 1.267491 |
| 5nM+NA | -0.60722 | -0.60722 | -0.55163 | -0.43459 | -0.31423 | -0.28682 | -0.28682 |
| +NA | -1.50593 | -1.50593 | -1.4034 | -1.2085 | -0.98784 | -0.98453 | -0.98453 |

### Oneway Anova

#### Summary of Fit

| | |
|---|---|
| Rsquare | 0.950266 |
| Adj Rsquare | 0.941978 |
| Root Mean Square Error | 0.278081 |
| Mean of Response | -5.56e-9 |
| Observations (or Sum Wgts) | 36 |

#### Analysis of Variance

| Source | DF | Sum of Squares | Mean Square | F Ratio | Prob > F |
|---|---|---|---|---|---|
| Label | 5 | 44.325992 | 8.86520 | 114.6429 | <.0001 * |
| Error | 30 | 2.319865 | 0.07733 | | |
| C. Total | 35 | 46.645857 | | | |

#### Means for Oneway Anova

| Level | Number | Mean | Std Error | Lower 95% | Upper 95% |
|---|---|---|---|---|---|
| 50nM-NA | 6 | 1.8568 | 0.11353 | 1.625 | 2.089 |
| 5nM-NA | 6 | -0.0755 | 0.11353 | -0.307 | 0.156 |
| -NA | 6 | -1.1318 | 0.11353 | -1.364 | -0.900 |
| 50nM+NA | 6 | 0.9980 | 0.11353 | 0.766 | 1.230 |
| 5nM+NA | 6 | -0.4366 | 0.11353 | -0.668 | -0.205 |
| +NA | 6 | -1.2109 | 0.11353 | -1.443 | -0.979 |

Std Error uses a pooled estimate of error variance

### Means Comparisons

#### Comparisons with a control using Dunnett's Method

## Oneway Analysis of Spermine By Label

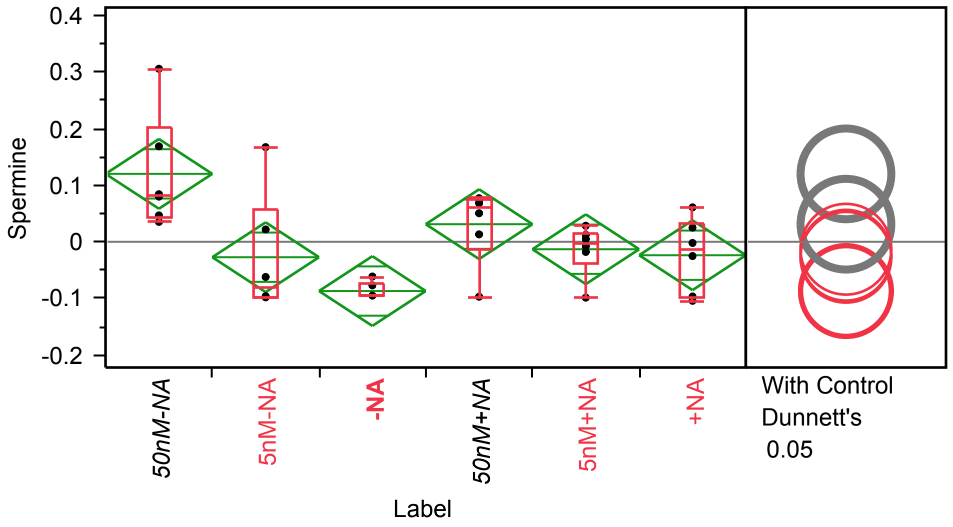


### Quantiles

| Level | Minimum | 10% | 25% | Median | 75% | 90% | Maximum |
|---|---|---|---|---|---|---|---|
| 50nM-NA | 0.035177 | 0.035177 | 0.043788 | 0.081927 | 0.202904 | 0.305657 | 0.305657 |
| 5nM-NA | -0.0981 | -0.0981 | -0.09664 | -0.0791 | 0.058152 | 0.167246 | 0.167246 |
| -NA | -0.09655 | -0.09655 | -0.09617 | -0.09552 | -0.07306 | -0.06135 | -0.06135 |
| 50nM+NA | -0.09707 | -0.09707 | -0.01441 | 0.059362 | 0.075349 | 0.077014 | 0.077014 |
| 5nM+NA | -0.09861 | -0.09861 | -0.03808 | -0.00153 | 0.016335 | 0.028343 | 0.028343 |
| +NA | -0.10425 | -0.10425 | -0.09852 | -0.01361 | 0.033895 | 0.060828 | 0.060828 |

### Oneway Anova

#### Summary of Fit

| | |
|---|---|
| Rsquare | 0.472232 |
| Adj Rsquare | 0.38427 |
| Root Mean Square Error | 0.07387 |
| Mean of Response | -5.6e-11 |
| Observations (or Sum Wgts) | 36 |

#### Analysis of Variance

| Source | DF | Sum of Squares | Mean Square | F Ratio | Prob > F |
|---|---|---|---|---|---|
| Label | 5 | 0.14647575 | 0.029295 | 5.3686 | 0.0012 * |
| Error | 30 | 0.16370204 | 0.005457 | | |
| C. Total | 35 | 0.31017780 | | | |

#### Means for Oneway Anova

| Level | Number | Mean | Std Error | Lower 95% | Upper 95% |
|---|---|---|---|---|---|
| 50nM-NA | 6 | 0.12000 | 0.03016 | 0.0584 | 0.1816 |
| 5nM-NA | 6 | -0.02724 | 0.03016 | -0.0888 | 0.0344 |
| -NA | 6 | -0.08699 | 0.03016 | -0.1486 | -0.0254 |
| 50nM+NA | 6 | 0.03110 | 0.03016 | -0.0305 | 0.0927 |
| 5nM+NA | 6 | -0.01315 | 0.03016 | -0.0747 | 0.0484 |
| +NA | 6 | -0.02372 | 0.03016 | -0.0853 | 0.0379 |

Std Error uses a pooled estimate of error variance

### Means Comparisons

#### Comparisons with a control using Dunnett's Method

## Oneway Analysis of Stearic acid By Label

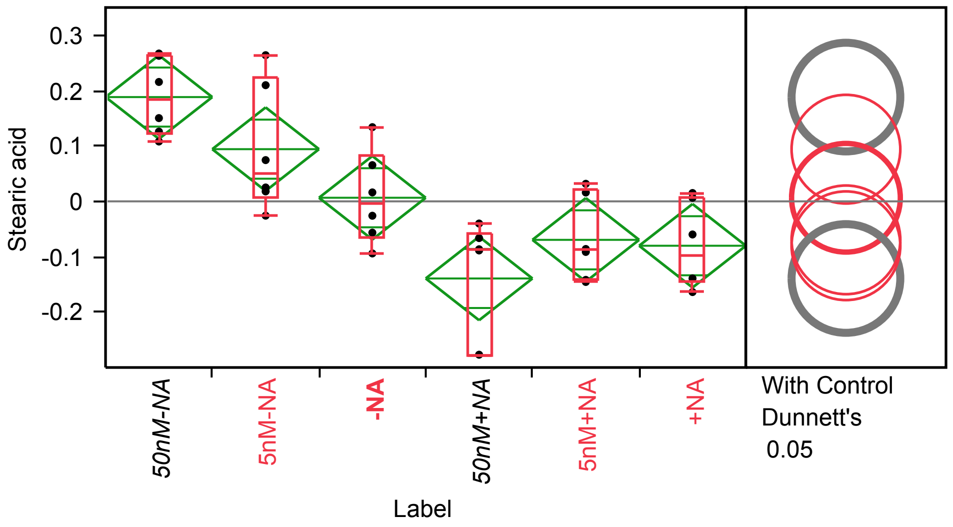


### Quantiles

| Level | Minimum | 10% | 25% | Median | 75% | 90% | Maximum |
|---|---|---|---|---|---|---|---|
| 50nM-NA | 0.108069 | 0.108069 | 0.12144 | 0.18293 | 0.263818 | 0.266905 | 0.266905 |
| 5nM-NA | -0.02583 | -0.02583 | 0.007005 | 0.049741 | 0.223281 | 0.263878 | 0.263878 |
| -NA | -0.09405 | -0.09405 | -0.06591 | -0.00494 | 0.082537 | 0.13394 | 0.13394 |
| 50nM+NA | -0.27702 | -0.27702 | -0.27694 | -0.0876 | -0.05975 | -0.04006 | -0.04006 |
| 5nM+NA | -0.14554 | -0.14554 | -0.14274 | -0.08869 | 0.019883 | 0.031673 | 0.031673 |
| +NA | -0.16379 | -0.16379 | -0.14577 | -0.09948 | 0.008597 | 0.015032 | 0.015032 |

### Oneway Anova

#### Summary of Fit

| | |
|---|---|
| Rsquare | 0.647079 |
| Adj Rsquare | 0.588259 |
| Root Mean Square Error | 0.090453 |
| Mean of Response | -2.8e-11 |
| Observations (or Sum Wgts) | 36 |

#### Analysis of Variance

| Source | DF | Sum of Squares | Mean Square | F Ratio | Prob > F |
|---|---|---|---|---|---|
| Label | 5 | 0.45003864 | 0.090008 | 11.0010 | <.0001 * |
| Error | 30 | 0.24545372 | 0.008182 | | |
| C. Total | 35 | 0.69549236 | | | |

#### Means for Oneway Anova

| Level | Number | Mean | Std Error | Lower 95% | Upper 95% |
|---|---|---|---|---|---|
| 50nM-NA | 6 | 0.18825 | 0.03693 | 0.1128 | 0.2637 |
| 5nM-NA | 6 | 0.09420 | 0.03693 | 0.0188 | 0.1696 |
| -NA | 6 | 0.00648 | 0.03693 | -0.0689 | 0.0819 |
| 50nM+NA | 6 | -0.13925 | 0.03693 | -0.2147 | -0.0638 |
| 5nM+NA | 6 | -0.06952 | 0.03693 | -0.1449 | 0.0059 |
| +NA | 6 | -0.08017 | 0.03693 | -0.1556 | -0.0048 |

Std Error uses a pooled estimate of error variance

### Means Comparisons

#### Comparisons with a control using Dunnett's Method

## Oneway Analysis of succinate By Label

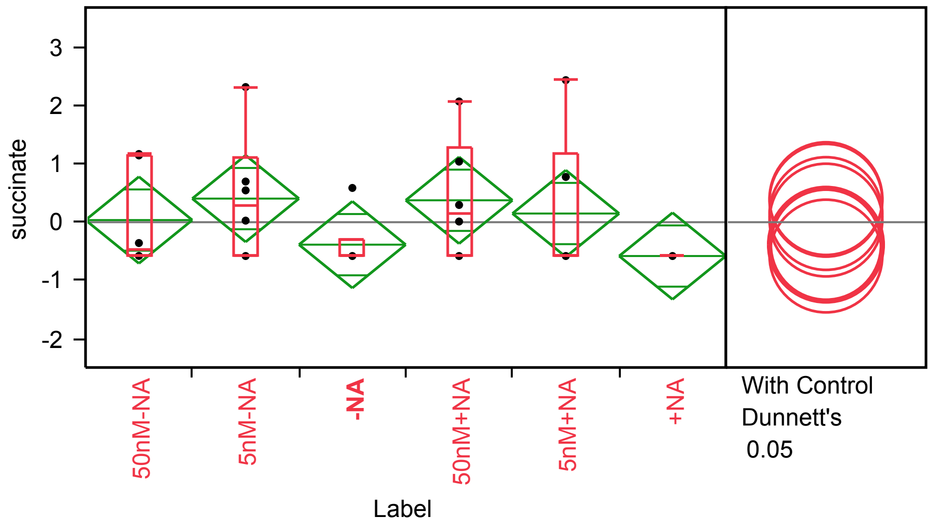


### Quantiles

| Level | Minimum | 10% | 25% | Median | 75% | 90% | Maximum |
|---|---|---|---|---|---|---|---|
| 50nM-NA | -0.5814 | -0.5814 | -0.58129 | -0.46866 | 1.153699 | 1.171845 | 1.171845 |
| 5nM-NA | -0.58119 | -0.58119 | -0.58118 | 0.286904 | 1.104726 | 2.322371 | 2.322371 |
| -NA | -0.58128 | -0.58128 | -0.58122 | -0.58117 | -0.28865 | 0.588637 | 0.588637 |
| 50nM+NA | -0.58133 | -0.58133 | -0.58133 | 0.153704 | 1.299436 | 2.074219 | 2.074219 |
| 5nM+NA | -0.5814 | -0.5814 | -0.58137 | -0.58121 | 1.193406 | 2.441066 | 2.441066 |
| +NA | -0.58203 | -0.58203 | -0.5816 | -0.58126 | -0.58124 | -0.58123 | -0.58123 |

### Oneway Anova

#### Summary of Fit

| | |
|---|---|
| Rsquare | 0.170113 |
| Adj Rsquare | 0.031798 |
| Root Mean Square Error | 0.89282 |
| Mean of Response | 8.33e-10 |
| Observations (or Sum Wgts) | 36 |

#### Analysis of Variance

| Source | DF | Sum of Squares | Mean Square | F Ratio | Prob > F |
|---|---|---|---|---|---|
| Label | 5 | 4.901921 | 0.980384 | 1.2299 | 0.3197 |
| Error | 30 | 23.913835 | 0.797128 | | |
| C. Total | 35 | 28.815755 | | | |

#### Means for Oneway Anova

| Level | Number | Mean | Std Error | Lower 95% | Upper 95% |
|---|---|---|---|---|---|
| 50nM-NA | 6 | 0.03659 | 0.36449 | -0.708 | 0.7810 |
| 5nM-NA | 6 | 0.40544 | 0.36449 | -0.339 | 1.1498 |
| -NA | 6 | -0.38621 | 0.36449 | -1.131 | 0.3582 |
| 50nM+NA | 6 | 0.37669 | 0.36449 | -0.368 | 1.1211 |
| 5nM+NA | 6 | 0.14890 | 0.36449 | -0.595 | 0.8933 |
| +NA | 6 | -0.58141 | 0.36449 | -1.326 | 0.1630 |

Std Error uses a pooled estimate of error variance

### Means Comparisons

#### Comparisons with a control using Dunnett's Method

## Oneway Analysis of succinyl-CoA By Label

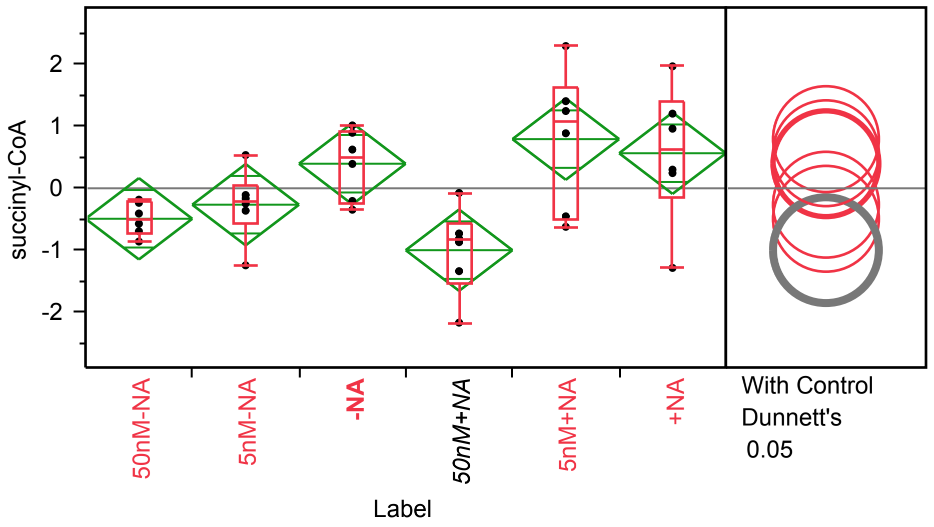


### Quantiles

| Level | Minimum | 10% | 25% | Median | 75% | 90% | Maximum |
|---|---|---|---|---|---|---|---|
| 50nM-NA | -0.85943 | -0.85943 | -0.7351 | -0.48941 | -0.22215 | -0.18394 | -0.18394 |
| 5nM-NA | -1.24289 | -1.24289 | -0.58203 | -0.20545 | 0.05241 | 0.535638 | 0.535638 |
| -NA | -0.34566 | -0.34566 | -0.24001 | 0.508779 | 0.92531 | 1.01415 | 1.01415 |
| 50nM+NA | -2.16759 | -2.16759 | -1.54458 | -0.8411 | -0.56554 | -0.07508 | -0.07508 |
| 5nM+NA | -0.6216 | -0.6216 | -0.49486 | 1.066945 | 1.627643 | 2.294303 | 2.294303 |
| +NA | -1.28477 | -1.28477 | -0.13727 | 0.630904 | 1.394883 | 1.967444 | 1.967444 |

### Oneway Anova

#### Summary of Fit

| | |
|---|---|
| Rsquare | 0.438335 |
| Adj Rsquare | 0.344725 |
| Root Mean Square Error | 0.786697 |
| Mean of Response | 8.33e-10 |
| Observations (or Sum Wgts) | 36 |

#### Analysis of Variance

| Source | DF | Sum of Squares | Mean Square | F Ratio | Prob > F |
|---|---|---|---|---|---|
| Label | 5 | 14.489902 | 2.89798 | 4.6825 | 0.0028 * |
| Error | 30 | 18.566759 | 0.61889 | | |
| C. Total | 35 | 33.056662 | | | |

#### Means for Oneway Anova

| Level | Number | Mean | Std Error | Lower 95% | Upper 95% |
|---|---|---|---|---|---|
| 50nM-NA | 6 | -0.49179 | 0.32117 | -1.148 | 0.164 |
| 5nM-NA | 6 | -0.26476 | 0.32117 | -0.921 | 0.391 |
| -NA | 6 | 0.39616 | 0.32117 | -0.260 | 1.052 |
| 50nM+NA | 6 | -0.99847 | 0.32117 | -1.654 | -0.343 |
| 5nM+NA | 6 | 0.79323 | 0.32117 | 0.137 | 1.449 |
| +NA | 6 | 0.56562 | 0.32117 | -0.090 | 1.222 |

Std Error uses a pooled estimate of error variance

### Means Comparisons

#### Comparisons with a control using Dunnett's Method

## Oneway Analysis of taurine By Label

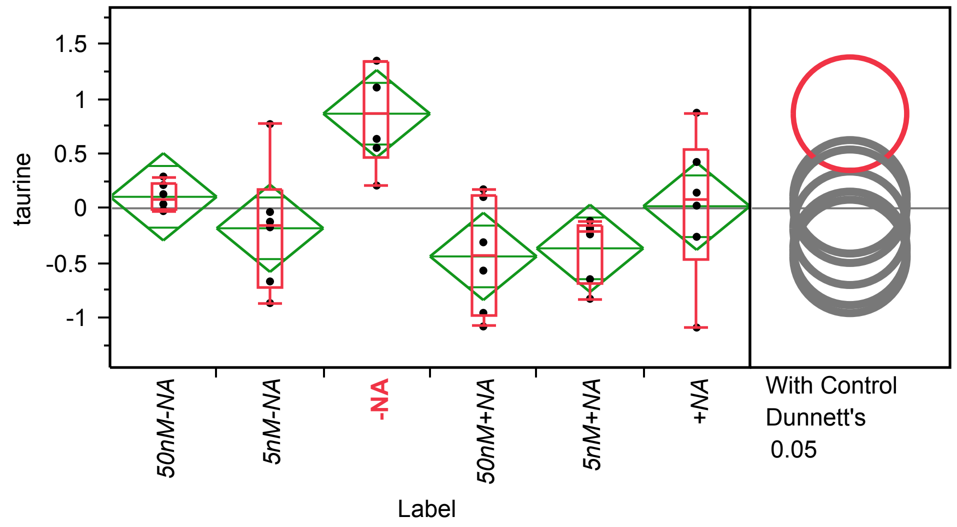


## Quantiles

| Level | Minimum | 10% | 25% | Median | 75% | 90% | Maximum |
|---|---|---|---|---|---|---|---|
| 50nM-NA | -0.02562 | -0.02562 | -0.01814 | 0.0839 | 0.232259 | 0.290243 | 0.290243 |
| 5nM-NA | -0.86663 | -0.86663 | -0.71823 | -0.1483 | 0.167176 | 0.769805 | 0.769805 |
| -NA | 0.207857 | 0.207857 | 0.466182 | 0.869531 | 1.343458 | 1.349467 | 1.349467 |
| 50nM+NA | -1.08042 | -1.08042 | -0.98722 | -0.44024 | 0.120682 | 0.173723 | 0.173723 |
| 5nM+NA | -0.82758 | -0.82758 | -0.69308 | -0.21467 | -0.16099 | -0.11526 | -0.11526 |
| +NA | -1.09056 | -1.09056 | -0.46814 | 0.083642 | 0.535911 | 0.873678 | 0.873678 |

## Oneway Anova

### Summary of Fit

| | |
|---|---|
| Rsquare | 0.494985 |
| Adj Rsquare | 0.410815 |
| Root Mean Square Error | 0.478203 |
| Mean of Response | -2.8e-10 |
| Observations (or Sum Wgts) | 36 |

### Analysis of Variance

| Source | DF | Sum of Squares | Mean Square | F Ratio | Prob > F |
|---|---|---|---|---|---|
| Label | 5 | 6.724081 | 1.34482 | 5.8808 | 0.0007 * |
| Error | 30 | 6.860341 | 0.22868 | | |
| C. Total | 35 | 13.584421 | | | |

### Means for Oneway Anova

| Level | Number | Mean | Std Error | Lower 95% | Upper 95% |
|---|---|---|---|---|---|
| 50nM-NA | 6 | 0.10495 | 0.19523 | -0.2938 | 0.504 |
| 5nM-NA | 6 | -0.18265 | 0.19523 | -0.5814 | 0.216 |
| -NA | 6 | 0.86502 | 0.19523 | 0.4663 | 1.264 |
| 50nM+NA | 6 | -0.44006 | 0.19523 | -0.8388 | -0.041 |
| 5nM+NA | 6 | -0.36611 | 0.19523 | -0.7648 | 0.033 |
| +NA | 6 | 0.01884 | 0.19523 | -0.3799 | 0.418 |

Std Error uses a pooled estimate of error variance

## Means Comparisons

### Comparisons with a control using Dunnett's Method

## Oneway Analysis of Taurodeoxycholic acid By Label

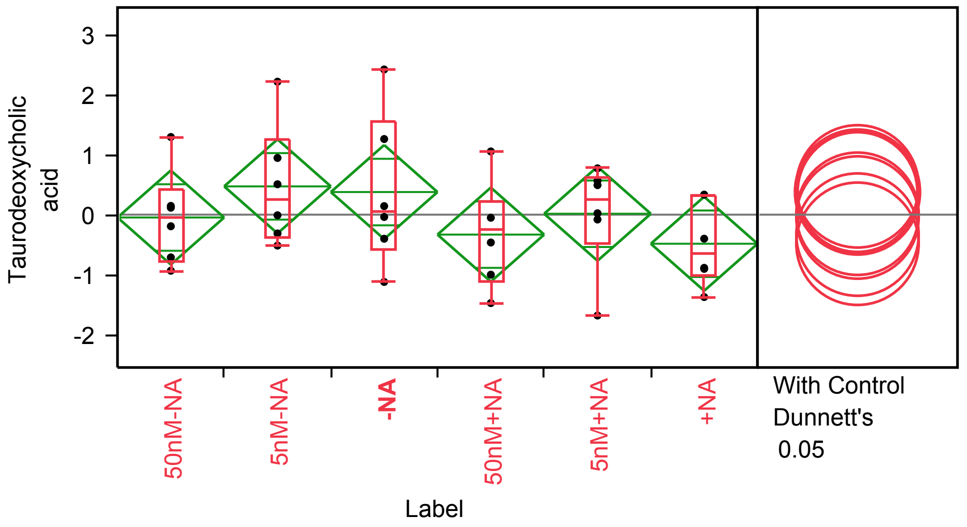


### Quantiles

| Level | Minimum | 10% | 25% | Median | 75% | 90% | Maximum |
|---|---|---|---|---|---|---|---|
| 50nM-NA | -0.93338 | -0.93338 | -0.76706 | -0.03853 | 0.432708 | 1.293274 | 1.293274 |
| 5nM-NA | -0.51455 | -0.51455 | -0.36362 | 0.248243 | 1.261925 | 2.214313 | 2.214313 |
| -NA | -1.1201 | -1.1201 | -0.581 | 0.052118 | 1.54917 | 2.417607 | 2.417607 |
| 50nM+NA | -1.47195 | -1.47195 | -1.11775 | -0.26076 | 0.224845 | 1.052753 | 1.052753 |
| 5nM+NA | -1.67941 | -1.67941 | -0.4802 | 0.257736 | 0.61678 | 0.771452 | 0.771452 |
| +NA | -1.37149 | -1.37149 | -1.01958 | -0.64287 | 0.323724 | 0.333762 | 0.333762 |

### Oneway Anova

#### Summary of Fit

| | |
|---|---|
| Rsquare | 0.138797 |
| Adj Rsquare | -0.00474 |
| Root Mean Square Error | 0.939363 |
| Mean of Response | -5.6e-11 |
| Observations (or Sum Wgts) | 36 |

#### Analysis of Variance

| Source | DF | Sum of Squares | Mean Square | F Ratio | Prob > F |
|---|---|---|---|---|---|
| Label | 5 | 4.266399 | 0.853280 | 0.9670 | 0.4536 |
| Error | 30 | 26.472105 | 0.882404 | | |
| C. Total | 35 | 30.738505 | | | |

#### Means for Oneway Anova

| Level | Number | Mean | Std Error | Lower 95% | Upper 95% |
|---|---|---|---|---|---|
| 50nM-NA | 6 | -0.04716 | 0.38349 | -0.830 | 0.7360 |
| 5nM-NA | 6 | 0.47123 | 0.38349 | -0.312 | 1.2544 |
| -NA | 6 | 0.37669 | 0.38349 | -0.407 | 1.1599 |
| 50nM+NA | 6 | -0.33192 | 0.38349 | -1.115 | 0.4513 |
| 5nM+NA | 6 | 0.01538 | 0.38349 | -0.768 | 0.7986 |
| +NA | 6 | -0.48423 | 0.38349 | -1.267 | 0.2990 |

Std Error uses a pooled estimate of error variance

### Means Comparisons

#### Comparisons with a control using Dunnett's Method

## Oneway Analysis of thiamine By Label

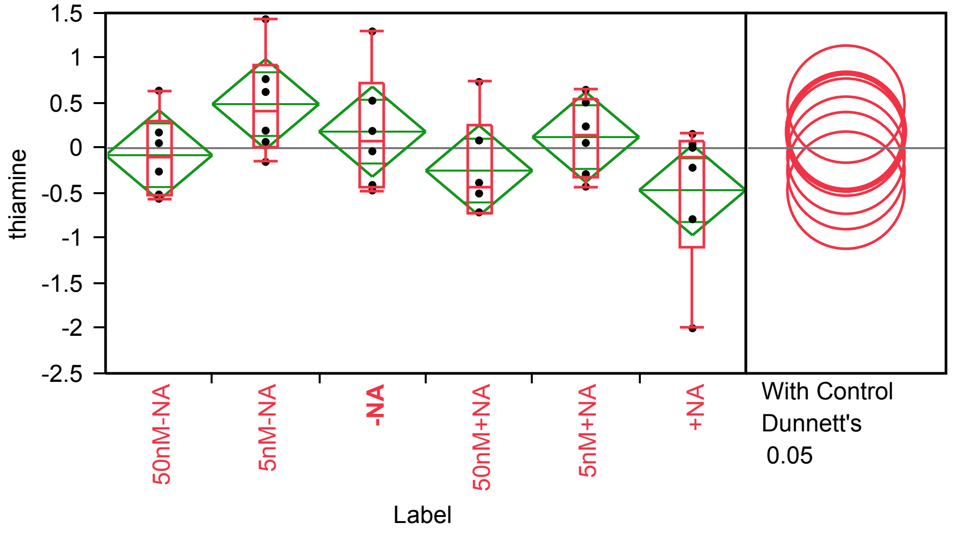


## Quantiles

| Level | Minimum | 10% | 25% | Median | 75% | 90% | Maximum |
|---|---|---|---|---|---|---|---|
| 50nM-NA | -0.56206 | -0.56206 | -0.5271 | -0.10237 | 0.289659 | 0.637949 | 0.637949 |
| 5nM-NA | -0.15249 | -0.15249 | 0.013563 | 0.408701 | 0.93275 | 1.431363 | 1.431363 |
| -NA | -0.46981 | -0.46981 | -0.42452 | 0.07764 | 0.718954 | 1.295683 | 1.295683 |
| 50nM+NA | -0.71366 | -0.71366 | -0.713 | -0.44395 | 0.247378 | 0.733857 | 0.733857 |
| 5nM+NA | -0.42732 | -0.42732 | -0.32374 | 0.149526 | 0.541473 | 0.646879 | 0.646879 |
| +NA | -1.99922 | -1.99922 | -1.09167 | -0.10638 | 0.07247 | 0.156909 | 0.156909 |

## Oneway Anova

### Summary of Fit

| | |
|---|---|
| Rsquare | 0.241865 |
| Adj Rsquare | 0.115509 |
| Root Mean Square Error | 0.599519 |
| Mean of Response | -8.3e-11 |
| Observations (or Sum Wgts) | 36 |

### Analysis of Variance

| Source | DF | Sum of Squares | Mean Square | F Ratio | Prob > F |
|---|---|---|---|---|---|
| Label | 5 | 3.439965 | 0.687993 | 1.9142 | 0.1214 |
| Error | 30 | 10.782693 | 0.359423 | | |
| C. Total | 35 | 14.222658 | | | |

### Means for Oneway Anova

| Level | Number | Mean | Std Error | Lower 95% | Upper 95% |
|---|---|---|---|---|---|
| 50nM-NA | 6 | -0.07845 | 0.24475 | -0.5783 | 0.42140 |
| 5nM-NA | 6 | 0.48862 | 0.24475 | -0.0112 | 0.98847 |
| -NA | 6 | 0.18307 | 0.24475 | -0.3168 | 0.68292 |
| 50nM+NA | 6 | -0.24921 | 0.24475 | -0.7491 | 0.25064 |
| 5nM+NA | 6 | 0.12262 | 0.24475 | -0.3772 | 0.62247 |
| +NA | 6 | -0.46665 | 0.24475 | -0.9665 | 0.03320 |

Std Error uses a pooled estimate of error variance

## Means Comparisons

### Comparisons with a control using Dunnett's Method

## Oneway Analysis of Thiamine pyrophosphate By Label

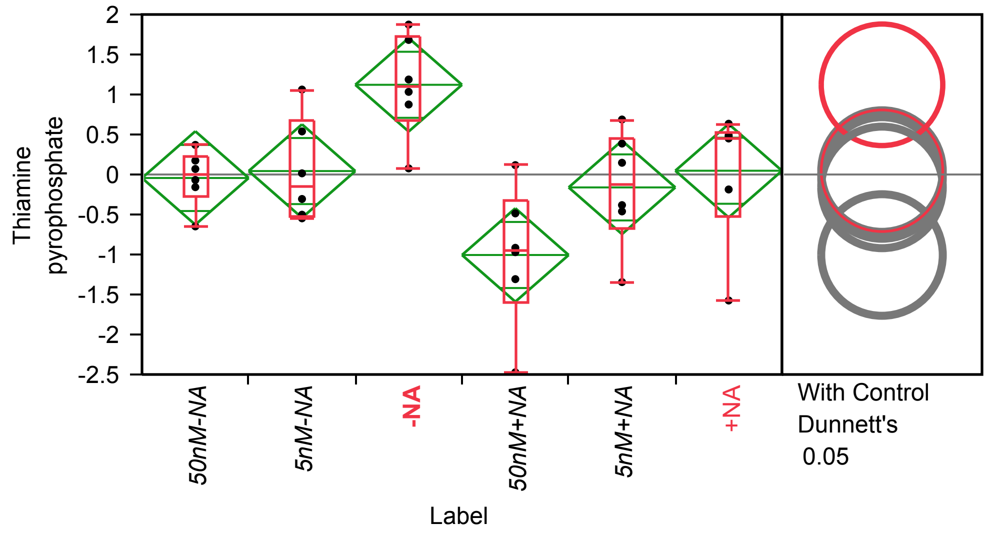


### Quantiles

| Level | Minimum | 10% | 25% | Median | 75% | 90% | Maximum |
|---|---|---|---|---|---|---|---|
| 50nM-NA | -0.64848 | -0.64848 | -0.28078 | 0.000494 | 0.222773 | 0.369871 | 0.369871 |
| 5nM-NA | -0.5476 | -0.5476 | -0.514 | -0.14576 | 0.669296 | 1.061585 | 1.061585 |
| -NA | 0.076468 | 0.076468 | 0.675339 | 1.110067 | 1.730078 | 1.873801 | 1.873801 |
| 50nM+NA | -2.473 | -2.473 | -1.59925 | -0.94374 | -0.33531 | 0.116909 | 0.116909 |
| 5nM+NA | -1.34461 | -1.34461 | -0.68207 | -0.1181 | 0.461731 | 0.687201 | 0.687201 |
| +NA | -1.5749 | -1.5749 | -0.53406 | 0.460211 | 0.525736 | 0.634288 | 0.634288 |

### Oneway Anova

#### Summary of Fit

| | |
|---|---|
| Rsquare | 0.484167 |
| Adj Rsquare | 0.398195 |
| Root Mean Square Error | 0.700336 |
| Mean of Response | 2.78e-10 |
| Observations (or Sum Wgts) | 36 |

#### Analysis of Variance

| Source | DF | Sum of Squares | Mean Square | F Ratio | Prob > F |
|---|---|---|---|---|---|
| Label | 5 | 13.810865 | 2.76217 | 5.6317 | 0.0009 * |
| Error | 30 | 14.714111 | 0.49047 | | |
| C. Total | 35 | 28.524976 | | | |

#### Means for Oneway Anova

| Level | Number | Mean | Std Error | Lower 95% | Upper 95% |
|---|---|---|---|---|---|
| 50nM-NA | 6 | -0.0437 | 0.28591 | -0.628 | 0.540 |
| 5nM-NA | 6 | 0.0430 | 0.28591 | -0.541 | 0.627 |
| -NA | 6 | 1.1213 | 0.28591 | 0.537 | 1.705 |
| 50nM+NA | 6 | -1.0063 | 0.28591 | -1.590 | -0.422 |
| 5nM+NA | 6 | -0.1614 | 0.28591 | -0.745 | 0.423 |
| +NA | 6 | 0.0470 | 0.28591 | -0.537 | 0.631 |

Std Error uses a pooled estimate of error variance

### Means Comparisons

#### Comparisons with a control using Dunnett's Method

# Oneway Analysis of threonine By Label

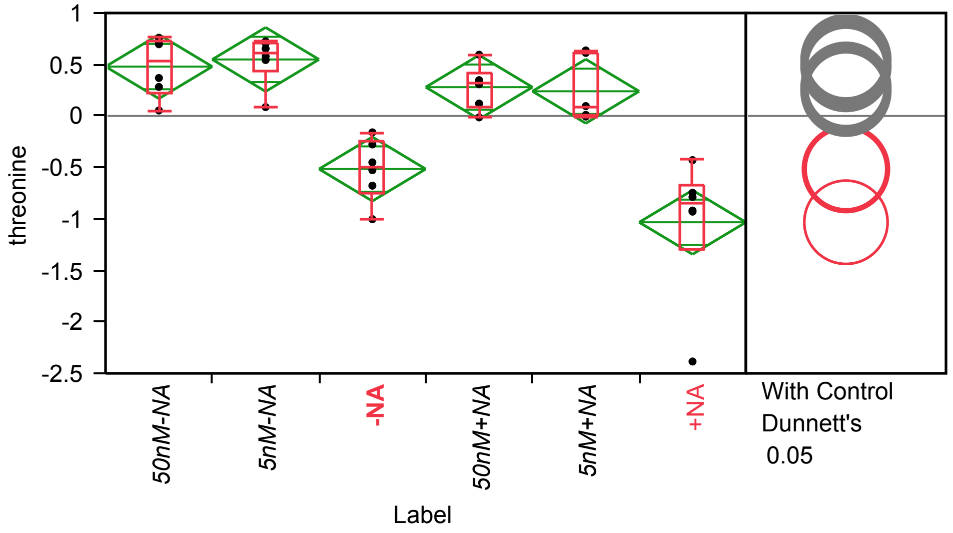


## Quantiles

| Level | Minimum | 10% | 25% | Median | 75% | 90% | Maximum |
|---|---|---|---|---|---|---|---|
| 50nM-NA | 0.053458 | 0.053458 | 0.225107 | 0.53281 | 0.728416 | 0.757369 | 0.757369 |
| 5nM-NA | 0.086544 | 0.086544 | 0.429257 | 0.616072 | 0.710496 | 0.725106 | 0.725106 |
| -NA | -1.00226 | -1.00226 | -0.7584 | -0.48866 | -0.24713 | -0.15946 | -0.15946 |
| 50nM+NA | -0.01363 | -0.01363 | 0.08704 | 0.314368 | 0.409646 | 0.594901 | 0.594901 |
| 5nM+NA | -0.00582 | -0.00582 | 0.006397 | 0.092446 | 0.618658 | 0.636386 | 0.636386 |
| +NA | -2.38491 | -2.38491 | -1.29329 | -0.853 | -0.66652 | -0.42913 | -0.42913 |

## Oneway Anova

### Summary of Fit

| | |
|---|---|
| Rsquare | 0.741686 |
| Adj Rsquare | 0.698633 |
| Root Mean Square Error | 0.373126 |
| Mean of Response | 0 |
| Observations (or Sum Wgts) | 36 |

### Analysis of Variance

| Source | DF | Sum of Squares | Mean Square | F Ratio | Prob > F |
|---|---|---|---|---|---|
| Label | 5 | 11.992300 | 2.39846 | 17.2275 | <.0001 * |
| Error | 30 | 4.176681 | 0.13922 | | |
| C. Total | 35 | 16.168982 | | | |

### Means for Oneway Anova

| Level | Number | Mean | Std Error | Lower 95% | Upper 95% |
|---|---|---|---|---|---|
| 50nM-NA | 6 | 0.4796 | 0.15233 | 0.168 | 0.7907 |
| 5nM-NA | 6 | 0.5488 | 0.15233 | 0.238 | 0.8599 |
| -NA | 6 | -0.5154 | 0.15233 | -0.827 | -0.2043 |
| 50nM+NA | 6 | 0.2798 | 0.15233 | -0.031 | 0.5908 |
| 5nM+NA | 6 | 0.2398 | 0.15233 | -0.071 | 0.5509 |
| +NA | 6 | -1.0325 | 0.15233 | -1.344 | -0.7214 |

Std Error uses a pooled estimate of error variance

## Means Comparisons

### Comparisons with a control using Dunnett's Method

## Oneway Analysis of thymidine By Label

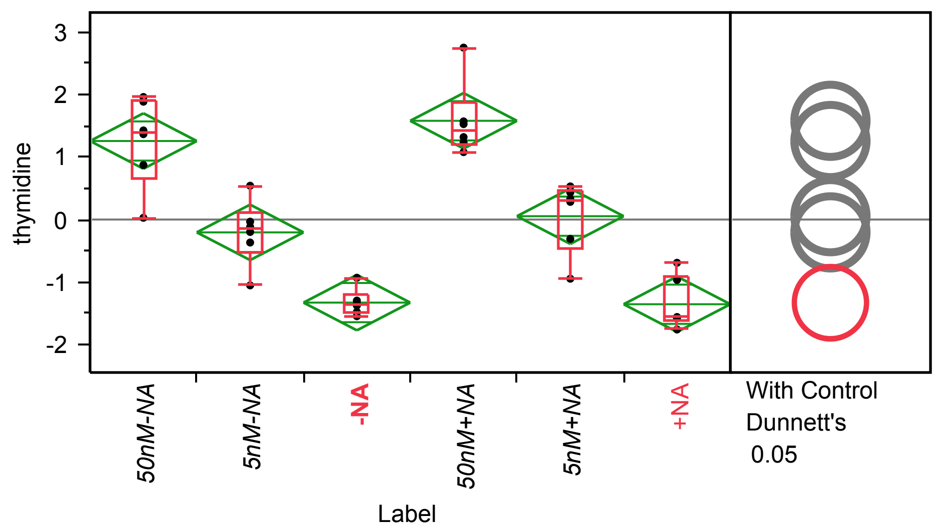


## Quantiles

| Level | Minimum | 10% | 25% | Median | 75% | 90% | Maximum |
|---|---|---|---|---|---|---|---|
| 50nM-NA | 0.027931 | 0.027931 | 0.660207 | 1.392875 | 1.900162 | 1.956319 | 1.956319 |
| 5nM-NA | -1.05133 | -1.05133 | -0.53807 | -0.15807 | 0.109302 | 0.53946 | 0.53946 |
| -NA | -1.54346 | -1.54346 | -1.49215 | -1.35744 | -1.20301 | -0.93014 | -0.93014 |
| 50nM+NA | 1.076407 | 1.076407 | 1.192145 | 1.419719 | 1.861351 | 2.744561 | 2.744561 |
| 5nM+NA | -0.94463 | -0.94463 | -0.46787 | 0.306041 | 0.456214 | 0.52888 | 0.52888 |
| +NA | -1.75543 | -1.75543 | -1.62052 | -1.56368 | -0.89598 | -0.69047 | -0.69047 |

## Oneway Anova

### Summary of Fit

| | |
|---|---|
| Rsquare | 0.845018 |
| Adj Rsquare | 0.819187 |
| Root Mean Square Error | 0.531076 |
| Mean of Response | 5.56e-10 |
| Observations (or Sum Wgts) | 36 |

### Analysis of Variance

| Source | DF | Sum of Squares | Mean Square | F Ratio | Prob > F |
|---|---|---|---|---|---|
| Label | 5 | 46.133648 | 9.22673 | 32.7141 | <.0001 * |
| Error | 30 | 8.461249 | 0.28204 | | |
| C. Total | 35 | 54.594897 | | | |

### Means for Oneway Anova

| Level | Number | Mean | Std Error | Lower 95% | Upper 95% |
|---|---|---|---|---|---|
| 50nM-NA | 6 | 1.2537 | 0.21681 | 0.811 | 1.697 |
| 5nM-NA | 6 | -0.2048 | 0.21681 | -0.648 | 0.238 |
| -NA | 6 | -1.3262 | 0.21681 | -1.769 | -0.883 |
| 50nM+NA | 6 | 1.5763 | 0.21681 | 1.134 | 2.019 |
| 5nM+NA | 6 | 0.0532 | 0.21681 | -0.390 | 0.496 |
| +NA | 6 | -1.3522 | 0.21681 | -1.795 | -0.909 |

Std Error uses a pooled estimate of error variance

## Means Comparisons

### Comparisons with a control using Dunnett's Method

## Oneway Analysis of tryptophan By Label

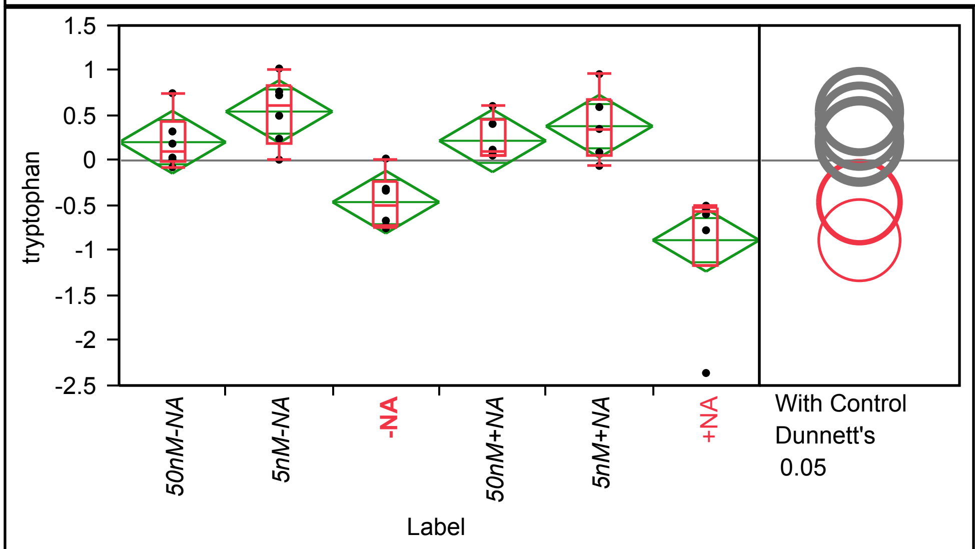


## Quantiles

| Level | Minimum | 10% | 25% | Median | 75% | 90% | Maximum |
|---|---|---|---|---|---|---|---|
| 50nM-NA | -0.0751 | -0.0751 | -0.01395 | 0.109927 | 0.428962 | 0.747655 | 0.747655 |
| 5nM-NA | 0.00905 | 0.00905 | 0.183872 | 0.611294 | 0.829548 | 1.021918 | 1.021918 |
| -NA | -0.75257 | -0.75257 | -0.73199 | -0.50173 | -0.22993 | 0.021553 | 0.021553 |
| 50nM+NA | 0.0551 | 0.0551 | 0.058743 | 0.094001 | 0.456159 | 0.605219 | 0.605219 |
| 5nM+NA | -0.05935 | -0.05935 | 0.054115 | 0.35061 | 0.685647 | 0.959398 | 0.959398 |
| +NA | -2.36114 | -2.36114 | -1.17166 | -0.56908 | -0.52669 | -0.50247 | -0.50247 |

## Oneway Anova

### Summary of Fit

| | |
|---|---|
| Rsquare | 0.637863 |
| Adj Rsquare | 0.577506 |
| Root Mean Square Error | 0.416482 |
| Mean of Response | -2.8e-11 |
| Observations (or Sum Wgts) | 36 |

### Analysis of Variance

| Source | DF | Sum of Squares | Mean Square | F Ratio | Prob > F |
|---|---|---|---|---|---|
| Label | 5 | 9.165748 | 1.83315 | 10.5683 | <.0001 * |
| Error | 30 | 5.203724 | 0.17346 | | |
| C. Total | 35 | 14.369472 | | | |

### Means for Oneway Anova

| Level | Number | Mean | Std Error | Lower 95% | Upper 95% |
|---|---|---|---|---|---|
| 50nM-NA | 6 | 0.20360 | 0.17003 | -0.144 | 0.5508 |
| 5nM-NA | 6 | 0.54352 | 0.17003 | 0.196 | 0.8908 |
| -NA | 6 | -0.46223 | 0.17003 | -0.809 | -0.1150 |
| 50nM+NA | 6 | 0.21913 | 0.17003 | -0.128 | 0.5664 |
| 5nM+NA | 6 | 0.38127 | 0.17003 | 0.034 | 0.7285 |
| +NA | 6 | -0.88528 | 0.17003 | -1.233 | -0.5380 |

Std Error uses a pooled estimate of error variance

## Means Comparisons

### Comparisons with a control using Dunnett's Method

## Oneway Analysis of tyrosine By Label

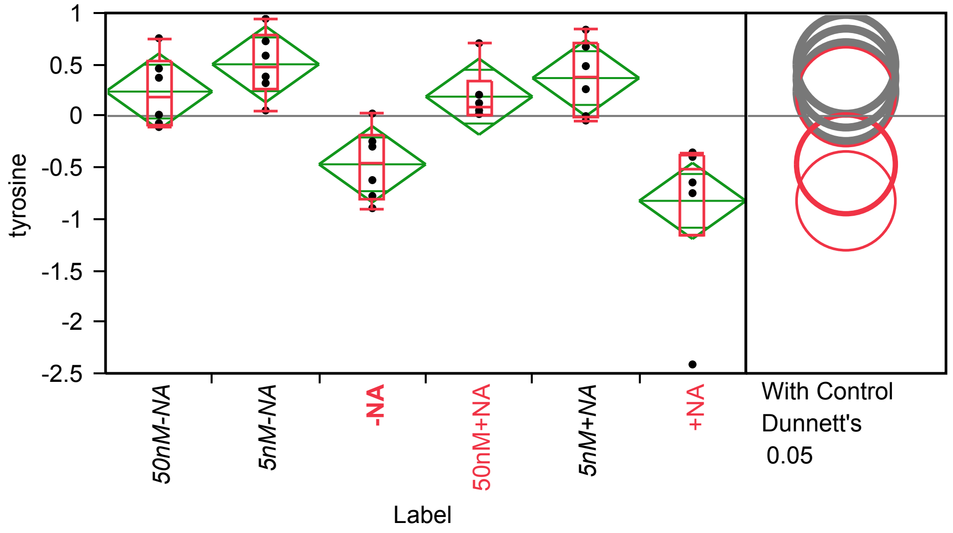


### Quantiles

| Level | Minimum | 10% | 25% | Median | 75% | 90% | Maximum |
|---|---|---|---|---|---|---|---|
| 50nM-NA | -0.10749 | -0.10749 | -0.07993 | 0.191518 | 0.534315 | 0.755137 | 0.755137 |
| 5nM-NA | 0.053672 | 0.053672 | 0.252766 | 0.484107 | 0.780514 | 0.943725 | 0.943725 |
| -NA | -0.89588 | -0.89588 | -0.80697 | -0.46044 | -0.17969 | 0.023672 | 0.023672 |
| 50nM+NA | 0.015703 | 0.015703 | 0.017824 | 0.089417 | 0.330702 | 0.706266 | 0.706266 |
| 5nM+NA | -0.04434 | -0.04434 | -0.01247 | 0.372257 | 0.714031 | 0.837656 | 0.837656 |
| +NA | -2.41347 | -2.41347 | -1.16566 | -0.52164 | -0.3784 | -0.35438 | -0.35438 |

### Oneway Anova

#### Summary of Fit

| | |
|---|---|
| Rsquare | 0.584718 |
| Adj Rsquare | 0.515504 |
| Root Mean Square Error | 0.442582 |
| Mean of Response | -5.6e-10 |
| Observations (or Sum Wgts) | 36 |

#### Analysis of Variance

| Source | DF | Sum of Squares | Mean Square | F Ratio | Prob > F |
|---|---|---|---|---|---|
| Label | 5 | 8.273931 | 1.65479 | 8.4480 | <.0001 * |
| Error | 30 | 5.876359 | 0.19588 | | |
| C. Total | 35 | 14.150290 | | | |

#### Means for Oneway Anova

| Level | Number | Mean | Std Error | Lower 95% | Upper 95% |
|---|---|---|---|---|---|
| 50nM-NA | 6 | 0.23678 | 0.18068 | -0.132 | 0.6058 |
| 5nM-NA | 6 | 0.50181 | 0.18068 | 0.133 | 0.8708 |
| -NA | 6 | -0.46965 | 0.18068 | -0.839 | -0.1006 |
| 50nM+NA | 6 | 0.18747 | 0.18068 | -0.182 | 0.5565 |
| 5nM+NA | 6 | 0.36814 | 0.18068 | -0.0009 | 0.7371 |
| +NA | 6 | -0.82454 | 0.18068 | -1.194 | -0.4555 |

Std Error uses a pooled estimate of error variance

### Means Comparisons

#### Comparisons with a control using Dunnett's Method

## Oneway Analysis of UDP By Label

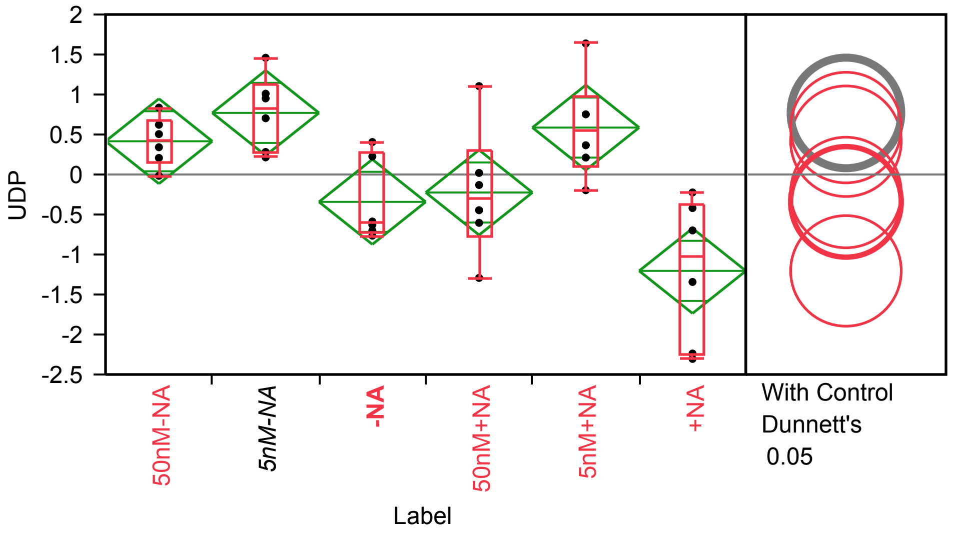


### Quantiles

| Level | Minimum | 10% | 25% | Median | 75% | 90% | Maximum |
|---|---|---|---|---|---|---|---|
| 50nM-NA | -0.0161 | -0.0161 | 0.151482 | 0.422856 | 0.675363 | 0.835872 | 0.835872 |
| 5nM-NA | 0.214115 | 0.214115 | 0.264427 | 0.827264 | 1.122157 | 1.459107 | 1.459107 |
| -NA | -0.76436 | -0.76436 | -0.71748 | -0.60938 | 0.270881 | 0.405568 | 0.405568 |
| 50nM+NA | -1.2928 | -1.2928 | -0.77622 | -0.28888 | 0.290903 | 1.104865 | 1.104865 |
| 5nM+NA | -0.19603 | -0.19603 | 0.109647 | 0.556636 | 0.97375 | 1.638252 | 1.638252 |
| +NA | -2.30479 | -2.30479 | -2.25476 | -1.02108 | -0.37084 | -0.22455 | -0.22455 |

### Oneway Anova

#### Summary of Fit

| | |
|---|---|
| Rsquare | 0.573537 |
| Adj Rsquare | 0.50246 |
| Root Mean Square Error | 0.637051 |
| Mean of Response | -2.8e-10 |
| Observations (or Sum Wgts) | 36 |

#### Analysis of Variance

| Source | DF | Sum of Squares | Mean Square | F Ratio | Prob > F |
|---|---|---|---|---|---|
| Label | 5 | 16.373801 | 3.27476 | 8.0692 | <.0001 * |
| Error | 30 | 12.175012 | 0.40583 | | |
| C. Total | 35 | 28.548812 | | | |

#### Means for Oneway Anova

| Level | Number | Mean | Std Error | Lower 95% | Upper 95% |
|---|---|---|---|---|---|
| 50nM-NA | 6 | 0.4158 | 0.26007 | -0.115 | 0.947 |
| 5nM-NA | 6 | 0.7698 | 0.26007 | 0.239 | 1.301 |
| -NA | 6 | -0.3422 | 0.26007 | -0.873 | 0.189 |
| 50nM+NA | 6 | -0.2250 | 0.26007 | -0.756 | 0.306 |
| 5nM+NA | 6 | 0.5865 | 0.26007 | 0.055 | 1.118 |
| +NA | 6 | -1.2049 | 0.26007 | -1.736 | -0.674 |

Std Error uses a pooled estimate of error variance

### Means Comparisons

#### Comparisons with a control using Dunnett's Method

## Oneway Analysis of UDP-D-glucose By Label

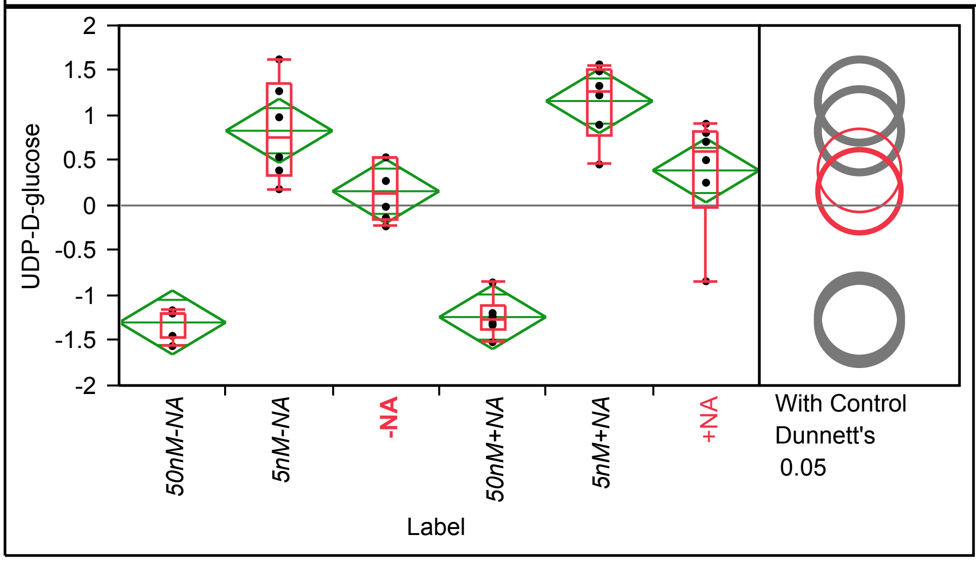


### Quantiles

| Level | Minimum | 10% | 25% | Median | 75% | 90% | Maximum |
|---|---|---|---|---|---|---|---|
| 50nM-NA | -1.56212 | -1.56212 | -1.47715 | -1.20989 | -1.19183 | -1.16498 | -1.16498 |
| 5nM-NA | 0.181401 | 0.181401 | 0.337238 | 0.759169 | 1.358584 | 1.623432 | 1.623432 |
| -NA | -0.23139 | -0.23139 | -0.1613 | 0.129291 | 0.5289 | 0.535191 | 0.535191 |
| 50nM+NA | -1.51876 | -1.51876 | -1.37636 | -1.27036 | -1.11035 | -0.85494 | -0.85494 |
| 5nM+NA | 0.456443 | 0.456443 | 0.786601 | 1.276531 | 1.50979 | 1.566503 | 1.566503 |
| +NA | -0.84139 | -0.84139 | -0.02034 | 0.605604 | 0.832226 | 0.907548 | 0.907548 |

### Oneway Anova

#### Summary of Fit

| | |
|---|---|
| Rsquare | 0.857026 |
| Adj Rsquare | 0.833197 |
| Root Mean Square Error | 0.426002 |
| Mean of Response | -8.3e-10 |
| Observations (or Sum Wgts) | 36 |

#### Analysis of Variance

| Source | DF | Sum of Squares | Mean Square | F Ratio | Prob > F |
|---|---|---|---|---|---|
| Label | 5 | 32.634849 | 6.52697 | 35.9657 | <.0001 * |
| Error | 30 | 5.444331 | 0.18148 | | |
| C. Total | 35 | 38.079180 | | | |

#### Means for Oneway Anova

| Level | Number | Mean | Std Error | Lower 95% | Upper 95% |
|---|---|---|---|---|---|
| 50nM-NA | 6 | -1.2994 | 0.17391 | -1.655 | -0.944 |
| 5nM-NA | 6 | 0.8304 | 0.17391 | 0.475 | 1.186 |
| -NA | 6 | 0.1585 | 0.17391 | -0.197 | 0.514 |
| 50nM+NA | 6 | -1.2398 | 0.17391 | -1.595 | -0.885 |
| 5nM+NA | 6 | 1.1606 | 0.17391 | 0.805 | 1.516 |
| +NA | 6 | 0.3896 | 0.17391 | 0.034 | 0.745 |

Std Error uses a pooled estimate of error variance

### Means Comparisons

#### Comparisons with a control using Dunnett's Method

## Oneway Analysis of UDP-D-glucuronate By Label

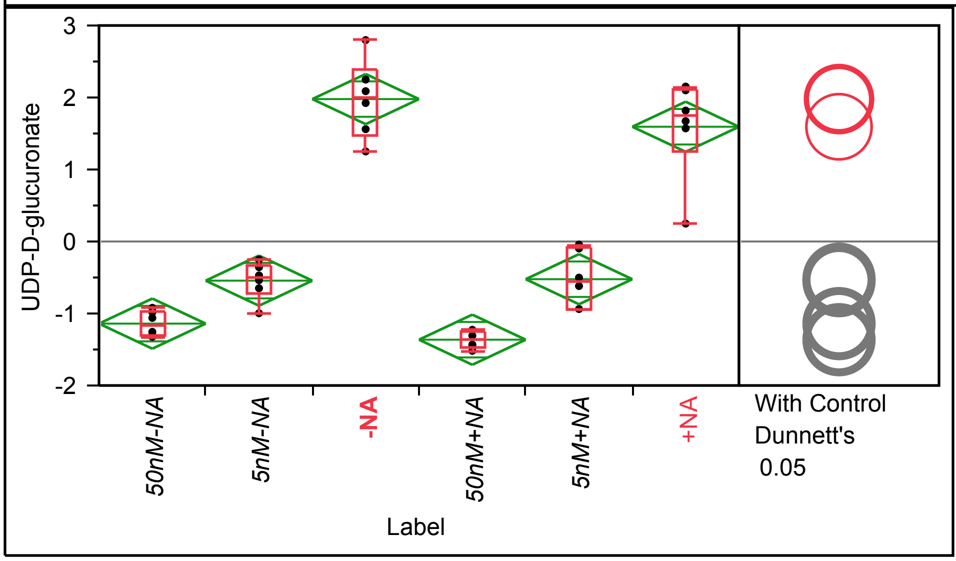


### Quantiles

| Level | Minimum | 10% | 25% | Median | 75% | 90% | Maximum |
|---|---|---|---|---|---|---|---|
| 50nM-NA | -1.32358 | -1.32358 | -1.30521 | -1.15812 | -0.9675 | -0.92102 | -0.92102 |
| 5nM-NA | -0.99638 | -0.99638 | -0.73593 | -0.5045 | -0.33058 | -0.25154 | -0.25154 |
| -NA | 1.251124 | 1.251124 | 1.482838 | 2.005629 | 2.385819 | 2.797112 | 2.797112 |
| 50nM+NA | -1.51863 | -1.51863 | -1.47364 | -1.36999 | -1.23611 | -1.22635 | -1.22635 |
| 5nM+NA | -0.94342 | -0.94342 | -0.93928 | -0.56036 | -0.08319 | -0.04292 | -0.04292 |
| +NA | 0.248746 | 0.248746 | 1.240968 | 1.745306 | 2.112761 | 2.151078 | 2.151078 |

### Oneway Anova

#### Summary of Fit

| | |
|---|---|
| Rsquare | 0.9211 |
| Adj Rsquare | 0.907949 |
| Root Mean Square Error | 0.417679 |
| Mean of Response | 2.78e-10 |
| Observations (or Sum Wgts) | 36 |

#### Analysis of Variance

| Source | DF | Sum of Squares | Mean Square | F Ratio | Prob > F |
|---|---|---|---|---|---|
| Label | 5 | 61.098926 | 12.2198 | 70.0452 | <.0001 * |
| Error | 30 | 5.233674 | 0.1745 | | |
| C. Total | 35 | 66.332600 | | | |

#### Means for Oneway Anova

| Level | Number | Mean | Std Error | Lower 95% | Upper 95% |
|---|---|---|---|---|---|
| 50nM-NA | 6 | -1.1405 | 0.17052 | -1.489 | -0.792 |
| 5nM-NA | 6 | -0.5438 | 0.17052 | -0.892 | -0.196 |
| -NA | 6 | 1.9780 | 0.17052 | 1.630 | 2.326 |
| 50nM+NA | 6 | -1.3638 | 0.17052 | -1.712 | -1.016 |
| 5nM+NA | 6 | -0.5236 | 0.17052 | -0.872 | -0.175 |
| +NA | 6 | 1.5937 | 0.17052 | 1.245 | 1.942 |

Std Error uses a pooled estimate of error variance

### Means Comparisons

#### Comparisons with a control using Dunnett's Method

## Oneway Analysis of UDP-N-acetyl-glucosamine By Label

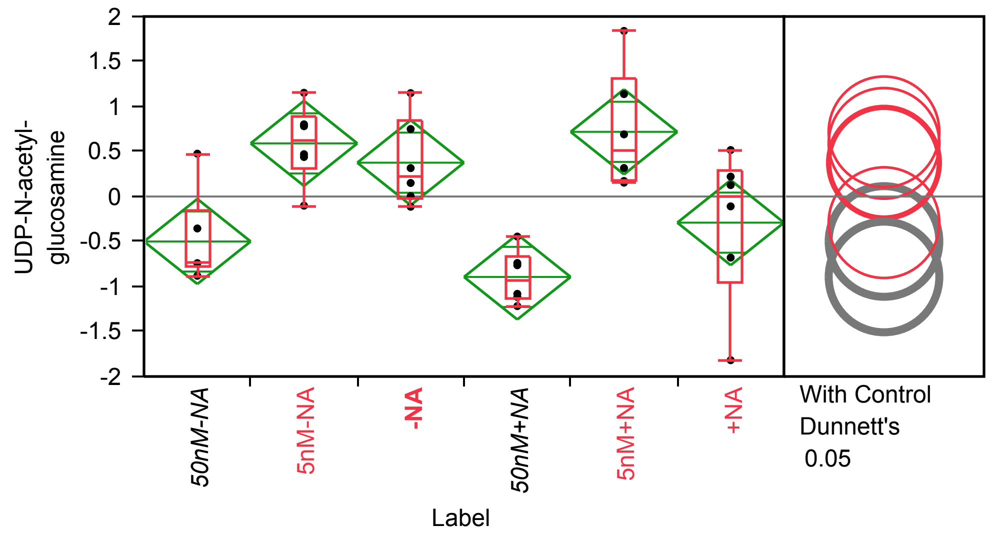


## Quantiles

| Level | Minimum | 10% | 25% | Median | 75% | 90% | Maximum |
|---|---|---|---|---|---|---|---|
| 50nM-NA | -0.88104 | -0.88104 | -0.78201 | -0.7444 | -0.1473 | 0.475667 | 0.475667 |
| 5nM-NA | -0.10193 | -0.10193 | 0.303712 | 0.625371 | 0.889444 | 1.152203 | 1.152203 |
| -NA | -0.11381 | -0.11381 | -0.0255 | 0.232764 | 0.849207 | 1.14853 | 1.14853 |
| 50nM+NA | -1.2178 | -1.2178 | -1.14222 | -0.92262 | -0.66542 | -0.44532 | -0.44532 |
| 5nM+NA | 0.153824 | 0.153824 | 0.167633 | 0.503269 | 1.313515 | 1.839819 | 1.839819 |
| +NA | -1.81865 | -1.81865 | -0.96404 | 0.008515 | 0.294754 | 0.516225 | 0.516225 |

## Oneway Anova

### Summary of Fit

| | |
|---|---|
| Rsquare | 0.571237 |
| Adj Rsquare | 0.499777 |
| Root Mean Square Error | 0.566667 |
| Mean of Response | 5.56e-11 |
| Observations (or Sum Wgts) | 36 |

### Analysis of Variance

| Source | DF | Sum of Squares | Mean Square | F Ratio | Prob > F |
|---|---|---|---|---|---|
| Label | 5 | 12.834462 | 2.56689 | 7.9938 | <.0001 * |
| Error | 30 | 9.633361 | 0.32111 | | |
| C. Total | 35 | 22.467823 | | | |

### Means for Oneway Anova

| Level | Number | Mean | Std Error | Lower 95% | Upper 95% |
|---|---|---|---|---|---|
| 50nM-NA | 6 | -0.49969 | 0.23134 | -0.972 | -0.027 |
| 5nM-NA | 6 | 0.59030 | 0.23134 | 0.118 | 1.063 |
| -NA | 6 | 0.37560 | 0.23134 | -0.097 | 0.848 |
| 50nM+NA | 6 | -0.89403 | 0.23134 | -1.366 | -0.422 |
| 5nM+NA | 6 | 0.71842 | 0.23134 | 0.246 | 1.191 |
| +NA | 6 | -0.29061 | 0.23134 | -0.763 | 0.182 |

Std Error uses a pooled estimate of error variance

## Means Comparisons

### Comparisons with a control using Dunnett's Method

## Oneway Analysis of Urea By Label

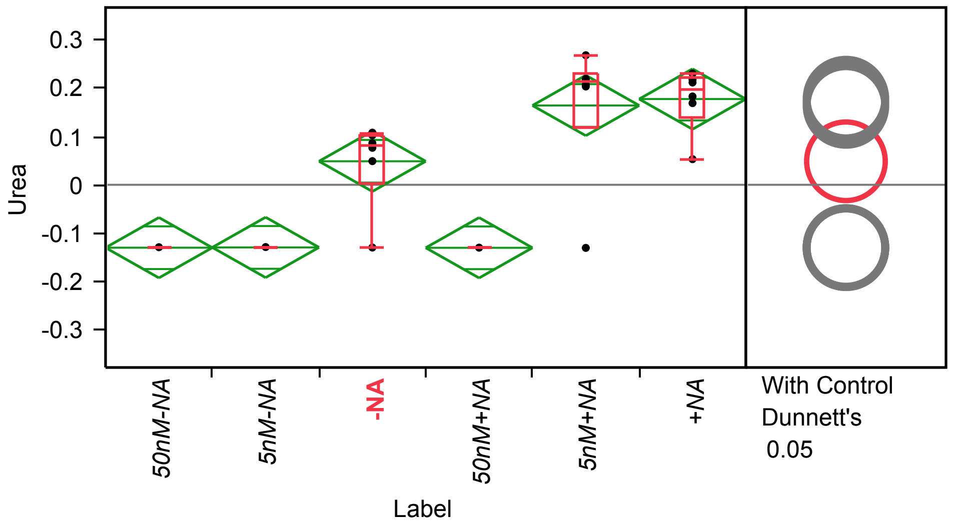


### Quantiles

| Level | Minimum | 10% | 25% | Median | 75% | 90% | Maximum |
|---|---|---|---|---|---|---|---|
| 50nM-NA | -0.13122 | -0.13122 | -0.13102 | -0.1308 | -0.13015 | -0.12957 | -0.12957 |
| 5nM-NA | -0.13116 | -0.13116 | -0.13051 | -0.12995 | -0.12943 | -0.12926 | -0.12926 |
| -NA | -0.13021 | -0.13021 | 0.004263 | 0.08154 | 0.104112 | 0.108186 | 0.108186 |
| 50nM+NA | -0.13116 | -0.13116 | -0.13098 | -0.13082 | -0.13053 | -0.13053 | -0.13053 |
| 5nM+NA | -0.13099 | -0.13099 | 0.12029 | 0.212943 | 0.232185 | 0.268921 | 0.268921 |
| +NA | 0.0532 | 0.0532 | 0.140466 | 0.197857 | 0.221574 | 0.230959 | 0.230959 |

### Oneway Anova

#### Summary of Fit

| | |
|---|---|
| Rsquare | 0.798846 |
| Adj Rsquare | 0.76532 |
| Root Mean Square Error | 0.075182 |
| Mean of Response | -2.8e-10 |
| Observations (or Sum Wgts) | 36 |

#### Analysis of Variance

| Source | DF | Sum of Squares | Mean Square | F Ratio | Prob > F |
|---|---|---|---|---|---|
| Label | 5 | 0.67341767 | 0.134684 | 23.8278 | <.0001 * |
| Error | 30 | 0.16957077 | 0.005652 | | |
| C. Total | 35 | 0.84298844 | | | |

#### Means for Oneway Anova

| Level | Number | Mean | Std Error | Lower 95% | Upper 95% |
|---|---|---|---|---|---|
| 50nM-NA | 6 | -0.13061 | 0.03069 | -0.1933 | -0.0679 |
| 5nM-NA | 6 | -0.13002 | 0.03069 | -0.1927 | -0.0673 |
| -NA | 6 | 0.04882 | 0.03069 | -0.0139 | 0.1115 |
| 50nM+NA | 6 | -0.13080 | 0.03069 | -0.1935 | -0.0681 |
| 5nM+NA | 6 | 0.16463 | 0.03069 | 0.1020 | 0.2273 |
| +NA | 6 | 0.17798 | 0.03069 | 0.1153 | 0.2407 |

Std Error uses a pooled estimate of error variance

### Means Comparisons

#### Comparisons with a control using Dunnett's Method

## Oneway Analysis of Uric acid By Label

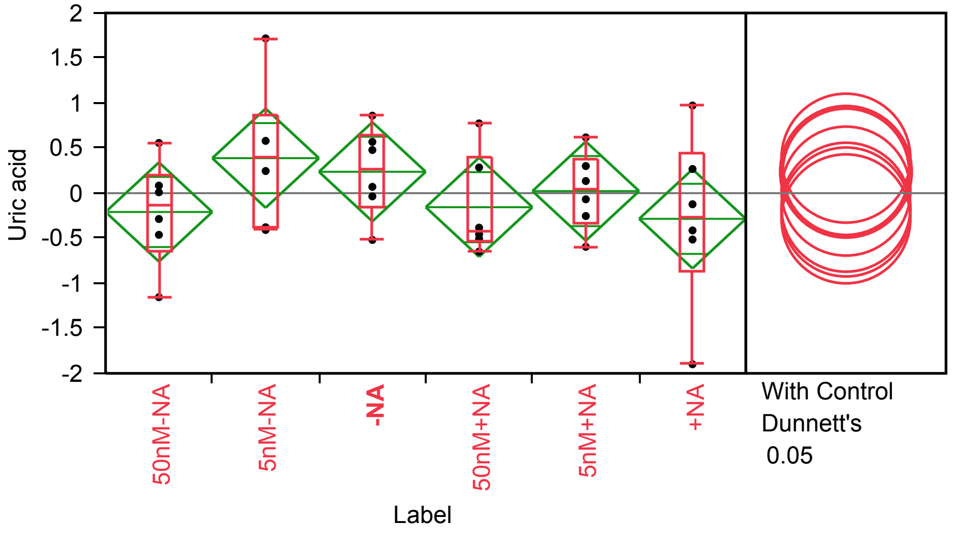


### Quantiles

| Level | Minimum | 10% | 25% | Median | 75% | 90% | Maximum |
|---|---|---|---|---|---|---|---|
| 50nM-NA | -1.15479 | -1.15479 | -0.63543 | -0.13831 | 0.202394 | 0.557297 | 0.557297 |
| 5nM-NA | -0.40815 | -0.40815 | -0.38595 | 0.409919 | 0.865069 | 1.718225 | 1.718225 |
| -NA | -0.5185 | -0.5185 | -0.15626 | 0.274785 | 0.642265 | 0.859976 | 0.859976 |
| 50nM+NA | -0.64842 | -0.64842 | -0.54222 | -0.41956 | 0.407642 | 0.774395 | 0.774395 |
| 5nM+NA | -0.59533 | -0.59533 | -0.33897 | 0.033504 | 0.380777 | 0.6192 | 0.6192 |
| +NA | -1.89911 | -1.89911 | -0.86072 | -0.26828 | 0.444443 | 0.972946 | 0.972946 |

### Oneway Anova

#### Summary of Fit

| | |
|---|---|
| Rsquare | 0.14082 |
| Adj Rsquare | -0.00238 |
| Root Mean Square Error | 0.660011 |
| Mean of Response | -6.2e-18 |
| Observations (or Sum Wgts) | 36 |

#### Analysis of Variance

| Source | DF | Sum of Squares | Mean Square | F Ratio | Prob > F |
|---|---|---|---|---|---|
| Label | 5 | 2.141917 | 0.428383 | 0.9834 | 0.4441 |
| Error | 30 | 13.068433 | 0.435614 | | |
| C. Total | 35 | 15.210351 | | | |

#### Means for Oneway Anova

| Level | Number | Mean | Std Error | Lower 95% | Upper 95% |
|---|---|---|---|---|---|
| 50nM-NA | 6 | -0.20872 | 0.26945 | -0.7590 | 0.34156 |
| 5nM-NA | 6 | 0.38868 | 0.26945 | -0.1616 | 0.93896 |
| -NA | 6 | 0.23754 | 0.26945 | -0.3127 | 0.78782 |
| 50nM+NA | 6 | -0.15576 | 0.26945 | -0.7060 | 0.39453 |
| 5nM+NA | 6 | 0.02311 | 0.26945 | -0.5272 | 0.57340 |
| +NA | 6 | -0.28484 | 0.26945 | -0.8351 | 0.26545 |

Std Error uses a pooled estimate of error variance

### Means Comparisons

#### Comparisons with a control using Dunnett's Method

## Oneway Analysis of uridine By Label

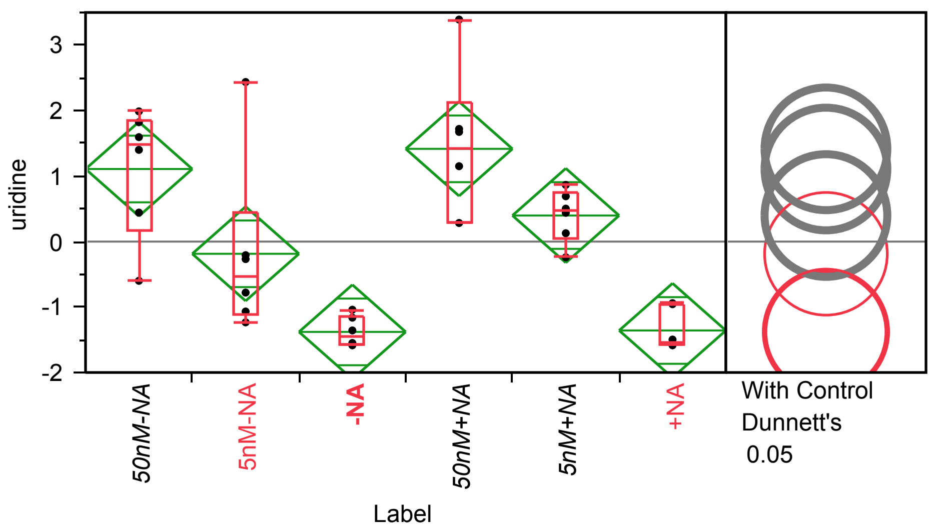


## Quantiles

| Level | Minimum | 10% | 25% | Median | 75% | 90% | Maximum |
|---|---|---|---|---|---|---|---|
| 50nM-NA | -0.59891 | -0.59891 | 0.182365 | 1.498594 | 1.865107 | 1.989222 | 1.989222 |
| 5nM-NA | -1.2338 | -1.2338 | -1.10972 | -0.52309 | 0.45375 | 2.437687 | 2.437687 |
| -NA | -1.58434 | -1.58434 | -1.58432 | -1.45331 | -1.13189 | -1.04112 | -1.04112 |
| 50nM+NA | 0.278015 | 0.278015 | 0.282486 | 1.413621 | 2.138696 | 3.391651 | 3.391651 |
| 5nM+NA | -0.23949 | -0.23949 | 0.035949 | 0.471266 | 0.735778 | 0.865941 | 0.865941 |
| +NA | -1.58489 | -1.58489 | -1.5846 | -1.5407 | -0.95263 | -0.94104 | -0.94104 |

## Oneway Anova

### Summary of Fit

| | |
|---|---|
| Rsquare | 0.658092 |
| Adj Rsquare | 0.601107 |
| Root Mean Square Error | 0.863695 |
| Mean of Response | 2.778e-9 |
| Observations (or Sum Wgts) | 36 |

### Analysis of Variance

| Source | DF | Sum of Squares | Mean Square | F Ratio | Prob > F |
|---|---|---|---|---|---|
| Label | 5 | 43.074416 | 8.61488 | 11.5486 | <.0001 * |
| Error | 30 | 22.379097 | 0.74597 | | |
| C. Total | 35 | 65.453513 | | | |

### Means for Oneway Anova

| Level | Number | Mean | Std Error | Lower 95% | Upper 95% |
|---|---|---|---|---|---|
| 50nM-NA | 6 | 1.1090 | 0.35260 | 0.389 | 1.829 |
| 5nM-NA | 6 | -0.1864 | 0.35260 | -0.906 | 0.534 |
| -NA | 6 | -1.3798 | 0.35260 | -2.100 | -0.660 |
| 50nM+NA | 6 | 1.4170 | 0.35260 | 0.697 | 2.137 |
| 5nM+NA | 6 | 0.3982 | 0.35260 | -0.322 | 1.118 |
| +NA | 6 | -1.3581 | 0.35260 | -2.078 | -0.638 |

Std Error uses a pooled estimate of error variance

## Means Comparisons

### Comparisons with a control using Dunnett's Method

## Oneway Analysis of UTP By Label

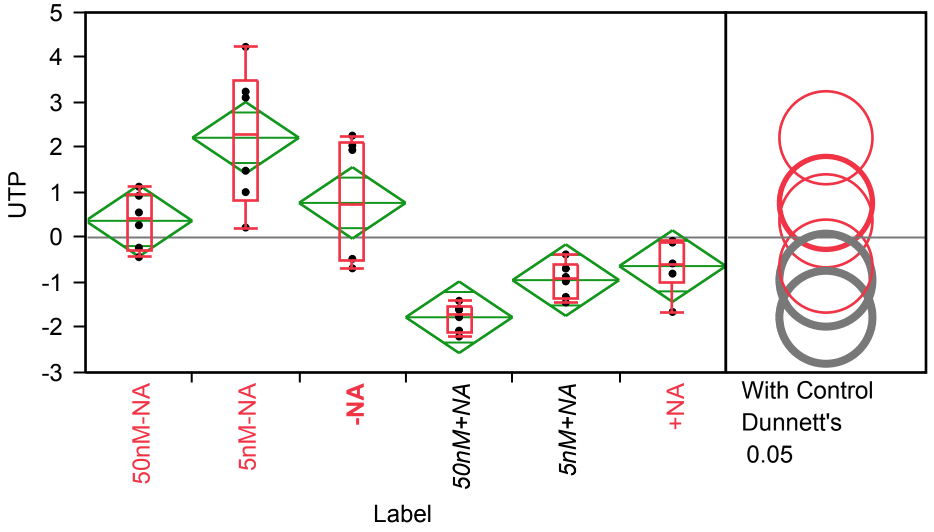


## Quantiles

| Level | Minimum | 10% | 25% | Median | 75% | 90% | Maximum |
|---|---|---|---|---|---|---|---|
| 50nM-NA | -0.43618 | -0.43618 | -0.27869 | 0.414739 | 0.977601 | 1.128711 | 1.128711 |
| 5nM-NA | 0.220942 | 0.220942 | 0.811522 | 2.296008 | 3.490053 | 4.235981 | 4.235981 |
| -NA | -0.68484 | -0.68484 | -0.53144 | 0.73489 | 2.099119 | 2.264122 | 2.264122 |
| 50nM+NA | -2.20113 | -2.20113 | -2.10255 | -1.68904 | -1.53685 | -1.40702 | -1.40702 |
| 5nM+NA | -1.44707 | -1.44707 | -1.35235 | -0.92587 | -0.61198 | -0.37579 | -0.37579 |
| +NA | -1.65396 | -1.65396 | -1.01567 | -0.58419 | -0.10322 | -0.07444 | -0.07444 |

## Oneway Anova

### Summary of Fit

| | |
|---|---|
| Rsquare | 0.689721 |
| Adj Rsquare | 0.638007 |
| Root Mean Square Error | 0.952573 |
| Mean of Response | 2.78e-10 |
| Observations (or Sum Wgts) | 36 |

### Analysis of Variance

| Source | DF | Sum of Squares | Mean Square | F Ratio | Prob > F |
|---|---|---|---|---|---|
| Label | 5 | 60.511535 | 12.1023 | 13.3374 | <.0001 * |
| Error | 30 | 27.221865 | 0.9074 | | |
| C. Total | 35 | 87.733400 | | | |

### Means for Oneway Anova

| Level | Number | Mean | Std Error | Lower 95% | Upper 95% |
|---|---|---|---|---|---|
| 50nM-NA | 6 | 0.3705 | 0.38889 | -0.424 | 1.165 |
| 5nM-NA | 6 | 2.2165 | 0.38889 | 1.422 | 3.011 |
| -NA | 6 | 0.7688 | 0.38889 | -0.025 | 1.563 |
| 50nM+NA | 6 | -1.7727 | 0.38889 | -2.567 | -0.978 |
| 5nM+NA | 6 | -0.9477 | 0.38889 | -1.742 | -0.153 |
| +NA | 6 | -0.6354 | 0.38889 | -1.430 | 0.159 |

Std Error uses a pooled estimate of error variance

## Means Comparisons

### Comparisons with a control using Dunnett's Method

## Oneway Analysis of Valine By Label

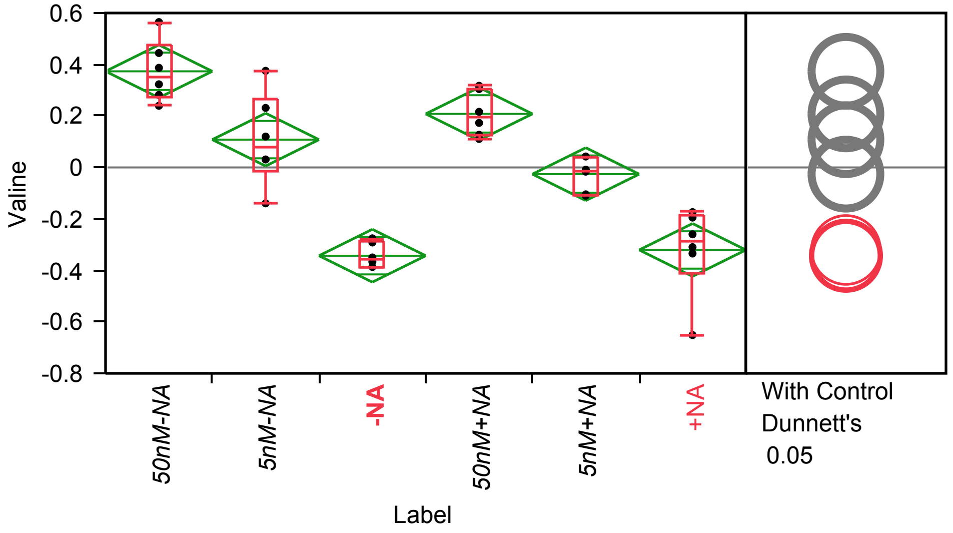


## Quantiles

| Level | Minimum | 10% | 25% | Median | 75% | 90% | Maximum |
|---|---|---|---|---|---|---|---|
| 50nM-NA | 0.239597 | 0.239597 | 0.271379 | 0.355 | 0.474639 | 0.564752 | 0.564752 |
| 5nM-NA | -0.13938 | -0.13938 | -0.01237 | 0.075466 | 0.267317 | 0.375406 | 0.375406 |
| -NA | -0.38787 | -0.38787 | -0.38695 | -0.35819 | -0.28748 | -0.27552 | -0.27552 |
| 50nM+NA | 0.110683 | 0.110683 | 0.122838 | 0.194546 | 0.307701 | 0.317169 | 0.317169 |
| 5nM+NA | -0.10971 | -0.10971 | -0.10587 | -0.01255 | 0.041242 | 0.042477 | 0.042477 |
| +NA | -0.65135 | -0.65135 | -0.41364 | -0.28472 | -0.18941 | -0.17367 | -0.17367 |

## Oneway Anova

### Summary of Fit

| | |
|---|---|
| Rsquare | 0.845779 |
| Adj Rsquare | 0.820076 |
| Root Mean Square Error | 0.123102 |
| Mean of Response | -8.3e-11 |
| Observations (or Sum Wgts) | 36 |

### Analysis of Variance

| Source | DF | Sum of Squares | Mean Square | F Ratio | Prob > F |
|---|---|---|---|---|---|
| Label | 5 | 2.4932621 | 0.498652 | 32.9052 | <.0001 * |
| Error | 30 | 0.4546257 | 0.015154 | | |
| C. Total | 35 | 2.9478879 | | | |

### Means for Oneway Anova

| Level | Number | Mean | Std Error | Lower 95% | Upper 95% |
|---|---|---|---|---|---|
| 50nM-NA | 6 | 0.37349 | 0.05026 | 0.2709 | 0.4761 |
| 5nM-NA | 6 | 0.10803 | 0.05026 | 0.0054 | 0.2107 |
| -NA | 6 | -0.34298 | 0.05026 | -0.4456 | -0.2403 |
| 50nM+NA | 6 | 0.20806 | 0.05026 | 0.1054 | 0.3107 |
| 5nM+NA | 6 | -0.02602 | 0.05026 | -0.1287 | 0.0766 |
| +NA | 6 | -0.32059 | 0.05026 | -0.4232 | -0.2179 |

Std Error uses a pooled estimate of error variance

## Means Comparisons

### Comparisons with a control using Dunnett's Method

## Oneway Analysis of xanthine By Label

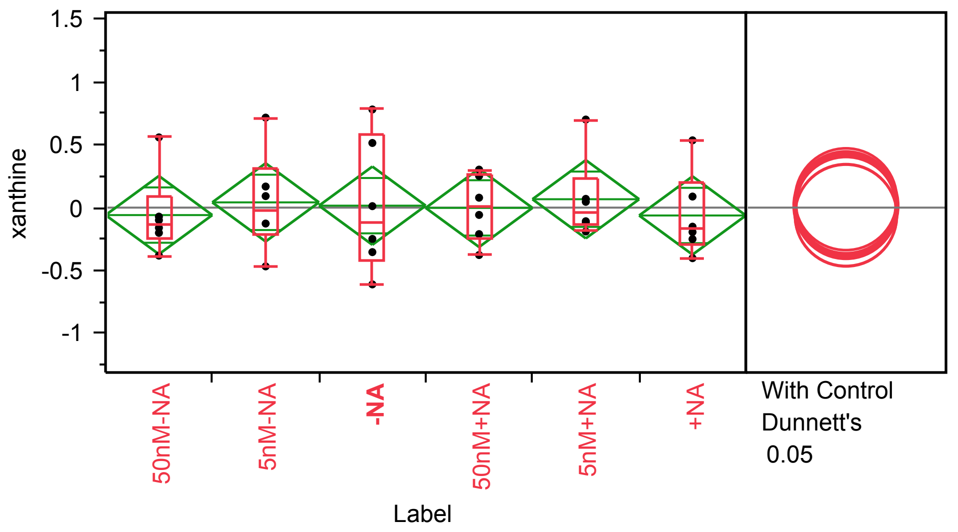


### Quantiles

| Level | Minimum | 10% | 25% | Median | 75% | 90% | Maximum |
|---|---|---|---|---|---|---|---|
| 50nM-NA | -0.3822 | -0.3822 | -0.24636 | -0.13037 | 0.086029 | 0.55936 | 0.55936 |
| 5nM-NA | -0.46799 | -0.46799 | -0.21514 | -0.01752 | 0.305169 | 0.716815 | 0.716815 |
| -NA | -0.61134 | -0.61134 | -0.41868 | -0.11879 | 0.581641 | 0.781686 | 0.781686 |
| 50nM+NA | -0.37757 | -0.37757 | -0.25267 | 0.010427 | 0.264693 | 0.302625 | 0.302625 |
| 5nM+NA | -0.19002 | -0.19002 | -0.13554 | -0.03129 | 0.227627 | 0.701405 | 0.701405 |
| +NA | -0.40256 | -0.40256 | -0.28864 | -0.17331 | 0.200069 | 0.535944 | 0.535944 |

### Oneway Anova

#### Summary of Fit

| | |
|---|---|
| Rsquare | 0.019535 |
| Adj Rsquare | -0.14388 |
| Root Mean Square Error | 0.373754 |
| Mean of Response | -5.6e-10 |
| Observations (or Sum Wgts) | 36 |

#### Analysis of Variance

| Source | DF | Sum of Squares | Mean Square | F Ratio | Prob > F |
|---|---|---|---|---|---|
| Label | 5 | 0.0834977 | 0.016700 | 0.1195 | 0.9870 |
| Error | 30 | 4.1907651 | 0.139692 | | |
| C. Total | 35 | 4.2742627 | | | |

#### Means for Oneway Anova

| Level | Number | Mean | Std Error | Lower 95% | Upper 95% |
|---|---|---|---|---|---|
| 50nM-NA | 6 | -0.05940 | 0.15258 | -0.3710 | 0.25222 |
| 5nM-NA | 6 | 0.04182 | 0.15258 | -0.2698 | 0.35343 |
| -NA | 6 | 0.01555 | 0.15258 | -0.2961 | 0.32716 |
| 50nM+NA | 6 | -0.00218 | 0.15258 | -0.3138 | 0.30944 |
| 5nM+NA | 6 | 0.06685 | 0.15258 | -0.2448 | 0.37847 |
| +NA | 6 | -0.06263 | 0.15258 | -0.3743 | 0.24898 |

Std Error uses a pooled estimate of error variance

### Means Comparisons

#### Comparisons with a control using Dunnett's Method

## Oneway Analysis of Xanthurenic acid By Label

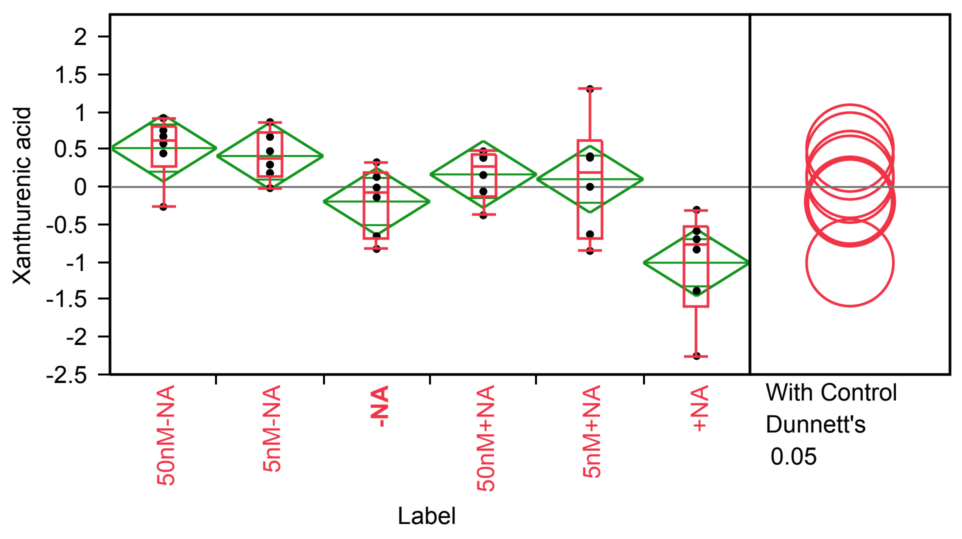


## Quantiles

| Level | Minimum | 10% | 25% | Median | 75% | 90% | Maximum |
|---|---|---|---|---|---|---|---|
| 50nM-NA | -0.26439 | -0.26439 | 0.269869 | 0.627561 | 0.795472 | 0.917001 | 0.917001 |
| 5nM-NA | -0.01473 | -0.01473 | 0.135834 | 0.387631 | 0.71772 | 0.865069 | 0.865069 |
| -NA | -0.82577 | -0.82577 | -0.69847 | -0.07252 | 0.182874 | 0.329697 | 0.329697 |
| 50nM+NA | -0.3769 | -0.3769 | -0.13896 | 0.273867 | 0.434442 | 0.475813 | 0.475813 |
| 5nM+NA | -0.8518 | -0.8518 | -0.68609 | 0.196553 | 0.63289 | 1.304845 | 1.304845 |
| +NA | -2.25296 | -2.25296 | -1.60144 | -0.76496 | -0.51686 | -0.30481 | -0.30481 |

## Oneway Anova

### Summary of Fit

| | |
|---|---|
| Rsquare | 0.518999 |
| Adj Rsquare | 0.438832 |
| Root Mean Square Error | 0.533617 |
| Mean of Response | -1.1e-10 |
| Observations (or Sum Wgts) | 36 |

### Analysis of Variance

| Source | DF | Sum of Squares | Mean Square | F Ratio | Prob > F |
|---|---|---|---|---|---|
| Label | 5 | 9.217244 | 1.84345 | 6.4740 | 0.0003 * |
| Error | 30 | 8.542415 | 0.28475 | | |
| C. Total | 35 | 17.759659 | | | |

### Means for Oneway Anova

| Level | Number | Mean | Std Error | Lower 95% | Upper 95% |
|---|---|---|---|---|---|
| 50nM-NA | 6 | 0.5184 | 0.21785 | 0.074 | 0.9633 |
| 5nM-NA | 6 | 0.4134 | 0.21785 | -0.032 | 0.8583 |
| -NA | 6 | -0.1939 | 0.21785 | -0.639 | 0.2510 |
| 50nM+NA | 6 | 0.1679 | 0.21785 | -0.277 | 0.6128 |
| 5nM+NA | 6 | 0.1040 | 0.21785 | -0.341 | 0.5489 |
| +NA | 6 | -1.0099 | 0.21785 | -1.455 | -0.5650 |

Std Error uses a pooled estimate of error variance

## Means Comparisons

### Comparisons with a control using Dunnett's Method

# Oneway Analysis of 1-(5Z,8Z,11Z,14Z-eicosatetraenoyl)-sn-glycero-3-phosphocholine By Labe

## Means Comparisons

### Comparisons with a control using Dunnett's Method

Control Group = -NA

| \|d\| | Alpha |
|---|---|
| 2.65691 | 0.05 |

| Level | Abs(Dif)-LSD | p-Value | |
|---|---|---|---|
| 50nM+NA | -0.01 | 0.0570 | |
| -NA | -0.42 | 1.0000 | |
| 5nM-NA | 0.265 | 0.0007 | * |
| 50nM-NA | 0.363 | 0.0001 | * |
| 5nM+NA | 0.595 | <.0001 | * |
| +NA | 0.803 | <.0001 | * |

Positive values show pairs of means that are significantly different.

## l Oneway Analysis of 1,3-diphopshateglycerate By Label

### Means Comparisons

#### Comparisons with a control using Dunnett's Method

Control Group = -NA

| \|d\| | Alpha |
|---|---|
| 2.65691 | 0.05 |

| Level | Abs(Dif)-LSD | p-Value | |
|---|---|---|---|
| -NA | -1.04 | 1.0000 | |
| 5nM-NA | -0.92 | 0.9984 | |
| 5nM+NA | -0.3 | 0.2384 | |
| +NA | -0.23 | 0.1683 | |
| 50nM-NA | -0.09 | 0.0841 | |
| 50nM+NA | 0.868 | 0.0002 | * |

Positive values show pairs of means that are significantly different.

# Oneway Analysis of 1-Aminocyclopropanecarboxylic acid By Label

## Means Comparisons

### Comparisons with a control using Dunnett's Method

Control Group = -NA

| \|d\| | Alpha |
|---|---|
| 2.65691 | 0.05 |

| Level | Abs(Dif)-LSD | p-Value | |
|---|---|---|---|
| 5nM-NA | 0.037 | 0.0036 | * |
| 50nM+NA | 0.013 | 0.0206 | * |
| 5nM+NA | -0.02 | 0.1980 | |
| +NA | -0.05 | 0.5714 | |
| -NA | -0.09 | 1.0000 | |
| 50nM-NA | -0.07 | 0.9645 | |

Positive values show pairs of means that are significantly different.

# Oneway Analysis of 1-Hexadecyl-2-arachidonoyl-glycerol By Label

## Means Comparisons

### Comparisons with a control using Dunnett's Method

Control Group = -NA

| \|d\| | Alpha |
|---|---|
| 2.65691 | 0.05 |

| Level | Abs(Dif)-LSD | p-Value | |
|---|---|---|---|
| 50nM+NA | 1.038 | 0.0006 | * |
| 50nM-NA | -0.07 | 0.0644 | |
| 5nM+NA | -1.4 | 0.9984 | |
| 5nM-NA | -1.56 | 1.0000 | |
| -NA | -1.57 | 1.0000 | |
| +NA | -1.02 | 0.8170 | |

Positive values show pairs of means that are significantly different.

# Oneway Analysis of 1-Methyladenosine By Label

## Means Comparisons

### Comparisons with a control using Dunnett's Method

Control Group = -NA

| \|d\| | Alpha |
|---|---|
| 2.65691 | 0.05 |

| Level | Abs(Dif)-LSD | p-Value |
|---|---|---|
| 5nM-NA | -0.56 | 0.6550 |
| 5nM+NA | -0.62 | 0.7678 |
| 50nM+NA | -0.85 | 0.9947 |
| -NA | -1 | 1.0000 |
| 50nM-NA | -0.93 | 0.9999 |
| +NA | -0.41 | 0.3959 |

Positive values show pairs of means that are significantly different.

# Oneway Analysis of 1-Methyl-Histidine By Label

## Means Comparisons

### Comparisons with a control using Dunnett's Method

Control Group = -NA

| \|d\| | Alpha |
|---|---|
| 2.65691 | 0.05 |

| Level | Abs(Dif)-LSD | p-Value | |
|---|---|---|---|
| 5nM-NA | -0.9 | 1.0000 | |
| -NA | -0.92 | 1.0000 | |
| 5nM+NA | -0.58 | 0.7825 | |
| +NA | -0.17 | 0.1374 | |
| 50nM-NA | -0.13 | 0.1081 | |
| 50nM+NA | 0.016 | 0.0451 | * |

Positive values show pairs of means that are significantly different.

# Oneway Analysis of 1-Octadecanoyl-sn-glycero-3-phosphocholine By Label

## Means Comparisons

### Comparisons with a control using Dunnett's Method

Control Group = -NA

| \|d\| | Alpha |
|---|---|
| 2.65691 | 0.05 |

| Level | Abs(Dif)-LSD | p-Value |
|---|---|---|
| 50nM+NA | -1.14 | 0.9996 |
| -NA | -1.24 | 1.0000 |
| 5nM-NA | 0.41 | 0.0059 * |
| 5nM+NA | 0.926 | 0.0003 * |
| 50nM-NA | 1.143 | <.0001 * |
| +NA | 2.964 | <.0001 * |

Positive values show pairs of means that are significantly different.

# Oneway Analysis of 1-oleoyl-2-hydroxy-sn-glycero-3-phosphocholine By Label

## Means Comparisons

### Comparisons with a control using Dunnett's Method

Control Group = -NA

| \|d\| | Alpha |
|---|---|
| 2.65691 | 0.05 |

| Level | Abs(Dif)-LSD | p-Value | |
|---|---|---|---|
| 50nM+NA | 1.335 | 0.0006 | * |
| -NA | -2.05 | 1.0000 | |
| 5nM-NA | 1.476 | 0.0004 | * |
| 5nM+NA | 1.502 | 0.0003 | * |
| 50nM-NA | 2.247 | <.0001 | * |
| +NA | 3.043 | <.0001 | * |

Positive values show pairs of means that are significantly different.

# Oneway Analysis of 2-Aminooctanoic acid By Label

## Means Comparisons

### Comparisons with a control using Dunnett's Method

Control Group = -NA

| \|d\| | Alpha |
|---|---|
| 2.65691 | 0.05 |

| Level | Abs(Dif)-LSD | p-Value |
|---|---|---|
| 5nM-NA | -0.58 | 0.8111 |
| -NA | -0.89 | 1.0000 |
| 50nM-NA | -0.86 | 1.0000 |
| 5nM+NA | -0.79 | 0.9984 |
| +NA | -0.63 | 0.8944 |
| 50nM+NA | -0.54 | 0.7399 |

Positive values show pairs of means that are significantly different.

# Oneway Analysis of 2-dehydro-D-gluconate By Label

## Means Comparisons

### Comparisons with a control using Dunnett's Method

Control Group = -NA

| \|d\| | Alpha |
|---|---|
| 2.65691 | 0.05 |

| Level | Abs(Dif)-LSD | p-Value |
|---|---|---|
| -NA | -1.31 | 1.0000 |
| 5nM+NA | -0.77 | 0.7158 |
| 50nM-NA | -0.66 | 0.5516 |
| +NA | -0.41 | 0.2627 |
| 5nM-NA | -0.25 | 0.1469 |
| 50nM+NA | -0.11 | 0.0817 |

Positive values show pairs of means that are significantly different.

# Oneway Analysis of 2-deoxyglucose-6-phosphate By Label

## Means Comparisons

### Comparisons with a control using Dunnett's Method

Control Group = -NA

| \|d\| | Alpha |
|---|---|
| 2.65691 | 0.05 |

| Level | Abs(Dif)-LSD | p-Value | |
|---|---|---|---|
| 5nM+NA | -0.33 | 0.2926 | |
| +NA | -0.92 | 0.9999 | |
| -NA | -0.99 | 1.0000 | |
| 5nM-NA | -0.96 | 1.0000 | |
| 50nM+NA | -0.39 | 0.3708 | |
| 50nM-NA | 0.002 | 0.0493 | * |

Positive values show pairs of means that are significantly different.

# Oneway Analysis of 2-Hydroxy-2-methylbutanedioic acid By Label

## Means Comparisons

### Comparisons with a control using Dunnett's Method

Control Group = -NA

| \|d\| | Alpha |
|---|---|
| 2.65691 | 0.05 |

| Level | Abs(Dif)-LSD | p-Value |
|---|---|---|
| +NA | -1.03 | 1.0000 |
| -NA | -1.04 | 1.0000 |
| 5nM-NA | 0.027 | 0.0428 * |
| 50nM+NA | 0.3 | 0.0078 * |
| 5nM+NA | 0.327 | 0.0065 * |
| 50nM-NA | 0.481 | 0.0023 * |

Positive values show pairs of means that are significantly different.

# Oneway Analysis of 2-hydroxyglutarate By Label

## Means Comparisons

### Comparisons with a control using Dunnett's Method

Control Group = -NA

| \|d\| | Alpha |
|---|---|
| 2.65691 | 0.05 |

| Level | Abs(Dif)-LSD | p-Value | |
|---|---|---|---|
| 50nM-NA | 0.157 | 0.0170 | * |
| 5nM-NA | 0.029 | 0.0412 | * |
| 5nM+NA | -0.07 | 0.0760 | |
| 50nM+NA | -0.37 | 0.3915 | |
| +NA | -0.68 | 0.9467 | |
| -NA | -0.9 | 1.0000 | |

Positive values show pairs of means that are significantly different.

# Oneway Analysis of 2-Isopropylmalic acid By Label

## Means Comparisons

### Comparisons with a control using Dunnett's Method

Control Group = -NA

| \|d\| | Alpha |
|---|---|
| 2.65691 | 0.05 |

| Level | Abs(Dif)-LSD | p-Value |
|---|---|---|
| 5nM-NA | -0.67 | 0.7332 |
| 50nM-NA | -0.7 | 0.7855 |
| -NA | -1.11 | 1.0000 |
| 5nM+NA | -0.87 | 0.9678 |
| 50nM+NA | -0.77 | 0.8743 |
| +NA | -0.69 | 0.7716 |

Positive values show pairs of means that are significantly different.

# Oneway Analysis of 2-ketoglutarate By Label

## Means Comparisons

### Comparisons with a control using Dunnett's Method

Control Group = -NA

| \|d\| | Alpha |
|---|---|
| 2.65691 | 0.05 |

| Level | Abs(Dif)-LSD | p-Value | |
|---|---|---|---|
| -NA | -0.67 | 1.0000 | |
| 5nM-NA | -0.6 | 0.9988 | |
| 50nM-NA | -0.54 | 0.9798 | |
| 50nM+NA | 0.187 | 0.0084 | * |
| 5nM+NA | 0.49 | 0.0003 | * |
| +NA | 1.045 | <.0001 | * |

Positive values show pairs of means that are significantly different.

# Oneway Analysis of 2-Oxo-4-hydroxy-5-aminovalerate By Label

## Means Comparisons

### Comparisons with a control using Dunnett's Method

Control Group = -NA

| \|d\| | Alpha |
|---|---|
| 2.65691 | 0.05 |

| Level | Abs(Dif)-LSD | p-Value | |
|---|---|---|---|
| -NA | -0.12 | 1.0000 | |
| 5nM+NA | -0.1 | 0.9934 | |
| 50nM+NA | -0.09 | 0.9588 | |
| 5nM-NA | 0.014 | 0.0247 | * |
| 50nM-NA | 0.109 | 0.0001 | * |
| +NA | 0.11 | <.0001 | * |

Positive values show pairs of means that are significantly different.

# Oneway Analysis of 2-oxobutanoate By Label

## Means Comparisons

### Comparisons with a control using Dunnett's Method

Control Group = -NA

| \|d\| | Alpha |
|---|---|
| 2.65691 | 0.05 |

| Level | Abs(Dif)-LSD | p-Value | |
|---|---|---|---|
| 50nM-NA | -0.33 | 0.4062 | |
| 5nM-NA | -0.49 | 0.7506 | |
| -NA | -0.8 | 1.0000 | |
| 50nM+NA | -0.43 | 0.6113 | |
| 5nM+NA | -0.08 | 0.0887 | |
| +NA | 0.034 | 0.0387 | * |

Positive values show pairs of means that are significantly different.

# Oneway Analysis of 3-phosphoglycerate By Label

## Means Comparisons

### Comparisons with a control using Dunnett's Method

Control Group = -NA

| \|d\| | Alpha |
|---|---|
| 2.65691 | 0.05 |

| Level | Abs(Dif)-LSD | p-Value |
|---|---|---|
| 5nM-NA | -0.38 | 0.3658 |
| -NA | -0.97 | 1.0000 |
| 50nM-NA | -0.84 | 0.9964 |
| 5nM+NA | -0.71 | 0.9211 |
| +NA | -0.13 | 0.1083 |
| 50nM+NA | -0.06 | 0.0708 |

Positive values show pairs of means that are significantly different.

# Oneway Analysis of 3-phospho-serine By Label

## Means Comparisons

### Comparisons with a control using Dunnett's Method

Control Group = -NA

| \|d\| | Alpha |
|---|---|
| 2.65691 | 0.05 |

| Level | Abs(Dif)-LSD | p-Value | |
|---|---|---|---|
| -NA | -0.91 | 1.0000 | |
| 5nM-NA | -0.52 | 0.6760 | |
| 50nM-NA | -0.28 | 0.2602 | |
| 5nM+NA | -0.09 | 0.0879 | |
| 50nM+NA | 0.051 | 0.0356 | * |
| +NA | 0.612 | 0.0005 | * |

Positive values show pairs of means that are significantly different.

# Oneway Analysis of 4-aminobutyrate By Label

## Means Comparisons

### Comparisons with a control using Dunnett's Method

Control Group = -NA

| \|d\| | Alpha |
|---|---|
| 2.65691 | 0.05 |

| Level | Abs(Dif)-LSD | p-Value |
|---|---|---|
| 5nM-NA | -0.45 | 0.9997 |
| -NA | -0.49 | 1.0000 |
| 50nM+NA | -0.3 | 0.7730 |
| +NA | -0.24 | 0.5320 |
| 50nM-NA | -0.18 | 0.3308 |
| 5nM+NA | -0.12 | 0.1920 |

Positive values show pairs of means that are significantly different.

# Oneway Analysis of 4-Pyridoxic acid By Label

## Means Comparisons

### Comparisons with a control using Dunnett's Method

Control Group = -NA

| \|d\| | Alpha |
|---|---|
| 2.65691 | 0.05 |

| Level | Abs(Dif)-LSD | p-Value |
|---|---|---|
| 5nM-NA | -1.12 | 0.9429 |
| -NA | -1.49 | 1.0000 |
| +NA | -1.1 | 0.9291 |
| 5nM+NA | -0.92 | 0.7634 |
| 50nM-NA | -0.85 | 0.6775 |
| 50nM+NA | -0.49 | 0.2808 |

Positive values show pairs of means that are significantly different.

# Oneway Analysis of 6-phospho-D-gluconate By Label

## Means Comparisons

### Comparisons with a control using Dunnett's Method

Control Group = -NA

| \|d\| | Alpha |
|---|---|
| 2.65691 | 0.05 |

| Level | Abs(Dif)-LSD | p-Value |
|---|---|---|
| 5nM-NA | -0.98 | 0.8835 |
| -NA | -1.41 | 1.0000 |
| 5nM+NA | -1.24 | 0.9973 |
| +NA | -0.71 | 0.5555 |
| 50nM+NA | -0.39 | 0.2215 |
| 50nM-NA | -0.11 | 0.0800 |

Positive values show pairs of means that are significantly different.

# Oneway Analysis of 6-phospho-D-glucono-1,5-lactone By Label

## Means Comparisons

### Comparisons with a control using Dunnett's Method

Control Group = -NA

| \|d\| | Alpha |
|---|---|
| 2.65691 | 0.05 |

| Level | Abs(Dif)-LSD | p-Value |
|---|---|---|
| 5nM+NA | -1.39 | 0.9380 |
| 5nM-NA | -1.74 | 0.9998 |
| -NA | -1.87 | 1.0000 |
| +NA | -1.18 | 0.7862 |
| 50nM+NA | -0.66 | 0.3134 |
| 50nM-NA | -0.29 | 0.1185 |

Positive values show pairs of means that are significantly different.

# Oneway Analysis of 7-methylguanosine By Label

## Means Comparisons

### Comparisons with a control using Dunnett's Method

Control Group = -NA

| \|d\| | Alpha |
|---|---|
| 2.65691 | 0.05 |

| Level | Abs(Dif)-LSD | p-Value |
|---|---|---|
| 5nM+NA | -1.04 | 1.0000 |
| +NA | -1.04 | 1.0000 |
| -NA | -1.07 | 1.0000 |
| 5nM-NA | -0.67 | 0.7728 |
| 50nM-NA | -0.08 | 0.0751 |
| 50nM+NA | 0.129 | 0.0239 * |

Positive values show pairs of means that are significantly different.

# Oneway Analysis of acetoacetate By Label

## Means Comparisons

### Comparisons with a control using Dunnett's Method

Control Group = -NA

| \|d\| | Alpha |
|---|---|
| 2.65691 | 0.05 |

| Level | Abs(Dif)-LSD | p-Value |
|---|---|---|
| 5nM-NA | -0.25 | 0.2201 |
| 50nM-NA | -0.63 | 0.8806 |
| -NA | -0.91 | 1.0000 |
| 50nM+NA | -0.55 | 0.7414 |
| 5nM+NA | -0.32 | 0.3159 |
| +NA | -0.05 | 0.0711 |

Positive values show pairs of means that are significantly different.

# Oneway Analysis of Acetylcarnitine By Label

## Means Comparisons

### Comparisons with a control using Dunnett's Method

Control Group = -NA

| \|d\| | Alpha |
|---|---|
| 2.65691 | 0.05 |

| Level | Abs(Dif)-LSD | p-Value | |
|---|---|---|---|
| 50nM+NA | 0.125 | 0.0117 | * |
| 50nM-NA | -0.24 | 0.4589 | |
| -NA | -0.54 | 1.0000 | |
| 5nM+NA | -0.5 | 0.9997 | |
| 5nM-NA | -0.34 | 0.7604 | |
| +NA | -0.12 | 0.1743 | |

Positive values show pairs of means that are significantly different.

# Oneway Analysis of acetyl-CoA By Label

## Means Comparisons

### Comparisons with a control using Dunnett's Method

Control Group = -NA

| \|d\| | Alpha |
|---|---|
| 2.65691 | 0.05 |

| Level | Abs(Dif)-LSD | p-Value | |
|---|---|---|---|
| -NA | -0.9 | 1.0000 | |
| 50nM-NA | 0.072 | 0.0306 | * |
| 50nM+NA | 0.17 | 0.0153 | * |
| +NA | 0.217 | 0.0108 | * |
| 5nM-NA | 0.248 | 0.0086 | * |
| 5nM+NA | 0.675 | 0.0003 | * |

Positive values show pairs of means that are significantly different.

# Oneway Analysis of Acetyllysine By Label

## Means Comparisons

### Comparisons with a control using Dunnett's Method

Control Group = -NA

| \|d\| | Alpha |
|---|---|
| 2.65691 | 0.05 |

| Level | Abs(Dif)-LSD | p-Value | |
|---|---|---|---|
| 5nM+NA | 0.724 | <.0001 | * |
| 5nM-NA | 0.676 | <.0001 | * |
| 50nM-NA | 0.174 | 0.0064 | * |
| 50nM+NA | -0.01 | 0.0559 | |
| -NA | -0.55 | 1.0000 | |
| +NA | -0.34 | 0.7660 | |

Positive values show pairs of means that are significantly different.

# Oneway Analysis of aconitate By Label

## Means Comparisons

### Comparisons with a control using Dunnett's Method

Control Group = -NA

| \|d\| | Alpha |
|---|---|
| 2.65691 | 0.05 |

| Level | Abs(Dif)-LSD | p-Value | |
|---|---|---|---|
| -NA | -0.9 | 1.0000 | |
| 5nM+NA | -0.06 | 0.0739 | |
| +NA | 0.065 | 0.0323 | * |
| 5nM-NA | 1.13 | <.0001 | * |
| 50nM-NA | 1.282 | <.0001 | * |
| 50nM+NA | 1.387 | <.0001 | * |

Positive values show pairs of means that are significantly different.

# Oneway Analysis of Adenine By Label

## Means Comparisons

### Comparisons with a control using Dunnett's Method

Control Group = -NA

| \|d\| | Alpha |
|---|---|
| 2.65691 | 0.05 |

| Level | Abs(Dif)-LSD | p-Value | |
|---|---|---|---|
| 50nM+NA | 3.571 | <.0001 | * |
| 50nM-NA | 2.544 | <.0001 | * |
| 5nM-NA | 0.732 | 0.0009 | * |
| 5nM+NA | -0.09 | 0.0749 | |
| -NA | -1.24 | 1.0000 | |
| +NA | -0.55 | 0.4566 | |

Positive values show pairs of means that are significantly different.

# Oneway Analysis of adenosine By Label

## Means Comparisons

### Comparisons with a control using Dunnett's Method

Control Group = -NA

| \|d\| | Alpha |
|---|---|
| 2.65691 | 0.05 |

| Level | Abs(Dif)-LSD | p-Value | |
|---|---|---|---|
| 50nM+NA | 0.839 | <.0001 | * |
| 50nM-NA | 0.671 | <.0001 | * |
| 5nM-NA | 0.428 | 0.0005 | * |
| 5nM+NA | 0.168 | 0.0090 | * |
| +NA | -0.45 | 0.9244 | |
| -NA | -0.62 | 1.0000 | |

Positive values show pairs of means that are significantly different.

# Oneway Analysis of Adipic acid By Label

## Means Comparisons

### Comparisons with a control using Dunnett's Method

Control Group = -NA

| \|d\| | Alpha |
|---|---|
| 2.65691 | 0.05 |

| Level | Abs(Dif)-LSD | p-Value | |
|---|---|---|---|
| 50nM-NA | 0.446 | 0.0012 | * |
| +NA | -0.25 | 0.2709 | |
| 50nM+NA | -0.53 | 0.8498 | |
| 5nM+NA | -0.56 | 0.8882 | |
| 5nM-NA | -0.73 | 0.9997 | |
| -NA | -0.8 | 1.0000 | |

Positive values show pairs of means that are significantly different.

# Oneway Analysis of ADP By Label

## Means Comparisons

### Comparisons with a control using Dunnett's Method

Control Group = -NA

| \|d\| | Alpha |
|---|---|
| 2.65691 | 0.05 |

| Level | Abs(Dif)-LSD | p-Value | |
|---|---|---|---|
| 50nM+NA | 1.311 | <.0001 | * |
| 50nM-NA | 0.799 | <.0001 | * |
| 5nM+NA | 0.557 | 0.0003 | * |
| 5nM-NA | 0.067 | 0.0284 | * |
| +NA | -0.61 | 0.9904 | |
| -NA | -0.73 | 1.0000 | |

Positive values show pairs of means that are significantly different.

# Oneway Analysis of AICAR 3,5-Cyclic Phosphate By Label

## Means Comparisons

### Comparisons with a control using Dunnett's Method

Control Group = -NA

| \|d\| | Alpha |
|---|---|
| 2.65691 | 0.05 |

| Level | Abs(Dif)-LSD | p-Value | |
|---|---|---|---|
| 50nM+NA | 1.912 | <.0001 | * |
| 50nM-NA | 1.034 | <.0001 | * |
| 5nM+NA | 0.417 | <.0001 | * |
| 5nM-NA | 0.139 | 0.0038 | * |
| -NA | -0.35 | 1.0000 | |
| +NA | -0.34 | 1.0000 | |

Positive values show pairs of means that are significantly different.

# Oneway Analysis of Ala-Ala By Label

## Means Comparisons

### Comparisons with a control using Dunnett's Method

Control Group = -NA

| \|d\| | Alpha |
|---|---|
| 2.65691 | 0.05 |

| Level | Abs(Dif)-LSD | p-Value | |
|---|---|---|---|
| 50nM-NA | 0.229 | 0.0026 | * |
| 5nM+NA | -0.25 | 0.5120 | |
| 50nM+NA | -0.38 | 0.9266 | |
| 5nM-NA | -0.42 | 0.9872 | |
| -NA | -0.51 | 1.0000 | |
| +NA | -0.51 | 1.0000 | |

Positive values show pairs of means that are significantly different.

# Oneway Analysis of Ala-Gly By Label

## Means Comparisons

### Comparisons with a control using Dunnett's Method

Control Group = -NA

| \|d\| | Alpha |
|---|---|
| 2.65691 | 0.05 |

| Level | Abs(Dif)-LSD | p-Value |
|---|---|---|
| 5nM+NA | -0.49 | 0.2179 |
| 50nM-NA | -1.71 | 1.0000 |
| 50nM+NA | -1.72 | 1.0000 |
| 5nM-NA | -1.73 | 1.0000 |
| -NA | -1.79 | 1.0000 |
| +NA | -1.65 | 0.9997 |

Positive values show pairs of means that are significantly different.

# Oneway Analysis of alanine By Label

## Means Comparisons

### Comparisons with a control using Dunnett's Method

Control Group = -NA

| \|d\| | Alpha |
|---|---|
| 2.65691 | 0.05 |

| Level | Abs(Dif)-LSD | p-Value | |
|---|---|---|---|
| 50nM-NA | 1.141 | <.0001 | * |
| 50nM+NA | 1.05 | <.0001 | * |
| 5nM-NA | 0.833 | <.0001 | * |
| 5nM+NA | 0.776 | <.0001 | * |
| -NA | -0.56 | 1.0000 | |
| +NA | -0.48 | 0.9943 | |

Positive values show pairs of means that are significantly different.

# Oneway Analysis of allantoate By Label

## Means Comparisons

### Comparisons with a control using Dunnett's Method

Control Group = -NA

| \|d\| | Alpha |
|---|---|
| 2.65691 | 0.05 |

| Level | Abs(Dif)-LSD | p-Value | |
|---|---|---|---|
| -NA | -0.8 | 1.0000 | |
| +NA | 1.913 | <.0001 | * |
| 5nM-NA | 3.46 | <.0001 | * |
| 5nM+NA | 3.991 | <.0001 | * |
| 50nM-NA | 4.224 | <.0001 | * |
| 50nM+NA | 4.492 | <.0001 | * |

Positive values show pairs of means that are significantly different.

# Oneway Analysis of Aminoadipic acid By Label

## Means Comparisons

### Comparisons with a control using Dunnett's Method

Control Group = -NA

| \|d\| | Alpha |
|---|---|
| 2.65691 | 0.05 |

| Level | Abs(Dif)-LSD | p-Value | |
|---|---|---|---|
| -NA | -1.03 | 1.0000 | |
| +NA | -0.14 | 0.1084 | |
| 50nM-NA | 0.127 | 0.0234 | * |
| 5nM-NA | 0.328 | 0.0064 | * |
| 50nM+NA | 0.944 | <.0001 | * |
| 5nM+NA | 1.015 | <.0001 | * |

Positive values show pairs of means that are significantly different.

# Oneway Analysis of AICAR(ZMP) By Label

## Means Comparisons

### Comparisons with a control using Dunnett's Method

Control Group = -NA

| \|d\| | Alpha |
|---|---|
| 2.65691 | 0.05 |

| Level | Abs(Dif)-LSD | p-Value | |
|---|---|---|---|
| +NA | -0.43 | 0.4459 | |
| -NA | -0.98 | 1.0000 | |
| 5nM-NA | 3.136 | <.0001 | * |
| 5nM+NA | 3.765 | <.0001 | * |
| 50nM-NA | 4.316 | <.0001 | * |
| 50nM+NA | 4.443 | <.0001 | * |

Positive values show pairs of means that are significantly different.

# Oneway Analysis of Aminomalonic acid By Label

## Means Comparisons

### Comparisons with a control using Dunnett's Method

Control Group = -NA

| \|d\| | Alpha |
|---|---|
| 2.65691 | 0.05 |

| Level | Abs(Dif)-LSD | p-Value | |
|---|---|---|---|
| +NA | 2.383 | 0.0006 | * |
| 5nM+NA | 1.926 | 0.0014 | * |
| 50nM+NA | -0.41 | 0.0959 | |
| 50nM-NA | -2.29 | 0.7994 | |
| 5nM-NA | -3.09 | 0.9950 | |
| -NA | -3.59 | 1.0000 | |

Positive values show pairs of means that are significantly different.

# Oneway Analysis of AMP By Label

## Means Comparisons

### Comparisons with a control using Dunnett's Method

Control Group = -NA

| \|d\| | Alpha |
|---|---|
| 2.65691 | 0.05 |

| Level | Abs(Dif)-LSD | p-Value |
|---|---|---|
| 50nM+NA | 2.488 | <.0001 * |
| 50nM-NA | 2.142 | <.0001 * |
| 5nM-NA | 1.31 | <.0001 * |
| 5nM+NA | 0.696 | <.0001 * |
| -NA | -0.45 | 1.0000 |
| +NA | -0.37 | 0.9861 |

Positive values show pairs of means that are significantly different.

# Oneway Analysis of Anhydrosorbitol By Label

## Means Comparisons

### Comparisons with a control using Dunnett's Method

Control Group = -NA

| \|d\| | Alpha |
|---|---|
| 2.65691 | 0.05 |

| Level | Abs(Dif)-LSD | p-Value |
|---|---|---|
| 5nM+NA | -2.2 | 0.3609 |
| +NA | -5.35 | 0.9999 |
| 5nM-NA | -5.43 | 1.0000 |
| -NA | -5.69 | 1.0000 |
| 50nM-NA | -3.88 | 0.8640 |
| 50nM+NA | -3.24 | 0.6766 |

Positive values show pairs of means that are significantly different.

# Oneway Analysis of Arabitol By Label

## Means Comparisons

### Comparisons with a control using Dunnett's Method

Control Group = -NA

| \|d\| | Alpha |
|---|---|
| 2.65691 | 0.05 |

| Level | Abs(Dif)-LSD | p-Value |
|---|---|---|
| 50nM-NA | -0.94 | 0.2168 |
| 5nM-NA | -3.32 | 1.0000 |
| -NA | -3.45 | 1.0000 |
| 50nM+NA | -2.16 | 0.7787 |
| 5nM+NA | -1.91 | 0.6495 |
| +NA | -0.27 | 0.0792 |

Positive values show pairs of means that are significantly different.

# Oneway Analysis of Arginine By Label

## Means Comparisons

### Comparisons with a control using Dunnett's Method

Control Group = -NA

| \|d\| | Alpha |
|---|---|
| 2.65691 | 0.05 |

| Level | Abs(Dif)-LSD | p-Value | |
|---|---|---|---|
| 5nM+NA | 4.53 | <.0001 | * |
| 50nM+NA | 4.403 | <.0001 | * |
| +NA | -0.24 | 0.0970 | |
| 5nM-NA | -0.43 | 0.1573 | |
| 50nM-NA | -1.1 | 0.6057 | |
| -NA | -2.06 | 1.0000 | |

Positive values show pairs of means that are significantly different.

# Oneway Analysis of Asparagine By Label

## Means Comparisons

### Comparisons with a control using Dunnett's Method

Control Group = -NA

| \|d\| | Alpha |
|---|---|
| 2.65691 | 0.05 |

| Level | Abs(Dif)-LSD | p-Value | |
|---|---|---|---|
| 5nM+NA | 1.447 | 0.0003 | * |
| 50nM+NA | 0.916 | 0.0020 | * |
| 50nM-NA | -0.29 | 0.1182 | |
| 5nM-NA | -1.27 | 0.8552 | |
| +NA | -1.7 | 0.9992 | |
| -NA | -1.89 | 1.0000 | |

Positive values show pairs of means that are significantly different.

# Oneway Analysis of aspartate By Label

## Means Comparisons

### Comparisons with a control using Dunnett's Method

Control Group = -NA

| \|d\| | Alpha |
|---|---|
| 2.65691 | 0.05 |

| Level | Abs(Dif)-LSD | p-Value | |
|---|---|---|---|
| 50nM+NA | 3.328 | <.0001 | * |
| 50nM-NA | 3.219 | <.0001 | * |
| 5nM+NA | 2.43 | <.0001 | * |
| 5nM-NA | 2.394 | <.0001 | * |
| -NA | -0.44 | 1.0000 | |
| +NA | -0.39 | 0.9977 | |

Positive values show pairs of means that are significantly different.

# Oneway Analysis of ATP By Label

## Means Comparisons

### Comparisons with a control using Dunnett's Method

Control Group = -NA

| \|d\| | Alpha |
|---|---|
| 2.65691 | 0.05 |

| Level | Abs(Dif)-LSD | p-Value | |
|---|---|---|---|
| 5nM-NA | 1.398 | 0.0001 | * |
| 50nM-NA | -0.44 | 0.2109 | |
| -NA | -1.64 | 1.0000 | |
| 5nM+NA | 0.542 | 0.0059 | * |
| +NA | 0.761 | 0.0023 | * |
| 50nM+NA | 0.797 | 0.0020 | * |

Positive values show pairs of means that are significantly different.

# Oneway Analysis of Atrolactic acid By Label

## Means Comparisons

### Comparisons with a control using Dunnett's Method

Control Group = -NA

| \|d\| | Alpha |
|---|---|
| 2.65691 | 0.05 |

| Level | Abs(Dif)-LSD | p-Value | |
|---|---|---|---|
| -NA | -1 | 1.0000 | |
| +NA | 0.279 | 0.0084 | * |
| 5nM+NA | 1.694 | <.0001 | * |
| 5nM-NA | 1.725 | <.0001 | * |
| 50nM-NA | 2.113 | <.0001 | * |
| 50nM+NA | 2.282 | <.0001 | * |

Positive values show pairs of means that are significantly different.

# Oneway Analysis of Benzoic acid By Label

## Means Comparisons

### Comparisons with a control using Dunnett's Method

Control Group = -NA

| \|d\| | Alpha |
|---|---|
| 2.65691 | 0.05 |

| Level | Abs(Dif)-LSD | p-Value |
|---|---|---|
| 50nM-NA | -1.24 | 0.6791 |
| 5nM-NA | -1.58 | 0.9186 |
| 5nM+NA | -1.58 | 0.9206 |
| 50nM+NA | -1.62 | 0.9379 |
| +NA | -1.97 | 0.9991 |
| -NA | -2.18 | 1.0000 |

Positive values show pairs of means that are significantly different.

# Oneway Analysis of beta-Alanine By Label

## Means Comparisons

### Comparisons with a control using Dunnett's Method

Control Group = -NA

| \|d\| | Alpha |
|---|---|
| 2.65691 | 0.05 |

| Level | Abs(Dif)-LSD | p-Value |
|---|---|---|
| +NA | -1.89 | 0.9787 |
| -NA | -2.34 | 1.0000 |
| 50nM-NA | -1.96 | 0.9900 |
| 5nM-NA | -0.67 | 0.2310 |
| 5nM+NA | -0.67 | 0.2310 |
| 50nM+NA | -0.67 | 0.2310 |

Positive values show pairs of means that are significantly different.

# Oneway Analysis of beta-D-Glucopyranose By Label

## Means Comparisons

### Comparisons with a control using Dunnett's Method

Control Group = -NA

| \|d\| | Alpha |
|---|---|
| 2.65691 | 0.05 |

| Level | Abs(Dif)-LSD | p-Value |
|---|---|---|
| 5nM-NA | -0.78 | 0.9986 |
| 5nM+NA | -0.8 | 0.9996 |
| -NA | -0.87 | 1.0000 |
| 50nM-NA | -0.86 | 1.0000 |
| 50nM+NA | -0.64 | 0.9257 |
| +NA | -0.37 | 0.4144 |

Positive values show pairs of means that are significantly different.

# Oneway Analysis of betaine By Label

## Means Comparisons

### Comparisons with a control using Dunnett's Method

Control Group = -NA

| \|d\| | Alpha |
|---|---|
| 2.65691 | 0.05 |

| Level | Abs(Dif)-LSD | p-Value | |
|---|---|---|---|
| 5nM-NA | 0.246 | 0.0031 | * |
| 50nM-NA | -0.37 | 0.8062 | |
| -NA | -0.58 | 1.0000 | |
| 5nM+NA | -0.14 | 0.1934 | |
| 50nM+NA | 0.27 | 0.0023 | * |
| +NA | 0.571 | <.0001 | * |

Positive values show pairs of means that are significantly different.

# Oneway Analysis of betaine aldehyde By Label

## Means Comparisons

### Comparisons with a control using Dunnett's Method

Control Group = -NA

| \|d\| | Alpha |
|---|---|
| 2.65691 | 0.05 |

| Level | Abs(Dif)-LSD | p-Value |
|---|---|---|
| 5nM-NA | -0.59 | 0.5840 |
| 5nM+NA | -0.87 | 0.9599 |
| 50nM-NA | -0.98 | 0.9956 |
| -NA | -1.13 | 1.0000 |
| +NA | -0.68 | 0.7319 |
| 50nM+NA | -0.43 | 0.3513 |

Positive values show pairs of means that are significantly different.

# Oneway Analysis of Butyrylcarnitine By Label

## Means Comparisons

### Comparisons with a control using Dunnett's Method

Control Group = -NA

| \|d\| | Alpha |
|---|---|
| 2.65691 | 0.05 |

| Level | Abs(Dif)-LSD | p-Value |
|---|---|---|
| -NA | -0.57 | 1.0000 |
| +NA | 1.42 | <.0001 * |
| 5nM-NA | 2.529 | <.0001 * |
| 5nM+NA | 3.523 | <.0001 * |
| 50nM-NA | 4.022 | <.0001 * |
| 50nM+NA | 4.24 | <.0001 * |

Positive values show pairs of means that are significantly different.

# Oneway Analysis of Carbamoyl phosphate By Label

## Means Comparisons

### Comparisons with a control using Dunnett's Method

Control Group = -NA

| \|d\| | Alpha |
|---|---|
| 2.65691 | 0.05 |

| Level | Abs(Dif)-LSD | p-Value | |
|---|---|---|---|
| 50nM-NA | 0.119 | 0.0135 | * |
| 50nM+NA | -0.07 | 0.1004 | |
| 5nM+NA | -0.29 | 0.5664 | |
| 5nM-NA | -0.55 | 1.0000 | |
| -NA | -0.57 | 1.0000 | |
| +NA | -0.03 | 0.0705 | |

Positive values show pairs of means that are significantly different.

# Oneway Analysis of carnitine By Label

## Means Comparisons

### Comparisons with a control using Dunnett's Method

Control Group = -NA

| \|d\| | Alpha |
|---|---|
| 2.65691 | 0.05 |

| Level | Abs(Dif)-LSD | p-Value |
|---|---|---|
| 5nM-NA | 1.552 | <.0001 * |
| 50nM-NA | 1.365 | <.0001 * |
| 5nM+NA | 1.168 | <.0001 * |
| 50nM+NA | 0.872 | <.0001 * |
| +NA | -0.38 | 1.0000 |
| -NA | -0.39 | 1.0000 |

Positive values show pairs of means that are significantly different.

# Oneway Analysis of CDP By Label

## Means Comparisons

### Comparisons with a control using Dunnett's Method

Control Group = -NA

| \|d\| | Alpha |
|---|---|
| 2.65691 | 0.05 |

| Level | Abs(Dif)-LSD | p-Value |
|---|---|---|
| 50nM-NA | -0.13 | 0.1036 |
| 5nM-NA | -0.3 | 0.2339 |
| 50nM+NA | -0.33 | 0.2619 |
| 5nM+NA | -0.72 | 0.8727 |
| -NA | -1.05 | 1.0000 |
| +NA | -0.86 | 0.9851 |

Positive values show pairs of means that are significantly different.

# Oneway Analysis of Cholesterol By Label

## Means Comparisons

### Comparisons with a control using Dunnett's Method

Control Group = -NA

| \|d\| | Alpha |
|---|---|
| 2.65691 | 0.05 |

| Level | Abs(Dif)-LSD | p-Value | |
|---|---|---|---|
| 50nM+NA | 1.043 | 0.0026 | * |
| +NA | -1 | 0.4288 | |
| 5nM+NA | -1.5 | 0.8075 | |
| 50nM-NA | -2.03 | 0.9969 | |
| -NA | -2.32 | 1.0000 | |
| 5nM-NA | -2.13 | 0.9996 | |

Positive values show pairs of means that are significantly different.

# Oneway Analysis of choline By Label

## Means Comparisons

### Comparisons with a control using Dunnett's Method

Control Group = -NA

| \|d\| | Alpha |
|---|---|
| 2.65691 | 0.05 |

| Level | Abs(Dif)-LSD | p-Value |
|---|---|---|
| 50nM-NA | -0.29 | 0.3843 |
| 50nM+NA | -0.33 | 0.4679 |
| 5nM-NA | -0.6 | 0.9858 |
| 5nM+NA | -0.7 | 1.0000 |
| -NA | -0.73 | 1.0000 |
| +NA | -0.27 | 0.3356 |

Positive values show pairs of means that are significantly different.

# Oneway Analysis of Chondroitin By Label

## Means Comparisons

### Comparisons with a control using Dunnett's Method

Control Group = -NA

| \|d\| | Alpha |
|---|---|
| 2.65691 | 0.05 |

| Level | Abs(Dif)-LSD | p-Value |
|---|---|---|
| 5nM+NA | -1.2 | 0.9905 |
| 50nM+NA | -1.35 | 0.9999 |
| -NA | -1.43 | 1.0000 |
| 5nM-NA | -0.68 | 0.5091 |
| 50nM-NA | -0.44 | 0.2592 |
| +NA | 0.157 | 0.0255 * |

Positive values show pairs of means that are significantly different.

# Oneway Analysis of Citric acid By Label

## Means Comparisons

### Comparisons with a control using Dunnett's Method

Control Group = -NA

| \|d\| | Alpha |
|---|---|
| 2.65691 | 0.05 |

| Level | Abs(Dif)-LSD | p-Value | |
|---|---|---|---|
| +NA | 0.348 | 0.0273 | * |
| 5nM+NA | -3.47 | 1.0000 | |
| -NA | -3.52 | 1.0000 | |
| 50nM+NA | -2.99 | 0.9932 | |
| 50nM-NA | -2.33 | 0.8370 | |
| 5nM-NA | 1.411 | 0.0036 | * |

Positive values show pairs of means that are significantly different.

# Oneway Analysis of citrulline By Label

## Means Comparisons

### Comparisons with a control using Dunnett's Method

Control Group = -NA

| \|d\| | Alpha |
|---|---|
| 2.65691 | 0.05 |

| Level | Abs(Dif)-LSD | p-Value | |
|---|---|---|---|
| +NA | 0.489 | 0.0003 | * |
| 5nM+NA | -0.02 | 0.0616 | |
| -NA | -0.65 | 1.0000 | |
| 5nM-NA | -0.46 | 0.9088 | |
| 50nM+NA | -0.14 | 0.1689 | |
| 50nM-NA | 0.423 | 0.0006 | * |

Positive values show pairs of means that are significantly different.

# Oneway Analysis of CMP By Label

## Means Comparisons

### Comparisons with a control using Dunnett's Method

Control Group = -NA

| \|d\| | Alpha |
|---|---|
| 2.65691 | 0.05 |

| Level | Abs(Dif)-LSD | p-Value | |
|---|---|---|---|
| 5nM-NA | -0.47 | 0.5855 | |
| 5nM+NA | -0.73 | 0.9792 | |
| -NA | -0.91 | 1.0000 | |
| +NA | -0.69 | 0.9520 | |
| 50nM-NA | 0.173 | 0.0152 | * |
| 50nM+NA | 0.409 | 0.0026 | * |

Positive values show pairs of means that are significantly different.

# Oneway Analysis of CMP-N-acetylneuraminic acid By Label

## Means Comparisons

### Comparisons with a control using Dunnett's Method

Control Group = -NA

| \|d\| | Alpha |
|---|---|
| 2.65691 | 0.05 |

| Level | Abs(Dif)-LSD | p-Value |
|---|---|---|
| +NA | 0.678 | 0.0015 * |
| -NA | -1.29 | 1.0000 |
| 50nM+NA | -0.9 | 0.8928 |
| 5nM+NA | -0.75 | 0.6977 |
| 5nM-NA | -0.02 | 0.0554 |
| 50nM-NA | 0.172 | 0.0219 * |

Positive values show pairs of means that are significantly different.

# Oneway Analysis of creatine By Label

## Means Comparisons

### Comparisons with a control using Dunnett's Method

Control Group = -NA

| \|d\| | Alpha |
|---|---|
| 2.65691 | 0.05 |

| Level | Abs(Dif)-LSD | p-Value | |
|---|---|---|---|
| 5nM-NA | 0.492 | 0.0004 | * |
| 5nM+NA | 0.256 | 0.0047 | * |
| 50nM-NA | 0.098 | 0.0211 | * |
| 50nM+NA | 0.017 | 0.0432 | * |
| -NA | -0.7 | 1.0000 | |
| +NA | -0.51 | 0.9216 | |

Positive values show pairs of means that are significantly different.

# Oneway Analysis of Creatinine By Label

## Means Comparisons

### Comparisons with a control using Dunnett's Method

Control Group = -NA

| \|d\| | Alpha |
|---|---|
| 2.65691 | 0.05 |

| Level | Abs(Dif)-LSD | p-Value |
|---|---|---|
| 50nM+NA | 1.843 | <.0001 * |
| 50nM-NA | 1.538 | <.0001 * |
| 5nM+NA | 0.932 | <.0001 * |
| 5nM-NA | 0.258 | 0.0011 * |
| +NA | 0.03 | 0.0335 * |
| -NA | -0.46 | 1.0000 |

Positive values show pairs of means that are significantly different.

# Oneway Analysis of Cyclic ADP-ribose By Label

## Means Comparisons

### Comparisons with a control using Dunnett's Method

Control Group = -NA

| \|d\| | Alpha |
|---|---|
| 2.65691 | 0.05 |

| Level | Abs(Dif)-LSD | p-Value |
|---|---|---|
| +NA | -0.48 | 0.6942 |
| -NA | -0.82 | 1.0000 |
| 5nM+NA | 4.653 | <.0001 * |
| 50nM+NA | 4.672 | <.0001 * |
| 5nM-NA | 4.805 | <.0001 * |
| 50nM-NA | 4.87 | <.0001 * |

Positive values show pairs of means that are significantly different.

# Oneway Analysis of cyclic-AMP By Label

## Means Comparisons

### Comparisons with a control using Dunnett's Method

Control Group = -NA

| \|d\| | Alpha |
|---|---|
| 2.65691 | 0.05 |

| Level | Abs(Dif)-LSD | p-Value |
|---|---|---|
| -NA | -0.96 | 1.0000 |
| +NA | -0.69 | 0.9192 |
| 5nM-NA | -0.02 | 0.0570 |
| 50nM-NA | -4e-3 | 0.0513 |
| 5nM+NA | 0.125 | 0.0223 * |
| 50nM+NA | 0.599 | 0.0007 * |

Positive values show pairs of means that are significantly different.

# Oneway Analysis of cystathionine By Label

## Means Comparisons

### Comparisons with a control using Dunnett's Method

Control Group = -NA

| \|d\| | Alpha |
|---|---|
| 2.65691 | 0.05 |

| Level | Abs(Dif)-LSD | p-Value |
|---|---|---|
| -NA | -0.9 | 1.0000 |
| +NA | 0.073 | 0.0304 * |
| 50nM+NA | 1.452 | <.0001 * |
| 50nM-NA | 1.459 | <.0001 * |
| 5nM-NA | 1.6 | <.0001 * |
| 5nM+NA | 1.767 | <.0001 * |

Positive values show pairs of means that are significantly different.

# Oneway Analysis of Cysteineglutathione disulfide By Label

## Means Comparisons

### Comparisons with a control using Dunnett's Method

Control Group = -NA

| \|d\| | Alpha |
|---|---|
| 2.65691 | 0.05 |

| Level | Abs(Dif)-LSD | p-Value | |
|---|---|---|---|
| +NA | 1.843 | <.0001 | * |
| -NA | -0.6 | 1.0000 | |
| 5nM+NA | 0.15 | 0.0101 | * |
| 5nM-NA | 1.189 | <.0001 | * |
| 50nM-NA | 3.011 | <.0001 | * |
| 50nM+NA | 3.063 | <.0001 | * |

Positive values show pairs of means that are significantly different.

# Oneway Analysis of Cystine By Label

## Means Comparisons

### Comparisons with a control using Dunnett's Method

Control Group = -NA

| \|d\| | Alpha |
|---|---|
| 2.65691 | 0.05 |

| Level | Abs(Dif)-LSD | p-Value |
|---|---|---|
| 5nM-NA | -0.94 | 0.9991 |
| -NA | -1.04 | 1.0000 |
| +NA | -0.08 | 0.0800 |
| 5nM+NA | -0.04 | 0.0612 |
| 50nM-NA | 1.318 | <.0001 * |
| 50nM+NA | 2.62 | <.0001 * |

Positive values show pairs of means that are significantly different.

# Oneway Analysis of cytidine By Label

## Means Comparisons

### Comparisons with a control using Dunnett's Method

Control Group = -NA

| \|d\| | Alpha |
|---|---|
| 2.65691 | 0.05 |

| Level | Abs(Dif)-LSD | p-Value | |
|---|---|---|---|
| 50nM+NA | 4.089 | <.0001 | * |
| 50nM-NA | 2.877 | <.0001 | * |
| 5nM+NA | 1.664 | <.0001 | * |
| 5nM-NA | 1.649 | <.0001 | * |
| +NA | -0.63 | 1.0000 | |
| -NA | -0.65 | 1.0000 | |

Positive values show pairs of means that are significantly different.

# Oneway Analysis of cytosine By Label

## Means Comparisons

### Comparisons with a control using Dunnett's Method

Control Group = -NA

| \|d\| | Alpha |
|---|---|
| 2.65691 | 0.05 |

| Level | Abs(Dif)-LSD | p-Value | |
|---|---|---|---|
| 5nM+NA | 0.213 | 0.0101 | * |
| 5nM-NA | -0.07 | 0.0830 | |
| 50nM-NA | -0.13 | 0.1174 | |
| 50nM+NA | -0.29 | 0.2958 | |
| -NA | -0.85 | 1.0000 | |
| +NA | 0.043 | 0.0367 | * |

Positive values show pairs of means that are significantly different.

# Oneway Analysis of dAMP By Label

## Means Comparisons

### Comparisons with a control using Dunnett's Method

Control Group = -NA

| \|d\| | Alpha |
|---|---|
| 2.65691 | 0.05 |

| Level | Abs(Dif)-LSD | p-Value |
|---|---|---|
| -NA | -0.42 | 1.0000 |
| +NA | 0.6 | <.0001 * |
| 5nM-NA | 3.83 | <.0001 * |
| 50nM-NA | 3.83 | <.0001 * |
| 5nM+NA | 3.83 | <.0001 * |
| 50nM+NA | 3.83 | <.0001 * |

Positive values show pairs of means that are significantly different.

# Oneway Analysis of dCMP By Label

## Means Comparisons

### Comparisons with a control using Dunnett's Method

Control Group = -NA

| \|d\| | Alpha |
|---|---|
| 2.65691 | 0.05 |

| Level | Abs(Dif)-LSD | p-Value | |
|---|---|---|---|
| 5nM-NA | 1.289 | <.0001 | * |
| 5nM+NA | 0.501 | 0.0009 | * |
| +NA | -0.5 | 0.7397 | |
| -NA | -0.84 | 1.0000 | |
| 50nM-NA | -0.04 | 0.0684 | |
| 50nM+NA | 0.461 | 0.0012 | * |

Positive values show pairs of means that are significantly different.

# Oneway Analysis of deoxyadenosine By Label

## Means Comparisons

### Comparisons with a control using Dunnett's Method

Control Group = -NA

| \|d\| | Alpha |
|---|---|
| 2.65691 | 0.05 |

| Level | Abs(Dif)-LSD | p-Value | |
|---|---|---|---|
| 5nM+NA | -0.01 | 0.0564 | |
| +NA | -0.4 | 0.6490 | |
| -NA | -0.72 | 1.0000 | |
| 5nM-NA | -0.53 | 0.9227 | |
| 50nM+NA | -0.11 | 0.1169 | |
| 50nM-NA | 0.041 | 0.0356 | * |

Positive values show pairs of means that are significantly different.

# Oneway Analysis of Deoxyheptulose-7-phosphate By Label

## Means Comparisons

### Comparisons with a control using Dunnett's Method

Control Group = -NA

| \|d\| | Alpha |
|---|---|
| 2.65691 | 0.05 |

| Level | Abs(Dif)-LSD | p-Value | |
|---|---|---|---|
| +NA | -0.36 | 0.6229 | |
| -NA | -0.67 | 1.0000 | |
| 5nM+NA | -0.36 | 0.6173 | |
| 50nM-NA | 0.565 | 0.0001 | * |
| 5nM-NA | 0.59 | 0.0001 | * |
| 50nM+NA | 0.791 | <.0001 | * |

Positive values show pairs of means that are significantly different.

# Oneway Analysis of deoxyinosine By Label

## Means Comparisons

### Comparisons with a control using Dunnett's Method

Control Group = -NA

| \|d\| | Alpha |
|---|---|
| 2.65691 | 0.05 |

| Level | Abs(Dif)-LSD | p-Value |
|---|---|---|
| 5nM-NA | -0.74 | 0.5634 |
| 5nM+NA | -1.06 | 0.9142 |
| -NA | -1.47 | 1.0000 |
| +NA | -1 | 0.8677 |
| 50nM+NA | -0.98 | 0.8471 |
| 50nM-NA | -0.62 | 0.4162 |

Positive values show pairs of means that are significantly different.

# Oneway Analysis of deoxyribose-phosphate By Label

## Means Comparisons

### Comparisons with a control using Dunnett's Method

Control Group = -NA

| \|d\| | Alpha |
|---|---|
| 2.65691 | 0.05 |

| Level | Abs(Dif)-LSD | p-Value |
|---|---|---|
| 5nM+NA | -0.95 | 0.8959 |
| 50nM-NA | -1.01 | 0.9442 |
| +NA | -1.11 | 0.9874 |
| 5nM-NA | -1.16 | 0.9955 |
| -NA | -1.34 | 1.0000 |
| 50nM+NA | -1.16 | 0.9955 |

Positive values show pairs of means that are significantly different.

# Oneway Analysis of D-erythrose-4-phosphate By Label

## Means Comparisons

### Comparisons with a control using Dunnett's Method

Control Group = -NA

| \|d\| | Alpha |
|---|---|
| 2.65691 | 0.05 |

| Level | Abs(Dif)-LSD | p-Value | |
|---|---|---|---|
| -NA | -0.94 | 1.0000 | |
| +NA | 0.314 | 0.0058 | * |
| 5nM-NA | 1.329 | <.0001 | * |
| 50nM-NA | 1.708 | <.0001 | * |
| 5nM+NA | 1.994 | <.0001 | * |
| 50nM+NA | 2.045 | <.0001 | * |

Positive values show pairs of means that are significantly different.

# Oneway Analysis of dGDP By Label

## Means Comparisons

### Comparisons with a control using Dunnett's Method

Control Group = -NA

| \|d\| | Alpha |
|---|---|
| 2.65691 | 0.05 |

| Level | Abs(Dif)-LSD | p-Value | |
|---|---|---|---|
| 50nM+NA | 1.009 | <.0001 | * |
| 50nM-NA | 0.955 | <.0001 | * |
| 5nM-NA | 0.837 | <.0001 | * |
| 5nM+NA | 0.75 | <.0001 | * |
| -NA | -0.56 | 1.0000 | |
| +NA | -0.01 | 0.0538 | |

Positive values show pairs of means that are significantly different.

# Oneway Analysis of D-gluconate By Label

## Means Comparisons

### Comparisons with a control using Dunnett's Method

Control Group = -NA

| \|d\| | Alpha |
|---|---|
| 2.65691 | 0.05 |

| Level | Abs(Dif)-LSD | p-Value |
|---|---|---|
| 5nM-NA | -0.42 | 0.3859 |
| 50nM-NA | -0.58 | 0.6558 |
| 5nM+NA | -0.93 | 0.9987 |
| -NA | -1.03 | 1.0000 |
| 50nM+NA | -0.78 | 0.9423 |
| +NA | 0.001 | 0.0497 * |

Positive values show pairs of means that are significantly different.

# Oneway Analysis of D-glyceraldehdye-3-phosphate By Label

## Means Comparisons

### Comparisons with a control using Dunnett's Method

Control Group = -NA

| \|d\| | Alpha |
|---|---|
| 2.65691 | 0.05 |

| Level | Abs(Dif)-LSD | p-Value | |
|---|---|---|---|
| 50nM+NA | 3.797 | <.0001 | * |
| 50nM-NA | 3.183 | <.0001 | * |
| 5nM-NA | 0.965 | <.0001 | * |
| 5nM+NA | 0.848 | 0.0001 | * |
| -NA | -0.99 | 1.0000 | |
| +NA | -0.94 | 1.0000 | |

Positive values show pairs of means that are significantly different.

# Oneway Analysis of dGTP By Label

## Means Comparisons

### Comparisons with a control using Dunnett's Method

Control Group = -NA

| \|d\| | Alpha |
|---|---|
| 2.65691 | 0.05 |

| Level | Abs(Dif)-LSD | p-Value |
|---|---|---|
| 5nM-NA | -0.08 | 0.0718 |
| 5nM+NA | -0.74 | 0.7508 |
| -NA | -1.22 | 1.0000 |
| 50nM-NA | -1.19 | 1.0000 |
| +NA | -0.7 | 0.6897 |
| 50nM+NA | -0.12 | 0.0896 |

Positive values show pairs of means that are significantly different.

# Oneway Analysis of dihydroxy-acetone-phosphate By Label

## Means Comparisons

### Comparisons with a control using Dunnett's Method

Control Group = -NA

| \|d\| | Alpha |
|---|---|
| 2.65691 | 0.05 |

| Level | Abs(Dif)-LSD | p-Value | |
|---|---|---|---|
| -NA | -1.18 | 1.0000 | |
| +NA | -1.01 | 0.9937 | |
| 5nM+NA | 0.02 | 0.0453 | * |
| 5nM-NA | 0.067 | 0.0355 | * |
| 50nM-NA | 0.627 | 0.0015 | * |
| 50nM+NA | 0.827 | 0.0004 | * |

Positive values show pairs of means that are significantly different.

# Oneway Analysis of dimethylglycine By Label

## Means Comparisons

### Comparisons with a control using Dunnett's Method

Control Group = -NA

| \|d\| | Alpha |
|---|---|
| 2.65691 | 0.05 |

| Level | Abs(Dif)-LSD | p-Value |
|---|---|---|
| 50nM-NA | -0.08 | 0.0945 |
| 50nM+NA | -0.17 | 0.1749 |
| 5nM-NA | -0.63 | 0.9935 |
| 5nM+NA | -0.65 | 0.9974 |
| -NA | -0.74 | 1.0000 |
| +NA | -0.34 | 0.4728 |

Positive values show pairs of means that are significantly different.

# Oneway Analysis of DL-Pipecolic acid By Label

## Means Comparisons

### Comparisons with a control using Dunnett's Method

Control Group = -NA

| \|d\| | Alpha |
|---|---|
| 2.65691 | 0.05 |

| Level | Abs(Dif)-LSD | p-Value |
|---|---|---|
| 5nM-NA | -0.89 | 0.9948 |
| 5nM+NA | -0.95 | 0.9995 |
| -NA | -1.04 | 1.0000 |
| 50nM-NA | -0.48 | 0.4764 |
| +NA | -0.34 | 0.2826 |
| 50nM+NA | -0.23 | 0.1679 |

Positive values show pairs of means that are significantly different.

# Oneway Analysis of D-Mannitol By Label

## Means Comparisons

### Comparisons with a control using Dunnett's Method

Control Group = -NA

| \|d\| | Alpha |
|---|---|
| 2.65691 | 0.05 |

| Level | Abs(Dif)-LSD | p-Value | |
|---|---|---|---|
| 5nM-NA | -0.36 | 0.8603 | |
| -NA | -0.54 | 1.0000 | |
| 50nM-NA | -0.41 | 0.9461 | |
| 50nM+NA | -0.06 | 0.0978 | |
| 5nM+NA | 0.206 | 0.0041 | * |
| +NA | 0.347 | 0.0006 | * |

Positive values show pairs of means that are significantly different.

# Oneway Analysis of Sedoheptulose-1-7-bisphosphate By Label

## Means Comparisons

### Comparisons with a control using Dunnett's Method

Control Group = -NA

| \|d\| | Alpha |
|---|---|
| 2.65691 | 0.05 |

| Level | Abs(Dif)-LSD | p-Value | |
|---|---|---|---|
| 50nM+NA | 3.684 | <.0001 | * |
| 50nM-NA | 3.536 | <.0001 | * |
| 5nM+NA | 1.524 | <.0001 | * |
| 5nM-NA | 1.271 | <.0001 | * |
| -NA | -0.54 | 1.0000 | |
| +NA | -0.45 | 0.9898 | |

Positive values show pairs of means that are significantly different.

# Oneway Analysis of FAD By Label

## Means Comparisons

### Comparisons with a control using Dunnett's Method

Control Group = -NA

| \|d\| | Alpha |
|---|---|
| 2.65691 | 0.05 |

| Level | Abs(Dif)-LSD | p-Value | |
|---|---|---|---|
| -NA | -0.94 | 1.0000 | |
| +NA | -0.84 | 0.9985 | |
| 5nM+NA | -0.53 | 0.6609 | |
| 5nM-NA | -0.28 | 0.2459 | |
| 50nM+NA | 0.08 | 0.0297 | * |
| 50nM-NA | 0.279 | 0.0075 | * |

Positive values show pairs of means that are significantly different.

# Oneway Analysis of folate By Label

## Means Comparisons

### Comparisons with a control using Dunnett's Method

Control Group = -NA

| \|d\| | Alpha |
|---|---|
| 2.65691 | 0.05 |

| Level | Abs(Dif)-LSD | p-Value |
|---|---|---|
| 5nM-NA | -0.75 | 0.8899 |
| 5nM+NA | -0.91 | 0.9928 |
| -NA | -1.08 | 1.0000 |
| +NA | -0.68 | 0.7838 |
| 50nM+NA | -0.55 | 0.5765 |
| 50nM-NA | -0.36 | 0.2831 |

Positive values show pairs of means that are significantly different.

# Oneway Analysis of fructose-1,6-bisphosphate By Label

## Means Comparisons

### Comparisons with a control using Dunnett's Method

Control Group = -NA

| \|d\| | Alpha |
|---|---|
| 2.65691 | 0.05 |

| Level | Abs(Dif)-LSD | p-Value | |
|---|---|---|---|
| 50nM-NA | 3.992 | <.0001 | * |
| 50nM+NA | 3.957 | <.0001 | * |
| 5nM+NA | 0.754 | <.0001 | * |
| 5nM-NA | 0.668 | <.0001 | * |
| +NA | -0.58 | 1.0000 | |
| -NA | -0.58 | 1.0000 | |

Positive values show pairs of means that are significantly different.

# Oneway Analysis of fructose-6-phosphate By Label

## Means Comparisons

### Comparisons with a control using Dunnett's Method

Control Group = -NA

| \|d\| | Alpha |
|---|---|
| 2.65691 | 0.05 |

| Level | Abs(Dif)-LSD | p-Value | |
|---|---|---|---|
| 50nM-NA | 3.969 | <.0001 | * |
| 50nM+NA | 3.915 | <.0001 | * |
| 5nM+NA | 0.756 | <.0001 | * |
| 5nM-NA | 0.594 | <.0001 | * |
| -NA | -0.44 | 1.0000 | |
| +NA | -0.44 | 1.0000 | |

Positive values show pairs of means that are significantly different.

# Oneway Analysis of fumarate By Label

## Means Comparisons

### Comparisons with a control using Dunnett's Method

Control Group = -NA

| \|d\| | Alpha |
|---|---|
| 2.65691 | 0.05 |

| Level | Abs(Dif)-LSD | p-Value | |
|---|---|---|---|
| 5nM+NA | 0.975 | 0.0001 | * |
| 5nM-NA | 0.752 | 0.0006 | * |
| +NA | -0.06 | 0.0681 | |
| 50nM+NA | -1.13 | 1.0000 | |
| -NA | -1.14 | 1.0000 | |
| 50nM-NA | -1.07 | 0.9999 | |

Positive values show pairs of means that are significantly different.

# Oneway Analysis of Geranyl-PP By Label

## Means Comparisons

### Comparisons with a control using Dunnett's Method

Control Group = -NA

| \|d\| | Alpha |
|---|---|
| 2.65691 | 0.05 |

| Level | Abs(Dif)-LSD | p-Value | |
|---|---|---|---|
| 50nM-NA | 1.637 | <.0001 | * |
| 50nM+NA | 1.324 | <.0001 | * |
| 5nM+NA | 0.841 | 0.0001 | * |
| 5nM-NA | -0.04 | 0.0655 | |
| +NA | -0.84 | 0.9988 | |
| -NA | -0.94 | 1.0000 | |

Positive values show pairs of means that are significantly different.

# Oneway Analysis of Glucaric acid By Label

## Means Comparisons

### Comparisons with a control using Dunnett's Method

Control Group = -NA

| \|d\| | Alpha |
|---|---|
| 2.65691 | 0.05 |

| Level | Abs(Dif)-LSD | p-Value | |
|---|---|---|---|
| 50nM-NA | 2.304 | 0.0017 | * |
| 5nM+NA | 0.869 | 0.0151 | * |
| +NA | -1.69 | 0.3360 | |
| 50nM+NA | -2.87 | 0.7852 | |
| 5nM-NA | -4.54 | 1.0000 | |
| -NA | -4.56 | 1.0000 | |

Positive values show pairs of means that are significantly different.

# Oneway Analysis of glucono-D-lactone By Label

## Means Comparisons

### Comparisons with a control using Dunnett's Method

Control Group = -NA

| \|d\| | Alpha |
|---|---|
| 2.65691 | 0.05 |

| Level | Abs(Dif)-LSD | p-Value | |
|---|---|---|---|
| 50nM-NA | 0.221 | 0.0190 | * |
| 5nM-NA | -0.02 | 0.0534 | |
| 5nM+NA | -0.34 | 0.1836 | |
| 50nM+NA | -0.57 | 0.3851 | |
| -NA | -1.41 | 1.0000 | |
| +NA | -1.19 | 0.9918 | |

Positive values show pairs of means that are significantly different.

# Oneway Analysis of Glucose By Label

## Means Comparisons

### Comparisons with a control using Dunnett's Method

Control Group = -NA

| \|d\| | Alpha |
|---|---|
| 2.65691 | 0.05 |

| Level | Abs(Dif)-LSD | p-Value | |
|---|---|---|---|
| 5nM-NA | -1.32 | 1.0000 | |
| -NA | -1.35 | 1.0000 | |
| 50nM+NA | -0.14 | 0.0922 | |
| 5nM+NA | 0.157 | 0.0244 | * |
| 50nM-NA | 0.492 | 0.0047 | * |
| +NA | 0.767 | 0.0011 | * |

Positive values show pairs of means that are significantly different.

# Oneway Analysis of glucose-6-phosphate By Label

## Means Comparisons

### Comparisons with a control using Dunnett's Method

Control Group = -NA

| \|d\| | Alpha |
|---|---|
| 2.65691 | 0.05 |

| Level | Abs(Dif)-LSD | p-Value |
|---|---|---|
| 5nM+NA | -0.17 | 0.1086 |
| +NA | -0.81 | 0.7865 |
| 5nM-NA | -1.15 | 0.9991 |
| -NA | -1.28 | 1.0000 |
| 50nM+NA | -0.75 | 0.7086 |
| 50nM-NA | -0.25 | 0.1457 |

Positive values show pairs of means that are significantly different.

# Oneway Analysis of glutamate By Label

## Means Comparisons

### Comparisons with a control using Dunnett's Method

Control Group = -NA

| \|d\| | Alpha |
|---|---|
| 2.65691 | 0.05 |

| Level | Abs(Dif)-LSD | p-Value |
|---|---|---|
| 50nM+NA | -0.54 | 0.7700 |
| 5nM-NA | -0.62 | 0.9116 |
| 50nM-NA | -0.62 | 0.9145 |
| 5nM+NA | -0.7 | 0.9812 |
| -NA | -0.87 | 1.0000 |
| +NA | -0.35 | 0.3878 |

Positive values show pairs of means that are significantly different.

# Oneway Analysis of glutamine By Label

## Means Comparisons

### Comparisons with a control using Dunnett's Method

Control Group = -NA

| \|d\| | Alpha |
|---|---|
| 2.65691 | 0.05 |

| Level | Abs(Dif)-LSD | p-Value |
|---|---|---|
| 5nM-NA | -0.62 | 0.8562 |
| -NA | -0.92 | 1.0000 |
| 5nM+NA | -0.91 | 1.0000 |
| 50nM-NA | -0.7 | 0.9578 |
| +NA | -0.4 | 0.4390 |
| 50nM+NA | -0.1 | 0.0954 |

Positive values show pairs of means that are significantly different.

# Oneway Analysis of glutathione By Label

## Means Comparisons

### Comparisons with a control using Dunnett's Method

Control Group = -NA

| \|d\| | Alpha |
|---|---|
| 2.65691 | 0.05 |

| Level | Abs(Dif)-LSD | p-Value |
|---|---|---|
| -NA | -0.64 | 1.0000 |
| 50nM-NA | -0.45 | 0.8865 |
| 50nM+NA | -0.21 | 0.2737 |
| +NA | -0.04 | 0.0719 |
| 5nM-NA | 0.347 | 0.0014 * |
| 5nM+NA | 1.052 | <.0001 * |

Positive values show pairs of means that are significantly different.

# Oneway Analysis of glutathione disulfide By Label

## Means Comparisons

### Comparisons with a control using Dunnett's Method

Control Group = -NA

| \|d\| | Alpha |
|---|---|
| 2.65691 | 0.05 |

| Level | Abs(Dif)-LSD | p-Value | |
|---|---|---|---|
| -NA | -0.74 | 1.0000 | |
| +NA | -0.21 | 0.2306 | |
| 50nM-NA | 0.117 | 0.0187 | * |
| 5nM-NA | 0.555 | 0.0003 | * |
| 50nM+NA | 0.621 | 0.0002 | * |
| 5nM+NA | 0.721 | <.0001 | * |

Positive values show pairs of means that are significantly different.

# Oneway Analysis of Glycerophosphocholine By Label

## Means Comparisons

### Comparisons with a control using Dunnett's Method

Control Group = -NA

| \|d\| | Alpha |
|---|---|
| 2.65691 | 0.05 |

| Level | Abs(Dif)-LSD | p-Value | |
|---|---|---|---|
| 50nM+NA | 1.816 | <.0001 | * |
| 50nM-NA | 1.684 | <.0001 | * |
| 5nM+NA | 0.53 | <.0001 | * |
| 5nM-NA | 0.368 | 0.0001 | * |
| -NA | -0.44 | 1.0000 | |
| +NA | -0.2 | 0.4796 | |

Positive values show pairs of means that are significantly different.

# Oneway Analysis of glycine By Label

## Means Comparisons

### Comparisons with a control using Dunnett's Method

Control Group = -NA

| \|d\| | Alpha |
|---|---|
| 2.65691 | 0.05 |

| Level | Abs(Dif)-LSD | p-Value | |
|---|---|---|---|
| -NA | -0.87 | 1.0000 | |
| 5nM+NA | -0.83 | 1.0000 | |
| 5nM-NA | -0.75 | 0.9965 | |
| 50nM+NA | -0.72 | 0.9901 | |
| 50nM-NA | -0.53 | 0.7485 | |
| +NA | 0.092 | 0.0261 | * |

Positive values show pairs of means that are significantly different.

# Oneway Analysis of GMP By Label

## Means Comparisons

### Comparisons with a control using Dunnett's Method

Control Group = -NA

| \|d\| | Alpha |
|---|---|
| 2.65691 | 0.05 |

| Level | Abs(Dif)-LSD | p-Value | |
|---|---|---|---|
| +NA | 0.419 | 0.0050 | * |
| -NA | -1.18 | 1.0000 | |
| 5nM+NA | -0.29 | 0.1911 | |
| 5nM-NA | 0.172 | 0.0201 | * |
| 50nM+NA | 0.212 | 0.0162 | * |
| 50nM-NA | 0.428 | 0.0047 | * |

Positive values show pairs of means that are significantly different.

# Oneway Analysis of Guanidoacetic acid By Label

## Means Comparisons

### Comparisons with a control using Dunnett's Method

Control Group = -NA

| \|d\| | Alpha |
|---|---|
| 2.65691 | 0.05 |

| Level | Abs(Dif)-LSD | p-Value | |
|---|---|---|---|
| -NA | -0.68 | 1.0000 | |
| +NA | -0.17 | 0.1974 | |
| 5nM+NA | 0.74 | <.0001 | * |
| 5nM-NA | 0.822 | <.0001 | * |
| 50nM-NA | 1.159 | <.0001 | * |
| 50nM+NA | 1.279 | <.0001 | * |

Positive values show pairs of means that are significantly different.

# Oneway Analysis of histidine By Label

## Means Comparisons

### Comparisons with a control using Dunnett's Method

Control Group = -NA

| \|d\| | Alpha |
|---|---|
| 2.65691 | 0.05 |

| Level | Abs(Dif)-LSD | p-Value | |
|---|---|---|---|
| 5nM-NA | 0.656 | <.0001 | * |
| 50nM-NA | 0.644 | <.0001 | * |
| 5nM+NA | 0.281 | 0.0024 | * |
| 50nM+NA | 0.165 | 0.0089 | * |
| -NA | -0.61 | 1.0000 | |
| +NA | -0.12 | 0.1507 | |

Positive values show pairs of means that are significantly different.

# Oneway Analysis of homocysteine By Label

## Means Comparisons

### Comparisons with a control using Dunnett's Method

Control Group = -NA

| \|d\| | Alpha |
|---|---|
| 2.65691 | 0.05 |

| Level | Abs(Dif)-LSD | p-Value |
|---|---|---|
| +NA | -0.07 | 0.2942 |
| 5nM-NA | -0.19 | 0.9956 |
| -NA | -0.22 | 1.0000 |
| 5nM+NA | -0.2 | 0.9997 |
| 50nM-NA | -0.05 | 0.1619 |
| 50nM+NA | -0.04 | 0.1487 |

Positive values show pairs of means that are significantly different.

# Oneway Analysis of homoserine By Label

## Means Comparisons

### Comparisons with a control using Dunnett's Method

Control Group = -NA

| \|d\| | Alpha |
|---|---|
| 2.65691 | 0.05 |

| Level | Abs(Dif)-LSD | p-Value | |
|---|---|---|---|
| 50nM-NA | 0.761 | 0.0019 | * |
| 5nM-NA | -0.3 | 0.1491 | |
| +NA | -0.63 | 0.3885 | |
| 5nM+NA | -0.67 | 0.4350 | |
| -NA | -1.54 | 1.0000 | |
| 50nM+NA | -1.46 | 0.9999 | |

Positive values show pairs of means that are significantly different.

# Oneway Analysis of Hydroxyisocaproic acid By Label

## Means Comparisons

### Comparisons with a control using Dunnett's Method

Control Group = -NA

| \|d\| | Alpha |
|---|---|
| 2.65691 | 0.05 |

| Level | Abs(Dif)-LSD | p-Value |
|---|---|---|
| +NA | -0.83 | 0.9913 |
| -NA | -0.99 | 1.0000 |
| 5nM+NA | 1.404 | <.0001 * |
| 5nM-NA | 1.447 | <.0001 * |
| 50nM-NA | 1.818 | <.0001 * |
| 50nM+NA | 1.833 | <.0001 * |

Positive values show pairs of means that are significantly different.

# Oneway Analysis of Hydroxylamine By Label

## Means Comparisons

### Comparisons with a control using Dunnett's Method

Control Group = -NA

| \|d\| | Alpha |
|---|---|
| 2.65691 | 0.05 |

| Level | Abs(Dif)-LSD | p-Value |
|---|---|---|
| 50nM-NA | -1.64 | 0.7546 |
| 5nM+NA | -2.33 | 0.9961 |
| -NA | -2.69 | 1.0000 |
| +NA | -2.68 | 1.0000 |
| 50nM+NA | -1.95 | 0.9197 |
| 5nM-NA | -1.37 | 0.5695 |

Positive values show pairs of means that are significantly different.

# Oneway Analysis of hydroxyphenylpyruvate By Label

## Means Comparisons

### Comparisons with a control using Dunnett's Method

Control Group = -NA

| \|d\| | Alpha |
|---|---|
| 2.65691 | 0.05 |

| Level | Abs(Dif)-LSD | p-Value |
|---|---|---|
| -NA | -1.69 | 1.0000 |
| 5nM-NA | -1.3 | 0.9576 |
| 5nM+NA | -1.04 | 0.7569 |
| 50nM+NA | -0.95 | 0.6588 |
| 50nM-NA | -0.62 | 0.3331 |
| +NA | -0.54 | 0.2731 |

Positive values show pairs of means that are significantly different.

# Oneway Analysis of hydroxyproline By Label

## Means Comparisons

### Comparisons with a control using Dunnett's Method

Control Group = -NA

| \|d\| | Alpha |
|---|---|
| 2.65691 | 0.05 |

| Level | Abs(Dif)-LSD | p-Value | |
|---|---|---|---|
| 5nM+NA | 0.313 | 0.0033 | * |
| 50nM+NA | 0.195 | 0.0098 | * |
| 50nM-NA | -0.1 | 0.1097 | |
| 5nM-NA | -0.18 | 0.1849 | |
| +NA | -0.6 | 0.9723 | |
| -NA | -0.76 | 1.0000 | |

Positive values show pairs of means that are significantly different.

# Oneway Analysis of IDP By Label

## Means Comparisons

### Comparisons with a control using Dunnett's Method

Control Group = -NA

| \|d\| | Alpha |
|---|---|
| 2.65691 | 0.05 |

| Level | Abs(Dif)-LSD | p-Value | |
|---|---|---|---|
| 50nM+NA | 1.066 | <.0001 | * |
| 50nM-NA | 0.201 | 0.0136 | * |
| 5nM+NA | -0.01 | 0.0519 | |
| 5nM-NA | -0.46 | 0.5086 | |
| -NA | -0.97 | 1.0000 | |
| +NA | -0.35 | 0.3179 | |

Positive values show pairs of means that are significantly different.

# Oneway Analysis of Imidazoleacetic acid By Label

## Means Comparisons

### Comparisons with a control using Dunnett's Method

Control Group = -NA

| \|d\| | Alpha |
|---|---|
| 2.65691 | 0.05 |

| Level | Abs(Dif)-LSD | p-Value | |
|---|---|---|---|
| +NA | -0.76 | 0.9998 | |
| -NA | -0.82 | 1.0000 | |
| 5nM+NA | 0.001 | 0.0496 | * |
| 5nM-NA | 0.428 | 0.0015 | * |
| 50nM-NA | 0.774 | <.0001 | * |
| 50nM+NA | 1.059 | <.0001 | * |

Positive values show pairs of means that are significantly different.

# Oneway Analysis of IMP By Label

## Means Comparisons

### Comparisons with a control using Dunnett's Method

Control Group = -NA

| \|d\| | Alpha |
|---|---|
| 2.65691 | 0.05 |

| Level | Abs(Dif)-LSD | p-Value | |
|---|---|---|---|
| 50nM+NA | 2.238 | <.0001 | * |
| 50nM-NA | 1.776 | <.0001 | * |
| 5nM-NA | 0.823 | <.0001 | * |
| 5nM+NA | 0.459 | 0.0007 | * |
| -NA | -0.72 | 1.0000 | |
| +NA | -0.57 | 0.9719 | |

Positive values show pairs of means that are significantly different.

# Oneway Analysis of indole By Label

## Means Comparisons

### Comparisons with a control using Dunnett's Method

Control Group = -NA

| \|d\| | Alpha |
|---|---|
| 2.65691 | 0.05 |

| Level | Abs(Dif)-LSD | p-Value | |
|---|---|---|---|
| 5nM+NA | 0.59 | 0.0002 | * |
| 5nM-NA | 0.457 | 0.0009 | * |
| 50nM-NA | 0.217 | 0.0081 | * |
| 50nM+NA | 0.051 | 0.0332 | * |
| -NA | -0.76 | 1.0000 | |
| +NA | -0.5 | 0.8269 | |

Positive values show pairs of means that are significantly different.

# Oneway Analysis of Indole-3-carboxylic acid By Label

## Means Comparisons

### Comparisons with a control using Dunnett's Method

Control Group = -NA

| \|d\| | Alpha |
|---|---|
| 2.65691 | 0.05 |

| Level | Abs(Dif)-LSD | p-Value | |
|---|---|---|---|
| -NA | -1.17 | 1.0000 | |
| +NA | -0.5 | 0.4198 | |
| 5nM-NA | 0.462 | 0.0038 | * |
| 5nM+NA | 0.671 | 0.0011 | * |
| 50nM+NA | 1.175 | <.0001 | * |
| 50nM-NA | 1.178 | <.0001 | * |

Positive values show pairs of means that are significantly different.

# Oneway Analysis of Indoleacrylic acid By Label

## Means Comparisons

### Comparisons with a control using Dunnett's Method

Control Group = -NA

| \|d\| | Alpha |
|---|---|
| 2.65691 | 0.05 |

| Level | Abs(Dif)-LSD | p-Value |
|---|---|---|
| 5nM+NA | -0.17 | 0.1175 |
| 5nM-NA | -0.17 | 0.1186 |
| 50nM+NA | -0.46 | 0.3980 |
| 50nM-NA | -0.59 | 0.6014 |
| -NA | -1.12 | 1.0000 |
| +NA | -0.99 | 0.9983 |

Positive values show pairs of means that are significantly different.

# Oneway Analysis of inosine By Label

## Means Comparisons

### Comparisons with a control using Dunnett's Method

Control Group = -NA

| \|d\| | Alpha |
|---|---|
| 2.65691 | 0.05 |

| Level | Abs(Dif)-LSD | p-Value | |
|---|---|---|---|
| 5nM-NA | 2.803 | <.0001 | * |
| 50nM-NA | 1.099 | <.0001 | * |
| 50nM+NA | -0.34 | 0.3203 | |
| 5nM+NA | -0.42 | 0.4489 | |
| -NA | -0.95 | 1.0000 | |
| +NA | -0.74 | 0.9653 | |

Positive values show pairs of means that are significantly different.

# Oneway Analysis of isocitrate By Label

## Means Comparisons

### Comparisons with a control using Dunnett's Method

Control Group = -NA

| \|d\| | Alpha |
|---|---|
| 2.65691 | 0.05 |

| Level | Abs(Dif)-LSD | p-Value |
|---|---|---|
| 5nM-NA | -2.07 | 1.0000 |
| -NA | -2.14 | 1.0000 |
| 5nM+NA | -1.66 | 0.9626 |
| +NA | -1.51 | 0.8987 |
| 50nM+NA | -1.03 | 0.5136 |
| 50nM-NA | -0.68 | 0.2696 |

Positive values show pairs of means that are significantly different.

# Oneway Analysis of Isoleucine By Label

## Means Comparisons

### Comparisons with a control using Dunnett's Method

Control Group = -NA

| \|d\| | Alpha |
|---|---|
| 2.65691 | 0.05 |

| Level | Abs(Dif)-LSD | p-Value | |
|---|---|---|---|
| 5nM-NA | 0.136 | 0.0076 | * |
| 50nM+NA | -0.03 | 0.0725 | |
| 50nM-NA | -0.18 | 0.3788 | |
| 5nM+NA | -0.3 | 0.8235 | |
| -NA | -0.46 | 1.0000 | |
| +NA | -0.34 | 0.9195 | |

Positive values show pairs of means that are significantly different.

# Oneway Analysis of Ketohexanoic acid By Label

## Means Comparisons

### Comparisons with a control using Dunnett's Method

Control Group = -NA

| \|d\| | Alpha |
|---|---|
| 2.65691 | 0.05 |

| Level | Abs(Dif)-LSD | p-Value | |
|---|---|---|---|
| 5nM-NA | 2.618 | 0.0003 | * |
| +NA | -0.86 | 0.1843 | |
| 5nM+NA | -1.62 | 0.4608 | |
| 50nM+NA | -2.21 | 0.7555 | |
| -NA | -3.61 | 1.0000 | |
| 50nM-NA | -2.1 | 0.7026 | |

Positive values show pairs of means that are significantly different.

# Oneway Analysis of Ketoleucine By Label

## Means Comparisons

### Comparisons with a control using Dunnett's Method

Control Group = -NA

| \|d\| | Alpha |
|---|---|
| 2.65691 | 0.05 |

| Level | Abs(Dif)-LSD | p-Value |
|---|---|---|
| -NA | -0.99 | 1.0000 |
| +NA | -0.59 | 0.7269 |
| 5nM-NA | 0.123 | 0.0234 * |
| 5nM+NA | 0.828 | 0.0002 * |
| 50nM+NA | 1.056 | <.0001 * |
| 50nM-NA | 1.501 | <.0001 * |

Positive values show pairs of means that are significantly different.

# Oneway Analysis of Kynurenic acid By Label

## Means Comparisons

### Comparisons with a control using Dunnett's Method

Control Group = -NA

| \|d\| | Alpha |
|---|---|
| 2.65691 | 0.05 |

| Level | Abs(Dif)-LSD | p-Value | |
|---|---|---|---|
| -NA | -0.32 | 1.0000 | |
| +NA | 0.187 | 0.0010 | * |
| 5nM-NA | 1.311 | <.0001 | * |
| 5nM+NA | 1.347 | <.0001 | * |
| 50nM-NA | 1.397 | <.0001 | * |
| 50nM+NA | 1.589 | <.0001 | * |

Positive values show pairs of means that are significantly different.

# Oneway Analysis of Kynurenine By Label

## Means Comparisons

### Comparisons with a control using Dunnett's Method

Control Group = -NA

| \|d\| | Alpha |
|---|---|
| 2.65691 | 0.05 |

| Level | Abs(Dif)-LSD | p-Value |
|---|---|---|
| 5nM-NA | -0.08 | 0.0777 |
| 5nM+NA | -0.33 | 0.2779 |
| 50nM+NA | -0.63 | 0.7876 |
| 50nM-NA | -0.64 | 0.7965 |
| -NA | -1 | 1.0000 |
| +NA | -0.73 | 0.9259 |

Positive values show pairs of means that are significantly different.

# Oneway Analysis of Lactic acid By Label

## Means Comparisons

### Comparisons with a control using Dunnett's Method

Control Group = -NA

| \|d\| | Alpha |
|---|---|
| 2.65691 | 0.05 |

| Level | Abs(Dif)-LSD | p-Value | |
|---|---|---|---|
| -NA | -1.46 | 1.0000 | |
| 5nM-NA | -1.09 | 0.9355 | |
| 50nM-NA | 1.344 | <.0001 | * |
| 50nM+NA | 1.5 | <.0001 | * |
| 5nM+NA | 1.797 | <.0001 | * |
| +NA | 2.192 | <.0001 | * |

Positive values show pairs of means that are significantly different.

# Oneway Analysis of leucine By Label

## Means Comparisons

### Comparisons with a control using Dunnett's Method

Control Group = -NA

| \|d\| | Alpha |
|---|---|
| 2.65691 | 0.05 |

| Level | Abs(Dif)-LSD | p-Value | |
|---|---|---|---|
| 5nM-NA | 0.726 | <.0001 | * |
| 50nM-NA | 0.423 | 0.0004 | * |
| 50nM+NA | 0.423 | 0.0004 | * |
| 5nM+NA | 0.353 | 0.0009 | * |
| -NA | -0.59 | 1.0000 | |
| +NA | -0.28 | 0.5151 | |

Positive values show pairs of means that are significantly different.

# Oneway Analysis of lysine By Label

## Means Comparisons

### Comparisons with a control using Dunnett's Method

Control Group = -NA

| \|d\| | Alpha |
|---|---|
| 2.65691 | 0.05 |

| Level | Abs(Dif)-LSD | p-Value |
|---|---|---|
| 5nM-NA | 0.636 | <.0001 * |
| 5nM+NA | 0.467 | 0.0006 * |
| 50nM-NA | 0.413 | 0.0010 * |
| 50nM+NA | 0.369 | 0.0016 * |
| -NA | -0.71 | 1.0000 |
| +NA | -0.42 | 0.7206 |

Positive values show pairs of means that are significantly different.

# Oneway Analysis of LysoPC(20:4) By Label

## Means Comparisons

### Comparisons with a control using Dunnett's Method

Control Group = -NA

| \|d\| | Alpha |
|---|---|
| 2.65691 | 0.05 |

| Level | Abs(Dif)-LSD | p-Value |
|---|---|---|
| -NA | -1.46 | 1.0000 |
| 50nM-NA | 1.002 | 0.0005 * |
| 5nM-NA | 1.227 | 0.0002 * |
| +NA | 2.165 | <.0001 * |
| 50nM+NA | 2.354 | <.0001 * |
| 5nM+NA | 3.625 | <.0001 * |

Positive values show pairs of means that are significantly different.

# Oneway Analysis of Maleic acid By Label

## Means Comparisons

### Comparisons with a control using Dunnett's Method

Control Group = -NA

| \|d\| | Alpha |
|---|---|
| 2.65691 | 0.05 |

| Level | Abs(Dif)-LSD | p-Value |
|---|---|---|
| 5nM+NA | 0.962 | <.0001 * |
| 5nM-NA | 0.878 | <.0001 * |
| 50nM+NA | 0.8 | <.0001 * |
| 50nM-NA | 0.724 | <.0001 * |
| +NA | -0.25 | 0.6558 |
| -NA | -0.44 | 1.0000 |

Positive values show pairs of means that are significantly different.

# Oneway Analysis of Malic acid By Label

## Means Comparisons

### Comparisons with a control using Dunnett's Method

Control Group = -NA

| \|d\| | Alpha |
|---|---|
| 2.65691 | 0.05 |

| Level | Abs(Dif)-LSD | p-Value | |
|---|---|---|---|
| 5nM+NA | 5.955 | <.0001 | * |
| 5nM-NA | 2.762 | <.0001 | * |
| +NA | -0.89 | 0.2536 | |
| -NA | -2.9 | 1.0000 | |
| 50nM+NA | -0.93 | 0.2725 | |
| 50nM-NA | -0.69 | 0.1826 | |

Positive values show pairs of means that are significantly different.

# Oneway Analysis of Mannose By Label

## Means Comparisons

### Comparisons with a control using Dunnett's Method

Control Group = -NA

| \|d\| | Alpha |
|---|---|
| 2.65691 | 0.05 |

| Level | Abs(Dif)-LSD | p-Value |
|---|---|---|
| 5nM+NA | -0.36 | 0.7199 |
| +NA | -0.37 | 0.7538 |
| 50nM-NA | -0.37 | 0.7618 |
| 50nM+NA | -0.41 | 0.8603 |
| 5nM-NA | -0.5 | 0.9869 |
| -NA | -0.61 | 1.0000 |

Positive values show pairs of means that are significantly different.

# Oneway Analysis of methionine By Label

## Means Comparisons

### Comparisons with a control using Dunnett's Method

Control Group = -NA

| \|d\| | Alpha |
|---|---|
| 2.65691 | 0.05 |

| Level | Abs(Dif)-LSD | p-Value | |
|---|---|---|---|
| 5nM-NA | 0.469 | 0.0003 | * |
| 50nM-NA | 0.287 | 0.0023 | * |
| 5nM+NA | 0.156 | 0.0101 | * |
| 50nM+NA | 0.093 | 0.0196 | * |
| -NA | -0.62 | 1.0000 | |
| +NA | -0.11 | 0.1376 | |

Positive values show pairs of means that are significantly different.

# Oneway Analysis of Methionine sulfoxide By Label

## Means Comparisons

### Comparisons with a control using Dunnett's Method

Control Group = -NA

| \|d\| | Alpha |
|---|---|
| 2.65691 | 0.05 |

| Level | Abs(Dif)-LSD | p-Value | |
|---|---|---|---|
| 5nM-NA | 0.17 | 0.0158 | * |
| 5nM+NA | 0.142 | 0.0192 | * |
| 50nM+NA | -0.28 | 0.2572 | |
| 50nM-NA | -0.61 | 0.8398 | |
| +NA | -0.85 | 0.9997 | |
| -NA | -0.92 | 1.0000 | |

Positive values show pairs of means that are significantly different.

# Oneway Analysis of Methylcysteine By Label

## Means Comparisons

### Comparisons with a control using Dunnett's Method

Control Group = -NA

| \|d\| | Alpha |
|---|---|
| 2.65691 | 0.05 |

| Level | Abs(Dif)-LSD | p-Value | |
|---|---|---|---|
| 5nM-NA | 0.118 | 0.0176 | * |
| 5nM+NA | -0.1 | 0.1156 | |
| 50nM-NA | -0.14 | 0.1476 | |
| 50nM+NA | -0.23 | 0.2745 | |
| -NA | -0.71 | 1.0000 | |
| +NA | -0.18 | 0.2043 | |

Positive values show pairs of means that are significantly different.

# Oneway Analysis of Methylmalonic acid By Label

## Means Comparisons

### Comparisons with a control using Dunnett's Method

Control Group = -NA

| \|d\| | Alpha |
|---|---|
| 2.65691 | 0.05 |

| Level | Abs(Dif)-LSD | p-Value | |
|---|---|---|---|
| -NA | -0.67 | 1.0000 | |
| 5nM-NA | 0.324 | 0.0021 | * |
| 5nM+NA | 0.586 | 0.0001 | * |
| +NA | 0.732 | <.0001 | * |
| 50nM+NA | 0.79 | <.0001 | * |
| 50nM-NA | 0.812 | <.0001 | * |

Positive values show pairs of means that are significantly different.

# Oneway Analysis of myo-inositol By Label

## Means Comparisons

### Comparisons with a control using Dunnett's Method

Control Group = -NA

| \|d\| | Alpha |
|---|---|
| 2.65691 | 0.05 |

| Level | Abs(Dif)-LSD | p-Value |
|---|---|---|
| -NA | -1.25 | 1.0000 |
| 50nM-NA | -0.92 | 0.9243 |
| 5nM+NA | -0.85 | 0.8573 |
| 5nM-NA | -0.83 | 0.8354 |
| +NA | -0.65 | 0.5798 |
| 50nM+NA | -0.26 | 0.1586 |

Positive values show pairs of means that are significantly different.

# Oneway Analysis of N6-Acetyl-L-lysine By Label

## Means Comparisons

### Comparisons with a control using Dunnett's Method

Control Group = -NA

| \|d\| | Alpha |
|---|---|
| 2.65691 | 0.05 |

| Level | Abs(Dif)-LSD | p-Value |
|---|---|---|
| 5nM-NA | 0.605 | <.0001 * |
| 5nM+NA | 0.516 | 0.0001 * |
| 50nM-NA | 0.084 | 0.0202 * |
| 50nM+NA | -0.1 | 0.1372 |
| -NA | -0.58 | 1.0000 |
| +NA | -0.23 | 0.3798 |

Positive values show pairs of means that are significantly different.

# Oneway Analysis of N-Acetylaspartylglutamic acid By Label

## Means Comparisons

### Comparisons with a control using Dunnett's Method

Control Group = -NA

| \|d\| | Alpha |
|---|---|
| 2.65691 | 0.05 |

| Level | Abs(Dif)-LSD | p-Value | |
|---|---|---|---|
| 50nM+NA | -0.17 | 0.1728 | |
| 5nM+NA | -0.25 | 0.3016 | |
| -NA | -0.74 | 1.0000 | |
| +NA | -0.69 | 0.9998 | |
| 5nM-NA | -0.01 | 0.0545 | |
| 50nM-NA | 0.089 | 0.0237 | * |

Positive values show pairs of means that are significantly different.

# Oneway Analysis of N-acetyl-glucosamine-1-phosphate By Label

## Means Comparisons

### Comparisons with a control using Dunnett's Method

Control Group = -NA

| \|d\| | Alpha |
|---|---|
| 2.65691 | 0.05 |

| Level | Abs(Dif)-LSD | p-Value |
|---|---|---|
| 50nM+NA | -0.56 | 0.3838 |
| 50nM-NA | -0.72 | 0.5739 |
| 5nM-NA | -1.07 | 0.9561 |
| -NA | -1.4 | 1.0000 |
| +NA | -1.23 | 0.9974 |
| 5nM+NA | -1.14 | 0.9824 |

Positive values show pairs of means that are significantly different.

# Oneway Analysis of N-acetyl-glutamine By Label

## Means Comparisons

### Comparisons with a control using Dunnett's Method

Control Group = -NA

| \|d\| | Alpha |
|---|---|
| 2.65691 | 0.05 |

| Level | Abs(Dif)-LSD | p-Value | |
|---|---|---|---|
| 5nM+NA | 0.765 | <.0001 | * |
| 5nM-NA | 0.626 | <.0001 | * |
| 50nM-NA | 0.096 | 0.0159 | * |
| 50nM+NA | 0.001 | 0.0494 | * |
| -NA | -0.53 | 1.0000 | |
| +NA | -0.15 | 0.2211 | |

Positive values show pairs of means that are significantly different.

# Oneway Analysis of N-Acetyl-L-alanine By Label

## Means Comparisons

### Comparisons with a control using Dunnett's Method

Control Group = -NA

| \|d\| | Alpha |
|---|---|
| 2.65691 | 0.05 |

| Level | Abs(Dif)-LSD | p-Value | |
|---|---|---|---|
| -NA | -0.51 | 1.0000 | |
| +NA | 0.267 | 0.0016 | * |
| 5nM+NA | 1.629 | <.0001 | * |
| 5nM-NA | 1.641 | <.0001 | * |
| 50nM-NA | 2.47 | <.0001 | * |
| 50nM+NA | 2.602 | <.0001 | * |

Positive values show pairs of means that are significantly different.

# Oneway Analysis of N-acetyl-L-ornithine By Label

## Means Comparisons

### Comparisons with a control using Dunnett's Method

Control Group = -NA

| \|d\| | Alpha |
|---|---|
| 2.65691 | 0.05 |

| Level | Abs(Dif)-LSD | p-Value |
|---|---|---|
| 5nM-NA | 0.955 | <.0001 * |
| 50nM-NA | 0.677 | <.0001 * |
| 5nM+NA | 0.652 | <.0001 * |
| 50nM+NA | 0.127 | 0.0146 * |
| -NA | -0.65 | 1.0000 |
| +NA | -0.07 | 0.0920 |

Positive values show pairs of means that are significantly different.

# Oneway Analysis of N-Acetylputrescine By Label

## Means Comparisons

### Comparisons with a control using Dunnett's Method

Control Group = -NA

| \|d\| | Alpha |
|---|---|
| 2.65691 | 0.05 |

| Level | Abs(Dif)-LSD | p-Value | |
|---|---|---|---|
| -NA | -0.22 | 1.0000 | |
| +NA | 0.194 | 0.0001 | * |
| 5nM-NA | 0.3 | <.0001 | * |
| 50nM-NA | 0.352 | <.0001 | * |
| 5nM+NA | 0.376 | <.0001 | * |
| 50nM+NA | 0.465 | <.0001 | * |

Positive values show pairs of means that are significantly different.

# Oneway Analysis of NAD By Label

## Means Comparisons

### Comparisons with a control using Dunnett's Method

Control Group = -NA

| \|d\| | Alpha |
|---|---|
| 2.65691 | 0.05 |

| Level | Abs(Dif)-LSD | p-Value | |
|---|---|---|---|
| +NA | -1 | 1.0000 | |
| -NA | -1.01 | 1.0000 | |
| 5nM-NA | 5.529 | <.0001 | * |
| 5nM+NA | 5.534 | <.0001 | * |
| 50nM-NA | 5.541 | <.0001 | * |
| 50nM+NA | 5.542 | <.0001 | * |

Positive values show pairs of means that are significantly different.

# Oneway Analysis of NADH By Label

## Means Comparisons

### Comparisons with a control using Dunnett's Method

Control Group = -NA

| \|d\| | Alpha |
|---|---|
| 2.65691 | 0.05 |

| Level | Abs(Dif)-LSD | p-Value |
|---|---|---|
| +NA | -0.65 | 0.9993 |
| -NA | -0.71 | 1.0000 |
| 5nM-NA | 3.342 | <.0001 * |
| 5nM+NA | 3.357 | <.0001 * |
| 50nM-NA | 3.446 | <.0001 * |
| 50nM+NA | 3.463 | <.0001 * |

Positive values show pairs of means that are significantly different.

# Oneway Analysis of NADP By Label

## Means Comparisons

### Comparisons with a control using Dunnett's Method

Control Group = -NA

| \|d\| | Alpha |
|---|---|
| 2.65691 | 0.05 |

| Level | Abs(Dif)-LSD | p-Value | |
|---|---|---|---|
| -NA | -0.49 | 1.0000 | |
| +NA | 0.026 | 0.0365 | * |
| 5nM+NA | 2.2 | <.0001 | * |
| 5nM-NA | 2.28 | <.0001 | * |
| 50nM-NA | 2.741 | <.0001 | * |
| 50nM+NA | 2.826 | <.0001 | * |

Positive values show pairs of means that are significantly different.

# Oneway Analysis of N-carbamoyl-L-aspartate By Label

## Means Comparisons

### Comparisons with a control using Dunnett's Method

Control Group = -NA

| \|d\| | Alpha |
|---|---|
| 2.65691 | 0.05 |

| Level | Abs(Dif)-LSD | p-Value | |
|---|---|---|---|
| -NA | -0.75 | 1.0000 | |
| +NA | 0.775 | <.0001 | * |
| 5nM-NA | 2.213 | <.0001 | * |
| 5nM+NA | 3.011 | <.0001 | * |
| 50nM-NA | 3.032 | <.0001 | * |
| 50nM+NA | 3.312 | <.0001 | * |

Positive values show pairs of means that are significantly different.

# Oneway Analysis of N-Formylglycinamide ribonucleotide By Label

## Means Comparisons

### Comparisons with a control using Dunnett's Method

Control Group = -NA

| \|d\| | Alpha |
|---|---|
| 2.65691 | 0.05 |

| Level | Abs(Dif)-LSD | p-Value | |
|---|---|---|---|
| 50nM+NA | 1.387 | <.0001 | * |
| 50nM-NA | 1.102 | <.0001 | * |
| 5nM+NA | 0.528 | <.0001 | * |
| 5nM-NA | 0.154 | 0.0029 | * |
| +NA | -0.33 | 0.9999 | |
| -NA | -0.35 | 1.0000 | |

Positive values show pairs of means that are significantly different.

# Oneway Analysis of Ng,NG-dimethyl-L-arginine By Label

## Means Comparisons

### Comparisons with a control using Dunnett's Method

Control Group = -NA

| \|d\| | Alpha |
|---|---|
| 2.65691 | 0.05 |

| Level | Abs(Dif)-LSD | p-Value |
|---|---|---|
| 5nM-NA | -0.1 | 0.0905 |
| 5nM+NA | -0.21 | 0.1679 |
| 50nM-NA | -0.62 | 0.8288 |
| 50nM+NA | -0.75 | 0.9725 |
| -NA | -0.94 | 1.0000 |
| +NA | -0.83 | 0.9972 |

Positive values show pairs of means that are significantly different.

# Oneway Analysis of Nicotinamide By Label

## Means Comparisons

### Comparisons with a control using Dunnett's Method

Control Group = -NA

| \|d\| | Alpha |
|---|---|
| 2.65691 | 0.05 |

| Level | Abs(Dif)-LSD | p-Value |
|---|---|---|
| 5nM-NA | -0.79 | 0.6915 |
| 5nM+NA | -1.3 | 1.0000 |
| -NA | -1.36 | 1.0000 |
| 50nM-NA | -1.24 | 0.9993 |
| +NA | -0.79 | 0.7004 |
| 50nM+NA | -0.32 | 0.1779 |

Positive values show pairs of means that are significantly different.

# Oneway Analysis of Nicotinamide ribotide By Label

## Means Comparisons

### Comparisons with a control using Dunnett's Method

Control Group = -NA

| \|d\| | Alpha |
|---|---|
| 2.65691 | 0.05 |

| Level | Abs(Dif)-LSD | p-Value |
|---|---|---|
| -NA | -0.59 | 1.0000 |
| +NA | -0.15 | 0.1990 |
| 5nM-NA | 3.359 | <.0001 * |
| 50nM-NA | 3.359 | <.0001 * |
| 5nM+NA | 3.359 | <.0001 * |
| 50nM+NA | 3.359 | <.0001 * |

Positive values show pairs of means that are significantly different.

# Oneway Analysis of Nicotinic acid By Label

## Means Comparisons

### Comparisons with a control using Dunnett's Method

Control Group = -NA

| \|d\| | Alpha |
|---|---|
| 2.65691 | 0.05 |

| Level | Abs(Dif)-LSD | p-Value | |
|---|---|---|---|
| 5nM+NA | 4.355 | <.0001 | * |
| +NA | 3.618 | <.0001 | * |
| 50nM+NA | 2.991 | <.0001 | * |
| -NA | -0.87 | 1.0000 | |
| 5nM-NA | -0.87 | 1.0000 | |
| 50nM-NA | -0.87 | 1.0000 | |

Positive values show pairs of means that are significantly different.

# Oneway Analysis of Nicotyrine By Label

## Means Comparisons

### Comparisons with a control using Dunnett's Method

Control Group = -NA

| \|d\| | Alpha |
|---|---|
| 2.65691 | 0.05 |

| Level | Abs(Dif)-LSD | p-Value |
|---|---|---|
| 50nM+NA | 0.036 | 0.0016 * |
| 50nM-NA | -0.02 | 0.2920 |
| 5nM-NA | -0.06 | 0.9946 |
| -NA | -0.07 | 1.0000 |
| 5nM+NA | -0.03 | 0.4708 |
| +NA | -0.02 | 0.2033 |

Positive values show pairs of means that are significantly different.

# Oneway Analysis of O-acetyl-L-serine By Label

## Means Comparisons

### Comparisons with a control using Dunnett's Method

Control Group = -NA

| \|d\| | Alpha |
|---|---|
| 2.65691 | 0.05 |

| Level | Abs(Dif)-LSD | p-Value | |
|---|---|---|---|
| -NA | -0.52 | 1.0000 | |
| +NA | 0.034 | 0.0335 | * |
| 5nM-NA | 0.806 | <.0001 | * |
| 5nM+NA | 1.247 | <.0001 | * |
| 50nM-NA | 1.279 | <.0001 | * |
| 50nM+NA | 1.648 | <.0001 | * |

Positive values show pairs of means that are significantly different.

# Oneway Analysis of octulose-phosphate By Label

## Means Comparisons

### Comparisons with a control using Dunnett's Method

Control Group = -NA

| \|d\| | Alpha |
|---|---|
| 2.65691 | 0.05 |

| Level | Abs(Dif)-LSD | p-Value | |
|---|---|---|---|
| 50nM+NA | 3.741 | <.0001 | * |
| 50nM-NA | 3.618 | <.0001 | * |
| 5nM+NA | 1.542 | <.0001 | * |
| 5nM-NA | 1.422 | <.0001 | * |
| -NA | -0.47 | 1.0000 | |
| +NA | -0.35 | 0.9434 | |

Positive values show pairs of means that are significantly different.

# Oneway Analysis of ornithine By Label

## Means Comparisons

### Comparisons with a control using Dunnett's Method

Control Group = -NA

| \|d\| | Alpha |
|---|---|
| 2.65691 | 0.05 |

| Level | Abs(Dif)-LSD | p-Value | |
|---|---|---|---|
| 5nM-NA | 0.664 | <.0001 | * |
| 50nM-NA | 0.446 | 0.0007 | * |
| 5nM+NA | 0.312 | 0.0028 | * |
| 50nM+NA | 0.285 | 0.0037 | * |
| -NA | -0.71 | 1.0000 | |
| +NA | -0.63 | 0.9977 | |

Positive values show pairs of means that are significantly different.

# Oneway Analysis of orotate By Label

## Means Comparisons

### Comparisons with a control using Dunnett's Method

Control Group = -NA

| \|d\| | Alpha |
|---|---|
| 2.65691 | 0.05 |

| Level | Abs(Dif)-LSD | p-Value |
|---|---|---|
| 50nM-NA | 0.14 | 0.0192 * |
| 5nM+NA | -0.02 | 0.0553 |
| 5nM-NA | -0.06 | 0.0756 |
| 50nM+NA | -0.19 | 0.1636 |
| -NA | -0.91 | 1.0000 |
| +NA | -0.71 | 0.9698 |

Positive values show pairs of means that are significantly different.

# Oneway Analysis of Orotidine By Label

## Means Comparisons

### Comparisons with a control using Dunnett's Method

Control Group = -NA

| \|d\| | Alpha |
|---|---|
| 2.65691 | 0.05 |

| Level | Abs(Dif)-LSD | p-Value | |
|---|---|---|---|
| 50nM+NA | 3.675 | <.0001 | * |
| 50nM-NA | 2.557 | <.0001 | * |
| 5nM+NA | 2.474 | <.0001 | * |
| 5nM-NA | 2.024 | <.0001 | * |
| -NA | -0.35 | 1.0000 | |
| +NA | -0.34 | 1.0000 | |

Positive values show pairs of means that are significantly different.

# Oneway Analysis of Palmitic acid By Label

## Means Comparisons

### Comparisons with a control using Dunnett's Method

Control Group = -NA

| \|d\| | Alpha |
|---|---|
| 2.65691 | 0.05 |

| Level | Abs(Dif)-LSD | p-Value | |
|---|---|---|---|
| 50nM-NA | 0.166 | <.0001 | * |
| 5nM-NA | -0.09 | 0.9999 | |
| -NA | -0.1 | 1.0000 | |
| 5nM+NA | -4e-3 | 0.0643 | |
| 50nM+NA | 0.003 | 0.0417 | * |
| +NA | 0.004 | 0.0389 | * |

Positive values show pairs of means that are significantly different.

# Oneway Analysis of pantothenate By Label

## Means Comparisons

### Comparisons with a control using Dunnett's Method

Control Group = -NA

| \|d\| | Alpha |
|---|---|
| 2.65691 | 0.05 |

| Level | Abs(Dif)-LSD | p-Value | |
|---|---|---|---|
| 50nM-NA | -0.44 | 0.6731 | |
| -NA | -0.77 | 1.0000 | |
| 5nM-NA | -0.56 | 0.9247 | |
| 50nM+NA | -0.31 | 0.3748 | |
| +NA | 0.468 | 0.0008 | * |
| 5nM+NA | 0.485 | 0.0007 | * |

Positive values show pairs of means that are significantly different.

# Oneway Analysis of PC (13:0) By Label

## Means Comparisons

### Comparisons with a control using Dunnett's Method

Control Group = -NA

| \|d\| | Alpha |
|---|---|
| 2.65691 | 0.05 |

| Level | Abs(Dif)-LSD | p-Value | |
|---|---|---|---|
| 50nM+NA | 0.318 | 0.0004 | * |
| -NA | -0.45 | 1.0000 | |
| +NA | -0.37 | 0.9885 | |
| 50nM-NA | -0.35 | 0.9704 | |
| 5nM+NA | -0.3 | 0.8644 | |
| 5nM-NA | -0.09 | 0.1491 | |

Positive values show pairs of means that are significantly different.

# Oneway Analysis of PC (14:0) By Label

## Means Comparisons

### Comparisons with a control using Dunnett's Method

Control Group = -NA

| \|d\| | Alpha |
|---|---|
| 2.65691 | 0.05 |

| Level | Abs(Dif)-LSD | p-Value |
|---|---|---|
| -NA | -0.7 | 1.0000 |
| 50nM-NA | 0.443 | 0.0007 * |
| 50nM+NA | 0.514 | 0.0003 * |
| 5nM-NA | 0.66 | <.0001 * |
| +NA | 0.666 | <.0001 * |
| 5nM+NA | 0.785 | <.0001 * |

Positive values show pairs of means that are significantly different.

# Oneway Analysis of PC (15:1) By Label

## Means Comparisons

### Comparisons with a control using Dunnett's Method

Control Group = -NA

| \|d\| | Alpha |
|---|---|
| 2.65691 | 0.05 |

| Level | Abs(Dif)-LSD | p-Value | |
|---|---|---|---|
| 50nM-NA | -0.12 | 0.5329 | |
| -NA | -0.24 | 1.0000 | |
| 5nM-NA | -0.1 | 0.3684 | |
| 50nM+NA | 0.432 | <.0001 | * |
| +NA | 0.506 | <.0001 | * |
| 5nM+NA | 0.579 | <.0001 | * |

Positive values show pairs of means that are significantly different.

# Oneway Analysis of PC(15:0) By Label

## Means Comparisons

### Comparisons with a control using Dunnett's Method

Control Group = -NA

| \|d\| | Alpha |
|---|---|
| 2.65691 | 0.05 |

| Level | Abs(Dif)-LSD | p-Value | |
|---|---|---|---|
| 50nM+NA | -0.27 | 0.1017 | |
| -NA | -2.14 | 1.0000 | |
| 5nM+NA | -0.02 | 0.0524 | |
| 5nM-NA | 1.672 | 0.0002 | * |
| +NA | 2.698 | <.0001 | * |
| 50nM-NA | 2.969 | <.0001 | * |

Positive values show pairs of means that are significantly different.

# Oneway Analysis of PC(16:1) By Label

## Means Comparisons

### Comparisons with a control using Dunnett's Method

Control Group = -NA

| \|d\| | Alpha |
|---|---|
| 2.65691 | 0.05 |

| Level | Abs(Dif)-LSD | p-Value |
|---|---|---|
| 50nM+NA | 0.59 | 0.0005 * |
| -NA | -0.86 | 1.0000 |
| 5nM-NA | 0.093 | 0.0258 * |
| 5nM+NA | 0.153 | 0.0165 * |
| +NA | 0.158 | 0.0159 * |
| 50nM-NA | 0.18 | 0.0135 * |

Positive values show pairs of means that are significantly different.

# Oneway Analysis of PC(17:1) By Label

## Means Comparisons

### Comparisons with a control using Dunnett's Method

Control Group = -NA

| \|d\| | Alpha |
|---|---|
| 2.65691 | 0.05 |

| Level | Abs(Dif)-LSD | p-Value |
|---|---|---|
| -NA | -0.44 | 1.0000 |
| 50nM-NA | 0.267 | 0.0009 * |
| 5nM-NA | 0.314 | 0.0004 * |
| 50nM+NA | 0.526 | <.0001 * |
| 5nM+NA | 0.976 | <.0001 * |
| +NA | 1.212 | <.0001 * |

Positive values show pairs of means that are significantly different.

# Oneway Analysis of PC(17:2) By Label

## Means Comparisons

### Comparisons with a control using Dunnett's Method

Control Group = -NA

| \|d\| | Alpha |
|---|---|
| 2.65691 | 0.05 |

| Level | Abs(Dif)-LSD | p-Value |
|---|---|---|
| -NA | -0.2 | 1.0000 |
| 50nM-NA | -0.12 | 0.7661 |
| 5nM-NA | 0.022 | 0.0258 * |
| +NA | 0.196 | <.0001 * |
| 5nM+NA | 0.266 | <.0001 * |
| 50nM+NA | 0.354 | <.0001 * |

Positive values show pairs of means that are significantly different.

# Oneway Analysis of p-coumaric acid By Label

## Means Comparisons

### Comparisons with a control using Dunnett's Method

Control Group = -NA

| \|d\| | Alpha |
|---|---|
| 2.65691 | 0.05 |

| Level | Abs(Dif)-LSD | p-Value |
|---|---|---|
| 50nM+NA | -6e-4 | 0.0508 |
| 5nM-NA | -0.02 | 0.0903 |
| 5nM+NA | -0.13 | 0.7339 |
| 50nM-NA | -0.15 | 0.8602 |
| -NA | -0.22 | 1.0000 |
| +NA | -0.15 | 0.8694 |

Positive values show pairs of means that are significantly different.

# Oneway Analysis of Pentadecanal By Label

## Means Comparisons

### Comparisons with a control using Dunnett's Method

Control Group = -NA

| \|d\| | Alpha |
|---|---|
| 2.65691 | 0.05 |

| Level | Abs(Dif)-LSD | p-Value | |
|---|---|---|---|
| 5nM-NA | 0.705 | <.0001 | * |
| 50nM-NA | 0.569 | <.0001 | * |
| 5nM+NA | 0.483 | <.0001 | * |
| 50nM+NA | -0.18 | 0.2907 | |
| -NA | -0.52 | 1.0000 | |
| +NA | -0.52 | 1.0000 | |

Positive values show pairs of means that are significantly different.

# Oneway Analysis of phenylalanine By Label

## Means Comparisons

### Comparisons with a control using Dunnett's Method

Control Group = -NA

| \|d\| | Alpha |
|---|---|
| 2.65691 | 0.05 |

| Level | Abs(Dif)-LSD | p-Value | |
|---|---|---|---|
| 5nM-NA | 0.344 | 0.0017 | * |
| 5nM+NA | 0.075 | 0.0254 | * |
| 50nM-NA | 0.039 | 0.0352 | * |
| 50nM+NA | -0.13 | 0.1466 | |
| -NA | -0.68 | 1.0000 | |
| +NA | -0.29 | 0.4279 | |

Positive values show pairs of means that are significantly different.

# Oneway Analysis of Phenyllactic acid By Label

## Means Comparisons

### Comparisons with a control using Dunnett's Method

Control Group = -NA

| \|d\| | Alpha |
|---|---|
| 2.65691 | 0.05 |

| Level | Abs(Dif)-LSD | p-Value |
|---|---|---|
| 5nM-NA | -0.25 | 0.2381 |
| 5nM+NA | -0.31 | 0.3155 |
| 50nM-NA | -0.7 | 0.9821 |
| 50nM+NA | -0.75 | 0.9963 |
| -NA | -0.87 | 1.0000 |
| +NA | -0.11 | 0.1044 |

Positive values show pairs of means that are significantly different.

# Oneway Analysis of Phosphate By Label

## Means Comparisons

### Comparisons with a control using Dunnett's Method

Control Group = -NA

| \|d\| | Alpha |
|---|---|
| 2.65691 | 0.05 |

| Level | Abs(Dif)-LSD | p-Value | |
|---|---|---|---|
| 50nM+NA | 0.588 | <.0001 | * |
| 50nM-NA | 0.433 | 0.0002 | * |
| 5nM+NA | 0.194 | 0.0046 | * |
| +NA | -0.03 | 0.0699 | |
| 5nM-NA | -0.37 | 0.8942 | |
| -NA | -0.53 | 1.0000 | |

Positive values show pairs of means that are significantly different.

# Oneway Analysis of Phosphatidylglycerol By Label

## Means Comparisons

### Comparisons with a control using Dunnett's Method

Control Group = -NA

| \|d\| | Alpha |
|---|---|
| 2.65691 | 0.05 |

| Level | Abs(Dif)-LSD | p-Value | |
|---|---|---|---|
| 5nM+NA | 2.88 | <.0001 | * |
| +NA | 2.389 | <.0001 | * |
| 50nM+NA | -0.24 | 0.0864 | |
| 5nM-NA | -0.5 | 0.1464 | |
| -NA | -2.57 | 1.0000 | |
| 50nM-NA | -1.68 | 0.8249 | |

Positive values show pairs of means that are significantly different.

# Oneway Analysis of phosphoenolpyruvate By Label

## Means Comparisons

### Comparisons with a control using Dunnett's Method

Control Group = -NA

| \|d\| | Alpha |
|---|---|
| 2.65691 | 0.05 |

| Level | Abs(Dif)-LSD | p-Value |
|---|---|---|
| -NA | -0.93 | 1.0000 |
| 5nM-NA | -0.88 | 1.0000 |
| 5nM+NA | -0.82 | 0.9978 |
| 50nM-NA | -0.58 | 0.7671 |
| +NA | -0.3 | 0.2710 |
| 50nM+NA | -0.24 | 0.2036 |

Positive values show pairs of means that are significantly different.

# Oneway Analysis of Phosphorylcholine By Label

## Means Comparisons

### Comparisons with a control using Dunnett's Method

Control Group = -NA

| \|d\| | Alpha |
|---|---|
| 2.65691 | 0.05 |

| Level | Abs(Dif)-LSD | p-Value | |
|---|---|---|---|
| 5nM-NA | -0.54 | 0.8961 | |
| -NA | -0.77 | 1.0000 | |
| 50nM-NA | -0.65 | 0.9915 | |
| 5nM+NA | -0.42 | 0.6332 | |
| 50nM+NA | -0.34 | 0.4473 | |
| +NA | 0.234 | 0.0071 | * |

Positive values show pairs of means that are significantly different.

# Oneway Analysis of p-hydroxybenzoate By Label

## Means Comparisons

### Comparisons with a control using Dunnett's Method

Control Group = -NA

| \|d\| | Alpha |
|---|---|
| 2.65691 | 0.05 |

| Level | Abs(Dif)-LSD | p-Value | |
|---|---|---|---|
| 5nM-NA | 0.542 | 0.0002 | * |
| 5nM+NA | 0.425 | 0.0008 | * |
| 50nM-NA | 0.232 | 0.0057 | * |
| 50nM+NA | 0.074 | 0.0258 | * |
| -NA | -0.69 | 1.0000 | |
| +NA | -0.4 | 0.6948 | |

Positive values show pairs of means that are significantly different.

# Oneway Analysis of PI(16:0/20:2) By Label

## Means Comparisons

### Comparisons with a control using Dunnett's Method

Control Group = -NA

| \|d\| | Alpha |
|---|---|
| 2.65691 | 0.05 |

| Level | Abs(Dif)-LSD | p-Value | |
|---|---|---|---|
| 50nM-NA | 2.165 | <.0001 | * |
| 5nM-NA | -0.36 | 0.1718 | |
| 50nM+NA | -1.56 | 1.0000 | |
| -NA | -1.62 | 1.0000 | |
| +NA | 0.64 | 0.0038 | * |
| 5nM+NA | 1.111 | 0.0005 | * |

Positive values show pairs of means that are significantly different.

# Oneway Analysis of PI(16:0/22:4) By Label

## Means Comparisons

### Comparisons with a control using Dunnett's Method

Control Group = -NA

| \|d\| | Alpha |
|---|---|
| 2.65691 | 0.05 |

| Level | Abs(Dif)-LSD | p-Value | |
|---|---|---|---|
| 50nM-NA | 5.501 | <.0001 | * |
| 50nM+NA | 0.383 | 0.0248 | * |
| 5nM-NA | -0.3 | 0.0833 | |
| -NA | -3.36 | 1.0000 | |
| 5nM+NA | -0.46 | 0.1087 | |
| +NA | -0.15 | 0.0650 | |

Positive values show pairs of means that are significantly different.

# Oneway Analysis of PI(16:1/22:4) By Label

## Means Comparisons

### Comparisons with a control using Dunnett's Method

Control Group = -NA

| \|d\| | Alpha |
|---|---|
| 2.65691 | 0.05 |

| Level | Abs(Dif)-LSD | p-Value | |
|---|---|---|---|
| 50nM-NA | 5.697 | <.0001 | * |
| 50nM+NA | 1.501 | 0.0007 | * |
| 5nM-NA | 1.202 | 0.0017 | * |
| -NA | -2.36 | 1.0000 | |
| 5nM+NA | -0.37 | 0.1204 | |
| +NA | 0.232 | 0.0274 | * |

Positive values show pairs of means that are significantly different.

# Oneway Analysis of Piperidine By Label

## Means Comparisons

### Comparisons with a control using Dunnett's Method

Control Group = -NA

| \|d\| | Alpha |
|---|---|
| 2.65691 | 0.05 |

| Level | Abs(Dif)-LSD | p-Value | |
|---|---|---|---|
| 50nM+NA | 0.031 | 0.0208 | * |
| 50nM-NA | -0.07 | 0.2672 | |
| 5nM-NA | -0.15 | 0.8931 | |
| 5nM+NA | -0.21 | 1.0000 | |
| -NA | -0.22 | 1.0000 | |
| +NA | -0.21 | 0.9999 | |

Positive values show pairs of means that are significantly different.

# Oneway Analysis of proline By Label

## Means Comparisons

### Comparisons with a control using Dunnett's Method

Control Group = -NA

| \|d\| | Alpha |
|---|---|
| 2.65691 | 0.05 |

| Level | Abs(Dif)-LSD | p-Value | |
|---|---|---|---|
| 5nM-NA | 1.881 | <.0001 | * |
| 5nM+NA | 1.096 | <.0001 | * |
| 50nM+NA | 0.741 | <.0001 | * |
| 50nM-NA | 0.736 | <.0001 | * |
| -NA | -0.66 | 1.0000 | |
| +NA | -0.66 | 1.0000 | |

Positive values show pairs of means that are significantly different.

# Oneway Analysis of purine By Label

## Means Comparisons

### Comparisons with a control using Dunnett's Method

Control Group = -NA

| \|d\| | Alpha |
|---|---|
| 2.65691 | 0.05 |

| Level | Abs(Dif)-LSD | p-Value | |
|---|---|---|---|
| 5nM-NA | 0.361 | 0.0014 | * |
| 5nM+NA | 0.01 | 0.0457 | * |
| 50nM-NA | -0.06 | 0.0845 | |
| 50nM+NA | -0.37 | 0.6434 | |
| -NA | -0.68 | 1.0000 | |
| +NA | -0.23 | 0.2857 | |

Positive values show pairs of means that are significantly different.

# Oneway Analysis of pyridoxine By Label

## Means Comparisons

### Comparisons with a control using Dunnett's Method

Control Group = -NA

| \|d\| | Alpha |
|---|---|
| 2.65691 | 0.05 |

| Level | Abs(Dif)-LSD | p-Value |
|---|---|---|
| 5nM-NA | -0.87 | 0.9976 |
| -NA | -0.99 | 1.0000 |
| 5nM+NA | -0.81 | 0.9842 |
| 50nM-NA | -0.41 | 0.4108 |
| +NA | -0.31 | 0.2577 |
| 50nM+NA | -0.25 | 0.2026 |

Positive values show pairs of means that are significantly different.

# Oneway Analysis of Pyroglutamate By Label

## Means Comparisons

### Comparisons with a control using Dunnett's Method

Control Group = -NA

| \|d\| | Alpha |
|---|---|
| 2.65691 | 0.05 |

| Level | Abs(Dif)-LSD | p-Value |
|---|---|---|
| +NA | -4.3 | 0.3052 |
| 50nM+NA | -12.4 | 1.0000 |
| 50nM-NA | -12.4 | 1.0000 |
| 5nM-NA | -12.4 | 1.0000 |
| 5nM+NA | -12.4 | 1.0000 |
| -NA | -12.4 | 1.0000 |

Positive values show pairs of means that are significantly different.

# Oneway Analysis of pyruvate By Label

## Means Comparisons

### Comparisons with a control using Dunnett's Method

Control Group = -NA

| \|d\| | Alpha |
|---|---|
| 2.65691 | 0.05 |

| Level | Abs(Dif)-LSD | p-Value | |
|---|---|---|---|
| -NA | -1.06 | 1.0000 | |
| +NA | -0.59 | 0.6622 | |
| 5nM+NA | -0.39 | 0.3302 | |
| 5nM-NA | -0.15 | 0.1102 | |
| 50nM-NA | 0.705 | 0.0005 | * |
| 50nM+NA | 0.712 | 0.0005 | * |

Positive values show pairs of means that are significantly different.

# Oneway Analysis of riboflavin By Label

## Means Comparisons

### Comparisons with a control using Dunnett's Method

Control Group = -NA

| \|d\| | Alpha |
|---|---|
| 2.65691 | 0.05 |

| Level | Abs(Dif)-LSD | p-Value |
|---|---|---|
| 5nM-NA | -0.94 | 0.9999 |
| -NA | -1 | 1.0000 |
| 5nM+NA | -0.73 | 0.9237 |
| 50nM-NA | -0.36 | 0.3305 |
| +NA | -0.32 | 0.2732 |
| 50nM+NA | -0.3 | 0.2511 |

Positive values show pairs of means that are significantly different.

# Oneway Analysis of ribose-5-phosphate By Label

## Means Comparisons

### Comparisons with a control using Dunnett's Method

Control Group = -NA

| \|d\| | Alpha |
|---|---|
| 2.65691 | 0.05 |

| Level | Abs(Dif)-LSD | p-Value | |
|---|---|---|---|
| 50nM+NA | 2.526 | <.0001 | * |
| 50nM-NA | 1.191 | <.0001 | * |
| 5nM+NA | 0.61 | 0.0009 | * |
| 5nM-NA | 0.216 | 0.0134 | * |
| -NA | -1.03 | 1.0000 | |
| +NA | -0.94 | 0.9994 | |

Positive values show pairs of means that are significantly different.

# Oneway Analysis of S-adenosyl-L-homoCysteine By Label

## Means Comparisons

### Comparisons with a control using Dunnett's Method

Control Group = -NA

| \|d\| | Alpha |
|---|---|
| 2.65691 | 0.05 |

| Level | Abs(Dif)-LSD | p-Value |
|---|---|---|
| 5nM-NA | -0.28 | 0.4747 |
| 50nM+NA | -0.31 | 0.5325 |
| 50nM-NA | -0.31 | 0.5604 |
| -NA | -0.62 | 1.0000 |
| 5nM+NA | -0.43 | 0.8698 |
| +NA | 0.498 | 0.0002 * |

Positive values show pairs of means that are significantly different.

## Oneway Analysis of S-adenosyl-L-methioninamine By Label

### Means Comparisons

#### Comparisons with a control using Dunnett's Method

Control Group = -NA

| \|d\| | Alpha |
|---|---|
| 2.65691 | 0.05 |

| Level | Abs(Dif)-LSD | p-Value | |
|---|---|---|---|
| -NA | -1.23 | 1.0000 | |
| +NA | -0.36 | 0.2417 | |
| 5nM-NA | -0.33 | 0.2136 | |
| 50nM-NA | -0.23 | 0.1432 | |
| 5nM+NA | 0.669 | 0.0013 | * |
| 50nM+NA | 0.8 | 0.0006 | * |

Positive values show pairs of means that are significantly different.

# Oneway Analysis of S-adenosyl-L-methionine By Label

## Means Comparisons

### Comparisons with a control using Dunnett's Method

Control Group = -NA

| \|d\| | Alpha |
|---|---|
| 2.65691 | 0.05 |

| Level | Abs(Dif)-LSD | p-Value | |
|---|---|---|---|
| 50nM-NA | 2.672 | <.0001 | * |
| 50nM+NA | 2.46 | <.0001 | * |
| 5nM-NA | 1.703 | <.0001 | * |
| 5nM+NA | 1.453 | <.0001 | * |
| -NA | -0.47 | 1.0000 | |
| +NA | -0.39 | 0.9874 | |

Positive values show pairs of means that are significantly different.

# Oneway Analysis of sarcosine By Label

## Means Comparisons

### Comparisons with a control using Dunnett's Method

Control Group = -NA

| \|d\| | Alpha |
|---|---|
| 2.65691 | 0.05 |

| Level | Abs(Dif)-LSD | p-Value | |
|---|---|---|---|
| 50nM+NA | 1.617 | <.0001 | * |
| 50nM-NA | 1.427 | <.0001 | * |
| 5nM+NA | 0.798 | <.0001 | * |
| 5nM-NA | 0.433 | <.0001 | * |
| +NA | -0.07 | 0.1462 | |
| -NA | -0.36 | 1.0000 | |

Positive values show pairs of means that are significantly different.

# Oneway Analysis of Sedoheptulose 7-phosphate By Label

## Means Comparisons

### Comparisons with a control using Dunnett's Method

Control Group = -NA

| \|d\| | Alpha |
|---|---|
| 2.65691 | 0.05 |

| Level | Abs(Dif)-LSD | p-Value |
|---|---|---|
| 50nM+NA | 3.216 | <.0001 * |
| 50nM-NA | 1.799 | <.0001 * |
| 5nM+NA | 1.111 | <.0001 * |
| 5nM-NA | 0.567 | <.0001 * |
| +NA | -0.34 | 1.0000 |
| -NA | -0.36 | 1.0000 |

Positive values show pairs of means that are significantly different.

# Oneway Analysis of serine By Label

## Means Comparisons

### Comparisons with a control using Dunnett's Method

Control Group = -NA

| \|d\| | Alpha |
|---|---|
| 2.65691 | 0.05 |

| Level | Abs(Dif)-LSD | p-Value |
|---|---|---|
| 50nM+NA | -0.63 | 0.5867 |
| 50nM-NA | -0.9 | 0.9362 |
| +NA | -1.08 | 0.9986 |
| -NA | -1.21 | 1.0000 |
| 5nM+NA | -0.93 | 0.9548 |
| 5nM-NA | -0.91 | 0.9393 |

Positive values show pairs of means that are significantly different.

# Oneway Analysis of S-methyl-5-thioadenosine By Label

## Means Comparisons

### Comparisons with a control using Dunnett's Method

Control Group = -NA

| \|d\| | Alpha |
|---|---|
| 2.65691 | 0.05 |

| Level | Abs(Dif)-LSD | p-Value | |
|---|---|---|---|
| -NA | -0.92 | 1.0000 | |
| +NA | 1.04 | <.0001 | * |
| 5nM-NA | 2.944 | <.0001 | * |
| 50nM-NA | 4.748 | <.0001 | * |
| 5nM+NA | 4.993 | <.0001 | * |
| 50nM+NA | 5.329 | <.0001 | * |

Positive values show pairs of means that are significantly different.

# Oneway Analysis of sn-glycerol-3-phosphate By Label

## Means Comparisons

### Comparisons with a control using Dunnett's Method

Control Group = -NA

| \|d\| | Alpha |
|---|---|
| 2.65691 | 0.05 |

| Level | Abs(Dif)-LSD | p-Value | |
|---|---|---|---|
| 50nM-NA | 1.417 | <.0001 | * |
| 50nM+NA | 1.277 | <.0001 | * |
| 5nM+NA | -0.15 | 0.0988 | |
| 5nM-NA | -0.59 | 0.5130 | |
| -NA | -1.24 | 1.0000 | |
| +NA | -1.05 | 0.9928 | |

Positive values show pairs of means that are significantly different.

# Oneway Analysis of Spermidine By Label

## Means Comparisons

### Comparisons with a control using Dunnett's Method

Control Group = -NA

| \|d\| | Alpha |
|---|---|
| 2.65691 | 0.05 |

| Level | Abs(Dif)-LSD | p-Value | |
|---|---|---|---|
| 50nM-NA | 2.562 | <.0001 | * |
| 50nM+NA | 1.703 | <.0001 | * |
| 5nM-NA | 0.63 | <.0001 | * |
| 5nM+NA | 0.269 | 0.0007 | * |
| -NA | -0.43 | 1.0000 | |
| +NA | -0.35 | 0.9832 | |

Positive values show pairs of means that are significantly different.

# Oneway Analysis of Spermine By Label

## Means Comparisons

### Comparisons with a control using Dunnett's Method

Control Group = -NA

| \|d\| | Alpha |
|---|---|
| 2.65691 | 0.05 |

| Level | Abs(Dif)-LSD | p-Value |
|---|---|---|
| 50nM-NA | 0.094 | 0.0002 * |
| 50nM+NA | 0.005 | 0.0388 * |
| 5nM+NA | -0.04 | 0.3065 |
| +NA | -0.05 | 0.4470 |
| 5nM-NA | -0.05 | 0.5003 |
| -NA | -0.11 | 1.0000 |

Positive values show pairs of means that are significantly different.

# Oneway Analysis of Stearic acid By Label

## Means Comparisons

### Comparisons with a control using Dunnett's Method

Control Group = -NA

| \|d\| | Alpha |
|---|---|
| 2.65691 | 0.05 |

| Level | Abs(Dif)-LSD | p-Value | |
|---|---|---|---|
| 50nM-NA | 0.043 | 0.0068 | * |
| 5nM-NA | -0.05 | 0.3331 | |
| -NA | -0.14 | 1.0000 | |
| 5nM+NA | -0.06 | 0.4650 | |
| +NA | -0.05 | 0.3441 | |
| 50nM+NA | 0.007 | 0.0369 | * |

Positive values show pairs of means that are significantly different.

# Oneway Analysis of succinate By Label

## Means Comparisons

### Comparisons with a control using Dunnett's Method

Control Group = -NA

| \|d\| | Alpha |
|---|---|
| 2.65691 | 0.05 |

| Level | Abs(Dif)-LSD | p-Value |
|---|---|---|
| 5nM-NA | -0.58 | 0.4148 |
| 50nM+NA | -0.61 | 0.4492 |
| 5nM+NA | -0.83 | 0.7494 |
| 50nM-NA | -0.95 | 0.8782 |
| -NA | -1.37 | 1.0000 |
| +NA | -1.17 | 0.9948 |

Positive values show pairs of means that are significantly different.

# Oneway Analysis of succinyl-CoA By Label

## Means Comparisons

### Comparisons with a control using Dunnett's Method

Control Group = -NA

| \|d\| | Alpha |
|---|---|
| 2.65691 | 0.05 |

| Level | Abs(Dif)-LSD | p-Value | |
|---|---|---|---|
| 5nM+NA | -0.81 | 0.8498 | |
| +NA | -1.04 | 0.9951 | |
| -NA | -1.21 | 1.0000 | |
| 5nM-NA | -0.55 | 0.4651 | |
| 50nM-NA | -0.32 | 0.2087 | |
| 50nM+NA | 0.188 | 0.0190 | * |

Positive values show pairs of means that are significantly different.

# Oneway Analysis of taurine By Label

## Means Comparisons

### Comparisons with a control using Dunnett's Method

Control Group = -NA

| \|d\| | Alpha |
|---|---|
| 2.65691 | 0.05 |

| Level | Abs(Dif)-LSD | p-Value | |
|---|---|---|---|
| -NA | -0.73 | 1.0000 | |
| 50nM-NA | 0.027 | 0.0402 | * |
| +NA | 0.113 | 0.0193 | * |
| 5nM-NA | 0.314 | 0.0030 | * |
| 5nM+NA | 0.498 | 0.0005 | * |
| 50nM+NA | 0.572 | 0.0002 | * |

Positive values show pairs of means that are significantly different.

# Oneway Analysis of Taurodeoxycholic acid By Label

## Means Comparisons

### Comparisons with a control using Dunnett's Method

Control Group = -NA

| \|d\| | Alpha |
|---|---|
| 2.65691 | 0.05 |

| Level | Abs(Dif)-LSD | p-Value |
|---|---|---|
| 5nM-NA | -1.35 | 0.9999 |
| -NA | -1.44 | 1.0000 |
| 5nM+NA | -1.08 | 0.9424 |
| 50nM-NA | -1.02 | 0.8968 |
| 50nM+NA | -0.73 | 0.5642 |
| +NA | -0.58 | 0.3843 |

Positive values show pairs of means that are significantly different.

# Oneway Analysis of thiamine By Label

## Means Comparisons

### Comparisons with a control using Dunnett's Method

Control Group = -NA

| \|d\| | Alpha |
|---|---|
| 2.65691 | 0.05 |

| Level | Abs(Dif)-LSD | p-Value |
|---|---|---|
| 5nM-NA | -0.61 | 0.8450 |
| -NA | -0.92 | 1.0000 |
| 5nM+NA | -0.86 | 0.9999 |
| 50nM-NA | -0.66 | 0.9083 |
| 50nM+NA | -0.49 | 0.6042 |
| +NA | -0.27 | 0.2397 |

Positive values show pairs of means that are significantly different.

# Oneway Analysis of Thiamine pyrophosphate By Label

## Means Comparisons

### Comparisons with a control using Dunnett's Method

Control Group = -NA

| \|d\| | Alpha |
|---|---|
| 2.65691 | 0.05 |

| Level | Abs(Dif)-LSD | p-Value |
|---|---|---|
| -NA | -1.07 | 1.0000 |
| +NA | -8e-5 | 0.0500 |
| 5nM-NA | 0.004 | 0.0489 * |
| 50nM-NA | 0.091 | 0.0299 * |
| 5nM+NA | 0.208 | 0.0148 * |
| 50nM+NA | 1.053 | <.0001 * |

Positive values show pairs of means that are significantly different.

# Oneway Analysis of threonine By Label

## Means Comparisons

### Comparisons with a control using Dunnett's Method

Control Group = -NA

| \|d\| | Alpha |
|---|---|
| 2.65691 | 0.05 |

| Level | Abs(Dif)-LSD | p-Value | |
|---|---|---|---|
| 5nM-NA | 0.492 | 0.0001 | * |
| 50nM-NA | 0.423 | 0.0003 | * |
| 50nM+NA | 0.223 | 0.0039 | * |
| 5nM+NA | 0.183 | 0.0064 | * |
| -NA | -0.57 | 1.0000 | |
| +NA | -0.06 | 0.0873 | |

Positive values show pairs of means that are significantly different.

# Oneway Analysis of thymidine By Label

## Means Comparisons

### Comparisons with a control using Dunnett's Method

Control Group = -NA

| \|d\| | Alpha |
|---|---|
| 2.65691 | 0.05 |

| Level | Abs(Dif)-LSD | p-Value | |
|---|---|---|---|
| 50nM+NA | 2.088 | <.0001 | * |
| 50nM-NA | 1.765 | <.0001 | * |
| 5nM+NA | 0.565 | 0.0004 | * |
| 5nM-NA | 0.307 | 0.0043 | * |
| -NA | -0.81 | 1.0000 | |
| +NA | -0.79 | 1.0000 | |

Positive values show pairs of means that are significantly different.

# Oneway Analysis of tryptophan By Label

## Means Comparisons

### Comparisons with a control using Dunnett's Method

Control Group = -NA

| \|d\| | Alpha |
|---|---|
| 2.65691 | 0.05 |

| Level | Abs(Dif)-LSD | p-Value | |
|---|---|---|---|
| 5nM-NA | 0.367 | 0.0011 | * |
| 5nM+NA | 0.205 | 0.0064 | * |
| 50nM+NA | 0.042 | 0.0334 | * |
| 50nM-NA | 0.027 | 0.0388 | * |
| -NA | -0.64 | 1.0000 | |
| +NA | -0.22 | 0.2928 | |

Positive values show pairs of means that are significantly different.

# Oneway Analysis of tyrosine By Label

## Means Comparisons

### Comparisons with a control using Dunnett's Method

Control Group = -NA

| \|d\| | Alpha |
|---|---|
| 2.65691 | 0.05 |

| Level | Abs(Dif)-LSD | p-Value | |
|---|---|---|---|
| 5nM-NA | 0.293 | 0.0029 | * |
| 5nM+NA | 0.159 | 0.0114 | * |
| 50nM-NA | 0.028 | 0.0392 | * |
| 50nM+NA | -0.02 | 0.0604 | |
| -NA | -0.68 | 1.0000 | |
| +NA | -0.32 | 0.5084 | |

Positive values show pairs of means that are significantly different.

# Oneway Analysis of UDP By Label

## Means Comparisons

### Comparisons with a control using Dunnett's Method

Control Group = -NA

| \|d\| | Alpha |
|---|---|
| 2.65691 | 0.05 |

| Level | Abs(Dif)-LSD | p-Value | |
|---|---|---|---|
| 5nM-NA | 0.135 | 0.0213 | * |
| 5nM+NA | -0.05 | 0.0668 | |
| 50nM-NA | -0.22 | 0.1717 | |
| 50nM+NA | -0.86 | 0.9976 | |
| -NA | -0.98 | 1.0000 | |
| +NA | -0.11 | 0.0979 | |

Positive values show pairs of means that are significantly different.

# Oneway Analysis of UDP-D-glucose By Label

## Means Comparisons

### Comparisons with a control using Dunnett's Method

Control Group = -NA

| \|d\| | Alpha |
|---|---|
| 2.65691 | 0.05 |

| Level | Abs(Dif)-LSD | p-Value |
|---|---|---|
| 5nM+NA | 0.349 | 0.0014 * |
| 5nM-NA | 0.018 | 0.0422 * |
| +NA | -0.42 | 0.8119 |
| -NA | -0.65 | 1.0000 |
| 50nM+NA | 0.745 | <.0001 * |
| 50nM-NA | 0.804 | <.0001 * |

Positive values show pairs of means that are significantly different.

# Oneway Analysis of UDP-D-glucuronate By Label

## Means Comparisons

### Comparisons with a control using Dunnett's Method

Control Group = -NA

| \|d\| | Alpha |
|---|---|
| 2.65691 | 0.05 |

| Level | Abs(Dif)-LSD | p-Value |
|---|---|---|
| -NA | -0.64 | 1.0000 |
| +NA | -0.26 | 0.3805 |
| 5nM+NA | 1.861 | <.0001 * |
| 5nM-NA | 1.881 | <.0001 * |
| 50nM-NA | 2.478 | <.0001 * |
| 50nM+NA | 2.701 | <.0001 * |

Positive values show pairs of means that are significantly different.

# Oneway Analysis of UDP-N-acetyl-glucosamine By Label

## Means Comparisons

### Comparisons with a control using Dunnett's Method

Control Group = -NA

| \|d\| | Alpha |
|---|---|
| 2.65691 | 0.05 |

| Level | Abs(Dif)-LSD | p-Value | |
|---|---|---|---|
| 5nM+NA | -0.53 | 0.7429 | |
| 5nM-NA | -0.65 | 0.9456 | |
| -NA | -0.87 | 1.0000 | |
| +NA | -0.2 | 0.1798 | |
| 50nM-NA | 0.006 | 0.0480 | * |
| 50nM+NA | 0.4 | 0.0024 | * |

Positive values show pairs of means that are significantly different.

# Oneway Analysis of Urea By Label

## Means Comparisons

### Comparisons with a control using Dunnett's Method

Control Group = -NA

| \|d\| | Alpha |
|---|---|
| 2.65691 | 0.05 |

| Level | Abs(Dif)-LSD | p-Value |
|---|---|---|
| +NA | 0.014 | 0.0239 * |
| 5nM+NA | 5e-4 | 0.0487 * |
| -NA | -0.12 | 1.0000 |
| 5nM-NA | 0.064 | 0.0013 * |
| 50nM-NA | 0.064 | 0.0012 * |
| 50nM+NA | 0.064 | 0.0012 * |

Positive values show pairs of means that are significantly different.

# Oneway Analysis of Uric acid By Label

## Means Comparisons

### Comparisons with a control using Dunnett's Method

Control Group = -NA

| \|d\| | Alpha |
|---|---|
| 2.65691 | 0.05 |

| Level | Abs(Dif)-LSD | p-Value |
|---|---|---|
| 5nM-NA | -0.86 | 0.9935 |
| -NA | -1.01 | 1.0000 |
| 5nM+NA | -0.8 | 0.9705 |
| 50nM+NA | -0.62 | 0.7533 |
| 50nM-NA | -0.57 | 0.6585 |
| +NA | -0.49 | 0.5204 |

Positive values show pairs of means that are significantly different.

# Oneway Analysis of uridine By Label

## Means Comparisons

### Comparisons with a control using Dunnett's Method

Control Group = -NA

| \|d\| | Alpha |
|---|---|
| 2.65691 | 0.05 |

| Level | Abs(Dif)-LSD | p-Value | |
|---|---|---|---|
| 50nM+NA | 1.472 | <.0001 | * |
| 50nM-NA | 1.164 | 0.0001 | * |
| 5nM+NA | 0.453 | 0.0055 | * |
| 5nM-NA | -0.13 | 0.0886 | |
| +NA | -1.3 | 1.0000 | |
| -NA | -1.32 | 1.0000 | |

Positive values show pairs of means that are significantly different.

# Oneway Analysis of UTP By Label

## Means Comparisons

### Comparisons with a control using Dunnett's Method

Control Group = -NA

| \|d\| | Alpha |
|---|---|
| 2.65691 | 0.05 |

| Level | Abs(Dif)-LSD | p-Value | |
|---|---|---|---|
| 5nM-NA | -0.01 | 0.0528 | |
| -NA | -1.46 | 1.0000 | |
| 50nM-NA | -1.06 | 0.9213 | |
| +NA | -0.06 | 0.0629 | |
| 5nM+NA | 0.255 | 0.0168 | * |
| 50nM+NA | 1.08 | 0.0003 | * |

Positive values show pairs of means that are significantly different.

# Oneway Analysis of Valine By Label

## Means Comparisons

### Comparisons with a control using Dunnett's Method

Control Group = -NA

| \|d\| | Alpha |
|---|---|
| 2.65691 | 0.05 |

| Level | Abs(Dif)-LSD | p-Value | |
|---|---|---|---|
| 50nM-NA | 0.528 | <.0001 | * |
| 50nM+NA | 0.362 | <.0001 | * |
| 5nM-NA | 0.262 | <.0001 | * |
| 5nM+NA | 0.128 | 0.0005 | * |
| +NA | -0.17 | 0.9978 | |
| -NA | -0.19 | 1.0000 | |

Positive values show pairs of means that are significantly different.

# Oneway Analysis of xanthine By Label

## Means Comparisons

### Comparisons with a control using Dunnett's Method

Control Group = -NA

| \|d\| | Alpha |
|---|---|
| 2.65691 | 0.05 |

| Level | Abs(Dif)-LSD | p-Value |
|---|---|---|
| 5nM+NA | -0.52 | 0.9994 |
| 5nM-NA | -0.55 | 1.0000 |
| -NA | -0.57 | 1.0000 |
| 50nM+NA | -0.56 | 1.0000 |
| 50nM-NA | -0.5 | 0.9965 |
| +NA | -0.5 | 0.9957 |

Positive values show pairs of means that are significantly different.

# Oneway Analysis of Xanthurenic acid By Label

## Means Comparisons

### Comparisons with a control using Dunnett's Method

Control Group = -NA

| \|d\| | Alpha |
|---|---|
| 2.65691 | 0.05 |

| Level | Abs(Dif)-LSD | p-Value |
|---|---|---|
| 50nM-NA | -0.11 | 0.1048 |
| 5nM-NA | -0.21 | 0.2027 |
| 50nM+NA | -0.46 | 0.6562 |
| 5nM+NA | -0.52 | 0.7952 |
| -NA | -0.82 | 1.0000 |
| +NA | -3e-3 | 0.0509 |

Positive values show pairs of means that are significantly different.
