# Supplementary material for: Metabolomics Analysis of Metabolic Effects of Nicotinamide Phosphoribosyltransferase (NAMPT) Inhibition on Human Cancer Cells
Source: PLoS One. 2014 Dec 8;9(12):e114019. doi: 10.1371/journal.pone.0114019 (PMC4259317; doi:10.1371/journal.pone.0114019)
Supplement: S5 File — HCT-116 one-way analysis of variance results. (PDF) [file pone.0114019.s005.pdf]

Oneway Analysis of 1-Aminocyclopropanecarboxylic acid By Label

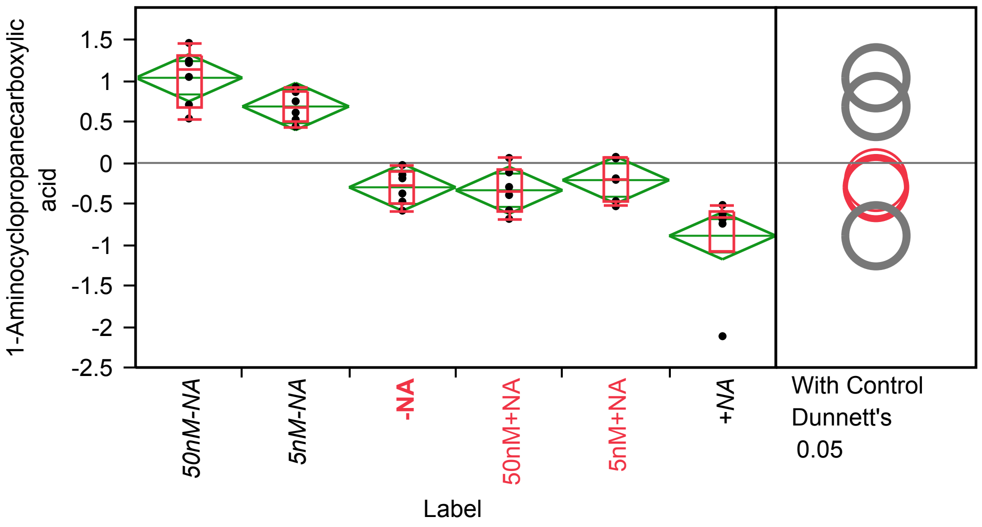

Quantiles

Level	Minimum	10%	25%	Median	75%	90%	Maximum
50nM-NA	0.543127	0.543127	0.669624	1.13685	1.303384	1.467074	1.467074
5nM-NA	0.445404	0.445404	0.509714	0.684544	0.882072	0.927216	0.927216
-NA	-0.58267	-0.58267	-0.49975	-0.28042	-0.11382	-0.02552	-0.02552
50nM+NA	-0.68367	-0.68367	-0.60502	-0.34282	-0.07115	0.061642	0.061642
5nM+NA	-0.52883	-0.52883	-0.48178	-0.19715	0.060677	0.074526	0.074526
+NA	-2.11749	-2.11749	-1.08608	-0.6728	-0.59371	-0.51377	-0.51377

Oneway Anova

Summary of Fit

Rsquare	0.813383
Adj Rsquare	0.782281
Root Mean Square Error	0.34507
Mean of Response	5.56e-10
Observations (or Sum Wgts)	36

Analysis of Variance

Source	DF	Sum of Squares	Mean Square	F Ratio	Prob > F
Label	5	15.569728	3.11395	26.1515	<.0001 *
Error	30	3.572202	0.11907		
C. Total	35	19.141930			

Means for Oneway Anova

Level	Number	Mean	Std Error	Lower 95%	Upper 95%
50nM-NA	6	1.0408	0.14087	0.753	1.328
5nM-NA	6	0.6900	0.14087	0.402	0.978
-NA	6	-0.2974	0.14087	-0.585	-0.0097
50nM+NA	6	-0.3336	0.14087	-0.621	-0.046
5nM+NA	6	-0.2098	0.14087	-0.497	0.078
+NA	6	-0.8899	0.14087	-1.178	-0.602

Std Error uses a pooled estimate of error variance

Means Comparisons

Comparisons with a control using Dunnett's Method

Oneway Analysis of 1-Hexadecyl-2-arachidonoyl-glycerol By Label

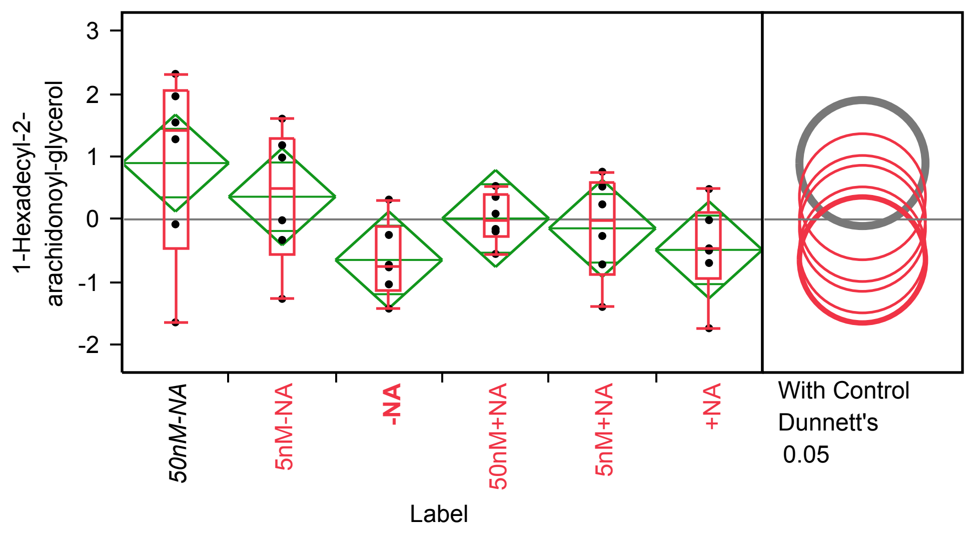

Quantiles

Level	Minimum	10%	25%	Median	75%	90%	Maximum
50nM-NA	-1.64164	-1.64164	-0.46902	1.41281	2.053175	2.320703	2.320703
5nM-NA	-1.26671	-1.26671	-0.56232	0.486967	1.28942	1.605995	1.605995
-NA	-1.42267	-1.42267	-1.13207	-0.74472	-0.1069	0.31564	0.31564
50nM+NA	-0.55025	-0.55025	-0.28134	-0.03091	0.403047	0.532881	0.532881
5nM+NA	-1.39542	-1.39542	-0.88714	-0.01221	0.582763	0.760084	0.760084
+NA	-1.73549	-1.73549	-0.95491	-0.47915	0.112521	0.48405	0.48405

Oneway Anova

Summary of Fit

Rsquare	0.273155
Adj Rsquare	0.152015
Root Mean Square Error	0.926391
Mean of Response	-5.6e-10
Observations (or Sum Wgts)	36

Analysis of Variance

Source	DF	Sum of Squares	Mean Square	F Ratio	Prob > F
Label	5	9.675599	1.93512	2.2549	0.0744
Error	30	25.746002	0.85820		
C. Total	35	35.421601			

Means for Oneway Anova

Level	Number	Mean	Std Error	Lower 95%	Upper 95%
50nM-NA	6	0.89842	0.37820	0.126	1.6708
5nM-NA	6	0.36160	0.37820	-0.411	1.1340
-NA	6	-0.64657	0.37820	-1.419	0.1258
50nM+NA	6	0.01481	0.37820	-0.758	0.7872
5nM+NA	6	-0.14230	0.37820	-0.915	0.6301
+NA	6	-0.48596	0.37820	-1.258	0.2864

Std Error uses a pooled estimate of error variance

Means Comparisons

Comparisons with a control using Dunnett's Method

Oneway Analysis of 1-Methyladenosine By Label

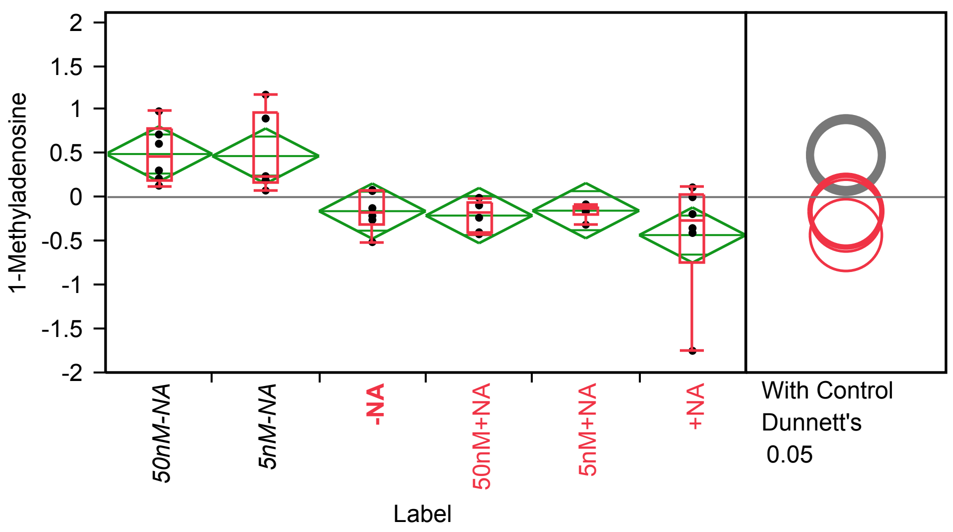

Quantiles

Level	Minimum	10%	25%	Median	75%	90%	Maximum
50nM-NA	0.130223	0.130223	0.19192	0.455732	0.780225	0.97827	0.97827
5nM-NA	0.074568	0.074568	0.162686	0.238575	0.963913	1.167352	1.167352
-NA	-0.51319	-0.51319	-0.32343	-0.16982	0.072525	0.081542	0.081542
50nM+NA	-0.42344	-0.42344	-0.40693	-0.16826	-0.07261	-0.00861	-0.00861
5nM+NA	-0.31491	-0.31491	-0.20366	-0.12418	-0.10767	-0.08328	-0.08328
+NA	-1.75251	-1.75251	-0.74486	-0.27371	0.026985	0.110772	0.110772

Oneway Anova

Summary of Fit

Rsquare	0.510193
Adj Rsquare	0.428558
Root Mean Square Error	0.377398
Mean of Response	2.78e-11
Observations (or Sum Wgts)	36

Analysis of Variance

Source	DF	Sum of Squares	Mean Square	F Ratio	Prob > F
Label	5	4.4507121	0.890142	6.2497	0.0004 *
Error	30	4.2728806	0.142429		
C. Total	35	8.7235928			

Means for Oneway Anova

Level	Number	Mean	Std Error	Lower 95%	Upper 95%
50nM-NA	6	0.49111	0.15407	0.1765	0.8058
5nM-NA	6	0.46787	0.15407	0.1532	0.7825
-NA	6	-0.16032	0.15407	-0.4750	0.1543
50nM+NA	6	-0.21066	0.15407	-0.5253	0.1040
5nM+NA	6	-0.15482	0.15407	-0.4695	0.1598
+NA	6	-0.43318	0.15407	-0.7478	-0.1185

Std Error uses a pooled estimate of error variance

Means Comparisons

Comparisons with a control using Dunnett's Method

Oneway Analysis of 1-Methyl-Histidine By Label

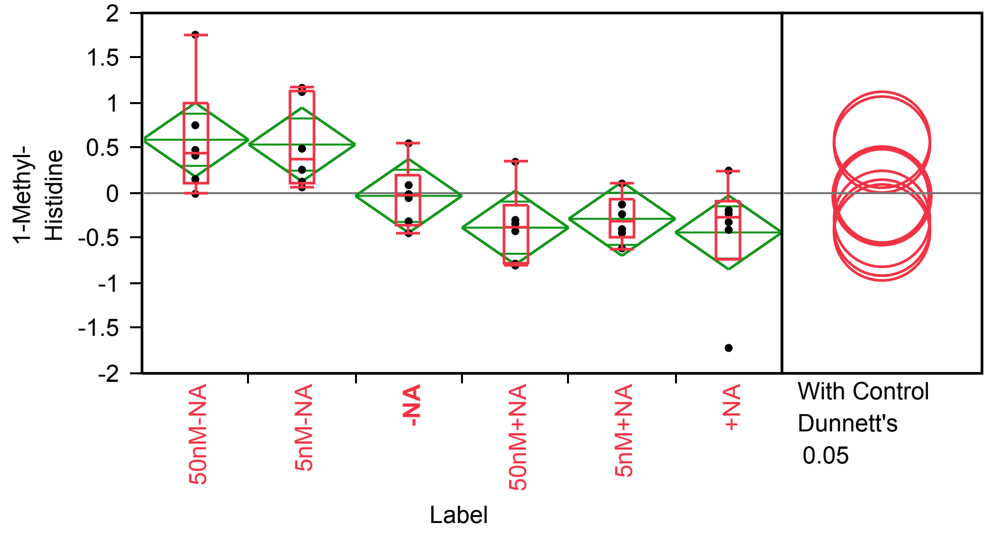

Quantiles

Level	Minimum	10%	25%	Median	75%	90%	Maximum
50nM-NA	-0.00727	-0.00727	0.114973	0.448367	1.005076	1.758836	1.758836
5nM-NA	0.064976	0.064976	0.111971	0.377074	1.134843	1.168954	1.168954
-NA	-0.44398	-0.44398	-0.34533	-0.03242	0.205235	0.553151	0.553151
50nM+NA	-0.80302	-0.80302	-0.78785	-0.38086	-0.13587	0.348058	0.348058
5nM+NA	-0.6126	-0.6126	-0.48398	-0.31629	-0.06798	0.106279	0.106279
+NA	-1.71824	-1.71824	-0.73483	-0.27552	-0.07949	0.250235	0.250235

Oneway Anova

Summary of Fit

Rsquare	0.467995
Adj Rsquare	0.379327
Root Mean Square Error	0.491226
Mean of Response	2.78e-11
Observations (or Sum Wgts)	36

Analysis of Variance

Source	DF	Sum of Squares	Mean Square	F Ratio	Prob > F
Label	5	6.368084	1.27362	5.2781	0.0014 *
Error	30	7.239080	0.24130		
C. Total	35	13.607164			

Means for Oneway Anova

Level	Number	Mean	Std Error	Lower 95%	Upper 95%
50nM-NA	6	0.59297	0.20054	0.1834	1.003
5nM-NA	6	0.53986	0.20054	0.1303	0.949
-NA	6	-0.02981	0.20054	-0.4394	0.380
50nM+NA	6	-0.38277	0.20054	-0.7923	0.027
5nM+NA	6	-0.28434	0.20054	-0.6939	0.125
+NA	6	-0.43591	0.20054	-0.8455	-0.026

Std Error uses a pooled estimate of error variance

Means Comparisons

Comparisons with a control using Dunnett's Method

Oneway Analysis of 2-Aminooctanoic acid By Label

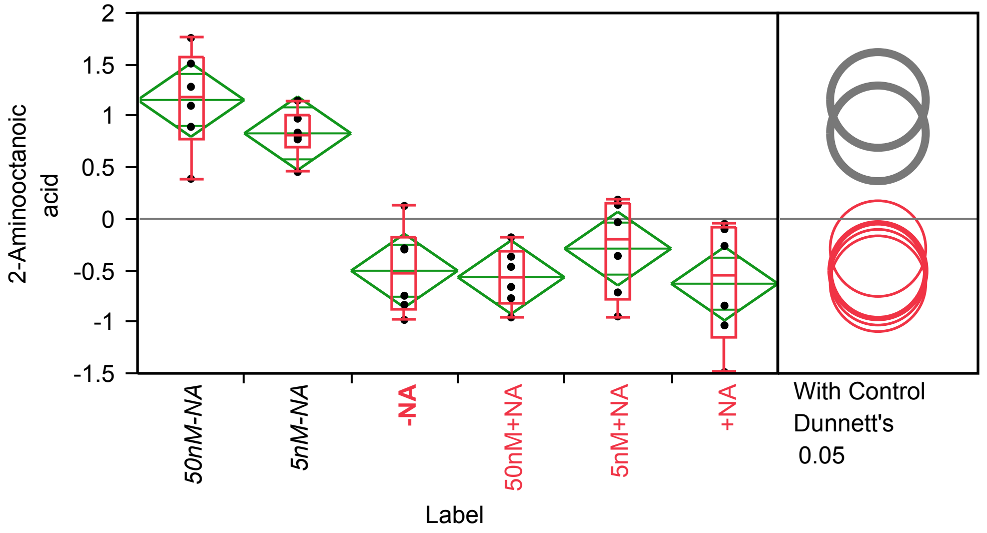

Quantiles

Level	Minimum	10%	25%	Median	75%	90%	Maximum
50nM-NA	0.389922	0.389922	0.768251	1.19119	1.571194	1.7574	1.7574
5nM-NA	0.455962	0.455962	0.693667	0.817574	1.017982	1.148849	1.148849
-NA	-0.98093	-0.98093	-0.87118	-0.52148	-0.18296	0.126262	0.126262
50nM+NA	-0.95671	-0.95671	-0.81703	-0.56297	-0.31961	-0.17901	-0.17901
5nM+NA	-0.94698	-0.94698	-0.77229	-0.19612	0.14944	0.18688	0.18688
+NA	-1.48788	-1.48788	-1.14701	-0.55232	-0.08698	-0.0483	-0.0483

Oneway Anova

Summary of Fit

Rsquare	0.770417
Adj Rsquare	0.732154
Root Mean Square Error	0.428363
Mean of Response	-3.7e-17
Observations (or Sum Wgts)	36

Analysis of Variance

Source	DF	Sum of Squares	Mean Square	F Ratio	Prob > F
Label	5	18.472769	3.69455	20.1344	<.0001 *
Error	30	5.504846	0.18349		
C. Total	35	23.977614			

Means for Oneway Anova

Level	Number	Mean	Std Error	Lower 95%	Upper 95%
50nM-NA	6	1.1555	0.17488	0.7984	1.513
5nM-NA	6	0.8312	0.17488	0.4741	1.188
-NA	6	-0.5030	0.17488	-0.8602	-0.146
50nM+NA	6	-0.5664	0.17488	-0.9236	-0.209
5nM+NA	6	-0.2882	0.17488	-0.6454	0.069
+NA	6	-0.6290	0.17488	-0.9862	-0.272

Std Error uses a pooled estimate of error variance

Means Comparisons

Comparisons with a control using Dunnett's Method

Oneway Analysis of 2-deoxyglucose-6-phosphate By Label

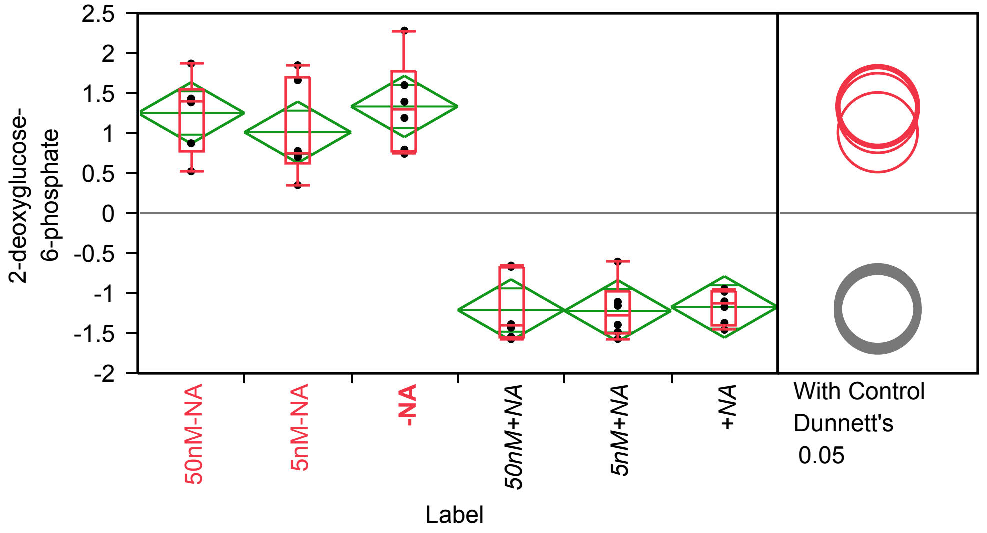

Quantiles

Level	Minimum	10%	25%	Median	75%	90%	Maximum
50nM-NA	0.524052	0.524052	0.786842	1.408044	1.542795	1.871559	1.871559
5nM-NA	0.351829	0.351829	0.614463	0.754141	1.709205	1.849596	1.849596
-NA	0.7432	0.7432	0.78168	1.292636	1.771999	2.283118	2.283118
50nM+NA	-1.57369	-1.57369	-1.55162	-1.40789	-0.6656	-0.65407	-0.65407
5nM+NA	-1.57143	-1.57143	-1.50599	-1.27478	-0.98125	-0.60508	-0.60508
+NA	-1.45642	-1.45642	-1.39239	-1.13659	-0.97198	-0.94566	-0.94566

Oneway Anova

Summary of Fit

Rsquare	0.891953
Adj Rsquare	0.873945
Root Mean Square Error	0.459061
Mean of Response	5.556e-9
Observations (or Sum Wgts)	36

Analysis of Variance

Source	DF	Sum of Squares	Mean Square	F Ratio	Prob > F
Label	5	52.190530	10.4381	49.5313	<.0001 *
Error	30	6.322123	0.2107		
C. Total	35	58.512654			

Means for Oneway Anova

Level	Number	Mean	Std Error	Lower 95%	Upper 95%
50nM-NA	6	1.2532	0.18741	0.870	1.636
5nM-NA	6	1.0124	0.18741	0.630	1.395
-NA	6	1.3346	0.18741	0.952	1.717
50nM+NA	6	-1.2095	0.18741	-1.592	-0.827
5nM+NA	6	-1.2195	0.18741	-1.602	-0.837
+NA	6	-1.1712	0.18741	-1.554	-0.788

Std Error uses a pooled estimate of error variance

Means Comparisons

Comparisons with a control using Dunnett's Method

Oneway Analysis of 2-Hydroxy-2-methylbutanedioic acid By Label

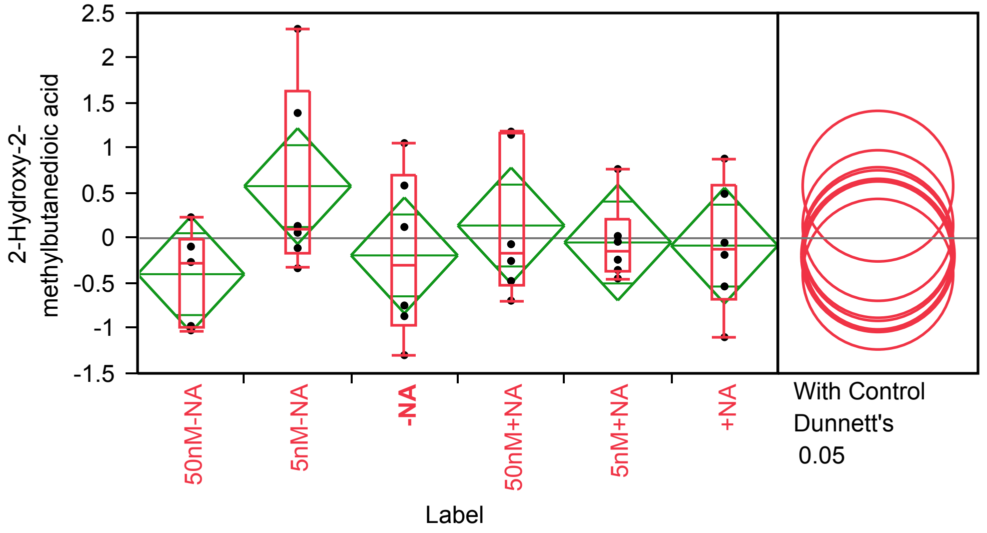

Quantiles

Level	Minimum	10%	25%	Median	75%	90%	Maximum
50nM-NA	-1.0226	-1.0226	-0.98635	-0.26869	-0.01156	0.232802	0.232802
5nM-NA	-0.33291	-0.33291	-0.16538	0.098472	1.623777	2.323914	2.323914
-NA	-1.29817	-1.29817	-0.9734	-0.31104	0.702719	1.055813	1.055813
50nM+NA	-0.69324	-0.69324	-0.52923	-0.15942	1.157137	1.183358	1.183358
5nM+NA	-0.44653	-0.44653	-0.37621	-0.13873	0.210597	0.767744	0.767744
+NA	-1.09835	-1.09835	-0.67584	-0.11696	0.590577	0.883569	0.883569

Oneway Anova

Summary of Fit

Rsquare	0.15839
Adj Rsquare	0.018121
Root Mean Square Error	0.770518
Mean of Response	2.78e-10
Observations (or Sum Wgts)	36

Analysis of Variance

Source	DF	Sum of Squares	Mean Square	F Ratio	Prob > F
Label	5	3.351988	0.670398	1.1292	0.3664
Error	30	17.810943	0.593698		
C. Total	35	21.162931			

Means for Oneway Anova

Level	Number	Mean	Std Error	Lower 95%	Upper 95%
50nM-NA	6	-0.39908	0.31456	-1.041	0.2433
5nM-NA	6	0.57814	0.31456	-0.064	1.2206
-NA	6	-0.19076	0.31456	-0.833	0.4517
50nM+NA	6	0.14085	0.31456	-0.502	0.7833
5nM+NA	6	-0.04736	0.31456	-0.690	0.5951
+NA	6	-0.08180	0.31456	-0.724	0.5606

Std Error uses a pooled estimate of error variance

Means Comparisons

Comparisons with a control using Dunnett's Method

Oneway Analysis of 2-hydroxyglutarate By Label

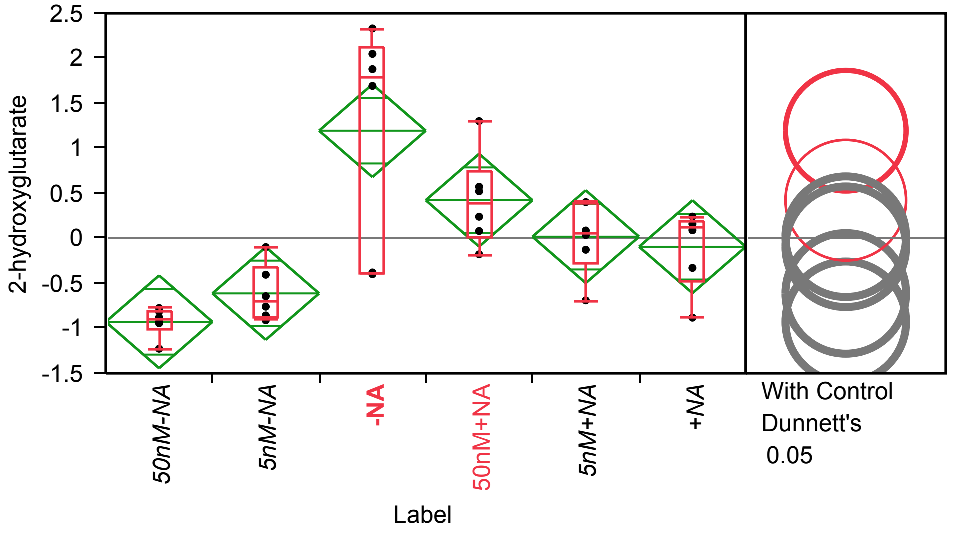

Quantiles

Level	Minimum	10%	25%	Median	75%	90%	Maximum
50nM-NA	-1.22727	-1.22727	-1.01465	-0.90553	-0.80427	-0.77513	-0.77513
5nM-NA	-0.91106	-0.91106	-0.87114	-0.70197	-0.32836	-0.09831	-0.09831
-NA	-0.3993	-0.3993	-0.38512	1.785905	2.119122	2.330938	2.330938
50nM+NA	-0.17829	-0.17829	0.015715	0.380797	0.754501	1.301383	1.301383
5nM+NA	-0.68977	-0.68977	-0.26806	0.060796	0.399448	0.401575	0.401575
+NA	-0.88213	-0.88213	-0.46763	0.122871	0.179554	0.240527	0.240527

Oneway Anova

Summary of Fit

Rsquare	0.599297
Adj Rsquare	0.532513
Root Mean Square Error	0.617806
Mean of Response	-5.6e-10
Observations (or Sum Wgts)	36

Analysis of Variance

Source	DF	Sum of Squares	Mean Square	F Ratio	Prob > F
Label	5	17.125542	3.42511	8.9737	<.0001 *
Error	30	11.450532	0.38168		
C. Total	35	28.576074			

Means for Oneway Anova

Level	Number	Mean	Std Error	Lower 95%	Upper 95%
50nM-NA	6	-0.9285	0.25222	-1.444	-0.413
5nM-NA	6	-0.6127	0.25222	-1.128	-0.098
-NA	6	1.1953	0.25222	0.680	1.710
50nM+NA	6	0.4229	0.25222	-0.092	0.938
5nM+NA	6	0.0174	0.25222	-0.498	0.533
+NA	6	-0.0943	0.25222	-0.609	0.421

Std Error uses a pooled estimate of error variance

Means Comparisons

Comparisons with a control using Dunnett's Method

Oneway Analysis of 2-Isopropylmalic acid By Label

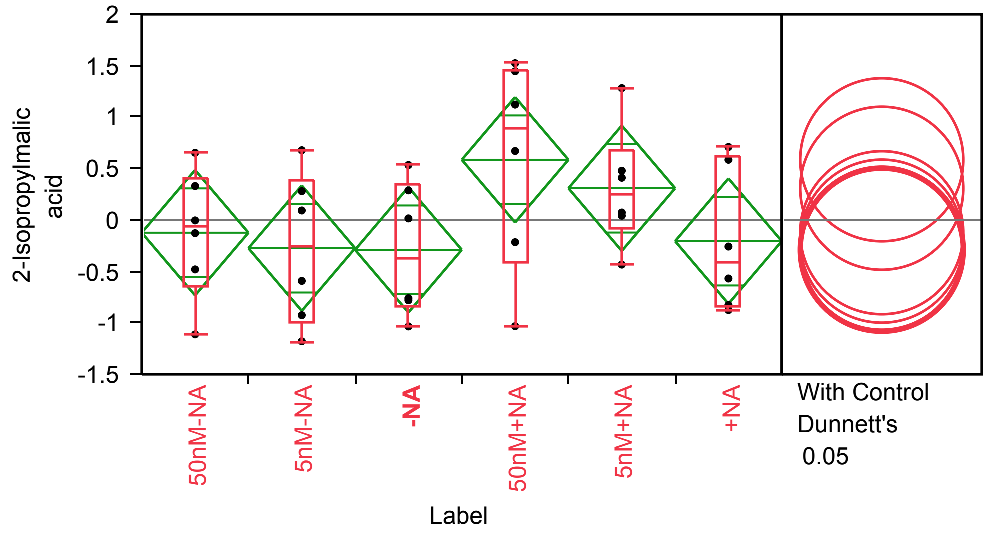

Quantiles

Level	Minimum	10%	25%	Median	75%	90%	Maximum
50nM-NA	-1.11205	-1.11205	-0.63848	-0.06638	0.410731	0.653001	0.653001
5nM-NA	-1.17917	-1.17917	-0.98911	-0.25008	0.379675	0.678017	0.678017
-NA	-1.03494	-1.03494	-0.84422	-0.37272	0.34939	0.534017	0.534017
50nM+NA	-1.03267	-1.03267	-0.42019	0.896064	1.4652	1.523924	1.523924
5nM+NA	-0.43367	-0.43367	-0.07735	0.241331	0.679523	1.281893	1.281893
+NA	-0.87586	-0.87586	-0.83917	-0.41371	0.616308	0.709369	0.709369

Oneway Anova

Summary of Fit

Rsquare	0.197029
Adj Rsquare	0.0632
Root Mean Square Error	0.730699
Mean of Response	2.78e-11
Observations (or Sum Wgts)	36

Analysis of Variance

Source	DF	Sum of Squares	Mean Square	F Ratio	Prob > F
Label	5	3.930320	0.786064	1.4722	0.2282
Error	30	16.017643	0.533921		
C. Total	35	19.947963			

Means for Oneway Anova

Level	Number	Mean	Std Error	Lower 95%	Upper 95%
50nM-NA	6	-0.12374	0.29831	-0.7330	0.4855
5nM-NA	6	-0.27447	0.29831	-0.8837	0.3348
-NA	6	-0.28986	0.29831	-0.8991	0.3194
50nM+NA	6	0.58550	0.29831	-0.0237	1.1947
5nM+NA	6	0.30851	0.29831	-0.3007	0.9177
+NA	6	-0.20593	0.29831	-0.8152	0.4033

Std Error uses a pooled estimate of error variance

Means Comparisons

Comparisons with a control using Dunnett's Method

Oneway Analysis of 2-keto-isovalerate By Label

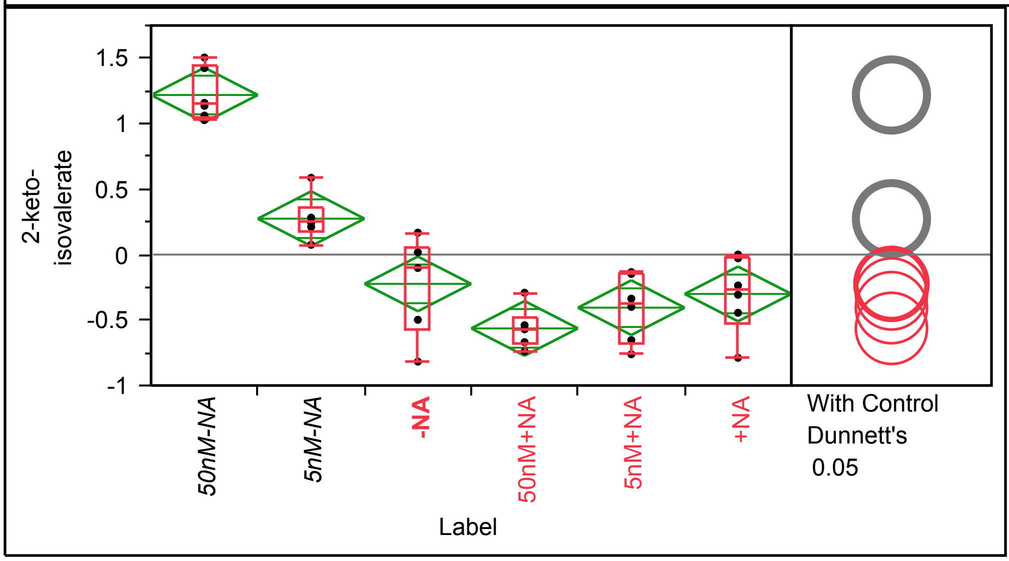

Quantiles

Level	Minimum	10%	25%	Median	75%	90%	Maximum
50nM-NA	1.027225	1.027225	1.053202	1.147742	1.44464	1.505459	1.505459
5nM-NA	0.07463	0.07463	0.177163	0.245615	0.358231	0.58668	0.58668
-NA	-0.81736	-0.81736	-0.57801	-0.10572	0.054879	0.167516	0.167516
50nM+NA	-0.73835	-0.73835	-0.68632	-0.57204	-0.4774	-0.29142	-0.29142
5nM+NA	-0.76126	-0.76126	-0.68031	-0.36812	-0.14433	-0.13358	-0.13358
+NA	-0.78852	-0.78852	-0.53047	-0.27127	-0.02127	0.000352	0.000352

Oneway Anova

Summary of Fit

Rsquare	0.874341
Adj Rsquare	0.853398
Root Mean Square Error	0.250545
Mean of Response	2.78e-11
Observations (or Sum Wgts)	36

Analysis of Variance

Source	DF	Sum of Squares	Mean Square	F Ratio	Prob > F
Label	5	13.103285	2.62066	41.7485	<.0001 *
Error	30	1.883177	0.06277		
C. Total	35	14.986461			

Means for Oneway Anova

Level	Number	Mean	Std Error	Lower 95%	Upper 95%
50nM-NA	6	1.2191	0.10228	1.010	1.428
5nM-NA	6	0.2743	0.10228	0.065	0.483
-NA	6	-0.2237	0.10228	-0.433	-0.015
50nM+NA	6	-0.5637	0.10228	-0.773	-0.355
5nM+NA	6	-0.4054	0.10228	-0.614	-0.196
+NA	6	-0.3006	0.10228	-0.509	-0.092

Std Error uses a pooled estimate of error variance

Means Comparisons

Comparisons with a control using Dunnett's Method

Oneway Analysis of 2-oxobutanoate By Label

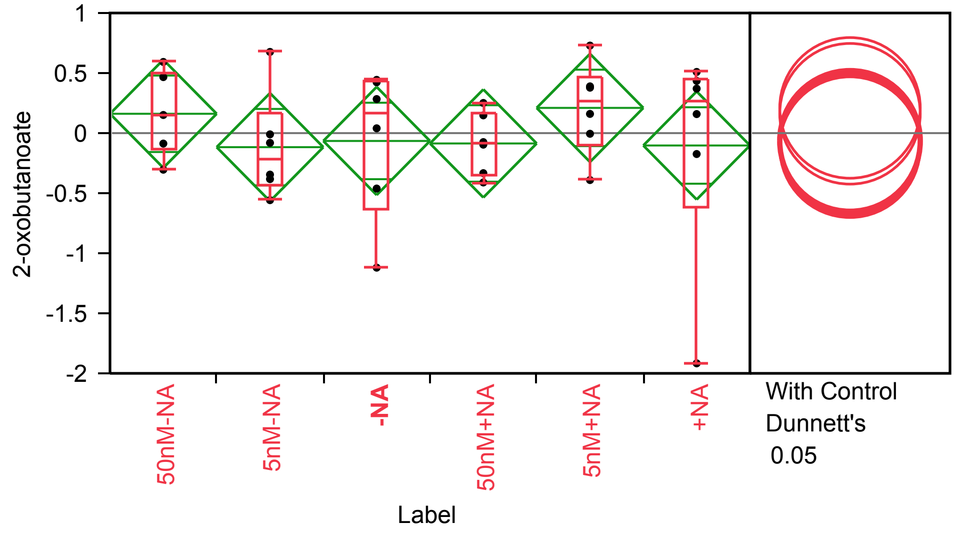

Quantiles

Level	Minimum	10%	25%	Median	75%	90%	Maximum
50nM-NA	-0.30433	-0.30433	-0.14141	0.149449	0.497914	0.592478	0.592478
5nM-NA	-0.558	-0.558	-0.42653	-0.21327	0.16127	0.67591	0.67591
-NA	-1.1199	-1.1199	-0.62608	0.161693	0.42829	0.443213	0.443213
50nM+NA	-0.41071	-0.41071	-0.35269	-0.08542	0.173724	0.250904	0.250904
5nM+NA	-0.39055	-0.39055	-0.10167	0.269167	0.474117	0.728516	0.728516
+NA	-1.9166	-1.9166	-0.60966	0.26453	0.453399	0.509255	0.509255

Oneway Anova

Summary of Fit

Rsquare	0.067814
Adj Rsquare	-0.08755
Root Mean Square Error	0.539766
Mean of Response	-6.2e-18
Observations (or Sum Wgts)	36

Analysis of Variance

Source	DF	Sum of Squares	Mean Square	F Ratio	Prob > F
Label	5	0.6358458	0.127169	0.4365	0.8195
Error	30	8.7404339	0.291348		
C. Total	35	9.3762797			

Means for Oneway Anova

Level	Number	Mean	Std Error	Lower 95%	Upper 95%
50nM-NA	6	0.16106	0.22036	-0.2890	0.61109
5nM-NA	6	-0.11693	0.22036	-0.5670	0.33310
-NA	6	-0.06524	0.22036	-0.5153	0.38479
50nM+NA	6	-0.08600	0.22036	-0.5360	0.36403
5nM+NA	6	0.21004	0.22036	-0.2400	0.66007
+NA	6	-0.10292	0.22036	-0.5530	0.34711

Std Error uses a pooled estimate of error variance

Means Comparisons

Comparisons with a control using Dunnett's Method

Oneway Analysis of 3-phospho-serine By Label

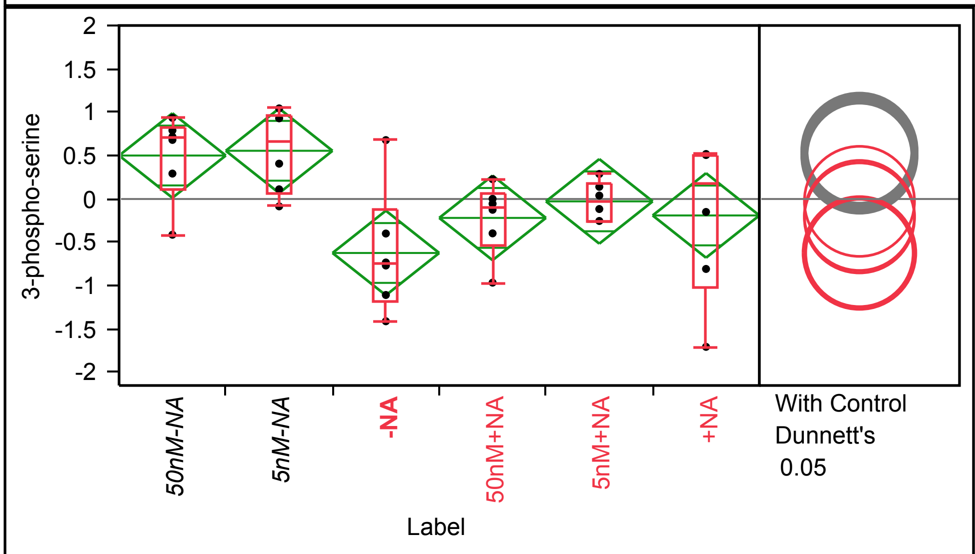

Quantiles

Level	Minimum	10%	25%	Median	75%	90%	Maximum
50nM-NA	-0.41291	-0.41291	0.117149	0.701611	0.829122	0.93808	0.93808
5nM-NA	-0.08484	-0.08484	0.062844	0.66861	0.961357	1.047269	1.047269
-NA	-1.41302	-1.41302	-1.18544	-0.75135	-0.12915	0.680662	0.680662
50nM+NA	-0.96661	-0.96661	-0.53948	-0.08942	0.058786	0.228976	0.228976
5nM+NA	-0.26358	-0.26358	-0.25634	-0.03812	0.177967	0.289527	0.289527
+NA	-1.70898	-1.70898	-1.03359	0.174139	0.511285	0.519995	0.519995

Oneway Anova

Summary of Fit

Rsquare	0.376354
Adj Rsquare	0.272413
Root Mean Square Error	0.586298
Mean of Response	-5.6e-11
Observations (or Sum Wgts)	36

Analysis of Variance

Source	DF	Sum of Squares	Mean Square	F Ratio	Prob > F
Label	5	6.223224	1.24464	3.6208	0.0111 *
Error	30	10.312348	0.34374		
C. Total	35	16.535572			

Means for Oneway Anova

Level	Number	Mean	Std Error	Lower 95%	Upper 95%
50nM-NA	6	0.50250	0.23936	0.014	0.991
5nM-NA	6	0.55741	0.23936	0.069	1.046
-NA	6	-0.62395	0.23936	-1.113	-0.135
50nM+NA	6	-0.21859	0.23936	-0.707	0.270
5nM+NA	6	-0.02724	0.23936	-0.516	0.462
+NA	6	-0.19013	0.23936	-0.679	0.299

Std Error uses a pooled estimate of error variance

Means Comparisons

Comparisons with a control using Dunnett's Method

Oneway Analysis of 4-aminobutyrate By Label

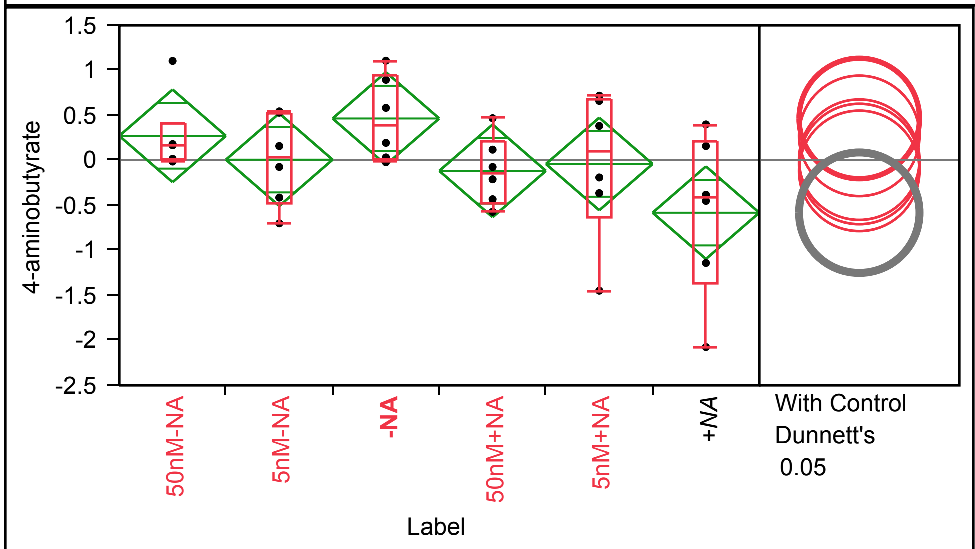

Quantiles

Level	Minimum	10%	25%	Median	75%	90%	Maximum
50nM-NA	-0.014	-0.014	0.008383	0.170562	0.410526	1.103667	1.103667
5nM-NA	-0.70091	-0.70091	-0.48645	0.041806	0.532044	0.543134	0.543134
-NA	-0.02191	-0.02191	0.018111	0.387709	0.946903	1.106217	1.106217
50nM+NA	-0.57263	-0.57263	-0.46792	-0.14319	0.205175	0.467237	0.467237
5nM+NA	-1.44935	-1.44935	-0.63642	0.095179	0.673889	0.717467	0.717467
+NA	-2.07536	-2.07536	-1.3749	-0.41746	0.217874	0.398193	0.398193

Oneway Anova

Summary of Fit

Rsquare	0.252781
Adj Rsquare	0.128244
Root Mean Square Error	0.617024
Mean of Response	-8.3e-10
Observations (or Sum Wgts)	36

Analysis of Variance

Source	DF	Sum of Squares	Mean Square	F Ratio	Prob > F
Label	5	3.863862	0.772772	2.0298	0.1028
Error	30	11.421562	0.380719		
C. Total	35	15.285424			

Means for Oneway Anova

Level	Number	Mean	Std Error	Lower 95%	Upper 95%
50nM-NA	6	0.27102	0.25190	-0.243	0.7855
5nM-NA	6	0.00654	0.25190	-0.508	0.5210
-NA	6	0.46416	0.25190	-0.050	0.9786
50nM+NA	6	-0.11783	0.25190	-0.632	0.3966
5nM+NA	6	-0.04127	0.25190	-0.556	0.4732
+NA	6	-0.58262	0.25190	-1.097	-0.0682

Std Error uses a pooled estimate of error variance

Means Comparisons

Comparisons with a control using Dunnett's Method

Oneway Analysis of 4-Aminomethylpyrimidine By Label

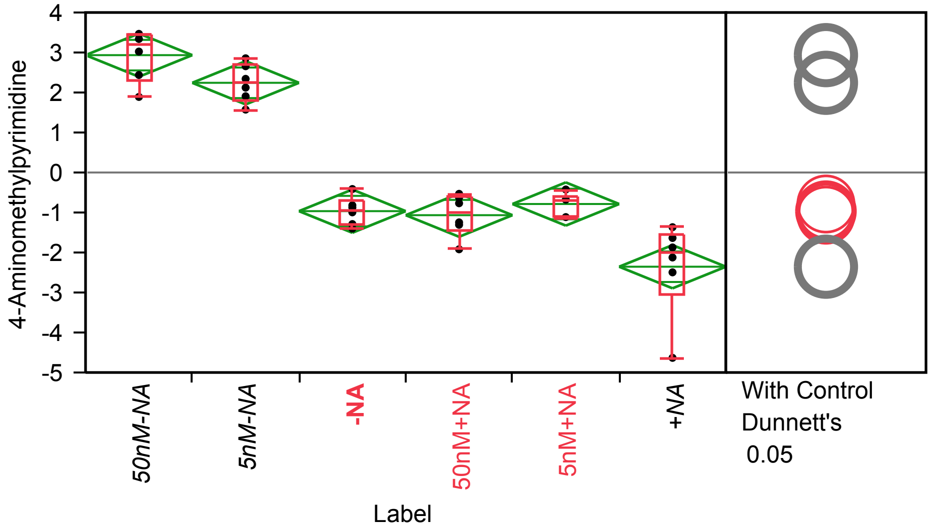

Quantiles

Level	Minimum	10%	25%	Median	75%	90%	Maximum
50nM-NA	1.88933	1.88933	2.300626	3.181083	3.452527	3.465959	3.465959
5nM-NA	1.572658	1.572658	1.822614	2.23159	2.706501	2.854819	2.854819
-NA	-1.40077	-1.40077	-1.3174	-0.9361	-0.71875	-0.41379	-0.41379
50nM+NA	-1.91928	-1.91928	-1.46013	-1.0078	-0.60314	-0.53361	-0.53361
5nM+NA	-1.13901	-1.13901	-1.12101	-0.68402	-0.61541	-0.42513	-0.42513
+NA	-4.63359	-4.63359	-3.03039	-1.99907	-1.56956	-1.36759	-1.36759

Oneway Anova

Summary of Fit

Rsquare	0.912502
Adj Rsquare	0.897919
Root Mean Square Error	0.647703
Mean of Response	2.778e-9
Observations (or Sum Wgts)	36

Analysis of Variance

Source	DF	Sum of Squares	Mean Square	F Ratio	Prob > F
Label	5	131.25256	26.2505	62.5729	<.0001 *
Error	30	12.58556	0.4195		
C. Total	35	143.83812			

Means for Oneway Anova

Level	Number	Mean	Std Error	Lower 95%	Upper 95%
50nM-NA	6	2.9339	0.26442	2.394	3.474
5nM-NA	6	2.2423	0.26442	1.702	2.782
-NA	6	-0.9661	0.26442	-1.506	-0.426
50nM+NA	6	-1.0670	0.26442	-1.607	-0.527
5nM+NA	6	-0.7877	0.26442	-1.328	-0.248
+NA	6	-2.3554	0.26442	-2.895	-1.815

Std Error uses a pooled estimate of error variance

Means Comparisons

Comparisons with a control using Dunnett's Method

Oneway Analysis of 4-Pyridoxic acid By Label

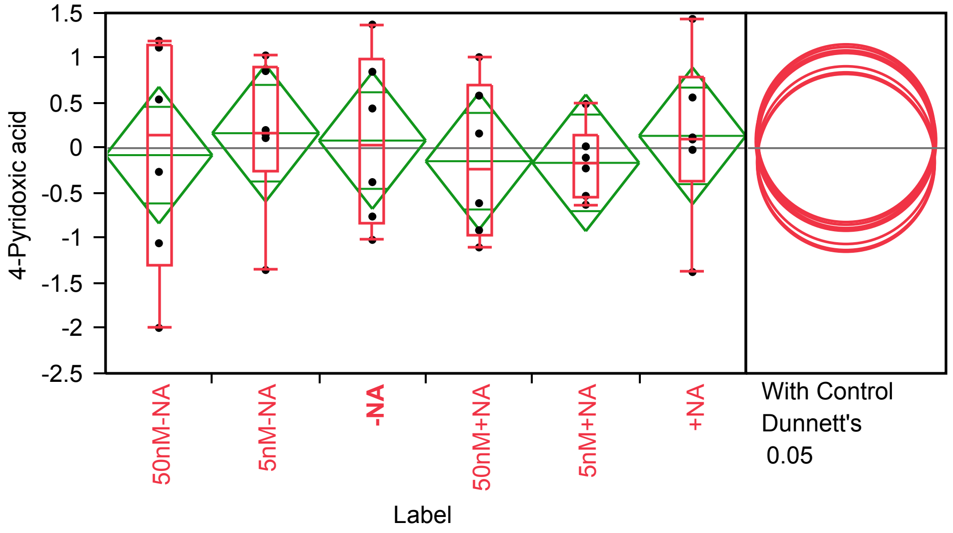

Quantiles

Level	Minimum	10%	25%	Median	75%	90%	Maximum
50nM-NA	-1.99557	-1.99557	-1.29075	0.138434	1.135262	1.191952	1.191952
5nM-NA	-1.35279	-1.35279	-0.25417	0.175735	0.89873	1.028714	1.028714
-NA	-1.01838	-1.01838	-0.82394	0.031405	0.978496	1.371441	1.371441
50nM+NA	-1.10215	-1.10215	-0.95932	-0.2243	0.689529	1.009565	1.009565
5nM+NA	-0.62872	-0.62872	-0.55476	-0.16522	0.13702	0.489375	0.489375
+NA	-1.37554	-1.37554	-0.35818	0.107579	0.780379	1.434624	1.434624

Oneway Anova

Summary of Fit

Rsquare	0.025204
Adj Rsquare	-0.13726
Root Mean Square Error	0.909238
Mean of Response	-2.8e-10
Observations (or Sum Wgts)	36

Analysis of Variance

Source	DF	Sum of Squares	Mean Square	F Ratio	Prob > F
Label	5	0.641265	0.128253	0.1551	0.9768
Error	30	24.801391	0.826713		
C. Total	35	25.442656			

Means for Oneway Anova

Level	Number	Mean	Std Error	Lower 95%	Upper 95%
50nM-NA	6	-0.07770	0.37119	-0.8358	0.68038
5nM-NA	6	0.16581	0.37119	-0.5923	0.92389
-NA	6	0.08404	0.37119	-0.6740	0.84212
50nM+NA	6	-0.14501	0.37119	-0.9031	0.61307
5nM+NA	6	-0.16339	0.37119	-0.9215	0.59469
+NA	6	0.13625	0.37119	-0.6218	0.89433

Std Error uses a pooled estimate of error variance

Means Comparisons

Comparisons with a control using Dunnett's Method

Oneway Analysis of 5,6-Dihydroxyindole-2-carboxylic acid By Label

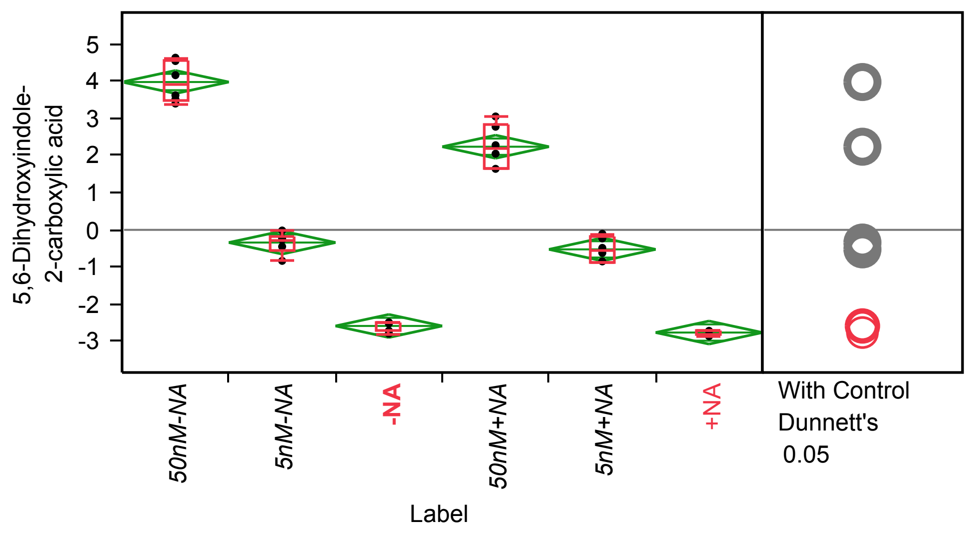

Quantiles

Level	Minimum	10%	25%	Median	75%	90%	Maximum
50nM-NA	3.395595	3.395595	3.48174	3.887874	4.561671	4.628571	4.628571
5nM-NA	-0.83323	-0.83323	-0.54497	-0.2639	-0.16862	-0.01755	-0.01755
-NA	-2.79268	-2.79268	-2.73209	-2.51375	-2.48571	-2.48508	-2.48508
50nM+NA	1.627311	1.627311	1.637509	2.161737	2.841799	3.048587	3.048587
5nM+NA	-0.85992	-0.85992	-0.85099	-0.55242	-0.19863	-0.10629	-0.10629
+NA	-2.85972	-2.85972	-2.83373	-2.72464	-2.71555	-2.71518	-2.71518

Oneway Anova

Summary of Fit

Rsquare	0.980859
Adj Rsquare	0.977669
Root Mean Square Error	0.37216
Mean of Response	1.111e-9
Observations (or Sum Wgts)	36

Analysis of Variance

Source	DF	Sum of Squares	Mean Square	F Ratio	Prob > F
Label	5	212.92750	42.5855	307.4703	<.0001 *
Error	30	4.15508	0.1385		
C. Total	35	217.08258			

Means for Oneway Anova

Level	Number	Mean	Std Error	Lower 95%	Upper 95%
50nM-NA	6	3.9750	0.15193	3.665	4.285
5nM-NA	6	-0.3411	0.15193	-0.651	-0.031
-NA	6	-2.5838	0.15193	-2.894	-2.274
50nM+NA	6	2.2355	0.15193	1.925	2.546
5nM+NA	6	-0.5247	0.15193	-0.835	-0.214
+NA	6	-2.7608	0.15193	-3.071	-2.451

Std Error uses a pooled estimate of error variance

Means Comparisons

Comparisons with a control using Dunnett's Method

Oneway Analysis of 7-methylguanosine By Label

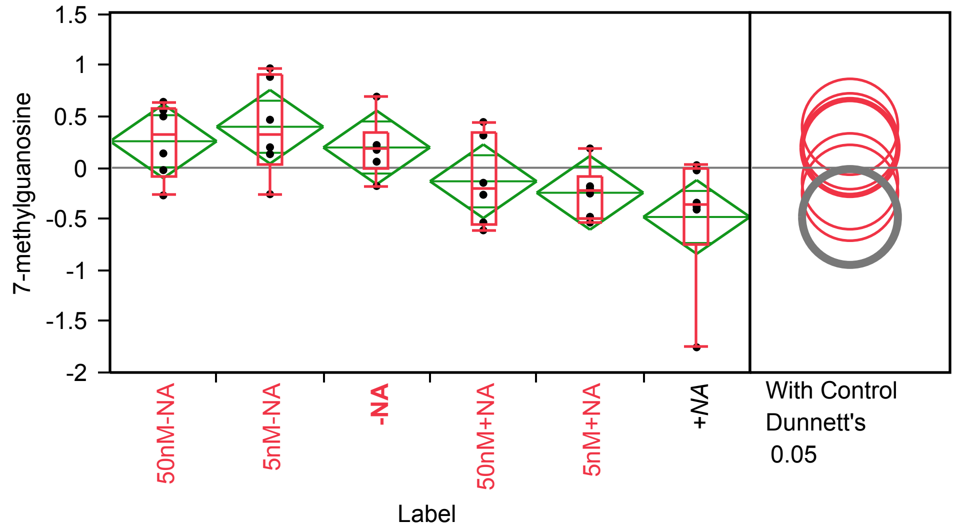

Quantiles

Level	Minimum	10%	25%	Median	75%	90%	Maximum
50nM-NA	-0.26986	-0.26986	-0.08464	0.320826	0.581712	0.643727	0.643727
5nM-NA	-0.25818	-0.25818	0.03516	0.334372	0.908546	0.971286	0.971286
-NA	-0.17823	-0.17823	-0.00013	0.195245	0.339979	0.694327	0.694327
50nM+NA	-0.61092	-0.61092	-0.55352	-0.20533	0.348565	0.446549	0.446549
5nM+NA	-0.5348	-0.5348	-0.49326	-0.2294	-0.08839	0.188426	0.188426
+NA	-1.75383	-1.75383	-0.74471	-0.36127	-0.01277	0.02509	0.02509

Oneway Anova

Summary of Fit

Rsquare	0.38166
Adj Rsquare	0.278603
Root Mean Square Error	0.431601
Mean of Response	-1.39e-9
Observations (or Sum Wgts)	36

Analysis of Variance

Source	DF	Sum of Squares	Mean Square	F Ratio	Prob > F
Label	5	3.4493391	0.689868	3.7034	0.0099 *
Error	30	5.5883854	0.186280		
C. Total	35	9.0377245			

Means for Oneway Anova

Level	Number	Mean	Std Error	Lower 95%	Upper 95%
50nM-NA	6	0.25894	0.17620	-0.1009	0.6188
5nM-NA	6	0.40040	0.17620	0.0406	0.7603
-NA	6	0.19795	0.17620	-0.1619	0.5578
50nM+NA	6	-0.13225	0.17620	-0.4921	0.2276
5nM+NA	6	-0.24421	0.17620	-0.6041	0.1156
+NA	6	-0.48084	0.17620	-0.8407	-0.1210

Std Error uses a pooled estimate of error variance

Means Comparisons

Comparisons with a control using Dunnett's Method

Oneway Analysis of acetoacetate By Label

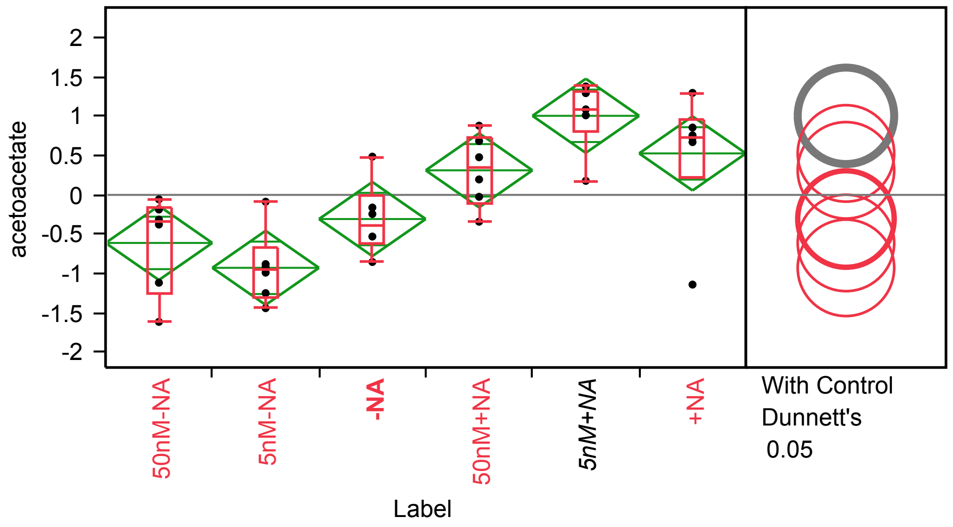

Quantiles

Level	Minimum	10%	25%	Median	75%	90%	Maximum
50nM-NA	-1.61459	-1.61459	-1.24132	-0.34584	-0.15361	-0.05611	-0.05611
5nM-NA	-1.44024	-1.44024	-1.29709	-0.95407	-0.68163	-0.08684	-0.08684
-NA	-0.85319	-0.85319	-0.61939	-0.3865	0.000867	0.486136	0.486136
50nM+NA	-0.341	-0.341	-0.1036	0.337675	0.733548	0.879972	0.879972
5nM+NA	0.178584	0.178584	0.80371	1.084422	1.315134	1.378987	1.378987
+NA	-1.14047	-1.14047	0.21823	0.740803	0.966333	1.296944	1.296944

Oneway Anova

Summary of Fit

Rsquare	0.629475
Adj Rsquare	0.567721
Root Mean Square Error	0.565282
Mean of Response	-5.6e-10
Observations (or Sum Wgts)	36

Analysis of Variance

Source	DF	Sum of Squares	Mean Square	F Ratio	Prob > F
Label	5	16.285981	3.25720	10.1933	<.0001 *
Error	30	9.586323	0.31954		
C. Total	35	25.872304			

Means for Oneway Anova

Level	Number	Mean	Std Error	Lower 95%	Upper 95%
50nM-NA	6	-0.6109	0.23078	-1.082	-0.140
5nM-NA	6	-0.9274	0.23078	-1.399	-0.456
-NA	6	-0.3071	0.23078	-0.778	0.164
50nM+NA	6	0.3124	0.23078	-0.159	0.784
5nM+NA	6	1.0054	0.23078	0.534	1.477
+NA	6	0.5276	0.23078	0.056	0.999

Std Error uses a pooled estimate of error variance

Means Comparisons

Comparisons with a control using Dunnett's Method

Oneway Analysis of acetoacetyl-CoA By Label

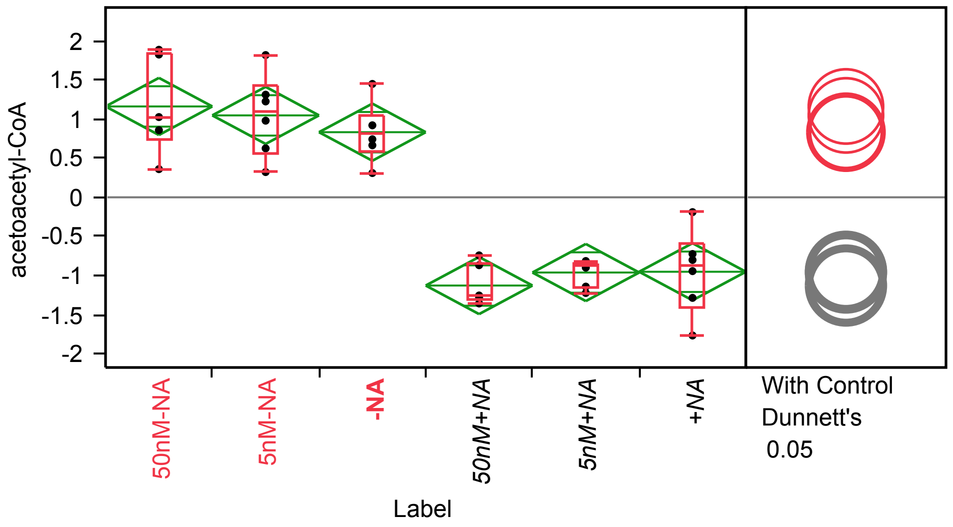

Quantiles

Level	Minimum	10%	25%	Median	75%	90%	Maximum
50nM-NA	0.35964	0.35964	0.732198	1.018396	1.837026	1.88239	1.88239
5nM-NA	0.322191	0.322191	0.549042	1.100699	1.435656	1.814986	1.814986
-NA	0.309725	0.309725	0.575431	0.823377	1.050834	1.44678	1.44678
50nM+NA	-1.3544	-1.3544	-1.30071	-1.25529	-0.83515	-0.74126	-0.74126
5nM+NA	-1.22087	-1.22087	-1.16025	-0.87206	-0.83388	-0.81338	-0.81338
+NA	-1.76335	-1.76335	-1.40339	-0.8707	-0.59004	-0.18922	-0.18922

Oneway Anova

Summary of Fit

Rsquare	0.866783
Adj Rsquare	0.844581
Root Mean Square Error	0.437247
Mean of Response	2.778e-9
Observations (or Sum Wgts)	36

Analysis of Variance

Source	DF	Sum of Squares	Mean Square	F Ratio	Prob > F
Label	5	37.318752	7.46375	39.0394	<.0001 *
Error	30	5.735551	0.19119		
C. Total	35	43.054303			

Means for Oneway Anova

Level	Number	Mean	Std Error	Lower 95%	Upper 95%
50nM-NA	6	1.1595	0.17851	0.795	1.524
5nM-NA	6	1.0454	0.17851	0.681	1.410
-NA	6	0.8310	0.17851	0.466	1.196
50nM+NA	6	-1.1259	0.17851	-1.490	-0.761
5nM+NA	6	-0.9599	0.17851	-1.324	-0.595
+NA	6	-0.9502	0.17851	-1.315	-0.586

Std Error uses a pooled estimate of error variance

Means Comparisons

Comparisons with a control using Dunnett's Method

Oneway Analysis of Acetylcarnitine By Label

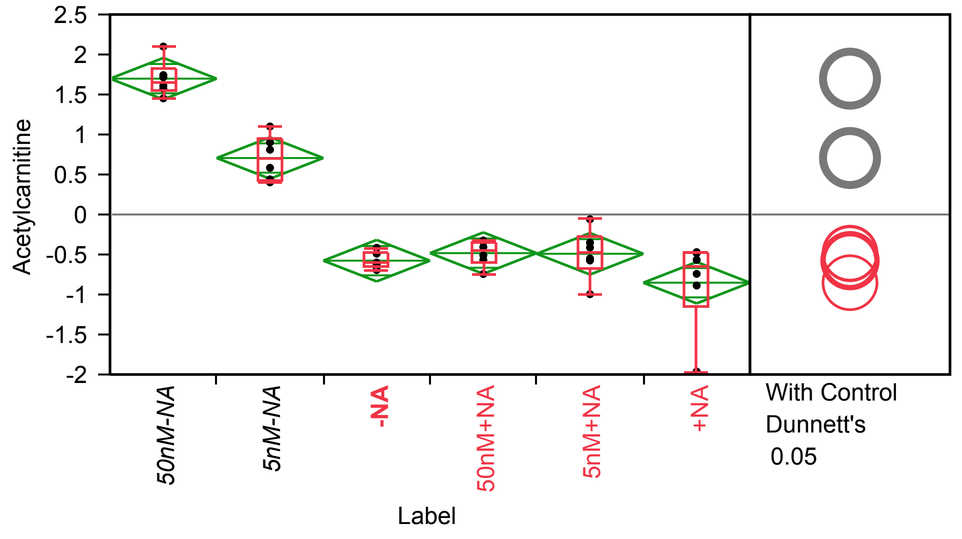

Quantiles

Level	Minimum	10%	25%	Median	75%	90%	Maximum
50nM-NA	1.451836	1.451836	1.541049	1.661973	1.831609	2.098868	2.098868
5nM-NA	0.402519	0.402519	0.429722	0.696512	0.950654	1.099695	1.099695
-NA	-0.69392	-0.69392	-0.65587	-0.61013	-0.4713	-0.41639	-0.41639
50nM+NA	-0.74395	-0.74395	-0.61214	-0.45785	-0.33919	-0.32534	-0.32534
5nM+NA	-0.99577	-0.99577	-0.68026	-0.48224	-0.2788	-0.05997	-0.05997
+NA	-1.96373	-1.96373	-1.15653	-0.65472	-0.48091	-0.47053	-0.47053

Oneway Anova

Summary of Fit

Rsquare	0.910552
Adj Rsquare	0.895644
Root Mean Square Error	0.310776
Mean of Response	2.78e-10
Observations (or Sum Wgts)	36

Analysis of Variance

Source	DF	Sum of Squares	Mean Square	F Ratio	Prob > F
Label	5	29.495197	5.89904	61.0782	<.0001 *
Error	30	2.897453	0.09658		
C. Total	35	32.392650			

Means for Oneway Anova

Level	Number	Mean	Std Error	Lower 95%	Upper 95%
50nM-NA	6	1.6980	0.12687	1.439	1.957
5nM-NA	6	0.7058	0.12687	0.447	0.965
-NA	6	-0.5772	0.12687	-0.836	-0.318
50nM+NA	6	-0.4828	0.12687	-0.742	-0.224
5nM+NA	6	-0.4912	0.12687	-0.750	-0.232
+NA	6	-0.8526	0.12687	-1.112	-0.593

Std Error uses a pooled estimate of error variance

Means Comparisons

Comparisons with a control using Dunnett's Method

Oneway Analysis of Acetylcholine By Label

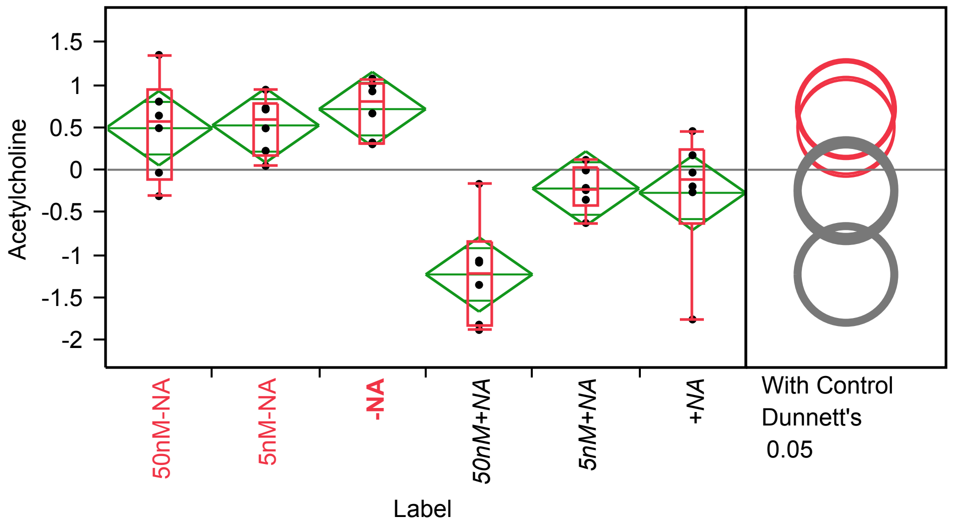

Quantiles

Level	Minimum	10%	25%	Median	75%	90%	Maximum
50nM-NA	-0.3102	-0.3102	-0.10393	0.563196	0.938756	1.350294	1.350294
5nM-NA	0.04521	0.04521	0.178399	0.598152	0.778601	0.940769	0.940769
-NA	0.299941	0.299941	0.31562	0.792813	1.01811	1.071842	1.071842
50nM+NA	-1.88394	-1.88394	-1.83779	-1.22277	-0.84325	-0.16672	-0.16672
5nM+NA	-0.62621	-0.62621	-0.4214	-0.2266	0.023143	0.115384	0.115384
+NA	-1.76028	-1.76028	-0.63825	-0.11466	0.241761	0.454025	0.454025

Oneway Anova

Summary of Fit

Rsquare	0.659372
Adj Rsquare	0.602601
Root Mean Square Error	0.524029
Mean of Response	5.56e-11
Observations (or Sum Wgts)	36

Analysis of Variance

Source	DF	Sum of Squares	Mean Square	F Ratio	Prob > F
Label	5	15.947136	3.18943	11.6145	<.0001 *
Error	30	8.238193	0.27461		
C. Total	35	24.185329			

Means for Oneway Anova

Level	Number	Mean	Std Error	Lower 95%	Upper 95%
50nM-NA	6	0.4888	0.21393	0.052	0.926
5nM-NA	6	0.5216	0.21393	0.085	0.959
-NA	6	0.7131	0.21393	0.276	1.150
50nM+NA	6	-1.2312	0.21393	-1.668	-0.794
5nM+NA	6	-0.2208	0.21393	-0.658	0.216
+NA	6	-0.2715	0.21393	-0.708	0.165

Std Error uses a pooled estimate of error variance

Means Comparisons

Comparisons with a control using Dunnett's Method

Oneway Analysis of acetyl-CoA By Label

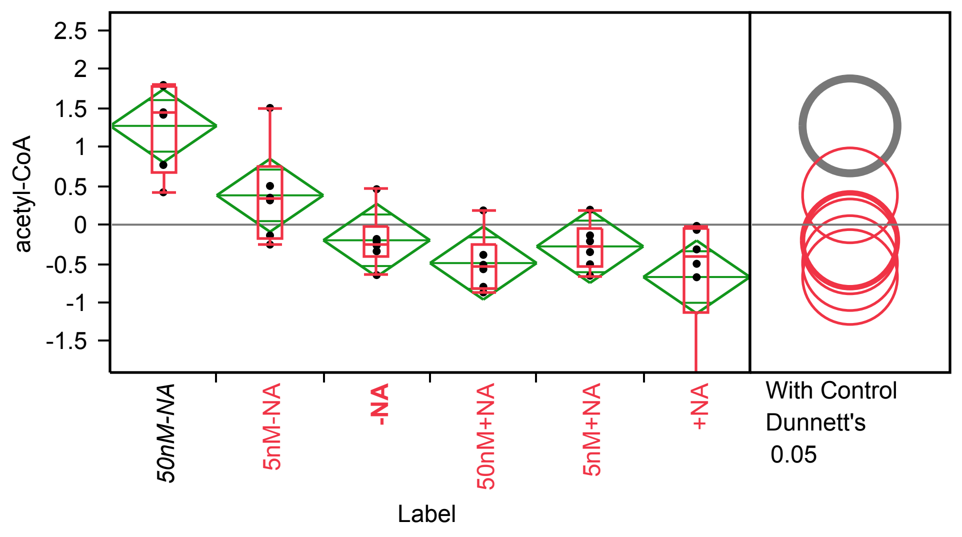

Quantiles

Level	Minimum	10%	25%	Median	75%	90%	Maximum
50nM-NA	0.416728	0.416728	0.68027	1.430849	1.787412	1.797572	1.797572
5nM-NA	-0.25641	-0.25641	-0.1682	0.329681	0.749143	1.50188	1.50188
-NA	-0.64873	-0.64873	-0.41886	-0.24303	-0.02405	0.456726	0.456726
50nM+NA	-0.87179	-0.87179	-0.81791	-0.54466	-0.24554	0.183348	0.183348
5nM+NA	-0.65709	-0.65709	-0.54653	-0.28323	-0.0554	0.191924	0.191924
+NA	-2.46177	-2.46177	-1.12272	-0.41068	-0.05408	-0.01626	-0.01626

Oneway Anova

Summary of Fit

Rsquare	0.619329
Adj Rsquare	0.555884
Root Mean Square Error	0.562702
Mean of Response	-5.6e-10
Observations (or Sum Wgts)	36

Analysis of Variance

Source	DF	Sum of Squares	Mean Square	F Ratio	Prob > F
Label	5	15.454311	3.09086	9.7616	<.0001 *
Error	30	9.499003	0.31663		
C. Total	35	24.953313			

Means for Oneway Anova

Level	Number	Mean	Std Error	Lower 95%	Upper 95%
50nM-NA	6	1.2714	0.22972	0.802	1.741
5nM-NA	6	0.3774	0.22972	-0.092	0.847
-NA	6	-0.2008	0.22972	-0.670	0.268
50nM+NA	6	-0.4944	0.22972	-0.964	-0.025
5nM+NA	6	-0.2799	0.22972	-0.749	0.189
+NA	6	-0.6737	0.22972	-1.143	-0.205

Std Error uses a pooled estimate of error variance

Means Comparisons

Comparisons with a control using Dunnett's Method

Oneway Analysis of Acetyllysine By Label

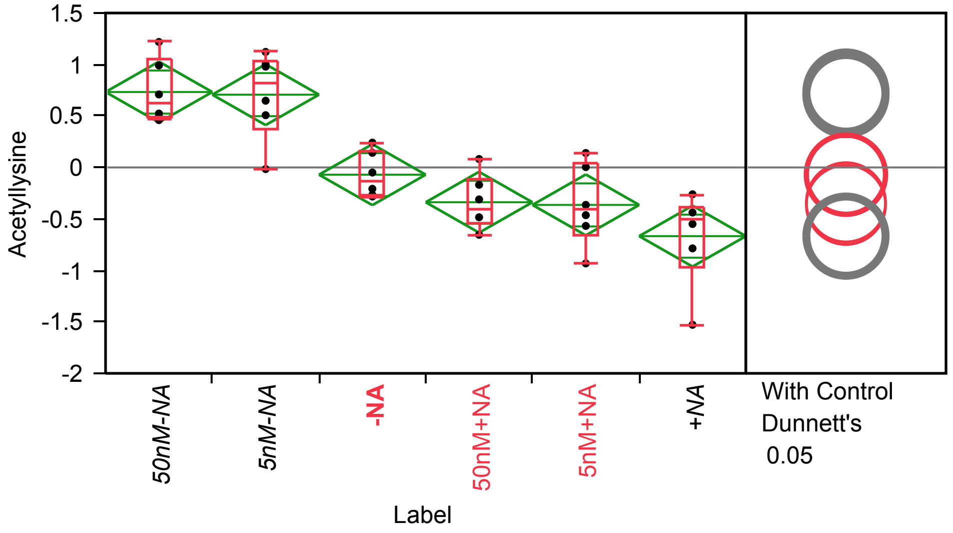

Quantiles

Level	Minimum	10%	25%	Median	75%	90%	Maximum
50nM-NA	0.459884	0.459884	0.485708	0.616287	1.04758	1.218643	1.218643
5nM-NA	-0.01554	-0.01554	0.37663	0.813423	1.031317	1.122135	1.122135
-NA	-0.28222	-0.28222	-0.27009	-0.12851	0.166814	0.240832	0.240832
50nM+NA	-0.65414	-0.65414	-0.5331	-0.39739	-0.1059	0.081717	0.081717
5nM+NA	-0.93204	-0.93204	-0.65901	-0.41368	0.03788	0.140133	0.140133
+NA	-1.52811	-1.52811	-0.97165	-0.49536	-0.39356	-0.26082	-0.26082

Oneway Anova

Summary of Fit

Rsquare	0.733793
Adj Rsquare	0.689426
Root Mean Square Error	0.354653
Mean of Response	-5.6e-11
Observations (or Sum Wgts)	36

Analysis of Variance

Source	DF	Sum of Squares	Mean Square	F Ratio	Prob > F
Label	5	10.401181	2.08024	16.5389	<.0001 *
Error	30	3.773357	0.12578		
C. Total	35	14.174538			

Means for Oneway Anova

Level	Number	Mean	Std Error	Lower 95%	Upper 95%
50nM-NA	6	0.73266	0.14479	0.4370	1.028
5nM-NA	6	0.70697	0.14479	0.4113	1.003
-NA	6	-0.07039	0.14479	-0.3661	0.225
50nM+NA	6	-0.33807	0.14479	-0.6338	-0.042
5nM+NA	6	-0.36391	0.14479	-0.6596	-0.068
+NA	6	-0.66727	0.14479	-0.9630	-0.372

Std Error uses a pooled estimate of error variance

Means Comparisons

Comparisons with a control using Dunnett's Method

Oneway Analysis of acetylphosphate By Label

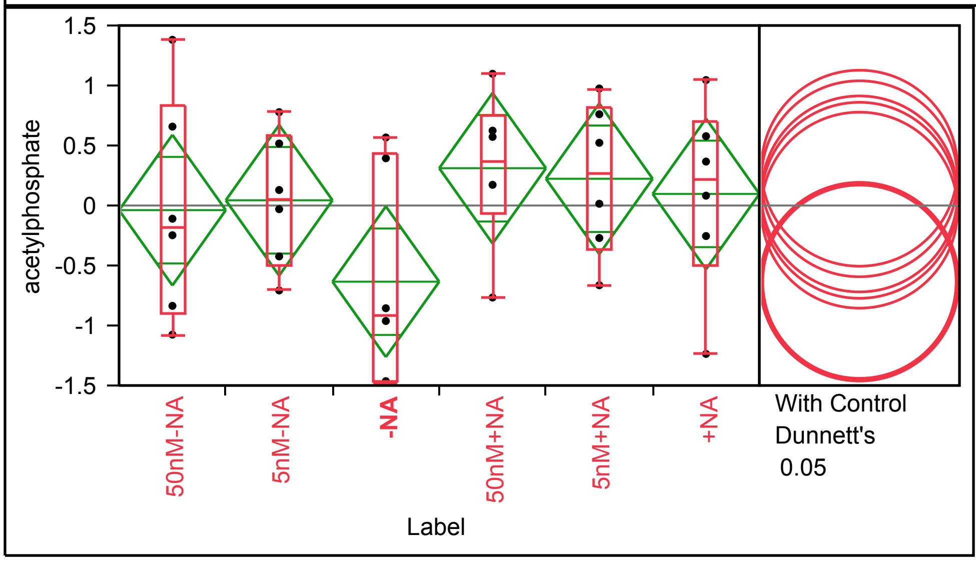

Quantiles

Level	Minimum	10%	25%	Median	75%	90%	Maximum
50nM-NA	-1.07595	-1.07595	-0.89634	-0.17899	0.838193	1.3807	1.3807
5nM-NA	-0.70761	-0.70761	-0.49512	0.049212	0.581712	0.777793	0.777793
-NA	-1.48693	-1.48693	-1.46957	-0.90934	0.436784	0.566676	0.566676
50nM+NA	-0.76671	-0.76671	-0.06682	0.371977	0.74202	1.097306	1.097306
5nM+NA	-0.66442	-0.66442	-0.36936	0.268538	0.814107	0.974837	0.974837
+NA	-1.23658	-1.23658	-0.50008	0.223894	0.69441	1.045154	1.045154

Oneway Anova

Summary of Fit

Rsquare	0.165534
Adj Rsquare	0.026457
Root Mean Square Error	0.75274
Mean of Response	2.78e-10
Observations (or Sum Wgts)	36

Analysis of Variance

Source	DF	Sum of Squares	Mean Square	F Ratio	Prob > F
Label	5	3.372034	0.674407	1.1902	0.3374
Error	30	16.998548	0.566618		
C. Total	35	20.370582			

Means for Oneway Anova

Level	Number	Mean	Std Error	Lower 95%	Upper 95%
50nM-NA	6	-0.03872	0.30731	-0.666	0.5889
5nM-NA	6	0.04344	0.30731	-0.584	0.6710
-NA	6	-0.63487	0.30731	-1.262	-0.0073
50nM+NA	6	0.31077	0.30731	-0.317	0.9384
5nM+NA	6	0.22283	0.30731	-0.405	0.8504
+NA	6	0.09655	0.30731	-0.531	0.7241

Std Error uses a pooled estimate of error variance

Means Comparisons

Comparisons with a control using Dunnett's Method

Oneway Analysis of adenine By Label

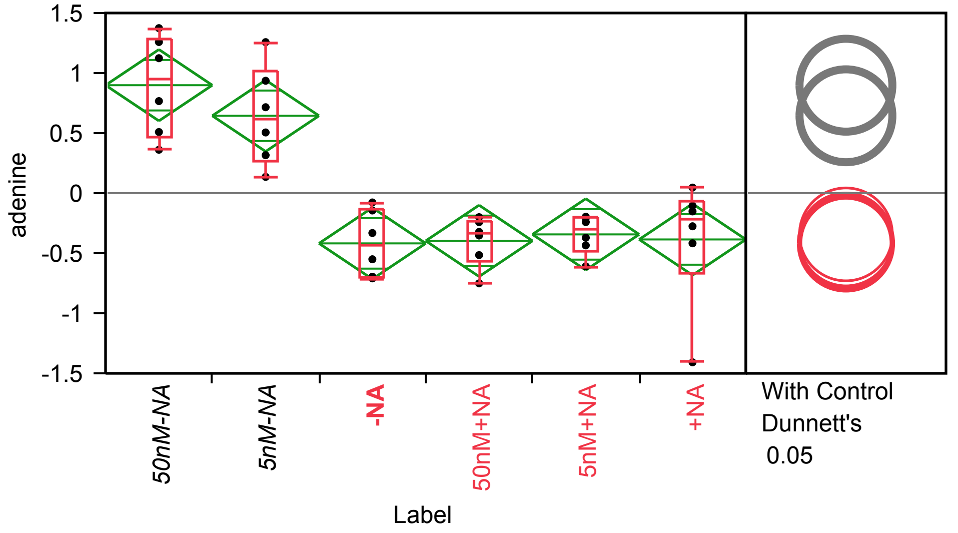

Quantiles

Level	Minimum	10%	25%	Median	75%	90%	Maximum
50nM-NA	0.361597	0.361597	0.472346	0.945151	1.287433	1.373934	1.373934
5nM-NA	0.13564	0.13564	0.271053	0.610561	1.016356	1.257448	1.257448
-NA	-0.70905	-0.70905	-0.6991	-0.4409	-0.12729	-0.07734	-0.07734
50nM+NA	-0.75067	-0.75067	-0.57429	-0.33745	-0.23082	-0.19972	-0.19972
5nM+NA	-0.60902	-0.60902	-0.47817	-0.3058	-0.20455	-0.19637	-0.19637
+NA	-1.40795	-1.40795	-0.66484	-0.21364	-0.06746	0.046383	0.046383

Oneway Anova

Summary of Fit

Rsquare	0.741453
Adj Rsquare	0.698361
Root Mean Square Error	0.356448
Mean of Response	-8.3e-10
Observations (or Sum Wgts)	36

Analysis of Variance

Source	DF	Sum of Squares	Mean Square	F Ratio	Prob > F
Label	5	10.930925	2.18619	17.2066	<.0001 *
Error	30	3.811655	0.12706		
C. Total	35	14.742581			

Means for Oneway Anova

Level	Number	Mean	Std Error	Lower 95%	Upper 95%
50nM-NA	6	0.89895	0.14552	0.6018	1.196
5nM-NA	6	0.64440	0.14552	0.3472	0.942
-NA	6	-0.41798	0.14552	-0.7152	-0.121
50nM+NA	6	-0.39699	0.14552	-0.6942	-0.100
5nM+NA	6	-0.34314	0.14552	-0.6403	-0.046
+NA	6	-0.38523	0.14552	-0.6824	-0.088

Std Error uses a pooled estimate of error variance

Means Comparisons

Comparisons with a control using Dunnett's Method

Oneway Analysis of adenosine By Label

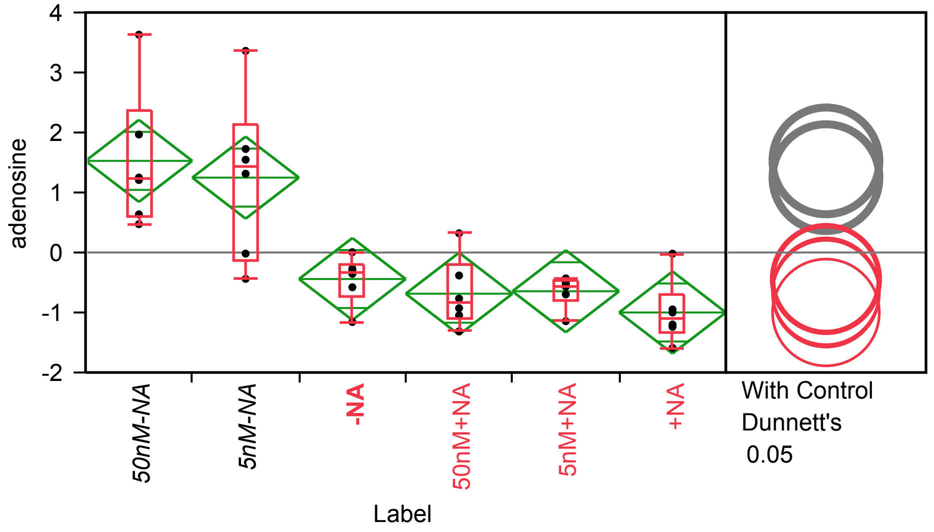

Quantiles

Level	Minimum	10%	25%	Median	75%	90%	Maximum
50nM-NA	0.472367	0.472367	0.594415	1.228634	2.382603	3.630351	3.630351
5nM-NA	-0.43933	-0.43933	-0.1238	1.42948	2.134335	3.360068	3.360068
-NA	-1.15801	-1.15801	-0.72393	-0.32635	-0.20023	0.005269	0.005269
50nM+NA	-1.31414	-1.31414	-1.11399	-0.84826	-0.20751	0.319997	0.319997
5nM+NA	-1.14456	-1.14456	-0.80986	-0.55236	-0.48007	-0.43202	-0.43202
+NA	-1.59074	-1.59074	-1.32689	-1.1001	-0.71635	-0.01982	-0.01982

Oneway Anova

Summary of Fit

Rsquare	0.639869
Adj Rsquare	0.579847
Root Mean Square Error	0.819943
Mean of Response	-8.3e-11
Observations (or Sum Wgts)	36

Analysis of Variance

Source	DF	Sum of Squares	Mean Square	F Ratio	Prob > F
Label	5	35.835981	7.16720	10.6606	<.0001 *
Error	30	20.169204	0.67231		
C. Total	35	56.005184			

Means for Oneway Anova

Level	Number	Mean	Std Error	Lower 95%	Upper 95%
50nM-NA	6	1.5270	0.33474	0.843	2.211
5nM-NA	6	1.2478	0.33474	0.564	1.931
-NA	6	-0.4422	0.33474	-1.126	0.241
50nM+NA	6	-0.6869	0.33474	-1.371	-0.0033
5nM+NA	6	-0.6459	0.33474	-1.330	0.038
+NA	6	-0.9997	0.33474	-1.683	-0.316

Std Error uses a pooled estimate of error variance

Means Comparisons

Comparisons with a control using Dunnett's Method

Oneway Analysis of Adipic acid By Label

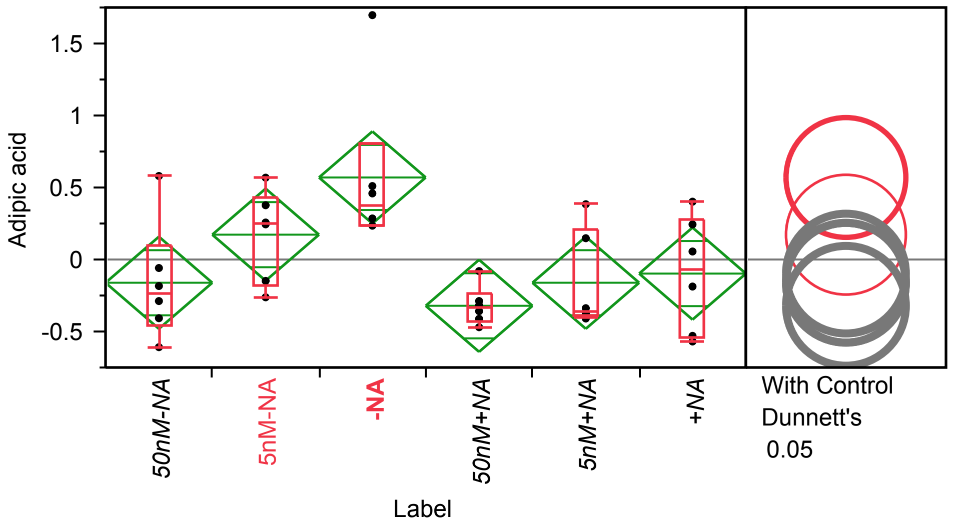

Quantiles

Level	Minimum	10%	25%	Median	75%	90%	Maximum
50nM-NA	-0.60882	-0.60882	-0.45793	-0.23641	0.100638	0.579838	0.579838
5nM-NA	-0.26246	-0.26246	-0.17719	0.250464	0.424652	0.568165	0.568165
-NA	0.232196	0.232196	0.234266	0.371969	0.806697	1.696972	1.696972
50nM+NA	-0.47017	-0.47017	-0.4264	-0.33894	-0.23656	-0.08065	-0.08065
5nM+NA	-0.40964	-0.40964	-0.38656	-0.35579	0.206856	0.383829	0.383829
+NA	-0.56928	-0.56928	-0.54011	-0.0661	0.283139	0.401461	0.401461

Oneway Anova

Summary of Fit

Rsquare	0.414028
Adj Rsquare	0.316366
Root Mean Square Error	0.383394
Mean of Response	-1.39e-9
Observations (or Sum Wgts)	36

Analysis of Variance

Source	DF	Sum of Squares	Mean Square	F Ratio	Prob > F
Label	5	3.1157594	0.623152	4.2394	0.0049 *
Error	30	4.4097181	0.146991		
C. Total	35	7.5254776			

Means for Oneway Anova

Level	Number	Mean	Std Error	Lower 95%	Upper 95%
50nM-NA	6	-0.16142	0.15652	-0.4811	0.1582
5nM-NA	6	0.17245	0.15652	-0.1472	0.4921
-NA	6	0.56967	0.15652	0.2500	0.8893
50nM+NA	6	-0.32151	0.15652	-0.6412	-0.0018
5nM+NA	6	-0.16140	0.15652	-0.4811	0.1583
+NA	6	-0.09779	0.15652	-0.4174	0.2219

Std Error uses a pooled estimate of error variance

Means Comparisons

Comparisons with a control using Dunnett's Method

Oneway Analysis of ADP By Label

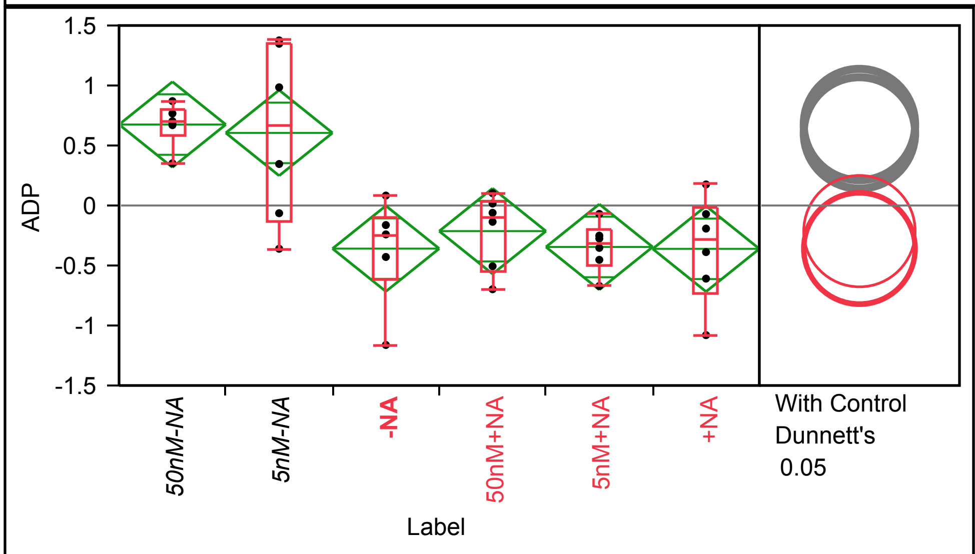

Quantiles

Level	Minimum	10%	25%	Median	75%	90%	Maximum
50nM-NA	0.351318	0.351318	0.590116	0.695331	0.792507	0.869728	0.869728
5nM-NA	-0.36045	-0.36045	-0.13828	0.665316	1.354778	1.375931	1.375931
-NA	-1.16213	-1.16213	-0.61218	-0.24205	-0.10165	0.082558	0.082558
50nM+NA	-0.69768	-0.69768	-0.55409	-0.09838	0.037821	0.102164	0.102164
5nM+NA	-0.67084	-0.67084	-0.50734	-0.31416	-0.206	-0.07013	-0.07013
+NA	-1.08028	-1.08028	-0.72714	-0.29038	-0.00981	0.17534	0.17534

Oneway Anova

Summary of Fit

Rsquare	0.576585
Adj Rsquare	0.506016
Root Mean Square Error	0.427736
Mean of Response	2.78e-10
Observations (or Sum Wgts)	36

Analysis of Variance

Source	DF	Sum of Squares	Mean Square	F Ratio	Prob > F
Label	5	7.474313	1.49486	8.1705	<.0001 *
Error	30	5.488750	0.18296		
C. Total	35	12.963063			

Means for Oneway Anova

Level	Number	Mean	Std Error	Lower 95%	Upper 95%
50nM-NA	6	0.67470	0.17462	0.3181	1.031
5nM-NA	6	0.60494	0.17462	0.2483	0.962
-NA	6	-0.35927	0.17462	-0.7159	-0.0026
50nM+NA	6	-0.21369	0.17462	-0.5703	0.143
5nM+NA	6	-0.34557	0.17462	-0.7022	0.011
+NA	6	-0.36111	0.17462	-0.7177	-0.0045

Std Error uses a pooled estimate of error variance

Means Comparisons

Comparisons with a control using Dunnett's Method

Oneway Analysis of AICAR 3,5-Cyclic Phosphate By Label

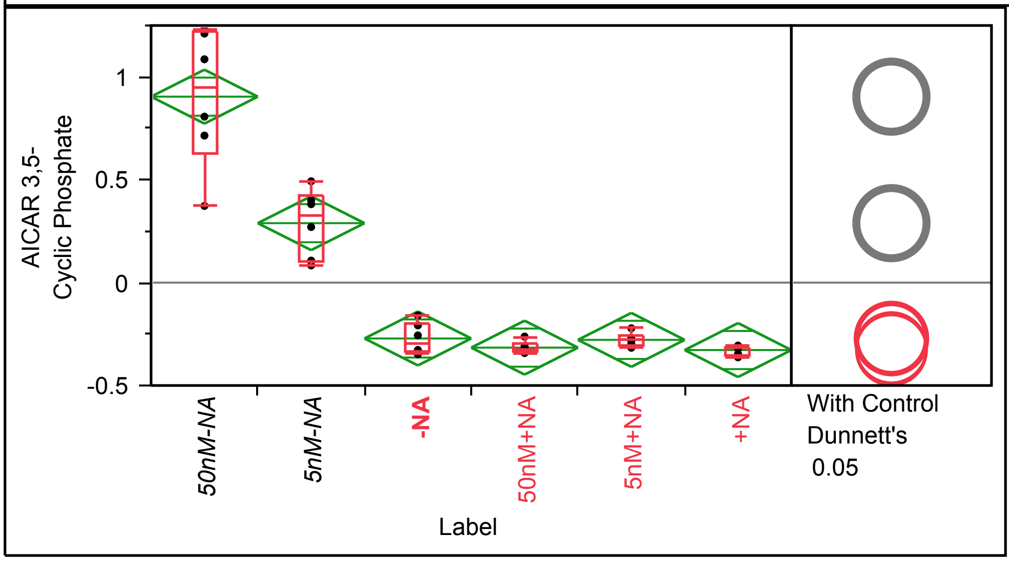

Quantiles

Level	Minimum	10%	25%	Median	75%	90%	Maximum
50nM-NA	0.372871	0.372871	0.629357	0.946287	1.216381	1.233304	1.233304
5nM-NA	0.08376	0.08376	0.10124	0.325903	0.422721	0.492581	0.492581
-NA	-0.34808	-0.34808	-0.33365	-0.29192	-0.19575	-0.15992	-0.15992
50nM+NA	-0.34221	-0.34221	-0.33433	-0.3249	-0.29786	-0.26224	-0.26224
5nM+NA	-0.31667	-0.31667	-0.30336	-0.28029	-0.25868	-0.22186	-0.22186
+NA	-0.36307	-0.36307	-0.35103	-0.3185	-0.31115	-0.30599	-0.30599

Oneway Anova

Summary of Fit

Rsquare	0.910641
Adj Rsquare	0.895748
Root Mean Square Error	0.157197
Mean of Response	-1.2e-17
Observations (or Sum Wgts)	36

Analysis of Variance

Source	DF	Sum of Squares	Mean Square	F Ratio	Prob > F
Label	5	7.5546943	1.51094	61.1449	<.0001 *
Error	30	0.7413240	0.02471		
C. Total	35	8.2960183			

Means for Oneway Anova

Level	Number	Mean	Std Error	Lower 95%	Upper 95%
50nM-NA	6	0.90406	0.06418	0.7730	1.035
5nM-NA	6	0.28911	0.06418	0.1580	0.420
-NA	6	-0.27139	0.06418	-0.4025	-0.140
50nM+NA	6	-0.31595	0.06418	-0.4470	-0.185
5nM+NA	6	-0.27816	0.06418	-0.4092	-0.147
+NA	6	-0.32766	0.06418	-0.4587	-0.197

Std Error uses a pooled estimate of error variance

Means Comparisons

Comparisons with a control using Dunnett's Method

Oneway Analysis of AICAR(ZMP) By Label

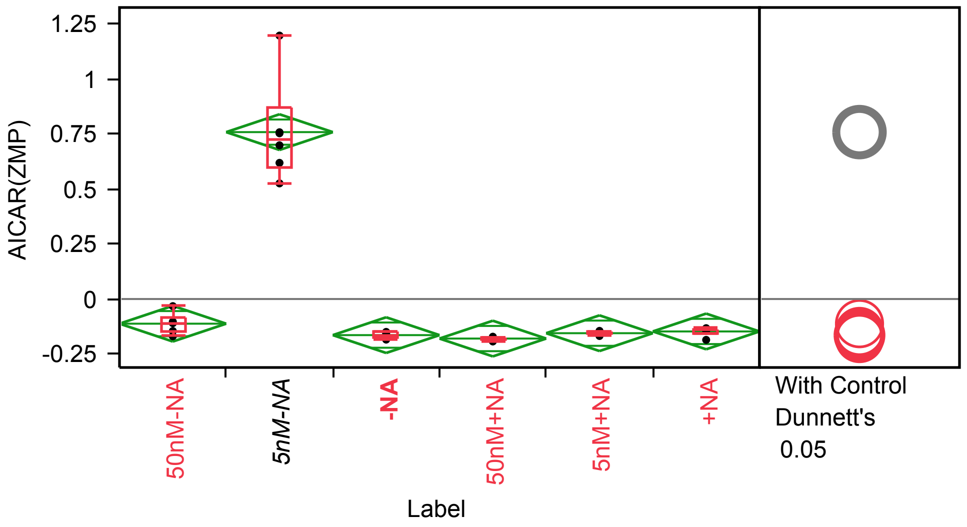

Quantiles

Level	Minimum	10%	25%	Median	75%	90%	Maximum
50nM-NA	-0.16912	-0.16912	-0.15141	-0.11121	-0.08547	-0.03245	-0.03245
5nM-NA	0.525644	0.525644	0.595543	0.726007	0.867752	1.197175	1.197175
-NA	-0.18442	-0.18442	-0.17424	-0.16355	-0.15189	-0.14955	-0.14955
50nM+NA	-0.1929	-0.1929	-0.18471	-0.17884	-0.17357	-0.17139	-0.17139
5nM+NA	-0.16798	-0.16798	-0.15961	-0.15579	-0.14928	-0.14488	-0.14488
+NA	-0.18574	-0.18574	-0.15489	-0.14091	-0.1377	-0.13261	-0.13261

Oneway Anova

Summary of Fit

Rsquare	0.936097
Adj Rsquare	0.925446
Root Mean Square Error	0.097279
Mean of Response	-2.8e-10
Observations (or Sum Wgts)	36

Analysis of Variance

Source	DF	Sum of Squares	Mean Square	F Ratio	Prob > F
Label	5	4.1586762	0.831735	87.8921	<.0001 *
Error	30	0.2838944	0.009463		
C. Total	35	4.4425705			

Means for Oneway Anova

Level	Number	Mean	Std Error	Lower 95%	Upper 95%
50nM-NA	6	-0.11211	0.03971	-0.1932	-0.0310
5nM-NA	6	0.75860	0.03971	0.6775	0.8397
-NA	6	-0.16410	0.03971	-0.2452	-0.0830
50nM+NA	6	-0.17971	0.03971	-0.2608	-0.0986
5nM+NA	6	-0.15533	0.03971	-0.2364	-0.0742
+NA	6	-0.14736	0.03971	-0.2285	-0.0663

Std Error uses a pooled estimate of error variance

Means Comparisons

Comparisons with a control using Dunnett's Method

Oneway Analysis of 2-ketoglutarate By Label

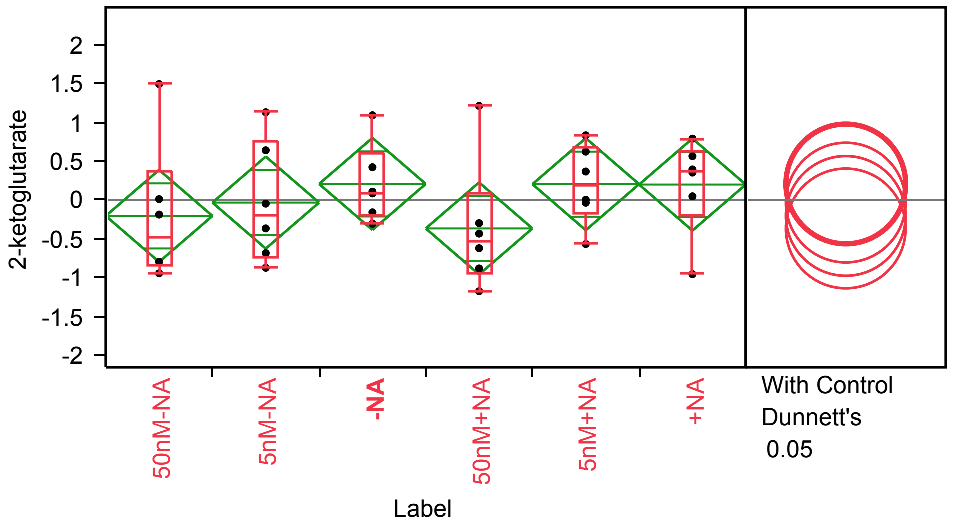

Quantiles

Level	Minimum	10%	25%	Median	75%	90%	Maximum
50nM-NA	-0.94591	-0.94591	-0.84239	-0.49318	0.381148	1.493523	1.493523
5nM-NA	-0.87473	-0.87473	-0.73283	-0.20739	0.762619	1.130293	1.130293
-NA	-0.30678	-0.30678	-0.19662	0.095143	0.589327	1.091615	1.091615
50nM+NA	-1.17701	-1.17701	-0.95663	-0.52832	0.078195	1.211892	1.211892
5nM+NA	-0.56835	-0.56835	-0.16919	0.183976	0.675135	0.832968	0.832968
+NA	-0.95699	-0.95699	-0.2042	0.372822	0.620723	0.789757	0.789757

Oneway Anova

Summary of Fit

Rsquare	0.106519
Adj Rsquare	-0.0424
Root Mean Square Error	0.711456
Mean of Response	8.33e-10
Observations (or Sum Wgts)	36

Analysis of Variance

Source	DF	Sum of Squares	Mean Square	F Ratio	Prob > F
Label	5	1.810328	0.362066	0.7153	0.6169
Error	30	15.185105	0.506170		
C. Total	35	16.995433			

Means for Oneway Anova

Level	Number	Mean	Std Error	Lower 95%	Upper 95%
50nM-NA	6	-0.20605	0.29045	-0.7992	0.38713
5nM-NA	6	-0.03411	0.29045	-0.6273	0.55907
-NA	6	0.20619	0.29045	-0.3870	0.79937
50nM+NA	6	-0.36744	0.29045	-0.9606	0.22574
5nM+NA	6	0.20316	0.29045	-0.3900	0.79634
+NA	6	0.19825	0.29045	-0.3949	0.79143

Std Error uses a pooled estimate of error variance

Means Comparisons

Comparisons with a control using Dunnett's Method

Oneway Analysis of Ala-Ala By Label

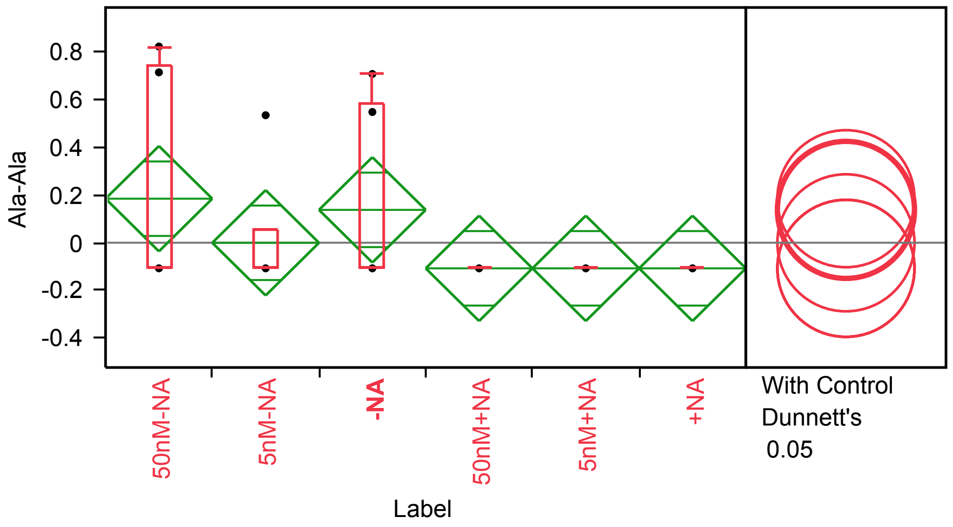

Quantiles

Level	Minimum	10%	25%	Median	75%	90%	Maximum
50nM-NA	-0.10719	-0.10719	-0.10717	-0.10715	0.74087	0.821913	0.821913
5nM-NA	-0.10723	-0.10723	-0.10722	-0.10717	0.053162	0.534126	0.534126
-NA	-0.1072	-0.1072	-0.1072	-0.10719	0.587501	0.706901	0.706901
50nM+NA	-0.10731	-0.10731	-0.10731	-0.10729	-0.10726	-0.10725	-0.10725
5nM+NA	-0.10732	-0.10732	-0.10728	-0.10726	-0.10725	-0.10723	-0.10723
+NA	-0.10749	-0.10749	-0.10733	-0.10726	-0.10726	-0.10725	-0.10725

Oneway Anova

Summary of Fit

Rsquare	0.199925
Adj Rsquare	0.066579
Root Mean Square Error	0.26467
Mean of Response	-3.1e-18
Observations (or Sum Wgts)	36

Analysis of Variance

Source	DF	Sum of Squares	Mean Square	F Ratio	Prob > F
Label	5	0.5251301	0.105026	1.4993	0.2196
Error	30	2.1015027	0.070050		
C. Total	35	2.6266328			

Means for Oneway Anova

Level	Number	Mean	Std Error	Lower 95%	Upper 95%
50nM-NA	6	0.18452	0.10805	-0.0361	0.40519
5nM-NA	6	-0.00030	0.10805	-0.2210	0.22037
-NA	6	0.13764	0.10805	-0.0830	0.35831
50nM+NA	6	-0.10729	0.10805	-0.3280	0.11338
5nM+NA	6	-0.10727	0.10805	-0.3279	0.11340
+NA	6	-0.10730	0.10805	-0.3280	0.11337

Std Error uses a pooled estimate of error variance

Means Comparisons

Comparisons with a control using Dunnett's Method

Oneway Analysis of Ala-Gln By Label

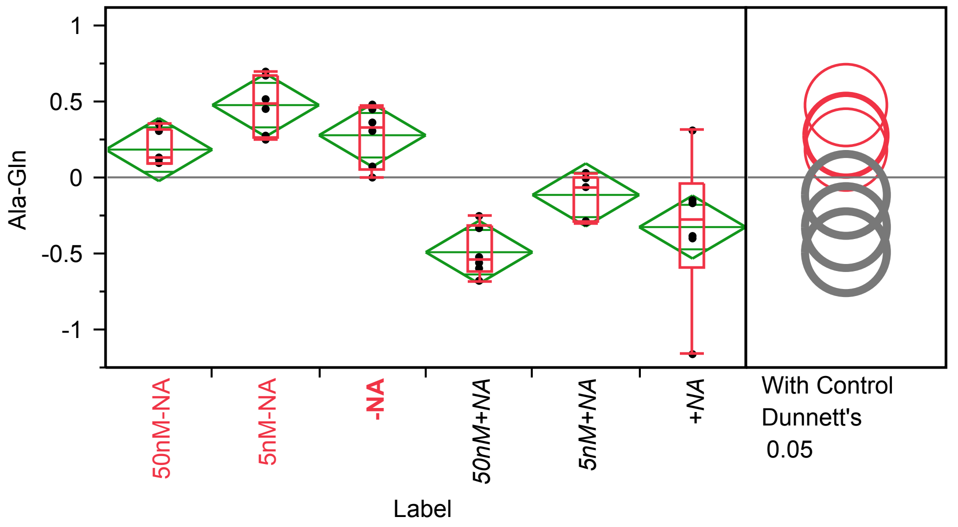

Quantiles

Level	Minimum	10%	25%	Median	75%	90%	Maximum
50nM-NA	0.093716	0.093716	0.09404	0.125679	0.316317	0.350832	0.350832
5nM-NA	0.247914	0.247914	0.267383	0.481877	0.675582	0.695034	0.695034
-NA	-0.00126	-0.00126	0.052998	0.332624	0.455643	0.478235	0.478235
50nM+NA	-0.67983	-0.67983	-0.61878	-0.54303	-0.31404	-0.25411	-0.25411
5nM+NA	-0.29731	-0.29731	-0.2901	-0.0651	0.002759	0.028823	0.028823
+NA	-1.16246	-1.16246	-0.59149	-0.27786	-0.0355	0.308216	0.308216

Oneway Anova

Summary of Fit

Rsquare	0.694225
Adj Rsquare	0.643263
Root Mean Square Error	0.247938
Mean of Response	2.78e-11
Observations (or Sum Wgts)	36

Analysis of Variance

Source	DF	Sum of Squares	Mean Square	F Ratio	Prob > F
Label	5	4.1870285	0.837406	13.6223	<.0001 *
Error	30	1.8441945	0.061473		
C. Total	35	6.0312229			

Means for Oneway Anova

Level	Number	Mean	Std Error	Lower 95%	Upper 95%
50nM-NA	6	0.18248	0.10122	-0.0242	0.3892
5nM-NA	6	0.47495	0.10122	0.2682	0.6817
-NA	6	0.27690	0.10122	0.0702	0.4836
50nM+NA	6	-0.49207	0.10122	-0.6988	-0.2854
5nM+NA	6	-0.11539	0.10122	-0.3221	0.0913
+NA	6	-0.32687	0.10122	-0.5336	-0.1201

Std Error uses a pooled estimate of error variance

Means Comparisons

Comparisons with a control using Dunnett's Method

Oneway Analysis of Ala-Gly By Label

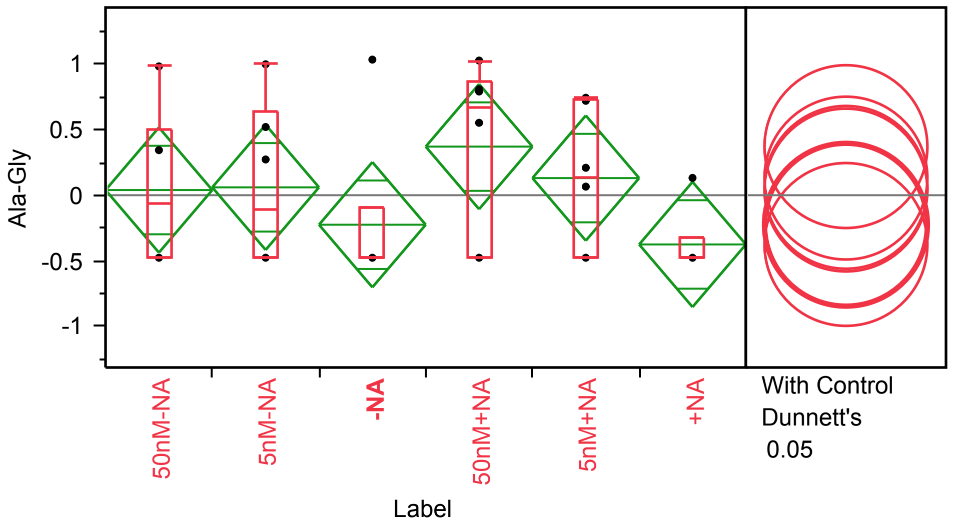

Quantiles

Level	Minimum	10%	25%	Median	75%	90%	Maximum
50nM-NA	-0.47801	-0.47801	-0.47801	-0.06801	0.504391	0.984552	0.984552
5nM-NA	-0.47803	-0.47803	-0.47803	-0.10241	0.641164	1.000718	1.000718
-NA	-0.47804	-0.47804	-0.47803	-0.47802	-0.09942	1.036363	1.036363
50nM+NA	-0.47806	-0.47806	-0.47805	0.673125	0.864374	1.029091	1.029091
5nM+NA	-0.47808	-0.47808	-0.47806	0.13759	0.727369	0.744591	0.744591
+NA	-0.47816	-0.47816	-0.47808	-0.47805	-0.3255	0.132148	0.132148

Oneway Anova

Summary of Fit

Rsquare	0.176612
Adj Rsquare	0.039381
Root Mean Square Error	0.573587
Mean of Response	2.47e-17
Observations (or Sum Wgts)	36

Analysis of Variance

Source	DF	Sum of Squares	Mean Square	F Ratio	Prob > F
Label	5	2.117076	0.423415	1.2870	0.2955
Error	30	9.870068	0.329002		
C. Total	35	11.987144			

Means for Oneway Anova

Level	Number	Mean	Std Error	Lower 95%	Upper 95%
50nM-NA	6	0.03947	0.23417	-0.4388	0.51770
5nM-NA	6	0.06019	0.23417	-0.4180	0.53842
-NA	6	-0.22563	0.23417	-0.7039	0.25261
50nM+NA	6	0.37145	0.23417	-0.1068	0.84968
5nM+NA	6	0.13088	0.23417	-0.3474	0.60911
+NA	6	-0.37637	0.23417	-0.8546	0.10186

Std Error uses a pooled estimate of error variance

Means Comparisons

Comparisons with a control using Dunnett's Method

Oneway Analysis of alanine By Label

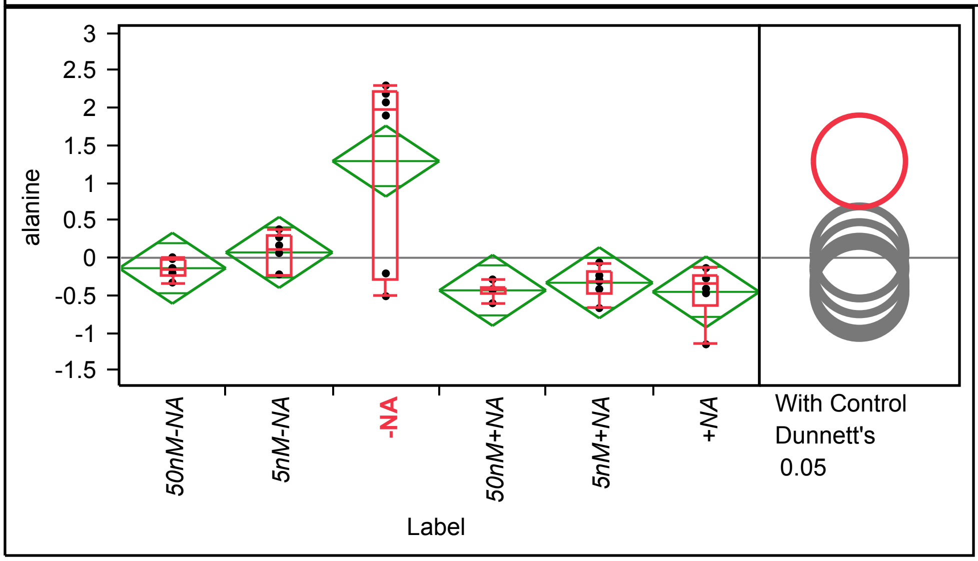

Quantiles

Level	Minimum	10%	25%	Median	75%	90%	Maximum
50nM-NA	-0.33011	-0.33011	-0.23597	-0.14537	-0.01028	0.006806	0.006806
5nM-NA	-0.2316	-0.2316	-0.22407	0.114643	0.300596	0.380095	0.380095
-NA	-0.51247	-0.51247	-0.28452	1.986476	2.216588	2.29958	2.29958
50nM+NA	-0.60609	-0.60609	-0.4763	-0.43115	-0.38521	-0.28922	-0.28922
5nM+NA	-0.6715	-0.6715	-0.48095	-0.30507	-0.19319	-0.06327	-0.06327
+NA	-1.15486	-1.15486	-0.64374	-0.34482	-0.24025	-0.13694	-0.13694

Oneway Anova

Summary of Fit

Rsquare	0.577916
Adj Rsquare	0.507568
Root Mean Square Error	0.56632
Mean of Response	8.33e-11
Observations (or Sum Wgts)	36

Analysis of Variance

Source	DF	Sum of Squares	Mean Square	F Ratio	Prob > F
Label	5	13.173758	2.63475	8.2152	<.0001 *
Error	30	9.621542	0.32072		
C. Total	35	22.795300			

Means for Oneway Anova

Level	Number	Mean	Std Error	Lower 95%	Upper 95%
50nM-NA	6	-0.1391	0.23120	-0.6113	0.3331
5nM-NA	6	0.0717	0.23120	-0.4005	0.5439
-NA	6	1.2901	0.23120	0.8179	1.7622
50nM+NA	6	-0.4346	0.23120	-0.9068	0.0375
5nM+NA	6	-0.3331	0.23120	-0.8053	0.1390
+NA	6	-0.4549	0.23120	-0.9271	0.0173

Std Error uses a pooled estimate of error variance

Means Comparisons

Comparisons with a control using Dunnett's Method

Oneway Analysis of allantoate By Label

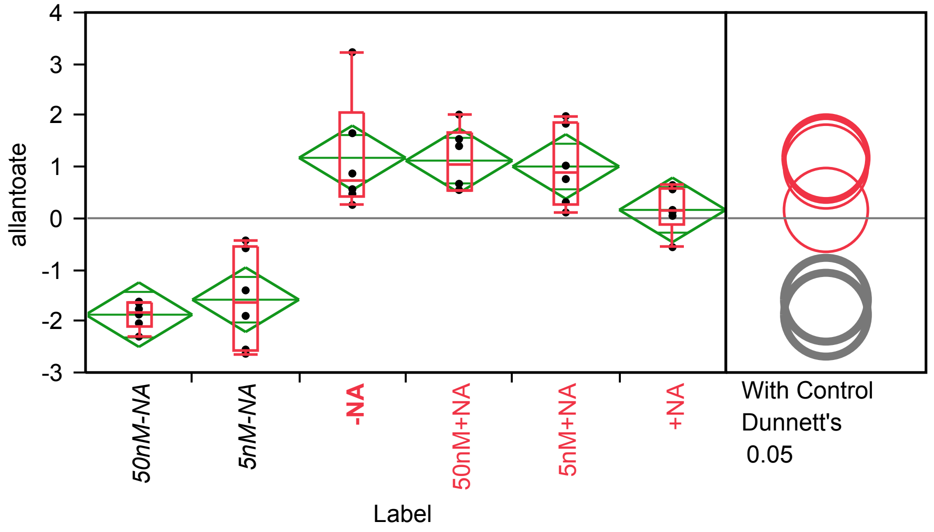

Quantiles

Level	Minimum	10%	25%	Median	75%	90%	Maximum
50nM-NA	-2.3021	-2.3021	-2.10784	-1.81933	-1.6422	-1.62262	-1.62262
5nM-NA	-2.63509	-2.63509	-2.57464	-1.65102	-0.54585	-0.43303	-0.43303
-NA	0.259112	0.259112	0.418752	0.716336	2.046761	3.227707	3.227707
50nM+NA	0.544888	0.544888	0.54753	1.035668	1.658203	2.014638	2.014638
5nM+NA	0.112245	0.112245	0.262022	0.891831	1.874415	1.984756	1.984756
+NA	-0.56335	-0.56335	-0.10648	0.1412	0.581906	0.643605	0.643605

Oneway Anova

Summary of Fit

Rsquare	0.77469
Adj Rsquare	0.737139
Root Mean Square Error	0.7511
Mean of Response	-8e-17
Observations (or Sum Wgts)	36

Analysis of Variance

Source	DF	Sum of Squares	Mean Square	F Ratio	Prob > F
Label	5	58.192313	11.6385	20.6300	<.0001 *
Error	30	16.924555	0.5642		
C. Total	35	75.116869			

Means for Oneway Anova

Level	Number	Mean	Std Error	Lower 95%	Upper 95%
50nM-NA	6	-1.8759	0.30664	-2.502	-1.250
5nM-NA	6	-1.5847	0.30664	-2.211	-0.958
-NA	6	1.1741	0.30664	0.548	1.800
50nM+NA	6	1.1198	0.30664	0.494	1.746
5nM+NA	6	1.0050	0.30664	0.379	1.631
+NA	6	0.1616	0.30664	-0.465	0.788

Std Error uses a pooled estimate of error variance

Means Comparisons

Comparisons with a control using Dunnett's Method

Oneway Analysis of Aminoadipic acid By Label

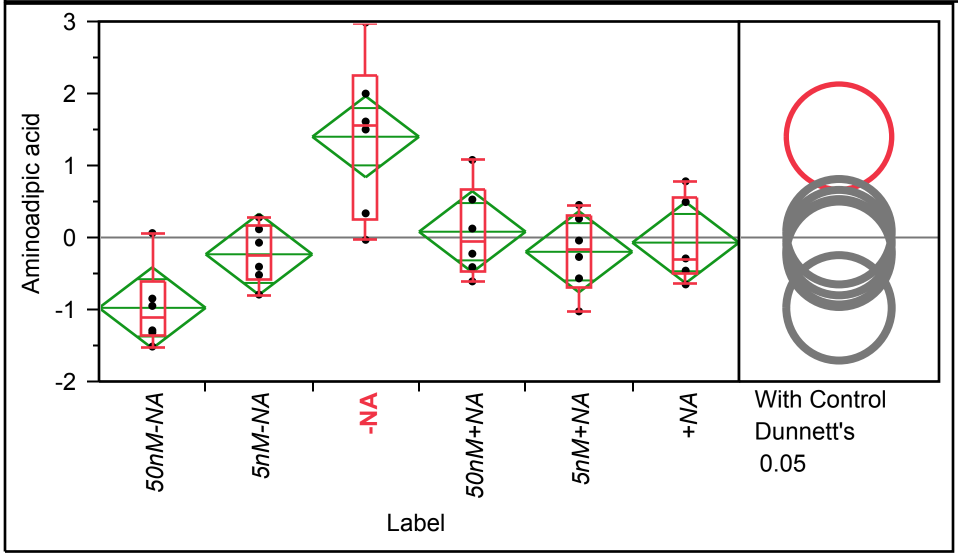

Quantiles

Level	Minimum	10%	25%	Median	75%	90%	Maximum
50nM-NA	-1.515	-1.515	-1.36867	-1.11999	-0.6213	0.061246	0.061246
5nM-NA	-0.79287	-0.79287	-0.58837	-0.23914	0.155611	0.278174	0.278174
-NA	-0.03214	-0.03214	0.2435	1.555245	2.24509	2.985088	2.985088
50nM+NA	-0.60954	-0.60954	-0.46123	-0.05075	0.664668	1.076757	1.076757
5nM+NA	-1.02579	-1.02579	-0.68241	-0.15598	0.309536	0.451935	0.451935
+NA	-0.65135	-0.65135	-0.50822	-0.29321	0.564164	0.780206	0.780206

Oneway Anova

Summary of Fit

Rsquare	0.569984
Adj Rsquare	0.498315
Root Mean Square Error	0.674886
Mean of Response	8.33e-10
Observations (or Sum Wgts)	36

Analysis of Variance

Source	DF	Sum of Squares	Mean Square	F Ratio	Prob > F
Label	5	18.111773	3.62235	7.9530	<.0001 *
Error	30	13.664132	0.45547		
C. Total	35	31.775904			

Means for Oneway Anova

Level	Number	Mean	Std Error	Lower 95%	Upper 95%
50nM-NA	6	-0.9771	0.27552	-1.540	-0.414
5nM-NA	6	-0.2331	0.27552	-0.796	0.330
-NA	6	1.3995	0.27552	0.837	1.962
50nM+NA	6	0.0802	0.27552	-0.482	0.643
5nM+NA	6	-0.1986	0.27552	-0.761	0.364
+NA	6	-0.0710	0.27552	-0.634	0.492

Std Error uses a pooled estimate of error variance

Means Comparisons

Comparisons with a control using Dunnett's Method

Oneway Analysis of AMP By Label

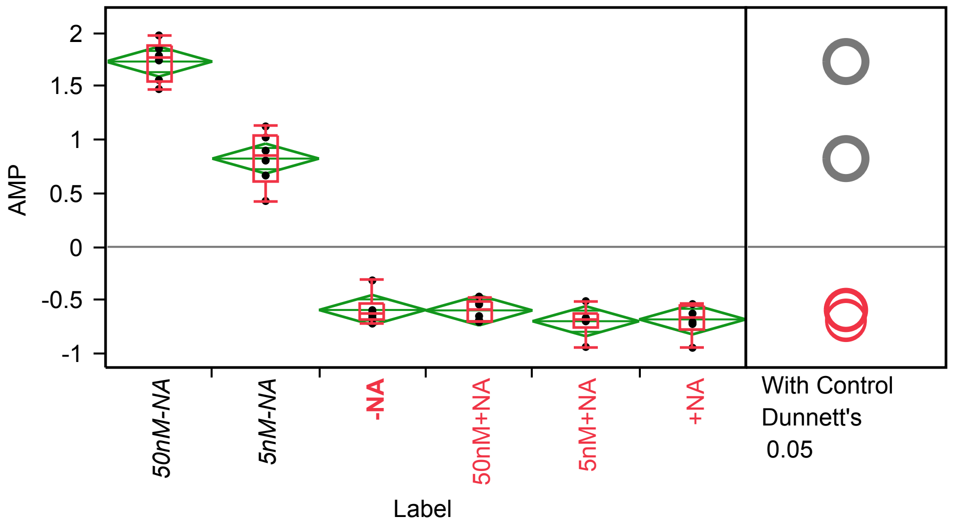

Quantiles

Level	Minimum	10%	25%	Median	75%	90%	Maximum
50nM-NA	1.471958	1.471958	1.535702	1.76212	1.879331	1.972619	1.972619
5nM-NA	0.427989	0.427989	0.606297	0.851264	1.046609	1.123576	1.123576
-NA	-0.716	-0.716	-0.67488	-0.62631	-0.52142	-0.31329	-0.31329
50nM+NA	-0.70101	-0.70101	-0.68818	-0.59201	-0.50856	-0.46456	-0.46456
5nM+NA	-0.93397	-0.93397	-0.75445	-0.6821	-0.62414	-0.50677	-0.50677
+NA	-0.94168	-0.94168	-0.77513	-0.66224	-0.5395	-0.53435	-0.53435

Oneway Anova

Summary of Fit

Rsquare	0.974158
Adj Rsquare	0.969852
Root Mean Square Error	0.167771
Mean of Response	5.556e-9
Observations (or Sum Wgts)	36

Analysis of Variance

Source	DF	Sum of Squares	Mean Square	F Ratio	Prob > F
Label	5	31.832244	6.36645	226.1842	<.0001 *
Error	30	0.844415	0.02815		
C. Total	35	32.676660			

Means for Oneway Anova

Level	Number	Mean	Std Error	Lower 95%	Upper 95%
50nM-NA	6	1.7290	0.06849	1.589	1.869
5nM-NA	6	0.8235	0.06849	0.684	0.963
-NA	6	-0.5890	0.06849	-0.729	-0.449
50nM+NA	6	-0.5928	0.06849	-0.733	-0.453
5nM+NA	6	-0.6938	0.06849	-0.834	-0.554
+NA	6	-0.6769	0.06849	-0.817	-0.537

Std Error uses a pooled estimate of error variance

Means Comparisons

Comparisons with a control using Dunnett's Method

Oneway Analysis of Arabitol By Label

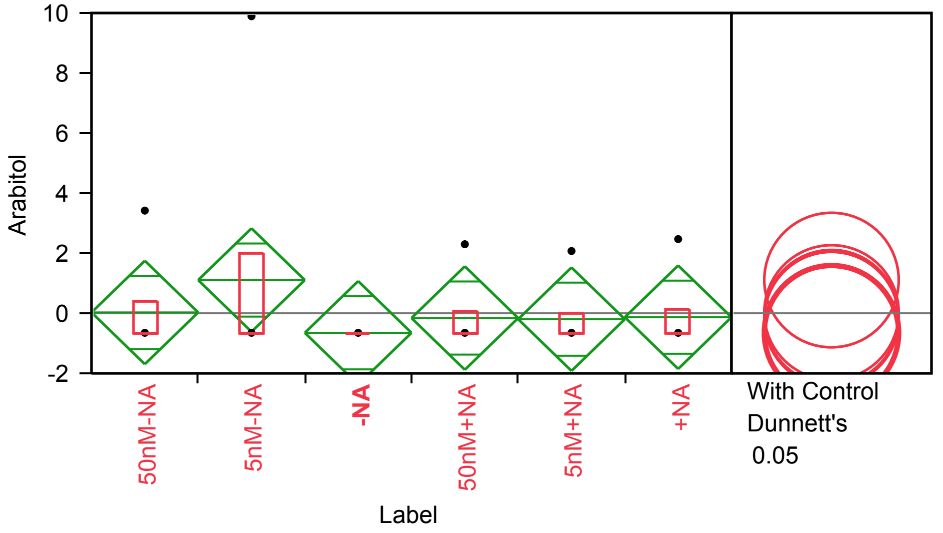

Quantiles

Level	Minimum	10%	25%	Median	75%	90%	Maximum
50nM-NA	-0.65021	-0.65021	-0.65021	-0.6502	0.367214	3.419454	3.419454
5nM-NA	-0.65021	-0.65021	-0.65021	-0.6502	1.985248	9.891594	9.891594
-NA	-0.65021	-0.65021	-0.65021	-0.65021	-0.65021	-0.65021	-0.65021
50nM+NA	-0.65022	-0.65022	-0.65022	-0.65022	0.087366	2.300105	2.300105
5nM+NA	-0.65022	-0.65022	-0.65022	-0.65022	0.030781	2.073764	2.073764
+NA	-0.65025	-0.65025	-0.65022	-0.65022	0.130257	2.471673	2.471673

Oneway Anova

Summary of Fit

Rsquare	0.074987
Adj Rsquare	-0.07918
Root Mean Square Error	2.065338
Mean of Response	5.556e-9
Observations (or Sum Wgts)	36

Analysis of Variance

Source	DF	Sum of Squares	Mean Square	F Ratio	Prob > F
Label	5	10.37389	2.07478	0.4864	0.7836
Error	30	127.96863	4.26562		
C. Total	35	138.34252			

Means for Oneway Anova

Level	Number	Mean	Std Error	Lower 95%	Upper 95%
50nM-NA	6	0.0281	0.84317	-1.694	1.7501
5nM-NA	6	1.1068	0.84317	-0.615	2.8287
-NA	6	-0.6502	0.84317	-2.372	1.0718
50nM+NA	6	-0.1585	0.84317	-1.880	1.5635
5nM+NA	6	-0.1962	0.84317	-1.918	1.5258
+NA	6	-0.1299	0.84317	-1.852	1.5921

Std Error uses a pooled estimate of error variance

Means Comparisons

Comparisons with a control using Dunnett's Method

Oneway Analysis of arginine By Label

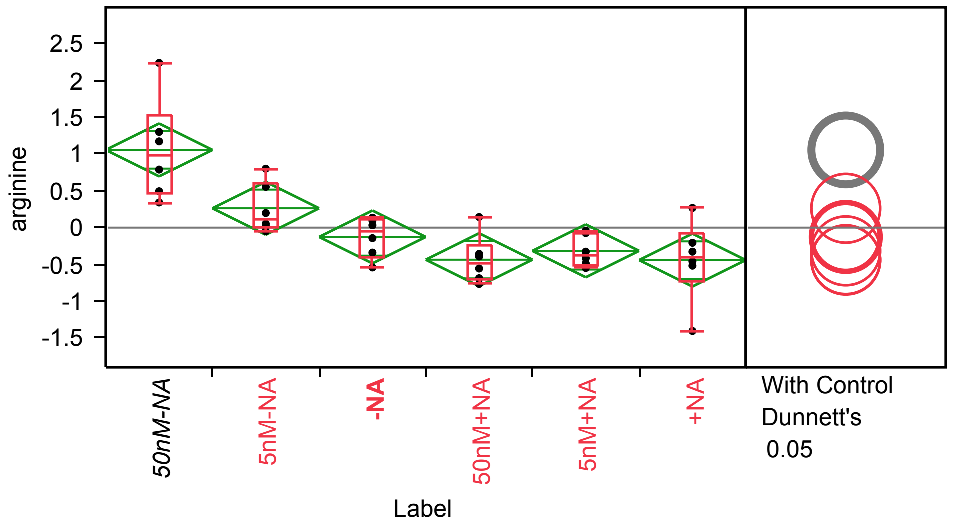

Quantiles

Level	Minimum	10%	25%	Median	75%	90%	Maximum
50nM-NA	0.341555	0.341555	0.455516	0.979464	1.533235	2.236059	2.236059
5nM-NA	-0.04755	-0.04755	0.002524	0.125067	0.615004	0.799815	0.799815
-NA	-0.54061	-0.54061	-0.38934	-0.0552	0.106139	0.134584	0.134584
50nM+NA	-0.76518	-0.76518	-0.70643	-0.47331	-0.23118	0.143738	0.143738
5nM+NA	-0.54781	-0.54781	-0.50176	-0.37062	-0.06594	-0.0423	-0.0423
+NA	-1.40326	-1.40326	-0.73821	-0.39207	-0.0877	0.267267	0.267267

Oneway Anova

Summary of Fit

Rsquare	0.644603
Adj Rsquare	0.585371
Root Mean Square Error	0.430451
Mean of Response	-2.8e-10
Observations (or Sum Wgts)	36

Analysis of Variance

Source	DF	Sum of Squares	Mean Square	F Ratio	Prob > F
Label	5	10.082020	2.01640	10.8825	<.0001 *
Error	30	5.558637	0.18529		
C. Total	35	15.640657			

Means for Oneway Anova

Level	Number	Mean	Std Error	Lower 95%	Upper 95%
50nM-NA	6	1.0548	0.17573	0.6959	1.414
5nM-NA	6	0.2625	0.17573	-0.0964	0.621
-NA	6	-0.1264	0.17573	-0.4853	0.232
50nM+NA	6	-0.4352	0.17573	-0.7941	-0.076
5nM+NA	6	-0.3153	0.17573	-0.6742	0.044
+NA	6	-0.4404	0.17573	-0.7993	-0.082

Std Error uses a pooled estimate of error variance

Means Comparisons

Comparisons with a control using Dunnett's Method

Oneway Analysis of asparagine By Label

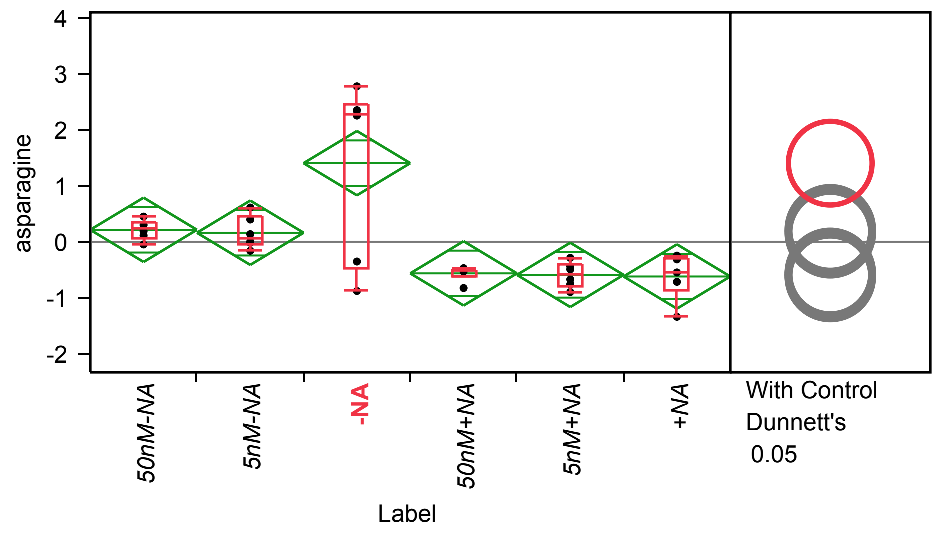

Quantiles

Level	Minimum	10%	25%	Median	75%	90%	Maximum
50nM-NA	-0.04822	-0.04822	0.071195	0.233942	0.336102	0.447841	0.447841
5nM-NA	-0.16099	-0.16099	-0.05458	0.068104	0.449644	0.609587	0.609587
-NA	-0.87804	-0.87804	-0.48414	2.26082	2.451371	2.770718	2.770718
50nM+NA	-0.82559	-0.82559	-0.60599	-0.5253	-0.49494	-0.47147	-0.47147
5nM+NA	-0.89666	-0.89666	-0.79275	-0.58188	-0.4007	-0.28797	-0.28797
+NA	-1.33816	-1.33816	-0.87074	-0.55109	-0.29798	-0.24785	-0.24785

Oneway Anova

Summary of Fit

Rsquare	0.566138
Adj Rsquare	0.493827
Root Mean Square Error	0.687629
Mean of Response	5.56e-11
Observations (or Sum Wgts)	36

Analysis of Variance

Source	DF	Sum of Squares	Mean Square	F Ratio	Prob > F
Label	5	18.509724	3.70194	7.8293	<.0001 *
Error	30	14.185020	0.47283		
C. Total	35	32.694743			

Means for Oneway Anova

Level	Number	Mean	Std Error	Lower 95%	Upper 95%
50nM-NA	6	0.2129	0.28072	-0.360	0.786
5nM-NA	6	0.1603	0.28072	-0.413	0.734
-NA	6	1.4011	0.28072	0.828	1.974
50nM+NA	6	-0.5639	0.28072	-1.137	0.00944
5nM+NA	6	-0.5908	0.28072	-1.164	-0.017
+NA	6	-0.6196	0.28072	-1.193	-0.046

Std Error uses a pooled estimate of error variance

Means Comparisons

Comparisons with a control using Dunnett's Method

Oneway Analysis of aspartate By Label

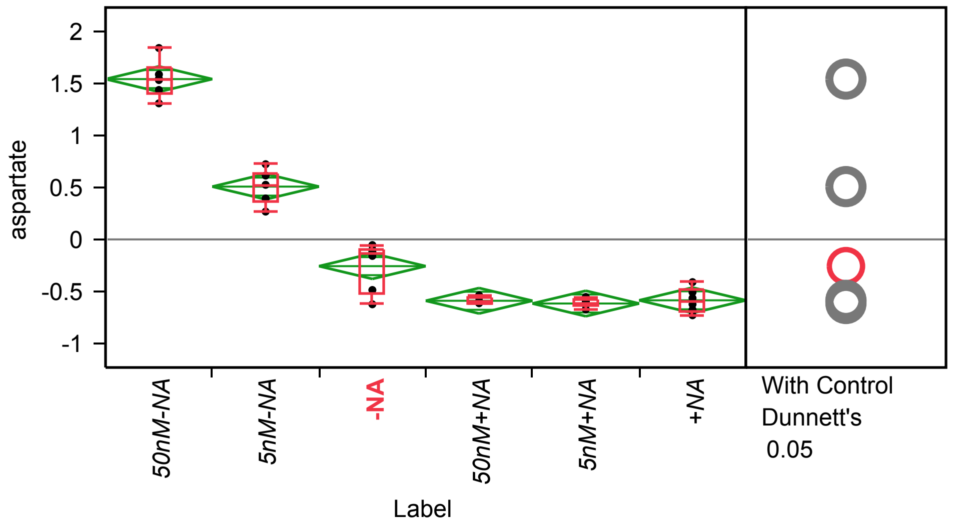

Quantiles

Level	Minimum	10%	25%	Median	75%	90%	Maximum
50nM-NA	1.310418	1.310418	1.406893	1.540827	1.652208	1.843152	1.843152
5nM-NA	0.267961	0.267961	0.362721	0.524807	0.642218	0.724118	0.724118
-NA	-0.62475	-0.62475	-0.52173	-0.13981	-0.08784	-0.05468	-0.05468
50nM+NA	-0.62368	-0.62368	-0.61783	-0.60503	-0.55265	-0.53863	-0.53863
5nM+NA	-0.67387	-0.67387	-0.64451	-0.62225	-0.58568	-0.55471	-0.55471
+NA	-0.73026	-0.73026	-0.69062	-0.59538	-0.48469	-0.41152	-0.41152

Oneway Anova

Summary of Fit

Rsquare	0.972133
Adj Rsquare	0.967488
Root Mean Square Error	0.147261
Mean of Response	-1.2e-17
Observations (or Sum Wgts)	36

Analysis of Variance

Source	DF	Sum of Squares	Mean Square	F Ratio	Prob > F
Label	5	22.694861	4.53897	209.3072	<.0001 *
Error	30	0.650571	0.02169		
C. Total	35	23.345432			

Means for Oneway Anova

Level	Number	Mean	Std Error	Lower 95%	Upper 95%
50nM-NA	6	1.5438	0.06012	1.421	1.667
5nM-NA	6	0.5085	0.06012	0.386	0.631
-NA	6	-0.2576	0.06012	-0.380	-0.135
50nM+NA	6	-0.5909	0.06012	-0.714	-0.468
5nM+NA	6	-0.6173	0.06012	-0.740	-0.495
+NA	6	-0.5865	0.06012	-0.709	-0.464

Std Error uses a pooled estimate of error variance

Means Comparisons

Comparisons with a control using Dunnett's Method

Oneway Analysis of ATP By Label

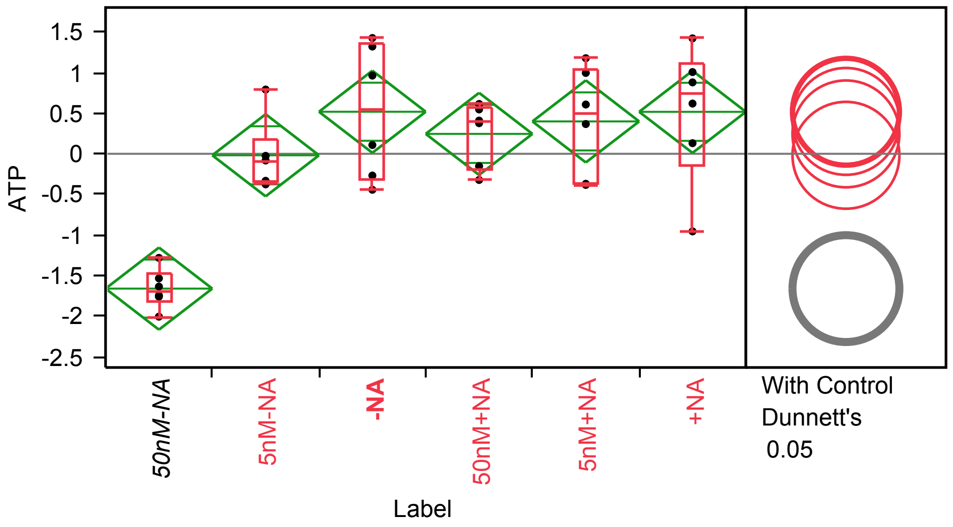

Quantiles

Level	Minimum	10%	25%	Median	75%	90%	Maximum
50nM-NA	-2.00361	-2.00361	-1.81882	-1.68846	-1.4693	-1.28062	-1.28062
5nM-NA	-0.37574	-0.37574	-0.34314	-0.08349	0.178539	0.790023	0.790023
-NA	-0.44238	-0.44238	-0.31148	0.535008	1.345649	1.424214	1.424214
50nM+NA	-0.3219	-0.3219	-0.19497	0.392421	0.562703	0.612926	0.612926
5nM+NA	-0.38284	-0.38284	-0.37244	0.486058	1.040028	1.175568	1.175568
+NA	-0.95448	-0.95448	-0.14069	0.748144	1.108922	1.419803	1.419803

Oneway Anova

Summary of Fit

Rsquare	0.656193
Adj Rsquare	0.598892
Root Mean Square Error	0.605909
Mean of Response	5.56e-10
Observations (or Sum Wgts)	36

Analysis of Variance

Source	DF	Sum of Squares	Mean Square	F Ratio	Prob > F
Label	5	21.020975	4.20419	11.4517	<.0001 *
Error	30	11.013769	0.36713		
C. Total	35	32.034743			

Means for Oneway Anova

Level	Number	Mean	Std Error	Lower 95%	Upper 95%
50nM-NA	6	-1.6584	0.24736	-2.164	-1.153
5nM-NA	6	-0.0184	0.24736	-0.524	0.487
-NA	6	0.5172	0.24736	0.012	1.022
50nM+NA	6	0.2449	0.24736	-0.260	0.750
5nM+NA	6	0.3985	0.24736	-0.107	0.904
+NA	6	0.5162	0.24736	0.011	1.021

Std Error uses a pooled estimate of error variance

Means Comparisons

Comparisons with a control using Dunnett's Method

Oneway Analysis of benzenesulfonamine By Label

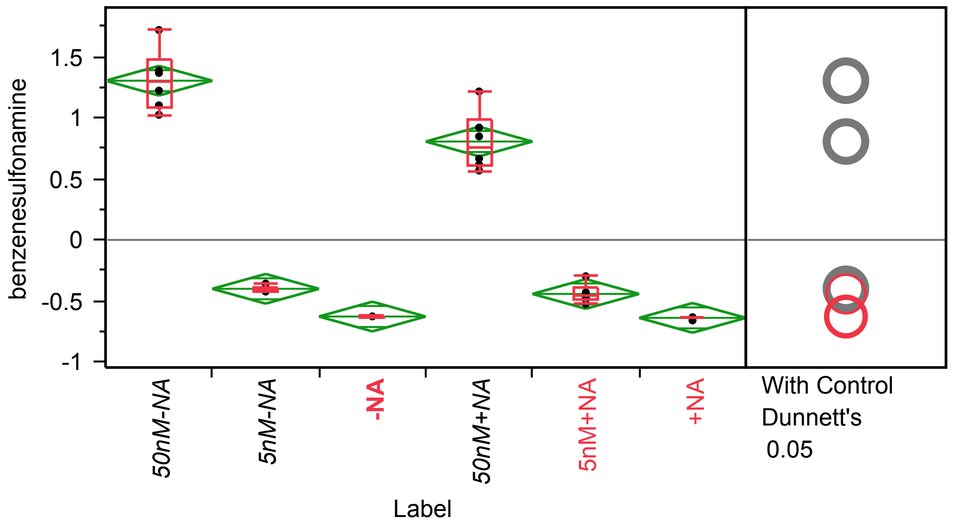

Quantiles

Level	Minimum	10%	25%	Median	75%	90%	Maximum
50nM-NA	1.024381	1.024381	1.082341	1.294399	1.469725	1.718313	1.718313
5nM-NA	-0.42285	-0.42285	-0.41709	-0.40847	-0.38182	-0.35763	-0.35763
-NA	-0.63203	-0.63203	-0.6283	-0.62647	-0.62622	-0.62619	-0.62619
50nM+NA	0.567983	0.567983	0.603761	0.756822	0.993552	1.215238	1.215238
5nM+NA	-0.52353	-0.52353	-0.49135	-0.46168	-0.39655	-0.29768	-0.29768
+NA	-0.65946	-0.65946	-0.64179	-0.63432	-0.63347	-0.63293	-0.63293

Oneway Anova

Summary of Fit

Rsquare	0.970626
Adj Rsquare	0.965731
Root Mean Square Error	0.145642
Mean of Response	2.778e-9
Observations (or Sum Wgts)	36

Analysis of Variance

Source	DF	Sum of Squares	Mean Square	F Ratio	Prob > F
Label	5	21.027791	4.20556	198.2657	<.0001 *
Error	30	0.636352	0.02121		
C. Total	35	21.664143			

Means for Oneway Anova

Level	Number	Mean	Std Error	Lower 95%	Upper 95%
50nM-NA	6	1.3033	0.05946	1.182	1.425
5nM-NA	6	-0.4004	0.05946	-0.522	-0.279
-NA	6	-0.6274	0.05946	-0.749	-0.506
50nM+NA	6	0.8054	0.05946	0.684	0.927
5nM+NA	6	-0.4425	0.05946	-0.564	-0.321
+NA	6	-0.6384	0.05946	-0.760	-0.517

Std Error uses a pooled estimate of error variance

Means Comparisons

Comparisons with a control using Dunnett's Method

Oneway Analysis of Benzoic acid By Label

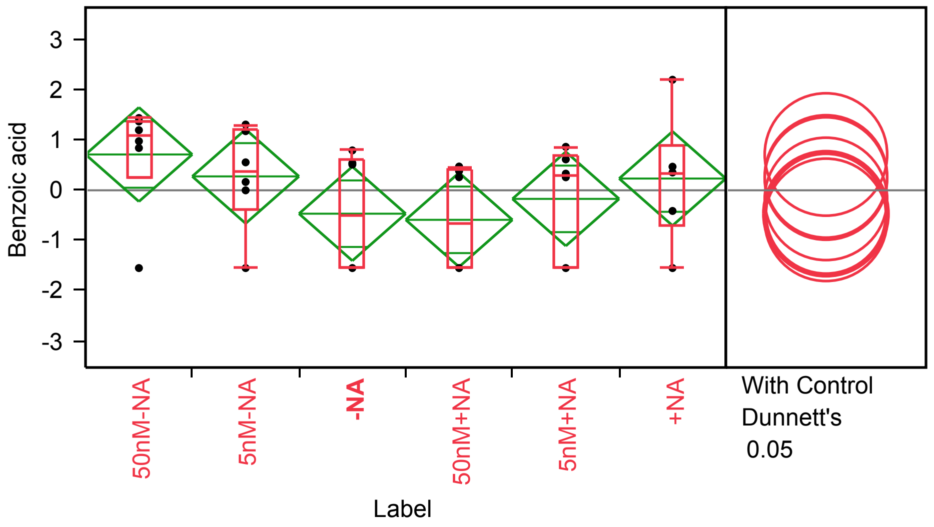

Quantiles

Level	Minimum	10%	25%	Median	75%	90%	Maximum
50nM-NA	-1.55259	-1.55259	0.24538	1.091126	1.392079	1.443449	1.443449
5nM-NA	-1.55259	-1.55259	-0.38849	0.362338	1.213988	1.312587	1.312587
-NA	-1.5526	-1.5526	-1.5526	-0.51931	0.607826	0.796636	0.796636
50nM+NA	-1.55263	-1.55263	-1.55262	-0.64557	0.407515	0.474107	0.474107
5nM+NA	-1.55261	-1.55261	-1.55261	0.296316	0.679866	0.867563	0.867563
+NA	-1.55261	-1.55261	-0.69742	0.34799	0.904915	2.207002	2.207002

Oneway Anova

Summary of Fit

Rsquare	0.163315
Adj Rsquare	0.023867
Root Mean Square Error	1.126746
Mean of Response	-2.8e-11
Observations (or Sum Wgts)	36

Analysis of Variance

Source	DF	Sum of Squares	Mean Square	F Ratio	Prob > F
Label	5	7.434239	1.48685	1.1712	0.3463
Error	30	38.086671	1.26956		
C. Total	35	45.520910			

Means for Oneway Anova

Level	Number	Mean	Std Error	Lower 95%	Upper 95%
50nM-NA	6	0.71546	0.45999	-0.224	1.6549
5nM-NA	6	0.27756	0.45999	-0.662	1.2170
-NA	6	-0.46705	0.45999	-1.406	0.4724
50nM+NA	6	-0.58950	0.45999	-1.529	0.3499
5nM+NA	6	-0.17129	0.45999	-1.111	0.7681
+NA	6	0.23482	0.45999	-0.705	1.1742

Std Error uses a pooled estimate of error variance

Means Comparisons

Comparisons with a control using Dunnett's Method

Oneway Analysis of beta-Alanine By Label

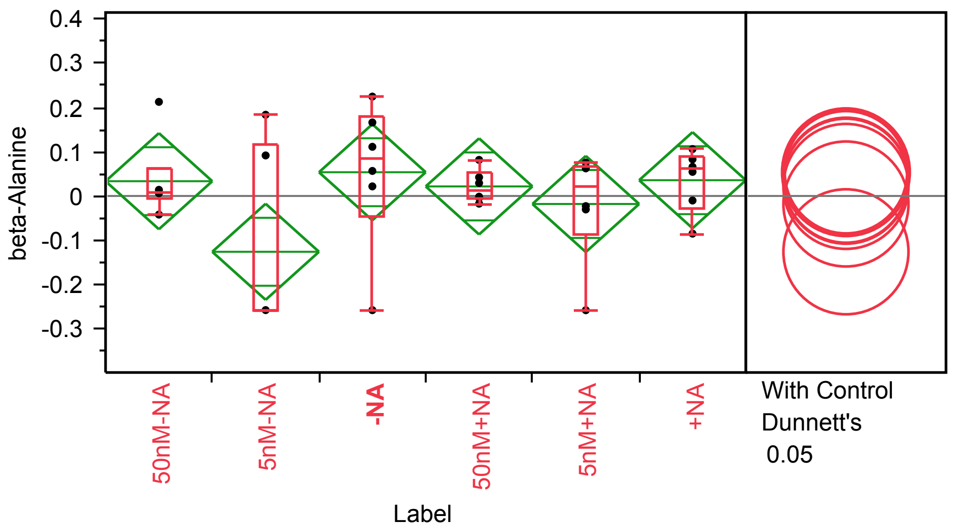

Quantiles

Level	Minimum	10%	25%	Median	75%	90%	Maximum
50nM-NA	-0.04208	-0.04208	-0.00731	0.005731	0.063623	0.212858	0.212858
5nM-NA	-0.25832	-0.25832	-0.25831	-0.25818	0.114684	0.183674	0.183674
-NA	-0.25826	-0.25826	-0.04831	0.083901	0.180879	0.224735	0.224735
50nM+NA	-0.0174	-0.0174	-0.00755	0.013817	0.05206	0.081597	0.081597
5nM+NA	-0.25839	-0.25839	-0.08746	0.01974	0.067751	0.075451	0.075451
+NA	-0.08517	-0.08517	-0.02899	0.060211	0.088725	0.106042	0.106042

Oneway Anova

Summary of Fit

Rsquare	0.205839
Adj Rsquare	0.073479
Root Mean Square Error	0.130328
Mean of Response	-5.6e-11
Observations (or Sum Wgts)	36

Analysis of Variance

Source	DF	Sum of Squares	Mean Square	F Ratio	Prob > F
Label	5	0.13207366	0.026415	1.5551	0.2029
Error	30	0.50956281	0.016985		
C. Total	35	0.64163646			

Means for Oneway Anova

Level	Number	Mean	Std Error	Lower 95%	Upper 95%
50nM-NA	6	0.03340	0.05321	-0.0753	0.1421
5nM-NA	6	-0.12627	0.05321	-0.2349	-0.0176
-NA	6	0.05370	0.05321	-0.0550	0.1624
50nM+NA	6	0.02163	0.05321	-0.0870	0.1303
5nM+NA	6	-0.01813	0.05321	-0.1268	0.0905
+NA	6	0.03567	0.05321	-0.0730	0.1443

Std Error uses a pooled estimate of error variance

Means Comparisons

Comparisons with a control using Dunnett's Method

Oneway Analysis of beta-D-Glucopyranose By Label

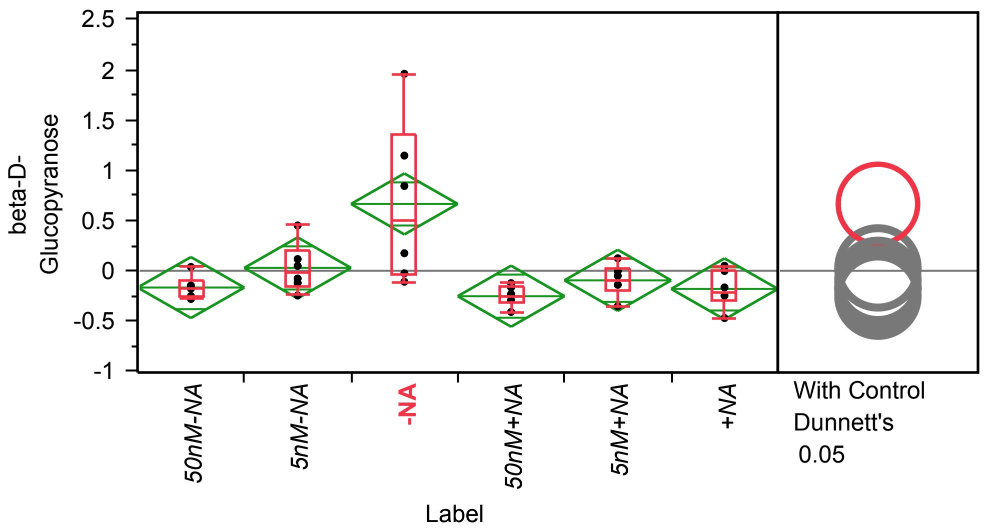

Quantiles

Level	Minimum	10%	25%	Median	75%	90%	Maximum
50nM-NA	-0.28035	-0.28035	-0.25856	-0.17994	-0.09994	0.038571	0.038571
5nM-NA	-0.24528	-0.24528	-0.15185	-0.01427	0.200488	0.450881	0.450881
-NA	-0.10862	-0.10862	-0.04574	0.509255	1.34933	1.959733	1.959733
50nM+NA	-0.40956	-0.40956	-0.32392	-0.26129	-0.15447	-0.12243	-0.12243
5nM+NA	-0.35277	-0.35277	-0.19958	-0.09476	0.029533	0.124679	0.124679
+NA	-0.4699	-0.4699	-0.30319	-0.20627	0.011225	0.050239	0.050239

Oneway Anova

Summary of Fit

Rsquare	0.465255
Adj Rsquare	0.376131
Root Mean Square Error	0.363931
Mean of Response	-5.6e-11
Observations (or Sum Wgts)	36

Analysis of Variance

Source	DF	Sum of Squares	Mean Square	F Ratio	Prob > F
Label	5	3.4570469	0.691409	5.2203	0.0015 *
Error	30	3.9733804	0.132446		
C. Total	35	7.4304273			

Means for Oneway Anova

Level	Number	Mean	Std Error	Lower 95%	Upper 95%
50nM-NA	6	-0.16651	0.14857	-0.4699	0.13692
5nM-NA	6	0.02889	0.14857	-0.2745	0.33232
-NA	6	0.66512	0.14857	0.3617	0.96855
50nM+NA	6	-0.25252	0.14857	-0.5559	0.05091
5nM+NA	6	-0.09472	0.14857	-0.3981	0.20871
+NA	6	-0.18027	0.14857	-0.4837	0.12316

Std Error uses a pooled estimate of error variance

Means Comparisons

Comparisons with a control using Dunnett's Method

Oneway Analysis of betaine By Label

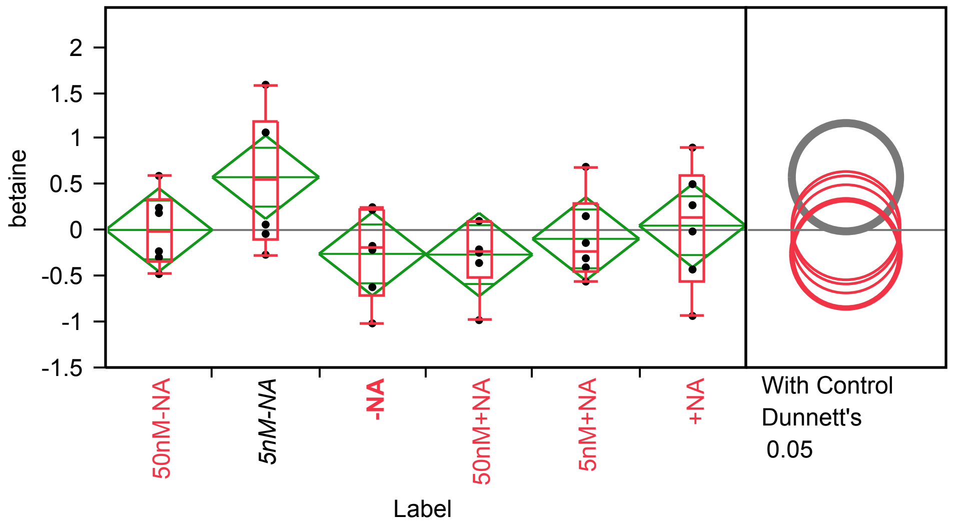

Quantiles

Level	Minimum	10%	25%	Median	75%	90%	Maximum
50nM-NA	-0.48059	-0.48059	-0.34412	-0.02346	0.329556	0.590633	0.590633
5nM-NA	-0.26988	-0.26988	-0.09954	0.560723	1.194409	1.584702	1.584702
-NA	-1.01834	-1.01834	-0.72222	-0.19476	0.22749	0.248821	0.248821
50nM+NA	-0.98315	-0.98315	-0.51557	-0.22996	0.095243	0.098793	0.098793
5nM+NA	-0.56225	-0.56225	-0.4462	-0.22474	0.285814	0.689184	0.689184
+NA	-0.9374	-0.9374	-0.55737	0.127495	0.599841	0.898891	0.898891

Oneway Anova

Summary of Fit

Rsquare	0.24602
Adj Rsquare	0.120357
Root Mean Square Error	0.544377
Mean of Response	2.78e-10
Observations (or Sum Wgts)	36

Analysis of Variance

Source	DF	Sum of Squares	Mean Square	F Ratio	Prob > F
Label	5	2.900898	0.580180	1.9578	0.1141
Error	30	8.890388	0.296346		
C. Total	35	11.791286			

Means for Oneway Anova

Level	Number	Mean	Std Error	Lower 95%	Upper 95%
50nM-NA	6	0.00117	0.22224	-0.4527	0.4550
5nM-NA	6	0.57630	0.22224	0.1224	1.0302
-NA	6	-0.26036	0.22224	-0.7142	0.1935
50nM+NA	6	-0.26832	0.22224	-0.7222	0.1856
5nM+NA	6	-0.09645	0.22224	-0.5503	0.3574
+NA	6	0.04766	0.22224	-0.4062	0.5015

Std Error uses a pooled estimate of error variance

Means Comparisons

Comparisons with a control using Dunnett's Method

Oneway Analysis of betaine aldehyde By Label

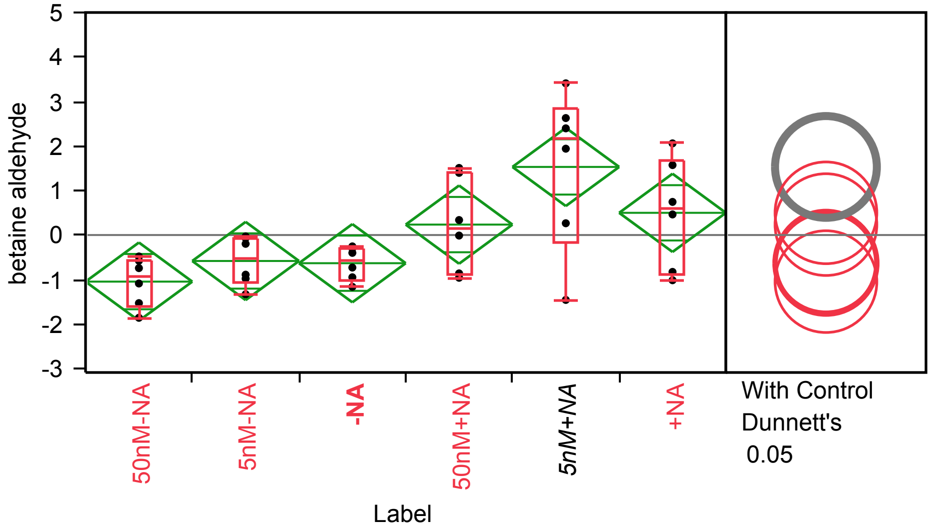

Quantiles

Level	Minimum	10%	25%	Median	75%	90%	Maximum
50nM-NA	-1.85923	-1.85923	-1.61262	-0.91587	-0.55408	-0.48166	-0.48166
5nM-NA	-1.3312	-1.3312	-1.06732	-0.54435	-0.06003	-0.03038	-0.03038
-NA	-1.16369	-1.16369	-1.00305	-0.56581	-0.28751	-0.25555	-0.25555
50nM+NA	-0.96032	-0.96032	-0.88684	0.162134	1.424749	1.506961	1.506961
5nM+NA	-1.45797	-1.45797	-0.16604	2.168534	2.823115	3.40828	3.40828
+NA	-1.01558	-1.01558	-0.87544	0.603374	1.693602	2.059235	2.059235

Oneway Anova

Summary of Fit

Rsquare	0.446639
Adj Rsquare	0.354412
Root Mean Square Error	1.053807
Mean of Response	-5.6e-10
Observations (or Sum Wgts)	36

Analysis of Variance

Source	DF	Sum of Squares	Mean Square	F Ratio	Prob > F
Label	5	26.890027	5.37801	4.8428	0.0023 *
Error	30	33.315265	1.11051		
C. Total	35	60.205292			

Means for Oneway Anova

Level	Number	Mean	Std Error	Lower 95%	Upper 95%
50nM-NA	6	-1.0469	0.43021	-1.925	-0.168
5nM-NA	6	-0.5833	0.43021	-1.462	0.295
-NA	6	-0.6331	0.43021	-1.512	0.246
50nM+NA	6	0.2343	0.43021	-0.644	1.113
5nM+NA	6	1.5300	0.43021	0.651	2.409
+NA	6	0.4989	0.43021	-0.380	1.378

Std Error uses a pooled estimate of error variance

Means Comparisons

Comparisons with a control using Dunnett's Method

Oneway Analysis of Butyrylcarnitine By Label

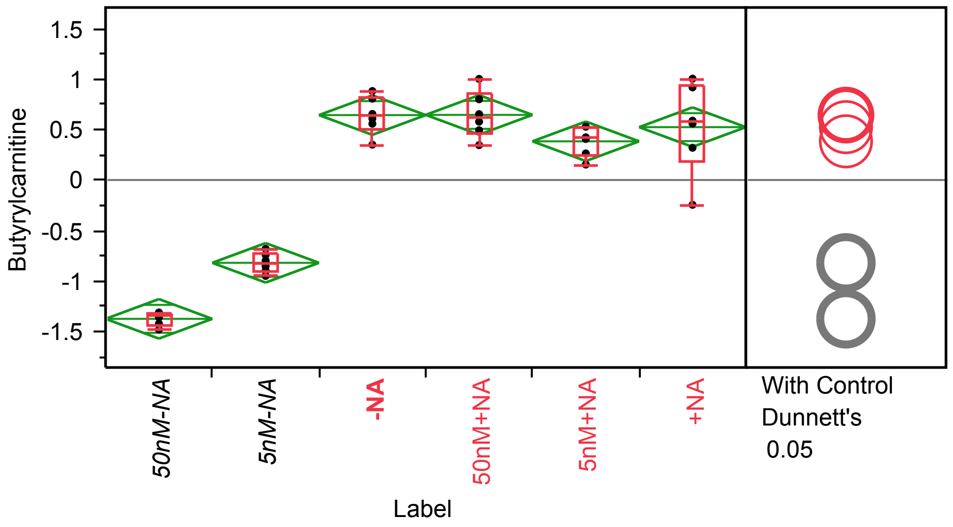

Quantiles

Level	Minimum	10%	25%	Median	75%	90%	Maximum
50nM-NA	-1.4806	-1.4806	-1.43706	-1.34896	-1.32941	-1.31192	-1.31192
5nM-NA	-0.94974	-0.94974	-0.91012	-0.82517	-0.72622	-0.68147	-0.68147
-NA	0.350138	0.350138	0.505764	0.631652	0.824414	0.880772	0.880772
50nM+NA	0.345464	0.345464	0.456256	0.615103	0.85001	1.001311	1.001311
5nM+NA	0.153109	0.153109	0.235646	0.414672	0.52601	0.528238	0.528238
+NA	-0.24528	-0.24528	0.178164	0.573357	0.938773	1.003548	1.003548

Oneway Anova

Summary of Fit

Rsquare	0.932719
Adj Rsquare	0.921505
Root Mean Square Error	0.234523
Mean of Response	5.556e-9
Observations (or Sum Wgts)	36

Analysis of Variance

Source	DF	Sum of Squares	Mean Square	F Ratio	Prob > F
Label	5	22.874243	4.57485	83.1778	<.0001 *
Error	30	1.650026	0.05500		
C. Total	35	24.524269			

Means for Oneway Anova

Level	Number	Mean	Std Error	Lower 95%	Upper 95%
50nM-NA	6	-1.3747	0.09574	-1.570	-1.179
5nM-NA	6	-0.8199	0.09574	-1.015	-0.624
-NA	6	0.6429	0.09574	0.447	0.838
50nM+NA	6	0.6450	0.09574	0.449	0.840
5nM+NA	6	0.3832	0.09574	0.188	0.579
+NA	6	0.5236	0.09574	0.328	0.719

Std Error uses a pooled estimate of error variance

Means Comparisons

Comparisons with a control using Dunnett's Method

Oneway Analysis of Carbamoyl phosphate By Label

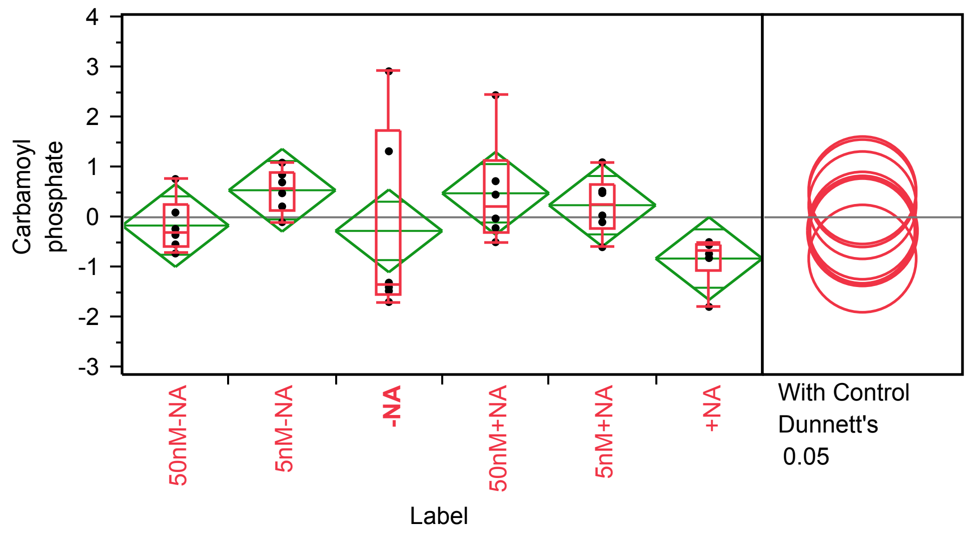

Quantiles

Level	Minimum	10%	25%	Median	75%	90%	Maximum
50nM-NA	-0.72338	-0.72338	-0.58804	-0.29266	0.263032	0.765437	0.765437
5nM-NA	-0.09676	-0.09676	0.13828	0.590331	0.912721	1.09159	1.09159
-NA	-1.69429	-1.69429	-1.52831	-1.35302	1.720078	2.919458	2.919458
50nM+NA	-0.49756	-0.49756	-0.28602	0.214113	1.152137	2.441499	2.441499
5nM+NA	-0.59713	-0.59713	-0.22241	0.260619	0.668512	1.099196	1.099196
+NA	-1.79148	-1.79148	-1.05872	-0.64714	-0.53956	-0.49669	-0.49669

Oneway Anova

Summary of Fit

Rsquare	0.217538
Adj Rsquare	0.087128
Root Mean Square Error	0.990379
Mean of Response	2.78e-10
Observations (or Sum Wgts)	36

Analysis of Variance

Source	DF	Sum of Squares	Mean Square	F Ratio	Prob > F
Label	5	8.180805	1.63616	1.6681	0.1728
Error	30	29.425496	0.98085		
C. Total	35	37.606301			

Means for Oneway Anova

Level	Number	Mean	Std Error	Lower 95%	Upper 95%
50nM-NA	6	-0.16511	0.40432	-0.991	0.6606
5nM-NA	6	0.54087	0.40432	-0.285	1.3666
-NA	6	-0.27226	0.40432	-1.098	0.5535
50nM+NA	6	0.47983	0.40432	-0.346	1.3056
5nM+NA	6	0.24179	0.40432	-0.584	1.0675
+NA	6	-0.82513	0.40432	-1.651	0.0006

Std Error uses a pooled estimate of error variance

Means Comparisons

Comparisons with a control using Dunnett's Method

Oneway Analysis of Carnitine By Label

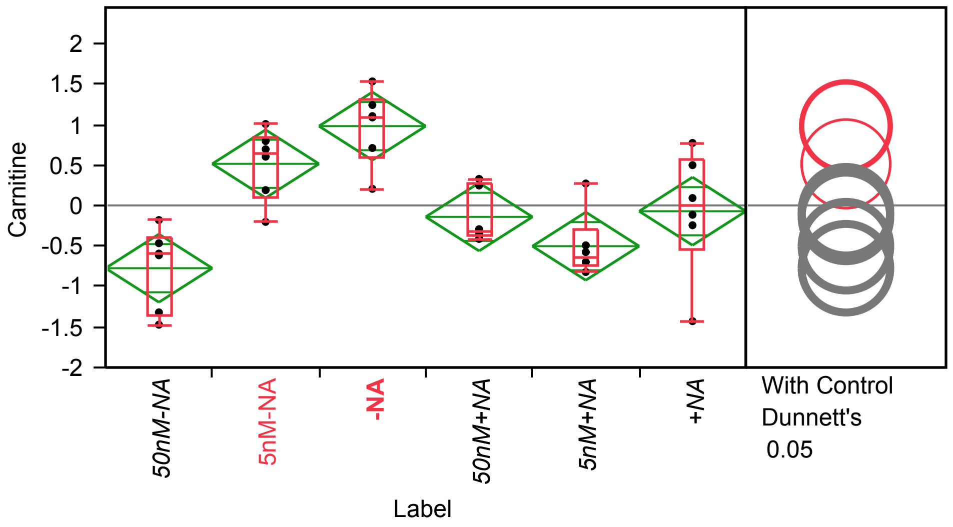

Quantiles

Level	Minimum	10%	25%	Median	75%	90%	Maximum
50nM-NA	-1.47478	-1.47478	-1.3606	-0.60606	-0.396	-0.18135	-0.18135
5nM-NA	-0.20357	-0.20357	0.090758	0.64861	0.848176	1.006336	1.006336
-NA	0.205224	0.205224	0.583619	1.096082	1.314398	1.533388	1.533388
50nM+NA	-0.41446	-0.41446	-0.37907	-0.32211	0.266673	0.327845	0.327845
5nM+NA	-0.82354	-0.82354	-0.73184	-0.63852	-0.30276	0.270665	0.270665
+NA	-1.43128	-1.43128	-0.54178	-0.01236	0.566524	0.772391	0.772391

Oneway Anova

Summary of Fit

Rsquare	0.623278
Adj Rsquare	0.560491
Root Mean Square Error	0.50475
Mean of Response	-1.2e-17
Observations (or Sum Wgts)	36

Analysis of Variance

Source	DF	Sum of Squares	Mean Square	F Ratio	Prob > F
Label	5	12.645458	2.52909	9.9269	<.0001 *
Error	30	7.643172	0.25477		
C. Total	35	20.288630			

Means for Oneway Anova

Level	Number	Mean	Std Error	Lower 95%	Upper 95%
50nM-NA	6	-0.77639	0.20606	-1.197	-0.356
5nM-NA	6	0.51405	0.20606	0.093	0.935
-NA	6	0.98032	0.20606	0.559	1.401
50nM+NA	6	-0.14197	0.20606	-0.563	0.279
5nM+NA	6	-0.50418	0.20606	-0.925	-0.083
+NA	6	-0.07183	0.20606	-0.493	0.349

Std Error uses a pooled estimate of error variance

Means Comparisons

Comparisons with a control using Dunnett's Method

Oneway Analysis of CDP By Label

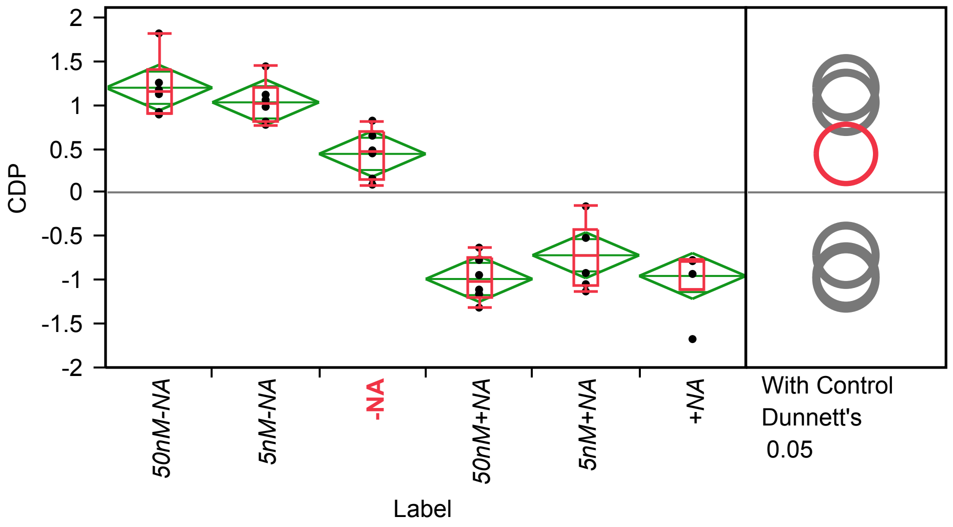

Quantiles

Level	Minimum	10%	25%	Median	75%	90%	Maximum
50nM-NA	0.889531	0.889531	0.910671	1.146803	1.393167	1.815313	1.815313
5nM-NA	0.771454	0.771454	0.799695	1.016577	1.196407	1.439235	1.439235
-NA	0.088101	0.088101	0.13975	0.462671	0.689955	0.818808	0.818808
50nM+NA	-1.31604	-1.31604	-1.20032	-1.02718	-0.73701	-0.6314	-0.6314
5nM+NA	-1.13081	-1.13081	-1.06932	-0.72554	-0.42968	-0.15936	-0.15936
+NA	-1.67547	-1.67547	-1.11753	-0.78555	-0.77561	-0.77305	-0.77305

Oneway Anova

Summary of Fit

Rsquare	0.912414
Adj Rsquare	0.897817
Root Mean Square Error	0.312401
Mean of Response	-4.9e-17
Observations (or Sum Wgts)	36

Analysis of Variance

Source	DF	Sum of Squares	Mean Square	F Ratio	Prob > F
Label	5	30.500336	6.10007	62.5045	<.0001 *
Error	30	2.927822	0.09759		
C. Total	35	33.428158			

Means for Oneway Anova

Level	Number	Mean	Std Error	Lower 95%	Upper 95%
50nM-NA	6	1.1948	0.12754	0.934	1.455
5nM-NA	6	1.0281	0.12754	0.768	1.289
-NA	6	0.4394	0.12754	0.179	0.700
50nM+NA	6	-0.9893	0.12754	-1.250	-0.729
5nM+NA	6	-0.7183	0.12754	-0.979	-0.458
+NA	6	-0.9546	0.12754	-1.215	-0.694

Std Error uses a pooled estimate of error variance

Means Comparisons

Comparisons with a control using Dunnett's Method

Oneway Analysis of Chodroitin By Label

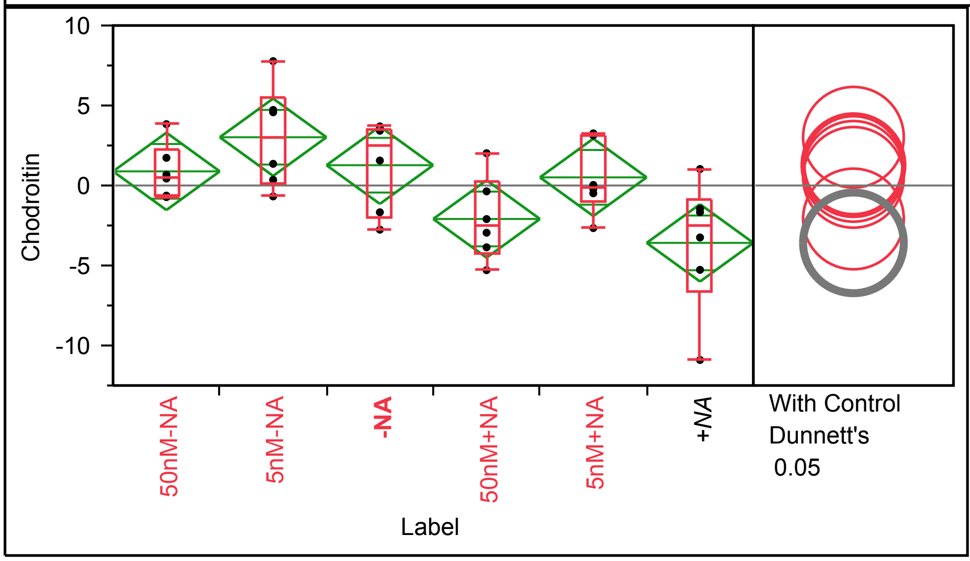

Quantiles

Level	Minimum	10%	25%	Median	75%	90%	Maximum
50nM-NA	-0.72371	-0.72371	-0.6575	0.552296	2.255877	3.83175	3.83175
5nM-NA	-0.68085	-0.68085	0.09562	2.965578	5.48302	7.771999	7.771999
-NA	-2.7605	-2.7605	-1.94561	2.464566	3.490823	3.693914	3.693914
50nM+NA	-5.28955	-5.28955	-4.2195	-2.52503	0.229986	2.016652	2.016652
5nM+NA	-2.66216	-2.66216	-1.03167	-0.09413	3.163831	3.256941	3.256941
+NA	-10.9036	-10.9036	-6.68022	-2.45846	-0.83161	1.028487	1.028487

Oneway Anova

Summary of Fit

Rsquare	0.408135
Adj Rsquare	0.309491
Root Mean Square Error	2.899324
Mean of Response	8.33e-10
Observations (or Sum Wgts)	36

Analysis of Variance

Source	DF	Sum of Squares	Mean Square	F Ratio	Prob > F
Label	5	173.89853	34.7797	4.1374	0.0056 *
Error	30	252.18240	8.4061		
C. Total	35	426.08093			

Means for Oneway Anova

Level	Number	Mean	Std Error	Lower 95%	Upper 95%
50nM-NA	6	0.8846	1.1836	-1.533	3.302
5nM-NA	6	3.0161	1.1836	0.599	5.433
-NA	6	1.2686	1.1836	-1.149	3.686
50nM+NA	6	-2.0919	1.1836	-4.509	0.325
5nM+NA	6	0.5085	1.1836	-1.909	2.926
+NA	6	-3.5860	1.1836	-6.003	-1.169

Std Error uses a pooled estimate of error variance

Means Comparisons

Comparisons with a control using Dunnett's Method

Oneway Analysis of Cholesterol By Label

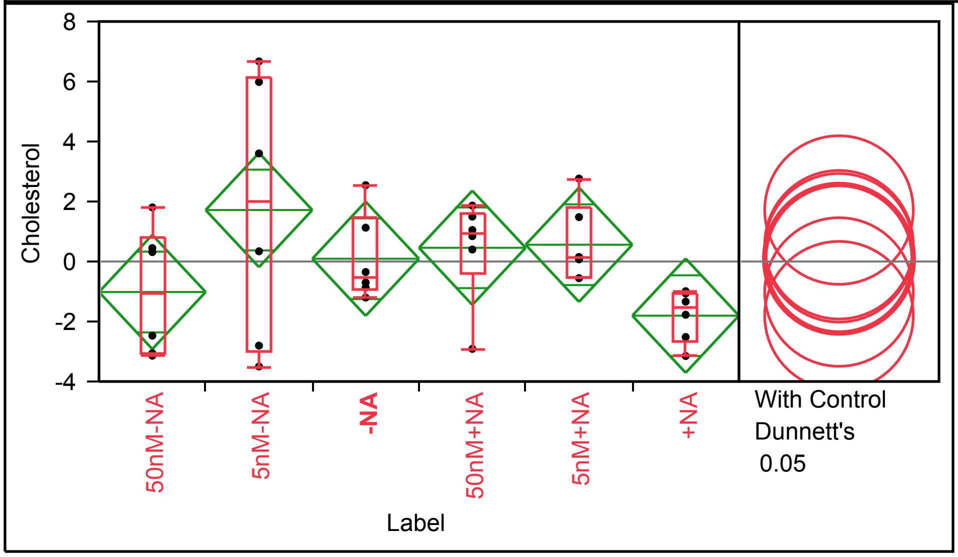

Quantiles

Level	Minimum	10%	25%	Median	75%	90%	Maximum
50nM-NA	-3.13935	-3.13935	-3.07751	-1.07891	0.786302	1.804371	1.804371
5nM-NA	-3.50086	-3.50086	-2.97892	1.971748	6.155661	6.670285	6.670285
-NA	-1.20372	-1.20372	-0.94841	-0.52484	1.480737	2.542551	2.542551
50nM+NA	-2.91207	-2.91207	-0.429	0.947408	1.588432	1.863236	1.863236
5nM+NA	-0.55707	-0.55707	-0.55635	0.111458	1.800672	2.764381	2.764381
+NA	-3.15003	-3.15003	-2.6756	-1.55659	-1.04664	-0.9913	-0.9913

Oneway Anova

Summary of Fit

Rsquare	0.229747
Adj Rsquare	0.101371
Root Mean Square Error	2.282374
Mean of Response	-2.8e-10
Observations (or Sum Wgts)	36

Analysis of Variance

Source	DF	Sum of Squares	Mean Square	F Ratio	Prob > F
Label	5	46.61333	9.32267	1.7896	0.1452
Error	30	156.27695	5.20923		
C. Total	35	202.89028			

Means for Oneway Anova

Level	Number	Mean	Std Error	Lower 95%	Upper 95%
50nM-NA	6	-1.0171	0.93178	-2.920	0.8858
5nM-NA	6	1.7153	0.93178	-0.188	3.6183
-NA	6	0.0921	0.93178	-1.811	1.9950
50nM+NA	6	0.4569	0.93178	-1.446	2.3599
5nM+NA	6	0.5589	0.93178	-1.344	2.4619
+NA	6	-1.8062	0.93178	-3.709	0.0968

Std Error uses a pooled estimate of error variance

Means Comparisons

Comparisons with a control using Dunnett's Method

Oneway Analysis of cholesteryl sulfate By Label

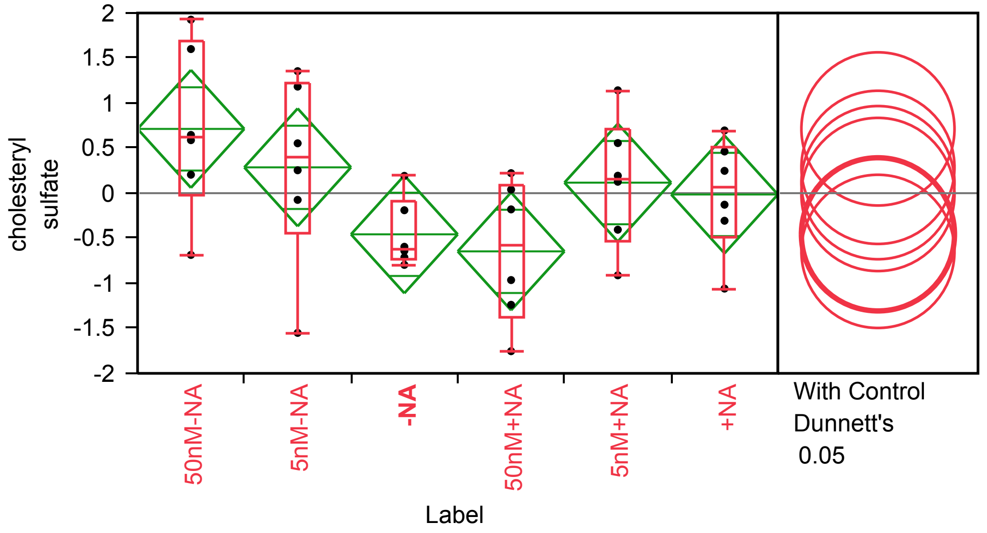

Quantiles

Level	Minimum	10%	25%	Median	75%	90%	Maximum
50nM-NA	-0.68527	-0.68527	-0.01745	0.617489	1.681301	1.925807	1.925807
5nM-NA	-1.55072	-1.55072	-0.44348	0.404022	1.225982	1.354371	1.354371
-NA	-0.79585	-0.79585	-0.73314	-0.61786	-0.09404	0.191973	0.191973
50nM+NA	-1.75393	-1.75393	-1.36792	-0.57224	0.085337	0.222183	0.222183
5nM+NA	-0.91167	-0.91167	-0.53198	0.160353	0.702829	1.141863	1.141863
+NA	-1.05911	-1.05911	-0.49434	0.059999	0.521227	0.696385	0.696385

Oneway Anova

Summary of Fit

Rsquare	0.286089
Adj Rsquare	0.167104
Root Mean Square Error	0.783838
Mean of Response	-2.8e-10
Observations (or Sum Wgts)	36

Analysis of Variance

Source	DF	Sum of Squares	Mean Square	F Ratio	Prob > F
Label	5	7.386376	1.47728	2.4044	0.0601
Error	30	18.432074	0.61440		
C. Total	35	25.818450			

Means for Oneway Anova

Level	Number	Mean	Std Error	Lower 95%	Upper 95%
50nM-NA	6	0.71341	0.32000	0.060	1.3669
5nM-NA	6	0.28675	0.32000	-0.367	0.9403
-NA	6	-0.45687	0.32000	-1.110	0.1967
50nM+NA	6	-0.64596	0.32000	-1.299	0.0076
5nM+NA	6	0.11700	0.32000	-0.537	0.7705
+NA	6	-0.01433	0.32000	-0.668	0.6392

Std Error uses a pooled estimate of error variance

Means Comparisons

Comparisons with a control using Dunnett's Method

Oneway Analysis of choline By Label

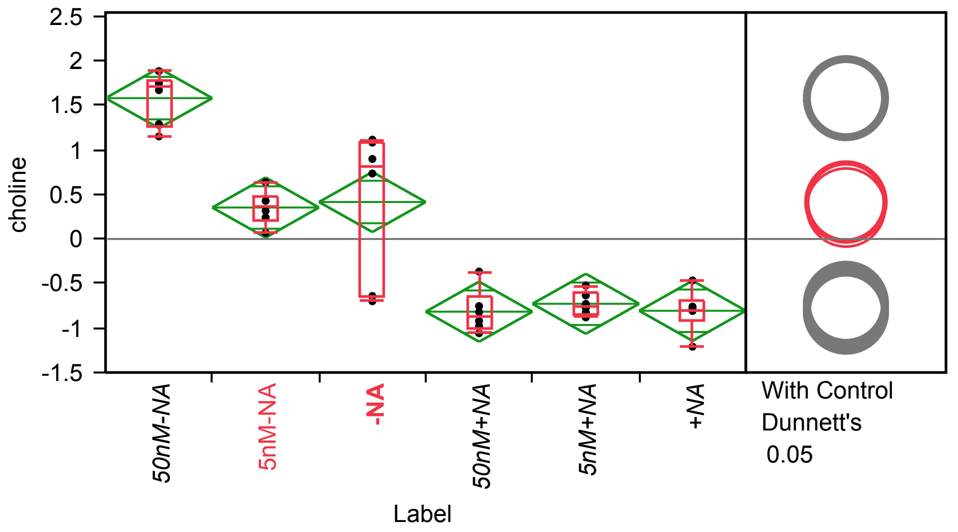

Quantiles

Level	Minimum	10%	25%	Median	75%	90%	Maximum
50nM-NA	1.152442	1.152442	1.256047	1.707787	1.783786	1.884712	1.884712
5nM-NA	0.069399	0.069399	0.199319	0.37165	0.480433	0.637045	0.637045
-NA	-0.69928	-0.69928	-0.65573	0.817221	1.091129	1.115936	1.115936
50nM+NA	-1.05872	-1.05872	-0.99915	-0.87084	-0.65697	-0.36627	-0.36627
5nM+NA	-0.88072	-0.88072	-0.83929	-0.75276	-0.60584	-0.52273	-0.52273
+NA	-1.2097	-1.2097	-0.91347	-0.79484	-0.68594	-0.46539	-0.46539

Oneway Anova

Summary of Fit

Rsquare	0.850268
Adj Rsquare	0.825313
Root Mean Square Error	0.404615
Mean of Response	-4.9e-17
Observations (or Sum Wgts)	36

Analysis of Variance

Source	DF	Sum of Squares	Mean Square	F Ratio	Prob > F
Label	5	27.889957	5.57799	34.0717	<.0001 *
Error	30	4.911400	0.16371		
C. Total	35	32.801357			

Means for Oneway Anova

Level	Number	Mean	Std Error	Lower 95%	Upper 95%
50nM-NA	6	1.5822	0.16518	1.245	1.920
5nM-NA	6	0.3534	0.16518	0.016	0.691
-NA	6	0.4155	0.16518	0.078	0.753
50nM+NA	6	-0.8166	0.16518	-1.154	-0.479
5nM+NA	6	-0.7280	0.16518	-1.065	-0.391
+NA	6	-0.8065	0.16518	-1.144	-0.469

Std Error uses a pooled estimate of error variance

Means Comparisons

Comparisons with a control using Dunnett's Method

Oneway Analysis of Citric acid By Label

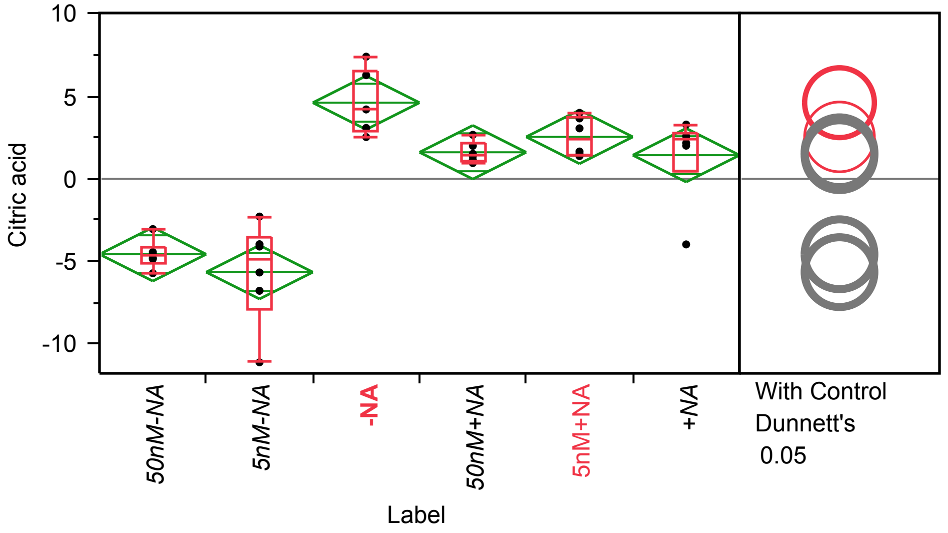

Quantiles

Level	Minimum	10%	25%	Median	75%	90%	Maximum
50nM-NA	-5.71717	-5.71717	-5.06998	-4.67751	-4.08585	-3.04003	-3.04003
5nM-NA	-11.1162	-11.1162	-7.86419	-4.88505	-3.54019	-2.28341	-2.28341
-NA	2.538062	2.538062	2.952211	4.196627	6.56697	7.408101	7.408101
50nM+NA	0.960409	0.960409	1.106328	1.4476	2.170744	2.673071	2.673071
5nM+NA	1.384904	1.384904	1.471934	2.362255	3.751125	4.002758	4.002758
+NA	-3.97016	-3.97016	0.518535	2.350975	2.763634	3.292468	3.292468

Oneway Anova

Summary of Fit

Rsquare	0.817739
Adj Rsquare	0.787362
Root Mean Square Error	1.949902
Mean of Response	2.778e-9
Observations (or Sum Wgts)	36

Analysis of Variance

Source	DF	Sum of Squares	Mean Square	F Ratio	Prob > F
Label	5	511.76104	102.352	26.9198	<.0001 *
Error	30	114.06349	3.802		
C. Total	35	625.82453			

Means for Oneway Anova

Level	Number	Mean	Std Error	Lower 95%	Upper 95%
50nM-NA	6	-4.5668	0.79604	-6.193	-2.941
5nM-NA	6	-5.6515	0.79604	-7.277	-4.026
-NA	6	4.6194	0.79604	2.994	6.245
50nM+NA	6	1.6145	0.79604	-0.011	3.240
5nM+NA	6	2.5467	0.79604	0.921	4.172
+NA	6	1.4377	0.79604	-0.188	3.063

Std Error uses a pooled estimate of error variance

Means Comparisons

Comparisons with a control using Dunnett's Method

Oneway Analysis of citrulline By Label

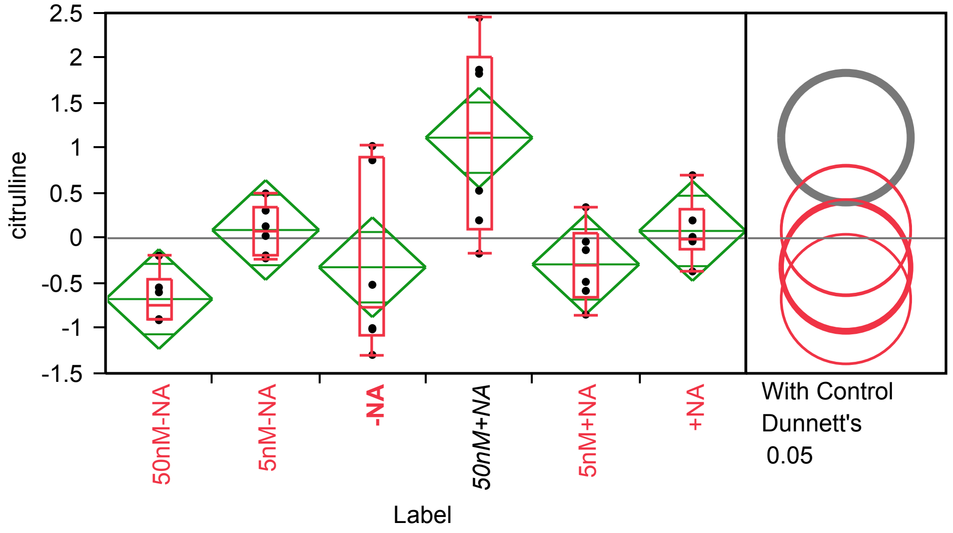

Quantiles

Level	Minimum	10%	25%	Median	75%	90%	Maximum
50nM-NA	-0.90986	-0.90986	-0.90518	-0.74935	-0.45876	-0.19519	-0.19519
5nM-NA	-0.22348	-0.22348	-0.19939	0.078916	0.354267	0.497012	0.497012
-NA	-1.29531	-1.29531	-1.08198	-0.75746	0.905208	1.023297	1.023297
50nM+NA	-0.17078	-0.17078	0.106103	1.176679	2.013871	2.446711	2.446711
5nM+NA	-0.84803	-0.84803	-0.65009	-0.30798	0.057118	0.343922	0.343922
+NA	-0.36866	-0.36866	-0.1184	-0.00403	0.322802	0.698915	0.698915

Oneway Anova

Summary of Fit

Rsquare	0.464457
Adj Rsquare	0.3752
Root Mean Square Error	0.662862
Mean of Response	-8.3e-10
Observations (or Sum Wgts)	36

Analysis of Variance

Source	DF	Sum of Squares	Mean Square	F Ratio	Prob > F
Label	5	11.431912	2.28638	5.2036	0.0015 *
Error	30	13.181593	0.43939		
C. Total	35	24.613505			

Means for Oneway Anova

Level	Number	Mean	Std Error	Lower 95%	Upper 95%
50nM-NA	6	-0.6757	0.27061	-1.228	-0.123
5nM-NA	6	0.0911	0.27061	-0.462	0.644
-NA	6	-0.3220	0.27061	-0.875	0.231
50nM+NA	6	1.1162	0.27061	0.564	1.669
5nM+NA	6	-0.2904	0.27061	-0.843	0.262
+NA	6	0.0808	0.27061	-0.472	0.633

Std Error uses a pooled estimate of error variance

Means Comparisons

Comparisons with a control using Dunnett's Method

Oneway Analysis of CMP By Label

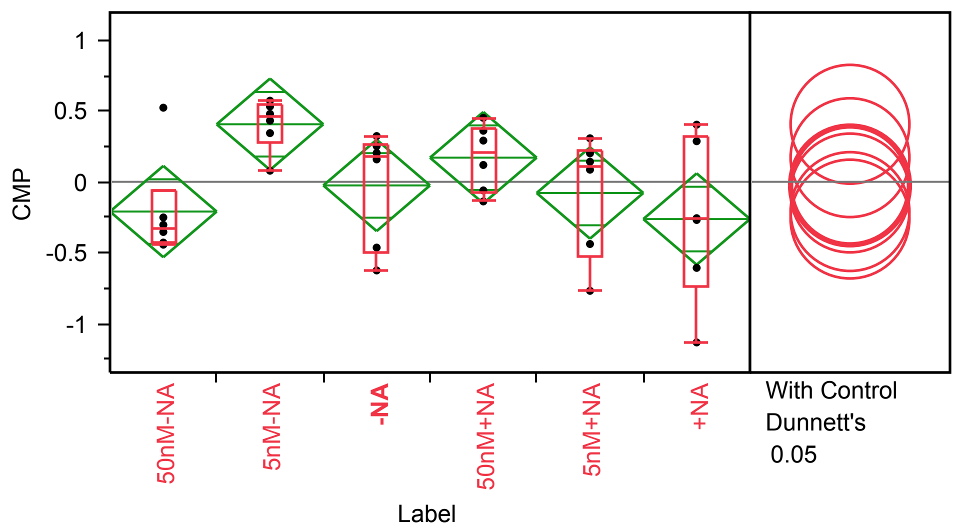

Quantiles

Level	Minimum	10%	25%	Median	75%	90%	Maximum
50nM-NA	-0.44336	-0.44336	-0.4324	-0.32869	-0.05787	0.522832	0.522832
5nM-NA	0.078505	0.078505	0.277245	0.455541	0.54168	0.57196	0.57196
-NA	-0.62498	-0.62498	-0.50438	0.180933	0.269818	0.322696	0.322696
50nM+NA	-0.13822	-0.13822	-0.08006	0.204821	0.382974	0.451212	0.451212
5nM+NA	-0.76892	-0.76892	-0.52134	0.113627	0.227635	0.307017	0.307017
+NA	-1.13277	-1.13277	-0.73844	-0.26281	0.315586	0.402851	0.402851

Oneway Anova

Summary of Fit

Rsquare	0.296009
Adj Rsquare	0.178678
Root Mean Square Error	0.386311
Mean of Response	6.17e-18
Observations (or Sum Wgts)	36

Analysis of Variance

Source	DF	Sum of Squares	Mean Square	F Ratio	Prob > F
Label	5	1.8824914	0.376498	2.5228	0.0508
Error	30	4.4770746	0.149236		
C. Total	35	6.3595660			

Means for Oneway Anova

Level	Number	Mean	Std Error	Lower 95%	Upper 95%
50nM-NA	6	-0.20968	0.15771	-0.5318	0.11241
5nM-NA	6	0.40610	0.15771	0.0840	0.72819
-NA	6	-0.02540	0.15771	-0.3475	0.29669
50nM+NA	6	0.17036	0.15771	-0.1517	0.49245
5nM+NA	6	-0.07871	0.15771	-0.4008	0.24337
+NA	6	-0.26267	0.15771	-0.5848	0.05942

Std Error uses a pooled estimate of error variance

Means Comparisons

Comparisons with a control using Dunnett's Method

Oneway Analysis of CMP-N-acetylneuraminic acid By Label

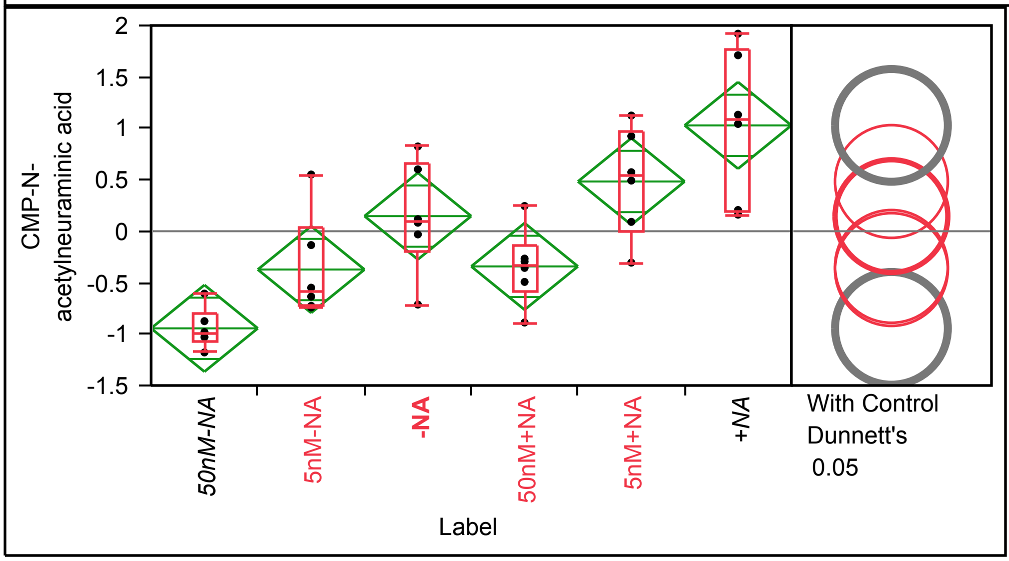

Quantiles

Level	Minimum	10%	25%	Median	75%	90%	Maximum
50nM-NA	-1.17831	-1.17831	-1.06693	-0.98796	-0.80756	-0.60682	-0.60682
5nM-NA	-0.74113	-0.74113	-0.72949	-0.59233	0.036708	0.551158	0.551158
-NA	-0.71312	-0.71312	-0.20157	0.099652	0.657117	0.823907	0.823907
50nM+NA	-0.88769	-0.88769	-0.59159	-0.32557	-0.13863	0.244819	0.244819
5nM+NA	-0.30541	-0.30541	-0.00814	0.532928	0.974414	1.12443	1.12443
+NA	0.161075	0.161075	0.194851	1.087396	1.763168	1.920053	1.920053

Oneway Anova

Summary of Fit

Rsquare	0.658446
Adj Rsquare	0.60152
Root Mean Square Error	0.505298
Mean of Response	-2.8e-10
Observations (or Sum Wgts)	36

Analysis of Variance

Source	DF	Sum of Squares	Mean Square	F Ratio	Prob > F
Label	5	14.766482	2.95330	11.5668	<.0001 *
Error	30	7.659789	0.25533		
C. Total	35	22.426271			

Means for Oneway Anova

Level	Number	Mean	Std Error	Lower 95%	Upper 95%
50nM-NA	6	-0.9442	0.20629	-1.366	-0.523
5nM-NA	6	-0.3725	0.20629	-0.794	0.049
-NA	6	0.1468	0.20629	-0.275	0.568
50nM+NA	6	-0.3422	0.20629	-0.764	0.079
5nM+NA	6	0.4834	0.20629	0.062	0.905
+NA	6	1.0288	0.20629	0.608	1.450

Std Error uses a pooled estimate of error variance

Means Comparisons

Comparisons with a control using Dunnett's Method

Oneway Analysis of creatine By Label

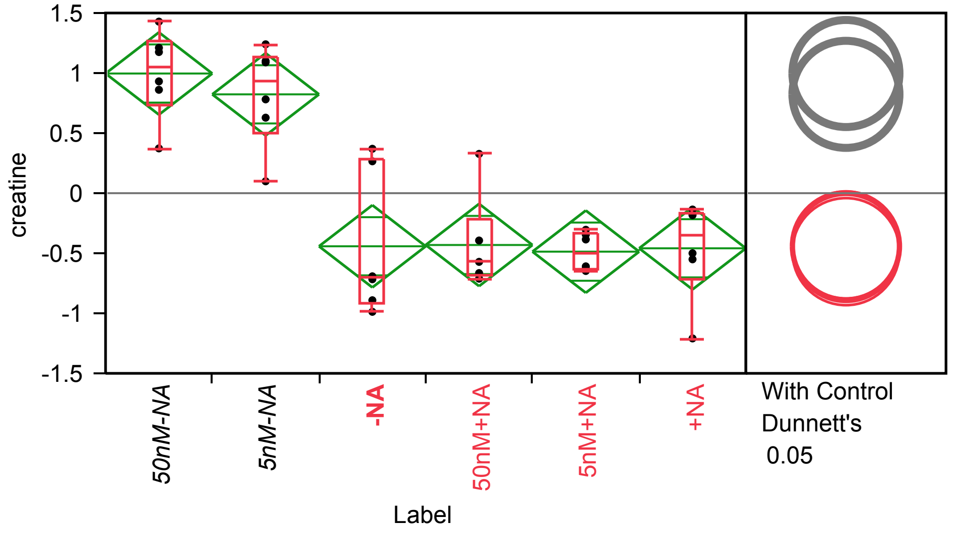

Quantiles

Level	Minimum	10%	25%	Median	75%	90%	Maximum
50nM-NA	0.372953	0.372953	0.738812	1.052888	1.264776	1.428074	1.428074
5nM-NA	0.099008	0.099008	0.496172	0.935113	1.134949	1.239472	1.239472
-NA	-0.98767	-0.98767	-0.91552	-0.70439	0.291499	0.369515	0.369515
50nM+NA	-0.71178	-0.71178	-0.67692	-0.57204	-0.21376	0.327054	0.327054
5nM+NA	-0.6476	-0.6476	-0.63287	-0.49729	-0.33524	-0.30488	-0.30488
+NA	-1.20921	-1.20921	-0.7153	-0.34167	-0.16488	-0.13618	-0.13618

Oneway Anova

Summary of Fit

Rsquare	0.747494
Adj Rsquare	0.70541
Root Mean Square Error	0.410889
Mean of Response	-2.8e-10
Observations (or Sum Wgts)	36

Analysis of Variance

Source	DF	Sum of Squares	Mean Square	F Ratio	Prob > F
Label	5	14.993634	2.99873	17.7618	<.0001 *
Error	30	5.064899	0.16883		
C. Total	35	20.058533			

Means for Oneway Anova

Level	Number	Mean	Std Error	Lower 95%	Upper 95%
50nM-NA	6	0.99632	0.16774	0.6537	1.339
5nM-NA	6	0.82290	0.16774	0.4803	1.165
-NA	6	-0.44215	0.16774	-0.7847	-0.100
50nM+NA	6	-0.43136	0.16774	-0.7739	-0.089
5nM+NA	6	-0.48673	0.16774	-0.8293	-0.144
+NA	6	-0.45897	0.16774	-0.8016	-0.116

Std Error uses a pooled estimate of error variance

Means Comparisons

Comparisons with a control using Dunnett's Method

Oneway Analysis of Creatinine By Label

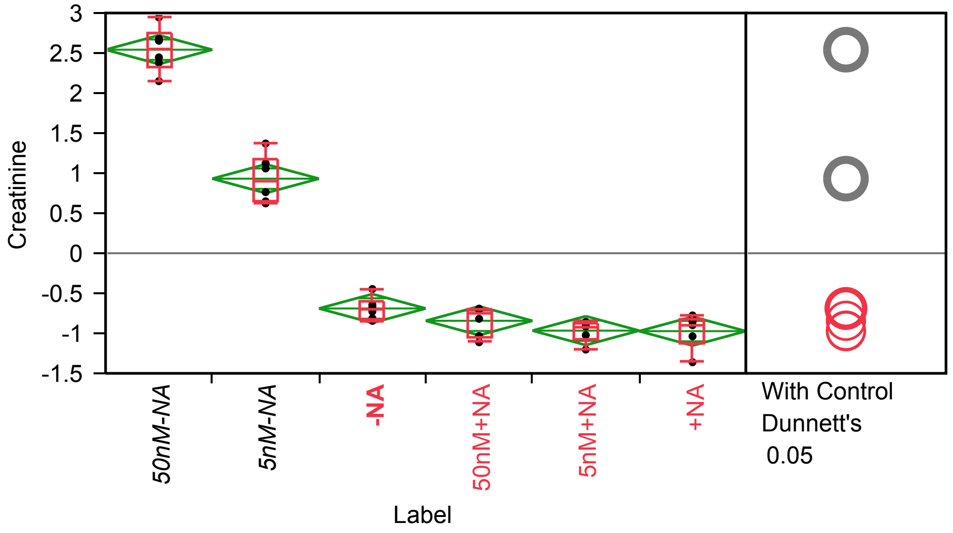

Quantiles

Level	Minimum	10%	25%	Median	75%	90%	Maximum
50nM-NA	2.149003	2.149003	2.322406	2.549877	2.743573	2.944872	2.944872
5nM-NA	0.623025	0.623025	0.641936	0.910877	1.185674	1.369927	1.369927
-NA	-0.84424	-0.84424	-0.82052	-0.69685	-0.59257	-0.44716	-0.44716
50nM+NA	-1.11127	-1.11127	-1.05337	-0.7623	-0.69769	-0.6918	-0.6918
5nM+NA	-1.2029	-1.2029	-1.06795	-0.91906	-0.87312	-0.85722	-0.85722
+NA	-1.36118	-1.36118	-1.11865	-0.9037	-0.83506	-0.77686	-0.77686

Oneway Anova

Summary of Fit

Rsquare	0.977955
Adj Rsquare	0.974281
Root Mean Square Error	0.21649
Mean of Response	0
Observations (or Sum Wgts)	36

Analysis of Variance

Source	DF	Sum of Squares	Mean Square	F Ratio	Prob > F
Label	5	62.375091	12.4750	266.1742	<.0001 *
Error	30	1.406036	0.0469		
C. Total	35	63.781127			

Means for Oneway Anova

Level	Number	Mean	Std Error	Lower 95%	Upper 95%
50nM-NA	6	2.5417	0.08838	2.361	2.722
5nM-NA	6	0.9312	0.08838	0.751	1.112
-NA	6	-0.6898	0.08838	-0.870	-0.509
50nM+NA	6	-0.8436	0.08838	-1.024	-0.663
5nM+NA	6	-0.9666	0.08838	-1.147	-0.786
+NA	6	-0.9730	0.08838	-1.153	-0.792

Std Error uses a pooled estimate of error variance

Means Comparisons

Comparisons with a control using Dunnett's Method

Oneway Analysis of Cyclic ADP-ribose By Label

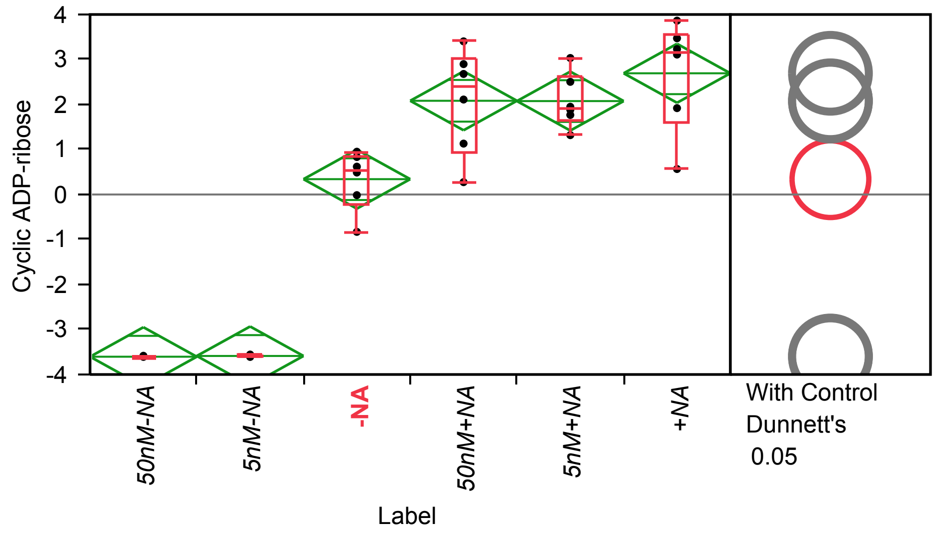

Quantiles

Level	Minimum	10%	25%	Median	75%	90%	Maximum
50nM-NA	-3.62967	-3.62967	-3.61519	-3.6051	-3.59169	-3.58421	-3.58421
5nM-NA	-3.60934	-3.60934	-3.60321	-3.59017	-3.57512	-3.55673	-3.55673
-NA	-0.83012	-0.83012	-0.21837	0.553417	0.862362	0.950427	0.950427
50nM+NA	0.274608	0.274608	0.916792	2.394458	3.024057	3.403034	3.403034
5nM+NA	1.319347	1.319347	1.655421	1.916125	2.635789	3.03296	3.03296
+NA	0.566487	0.566487	1.581244	3.1702	3.56968	3.862899	3.862899

Oneway Anova

Summary of Fit

Rsquare	0.931334
Adj Rsquare	0.919889
Root Mean Square Error	0.785911
Mean of Response	-2.8e-10
Observations (or Sum Wgts)	36

Analysis of Variance

Source	DF	Sum of Squares	Mean Square	F Ratio	Prob > F
Label	5	251.32062	50.2641	81.3789	<.0001 *
Error	30	18.52967	0.6177		
C. Total	35	269.85029			

Means for Oneway Anova

Level	Number	Mean	Std Error	Lower 95%	Upper 95%
50nM-NA	6	-3.6048	0.32085	-4.260	-2.950
5nM-NA	6	-3.5881	0.32085	-4.243	-2.933
-NA	6	0.3409	0.32085	-0.314	0.996
50nM+NA	6	2.0825	0.32085	1.427	2.738
5nM+NA	6	2.0759	0.32085	1.421	2.731
+NA	6	2.6935	0.32085	2.038	3.349

Std Error uses a pooled estimate of error variance

Means Comparisons

Comparisons with a control using Dunnett's Method

Oneway Analysis of cyclic-AMP By Label

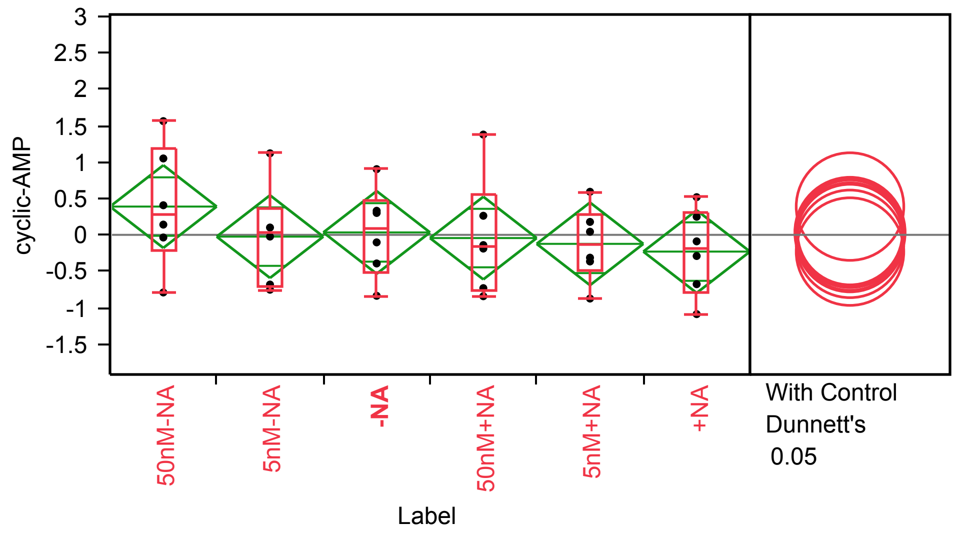

Quantiles

Level	Minimum	10%	25%	Median	75%	90%	Maximum
50nM-NA	-0.7924	-0.7924	-0.22546	0.274079	1.179531	1.56446	1.56446
5nM-NA	-0.75475	-0.75475	-0.70167	0.035191	0.356083	1.12194	1.12194
-NA	-0.83846	-0.83846	-0.50483	0.099028	0.474284	0.903911	0.903911
50nM+NA	-0.84435	-0.84435	-0.76106	-0.16485	0.541787	1.379563	1.379563
5nM+NA	-0.87621	-0.87621	-0.49364	-0.13576	0.281597	0.592479	0.592479
+NA	-1.09056	-1.09056	-0.78156	-0.19008	0.316559	0.518319	0.518319

Oneway Anova

Summary of Fit

Rsquare	0.087609
Adj Rsquare	-0.06446
Root Mean Square Error	0.682273
Mean of Response	-5.6e-10
Observations (or Sum Wgts)	36

Analysis of Variance

Source	DF	Sum of Squares	Mean Square	F Ratio	Prob > F
Label	5	1.340929	0.268186	0.5761	0.7178
Error	30	13.964901	0.465497		
C. Total	35	15.305829			

Means for Oneway Anova

Level	Number	Mean	Std Error	Lower 95%	Upper 95%
50nM-NA	6	0.38916	0.27854	-0.1797	0.95801
5nM-NA	6	-0.02427	0.27854	-0.5931	0.54458
-NA	6	0.03349	0.27854	-0.5354	0.60234
50nM+NA	6	-0.04421	0.27854	-0.6131	0.52464
5nM+NA	6	-0.12390	0.27854	-0.6927	0.44495
+NA	6	-0.23028	0.27854	-0.7991	0.33857

Std Error uses a pooled estimate of error variance

Means Comparisons

Comparisons with a control using Dunnett's Method

Oneway Analysis of cystathionine By Label

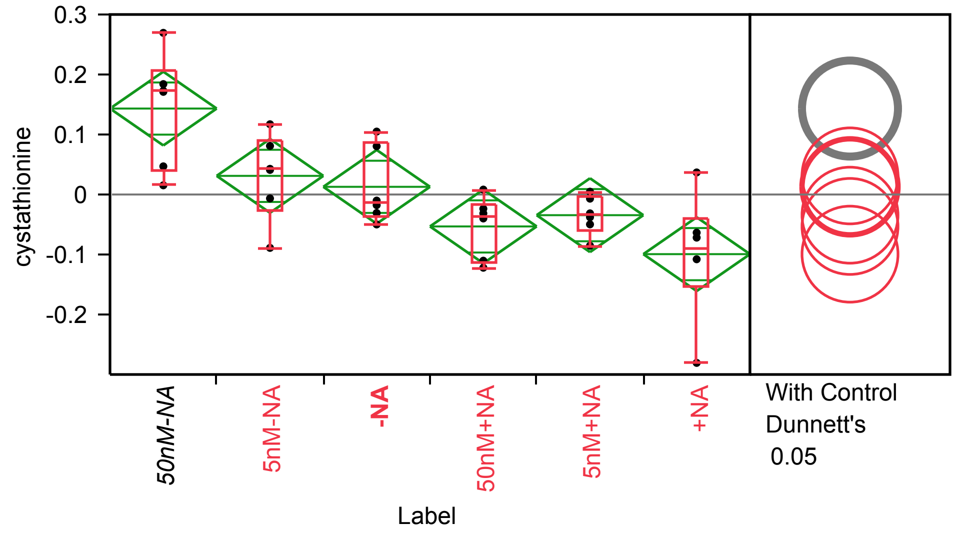

Quantiles

Level	Minimum	10%	25%	Median	75%	90%	Maximum
50nM-NA	0.015326	0.015326	0.039014	0.171939	0.205333	0.269409	0.269409
5nM-NA	-0.08896	-0.08896	-0.02711	0.041859	0.089989	0.117062	0.117062
-NA	-0.04988	-0.04988	-0.03557	-0.01395	0.086577	0.104832	0.104832
50nM+NA	-0.12203	-0.12203	-0.11327	-0.03569	-0.01575	0.008136	0.008136
5nM+NA	-0.0854	-0.0854	-0.05867	-0.03467	-0.00402	0.004678	0.004678
+NA	-0.28061	-0.28061	-0.15206	-0.08989	-0.03851	0.036737	0.036737

Oneway Anova

Summary of Fit

Rsquare	0.566611
Adj Rsquare	0.494379
Root Mean Square Error	0.073745
Mean of Response	3.08e-18
Observations (or Sum Wgts)	36

Analysis of Variance

Source	DF	Sum of Squares	Mean Square	F Ratio	Prob > F
Label	5	0.21330145	0.042660	7.8444	<.0001 *
Error	30	0.16315007	0.005438		
C. Total	35	0.37645152			

Means for Oneway Anova

Level	Number	Mean	Std Error	Lower 95%	Upper 95%
50nM-NA	6	0.14325	0.03011	0.0818	0.2047
5nM-NA	6	0.03105	0.03011	-0.0304	0.0925
-NA	6	0.01279	0.03011	-0.0487	0.0743
50nM+NA	6	-0.05322	0.03011	-0.1147	0.0083
5nM+NA	6	-0.03446	0.03011	-0.0959	0.0270
+NA	6	-0.09941	0.03011	-0.1609	-0.0379

Std Error uses a pooled estimate of error variance

Means Comparisons

Comparisons with a control using Dunnett's Method

Oneway Analysis of Cysteineglutathione disulfide By Label

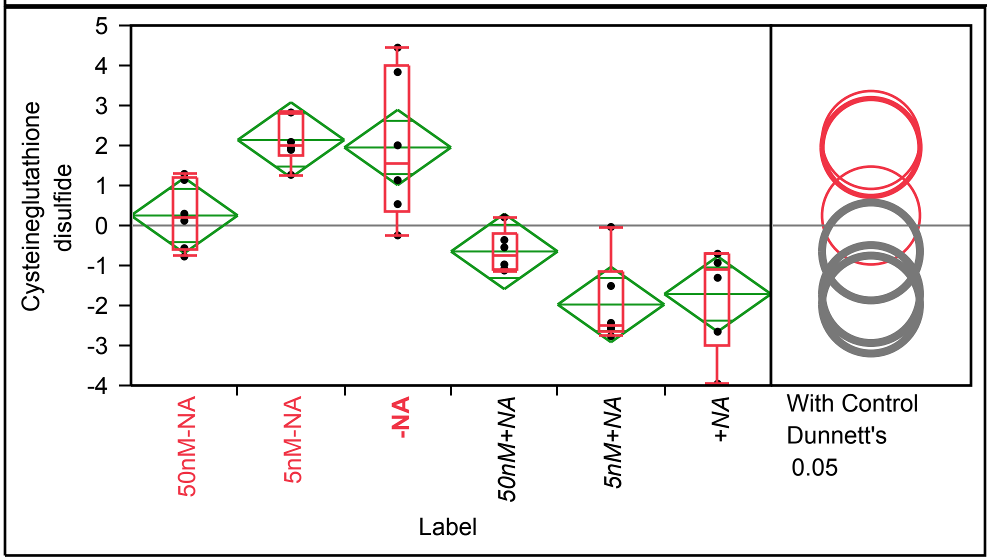

Quantiles

Level	Minimum	10%	25%	Median	75%	90%	Maximum
50nM-NA	-0.77489	-0.77489	-0.61994	0.206601	1.176197	1.289853	1.289853
5nM-NA	1.265815	1.265815	1.734166	2.017103	2.816797	2.825021	2.825021
-NA	-0.24753	-0.24753	0.338674	1.568538	3.987306	4.445479	4.445479
50nM+NA	-1.12537	-1.12537	-1.09568	-0.7608	-0.22153	0.204056	0.204056
5nM+NA	-2.76485	-2.76485	-2.62585	-2.48188	-1.14272	-0.03686	-0.03686
+NA	-3.95614	-3.95614	-2.98058	-1.12086	-0.71881	-0.70678	-0.70678

Oneway Anova

Summary of Fit

Rsquare	0.711562
Adj Rsquare	0.663489
Root Mean Square Error	1.128267
Mean of Response	-5.6e-10
Observations (or Sum Wgts)	36

Analysis of Variance

Source	DF	Sum of Squares	Mean Square	F Ratio	Prob > F
Label	5	94.21189	18.8424	14.8017	<.0001 *
Error	30	38.18959	1.2730		
C. Total	35	132.40148			

Means for Oneway Anova

Level	Number	Mean	Std Error	Lower 95%	Upper 95%
50nM-NA	6	0.2497	0.46061	-0.691	1.190
5nM-NA	6	2.1382	0.46061	1.198	3.079
-NA	6	1.9506	0.46061	1.010	2.891
50nM+NA	6	-0.6487	0.46061	-1.589	0.292
5nM+NA	6	-1.9761	0.46061	-2.917	-1.035
+NA	6	-1.7138	0.46061	-2.655	-0.773

Std Error uses a pooled estimate of error variance

Means Comparisons

Comparisons with a control using Dunnett's Method

Oneway Analysis of Cystine By Label

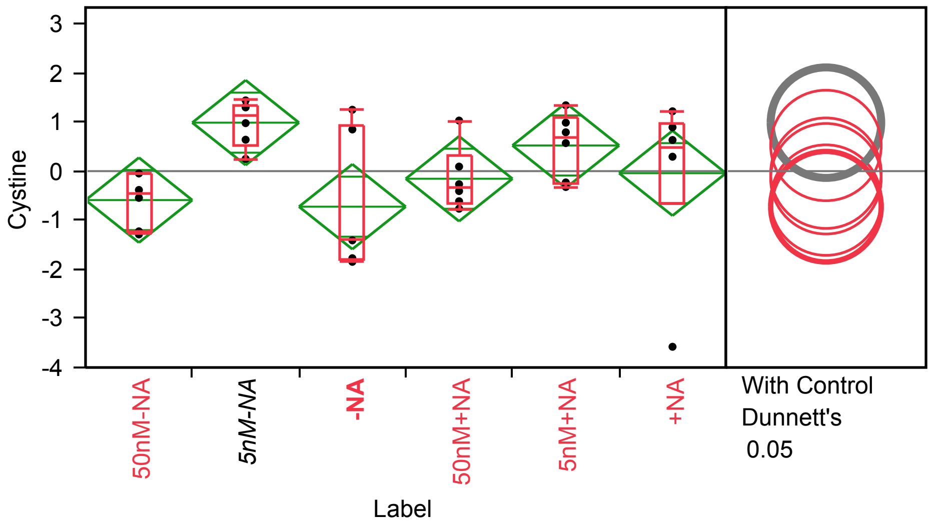

Quantiles

Level	Minimum	10%	25%	Median	75%	90%	Maximum
50nM-NA	-1.28865	-1.28865	-1.24226	-0.46368	-0.04934	-0.04735	-0.04735
5nM-NA	0.249069	0.249069	0.542096	1.138155	1.350061	1.440672	1.440672
-NA	-1.84747	-1.84747	-1.79258	-1.41105	0.949596	1.249834	1.249834
50nM+NA	-0.76535	-0.76535	-0.65144	-0.33599	0.32668	1.029773	1.029773
5nM+NA	-0.32295	-0.32295	-0.25338	0.681295	1.075681	1.341417	1.341417
+NA	-3.578	-3.578	-0.67831	0.463627	0.974654	1.219046	1.219046

Oneway Anova

Summary of Fit

Rsquare	0.285138
Adj Rsquare	0.165994
Root Mean Square Error	1.037571
Mean of Response	5.56e-10
Observations (or Sum Wgts)	36

Analysis of Variance

Source	DF	Sum of Squares	Mean Square	F Ratio	Prob > F
Label	5	12.882190	2.57644	2.3932	0.0611
Error	30	32.296599	1.07655		
C. Total	35	45.178788			

Means for Oneway Anova

Level	Number	Mean	Std Error	Lower 95%	Upper 95%
50nM-NA	6	-0.59003	0.42359	-1.455	0.2751
5nM-NA	6	0.98761	0.42359	0.123	1.8527
-NA	6	-0.72408	0.42359	-1.589	0.1410
50nM+NA	6	-0.15479	0.42359	-1.020	0.7103
5nM+NA	6	0.52299	0.42359	-0.342	1.3881
+NA	6	-0.04171	0.42359	-0.907	0.8234

Std Error uses a pooled estimate of error variance

Means Comparisons

Comparisons with a control using Dunnett's Method

Oneway Analysis of cytidine By Label

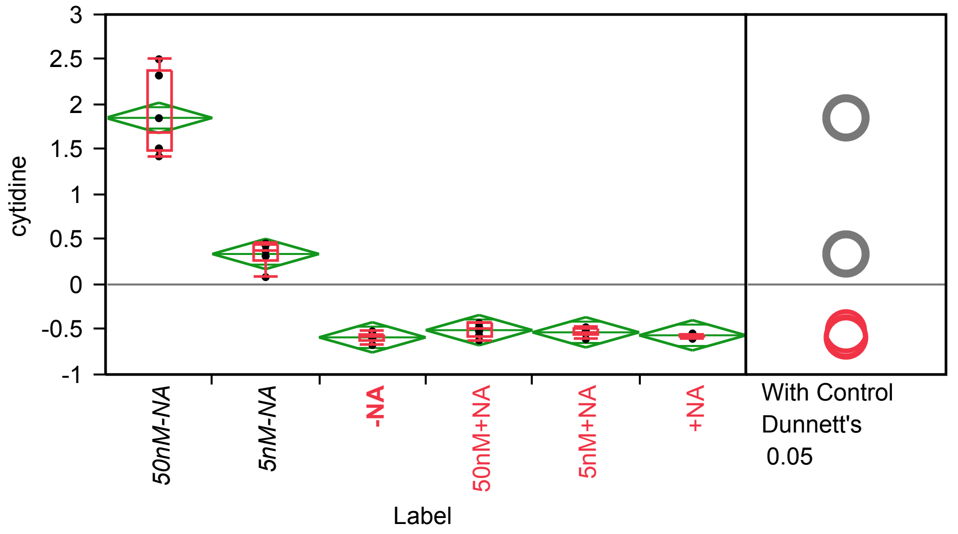

Quantiles

Level	Minimum	10%	25%	Median	75%	90%	Maximum
50nM-NA	1.423975	1.423975	1.480996	1.679897	2.367293	2.50255	2.50255
5nM-NA	0.081869	0.081869	0.256745	0.373236	0.44003	0.457015	0.457015
-NA	-0.6719	-0.6719	-0.61997	-0.58274	-0.55301	-0.52022	-0.52022
50nM+NA	-0.62108	-0.62108	-0.58068	-0.49654	-0.43263	-0.42922	-0.42922
5nM+NA	-0.60727	-0.60727	-0.56413	-0.53175	-0.48832	-0.47506	-0.47506
+NA	-0.60317	-0.60317	-0.5824	-0.55897	-0.54508	-0.54457	-0.54457

Oneway Anova

Summary of Fit

Rsquare	0.959084
Adj Rsquare	0.952264
Root Mean Square Error	0.201236
Mean of Response	5.56e-10
Observations (or Sum Wgts)	36

Analysis of Variance

Source	DF	Sum of Squares	Mean Square	F Ratio	Prob > F
Label	5	28.476822	5.69536	140.6408	<.0001 *
Error	30	1.214874	0.04050		
C. Total	35	29.691696			

Means for Oneway Anova

Level	Number	Mean	Std Error	Lower 95%	Upper 95%
50nM-NA	6	1.8514	0.08215	1.684	2.019
5nM-NA	6	0.3391	0.08215	0.171	0.507
-NA	6	-0.5874	0.08215	-0.755	-0.420
50nM+NA	6	-0.5074	0.08215	-0.675	-0.340
5nM+NA	6	-0.5314	0.08215	-0.699	-0.364
+NA	6	-0.5644	0.08215	-0.732	-0.397

Std Error uses a pooled estimate of error variance

Means Comparisons

Comparisons with a control using Dunnett's Method

Oneway Analysis of cytosine By Label

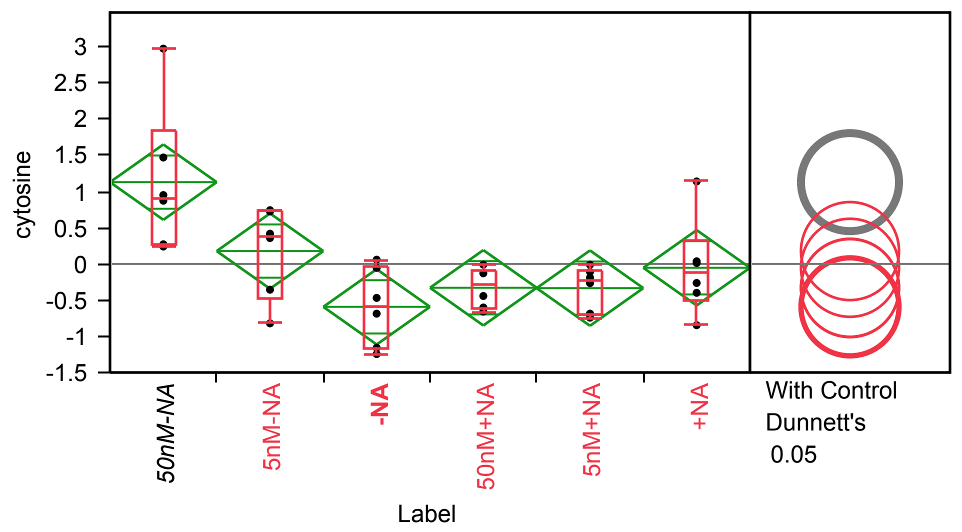

Quantiles

Level	Minimum	10%	25%	Median	75%	90%	Maximum
50nM-NA	0.240578	0.240578	0.263835	0.912864	1.847201	2.975105	2.975105
5nM-NA	-0.82075	-0.82075	-0.47194	0.388078	0.729987	0.742497	0.742497
-NA	-1.24526	-1.24526	-1.1803	-0.57803	-0.02581	0.058657	0.058657
50nM+NA	-0.65884	-0.65884	-0.61631	-0.28737	-0.09954	-0.01052	-0.01052
5nM+NA	-0.74201	-0.74201	-0.70055	-0.22707	-0.08552	-0.0101	-0.0101
+NA	-0.84617	-0.84617	-0.51061	-0.12486	0.312621	1.139833	1.139833

Oneway Anova

Summary of Fit

Rsquare	0.491431
Adj Rsquare	0.40667
Root Mean Square Error	0.624454
Mean of Response	-2.8e-10
Observations (or Sum Wgts)	36

Analysis of Variance

Source	DF	Sum of Squares	Mean Square	F Ratio	Prob > F
Label	5	11.304079	2.26082	5.7978	0.0007 *
Error	30	11.698288	0.38994		
C. Total	35	23.002367			

Means for Oneway Anova

Level	Number	Mean	Std Error	Lower 95%	Upper 95%
50nM-NA	6	1.1307	0.25493	0.610	1.651
5nM-NA	6	0.1780	0.25493	-0.343	0.699
-NA	6	-0.5925	0.25493	-1.113	-0.072
50nM+NA	6	-0.3292	0.25493	-0.850	0.191
5nM+NA	6	-0.3339	0.25493	-0.855	0.187
+NA	6	-0.0530	0.25493	-0.574	0.468

Std Error uses a pooled estimate of error variance

Means Comparisons

Comparisons with a control using Dunnett's Method

Oneway Analysis of dAMP By Label

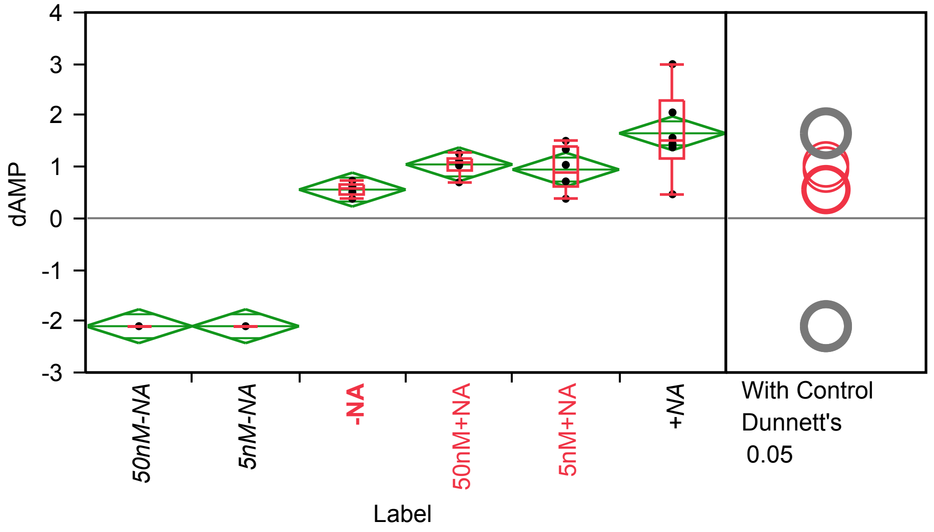

Quantiles

Level	Minimum	10%	25%	Median	75%	90%	Maximum
50nM-NA	-2.09967	-2.09967	-2.09967	-2.09966	-2.09966	-2.09966	-2.09966
5nM-NA	-2.09967	-2.09967	-2.09967	-2.09967	-2.09966	-2.09966	-2.09966
-NA	0.377039	0.377039	0.46159	0.561009	0.647794	0.728754	0.728754
50nM+NA	0.698046	0.698046	0.942955	1.078497	1.166149	1.259855	1.259855
5nM+NA	0.378552	0.378552	0.62093	0.876011	1.380752	1.504136	1.504136
+NA	0.465208	0.465208	1.149622	1.505957	2.291368	2.995651	2.995651

Oneway Anova

Summary of Fit

Rsquare	0.946806
Adj Rsquare	0.937941
Root Mean Square Error	0.394375
Mean of Response	-5.56e-9
Observations (or Sum Wgts)	36

Analysis of Variance

Source	DF	Sum of Squares	Mean Square	F Ratio	Prob > F
Label	5	83.050155	16.6100	106.7951	<.0001 *
Error	30	4.665953	0.1555		
C. Total	35	87.716108			

Means for Oneway Anova

Level	Number	Mean	Std Error	Lower 95%	Upper 95%
50nM-NA	6	-2.0997	0.16100	-2.428	-1.771
5nM-NA	6	-2.0997	0.16100	-2.428	-1.771
-NA	6	0.5564	0.16100	0.228	0.885
50nM+NA	6	1.0457	0.16100	0.717	1.375
5nM+NA	6	0.9460	0.16100	0.617	1.275
+NA	6	1.6512	0.16100	1.322	1.980

Std Error uses a pooled estimate of error variance

Means Comparisons

Comparisons with a control using Dunnett's Method

Oneway Analysis of dCMP By Label

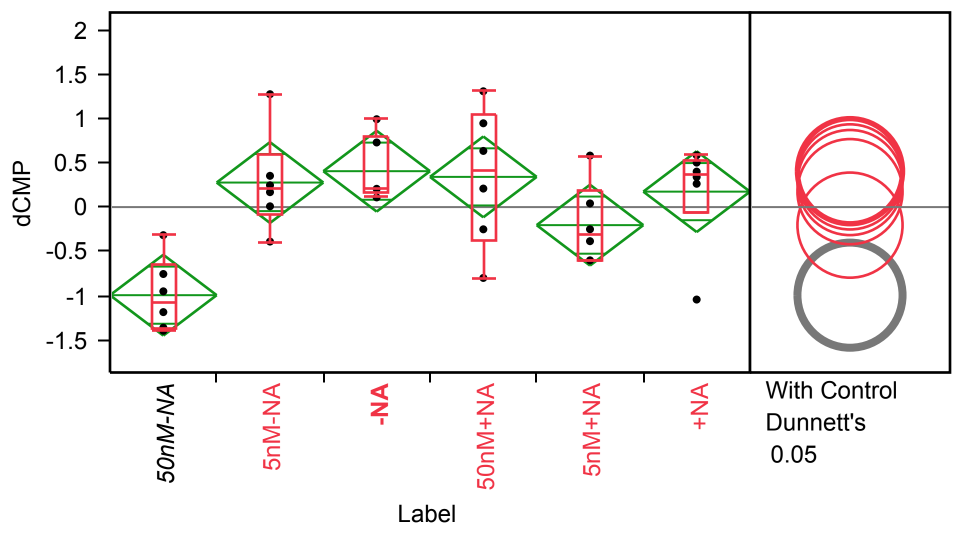

Quantiles

Level	Minimum	10%	25%	Median	75%	90%	Maximum
50nM-NA	-1.39786	-1.39786	-1.37206	-1.06982	-0.6468	-0.3177	-0.3177
5nM-NA	-0.38985	-0.38985	-0.0908	0.208031	0.584169	1.276256	1.276256
-NA	0.109779	0.109779	0.174996	0.2029	0.796289	0.993401	0.993401
50nM+NA	-0.80092	-0.80092	-0.38865	0.422194	1.037977	1.309208	1.309208
5nM+NA	-0.60935	-0.60935	-0.6039	-0.31791	0.176712	0.582179	0.582179
+NA	-1.0453	-1.0453	-0.06342	0.370493	0.523818	0.585837	0.585837

Oneway Anova

Summary of Fit

Rsquare	0.486653
Adj Rsquare	0.401095
Root Mean Square Error	0.547791
Mean of Response	0
Observations (or Sum Wgts)	36

Analysis of Variance

Source	DF	Sum of Squares	Mean Square	F Ratio	Prob > F
Label	5	8.534134	1.70683	5.6880	0.0008 *
Error	30	9.002246	0.30007		
C. Total	35	17.536380			

Means for Oneway Anova

Level	Number	Mean	Std Error	Lower 95%	Upper 95%
50nM-NA	6	-0.99586	0.22363	-1.453	-0.5391
5nM-NA	6	0.27747	0.22363	-0.179	0.7342
-NA	6	0.40605	0.22363	-0.051	0.8628
50nM+NA	6	0.34150	0.22363	-0.115	0.7982
5nM+NA	6	-0.20392	0.22363	-0.661	0.2528
+NA	6	0.17476	0.22363	-0.282	0.6315

Std Error uses a pooled estimate of error variance

Means Comparisons

Comparisons with a control using Dunnett's Method

Oneway Analysis of deoxyadenosine By Label

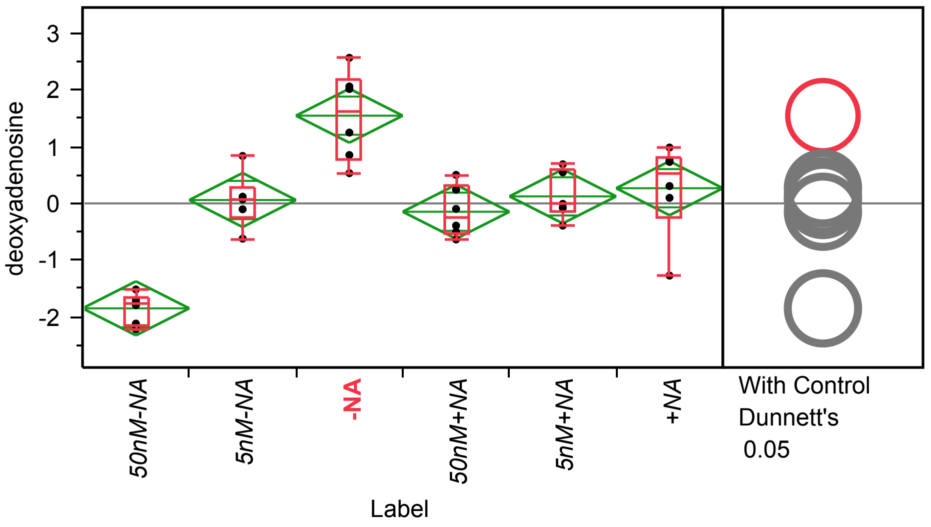

Quantiles

Level	Minimum	10%	25%	Median	75%	90%	Maximum
50nM-NA	-2.2233	-2.2233	-2.14734	-1.77148	-1.6614	-1.51974	-1.51974
5nM-NA	-0.62238	-0.62238	-0.23349	0.064526	0.298013	0.836866	0.836866
-NA	0.533422	0.533422	0.773582	1.635041	2.190738	2.569893	2.569893
50nM+NA	-0.63657	-0.63657	-0.5352	-0.24534	0.306386	0.503005	0.503005
5nM+NA	-0.39252	-0.39252	-0.15599	-0.01235	0.583736	0.690364	0.690364
+NA	-1.27495	-1.27495	-0.24526	0.519073	0.817145	0.985658	0.985658

Oneway Anova

Summary of Fit

Rsquare	0.784079
Adj Rsquare	0.748092
Root Mean Square Error	0.572133
Mean of Response	2.78e-11
Observations (or Sum Wgts)	36

Analysis of Variance

Source	DF	Sum of Squares	Mean Square	F Ratio	Prob > F
Label	5	35.659954	7.13199	21.7880	<.0001 *
Error	30	9.820091	0.32734		
C. Total	35	45.480046			

Means for Oneway Anova

Level	Number	Mean	Std Error	Lower 95%	Upper 95%
50nM-NA	6	-1.8528	0.23357	-2.330	-1.376
5nM-NA	6	0.0597	0.23357	-0.417	0.537
-NA	6	1.5486	0.23357	1.072	2.026
50nM+NA	6	-0.1475	0.23357	-0.624	0.330
5nM+NA	6	0.1240	0.23357	-0.353	0.601
+NA	6	0.2680	0.23357	-0.209	0.745

Std Error uses a pooled estimate of error variance

Means Comparisons

Comparisons with a control using Dunnett's Method

Oneway Analysis of Deoxyheptulose-7-phosphate By Label

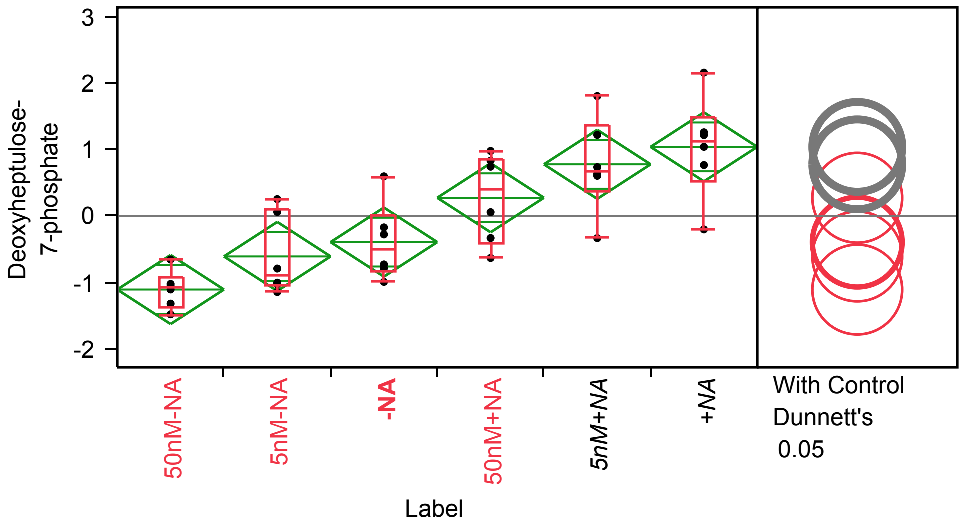

Quantiles

Level	Minimum	10%	25%	Median	75%	90%	Maximum
50nM-NA	-1.47719	-1.47719	-1.35665	-1.07897	-0.9282	-0.65311	-0.65311
5nM-NA	-1.14152	-1.14152	-1.05433	-0.89461	0.11167	0.25663	0.25663
-NA	-0.98897	-0.98897	-0.83368	-0.50049	0.023151	0.59947	0.59947
50nM+NA	-0.62932	-0.62932	-0.40413	0.402282	0.871292	0.981648	0.981648
5nM+NA	-0.33004	-0.33004	0.373183	0.687207	1.369107	1.807507	1.807507
+NA	-0.19892	-0.19892	0.527441	1.131897	1.486899	2.160379	2.160379

Oneway Anova

Summary of Fit

Rsquare	0.643951
Adj Rsquare	0.58461
Root Mean Square Error	0.623184
Mean of Response	2.78e-10
Observations (or Sum Wgts)	36

Analysis of Variance

Source	DF	Sum of Squares	Mean Square	F Ratio	Prob > F
Label	5	21.071595	4.21432	10.8516	<.0001 *
Error	30	11.650744	0.38836		
C. Total	35	32.722339			

Means for Oneway Anova

Level	Number	Mean	Std Error	Lower 95%	Upper 95%
50nM-NA	6	-1.1041	0.25441	-1.624	-0.585
5nM-NA	6	-0.6060	0.25441	-1.126	-0.086
-NA	6	-0.3902	0.25441	-0.910	0.129
50nM+NA	6	0.2771	0.25441	-0.243	0.797
5nM+NA	6	0.7804	0.25441	0.261	1.300
+NA	6	1.0429	0.25441	0.523	1.562

Std Error uses a pooled estimate of error variance

Means Comparisons

Comparisons with a control using Dunnett's Method

Oneway Analysis of deoxyribose-phosphate By Label

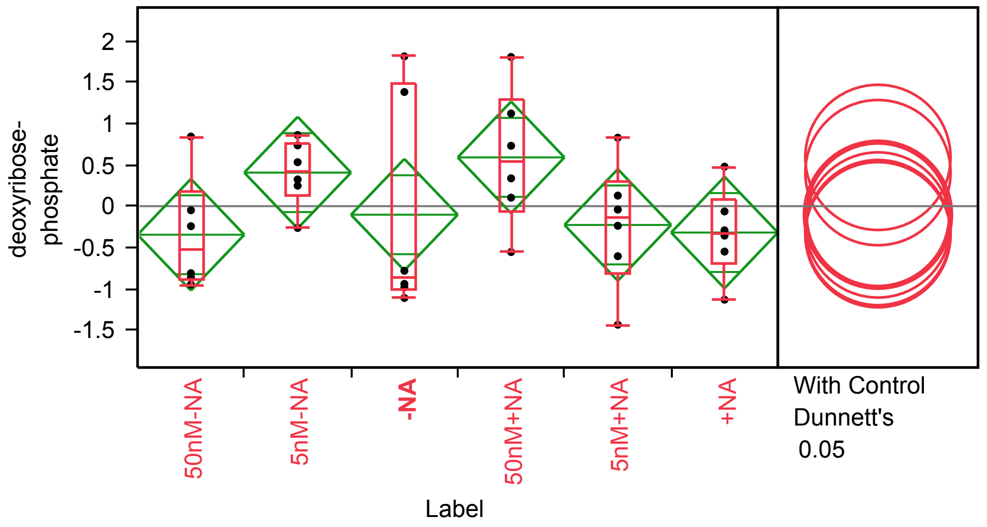

Quantiles

Level	Minimum	10%	25%	Median	75%	90%	Maximum
50nM-NA	-0.95023	-0.95023	-0.88626	-0.52795	0.173462	0.843538	0.843538
5nM-NA	-0.26443	-0.26443	0.119989	0.428234	0.768139	0.862741	0.862741
-NA	-1.11299	-1.11299	-1.01609	-0.86301	1.491686	1.815733	1.815733
50nM+NA	-0.55628	-0.55628	-0.06198	0.53453	1.294672	1.808679	1.808679
5nM+NA	-1.43746	-1.43746	-0.81414	-0.13993	0.303489	0.829524	0.829524
+NA	-1.13366	-1.13366	-0.69669	-0.32501	0.071748	0.478221	0.478221

Oneway Anova

Summary of Fit

Rsquare	0.195506
Adj Rsquare	0.061423
Root Mean Square Error	0.811332
Mean of Response	2.78e-10
Observations (or Sum Wgts)	36

Analysis of Variance

Source	DF	Sum of Squares	Mean Square	F Ratio	Prob > F
Label	5	4.799048	0.959810	1.4581	0.2328
Error	30	19.747807	0.658260		
C. Total	35	24.546854			

Means for Oneway Anova

Level	Number	Mean	Std Error	Lower 95%	Upper 95%
50nM-NA	6	-0.34624	0.33123	-1.023	0.3302
5nM-NA	6	0.40659	0.33123	-0.270	1.0830
-NA	6	-0.10390	0.33123	-0.780	0.5726
50nM+NA	6	0.59126	0.33123	-0.085	1.2677
5nM+NA	6	-0.22767	0.33123	-0.904	0.4488
+NA	6	-0.32004	0.33123	-0.996	0.3564

Std Error uses a pooled estimate of error variance

Means Comparisons

Comparisons with a control using Dunnett's Method

Oneway Analysis of deoxyuridine By Label

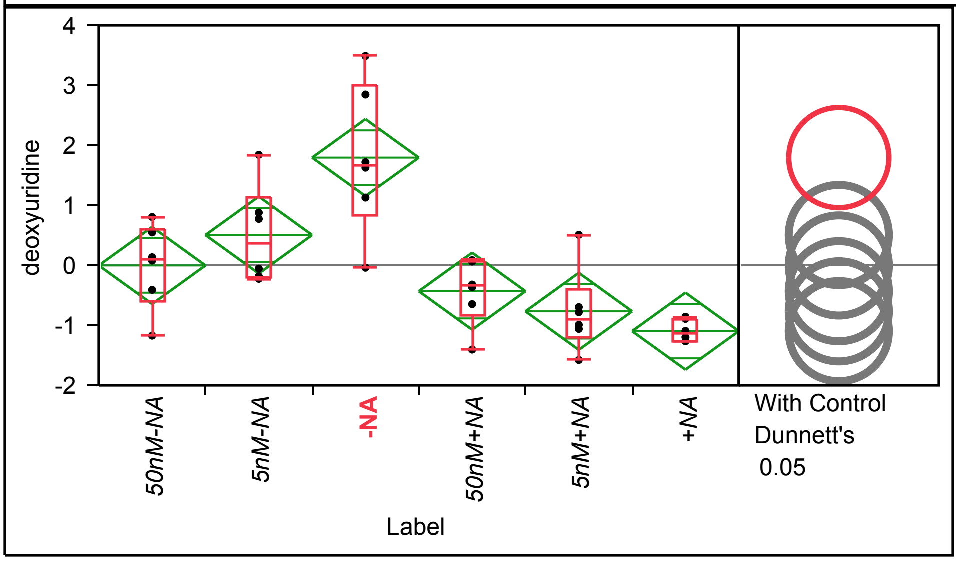

Quantiles

Level	Minimum	10%	25%	Median	75%	90%	Maximum
50nM-NA	-1.17237	-1.17237	-0.60014	0.106233	0.611868	0.804521	0.804521
5nM-NA	-0.22543	-0.22543	-0.1929	0.358363	1.11848	1.840688	1.840688
-NA	-0.04041	-0.04041	0.836287	1.673092	3.006336	3.487991	3.487991
50nM+NA	-1.40479	-1.40479	-0.83674	-0.34353	0.068433	0.085826	0.085826
5nM+NA	-1.57795	-1.57795	-1.18989	-0.88715	-0.39625	0.506966	0.506966
+NA	-1.27615	-1.27615	-1.26834	-1.14564	-0.88429	-0.85869	-0.85869

Oneway Anova

Summary of Fit

Rsquare	0.648241
Adj Rsquare	0.589614
Root Mean Square Error	0.769421
Mean of Response	-2.5e-17
Observations (or Sum Wgts)	36

Analysis of Variance

Source	DF	Sum of Squares	Mean Square	F Ratio	Prob > F
Label	5	32.729600	6.54592	11.0571	<.0001 *
Error	30	17.760267	0.59201		
C. Total	35	50.489867			

Means for Oneway Anova

Level	Number	Mean	Std Error	Lower 95%	Upper 95%
50nM-NA	6	-0.0029	0.31411	-0.644	0.639
5nM-NA	6	0.5046	0.31411	-0.137	1.146
-NA	6	1.7947	0.31411	1.153	2.436
50nM+NA	6	-0.4318	0.31411	-1.073	0.210
5nM+NA	6	-0.7672	0.31411	-1.409	-0.126
+NA	6	-1.0974	0.31411	-1.739	-0.456

Std Error uses a pooled estimate of error variance

Means Comparisons

Comparisons with a control using Dunnett's Method

Oneway Analysis of dGDP By Label

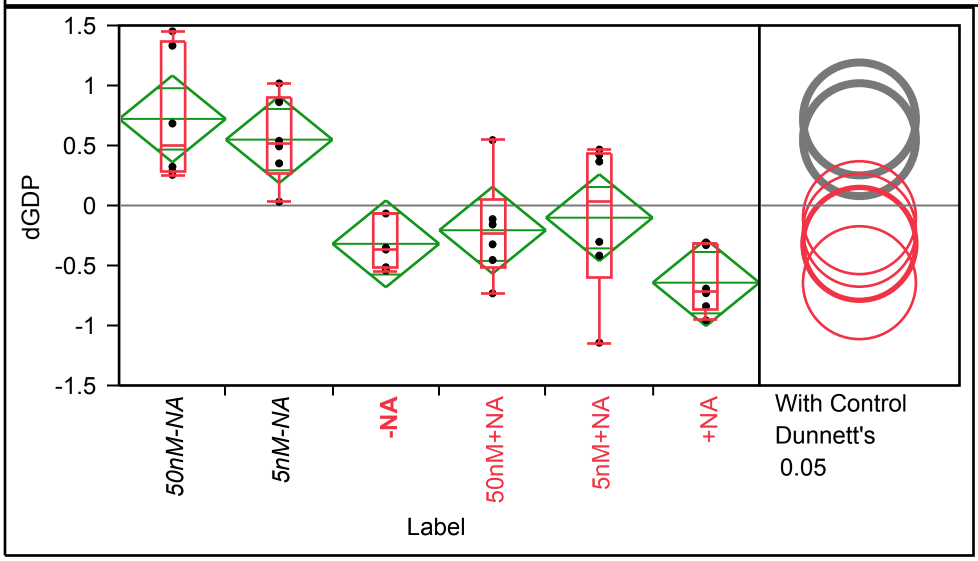

Quantiles

Level	Minimum	10%	25%	Median	75%	90%	Maximum
50nM-NA	0.254939	0.254939	0.277801	0.503026	1.361674	1.451872	1.451872
5nM-NA	0.031607	0.031607	0.271317	0.515715	0.900306	1.017365	1.017365
-NA	-0.54225	-0.54225	-0.52151	-0.35985	-0.07191	-0.0669	-0.0669
50nM+NA	-0.73209	-0.73209	-0.52367	-0.24089	0.051041	0.544771	0.544771
5nM+NA	-1.14339	-1.14339	-0.59911	0.031749	0.433052	0.465778	0.465778
+NA	-0.95741	-0.95741	-0.86885	-0.71183	-0.32492	-0.30852	-0.30852

Oneway Anova

Summary of Fit

Rsquare	0.596635
Adj Rsquare	0.529407
Root Mean Square Error	0.433623
Mean of Response	5.56e-10
Observations (or Sum Wgts)	36

Analysis of Variance

Source	DF	Sum of Squares	Mean Square	F Ratio	Prob > F
Label	5	8.343634	1.66873	8.8749	<.0001 *
Error	30	5.640855	0.18803		
C. Total	35	13.984489			

Means for Oneway Anova

Level	Number	Mean	Std Error	Lower 95%	Upper 95%
50nM-NA	6	0.72165	0.17703	0.360	1.083
5nM-NA	6	0.54882	0.17703	0.187	0.910
-NA	6	-0.31950	0.17703	-0.681	0.042
50nM+NA	6	-0.20614	0.17703	-0.568	0.155
5nM+NA	6	-0.10161	0.17703	-0.463	0.260
+NA	6	-0.64322	0.17703	-1.005	-0.282

Std Error uses a pooled estimate of error variance

Means Comparisons

Comparisons with a control using Dunnett's Method

Oneway Analysis of D-gluconate By Label

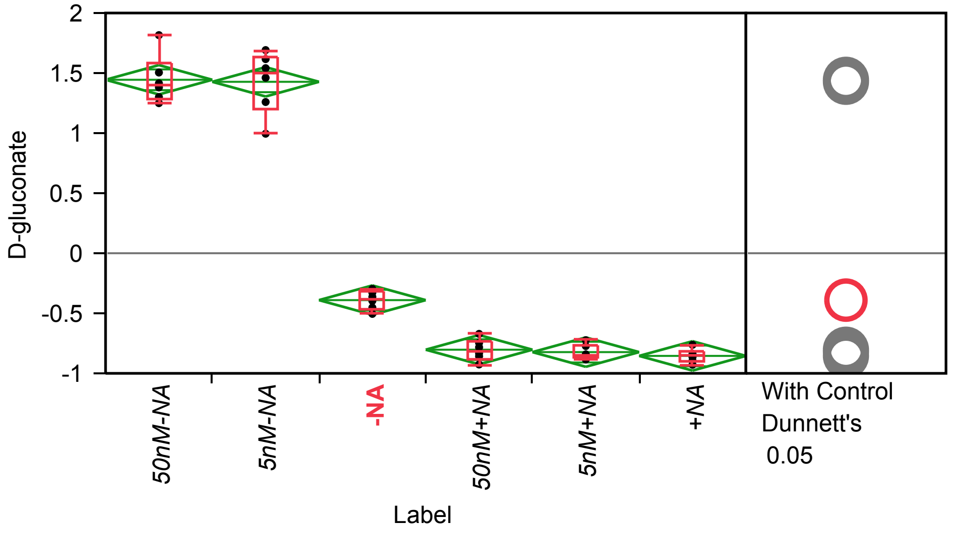

Quantiles

Level	Minimum	10%	25%	Median	75%	90%	Maximum
50nM-NA	1.249535	1.249535	1.287454	1.397604	1.582301	1.815725	1.815725
5nM-NA	0.995236	0.995236	1.193076	1.500845	1.635035	1.691199	1.691199
-NA	-0.5048	-0.5048	-0.46558	-0.37978	-0.32025	-0.29756	-0.29756
50nM+NA	-0.92619	-0.92619	-0.87511	-0.80841	-0.72742	-0.67149	-0.67149
5nM+NA	-0.88436	-0.88436	-0.86881	-0.847	-0.76053	-0.72429	-0.72429
+NA	-0.92818	-0.92818	-0.90345	-0.85261	-0.81883	-0.76257	-0.76257

Oneway Anova

Summary of Fit

Rsquare	0.983297
Adj Rsquare	0.980514
Root Mean Square Error	0.146625
Mean of Response	0
Observations (or Sum Wgts)	36

Analysis of Variance

Source	DF	Sum of Squares	Mean Square	F Ratio	Prob > F
Label	5	37.969691	7.59394	353.2245	<.0001 *
Error	30	0.644967	0.02150		
C. Total	35	38.614658			

Means for Oneway Anova

Level	Number	Mean	Std Error	Lower 95%	Upper 95%
50nM-NA	6	1.4442	0.05986	1.322	1.566
5nM-NA	6	1.4272	0.05986	1.305	1.549
-NA	6	-0.3904	0.05986	-0.513	-0.268
50nM+NA	6	-0.8031	0.05986	-0.925	-0.681
5nM+NA	6	-0.8231	0.05986	-0.945	-0.701
+NA	6	-0.8548	0.05986	-0.977	-0.733

Std Error uses a pooled estimate of error variance

Means Comparisons

Comparisons with a control using Dunnett's Method

Oneway Analysis of dihydroxy-acetone-phosphate By Label

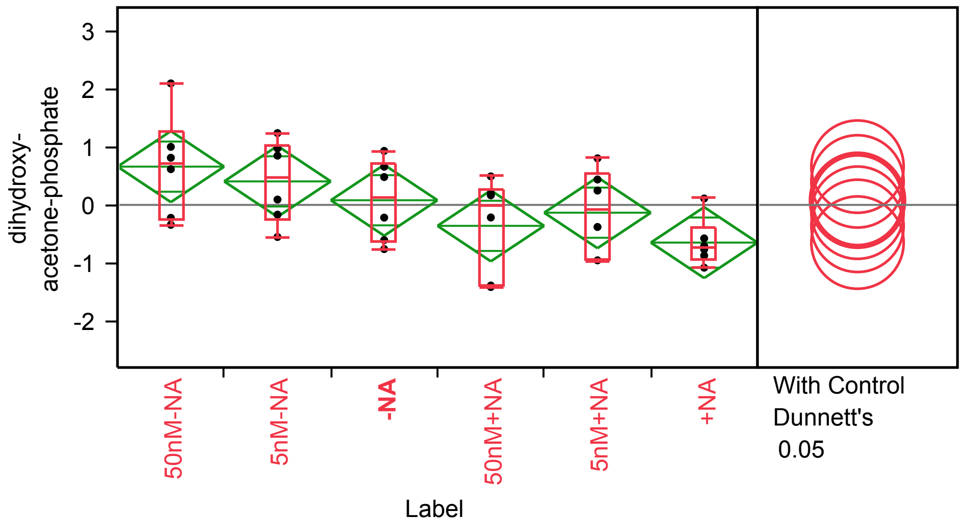

Quantiles

Level	Minimum	10%	25%	Median	75%	90%	Maximum
50nM-NA	-0.34399	-0.34399	-0.25379	0.716072	1.277244	2.099365	2.099365
5nM-NA	-0.55415	-0.55415	-0.26482	0.47189	1.042232	1.240734	1.240734
-NA	-0.76312	-0.76312	-0.64638	0.130587	0.728258	0.933143	0.933143
50nM+NA	-1.41867	-1.41867	-1.39976	-0.02586	0.272552	0.492885	0.492885
5nM+NA	-0.96115	-0.96115	-0.95846	-0.06687	0.530357	0.804719	0.804719
+NA	-1.08463	-1.08463	-0.92878	-0.73971	-0.40591	0.111053	0.111053

Oneway Anova

Summary of Fit

Rsquare	0.304258
Adj Rsquare	0.188302
Root Mean Square Error	0.735297
Mean of Response	2.78e-10
Observations (or Sum Wgts)	36

Analysis of Variance

Source	DF	Sum of Squares	Mean Square	F Ratio	Prob > F
Label	5	7.093190	1.41864	2.6239	0.0440 *
Error	30	16.219848	0.54066		
C. Total	35	23.313038			

Means for Oneway Anova

Level	Number	Mean	Std Error	Lower 95%	Upper 95%
50nM-NA	6	0.66117	0.30018	0.048	1.274
5nM-NA	6	0.40634	0.30018	-0.207	1.019
-NA	6	0.08062	0.30018	-0.532	0.694
50nM+NA	6	-0.36198	0.30018	-0.975	0.251
5nM+NA	6	-0.13481	0.30018	-0.748	0.478
+NA	6	-0.65134	0.30018	-1.264	-0.038

Std Error uses a pooled estimate of error variance

Means Comparisons

Comparisons with a control using Dunnett's Method

Oneway Analysis of dimethylglycine By Label

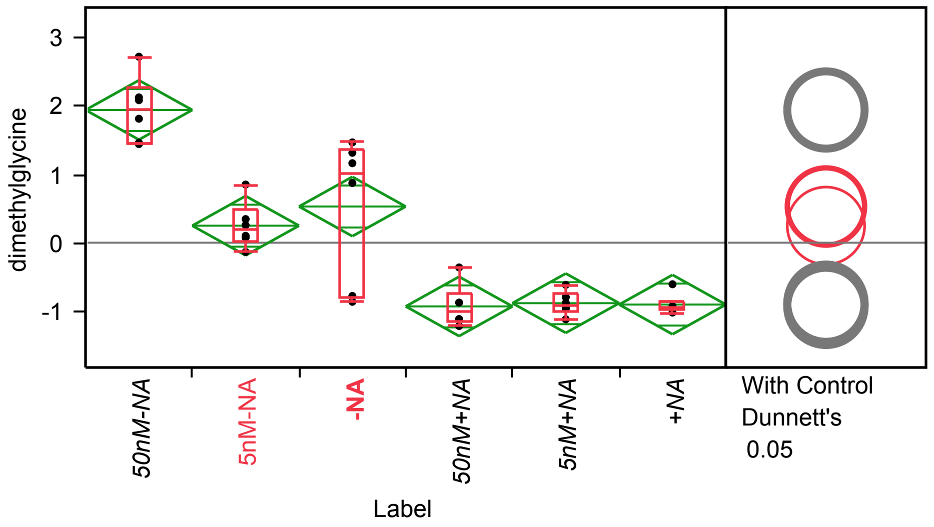

Quantiles

Level	Minimum	10%	25%	Median	75%	90%	Maximum
50nM-NA	1.446557	1.446557	1.460552	1.954373	2.28077	2.72813	2.72813
5nM-NA	-0.13513	-0.13513	0.022158	0.179646	0.473231	0.852759	0.852759
-NA	-0.86627	-0.86627	-0.80216	1.021203	1.357221	1.471435	1.471435
50nM+NA	-1.22184	-1.22184	-1.15542	-1.00393	-0.74873	-0.36265	-0.36265
5nM+NA	-1.12306	-1.12306	-1.00531	-0.90756	-0.75284	-0.61801	-0.61801
+NA	-1.02426	-1.02426	-0.99029	-0.95029	-0.84866	-0.609	-0.609

Oneway Anova

Summary of Fit

Rsquare	0.829037
Adj Rsquare	0.800543
Root Mean Square Error	0.522318
Mean of Response	-5.6e-10
Observations (or Sum Wgts)	36

Analysis of Variance

Source	DF	Sum of Squares	Mean Square	F Ratio	Prob > F
Label	5	39.688270	7.93765	29.0952	<.0001 *
Error	30	8.184485	0.27282		
C. Total	35	47.872755			

Means for Oneway Anova

Level	Number	Mean	Std Error	Lower 95%	Upper 95%
50nM-NA	6	1.9467	0.21324	1.511	2.382
5nM-NA	6	0.2497	0.21324	-0.186	0.685
-NA	6	0.5310	0.21324	0.096	0.966
50nM+NA	6	-0.9338	0.21324	-1.369	-0.498
5nM+NA	6	-0.8867	0.21324	-1.322	-0.451
+NA	6	-0.9069	0.21324	-1.342	-0.471

Std Error uses a pooled estimate of error variance

Means Comparisons

Comparisons with a control using Dunnett's Method

Oneway Analysis of DL-Pipecolic acid By Label

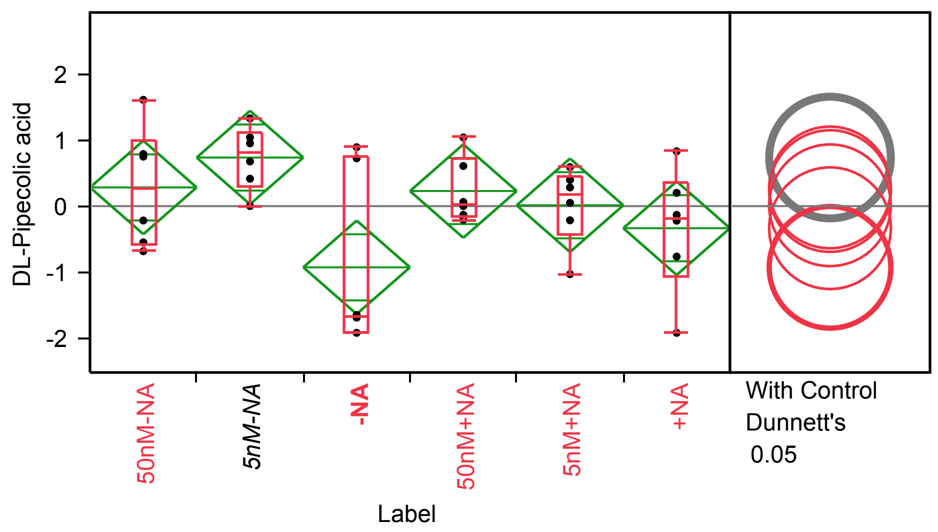

Quantiles

Level	Minimum	10%	25%	Median	75%	90%	Maximum
50nM-NA	-0.683	-0.683	-0.5825	0.266	0.99425	1.61	1.61
5nM-NA	0.00198	0.00198	0.311745	0.814	1.1125	1.33	1.33
-NA	-1.92	-1.92	-1.92	-1.67	0.766	0.892	0.892
50nM+NA	-0.208	-0.208	-0.151	0.031337	0.716	1.04	1.04
5nM+NA	-1.03	-1.03	-0.41875	0.16625	0.446	0.59	0.59
+NA	-1.92	-1.92	-1.05375	-0.177	0.35825	0.83	0.83

Oneway Anova

Summary of Fit

Rsquare	0.313223
Adj Rsquare	0.198761
Root Mean Square Error	0.849572
Mean of Response	-0.00033
Observations (or Sum Wgts)	36

Analysis of Variance

Source	DF	Sum of Squares	Mean Square	F Ratio	Prob > F
Label	5	9.875539	1.97511	2.7365	0.0375 *
Error	30	21.653198	0.72177		
C. Total	35	31.528738			

Means for Oneway Anova

Level	Number	Mean	Std Error	Lower 95%	Upper 95%
50nM-NA	6	0.28317	0.34684	-0.425	0.992
5nM-NA	6	0.73583	0.34684	0.027	1.444
-NA	6	-0.92733	0.34684	-1.636	-0.219
50nM+NA	6	0.22845	0.34684	-0.480	0.937
5nM+NA	6	0.01258	0.34684	-0.696	0.721
+NA	6	-0.33467	0.34684	-1.043	0.374

Std Error uses a pooled estimate of error variance

Means Comparisons

Comparisons with a control using Dunnett's Method

Oneway Analysis of D-sedoheptulose-1-7-bisphosphate By Label

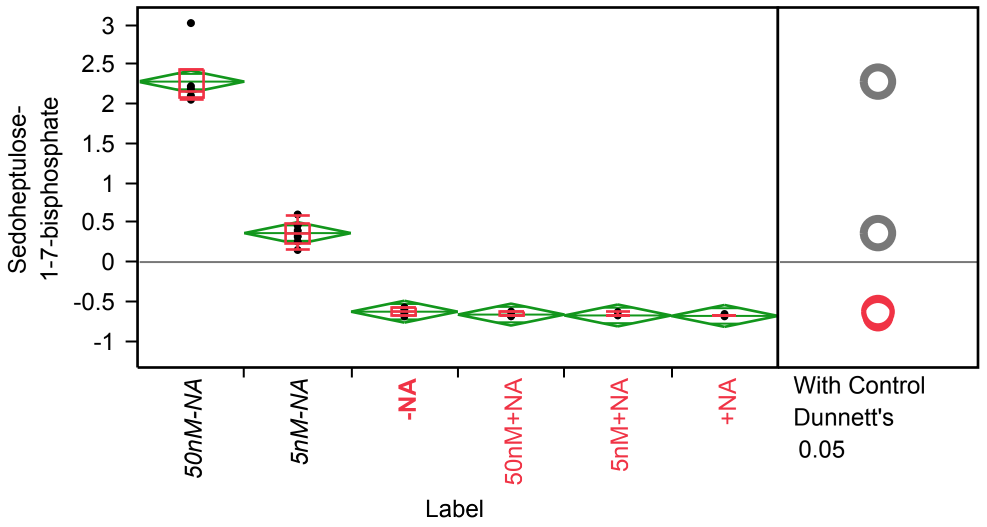

Quantiles

Level	Minimum	10%	25%	Median	75%	90%	Maximum
50nM-NA	2.051149	2.051149	2.086099	2.144911	2.421234	3.019535	3.019535
5nM-NA	0.15272	0.15272	0.232365	0.359733	0.497558	0.600717	0.600717
-NA	-0.68524	-0.68524	-0.68524	-0.62168	-0.57492	-0.5729	-0.5729
50nM+NA	-0.68527	-0.68527	-0.68527	-0.67879	-0.62723	-0.62607	-0.62607
5nM+NA	-0.68527	-0.68527	-0.68526	-0.68525	-0.66384	-0.63256	-0.63256
+NA	-0.68531	-0.68531	-0.68527	-0.68526	-0.67812	-0.65673	-0.65673

Oneway Anova

Summary of Fit

Rsquare	0.981038
Adj Rsquare	0.977878
Root Mean Square Error	0.165483
Mean of Response	8.333e-9
Observations (or Sum Wgts)	36

Analysis of Variance

Source	DF	Sum of Squares	Mean Square	F Ratio	Prob > F
Label	5	42.504700	8.50094	310.4282	<.0001 *
Error	30	0.821537	0.02738		
C. Total	35	43.326237			

Means for Oneway Anova

Level	Number	Mean	Std Error	Lower 95%	Upper 95%
50nM-NA	6	2.2800	0.06756	2.142	2.418
5nM-NA	6	0.3658	0.06756	0.228	0.504
-NA	6	-0.6271	0.06756	-0.765	-0.489
50nM+NA	6	-0.6636	0.06756	-0.802	-0.526
5nM+NA	6	-0.6746	0.06756	-0.813	-0.537
+NA	6	-0.6805	0.06756	-0.818	-0.543

Std Error uses a pooled estimate of error variance

Means Comparisons

Comparisons with a control using Dunnett's Method

Oneway Analysis of FAD By Label

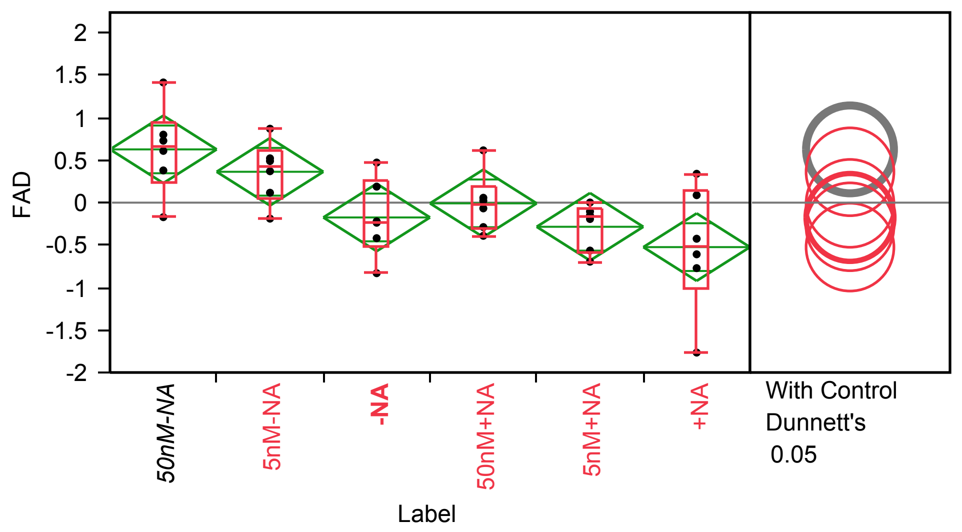

Quantiles

Level	Minimum	10%	25%	Median	75%	90%	Maximum
50nM-NA	-0.1708	-0.1708	0.241794	0.669376	0.953965	1.414896	1.414896
5nM-NA	-0.18804	-0.18804	0.040213	0.430125	0.608889	0.870131	0.870131
-NA	-0.83057	-0.83057	-0.52141	-0.22853	0.259318	0.471937	0.471937
50nM+NA	-0.39048	-0.39048	-0.31355	-0.02219	0.196912	0.615628	0.615628
5nM+NA	-0.69509	-0.69509	-0.59721	-0.17145	-0.0779	0.001435	0.001435
+NA	-1.76525	-1.76525	-1.01971	-0.51663	0.15088	0.339716	0.339716

Oneway Anova

Summary of Fit

Rsquare	0.444938
Adj Rsquare	0.352427
Root Mean Square Error	0.476695
Mean of Response	2.78e-11
Observations (or Sum Wgts)	36

Analysis of Variance

Source	DF	Sum of Squares	Mean Square	F Ratio	Prob > F
Label	5	5.464613	1.09292	4.8096	0.0024 *
Error	30	6.817139	0.22724		
C. Total	35	12.281752			

Means for Oneway Anova

Level	Number	Mean	Std Error	Lower 95%	Upper 95%
50nM-NA	6	0.62708	0.19461	0.2296	1.025
5nM-NA	6	0.36341	0.19461	-0.0340	0.761
-NA	6	-0.17427	0.19461	-0.5717	0.223
50nM+NA	6	-0.00830	0.19461	-0.4057	0.389
5nM+NA	6	-0.28425	0.19461	-0.6817	0.113
+NA	6	-0.52368	0.19461	-0.9211	-0.126

Std Error uses a pooled estimate of error variance

Means Comparisons

Comparisons with a control using Dunnett's Method

Oneway Analysis of folate By Label

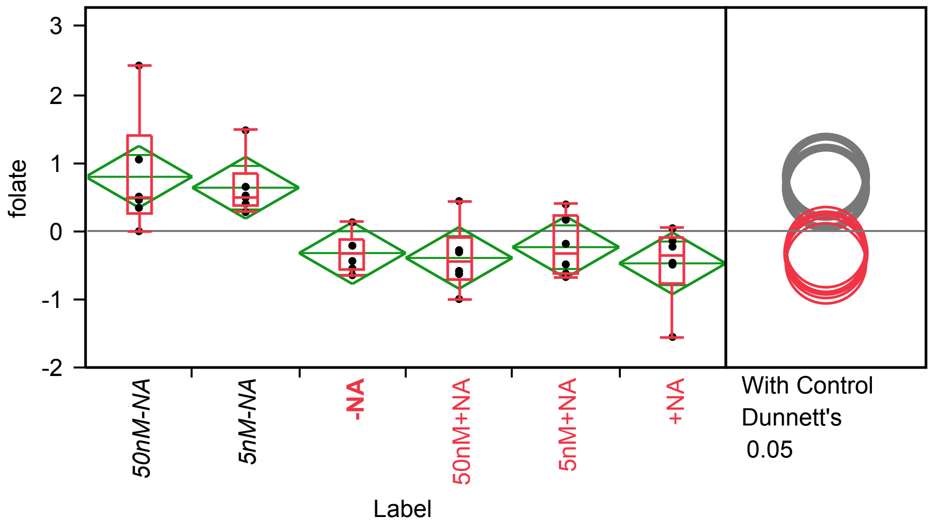

Quantiles

Level	Minimum	10%	25%	Median	75%	90%	Maximum
50nM-NA	-0.00328	-0.00328	0.253586	0.479816	1.391262	2.423598	2.423598
5nM-NA	0.277969	0.277969	0.368118	0.50411	0.854545	1.474808	1.474808
-NA	-0.64752	-0.64752	-0.57345	-0.32844	-0.12995	0.127745	0.127745
50nM+NA	-0.99686	-0.99686	-0.72248	-0.44876	-0.10099	0.438236	0.438236
5nM+NA	-0.67403	-0.67403	-0.63609	-0.33806	0.220616	0.388956	0.388956
+NA	-1.55178	-1.55178	-0.75736	-0.34733	-0.09881	0.04011	0.04011

Oneway Anova

Summary of Fit

Rsquare	0.517703
Adj Rsquare	0.43732
Root Mean Square Error	0.541674
Mean of Response	5.56e-11
Observations (or Sum Wgts)	36

Analysis of Variance

Source	DF	Sum of Squares	Mean Square	F Ratio	Prob > F
Label	5	9.448501	1.88970	6.4405	0.0004 *
Error	30	8.802322	0.29341		
C. Total	35	18.250823			

Means for Oneway Anova

Level	Number	Mean	Std Error	Lower 95%	Upper 95%
50nM-NA	6	0.79438	0.22114	0.3428	1.246
5nM-NA	6	0.63449	0.22114	0.1829	1.086
-NA	6	-0.32354	0.22114	-0.7752	0.128
50nM+NA	6	-0.39465	0.22114	-0.8463	0.057
5nM+NA	6	-0.23669	0.22114	-0.6883	0.215
+NA	6	-0.47400	0.22114	-0.9256	-0.022

Std Error uses a pooled estimate of error variance

Means Comparisons

Comparisons with a control using Dunnett's Method

Oneway Analysis of fructose-1,6-bisphosphate By Label

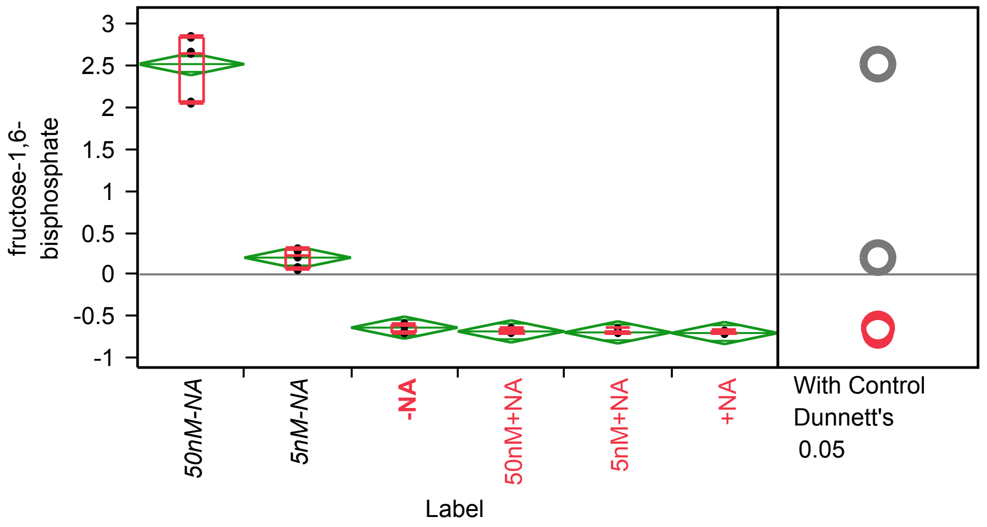

Quantiles

Level	Minimum	10%	25%	Median	75%	90%	Maximum
50nM-NA	2.046449	2.046449	2.065396	2.65715	2.842253	2.857596	2.857596
5nM-NA	0.050613	0.050613	0.078231	0.238146	0.286251	0.312443	0.312443
-NA	-0.70916	-0.70916	-0.68786	-0.62386	-0.60185	-0.58749	-0.58749
50nM+NA	-0.71561	-0.71561	-0.70707	-0.68324	-0.6668	-0.64291	-0.64291
5nM+NA	-0.7156	-0.7156	-0.71526	-0.70781	-0.67367	-0.6452	-0.6452
+NA	-0.7156	-0.7156	-0.7156	-0.7156	-0.68251	-0.6729	-0.6729

Oneway Anova

Summary of Fit

Rsquare	0.984948
Adj Rsquare	0.98244
Root Mean Square Error	0.158807
Mean of Response	-5.6e-10
Observations (or Sum Wgts)	36

Analysis of Variance

Source	DF	Sum of Squares	Mean Square	F Ratio	Prob > F
Label	5	49.508818	9.90176	392.6227	<.0001 *
Error	30	0.756586	0.02522		
C. Total	35	50.265404			

Means for Oneway Anova

Level	Number	Mean	Std Error	Lower 95%	Upper 95%
50nM-NA	6	2.5212	0.06483	2.389	2.654
5nM-NA	6	0.2007	0.06483	0.068	0.333
-NA	6	-0.6386	0.06483	-0.771	-0.506
50nM+NA	6	-0.6840	0.06483	-0.816	-0.552
5nM+NA	6	-0.6958	0.06483	-0.828	-0.563
+NA	6	-0.7035	0.06483	-0.836	-0.571

Std Error uses a pooled estimate of error variance

Means Comparisons

Comparisons with a control using Dunnett's Method

Oneway Analysis of fructose-6-phosphate By Label

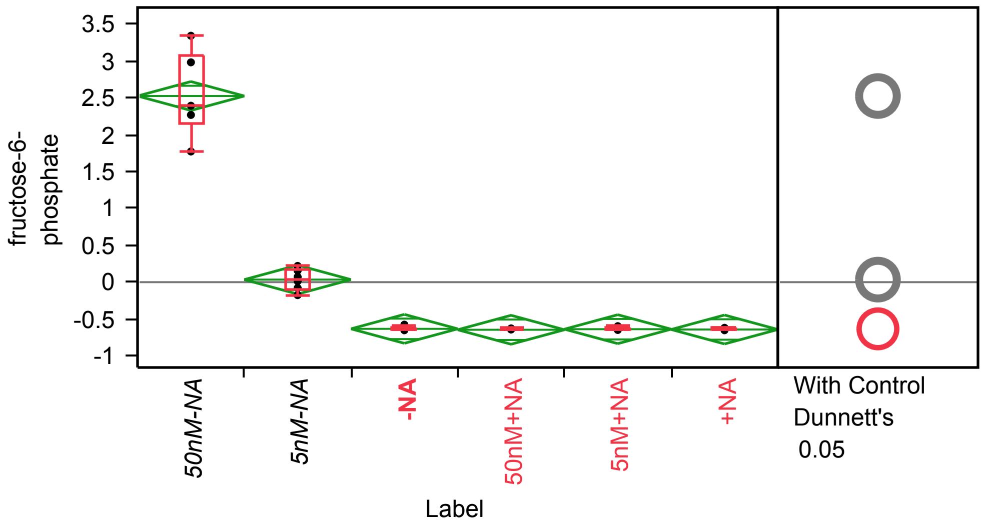

Quantiles

Level	Minimum	10%	25%	Median	75%	90%	Maximum
50nM-NA	1.772113	1.772113	2.144052	2.392025	3.071408	3.341539	3.341539
5nM-NA	-0.17582	-0.17582	-0.10314	0.042163	0.172451	0.219984	0.219984
-NA	-0.65233	-0.65233	-0.65233	-0.65232	-0.60439	-0.57645	-0.57645
50nM+NA	-0.65235	-0.65235	-0.65235	-0.64979	-0.63879	-0.62719	-0.62719
5nM+NA	-0.65235	-0.65235	-0.65234	-0.65049	-0.61773	-0.59709	-0.59709
+NA	-0.65234	-0.65234	-0.65234	-0.65234	-0.62253	-0.61944	-0.61944

Oneway Anova

Summary of Fit

Rsquare	0.966601
Adj Rsquare	0.961035
Root Mean Square Error	0.235294
Mean of Response	2.78e-10
Observations (or Sum Wgts)	36

Analysis of Variance

Source	DF	Sum of Squares	Mean Square	F Ratio	Prob > F
Label	5	48.068497	9.61370	173.6470	<.0001 *
Error	30	1.660904	0.05536		
C. Total	35	49.729400			

Means for Oneway Anova

Level	Number	Mean	Std Error	Lower 95%	Upper 95%
50nM-NA	6	2.5245	0.09606	2.328	2.721
5nM-NA	6	0.0344	0.09606	-0.162	0.231
-NA	6	-0.6332	0.09606	-0.829	-0.437
50nM+NA	6	-0.6457	0.09606	-0.842	-0.450
5nM+NA	6	-0.6379	0.09606	-0.834	-0.442
+NA	6	-0.6421	0.09606	-0.838	-0.446

Std Error uses a pooled estimate of error variance

Means Comparisons

Comparisons with a control using Dunnett's Method

Oneway Analysis of Geranyl-PP By Label

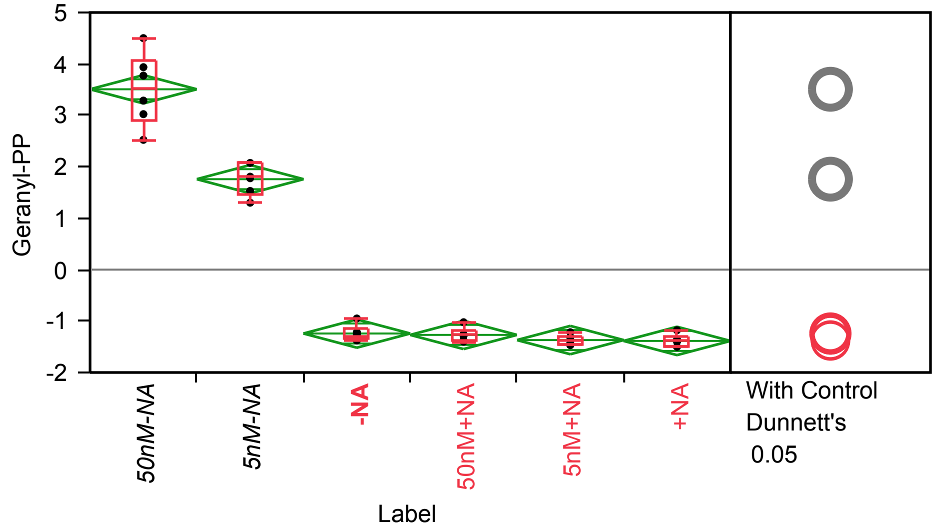

Quantiles

Level	Minimum	10%	25%	Median	75%	90%	Maximum
50nM-NA	2.524189	2.524189	2.898344	3.529392	4.077509	4.501268	4.501268
5nM-NA	1.301823	1.301823	1.472988	1.792545	2.065302	2.07496	2.07496
-NA	-1.37801	-1.37801	-1.35157	-1.28109	-1.15091	-0.94993	-0.94993
50nM+NA	-1.39915	-1.39915	-1.3795	-1.28052	-1.19882	-1.02272	-1.02272
5nM+NA	-1.46858	-1.46858	-1.45184	-1.38421	-1.29107	-1.21752	-1.21752
+NA	-1.50691	-1.50691	-1.50177	-1.38416	-1.31705	-1.17855	-1.17855

Oneway Anova

Summary of Fit

Rsquare	0.976158
Adj Rsquare	0.972185
Root Mean Square Error	0.330371
Mean of Response	2.778e-9
Observations (or Sum Wgts)	36

Analysis of Variance

Source	DF	Sum of Squares	Mean Square	F Ratio	Prob > F
Label	5	134.06295	26.8126	245.6602	<.0001 *
Error	30	3.27435	0.1091		
C. Total	35	137.33730			

Means for Oneway Anova

Level	Number	Mean	Std Error	Lower 95%	Upper 95%
50nM-NA	6	3.5073	0.13487	3.232	3.783
5nM-NA	6	1.7590	0.13487	1.484	2.034
-NA	6	-1.2418	0.13487	-1.517	-0.966
50nM+NA	6	-1.2689	0.13487	-1.544	-0.993
5nM+NA	6	-1.3694	0.13487	-1.645	-1.094
+NA	6	-1.3862	0.13487	-1.662	-1.111

Std Error uses a pooled estimate of error variance

Means Comparisons

Comparisons with a control using Dunnett's Method

Oneway Analysis of Glucaric acid By Label

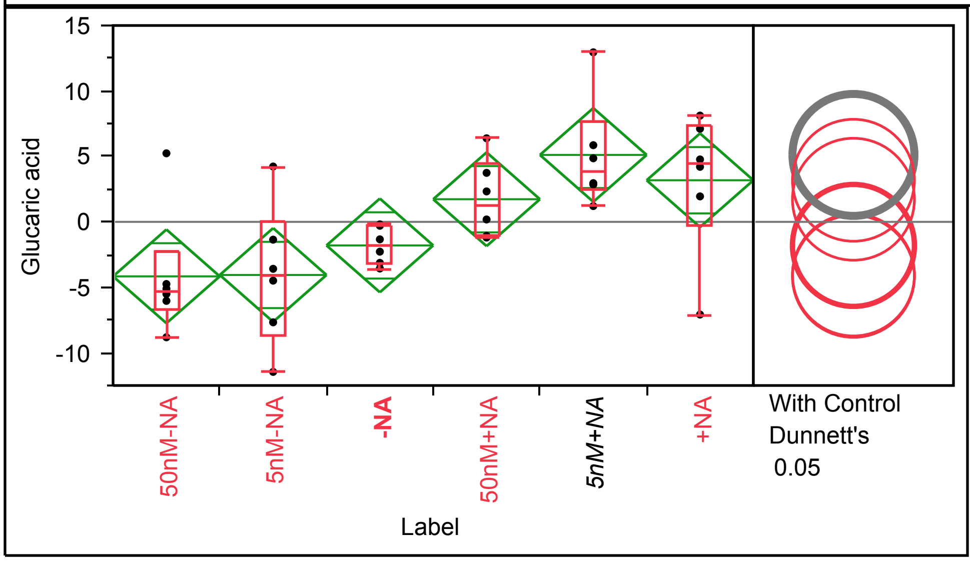

Quantiles

Level	Minimum	10%	25%	Median	75%	90%	Maximum
50nM-NA	-8.81636	-8.81636	-6.73043	-5.30733	-2.25223	5.236084	5.236084
5nM-NA	-11.4602	-11.4602	-8.62029	-4.03851	0.036419	4.225399	4.225399
-NA	-3.563	-3.563	-3.2395	-1.80589	-0.27079	-0.1923	-0.1923
50nM+NA	-1.19701	-1.19701	-1.10022	1.254928	4.40393	6.382882	6.382882
5nM+NA	1.207281	1.207281	2.440502	3.906316	7.630399	12.95026	12.95026
+NA	-7.08051	-7.08051	-0.31047	4.487595	7.362574	8.116358	8.116358

Oneway Anova

Summary of Fit

Rsquare	0.452797
Adj Rsquare	0.361596
Root Mean Square Error	4.293393
Mean of Response	-2.78e-9
Observations (or Sum Wgts)	36

Analysis of Variance

Source	DF	Sum of Squares	Mean Square	F Ratio	Prob > F
Label	5	457.5911	91.5182	4.9649	0.0020 *
Error	30	552.9968	18.4332		
C. Total	35	1010.5879			

Means for Oneway Anova

Level	Number	Mean	Std Error	Lower 95%	Upper 95%
50nM-NA	6	-4.1631	1.7528	-7.743	-0.583
5nM-NA	6	-4.0576	1.7528	-7.637	-0.478
-NA	6	-1.7993	1.7528	-5.379	1.780
50nM+NA	6	1.7287	1.7528	-1.851	5.308
5nM+NA	6	5.1131	1.7528	1.534	8.693
+NA	6	3.1781	1.7528	-0.402	6.758

Std Error uses a pooled estimate of error variance

Means Comparisons

Comparisons with a control using Dunnett's Method

Oneway Analysis of Glucose By Label

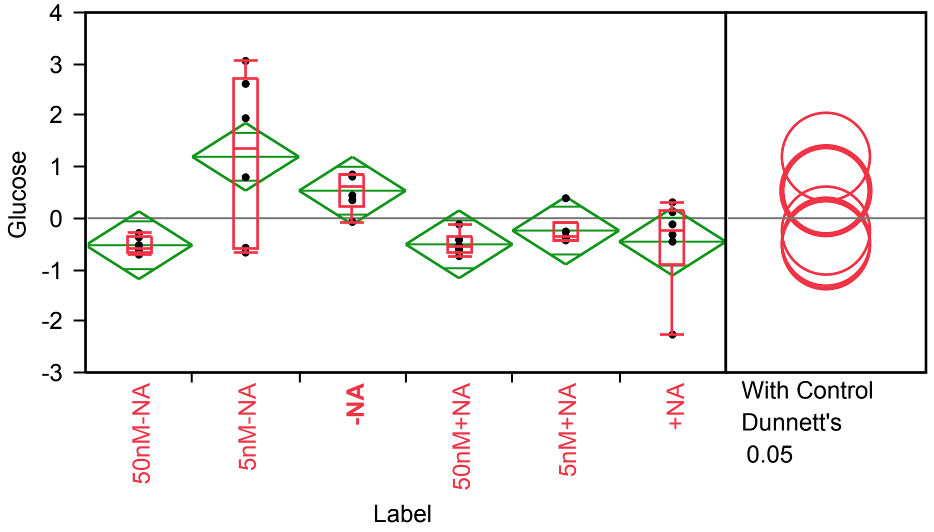

Quantiles

Level	Minimum	10%	25%	Median	75%	90%	Maximum
50nM-NA	-0.70892	-0.70892	-0.65504	-0.57261	-0.35158	-0.28835	-0.28835
5nM-NA	-0.66795	-0.66795	-0.59878	1.370437	2.7236	3.057406	3.057406
-NA	-0.07273	-0.07273	0.243188	0.625847	0.837046	0.850477	0.850477
50nM+NA	-0.7384	-0.7384	-0.67263	-0.55927	-0.3449	-0.12101	-0.12101
5nM+NA	-0.43115	-0.43115	-0.41412	-0.36549	-0.09719	0.388833	0.388833
+NA	-2.26345	-2.26345	-0.90783	-0.2218	0.165478	0.309427	0.309427

Oneway Anova

Summary of Fit

Rsquare	0.446935
Adj Rsquare	0.354757
Root Mean Square Error	0.788614
Mean of Response	2.78e-10
Observations (or Sum Wgts)	36

Analysis of Variance

Source	DF	Sum of Squares	Mean Square	F Ratio	Prob > F
Label	5	15.077110	3.01542	4.8486	0.0023 *
Error	30	18.657361	0.62191		
C. Total	35	33.734471			

Means for Oneway Anova

Level	Number	Mean	Std Error	Lower 95%	Upper 95%
50nM-NA	6	-0.5254	0.32195	-1.183	0.1321
5nM-NA	6	1.1945	0.32195	0.537	1.8520
-NA	6	0.5351	0.32195	-0.122	1.1926
50nM+NA	6	-0.5080	0.32195	-1.166	0.1495
5nM+NA	6	-0.2402	0.32195	-0.898	0.4174
+NA	6	-0.4560	0.32195	-1.114	0.2015

Std Error uses a pooled estimate of error variance

Means Comparisons

Comparisons with a control using Dunnett's Method

Oneway Analysis of glucose-6-phosphate By Label

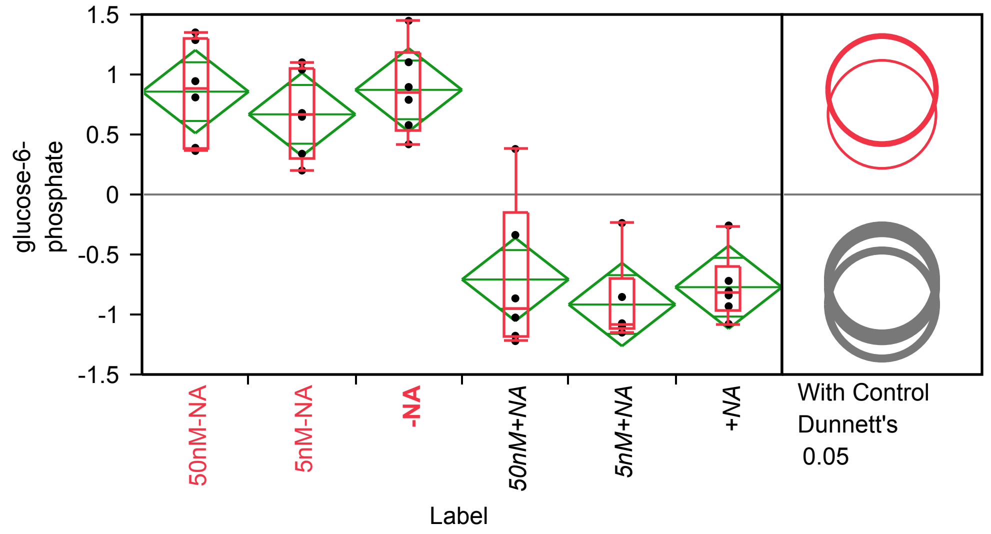

Quantiles

Level	Minimum	10%	25%	Median	75%	90%	Maximum
50nM-NA	0.363238	0.363238	0.382389	0.876824	1.303125	1.350594	1.350594
5nM-NA	0.200033	0.200033	0.304416	0.663292	1.055908	1.101032	1.101032
-NA	0.418699	0.418699	0.538559	0.842261	1.188711	1.447365	1.447365
50nM+NA	-1.22137	-1.22137	-1.18855	-0.94565	-0.15818	0.379149	0.379149
5nM+NA	-1.15063	-1.15063	-1.1153	-1.07725	-0.70008	-0.23784	-0.23784
+NA	-1.07905	-1.07905	-0.9681	-0.82268	-0.60452	-0.25836	-0.25836

Oneway Anova

Summary of Fit

Rsquare	0.818374
Adj Rsquare	0.788103
Root Mean Square Error	0.414975
Mean of Response	5.556e-9
Observations (or Sum Wgts)	36

Analysis of Variance

Source	DF	Sum of Squares	Mean Square	F Ratio	Prob > F
Label	5	23.277660	4.65553	27.0350	<.0001 *
Error	30	5.166121	0.17220		
C. Total	35	28.443782			

Means for Oneway Anova

Level	Number	Mean	Std Error	Lower 95%	Upper 95%
50nM-NA	6	0.85726	0.16941	0.511	1.203
5nM-NA	6	0.66795	0.16941	0.322	1.014
-NA	6	0.87193	0.16941	0.526	1.218
50nM+NA	6	-0.70807	0.16941	-1.054	-0.362
5nM+NA	6	-0.91678	0.16941	-1.263	-0.571
+NA	6	-0.77230	0.16941	-1.118	-0.426

Std Error uses a pooled estimate of error variance

Means Comparisons

Comparisons with a control using Dunnett's Method

Oneway Analysis of glutamate By Label

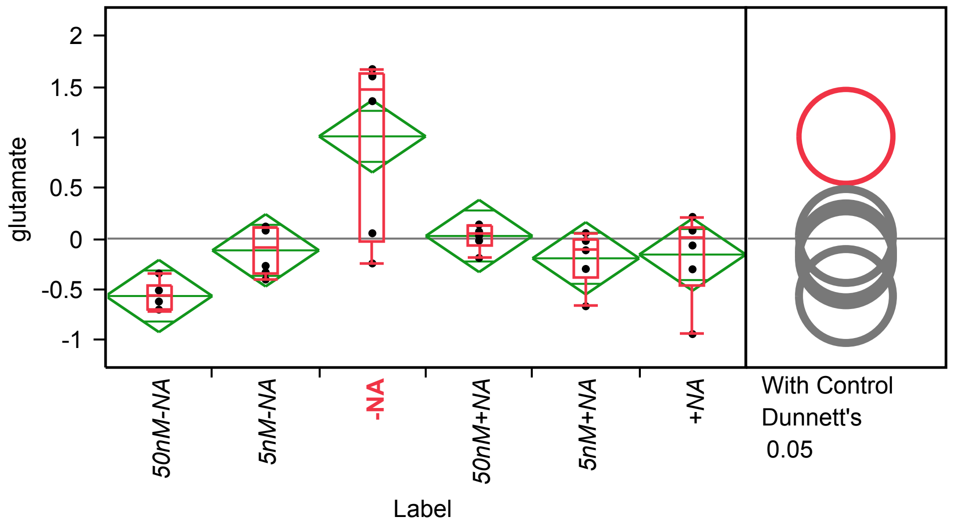

Quantiles

Level	Minimum	10%	25%	Median	75%	90%	Maximum
50nM-NA	-0.71118	-0.71118	-0.70405	-0.56747	-0.4704	-0.34368	-0.34368
5nM-NA	-0.40437	-0.40437	-0.35157	-0.09526	0.114985	0.118607	0.118607
-NA	-0.24533	-0.24533	-0.02192	1.480174	1.632984	1.674433	1.674433
50nM+NA	-0.19139	-0.19139	-0.06375	0.048792	0.132903	0.139191	0.139191
5nM+NA	-0.66835	-0.66835	-0.39212	-0.11588	-0.00361	0.053795	0.053795
+NA	-0.94353	-0.94353	-0.46335	0.003628	0.112731	0.214178	0.214178

Oneway Anova

Summary of Fit

Rsquare	0.608258
Adj Rsquare	0.542968
Root Mean Square Error	0.427583
Mean of Response	-8.3e-10
Observations (or Sum Wgts)	36

Analysis of Variance

Source	DF	Sum of Squares	Mean Square	F Ratio	Prob > F
Label	5	8.516255	1.70325	9.3162	<.0001 *
Error	30	5.484804	0.18283		
C. Total	35	14.001059			

Means for Oneway Anova

Level	Number	Mean	Std Error	Lower 95%	Upper 95%
50nM-NA	6	-0.5674	0.17456	-0.9239	-0.211
5nM-NA	6	-0.1161	0.17456	-0.4726	0.240
-NA	6	1.0102	0.17456	0.6537	1.367
50nM+NA	6	0.0258	0.17456	-0.3307	0.382
5nM+NA	6	-0.1949	0.17456	-0.5514	0.162
+NA	6	-0.1577	0.17456	-0.5142	0.199

Std Error uses a pooled estimate of error variance

Means Comparisons

Comparisons with a control using Dunnett's Method

Oneway Analysis of glutamine By Label

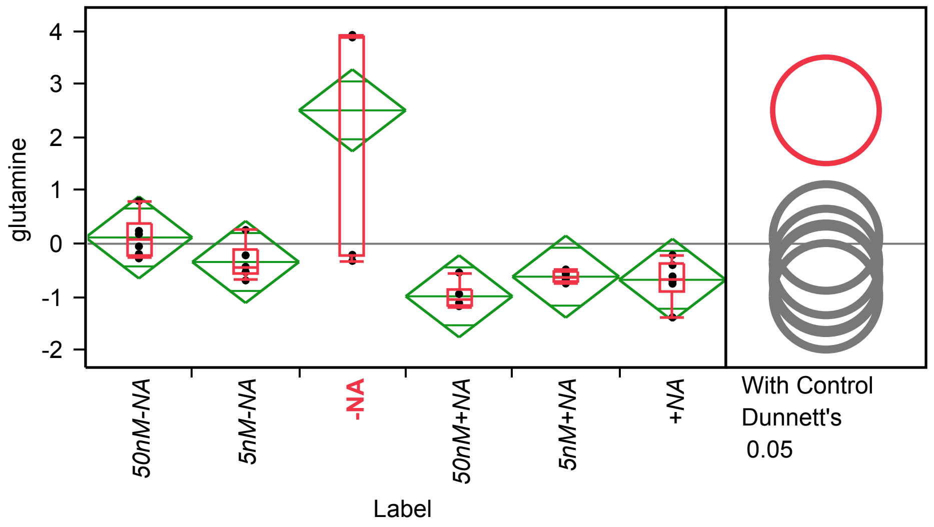

Quantiles

Level	Minimum	10%	25%	Median	75%	90%	Maximum
50nM-NA	-0.27393	-0.27393	-0.2124	0.06586	0.382223	0.802359	0.802359
5nM-NA	-0.68747	-0.68747	-0.5697	-0.43754	-0.0979	0.258	0.258
-NA	-0.32165	-0.32165	-0.23606	3.867905	3.901309	3.918465	3.918465
50nM+NA	-1.19013	-1.19013	-1.17619	-1.03631	-0.84185	-0.5396	-0.5396
5nM+NA	-0.74565	-0.74565	-0.71309	-0.62244	-0.51838	-0.48292	-0.48292
+NA	-1.3809	-1.3809	-0.90706	-0.65801	-0.35244	-0.21188	-0.21188

Oneway Anova

Summary of Fit

Rsquare	0.659275
Adj Rsquare	0.602488
Root Mean Square Error	0.921162
Mean of Response	5.56e-10
Observations (or Sum Wgts)	36

Analysis of Variance

Source	DF	Sum of Squares	Mean Square	F Ratio	Prob > F
Label	5	49.255617	9.85112	11.6095	<.0001 *
Error	30	25.456174	0.84854		
C. Total	35	74.711791			

Means for Oneway Anova

Level	Number	Mean	Std Error	Lower 95%	Upper 95%
50nM-NA	6	0.1184	0.37606	-0.650	0.886
5nM-NA	6	-0.3419	0.37606	-1.110	0.426
-NA	6	2.5034	0.37606	1.735	3.271
50nM+NA	6	-0.9861	0.37606	-1.754	-0.218
5nM+NA	6	-0.6176	0.37606	-1.386	0.150
+NA	6	-0.6762	0.37606	-1.444	0.092

Std Error uses a pooled estimate of error variance

Means Comparisons

Comparisons with a control using Dunnett's Method

Oneway Analysis of Glutathionedisufide By Label

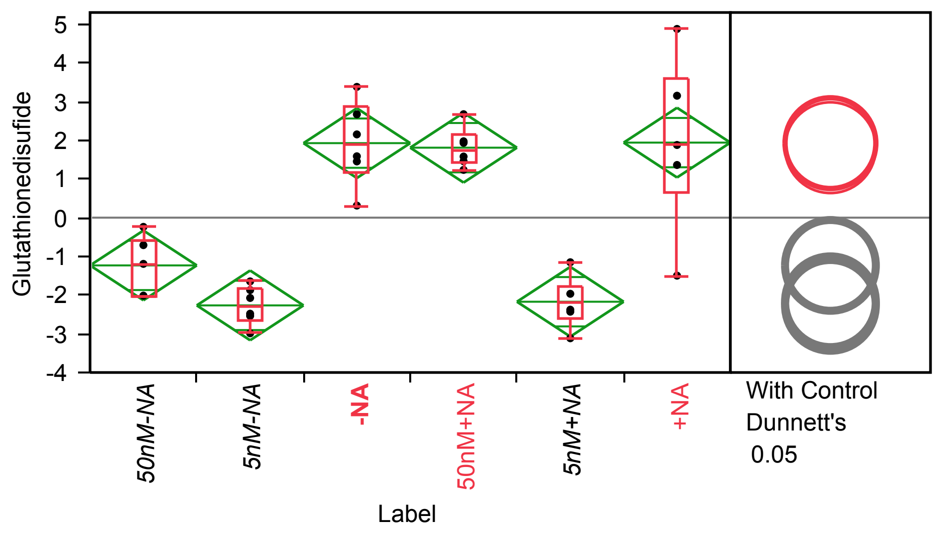

Quantiles

Level	Minimum	10%	25%	Median	75%	90%	Maximum
50nM-NA	-2.027	-2.027	-2.01739	-1.2056	-0.59171	-0.2382	-0.2382
5nM-NA	-2.98708	-2.98708	-2.65039	-2.27779	-1.81437	-1.64682	-1.64682
-NA	0.312319	0.312319	1.167857	1.868264	2.84698	3.377978	3.377978
50nM+NA	1.233991	1.233991	1.401866	1.754873	2.147729	2.672373	2.672373
5nM+NA	-3.11517	-3.11517	-2.6007	-2.18419	-1.76299	-1.15212	-1.15212
+NA	-1.49789	-1.49789	0.645958	1.87243	3.581355	4.875277	4.875277

Oneway Anova

Summary of Fit

Rsquare	0.791077
Adj Rsquare	0.756257
Root Mean Square Error	1.0806
Mean of Response	-5.56e-9
Observations (or Sum Wgts)	36

Analysis of Variance

Source	DF	Sum of Squares	Mean Square	F Ratio	Prob > F
Label	5	132.64316	26.5286	22.7188	<.0001 *
Error	30	35.03091	1.1677		
C. Total	35	167.67407			

Means for Oneway Anova

Level	Number	Mean	Std Error	Lower 95%	Upper 95%
50nM-NA	6	-1.2334	0.44115	-2.134	-0.332
5nM-NA	6	-2.2663	0.44115	-3.167	-1.365
-NA	6	1.9250	0.44115	1.024	2.826
50nM+NA	6	1.8078	0.44115	0.907	2.709
5nM+NA	6	-2.1719	0.44115	-3.073	-1.271
+NA	6	1.9388	0.44115	1.038	2.840

Std Error uses a pooled estimate of error variance

Means Comparisons

Comparisons with a control using Dunnett's Method

Oneway Analysis of Glutathione By Label

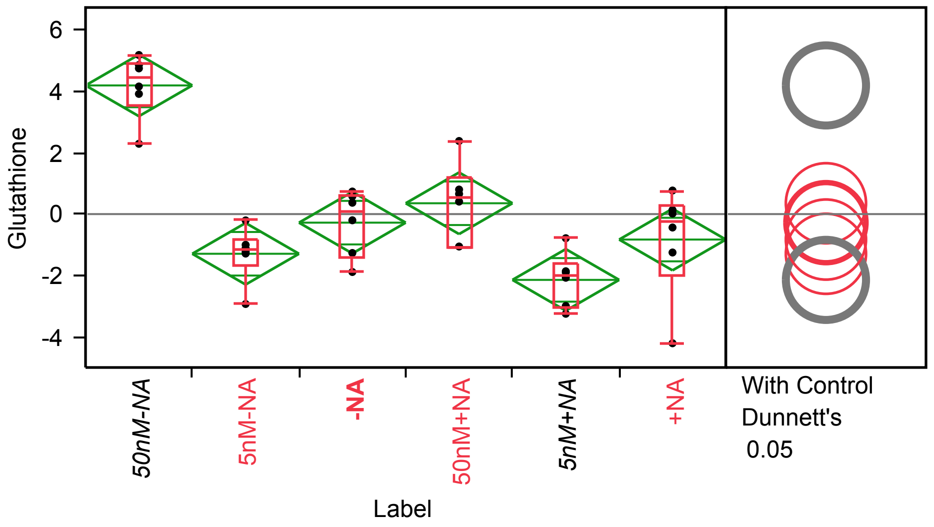

Quantiles

Level	Minimum	10%	25%	Median	75%	90%	Maximum
50nM-NA	2.291961	2.291961	3.511113	4.453618	4.940531	5.18516	5.18516
5nM-NA	-2.92972	-2.92972	-1.69757	-1.16762	-0.80271	-0.20538	-0.20538
-NA	-1.88716	-1.88716	-1.4228	0.084324	0.616606	0.734809	0.734809
50nM+NA	-1.07198	-1.07198	-1.06095	0.532814	1.195318	2.38212	2.38212
5nM+NA	-3.24846	-3.24846	-3.05783	-1.98691	-1.59407	-0.78659	-0.78659
+NA	-4.20943	-4.20943	-1.9912	-0.20985	0.29235	0.767958	0.767958

Oneway Anova

Summary of Fit

Rsquare	0.7735
Adj Rsquare	0.73575
Root Mean Square Error	1.203875
Mean of Response	9.87e-17
Observations (or Sum Wgts)	36

Analysis of Variance

Source	DF	Sum of Squares	Mean Square	F Ratio	Prob > F
Label	5	148.48284	29.6966	20.4901	<.0001 *
Error	30	43.47942	1.4493		
C. Total	35	191.96226			

Means for Oneway Anova

Level	Number	Mean	Std Error	Lower 95%	Upper 95%
50nM-NA	6	4.1935	0.49148	3.190	5.197
5nM-NA	6	-1.2932	0.49148	-2.297	-0.289
-NA	6	-0.2791	0.49148	-1.283	0.725
50nM+NA	6	0.3530	0.49148	-0.651	1.357
5nM+NA	6	-2.1444	0.49148	-3.148	-1.141
+NA	6	-0.8299	0.49148	-1.834	0.174

Std Error uses a pooled estimate of error variance

Means Comparisons

Comparisons with a control using Dunnett's Method

Oneway Analysis of Glycerophosphocholine By Label

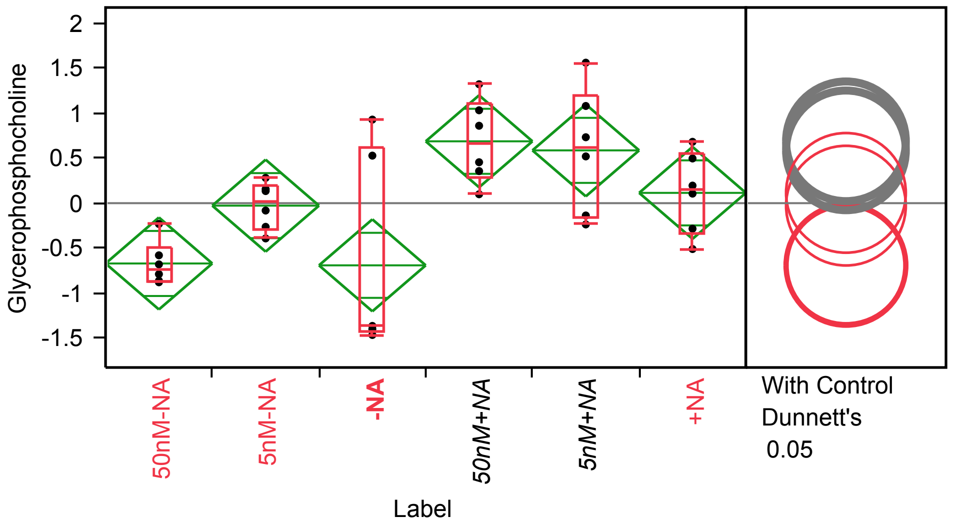

Quantiles

Level	Minimum	10%	25%	Median	75%	90%	Maximum
50nM-NA	-0.8792	-0.8792	-0.86686	-0.73449	-0.49261	-0.23136	-0.23136
5nM-NA	-0.39173	-0.39173	-0.29523	0.026328	0.187457	0.280789	0.280789
-NA	-1.46514	-1.46514	-1.41583	-1.36955	0.628447	0.928805	0.928805
50nM+NA	0.099205	0.099205	0.292258	0.657757	1.104913	1.321896	1.321896
5nM+NA	-0.23627	-0.23627	-0.16176	0.626334	1.200655	1.561521	1.561521
+NA	-0.50983	-0.50983	-0.34044	0.150648	0.543191	0.681841	0.681841

Oneway Anova

Summary of Fit

Rsquare	0.48325
Adj Rsquare	0.397125
Root Mean Square Error	0.613179
Mean of Response	-2.8e-10
Observations (or Sum Wgts)	36

Analysis of Variance

Source	DF	Sum of Squares	Mean Square	F Ratio	Prob > F
Label	5	10.548381	2.10968	5.6110	0.0009 *
Error	30	11.279637	0.37599		
C. Total	35	21.828018			

Means for Oneway Anova

Level	Number	Mean	Std Error	Lower 95%	Upper 95%
50nM-NA	6	-0.67033	0.25033	-1.182	-0.159
5nM-NA	6	-0.02750	0.25033	-0.539	0.484
-NA	6	-0.69108	0.25033	-1.202	-0.180
50nM+NA	6	0.68763	0.25033	0.176	1.199
5nM+NA	6	0.58689	0.25033	0.076	1.098
+NA	6	0.11438	0.25033	-0.397	0.626

Std Error uses a pooled estimate of error variance

Means Comparisons

Comparisons with a control using Dunnett's Method

Oneway Analysis of glycine By Label

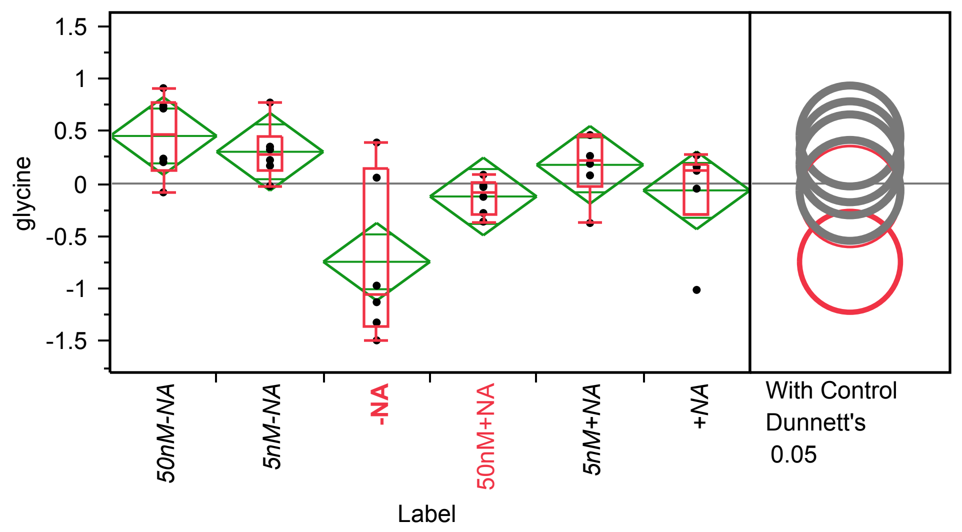

Quantiles

Level	Minimum	10%	25%	Median	75%	90%	Maximum
50nM-NA	-0.0837	-0.0837	0.131481	0.474619	0.779443	0.90627	0.90627
5nM-NA	-0.02348	-0.02348	0.1211	0.272628	0.454923	0.769412	0.769412
-NA	-1.48964	-1.48964	-1.36224	-1.04841	0.139235	0.389525	0.389525
50nM+NA	-0.36181	-0.36181	-0.30046	-0.07944	0.005856	0.083247	0.083247
5nM+NA	-0.3749	-0.3749	-0.0355	0.22495	0.454154	0.460549	0.460549
+NA	-1.01049	-1.01049	-0.28903	0.120895	0.186463	0.268468	0.268468

Oneway Anova

Summary of Fit

Rsquare	0.479417
Adj Rsquare	0.392654
Root Mean Square Error	0.441858
Mean of Response	5.56e-10
Observations (or Sum Wgts)	36

Analysis of Variance

Source	DF	Sum of Squares	Mean Square	F Ratio	Prob > F
Label	5	5.394005	1.07880	5.5255	0.0010 *
Error	30	5.857161	0.19524		
C. Total	35	11.251166			

Means for Oneway Anova

Level	Number	Mean	Std Error	Lower 95%	Upper 95%
50nM-NA	6	0.45203	0.18039	0.084	0.8204
5nM-NA	6	0.30176	0.18039	-0.067	0.6702
-NA	6	-0.74348	0.18039	-1.112	-0.3751
50nM+NA	6	-0.12290	0.18039	-0.491	0.2455
5nM+NA	6	0.17753	0.18039	-0.191	0.5459
+NA	6	-0.06494	0.18039	-0.433	0.3035

Std Error uses a pooled estimate of error variance

Means Comparisons

Comparisons with a control using Dunnett's Method

Oneway Analysis of GMP By Label

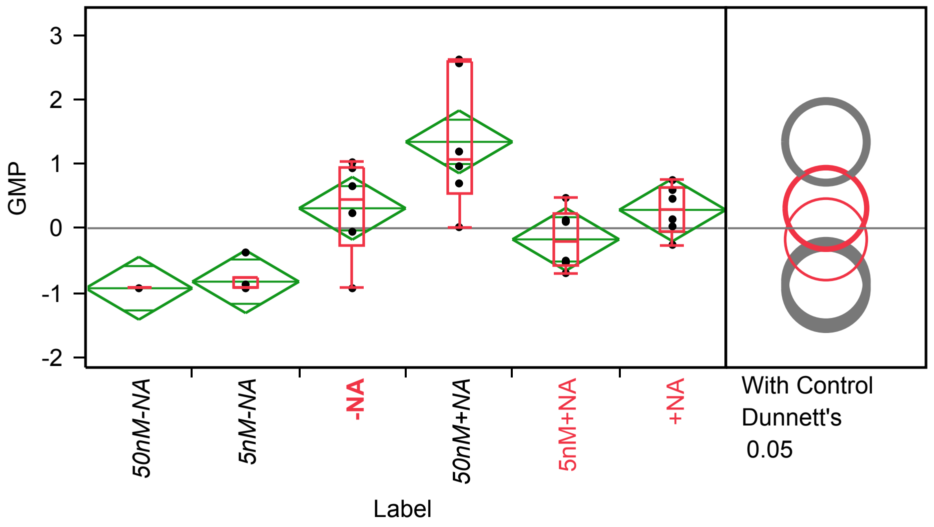

Quantiles

Level	Minimum	10%	25%	Median	75%	90%	Maximum
50nM-NA	-0.93246	-0.93246	-0.93245	-0.93244	-0.93244	-0.93244	-0.93244
5nM-NA	-0.93245	-0.93245	-0.93245	-0.93244	-0.75005	-0.37669	-0.37669
-NA	-0.93245	-0.93245	-0.27404	0.444087	0.955556	1.02413	1.02413
50nM+NA	0.016578	0.016578	0.526376	1.076744	2.576871	2.619646	2.619646
5nM+NA	-0.69734	-0.69734	-0.57535	-0.20248	0.21346	0.470014	0.470014
+NA	-0.25783	-0.25783	-0.04343	0.298047	0.632571	0.7475	0.7475

Oneway Anova

Summary of Fit

Rsquare	0.676585
Adj Rsquare	0.622682
Root Mean Square Error	0.5838
Mean of Response	8.64e-17
Observations (or Sum Wgts)	36

Analysis of Variance

Source	DF	Sum of Squares	Mean Square	F Ratio	Prob > F
Label	5	21.389996	4.27800	12.5520	<.0001 *
Error	30	10.224664	0.34082		
C. Total	35	31.614660			

Means for Oneway Anova

Level	Number	Mean	Std Error	Lower 95%	Upper 95%
50nM-NA	6	-0.9324	0.23834	-1.419	-0.446
5nM-NA	6	-0.8302	0.23834	-1.317	-0.343
-NA	6	0.3097	0.23834	-0.177	0.796
50nM+NA	6	1.3414	0.23834	0.855	1.828
5nM+NA	6	-0.1732	0.23834	-0.660	0.314
+NA	6	0.2847	0.23834	-0.202	0.771

Std Error uses a pooled estimate of error variance

Means Comparisons

Comparisons with a control using Dunnett's Method

Oneway Analysis of Guanidoacetic acid By Label

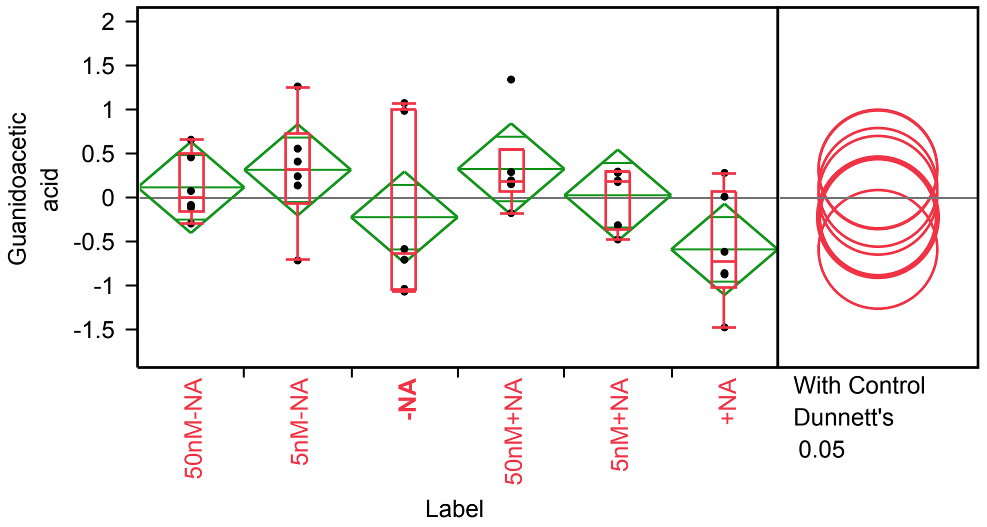

Quantiles

Level	Minimum	10%	25%	Median	75%	90%	Maximum
50nM-NA	-0.29067	-0.29067	-0.15404	0.00022	0.512286	0.662661	0.662661
5nM-NA	-0.70971	-0.70971	-0.07051	0.330772	0.738156	1.268162	1.268162
-NA	-1.06466	-1.06466	-1.04151	-0.64141	1.0136	1.080081	1.080081
50nM+NA	-0.17215	-0.17215	0.071861	0.17859	0.557645	1.347148	1.347148
5nM+NA	-0.47174	-0.47174	-0.35004	0.189997	0.290564	0.30002	0.30002
+NA	-1.47141	-1.47141	-1.01961	-0.7331	0.082552	0.28516	0.28516

Oneway Anova

Summary of Fit

Rsquare	0.241568
Adj Rsquare	0.115163
Root Mean Square Error	0.622188
Mean of Response	5.56e-10
Observations (or Sum Wgts)	36

Analysis of Variance

Source	DF	Sum of Squares	Mean Square	F Ratio	Prob > F
Label	5	3.699033	0.739807	1.9111	0.1220
Error	30	11.613541	0.387118		
C. Total	35	15.312574			

Means for Oneway Anova

Level	Number	Mean	Std Error	Lower 95%	Upper 95%
50nM-NA	6	0.12102	0.25401	-0.398	0.6398
5nM-NA	6	0.32067	0.25401	-0.198	0.8394
-NA	6	-0.21829	0.25401	-0.737	0.3005
50nM+NA	6	0.32998	0.25401	-0.189	0.8487
5nM+NA	6	0.03103	0.25401	-0.488	0.5498
+NA	6	-0.58441	0.25401	-1.103	-0.0657

Std Error uses a pooled estimate of error variance

Means Comparisons

Comparisons with a control using Dunnett's Method

Oneway Analysis of Histidine By Label

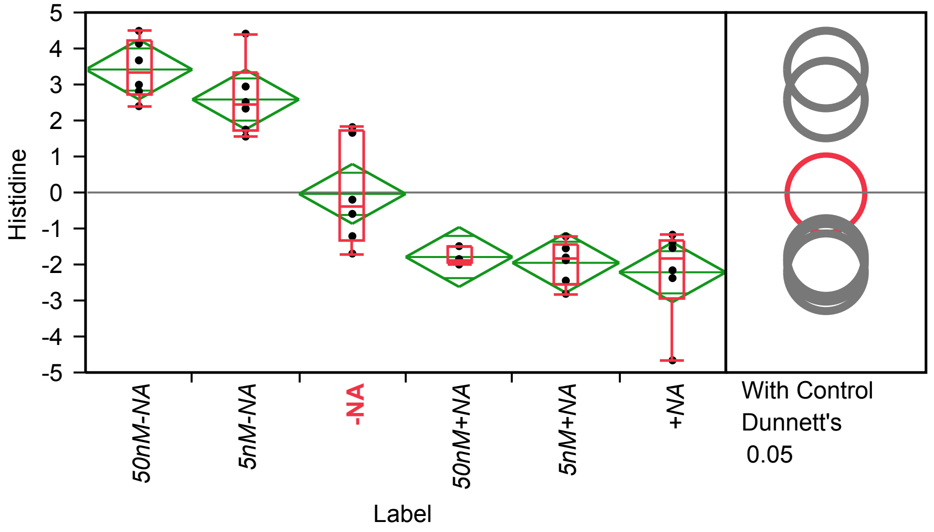

Quantiles

Level	Minimum	10%	25%	Median	75%	90%	Maximum
50nM-NA	2.396812	2.396812	2.70878	3.331554	4.22419	4.489771	4.489771
5nM-NA	1.552602	1.552602	1.697271	2.422843	3.310296	4.411681	4.411681
-NA	-1.69649	-1.69649	-1.33369	-0.39642	1.697261	1.816042	1.816042
50nM+NA	-2.00038	-2.00038	-1.97109	-1.90063	-1.49734	-1.49038	-1.49038
5nM+NA	-2.81518	-2.81518	-2.53983	-1.84318	-1.46917	-1.21841	-1.21841
+NA	-4.66148	-4.66148	-2.94884	-1.85183	-1.32736	-1.17257	-1.17257

Oneway Anova

Summary of Fit

Rsquare	0.859719
Adj Rsquare	0.836339
Root Mean Square Error	0.99411
Mean of Response	-2.78e-9
Observations (or Sum Wgts)	36

Analysis of Variance

Source	DF	Sum of Squares	Mean Square	F Ratio	Prob > F
Label	5	181.69713	36.3394	36.7713	<.0001 *
Error	30	29.64763	0.9883		
C. Total	35	211.34476			

Means for Oneway Anova

Level	Number	Mean	Std Error	Lower 95%	Upper 95%
50nM-NA	6	3.4164	0.40584	2.588	4.245
5nM-NA	6	2.5831	0.40584	1.754	3.412
-NA	6	-0.0381	0.40584	-0.867	0.791
50nM+NA	6	-1.7922	0.40584	-2.621	-0.963
5nM+NA	6	-1.9535	0.40584	-2.782	-1.125
+NA	6	-2.2158	0.40584	-3.045	-1.387

Std Error uses a pooled estimate of error variance

Means Comparisons

Comparisons with a control using Dunnett's Method

Oneway Analysis of homocysteine By Label

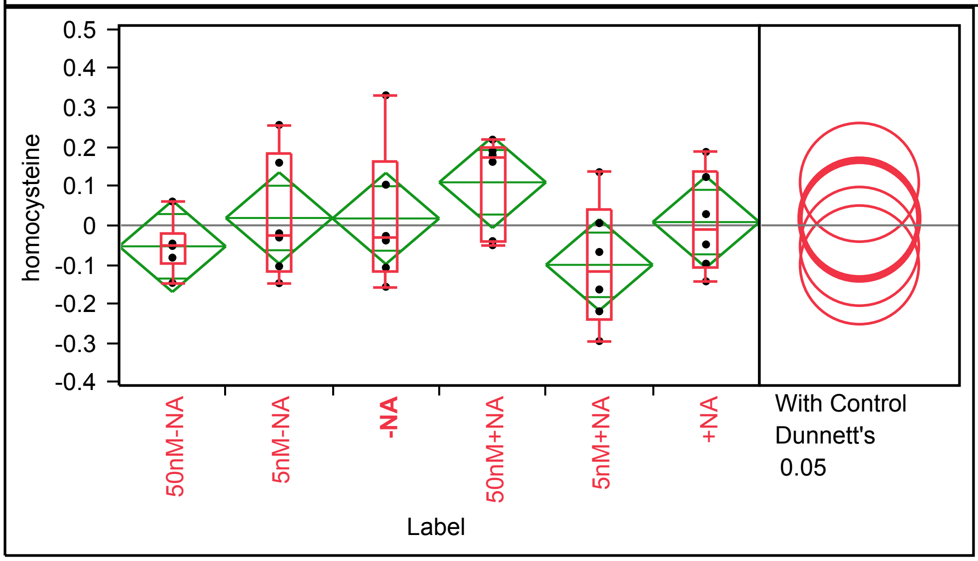

Quantiles

Level	Minimum	10%	25%	Median	75%	90%	Maximum
50nM-NA	-0.14661	-0.14661	-0.09839	-0.05292	-0.01968	0.060654	0.060654
5nM-NA	-0.14717	-0.14717	-0.11505	-0.02564	0.183488	0.255683	0.255683
-NA	-0.1565	-0.1565	-0.11977	-0.03279	0.160391	0.331162	0.331162
50nM+NA	-0.0506	-0.0506	-0.04278	0.170271	0.196664	0.218087	0.218087
5nM+NA	-0.29468	-0.29468	-0.23783	-0.11563	0.038025	0.135448	0.135448
+NA	-0.14258	-0.14258	-0.10892	-0.01006	0.139041	0.18701	0.18701

Oneway Anova

Summary of Fit

Rsquare	0.209479
Adj Rsquare	0.077725
Root Mean Square Error	0.139306
Mean of Response	2.31e-18
Observations (or Sum Wgts)	36

Analysis of Variance

Source	DF	Sum of Squares	Mean Square	F Ratio	Prob > F
Label	5	0.15427167	0.030854	1.5899	0.1931
Error	30	0.58218393	0.019406		
C. Total	35	0.73645560			

Means for Oneway Anova

Level	Number	Mean	Std Error	Lower 95%	Upper 95%
50nM-NA	6	-0.05343	0.05687	-0.1696	0.06272
5nM-NA	6	0.01872	0.05687	-0.0974	0.13486
-NA	6	0.01751	0.05687	-0.0986	0.13365
50nM+NA	6	0.10956	0.05687	-0.0066	0.22571
5nM+NA	6	-0.10064	0.05687	-0.2168	0.01551
+NA	6	0.00828	0.05687	-0.1079	0.12443

Std Error uses a pooled estimate of error variance

Means Comparisons

Comparisons with a control using Dunnett's Method

Oneway Analysis of homoserine By Label

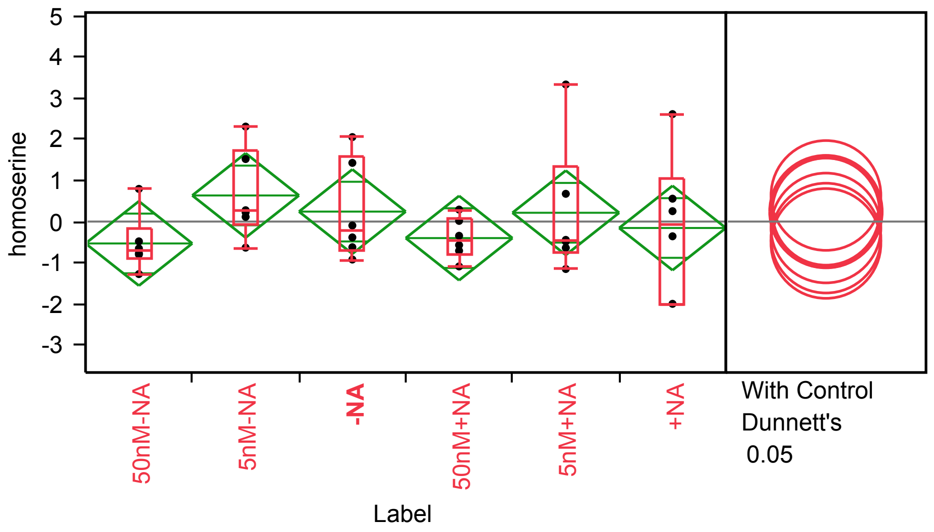

Quantiles

Level	Minimum	10%	25%	Median	75%	90%	Maximum
50nM-NA	-1.27796	-1.27796	-0.9208	-0.71518	-0.1627	0.795204	0.795204
5nM-NA	-0.63496	-0.63496	-0.07339	0.250005	1.719095	2.311178	2.311178
-NA	-0.92388	-0.92388	-0.69933	-0.2408	1.585252	2.055821	2.055821
50nM+NA	-1.09197	-1.09197	-0.80649	-0.4597	0.085389	0.291284	0.291284
5nM+NA	-1.1546	-1.1546	-0.76882	-0.46635	1.3407	3.336053	3.336053
+NA	-2.00254	-2.00254	-2.00254	-0.05258	1.071292	2.614902	2.614902

Oneway Anova

Summary of Fit

Rsquare	0.114228
Adj Rsquare	-0.0334
Root Mean Square Error	1.231863
Mean of Response	-2.5e-17
Observations (or Sum Wgts)	36

Analysis of Variance

Source	DF	Sum of Squares	Mean Square	F Ratio	Prob > F
Label	5	5.870812	1.17416	0.7738	0.5763
Error	30	45.524611	1.51749		
C. Total	35	51.395423			

Means for Oneway Anova

Level	Number	Mean	Std Error	Lower 95%	Upper 95%
50nM-NA	6	-0.53281	0.50291	-1.560	0.4943
5nM-NA	6	0.63529	0.50291	-0.392	1.6624
-NA	6	0.24238	0.50291	-0.785	1.2694
50nM+NA	6	-0.40244	0.50291	-1.430	0.6246
5nM+NA	6	0.21402	0.50291	-0.813	1.2411
+NA	6	-0.15643	0.50291	-1.184	0.8706

Std Error uses a pooled estimate of error variance

Means Comparisons

Comparisons with a control using Dunnett's Method

Oneway Analysis of Homoserine lacton By Label

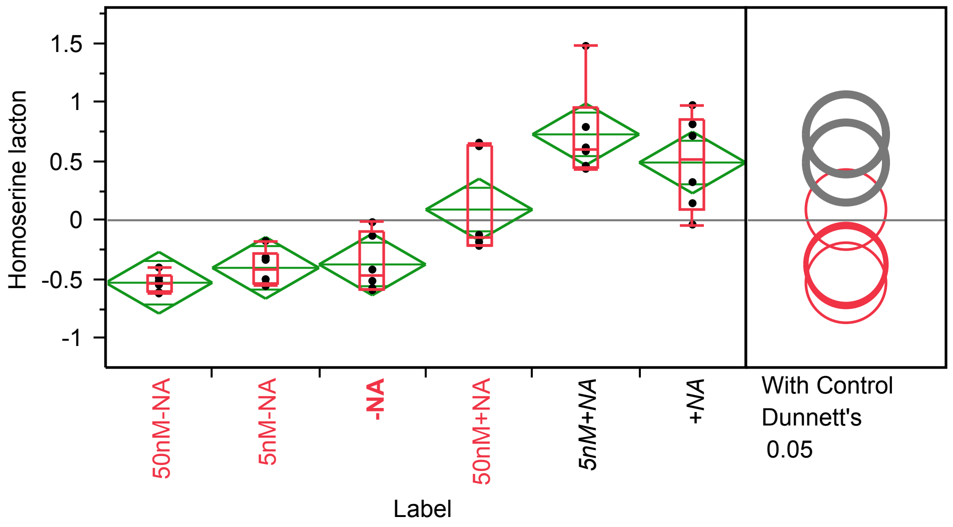

Quantiles

Level	Minimum	10%	25%	Median	75%	90%	Maximum
50nM-NA	-0.61904	-0.61904	-0.61119	-0.52901	-0.46454	-0.40012	-0.40012
5nM-NA	-0.55673	-0.55673	-0.54132	-0.41702	-0.27948	-0.17558	-0.17558
-NA	-0.5909	-0.5909	-0.57853	-0.46538	-0.10251	-0.01632	-0.01632
50nM+NA	-0.21739	-0.21739	-0.21667	-0.1532	0.633898	0.656158	0.656158
5nM+NA	0.435353	0.435353	0.454057	0.600126	0.960508	1.474697	1.474697
+NA	-0.03644	-0.03644	0.098973	0.517953	0.852732	0.974044	0.974044

Oneway Anova

Summary of Fit

Rsquare	0.735564
Adj Rsquare	0.691492
Root Mean Square Error	0.312257
Mean of Response	-8.3e-10
Observations (or Sum Wgts)	36

Analysis of Variance

Source	DF	Sum of Squares	Mean Square	F Ratio	Prob > F
Label	5	8.136639	1.62733	16.6898	<.0001 *
Error	30	2.925125	0.09750		
C. Total	35	11.061765			

Means for Oneway Anova

Level	Number	Mean	Std Error	Lower 95%	Upper 95%
50nM-NA	6	-0.52863	0.12748	-0.7890	-0.2683
5nM-NA	6	-0.40277	0.12748	-0.6631	-0.1424
-NA	6	-0.37394	0.12748	-0.6343	-0.1136
50nM+NA	6	0.09040	0.12748	-0.1699	0.3507
5nM+NA	6	0.72662	0.12748	0.4663	0.9870
+NA	6	0.48832	0.12748	0.2280	0.7487

Std Error uses a pooled estimate of error variance

Means Comparisons

Comparisons with a control using Dunnett's Method

Oneway Analysis of Hydroxyisocaproic acid By Label

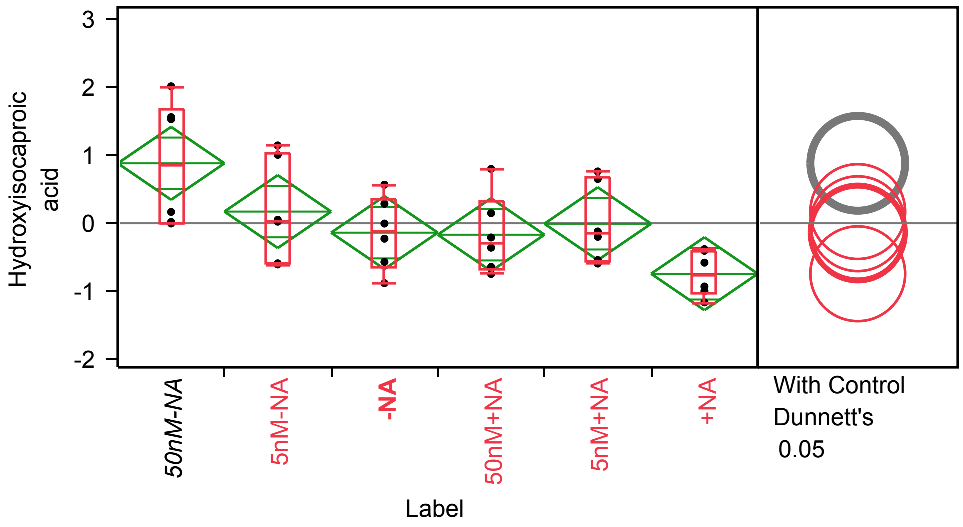

Quantiles

Level	Minimum	10%	25%	Median	75%	90%	Maximum
50nM-NA	-0.00278	-0.00278	0.014133	0.851967	1.681041	2.022351	2.022351
5nM-NA	-0.60731	-0.60731	-0.5951	0.037873	1.045042	1.149465	1.149465
-NA	-0.88346	-0.88346	-0.64868	-0.11588	0.357721	0.568942	0.568942
50nM+NA	-0.74866	-0.74866	-0.66973	-0.285	0.312579	0.802656	0.802656
5nM+NA	-0.59454	-0.59454	-0.55547	-0.16044	0.681733	0.765421	0.765421
+NA	-1.16745	-1.16745	-1.04159	-0.75732	-0.40004	-0.38155	-0.38155

Oneway Anova

Summary of Fit

Rsquare	0.404951
Adj Rsquare	0.305776
Root Mean Square Error	0.645013
Mean of Response	2.78e-11
Observations (or Sum Wgts)	36

Analysis of Variance

Source	DF	Sum of Squares	Mean Square	F Ratio	Prob > F
Label	5	8.493919	1.69878	4.0832	0.0060 *
Error	30	12.481271	0.41604		
C. Total	35	20.975191			

Means for Oneway Anova

Level	Number	Mean	Std Error	Lower 95%	Upper 95%
50nM-NA	6	0.88509	0.26333	0.347	1.423
5nM-NA	6	0.17285	0.26333	-0.365	0.711
-NA	6	-0.13823	0.26333	-0.676	0.400
50nM+NA	6	-0.16837	0.26333	-0.706	0.369
5nM+NA	6	-0.00643	0.26333	-0.544	0.531
+NA	6	-0.74491	0.26333	-1.283	-0.207

Std Error uses a pooled estimate of error variance

Means Comparisons

Comparisons with a control using Dunnett's Method

Oneway Analysis of Hydroxylamine By Label

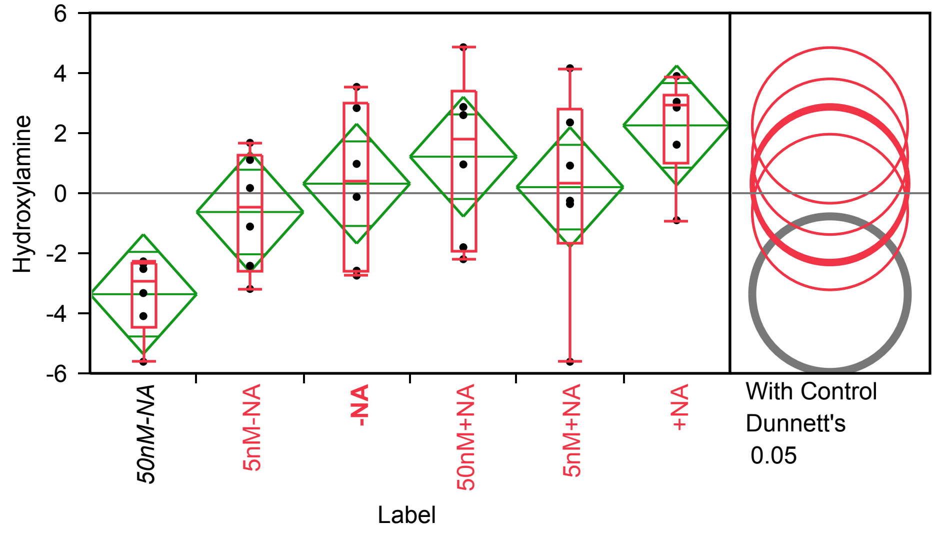

Quantiles

Level	Minimum	10%	25%	Median	75%	90%	Maximum
50nM-NA	-5.61278	-5.61278	-4.47355	-2.92529	-2.33201	-2.27141	-2.27141
5nM-NA	-3.18794	-3.18794	-2.61104	-0.47017	1.249926	1.676085	1.676085
-NA	-2.74385	-2.74385	-2.62208	0.426193	3.011292	3.538255	3.538255
50nM+NA	-2.19812	-2.19812	-1.90011	1.778926	3.367574	4.859246	4.859246
5nM+NA	-5.61279	-5.61279	-1.67494	0.333926	2.806695	4.160799	4.160799
+NA	-0.90069	-0.90069	0.9863	2.944526	3.256548	3.899624	3.899624

Oneway Anova

Summary of Fit

Rsquare	0.392356
Adj Rsquare	0.291083
Root Mean Square Error	2.38857
Mean of Response	8.333e-9
Observations (or Sum Wgts)	36

Analysis of Variance

Source	DF	Sum of Squares	Mean Square	F Ratio	Prob > F
Label	5	110.51703	22.1034	3.8742	0.0079 *
Error	30	171.15800	5.7053		
C. Total	35	281.67503			

Means for Oneway Anova

Level	Number	Mean	Std Error	Lower 95%	Upper 95%
50nM-NA	6	-3.3635	0.97513	-5.355	-1.372
5nM-NA	6	-0.6272	0.97513	-2.619	1.364
-NA	6	0.3168	0.97513	-1.675	2.308
50nM+NA	6	1.2148	0.97513	-0.777	3.206
5nM+NA	6	0.2015	0.97513	-1.790	2.193
+NA	6	2.2576	0.97513	0.266	4.249

Std Error uses a pooled estimate of error variance

Means Comparisons

Comparisons with a control using Dunnett's Method

Oneway Analysis of hydroxyproline By Label

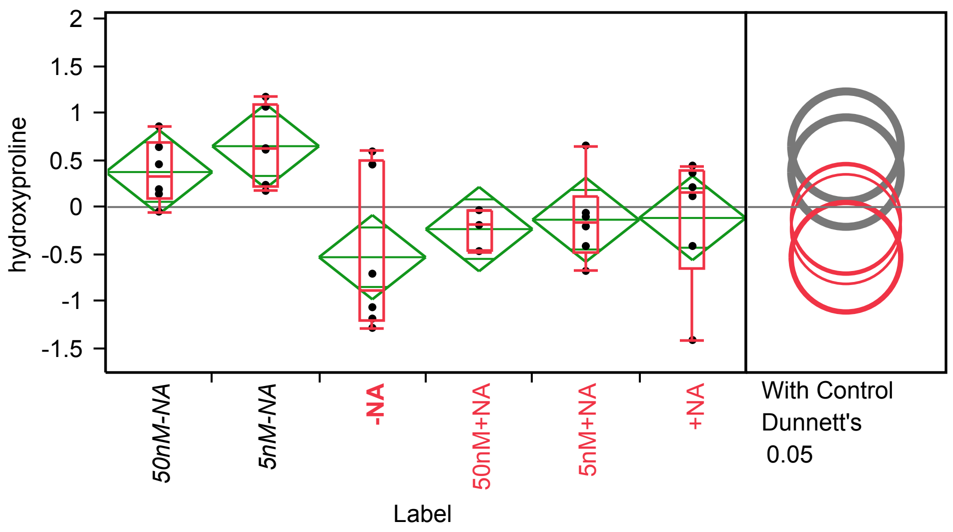

Quantiles

Level	Minimum	10%	25%	Median	75%	90%	Maximum
50nM-NA	-0.04847	-0.04847	0.0926	0.322093	0.692487	0.857381	0.857381
5nM-NA	0.172243	0.172243	0.219486	0.617522	1.092132	1.171232	1.171232
-NA	-1.28353	-1.28353	-1.20955	-0.88595	0.486795	0.590891	0.590891
50nM+NA	-0.47627	-0.47627	-0.47019	-0.19579	-0.03702	-0.03311	-0.03311
5nM+NA	-0.67817	-0.67817	-0.4803	-0.15413	0.11735	0.653481	0.653481
+NA	-1.41274	-1.41274	-0.6634	0.163305	0.382484	0.440172	0.440172

Oneway Anova

Summary of Fit

Rsquare	0.391896
Adj Rsquare	0.290546
Root Mean Square Error	0.536324
Mean of Response	-2.8e-10
Observations (or Sum Wgts)	36

Analysis of Variance

Source	DF	Sum of Squares	Mean Square	F Ratio	Prob > F
Label	5	5.561218	1.11224	3.8667	0.0080 *
Error	30	8.629311	0.28764		
C. Total	35	14.190530			

Means for Oneway Anova

Level	Number	Mean	Std Error	Lower 95%	Upper 95%
50nM-NA	6	0.37171	0.21895	-0.0755	0.819
5nM-NA	6	0.64659	0.21895	0.1994	1.094
-NA	6	-0.53289	0.21895	-0.9801	-0.086
50nM+NA	6	-0.23458	0.21895	-0.6817	0.213
5nM+NA	6	-0.13477	0.21895	-0.5819	0.312
+NA	6	-0.11605	0.21895	-0.5632	0.331

Std Error uses a pooled estimate of error variance

Means Comparisons

Comparisons with a control using Dunnett's Method

Oneway Analysis of IDP By Label

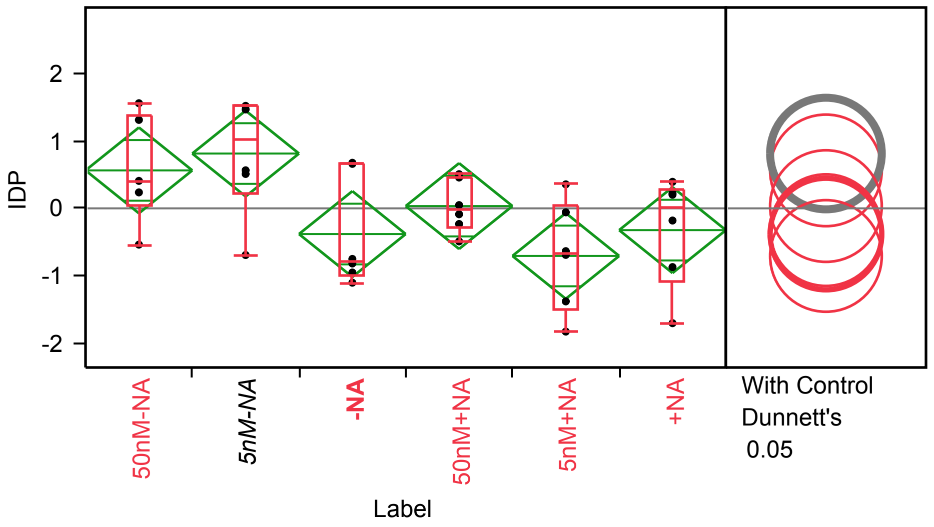

Quantiles

Level	Minimum	10%	25%	Median	75%	90%	Maximum
50nM-NA	-0.53759	-0.53759	0.0431	0.404236	1.373041	1.55847	1.55847
5nM-NA	-0.692	-0.692	0.209356	1.015453	1.510742	1.51925	1.51925
-NA	-1.10205	-1.10205	-0.989	-0.78366	0.663555	0.675465	0.675465
50nM+NA	-0.48976	-0.48976	-0.29627	-0.01965	0.469466	0.506616	0.506616
5nM+NA	-1.82722	-1.82722	-1.49128	-0.66227	0.044888	0.355884	0.355884
+NA	-1.70237	-1.70237	-1.07867	0.010763	0.264826	0.393177	0.393177

Oneway Anova

Summary of Fit

Rsquare	0.372368
Adj Rsquare	0.267763
Root Mean Square Error	0.762693
Mean of Response	-2.8e-10
Observations (or Sum Wgts)	36

Analysis of Variance

Source	DF	Sum of Squares	Mean Square	F Ratio	Prob > F
Label	5	10.353524	2.07070	3.5597	0.0121 *
Error	30	17.450997	0.58170		
C. Total	35	27.804521			

Means for Oneway Anova

Level	Number	Mean	Std Error	Lower 95%	Upper 95%
50nM-NA	6	0.56287	0.31137	-0.073	1.199
5nM-NA	6	0.81264	0.31137	0.177	1.449
-NA	6	-0.38094	0.31137	-1.017	0.255
50nM+NA	6	0.03381	0.31137	-0.602	0.670
5nM+NA	6	-0.70566	0.31137	-1.342	-0.070
+NA	6	-0.32273	0.31137	-0.959	0.313

Std Error uses a pooled estimate of error variance

Means Comparisons

Comparisons with a control using Dunnett's Method

Oneway Analysis of Imidazoleacetic acid By Label

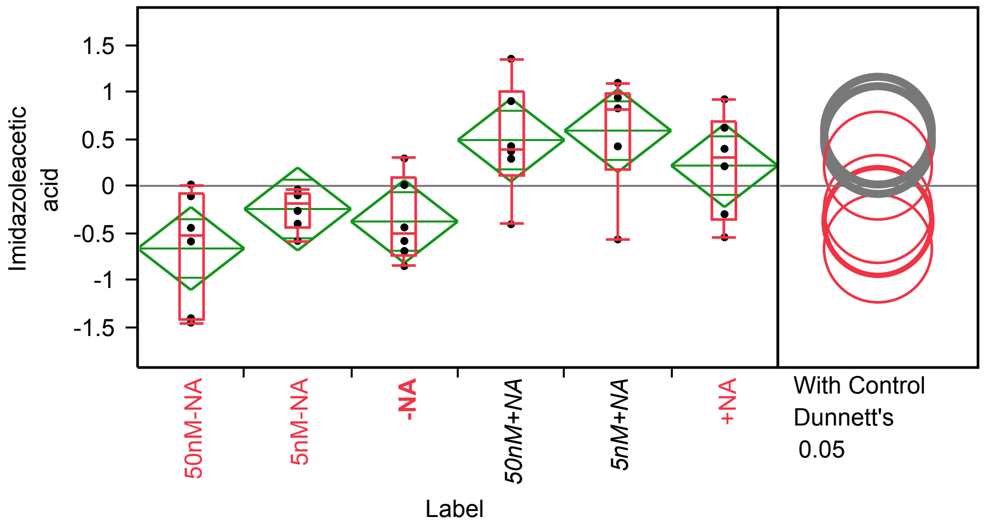

Quantiles

Level	Minimum	10%	25%	Median	75%	90%	Maximum
50nM-NA	-1.45674	-1.45674	-1.42161	-0.5193	-0.07912	0.014014	0.014014
5nM-NA	-0.58616	-0.58616	-0.44895	-0.18325	-0.07175	-0.03864	-0.03864
-NA	-0.85429	-0.85429	-0.73388	-0.51441	0.079695	0.291872	0.291872
50nM+NA	-0.40837	-0.40837	0.114189	0.395688	1.01571	1.353559	1.353559
5nM+NA	-0.57002	-0.57002	0.172999	0.820869	0.98005	1.097334	1.097334
+NA	-0.54773	-0.54773	-0.363	0.30193	0.694785	0.922346	0.922346

Oneway Anova

Summary of Fit

Rsquare	0.477764
Adj Rsquare	0.390724
Root Mean Square Error	0.529061
Mean of Response	-2.8e-11
Observations (or Sum Wgts)	36

Analysis of Variance

Source	DF	Sum of Squares	Mean Square	F Ratio	Prob > F
Label	5	7.682079	1.53642	5.4891	0.0011 *
Error	30	8.397162	0.27991		
C. Total	35	16.079241			

Means for Oneway Anova

Level	Number	Mean	Std Error	Lower 95%	Upper 95%
50nM-NA	6	-0.66690	0.21599	-1.108	-0.226
5nM-NA	6	-0.24622	0.21599	-0.687	0.195
-NA	6	-0.37933	0.21599	-0.820	0.062
50nM+NA	6	0.48801	0.21599	0.047	0.929
5nM+NA	6	0.58845	0.21599	0.147	1.030
+NA	6	0.21600	0.21599	-0.225	0.657

Std Error uses a pooled estimate of error variance

Means Comparisons

Comparisons with a control using Dunnett's Method

Oneway Analysis of IMP By Label

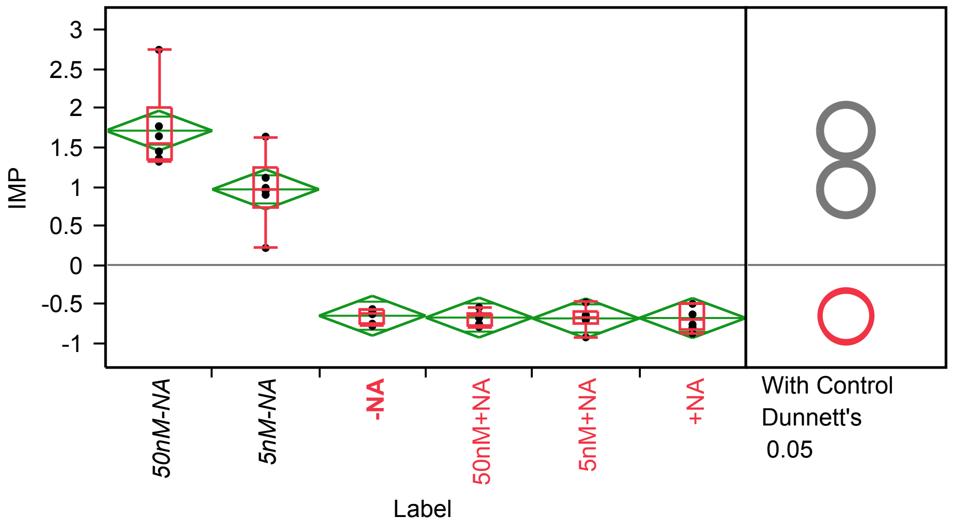

Quantiles

Level	Minimum	10%	25%	Median	75%	90%	Maximum
50nM-NA	1.319122	1.319122	1.349115	1.547533	2.016059	2.748199	2.748199
5nM-NA	0.217359	0.217359	0.726491	0.963936	1.245102	1.638748	1.638748
-NA	-0.77963	-0.77963	-0.75778	-0.61065	-0.57346	-0.5641	-0.5641
50nM+NA	-0.79305	-0.79305	-0.76437	-0.65512	-0.60978	-0.54225	-0.54225
5nM+NA	-0.92439	-0.92439	-0.75948	-0.67411	-0.60001	-0.47938	-0.47938
+NA	-0.8755	-0.8755	-0.81852	-0.69585	-0.5011	-0.49692	-0.49692

Oneway Anova

Summary of Fit

Rsquare	0.924426
Adj Rsquare	0.91183
Root Mean Square Error	0.304579
Mean of Response	5.556e-9
Observations (or Sum Wgts)	36

Analysis of Variance

Source	DF	Sum of Squares	Mean Square	F Ratio	Prob > F
Label	5	34.042445	6.80849	73.3924	<.0001 *
Error	30	2.783049	0.09277		
C. Total	35	36.825494			

Means for Oneway Anova

Level	Number	Mean	Std Error	Lower 95%	Upper 95%
50nM-NA	6	1.7156	0.12434	1.462	1.970
5nM-NA	6	0.9657	0.12434	0.712	1.220
-NA	6	-0.6487	0.12434	-0.903	-0.395
50nM+NA	6	-0.6721	0.12434	-0.926	-0.418
5nM+NA	6	-0.6828	0.12434	-0.937	-0.429
+NA	6	-0.6777	0.12434	-0.932	-0.424

Std Error uses a pooled estimate of error variance

Means Comparisons

Comparisons with a control using Dunnett's Method

Oneway Analysis of indole By Label

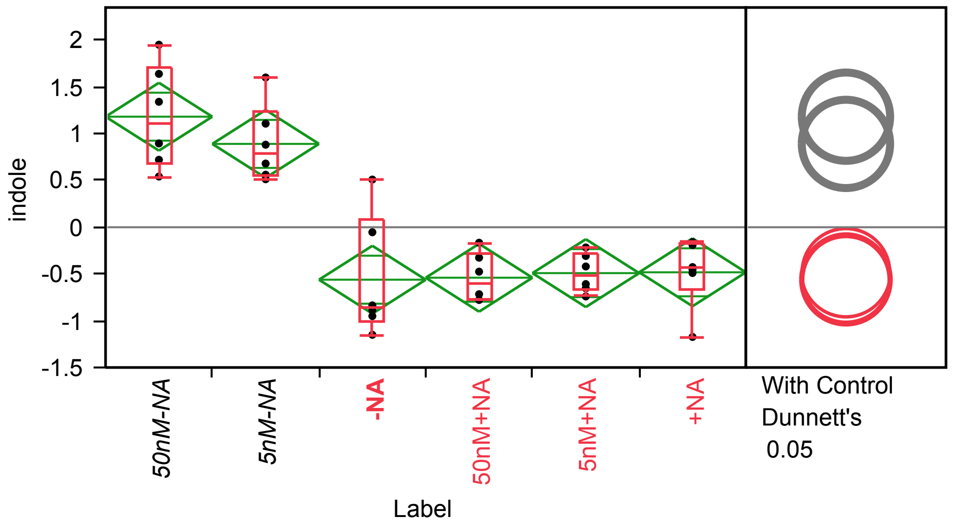

Quantiles

Level	Minimum	10%	25%	Median	75%	90%	Maximum
50nM-NA	0.542338	0.542338	0.676643	1.118914	1.717934	1.95176	1.95176
5nM-NA	0.513309	0.513309	0.549498	0.781787	1.231237	1.601443	1.601443
-NA	-1.15028	-1.15028	-1.00011	-0.86122	0.087187	0.509632	0.509632
50nM+NA	-0.77843	-0.77843	-0.77804	-0.59559	-0.28589	-0.16577	-0.16577
5nM+NA	-0.73998	-0.73998	-0.67483	-0.51375	-0.28455	-0.21645	-0.21645
+NA	-1.17314	-1.17314	-0.66034	-0.43906	-0.18279	-0.15956	-0.15956

Oneway Anova

Summary of Fit

Rsquare	0.775472
Adj Rsquare	0.73805
Root Mean Square Error	0.435239
Mean of Response	-2.8e-10
Observations (or Sum Wgts)	36

Analysis of Variance

Source	DF	Sum of Squares	Mean Square	F Ratio	Prob > F
Label	5	19.627760	3.92555	20.7227	<.0001 *
Error	30	5.682980	0.18943		
C. Total	35	25.310741			

Means for Oneway Anova

Level	Number	Mean	Std Error	Lower 95%	Upper 95%
50nM-NA	6	1.1822	0.17769	0.8193	1.545
5nM-NA	6	0.8913	0.17769	0.5284	1.254
-NA	6	-0.5611	0.17769	-0.9240	-0.198
50nM+NA	6	-0.5399	0.17769	-0.9028	-0.177
5nM+NA	6	-0.4907	0.17769	-0.8536	-0.128
+NA	6	-0.4818	0.17769	-0.8447	-0.119

Std Error uses a pooled estimate of error variance

Means Comparisons

Comparisons with a control using Dunnett's Method

Oneway Analysis of Indoleacrylic acid By Label

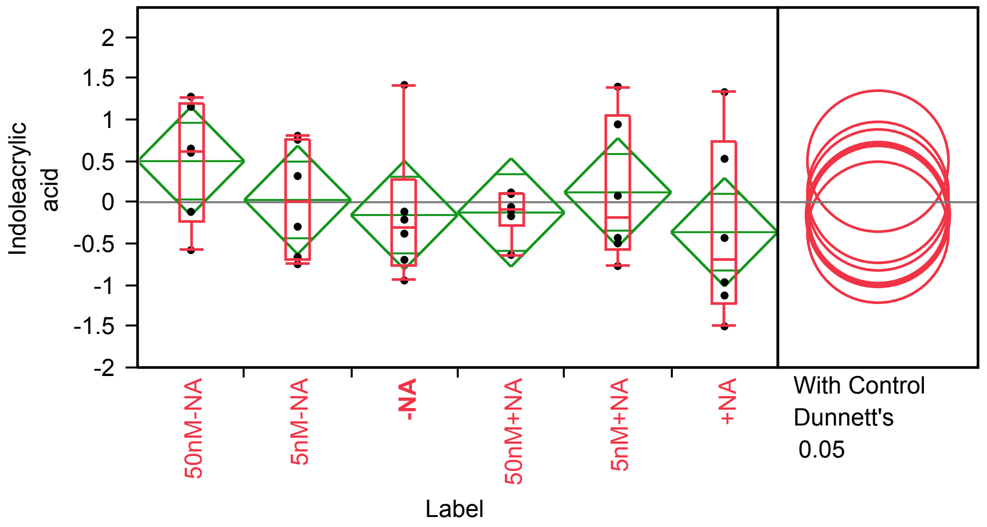

Quantiles

Level	Minimum	10%	25%	Median	75%	90%	Maximum
50nM-NA	-0.58008	-0.58008	-0.23229	0.623404	1.184442	1.276514	1.276514
5nM-NA	-0.75086	-0.75086	-0.68527	0.01	0.767273	0.80288	0.80288
-NA	-0.94702	-0.94702	-0.75992	-0.29633	0.268894	1.418125	1.418125
50nM+NA	-0.63739	-0.63739	-0.28621	-0.07988	0.103711	0.117284	0.117284
5nM+NA	-0.77274	-0.77274	-0.56642	-0.17682	1.055661	1.397441	1.397441
+NA	-1.50219	-1.50219	-1.22241	-0.70072	0.726347	1.331446	1.331446

Oneway Anova

Summary of Fit

Rsquare	0.122894
Adj Rsquare	-0.02329
Root Mean Square Error	0.78632
Mean of Response	2.78e-10
Observations (or Sum Wgts)	36

Analysis of Variance

Source	DF	Sum of Squares	Mean Square	F Ratio	Prob > F
Label	5	2.598955	0.519791	0.8407	0.5316
Error	30	18.548952	0.618298		
C. Total	35	21.147907			

Means for Oneway Anova

Level	Number	Mean	Std Error	Lower 95%	Upper 95%
50nM-NA	6	0.49677	0.32101	-0.159	1.1524
5nM-NA	6	0.02734	0.32101	-0.628	0.6829
-NA	6	-0.15555	0.32101	-0.811	0.5000
50nM+NA	6	-0.12497	0.32101	-0.781	0.5306
5nM+NA	6	0.11919	0.32101	-0.536	0.7748
+NA	6	-0.36278	0.32101	-1.018	0.2928

Std Error uses a pooled estimate of error variance

Means Comparisons

Comparisons with a control using Dunnett's Method

Oneway Analysis of inosine By Label

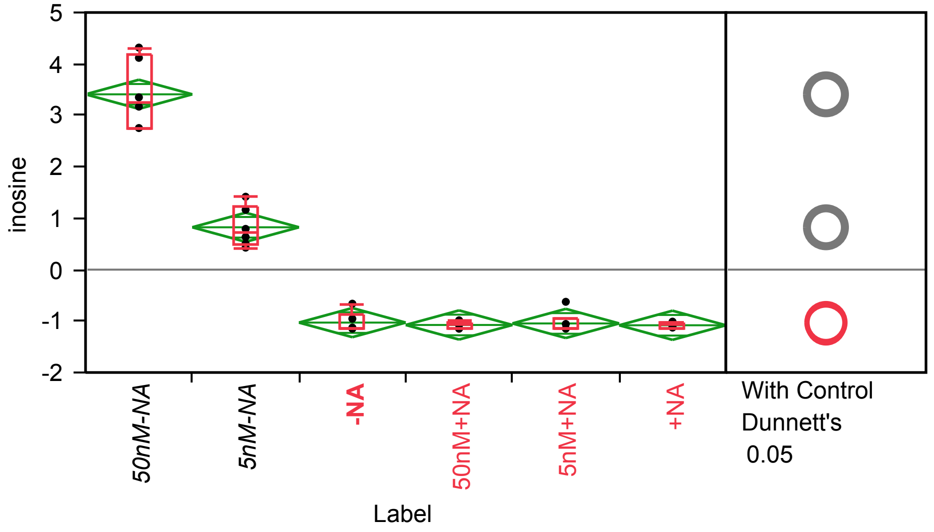

Quantiles

Level	Minimum	10%	25%	Median	75%	90%	Maximum
50nM-NA	2.746018	2.746018	2.751375	3.259972	4.168089	4.317371	4.317371
5nM-NA	0.42804	0.42804	0.485527	0.713905	1.227562	1.417477	1.417477
-NA	-1.14846	-1.14846	-1.14846	-1.13176	-0.88133	-0.66221	-0.66221
50nM+NA	-1.14847	-1.14847	-1.14847	-1.06416	-1.02942	-0.98535	-0.98535
5nM+NA	-1.14848	-1.14848	-1.14847	-1.14847	-0.94906	-0.62528	-0.62528
+NA	-1.14065	-1.14065	-1.13002	-1.08223	-1.03771	-1.01104	-1.01104

Oneway Anova

Summary of Fit

Rsquare	0.966742
Adj Rsquare	0.961199
Root Mean Square Error	0.339802
Mean of Response	-2.78e-9
Observations (or Sum Wgts)	36

Analysis of Variance

Source	DF	Sum of Squares	Mean Square	F Ratio	Prob > F
Label	5	100.69078	20.1382	174.4091	<.0001 *
Error	30	3.46395	0.1155		
C. Total	35	104.15474			

Means for Oneway Anova

Level	Number	Mean	Std Error	Lower 95%	Upper 95%
50nM-NA	6	3.4091	0.13872	3.126	3.692
5nM-NA	6	0.8237	0.13872	0.540	1.107
-NA	6	-1.0295	0.13872	-1.313	-0.746
50nM+NA	6	-1.0758	0.13872	-1.359	-0.792
5nM+NA	6	-1.0460	0.13872	-1.329	-0.763
+NA	6	-1.0815	0.13872	-1.365	-0.798

Std Error uses a pooled estimate of error variance

Means Comparisons

Comparisons with a control using Dunnett's Method

Oneway Analysis of isocitrate By Label

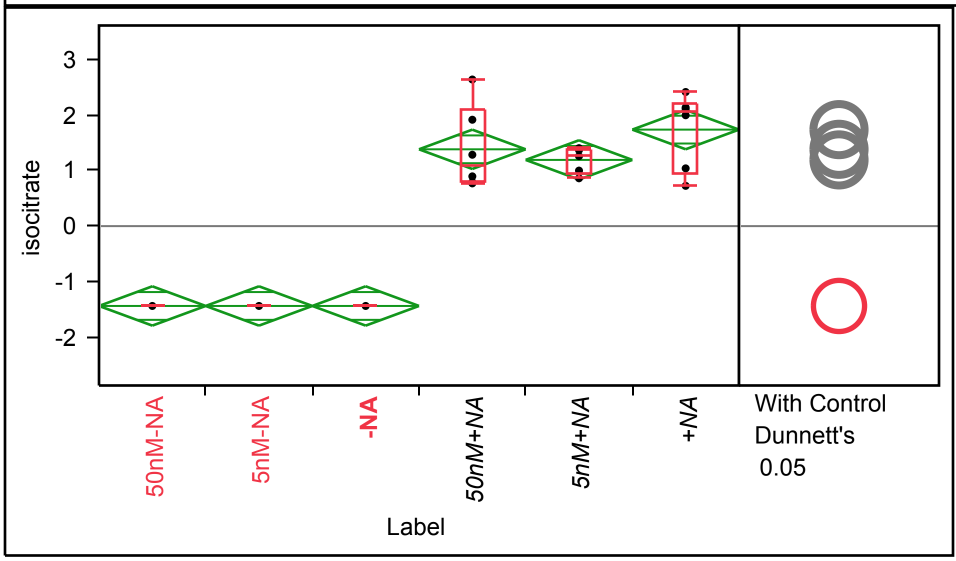

Quantiles

Level	Minimum	10%	25%	Median	75%	90%	Maximum
50nM-NA	-1.43268	-1.43268	-1.43268	-1.43267	-1.43267	-1.43267	-1.43267
5nM-NA	-1.43268	-1.43268	-1.43268	-1.43267	-1.43267	-1.43267	-1.43267
-NA	-1.43269	-1.43269	-1.43268	-1.43268	-1.43268	-1.43268	-1.43268
50nM+NA	0.766892	0.766892	0.784323	1.086777	2.089284	2.630685	2.630685
5nM+NA	0.858347	0.858347	0.961569	1.255465	1.373101	1.398595	1.398595
+NA	0.722842	0.722842	0.957997	2.04764	2.198102	2.406506	2.406506

Oneway Anova

Summary of Fit

Rsquare	0.933073
Adj Rsquare	0.921918
Root Mean Square Error	0.422911
Mean of Response	9.87e-17
Observations (or Sum Wgts)	36

Analysis of Variance

Source	DF	Sum of Squares	Mean Square	F Ratio	Prob > F
Label	5	74.805056	14.9610	83.6495	<.0001 *
Error	30	5.365606	0.1789		
C. Total	35	80.170662			

Means for Oneway Anova

Level	Number	Mean	Std Error	Lower 95%	Upper 95%
50nM-NA	6	-1.4327	0.17265	-1.785	-1.080
5nM-NA	6	-1.4327	0.17265	-1.785	-1.080
-NA	6	-1.4327	0.17265	-1.785	-1.080
50nM+NA	6	1.3783	0.17265	1.026	1.731
5nM+NA	6	1.1881	0.17265	0.835	1.541
+NA	6	1.7316	0.17265	1.379	2.084

Std Error uses a pooled estimate of error variance

Means Comparisons

Comparisons with a control using Dunnett's Method

Oneway Analysis of Isoleucine By Label

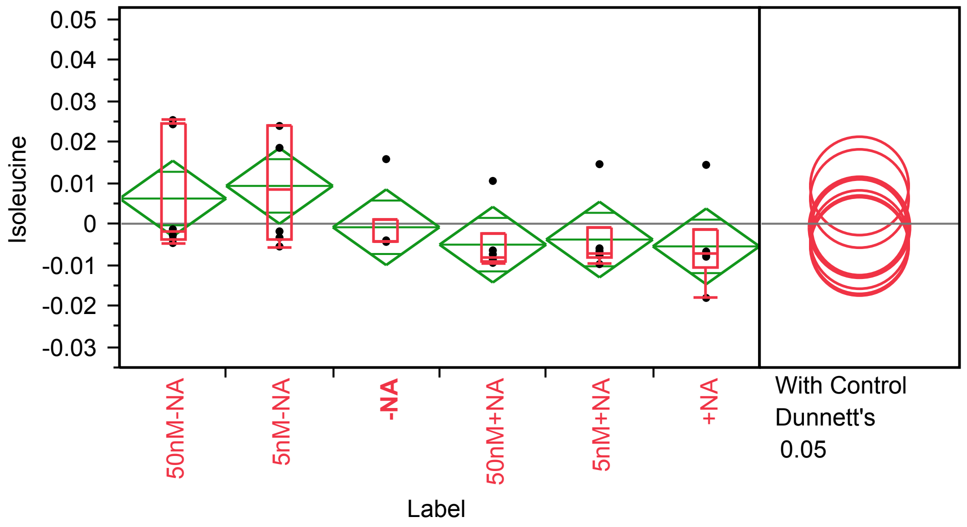

Quantiles

Level	Minimum	10%	25%	Median	75%	90%	Maximum
50nM-NA	-0.00472	-0.00472	-0.00403	-0.00208	0.024492	0.025271	0.025271
5nM-NA	-0.00556	-0.00556	-0.00393	0.008315	0.023802	0.023839	0.023839
-NA	-0.00443	-0.00443	-0.0043	-0.0041	0.000891	0.015748	0.015748
50nM+NA	-0.00954	-0.00954	-0.00911	-0.00802	-0.00227	0.010432	0.010432
5nM+NA	-0.00985	-0.00985	-0.00828	-0.00704	-0.00087	0.014518	0.014518
+NA	-0.01809	-0.01809	-0.01055	-0.0073	-0.0015	0.014348	0.014348

Oneway Anova

Summary of Fit

Rsquare	0.241838
Adj Rsquare	0.115478
Root Mean Square Error	0.011055
Mean of Response	2.78e-11
Observations (or Sum Wgts)	36

Analysis of Variance

Source	DF	Sum of Squares	Mean Square	F Ratio	Prob > F
Label	5	0.00116947	0.000234	1.9139	0.1215
Error	30	0.00366629	0.000122		
C. Total	35	0.00483576			

Means for Oneway Anova

Level	Number	Mean	Std Error	Lower 95%	Upper 95%
50nM-NA	6	0.00614	0.00451	-0.0031	0.01536
5nM-NA	6	0.00922	0.00451	1.33e-6	0.01844
-NA	6	-0.00086	0.00451	-0.0101	0.00835
50nM+NA	6	-0.00510	0.00451	-0.0143	0.00411
5nM+NA	6	-0.00386	0.00451	-0.0131	0.00535
+NA	6	-0.00553	0.00451	-0.0147	0.00369

Std Error uses a pooled estimate of error variance

Means Comparisons

Comparisons with a control using Dunnett's Method

Oneway Analysis of Ketohexanoic acid By Label

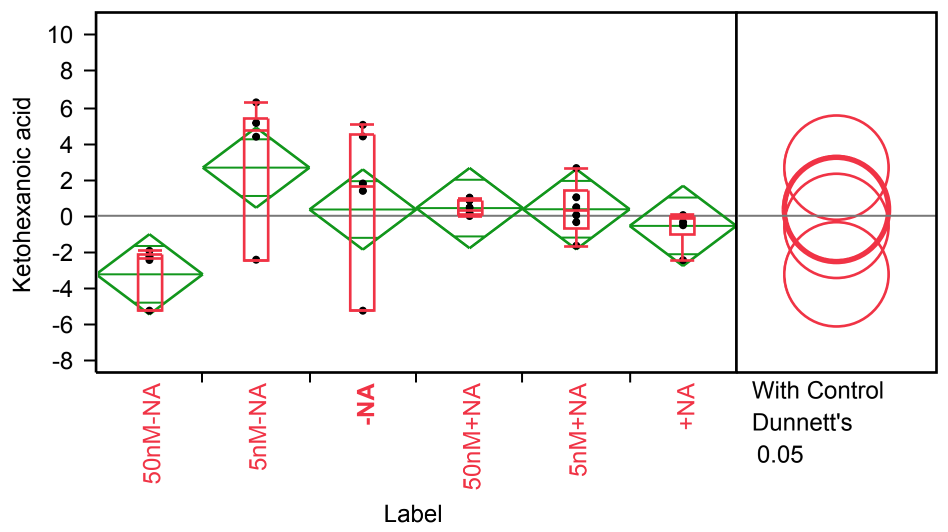

Quantiles

Level	Minimum	10%	25%	Median	75%	90%	Maximum
50nM-NA	-5.2599	-5.2599	-5.2599	-2.37732	-2.17182	-1.94481	-1.94481
5nM-NA	-2.45635	-2.45635	-2.44043	4.737859	5.428362	6.284452	6.284452
-NA	-5.2599	-5.2599	-5.2599	1.584947	4.559031	5.038674	5.038674
50nM+NA	-0.01285	-0.01285	0.080981	0.302995	0.886311	1.00483	1.00483
5nM+NA	-1.66027	-1.66027	-0.67889	0.256098	1.430646	2.643512	2.643512
+NA	-2.46033	-2.46033	-1.00763	-0.20238	0.002653	0.037529	0.037529

Oneway Anova

Summary of Fit

Rsquare	0.341388
Adj Rsquare	0.231619
Root Mean Square Error	2.664143
Mean of Response	-8.3e-11
Observations (or Sum Wgts)	36

Analysis of Variance

Source	DF	Sum of Squares	Mean Square	F Ratio	Prob > F
Label	5	110.37080	22.0742	3.1101	0.0223 *
Error	30	212.92978	7.0977		
C. Total	35	323.30058			

Means for Oneway Anova

Level	Number	Mean	Std Error	Lower 95%	Upper 95%
50nM-NA	6	-3.2445	1.0876	-5.466	-1.023
5nM-NA	6	2.6686	1.0876	0.447	4.890
-NA	6	0.3480	1.0876	-1.873	2.569
50nM+NA	6	0.4262	1.0876	-1.795	2.647
5nM+NA	6	0.3617	1.0876	-1.860	2.583
+NA	6	-0.5600	1.0876	-2.781	1.661

Std Error uses a pooled estimate of error variance

Means Comparisons

Comparisons with a control using Dunnett's Method

Oneway Analysis of Ketoleucine By Label

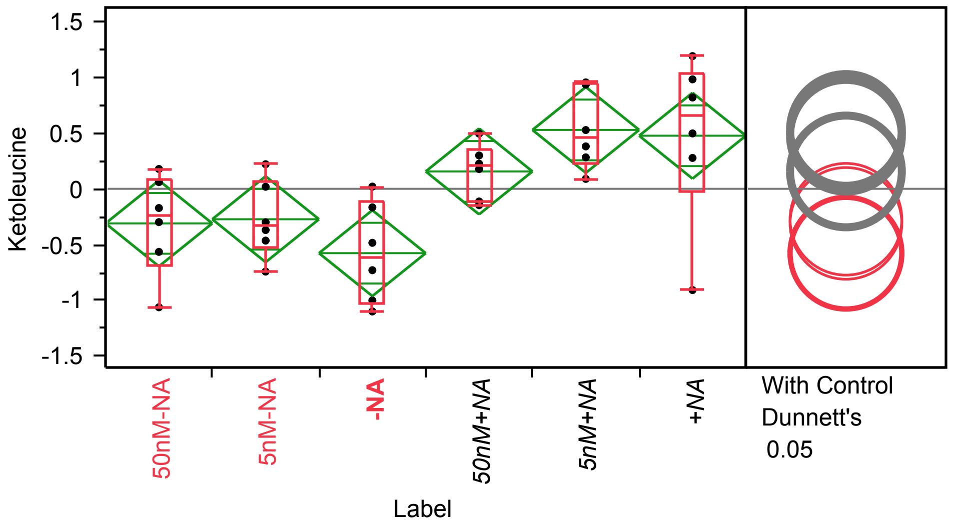

Quantiles

Level	Minimum	10%	25%	Median	75%	90%	Maximum
50nM-NA	-1.06152	-1.06152	-0.68921	-0.2354	0.089503	0.17784	0.17784
5nM-NA	-0.74157	-0.74157	-0.53354	-0.33476	0.069009	0.220404	0.220404
-NA	-1.09913	-1.09913	-1.02565	-0.60671	-0.1196	0.019781	0.019781
50nM+NA	-0.14654	-0.14654	-0.12175	0.200822	0.347821	0.494637	0.494637
5nM+NA	0.089046	0.089046	0.233636	0.453477	0.939213	0.953809	0.953809
+NA	-0.90738	-0.90738	-0.0193	0.657517	1.03316	1.189201	1.189201

Oneway Anova

Summary of Fit

Rsquare	0.492188
Adj Rsquare	0.407552
Root Mean Square Error	0.461483
Mean of Response	5.56e-10
Observations (or Sum Wgts)	36

Analysis of Variance

Source	DF	Sum of Squares	Mean Square	F Ratio	Prob > F
Label	5	6.192401	1.23848	5.8154	0.0007 *
Error	30	6.388984	0.21297		
C. Total	35	12.581385			

Means for Oneway Anova

Level	Number	Mean	Std Error	Lower 95%	Upper 95%
50nM-NA	6	-0.30992	0.18840	-0.6947	0.0748
5nM-NA	6	-0.27272	0.18840	-0.6575	0.1120
-NA	6	-0.57667	0.18840	-0.9614	-0.1919
50nM+NA	6	0.15586	0.18840	-0.2289	0.5406
5nM+NA	6	0.52766	0.18840	0.1429	0.9124
+NA	6	0.47579	0.18840	0.0910	0.8606

Std Error uses a pooled estimate of error variance

Means Comparisons

Comparisons with a control using Dunnett's Method

Oneway Analysis of Kynurenic acid By Label

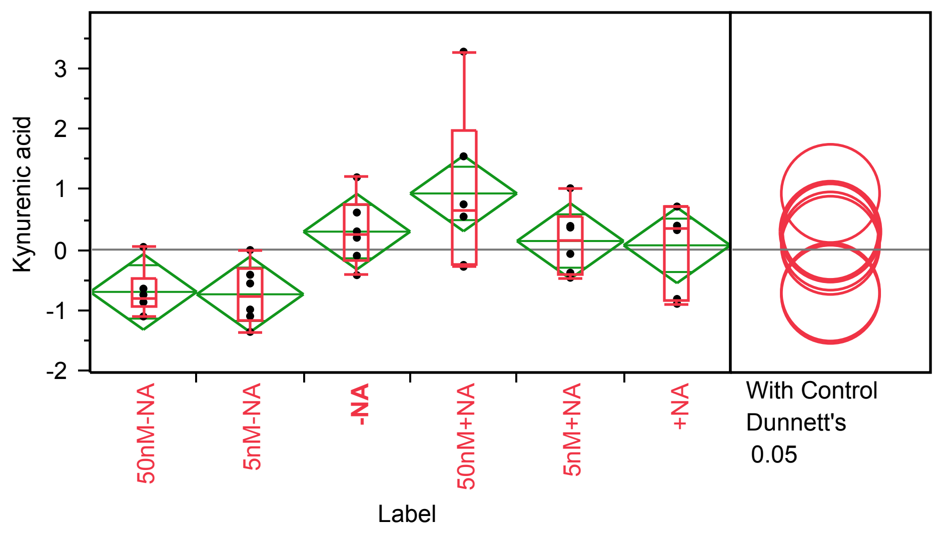

Quantiles

Level	Minimum	10%	25%	Median	75%	90%	Maximum
50nM-NA	-1.11174	-1.11174	-0.93613	-0.81373	-0.4761	0.044324	0.044324
5nM-NA	-1.36867	-1.36867	-1.17229	-0.77993	-0.31579	-0.00748	-0.00748
-NA	-0.42467	-0.42467	-0.18274	0.250328	0.763346	1.202976	1.202976
50nM+NA	-0.28531	-0.28531	-0.26291	0.651102	1.987167	3.292816	3.292816
5nM+NA	-0.46795	-0.46795	-0.40547	0.147821	0.550023	1.020516	1.020516
+NA	-0.9016	-0.9016	-0.84118	0.361097	0.712084	0.716731	0.716731

Oneway Anova

Summary of Fit

Rsquare	0.417851
Adj Rsquare	0.320826
Root Mean Square Error	0.753185
Mean of Response	5.56e-11
Observations (or Sum Wgts)	36

Analysis of Variance

Source	DF	Sum of Squares	Mean Square	F Ratio	Prob > F
Label	5	12.215491	2.44310	4.3066	0.0045 *
Error	30	17.018607	0.56729		
C. Total	35	29.234099			

Means for Oneway Anova

Level	Number	Mean	Std Error	Lower 95%	Upper 95%
50nM-NA	6	-0.70368	0.30749	-1.332	-0.076
5nM-NA	6	-0.74357	0.30749	-1.372	-0.116
-NA	6	0.29894	0.30749	-0.329	0.927
50nM+NA	6	0.93437	0.30749	0.306	1.562
5nM+NA	6	0.14279	0.30749	-0.485	0.771
+NA	6	0.07114	0.30749	-0.557	0.699

Std Error uses a pooled estimate of error variance

Means Comparisons

Comparisons with a control using Dunnett's Method

Oneway Analysis of Kynurenine By Label

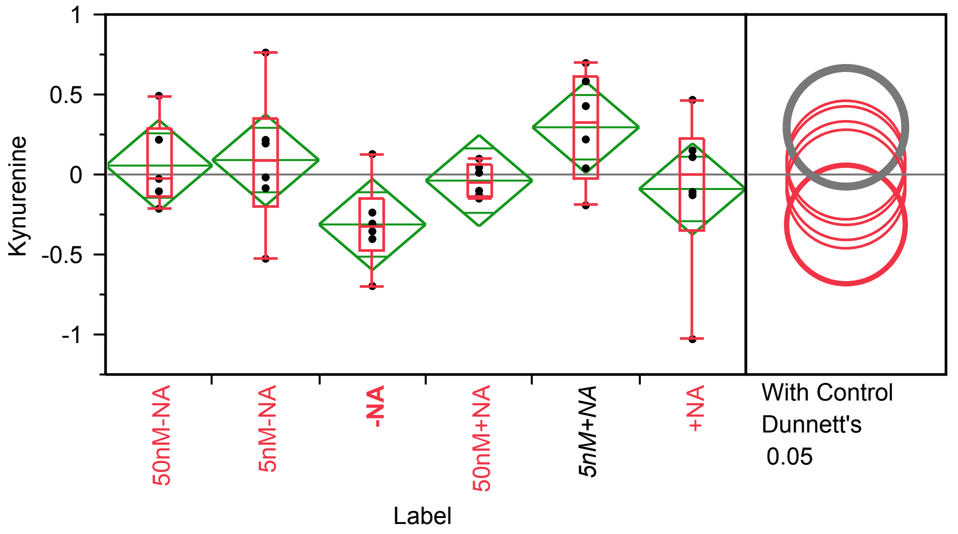

Quantiles

Level	Minimum	10%	25%	Median	75%	90%	Maximum
50nM-NA	-0.21414	-0.21414	-0.13248	-0.02758	0.28592	0.49172	0.49172
5nM-NA	-0.52661	-0.52661	-0.1959	0.087811	0.352725	0.761897	0.761897
-NA	-0.69731	-0.69731	-0.47687	-0.3312	-0.14679	0.127315	0.127315
50nM+NA	-0.15053	-0.15053	-0.13586	-0.04581	0.05845	0.098076	0.098076
5nM+NA	-0.19284	-0.19284	-0.01916	0.323218	0.610743	0.696295	0.696295
+NA	-1.02925	-1.02925	-0.35442	-0.00028	0.22901	0.466204	0.466204

Oneway Anova

Summary of Fit

Rsquare	0.260515
Adj Rsquare	0.137267
Root Mean Square Error	0.341622
Mean of Response	-5.6e-10
Observations (or Sum Wgts)	36

Analysis of Variance

Source	DF	Sum of Squares	Mean Square	F Ratio	Prob > F
Label	5	1.2334329	0.246687	2.1138	0.0911
Error	30	3.5011666	0.116706		
C. Total	35	4.7345995			

Means for Oneway Anova

Level	Number	Mean	Std Error	Lower 95%	Upper 95%
50nM-NA	6	0.05575	0.13947	-0.2291	0.3406
5nM-NA	6	0.09026	0.13947	-0.1946	0.3751
-NA	6	-0.31233	0.13947	-0.5972	-0.0275
50nM+NA	6	-0.03830	0.13947	-0.3231	0.2465
5nM+NA	6	0.29514	0.13947	0.0103	0.5800
+NA	6	-0.09052	0.13947	-0.3754	0.1943

Std Error uses a pooled estimate of error variance

Means Comparisons

Comparisons with a control using Dunnett's Method

Oneway Analysis of Lactic acid By Label

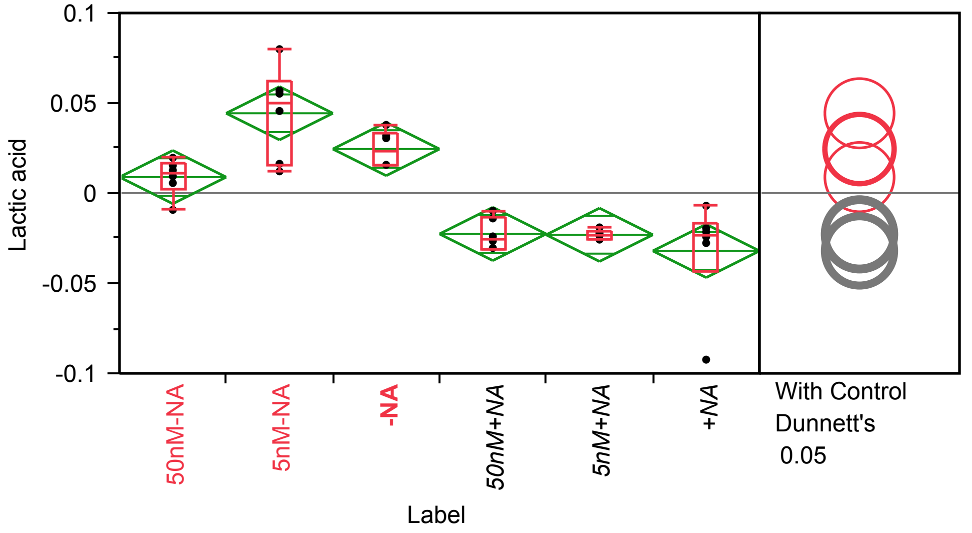

Quantiles

Level	Minimum	10%	25%	Median	75%	90%	Maximum
50nM-NA	-0.00925	-0.00925	0.001915	0.011054	0.016431	0.019633	0.019633
5nM-NA	0.012135	0.012135	0.015263	0.050424	0.062682	0.079917	0.079917
-NA	0.015368	0.015368	0.015515	0.023122	0.033402	0.037883	0.037883
50nM+NA	-0.03097	-0.03097	-0.03056	-0.02527	-0.01295	-0.00979	-0.00979
5nM+NA	-0.02583	-0.02583	-0.02577	-0.02303	-0.02119	-0.01901	-0.01901
+NA	-0.0924	-0.0924	-0.04385	-0.02285	-0.0164	-0.00701	-0.00701

Oneway Anova

Summary of Fit

Rsquare	0.750206
Adj Rsquare	0.708574
Root Mean Square Error	0.017728
Mean of Response	-8.3e-11
Observations (or Sum Wgts)	36

Analysis of Variance

Source	DF	Sum of Squares	Mean Square	F Ratio	Prob > F
Label	5	0.02831598	0.005663	18.0198	<.0001 *
Error	30	0.00942829	0.000314		
C. Total	35	0.03774427			

Means for Oneway Anova

Level	Number	Mean	Std Error	Lower 95%	Upper 95%
50nM-NA	6	0.00892	0.00724	-0.0059	0.0237
5nM-NA	6	0.04436	0.00724	0.0296	0.0591
-NA	6	0.02449	0.00724	0.0097	0.0393
50nM+NA	6	-0.02262	0.00724	-0.0374	-0.0078
5nM+NA	6	-0.02310	0.00724	-0.0379	-0.0083
+NA	6	-0.03205	0.00724	-0.0468	-0.0173

Std Error uses a pooled estimate of error variance

Means Comparisons

Comparisons with a control using Dunnett's Method

Oneway Analysis of leucine By Label

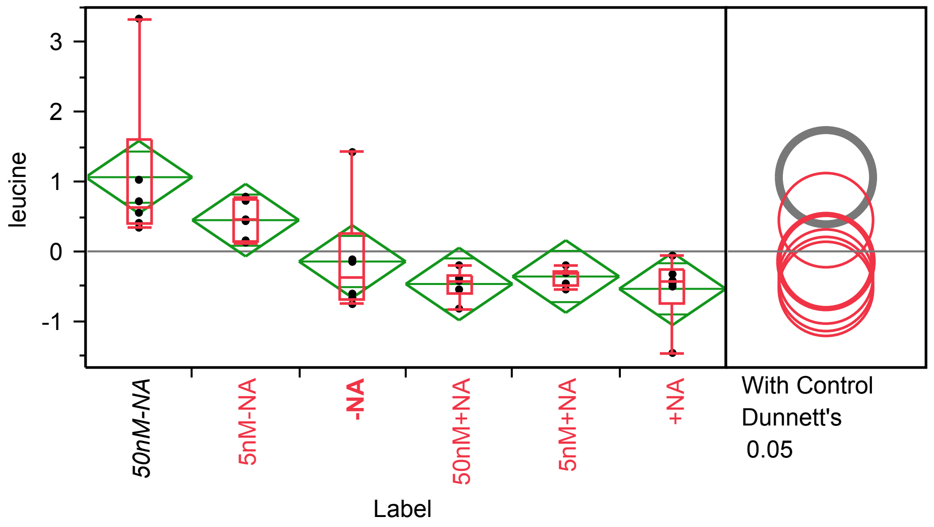

Quantiles

Level	Minimum	10%	25%	Median	75%	90%	Maximum
50nM-NA	0.340126	0.340126	0.391415	0.636457	1.60532	3.339438	3.339438
5nM-NA	0.126874	0.126874	0.149855	0.446841	0.740309	0.783878	0.783878
-NA	-0.75656	-0.75656	-0.69139	-0.37855	0.269448	1.42466	1.42466
50nM+NA	-0.82162	-0.82162	-0.61505	-0.42873	-0.33828	-0.19943	-0.19943
5nM+NA	-0.5464	-0.5464	-0.48321	-0.32309	-0.28323	-0.19965	-0.19965
+NA	-1.45739	-1.45739	-0.74277	-0.43845	-0.2604	-0.06135	-0.06135

Oneway Anova

Summary of Fit

Rsquare	0.506783
Adj Rsquare	0.42458
Root Mean Square Error	0.623035
Mean of Response	3.7e-17
Observations (or Sum Wgts)	36

Analysis of Variance

Source	DF	Sum of Squares	Mean Square	F Ratio	Prob > F
Label	5	11.965482	2.39310	6.1650	0.0005 *
Error	30	11.645173	0.38817		
C. Total	35	23.610655			

Means for Oneway Anova

Level	Number	Mean	Std Error	Lower 95%	Upper 95%
50nM-NA	6	1.0647	0.25435	0.545	1.584
5nM-NA	6	0.4480	0.25435	-0.072	0.967
-NA	6	-0.1457	0.25435	-0.665	0.374
50nM+NA	6	-0.4682	0.25435	-0.988	0.051
5nM+NA	6	-0.3609	0.25435	-0.880	0.159
+NA	6	-0.5378	0.25435	-1.057	-0.018

Std Error uses a pooled estimate of error variance

Means Comparisons

Comparisons with a control using Dunnett's Method

Oneway Analysis of Lysine By Label

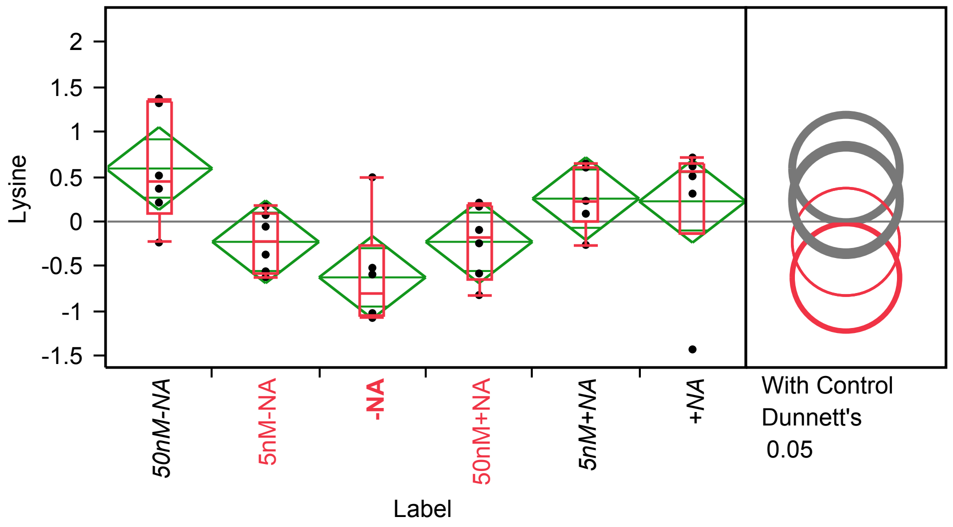

Quantiles

Level	Minimum	10%	25%	Median	75%	90%	Maximum
50nM-NA	-0.23263	-0.23263	0.100689	0.439218	1.331313	1.369411	1.369411
5nM-NA	-0.61703	-0.61703	-0.56977	-0.21198	0.097086	0.168956	0.168956
-NA	-1.07225	-1.07225	-1.03719	-0.80386	-0.26267	0.493238	0.493238
50nM+NA	-0.81839	-0.81839	-0.63771	-0.16719	0.178217	0.210599	0.210599
5nM+NA	-0.2598	-0.2598	0.000655	0.228295	0.612676	0.645479	0.645479
+NA	-1.42286	-1.42286	-0.12242	0.559837	0.653752	0.713035	0.713035

Oneway Anova

Summary of Fit

Rsquare	0.385506
Adj Rsquare	0.28309
Root Mean Square Error	0.551138
Mean of Response	5.56e-10
Observations (or Sum Wgts)	36

Analysis of Variance

Source	DF	Sum of Squares	Mean Square	F Ratio	Prob > F
Label	5	5.716830	1.14337	3.7641	0.0092 *
Error	30	9.112586	0.30375		
C. Total	35	14.829416			

Means for Oneway Anova

Level	Number	Mean	Std Error	Lower 95%	Upper 95%
50nM-NA	6	0.59094	0.22500	0.131	1.050
5nM-NA	6	-0.22549	0.22500	-0.685	0.234
-NA	6	-0.62114	0.22500	-1.081	-0.162
50nM+NA	6	-0.22537	0.22500	-0.685	0.234
5nM+NA	6	0.25525	0.22500	-0.204	0.715
+NA	6	0.22582	0.22500	-0.234	0.685

Std Error uses a pooled estimate of error variance

Means Comparisons

Comparisons with a control using Dunnett's Method

Oneway Analysis of LysoPC(20:4) By Label

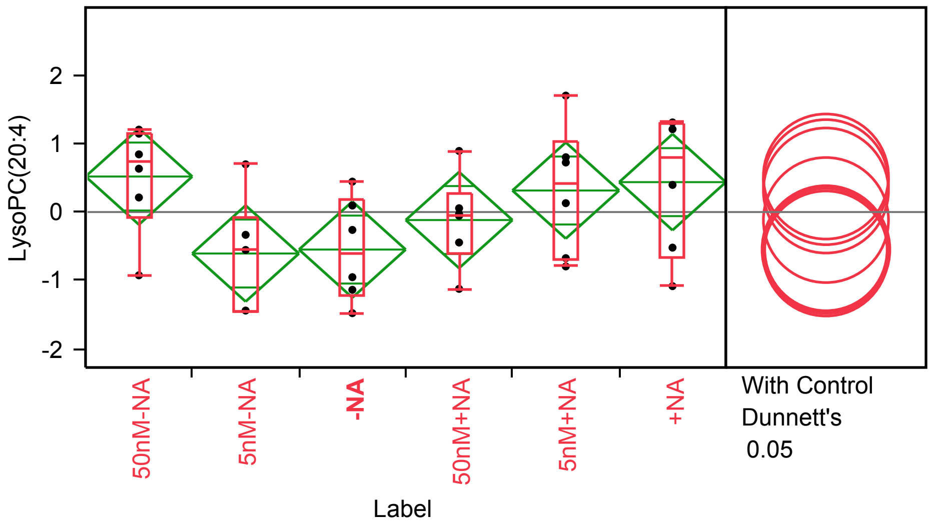

Quantiles

Level	Minimum	10%	25%	Median	75%	90%	Maximum
50nM-NA	-0.92482	-0.92482	-0.07138	0.738791	1.160998	1.204801	1.204801
5nM-NA	-1.45042	-1.45042	-1.44318	-0.55567	-0.07538	0.69827	0.69827
-NA	-1.47888	-1.47888	-1.22283	-0.60711	0.18244	0.444513	0.444513
50nM+NA	-1.12329	-1.12329	-0.61478	-0.04155	0.265077	0.893426	0.893426
5nM+NA	-0.79506	-0.79506	-0.70446	0.427443	1.027013	1.702661	1.702661
+NA	-1.08365	-1.08365	-0.66129	0.804391	1.309085	1.310922	1.310922

Oneway Anova

Summary of Fit

Rsquare	0.259055
Adj Rsquare	0.135564
Root Mean Square Error	0.843058
Mean of Response	2.78e-10
Observations (or Sum Wgts)	36

Analysis of Variance

Source	DF	Sum of Squares	Mean Square	F Ratio	Prob > F
Label	5	7.454895	1.49098	2.0978	0.0933
Error	30	21.322426	0.71075		
C. Total	35	28.777321			

Means for Oneway Anova

Level	Number	Mean	Std Error	Lower 95%	Upper 95%
50nM-NA	6	0.51951	0.34418	-0.183	1.2224
5nM-NA	6	-0.60625	0.34418	-1.309	0.0967
-NA	6	-0.54850	0.34418	-1.251	0.1544
50nM+NA	6	-0.11710	0.34418	-0.820	0.5858
5nM+NA	6	0.31500	0.34418	-0.388	1.0179
+NA	6	0.43734	0.34418	-0.266	1.1402

Std Error uses a pooled estimate of error variance

Means Comparisons

Comparisons with a control using Dunnett's Method

Oneway Analysis of Maleic acid By Label

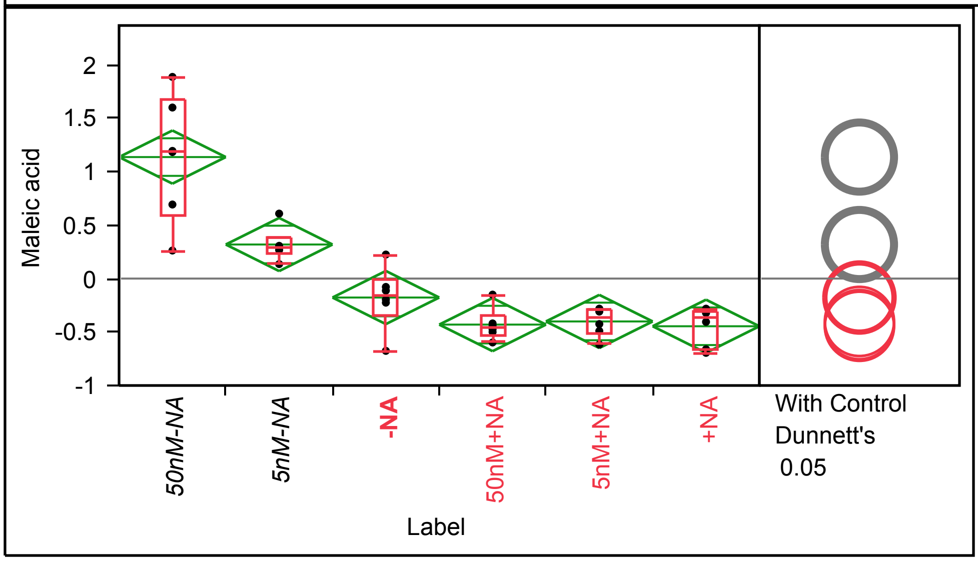

Quantiles

Level	Minimum	10%	25%	Median	75%	90%	Maximum
50nM-NA	0.262362	0.262362	0.584959	1.188512	1.671113	1.885878	1.885878
5nM-NA	0.13551	0.13551	0.23395	0.297634	0.381465	0.607473	0.607473
-NA	-0.67602	-0.67602	-0.33791	-0.15447	-0.00248	0.224532	0.224532
50nM+NA	-0.59756	-0.59756	-0.52457	-0.45759	-0.35222	-0.15027	-0.15027
5nM+NA	-0.61648	-0.61648	-0.52179	-0.36707	-0.28315	-0.28144	-0.28144
+NA	-0.69455	-0.69455	-0.66962	-0.36352	-0.30387	-0.28169	-0.28169

Oneway Anova

Summary of Fit

Rsquare	0.815885
Adj Rsquare	0.785199
Root Mean Square Error	0.298094
Mean of Response	3.7e-17
Observations (or Sum Wgts)	36

Analysis of Variance

Source	DF	Sum of Squares	Mean Square	F Ratio	Prob > F
Label	5	11.813197	2.36264	26.5883	<.0001 *
Error	30	2.665806	0.08886		
C. Total	35	14.479004			

Means for Oneway Anova

Level	Number	Mean	Std Error	Lower 95%	Upper 95%
50nM-NA	6	1.1362	0.12170	0.8877	1.385
5nM-NA	6	0.3185	0.12170	0.0700	0.567
-NA	6	-0.1773	0.12170	-0.4258	0.071
50nM+NA	6	-0.4305	0.12170	-0.6790	-0.182
5nM+NA	6	-0.4010	0.12170	-0.6495	-0.152
+NA	6	-0.4460	0.12170	-0.6945	-0.197

Std Error uses a pooled estimate of error variance

Means Comparisons

Comparisons with a control using Dunnett's Method

Oneway Analysis of Mannitol By Label

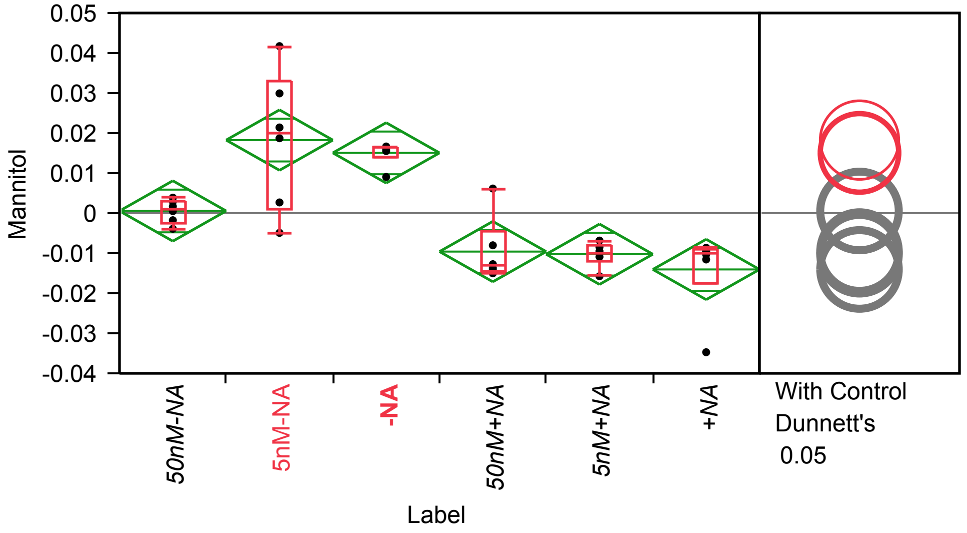

Quantiles

Level	Minimum	10%	25%	Median	75%	90%	Maximum
50nM-NA	-0.00389	-0.00389	-0.00229	0.001106	0.003144	0.00388	0.00388
5nM-NA	-0.00491	-0.00491	0.000787	0.020066	0.032877	0.041724	0.041724
-NA	0.009048	0.009048	0.013903	0.016283	0.016607	0.016654	0.016654
50nM+NA	-0.01501	-0.01501	-0.01441	-0.01322	-0.00448	0.006157	0.006157
5nM+NA	-0.01573	-0.01573	-0.01211	-0.00976	-0.00806	-0.00685	-0.00685
+NA	-0.03473	-0.03473	-0.01735	-0.01001	-0.00917	-0.00864	-0.00864

Oneway Anova

Summary of Fit

Rsquare	0.699991
Adj Rsquare	0.649989
Root Mean Square Error	0.009047
Mean of Response	1.11e-10
Observations (or Sum Wgts)	36

Analysis of Variance

Source	DF	Sum of Squares	Mean Square	F Ratio	Prob > F
Label	5	0.00572912	0.001146	13.9994	<.0001 *
Error	30	0.00245545	0.000082		
C. Total	35	0.00818456			

Means for Oneway Anova

Level	Number	Mean	Std Error	Lower 95%	Upper 95%
50nM-NA	6	0.00056	0.00369	-0.0070	0.0081
5nM-NA	6	0.01826	0.00369	0.0107	0.0258
-NA	6	0.01506	0.00369	0.0075	0.0226
50nM+NA	6	-0.00959	0.00369	-0.0171	-0.0020
5nM+NA	6	-0.01024	0.00369	-0.0178	-0.0027
+NA	6	-0.01405	0.00369	-0.0216	-0.0065

Std Error uses a pooled estimate of error variance

Means Comparisons

Comparisons with a control using Dunnett's Method

Oneway Analysis of methionine By Label

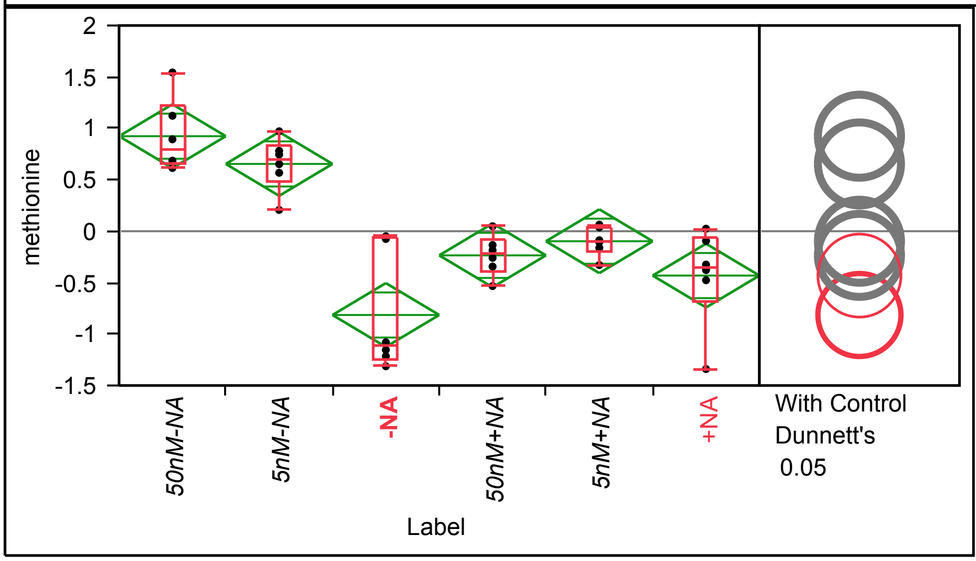

Quantiles

Level	Minimum	10%	25%	Median	75%	90%	Maximum
50nM-NA	0.614893	0.614893	0.665993	0.789622	1.227584	1.542737	1.542737
5nM-NA	0.206911	0.206911	0.477743	0.698681	0.828752	0.971141	0.971141
-NA	-1.31261	-1.31261	-1.24	-1.11701	-0.06859	-0.04944	-0.04944
50nM+NA	-0.53312	-0.53312	-0.39107	-0.22242	-0.08895	0.047893	0.047893
5nM+NA	-0.32881	-0.32881	-0.20347	-0.09714	0.04381	0.063647	0.063647
+NA	-1.33986	-1.33986	-0.69207	-0.3512	-0.06432	0.024174	0.024174

Oneway Anova

Summary of Fit

Rsquare	0.760327
Adj Rsquare	0.720381
Root Mean Square Error	0.372012
Mean of Response	5.56e-10
Observations (or Sum Wgts)	36

Analysis of Variance

Source	DF	Sum of Squares	Mean Square	F Ratio	Prob > F
Label	5	13.170918	2.63418	19.0341	<.0001 *
Error	30	4.151795	0.13839		
C. Total	35	17.322713			

Means for Oneway Anova

Level	Number	Mean	Std Error	Lower 95%	Upper 95%
50nM-NA	6	0.92374	0.15187	0.614	1.234
5nM-NA	6	0.65412	0.15187	0.344	0.964
-NA	6	-0.81447	0.15187	-1.125	-0.504
50nM+NA	6	-0.23473	0.15187	-0.545	0.075
5nM+NA	6	-0.09732	0.15187	-0.407	0.213
+NA	6	-0.43134	0.15187	-0.742	-0.121

Std Error uses a pooled estimate of error variance

Means Comparisons

Comparisons with a control using Dunnett's Method

Oneway Analysis of Methionine sulfoxide By Label

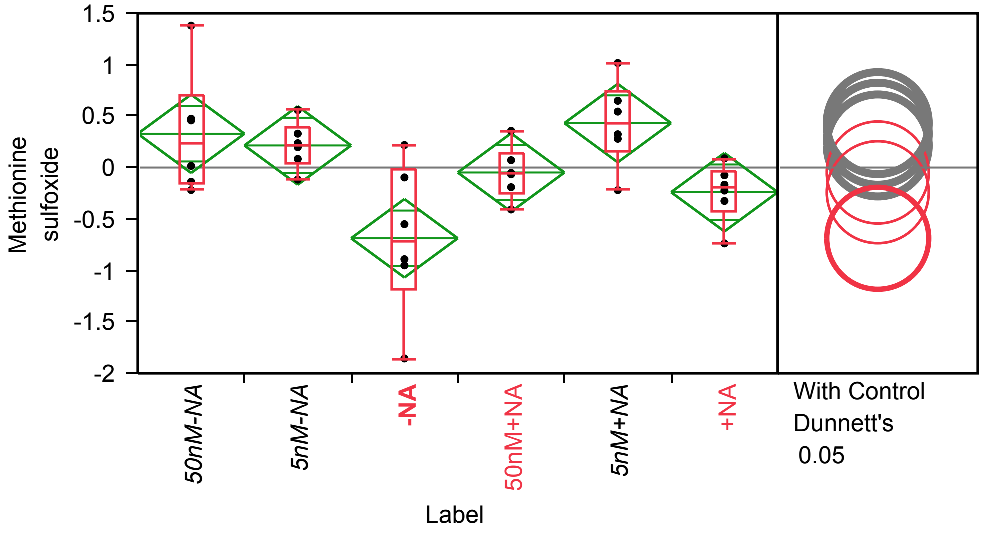

Quantiles

Level	Minimum	10%	25%	Median	75%	90%	Maximum
50nM-NA	-0.21868	-0.21868	-0.15826	0.237157	0.70089	1.379433	1.379433
5nM-NA	-0.11691	-0.11691	0.033535	0.21711	0.386642	0.558395	0.558395
-NA	-1.8561	-1.8561	-1.17727	-0.72075	-0.01744	0.218619	0.218619
50nM+NA	-0.4076	-0.4076	-0.24526	-0.05905	0.142791	0.356858	0.356858
5nM+NA	-0.21948	-0.21948	0.154844	0.433649	0.740775	1.016291	1.016291
+NA	-0.73738	-0.73738	-0.42804	-0.19536	-0.03377	0.089422	0.089422

Oneway Anova

Summary of Fit

Rsquare	0.455411
Adj Rsquare	0.364647
Root Mean Square Error	0.457077
Mean of Response	5.56e-10
Observations (or Sum Wgts)	36

Analysis of Variance

Source	DF	Sum of Squares	Mean Square	F Ratio	Prob > F
Label	5	5.241245	1.04825	5.0175	0.0019 *
Error	30	6.267571	0.20892		
C. Total	35	11.508816			

Means for Oneway Anova

Level	Number	Mean	Std Error	Lower 95%	Upper 95%
50nM-NA	6	0.32861	0.18660	-0.052	0.7097
5nM-NA	6	0.21480	0.18660	-0.166	0.5959
-NA	6	-0.68768	0.18660	-1.069	-0.3066
50nM+NA	6	-0.04809	0.18660	-0.429	0.3330
5nM+NA	6	0.43211	0.18660	0.051	0.8132
+NA	6	-0.23974	0.18660	-0.621	0.1413

Std Error uses a pooled estimate of error variance

Means Comparisons

Comparisons with a control using Dunnett's Method

Oneway Analysis of Methylcysteine By Label

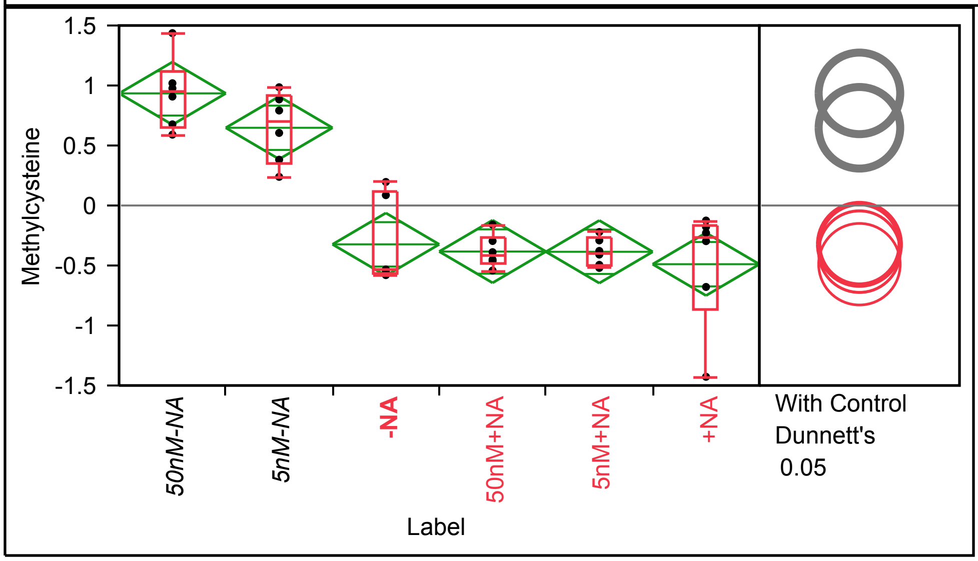

Quantiles

Level	Minimum	10%	25%	Median	75%	90%	Maximum
50nM-NA	0.590471	0.590471	0.654694	0.942237	1.12329	1.436392	1.436392
5nM-NA	0.238678	0.238678	0.345786	0.698135	0.909367	0.985395	0.985395
-NA	-0.58012	-0.58012	-0.57049	-0.5386	0.113707	0.196289	0.196289
50nM+NA	-0.54188	-0.54188	-0.48287	-0.41976	-0.26193	-0.15984	-0.15984
5nM+NA	-0.5201	-0.5201	-0.50098	-0.39378	-0.27356	-0.22098	-0.22098
+NA	-1.42746	-1.42746	-0.86589	-0.26254	-0.16536	-0.12548	-0.12548

Oneway Anova

Summary of Fit

Rsquare	0.797529
Adj Rsquare	0.763784
Root Mean Square Error	0.313238
Mean of Response	-2.8e-10
Observations (or Sum Wgts)	36

Analysis of Variance

Source	DF	Sum of Squares	Mean Square	F Ratio	Prob > F
Label	5	11.594558	2.31891	23.6339	<.0001 *
Error	30	2.943535	0.09812		
C. Total	35	14.538093			

Means for Oneway Anova

Level	Number	Mean	Std Error	Lower 95%	Upper 95%
50nM-NA	6	0.93439	0.12788	0.6732	1.196
5nM-NA	6	0.64764	0.12788	0.3865	0.909
-NA	6	-0.32369	0.12788	-0.5849	-0.063
50nM+NA	6	-0.38340	0.12788	-0.6446	-0.122
5nM+NA	6	-0.38572	0.12788	-0.6469	-0.125
+NA	6	-0.48923	0.12788	-0.7504	-0.228

Std Error uses a pooled estimate of error variance

Means Comparisons

Comparisons with a control using Dunnett's Method

Oneway Analysis of Methylmalonic acid By Label

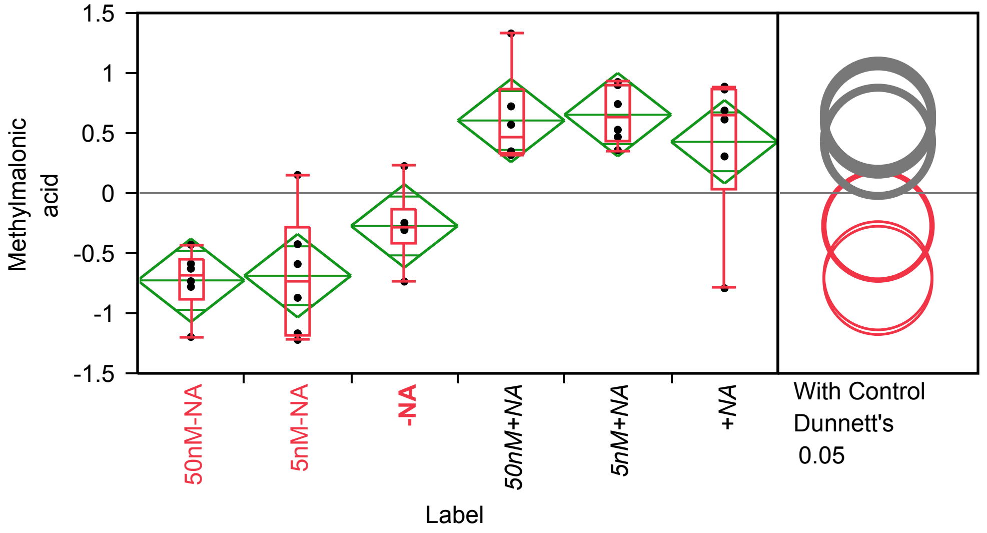

Quantiles

Level	Minimum	10%	25%	Median	75%	90%	Maximum
50nM-NA	-1.19704	-1.19704	-0.88408	-0.68085	-0.5482	-0.42973	-0.42973
5nM-NA	-1.22134	-1.22134	-1.18074	-0.73048	-0.28108	0.151133	0.151133
-NA	-0.7356	-0.7356	-0.41417	-0.28646	-0.12917	0.225243	0.225243
50nM+NA	0.316801	0.316801	0.336665	0.459194	0.874343	1.329626	1.329626
5nM+NA	0.357701	0.357701	0.440306	0.634847	0.905958	0.925406	0.925406
+NA	-0.79131	-0.79131	0.031685	0.650869	0.869222	0.886267	0.886267

Oneway Anova

Summary of Fit

Rsquare	0.703758
Adj Rsquare	0.654384
Root Mean Square Error	0.415419
Mean of Response	5.556e-9
Observations (or Sum Wgts)	36

Analysis of Variance

Source	DF	Sum of Squares	Mean Square	F Ratio	Prob > F
Label	5	12.299021	2.45980	14.2537	<.0001 *
Error	30	5.177187	0.17257		
C. Total	35	17.476208			

Means for Oneway Anova

Level	Number	Mean	Std Error	Lower 95%	Upper 95%
50nM-NA	6	-0.72599	0.16959	-1.072	-0.3796
5nM-NA	6	-0.68725	0.16959	-1.034	-0.3409
-NA	6	-0.27294	0.16959	-0.619	0.0734
50nM+NA	6	0.60511	0.16959	0.259	0.9515
5nM+NA	6	0.65335	0.16959	0.307	0.9997
+NA	6	0.42771	0.16959	0.081	0.7741

Std Error uses a pooled estimate of error variance

Means Comparisons

Comparisons with a control using Dunnett's Method

Oneway Analysis of Myo-Inositol By Label

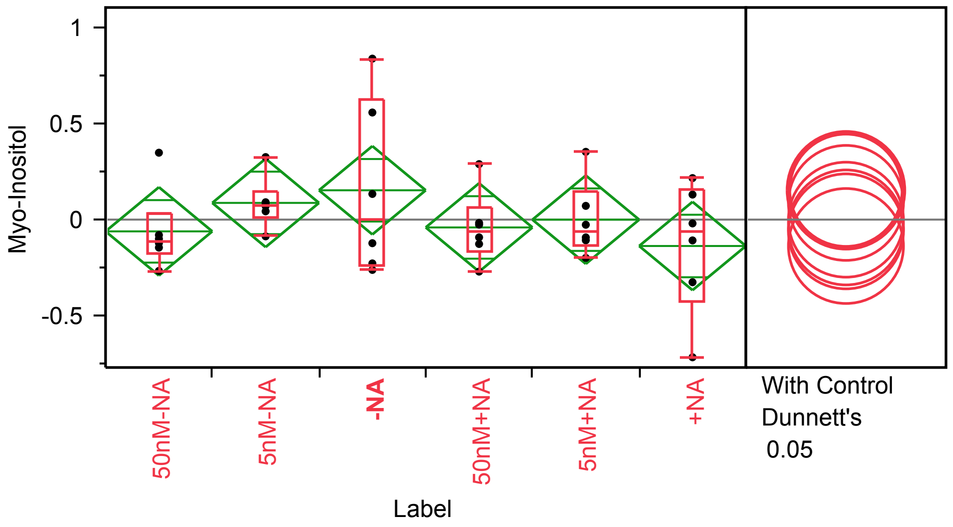

Quantiles

Level	Minimum	10%	25%	Median	75%	90%	Maximum
50nM-NA	-0.2675	-0.2675	-0.17627	-0.11128	0.026825	0.348618	0.348618
5nM-NA	-0.08716	-0.08716	0.01069	0.07505	0.149364	0.325859	0.325859
-NA	-0.26216	-0.26216	-0.23683	0.005367	0.628396	0.838897	0.838897
50nM+NA	-0.27092	-0.27092	-0.16296	-0.05946	0.059446	0.289118	0.289118
5nM+NA	-0.19953	-0.19953	-0.13068	-0.0598	0.142017	0.353205	0.353205
+NA	-0.71751	-0.71751	-0.42412	-0.06416	0.151914	0.216336	0.216336

Oneway Anova

Summary of Fit

Rsquare	0.126616
Adj Rsquare	-0.01895
Root Mean Square Error	0.276224
Mean of Response	5.56e-10
Observations (or Sum Wgts)	36

Analysis of Variance

Source	DF	Sum of Squares	Mean Square	F Ratio	Prob > F
Label	5	0.3318384	0.066368	0.8698	0.5128
Error	30	2.2889879	0.076300		
C. Total	35	2.6208263			

Means for Oneway Anova

Level	Number	Mean	Std Error	Lower 95%	Upper 95%
50nM-NA	6	-0.06129	0.11277	-0.2916	0.16901
5nM-NA	6	0.08711	0.11277	-0.1432	0.31741
-NA	6	0.15289	0.11277	-0.0774	0.38319
50nM+NA	6	-0.04080	0.11277	-0.2711	0.18950
5nM+NA	6	-0.00034	0.11277	-0.2306	0.22996
+NA	6	-0.13756	0.11277	-0.3679	0.09274

Std Error uses a pooled estimate of error variance

Means Comparisons

Comparisons with a control using Dunnett's Method

Oneway Analysis of N6-Acetyl-L-lysine By Label

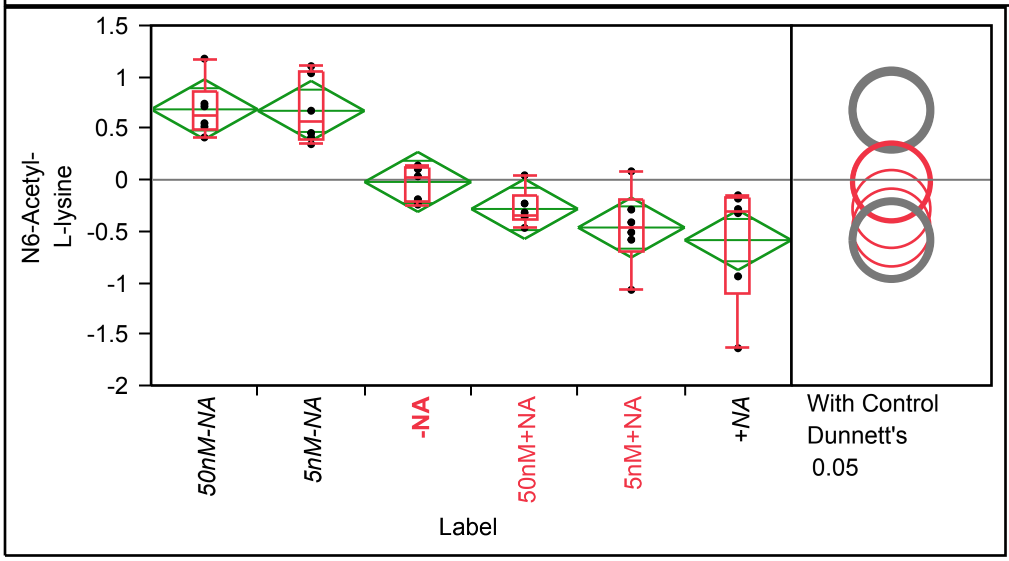

Quantiles

Level	Minimum	10%	25%	Median	75%	90%	Maximum
50nM-NA	0.411463	0.411463	0.490235	0.631556	0.849076	1.177555	1.177555
5nM-NA	0.346377	0.346377	0.394302	0.562975	1.053342	1.104786	1.104786
-NA	-0.24133	-0.24133	-0.20232	0.030891	0.112721	0.138461	0.138461
50nM+NA	-0.46791	-0.46791	-0.39178	-0.34014	-0.16221	0.043378	0.043378
5nM+NA	-1.07004	-1.07004	-0.70451	-0.46232	-0.19667	0.08237	0.08237
+NA	-1.63758	-1.63758	-1.11363	-0.30451	-0.17471	-0.15055	-0.15055

Oneway Anova

Summary of Fit

Rsquare	0.719417
Adj Rsquare	0.672654
Root Mean Square Error	0.348697
Mean of Response	2.78e-10
Observations (or Sum Wgts)	36

Analysis of Variance

Source	DF	Sum of Squares	Mean Square	F Ratio	Prob > F
Label	5	9.352703	1.87054	15.3841	<.0001 *
Error	30	3.647682	0.12159		
C. Total	35	13.000385			

Means for Oneway Anova

Level	Number	Mean	Std Error	Lower 95%	Upper 95%
50nM-NA	6	0.68470	0.14235	0.3940	0.9754
5nM-NA	6	0.67060	0.14235	0.3799	0.9613
-NA	6	-0.02104	0.14235	-0.3118	0.2697
50nM+NA	6	-0.28366	0.14235	-0.5744	0.0071
5nM+NA	6	-0.46411	0.14235	-0.7548	-0.1734
+NA	6	-0.58648	0.14235	-0.8772	-0.2958

Std Error uses a pooled estimate of error variance

Means Comparisons

Comparisons with a control using Dunnett's Method

Oneway Analysis of N-Acetylaspartylglutamic acid By Label

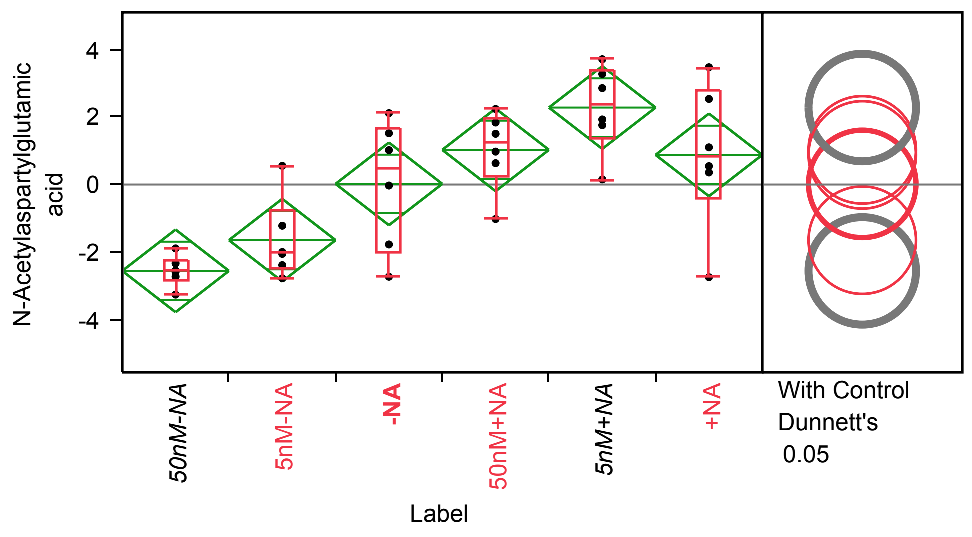

Quantiles

Level	Minimum	10%	25%	Median	75%	90%	Maximum
50nM-NA	-3.2649	-3.2649	-2.86426	-2.57019	-2.22753	-1.89513	-1.89513
5nM-NA	-2.78146	-2.78146	-2.48832	-2.02976	-0.77834	0.553562	0.553562
-NA	-2.73496	-2.73496	-2.0169	0.495194	1.670173	2.11761	2.11761
50nM+NA	-1.02296	-1.02296	0.219803	1.240819	1.940557	2.240481	2.240481
5nM+NA	0.152649	0.152649	1.359408	2.395811	3.388718	3.717227	3.717227
+NA	-2.7483	-2.7483	-0.41804	0.824347	2.773932	3.479115	3.479115

Oneway Anova

Summary of Fit

Rsquare	0.601958
Adj Rsquare	0.535617
Root Mean Square Error	1.469679
Mean of Response	8.33e-10
Observations (or Sum Wgts)	36

Analysis of Variance

Source	DF	Sum of Squares	Mean Square	F Ratio	Prob > F
Label	5	97.99478	19.5990	9.0738	<.0001 *
Error	30	64.79871	2.1600		
C. Total	35	162.79349			

Means for Oneway Anova

Level	Number	Mean	Std Error	Lower 95%	Upper 95%
50nM-NA	6	-2.5616	0.59999	-3.787	-1.336
5nM-NA	6	-1.6501	0.59999	-2.875	-0.425
-NA	6	0.0194	0.59999	-1.206	1.245
50nM+NA	6	1.0290	0.59999	-0.196	2.254
5nM+NA	6	2.2837	0.59999	1.058	3.509
+NA	6	0.8795	0.59999	-0.346	2.105

Std Error uses a pooled estimate of error variance

Means Comparisons

Comparisons with a control using Dunnett's Method

Oneway Analysis of N-acetyl-glucosamine-1-phosphate By Label

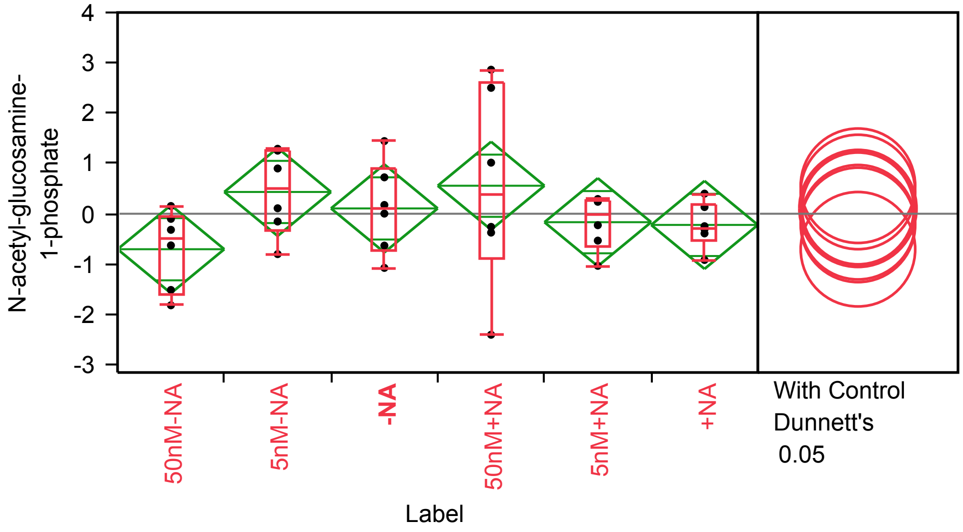

Quantiles

Level	Minimum	10%	25%	Median	75%	90%	Maximum
50nM-NA	-1.81936	-1.81936	-1.59109	-0.47434	-0.03783	0.153266	0.153266
5nM-NA	-0.80255	-0.80255	-0.31655	0.504439	1.263252	1.290829	1.290829
-NA	-1.07632	-1.07632	-0.74407	0.088386	0.903755	1.444037	1.444037
50nM+NA	-2.40501	-2.40501	-0.88235	0.377117	2.592505	2.862516	2.862516
5nM+NA	-1.0349	-1.0349	-0.65945	0.004717	0.268109	0.292596	0.292596
+NA	-0.91877	-0.91877	-0.52301	-0.27738	0.199281	0.399752	0.399752

Oneway Anova

Summary of Fit

Rsquare	0.164549
Adj Rsquare	0.025307
Root Mean Square Error	1.048721
Mean of Response	8.33e-10
Observations (or Sum Wgts)	36

Analysis of Variance

Source	DF	Sum of Squares	Mean Square	F Ratio	Prob > F
Label	5	6.498546	1.29971	1.1818	0.3413
Error	30	32.994473	1.09982		
C. Total	35	39.493018			

Means for Oneway Anova

Level	Number	Mean	Std Error	Lower 95%	Upper 95%
50nM-NA	6	-0.70522	0.42814	-1.580	0.1692
5nM-NA	6	0.43278	0.42814	-0.442	1.3072
-NA	6	0.10581	0.42814	-0.769	0.9802
50nM+NA	6	0.55657	0.42814	-0.318	1.4309
5nM+NA	6	-0.16787	0.42814	-1.042	0.7065
+NA	6	-0.22207	0.42814	-1.096	0.6523

Std Error uses a pooled estimate of error variance

Means Comparisons

Comparisons with a control using Dunnett's Method

Oneway Analysis of N-acetyl-glutamine By Label

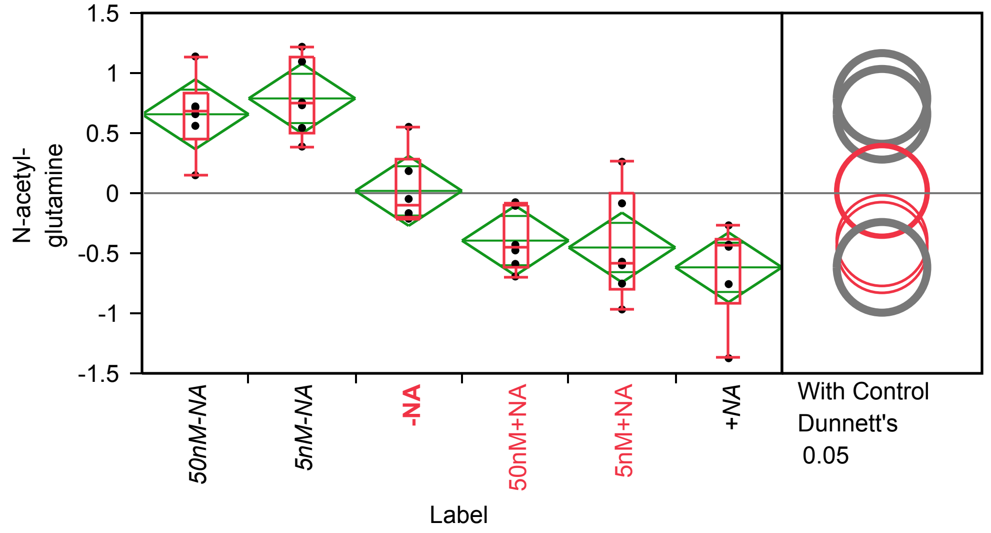

Quantiles

Level	Minimum	10%	25%	Median	75%	90%	Maximum
50nM-NA	0.149349	0.149349	0.457955	0.68565	0.826099	1.138019	1.138019
5nM-NA	0.388124	0.388124	0.504413	0.743647	1.126171	1.218504	1.218504
-NA	-0.21246	-0.21246	-0.19997	-0.10729	0.276341	0.551977	0.551977
50nM+NA	-0.69343	-0.69343	-0.61519	-0.45309	-0.09757	-0.07567	-0.07567
5nM+NA	-0.9678	-0.9678	-0.80665	-0.58493	0.001901	0.261517	0.261517
+NA	-1.37402	-1.37402	-0.91143	-0.43804	-0.38905	-0.26803	-0.26803

Oneway Anova

Summary of Fit

Rsquare	0.747965
Adj Rsquare	0.705959
Root Mean Square Error	0.347883
Mean of Response	-2.8e-10
Observations (or Sum Wgts)	36

Analysis of Variance

Source	DF	Sum of Squares	Mean Square	F Ratio	Prob > F
Label	5	10.774739	2.15495	17.8062	<.0001 *
Error	30	3.630671	0.12102		
C. Total	35	14.405410			

Means for Oneway Anova

Level	Number	Mean	Std Error	Lower 95%	Upper 95%
50nM-NA	6	0.65694	0.14202	0.3669	0.947
5nM-NA	6	0.78875	0.14202	0.4987	1.079
-NA	6	0.01893	0.14202	-0.2711	0.309
50nM+NA	6	-0.39488	0.14202	-0.6849	-0.105
5nM+NA	6	-0.45228	0.14202	-0.7423	-0.162
+NA	6	-0.61746	0.14202	-0.9075	-0.327

Std Error uses a pooled estimate of error variance

Means Comparisons

Comparisons with a control using Dunnett's Method

Oneway Analysis of N-Acetyl-L-alanine By Label

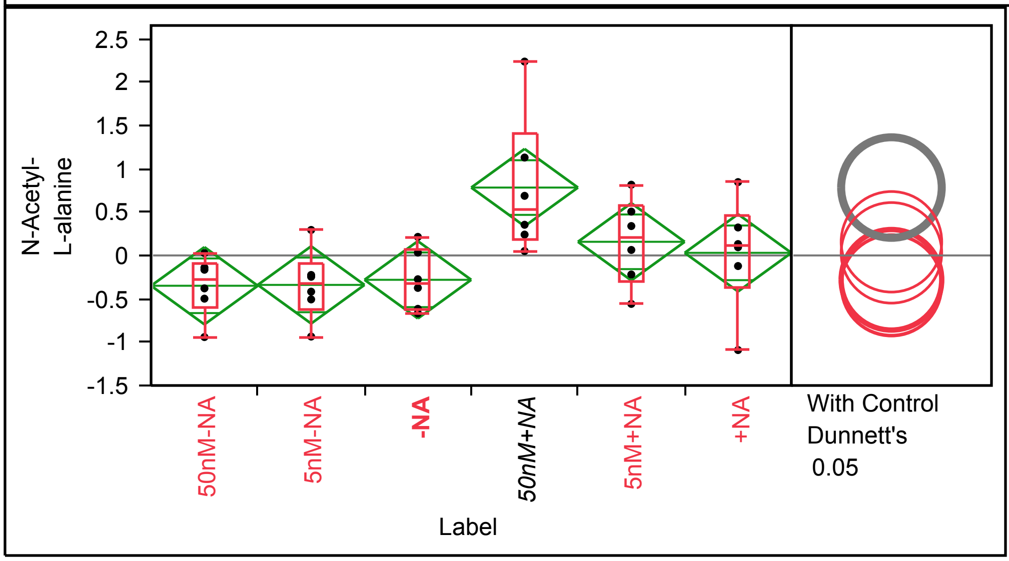

Quantiles

Level	Minimum	10%	25%	Median	75%	90%	Maximum
50nM-NA	-0.94145	-0.94145	-0.60917	-0.27232	-0.09826	0.026266	0.026266
5nM-NA	-0.93571	-0.93571	-0.61416	-0.33222	-0.09556	0.293931	0.293931
-NA	-0.66779	-0.66779	-0.62895	-0.32437	0.078055	0.214081	0.214081
50nM+NA	0.050806	0.050806	0.193007	0.520616	1.405967	2.235752	2.235752
5nM+NA	-0.55872	-0.55872	-0.30445	0.201073	0.582785	0.81535	0.81535
+NA	-1.09117	-1.09117	-0.3645	0.112801	0.45333	0.845724	0.845724

Oneway Anova

Summary of Fit

Rsquare	0.40077
Adj Rsquare	0.300898
Root Mean Square Error	0.534483
Mean of Response	-2.8e-10
Observations (or Sum Wgts)	36

Analysis of Variance

Source	DF	Sum of Squares	Mean Square	F Ratio	Prob > F
Label	5	5.731776	1.14636	4.0128	0.0066 *
Error	30	8.570150	0.28567		
C. Total	35	14.301925			

Means for Oneway Anova

Level	Number	Mean	Std Error	Lower 95%	Upper 95%
50nM-NA	6	-0.34967	0.21820	-0.7953	0.0960
5nM-NA	6	-0.33977	0.21820	-0.7854	0.1059
-NA	6	-0.28096	0.21820	-0.7266	0.1647
50nM+NA	6	0.78293	0.21820	0.3373	1.2286
5nM+NA	6	0.15739	0.21820	-0.2882	0.6030
+NA	6	0.03007	0.21820	-0.4156	0.4757

Std Error uses a pooled estimate of error variance

Means Comparisons

Comparisons with a control using Dunnett's Method

Oneway Analysis of N-acetyl-L-ornithine By Label

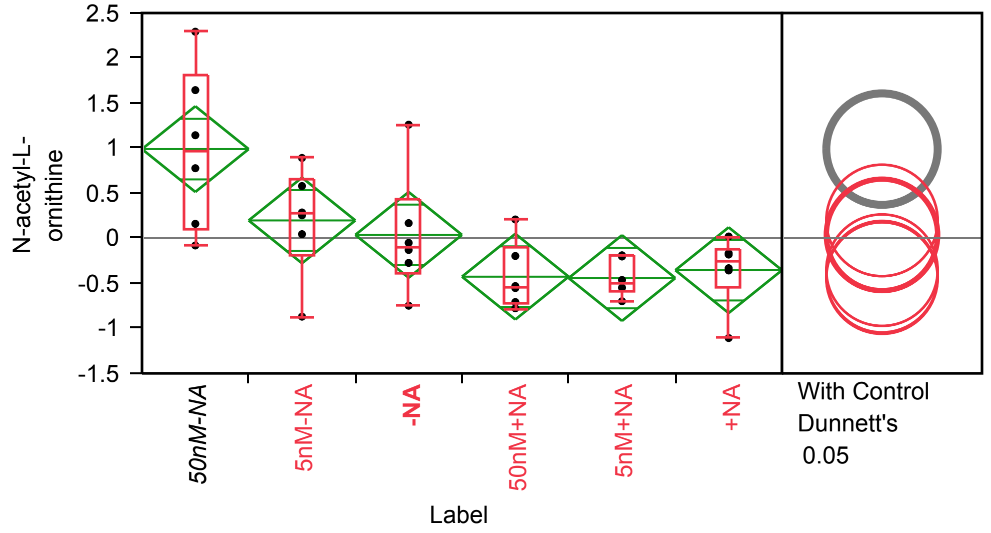

Quantiles

Level	Minimum	10%	25%	Median	75%	90%	Maximum
50nM-NA	-0.0805	-0.0805	0.099595	0.960043	1.806041	2.292351	2.292351
5nM-NA	-0.87059	-0.87059	-0.1839	0.269925	0.657248	0.890848	0.890848
-NA	-0.74892	-0.74892	-0.39287	-0.09099	0.440681	1.259964	1.259964
50nM+NA	-0.77797	-0.77797	-0.72592	-0.54228	-0.09586	0.207951	0.207951
5nM+NA	-0.69906	-0.69906	-0.58701	-0.50622	-0.19958	-0.18964	-0.18964
+NA	-1.10806	-1.10806	-0.54548	-0.2572	-0.12056	0.019034	0.019034

Oneway Anova

Summary of Fit

Rsquare	0.483426
Adj Rsquare	0.39733
Root Mean Square Error	0.570421
Mean of Response	2.78e-10
Observations (or Sum Wgts)	36

Analysis of Variance

Source	DF	Sum of Squares	Mean Square	F Ratio	Prob > F
Label	5	9.135038	1.82701	5.6150	0.0009 *
Error	30	9.761416	0.32538		
C. Total	35	18.896454			

Means for Oneway Anova

Level	Number	Mean	Std Error	Lower 95%	Upper 95%
50nM-NA	6	0.98925	0.23287	0.5137	1.4648
5nM-NA	6	0.19741	0.23287	-0.2782	0.6730
-NA	6	0.03708	0.23287	-0.4385	0.5127
50nM+NA	6	-0.42671	0.23287	-0.9023	0.0489
5nM+NA	6	-0.44228	0.23287	-0.9179	0.0333
+NA	6	-0.35474	0.23287	-0.8303	0.1208

Std Error uses a pooled estimate of error variance

Means Comparisons

Comparisons with a control using Dunnett's Method

Oneway Analysis of N-Acetylputrescine By Label

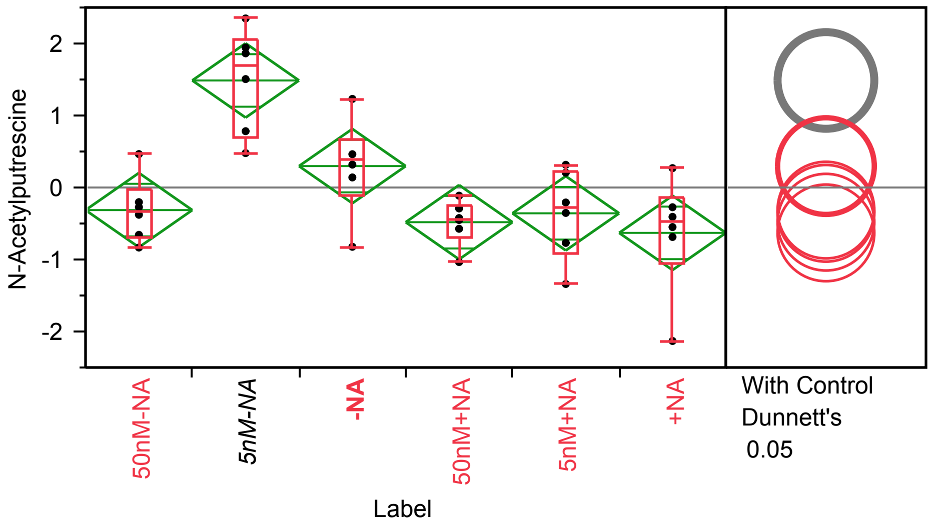

Quantiles

Level	Minimum	10%	25%	Median	75%	90%	Maximum
50nM-NA	-0.8339	-0.8339	-0.70173	-0.32594	-0.03665	0.46401	0.46401
5nM-NA	0.477884	0.477884	0.705997	1.684603	2.044639	2.348161	2.348161
-NA	-0.8235	-0.8235	-0.10092	0.387743	0.654163	1.230231	1.230231
50nM+NA	-1.03595	-1.03595	-0.68885	-0.43826	-0.24682	-0.11396	-0.11396
5nM+NA	-1.33994	-1.33994	-0.91199	-0.28064	0.233243	0.314764	0.314764
+NA	-2.13169	-2.13169	-1.04832	-0.4787	-0.13843	0.268818	0.268818

Oneway Anova

Summary of Fit

Rsquare	0.622299
Adj Rsquare	0.559348
Root Mean Square Error	0.618876
Mean of Response	5.556e-9
Observations (or Sum Wgts)	36

Analysis of Variance

Source	DF	Sum of Squares	Mean Square	F Ratio	Prob > F
Label	5	18.931233	3.78625	9.8856	<.0001 *
Error	30	11.490229	0.38301		
C. Total	35	30.421461			

Means for Oneway Anova

Level	Number	Mean	Std Error	Lower 95%	Upper 95%
50nM-NA	6	-0.3138	0.25266	-0.830	0.202
5nM-NA	6	1.4868	0.25266	0.971	2.003
-NA	6	0.2974	0.25266	-0.219	0.813
50nM+NA	6	-0.4818	0.25266	-0.998	0.034
5nM+NA	6	-0.3583	0.25266	-0.874	0.158
+NA	6	-0.6303	0.25266	-1.146	-0.114

Std Error uses a pooled estimate of error variance

Means Comparisons

Comparisons with a control using Dunnett's Method

Oneway Analysis of NAD By Label

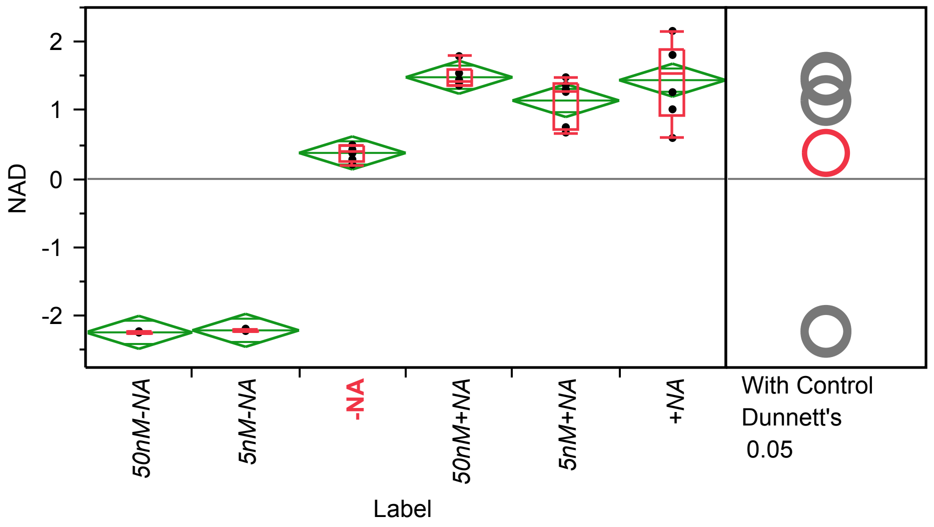

Quantiles

Level	Minimum	10%	25%	Median	75%	90%	Maximum
50nM-NA	-2.24897	-2.24897	-2.24516	-2.24183	-2.22549	-2.22207	-2.22207
5nM-NA	-2.22141	-2.22141	-2.21851	-2.21226	-2.19762	-2.1839	-2.1839
-NA	0.209614	0.209614	0.26634	0.401602	0.48611	0.494455	0.494455
50nM+NA	1.351313	1.351313	1.359352	1.425506	1.604079	1.791009	1.791009
5nM+NA	0.676387	0.676387	0.735092	1.287275	1.398977	1.482671	1.482671
+NA	0.597507	0.597507	0.910913	1.53172	1.895627	2.157939	2.157939

Oneway Anova

Summary of Fit

Rsquare	0.97418
Adj Rsquare	0.969877
Root Mean Square Error	0.287631
Mean of Response	-2.78e-9
Observations (or Sum Wgts)	36

Analysis of Variance

Source	DF	Sum of Squares	Mean Square	F Ratio	Prob > F
Label	5	93.644818	18.7290	226.3822	<.0001 *
Error	30	2.481948	0.0827		
C. Total	35	96.126766			

Means for Oneway Anova

Level	Number	Mean	Std Error	Lower 95%	Upper 95%
50nM-NA	6	-2.2375	0.11742	-2.477	-1.998
5nM-NA	6	-2.2083	0.11742	-2.448	-1.968
-NA	6	0.3793	0.11742	0.139	0.619
50nM+NA	6	1.4829	0.11742	1.243	1.723
5nM+NA	6	1.1432	0.11742	0.903	1.383
+NA	6	1.4404	0.11742	1.201	1.680

Std Error uses a pooled estimate of error variance

Means Comparisons

Comparisons with a control using Dunnett's Method

Oneway Analysis of NADH By Label

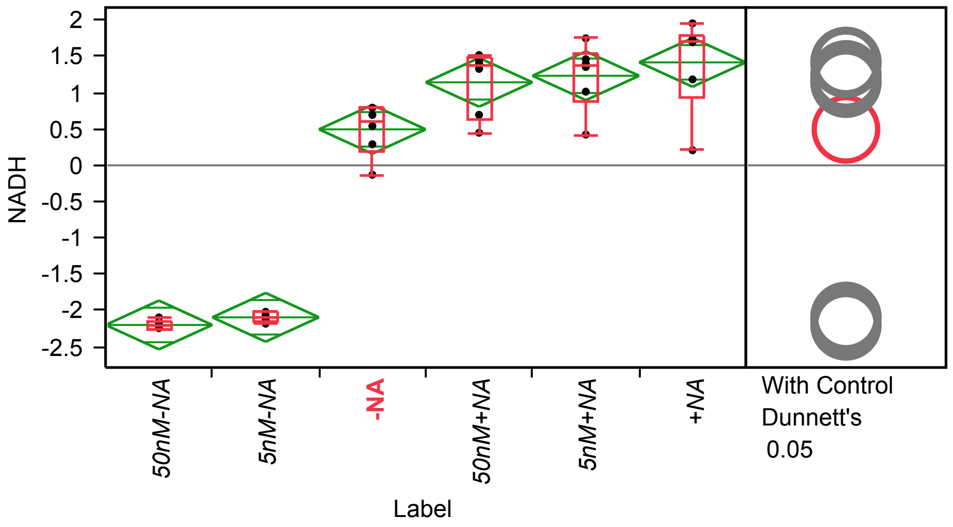

Quantiles

Level	Minimum	10%	25%	Median	75%	90%	Maximum
50nM-NA	-2.25544	-2.25544	-2.24921	-2.21527	-2.14888	-2.09643	-2.09643
5nM-NA	-2.18142	-2.18142	-2.15465	-2.09803	-2.02195	-2.01832	-2.01832
-NA	-0.13001	-0.13001	0.185121	0.617777	0.788719	0.793657	0.793657
50nM+NA	0.449379	0.449379	0.634142	1.369918	1.475598	1.519395	1.519395
5nM+NA	0.424097	0.424097	0.867603	1.376716	1.5339	1.750207	1.750207
+NA	0.208154	0.208154	0.939953	1.712849	1.793727	1.956698	1.956698

Oneway Anova

Summary of Fit

Rsquare	0.946326
Adj Rsquare	0.937381
Root Mean Square Error	0.402958
Mean of Response	-2.78e-9
Observations (or Sum Wgts)	36

Analysis of Variance

Source	DF	Sum of Squares	Mean Square	F Ratio	Prob > F
Label	5	85.885721	17.1771	105.7867	<.0001 *
Error	30	4.871260	0.1624		
C. Total	35	90.756981			

Means for Oneway Anova

Level	Number	Mean	Std Error	Lower 95%	Upper 95%
50nM-NA	6	-2.1993	0.16451	-2.535	-1.863
5nM-NA	6	-2.0941	0.16451	-2.430	-1.758
-NA	6	0.4961	0.16451	0.160	0.832
50nM+NA	6	1.1442	0.16451	0.808	1.480
5nM+NA	6	1.2342	0.16451	0.898	1.570
+NA	6	1.4190	0.16451	1.083	1.755

Std Error uses a pooled estimate of error variance

Means Comparisons

Comparisons with a control using Dunnett's Method

Oneway Analysis of NADP+ By Label

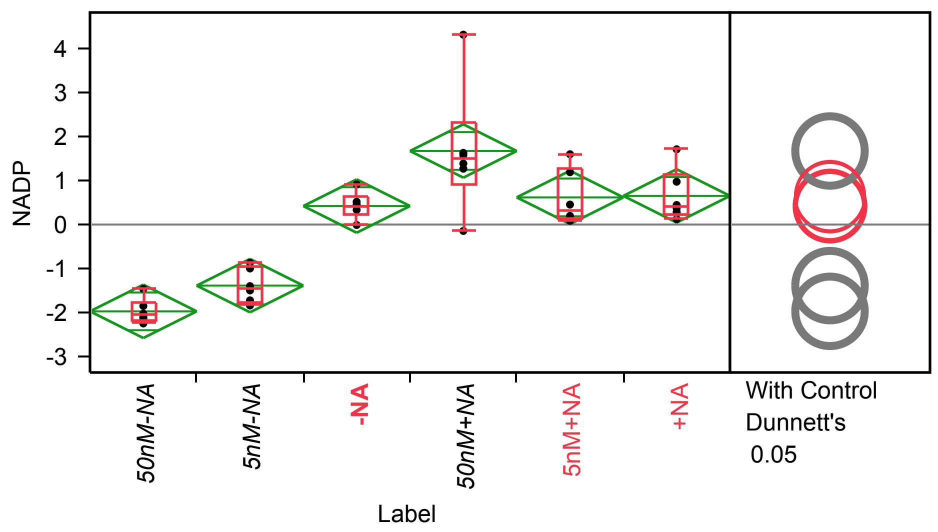

Quantiles

Level	Minimum	10%	25%	Median	75%	90%	Maximum
50nM-NA	-2.25251	-2.25251	-2.17287	-2.07015	-1.75686	-1.46487	-1.46487
5nM-NA	-1.84284	-1.84284	-1.75528	-1.45125	-0.9682	-0.87254	-0.87254
-NA	-0.01029	-0.01029	0.234566	0.395118	0.616979	0.913401	0.913401
50nM+NA	-0.14542	-0.14542	0.917224	1.484561	2.304887	4.323327	4.323327
5nM+NA	0.08732	0.08732	0.157226	0.325706	1.294124	1.600833	1.600833
+NA	0.125014	0.125014	0.244009	0.407463	1.159547	1.713533	1.713533

Oneway Anova

Summary of Fit

Rsquare	0.783878
Adj Rsquare	0.747857
Root Mean Square Error	0.728756
Mean of Response	2.78e-10
Observations (or Sum Wgts)	36

Analysis of Variance

Source	DF	Sum of Squares	Mean Square	F Ratio	Prob > F
Label	5	57.787607	11.5575	21.7621	<.0001 *
Error	30	15.932575	0.5311		
C. Total	35	73.720182			

Means for Oneway Anova

Level	Number	Mean	Std Error	Lower 95%	Upper 95%
50nM-NA	6	-1.9764	0.29751	-2.584	-1.369
5nM-NA	6	-1.3907	0.29751	-1.998	-0.783
-NA	6	0.4213	0.29751	-0.186	1.029
50nM+NA	6	1.6751	0.29751	1.067	2.283
5nM+NA	6	0.6187	0.29751	0.011	1.226
+NA	6	0.6520	0.29751	0.044	1.260

Std Error uses a pooled estimate of error variance

Means Comparisons

Comparisons with a control using Dunnett's Method

Oneway Analysis of N-carbamoyl-L-aspartate By Label

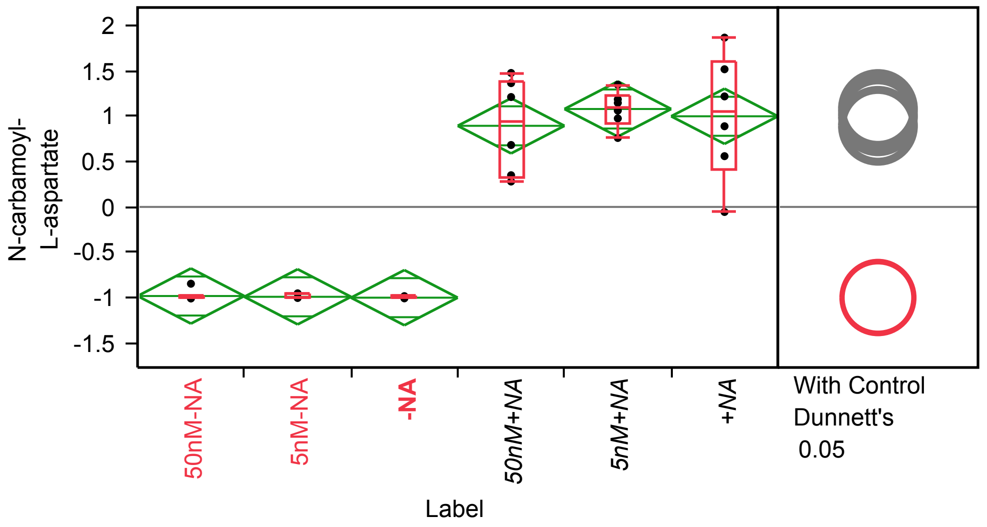

Quantiles

Level	Minimum	10%	25%	Median	75%	90%	Maximum
50nM-NA	-1.00729	-1.00729	-1.00729	-1.00728	-0.96671	-0.84503	-0.84503
5nM-NA	-1.0073	-1.0073	-1.00729	-1.00458	-0.95687	-0.95083	-0.95083
-NA	-1.00729	-1.00729	-1.00729	-1.00654	-0.98188	-0.98161	-0.98161
50nM+NA	0.279615	0.279615	0.331556	0.94531	1.390222	1.473899	1.473899
5nM+NA	0.758894	0.758894	0.920197	1.102323	1.224004	1.34458	1.34458
+NA	-0.0554	-0.0554	0.405075	1.051909	1.601939	1.862975	1.862975

Oneway Anova

Summary of Fit

Rsquare	0.898792
Adj Rsquare	0.881924
Root Mean Square Error	0.364174
Mean of Response	2.778e-9
Observations (or Sum Wgts)	36

Analysis of Variance

Source	DF	Sum of Squares	Mean Square	F Ratio	Prob > F
Label	5	35.333050	7.06661	53.2837	<.0001 *
Error	30	3.978674	0.13262		
C. Total	35	39.311724			

Means for Oneway Anova

Level	Number	Mean	Std Error	Lower 95%	Upper 95%
50nM-NA	6	-0.9802	0.14867	-1.284	-0.677
5nM-NA	6	-0.9889	0.14867	-1.293	-0.685
-NA	6	-0.9985	0.14867	-1.302	-0.695
50nM+NA	6	0.8926	0.14867	0.589	1.196
5nM+NA	6	1.0776	0.14867	0.774	1.381
+NA	6	0.9975	0.14867	0.694	1.301

Std Error uses a pooled estimate of error variance

Means Comparisons

Comparisons with a control using Dunnett's Method

Oneway Analysis of N-Formylglycinamide ribonucleotide By Label

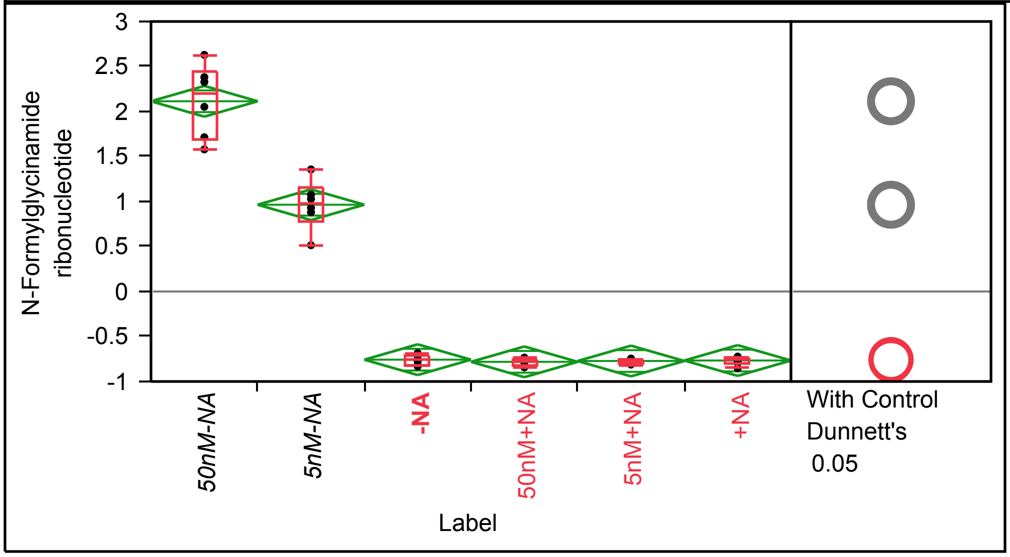

Quantiles

Level	Minimum	10%	25%	Median	75%	90%	Maximum
50nM-NA	1.578984	1.578984	1.679948	2.189743	2.443306	2.628926	2.628926
5nM-NA	0.514312	0.514312	0.788469	0.978665	1.146593	1.356577	1.356577
-NA	-0.83259	-0.83259	-0.82116	-0.74706	-0.70842	-0.68466	-0.68466
50nM+NA	-0.84318	-0.84318	-0.82617	-0.77131	-0.74456	-0.73437	-0.73437
5nM+NA	-0.81212	-0.81212	-0.79499	-0.76825	-0.74879	-0.74513	-0.74513
+NA	-0.85184	-0.85184	-0.80675	-0.75356	-0.72575	-0.72304	-0.72304

Oneway Anova

Summary of Fit

Rsquare	0.973734
Adj Rsquare	0.969356
Root Mean Square Error	0.204687
Mean of Response	-8.33e-9
Observations (or Sum Wgts)	36

Analysis of Variance

Source	DF	Sum of Squares	Mean Square	F Ratio	Prob > F
Label	5	46.595972	9.31919	222.4328	<.0001 *
Error	30	1.256900	0.04190		
C. Total	35	47.852872			

Means for Oneway Anova

Level	Number	Mean	Std Error	Lower 95%	Upper 95%
50nM-NA	6	2.1137	0.08356	1.943	2.284
5nM-NA	6	0.9641	0.08356	0.793	1.135
-NA	6	-0.7575	0.08356	-0.928	-0.587
50nM+NA	6	-0.7814	0.08356	-0.952	-0.611
5nM+NA	6	-0.7722	0.08356	-0.943	-0.602
+NA	6	-0.7667	0.08356	-0.937	-0.596

Std Error uses a pooled estimate of error variance

Means Comparisons

Comparisons with a control using Dunnett's Method

Oneway Analysis of Ng,NG-dimethyl-L-arginine By Label

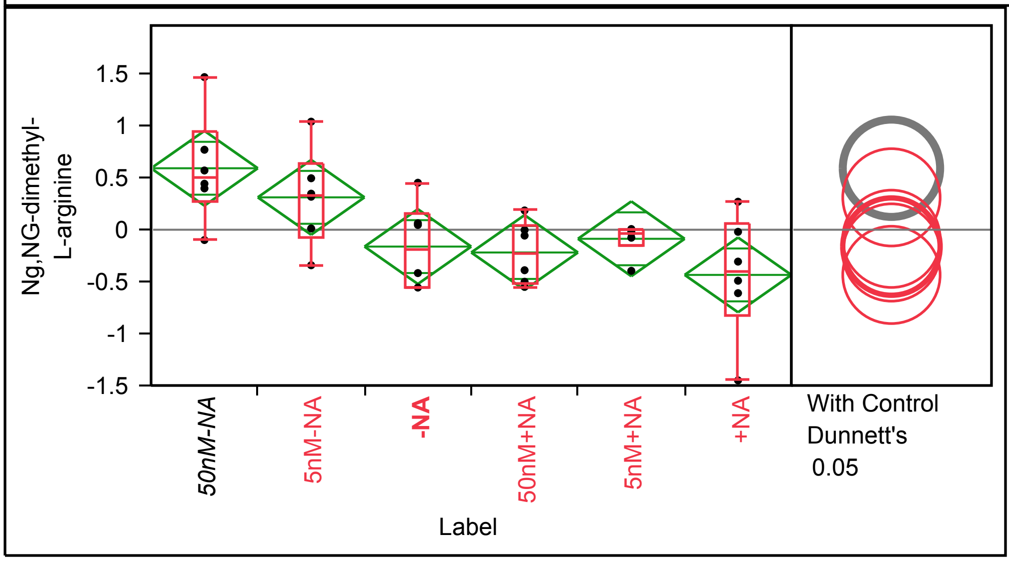

Quantiles

Level	Minimum	10%	25%	Median	75%	90%	Maximum
50nM-NA	-0.09913	-0.09913	0.273615	0.50663	0.945038	1.469638	1.469638
5nM-NA	-0.34194	-0.34194	-0.07632	0.332408	0.631947	1.040386	1.040386
-NA	-0.56029	-0.56029	-0.55765	-0.18576	0.159774	0.452268	0.452268
50nM+NA	-0.55018	-0.55018	-0.51344	-0.22326	0.044297	0.185686	0.185686
5nM+NA	-0.39577	-0.39577	-0.1566	-0.02757	-0.00033	0.006646	0.006646
+NA	-1.45054	-1.45054	-0.82077	-0.39906	0.052211	0.269494	0.269494

Oneway Anova

Summary of Fit

Rsquare	0.435227
Adj Rsquare	0.341098
Root Mean Square Error	0.431965
Mean of Response	-2.5e-17
Observations (or Sum Wgts)	36

Analysis of Variance

Source	DF	Sum of Squares	Mean Square	F Ratio	Prob > F
Label	5	4.3137945	0.862759	4.6237	0.0030 *
Error	30	5.5978086	0.186594		
C. Total	35	9.9116031			

Means for Oneway Anova

Level	Number	Mean	Std Error	Lower 95%	Upper 95%
50nM-NA	6	0.59197	0.17635	0.2318	0.9521
5nM-NA	6	0.31188	0.17635	-0.0483	0.6720
-NA	6	-0.16234	0.17635	-0.5225	0.1978
50nM+NA	6	-0.21917	0.17635	-0.5793	0.1410
5nM+NA	6	-0.08730	0.17635	-0.4475	0.2729
+NA	6	-0.43504	0.17635	-0.7952	-0.0749

Std Error uses a pooled estimate of error variance

Means Comparisons

Comparisons with a control using Dunnett's Method

Oneway Analysis of Nicotinamide By Label

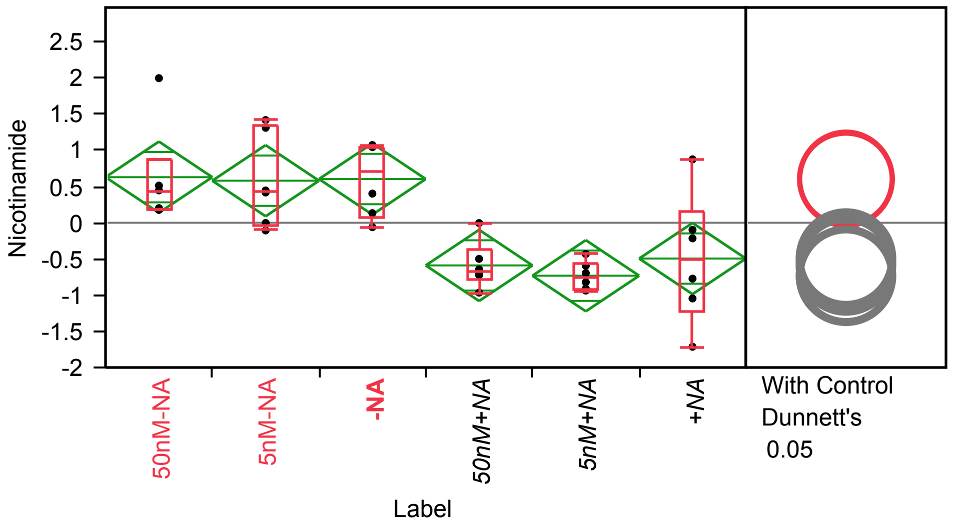

Quantiles

Level	Minimum	10%	25%	Median	75%	90%	Maximum
50nM-NA	0.177646	0.177646	0.195933	0.44531	0.884358	1.998141	1.998141
5nM-NA	-0.1059	-0.1059	-0.02887	0.432061	1.339371	1.419294	1.419294
-NA	-0.06032	-0.06032	0.084474	0.717513	1.052479	1.073869	1.073869
50nM+NA	-0.96516	-0.96516	-0.78368	-0.67266	-0.3751	-0.00445	-0.00445
5nM+NA	-0.93696	-0.93696	-0.91738	-0.75851	-0.55324	-0.43205	-0.43205
+NA	-1.71358	-1.71358	-1.21267	-0.49382	0.145915	0.876627	0.876627

Oneway Anova

Summary of Fit

Rsquare	0.561477
Adj Rsquare	0.488389
Root Mean Square Error	0.589927
Mean of Response	2.78e-11
Observations (or Sum Wgts)	36

Analysis of Variance

Source	DF	Sum of Squares	Mean Square	F Ratio	Prob > F
Label	5	13.367723	2.67354	7.6823	<.0001 *
Error	30	10.440430	0.34801		
C. Total	35	23.808153			

Means for Oneway Anova

Level	Number	Mean	Std Error	Lower 95%	Upper 95%
50nM-NA	6	0.63026	0.24084	0.138	1.122
5nM-NA	6	0.58117	0.24084	0.089	1.073
-NA	6	0.60444	0.24084	0.113	1.096
50nM+NA	6	-0.58946	0.24084	-1.081	-0.098
5nM+NA	6	-0.73176	0.24084	-1.224	-0.240
+NA	6	-0.49466	0.24084	-0.987	-0.0028

Std Error uses a pooled estimate of error variance

Means Comparisons

Comparisons with a control using Dunnett's Method

Oneway Analysis of Nicotinamide ribotide By Label

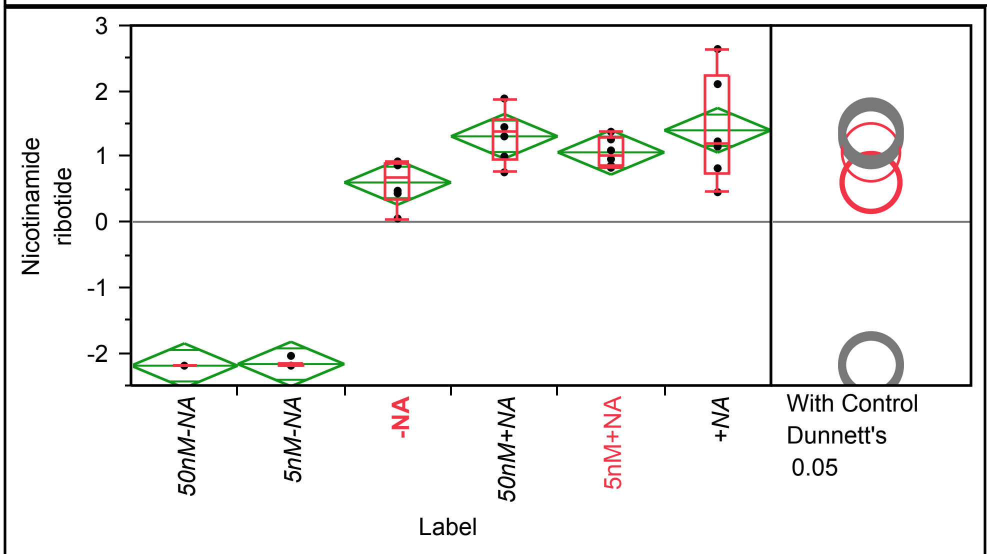

Quantiles

Level	Minimum	10%	25%	Median	75%	90%	Maximum
50nM-NA	-2.19747	-2.19747	-2.19747	-2.19747	-2.19746	-2.19746	-2.19746
5nM-NA	-2.19748	-2.19748	-2.19748	-2.19747	-2.15997	-2.04749	-2.04749
-NA	0.051029	0.051029	0.338819	0.66921	0.878556	0.923129	0.923129
50nM+NA	0.756894	0.756894	0.938281	1.375793	1.557227	1.884543	1.884543
5nM+NA	0.826685	0.826685	0.853419	1.024606	1.288926	1.376602	1.376602
+NA	0.452982	0.452982	0.727049	1.189176	2.238305	2.640088	2.640088

Oneway Anova

Summary of Fit

Rsquare	0.946539
Adj Rsquare	0.937629
Root Mean Square Error	0.407551
Mean of Response	2.778e-9
Observations (or Sum Wgts)	36

Analysis of Variance

Source	DF	Sum of Squares	Mean Square	F Ratio	Prob > F
Label	5	88.224461	17.6449	106.2322	<.0001 *
Error	30	4.982923	0.1661		
C. Total	35	93.207384			

Means for Oneway Anova

Level	Number	Mean	Std Error	Lower 95%	Upper 95%
50nM-NA	6	-2.1975	0.16638	-2.537	-1.858
5nM-NA	6	-2.1725	0.16638	-2.512	-1.833
-NA	6	0.6018	0.16638	0.262	0.942
50nM+NA	6	1.3066	0.16638	0.967	1.646
5nM+NA	6	1.0624	0.16638	0.723	1.402
+NA	6	1.3990	0.16638	1.059	1.739

Std Error uses a pooled estimate of error variance

Means Comparisons

Comparisons with a control using Dunnett's Method

Oneway Analysis of Nicotinic acid By Label

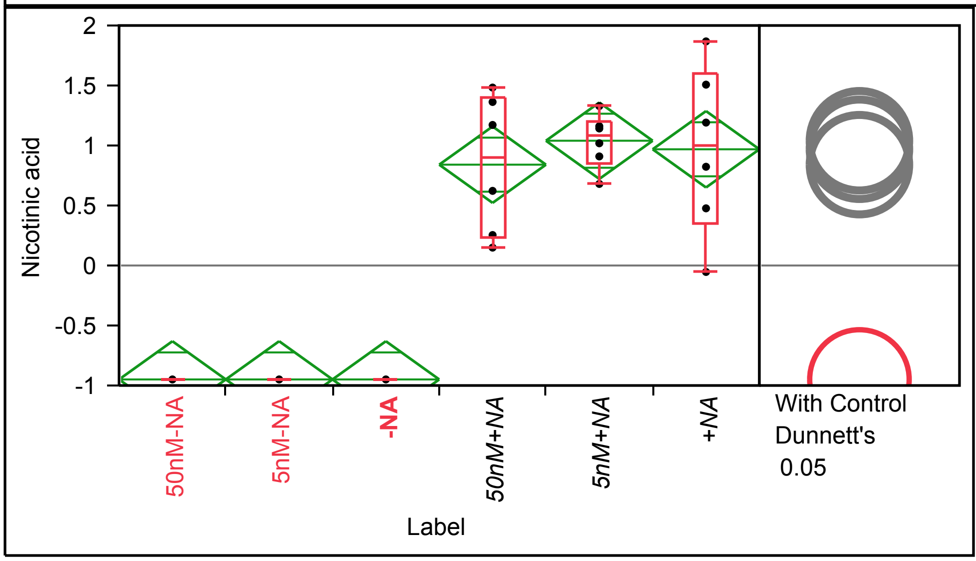

Quantiles

Level	Minimum	10%	25%	Median	75%	90%	Maximum
50nM-NA	-0.94949	-0.94949	-0.94949	-0.94948	-0.94947	-0.94947	-0.94947
5nM-NA	-0.9495	-0.9495	-0.94949	-0.94948	-0.94948	-0.94947	-0.94947
-NA	-0.9495	-0.9495	-0.94949	-0.94949	-0.94949	-0.94949	-0.94949
50nM+NA	0.149068	0.149068	0.227598	0.896574	1.392743	1.482626	1.482626
5nM+NA	0.679874	0.679874	0.851666	1.080129	1.202779	1.328627	1.328627
+NA	-0.05373	-0.05373	0.343848	1.006826	1.597139	1.867473	1.867473

Oneway Anova

Summary of Fit

Rsquare	0.881576
Adj Rsquare	0.861838
Root Mean Square Error	0.381934
Mean of Response	8.33e-10
Observations (or Sum Wgts)	36

Analysis of Variance

Source	DF	Sum of Squares	Mean Square	F Ratio	Prob > F
Label	5	32.577401	6.51548	44.6653	<.0001 *
Error	30	4.376201	0.14587		
C. Total	35	36.953603			

Means for Oneway Anova

Level	Number	Mean	Std Error	Lower 95%	Upper 95%
50nM-NA	6	-0.9495	0.15592	-1.268	-0.631
5nM-NA	6	-0.9495	0.15592	-1.268	-0.631
-NA	6	-0.9495	0.15592	-1.268	-0.631
50nM+NA	6	0.8402	0.15592	0.522	1.159
5nM+NA	6	1.0398	0.15592	0.721	1.358
+NA	6	0.9685	0.15592	0.650	1.287

Std Error uses a pooled estimate of error variance

Means Comparisons

Comparisons with a control using Dunnett's Method

Oneway Analysis of N-Methylantranilic acid By Label

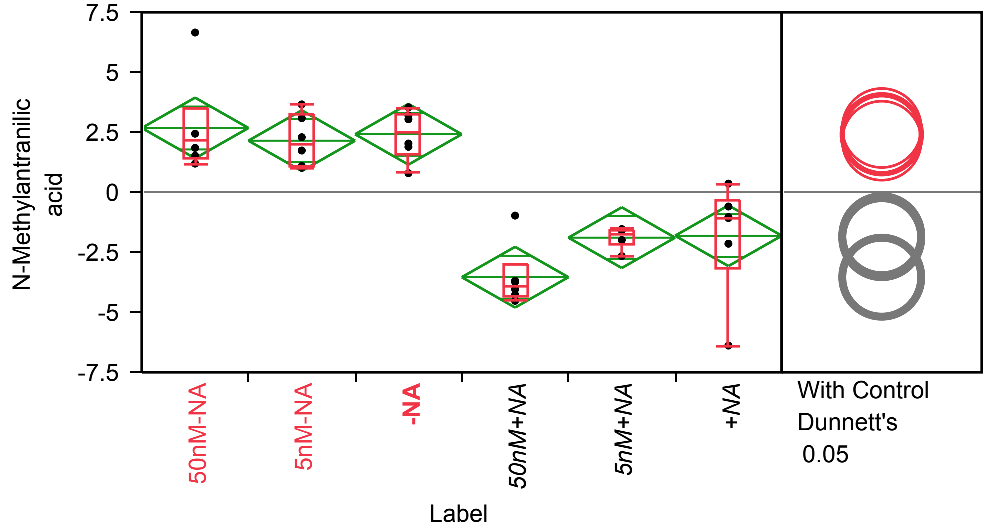

Quantiles

Level	Minimum	10%	25%	Median	75%	90%	Maximum
50nM-NA	1.193095	1.193095	1.439418	2.128231	3.493636	6.655462	6.655462
5nM-NA	1.021455	1.021455	1.074028	2.015678	3.231063	3.660355	3.660355
-NA	0.794815	0.794815	1.618562	2.535682	3.284603	3.53883	3.53883
50nM+NA	-4.52343	-4.52343	-4.33593	-3.88959	-3.00744	-0.97293	-0.97293
5nM+NA	-2.66015	-2.66015	-2.18556	-1.79009	-1.54496	-1.535	-1.535
+NA	-6.38735	-6.38735	-3.20529	-1.0524	-0.35566	0.359379	0.359379

Oneway Anova

Summary of Fit

Rsquare	0.762944
Adj Rsquare	0.723434
Root Mean Square Error	1.516607
Mean of Response	-1.11e-8
Observations (or Sum Wgts)	36

Analysis of Variance

Source	DF	Sum of Squares	Mean Square	F Ratio	Prob > F
Label	5	222.07946	44.4159	19.3105	<.0001 *
Error	30	69.00288	2.3001		
C. Total	35	291.08234			

Means for Oneway Anova

Level	Number	Mean	Std Error	Lower 95%	Upper 95%
50nM-NA	6	2.6777	0.61915	1.413	3.942
5nM-NA	6	2.1488	0.61915	0.884	3.413
-NA	6	2.4163	0.61915	1.152	3.681
50nM+NA	6	-3.5391	0.61915	-4.804	-2.275
5nM+NA	6	-1.8918	0.61915	-3.156	-0.627
+NA	6	-1.8119	0.61915	-3.076	-0.547

Std Error uses a pooled estimate of error variance

Means Comparisons

Comparisons with a control using Dunnett's Method

Oneway Analysis of O-acetyl-L-serine By Label

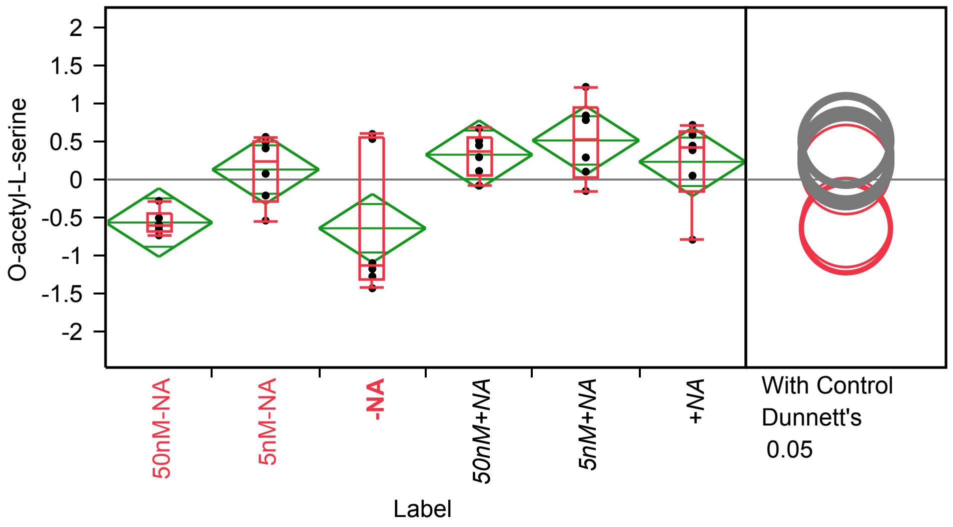

Quantiles

Level	Minimum	10%	25%	Median	75%	90%	Maximum
50nM-NA	-0.7377	-0.7377	-0.68396	-0.6069	-0.45232	-0.28201	-0.28201
5nM-NA	-0.54152	-0.54152	-0.29377	0.244636	0.511512	0.562855	0.562855
-NA	-1.43633	-1.43633	-1.31843	-1.14234	0.553022	0.601515	0.601515
50nM+NA	-0.07865	-0.07865	0.064724	0.373203	0.554132	0.676668	0.676668
5nM+NA	-0.15083	-0.15083	0.040264	0.539043	0.940393	1.224106	1.224106
+NA	-0.79465	-0.79465	-0.16007	0.41796	0.623979	0.720782	0.720782

Oneway Anova

Summary of Fit

Rsquare	0.445871
Adj Rsquare	0.353516
Root Mean Square Error	0.542565
Mean of Response	8.33e-10
Observations (or Sum Wgts)	36

Analysis of Variance

Source	DF	Sum of Squares	Mean Square	F Ratio	Prob > F
Label	5	7.105952	1.42119	4.8278	0.0023 *
Error	30	8.831301	0.29438		
C. Total	35	15.937253			

Means for Oneway Anova

Level	Number	Mean	Std Error	Lower 95%	Upper 95%
50nM-NA	6	-0.56811	0.22150	-1.020	-0.1157
5nM-NA	6	0.13230	0.22150	-0.320	0.5847
-NA	6	-0.64363	0.22150	-1.096	-0.1913
50nM+NA	6	0.32837	0.22150	-0.124	0.7807
5nM+NA	6	0.51686	0.22150	0.064	0.9692
+NA	6	0.23420	0.22150	-0.218	0.6866

Std Error uses a pooled estimate of error variance

Means Comparisons

Comparisons with a control using Dunnett's Method

Oneway Analysis of octulose-monophosphate By Label

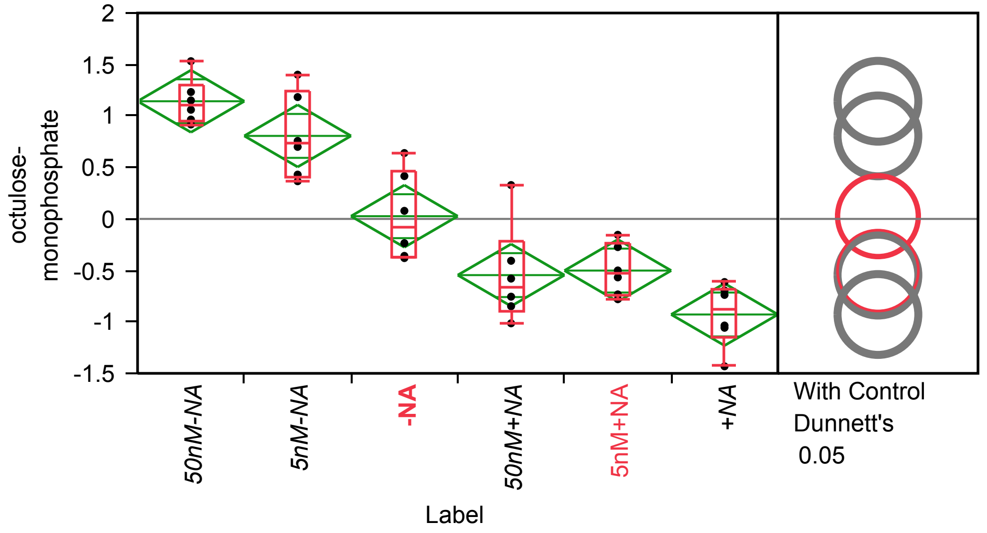

Quantiles

Level	Minimum	10%	25%	Median	75%	90%	Maximum
50nM-NA	0.91743	0.91743	0.952972	1.105549	1.307367	1.532007	1.532007
5nM-NA	0.3661	0.3661	0.414345	0.728616	1.237739	1.400774	1.400774
-NA	-0.38033	-0.38033	-0.36449	-0.0785	0.471987	0.63977	0.63977
50nM+NA	-1.01401	-1.01401	-0.88949	-0.66745	-0.22279	0.328796	0.328796
5nM+NA	-0.77997	-0.77997	-0.7449	-0.53426	-0.24392	-0.15452	-0.15452
+NA	-1.4318	-1.4318	-1.15093	-0.88675	-0.6742	-0.61326	-0.61326

Oneway Anova

Summary of Fit

Rsquare	0.837345
Adj Rsquare	0.810236
Root Mean Square Error	0.361766
Mean of Response	2.78e-10
Observations (or Sum Wgts)	36

Analysis of Variance

Source	DF	Sum of Squares	Mean Square	F Ratio	Prob > F
Label	5	20.212285	4.04246	30.8879	<.0001 *
Error	30	3.926247	0.13087		
C. Total	35	24.138532			

Means for Oneway Anova

Level	Number	Mean	Std Error	Lower 95%	Upper 95%
50nM-NA	6	1.1430	0.14769	0.841	1.445
5nM-NA	6	0.8063	0.14769	0.505	1.108
-NA	6	0.0265	0.14769	-0.275	0.328
50nM+NA	6	-0.5458	0.14769	-0.847	-0.244
5nM+NA	6	-0.5017	0.14769	-0.803	-0.200
+NA	6	-0.9284	0.14769	-1.230	-0.627

Std Error uses a pooled estimate of error variance

Means Comparisons

Comparisons with a control using Dunnett's Method

Oneway Analysis of ornithine By Label

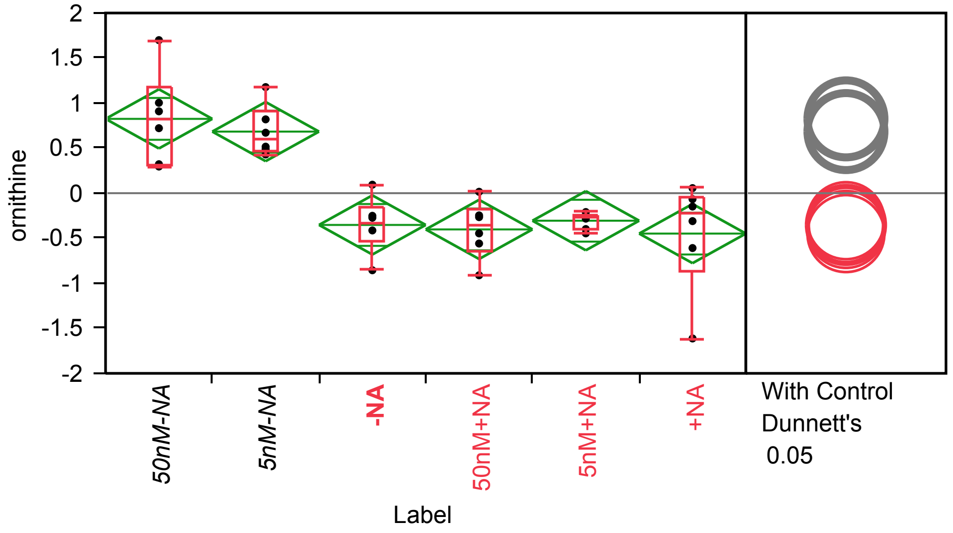

Quantiles

Level	Minimum	10%	25%	Median	75%	90%	Maximum
50nM-NA	0.295827	0.295827	0.315055	0.815098	1.177161	1.698805	1.698805
5nM-NA	0.429514	0.429514	0.47362	0.593711	0.90894	1.177517	1.177517
-NA	-0.85555	-0.85555	-0.52612	-0.34306	-0.16496	0.093936	0.093936
50nM+NA	-0.91137	-0.91137	-0.64686	-0.35607	-0.18107	0.015322	0.015322
5nM+NA	-0.44581	-0.44581	-0.41026	-0.26769	-0.23512	-0.20948	-0.20948
+NA	-1.61274	-1.61274	-0.85997	-0.22927	-0.03551	0.056038	0.056038

Oneway Anova

Summary of Fit

Rsquare	0.690171
Adj Rsquare	0.638533
Root Mean Square Error	0.393929
Mean of Response	4.93e-17
Observations (or Sum Wgts)	36

Analysis of Variance

Source	DF	Sum of Squares	Mean Square	F Ratio	Prob > F
Label	5	10.370326	2.07407	13.3655	<.0001 *
Error	30	4.655406	0.15518		
C. Total	35	15.025732			

Means for Oneway Anova

Level	Number	Mean	Std Error	Lower 95%	Upper 95%
50nM-NA	6	0.82493	0.16082	0.4965	1.153
5nM-NA	6	0.68370	0.16082	0.3553	1.012
-NA	6	-0.35255	0.16082	-0.6810	-0.024
50nM+NA	6	-0.40223	0.16082	-0.7307	-0.074
5nM+NA	6	-0.30546	0.16082	-0.6339	0.023
+NA	6	-0.44838	0.16082	-0.7768	-0.120

Std Error uses a pooled estimate of error variance

Means Comparisons

Comparisons with a control using Dunnett's Method

Oneway Analysis of orotate By Label

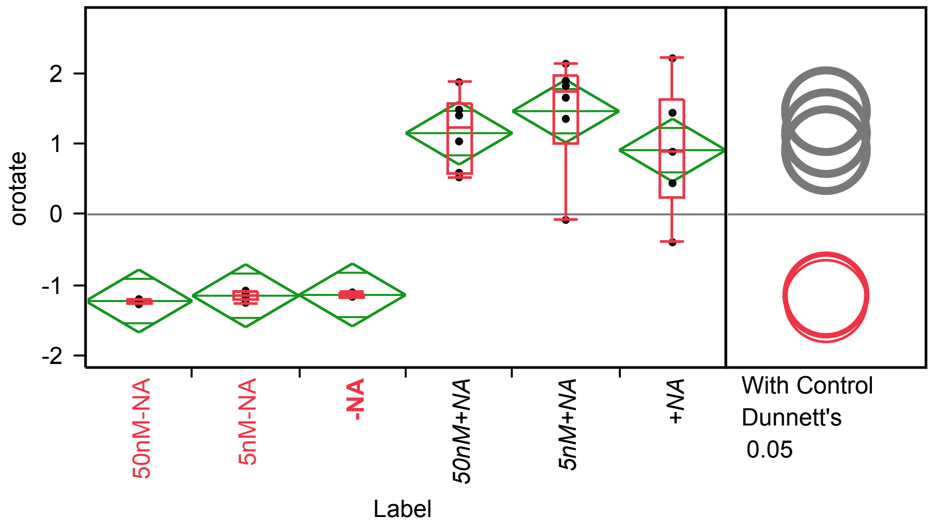

Quantiles

Level	Minimum	10%	25%	Median	75%	90%	Maximum
50nM-NA	-1.26978	-1.26978	-1.24684	-1.21455	-1.20783	-1.19795	-1.19795
5nM-NA	-1.24993	-1.24993	-1.21223	-1.1389	-1.08783	-1.07825	-1.07825
-NA	-1.17881	-1.17881	-1.16848	-1.13517	-1.1097	-1.10293	-1.10293
50nM+NA	0.523398	0.523398	0.571153	1.214077	1.575662	1.866089	1.866089
5nM+NA	-0.07859	-0.07859	0.991783	1.731076	1.947724	2.126855	2.126855
+NA	-0.39413	-0.39413	0.230953	0.879247	1.626829	2.20434	2.20434

Oneway Anova

Summary of Fit

Rsquare	0.855703
Adj Rsquare	0.831654
Root Mean Square Error	0.531635
Mean of Response	-7.4e-17
Observations (or Sum Wgts)	36

Analysis of Variance

Source	DF	Sum of Squares	Mean Square	F Ratio	Prob > F
Label	5	50.282324	10.0565	35.5810	<.0001 *
Error	30	8.479082	0.2826		
C. Total	35	58.761406			

Means for Oneway Anova

Level	Number	Mean	Std Error	Lower 95%	Upper 95%
50nM-NA	6	-1.2245	0.21704	-1.668	-0.781
5nM-NA	6	-1.1494	0.21704	-1.593	-0.706
-NA	6	-1.1382	0.21704	-1.581	-0.695
50nM+NA	6	1.1473	0.21704	0.704	1.591
5nM+NA	6	1.4578	0.21704	1.015	1.901
+NA	6	0.9071	0.21704	0.464	1.350

Std Error uses a pooled estimate of error variance

Means Comparisons

Comparisons with a control using Dunnett's Method

Oneway Analysis of Orotidine By Label

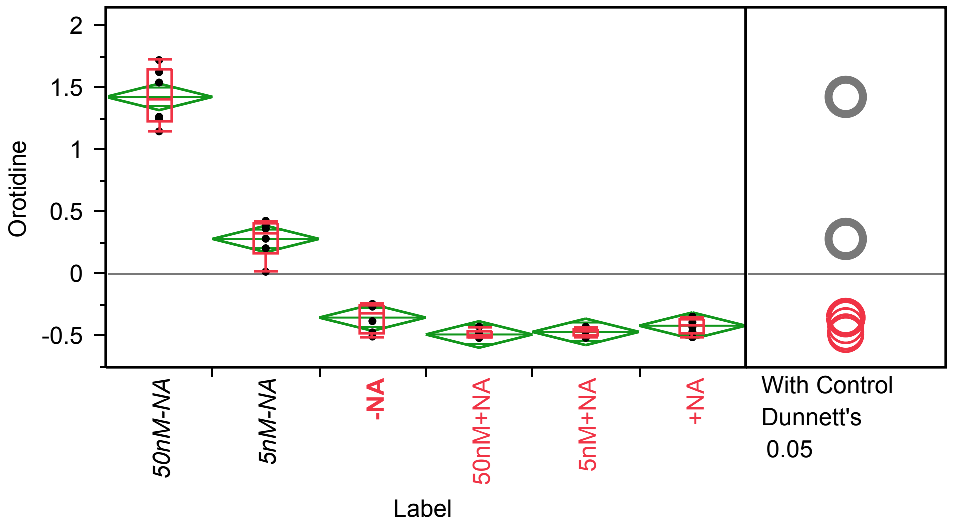

Quantiles

Level	Minimum	10%	25%	Median	75%	90%	Maximum
50nM-NA	1.151948	1.151948	1.230013	1.407545	1.654364	1.726587	1.726587
5nM-NA	0.020209	0.020209	0.162174	0.327239	0.403081	0.430836	0.430836
-NA	-0.50238	-0.50238	-0.47745	-0.31964	-0.24469	-0.23968	-0.23968
50nM+NA	-0.51416	-0.51416	-0.5075	-0.4975	-0.46382	-0.42229	-0.42229
5nM+NA	-0.51315	-0.51315	-0.49972	-0.45583	-0.43998	-0.42297	-0.42297
+NA	-0.50811	-0.50811	-0.47438	-0.40778	-0.35555	-0.34199	-0.34199

Oneway Anova

Summary of Fit

Rsquare	0.972298
Adj Rsquare	0.967682
Root Mean Square Error	0.127929
Mean of Response	-1.11e-9
Observations (or Sum Wgts)	36

Analysis of Variance

Source	DF	Sum of Squares	Mean Square	F Ratio	Prob > F
Label	5	17.232900	3.44658	210.5946	<.0001 *
Error	30	0.490978	0.01637		
C. Total	35	17.723878			

Means for Oneway Anova

Level	Number	Mean	Std Error	Lower 95%	Upper 95%
50nM-NA	6	1.4300	0.05223	1.323	1.537
5nM-NA	6	0.2848	0.05223	0.178	0.391
-NA	6	-0.3495	0.05223	-0.456	-0.243
50nM+NA	6	-0.4857	0.05223	-0.592	-0.379
5nM+NA	6	-0.4648	0.05223	-0.571	-0.358
+NA	6	-0.4148	0.05223	-0.521	-0.308

Std Error uses a pooled estimate of error variance

Means Comparisons

Comparisons with a control using Dunnett's Method

Oneway Analysis of Palmitic acid By Label

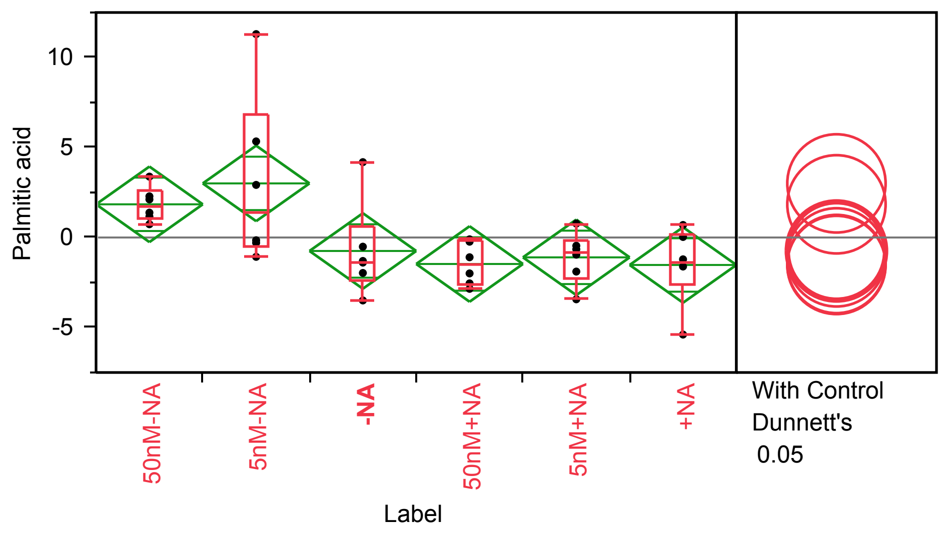

Quantiles

Level	Minimum	10%	25%	Median	75%	90%	Maximum
50nM-NA	0.736564	0.736564	1.053695	1.748289	2.561116	3.380835	3.380835
5nM-NA	-1.07402	-1.07402	-0.49108	1.38432	6.828279	11.29858	11.29858
-NA	-3.49675	-3.49675	-2.35222	-1.34916	0.663041	4.185627	4.185627
50nM+NA	-2.8505	-2.8505	-2.62467	-1.54642	-0.20013	-0.10223	-0.10223
5nM+NA	-3.43247	-3.43247	-2.27808	-0.80387	-0.15137	0.772145	0.772145
+NA	-5.39211	-5.39211	-2.59525	-1.41252	0.195825	0.694085	0.694085

Oneway Anova

Summary of Fit

Rsquare	0.371363
Adj Rsquare	0.26659
Root Mean Square Error	2.516463
Mean of Response	-5.6e-10
Observations (or Sum Wgts)	36

Analysis of Variance

Source	DF	Sum of Squares	Mean Square	F Ratio	Prob > F
Label	5	112.22810	22.4456	3.5445	0.0123 *
Error	30	189.97764	6.3326		
C. Total	35	302.20574			

Means for Oneway Anova

Level	Number	Mean	Std Error	Lower 95%	Upper 95%
50nM-NA	6	1.8435	1.0273	-0.255	3.9417
5nM-NA	6	3.0058	1.0273	0.908	5.1039
-NA	6	-0.7486	1.0273	-2.847	1.3496
50nM+NA	6	-1.4713	1.0273	-3.569	0.6268
5nM+NA	6	-1.1034	1.0273	-3.202	0.9947
+NA	6	-1.5260	1.0273	-3.624	0.5721

Std Error uses a pooled estimate of error variance

Means Comparisons

Comparisons with a control using Dunnett's Method

Oneway Analysis of pantothenate By Label

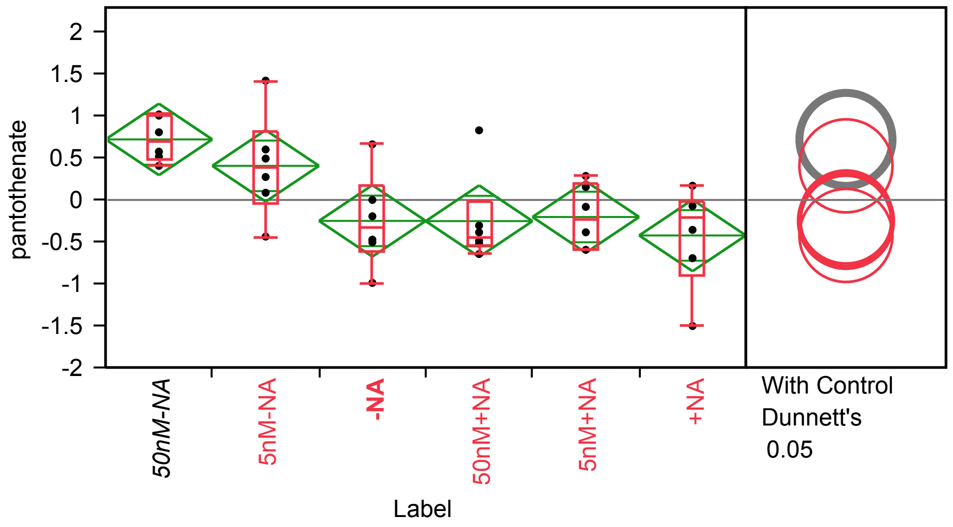

Quantiles

Level	Minimum	10%	25%	Median	75%	90%	Maximum
50nM-NA	0.407329	0.407329	0.489149	0.69236	1.010274	1.020622	1.020622
5nM-NA	-0.43735	-0.43735	-0.04508	0.383871	0.807595	1.423901	1.423901
-NA	-0.98979	-0.98979	-0.62616	-0.33457	0.166501	0.664041	0.664041
50nM+NA	-0.64544	-0.64544	-0.5519	-0.43749	-0.0218	0.831125	0.831125
5nM+NA	-0.59809	-0.59809	-0.5961	-0.23437	0.188178	0.286109	0.286109
+NA	-1.50457	-1.50457	-0.89739	-0.21998	-0.01175	0.169584	0.169584

Oneway Anova

Summary of Fit

Rsquare	0.44143
Adj Rsquare	0.348335
Root Mean Square Error	0.51181
Mean of Response	-4.9e-17
Observations (or Sum Wgts)	36

Analysis of Variance

Source	DF	Sum of Squares	Mean Square	F Ratio	Prob > F
Label	5	6.210452	1.24209	4.7417	0.0026 *
Error	30	7.858490	0.26195		
C. Total	35	14.068942			

Means for Oneway Anova

Level	Number	Mean	Std Error	Lower 95%	Upper 95%
50nM-NA	6	0.72265	0.20895	0.2959	1.1494
5nM-NA	6	0.40702	0.20895	-0.0197	0.8337
-NA	6	-0.24986	0.20895	-0.6766	0.1769
50nM+NA	6	-0.25269	0.20895	-0.6794	0.1740
5nM+NA	6	-0.20343	0.20895	-0.6302	0.2233
+NA	6	-0.42369	0.20895	-0.8504	0.0030

Std Error uses a pooled estimate of error variance

Means Comparisons

Comparisons with a control using Dunnett's Method

Oneway Analysis of PC(15:0) By Label

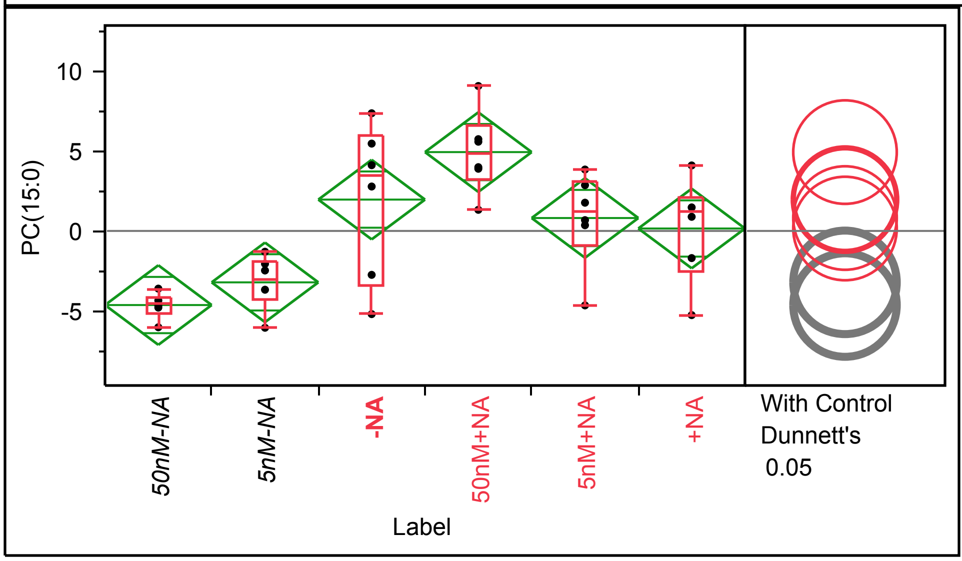

Quantiles

Level	Minimum	10%	25%	Median	75%	90%	Maximum
50nM-NA	-6.0111	-6.0111	-5.0904	-4.50541	-4.15608	-3.60084	-3.60084
5nM-NA	-6.04163	-6.04163	-4.29945	-3.06814	-1.86577	-1.2991	-1.2991
-NA	-5.19024	-5.19024	-3.35263	3.437032	5.922598	7.342681	7.342681
50nM+NA	1.318832	1.318832	3.23996	4.773788	6.555685	9.042641	9.042641
5nM+NA	-4.64536	-4.64536	-0.89418	1.217433	3.099722	3.830878	3.830878
+NA	-5.25276	-5.25276	-2.5838	1.152753	2.122812	4.086709	4.086709

Oneway Anova

Summary of Fit

Rsquare	0.57664
Adj Rsquare	0.50608
Root Mean Square Error	2.977539
Mean of Response	2.778e-9
Observations (or Sum Wgts)	36

Analysis of Variance

Source	DF	Sum of Squares	Mean Square	F Ratio	Prob > F
Label	5	362.26844	72.4537	8.1723	<.0001 *
Error	30	265.97220	8.8657		
C. Total	35	628.24065			

Means for Oneway Anova

Level	Number	Mean	Std Error	Lower 95%	Upper 95%
50nM-NA	6	-4.6246	1.2156	-7.107	-2.142
5nM-NA	6	-3.2084	1.2156	-5.691	-0.726
-NA	6	1.9559	1.2156	-0.527	4.438
50nM+NA	6	4.9193	1.2156	2.437	7.402
5nM+NA	6	0.8054	1.2156	-1.677	3.288
+NA	6	0.1522	1.2156	-2.330	2.635

Std Error uses a pooled estimate of error variance

Means Comparisons

Comparisons with a control using Dunnett's Method

Oneway Analysis of p-Coumaric acid By Label

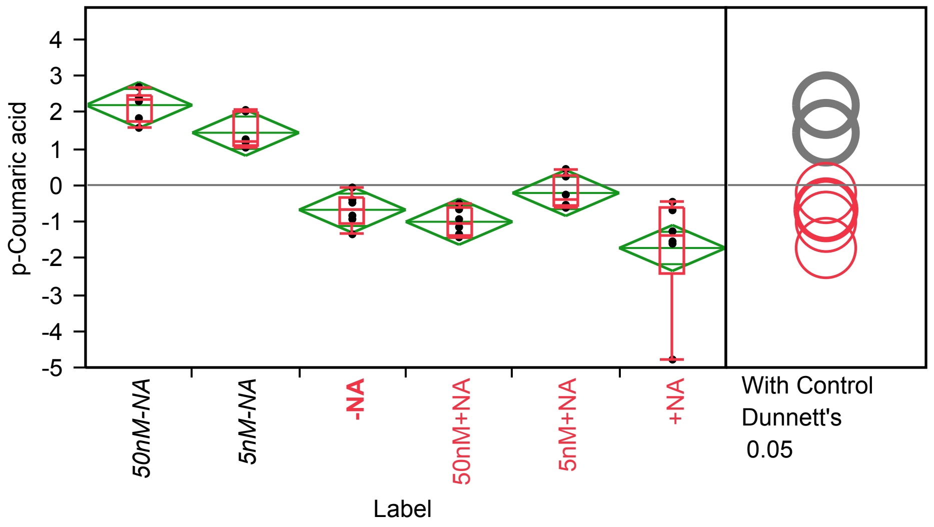

Quantiles

Level	Minimum	10%	25%	Median	75%	90%	Maximum
50nM-NA	1.568616	1.568616	1.767637	2.326255	2.474348	2.68842	2.68842
5nM-NA	1.026103	1.026103	1.059658	1.215462	2.028909	2.057468	2.057468
-NA	-1.34831	-1.34831	-1.0382	-0.65915	-0.31606	-0.0675	-0.0675
50nM+NA	-1.4305	-1.4305	-1.36692	-1.04454	-0.6246	-0.5046	-0.5046
5nM+NA	-0.6227	-0.6227	-0.56404	-0.40327	0.285436	0.439462	0.439462
+NA	-4.78182	-4.78182	-2.40064	-1.40548	-0.63179	-0.45993	-0.45993

Oneway Anova

Summary of Fit

Rsquare	0.800414
Adj Rsquare	0.76715
Root Mean Square Error	0.752371
Mean of Response	2.78e-10
Observations (or Sum Wgts)	36

Analysis of Variance

Source	DF	Sum of Squares	Mean Square	F Ratio	Prob > F
Label	5	68.103497	13.6207	24.0622	<.0001 *
Error	30	16.981857	0.5661		
C. Total	35	85.085355			

Means for Oneway Anova

Level	Number	Mean	Std Error	Lower 95%	Upper 95%
50nM-NA	6	2.1911	0.30715	1.564	2.818
5nM-NA	6	1.4341	0.30715	0.807	2.061
-NA	6	-0.6780	0.30715	-1.305	-0.051
50nM+NA	6	-1.0058	0.30715	-1.633	-0.378
5nM+NA	6	-0.2167	0.30715	-0.844	0.411
+NA	6	-1.7248	0.30715	-2.352	-1.097

Std Error uses a pooled estimate of error variance

Means Comparisons

Comparisons with a control using Dunnett's Method

Oneway Analysis of Pentadecanal By Label

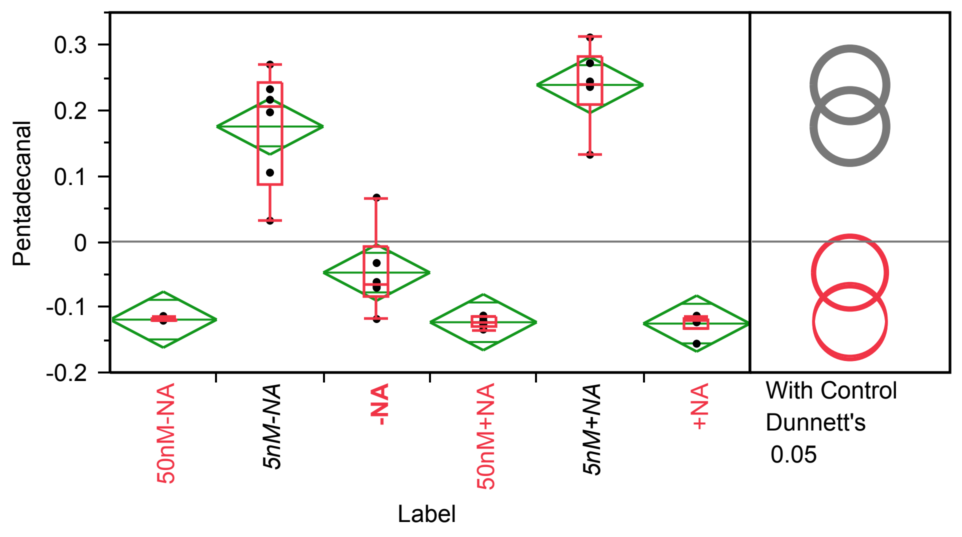

Quantiles

Level	Minimum	10%	25%	Median	75%	90%	Maximum
50nM-NA	-0.12177	-0.12177	-0.12141	-0.12011	-0.11722	-0.11374	-0.11374
5nM-NA	0.031972	0.031972	0.086976	0.20711	0.242158	0.270371	0.270371
-NA	-0.1183	-0.1183	-0.08247	-0.06497	-0.00777	0.067149	0.067149
50nM+NA	-0.13441	-0.13441	-0.12962	-0.12384	-0.11535	-0.11311	-0.11311
5nM+NA	0.132684	0.132684	0.210313	0.241399	0.282413	0.311855	0.311855
+NA	-0.15603	-0.15603	-0.13148	-0.12012	-0.11743	-0.11328	-0.11328

Oneway Anova

Summary of Fit

Rsquare	0.911598
Adj Rsquare	0.896865
Root Mean Square Error	0.051269
Mean of Response	8.33e-10
Observations (or Sum Wgts)	36

Analysis of Variance

Source	DF	Sum of Squares	Mean Square	F Ratio	Prob > F
Label	5	0.81315880	0.162632	61.8721	<.0001 *
Error	30	0.07885544	0.002629		
C. Total	35	0.89201424			

Means for Oneway Anova

Level	Number	Mean	Std Error	Lower 95%	Upper 95%
50nM-NA	6	-0.11923	0.02093	-0.1620	-0.0765
5nM-NA	6	0.17577	0.02093	0.1330	0.2185
-NA	6	-0.04740	0.02093	-0.0901	-0.0046
50nM+NA	6	-0.12322	0.02093	-0.1660	-0.0805
5nM+NA	6	0.23935	0.02093	0.1966	0.2821
+NA	6	-0.12528	0.02093	-0.1680	-0.0825

Std Error uses a pooled estimate of error variance

Means Comparisons

Comparisons with a control using Dunnett's Method

Oneway Analysis of Phenacylamine By Label

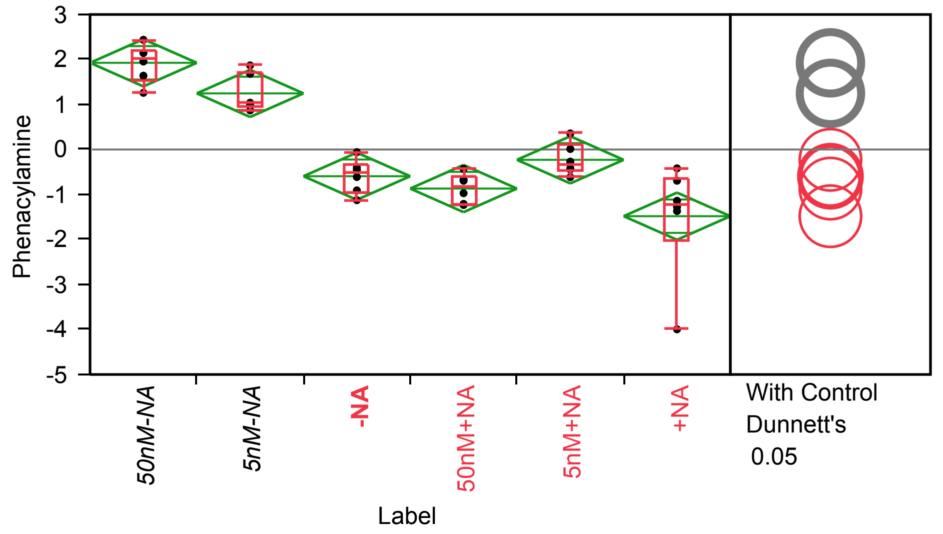

Quantiles

Level	Minimum	10%	25%	Median	75%	90%	Maximum
50nM-NA	1.259613	1.259613	1.544547	2.039386	2.216602	2.437282	2.437282
5nM-NA	0.870145	0.870145	0.959933	1.036145	1.728381	1.869342	1.869342
-NA	-1.12532	-1.12532	-0.96304	-0.53195	-0.31932	-0.05862	-0.05862
50nM+NA	-1.23335	-1.23335	-1.2152	-0.83279	-0.61009	-0.43065	-0.43065
5nM+NA	-0.61321	-0.61321	-0.48669	-0.34355	0.097768	0.356673	0.356673
+NA	-3.99524	-3.99524	-2.02473	-1.20853	-0.62507	-0.41898	-0.41898

Oneway Anova

Summary of Fit

Rsquare	0.813449
Adj Rsquare	0.782357
Root Mean Square Error	0.628859
Mean of Response	8.33e-10
Observations (or Sum Wgts)	36

Analysis of Variance

Source	DF	Sum of Squares	Mean Square	F Ratio	Prob > F
Label	5	51.732196	10.3464	26.1628	<.0001 *
Error	30	11.863918	0.3955		
C. Total	35	63.596113			

Means for Oneway Anova

Level	Number	Mean	Std Error	Lower 95%	Upper 95%
50nM-NA	6	1.9264	0.25673	1.402	2.451
5nM-NA	6	1.2472	0.25673	0.723	1.771
-NA	6	-0.5938	0.25673	-1.118	-0.070
50nM+NA	6	-0.8681	0.25673	-1.392	-0.344
5nM+NA	6	-0.2294	0.25673	-0.754	0.295
+NA	6	-1.4822	0.25673	-2.006	-0.958

Std Error uses a pooled estimate of error variance

Means Comparisons

Comparisons with a control using Dunnett's Method

Oneway Analysis of phenylalanine By Label

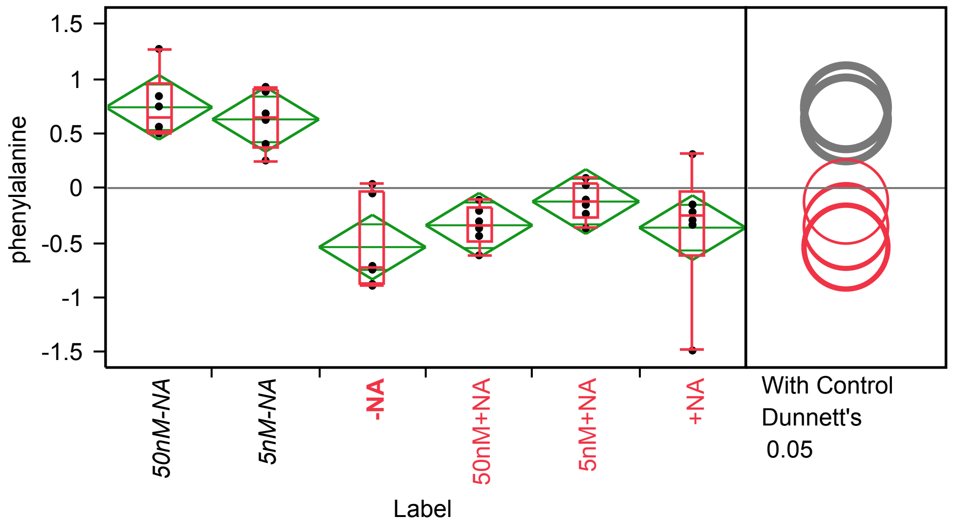

Quantiles

Level	Minimum	10%	25%	Median	75%	90%	Maximum
50nM-NA	0.500378	0.500378	0.508715	0.652833	0.947847	1.271129	1.271129
5nM-NA	0.251728	0.251728	0.365185	0.65224	0.892013	0.924404	0.924404
-NA	-0.89259	-0.89259	-0.88039	-0.72871	-0.02738	0.037103	0.037103
50nM+NA	-0.61485	-0.61485	-0.48216	-0.33609	-0.18311	-0.10805	-0.10805
5nM+NA	-0.36691	-0.36691	-0.2684	-0.12936	0.043246	0.092094	0.092094
+NA	-1.48454	-1.48454	-0.62312	-0.25547	-0.0354	0.309653	0.309653

Oneway Anova

Summary of Fit

Rsquare	0.704907
Adj Rsquare	0.655725
Root Mean Square Error	0.353427
Mean of Response	2.78e-10
Observations (or Sum Wgts)	36

Analysis of Variance

Source	DF	Sum of Squares	Mean Square	F Ratio	Prob > F
Label	5	8.951437	1.79029	14.3326	<.0001 *
Error	30	3.747316	0.12491		
C. Total	35	12.698753			

Means for Oneway Anova

Level	Number	Mean	Std Error	Lower 95%	Upper 95%
50nM-NA	6	0.73813	0.14429	0.4435	1.033
5nM-NA	6	0.62747	0.14429	0.3328	0.922
-NA	6	-0.53968	0.14429	-0.8344	-0.245
50nM+NA	6	-0.34019	0.14429	-0.6349	-0.046
5nM+NA	6	-0.12369	0.14429	-0.4184	0.171
+NA	6	-0.36204	0.14429	-0.6567	-0.067

Std Error uses a pooled estimate of error variance

Means Comparisons

Comparisons with a control using Dunnett's Method

Oneway Analysis of Phenyllactic acid By Label

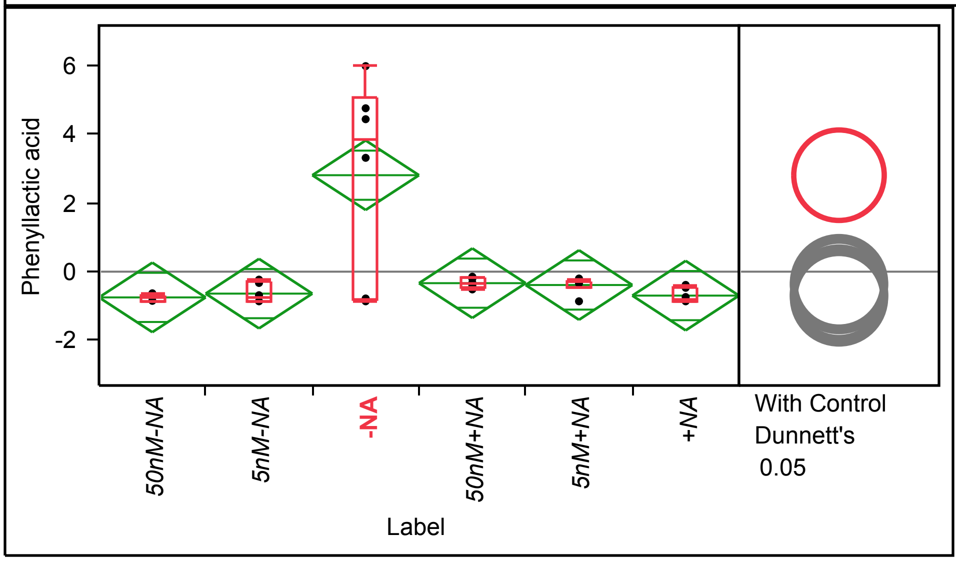

Quantiles

Level	Minimum	10%	25%	Median	75%	90%	Maximum
50nM-NA	-0.86261	-0.86261	-0.85247	-0.73173	-0.67494	-0.62955	-0.62955
5nM-NA	-0.86262	-0.86262	-0.86262	-0.77422	-0.30419	-0.23218	-0.23218
-NA	-0.86262	-0.86262	-0.80119	3.876452	5.063591	5.983731	5.983731
50nM+NA	-0.51607	-0.51607	-0.43693	-0.36416	-0.19107	-0.14746	-0.14746
5nM+NA	-0.86263	-0.86263	-0.47141	-0.31253	-0.27755	-0.19951	-0.19951
+NA	-0.86263	-0.86263	-0.86263	-0.80039	-0.44881	-0.3857	-0.3857

Oneway Anova

Summary of Fit

Rsquare	0.565858
Adj Rsquare	0.493501
Root Mean Square Error	1.214032
Mean of Response	-2.78e-9
Observations (or Sum Wgts)	36

Analysis of Variance

Source	DF	Sum of Squares	Mean Square	F Ratio	Prob > F
Label	5	57.63107	11.5262	7.8204	<.0001 *
Error	30	44.21620	1.4739		
C. Total	35	101.84727			

Means for Oneway Anova

Level	Number	Mean	Std Error	Lower 95%	Upper 95%
50nM-NA	6	-0.7491	0.49563	-1.761	0.2631
5nM-NA	6	-0.6390	0.49563	-1.651	0.3732
-NA	6	2.8084	0.49563	1.796	3.8206
50nM+NA	6	-0.3347	0.49563	-1.347	0.6775
5nM+NA	6	-0.3886	0.49563	-1.401	0.6236
+NA	6	-0.6969	0.49563	-1.709	0.3153

Std Error uses a pooled estimate of error variance

Means Comparisons

Comparisons with a control using Dunnett's Method

Oneway Analysis of Phosphate By Label

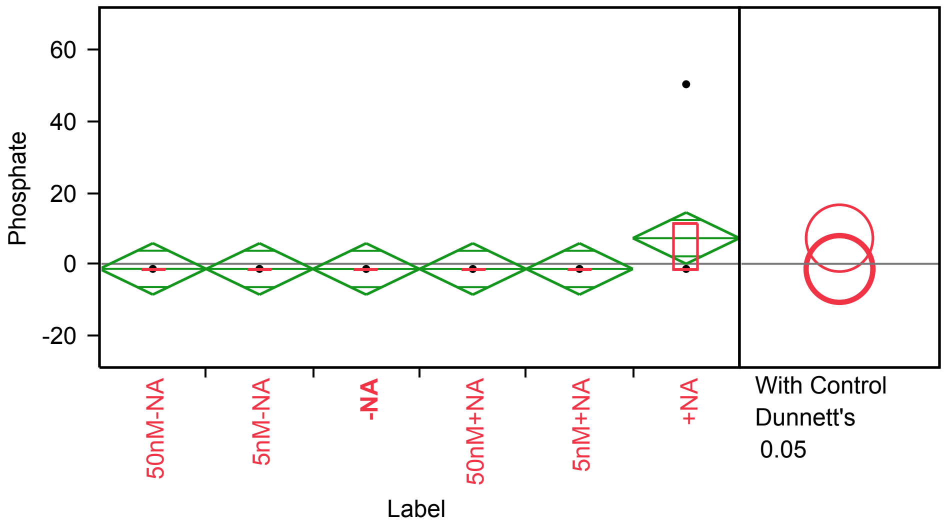

Quantiles

Level	Minimum	10%	25%	Median	75%	90%	Maximum
50nM-NA	-1.4391	-1.4391	-1.43009	-1.42512	-1.42045	-1.41872	-1.41872
5nM-NA	-1.46884	-1.46884	-1.45524	-1.43529	-1.42503	-1.42415	-1.42415
-NA	-1.46666	-1.46666	-1.46632	-1.45144	-1.44012	-1.43786	-1.43786
50nM+NA	-1.45565	-1.45565	-1.45359	-1.45148	-1.44591	-1.44105	-1.44105
5nM+NA	-1.45015	-1.45015	-1.44958	-1.44476	-1.437	-1.43282	-1.43282
+NA	-1.44658	-1.44658	-1.44622	-1.4399	11.54309	50.47844	50.47844

Oneway Anova

Summary of Fit

Rsquare	0.14288
Adj Rsquare	2.673e-5
Root Mean Square Error	8.65334
Mean of Response	-2e-16
Observations (or Sum Wgts)	36

Analysis of Variance

Source	DF	Sum of Squares	Mean Square	F Ratio	Prob > F
Label	5	374.4715	74.8943	1.0002	0.4345
Error	30	2246.4088	74.8803		
C. Total	35	2620.8804			

Means for Oneway Anova

Level	Number	Mean	Std Error	Lower 95%	Upper 95%
50nM-NA	6	-1.4260	3.5327	-8.641	5.789
5nM-NA	6	-1.4399	3.5327	-8.655	5.775
-NA	6	-1.4524	3.5327	-8.667	5.762
50nM+NA	6	-1.4500	3.5327	-8.665	5.765
5nM+NA	6	-1.4434	3.5327	-8.658	5.771
+NA	6	7.2118	3.5327	-0.003	14.427

Std Error uses a pooled estimate of error variance

Means Comparisons

Comparisons with a control using Dunnett's Method

Oneway Analysis of Phosphatidylglycerol By Label

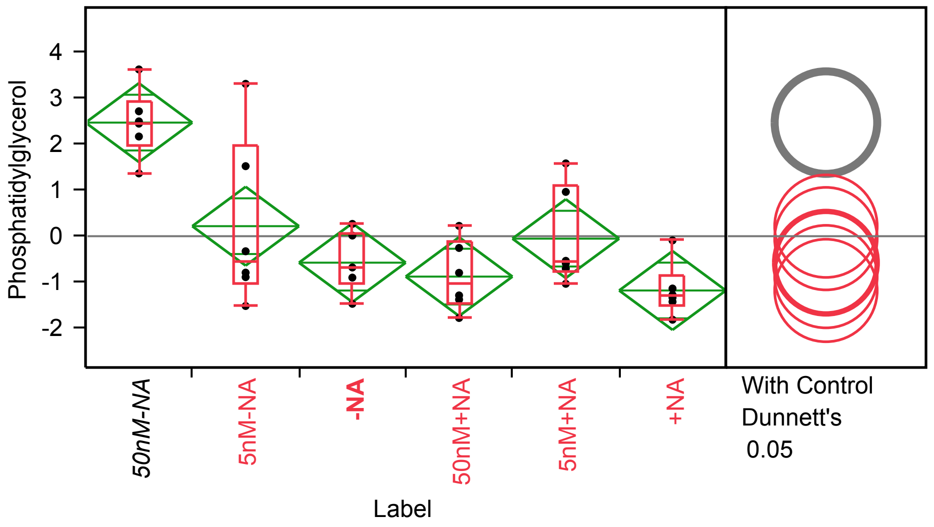

Quantiles

Level	Minimum	10%	25%	Median	75%	90%	Maximum
50nM-NA	1.364098	1.364098	1.964677	2.468859	2.939957	3.621591	3.621591
5nM-NA	-1.51534	-1.51534	-1.04553	-0.55927	1.967811	3.308483	3.308483
-NA	-1.46828	-1.46828	-1.04192	-0.68097	0.077199	0.266619	0.266619
50nM+NA	-1.77567	-1.77567	-1.4763	-1.04089	-0.13543	0.223889	0.223889
5nM+NA	-1.03344	-1.03344	-0.78608	-0.56236	1.116742	1.580587	1.580587
+NA	-1.81343	-1.81343	-1.52136	-1.30432	-0.87515	-0.09147	-0.09147

Oneway Anova

Summary of Fit

Rsquare	0.620965
Adj Rsquare	0.557792
Root Mean Square Error	1.026194
Mean of Response	-8.3e-10
Observations (or Sum Wgts)	36

Analysis of Variance

Source	DF	Sum of Squares	Mean Square	F Ratio	Prob > F
Label	5	51.756806	10.3514	9.8297	<.0001 *
Error	30	31.592225	1.0531		
C. Total	35	83.349031			

Means for Oneway Anova

Level	Number	Mean	Std Error	Lower 95%	Upper 95%
50nM-NA	6	2.4668	0.41894	1.611	3.322
5nM-NA	6	0.2178	0.41894	-0.638	1.073
-NA	6	-0.5749	0.41894	-1.430	0.281
50nM+NA	6	-0.8775	0.41894	-1.733	-0.022
5nM+NA	6	-0.0532	0.41894	-0.909	0.802
+NA	6	-1.1790	0.41894	-2.035	-0.323

Std Error uses a pooled estimate of error variance

Means Comparisons

Comparisons with a control using Dunnett's Method

Oneway Analysis of phosphoenolpyruvate By Label

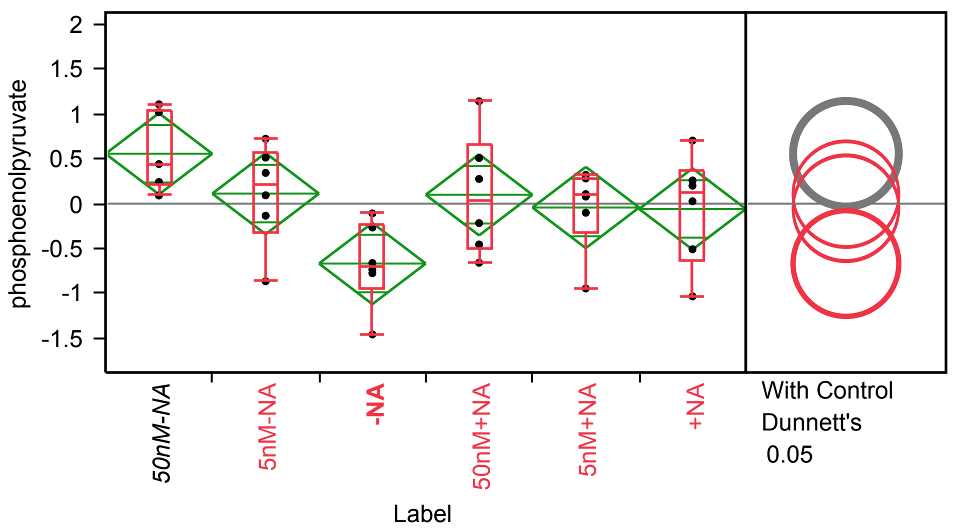

Quantiles

Level	Minimum	10%	25%	Median	75%	90%	Maximum
50nM-NA	0.091798	0.091798	0.205212	0.439536	1.04277	1.108814	1.108814
5nM-NA	-0.86571	-0.86571	-0.31723	0.218704	0.566813	0.724108	0.724108
-NA	-1.45867	-1.45867	-0.94617	-0.69956	-0.22598	-0.10845	-0.10845
50nM+NA	-0.65918	-0.65918	-0.50651	0.029693	0.66748	1.142844	1.142844
5nM+NA	-0.94562	-0.94562	-0.31186	0.093095	0.291533	0.321844	0.321844
+NA	-1.03391	-1.03391	-0.63999	0.113752	0.370229	0.703852	0.703852

Oneway Anova

Summary of Fit

Rsquare	0.346842
Adj Rsquare	0.237982
Root Mean Square Error	0.543501
Mean of Response	5.56e-10
Observations (or Sum Wgts)	36

Analysis of Variance

Source	DF	Sum of Squares	Mean Square	F Ratio	Prob > F
Label	5	4.705828	0.941166	3.1861	0.0200 *
Error	30	8.861810	0.295394		
C. Total	35	13.567639			

Means for Oneway Anova

Level	Number	Mean	Std Error	Lower 95%	Upper 95%
50nM-NA	6	0.55724	0.22188	0.104	1.010
5nM-NA	6	0.11263	0.22188	-0.341	0.566
-NA	6	-0.66779	0.22188	-1.121	-0.215
50nM+NA	6	0.09941	0.22188	-0.354	0.553
5nM+NA	6	-0.04279	0.22188	-0.496	0.410
+NA	6	-0.05870	0.22188	-0.512	0.394

Std Error uses a pooled estimate of error variance

Means Comparisons

Comparisons with a control using Dunnett's Method

Oneway Analysis of Phosphorylcholine By Label

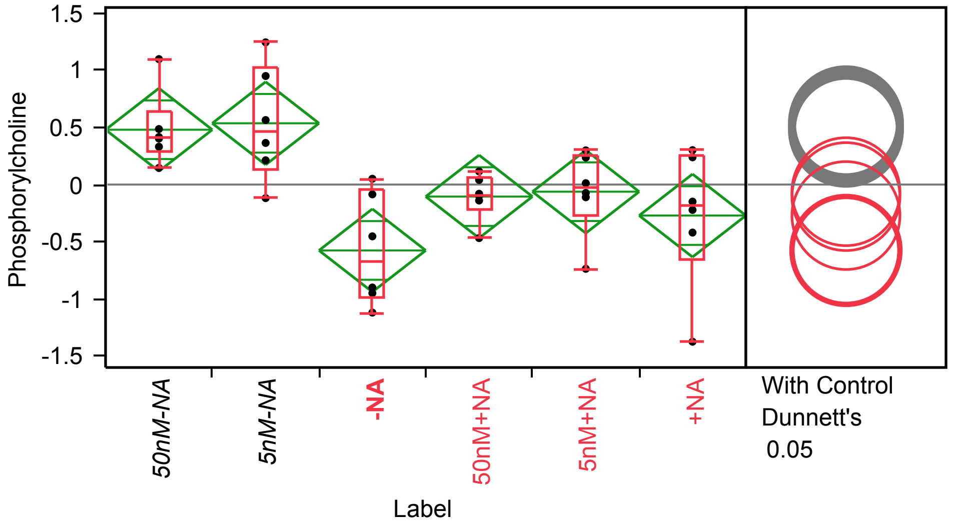

Quantiles

Level	Minimum	10%	25%	Median	75%	90%	Maximum
50nM-NA	0.145823	0.145823	0.284843	0.409227	0.638926	1.098043	1.098043
5nM-NA	-0.11878	-0.11878	0.12957	0.464459	1.0239	1.244937	1.244937
-NA	-1.12087	-1.12087	-0.99318	-0.67735	-0.05156	0.050722	0.050722
50nM+NA	-0.46869	-0.46869	-0.22202	-0.08954	0.058848	0.115141	0.115141
5nM+NA	-0.73752	-0.73752	-0.26787	-0.03085	0.252016	0.29926	0.29926
+NA	-1.37581	-1.37581	-0.65933	-0.18592	0.254196	0.301014	0.301014

Oneway Anova

Summary of Fit

Rsquare	0.497634
Adj Rsquare	0.413906
Root Mean Square Error	0.435493
Mean of Response	-2.8e-10
Observations (or Sum Wgts)	36

Analysis of Variance

Source	DF	Sum of Squares	Mean Square	F Ratio	Prob > F
Label	5	5.636041	1.12721	5.9435	0.0006 *
Error	30	5.689634	0.18965		
C. Total	35	11.325675			

Means for Oneway Anova

Level	Number	Mean	Std Error	Lower 95%	Upper 95%
50nM-NA	6	0.47990	0.17779	0.1168	0.8430
5nM-NA	6	0.53627	0.17779	0.1732	0.8994
-NA	6	-0.57685	0.17779	-0.9399	-0.2138
50nM+NA	6	-0.10539	0.17779	-0.4685	0.2577
5nM+NA	6	-0.06250	0.17779	-0.4256	0.3006
+NA	6	-0.27143	0.17779	-0.6345	0.0917

Std Error uses a pooled estimate of error variance

Means Comparisons

Comparisons with a control using Dunnett's Method

Oneway Analysis of p-hydroxybenzoate By Label

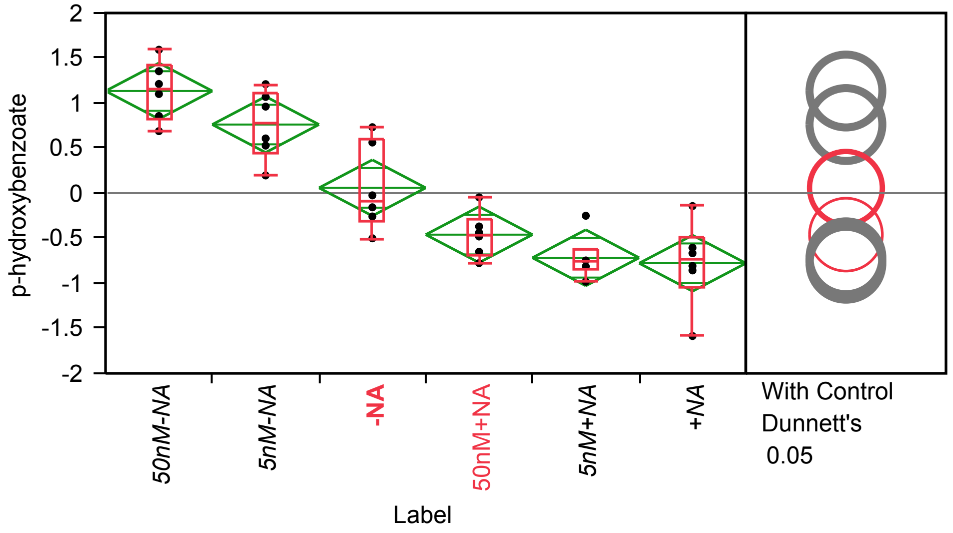

Quantiles

Level	Minimum	10%	25%	Median	75%	90%	Maximum
50nM-NA	0.688577	0.688577	0.814607	1.156671	1.413307	1.59205	1.59205
5nM-NA	0.197021	0.197021	0.445404	0.784159	1.103952	1.209972	1.209972
-NA	-0.50085	-0.50085	-0.31924	-0.09062	0.605008	0.73207	0.73207
50nM+NA	-0.77617	-0.77617	-0.68194	-0.45932	-0.28892	-0.04655	-0.04655
5nM+NA	-0.98226	-0.98226	-0.85538	-0.75473	-0.62299	-0.25077	-0.25077
+NA	-1.58548	-1.58548	-1.03932	-0.73743	-0.48979	-0.14318	-0.14318

Oneway Anova

Summary of Fit

Rsquare	0.821664
Adj Rsquare	0.791941
Root Mean Square Error	0.372768
Mean of Response	2.78e-10
Observations (or Sum Wgts)	36

Analysis of Variance

Source	DF	Sum of Squares	Mean Square	F Ratio	Prob > F
Label	5	19.206665	3.84133	27.6443	<.0001 *
Error	30	4.168675	0.13896		
C. Total	35	23.375341			

Means for Oneway Anova

Level	Number	Mean	Std Error	Lower 95%	Upper 95%
50nM-NA	6	1.1341	0.15218	0.823	1.445
5nM-NA	6	0.7620	0.15218	0.451	1.073
-NA	6	0.0590	0.15218	-0.252	0.370
50nM+NA	6	-0.4603	0.15218	-0.771	-0.149
5nM+NA	6	-0.7171	0.15218	-1.028	-0.406
+NA	6	-0.7777	0.15218	-1.088	-0.467

Std Error uses a pooled estimate of error variance

Means Comparisons

Comparisons with a control using Dunnett's Method

Oneway Analysis of PI(16:0/20:2) By Label

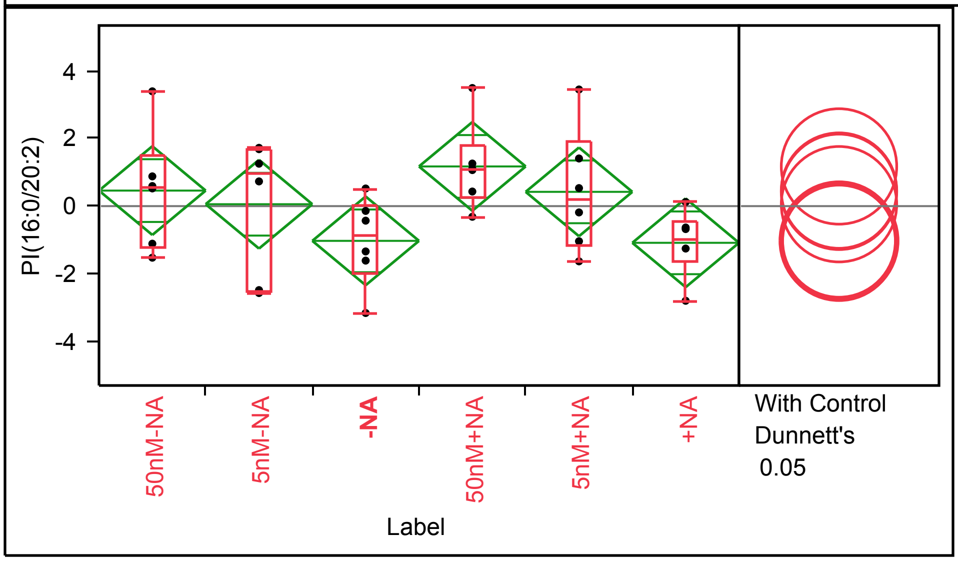

Quantiles

Level	Minimum	10%	25%	Median	75%	90%	Maximum
50nM-NA	-1.52238	-1.52238	-1.21273	0.557908	1.506337	3.389473	3.389473
5nM-NA	-2.56583	-2.56583	-2.50139	0.991304	1.691559	1.721066	1.721066
-NA	-3.1556	-3.1556	-1.99551	-0.88355	0.024061	0.520259	0.520259
50nM+NA	-0.31419	-0.31419	0.244461	1.084895	1.814674	3.48703	3.48703
5nM+NA	-1.62984	-1.62984	-1.18487	0.172959	1.916015	3.443508	3.443508
+NA	-2.7943	-2.7943	-1.64617	-0.96499	-0.4439	0.120164	0.120164

Oneway Anova

Summary of Fit

Rsquare	0.244291
Adj Rsquare	0.11834
Root Mean Square Error	1.571043
Mean of Response	-4.9e-17
Observations (or Sum Wgts)	36

Analysis of Variance

Source	DF	Sum of Squares	Mean Square	F Ratio	Prob > F
Label	5	23.935938	4.78719	1.9396	0.1171
Error	30	74.045255	2.46818		
C. Total	35	97.981193			

Means for Oneway Anova

Level	Number	Mean	Std Error	Lower 95%	Upper 95%
50nM-NA	6	0.4587	0.64138	-0.851	1.7685
5nM-NA	6	0.0566	0.64138	-1.253	1.3665
-NA	6	-1.0254	0.64138	-2.335	0.2844
50nM+NA	6	1.1718	0.64138	-0.138	2.4816
5nM+NA	6	0.4216	0.64138	-0.888	1.7315
+NA	6	-1.0833	0.64138	-2.393	0.2266

Std Error uses a pooled estimate of error variance

Means Comparisons

Comparisons with a control using Dunnett's Method

Oneway Analysis of PI(16:0/22:4) By Label

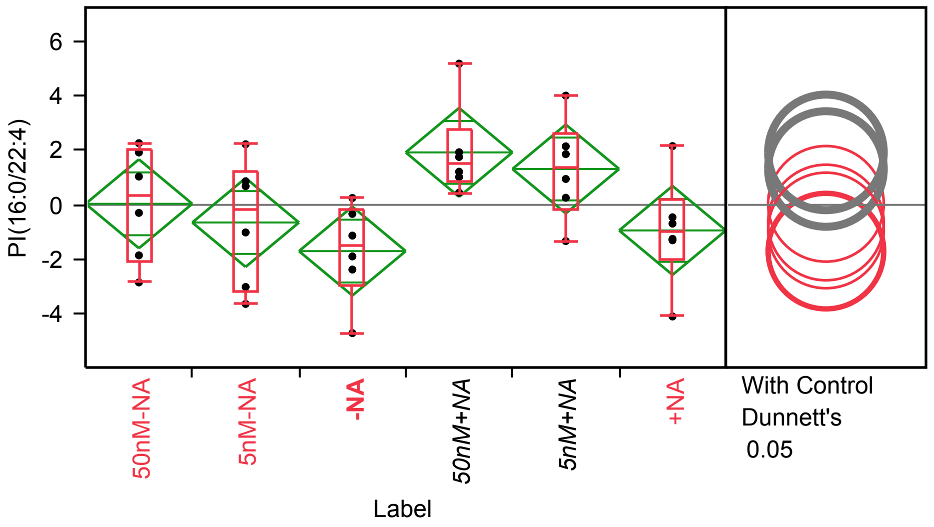

Quantiles

Level	Minimum	10%	25%	Median	75%	90%	Maximum
50nM-NA	-2.83733	-2.83733	-2.09515	0.371668	2.001726	2.257257	2.257257
5nM-NA	-3.6238	-3.6238	-3.15839	-0.16177	1.201808	2.217715	2.217715
-NA	-4.69284	-4.69284	-2.94823	-1.50585	-0.18433	0.251217	0.251217
50nM+NA	0.44537	0.44537	0.875207	1.482084	2.736117	5.167785	5.167785
5nM+NA	-1.32404	-1.32404	-0.13621	1.402335	2.605522	3.996665	3.996665
+NA	-4.08301	-4.08301	-1.99208	-0.96557	0.196928	2.150489	2.150489

Oneway Anova

Summary of Fit

Rsquare	0.334797
Adj Rsquare	0.223929
Root Mean Square Error	1.949667
Mean of Response	-7.4e-17
Observations (or Sum Wgts)	36

Analysis of Variance

Source	DF	Sum of Squares	Mean Square	F Ratio	Prob > F
Label	5	57.39428	11.4789	3.0198	0.0252 *
Error	30	114.03604	3.8012		
C. Total	35	171.43032			

Means for Oneway Anova

Level	Number	Mean	Std Error	Lower 95%	Upper 95%
50nM-NA	6	0.0387	0.79595	-1.587	1.664
5nM-NA	6	-0.6450	0.79595	-2.270	0.981
-NA	6	-1.6916	0.79595	-3.317	-0.066
50nM+NA	6	1.9202	0.79595	0.295	3.546
5nM+NA	6	1.3131	0.79595	-0.312	2.939
+NA	6	-0.9355	0.79595	-2.561	0.690

Std Error uses a pooled estimate of error variance

Means Comparisons

Comparisons with a control using Dunnett's Method

Oneway Analysis of PI(16:1/22:4) By Label

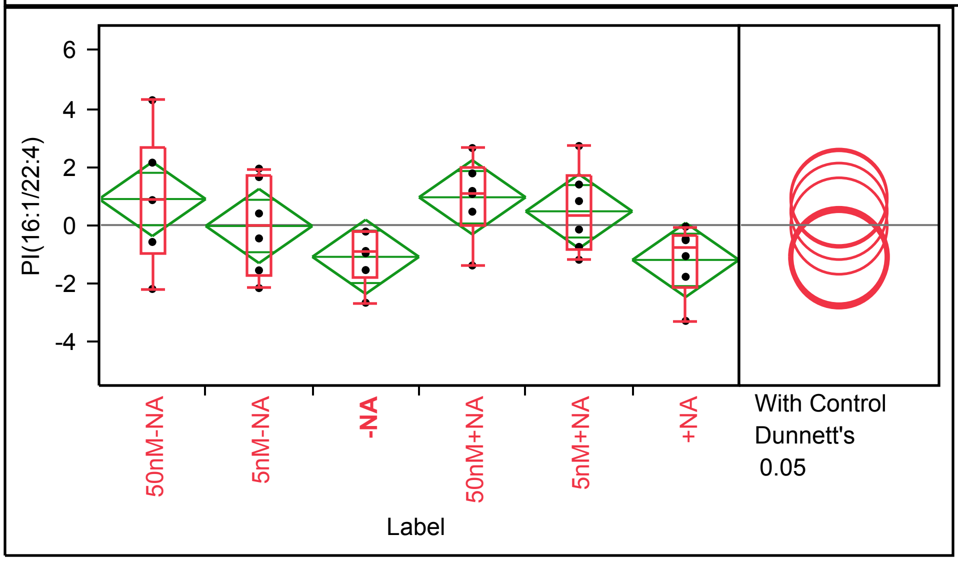

Quantiles

Level	Minimum	10%	25%	Median	75%	90%	Maximum
50nM-NA	-2.19382	-2.19382	-0.98366	0.853232	2.678389	4.286351	4.286351
5nM-NA	-2.16334	-2.16334	-1.70677	-0.02982	1.719236	1.936225	1.936225
-NA	-2.66615	-2.66615	-1.82529	-0.92695	-0.24766	-0.22351	-0.22351
50nM+NA	-1.38756	-1.38756	-0.00321	1.117559	1.991125	2.643096	2.643096
5nM+NA	-1.19089	-1.19089	-0.86002	0.334954	1.722166	2.713754	2.713754
+NA	-3.28857	-3.28857	-2.15364	-0.79221	-0.3708	-0.05564	-0.05564

Oneway Anova

Summary of Fit

Rsquare	0.281161
Adj Rsquare	0.161355
Root Mean Square Error	1.525969
Mean of Response	5.556e-9
Observations (or Sum Wgts)	36

Analysis of Variance

Source	DF	Sum of Squares	Mean Square	F Ratio	Prob > F
Label	5	27.323510	5.46470	2.3468	0.0653
Error	30	69.857467	2.32858		
C. Total	35	97.180977			

Means for Oneway Anova

Level	Number	Mean	Std Error	Lower 95%	Upper 95%
50nM-NA	6	0.8935	0.62297	-0.379	2.1658
5nM-NA	6	-0.0324	0.62297	-1.305	1.2399
-NA	6	-1.0907	0.62297	-2.363	0.1816
50nM+NA	6	0.9538	0.62297	-0.319	2.2261
5nM+NA	6	0.4724	0.62297	-0.800	1.7447
+NA	6	-1.1966	0.62297	-2.469	0.0756

Std Error uses a pooled estimate of error variance

Means Comparisons

Comparisons with a control using Dunnett's Method

Oneway Analysis of Piperidine By Label

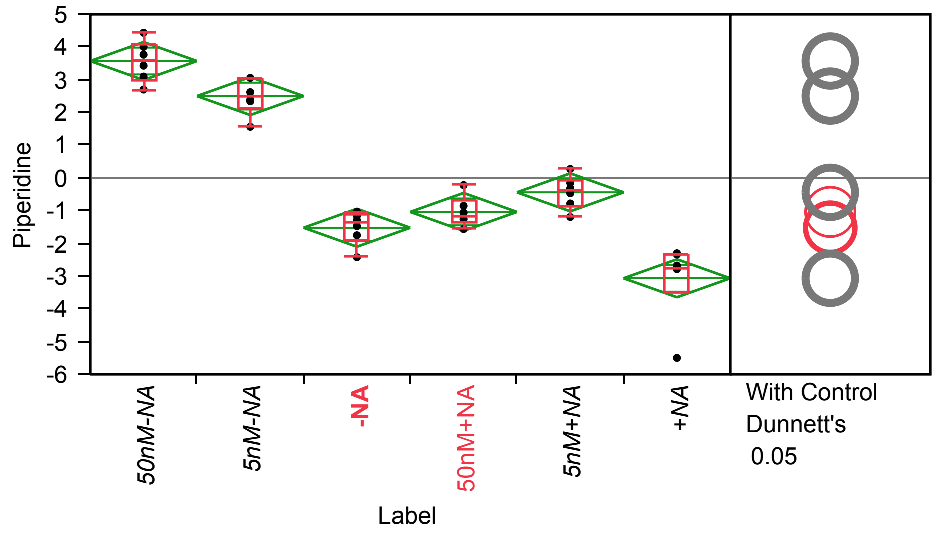

Quantiles

Level	Minimum	10%	25%	Median	75%	90%	Maximum
50nM-NA	2.700234	2.700234	2.997012	3.596998	4.110381	4.433493	4.433493
5nM-NA	1.561405	1.561405	2.146471	2.50338	3.040403	3.054542	3.054542
-NA	-2.4241	-2.4241	-1.92091	-1.38527	-1.12663	-1.03309	-1.03309
50nM+NA	-1.5633	-1.5633	-1.34529	-1.16362	-0.69635	-0.22045	-0.22045
5nM+NA	-1.19426	-1.19426	-0.88507	-0.39311	-0.04942	0.27164	0.27164
+NA	-5.50072	-5.50072	-3.46759	-2.73349	-2.33144	-2.30319	-2.30319

Oneway Anova

Summary of Fit

Rsquare	0.929776
Adj Rsquare	0.918072
Root Mean Square Error	0.695298
Mean of Response	2.778e-9
Observations (or Sum Wgts)	36

Analysis of Variance

Source	DF	Sum of Squares	Mean Square	F Ratio	Prob > F
Label	5	192.02484	38.4050	79.4410	<.0001 *
Error	30	14.50320	0.4834		
C. Total	35	206.52803			

Means for Oneway Anova

Level	Number	Mean	Std Error	Lower 95%	Upper 95%
50nM-NA	6	3.5711	0.28385	2.991	4.151
5nM-NA	6	2.5000	0.28385	1.920	3.080
-NA	6	-1.5231	0.28385	-2.103	-0.943
50nM+NA	6	-1.0398	0.28385	-1.619	-0.460
5nM+NA	6	-0.4412	0.28385	-1.021	0.138
+NA	6	-3.0669	0.28385	-3.647	-2.487

Std Error uses a pooled estimate of error variance

Means Comparisons

Comparisons with a control using Dunnett's Method

Oneway Analysis of proline By Label

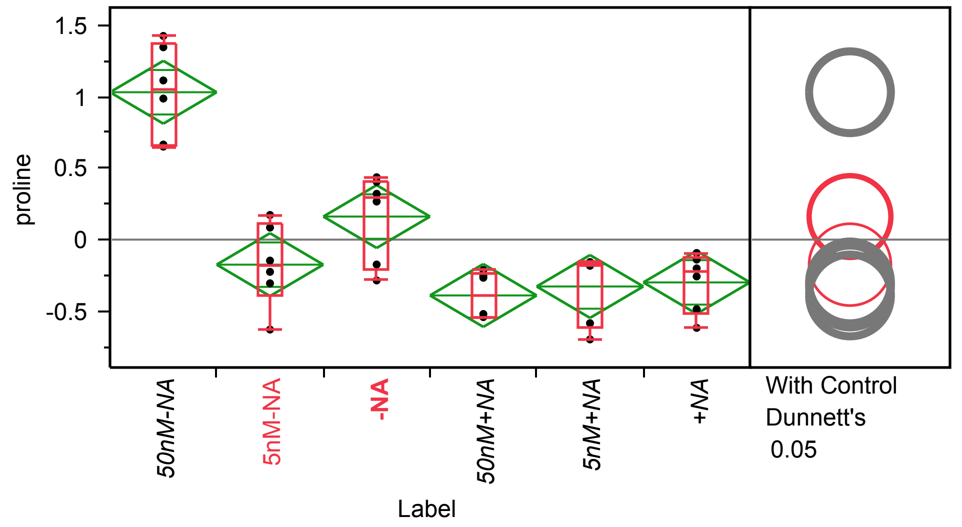

Quantiles

Level	Minimum	10%	25%	Median	75%	90%	Maximum
50nM-NA	0.651009	0.651009	0.661819	1.050886	1.36648	1.425785	1.425785
5nM-NA	-0.62946	-0.62946	-0.38672	-0.18628	0.106265	0.17257	0.17257
-NA	-0.2856	-0.2856	-0.20218	0.293765	0.412809	0.436724	0.436724
50nM+NA	-0.54786	-0.54786	-0.54357	-0.39383	-0.24365	-0.21278	-0.21278
5nM+NA	-0.69834	-0.69834	-0.61351	-0.17871	-0.16665	-0.15695	-0.15695
+NA	-0.61757	-0.61757	-0.51971	-0.2294	-0.12802	-0.09356	-0.09356

Oneway Anova

Summary of Fit

Rsquare	0.808585
Adj Rsquare	0.776683
Root Mean Square Error	0.263926
Mean of Response	5.56e-10
Observations (or Sum Wgts)	36

Analysis of Variance

Source	DF	Sum of Squares	Mean Square	F Ratio	Prob > F
Label	5	8.827449	1.76549	25.3456	<.0001 *
Error	30	2.089703	0.06966		
C. Total	35	10.917151			

Means for Oneway Anova

Level	Number	Mean	Std Error	Lower 95%	Upper 95%
50nM-NA	6	1.0318	0.10775	0.8117	1.252
5nM-NA	6	-0.1752	0.10775	-0.3952	0.045
-NA	6	0.1615	0.10775	-0.0585	0.382
50nM+NA	6	-0.3907	0.10775	-0.6108	-0.171
5nM+NA	6	-0.3280	0.10775	-0.5480	-0.108
+NA	6	-0.2994	0.10775	-0.5195	-0.079

Std Error uses a pooled estimate of error variance

Means Comparisons

Comparisons with a control using Dunnett's Method

Oneway Analysis of purine By Label

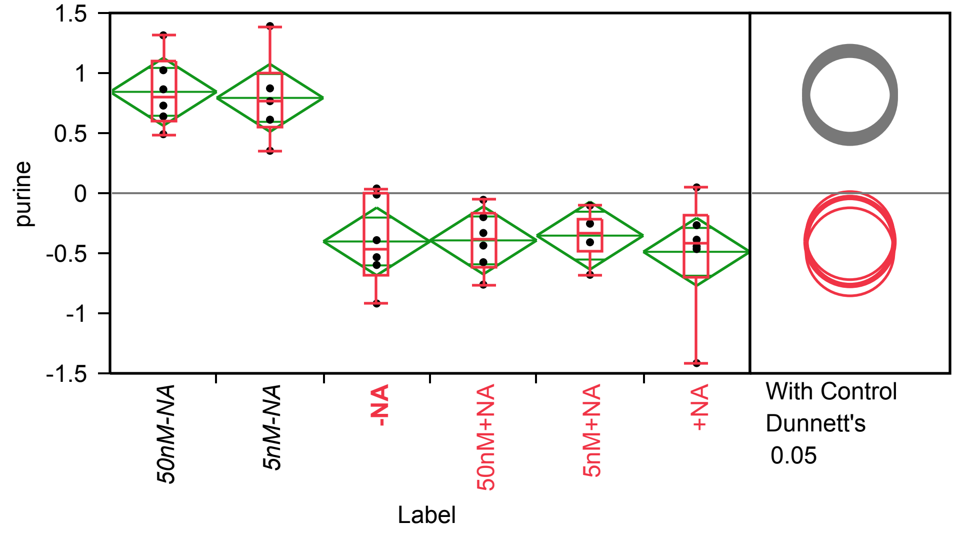

Quantiles

Level	Minimum	10%	25%	Median	75%	90%	Maximum
50nM-NA	0.490956	0.490956	0.60196	0.796907	1.096366	1.314238	1.314238
5nM-NA	0.352961	0.352961	0.54705	0.765187	1.002278	1.391232	1.391232
-NA	-0.91844	-0.91844	-0.67795	-0.46213	0.001149	0.039167	0.039167
50nM+NA	-0.76134	-0.76134	-0.62142	-0.38367	-0.16356	-0.05543	-0.05543
5nM+NA	-0.67949	-0.67949	-0.47966	-0.3351	-0.2163	-0.1035	-0.1035
+NA	-1.41477	-1.41477	-0.70261	-0.41535	-0.18787	0.048027	0.048027

Oneway Anova

Summary of Fit

Rsquare	0.7798
Adj Rsquare	0.7431
Root Mean Square Error	0.337778
Mean of Response	-5.6e-10
Observations (or Sum Wgts)	36

Analysis of Variance

Source	DF	Sum of Squares	Mean Square	F Ratio	Prob > F
Label	5	12.121294	2.42426	21.2479	<.0001 *
Error	30	3.422815	0.11409		
C. Total	35	15.544109			

Means for Oneway Anova

Level	Number	Mean	Std Error	Lower 95%	Upper 95%
50nM-NA	6	0.84362	0.13790	0.5620	1.125
5nM-NA	6	0.79316	0.13790	0.5115	1.075
-NA	6	-0.40214	0.13790	-0.6838	-0.121
50nM+NA	6	-0.39308	0.13790	-0.6747	-0.111
5nM+NA	6	-0.35336	0.13790	-0.6350	-0.072
+NA	6	-0.48820	0.13790	-0.7698	-0.207

Std Error uses a pooled estimate of error variance

Means Comparisons

Comparisons with a control using Dunnett's Method

Oneway Analysis of pyridoxine By Label

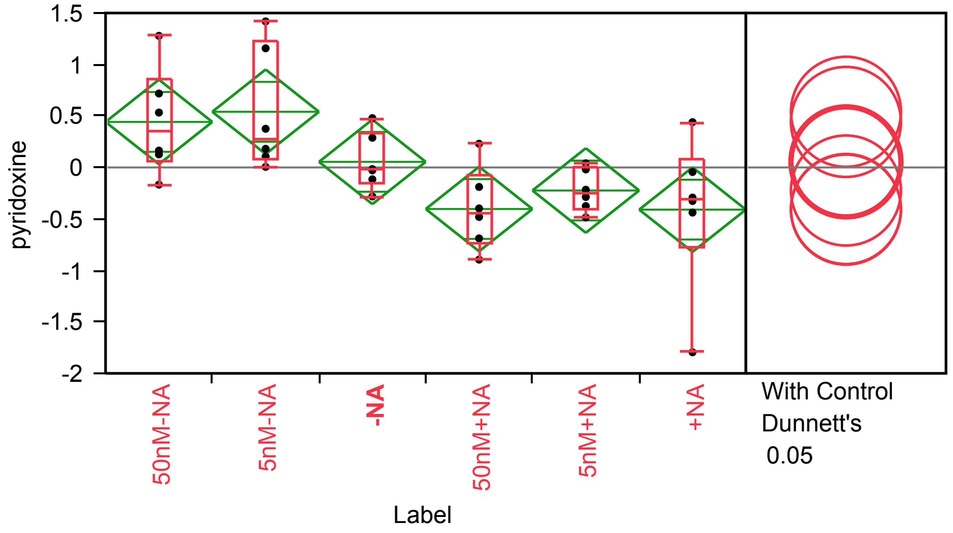

Quantiles

Level	Minimum	10%	25%	Median	75%	90%	Maximum
50nM-NA	-0.1668	-0.1668	0.053242	0.348405	0.858078	1.278137	1.278137
5nM-NA	0.005443	0.005443	0.08152	0.27809	1.222583	1.418447	1.418447
-NA	-0.28162	-0.28162	-0.15697	-0.02208	0.335844	0.478981	0.478981
50nM+NA	-0.89513	-0.89513	-0.7404	-0.43943	-0.08452	0.229885	0.229885
5nM+NA	-0.48533	-0.48533	-0.4027	-0.25186	-0.00459	0.040143	0.040143
+NA	-1.79539	-1.79539	-0.77759	-0.30926	0.076527	0.439601	0.439601

Oneway Anova

Summary of Fit

Rsquare	0.418017
Adj Rsquare	0.32102
Root Mean Square Error	0.49265
Mean of Response	-1.2e-17
Observations (or Sum Wgts)	36

Analysis of Variance

Source	DF	Sum of Squares	Mean Square	F Ratio	Prob > F
Label	5	5.229772	1.04595	4.3096	0.0045 *
Error	30	7.281128	0.24270		
C. Total	35	12.510900			

Means for Oneway Anova

Level	Number	Mean	Std Error	Lower 95%	Upper 95%
50nM-NA	6	0.44213	0.20112	0.0314	0.85288
5nM-NA	6	0.54071	0.20112	0.1300	0.95146
-NA	6	0.05432	0.20112	-0.3564	0.46507
50nM+NA	6	-0.40371	0.20112	-0.8145	0.00704
5nM+NA	6	-0.22393	0.20112	-0.6347	0.18682
+NA	6	-0.40952	0.20112	-0.8203	0.00123

Std Error uses a pooled estimate of error variance

Means Comparisons

Comparisons with a control using Dunnett's Method

Oneway Analysis of Pyroglutamate By Label

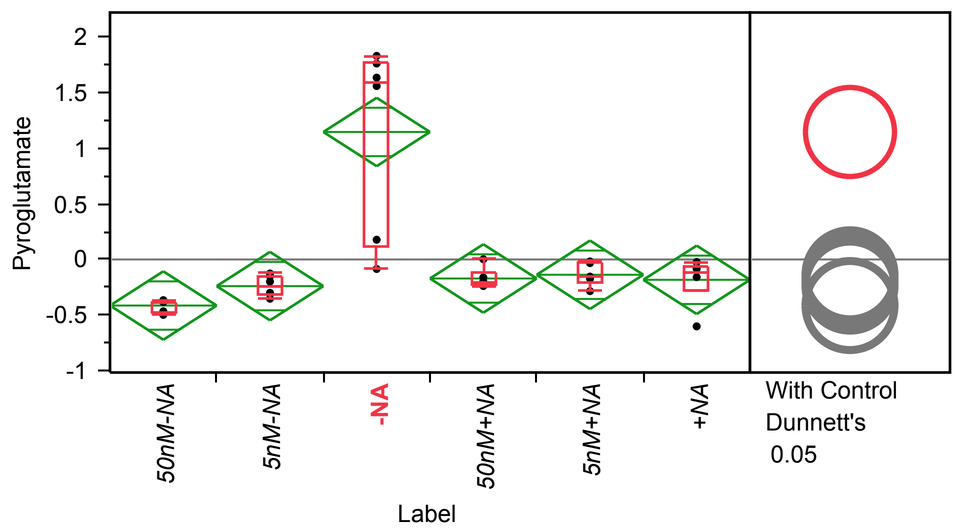

Quantiles

Level	Minimum	10%	25%	Median	75%	90%	Maximum
50nM-NA	-0.49692	-0.49692	-0.47294	-0.3851	-0.37795	-0.36472	-0.36472
5nM-NA	-0.35211	-0.35211	-0.32005	-0.24876	-0.14391	-0.12577	-0.12577
-NA	-0.08537	-0.08537	0.111188	1.592264	1.773535	1.824751	1.824751
50nM+NA	-0.23843	-0.23843	-0.22564	-0.20239	-0.1206	0.001689	0.001689
5nM+NA	-0.28394	-0.28394	-0.20051	-0.15823	-0.029	-0.01792	-0.01792
+NA	-0.59984	-0.59984	-0.26967	-0.12122	-0.05873	-0.02258	-0.02258

Oneway Anova

Summary of Fit

Rsquare	0.704794
Adj Rsquare	0.655593
Root Mean Square Error	0.367854
Mean of Response	-2.8e-10
Observations (or Sum Wgts)	36

Analysis of Variance

Source	DF	Sum of Squares	Mean Square	F Ratio	Prob > F
Label	5	9.691882	1.93838	14.3248	<.0001 *
Error	30	4.059488	0.13532		
C. Total	35	13.751370			

Means for Oneway Anova

Level	Number	Mean	Std Error	Lower 95%	Upper 95%
50nM-NA	6	-0.4132	0.15018	-0.7199	-0.106
5nM-NA	6	-0.2391	0.15018	-0.5458	0.068
-NA	6	1.1428	0.15018	0.8361	1.450
50nM+NA	6	-0.1707	0.15018	-0.4774	0.136
5nM+NA	6	-0.1373	0.15018	-0.4440	0.169
+NA	6	-0.1825	0.15018	-0.4892	0.124

Std Error uses a pooled estimate of error variance

Means Comparisons

Comparisons with a control using Dunnett's Method

Oneway Analysis of pyruvate By Label

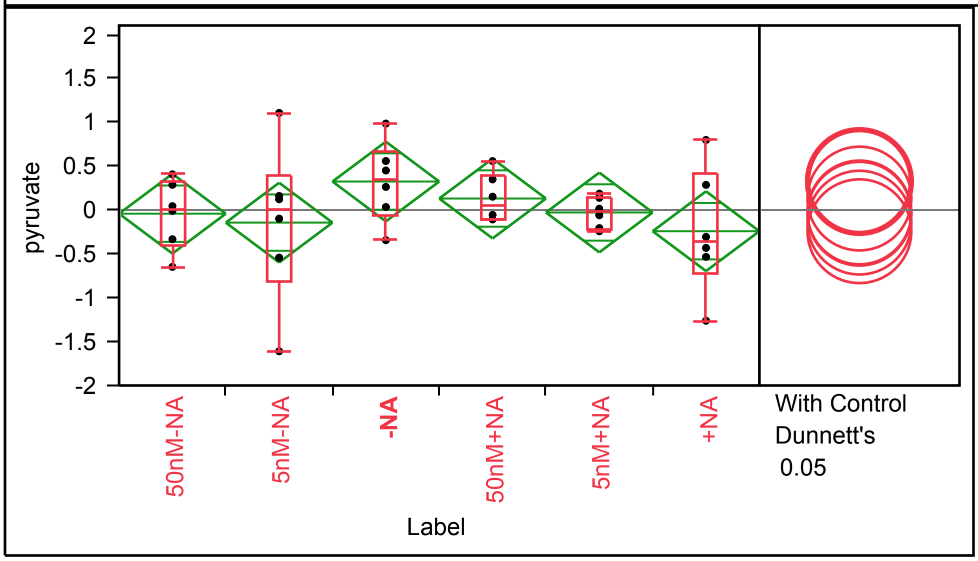

Quantiles

Level	Minimum	10%	25%	Median	75%	90%	Maximum
50nM-NA	-0.64783	-0.64783	-0.41165	0.017192	0.317336	0.406558	0.406558
5nM-NA	-1.60972	-1.60972	-0.80955	0.011913	0.396738	1.106607	1.106607
-NA	-0.3434	-0.3434	-0.06043	0.356659	0.664944	0.984693	0.984693
50nM+NA	-0.11084	-0.11084	-0.10781	0.047707	0.401433	0.557671	0.557671
5nM+NA	-0.24175	-0.24175	-0.21522	-0.02262	0.150829	0.186146	0.186146
+NA	-1.26167	-1.26167	-0.7161	-0.36907	0.414433	0.798305	0.798305

Oneway Anova

Summary of Fit

Rsquare	0.120761
Adj Rsquare	-0.02578
Root Mean Square Error	0.54494
Mean of Response	-6.2e-18
Observations (or Sum Wgts)	36

Analysis of Variance

Source	DF	Sum of Squares	Mean Square	F Ratio	Prob > F
Label	5	1.223592	0.244718	0.8241	0.5425
Error	30	8.908790	0.296960		
C. Total	35	10.132382			

Means for Oneway Anova

Level	Number	Mean	Std Error	Lower 95%	Upper 95%
50nM-NA	6	-0.04204	0.22247	-0.4964	0.41231
5nM-NA	6	-0.14367	0.22247	-0.5980	0.31068
-NA	6	0.32448	0.22247	-0.1299	0.77882
50nM+NA	6	0.13080	0.22247	-0.3235	0.58515
5nM+NA	6	-0.02803	0.22247	-0.4824	0.42632
+NA	6	-0.24155	0.22247	-0.6959	0.21280

Std Error uses a pooled estimate of error variance

Means Comparisons

Comparisons with a control using Dunnett's Method

Oneway Analysis of riboflavin By Label

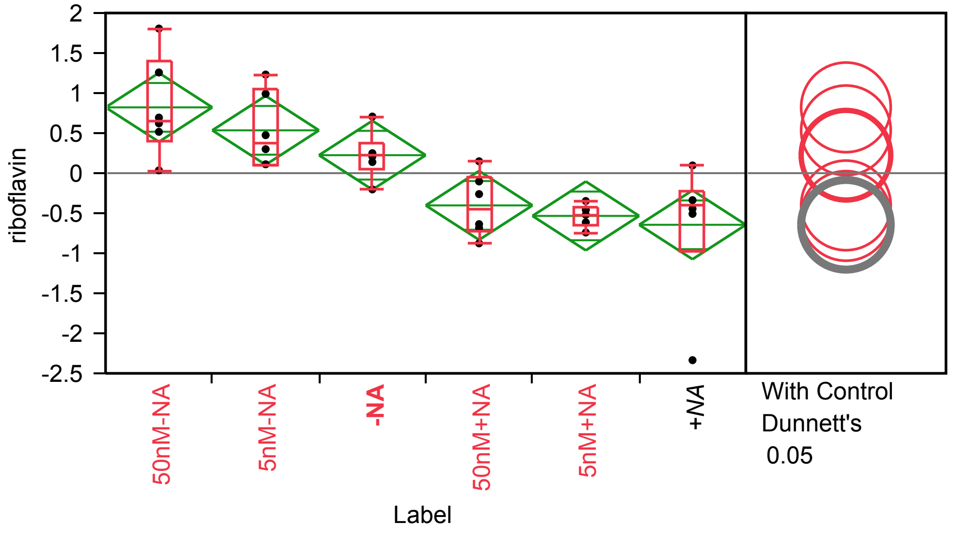

Quantiles

Level	Minimum	10%	25%	Median	75%	90%	Maximum
50nM-NA	0.034846	0.034846	0.395916	0.658204	1.394677	1.805881	1.805881
5nM-NA	0.102644	0.102644	0.108737	0.38718	1.050787	1.231192	1.231192
-NA	-0.2027	-0.2027	0.053623	0.226996	0.363773	0.706698	0.706698
50nM+NA	-0.8764	-0.8764	-0.73324	-0.449	-0.04042	0.14812	0.14812
5nM+NA	-0.74059	-0.74059	-0.64577	-0.52125	-0.43049	-0.34795	-0.34795
+NA	-2.33557	-2.33557	-0.96492	-0.39202	-0.22897	0.096083	0.096083

Oneway Anova

Summary of Fit

Rsquare	0.584834
Adj Rsquare	0.51564
Root Mean Square Error	0.515918
Mean of Response	-5.6e-10
Observations (or Sum Wgts)	36

Analysis of Variance

Source	DF	Sum of Squares	Mean Square	F Ratio	Prob > F
Label	5	11.248497	2.24970	8.4521	<.0001 *
Error	30	7.985148	0.26617		
C. Total	35	19.233645			

Means for Oneway Anova

Level	Number	Mean	Std Error	Lower 95%	Upper 95%
50nM-NA	6	0.82184	0.21062	0.392	1.252
5nM-NA	6	0.53494	0.21062	0.105	0.965
-NA	6	0.22442	0.21062	-0.206	0.655
50nM+NA	6	-0.40251	0.21062	-0.833	0.028
5nM+NA	6	-0.53387	0.21062	-0.964	-0.104
+NA	6	-0.64481	0.21062	-1.075	-0.215

Std Error uses a pooled estimate of error variance

Means Comparisons

Comparisons with a control using Dunnett's Method

Oneway Analysis of ribose-phosphate By Label

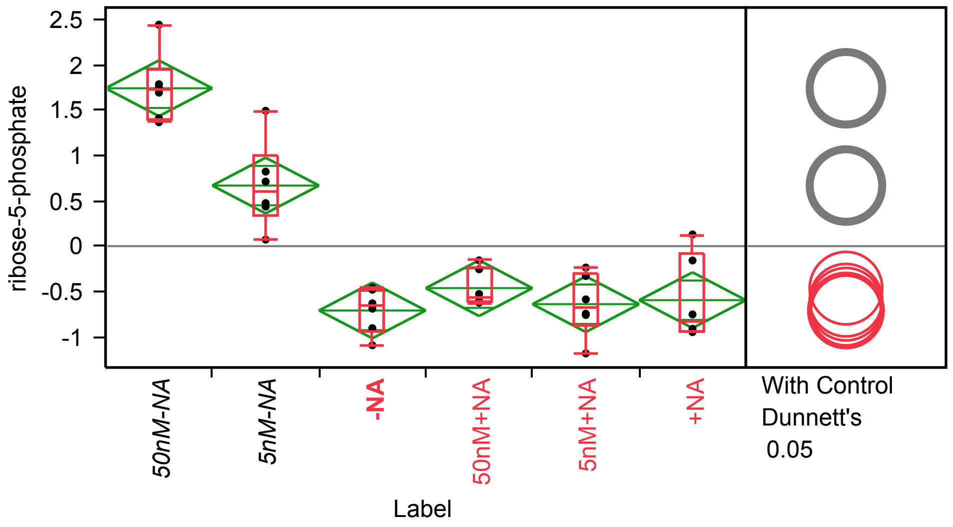

Quantiles

Level	Minimum	10%	25%	Median	75%	90%	Maximum
50nM-NA	1.36752	1.36752	1.40041	1.721312	1.950903	2.443475	2.443475
5nM-NA	0.072426	0.072426	0.345189	0.593513	0.989963	1.493507	1.493507
-NA	-1.0937	-1.0937	-0.95236	-0.66051	-0.47543	-0.45917	-0.45917
50nM+NA	-0.62499	-0.62499	-0.61368	-0.56685	-0.23057	-0.1572	-0.1572
5nM+NA	-1.18351	-1.18351	-0.8657	-0.66669	-0.30656	-0.23687	-0.23687
+NA	-0.94885	-0.94885	-0.93785	-0.83299	-0.08649	0.128355	0.128355

Oneway Anova

Summary of Fit

Rsquare	0.879611
Adj Rsquare	0.859546
Root Mean Square Error	0.368706
Mean of Response	-8.3e-10
Observations (or Sum Wgts)	36

Analysis of Variance

Source	DF	Sum of Squares	Mean Square	F Ratio	Prob > F
Label	5	29.797804	5.95956	43.8384	<.0001 *
Error	30	4.078317	0.13594		
C. Total	35	33.876121			

Means for Oneway Anova

Level	Number	Mean	Std Error	Lower 95%	Upper 95%
50nM-NA	6	1.7420	0.15052	1.435	2.049
5nM-NA	6	0.6685	0.15052	0.361	0.976
-NA	6	-0.7100	0.15052	-1.017	-0.403
50nM+NA	6	-0.4635	0.15052	-0.771	-0.156
5nM+NA	6	-0.6406	0.15052	-0.948	-0.333
+NA	6	-0.5965	0.15052	-0.904	-0.289

Std Error uses a pooled estimate of error variance

Means Comparisons

Comparisons with a control using Dunnett's Method

Oneway Analysis of S-adenosyl-L-homoCysteine By Label

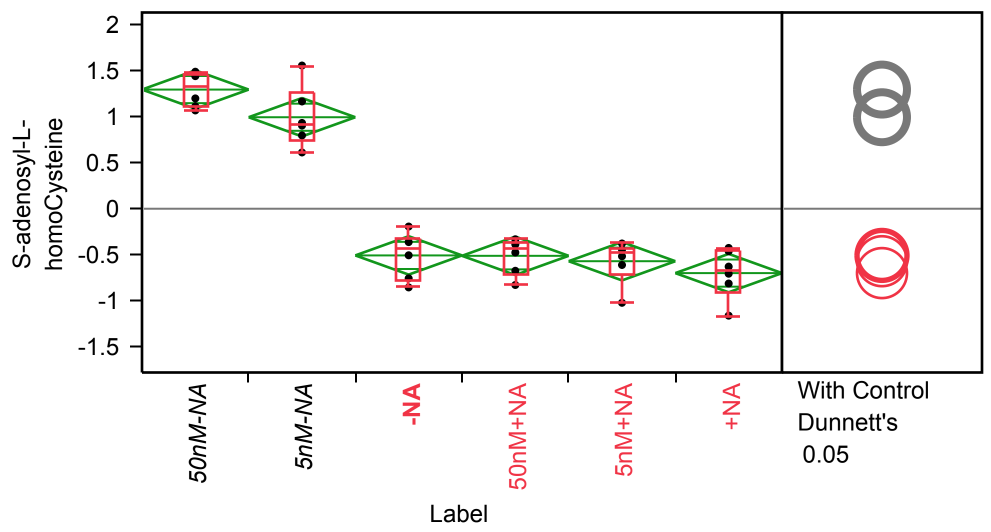

Quantiles

Level	Minimum	10%	25%	Median	75%	90%	Maximum
50nM-NA	1.070199	1.070199	1.103128	1.3221	1.469721	1.491266	1.491266
5nM-NA	0.612681	0.612681	0.751524	0.919522	1.265497	1.558623	1.558623
-NA	-0.85658	-0.85658	-0.78267	-0.43898	-0.32084	-0.19402	-0.19402
50nM+NA	-0.83008	-0.83008	-0.71421	-0.43272	-0.36253	-0.33273	-0.33273
5nM+NA	-1.02397	-1.02397	-0.7152	-0.48397	-0.42826	-0.37836	-0.37836
+NA	-1.16386	-1.16386	-0.90163	-0.66874	-0.45344	-0.42699	-0.42699

Oneway Anova

Summary of Fit

Rsquare	0.927399
Adj Rsquare	0.915299
Root Mean Square Error	0.250661
Mean of Response	-2.78e-9
Observations (or Sum Wgts)	36

Analysis of Variance

Source	DF	Sum of Squares	Mean Square	F Ratio	Prob > F
Label	5	24.078048	4.81561	76.6439	<.0001 *
Error	30	1.884928	0.06283		
C. Total	35	25.962976			

Means for Oneway Anova

Level	Number	Mean	Std Error	Lower 95%	Upper 95%
50nM-NA	6	1.2971	0.10233	1.088	1.506
5nM-NA	6	0.9960	0.10233	0.787	1.205
-NA	6	-0.5083	0.10233	-0.717	-0.299
50nM+NA	6	-0.5127	0.10233	-0.722	-0.304
5nM+NA	6	-0.5712	0.10233	-0.780	-0.362
+NA	6	-0.7008	0.10233	-0.910	-0.492

Std Error uses a pooled estimate of error variance

Means Comparisons

Comparisons with a control using Dunnett's Method

Oneway Analysis of S-adenosyl-L-methioninamine By Label

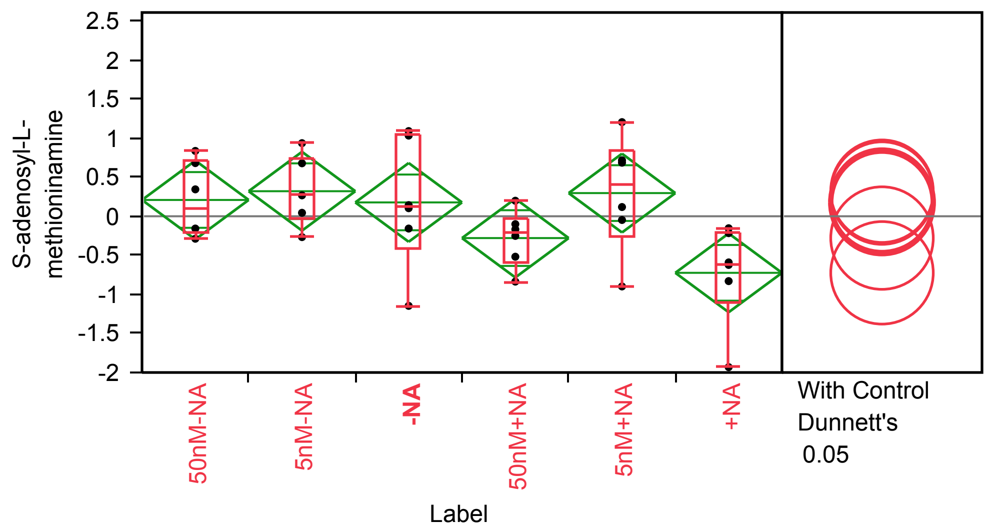

Quantiles

Level	Minimum	10%	25%	Median	75%	90%	Maximum
50nM-NA	-0.28761	-0.28761	-0.19509	0.093634	0.719483	0.836267	0.836267
5nM-NA	-0.26679	-0.26679	-0.03362	0.266391	0.74263	0.935685	0.935685
-NA	-1.14681	-1.14681	-0.40474	0.12275	1.047096	1.090212	1.090212
50nM+NA	-0.8364	-0.8364	-0.59756	-0.20946	-0.02599	0.198594	0.198594
5nM+NA	-0.8982	-0.8982	-0.26014	0.402003	0.83862	1.204845	1.204845
+NA	-1.93127	-1.93127	-1.10536	-0.60692	-0.20048	-0.15344	-0.15344

Oneway Anova

Summary of Fit

Rsquare	0.321833
Adj Rsquare	0.208805
Root Mean Square Error	0.604885
Mean of Response	-5.6e-10
Observations (or Sum Wgts)	36

Analysis of Variance

Source	DF	Sum of Squares	Mean Square	F Ratio	Prob > F
Label	5	5.209078	1.04182	2.8474	0.0321 *
Error	30	10.976564	0.36589		
C. Total	35	16.185642			

Means for Oneway Anova

Level	Number	Mean	Std Error	Lower 95%	Upper 95%
50nM-NA	6	0.20871	0.24694	-0.296	0.7130
5nM-NA	6	0.32068	0.24694	-0.184	0.8250
-NA	6	0.17737	0.24694	-0.327	0.6817
50nM+NA	6	-0.27925	0.24694	-0.784	0.2251
5nM+NA	6	0.29662	0.24694	-0.208	0.8009
+NA	6	-0.72413	0.24694	-1.228	-0.2198

Std Error uses a pooled estimate of error variance

Means Comparisons

Comparisons with a control using Dunnett's Method

Oneway Analysis of SAM By Label

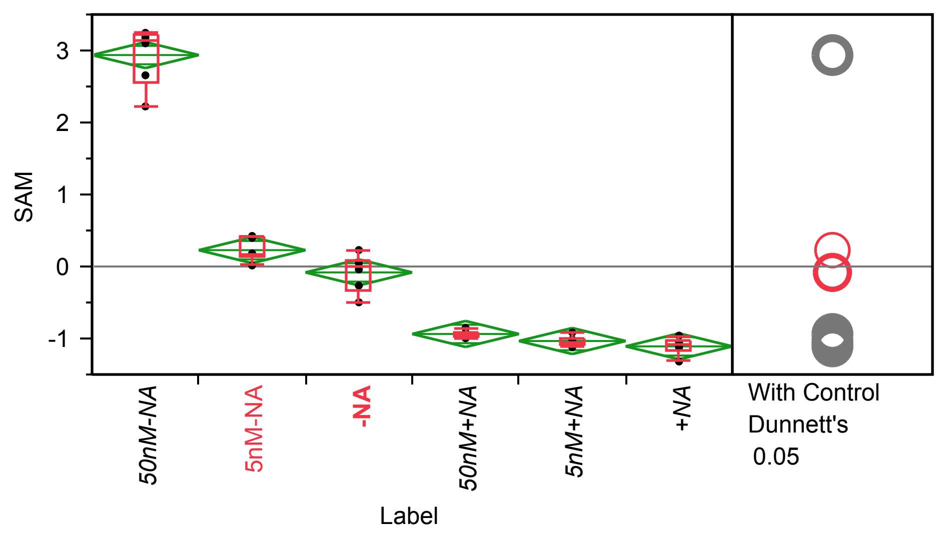

Quantiles

Level	Minimum	10%	25%	Median	75%	90%	Maximum
50nM-NA	2.223299	2.223299	2.54751	3.139784	3.222923	3.244946	3.244946
5nM-NA	0.014577	0.014577	0.131006	0.177963	0.40405	0.423854	0.423854
-NA	-0.49723	-0.49723	-0.3231	-0.00298	0.092695	0.228431	0.228431
50nM+NA	-0.99678	-0.99678	-0.96657	-0.94732	-0.90473	-0.8524	-0.8524
5nM+NA	-1.12004	-1.12004	-1.08616	-1.042	-0.98991	-0.91751	-0.91751
+NA	-1.31933	-1.31933	-1.17713	-1.09477	-1.03249	-0.96003	-0.96003

Oneway Anova

Summary of Fit

Rsquare	0.980593
Adj Rsquare	0.977359
Root Mean Square Error	0.216674
Mean of Response	5.56e-10
Observations (or Sum Wgts)	36

Analysis of Variance

Source	DF	Sum of Squares	Mean Square	F Ratio	Prob > F
Label	5	71.166602	14.2333	303.1743	<.0001 *
Error	30	1.408429	0.0469		
C. Total	35	72.575032			

Means for Oneway Anova

Level	Number	Mean	Std Error	Lower 95%	Upper 95%
50nM-NA	6	2.9365	0.08846	2.756	3.117
5nM-NA	6	0.2269	0.08846	0.046	0.408
-NA	6	-0.0821	0.08846	-0.263	0.099
50nM+NA	6	-0.9371	0.08846	-1.118	-0.756
5nM+NA	6	-1.0351	0.08846	-1.216	-0.854
+NA	6	-1.1092	0.08846	-1.290	-0.929

Std Error uses a pooled estimate of error variance

Means Comparisons

Comparisons with a control using Dunnett's Method

Oneway Analysis of sarcosine By Label

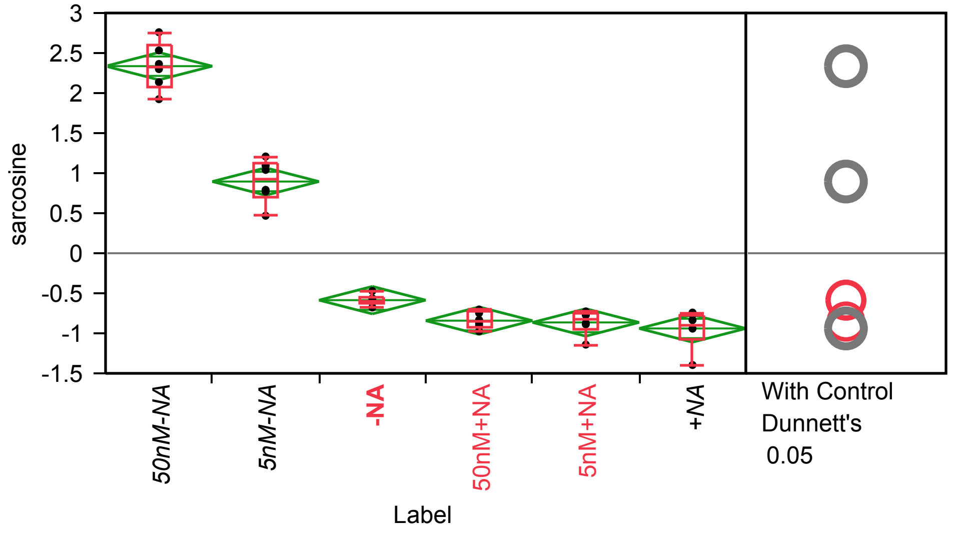

Quantiles

Level	Minimum	10%	25%	Median	75%	90%	Maximum
50nM-NA	1.923935	1.923935	2.083438	2.33157	2.589684	2.759978	2.759978
5nM-NA	0.468202	0.468202	0.693978	0.916151	1.123111	1.208169	1.208169
-NA	-0.67743	-0.67743	-0.629	-0.59586	-0.53966	-0.4709	-0.4709
50nM+NA	-0.97384	-0.97384	-0.92782	-0.85742	-0.73657	-0.70321	-0.70321
5nM+NA	-1.14095	-1.14095	-0.951	-0.82779	-0.76161	-0.72753	-0.72753
+NA	-1.3959	-1.3959	-1.06364	-0.88853	-0.76279	-0.74112	-0.74112

Oneway Anova

Summary of Fit

Rsquare	0.976846
Adj Rsquare	0.972987
Root Mean Square Error	0.205874
Mean of Response	1.111e-8
Observations (or Sum Wgts)	36

Analysis of Variance

Source	DF	Sum of Squares	Mean Square	F Ratio	Prob > F
Label	5	53.644813	10.7290	253.1363	<.0001 *
Error	30	1.271524	0.0424		
C. Total	35	54.916337			

Means for Oneway Anova

Level	Number	Mean	Std Error	Lower 95%	Upper 95%
50nM-NA	6	2.3361	0.08405	2.164	2.508
5nM-NA	6	0.8954	0.08405	0.724	1.067
-NA	6	-0.5859	0.08405	-0.758	-0.414
50nM+NA	6	-0.8420	0.08405	-1.014	-0.670
5nM+NA	6	-0.8641	0.08405	-1.036	-0.692
+NA	6	-0.9395	0.08405	-1.111	-0.768

Std Error uses a pooled estimate of error variance

Means Comparisons

Comparisons with a control using Dunnett's Method

Oneway Analysis of Sedoheptulose 7-phosphate By Label

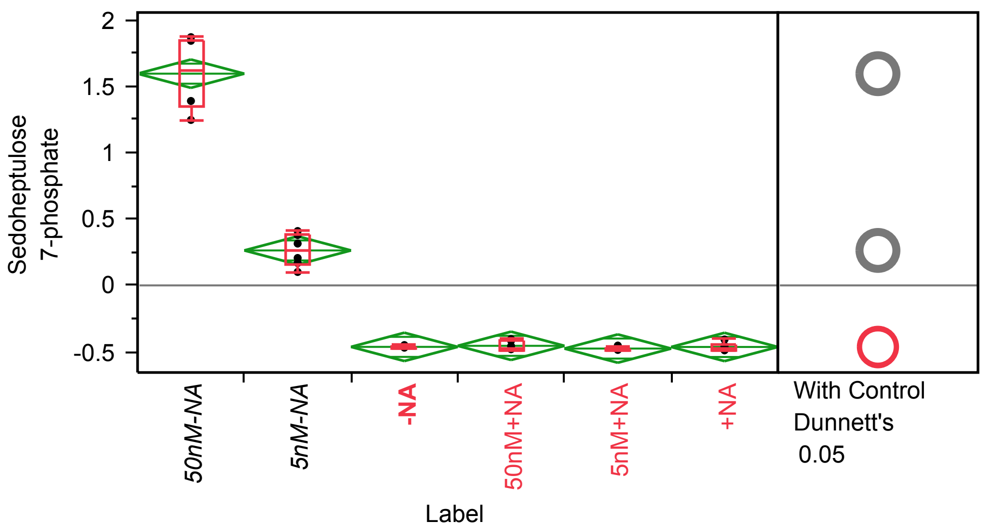

Quantiles

Level	Minimum	10%	25%	Median	75%	90%	Maximum
50nM-NA	1.246295	1.246295	1.350099	1.612653	1.849228	1.869874	1.869874
5nM-NA	0.101108	0.101108	0.151945	0.260713	0.386592	0.409338	0.409338
-NA	-0.4741	-0.4741	-0.47045	-0.4635	-0.45664	-0.44963	-0.44963
50nM+NA	-0.4885	-0.4885	-0.48504	-0.46917	-0.41308	-0.40467	-0.40467
5nM+NA	-0.49056	-0.49056	-0.48729	-0.47592	-0.46823	-0.45339	-0.45339
+NA	-0.48964	-0.48964	-0.48933	-0.47121	-0.44251	-0.40742	-0.40742

Oneway Anova

Summary of Fit

Rsquare	0.976969
Adj Rsquare	0.97313
Root Mean Square Error	0.128017
Mean of Response	8.333e-9
Observations (or Sum Wgts)	36

Analysis of Variance

Source	DF	Sum of Squares	Mean Square	F Ratio	Prob > F
Label	5	20.855198	4.17104	254.5134	<.0001 *
Error	30	0.491649	0.01639		
C. Total	35	21.346847			

Means for Oneway Anova

Level	Number	Mean	Std Error	Lower 95%	Upper 95%
50nM-NA	6	1.5948	0.05226	1.488	1.701
5nM-NA	6	0.2633	0.05226	0.157	0.370
-NA	6	-0.4632	0.05226	-0.570	-0.356
50nM+NA	6	-0.4552	0.05226	-0.562	-0.348
5nM+NA	6	-0.4759	0.05226	-0.583	-0.369
+NA	6	-0.4638	0.05226	-0.571	-0.357

Std Error uses a pooled estimate of error variance

Means Comparisons

Comparisons with a control using Dunnett's Method

Oneway Analysis of serine By Label

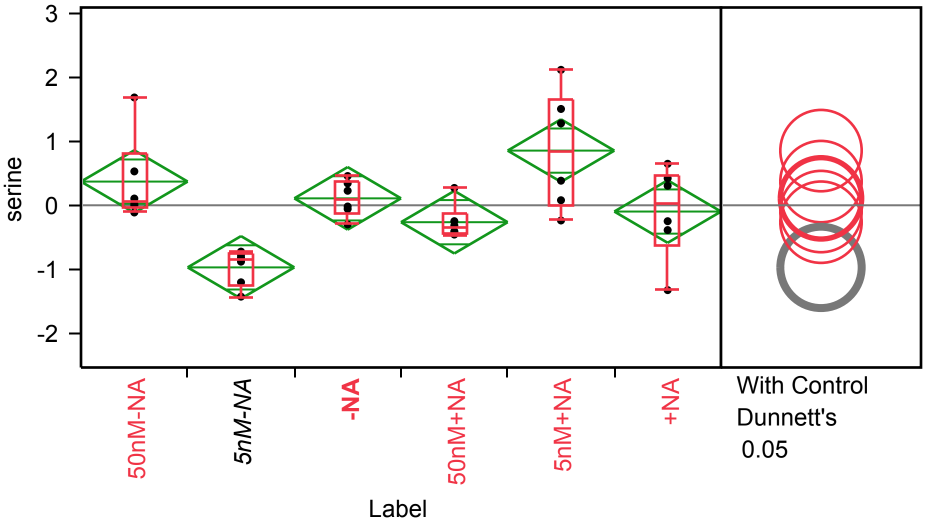

Quantiles

Level	Minimum	10%	25%	Median	75%	90%	Maximum
50nM-NA	-0.1128	-0.1128	-0.03148	0.062426	0.82075	1.689653	1.689653
5nM-NA	-1.43194	-1.43194	-1.26361	-0.85049	-0.7625	-0.72634	-0.72634
-NA	-0.3003	-0.3003	-0.11871	0.10402	0.372148	0.458249	0.458249
50nM+NA	-0.45886	-0.45886	-0.4355	-0.36322	-0.11894	0.271565	0.271565
5nM+NA	-0.23819	-0.23819	-0.00032	0.83422	1.662908	2.122642	2.122642
+NA	-1.32978	-1.32978	-0.62418	0.028276	0.479647	0.64999	0.64999

Oneway Anova

Summary of Fit

Rsquare	0.525293
Adj Rsquare	0.446175
Root Mean Square Error	0.58782
Mean of Response	5.56e-11
Observations (or Sum Wgts)	36

Analysis of Variance

Source	DF	Sum of Squares	Mean Square	F Ratio	Prob > F
Label	5	11.470604	2.29412	6.6394	0.0003 *
Error	30	10.365968	0.34553		
C. Total	35	21.836572			

Means for Oneway Anova

Level	Number	Mean	Std Error	Lower 95%	Upper 95%
50nM-NA	6	0.37141	0.23998	-0.119	0.862
5nM-NA	6	-0.97355	0.23998	-1.464	-0.483
-NA	6	0.10854	0.23998	-0.382	0.599
50nM+NA	6	-0.26509	0.23998	-0.755	0.225
5nM+NA	6	0.85692	0.23998	0.367	1.347
+NA	6	-0.09822	0.23998	-0.588	0.392

Std Error uses a pooled estimate of error variance

Means Comparisons

Comparisons with a control using Dunnett's Method

Oneway Analysis of S-methyl-5-thioadenosine By Label

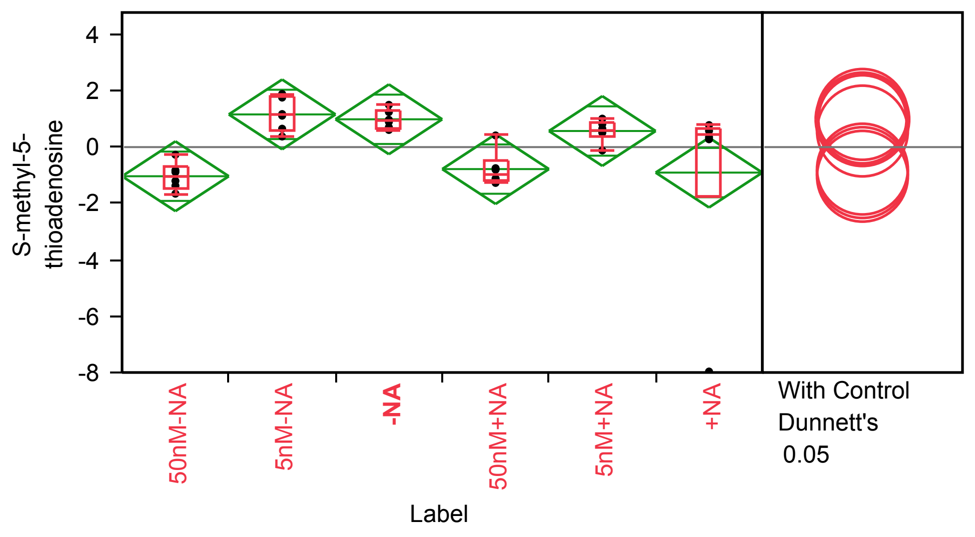

Quantiles

Level	Minimum	10%	25%	Median	75%	90%	Maximum
50nM-NA	-1.65321	-1.65321	-1.44206	-1.04289	-0.6847	-0.2709	-0.2709
5nM-NA	0.376373	0.376373	0.583673	1.144161	1.790213	1.886227	1.886227
-NA	0.608475	0.608475	0.680015	0.933656	1.307273	1.500063	1.500063
50nM+NA	-1.27072	-1.27072	-1.18846	-0.95774	-0.45745	0.413944	0.413944
5nM+NA	-0.11954	-0.11954	0.352292	0.607012	0.86402	1.00958	1.00958
+NA	-7.96591	-7.96591	-1.78295	0.450921	0.627676	0.781192	0.781192

Oneway Anova

Summary of Fit

Rsquare	0.318141
Adj Rsquare	0.204498
Root Mean Square Error	1.484897
Mean of Response	2.47e-17
Observations (or Sum Wgts)	36

Analysis of Variance

Source	DF	Sum of Squares	Mean Square	F Ratio	Prob > F
Label	5	30.863072	6.17261	2.7995	0.0343 *
Error	30	66.147597	2.20492		
C. Total	35	97.010669			

Means for Oneway Anova

Level	Number	Mean	Std Error	Lower 95%	Upper 95%
50nM-NA	6	-1.0340	0.60621	-2.272	0.2040
5nM-NA	6	1.1603	0.60621	-0.078	2.3984
-NA	6	0.9871	0.60621	-0.251	2.2252
50nM+NA	6	-0.7802	0.60621	-2.018	0.4578
5nM+NA	6	0.5715	0.60621	-0.667	1.8096
+NA	6	-0.9047	0.60621	-2.143	0.3333

Std Error uses a pooled estimate of error variance

Means Comparisons

Comparisons with a control using Dunnett's Method

Oneway Analysis of sn-glycerol-3-phosphate By Label

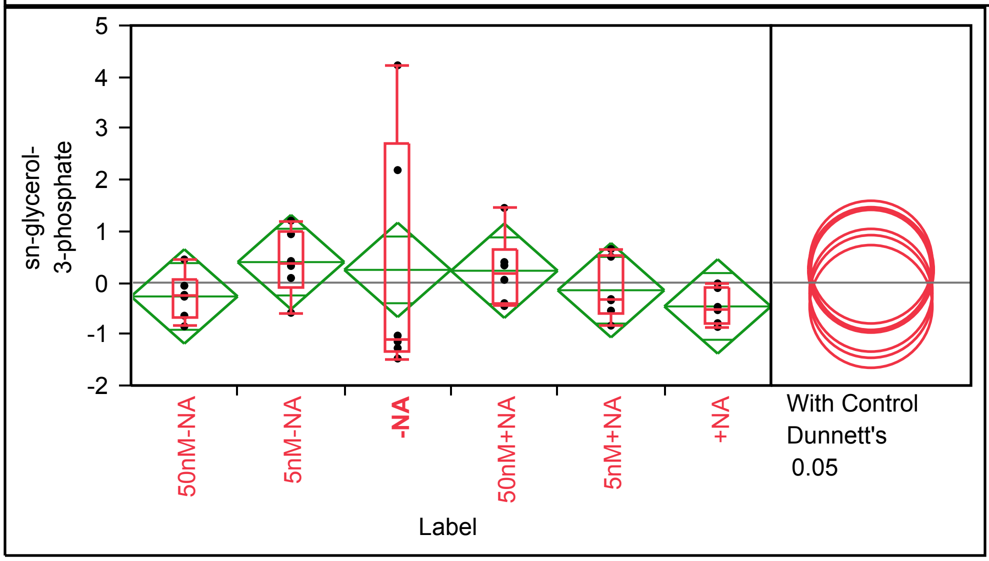

Quantiles

Level	Minimum	10%	25%	Median	75%	90%	Maximum
50nM-NA	-0.84933	-0.84933	-0.69428	-0.25504	0.067408	0.450624	0.450624
5nM-NA	-0.58666	-0.58666	-0.07868	0.372488	1.007551	1.204524	1.204524
-NA	-1.47565	-1.47565	-1.32362	-1.08765	2.697419	4.225186	4.225186
50nM+NA	-0.45447	-0.45447	-0.41285	0.19449	0.663132	1.453019	1.453019
5nM+NA	-0.83587	-0.83587	-0.61737	-0.33108	0.540809	0.653015	0.653015
+NA	-0.86134	-0.86134	-0.80601	-0.50291	-0.08262	-0.01575	-0.01575

Oneway Anova

Summary of Fit

Rsquare	0.087851
Adj Rsquare	-0.06417
Root Mean Square Error	1.09981
Mean of Response	0
Observations (or Sum Wgts)	36

Analysis of Variance

Source	DF	Sum of Squares	Mean Square	F Ratio	Prob > F
Label	5	3.494932	0.69899	0.5779	0.7165
Error	30	36.287458	1.20958		
C. Total	35	39.782390			

Means for Oneway Anova

Level	Number	Mean	Std Error	Lower 95%	Upper 95%
50nM-NA	6	-0.26862	0.44900	-1.186	0.6484
5nM-NA	6	0.39923	0.44900	-0.518	1.3162
-NA	6	0.24824	0.44900	-0.669	1.1652
50nM+NA	6	0.23140	0.44900	-0.686	1.1484
5nM+NA	6	-0.14769	0.44900	-1.065	0.7693
+NA	6	-0.46256	0.44900	-1.380	0.4544

Std Error uses a pooled estimate of error variance

Means Comparisons

Comparisons with a control using Dunnett's Method

Oneway Analysis of Spermidine By Label

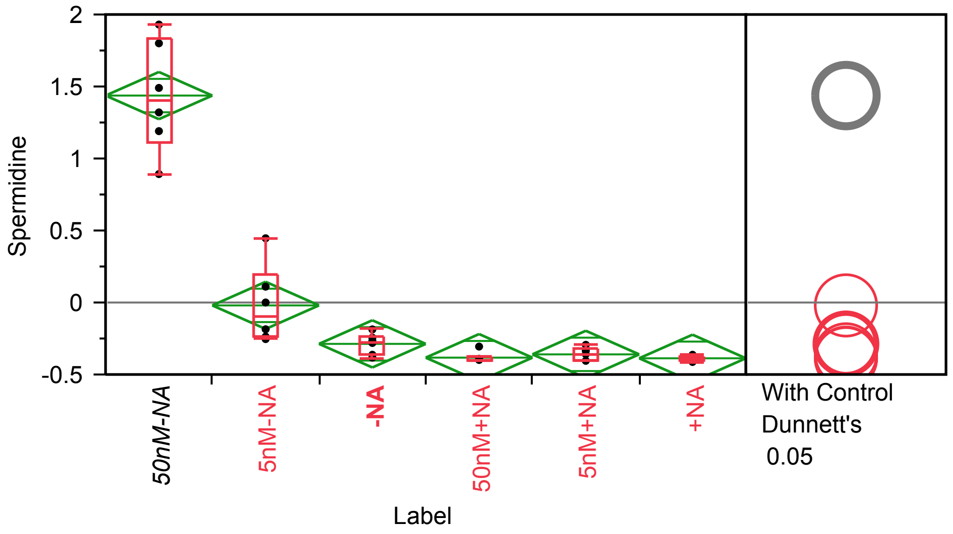

Quantiles

Level	Minimum	10%	25%	Median	75%	90%	Maximum
50nM-NA	0.892	0.892	1.1155	1.405	1.8325	1.93	1.93
5nM-NA	-0.254	-0.254	-0.24125	-0.09298	0.194	0.446	0.446
-NA	-0.383	-0.383	-0.368	-0.2735	-0.22975	-0.187	-0.187
50nM+NA	-0.405	-0.405	-0.40425	-0.396	-0.36975	-0.306	-0.306
5nM+NA	-0.406	-0.406	-0.40525	-0.3605	-0.32475	-0.294	-0.294
+NA	-0.413	-0.413	-0.40475	-0.387	-0.36975	-0.363	-0.363

Oneway Anova

Summary of Fit

Rsquare	0.93022
Adj Rsquare	0.918589
Root Mean Square Error	0.196528
Mean of Response	-0.00017
Observations (or Sum Wgts)	36

Analysis of Variance

Source	DF	Sum of Squares	Mean Square	F Ratio	Prob > F
Label	5	15.446231	3.08925	79.9839	<.0001 *
Error	30	1.158700	0.03862		
C. Total	35	16.604931			

Means for Oneway Anova

Level	Number	Mean	Std Error	Lower 95%	Upper 95%
50nM-NA	6	1.4370	0.08023	1.273	1.601
5nM-NA	6	-0.0202	0.08023	-0.184	0.144
-NA	6	-0.2873	0.08023	-0.451	-0.123
50nM+NA	6	-0.3830	0.08023	-0.547	-0.219
5nM+NA	6	-0.3602	0.08023	-0.524	-0.196
+NA	6	-0.3873	0.08023	-0.551	-0.223

Std Error uses a pooled estimate of error variance

Means Comparisons

Comparisons with a control using Dunnett's Method

Oneway Analysis of Spermine By Label

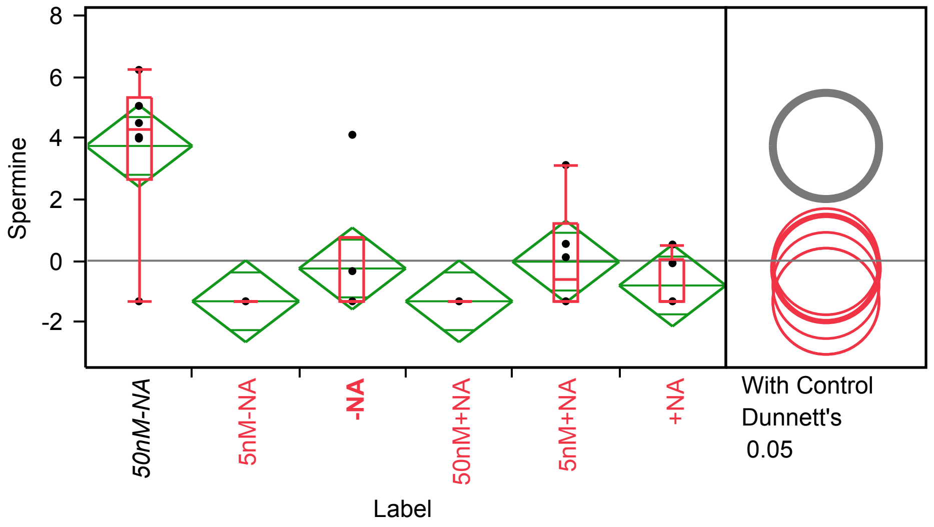

Quantiles

Level	Minimum	10%	25%	Median	75%	90%	Maximum
50nM-NA	-1.32251	-1.32251	2.654467	4.257795	5.336943	6.216519	6.216519
5nM-NA	-1.32251	-1.32251	-1.32251	-1.3225	-1.3225	-1.3225	-1.3225
-NA	-1.32251	-1.32251	-1.32251	-1.32251	0.770728	4.102761	4.102761
50nM+NA	-1.32252	-1.32252	-1.32252	-1.32252	-1.32252	-1.32251	-1.32251
5nM+NA	-1.32252	-1.32252	-1.32251	-0.60454	1.190142	3.116174	3.116174
+NA	-1.32254	-1.32254	-1.32252	-1.32252	0.069881	0.525947	0.525947

Oneway Anova

Summary of Fit

Rsquare	0.588476
Adj Rsquare	0.519889
Root Mean Square Error	1.595264
Mean of Response	-5.6e-10
Observations (or Sum Wgts)	36

Analysis of Variance

Source	DF	Sum of Squares	Mean Square	F Ratio	Prob > F
Label	5	109.17444	21.8349	8.5800	<.0001 *
Error	30	76.34604	2.5449		
C. Total	35	185.52048			

Means for Oneway Anova

Level	Number	Mean	Std Error	Lower 95%	Upper 95%
50nM-NA	6	3.7389	0.65126	2.409	5.0690
5nM-NA	6	-1.3225	0.65126	-2.653	0.0076
-NA	6	-0.2545	0.65126	-1.585	1.0755
50nM+NA	6	-1.3225	0.65126	-2.653	0.0075
5nM+NA	6	-0.0316	0.65126	-1.362	1.2984
+NA	6	-0.8077	0.65126	-2.138	0.5223

Std Error uses a pooled estimate of error variance

Means Comparisons

Comparisons with a control using Dunnett's Method

Oneway Analysis of Stearic acid By Label

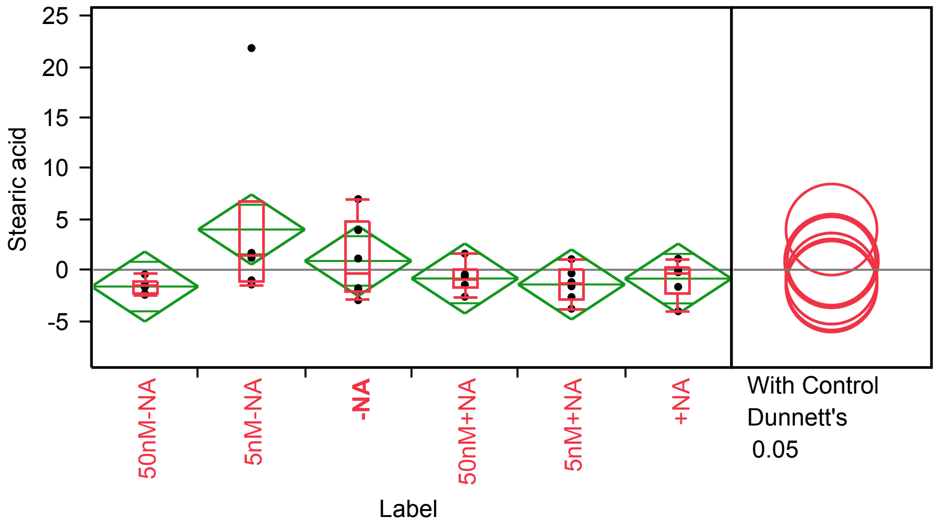

Quantiles

Level	Minimum	10%	25%	Median	75%	90%	Maximum
50nM-NA	-2.46954	-2.46954	-2.36871	-1.59973	-1.20328	-0.45499	-0.45499
5nM-NA	-1.4541	-1.4541	-1.13909	1.386842	6.70778	21.83766	21.83766
-NA	-2.99742	-2.99742	-2.22811	-0.34764	4.669731	6.96455	6.96455
50nM+NA	-2.65753	-2.65753	-1.81392	-1.05068	0.05499	1.594102	1.594102
5nM+NA	-3.84018	-3.84018	-2.97583	-1.4259	-0.00446	1.046058	1.046058
+NA	-4.08126	-4.08126	-2.28217	-0.2684	0.255786	1.083752	1.083752

Oneway Anova

Summary of Fit

Rsquare	0.210501
Adj Rsquare	0.078918
Root Mean Square Error	4.135223
Mean of Response	-2.8e-10
Observations (or Sum Wgts)	36

Analysis of Variance

Source	DF	Sum of Squares	Mean Square	F Ratio	Prob > F
Label	5	136.77992	27.3560	1.5998	0.1905
Error	30	513.00208	17.1001		
C. Total	35	649.78200			

Means for Oneway Anova

Level	Number	Mean	Std Error	Lower 95%	Upper 95%
50nM-NA	6	-1.6520	1.6882	-5.100	1.7958
5nM-NA	6	3.9646	1.6882	0.517	7.4124
-NA	6	0.8675	1.6882	-2.580	4.3153
50nM+NA	6	-0.8593	1.6882	-4.307	2.5885
5nM+NA	6	-1.4480	1.6882	-4.896	1.9997
+NA	6	-0.8728	1.6882	-4.321	2.5749

Std Error uses a pooled estimate of error variance

Means Comparisons

Comparisons with a control using Dunnett's Method

Oneway Analysis of succinate By Label

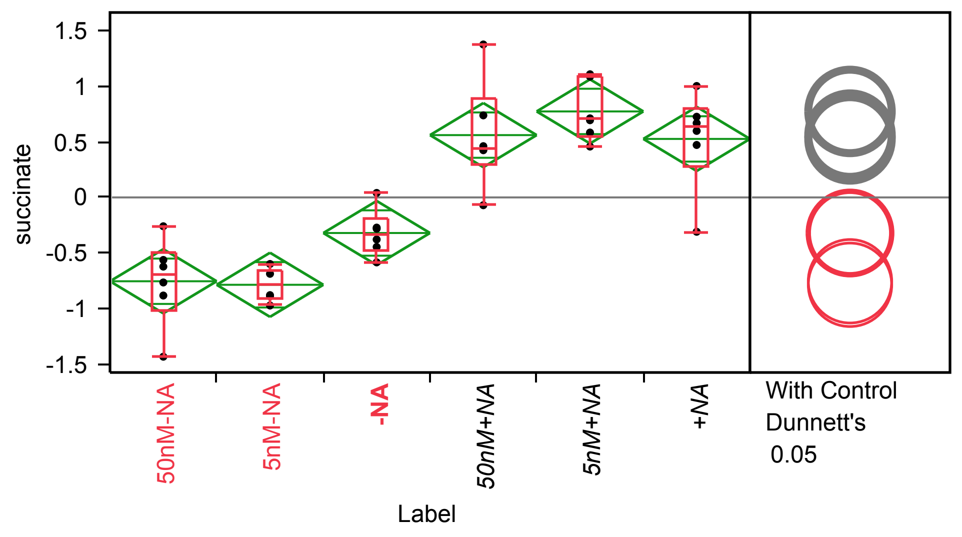

Quantiles

Level	Minimum	10%	25%	Median	75%	90%	Maximum
50nM-NA	-1.43791	-1.43791	-1.02373	-0.69613	-0.48861	-0.26051	-0.26051
5nM-NA	-0.97568	-0.97568	-0.91276	-0.78723	-0.66743	-0.60105	-0.60105
-NA	-0.58622	-0.58622	-0.48188	-0.33113	-0.19434	0.040451	0.040451
50nM+NA	-0.07029	-0.07029	0.302051	0.448925	0.900714	1.380463	1.380463
5nM+NA	0.459265	0.459265	0.55407	0.705224	1.095965	1.111752	1.111752
+NA	-0.31004	-0.31004	0.27755	0.635607	0.797505	1.005749	1.005749

Oneway Anova

Summary of Fit

Rsquare	0.80543
Adj Rsquare	0.773002
Root Mean Square Error	0.34727
Mean of Response	-2.8e-10
Observations (or Sum Wgts)	36

Analysis of Variance

Source	DF	Sum of Squares	Mean Square	F Ratio	Prob > F
Label	5	14.976412	2.99528	24.8373	<.0001 *
Error	30	3.617887	0.12060		
C. Total	35	18.594299			

Means for Oneway Anova

Level	Number	Mean	Std Error	Lower 95%	Upper 95%
50nM-NA	6	-0.75683	0.14177	-1.046	-0.467
5nM-NA	6	-0.78876	0.14177	-1.078	-0.499
-NA	6	-0.32129	0.14177	-0.611	-0.032
50nM+NA	6	0.56250	0.14177	0.273	0.852
5nM+NA	6	0.77631	0.14177	0.487	1.066
+NA	6	0.52807	0.14177	0.239	0.818

Std Error uses a pooled estimate of error variance

Means Comparisons

Comparisons with a control using Dunnett's Method

Oneway Analysis of taurine By Label

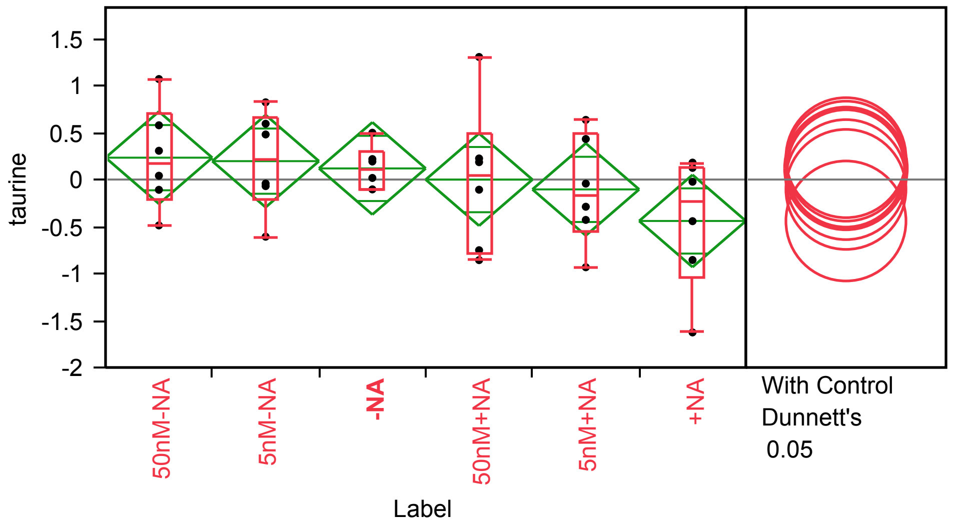

Quantiles

Level	Minimum	10%	25%	Median	75%	90%	Maximum
50nM-NA	-0.4881	-0.4881	-0.20402	0.173288	0.70032	1.069158	1.069158
5nM-NA	-0.60823	-0.60823	-0.20472	0.218647	0.650642	0.821713	0.821713
-NA	-0.10991	-0.10991	-0.10606	0.104354	0.287704	0.4978	0.4978
50nM+NA	-0.85736	-0.85736	-0.77834	0.038308	0.494284	1.303971	1.303971
5nM+NA	-0.93313	-0.93313	-0.55544	-0.16651	0.482074	0.634638	0.634638
+NA	-1.62673	-1.62673	-1.04864	-0.23336	0.138176	0.180028	0.180028

Oneway Anova

Summary of Fit

Rsquare	0.15255
Adj Rsquare	0.011308
Root Mean Square Error	0.588546
Mean of Response	2.78e-10
Observations (or Sum Wgts)	36

Analysis of Variance

Source	DF	Sum of Squares	Mean Square	F Ratio	Prob > F
Label	5	1.870590	0.374118	1.0801	0.3913
Error	30	10.391586	0.346386		
C. Total	35	12.262176			

Means for Oneway Anova

Level	Number	Mean	Std Error	Lower 95%	Upper 95%
50nM-NA	6	0.23261	0.24027	-0.2581	0.72332
5nM-NA	6	0.19570	0.24027	-0.2950	0.68640
-NA	6	0.11825	0.24027	-0.3725	0.60895
50nM+NA	6	-0.00073	0.24027	-0.4914	0.48997
5nM+NA	6	-0.10497	0.24027	-0.5957	0.38573
+NA	6	-0.44086	0.24027	-0.9316	0.04984

Std Error uses a pooled estimate of error variance

Means Comparisons

Comparisons with a control using Dunnett's Method

Oneway Analysis of thiamine By Label

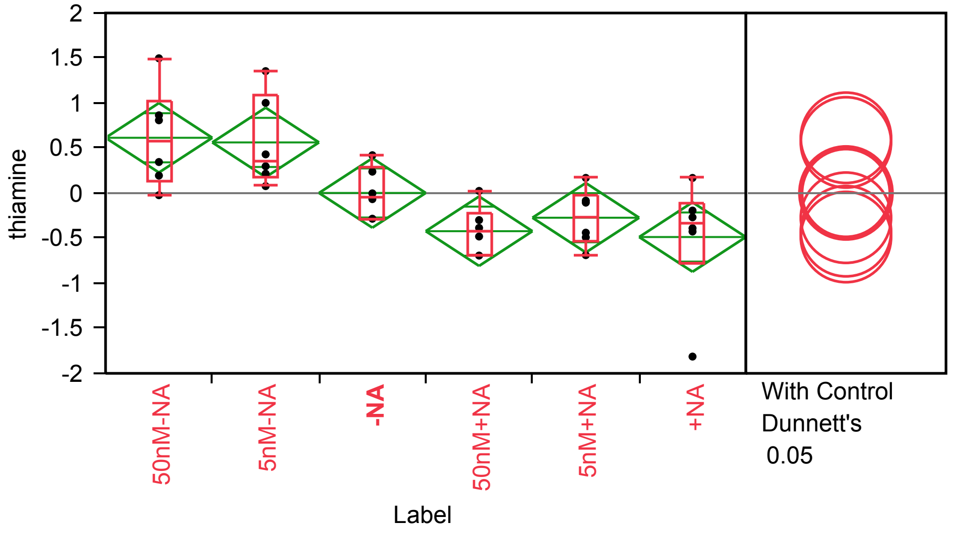

Quantiles

Level	Minimum	10%	25%	Median	75%	90%	Maximum
50nM-NA	-0.02248	-0.02248	0.140847	0.577605	1.022589	1.497344	1.497344
5nM-NA	0.07867	0.07867	0.181834	0.364766	1.090291	1.354683	1.354683
-NA	-0.29359	-0.29359	-0.28602	-0.03404	0.284551	0.421278	0.421278
50nM+NA	-0.69864	-0.69864	-0.69415	-0.43296	-0.21786	0.024988	0.024988
5nM+NA	-0.68771	-0.68771	-0.53955	-0.27162	-0.01991	0.169669	0.169669
+NA	-1.81393	-1.81393	-0.77268	-0.32854	-0.10249	0.169174	0.169174

Oneway Anova

Summary of Fit

Rsquare	0.525622
Adj Rsquare	0.446559
Root Mean Square Error	0.462455
Mean of Response	-2.8e-11
Observations (or Sum Wgts)	36

Analysis of Variance

Source	DF	Sum of Squares	Mean Square	F Ratio	Prob > F
Label	5	7.109016	1.42180	6.6481	0.0003 *
Error	30	6.415949	0.21386		
C. Total	35	13.524966			

Means for Oneway Anova

Level	Number	Mean	Std Error	Lower 95%	Upper 95%
50nM-NA	6	0.61495	0.18880	0.2294	1.001
5nM-NA	6	0.56354	0.18880	0.1780	0.949
-NA	6	0.00251	0.18880	-0.3831	0.388
50nM+NA	6	-0.42184	0.18880	-0.8074	-0.036
5nM+NA	6	-0.27242	0.18880	-0.6580	0.113
+NA	6	-0.48675	0.18880	-0.8723	-0.101

Std Error uses a pooled estimate of error variance

Means Comparisons

Comparisons with a control using Dunnett's Method

Oneway Analysis of threonine By Label

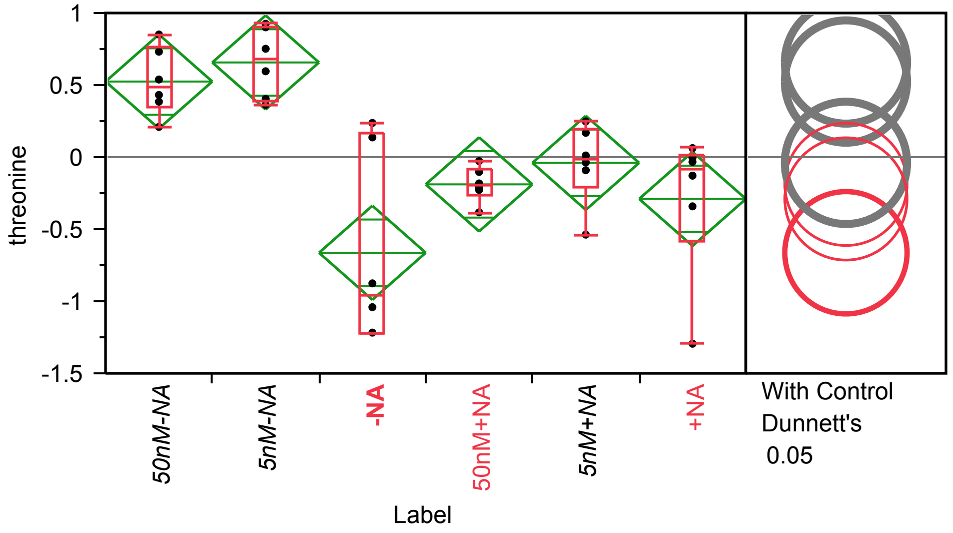

Quantiles

Level	Minimum	10%	25%	Median	75%	90%	Maximum
50nM-NA	0.209812	0.209812	0.341115	0.4851	0.761375	0.850769	0.850769
5nM-NA	0.363014	0.363014	0.394134	0.673831	0.906964	0.925232	0.925232
-NA	-1.22007	-1.22007	-1.21813	-0.95832	0.162208	0.237387	0.237387
50nM+NA	-0.3829	-0.3829	-0.2663	-0.19616	-0.08375	-0.02726	-0.02726
5nM+NA	-0.53756	-0.53756	-0.20249	-0.01287	0.188509	0.247631	0.247631
+NA	-1.29454	-1.29454	-0.57966	-0.08053	0.012383	0.062617	0.062617

Oneway Anova

Summary of Fit

Rsquare	0.623656
Adj Rsquare	0.560932
Root Mean Square Error	0.391157
Mean of Response	-5.6e-11
Observations (or Sum Wgts)	36

Analysis of Variance

Source	DF	Sum of Squares	Mean Square	F Ratio	Prob > F
Label	5	7.606461	1.52129	9.9428	<.0001 *
Error	30	4.590112	0.15300		
C. Total	35	12.196573			

Means for Oneway Anova

Level	Number	Mean	Std Error	Lower 95%	Upper 95%
50nM-NA	6	0.52454	0.15969	0.1984	0.8507
5nM-NA	6	0.65688	0.15969	0.3308	0.9830
-NA	6	-0.66328	0.15969	-0.9894	-0.3371
50nM+NA	6	-0.18875	0.15969	-0.5149	0.1374
5nM+NA	6	-0.03961	0.15969	-0.3657	0.2865
+NA	6	-0.28978	0.15969	-0.6159	0.0363

Std Error uses a pooled estimate of error variance

Means Comparisons

Comparisons with a control using Dunnett's Method

Oneway Analysis of thymidine By Label

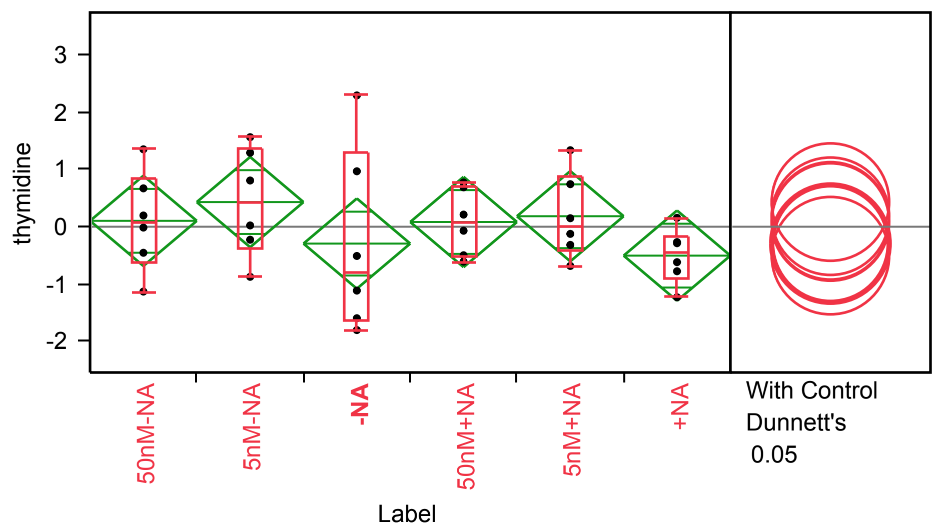

Quantiles

Level	Minimum	10%	25%	Median	75%	90%	Maximum
50nM-NA	-1.13003	-1.13003	-0.62299	0.090474	0.839021	1.349176	1.349176
5nM-NA	-0.87287	-0.87287	-0.3882	0.413995	1.356836	1.560696	1.560696
-NA	-1.80114	-1.80114	-1.64626	-0.8114	1.297044	2.288511	2.288511
50nM+NA	-0.60799	-0.60799	-0.52503	0.070832	0.708291	0.770689	0.770689
5nM+NA	-0.68344	-0.68344	-0.40832	0.011832	0.889645	1.33326	1.33326
+NA	-1.22917	-1.22917	-0.88914	-0.45246	-0.16216	0.155006	0.155006

Oneway Anova

Summary of Fit

Rsquare	0.114577
Adj Rsquare	-0.03299
Root Mean Square Error	0.943235
Mean of Response	1.23e-17
Observations (or Sum Wgts)	36

Analysis of Variance

Source	DF	Sum of Squares	Mean Square	F Ratio	Prob > F
Label	5	3.453870	0.690774	0.7764	0.5744
Error	30	26.690788	0.889693		
C. Total	35	30.144658			

Means for Oneway Anova

Level	Number	Mean	Std Error	Lower 95%	Upper 95%
50nM-NA	6	0.10251	0.38507	-0.684	0.8889
5nM-NA	6	0.42968	0.38507	-0.357	1.2161
-NA	6	-0.29391	0.38507	-1.080	0.4925
50nM+NA	6	0.08241	0.38507	-0.704	0.8688
5nM+NA	6	0.18311	0.38507	-0.603	0.9695
+NA	6	-0.50379	0.38507	-1.290	0.2826

Std Error uses a pooled estimate of error variance

Means Comparisons

Comparisons with a control using Dunnett's Method

Oneway Analysis of trans, trans-farnesyl diphosphate By Label

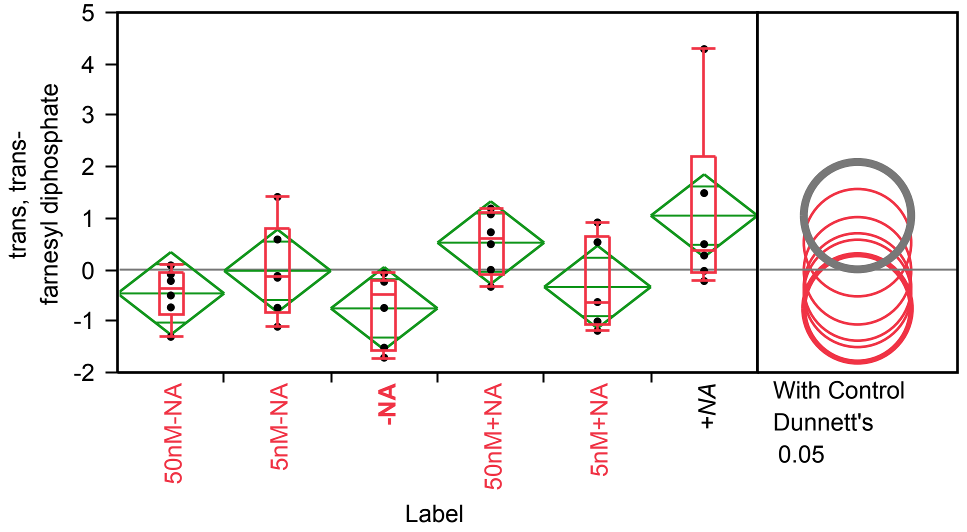

Quantiles

Level	Minimum	10%	25%	Median	75%	90%	Maximum
50nM-NA	-1.30665	-1.30665	-0.87724	-0.36122	-0.04805	0.082965	0.082965
5nM-NA	-1.11056	-1.11056	-0.83438	-0.14009	0.792783	1.412611	1.412611
-NA	-1.71209	-1.71209	-1.56656	-0.49132	-0.19458	-0.07156	-0.07156
50nM+NA	-0.33476	-0.33476	-0.08729	0.611066	1.103809	1.188144	1.188144
5nM+NA	-1.18956	-1.18956	-1.05309	-0.63738	0.629721	0.917205	0.917205
+NA	-0.22178	-0.22178	-0.07375	0.385215	2.188358	4.287777	4.287777

Oneway Anova

Summary of Fit

Rsquare	0.32913
Adj Rsquare	0.217319
Root Mean Square Error	0.962516
Mean of Response	5.56e-11
Observations (or Sum Wgts)	36

Analysis of Variance

Source	DF	Sum of Squares	Mean Square	F Ratio	Prob > F
Label	5	13.635377	2.72708	2.9436	0.0281 *
Error	30	27.793110	0.92644		
C. Total	35	41.428488			

Means for Oneway Anova

Level	Number	Mean	Std Error	Lower 95%	Upper 95%
50nM-NA	6	-0.4620	0.39295	-1.264	0.3405
5nM-NA	6	-0.0224	0.39295	-0.825	0.7801
-NA	6	-0.7533	0.39295	-1.556	0.0492
50nM+NA	6	0.5244	0.39295	-0.278	1.3269
5nM+NA	6	-0.3368	0.39295	-1.139	0.4657
+NA	6	1.0501	0.39295	0.248	1.8526

Std Error uses a pooled estimate of error variance

Means Comparisons

Comparisons with a control using Dunnett's Method

Oneway Analysis of tryptophan By Label

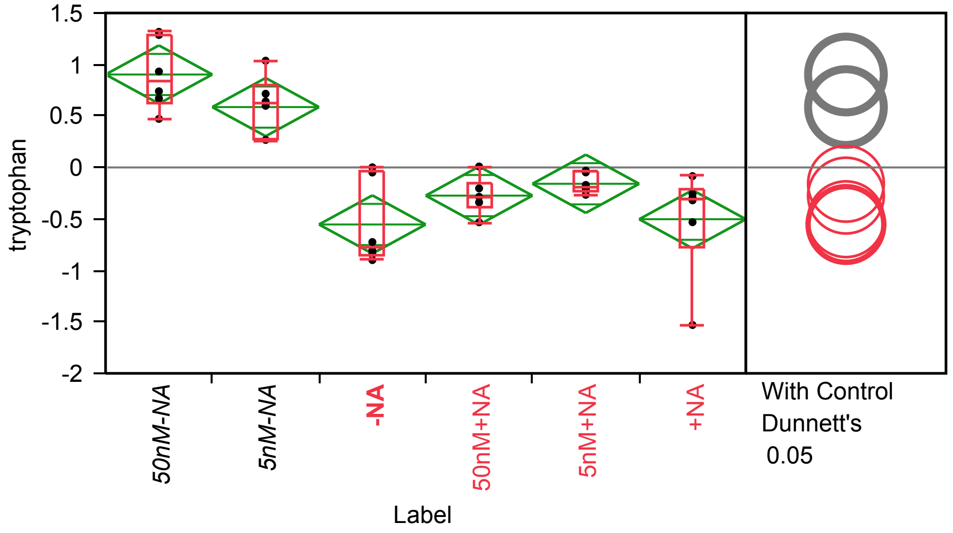

Quantiles

Level	Minimum	10%	25%	Median	75%	90%	Maximum
50nM-NA	0.471929	0.471929	0.620385	0.835644	1.294286	1.316788	1.316788
5nM-NA	0.258286	0.258286	0.265391	0.619707	0.795858	1.036676	1.036676
-NA	-0.89964	-0.89964	-0.85766	-0.76616	-0.03645	0.001094	0.001094
50nM+NA	-0.53259	-0.53259	-0.38729	-0.2878	-0.15018	0.00909	0.00909
5nM+NA	-0.26549	-0.26549	-0.23625	-0.19097	-0.04357	-0.03039	-0.03039
+NA	-1.53036	-1.53036	-0.78134	-0.3066	-0.2143	-0.08462	-0.08462

Oneway Anova

Summary of Fit

Rsquare	0.759698
Adj Rsquare	0.719648
Root Mean Square Error	0.339178
Mean of Response	2.78e-11
Observations (or Sum Wgts)	36

Analysis of Variance

Source	DF	Sum of Squares	Mean Square	F Ratio	Prob > F
Label	5	10.910873	2.18217	18.9686	<.0001 *
Error	30	3.451244	0.11504		
C. Total	35	14.362117			

Means for Oneway Anova

Level	Number	Mean	Std Error	Lower 95%	Upper 95%
50nM-NA	6	0.90278	0.13847	0.6200	1.186
5nM-NA	6	0.58629	0.13847	0.3035	0.869
-NA	6	-0.55392	0.13847	-0.8367	-0.271
50nM+NA	6	-0.27354	0.13847	-0.5563	0.00925
5nM+NA	6	-0.15871	0.13847	-0.4415	0.124
+NA	6	-0.50290	0.13847	-0.7857	-0.220

Std Error uses a pooled estimate of error variance

Means Comparisons

Comparisons with a control using Dunnett's Method

Oneway Analysis of tyrosine By Label

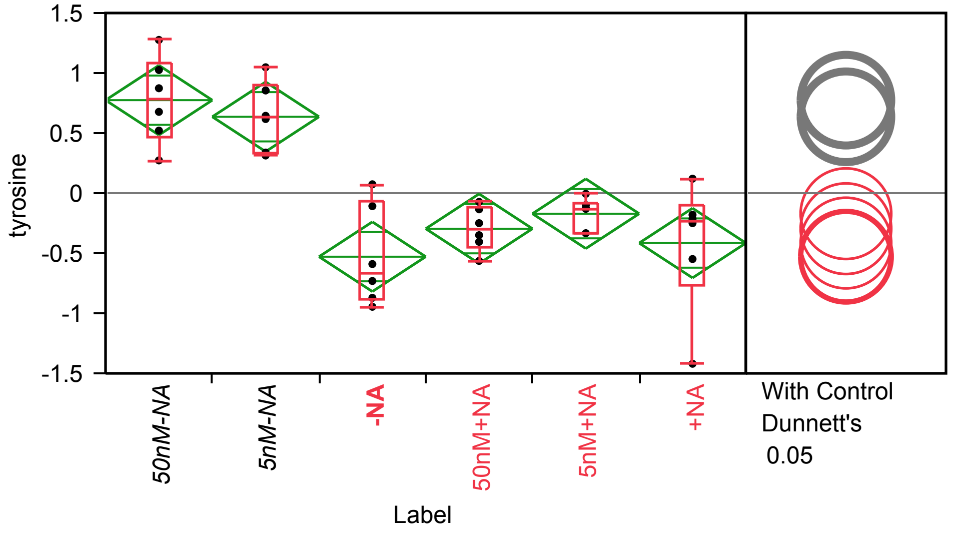

Quantiles

Level	Minimum	10%	25%	Median	75%	90%	Maximum
50nM-NA	0.272972	0.272972	0.45913	0.775351	1.088012	1.275921	1.275921
5nM-NA	0.313714	0.313714	0.331979	0.630705	0.903382	1.048178	1.048178
-NA	-0.94571	-0.94571	-0.89002	-0.66056	-0.06241	0.073837	0.073837
50nM+NA	-0.56387	-0.56387	-0.4446	-0.29976	-0.11875	-0.07241	-0.07241
5nM+NA	-0.33529	-0.33529	-0.3337	-0.12613	-0.07527	-0.00557	-0.00557
+NA	-1.42011	-1.42011	-0.76622	-0.23147	-0.10479	0.120443	0.120443

Oneway Anova

Summary of Fit

Rsquare	0.722226
Adj Rsquare	0.675931
Root Mean Square Error	0.347821
Mean of Response	-1.1e-10
Observations (or Sum Wgts)	36

Analysis of Variance

Source	DF	Sum of Squares	Mean Square	F Ratio	Prob > F
Label	5	9.436606	1.88732	15.6003	<.0001 *
Error	30	3.629393	0.12098		
C. Total	35	13.065999			

Means for Oneway Anova

Level	Number	Mean	Std Error	Lower 95%	Upper 95%
50nM-NA	6	0.77436	0.14200	0.4844	1.064
5nM-NA	6	0.63608	0.14200	0.3461	0.926
-NA	6	-0.52871	0.14200	-0.8187	-0.239
50nM+NA	6	-0.29581	0.14200	-0.5858	-0.0058
5nM+NA	6	-0.17080	0.14200	-0.4608	0.119
+NA	6	-0.41512	0.14200	-0.7051	-0.125

Std Error uses a pooled estimate of error variance

Means Comparisons

Comparisons with a control using Dunnett's Method

Oneway Analysis of UDP By Label

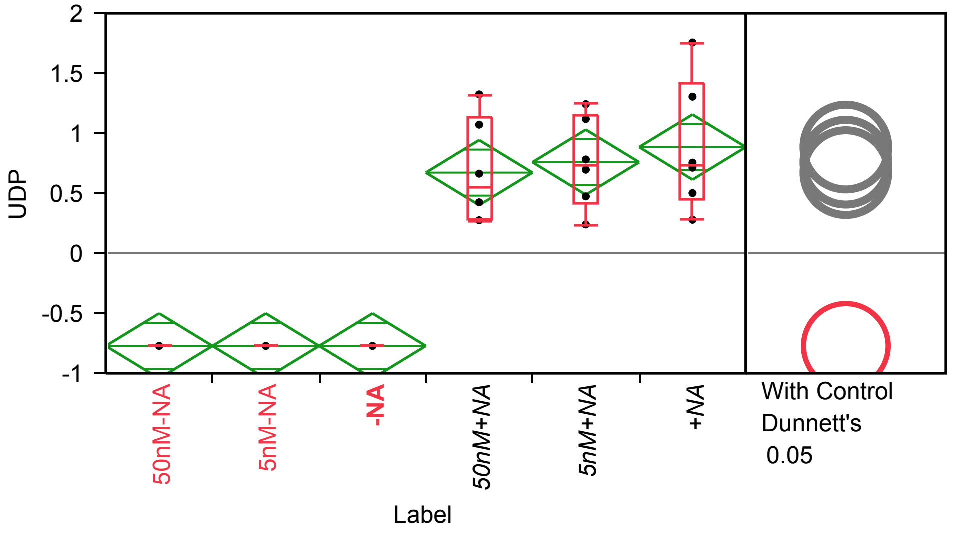

Quantiles

Level	Minimum	10%	25%	Median	75%	90%	Maximum
50nM-NA	-0.7721	-0.7721	-0.77209	-0.77208	-0.77208	-0.77207	-0.77207
5nM-NA	-0.7721	-0.7721	-0.7721	-0.77209	-0.77208	-0.77208	-0.77208
-NA	-0.77211	-0.77211	-0.7721	-0.77209	-0.77209	-0.77209	-0.77209
50nM+NA	0.274733	0.274733	0.27507	0.544072	1.134691	1.323925	1.323925
5nM+NA	0.240309	0.240309	0.415629	0.739233	1.149639	1.242242	1.242242
+NA	0.279399	0.279399	0.445966	0.734244	1.417547	1.756089	1.756089

Oneway Anova

Summary of Fit

Rsquare	0.871873
Adj Rsquare	0.850518
Root Mean Square Error	0.325266
Mean of Response	7.4e-17
Observations (or Sum Wgts)	36

Analysis of Variance

Source	DF	Sum of Squares	Mean Square	F Ratio	Prob > F
Label	5	21.597773	4.31955	40.8284	<.0001 *
Error	30	3.173934	0.10580		
C. Total	35	24.771707			

Means for Oneway Anova

Level	Number	Mean	Std Error	Lower 95%	Upper 95%
50nM-NA	6	-0.77208	0.13279	-1.043	-0.501
5nM-NA	6	-0.77209	0.13279	-1.043	-0.501
-NA	6	-0.77210	0.13279	-1.043	-0.501
50nM+NA	6	0.67227	0.13279	0.401	0.943
5nM+NA	6	0.75898	0.13279	0.488	1.030
+NA	6	0.88503	0.13279	0.614	1.156

Std Error uses a pooled estimate of error variance

Means Comparisons

Comparisons with a control using Dunnett's Method

Oneway Analysis of UDP-D-glucuronate By Label

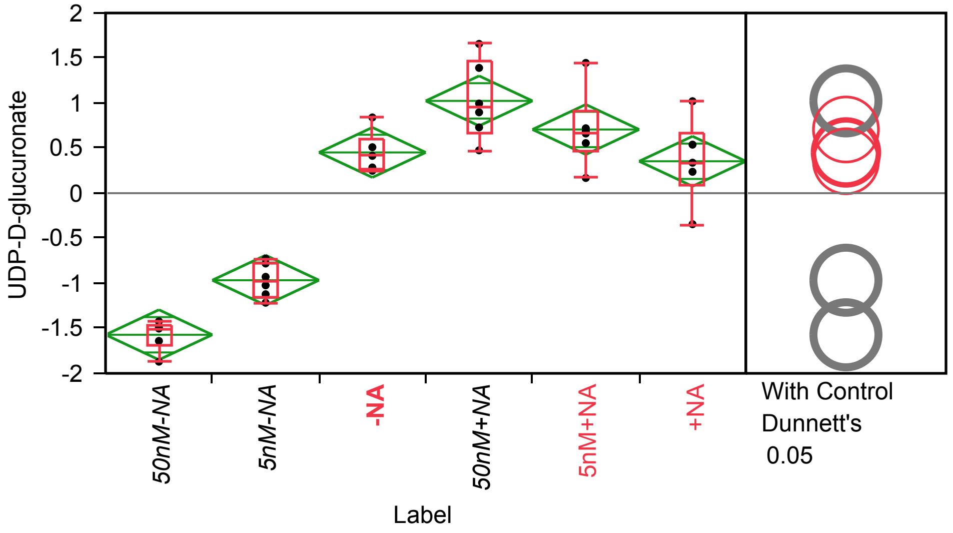

Quantiles

Level	Minimum	10%	25%	Median	75%	90%	Maximum
50nM-NA	-1.87012	-1.87012	-1.69766	-1.50432	-1.47281	-1.42242	-1.42242
5nM-NA	-1.21579	-1.21579	-1.14555	-0.97581	-0.7672	-0.72696	-0.72696
-NA	0.248811	0.248811	0.276075	0.414149	0.592813	0.840767	0.840767
50nM+NA	0.475666	0.475666	0.666557	0.946561	1.457302	1.657119	1.657119
5nM+NA	0.168882	0.168882	0.459183	0.675358	0.901938	1.445967	1.445967
+NA	-0.34445	-0.34445	0.092041	0.33671	0.659242	1.020738	1.020738

Oneway Anova

Summary of Fit

Rsquare	0.905151
Adj Rsquare	0.889342
Root Mean Square Error	0.332764
Mean of Response	4.93e-17
Observations (or Sum Wgts)	36

Analysis of Variance

Source	DF	Sum of Squares	Mean Square	F Ratio	Prob > F
Label	5	31.701593	6.34032	57.2582	<.0001 *
Error	30	3.321963	0.11073		
C. Total	35	35.023557			

Means for Oneway Anova

Level	Number	Mean	Std Error	Lower 95%	Upper 95%
50nM-NA	6	-1.5718	0.13585	-1.849	-1.294
5nM-NA	6	-0.9662	0.13585	-1.244	-0.689
-NA	6	0.4522	0.13585	0.175	0.730
50nM+NA	6	1.0245	0.13585	0.747	1.302
5nM+NA	6	0.7070	0.13585	0.430	0.984
+NA	6	0.3543	0.13585	0.077	0.632

Std Error uses a pooled estimate of error variance

Means Comparisons

Comparisons with a control using Dunnett's Method

Oneway Analysis of UDP-glucose By Label

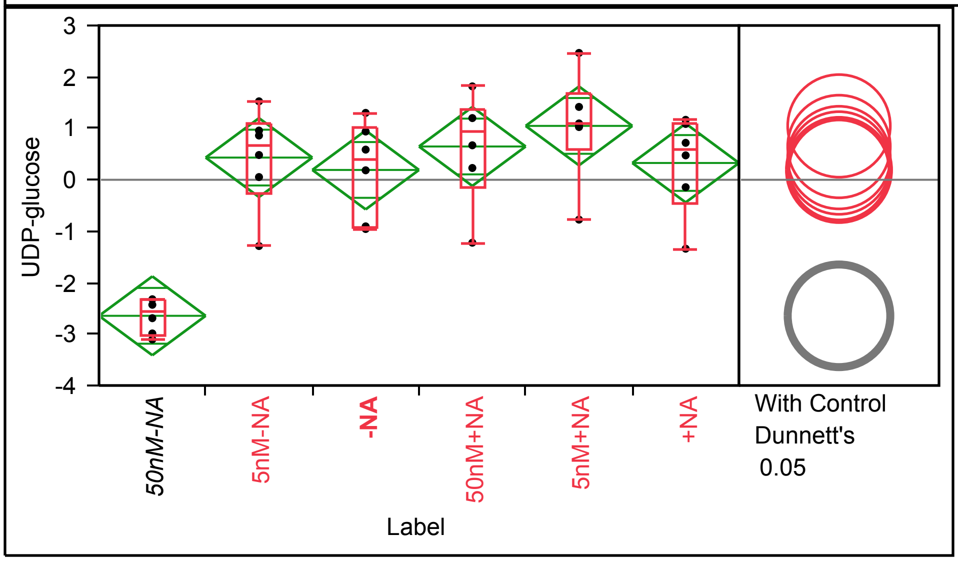

Quantiles

Level	Minimum	10%	25%	Median	75%	90%	Maximum
50nM-NA	-3.1041	-3.1041	-3.01702	-2.56067	-2.33103	-2.32291	-2.32291
5nM-NA	-1.28756	-1.28756	-0.28214	0.671244	1.09909	1.525452	1.525452
-NA	-0.95681	-0.95681	-0.91875	0.385407	1.029249	1.300876	1.300876
50nM+NA	-1.22321	-1.22321	-0.13382	0.931204	1.353874	1.816332	1.816332
5nM+NA	-0.7742	-0.7742	0.575758	1.075966	1.679213	2.466466	2.466466
+NA	-1.34007	-1.34007	-0.44318	0.596715	1.106029	1.16558	1.16558

Oneway Anova

Summary of Fit

Rsquare	0.676489
Adj Rsquare	0.62257
Root Mean Square Error	0.919659
Mean of Response	-2.8e-10
Observations (or Sum Wgts)	36

Analysis of Variance

Source	DF	Sum of Squares	Mean Square	F Ratio	Prob > F
Label	5	53.057362	10.6115	12.5465	<.0001 *
Error	30	25.373169	0.8458		
C. Total	35	78.430531			

Means for Oneway Anova

Level	Number	Mean	Std Error	Lower 95%	Upper 95%
50nM-NA	6	-2.6450	0.37545	-3.412	-1.878
5nM-NA	6	0.4317	0.37545	-0.335	1.198
-NA	6	0.1913	0.37545	-0.576	0.958
50nM+NA	6	0.6474	0.37545	-0.119	1.414
5nM+NA	6	1.0478	0.37545	0.281	1.815
+NA	6	0.3268	0.37545	-0.440	1.094

Std Error uses a pooled estimate of error variance

Means Comparisons

Comparisons with a control using Dunnett's Method

Oneway Analysis of UDP-N-acetyl-glucosamine By Label

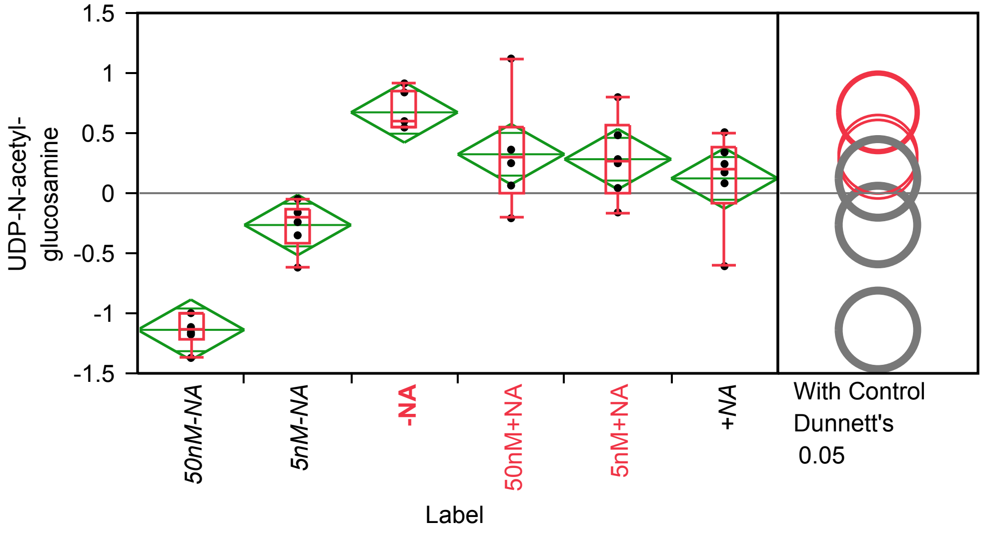

Quantiles

Level	Minimum	10%	25%	Median	75%	90%	Maximum
50nM-NA	-1.37157	-1.37157	-1.22497	-1.13759	-1.00388	-0.99849	-0.99849
5nM-NA	-0.61915	-0.61915	-0.41803	-0.20509	-0.13322	-0.04849	-0.04849
-NA	0.542175	0.542175	0.545475	0.59807	0.857917	0.913982	0.913982
50nM+NA	-0.20831	-0.20831	-0.00427	0.302848	0.551703	1.120617	1.120617
5nM+NA	-0.16024	-0.16024	-0.00796	0.266409	0.56171	0.799039	0.799039
+NA	-0.60777	-0.60777	-0.08996	0.208083	0.381867	0.507262	0.507262

Oneway Anova

Summary of Fit

Rsquare	0.816201
Adj Rsquare	0.785567
Root Mean Square Error	0.301471
Mean of Response	5.56e-10
Observations (or Sum Wgts)	36

Analysis of Variance

Source	DF	Sum of Squares	Mean Square	F Ratio	Prob > F
Label	5	12.107805	2.42156	26.6443	<.0001 *
Error	30	2.726546	0.09088		
C. Total	35	14.834350			

Means for Oneway Anova

Level	Number	Mean	Std Error	Lower 95%	Upper 95%
50nM-NA	6	-1.1378	0.12308	-1.389	-0.8865
5nM-NA	6	-0.2650	0.12308	-0.516	-0.0137
-NA	6	0.6730	0.12308	0.422	0.9244
50nM+NA	6	0.3240	0.12308	0.073	0.5753
5nM+NA	6	0.2828	0.12308	0.031	0.5342
+NA	6	0.1231	0.12308	-0.128	0.3744

Std Error uses a pooled estimate of error variance

Means Comparisons

Comparisons with a control using Dunnett's Method

Oneway Analysis of uracil By Label

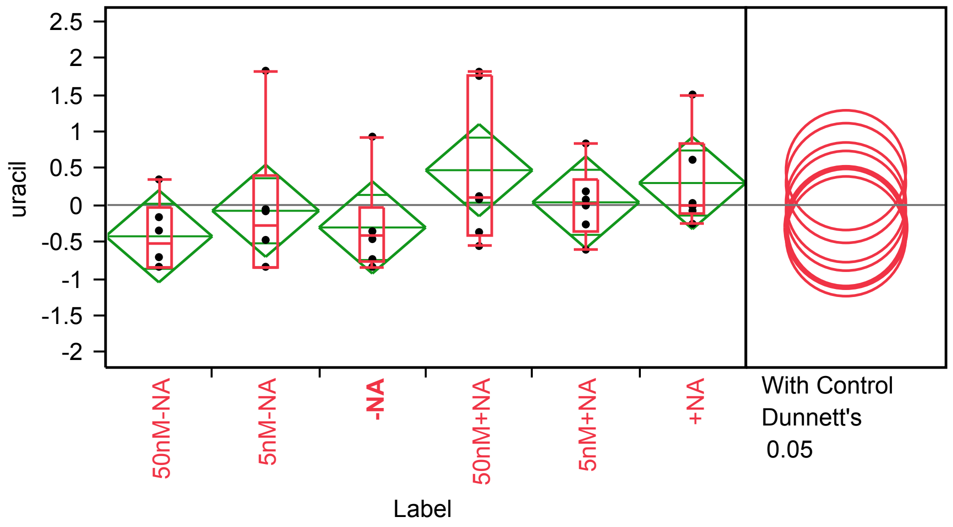

Quantiles

Level	Minimum	10%	25%	Median	75%	90%	Maximum
50nM-NA	-0.83924	-0.83924	-0.83923	-0.5275	-0.0366	0.347807	0.347807
5nM-NA	-0.83925	-0.83925	-0.83923	-0.27934	0.41313	1.823905	1.823905
-NA	-0.83924	-0.83924	-0.76117	-0.41365	-0.03544	0.928344	0.928344
50nM+NA	-0.55636	-0.55636	-0.41502	0.09936	1.768363	1.810266	1.810266
5nM+NA	-0.60631	-0.60631	-0.3493	0.033744	0.348051	0.836187	0.836187
+NA	-0.25527	-0.25527	-0.12155	0.004902	0.836047	1.50049	1.50049

Oneway Anova

Summary of Fit

Rsquare	0.174571
Adj Rsquare	0.037
Root Mean Square Error	0.749706
Mean of Response	2.78e-10
Observations (or Sum Wgts)	36

Analysis of Variance

Source	DF	Sum of Squares	Mean Square	F Ratio	Prob > F
Label	5	3.566121	0.713224	1.2689	0.3030
Error	30	16.861766	0.562059		
C. Total	35	20.427887			

Means for Oneway Anova

Level	Number	Mean	Std Error	Lower 95%	Upper 95%
50nM-NA	6	-0.42506	0.30607	-1.050	0.2000
5nM-NA	6	-0.07840	0.30607	-0.703	0.5467
-NA	6	-0.30501	0.30607	-0.930	0.3201
50nM+NA	6	0.47318	0.30607	-0.152	1.0983
5nM+NA	6	0.03651	0.30607	-0.589	0.6616
+NA	6	0.29877	0.30607	-0.326	0.9238

Std Error uses a pooled estimate of error variance

Means Comparisons

Comparisons with a control using Dunnett's Method

Oneway Analysis of Uric acid By Label

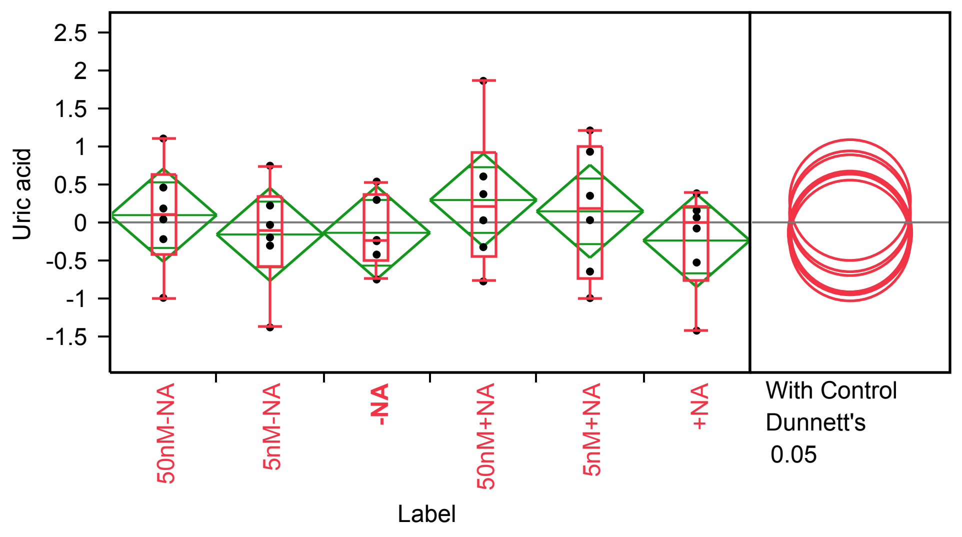

Quantiles

Level	Minimum	10%	25%	Median	75%	90%	Maximum
50nM-NA	-0.99156	-0.99156	-0.41331	0.110983	0.618757	1.101647	1.101647
5nM-NA	-1.37976	-1.37976	-0.57429	-0.11579	0.352343	0.74423	0.74423
-NA	-0.75027	-0.75027	-0.50519	-0.23936	0.355379	0.535605	0.535605
50nM+NA	-0.77588	-0.77588	-0.43802	0.199331	0.91715	1.859573	1.859573
5nM+NA	-0.99541	-0.99541	-0.73387	0.189207	0.998261	1.207734	1.207734
+NA	-1.42293	-1.42293	-0.75147	-0.00815	0.212532	0.381844	0.381844

Oneway Anova

Summary of Fit

Rsquare	0.074901
Adj Rsquare	-0.07928
Root Mean Square Error	0.731786
Mean of Response	8.33e-10
Observations (or Sum Wgts)	36

Analysis of Variance

Source	DF	Sum of Squares	Mean Square	F Ratio	Prob > F
Label	5	1.300745	0.260149	0.4858	0.7841
Error	30	16.065342	0.535511		
C. Total	35	17.366087			

Means for Oneway Anova

Level	Number	Mean	Std Error	Lower 95%	Upper 95%
50nM-NA	6	0.09488	0.29875	-0.5153	0.70501
5nM-NA	6	-0.15853	0.29875	-0.7687	0.45160
-NA	6	-0.13693	0.29875	-0.7471	0.47320
50nM+NA	6	0.29333	0.29875	-0.3168	0.90346
5nM+NA	6	0.14541	0.29875	-0.4647	0.75554
+NA	6	-0.23816	0.29875	-0.8483	0.37197

Std Error uses a pooled estimate of error variance

Means Comparisons

Comparisons with a control using Dunnett's Method

Oneway Analysis of UTP By Label

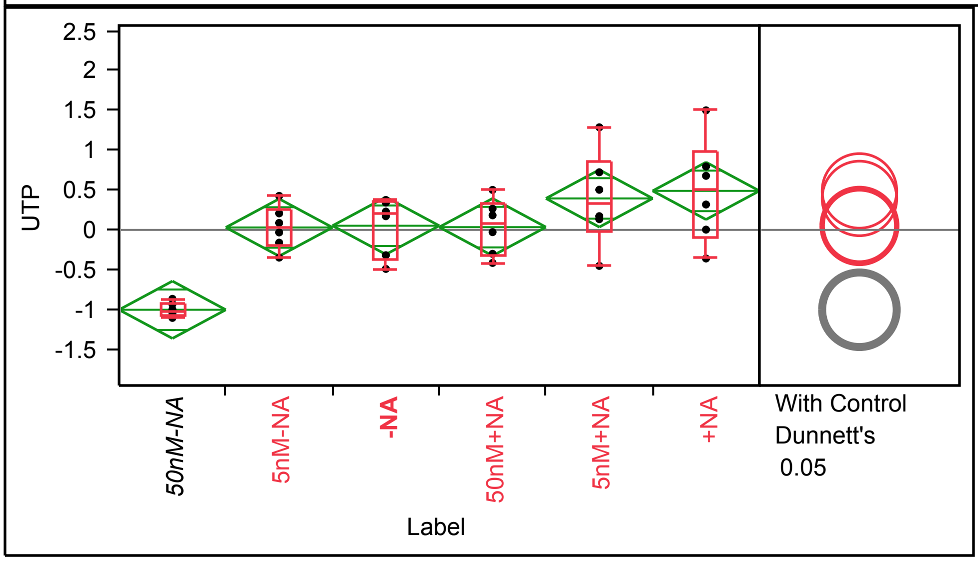

Quantiles

Level	Minimum	10%	25%	Median	75%	90%	Maximum
50nM-NA	-1.10348	-1.10348	-1.07892	-1.0193	-0.91572	-0.8648	-0.8648
5nM-NA	-0.34738	-0.34738	-0.20705	0.027993	0.262369	0.42664	0.42664
-NA	-0.48901	-0.48901	-0.36057	0.200817	0.350773	0.37541	0.37541
50nM+NA	-0.41397	-0.41397	-0.32861	0.077627	0.325077	0.501518	0.501518
5nM+NA	-0.45123	-0.45123	-0.01161	0.337807	0.863927	1.286805	1.286805
+NA	-0.35858	-0.35858	-0.08716	0.498364	0.971701	1.499718	1.499718

Oneway Anova

Summary of Fit

Rsquare	0.601807
Adj Rsquare	0.535442
Root Mean Square Error	0.431007
Mean of Response	5.56e-11
Observations (or Sum Wgts)	36

Analysis of Variance

Source	DF	Sum of Squares	Mean Square	F Ratio	Prob > F
Label	5	8.422751	1.68455	9.0681	<.0001 *
Error	30	5.573009	0.18577		
C. Total	35	13.995761			

Means for Oneway Anova

Level	Number	Mean	Std Error	Lower 95%	Upper 95%
50nM-NA	6	-1.0017	0.17596	-1.361	-0.6424
5nM-NA	6	0.0304	0.17596	-0.329	0.3898
-NA	6	0.0521	0.17596	-0.307	0.4115
50nM+NA	6	0.0348	0.17596	-0.325	0.3942
5nM+NA	6	0.3948	0.17596	0.035	0.7542
+NA	6	0.4895	0.17596	0.130	0.8488

Std Error uses a pooled estimate of error variance

Means Comparisons

Comparisons with a control using Dunnett's Method

Oneway Analysis of valine By Label

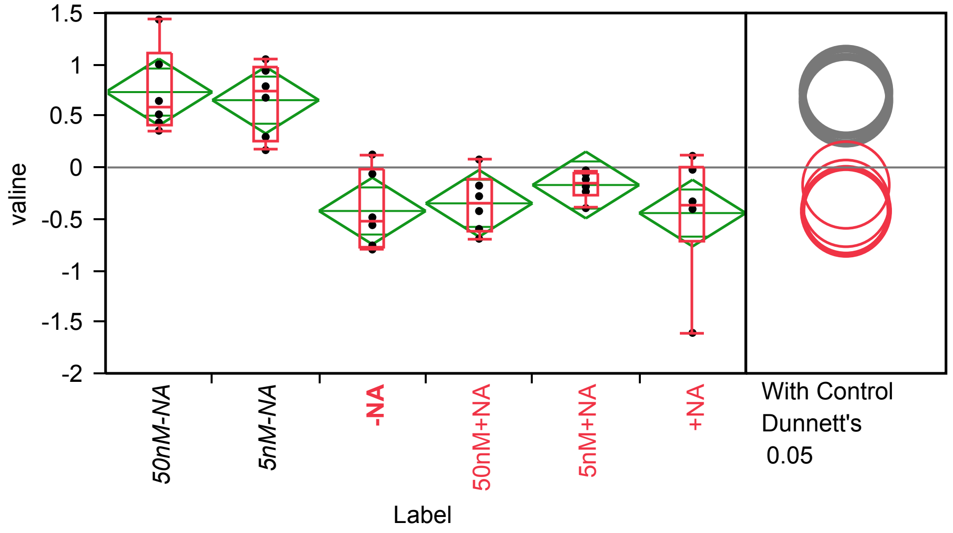

Quantiles

Level	Minimum	10%	25%	Median	75%	90%	Maximum
50nM-NA	0.357127	0.357127	0.416385	0.580466	1.109769	1.436303	1.436303
5nM-NA	0.168539	0.168539	0.265037	0.732806	0.966652	1.050723	1.050723
-NA	-0.79695	-0.79695	-0.76772	-0.52242	-0.01569	0.12531	0.12531
50nM+NA	-0.69245	-0.69245	-0.62138	-0.35157	-0.11309	0.07684	0.07684
5nM+NA	-0.39486	-0.39486	-0.27465	-0.15036	-0.05552	-0.02862	-0.02862
+NA	-1.60656	-1.60656	-0.70788	-0.36709	0.011589	0.112067	0.112067

Oneway Anova

Summary of Fit

Rsquare	0.663929
Adj Rsquare	0.607918
Root Mean Square Error	0.388205
Mean of Response	0
Observations (or Sum Wgts)	36

Analysis of Variance

Source	DF	Sum of Squares	Mean Square	F Ratio	Prob > F
Label	5	8.931719	1.78634	11.8534	<.0001 *
Error	30	4.521096	0.15070		
C. Total	35	13.452815			

Means for Oneway Anova

Level	Number	Mean	Std Error	Lower 95%	Upper 95%
50nM-NA	6	0.73190	0.15848	0.4082	1.056
5nM-NA	6	0.65345	0.15848	0.3298	0.977
-NA	6	-0.42285	0.15848	-0.7465	-0.099
50nM+NA	6	-0.34881	0.15848	-0.6725	-0.025
5nM+NA	6	-0.17055	0.15848	-0.4942	0.153
+NA	6	-0.44315	0.15848	-0.7668	-0.119

Std Error uses a pooled estimate of error variance

Means Comparisons

Comparisons with a control using Dunnett's Method

Oneway Analysis of xanthine By Label

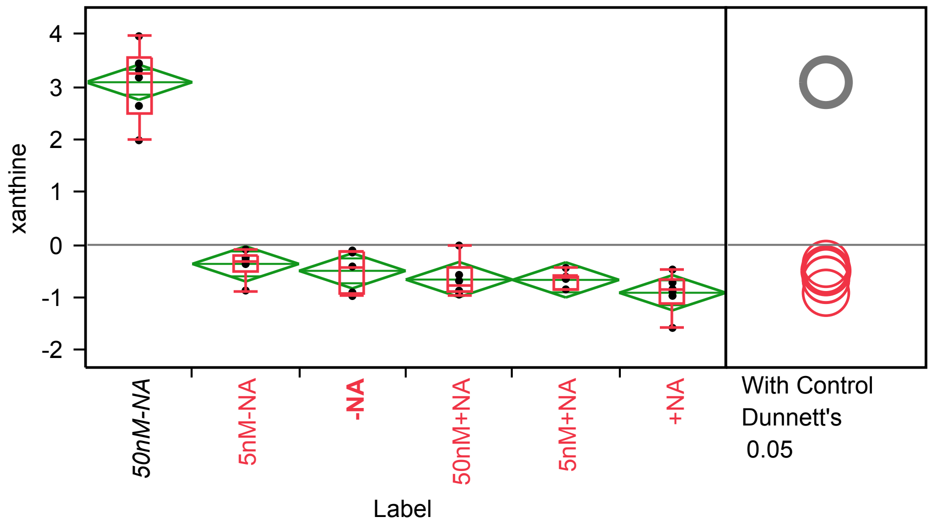

Quantiles

Level	Minimum	10%	25%	Median	75%	90%	Maximum
50nM-NA	1.982983	1.982983	2.471661	3.243654	3.568356	3.955351	3.955351
5nM-NA	-0.86571	-0.86571	-0.48907	-0.30301	-0.20293	-0.08825	-0.08825
-NA	-0.96994	-0.96994	-0.91872	-0.41515	-0.13511	-0.10755	-0.10755
50nM+NA	-0.94265	-0.94265	-0.88903	-0.7761	-0.4306	-0.01298	-0.01298
5nM+NA	-0.84534	-0.84534	-0.84457	-0.6265	-0.56196	-0.43783	-0.43783
+NA	-1.57404	-1.57404	-1.11799	-0.86227	-0.65382	-0.46831	-0.46831

Oneway Anova

Summary of Fit

Rsquare	0.9355
Adj Rsquare	0.92475
Root Mean Square Error	0.39956
Mean of Response	2.78e-10
Observations (or Sum Wgts)	36

Analysis of Variance

Source	DF	Sum of Squares	Mean Square	F Ratio	Prob > F
Label	5	69.465675	13.8931	87.0235	<.0001 *
Error	30	4.789445	0.1596		
C. Total	35	74.255119			

Means for Oneway Anova

Level	Number	Mean	Std Error	Lower 95%	Upper 95%
50nM-NA	6	3.0833	0.16312	2.750	3.416
5nM-NA	6	-0.3608	0.16312	-0.694	-0.028
-NA	6	-0.4923	0.16312	-0.825	-0.159
50nM+NA	6	-0.6581	0.16312	-0.991	-0.325
5nM+NA	6	-0.6640	0.16312	-0.997	-0.331
+NA	6	-0.9081	0.16312	-1.241	-0.575

Std Error uses a pooled estimate of error variance

Means Comparisons

Comparisons with a control using Dunnett's Method

Oneway Analysis of Xanthurenic acid By Label

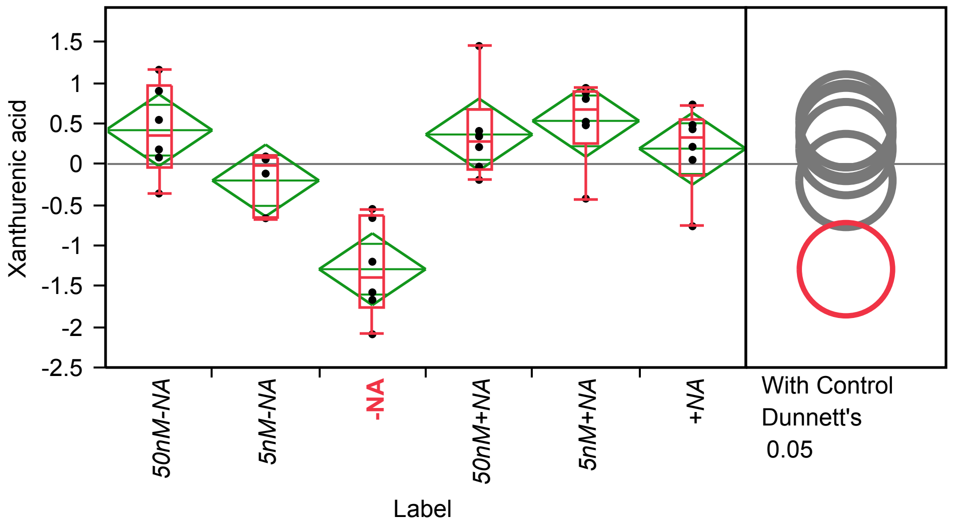

Quantiles

Level	Minimum	10%	25%	Median	75%	90%	Maximum
50nM-NA	-0.36246	-0.36246	-0.03097	0.359837	0.962106	1.156949	1.156949
5nM-NA	-0.67065	-0.67065	-0.66742	-0.03056	0.088682	0.092123	0.092123
-NA	-2.09172	-2.09172	-1.77479	-1.38901	-0.6357	-0.55303	-0.55303
50nM+NA	-0.19477	-0.19477	-0.07372	0.273458	0.666224	1.44923	1.44923
5nM+NA	-0.42559	-0.42559	0.25038	0.659068	0.889905	0.933594	0.933594
+NA	-0.76487	-0.76487	-0.15373	0.31898	0.542044	0.729834	0.729834

Oneway Anova

Summary of Fit

Rsquare	0.625541
Adj Rsquare	0.563131
Root Mean Square Error	0.528324
Mean of Response	2.78e-10
Observations (or Sum Wgts)	36

Analysis of Variance

Source	DF	Sum of Squares	Mean Square	F Ratio	Prob > F
Label	5	13.988540	2.79771	10.0231	<.0001 *
Error	30	8.373784	0.27913		
C. Total	35	22.362323			

Means for Oneway Anova

Level	Number	Mean	Std Error	Lower 95%	Upper 95%
50nM-NA	6	0.4151	0.21569	-0.025	0.8556
5nM-NA	6	-0.2031	0.21569	-0.644	0.2374
-NA	6	-1.2925	0.21569	-1.733	-0.8520
50nM+NA	6	0.3622	0.21569	-0.078	0.8027
5nM+NA	6	0.5295	0.21569	0.089	0.9700
+NA	6	0.1887	0.21569	-0.252	0.6292

Std Error uses a pooled estimate of error variance

Means Comparisons

Comparisons with a control using Dunnett's Method

Oneway Analysis of 1-Aminocyclopropanecarboxylic acid By Label

Means Comparisons

Comparisons with a control using Dunnett's Method

Control Group = -NA

|d|	Alpha
2.65691	0.05

Level	Abs(Dif)-LSD	p-Value	
50nM-NA	0.809	<.0001	*
5nM-NA	0.458	0.0001	*
5nM+NA	-0.44	0.9897	
-NA	-0.53	1.0000	
50nM+NA	-0.49	0.9998	
+NA	0.063	0.0240	*

Positive values show pairs of means that are significantly different.

Oneway Analysis of 1-Hexadecyl-2-arachidonoyl-glycerol By Label

Means Comparisons

Comparisons with a control using Dunnett's Method

Control Group = -NA

|d|	Alpha
2.65691	0.05

Level	Abs(Dif)-LSD	p-Value	
50nM-NA	0.124	0.0294	*
5nM-NA	-0.41	0.2364	
50nM+NA	-0.76	0.6128	
5nM+NA	-0.92	0.8100	
+NA	-1.26	0.9982	
-NA	-1.42	1.0000	

Positive values show pairs of means that are significantly different.

Oneway Analysis of 1-Methyladenosine By Label

Means Comparisons

Comparisons with a control using Dunnett's Method

Control Group = -NA

|d|	Alpha
2.65691	0.05

Level	Abs(Dif)-LSD	p-Value	
50nM-NA	0.073	0.0231	*
5nM-NA	0.049	0.0297	*
5nM+NA	-0.57	1.0000	
-NA	-0.58	1.0000	
50nM+NA	-0.53	0.9995	
+NA	-0.31	0.6018	

Positive values show pairs of means that are significantly different.

Oneway Analysis of 1-Methyl-Histidine By Label

Means Comparisons

Comparisons with a control using Dunnett's Method

Control Group = -NA

|d|	Alpha
2.65691	0.05

Level	Abs(Dif)-LSD	p-Value
50nM-NA	-0.13	0.1323
5nM-NA	-0.18	0.1892
-NA	-0.75	1.0000
5nM+NA	-0.5	0.8367
50nM+NA	-0.4	0.6072
+NA	-0.35	0.4800

Positive values show pairs of means that are significantly different.

Oneway Analysis of 2-Aminooctanoic acid By Label

Means Comparisons

Comparisons with a control using Dunnett's Method

Control Group = -NA

|d|	Alpha
2.65691	0.05

Level	Abs(Dif)-LSD	p-Value	
50nM-NA	1.001	<.0001	*
5nM-NA	0.677	<.0001	*
5nM+NA	-0.44	0.8529	
-NA	-0.66	1.0000	
50nM+NA	-0.59	0.9992	
+NA	-0.53	0.9806	

Positive values show pairs of means that are significantly different.

Oneway Analysis of 2-deoxyglucose-6-phosphate By Label

Means Comparisons

Comparisons with a control using Dunnett's Method

Control Group = -NA

|d|	Alpha
2.65691	0.05

Level	Abs(Dif)-LSD	p-Value	
-NA	-0.7	1.0000	
50nM-NA	-0.62	0.9980	
5nM-NA	-0.38	0.6272	
+NA	1.802	<.0001	*
50nM+NA	1.84	<.0001	*
5nM+NA	1.85	<.0001	*

Positive values show pairs of means that are significantly different.

Oneway Analysis of 2-Hydroxy-2-methylbutanedioic acid By Label

Means Comparisons

Comparisons with a control using Dunnett's Method

Control Group = -NA

|d|	Alpha
2.65691	0.05

Level	Abs(Dif)-LSD	p-Value
5nM-NA	-0.41	0.3081
50nM+NA	-0.85	0.9126
5nM+NA	-1.04	0.9975
+NA	-1.07	0.9993
-NA	-1.18	1.0000
50nM-NA	-0.97	0.9865

Positive values show pairs of means that are significantly different.

Oneway Analysis of 2-hydroxyglutarate By Label

Means Comparisons

Comparisons with a control using Dunnett's Method

Control Group = -NA

|d|	Alpha
2.65691	0.05

Level	Abs(Dif)-LSD	p-Value	
-NA	-0.95	1.0000	
50nM+NA	-0.18	0.1405	
5nM+NA	0.23	0.0107	*
+NA	0.342	0.0048	*
5nM-NA	0.86	<.0001	*
50nM-NA	1.176	<.0001	*

Positive values show pairs of means that are significantly different.

Oneway Analysis of 2-Isopropylmalic acid By Label

Means Comparisons

Comparisons with a control using Dunnett's Method

Control Group = -NA

|d|	Alpha
2.65691	0.05

Level	Abs(Dif)-LSD	p-Value
50nM+NA	-0.25	0.1672
5nM+NA	-0.52	0.4889
50nM-NA	-0.95	0.9937
+NA	-1.04	0.9998
5nM-NA	-1.11	1.0000
-NA	-1.12	1.0000

Positive values show pairs of means that are significantly different.

Oneway Analysis of 2-keto-isovalerate By Label

Means Comparisons

Comparisons with a control using Dunnett's Method

Control Group = -NA

|d|	Alpha
2.65691	0.05

Level	Abs(Dif)-LSD	p-Value	
50nM-NA	1.058	<.0001	*
5nM-NA	0.114	0.0075	*
-NA	-0.38	1.0000	
+NA	-0.31	0.9767	
5nM+NA	-0.2	0.5992	
50nM+NA	-0.04	0.0968	

Positive values show pairs of means that are significantly different.

Oneway Analysis of 2-oxobutanoate By Label

Means Comparisons

Comparisons with a control using Dunnett's Method

Control Group = -NA

|d|	Alpha
2.65691	0.05

Level	Abs(Dif)-LSD	p-Value
5nM+NA	-0.55	0.8447
50nM-NA	-0.6	0.9205
-NA	-0.83	1.0000
50nM+NA	-0.81	1.0000
+NA	-0.79	1.0000
5nM-NA	-0.78	0.9999

Positive values show pairs of means that are significantly different.

Oneway Analysis of 3-phospho-serine By Label

Means Comparisons

Comparisons with a control using Dunnett's Method

Control Group = -NA

|d|	Alpha
2.65691	0.05

Level	Abs(Dif)-LSD	p-Value	
5nM-NA	0.282	0.0067	*
50nM-NA	0.227	0.0101	*
5nM+NA	-0.3	0.2912	
+NA	-0.47	0.5814	
50nM+NA	-0.49	0.6400	
-NA	-0.9	1.0000	

Positive values show pairs of means that are significantly different.

Oneway Analysis of 4-aminobutyrate By Label

Means Comparisons

Comparisons with a control using Dunnett's Method

Control Group = -NA

|d|	Alpha
2.65691	0.05

Level	Abs(Dif)-LSD	p-Value	
-NA	-0.95	1.0000	
50nM-NA	-0.75	0.9747	
5nM-NA	-0.49	0.5794	
5nM+NA	-0.44	0.4886	
50nM+NA	-0.36	0.3581	
+NA	0.1	0.0261	*

Positive values show pairs of means that are significantly different.

Oneway Analysis of 4-Aminomethylpyrimidine By Label

Means Comparisons

Comparisons with a control using Dunnett's Method

Control Group = -NA

|d|	Alpha
2.65691	0.05

Level	Abs(Dif)-LSD	p-Value
50nM-NA	2.906	<.0001 *
5nM-NA	2.215	<.0001 *
5nM+NA	-0.82	0.9853
-NA	-0.99	1.0000
50nM+NA	-0.89	0.9989
+NA	0.396	0.0037 *

Positive values show pairs of means that are significantly different.

Oneway Analysis of 4-Pyridoxic acid By Label

Means Comparisons

Comparisons with a control using Dunnett's Method

Control Group = -NA

|d|	Alpha
2.65691	0.05

Level	Abs(Dif)-LSD	p-Value
5nM-NA	-1.31	0.9999
+NA	-1.34	1.0000
-NA	-1.39	1.0000
50nM-NA	-1.23	0.9980
50nM+NA	-1.17	0.9901
5nM+NA	-1.15	0.9861

Positive values show pairs of means that are significantly different.

Oneway Analysis of 5,6-Dihydroxyindole-2-carboxylic acid By Label

Means Comparisons

Comparisons with a control using Dunnett's Method

Control Group = -NA

|d|	Alpha
2.65691	0.05

Level	Abs(Dif)-LSD	p-Value	
50nM-NA	5.988	<.0001	*
50nM+NA	4.248	<.0001	*
5nM-NA	1.672	<.0001	*
5nM+NA	1.488	<.0001	*
-NA	-0.57	1.0000	
+NA	-0.39	0.8765	

Positive values show pairs of means that are significantly different.

Oneway Analysis of 7-methylguanosine By Label

Means Comparisons

Comparisons with a control using Dunnett's Method

Control Group = -NA

|d|	Alpha
2.65691	0.05

Level	Abs(Dif)-LSD	p-Value
5nM-NA	-0.46	0.8820
50nM-NA	-0.6	0.9993
-NA	-0.66	1.0000
50nM+NA	-0.33	0.5515
5nM+NA	-0.22	0.2856
+NA	0.017	0.0430 *

Positive values show pairs of means that are significantly different.

Oneway Analysis of acetoacetate By Label

Means Comparisons

Comparisons with a control using Dunnett's Method

Control Group = -NA

|d|	Alpha
2.65691	0.05

Level	Abs(Dif)-LSD	p-Value	
5nM+NA	0.445	0.0016	*
+NA	-0.03	0.0623	
50nM+NA	-0.25	0.2310	
-NA	-0.87	1.0000	
50nM-NA	-0.56	0.8171	
5nM-NA	-0.25	0.2299	

Positive values show pairs of means that are significantly different.

Oneway Analysis of acetoacetyl-CoA By Label

Means Comparisons

Comparisons with a control using Dunnett's Method

Control Group = -NA

|d|	Alpha
2.65691	0.05

Level	Abs(Dif)-LSD	p-Value
50nM-NA	-0.34	0.5678
5nM-NA	-0.46	0.8633
-NA	-0.67	1.0000
+NA	1.11	<.0001 *
5nM+NA	1.12	<.0001 *
50nM+NA	1.286	<.0001 *

Positive values show pairs of means that are significantly different.

Oneway Analysis of Acetylcarnitine By Label

Means Comparisons

Comparisons with a control using Dunnett's Method

Control Group = -NA

|d|	Alpha
2.65691	0.05

Level	Abs(Dif)-LSD	p-Value	
50nM-NA	1.798	<.0001	*
5nM-NA	0.806	<.0001	*
50nM+NA	-0.38	0.9777	
5nM+NA	-0.39	0.9850	
-NA	-0.48	1.0000	
+NA	-0.2	0.4155	

Positive values show pairs of means that are significantly different.

Oneway Analysis of Acetylcholine By Label

Means Comparisons

Comparisons with a control using Dunnett's Method

Control Group = -NA

|d|	Alpha
2.65691	0.05

Level	Abs(Dif)-LSD	p-Value	
-NA	-0.8	1.0000	
5nM-NA	-0.61	0.9528	
50nM-NA	-0.58	0.9144	
5nM+NA	0.13	0.0183	*
+NA	0.181	0.0121	*
50nM+NA	1.14	<.0001	*

Positive values show pairs of means that are significantly different.

Oneway Analysis of acetyl-CoA By Label

Means Comparisons

Comparisons with a control using Dunnett's Method

Control Group = -NA

|d|	Alpha
2.65691	0.05

Level	Abs(Dif)-LSD	p-Value	
50nM-NA	0.609	0.0004	*
5nM-NA	-0.29	0.2831	
-NA	-0.86	1.0000	
5nM+NA	-0.78	0.9993	
50nM+NA	-0.57	0.8331	
+NA	-0.39	0.4646	

Positive values show pairs of means that are significantly different.

Oneway Analysis of Acetyllysine By Label

Means Comparisons

Comparisons with a control using Dunnett's Method

Control Group = -NA

|d|	Alpha
2.65691	0.05

Level	Abs(Dif)-LSD	p-Value	
50nM-NA	0.259	0.0021	*
5nM-NA	0.233	0.0030	*
-NA	-0.54	1.0000	
50nM+NA	-0.28	0.5637	
5nM+NA	-0.25	0.4790	
+NA	0.053	0.0276	*

Positive values show pairs of means that are significantly different.

Oneway Analysis of acetylphosphate By Label

Means Comparisons

Comparisons with a control using Dunnett's Method

Control Group = -NA

|d|	Alpha
2.65691	0.05

Level	Abs(Dif)-LSD	p-Value
50nM+NA	-0.21	0.1377
5nM+NA	-0.3	0.2018
+NA	-0.42	0.3314
5nM-NA	-0.48	0.3998
50nM-NA	-0.56	0.5198
-NA	-1.15	1.0000

Positive values show pairs of means that are significantly different.

Oneway Analysis of adenine By Label

Means Comparisons

Comparisons with a control using Dunnett's Method

Control Group = -NA

abs(d)	Alpha
2.65691	0.05

Level	Abs(Dif)-LSD	p-Value	
50nM-NA	0.77	<.0001	*
5nM-NA	0.516	<.0001	*
5nM+NA	-0.47	0.9956	
+NA	-0.51	0.9999	
50nM+NA	-0.53	1.0000	
-NA	-0.55	1.0000	

Positive values show pairs of means that are significantly different.

Oneway Analysis of adenosine By Label

Means Comparisons

Comparisons with a control using Dunnett's Method

Control Group = -NA

|d|	Alpha
2.65691	0.05

Level	Abs(Dif)-LSD	p-Value	
50nM-NA	0.711	0.0011	*
5nM-NA	0.432	0.0054	*
-NA	-1.26	1.0000	
5nM+NA	-1.05	0.9907	
50nM+NA	-1.01	0.9793	
+NA	-0.7	0.6539	

Positive values show pairs of means that are significantly different.

Oneway Analysis of Adipic acid By Label

Means Comparisons

Comparisons with a control using Dunnett's Method

Control Group = -NA

|d|	Alpha
2.65691	0.05

Level	Abs(Dif)-LSD	p-Value
-NA	-0.59	1.0000
5nM-NA	-0.19	0.2761
+NA	0.079	0.0217 *
5nM+NA	0.143	0.0107 *
50nM-NA	0.143	0.0107 *
50nM+NA	0.303	0.0016 *

Positive values show pairs of means that are significantly different.

Oneway Analysis of ADP By Label

Means Comparisons

Comparisons with a control using Dunnett's Method

Control Group = -NA

|d|	Alpha
2.65691	0.05

Level	Abs(Dif)-LSD	p-Value	
50nM-NA	0.378	0.0010	*
5nM-NA	0.308	0.0022	*
50nM+NA	-0.51	0.9644	
5nM+NA	-0.64	1.0000	
-NA	-0.66	1.0000	
+NA	-0.65	1.0000	

Positive values show pairs of means that are significantly different.

Oneway Analysis of AICAR 3,5-Cyclic Phosphate By Label

Means Comparisons

Comparisons with a control using Dunnett's Method

Control Group = -NA

|d|	Alpha
2.65691	0.05

Level	Abs(Dif)-LSD	p-Value	
50nM-NA	0.934	<.0001	*
5nM-NA	0.319	<.0001	*
-NA	-0.24	1.0000	
5nM+NA	-0.23	1.0000	
50nM+NA	-0.2	0.9834	
+NA	-0.18	0.9565	

Positive values show pairs of means that are significantly different.

Oneway Analysis of AICAR(ZMP) By Label

Means Comparisons

Comparisons with a control using Dunnett's Method

Control Group = -NA

|d|	Alpha
2.65691	0.05

Level	Abs(Dif)-LSD	p-Value	
5nM-NA	0.773	<.0001	*
50nM-NA	-0.1	0.8203	
+NA	-0.13	0.9983	
5nM+NA	-0.14	0.9999	
-NA	-0.15	1.0000	
50nM+NA	-0.13	0.9988	

Positive values show pairs of means that are significantly different.

Oneway Analysis of 2-ketoglutarate By Label

Means Comparisons

Comparisons with a control using Dunnett's Method

Control Group = -NA

|d|	Alpha
2.65691	0.05

Level	Abs(Dif)-LSD	p-Value
-NA	-1.09	1.0000
5nM+NA	-1.09	1.0000
+NA	-1.08	1.0000
5nM-NA	-0.85	0.9655
50nM-NA	-0.68	0.7719
50nM+NA	-0.52	0.5033

Positive values show pairs of means that are significantly different.

Oneway Analysis of Ala-Ala By Label

Means Comparisons

Comparisons with a control using Dunnett's Method

Control Group = -NA

|d|	Alpha
2.65691	0.05

Level	Abs(Dif)-LSD	p-Value
50nM-NA	-0.36	0.9980
-NA	-0.41	1.0000
5nM-NA	-0.27	0.8337
5nM+NA	-0.16	0.3755
50nM+NA	-0.16	0.3754
+NA	-0.16	0.3754

Positive values show pairs of means that are significantly different.

Oneway Analysis of Ala-Gln By Label

Means Comparisons

Comparisons with a control using Dunnett's Method

Control Group = -NA

|d|	Alpha
2.65691	0.05

Level	Abs(Dif)-LSD	p-Value	
5nM-NA	-0.18	0.5120	
-NA	-0.38	1.0000	
50nM-NA	-0.29	0.9445	
5nM+NA	0.012	0.0414	*
+NA	0.223	0.0010	*
50nM+NA	0.389	<.0001	*

Positive values show pairs of means that are significantly different.

Oneway Analysis of Ala-Gly By Label

Means Comparisons

Comparisons with a control using Dunnett's Method

Control Group = -NA

|d|	Alpha
2.65691	0.05

Level	Abs(Dif)-LSD	p-Value
50nM+NA	-0.28	0.2722
5nM+NA	-0.52	0.7236
5nM-NA	-0.59	0.8559
50nM-NA	-0.61	0.8878
-NA	-0.88	1.0000
+NA	-0.73	0.9881

Positive values show pairs of means that are significantly different.

Oneway Analysis of alanine By Label

Means Comparisons

Comparisons with a control using Dunnett's Method

Control Group = -NA

|d|	Alpha
2.65691	0.05

Level	Abs(Dif)-LSD	p-Value	
-NA	-0.87	1.0000	
5nM-NA	0.35	0.0036	*
50nM-NA	0.56	0.0006	*
5nM+NA	0.754	0.0001	*
50nM+NA	0.856	<.0001	*
+NA	0.876	<.0001	*

Positive values show pairs of means that are significantly different.

Oneway Analysis of allantoate By Label

Means Comparisons

Comparisons with a control using Dunnett's Method

Control Group = -NA

|d|	Alpha
2.65691	0.05

Level	Abs(Dif)-LSD	p-Value
-NA	-1.15	1.0000
50nM+NA	-1.1	1.0000
5nM+NA	-0.98	0.9940
+NA	-0.14	0.1000
5nM-NA	1.607	<.0001 *
50nM-NA	1.898	<.0001 *

Positive values show pairs of means that are significantly different.

Oneway Analysis of Aminoadipic acid By Label

Means Comparisons

Comparisons with a control using Dunnett's Method

Control Group = -NA

|d|	Alpha
2.65691	0.05

Level	Abs(Dif)-LSD	p-Value	
-NA	-1.04	1.0000	
50nM+NA	0.284	0.0087	*
+NA	0.435	0.0032	*
5nM+NA	0.563	0.0013	*
5nM-NA	0.597	0.0010	*
50nM-NA	1.341	<.0001	*

Positive values show pairs of means that are significantly different.

Oneway Analysis of AMP By Label

Means Comparisons

Comparisons with a control using Dunnett's Method

Control Group = -NA

|d|	Alpha
2.65691	0.05

Level	Abs(Dif)-LSD	p-Value	
50nM-NA	2.061	<.0001	*
5nM-NA	1.155	<.0001	*
-NA	-0.26	1.0000	
50nM+NA	-0.25	1.0000	
+NA	-0.17	0.8309	
5nM+NA	-0.15	0.7198	

Positive values show pairs of means that are significantly different.

Oneway Analysis of Arabitol By Label

Means Comparisons

Comparisons with a control using Dunnett's Method

Control Group = -NA

|d|	Alpha
2.65691	0.05

Level	Abs(Dif)-LSD	p-Value
5nM-NA	-1.41	0.4534
50nM-NA	-2.49	0.9692
+NA	-2.65	0.9901
50nM+NA	-2.68	0.9923
5nM+NA	-2.71	0.9946
-NA	-3.17	1.0000

Positive values show pairs of means that are significantly different.

Oneway Analysis of arginine By Label

Means Comparisons

Comparisons with a control using Dunnett's Method

Control Group = -NA

|d|	Alpha
2.65691	0.05

Level	Abs(Dif)-LSD	p-Value	
50nM-NA	0.521	0.0002	*
5nM-NA	-0.27	0.3973	
-NA	-0.66	1.0000	
5nM+NA	-0.47	0.9065	
50nM+NA	-0.35	0.6088	
+NA	-0.35	0.5940	

Positive values show pairs of means that are significantly different.

Oneway Analysis of asparagine By Label

Means Comparisons

Comparisons with a control using Dunnett's Method

Control Group = -NA

|d|	Alpha
2.65691	0.05

Level	Abs(Dif)-LSD	p-Value	
-NA	-1.05	1.0000	
50nM-NA	0.133	0.0229	*
5nM-NA	0.186	0.0166	*
50nM+NA	0.91	0.0001	*
5nM+NA	0.937	0.0001	*
+NA	0.966	<.0001	*

Positive values show pairs of means that are significantly different.

Oneway Analysis of aspartate By Label

Means Comparisons

Comparisons with a control using Dunnett's Method

Control Group = -NA

|d|	Alpha
2.65691	0.05

Level	Abs(Dif)-LSD	p-Value	
50nM-NA	1.575	<.0001	*
5nM-NA	0.54	<.0001	*
-NA	-0.23	1.0000	
+NA	0.103	0.0025	*
50nM+NA	0.107	0.0021	*
5nM+NA	0.134	0.0009	*

Positive values show pairs of means that are significantly different.

Oneway Analysis of ATP By Label

Means Comparisons

Comparisons with a control using Dunnett's Method

Control Group = -NA

|d|	Alpha
2.65691	0.05

Level	Abs(Dif)-LSD	p-Value
-NA	-0.93	1.0000
+NA	-0.93	1.0000
5nM+NA	-0.81	0.9968
50nM+NA	-0.66	0.8981
5nM-NA	-0.39	0.4176
50nM-NA	1.246	<.0001 *

Positive values show pairs of means that are significantly different.

Oneway Analysis of benzenesulfonamine By Label

Means Comparisons

Comparisons with a control using Dunnett's Method

Control Group = -NA

|d|	Alpha
2.65691	0.05

Level	Abs(Dif)-LSD	p-Value	
50nM-NA	1.707	<.0001	*
50nM+NA	1.209	<.0001	*
5nM-NA	0.004	0.0454	*
5nM+NA	-0.04	0.1314	
-NA	-0.22	1.0000	
+NA	-0.21	1.0000	

Positive values show pairs of means that are significantly different.

Oneway Analysis of Benzoic acid By Label

Means Comparisons

Comparisons with a control using Dunnett's Method

Control Group = -NA

|d|	Alpha
2.65691	0.05

Level	Abs(Dif)-LSD	p-Value
50nM-NA	-0.55	0.2655
5nM-NA	-0.98	0.6769
+NA	-1.03	0.7220
5nM+NA	-1.43	0.9881
-NA	-1.73	1.0000
50nM+NA	-1.61	0.9998

Positive values show pairs of means that are significantly different.

Oneway Analysis of beta-Alanine By Label

Means Comparisons

Comparisons with a control using Dunnett's Method

Control Group = -NA

|d|	Alpha
2.65691	0.05

Level	Abs(Dif)-LSD	p-Value
-NA	-0.2	1.0000
+NA	-0.18	0.9994
50nM-NA	-0.18	0.9989
50nM+NA	-0.17	0.9911
5nM+NA	-0.13	0.8028
5nM-NA	-0.02	0.0888

Positive values show pairs of means that are significantly different.

Oneway Analysis of beta-D-Glucopyranose By Label

Means Comparisons

Comparisons with a control using Dunnett's Method

Control Group = -NA

|d|	Alpha
2.65691	0.05

Level	Abs(Dif)-LSD	p-Value
-NA	-0.56	1.0000
5nM-NA	0.078	0.0211 *
5nM+NA	0.202	0.0048 *
50nM-NA	0.273	0.0019 *
+NA	0.287	0.0016 *
50nM+NA	0.359	0.0006 *

Positive values show pairs of means that are significantly different.

Oneway Analysis of betaine By Label

Means Comparisons

Comparisons with a control using Dunnett's Method

Control Group = -NA

|d|	Alpha
2.65691	0.05

Level	Abs(Dif)-LSD	p-Value	
5nM-NA	0.002	0.0494	*
+NA	-0.53	0.7870	
50nM-NA	-0.57	0.8722	
5nM+NA	-0.67	0.9785	
-NA	-0.84	1.0000	
50nM+NA	-0.83	1.0000	

Positive values show pairs of means that are significantly different.

Oneway Analysis of betaine aldehyde By Label

Means Comparisons

Comparisons with a control using Dunnett's Method

Control Group = -NA

|d|	Alpha
2.65691	0.05

Level	Abs(Dif)-LSD	p-Value	
5nM+NA	0.547	0.0056	*
+NA	-0.48	0.2467	
50nM+NA	-0.75	0.4841	
5nM-NA	-1.57	1.0000	
-NA	-1.62	1.0000	
50nM-NA	-1.2	0.9377	

Positive values show pairs of means that are significantly different.

Oneway Analysis of Butyrylcarnitine By Label

Means Comparisons

Comparisons with a control using Dunnett's Method

Control Group = -NA

|d|	Alpha
2.65691	0.05

Level	Abs(Dif)-LSD	p-Value	
50nM+NA	-0.36	1.0000	
-NA	-0.36	1.0000	
+NA	-0.24	0.8458	
5nM+NA	-0.1	0.2229	
5nM-NA	1.103	<.0001	*
50nM-NA	1.658	<.0001	*

Positive values show pairs of means that are significantly different.

Oneway Analysis of Carbamoyl phosphate By Label

Means Comparisons

Comparisons with a control using Dunnett's Method

Control Group = -NA

|d|	Alpha
2.65691	0.05

Level	Abs(Dif)-LSD	p-Value
5nM-NA	-0.71	0.4865
50nM+NA	-0.77	0.5582
5nM+NA	-1.01	0.8358
50nM-NA	-1.41	0.9998
-NA	-1.52	1.0000
+NA	-0.97	0.7952

Positive values show pairs of means that are significantly different.

Oneway Analysis of Carnitine By Label

Means Comparisons

Comparisons with a control using Dunnett's Method

Control Group = -NA

|d|	Alpha
2.65691	0.05

Level	Abs(Dif)-LSD	p-Value	
-NA	-0.77	1.0000	
5nM-NA	-0.31	0.3770	
+NA	0.278	0.0049	*
50nM+NA	0.348	0.0026	*
5nM+NA	0.71	<.0001	*
50nM-NA	0.982	<.0001	*

Positive values show pairs of means that are significantly different.

Oneway Analysis of CDP By Label

Means Comparisons

Comparisons with a control using Dunnett's Method

Control Group = -NA

|d|	Alpha
2.65691	0.05

Level	Abs(Dif)-LSD	p-Value	
50nM-NA	0.276	0.0010	*
5nM-NA	0.109	0.0118	*
-NA	-0.48	1.0000	
5nM+NA	0.678	<.0001	*
+NA	0.915	<.0001	*
50nM+NA	0.949	<.0001	*

Positive values show pairs of means that are significantly different.

Oneway Analysis of Chodroitin By Label

Means Comparisons

Comparisons with a control using Dunnett's Method

Control Group = -NA

|d|	Alpha
2.65691	0.05

Level	Abs(Dif)-LSD	p-Value	
5nM-NA	-2.7	0.7455	
-NA	-4.45	1.0000	
50nM-NA	-4.06	0.9995	
5nM+NA	-3.69	0.9882	
50nM+NA	-1.09	0.1896	
+NA	0.407	0.0286	*

Positive values show pairs of means that are significantly different.

Oneway Analysis of Cholesterol By Label

Means Comparisons

Comparisons with a control using Dunnett's Method

Control Group = -NA

|d|	Alpha
2.65691	0.05

Level	Abs(Dif)-LSD	p-Value
5nM-NA	-1.88	0.6161
5nM+NA	-3.03	0.9961
50nM+NA	-3.14	0.9988
-NA	-3.5	1.0000
50nM-NA	-2.39	0.8672
+NA	-1.6	0.4744

Positive values show pairs of means that are significantly different.

Oneway Analysis of cholesteryl sulfate By Label

Means Comparisons

Comparisons with a control using Dunnett's Method

Control Group = -NA

|d|	Alpha
2.65691	0.05

Level	Abs(Dif)-LSD	p-Value
50nM-NA	-0.03	0.0585
5nM-NA	-0.46	0.3529
5nM+NA	-0.63	0.5908
+NA	-0.76	0.7883
-NA	-1.2	1.0000
50nM+NA	-1.01	0.9918

Positive values show pairs of means that are significantly different.

Oneway Analysis of choline By Label

Means Comparisons

Comparisons with a control using Dunnett's Method

Control Group = -NA

|d|	Alpha
2.65691	0.05

Level	Abs(Dif)-LSD	p-Value	
50nM-NA	0.546	0.0001	*
-NA	-0.62	1.0000	
5nM-NA	-0.56	0.9990	
5nM+NA	0.523	0.0001	*
+NA	0.601	<.0001	*
50nM+NA	0.611	<.0001	*

Positive values show pairs of means that are significantly different.

Oneway Analysis of Citric acid By Label

Means Comparisons

Comparisons with a control using Dunnett's Method

Control Group = -NA

|d|	Alpha
2.65691	0.05

Level	Abs(Dif)-LSD	p-Value	
-NA	-2.99	1.0000	
5nM+NA	-0.92	0.2551	
50nM+NA	0.014	0.0486	*
+NA	0.191	0.0340	*
50nM-NA	6.195	<.0001	*
5nM-NA	7.28	<.0001	*

Positive values show pairs of means that are significantly different.

Oneway Analysis of citrulline By Label

Means Comparisons

Comparisons with a control using Dunnett's Method

Control Group = -NA

|d|	Alpha
2.65691	0.05

Level	Abs(Dif)-LSD	p-Value	
50nM+NA	0.421	0.0033	*
5nM-NA	-0.6	0.7217	
+NA	-0.61	0.7398	
5nM+NA	-0.99	1.0000	
-NA	-1.02	1.0000	
50nM-NA	-0.66	0.8212	

Positive values show pairs of means that are significantly different.

Oneway Analysis of CMP By Label

Means Comparisons

Comparisons with a control using Dunnett's Method

Control Group = -NA

|d|	Alpha
2.65691	0.05

Level	Abs(Dif)-LSD	p-Value
5nM-NA	-0.16	0.2165
50nM+NA	-0.4	0.8478
-NA	-0.59	1.0000
5nM+NA	-0.54	0.9994
50nM-NA	-0.41	0.8752
+NA	-0.36	0.7322

Positive values show pairs of means that are significantly different.

Oneway Analysis of CMP-N-acetylneuraminic acid By Label

Means Comparisons

Comparisons with a control using Dunnett's Method

Control Group = -NA

|d|	Alpha
2.65691	0.05

Level	Abs(Dif)-LSD	p-Value
+NA	0.107	0.0213 *
5nM+NA	-0.44	0.6705
-NA	-0.78	1.0000
50nM+NA	-0.29	0.3351
5nM-NA	-0.26	0.2830
50nM-NA	0.316	0.0035 *

Positive values show pairs of means that are significantly different.

Oneway Analysis of creatine By Label

Means Comparisons

Comparisons with a control using Dunnett's Method

Control Group = -NA

|d|	Alpha
2.65691	0.05

Level	Abs(Dif)-LSD	p-Value
50nM-NA	0.808	<.0001 *
5nM-NA	0.635	<.0001 *
50nM+NA	-0.62	1.0000
-NA	-0.63	1.0000
+NA	-0.61	1.0000
5nM+NA	-0.59	0.9998

Positive values show pairs of means that are significantly different.

Oneway Analysis of Creatinine By Label

Means Comparisons

Comparisons with a control using Dunnett's Method

Control Group = -NA

|d|	Alpha
2.65691	0.05

Level	Abs(Dif)-LSD	p-Value
50nM-NA	2.899	<.0001 *
5nM-NA	1.289	<.0001 *
-NA	-0.33	1.0000
50nM+NA	-0.18	0.6171
5nM+NA	-0.06	0.1275
+NA	-0.05	0.1152

Positive values show pairs of means that are significantly different.

Oneway Analysis of Cyclic ADP-ribose By Label

Means Comparisons

Comparisons with a control using Dunnett's Method

Control Group = -NA

|d|	Alpha
2.65691	0.05

Level	Abs(Dif)-LSD	p-Value	
+NA	1.147	<.0001	*
50nM+NA	0.536	0.0027	*
5nM+NA	0.529	0.0028	*
-NA	-1.21	1.0000	
5nM-NA	2.724	<.0001	*
50nM-NA	2.74	<.0001	*

Positive values show pairs of means that are significantly different.

Oneway Analysis of cyclic-AMP By Label

Means Comparisons

Comparisons with a control using Dunnett's Method

Control Group = -NA

|d|	Alpha
2.65691	0.05

Level	Abs(Dif)-LSD	p-Value
50nM-NA	-0.69	0.8336
-NA	-1.05	1.0000
5nM-NA	-0.99	0.9999
50nM+NA	-0.97	0.9998
5nM+NA	-0.89	0.9933
+NA	-0.78	0.9413

Positive values show pairs of means that are significantly different.

Oneway Analysis of cystathionine By Label

Means Comparisons

Comparisons with a control using Dunnett's Method

Control Group = -NA

|d|	Alpha
2.65691	0.05

Level	Abs(Dif)-LSD	p-Value	
50nM-NA	0.017	0.0193	*
5nM-NA	-0.09	0.9908	
-NA	-0.11	1.0000	
5nM+NA	-0.07	0.7010	
50nM+NA	-0.05	0.4060	
+NA	-9e-4	0.0525	

Positive values show pairs of means that are significantly different.

Oneway Analysis of Cysteineglutathione disulfide By Label

Means Comparisons

Comparisons with a control using Dunnett's Method

Control Group = -NA

|d|	Alpha
2.65691	0.05

Level	Abs(Dif)-LSD	p-Value	
5nM-NA	-1.54	0.9985	
-NA	-1.73	1.0000	
50nM-NA	-0.03	0.0554	
50nM+NA	0.869	0.0018	*
+NA	1.934	<.0001	*
5nM+NA	2.196	<.0001	*

Positive values show pairs of means that are significantly different.

Oneway Analysis of Cystine By Label

Means Comparisons

Comparisons with a control using Dunnett's Method

Control Group = -NA

|d|	Alpha
2.65691	0.05

Level	Abs(Dif)-LSD	p-Value	
5nM-NA	0.12	0.0316	*
5nM+NA	-0.34	0.1650	
+NA	-0.91	0.6807	
50nM+NA	-1.02	0.8054	
50nM-NA	-1.46	0.9996	
-NA	-1.59	1.0000	

Positive values show pairs of means that are significantly different.

Oneway Analysis of cytidine By Label

Means Comparisons

Comparisons with a control using Dunnett's Method

Control Group = -NA

|d|	Alpha
2.65691	0.05

Level	Abs(Dif)-LSD	p-Value	
50nM-NA	2.13	<.0001	*
5nM-NA	0.618	<.0001	*
50nM+NA	-0.23	0.9348	
5nM+NA	-0.25	0.9847	
+NA	-0.29	0.9998	
-NA	-0.31	1.0000	

Positive values show pairs of means that are significantly different.

Oneway Analysis of cytosine By Label

Means Comparisons

Comparisons with a control using Dunnett's Method

Control Group = -NA

|d|	Alpha
2.65691	0.05

Level	Abs(Dif)-LSD	p-Value	
50nM-NA	0.765	0.0002	*
5nM-NA	-0.19	0.1484	
+NA	-0.42	0.4389	
50nM+NA	-0.69	0.9189	
5nM+NA	-0.7	0.9240	
-NA	-0.96	1.0000	

Positive values show pairs of means that are significantly different.

Oneway Analysis of dAMP By Label

Means Comparisons

Comparisons with a control using Dunnett's Method

Control Group = -NA

|d|	Alpha
2.65691	0.05

Level	Abs(Dif)-LSD	p-Value
+NA	0.49	0.0002 *
50nM+NA	-0.12	0.1450
5nM+NA	-0.22	0.3168
-NA	-0.6	1.0000
50nM-NA	2.051	<.0001 *
5nM-NA	2.051	<.0001 *

Positive values show pairs of means that are significantly different.

Oneway Analysis of dCMP By Label

Means Comparisons

Comparisons with a control using Dunnett's Method

Control Group = -NA

|d|	Alpha
2.65691	0.05

Level	Abs(Dif)-LSD	p-Value
-NA	-0.84	1.0000
50nM+NA	-0.78	0.9997
5nM-NA	-0.71	0.9928
+NA	-0.61	0.9185
5nM+NA	-0.23	0.2188
50nM-NA	0.562	0.0005 *

Positive values show pairs of means that are significantly different.

Oneway Analysis of deoxyadenosine By Label

Means Comparisons

Comparisons with a control using Dunnett's Method

Control Group = -NA

|d|	Alpha
2.65691	0.05

Level	Abs(Dif)-LSD	p-Value	
-NA	-0.88	1.0000	
+NA	0.403	0.0024	*
5nM+NA	0.547	0.0007	*
5nM-NA	0.611	0.0004	*
50nM+NA	0.818	<.0001	*
50nM-NA	2.524	<.0001	*

Positive values show pairs of means that are significantly different.

Oneway Analysis of Deoxyheptulose-7-phosphate By Label

Means Comparisons

Comparisons with a control using Dunnett's Method

Control Group = -NA

|d|	Alpha
2.65691	0.05

Level	Abs(Dif)-LSD	p-Value	
+NA	0.477	0.0018	*
5nM+NA	0.215	0.0121	*
50nM+NA	-0.29	0.2492	
-NA	-0.96	1.0000	
5nM-NA	-0.74	0.9618	
50nM-NA	-0.24	0.1980	

Positive values show pairs of means that are significantly different.

Oneway Analysis of deoxyribose-phosphate By Label

Means Comparisons

Comparisons with a control using Dunnett's Method

Control Group = -NA

|d|	Alpha
2.65691	0.05

Level	Abs(Dif)-LSD	p-Value
50nM+NA	-0.55	0.4467
5nM-NA	-0.73	0.7146
-NA	-1.24	1.0000
5nM+NA	-1.12	0.9990
+NA	-1.03	0.9873
50nM-NA	-1	0.9793

Positive values show pairs of means that are significantly different.

Oneway Analysis of deoxyuridine By Label

Means Comparisons

Comparisons with a control using Dunnett's Method

Control Group = -NA

|d|	Alpha
2.65691	0.05

Level	Abs(Dif)-LSD	p-Value	
-NA	-1.18	1.0000	
5nM-NA	0.11	0.0283	*
50nM-NA	0.617	0.0015	*
50nM+NA	1.046	0.0001	*
5nM+NA	1.382	<.0001	*
+NA	1.712	<.0001	*

Positive values show pairs of means that are significantly different.

Oneway Analysis of dGDP By Label

Means Comparisons

Comparisons with a control using Dunnett's Method

Control Group = -NA

|d|	Alpha
2.65691	0.05

Level	Abs(Dif)-LSD	p-Value
50nM-NA	0.376	0.0011 *
5nM-NA	0.203	0.0070 *
5nM+NA	-0.45	0.8519
50nM+NA	-0.55	0.9883
-NA	-0.67	1.0000
+NA	-0.34	0.5735

Positive values show pairs of means that are significantly different.

Oneway Analysis of D-gluconate By Label

Means Comparisons

Comparisons with a control using Dunnett's Method

Control Group = -NA

|d|	Alpha
2.65691	0.05

Level	Abs(Dif)-LSD	p-Value
50nM-NA	1.61	<.0001 *
5nM-NA	1.593	<.0001 *
-NA	-0.22	1.0000
50nM+NA	0.188	0.0002 *
5nM+NA	0.208	<.0001 *
+NA	0.24	<.0001 *

Positive values show pairs of means that are significantly different.

Oneway Analysis of dihydroxy-acetone-phosphate By Label

Means Comparisons

Comparisons with a control using Dunnett's Method

Control Group = -NA

|d|	Alpha
2.65691	0.05

Level	Abs(Dif)-LSD	p-Value
50nM-NA	-0.55	0.5227
5nM-NA	-0.8	0.9032
-NA	-1.13	1.0000
5nM+NA	-0.91	0.9809
50nM+NA	-0.69	0.7464
+NA	-0.4	0.3102

Positive values show pairs of means that are significantly different.

Oneway Analysis of dimethylglycine By Label

Means Comparisons

Comparisons with a control using Dunnett's Method

Control Group = -NA

|d|	Alpha
2.65691	0.05

Level	Abs(Dif)-LSD	p-Value	
50nM-NA	0.615	0.0003	*
-NA	-0.8	1.0000	
5nM-NA	-0.52	0.8161	
5nM+NA	0.616	0.0003	*
+NA	0.637	0.0002	*
50nM+NA	0.664	0.0002	*

Positive values show pairs of means that are significantly different.

Oneway Analysis of DL-Pipecolic acid By Label

Means Comparisons

Comparisons with a control using Dunnett's Method

Control Group = -NA

|d|	Alpha
2.65691	0.05

Level	Abs(Dif)-LSD	p-Value	
5nM-NA	0.36	0.0086	*
50nM-NA	-0.09	0.0756	
50nM+NA	-0.15	0.0957	
5nM+NA	-0.36	0.2237	
+NA	-0.71	0.6325	
-NA	-1.3	1.0000	

Positive values show pairs of means that are significantly different.

Oneway Analysis of D-sedoheptulose-1-7-bisphosphate By Label

Means Comparisons

Comparisons with a control using Dunnett's Method

Control Group = -NA

|d|	Alpha
2.65691	0.05

Level	Abs(Dif)-LSD	p-Value	
50nM-NA	2.653	<.0001	*
5nM-NA	0.739	<.0001	*
-NA	-0.25	1.0000	
50nM+NA	-0.22	0.9945	
5nM+NA	-0.21	0.9823	
+NA	-0.2	0.9712	

Positive values show pairs of means that are significantly different.

Oneway Analysis of FAD By Label

Means Comparisons

Comparisons with a control using Dunnett's Method

Control Group = -NA

|d|	Alpha
2.65691	0.05

Level	Abs(Dif)-LSD	p-Value	
50nM-NA	0.07	0.0278	*
5nM-NA	-0.19	0.2092	
50nM+NA	-0.57	0.9610	
-NA	-0.73	1.0000	
5nM+NA	-0.62	0.9933	
+NA	-0.38	0.5898	

Positive values show pairs of means that are significantly different.

Oneway Analysis of folate By Label

Means Comparisons

Comparisons with a control using Dunnett's Method

Control Group = -NA

|d|	Alpha
2.65691	0.05

Level	Abs(Dif)-LSD	p-Value	
50nM-NA	0.287	0.0053	*
5nM-NA	0.127	0.0193	*
5nM+NA	-0.74	0.9988	
-NA	-0.83	1.0000	
50nM+NA	-0.76	0.9995	
+NA	-0.68	0.9848	

Positive values show pairs of means that are significantly different.

Oneway Analysis of fructose-1,6-bisphosphate By Label

Means Comparisons

Comparisons with a control using Dunnett's Method

Control Group = -NA

|d|	Alpha
2.65691	0.05

Level	Abs(Dif)-LSD	p-Value
50nM-NA	2.916	<.0001 *
5nM-NA	0.596	<.0001 *
-NA	-0.24	1.0000
50nM+NA	-0.2	0.9828
5nM+NA	-0.19	0.9555
+NA	-0.18	0.9278

Positive values show pairs of means that are significantly different.

Oneway Analysis of fructose-6-phosphate By Label

Means Comparisons

Comparisons with a control using Dunnett's Method

Control Group = -NA

|d|	Alpha
2.65691	0.05

Level	Abs(Dif)-LSD	p-Value	
50nM-NA	2.797	<.0001	*
5nM-NA	0.307	0.0001	*
-NA	-0.36	1.0000	
5nM+NA	-0.36	1.0000	
+NA	-0.35	1.0000	
50nM+NA	-0.35	1.0000	

Positive values show pairs of means that are significantly different.

Oneway Analysis of Geranyl-PP By Label

Means Comparisons

Comparisons with a control using Dunnett's Method

Control Group = -NA

|d|	Alpha
2.65691	0.05

Level	Abs(Dif)-LSD	p-Value	
50nM-NA	4.242	<.0001	*
5nM-NA	2.494	<.0001	*
-NA	-0.51	1.0000	
50nM+NA	-0.48	1.0000	
5nM+NA	-0.38	0.9415	
+NA	-0.36	0.9077	

Positive values show pairs of means that are significantly different.

Oneway Analysis of Glucaric acid By Label

Means Comparisons

Comparisons with a control using Dunnett's Method

Control Group = -NA

|d|	Alpha
2.65691	0.05

Level	Abs(Dif)-LSD	p-Value	
5nM+NA	0.327	0.0371	*
+NA	-1.61	0.1895	
50nM+NA	-3.06	0.4857	
-NA	-6.59	1.0000	
5nM-NA	-4.33	0.8289	
50nM-NA	-4.22	0.8034	

Positive values show pairs of means that are significantly different.

Oneway Analysis of Glucose By Label

Means Comparisons

Comparisons with a control using Dunnett's Method

Control Group = -NA

|d|	Alpha
2.65691	0.05

Level	Abs(Dif)-LSD	p-Value
5nM-NA	-0.55	0.4695
-NA	-1.21	1.0000
5nM+NA	-0.43	0.3212
+NA	-0.22	0.1374
50nM+NA	-0.17	0.1094
50nM-NA	-0.15	0.1012

Positive values show pairs of means that are significantly different.

Oneway Analysis of glucose-6-phosphate By Label

Means Comparisons

Comparisons with a control using Dunnett's Method

Control Group = -NA

|d|	Alpha
2.65691	0.05

Level	Abs(Dif)-LSD	p-Value	
-NA	-0.64	1.0000	
50nM-NA	-0.62	1.0000	
5nM-NA	-0.43	0.8621	
50nM+NA	0.943	<.0001	*
+NA	1.008	<.0001	*
5nM+NA	1.152	<.0001	*

Positive values show pairs of means that are significantly different.

Oneway Analysis of glutamate By Label

Means Comparisons

Comparisons with a control using Dunnett's Method

Control Group = -NA

|d|	Alpha
2.65691	0.05

Level	Abs(Dif)-LSD	p-Value	
-NA	-0.66	1.0000	
50nM+NA	0.328	0.0018	*
5nM-NA	0.47	0.0004	*
+NA	0.512	0.0002	*
5nM+NA	0.549	0.0002	*
50nM-NA	0.922	<.0001	*

Positive values show pairs of means that are significantly different.

Oneway Analysis of glutamine By Label

Means Comparisons

Comparisons with a control using Dunnett's Method

Control Group = -NA

|d|	Alpha
2.65691	0.05

Level	Abs(Dif)-LSD	p-Value
-NA	-1.41	1.0000
50nM-NA	0.972	0.0005 *
5nM-NA	1.432	<.0001 *
5nM+NA	1.708	<.0001 *
+NA	1.767	<.0001 *
50nM+NA	2.076	<.0001 *

Positive values show pairs of means that are significantly different.

Oneway Analysis of Glutathionedisufide By Label

Means Comparisons

Comparisons with a control using Dunnett's Method

Control Group = -NA

|d|	Alpha
2.65691	0.05

Level	Abs(Dif)-LSD	p-Value	
+NA	-1.64	1.0000	
-NA	-1.66	1.0000	
50nM+NA	-1.54	0.9998	
50nM-NA	1.501	<.0001	*
5nM+NA	2.439	<.0001	*
5nM-NA	2.534	<.0001	*

Positive values show pairs of means that are significantly different.

Oneway Analysis of Glutathione By Label

Means Comparisons

Comparisons with a control using Dunnett's Method

Control Group = -NA

|d|	Alpha
2.65691	0.05

Level	Abs(Dif)-LSD	p-Value	
50nM-NA	2.626	<.0001	*
50nM+NA	-1.21	0.8298	
-NA	-1.85	1.0000	
+NA	-1.3	0.8917	
5nM-NA	-0.83	0.4626	
5nM+NA	0.019	0.0471	*

Positive values show pairs of means that are significantly different.

Oneway Analysis of Glycerophosphocholine By Label

Means Comparisons

Comparisons with a control using Dunnett's Method

Control Group = -NA

|d|	Alpha
2.65691	0.05

Level	Abs(Dif)-LSD	p-Value	
50nM+NA	0.438	0.0023	*
5nM+NA	0.337	0.0049	*
+NA	-0.14	0.1130	
5nM-NA	-0.28	0.2408	
50nM-NA	-0.92	1.0000	
-NA	-0.94	1.0000	

Positive values show pairs of means that are significantly different.

Oneway Analysis of glycine By Label

Means Comparisons

Comparisons with a control using Dunnett's Method

Control Group = -NA

|d|	Alpha
2.65691	0.05

Level	Abs(Dif)-LSD	p-Value	
50nM-NA	0.518	0.0003	*
5nM-NA	0.367	0.0013	*
5nM+NA	0.243	0.0049	*
+NA	0.001	0.0497	*
50nM+NA	-0.06	0.0815	
-NA	-0.68	1.0000	

Positive values show pairs of means that are significantly different.

Oneway Analysis of GMP By Label

Means Comparisons

Comparisons with a control using Dunnett's Method

Control Group = -NA

|d|	Alpha
2.65691	0.05

Level	Abs(Dif)-LSD	p-Value	
50nM+NA	0.136	0.0194	*
-NA	-0.9	1.0000	
+NA	-0.87	1.0000	
5nM+NA	-0.41	0.4797	
5nM-NA	0.244	0.0088	*
50nM-NA	0.347	0.0040	*

Positive values show pairs of means that are significantly different.

Oneway Analysis of Guanidoacetic acid By Label

Means Comparisons

Comparisons with a control using Dunnett's Method

Control Group = -NA

|d|	Alpha
2.65691	0.05

Level	Abs(Dif)-LSD	p-Value
50nM+NA	-0.41	0.4206
5nM-NA	-0.42	0.4365
50nM-NA	-0.62	0.8089
5nM+NA	-0.71	0.9327
-NA	-0.95	1.0000
+NA	-0.59	0.7618

Positive values show pairs of means that are significantly different.

Oneway Analysis of Histidine By Label

Means Comparisons

Comparisons with a control using Dunnett's Method

Control Group = -NA

|d|	Alpha
2.65691	0.05

Level	Abs(Dif)-LSD	p-Value	
50nM-NA	1.929	<.0001	*
5nM-NA	1.096	0.0004	*
-NA	-1.52	1.0000	
50nM+NA	0.229	0.0197	*
5nM+NA	0.39	0.0098	*
+NA	0.653	0.0030	*

Positive values show pairs of means that are significantly different.

Oneway Analysis of homocysteine By Label

Means Comparisons

Comparisons with a control using Dunnett's Method

Control Group = -NA

|d|	Alpha
2.65691	0.05

Level	Abs(Dif)-LSD	p-Value
50nM+NA	-0.12	0.6769
5nM-NA	-0.21	1.0000
-NA	-0.21	1.0000
+NA	-0.2	1.0000
50nM-NA	-0.14	0.8455
5nM+NA	-0.1	0.4563

Positive values show pairs of means that are significantly different.

Oneway Analysis of homoserine By Label

Means Comparisons

Comparisons with a control using Dunnett's Method

Control Group = -NA

|d|	Alpha
2.65691	0.05

Level	Abs(Dif)-LSD	p-Value
5nM-NA	-1.5	0.9727
-NA	-1.89	1.0000
5nM+NA	-1.86	1.0000
+NA	-1.49	0.9710
50nM+NA	-1.24	0.8314
50nM-NA	-1.11	0.7145

Positive values show pairs of means that are significantly different.

Oneway Analysis of Homoserine lacton By Label

Means Comparisons

Comparisons with a control using Dunnett's Method

Control Group = -NA

|d|	Alpha
2.65691	0.05

Level	Abs(Dif)-LSD	p-Value	
5nM+NA	0.622	<.0001	*
+NA	0.383	0.0002	*
50nM+NA	-0.01	0.0599	
-NA	-0.48	1.0000	
5nM-NA	-0.45	0.9999	
50nM-NA	-0.32	0.8586	

Positive values show pairs of means that are significantly different.

Oneway Analysis of Hydroxyisocaproic acid By Label

Means Comparisons

Comparisons with a control using Dunnett's Method

Control Group = -NA

|d|	Alpha
2.65691	0.05

Level	Abs(Dif)-LSD	p-Value	
50nM-NA	0.034	0.0407	*
5nM-NA	-0.68	0.8705	
5nM+NA	-0.86	0.9962	
-NA	-0.99	1.0000	
50nM+NA	-0.96	1.0000	
+NA	-0.38	0.3606	

Positive values show pairs of means that are significantly different.

Oneway Analysis of Hydroxylamine By Label

Means Comparisons

Comparisons with a control using Dunnett's Method

Control Group = -NA

|d|	Alpha
2.65691	0.05

Level	Abs(Dif)-LSD	p-Value
+NA	-1.72	0.4962
50nM+NA	-2.77	0.9472
-NA	-3.66	1.0000
5nM+NA	-3.55	1.0000
5nM-NA	-2.72	0.9362
50nM-NA	0.016	0.0487 *

Positive values show pairs of means that are significantly different.

Oneway Analysis of hydroxyproline By Label

Means Comparisons

Comparisons with a control using Dunnett's Method

Control Group = -NA

|d|	Alpha
2.65691	0.05

Level	Abs(Dif)-LSD	p-Value	
5nM-NA	0.357	0.0029	*
50nM-NA	0.082	0.0272	*
+NA	-0.41	0.5371	
5nM+NA	-0.42	0.5786	
50nM+NA	-0.52	0.7974	
-NA	-0.82	1.0000	

Positive values show pairs of means that are significantly different.

Oneway Analysis of IDP By Label

Means Comparisons

Comparisons with a control using Dunnett's Method

Control Group = -NA

|d|	Alpha
2.65691	0.05

Level	Abs(Dif)-LSD	p-Value	
5nM-NA	0.024	0.0443	*
50nM-NA	-0.23	0.1467	
50nM+NA	-0.76	0.8106	
+NA	-1.11	1.0000	
-NA	-1.17	1.0000	
5nM+NA	-0.85	0.9160	

Positive values show pairs of means that are significantly different.

Oneway Analysis of Imidazoleacetic acid By Label

Means Comparisons

Comparisons with a control using Dunnett's Method

Control Group = -NA

|d|	Alpha
2.65691	0.05

Level	Abs(Dif)-LSD	p-Value	
5nM+NA	0.156	0.0150	*
50nM+NA	0.056	0.0329	*
+NA	-0.22	0.2110	
5nM-NA	-0.68	0.9901	
-NA	-0.81	1.0000	
50nM-NA	-0.52	0.8108	

Positive values show pairs of means that are significantly different.

Oneway Analysis of IMP By Label

Means Comparisons

Comparisons with a control using Dunnett's Method

Control Group = -NA

|d|	Alpha
2.65691	0.05

Level	Abs(Dif)-LSD	p-Value	
50nM-NA	1.897	<.0001	*
5nM-NA	1.147	<.0001	*
-NA	-0.47	1.0000	
50nM+NA	-0.44	1.0000	
+NA	-0.44	0.9999	
5nM+NA	-0.43	0.9998	

Positive values show pairs of means that are significantly different.

Oneway Analysis of indole By Label

Means Comparisons

Comparisons with a control using Dunnett's Method

Control Group = -NA

|d|	Alpha
2.65691	0.05

Level	Abs(Dif)-LSD	p-Value
50nM-NA	1.076	<.0001 *
5nM-NA	0.785	<.0001 *
+NA	-0.59	0.9977
5nM+NA	-0.6	0.9987
50nM+NA	-0.65	1.0000
-NA	-0.67	1.0000

Positive values show pairs of means that are significantly different.

Oneway Analysis of Indoleacrylic acid By Label

Means Comparisons

Comparisons with a control using Dunnett's Method

Control Group = -NA

|d|	Alpha
2.65691	0.05

Level	Abs(Dif)-LSD	p-Value
50nM-NA	-0.55	0.4768
5nM+NA	-0.93	0.9604
5nM-NA	-1.02	0.9931
50nM+NA	-1.18	1.0000
-NA	-1.21	1.0000
+NA	-1	0.9879

Positive values show pairs of means that are significantly different.

Oneway Analysis of inosine By Label

Means Comparisons

Comparisons with a control using Dunnett's Method

Control Group = -NA

|d|	Alpha
2.65691	0.05

Level	Abs(Dif)-LSD	p-Value	
50nM-NA	3.917	<.0001	*
5nM-NA	1.332	<.0001	*
-NA	-0.52	1.0000	
5nM+NA	-0.5	1.0000	
50nM+NA	-0.47	0.9994	
+NA	-0.47	0.9990	

Positive values show pairs of means that are significantly different.

Oneway Analysis of isocitrate By Label

Means Comparisons

Comparisons with a control using Dunnett's Method

Control Group = -NA

|d|	Alpha
2.65691	0.05

Level	Abs(Dif)-LSD	p-Value	
+NA	2.516	<.0001	*
50nM+NA	2.162	<.0001	*
5nM+NA	1.972	<.0001	*
50nM-NA	-0.65	1.0000	
5nM-NA	-0.65	1.0000	
-NA	-0.65	1.0000	

Positive values show pairs of means that are significantly different.

Oneway Analysis of Isoleucine By Label

Means Comparisons

Comparisons with a control using Dunnett's Method

Control Group = -NA

|d|	Alpha
2.65691	0.05

Level	Abs(Dif)-LSD	p-Value
5nM-NA	-0.01	0.3887
50nM-NA	-0.01	0.7095
-NA	-0.02	1.0000
5nM+NA	-0.01	0.9863
50nM+NA	-0.01	0.9431
+NA	-0.01	0.9189

Positive values show pairs of means that are significantly different.

Oneway Analysis of Ketohexanoic acid By Label

Means Comparisons

Comparisons with a control using Dunnett's Method

Control Group = -NA

|d|	Alpha
2.65691	0.05

Level	Abs(Dif)-LSD	p-Value
5nM-NA	-1.77	0.4313
50nM+NA	-4.01	1.0000
5nM+NA	-4.07	1.0000
-NA	-4.09	1.0000
+NA	-3.18	0.9642
50nM-NA	-0.49	0.0999

Positive values show pairs of means that are significantly different.

Oneway Analysis of Ketoleucine By Label

Means Comparisons

Comparisons with a control using Dunnett's Method

Control Group = -NA

|d|	Alpha
2.65691	0.05

Level	Abs(Dif)-LSD	p-Value	
5nM+NA	0.396	0.0012	*
+NA	0.345	0.0020	*
50nM+NA	0.025	0.0406	*
5nM-NA	-0.4	0.6796	
50nM-NA	-0.44	0.7735	
-NA	-0.71	1.0000	

Positive values show pairs of means that are significantly different.

Oneway Analysis of Kynurenic acid By Label

Means Comparisons

Comparisons with a control using Dunnett's Method

Control Group = -NA

|d|	Alpha
2.65691	0.05

Level	Abs(Dif)-LSD	p-Value
50nM+NA	-0.52	0.4611
-NA	-1.16	1.0000
5nM+NA	-1	0.9959
+NA	-0.93	0.9781
50nM-NA	-0.15	0.1062
5nM-NA	-0.11	0.0878

Positive values show pairs of means that are significantly different.

Oneway Analysis of Kynurenine By Label

Means Comparisons

Comparisons with a control using Dunnett's Method

Control Group = -NA

|d|	Alpha
2.65691	0.05

Level	Abs(Dif)-LSD	p-Value	
5nM+NA	0.083	0.0186	*
5nM-NA	-0.12	0.1782	
50nM-NA	-0.16	0.2443	
50nM+NA	-0.25	0.5081	
+NA	-0.3	0.6908	
-NA	-0.52	1.0000	

Positive values show pairs of means that are significantly different.

Oneway Analysis of Lactic acid By Label

Means Comparisons

Comparisons with a control using Dunnett's Method

Control Group = -NA

|d|	Alpha
2.65691	0.05

Level	Abs(Dif)-LSD	p-Value	
5nM-NA	-0.01	0.2142	
-NA	-0.03	1.0000	
50nM-NA	-0.01	0.4231	
50nM+NA	0.02	0.0003	*
5nM+NA	0.02	0.0003	*
+NA	0.029	<.0001	*

Positive values show pairs of means that are significantly different.

Oneway Analysis of leucine By Label

Means Comparisons

Comparisons with a control using Dunnett's Method

Control Group = -NA

|d|	Alpha
2.65691	0.05

Level	Abs(Dif)-LSD	p-Value	
50nM-NA	0.255	0.0091	*
5nM-NA	-0.36	0.3489	
-NA	-0.96	1.0000	
5nM+NA	-0.74	0.9622	
50nM+NA	-0.63	0.8373	
+NA	-0.56	0.7144	

Positive values show pairs of means that are significantly different.

Oneway Analysis of Lysine By Label

Means Comparisons

Comparisons with a control using Dunnett's Method

Control Group = -NA

|d|	Alpha
2.65691	0.05

Level	Abs(Dif)-LSD	p-Value
50nM-NA	0.367	0.0029 *
5nM+NA	0.031	0.0401 *
+NA	0.002	0.0495 *
50nM+NA	-0.45	0.6077
5nM-NA	-0.45	0.6080
-NA	-0.85	1.0000

Positive values show pairs of means that are significantly different.

Oneway Analysis of LysoPC(20:4) By Label

Means Comparisons

Comparisons with a control using Dunnett's Method

Control Group = -NA

|d|	Alpha
2.65691	0.05

Level	Abs(Dif)-LSD	p-Value
50nM-NA	-0.23	0.1328
+NA	-0.31	0.1835
5nM+NA	-0.43	0.2858
50nM+NA	-0.86	0.8431
-NA	-1.29	1.0000
5nM-NA	-1.24	1.0000

Positive values show pairs of means that are significantly different.

Oneway Analysis of Maleic acid By Label

Means Comparisons

Comparisons with a control using Dunnett's Method

Control Group = -NA

|d|	Alpha
2.65691	0.05

Level	Abs(Dif)-LSD	p-Value	
50nM-NA	0.856	<.0001	*
5nM-NA	0.039	0.0299	*
-NA	-0.46	1.0000	
5nM+NA	-0.23	0.5689	
50nM+NA	-0.2	0.4549	
+NA	-0.19	0.3997	

Positive values show pairs of means that are significantly different.

Oneway Analysis of Mannitol By Label

Means Comparisons

Comparisons with a control using Dunnett's Method

Control Group = -NA

|d|	Alpha
2.65691	0.05

Level	Abs(Dif)-LSD	p-Value	
5nM-NA	-0.01	0.9586	
-NA	-0.01	1.0000	
50nM-NA	0.001	0.0380	*
50nM+NA	0.011	0.0002	*
5nM+NA	0.011	0.0002	*
+NA	0.015	<.0001	*

Positive values show pairs of means that are significantly different.

Oneway Analysis of methionine By Label

Means Comparisons

Comparisons with a control using Dunnett's Method

Control Group = -NA

|d|	Alpha
2.65691	0.05

Level	Abs(Dif)-LSD	p-Value	
50nM-NA	1.168	<.0001	*
5nM-NA	0.898	<.0001	*
5nM+NA	0.146	0.0098	*
50nM+NA	0.009	0.0455	*
+NA	-0.19	0.2811	
-NA	-0.57	1.0000	

Positive values show pairs of means that are significantly different.

Oneway Analysis of Methionine sulfoxide By Label

Means Comparisons

Comparisons with a control using Dunnett's Method

Control Group = -NA

|d|	Alpha
2.65691	0.05

Level	Abs(Dif)-LSD	p-Value	
5nM+NA	0.419	0.0009	*
50nM-NA	0.315	0.0026	*
5nM-NA	0.201	0.0080	*
50nM+NA	-0.06	0.0831	
+NA	-0.25	0.3239	
-NA	-0.7	1.0000	

Positive values show pairs of means that are significantly different.

Oneway Analysis of Methylcysteine By Label

Means Comparisons

Comparisons with a control using Dunnett's Method

Control Group = -NA

|d|	Alpha
2.65691	0.05

Level	Abs(Dif)-LSD	p-Value	
50nM-NA	0.778	<.0001	*
5nM-NA	0.491	<.0001	*
-NA	-0.48	1.0000	
50nM+NA	-0.42	0.9972	
5nM+NA	-0.42	0.9967	
+NA	-0.31	0.8264	

Positive values show pairs of means that are significantly different.

Oneway Analysis of Methylmalonic acid By Label

Means Comparisons

Comparisons with a control using Dunnett's Method

Control Group = -NA

|d|	Alpha
2.65691	0.05

Level	Abs(Dif)-LSD	p-Value	
5nM+NA	0.289	0.0025	*
50nM+NA	0.241	0.0043	*
+NA	0.063	0.0272	*
-NA	-0.64	1.0000	
5nM-NA	-0.22	0.3086	
50nM-NA	-0.18	0.2348	

Positive values show pairs of means that are significantly different.

Oneway Analysis of Myo-Inositol By Label

Means Comparisons

Comparisons with a control using Dunnett's Method

Control Group = -NA

|d|	Alpha
2.65691	0.05

Level	Abs(Dif)-LSD	p-Value
-NA	-0.42	1.0000
5nM-NA	-0.36	0.9923
5nM+NA	-0.27	0.7990
50nM+NA	-0.23	0.6282
50nM-NA	-0.21	0.5393
+NA	-0.13	0.2639

Positive values show pairs of means that are significantly different.

Oneway Analysis of N6-Acetyl-L-lysine By Label

Means Comparisons

Comparisons with a control using Dunnett's Method

Control Group = -NA

|d|	Alpha
2.65691	0.05

Level	Abs(Dif)-LSD	p-Value	
50nM-NA	0.171	0.0064	*
5nM-NA	0.157	0.0076	*
-NA	-0.53	1.0000	
50nM+NA	-0.27	0.5656	
5nM+NA	-0.09	0.1311	
+NA	0.031	0.0354	*

Positive values show pairs of means that are significantly different.

Oneway Analysis of N-Acetylaspartylglutamic acid By Label

Means Comparisons

Comparisons with a control using Dunnett's Method

Control Group = -NA

|d|	Alpha
2.65691	0.05

Level	Abs(Dif)-LSD	p-Value	
5nM+NA	0.01	0.0487	*
50nM+NA	-1.24	0.6454	
+NA	-1.39	0.7654	
-NA	-2.25	1.0000	
5nM-NA	-0.58	0.2040	
50nM-NA	0.327	0.0204	*

Positive values show pairs of means that are significantly different.

Oneway Analysis of N-acetyl-glucosamine-1-phosphate By Label

Means Comparisons

Comparisons with a control using Dunnett's Method

Control Group = -NA

|d|	Alpha
2.65691	0.05

Level	Abs(Dif)-LSD	p-Value
50nM+NA	-1.16	0.9130
5nM-NA	-1.28	0.9752
-NA	-1.61	1.0000
5nM+NA	-1.34	0.9884
+NA	-1.28	0.9749
50nM-NA	-0.8	0.5417

Positive values show pairs of means that are significantly different.

Oneway Analysis of N-acetyl-glutamine By Label

Means Comparisons

Comparisons with a control using Dunnett's Method

Control Group = -NA

|d|	Alpha
2.65691	0.05

Level	Abs(Dif)-LSD	p-Value	
5nM-NA	0.236	0.0027	*
50nM-NA	0.104	0.0147	*
-NA	-0.53	1.0000	
50nM+NA	-0.12	0.1719	
5nM+NA	-0.06	0.0977	
+NA	0.103	0.0150	*

Positive values show pairs of means that are significantly different.

Oneway Analysis of N-Acetyl-L-alanine By Label

Means Comparisons

Comparisons with a control using Dunnett's Method

Control Group = -NA

|d|	Alpha
2.65691	0.05

Level	Abs(Dif)-LSD	p-Value	
50nM+NA	0.244	0.0074	*
5nM+NA	-0.38	0.4875	
+NA	-0.51	0.7691	
-NA	-0.82	1.0000	
5nM-NA	-0.76	0.9998	
50nM-NA	-0.75	0.9996	

Positive values show pairs of means that are significantly different.

Oneway Analysis of N-acetyl-L-ornithine By Label

Means Comparisons

Comparisons with a control using Dunnett's Method

Control Group = -NA

|d|	Alpha
2.65691	0.05

Level	Abs(Dif)-LSD	p-Value	
50nM-NA	0.077	0.0292	*
5nM-NA	-0.71	0.9840	
-NA	-0.88	1.0000	
+NA	-0.48	0.6455	
50nM+NA	-0.41	0.4955	
5nM+NA	-0.4	0.4648	

Positive values show pairs of means that are significantly different.

Oneway Analysis of N-Acetylputrescine By Label

Means Comparisons

Comparisons with a control using Dunnett's Method

Control Group = -NA

|d|	Alpha
2.65691	0.05

Level	Abs(Dif)-LSD	p-Value	
5nM-NA	0.24	0.0100	*
-NA	-0.95	1.0000	
50nM-NA	-0.34	0.3171	
5nM+NA	-0.29	0.2578	
50nM+NA	-0.17	0.1364	
+NA	-0.02	0.0572	

Positive values show pairs of means that are significantly different.

Oneway Analysis of NAD By Label

Means Comparisons

Comparisons with a control using Dunnett's Method

Control Group = -NA

|d|	Alpha
2.65691	0.05

Level	Abs(Dif)-LSD	p-Value
50nM+NA	0.662	<.0001 *
+NA	0.62	<.0001 *
5nM+NA	0.323	0.0003 *
-NA	-0.44	1.0000
5nM-NA	2.146	<.0001 *
50nM-NA	2.176	<.0001 *

Positive values show pairs of means that are significantly different.

Oneway Analysis of NADH By Label

Means Comparisons

Comparisons with a control using Dunnett's Method

Control Group = -NA

|d|	Alpha
2.65691	0.05

Level	Abs(Dif)-LSD	p-Value
+NA	0.305	0.0019 *
5nM+NA	0.12	0.0148 *
50nM+NA	0.03	0.0373 *
-NA	-0.62	1.0000
5nM-NA	1.972	<.0001 *
50nM-NA	2.077	<.0001 *

Positive values show pairs of means that are significantly different.

Oneway Analysis of NADP+ By Label

Means Comparisons

Comparisons with a control using Dunnett's Method

Control Group = -NA

|d|	Alpha
2.65691	0.05

Level	Abs(Dif)-LSD	p-Value	
50nM+NA	0.136	0.0236	*
+NA	-0.89	0.9735	
5nM+NA	-0.92	0.9864	
-NA	-1.12	1.0000	
5nM-NA	0.694	0.0008	*
50nM-NA	1.28	<.0001	*

Positive values show pairs of means that are significantly different.

Oneway Analysis of N-carbamoyl-L-aspartate By Label

Means Comparisons

Comparisons with a control using Dunnett's Method

Control Group = -NA

|d|	Alpha
2.65691	0.05

Level	Abs(Dif)-LSD	p-Value	
5nM+NA	1.518	<.0001	*
+NA	1.437	<.0001	*
50nM+NA	1.332	<.0001	*
50nM-NA	-0.54	1.0000	
5nM-NA	-0.55	1.0000	
-NA	-0.56	1.0000	

Positive values show pairs of means that are significantly different.

Oneway Analysis of N-Formylglycinamide ribonucleotide By Label

Means Comparisons

Comparisons with a control using Dunnett's Method

Control Group = -NA

|d|	Alpha
2.65691	0.05

Level	Abs(Dif)-LSD	p-Value
50nM-NA	2.557	<.0001 *
5nM-NA	1.408	<.0001 *
-NA	-0.31	1.0000
+NA	-0.3	1.0000
5nM+NA	-0.3	1.0000
50nM+NA	-0.29	0.9997

Positive values show pairs of means that are significantly different.

Oneway Analysis of Ng,NG-dimethyl-L-arginine By Label

Means Comparisons

Comparisons with a control using Dunnett's Method

Control Group = -NA

|d|	Alpha
2.65691	0.05

Level	Abs(Dif)-LSD	p-Value	
50nM-NA	0.092	0.0212	*
5nM-NA	-0.19	0.2296	
5nM+NA	-0.59	0.9982	
-NA	-0.66	1.0000	
50nM+NA	-0.61	0.9995	
+NA	-0.39	0.7121	

Positive values show pairs of means that are significantly different.

Oneway Analysis of Nicotinamide By Label

Means Comparisons

Comparisons with a control using Dunnett's Method

Control Group = -NA

|d|	Alpha
2.65691	0.05

Level	Abs(Dif)-LSD	p-Value
50nM-NA	-0.88	1.0000
-NA	-0.9	1.0000
5nM-NA	-0.88	1.0000
+NA	0.194	0.0129 *
50nM+NA	0.289	0.0064 *
5nM+NA	0.431	0.0021 *

Positive values show pairs of means that are significantly different.

Oneway Analysis of Nicotinamide ribotide By Label

Means Comparisons

Comparisons with a control using Dunnett's Method

Control Group = -NA

|d|	Alpha
2.65691	0.05

Level	Abs(Dif)-LSD	p-Value	
+NA	0.172	0.0086	*
50nM+NA	0.08	0.0228	*
5nM+NA	-0.16	0.2078	
-NA	-0.63	1.0000	
5nM-NA	2.149	<.0001	*
50nM-NA	2.174	<.0001	*

Positive values show pairs of means that are significantly different.

Oneway Analysis of Nicotinic acid By Label

Means Comparisons

Comparisons with a control using Dunnett's Method

Control Group = -NA

|d|	Alpha
2.65691	0.05

Level	Abs(Dif)-LSD	p-Value	
5nM+NA	1.403	<.0001	*
+NA	1.332	<.0001	*
50nM+NA	1.204	<.0001	*
50nM-NA	-0.59	1.0000	
5nM-NA	-0.59	1.0000	
-NA	-0.59	1.0000	

Positive values show pairs of means that are significantly different.

Oneway Analysis of N-Methylantranilic acid By Label

Means Comparisons

Comparisons with a control using Dunnett's Method

Control Group = -NA

|d|	Alpha
2.65691	0.05

Level	Abs(Dif)-LSD	p-Value	
50nM-NA	-2.07	0.9983	
-NA	-2.33	1.0000	
5nM-NA	-2.06	0.9981	
+NA	1.902	0.0002	*
5nM+NA	1.982	0.0001	*
50nM+NA	3.629	<.0001	*

Positive values show pairs of means that are significantly different.

Oneway Analysis of O-acetyl-L-serine By Label

Means Comparisons

Comparisons with a control using Dunnett's Method

Control Group = -NA

|d|	Alpha
2.65691	0.05

Level	Abs(Dif)-LSD	p-Value	
5nM+NA	0.328	0.0038	*
50nM+NA	0.14	0.0176	*
+NA	0.046	0.0359	*
5nM-NA	-0.06	0.0742	
50nM-NA	-0.76	0.9994	
-NA	-0.83	1.0000	

Positive values show pairs of means that are significantly different.

Oneway Analysis of octulose-monophosphate By Label

Means Comparisons

Comparisons with a control using Dunnett's Method

Control Group = -NA

|d|	Alpha
2.65691	0.05

Level	Abs(Dif)-LSD	p-Value	
50nM-NA	0.561	<.0001	*
5nM-NA	0.225	0.0035	*
-NA	-0.55	1.0000	
5nM+NA	-0.03	0.0663	
50nM+NA	0.017	0.0414	*
+NA	0.4	0.0004	*

Positive values show pairs of means that are significantly different.

Oneway Analysis of ornithine By Label

Means Comparisons

Comparisons with a control using Dunnett's Method

Control Group = -NA

|d|	Alpha
2.65691	0.05

Level	Abs(Dif)-LSD	p-Value	
50nM-NA	0.573	<.0001	*
5nM-NA	0.432	0.0004	*
5nM+NA	-0.56	0.9997	
-NA	-0.6	1.0000	
50nM+NA	-0.55	0.9996	
+NA	-0.51	0.9915	

Positive values show pairs of means that are significantly different.

Oneway Analysis of orotate By Label

Means Comparisons

Comparisons with a control using Dunnett's Method

Control Group = -NA

|d|	Alpha
2.65691	0.05

Level	Abs(Dif)-LSD	p-Value	
5nM+NA	1.781	<.0001	*
50nM+NA	1.47	<.0001	*
+NA	1.23	<.0001	*
-NA	-0.82	1.0000	
5nM-NA	-0.8	1.0000	
50nM-NA	-0.73	0.9987	

Positive values show pairs of means that are significantly different.

Oneway Analysis of Orotidine By Label

Means Comparisons

Comparisons with a control using Dunnett's Method

Control Group = -NA

|d|	Alpha
2.65691	0.05

Level	Abs(Dif)-LSD	p-Value	
50nM-NA	1.583	<.0001	*
5nM-NA	0.438	<.0001	*
-NA	-0.2	1.0000	
+NA	-0.13	0.8440	
5nM+NA	-0.08	0.3996	
50nM+NA	-0.06	0.2535	

Positive values show pairs of means that are significantly different.

Oneway Analysis of Palmitic acid By Label

Means Comparisons

Comparisons with a control using Dunnett's Method

Control Group = -NA

|d|	Alpha
2.65691	0.05

Level	Abs(Dif)-LSD	p-Value
5nM-NA	-0.11	0.0588
50nM-NA	-1.27	0.2810
-NA	-3.86	1.0000
5nM+NA	-3.51	0.9993
50nM+NA	-3.14	0.9824
+NA	-3.08	0.9761

Positive values show pairs of means that are significantly different.

Oneway Analysis of pantothenate By Label

Means Comparisons

Comparisons with a control using Dunnett's Method

Control Group = -NA

|d|	Alpha
2.65691	0.05

Level	Abs(Dif)-LSD	p-Value	
50nM-NA	0.187	0.0110	*
5nM-NA	-0.13	0.1254	
5nM+NA	-0.74	0.9999	
-NA	-0.79	1.0000	
50nM+NA	-0.78	1.0000	
+NA	-0.61	0.9647	

Positive values show pairs of means that are significantly different.

Oneway Analysis of PC(15:0) By Label

Means Comparisons

Comparisons with a control using Dunnett's Method

Control Group = -NA

|d|	Alpha
2.65691	0.05

Level	Abs(Dif)-LSD	p-Value	
50nM+NA	-1.6	0.3104	
-NA	-4.57	1.0000	
5nM+NA	-3.42	0.9414	
+NA	-2.76	0.7420	
5nM-NA	0.597	0.0223	*
50nM-NA	2.013	0.0027	*

Positive values show pairs of means that are significantly different.

Oneway Analysis of p-Coumaric acid By Label

Means Comparisons

Comparisons with a control using Dunnett's Method

Control Group = -NA

|d|	Alpha
2.65691	0.05

Level	Abs(Dif)-LSD	p-Value	
50nM-NA	1.715	<.0001	*
5nM-NA	0.958	0.0002	*
5nM+NA	-0.69	0.7335	
-NA	-1.15	1.0000	
50nM+NA	-0.83	0.9088	
+NA	-0.11	0.0855	

Positive values show pairs of means that are significantly different.

Oneway Analysis of Pentadecanal By Label

Means Comparisons

Comparisons with a control using Dunnett's Method

Control Group = -NA

|d|	Alpha
2.65691	0.05

Level	Abs(Dif)-LSD	p-Value
5nM+NA	0.208	<.0001 *
5nM-NA	0.145	<.0001 *
-NA	-0.08	1.0000
50nM-NA	-0.01	0.0825
50nM+NA	-3e-3	0.0617
+NA	-8e-4	0.0530

Positive values show pairs of means that are significantly different.

Oneway Analysis of Phenacylamine By Label

Means Comparisons

Comparisons with a control using Dunnett's Method

Control Group = -NA

|d|	Alpha
2.65691	0.05

Level	Abs(Dif)-LSD	p-Value	
50nM-NA	1.556	<.0001	*
5nM-NA	0.876	<.0001	*
5nM+NA	-0.6	0.7719	
-NA	-0.96	1.0000	
50nM+NA	-0.69	0.9084	
+NA	-0.08	0.0791	

Positive values show pairs of means that are significantly different.

Oneway Analysis of phenylalanine By Label

Means Comparisons

Comparisons with a control using Dunnett's Method

Control Group = -NA

|d|	Alpha
2.65691	0.05

Level	Abs(Dif)-LSD	p-Value	
50nM-NA	0.736	<.0001	*
5nM-NA	0.625	<.0001	*
5nM+NA	-0.13	0.1790	
50nM+NA	-0.34	0.7884	
+NA	-0.36	0.8518	
-NA	-0.54	1.0000	

Positive values show pairs of means that are significantly different.

Oneway Analysis of Phenyllactic acid By Label

Means Comparisons

Comparisons with a control using Dunnett's Method

Control Group = -NA

|d|	Alpha
2.65691	0.05

Level	Abs(Dif)-LSD	p-Value	
-NA	-1.86	1.0000	
50nM+NA	1.281	0.0005	*
5nM+NA	1.335	0.0004	*
5nM-NA	1.585	0.0001	*
+NA	1.643	0.0001	*
50nM-NA	1.695	<.0001	*

Positive values show pairs of means that are significantly different.

Oneway Analysis of Phosphate By Label

Means Comparisons

Comparisons with a control using Dunnett's Method

Control Group = -NA

|d|	Alpha
2.65691	0.05

Level	Abs(Dif)-LSD	p-Value
+NA	-4.61	0.3052
50nM-NA	-13.2	1.0000
5nM-NA	-13.3	1.0000
5nM+NA	-13.3	1.0000
50nM+NA	-13.3	1.0000
-NA	-13.3	1.0000

Positive values show pairs of means that are significantly different.

Oneway Analysis of Phosphatidylglycerol By Label

Means Comparisons

Comparisons with a control using Dunnett's Method

Control Group = -NA

|d|	Alpha
2.65691	0.05

Level	Abs(Dif)-LSD	p-Value	
50nM-NA	1.468	<.0001	*
5nM-NA	-0.78	0.5427	
5nM+NA	-1.05	0.8463	
-NA	-1.57	1.0000	
50nM+NA	-1.27	0.9803	
+NA	-0.97	0.7615	

Positive values show pairs of means that are significantly different.

Oneway Analysis of phosphoenolpyruvate By Label

Means Comparisons

Comparisons with a control using Dunnett's Method

Control Group = -NA

|d|	Alpha
2.65691	0.05

Level	Abs(Dif)-LSD	p-Value	
50nM-NA	0.391	0.0022	*
5nM-NA	-0.05	0.0726	
50nM+NA	-0.07	0.0794	
5nM+NA	-0.21	0.1952	
+NA	-0.22	0.2140	
-NA	-0.83	1.0000	

Positive values show pairs of means that are significantly different.

Oneway Analysis of Phosphorylcholine By Label

Means Comparisons

Comparisons with a control using Dunnett's Method

Control Group = -NA

|d|	Alpha
2.65691	0.05

Level	Abs(Dif)-LSD	p-Value	
5nM-NA	0.445	0.0005	*
50nM-NA	0.389	0.0010	*
5nM+NA	-0.15	0.1767	
50nM+NA	-0.2	0.2405	
+NA	-0.36	0.6280	
-NA	-0.67	1.0000	

Positive values show pairs of means that are significantly different.

Oneway Analysis of p-hydroxybenzoate By Label

Means Comparisons

Comparisons with a control using Dunnett's Method

Control Group = -NA

|d|	Alpha
2.65691	0.05

Level	Abs(Dif)-LSD	p-Value	
50nM-NA	0.503	0.0001	*
5nM-NA	0.131	0.0117	*
-NA	-0.57	1.0000	
50nM+NA	-0.05	0.0850	
5nM+NA	0.204	0.0049	*
+NA	0.265	0.0023	*

Positive values show pairs of means that are significantly different.

Oneway Analysis of PI(16:0/20:2) By Label

Means Comparisons

Comparisons with a control using Dunnett's Method

Control Group = -NA

|d|	Alpha
2.65691	0.05

Level	Abs(Dif)-LSD	p-Value
50nM+NA	-0.21	0.0833
50nM-NA	-0.93	0.3567
5nM+NA	-0.96	0.3797
5nM-NA	-1.33	0.6432
-NA	-2.41	1.0000
+NA	-2.35	1.0000

Positive values show pairs of means that are significantly different.

Oneway Analysis of PI(16:0/22:4) By Label

Means Comparisons

Comparisons with a control using Dunnett's Method

Control Group = -NA

|d|	Alpha
2.65691	0.05

Level	Abs(Dif)-LSD	p-Value
50nM+NA	0.621	0.0135 *
5nM+NA	0.014	0.0486 *
50nM-NA	-1.26	0.4140
5nM-NA	-1.94	0.8178
+NA	-2.23	0.9406
-NA	-2.99	1.0000

Positive values show pairs of means that are significantly different.

Oneway Analysis of PI(16:1/22:4) By Label

Means Comparisons

Comparisons with a control using Dunnett's Method

Control Group = -NA

|d|	Alpha
2.65691	0.05

Level	Abs(Dif)-LSD	p-Value
50nM+NA	-0.3	0.1030
50nM-NA	-0.36	0.1183
5nM+NA	-0.78	0.2857
5nM-NA	-1.28	0.6374
-NA	-2.34	1.0000
+NA	-2.23	1.0000

Positive values show pairs of means that are significantly different.

Oneway Analysis of Piperidine By Label

Means Comparisons

Comparisons with a control using Dunnett's Method

Control Group = -NA

|d|	Alpha
2.65691	0.05

Level	Abs(Dif)-LSD	p-Value	
50nM-NA	4.028	<.0001	*
5nM-NA	2.957	<.0001	*
5nM+NA	0.015	0.0459	*
50nM+NA	-0.58	0.6355	
-NA	-1.07	1.0000	
+NA	0.477	0.0026	*

Positive values show pairs of means that are significantly different.

Oneway Analysis of proline By Label

Means Comparisons

Comparisons with a control using Dunnett's Method

Control Group = -NA

|d|	Alpha
2.65691	0.05

Level	Abs(Dif)-LSD	p-Value	
50nM-NA	0.465	<.0001	*
-NA	-0.4	1.0000	
5nM-NA	-0.07	0.1288	
+NA	0.056	0.0212	*
5nM+NA	0.085	0.0134	*
50nM+NA	0.147	0.0047	*

Positive values show pairs of means that are significantly different.

Oneway Analysis of purine By Label

Means Comparisons

Comparisons with a control using Dunnett's Method

Control Group = -NA

|d|	Alpha
2.65691	0.05

Level	Abs(Dif)-LSD	p-Value	
50nM-NA	0.728	<.0001	*
5nM-NA	0.677	<.0001	*
5nM+NA	-0.47	0.9993	
50nM+NA	-0.51	1.0000	
-NA	-0.52	1.0000	
+NA	-0.43	0.9896	

Positive values show pairs of means that are significantly different.

Oneway Analysis of pyridoxine By Label

Means Comparisons

Comparisons with a control using Dunnett's Method

Control Group = -NA

|d|	Alpha
2.65691	0.05

Level	Abs(Dif)-LSD	p-Value
5nM-NA	-0.27	0.3174
50nM-NA	-0.37	0.5254
-NA	-0.76	1.0000
5nM+NA	-0.48	0.7881
50nM+NA	-0.3	0.3712
+NA	-0.29	0.3597

Positive values show pairs of means that are significantly different.

Oneway Analysis of Pyroglutamate By Label

Means Comparisons

Comparisons with a control using Dunnett's Method

Control Group = -NA

|d|	Alpha
2.65691	0.05

Level	Abs(Dif)-LSD	p-Value
-NA	-0.56	1.0000
5nM+NA	0.716	<.0001 *
50nM+NA	0.749	<.0001 *
+NA	0.761	<.0001 *
5nM-NA	0.818	<.0001 *
50nM-NA	0.992	<.0001 *

Positive values show pairs of means that are significantly different.

Oneway Analysis of pyruvate By Label

Means Comparisons

Comparisons with a control using Dunnett's Method

Control Group = -NA

|d|	Alpha
2.65691	0.05

Level	Abs(Dif)-LSD	p-Value
-NA	-0.84	1.0000
50nM+NA	-0.64	0.9576
5nM+NA	-0.48	0.6936
50nM-NA	-0.47	0.6628
5nM-NA	-0.37	0.4442
+NA	-0.27	0.2740

Positive values show pairs of means that are significantly different.

Oneway Analysis of riboflavin By Label

Means Comparisons

Comparisons with a control using Dunnett's Method

Control Group = -NA

|d|	Alpha
2.65691	0.05

Level	Abs(Dif)-LSD	p-Value	
50nM-NA	-0.19	0.1903	
5nM-NA	-0.48	0.7465	
-NA	-0.79	1.0000	
50nM+NA	-0.16	0.1580	
5nM+NA	-0.03	0.0639	
+NA	0.078	0.0274	*

Positive values show pairs of means that are significantly different.

Oneway Analysis of ribose-phosphate By Label

Means Comparisons

Comparisons with a control using Dunnett's Method

Control Group = -NA

|d|	Alpha
2.65691	0.05

Level	Abs(Dif)-LSD	p-Value	
50nM-NA	1.886	<.0001	*
5nM-NA	0.813	<.0001	*
50nM+NA	-0.32	0.6675	
+NA	-0.45	0.9764	
5nM+NA	-0.5	0.9974	
-NA	-0.57	1.0000	

Positive values show pairs of means that are significantly different.

Oneway Analysis of S-adenosyl-L-homoCysteine By Label

Means Comparisons

Comparisons with a control using Dunnett's Method

Control Group = -NA

|d|	Alpha
2.65691	0.05

Level	Abs(Dif)-LSD	p-Value	
50nM-NA	1.421	<.0001	*
5nM-NA	1.12	<.0001	*
-NA	-0.38	1.0000	
50nM+NA	-0.38	1.0000	
5nM+NA	-0.32	0.9902	
+NA	-0.19	0.5479	

Positive values show pairs of means that are significantly different.

Oneway Analysis of S-adenosyl-L-methioninamine By Label

Means Comparisons

Comparisons with a control using Dunnett's Method

Control Group = -NA

|d|	Alpha
2.65691	0.05

Level	Abs(Dif)-LSD	p-Value
5nM-NA	-0.78	0.9925
5nM+NA	-0.81	0.9967
50nM-NA	-0.9	1.0000
-NA	-0.93	1.0000
50nM+NA	-0.47	0.5635
+NA	-0.03	0.0591

Positive values show pairs of means that are significantly different.

Oneway Analysis of SAM By Label

Means Comparisons

Comparisons with a control using Dunnett's Method

Control Group = -NA

|d|	Alpha
2.65691	0.05

Level	Abs(Dif)-LSD	p-Value
50nM-NA	2.686	<.0001 *
5nM-NA	-0.02	0.0753
-NA	-0.33	1.0000
50nM+NA	0.523	<.0001 *
5nM+NA	0.621	<.0001 *
+NA	0.695	<.0001 *

Positive values show pairs of means that are significantly different.

Oneway Analysis of sarcosine By Label

Means Comparisons

Comparisons with a control using Dunnett's Method

Control Group = -NA

|d|	Alpha
2.65691	0.05

Level	Abs(Dif)-LSD	p-Value	
50nM-NA	2.606	<.0001	*
5nM-NA	1.166	<.0001	*
-NA	-0.32	1.0000	
50nM+NA	-0.06	0.1435	
5nM+NA	-0.04	0.0988	
+NA	0.038	0.0239	*

Positive values show pairs of means that are significantly different.

Oneway Analysis of Sedoheptulose 7-phosphate By Label

Means Comparisons

Comparisons with a control using Dunnett's Method

Control Group = -NA

|d|	Alpha
2.65691	0.05

Level	Abs(Dif)-LSD	p-Value	
50nM-NA	1.862	<.0001	*
5nM-NA	0.53	<.0001	*
50nM+NA	-0.19	1.0000	
-NA	-0.2	1.0000	
+NA	-0.2	1.0000	
5nM+NA	-0.18	0.9999	

Positive values show pairs of means that are significantly different.

Oneway Analysis of serine By Label

Means Comparisons

Comparisons with a control using Dunnett's Method

Control Group = -NA

|d|	Alpha
2.65691	0.05

Level	Abs(Dif)-LSD	p-Value
5nM+NA	-0.15	0.1300
50nM-NA	-0.64	0.8999
-NA	-0.9	1.0000
+NA	-0.69	0.9594
50nM+NA	-0.53	0.7070
5nM-NA	0.18	0.0142 *

Positive values show pairs of means that are significantly different.

Oneway Analysis of S-methyl-5-thioadenosine By Label

Means Comparisons

Comparisons with a control using Dunnett's Method

Control Group = -NA

|d|	Alpha
2.65691	0.05

Level	Abs(Dif)-LSD	p-Value
5nM-NA	-2.1	0.9997
-NA	-2.28	1.0000
5nM+NA	-1.86	0.9843
50nM+NA	-0.51	0.1715
+NA	-0.39	0.1295
50nM-NA	-0.26	0.0954

Positive values show pairs of means that are significantly different.

Oneway Analysis of sn-glycerol-3-phosphate By Label

Means Comparisons

Comparisons with a control using Dunnett's Method

Control Group = -NA

|d|	Alpha
2.65691	0.05

Level	Abs(Dif)-LSD	p-Value
5nM-NA	-1.54	0.9994
-NA	-1.69	1.0000
50nM+NA	-1.67	1.0000
5nM+NA	-1.29	0.9554
50nM-NA	-1.17	0.8813
+NA	-0.98	0.6943

Positive values show pairs of means that are significantly different.

Oneway Analysis of Spermidine By Label

Means Comparisons

Comparisons with a control using Dunnett's Method

Control Group = -NA

|d|	Alpha
2.65691	0.05

Level	Abs(Dif)-LSD	p-Value	
50nM-NA	1.423	<.0001	*
5nM-NA	-0.03	0.0960	
-NA	-0.3	1.0000	
5nM+NA	-0.23	0.9501	
50nM+NA	-0.21	0.8665	
+NA	-0.2	0.8458	

Positive values show pairs of means that are significantly different.

Oneway Analysis of Spermine By Label

Means Comparisons

Comparisons with a control using Dunnett's Method

Control Group = -NA

|d|	Alpha
2.65691	0.05

Level	Abs(Dif)-LSD	p-Value	
50nM-NA	1.546	0.0007	*
5nM+NA	-2.22	0.9994	
-NA	-2.45	1.0000	
+NA	-1.89	0.9616	
5nM-NA	-1.38	0.6665	
50nM+NA	-1.38	0.6665	

Positive values show pairs of means that are significantly different.

Oneway Analysis of Stearic acid By Label

Means Comparisons

Comparisons with a control using Dunnett's Method

Control Group = -NA

|d|	Alpha
2.65691	0.05

Level	Abs(Dif)-LSD	p-Value
5nM-NA	-3.25	0.5706
-NA	-6.34	1.0000
50nM+NA	-4.62	0.9217
+NA	-4.6	0.9194
5nM+NA	-4.03	0.7933
50nM-NA	-3.82	0.7379

Positive values show pairs of means that are significantly different.

Oneway Analysis of succinate By Label

Means Comparisons

Comparisons with a control using Dunnett's Method

Control Group = -NA

|d|	Alpha
2.65691	0.05

Level	Abs(Dif)-LSD	p-Value	
5nM+NA	0.565	<.0001	*
50nM+NA	0.351	0.0006	*
+NA	0.317	0.0009	*
-NA	-0.53	1.0000	
50nM-NA	-0.1	0.1386	
5nM-NA	-0.07	0.1007	

Positive values show pairs of means that are significantly different.

Oneway Analysis of taurine By Label

Means Comparisons

Comparisons with a control using Dunnett's Method

Control Group = -NA

|d|	Alpha
2.65691	0.05

Level	Abs(Dif)-LSD	p-Value
50nM-NA	-0.79	0.9970
5nM-NA	-0.83	0.9995
-NA	-0.9	1.0000
50nM+NA	-0.78	0.9963
5nM+NA	-0.68	0.9454
+NA	-0.34	0.3516

Positive values show pairs of means that are significantly different.

Oneway Analysis of thiamine By Label

Means Comparisons

Comparisons with a control using Dunnett's Method

Control Group = -NA

|d|	Alpha
2.65691	0.05

Level	Abs(Dif)-LSD	p-Value
50nM-NA	-0.1	0.1088
5nM-NA	-0.15	0.1590
-NA	-0.71	1.0000
5nM+NA	-0.43	0.7549
50nM+NA	-0.29	0.3832
+NA	-0.22	0.2589

Positive values show pairs of means that are significantly different.

Oneway Analysis of threonine By Label

Means Comparisons

Comparisons with a control using Dunnett's Method

Control Group = -NA

|d|	Alpha
2.65691	0.05

Level	Abs(Dif)-LSD	p-Value	
5nM-NA	0.72	<.0001	*
50nM-NA	0.588	<.0001	*
5nM+NA	0.024	0.0394	*
50nM+NA	-0.13	0.1591	
+NA	-0.23	0.3470	
-NA	-0.6	1.0000	

Positive values show pairs of means that are significantly different.

Oneway Analysis of thymidine By Label

Means Comparisons

Comparisons with a control using Dunnett's Method

Control Group = -NA

|d|	Alpha
2.65691	0.05

Level	Abs(Dif)-LSD	p-Value
5nM-NA	-0.72	0.5490
5nM+NA	-0.97	0.8488
50nM-NA	-1.05	0.9198
50nM+NA	-1.07	0.9338
-NA	-1.45	1.0000
+NA	-1.24	0.9943

Positive values show pairs of means that are significantly different.

Oneway Analysis of trans, trans-farnesyl diphosphate By Label

Means Comparisons

Comparisons with a control using Dunnett's Method

Control Group = -NA

|d|	Alpha
2.65691	0.05

Level	Abs(Dif)-LSD	p-Value	
+NA	0.327	0.0124	*
50nM+NA	-0.2	0.1076	
5nM-NA	-0.75	0.5582	
5nM+NA	-1.06	0.9109	
50nM-NA	-1.19	0.9781	
-NA	-1.48	1.0000	

Positive values show pairs of means that are significantly different.

Oneway Analysis of tryptophan By Label

Means Comparisons

Comparisons with a control using Dunnett's Method

Control Group = -NA

|d|	Alpha
2.65691	0.05

Level	Abs(Dif)-LSD	p-Value
50nM-NA	0.936	<.0001 *
5nM-NA	0.62	<.0001 *
5nM+NA	-0.13	0.1859
50nM+NA	-0.24	0.4801
+NA	-0.47	0.9991
-NA	-0.52	1.0000

Positive values show pairs of means that are significantly different.

Oneway Analysis of tyrosine By Label

Means Comparisons

Comparisons with a control using Dunnett's Method

Control Group = -NA

|d|	Alpha
2.65691	0.05

Level	Abs(Dif)-LSD	p-Value	
50nM-NA	0.77	<.0001	*
5nM-NA	0.631	<.0001	*
5nM+NA	-0.18	0.2819	
50nM+NA	-0.3	0.6663	
+NA	-0.42	0.9699	
-NA	-0.53	1.0000	

Positive values show pairs of means that are significantly different.

Oneway Analysis of UDP By Label

Means Comparisons

Comparisons with a control using Dunnett's Method

Control Group = -NA

|d|	Alpha
2.65691	0.05

Level	Abs(Dif)-LSD	p-Value	
+NA	1.158	<.0001	*
5nM+NA	1.032	<.0001	*
50nM+NA	0.945	<.0001	*
50nM-NA	-0.5	1.0000	
5nM-NA	-0.5	1.0000	
-NA	-0.5	1.0000	

Positive values show pairs of means that are significantly different.

Oneway Analysis of UDP-D-glucuronate By Label

Means Comparisons

Comparisons with a control using Dunnett's Method

Control Group = -NA

|d|	Alpha
2.65691	0.05

Level	Abs(Dif)-LSD	p-Value
50nM+NA	0.062	0.0237 *
5nM+NA	-0.26	0.5506
-NA	-0.51	1.0000
+NA	-0.41	0.9806
5nM-NA	0.908	<.0001 *
50nM-NA	1.514	<.0001 *

Positive values show pairs of means that are significantly different.

Oneway Analysis of UDP-glucose By Label

Means Comparisons

Comparisons with a control using Dunnett's Method

Control Group = -NA

|d|	Alpha
2.65691	0.05

Level	Abs(Dif)-LSD	p-Value
5nM+NA	-0.55	0.3696
50nM+NA	-0.95	0.8580
5nM-NA	-1.17	0.9883
+NA	-1.28	0.9992
-NA	-1.41	1.0000
50nM-NA	1.426	<.0001 *

Positive values show pairs of means that are significantly different.

Oneway Analysis of UDP-N-acetyl-glucosamine By Label

Means Comparisons

Comparisons with a control using Dunnett's Method

Control Group = -NA

|d|	Alpha
2.65691	0.05

Level	Abs(Dif)-LSD	p-Value	
-NA	-0.46	1.0000	
50nM+NA	-0.11	0.1904	
5nM+NA	-0.07	0.1208	
+NA	0.088	0.0153	*
5nM-NA	0.476	<.0001	*
50nM-NA	1.348	<.0001	*

Positive values show pairs of means that are significantly different.

Oneway Analysis of uracil By Label

Means Comparisons

Comparisons with a control using Dunnett's Method

Control Group = -NA

|d|	Alpha
2.65691	0.05

Level	Abs(Dif)-LSD	p-Value
50nM+NA	-0.37	0.2746
+NA	-0.55	0.5044
5nM+NA	-0.81	0.8933
5nM-NA	-0.92	0.9782
-NA	-1.15	1.0000
50nM-NA	-1.03	0.9988

Positive values show pairs of means that are significantly different.

Oneway Analysis of Uric acid By Label

Means Comparisons

Comparisons with a control using Dunnett's Method

Control Group = -NA

|d|	Alpha
2.65691	0.05

Level	Abs(Dif)-LSD	p-Value
50nM+NA	-0.69	0.7623
5nM+NA	-0.84	0.9417
50nM-NA	-0.89	0.9735
-NA	-1.12	1.0000
5nM-NA	-1.1	1.0000
+NA	-1.02	0.9994

Positive values show pairs of means that are significantly different.

Oneway Analysis of UTP By Label

Means Comparisons

Comparisons with a control using Dunnett's Method

Control Group = -NA

|d|	Alpha
2.65691	0.05

Level	Abs(Dif)-LSD	p-Value	
+NA	-0.22	0.2937	
5nM+NA	-0.32	0.5162	
-NA	-0.66	1.0000	
50nM+NA	-0.64	1.0000	
5nM-NA	-0.64	1.0000	
50nM-NA	0.393	0.0009	*

Positive values show pairs of means that are significantly different.

Oneway Analysis of valine By Label

Means Comparisons

Comparisons with a control using Dunnett's Method

Control Group = -NA

|d|	Alpha
2.65691	0.05

Level	Abs(Dif)-LSD	p-Value	
50nM-NA	0.559	<.0001	*
5nM-NA	0.481	0.0002	*
5nM+NA	-0.34	0.6900	
50nM+NA	-0.52	0.9972	
-NA	-0.6	1.0000	
+NA	-0.58	1.0000	

Positive values show pairs of means that are significantly different.

Oneway Analysis of xanthine By Label

Means Comparisons

Comparisons with a control using Dunnett's Method

Control Group = -NA

|d|	Alpha
2.65691	0.05

Level	Abs(Dif)-LSD	p-Value	
50nM-NA	2.963	<.0001	*
5nM-NA	-0.48	0.9689	
-NA	-0.61	1.0000	
50nM+NA	-0.45	0.9234	
5nM+NA	-0.44	0.9132	
+NA	-0.2	0.2725	

Positive values show pairs of means that are significantly different.

Oneway Analysis of Xanthurenic acid By Label

Means Comparisons

Comparisons with a control using Dunnett's Method

Control Group = -NA

|d|	Alpha
2.65691	0.05

Level	Abs(Dif)-LSD	p-Value	
5nM+NA	1.012	<.0001	*
50nM-NA	0.897	<.0001	*
50nM+NA	0.844	<.0001	*
+NA	0.671	0.0002	*
5nM-NA	0.279	0.0054	*
-NA	-0.81	1.0000	

Positive values show pairs of means that are significantly different.
